# Associations between migrasome-related genes and long non-coding rnas in glioma and their prognostic relevance to the tumor microenvironment

**DOI:** 10.1016/j.ibneur.2026.06.013

**Published:** 2026-06-24

**Authors:** Biao Yang, Ren Li, Hubin Duan

**Affiliations:** a Department of Neurosurgery, First Hospital of Shanxi Medical University, Taiyuan, Shanxi Province 030001, China; b School of Public Health, Shanxi Medical University, Taiyuan, Shanxi Province 030001, China

**Keywords:** Glioma, Migrasomes, Long Non-Coding RNAs, Tumor Microenvironment, Prognostic Model

## Abstract

**Objective:**

This exploratory study evaluated associations between migrasome-related genes and long non-coding RNAs (lncRNAs) in glioma and examined whether migrasome-related lncRNA patterns were associated with prognosis and the tumor microenvironment.

**Methods:**

Transcriptomic, clinical, survival, and mutation data were obtained from the TCGA GDC portal. Migrasome-related genes were identified from published literature and GeneCards, and co-expressed lncRNAs were screened using |cor| > 0.4 and P < 0.05. A total of 674 glioma cases with complete survival information were randomly divided into training and internal testing cohorts (1:1; n = 337 each). Univariate Cox regression, LASSO Cox regression with tenfold cross-validation, and multivariate Cox regression were used to construct an eight-lncRNA risk model. External cohorts (CGGA325, CGGA693, and GSE16011) were screened for model-gene coverage. Because these platforms covered only part of the eight-lncRNA signature, we performed available-gene external survival analyses and available-gene partial-score analyses after within-cohort z-score normalization. Immune infiltration, TME scores, TMB, TIDE scores, and predicted drug sensitivity were analyzed as exploratory correlates.

**Results:**

The eight-lncRNA risk score was associated with overall survival in the training, internal testing, and overall TCGA cohorts (P < 0.001). In the overall TCGA cohort, the risk score showed an AUC of 0.880. Full external validation of the eight-lncRNA signature was not feasible because CGGA325 covered three model lncRNAs (CRNDE, AC007879.2, and LINC00092), CGGA693 covered two (CRNDE and LINC00092), and GSE16011 covered only CRNDE. Available-gene partial scores remained associated with overall survival in CGGA325 (n = 313, HR = 1.54, 95% CI: 1.37–1.74, P = 4.78 ×10^-13), CGGA693 (n = 657, HR = 1.34, 95% CI: 1.27–1.42, P = 8.98 ×10^-25), and GSE16011 (CRNDE only; n = 240, HR = 1.43, 95% CI: 1.25–1.64, P = 3.85 ×10^-7). High-risk tumors showed higher stromal, immune, and ESTIMATE scores and increased macrophage infiltration, including M2 macrophages. Higher TMB was associated with worse survival, and predicted drug-response analyses suggested candidate compounds for further validation.

**Conclusion:**

Migrasome-related lncRNA expression patterns were associated with glioma prognosis and immune microenvironment features in TCGA data. External cohorts provided partial support for the prognostic relevance of available model lncRNAs, particularly CRNDE, but did not permit full eight-lncRNA signature validation because of incomplete gene coverage. These findings remain hypothesis-generating and require complete external cohort validation and experimental studies before mechanistic or clinical application can be inferred.

## 1 Introduction

Glioma, a malignant tumor arising from glial cells, is the most prevalent form of central nervous system malignancy, comprising about 80% of these cases. Gliomas are characterized by high heterogeneity. According to the latest WHO guidelines, gliomas are classified into five major categories, with the most common type being adult-type diffuse gliomas (Louis et al., 2021, Weller et al., 2024). The age-standardized incidence rate (ASIR) of gliomas is 4.67 cases per 100,000 population in both the Northwest region of the United Kingdom and Finland, with a higher incidence observed in males compared to females (Larjavaara et al., 2007, Sehmer et al., 2014). There is currently an absence of global epidemiological studies on glioma that consider population classifications. However, existing evidence suggests that the incidence of gliomas varies across different regions. The incidence in Europe and the United States is significantly higher than in Asia, potentially approaching twofold differences (Leece et al., 2017). Additionally, the incidence rates of gliomas can vary significantly due to factors such as ethnicity, population, age, and geographical location (Lin et al., 2021). Gliomas are classified into four grades, with high-grade gliomas having a very short overall survival period of approximately 1.5–3 years (grades three and four) (Wang et al., 2023), while low-grade gliomas may have a survival period exceeding ten years (Louis et al., 2016).

Migrasomes are a newly discovered type of organelle that are generated at the tail end of migrating cells, producing retractile fibers upon cellular migration. Small vesicles, known as migrasomes, form at the connections or tips of these fibers, and their functions are closely related to cell migration (Wang and Yu, 2024). Cell migration is essential for biological development and cellular functions, significantly influencing angiogenesis, wound healing, immune response, and cancer cell invasion and metastasis (Aman and Piotrowski, 2010, Stuelten et al., 2018). The swift migration and invasion of cancer cells significantly contribute to the high malignancy of gliomas. Cancer cells can grow rapidly within the cranial cavity, transitioning from small intracranial lesions to extensive mass lesions in a very short period. This high invasive and migratory capability may be associated with the function of migrasomes in gliomas. Research indicates that migrasome production rises in highly migratory gliomas, aiding cancer cell survival (Lee et al., 2024). In rat models, a correlation has been observed between the increase in migrasomes and enhanced proliferation, invasion, and metastatic capabilities of gliomas. The results indicate that migrasomes are potentially crucial in glioma development and progression. Research on the link between migrasomes and gliomas is limited, with the specific mechanisms of their effects remaining unclear.

Long non-coding RNAs (lncRNAs) are RNA molecules exceeding 200 nucleotides that mainly regulate gene and protein expression. lncRNAs are less conserved across species and typically have lower expression levels than microRNAs, but they exhibit more pronounced tissue-specific expression (Mukherjee et al., 2017). This characteristic has garnered increasing attention from researchers regarding the role of lncRNAs in gliomas. LncRNAs are widely involved in influencing the disease progression of tumors, playing important roles in tumor proliferation, apoptosis, migration, invasion, and angiogenesis, and this is also true in the context of gliomas. Current research has identified numerous lncRNAs that significantly promote glioma migration and invasion, tumor angiogenesis, and anti-apoptosis (Wei et al., 2022, Chen et al., 2023, Shree et al., 2023, Shamshiripour et al., 2022). Furthermore, constructing prognostic and diagnostic models for gliomas based on lncRNAs with different biological functions also holds considerable value (Jin et al., 2023).

These observations suggest that migrasome biology and lncRNA regulation may converge on cell migration, invasion, and tumor microenvironment remodeling in glioma. However, direct molecular interactions between migrasomes and lncRNAs have not been established. Therefore, the present study was designed as an exploratory bioinformatics analysis to identify lncRNAs co-expressed with migrasome-related genes and to evaluate their prognostic associations in glioma. We used TCGA transcriptomic and clinical data to construct and internally test an eight-lncRNA risk model, then examined immune infiltration, TMB, predicted immunotherapy response, and drug-sensitivity correlates. The aim was to generate testable hypotheses rather than to demonstrate causality or immediate clinical utility.

## 2 Methods

### 2.1 Data source and data processing

The transcriptomic, clinical, survival, and tumor mutation data for glioma were obtained from the TCGA GDC portal (https://portal.gdc.cancer.gov/). The initial expression matrix included glioma tumor samples and five normal-adjacent samples; 674 tumor cases with complete survival information were retained for prognostic modeling. Gene reference files were used to differentiate coding and non-coding gene sets (the gene reference files are provided in the supplementary materials). Migrasome-related genes were sourced from published literature and GeneCards; after removing duplicated entries, 11 unique genes from 12 retrieved entries were analyzed (Zhu et al., 2021). Co-expression analysis identified lncRNAs associated with migrasome-related genes using |cor| > 0.4 and P < 0.05. Because co-expression alone cannot establish biological interaction or causality, these lncRNAs were considered migrasome-related candidates for downstream hypothesis-generating analyses. Data analyses utilized R software (v4.3.3) with the "limma" package (v3.60.6) and Perl language. We integrated survival, clinically relevant, and tumor mutation data for glioma. Additionally, clinical data from 33 other tumor types were obtained from TCGA, culminating in an analysis of 10,228 samples. All expression data were extracted in TPM format for analysis.

### 2.2 Construction of a prognostic model for gliomas and evaluation of model accuracy

To develop a prognostic model for gliomas while reducing multicollinearity and overfitting, we utilized the Least Absolute Shrinkage and Selection Operator (LASSO) Cox regression technique. The 674 eligible TCGA cases were randomly divided into a training cohort and an internal testing cohort at a 1:1 ratio (n = 337 each). Model training was performed in the training cohort. After univariate Cox regression analysis, eligible risk factors were incorporated into LASSO Cox analysis. Following tenfold cross-validation, the lambda.1se value was selected for model construction, and a multivariate Cox model generated the final risk score. The median risk score in the training cohort was used as the cutoff and then applied unchanged to the internal testing cohort and the overall TCGA cohort. Model performance was assessed using Kaplan-Meier survival curves, ROC and time-dependent ROC curves, univariate and multivariate Cox regression, a prognostic nomogram, calibration curves, and C-index analysis.

For external transferability assessment, three independent glioma datasets added in the revision were examined: CGGA325, CGGA693, and GSE16011. The expression matrices were screened for the eight model lncRNAs. CGGA325 contained CRNDE, AC007879.2, and LINC00092; CGGA693 contained CRNDE and LINC00092; and GSE16011 contained CRNDE only. Because the full eight-lncRNA signature could not be reconstructed in these platforms, we did not claim complete external validation. Instead, we performed exploratory survival analyses for available model lncRNAs and available-gene partial scores. For each external cohort, available genes were z-score normalized within that cohort, multiplied by their TCGA-derived Cox coefficients, and summed to create an available-gene partial score. Univariate Cox regression reported hazard ratios per 1 standard deviation increase, and Kaplan-Meier analysis used cohort-specific median expression or partial-score cutoffs.

### 2.3 Functional enrichment analysis of glioma risk groups

Principal component analysis (PCA) was initially conducted to examine separation between high-risk and low-risk groups. Differential gene analysis was then performed using |logFC| > 1 and FDR < 0.05 as selection criteria. Gene Ontology (GO), Kyoto Encyclopedia of Genes and Genomes (KEGG), and gene set enrichment analysis (GSEA) were performed on differentially expressed genes to characterize pathways associated with risk groups. These enrichment analyses were exploratory and were not used to infer causality. The R packages utilized included "clusterProfiler" (version 4.10.1) and "org. Hs.eg.db" (version 3.18.0). Supplementary materials include the primary reference materials.

### 2.4 Analysis of tumor microenvironment and immune function in gliomas

Using the "estimate" package, we evaluated tumor microenvironment scores in gliomas categorized as high-risk and low-risk (refer to supplementary materials for additional information). The CIBERSORT algorithm was employed to estimate the distribution of 22 immune cell types in high-risk and low-risk glioma patients (Newman et al., 2015). Immune function analysis was concurrently conducted (reference materials are included in the supplementary materials). These analyses were based on bulk transcriptomic deconvolution and therefore represent estimated immune profiles rather than direct cell-count measurements.

### 2.5 Analysis of tumor mutations and immunotherapy in glioma risk groups

We examined key gene mutation differences and dominant mutation types between high-risk and low-risk groups by integrating mutation and clinical data. Survival analysis was then performed according to mutation profiles. We used the TIDE database to estimate immunotherapy-related scores for gliomas, while noting that TIDE was developed mainly in melanoma and non-small cell lung cancer and may have limited validity for CNS tumors. We utilized the "oncoPredict" package to predict drug sensitivity in high-risk and low-risk groups using GDSC2 reference data. These predicted IC50 values were interpreted as computational hypotheses and not as evidence of clinical drug efficacy.

### 2.6 Clinical characteristics and prognostic analysis of lncRNA models

For 33 types of cancer, we analyzed pan-cancer survival associations for the eight model lncRNAs. We also examined the differential expression of individual lncRNAs according to glioma clinical features, including age, WHO grade, IDH mutation status, 1p/19q co-deletion, pathological subtype, and treatment status. ROC analysis was conducted to evaluate the ability of individual lncRNAs to classify these clinical features within the available TCGA data.

## 3 Results

### 3.1 Screening of Co-expressed lncRNAs and migrasome-related genes and construction of prognostic models

**Fig. 1:**
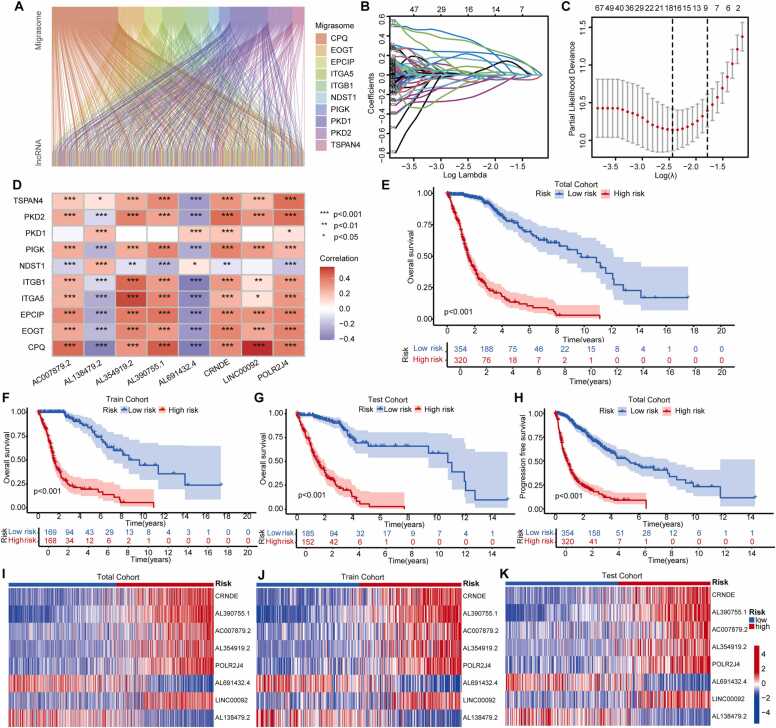
Screening for Migrasome-Related lncRNAs and Construction of the Prognostic Model. (A) Co-expression analysis of lncRNAs co-expressed with migrasome-related genes. (B-C) The coefficients of selected features are determined by the lambda parameter; the x-axis represents variable lambda values, while the y-axis represents the coefficients of variables. A plot illustrating the relationship between partial likelihood deviance and log(λ) using the LASSO Cox regression model is presented. (D) Expression correlation between model lncRNAs and migrasome-related genes; red indicates positive correlation, while blue indicates negative correlation. (E) Kaplan-Meier (KM) curve showing overall survival differences between high-risk and low-risk glioma patients. (F) High-risk patients in the training cohort exhibit lower survival probabilities. (G) In the internal testing cohort, high-risk patients demonstrate significantly lower survival probabilities. (H) In the overall glioma cohort, progression-free survival also indicates lower survival probabilities for high-risk patients. (I-K) Heatmaps of expression differences for model lncRNAs between high-risk and low-risk groups in the overall cohort, training cohort, and internal testing cohort. With the exception of AL691432.4 and AL138479.2, which show lower expression in the high-risk group, the other model lncRNAs are highly expressed in the high-risk group.

Based on published literature and online predictions, 12 migrasome-related gene entries were retrieved. Through co-expression analysis, 656 lncRNAs were identified as migrasome-related candidate lncRNAs (Supplementary Table 1) (Fig. 1A). After univariate Cox regression analysis (Supplementary Figure 1), eligible lncRNAs were included in LASSO Cox regression, which identified an eight-lncRNA prognostic model for gliomas (Figs. 1B, 1C). The risk score was calculated as follows: Risk score = (CRNDE * 0.26) + (AL390755.1 * 0.32) + (AC007879.2 * 0.52) + (AL354919.2 * 0.21) + (POLR2J4 * 0.52) + (AL691432.4 * −0.43) + (LINC00092 * 0.25) + (AL138479.2 * −0.36). The associated heatmap illustrates correlations between model lncRNAs and migrasome-related genes (Fig. 1D). Most pairs showed statistically significant correlations, whereas PKD1 showed no association with several model lncRNAs. Kaplan-Meier survival analysis indicated that in the overall glioma cohort, the high-risk group included 320 cases and the low-risk group included 354 cases. High-risk patients had lower survival probabilities than low-risk patients (P < 0.001) (Fig. 1E). The same risk-score formula and training-cohort cutoff separated high-risk and low-risk patients in both the training cohort and internal testing cohort (P < 0.001) (Figs. 1F, 1G). Consistent survival associations were also observed for progression-free survival in the overall cohort (Fig. 1H).

In both the training and internal testing cohorts, model lncRNA expression patterns were generally consistent with coefficient directions. CRNDE, AL390755.1, AC007879.2, AL354919.2, POLR2J4, and LINC00092 were relatively higher in high-risk tumors, whereas AL691432.4 and AL138479.2 were relatively lower in high-risk tumors (Fig. 1I-K). The complete risk curve is available in the supplementary materials.

### 3.2 Model evaluation and subgroup survival analysis

**Fig. 2:**
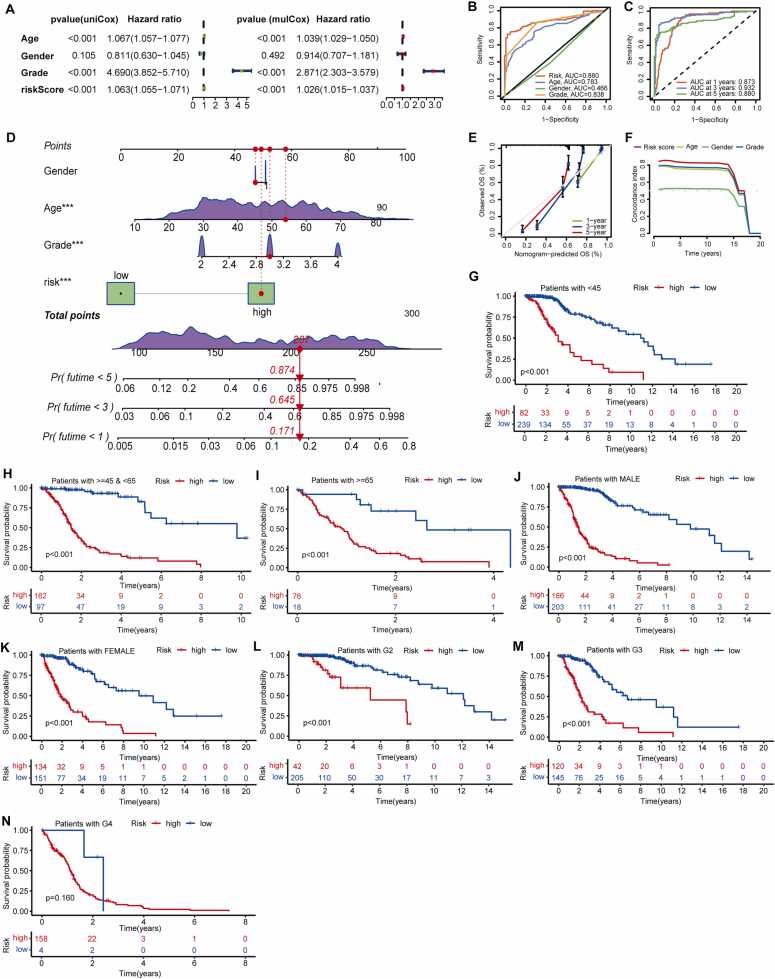
Evaluation of the Prognostic Model and Subgroup Survival Analysis. (A) Univariate and multivariate regression analyses indicate that the risk score is independently associated with survival in the TCGA cohort. (B) Internal ROC analysis shows an AUC value of 0.880 for the risk model. (C) Time-dependent ROC analysis of the risk model at 1, 3, and 5 years, with the highest AUC at 3 years (AUC = 0.932). (D) The prognostic nomogram displays the scoring of patient 207 in the comprehensive prognostic model. (G-I) Kaplan-Meier (KM) curves for age subgroups: high-risk patients exhibit lower survival probabilities in the age groups under 45 years, 45–65 years, and over 65 years. (J-K) KM curves for gender subgroups: high-risk glioma patients show reduced survival probabilities among both males and females. (L-M) KM curves for WHO grade subgroups: in G2 and G3 gliomas, high-risk patients have lower survival probabilities than low-risk patients; however, no significant difference is observed between high-risk and low-risk groups in G4 glioma patients.

Following model construction, we conducted univariate and multivariate Cox regression analyses with clinical characteristics of gliomas, including age, sex, and WHO grade. Age, WHO grade, and the risk score were independently associated with overall survival in the TCGA cohort (Fig. 2A). In internal ROC analysis, the risk model showed an AUC of 0.880, compared with 0.838 for WHO grade (Fig. 2B). The risk model demonstrated the highest time-dependent AUC at 3 years (AUC = 0.932) (Fig. 2C). These TCGA-based estimates represent internal performance and should not be interpreted as complete external validation.

Subsequently, prognostic nomograms and calibration curves suggested that the risk model retained predictive value at 1, 3, and 5 years in the available TCGA data (Figs. 2D, 2E). C-index analysis also showed that the risk model had a higher score than the evaluated clinical features (Fig. 2F). Subgroup survival analysis showed that high-risk patients had reduced survival probabilities across age groups (Fig. 2G-I). High-risk patients among both males and females also showed reduced survival, suggesting that the association between risk score and outcome was not limited to a single sex or age subgroup.

We next evaluated whether externally available model lncRNAs showed consistent survival associations in CGGA325, CGGA693, and GSE16011. The complete eight-lncRNA score could not be reconstructed because several model lncRNAs were absent from each external expression platform. In CGGA325, an available-gene partial score based on CRNDE, AC007879.2, and LINC00092 was associated with worse overall survival (n = 313; HR per 1 SD increase = 1.54, 95% CI: 1.37–1.74, Cox P = 4.78 ×10^−13; log-rank P = 9.32 ×10^−7). In CGGA693, a partial score based on CRNDE and LINC00092 was also associated with worse overall survival (n = 657; HR = 1.34, 95% CI: 1.27–1.42, Cox P = 8.98 ×10^−25; log-rank P = 3.23 ×10^−29). In GSE16011, only CRNDE was available and was associated with worse overall survival (n = 240; HR = 1.43, 95% CI: 1.25–1.64, Cox P = 3.85 ×10^−7; log-rank P = 2.52 ×10^−8). These results support the external prognostic relevance of available model lncRNAs but do not validate the complete eight-lncRNA model (Supplementary Figure 2 and Supplementary Table 6).

In further analysis of WHO grading, we found that high-risk groups among lower-grade gliomas had a significantly lower survival probability, whereas survival among high-grade gliomas was not significantly different (P = 0.16). It is worth noting that there were only four low-risk patients, which did not reach a sufficient statistical sample size, potentially accounting for this negative finding.

### 3.3 Evaluation of gene typing and enrichment analysis of differentially expressed genes

**Fig. 3:**
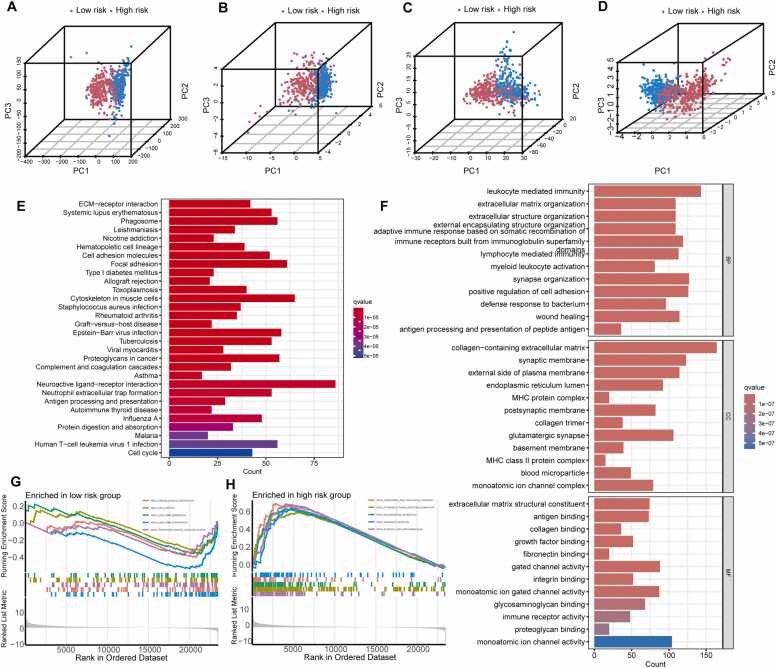
**Functional Enrichment Analysis of High-Risk Group.** (A-D) Analysis distinguishing high-risk and low-risk patients using different model types: A represents the classification based on all genes; B shows the analysis of migrasome-related genes; C illustrates the classification of co-expressed lncRNAs associated with migrasomes; D represents the classification of risk lncRNAs. The classification in D is particularly effective, significantly distinguishing between high-risk and low-risk glioma patients. (E) KEGG differential analysis between high-risk and low-risk groups. (F) GO analysis of high-risk and low-risk groups. Note: All results were FDR-corrected, meeting the criteria of FDR < 0.05. (G-H) GSEA analysis of differentially expressed genes: G indicates the main enriched pathways in the low-risk group, while H indicates the main enriched pathways in the high-risk group.

Principal component analysis (PCA) was employed to examine the separation between high-risk and low-risk glioma patients. This analysis was performed on all genes (Fig. 3A), migrasome-related genes (Fig. 3B), co-expressed lncRNAs associated with migrasome-related genes (Fig. 3C), and risk lncRNAs (Fig. 3D). The clearest separation was observed when risk lncRNAs were used. Based on these classifications, differential expression analysis identified 3852 differentially expressed genes (Supplementary Table 2). A total of 1466 GO terms (Supplementary Table 3) and 73 KEGG pathways (Supplementary Table 4) were obtained, with the top-ranking results presented.

The analysis identified that differentially expressed genes were mainly linked to pathways including ECM-receptor interaction, systemic lupus erythematosus, phagosome, leishmaniasis, and nicotine addiction (Fig. 3E). GO analysis revealed that the differentially expressed genes participated in biological processes like leukocyte-mediated immunity and extracellular matrix organization. In cellular components, these genes were associated with the collagen-containing extracellular matrix, synaptic membrane, and the external side of the plasma membrane. In molecular functions (MF), the differentially expressed genes were linked to extracellular matrix structural constituents, antigen binding, collagen binding, and growth factor binding (Fig. 3F).

Enrichment analysis of all differentially expressed genes revealed that in the low-risk group, these genes were mainly associated with pathways involving myocardial contraction, gap junctions, and long-term depression. Conversely, the high-risk group exhibited enrichment of differentially expressed genes in pathways including complement and coagulation cascades, cytokine-cytokine receptor interactions, and ECM-receptor interactions (Figs. 3G, 3H).

### 3.4 Analysis of tumor microenvironment and enrichment of immune cells and their functions

**Fig. 4:**
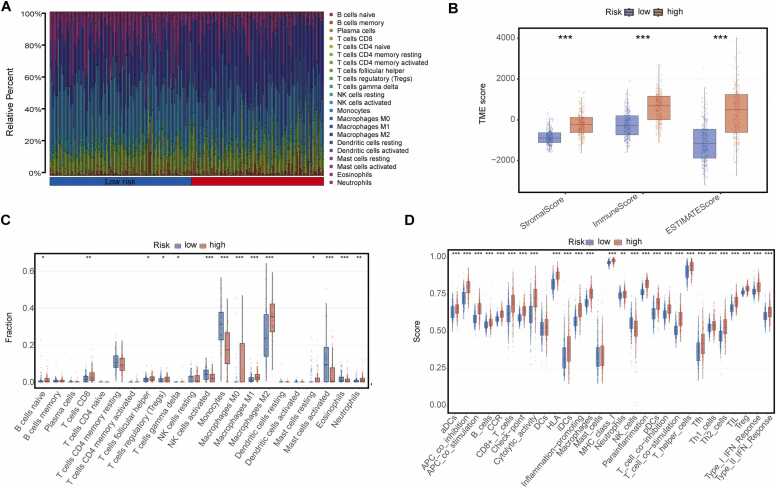
Tumor Immunity Correlation Analysis. (A) Immune landscape showing the distribution of 22 estimated immune cell types between high-risk and low-risk groups. (B) Differences in stromal scores, immune scores, and ESTIMATE scores between high-risk and low-risk groups, with individual data points overlaid. (C) Box plots illustrating differences in the 22 estimated immune cell types between high-risk and low-risk groups, with individual data points overlaid. (D) Differences in immune function scores between high-risk and low-risk groups, with individual data points overlaid.

We employed the CIBERSORT method to estimate differences in 22 immune cell types between high-risk and low-risk groups, resulting in a distribution landscape map (Fig. 4A). In the revised figure, individual data points were overlaid on the immune cell box plots as requested. The estimated fractions of macrophage subtypes M0, M1, and M2 were higher in the high-risk group, whereas monocytes and resting NK cells were lower (Fig. 4C).

The tumor microenvironment (TME) scores showed that stromal, immune, and ESTIMATE scores were higher in the high-risk group than in the low-risk group (Fig. 4B). Individual data points were added to the revised Fig. 4B-D panels. Immune function analysis identified differences between high-risk and low-risk groups; all evaluated functions except cytolytic activity and mast cells showed statistically significant differences, with higher estimated scores in the high-risk group (Fig. 4D).

### 3.5 Analysis of tumor mutational burden and immunotherapy

**Fig. 5:**
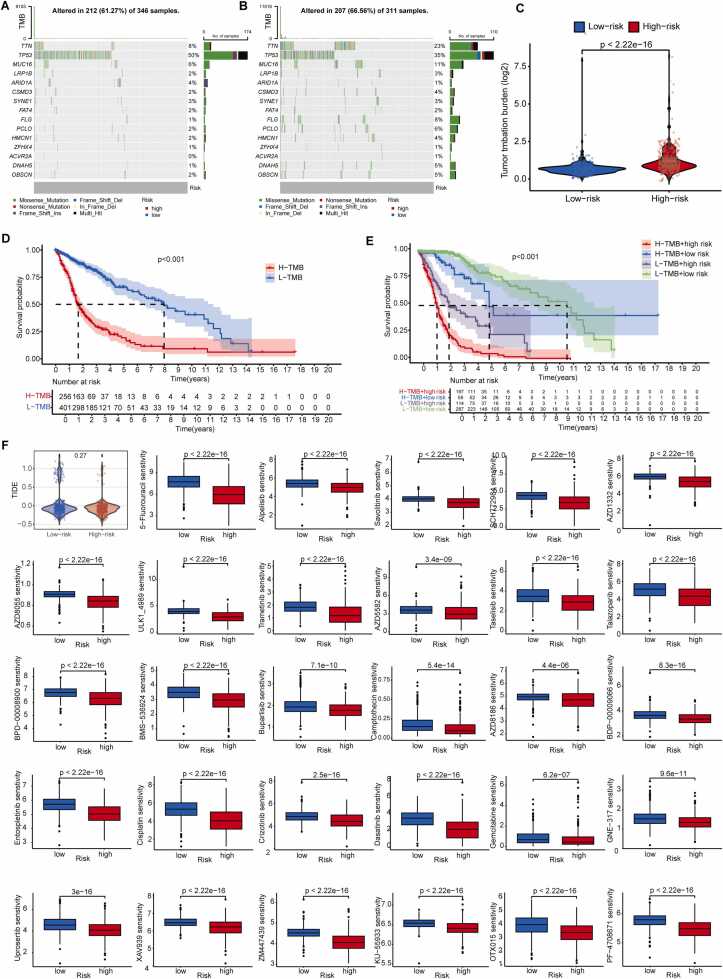
Tumor Mutational Burden and Immunotherapy Analysis. (A-B) Analysis of tumor mutational burden in high-risk and low-risk groups: A represents mutation status in the low-risk group, while B shows mutation status in the high-risk group. Differences in mutations of TTN, MUC16, and FLG between the two groups are notable. (C) Violin plots with individual data points show that TMB is higher in the high-risk group than in the low-risk group. (D) Kaplan-Meier (KM) survival curves show that patients in the high-TMB group have lower survival probabilities than those in the low-TMB group, with median survival times of 1.7 years for the high-TMB group and 8 years for the low-TMB group. (E) Combining risk score and TMB, comprehensive KM survival curves indicate that the high-risk/high-TMB group has the lowest survival probabilities, while the low-risk/low-TMB group shows the best survival outcomes. (F) TIDE analysis with individual data points evaluates predicted immunotherapy-related scores; because TIDE has limited glioma-specific validation, this result is exploratory. Drug-sensitivity analysis identifies candidate compounds for future validation; predicted IC50 values indicate computationally estimated drug response rather than confirmed treatment efficacy.

Using reference files, we categorized glioma patients with available clinical and tumor mutation data into high-TMB and low-TMB groups. The low-TMB group comprised 212 glioma patients, and the high-TMB group included 207 glioma patients. In the high-risk group, mutations in TTN, MUC16, FLG, and DNAH5 were more prevalent, whereas TP53 and ARID1A mutations were less common (Figs. 5A, 5B). Violin plots with individual data points indicated higher TMB in the high-risk group than in the low-risk group (Fig. 5C). Kaplan-Meier survival analysis showed that patients with elevated TMB had shorter survival than those with low TMB (Fig. 5D).

Integration with the risk model showed that patients with high-risk and high-TMB tumors had the lowest survival probabilities, whereas those with low-risk and low-TMB tumors had the best survival outcomes (Fig. 5E). The TIDE score analysis, shown with individual data points in the revised Fig. 5F, indicated no significant difference in predicted immunotherapy response between high-risk and low-risk groups. This result should be interpreted cautiously because TIDE was not developed specifically for glioma. We then used oncoPredict for exploratory drug-sensitivity prediction. Drugs with lower predicted IC50 values in the high-risk group were considered candidate compounds for future validation, not confirmed effective therapies (Fig. 5F).

### 3.6 Pan-cancer survival analysis and clinical features analysis of model genes

**Fig. 6:**
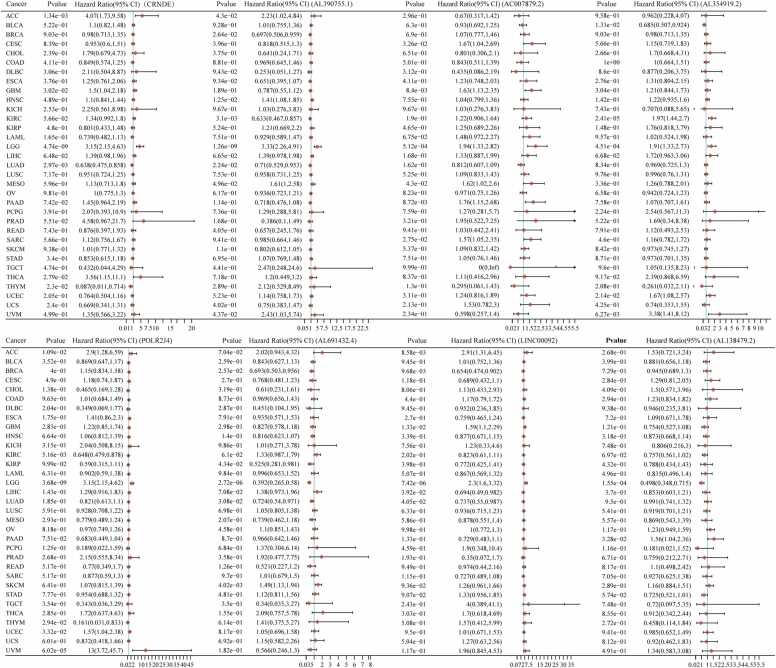
**Pan-Cancer Survival Forest Plot of Model lncRNAs.** A pan-cancer survival analysis was conducted on eight model lncRNAs. The model lncRNAs demonstrated specific associations with glioma risk.

After analyzing 33 types of cancer, we conducted Kaplan-Meier (KM) survival association analyses for the model lncRNAs (Fig. 6). CRNDE was associated with higher risk in ACC and LGG, with increased expression correlated with reduced survival in these two cancers. AL390755.1 was associated with higher risk in LGG. AC007879.2 was associated with higher risk in LGG, GBM, and PAAD. AL354919.2 was associated with higher risk in LGG and lower risk in UVM. POLR2J4 was associated with higher risk in both LGG and UVM. AL691432.4 was associated with lower risk in gliomas but higher risk in SKCM. LINC00092 was associated with higher risk, whereas AL138479.2 was associated with lower risk in LGG.

**Fig. 7:**
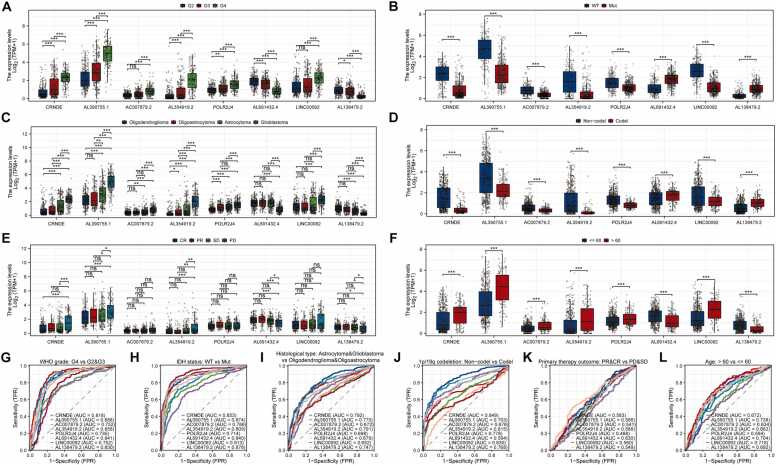
**Clinical Correlation Analysis of Major Model lncRNAs. (A)** Expression of the eight model genes differs across various WHO grades; except for AL691432.4 and AL138479.2, which decrease as the WHO grade increases, the remaining lncRNAs show increased expression with higher WHO grades. **(B)** With the exception of AL691432.4 and AL138479.2, which are significantly upregulated in the IDH mutant type, the others exhibit significant downregulation in this mutant type. **(C)** Among different pathological types of gliomas, risk lncRNAs are significantly upregulated in higher-grade astrocytomas and glioblastomas, while protective lncRNAs are downregulated. **(D)** In relation to 1p/19q co-deletion, protective lncRNAs are found to be upregulated in the coding group, while risk lncRNAs are downregulated. **(E)** In the analysis of glioma treatment, risk lncRNAs are upregulated during disease progression and downregulated during complete remission, while protective lncRNAs similarly decrease during disease progression. **(F)** Protective lncRNAs are downregulated in older patients, while risk lncRNAs are upregulated in this demographic. **(G-L)** ROC curve analysis evaluating the diagnostic capability of different model lncRNAs for glioma characteristics: apart from the relatively low diagnostic capabilities for glioma treatment during remission and progression phases, the remaining assessments demonstrate good diagnostic efficacy, with the lowest AUC value at 0.594 and the highest at 0.913.

We performed a clinical features analysis of the model lncRNAs in gliomas. With the exception of AL691432.4 and AL138479.2, which decreased in high-grade tumors, the remaining six model lncRNAs exhibited gradually increasing expression in high-grade gliomas (Fig. 7A). For IDH mutations, AL691432.4 and AL138479.2 showed increased expression in the mutated type, correlating with their protective effects and leading to reduced survival probabilities in gliomas (Fig. 7B). In the four glioma pathological types, higher malignancy correlated with elevated risk lncRNA expression and reduced protective lncRNA expression (Fig. 7C).

Regarding another clinical characteristic in gliomas, we observed that protective lncRNAs increased in the coding group with 1p/19q co-deletion, while risk lncRNAs decreased (Fig. 7D). Additionally, in clinical characteristics related to glioma treatment, we discovered that risk lncRNAs decreased during the complete remission phase but increased during disease progression, indicating that their elevation is associated with disease progression (Fig. 7E). Risk lncRNAs significantly increased in older patients, while protective lncRNAs significantly decreased (Fig. 7F).

We further evaluated the ability of individual lncRNAs to classify clinical characteristics. In distinguishing high-grade from low-grade gliomas, all eight model lncRNAs showed AUC values above chance, with AL390755.1 having the highest AUC value of 0.888 (Fig. 7G). LINC00092 showed an AUC of 0.913 for IDH mutation status (Fig. 7H). CRNDE showed an AUC of 0.792 for distinguishing pathological malignancy groups (Fig. 7I) and an AUC of 0.849 for 1p/19q co-deletion status (Fig. 7J). However, classification of treatment-related prognosis indicators was limited, with the highest AUC value being only 0.630 (Fig. 7K). AL390755.1 differentiated patients aged over and under 60 with an AUC of 0.738 (Fig. 7L).

## 4 Discussion

To our knowledge, analyses connecting migrasome-related gene expression with lncRNA-based glioma risk stratification remain limited. In this TCGA-based exploratory study, co-expression analysis followed by Cox and LASSO modeling identified eight candidate migrasome-related lncRNAs (CRNDE, AL390755.1, AC007879.2, AL354919.2, POLR2J4, AL691432.4, LINC00092, AL138479.2). The signature was associated with survival and with immune and TMB-related features. The apparent AUC of the risk model was higher than that of WHO grade in the internal TCGA analysis (0.880 vs. 0.838), but this should not be interpreted as definitive clinical performance. We therefore frame the model as hypothesis-generating rather than as a validated diagnostic or prognostic tool.

The main distinction from previously reported glioma lncRNA signatures is the migrasome-oriented candidate selection and the integration of immune infiltration, TMB, predicted immunotherapy response, drug sensitivity, pan-cancer survival, and glioma clinical features for the same eight-lncRNA set. This provides a biologically focused prioritization strategy for future mechanistic work, but it does not by itself prove a functional migrasome-lncRNA axis.

A major malignant characteristic of gliomas is their infiltrative and invasive capability; however, the specific mechanisms underlying this phenotype remain incompletely understood. Changes in the tumor microenvironment (TME), such as hypoxia, may contribute to glioma invasion. Prior studies have shown that hypoxia can increase glioblastoma cell migration and that hypoxia-inducible factors contribute to proliferation, invasion, and stem-like phenotypes (Tamai et al., 2022, Filatova et al., 2016, Lee et al., 2016). Immune infiltration is also relevant to glioma biology and treatment response (Zhang et al., 2021a, Ma et al., 2018, Zhang et al., 2021b). In our analysis, high-risk tumors showed higher estimated macrophage fractions, including M2-like macrophages, and higher ESTIMATE-derived stromal and immune scores. This is consistent with previous reports that microglia and macrophages constitute a substantial proportion of glioma tissue and that macrophage infiltration, especially M2-like phenotypes, is associated with glioma malignancy and poor prognosis (Hambardzumyan et al., 2016, Komohara et al., 2008, Tao et al., 2023). However, bulk RNA deconvolution cannot fully distinguish resident microglia from infiltrating macrophages and does not establish cell-cell mechanisms. Similarly, TMB and TIDE results require glioma-specific interpretation: CNS tumors differ from melanoma and non-small cell lung cancer in immune surveillance and immunotherapy response, and TIDE has not been optimized for glioma. Therefore, the TMB, TIDE, and drug-sensitivity analyses should be regarded as exploratory predictions requiring validation in glioma-specific clinical or experimental systems.

We also conducted pan-cancer survival association analysis of the eight candidate migrasome-related lncRNAs and evaluated their associations with glioma clinical features. The six risk-associated lncRNAs (CRNDE, AL390755.1, AC007879.2, AL354919.2, POLR2J4, LINC00092) tended to be upregulated in glioma groups with more malignant features, whereas the two protective lncRNAs (AL138479.2, AL691432.4) tended to show the opposite pattern. CRNDE (colorectal neoplasia differentially expressed) is overexpressed in several cancers and has been associated with tumor cell proliferation, invasion, and metastasis (Lu et al., 2020). In gliomas, elevated CRNDE levels have been associated with enhanced proliferation and migration, larger tumor volume, higher WHO grade, and poorer survival outcomes (Jing et al., 2016, Brat et al., 2004). Increased CRNDE expression may also influence glioma resistance through glycolytic pathways (Yang et al., 2024). Our findings are consistent with CRNDE being a risk-associated lncRNA in glioma, while whether this association involves migrasome biology remains to be experimentally tested.

Similar to CRNDE, AL390755.1 has been implicated in glioma biology through glycolytic pathways. Xu and colleagues reported lower AL390755.1 expression compared with normal glial cells (Xu et al., 2023), which differs from the pattern observed in our TCGA-based analysis. This discrepancy may reflect differences in sample composition, tumor stage, platform, or analytical strategy, and it requires further validation. POLR2J4 has been linked to hepatocellular carcinoma and metastatic breast cancer (Park et al., 2020, Ma and Deng, 2019), and other work has suggested that POLR2J4 may influence glioma biology through N6-methyladenosine-related pathways. AC007879.2, LINC00092, AL138479.2, and AL691432.4 remain less characterized in glioma. Our results nominate these lncRNAs as candidates for future study, but their functional roles and any relationship to migrasome biology require direct experimental validation.

This study has several limitations. First, all analyses were retrospective. Although three external datasets (CGGA325, CGGA693, and GSE16011) were added, their expression platforms did not cover all eight model lncRNAs. Therefore, we could perform only available-gene and partial-score external survival analyses, not full eight-lncRNA model validation. Complete external validation and cross-platform robustness testing are still required before clinical use. Second, the evidence connecting lncRNAs to migrasome biology is based on co-expression with a moderate correlation threshold and does not demonstrate direct molecular interaction, causality, or migrasome-dependent function. Third, batch effects, platform differences, missing clinical variables, and model-parameter choices may affect estimates. Fourth, immune deconvolution, TMB, TIDE, and oncoPredict analyses are computational approximations and require glioma-specific validation. Finally, prospective clinical data and experimental work in cells, animal models, or patient-derived samples are needed to test the proposed biological hypotheses.

In conclusion, an eight-lncRNA signature selected from migrasome-related co-expression candidates was associated with survival and immune microenvironment features in glioma TCGA data. External available-gene analyses in CGGA325, CGGA693, and GSE16011 partially supported the prognostic relevance of detectable model lncRNAs, especially CRNDE, but did not constitute full signature validation. The model may help prioritize lncRNAs for future studies of glioma migration, immune remodeling, and treatment-response hypotheses. Complete external validation and mechanistic experiments are required before the signature can be used for clinical decision-making or interpreted as evidence of a causal migrasome-lncRNA pathway.

## Ethics Statement

This study made no animal experimentation or personal human tissue involved and ethical approval was not applicable.

## Funding Sources

The current study received support from 10.13039/501100001809National Natural Science Foundation of China (30600637), National Key Clinical Specialty Construction Project Research Special Project (24090522), Teaching Reform and Innovation Project of Shanxi Medical University (XJ2025019), Key Country Science and Technology Cooperation Project of Shanxi Province (202204041101007)，Four “Batches” Innovation Project of Invigorating Medical through Science and Technology of Shanxi Province (2023XM006), Key Research and Development Project for Introducing High-level Scientific and Technological Talents of Luliang City (2023RC08, 2024RC19), Research Project Supported by Shanxi Scholarship Council of China (2022-188). Shanxi Province Overseas Students' Innovation and Entrepreneurship Start-up Support Program Project (SXLC-RY-2022), Shanxi Province Leading Talent Project in Science and Technology Innovation.

## Authorship

BY served as the main researcher, responsible for the overall research design and coordination, executing data collection and analysis, and playing a key role in constructing the prognostic model using machine learning algorithms such as LASSO regression analysis. RL participated in the literature review and data processing, identifying long non-coding RNAs (lncRNAs) related to migrasomes and conducting co-expression analysis, which ensured the scientific rigor of the models and methods employed. HBD, as the corresponding author, guided the research direction and methodology, coordinated team collaboration, provided academic support, and oversaw the writing and revision of research results to ensure the effectiveness and reliability of the findings.

## Conflict of Interest

The authors declare that they have no competing interests

## Data Availability

The data used in this study can be obtained from the TCGA official website, and the cleaned TPM data can also be requested from the corresponding author.

## Appendix A Supplementary material

Supplementary material

Fig.mentary Fig. 2. External survival analyses for available model lncRNAs. (A) Forest plot showing univariate Cox regression hazard ratios per 1 standard deviation increase for available-gene partial scores and individual detectable model lncRNAs in CGGA325, CGGA693, and GSE16011. (B) Kaplan-Meier survival curves for available-gene partial scores and individual detectable model lncRNAs. Because the external expression platforms did not cover all eight model lncRNAs, these analyses represent available-gene external support rather than full validation of the eight-lncRNA signature
