# Supplementary figures and images for: Associations between migrasome-related genes and long non-coding rnas in glioma and their prognostic relevance to the tumor microenvironment
Source: IBRO Neurosci Rep. 2026 Jun 24;21:279–90. doi: 10.1016/j.ibneur.2026.06.013 (PMC13356737; doi:10.1016/j.ibneur.2026.06.013)

## External survival associations for available model lncRNAs

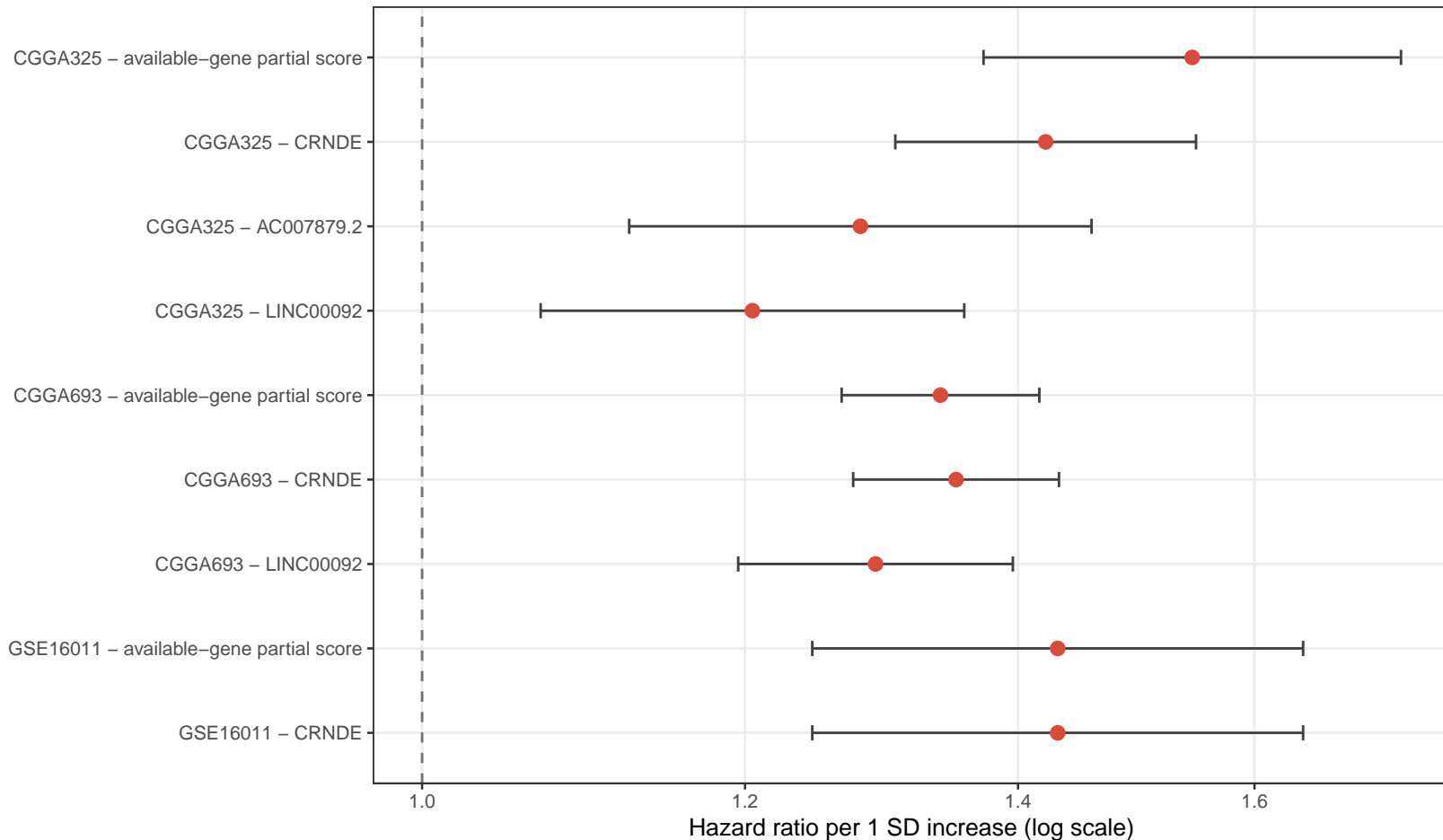

Supplement: Supplementary file 1 — Supplementary material [file mmc1.pdf]

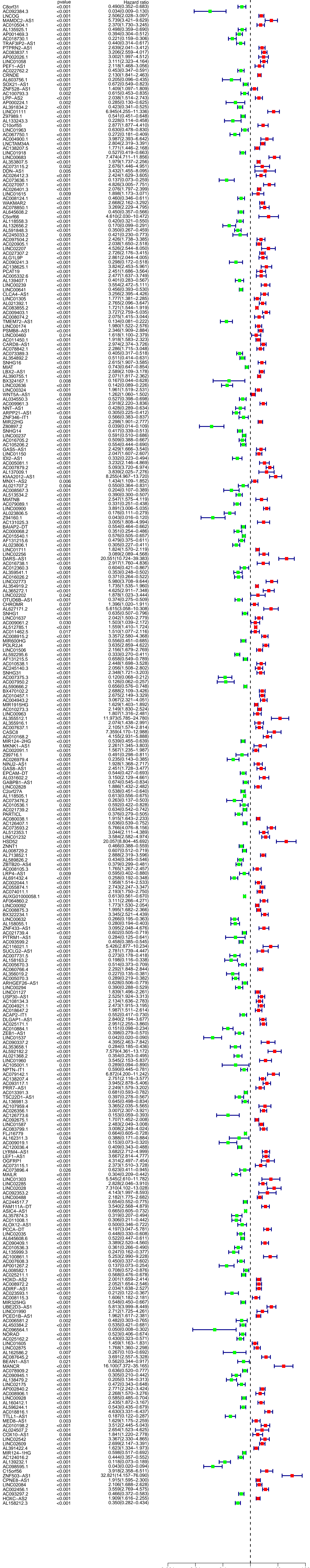

Supplement: Supplementary file 3 — Fig.mentary Fig. 2. External survival analyses for available model lncRNAs. (A) Forest plot showing univariate Cox regression hazard ratios per 1 standard deviation increase for available-gene partial scores and individual detectable model lncRNAs in CGGA325, CGGA693, and GSE16011. (B) Kaplan-Meier survival curves for available-gene partial scores and individual detectable model lncRNAs. Because the external expression platforms did not cover all eight model lncRNAs, these analyses represent available-gene external support rather than full validation of the eight-lncRNA signature [file mmc3.pdf]
