# Supplementary material for: Associations between migrasome-related genes and long non-coding rnas in glioma and their prognostic relevance to the tumor microenvironment
Source: IBRO Neurosci Rep. 2026 Jun 24;21:279–90. doi: 10.1016/j.ibneur.2026.06.013 (PMC13356737; doi:10.1016/j.ibneur.2026.06.013)
Supplement: Supplementary file 2 — Supplementary material [file mmc2.pdf]

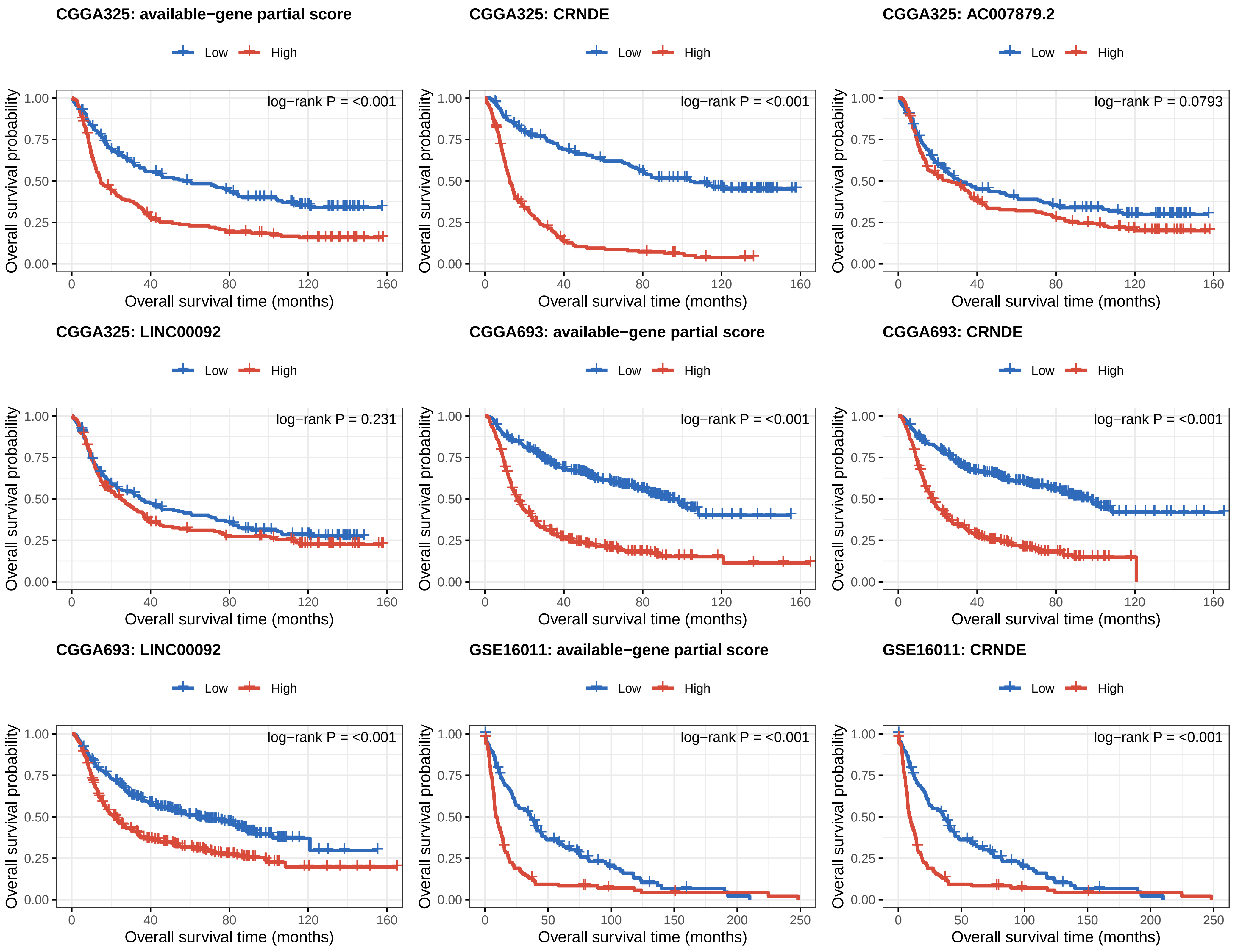
CGGA325: available−gene partial score
CGGA325: CRNDE
CGGA325: AC007879.2
CGGA325: LINC00092
CGGA693: available−gene partial score
CGGA693: CRNDE
CGGA693: LINC00092
GSE16011: available−gene partial score
GSE16011: CRNDE
Low
High
log−rank P = <0.001
log−rank P = 0.0793
log−rank P = 0.231
Overall survival probability
Overall survival time (months)
