# Supplementary material for: Associations between migrasome-related genes and long non-coding rnas in glioma and their prognostic relevance to the tumor microenvironment
Source: IBRO Neurosci Rep. 2026 Jun 24;21:279–90. doi: 10.1016/j.ibneur.2026.06.013 (PMC13356737; doi:10.1016/j.ibneur.2026.06.013)
Supplement: Supplementary file 4 — Supplementary material [file mmc4.docx]

Migrasome lncRNA cor pvalue

CPQ C8orf31 -0.468699404812245 8.69539853474363e-40

CPQ AC092384.3 -0.401657989315737 1.03213608600348e-28

ITGB1 LNCOG 0.462954701889995 9.72818238463284e-39

ITGA5 LNCOG 0.488416242725909 1.52910299062576e-43

EOGT LNCOG 0.433535043327253 1.13896527591675e-33

CPQ LNCOG 0.442400696085937 3.81030592326042e-35

PKD1 DLEU2L 0.499650638768458 8.56861676337819e-46

EPCIP MAMDC2-AS1 0.432669466892562 1.57850922195043e-33

CPQ AC010504.1 0.419197957088338 2.24927083639187e-31

CPQ AL135925.1 -0.443073463283065 2.9322341785873e-35

CPQ AP001469.3 -0.520065684367169 4.17470079280095e-50

ITGA5 AC018730.1 -0.408887801969429 8.62427610260348e-30

CPQ AC018730.1 -0.445430504607379 1.1657921715523e-35

CPQ TRAF3IP2-AS1 -0.411689393529315 3.24212315501213e-30

ITGB1 PTPRN2-AS1 0.506461687076484 3.36162502417623e-47

ITGA5 PTPRN2-AS1 0.417535853043967 4.08400591083493e-31

EOGT PTPRN2-AS1 0.446986323070018 6.31662929846545e-36

CPQ PTPRN2-AS1 0.494247546464908 1.06221225777047e-44

ITGA5 AC083837.1 0.452503000922102 7.00886422221837e-37

EOGT AC083837.1 0.466712534552496 2.01495889813896e-39

CPQ AC083837.1 0.51061022836654 4.51084691762781e-48

EPCIP AC083837.1 0.50425149933041 9.69163559963268e-47

PKD2 AC083837.1 0.432779850869733 1.51424007532885e-33

PKD2 AP002026.1 0.426442713478922 1.60643438832632e-32

PKD1 AP001432.1 0.504110766222364 1.03648696615771e-46

CPQ LINC01058 0.428550330013928 7.36471921379293e-33

PKD1 PEF1-AS1 0.430725689256137 3.27376049735372e-33

NDST1 NOP14-AS1 0.550422169193461 4.36805796006581e-57

ITGB1 AC022762.2 -0.414908414396618 1.04143925018421e-30

CPQ AC022762.2 -0.452983733940648 5.77577322795624e-37

PKD2 CRNDE 0.434130713097933 9.09352454912384e-34

PKD1 AL603756.1 0.574518109822387 3.68862842022862e-63

NDST1 SOX21-AS1 0.427035090003861 1.29093984105072e-32

PKD1 ZNF528-AS1 0.416502212906833 5.90816853215393e-31

PKD1 AC100793.3 0.544924967206089 9.0709052267352e-56

EPCIP LPP-AS2 0.445018925471218 1.37023553874805e-35

PKD1 AP000224.1 0.411142788753333 3.92685520716751e-30

NDST1 AL391834.2 0.406832045758074 1.75770817146795e-29

PKD1 AC114803.1 0.454554394143805 3.06270386338632e-37

CPQ LINC01111 0.415366415765829 8.85167885798078e-31

EPCIP LINC01111 0.413338251019306 1.81498546568605e-30

CPQ Z97989.1 -0.488012350908651 1.83583864881336e-43

PKD1 AL133243.3 0.400134666092653 1.72788218819128e-28

ITGB1 C10orf55 0.515317665310213 4.46471681325698e-49

ITGA5 C10orf55 0.467854660486392 1.24381963218562e-39

EOGT C10orf55 0.492539737914724 2.33203929873972e-44

PKD1 ATP2C2-AS1 0.455221212449532 2.33725218478023e-37

NDST1 LINC01963 0.472133969410523 2.00768150004293e-40

CPQ AC067750.1 -0.432262900300424 1.83941754138538e-33

PKD1 Z97832.2 0.468600207117335 9.06918987025984e-40

PKD1 AC090607.1 0.545582524891682 6.32928724636166e-56

PKD2 AC004900.1 0.405748463117213 2.55312566366457e-29

EOGT LNCTAM34A 0.461947416985239 1.47867851505039e-38

CPQ LNCTAM34A 0.607962404995944 1.79495953534642e-72

PKD2 LNCTAM34A 0.436132604636692 4.25300839044839e-34

EOGT AC138207.5 0.40943268688199 7.13509886017435e-30

CPQ AC138207.5 0.415459864508503 8.56259917213136e-31

CPQ LINC01918 -0.462065111201001 1.40817965024245e-38

CPQ LINC00683 0.49619481753578 4.30960770675889e-45

CPQ AL353807.5 0.460093932121819 3.18344203652517e-38

ITGB1 AC073115.2 0.478196835263918 1.44926554933413e-41

ITGA5 AC073115.2 0.451343365513991 1.11638508246824e-36

PKD1 DDN-AS1 0.488360700837314 1.56805593219075e-43

TSPAN4 AC012354.1 0.49622542028076 4.24874405917914e-45

PKD2 AC026412.3 0.482151059191922 2.53631885865524e-42

CPQ AC073636.1 -0.423420733840908 4.86806745062808e-32

PKD1 AL512791.1 0.554004036324402 5.87035359887219e-58

EOGT AC027097.1 0.406711977470184 1.83206444709496e-29

EOGT AC026401.3 0.413566622763811 1.67442477721124e-30

ITGB1 LINC01615 0.419923739248177 1.73169233692958e-31

ITGA5 LINC01615 0.551541033098774 2.33966405715165e-57

EOGT AP001189.1 0.43024843924474 3.91305054319083e-33

CPQ AC008124.1 -0.48453534541782 8.76966585683566e-43

EOGT WAKMAR2 0.504373948198741 9.14131397996129e-47

CPQ WAKMAR2 0.403627752315776 5.28021246695403e-29

PKD2 WAKMAR2 0.469815401512954 5.41044144466957e-40

ITGA5 AC078850.1 0.408166417325422 1.10784207688644e-29

CPQ AL645608.2 -0.430869693849307 3.10203988672775e-33

ITGB1 C5orf66 0.405515224588861 2.76624965199326e-29

PKD2 C5orf66 0.532149096288229 8.4492368920191e-53

PKD1 C5orf66 0.426581695134004 1.52616463033035e-32

CPQ AL118558.3 -0.40072929526634 1.4135165593861e-28

CPQ AL132656.2 -0.418833619330491 2.56418380583373e-31

CPQ AL591848.3 -0.400219811263183 1.67894656689921e-28

NDST1 AL591848.3 0.412751249678037 2.2321933746811e-30

PKD1 AC245033.2 0.447765263033002 4.64219426560743e-36

CPQ AC097504.2 0.491334480526765 4.05137590820838e-44

EPCIP AC020905.1 0.462754054070793 1.05755369185114e-38

NDST1 RAP2C-AS1 0.557207544042499 9.55218571908337e-59

ITGB1 LINC02207 0.421727321295282 9.01656911057703e-32

ITGA5 LINC02207 0.415505653699571 8.4243857549731e-31

EOGT LINC02207 0.406089914180977 2.27011925762174e-29

CPQ LINC02207 0.486170384204726 4.21298825049094e-43

ITGB1 AC027307.2 0.484538865987403 8.75587048891613e-43

ITGA5 AC027307.2 0.494912560867042 7.81088173743966e-45

EOGT AC027307.2 0.515526684867897 4.02558001958551e-49

CPQ AC027307.2 0.456657903897985 1.30280423196951e-37

PIGK AC027307.2 0.418714771874785 2.67606915247077e-31

TSPAN4 AC027307.2 0.429910619776301 4.43891203762342e-33

EPCIP AC027307.2 0.445978167715077 9.39930481636209e-36

PKD2 AC027307.2 0.481091526071426 4.05506830695654e-42

EPCIP ALG1L9P 0.445601035458337 1.09022851002774e-35

PKD2 ALG1L9P 0.465815819279409 2.93902656456747e-39

PKD1 AC090241.3 0.401115646710468 1.24033961376837e-28

PKD1 AC008079.1 0.5406452991903 9.25726537614111e-55

PKD1 AC025034.1 0.463148468822681 8.97391499521501e-39

EPCIP AC138625.1 0.431518033195675 2.43308920211263e-33

ITGA5 PCAT19 0.428551988219963 7.36018562349865e-33

EOGT PCAT19 0.476206937333909 3.45415189011124e-41

EPCIP AC005332.6 0.402842514772733 6.90121097173789e-29

PKD1 AL139407.1 0.443027569527674 2.98515666606411e-35

ITGB1 LINC00239 0.444893868569021 1.4391244935204e-35

ITGA5 LINC00239 0.561643193016282 7.48141298088953e-60

EOGT LINC00239 0.415770595319364 7.6669774070612e-31

CPQ LINC00239 0.465227232872395 3.76309018929281e-39

ITGA5 LINC00641 -0.442209888989879 4.10372190522619e-35

CPQ LINC00641 -0.473207370867233 1.26547286927687e-40

NDST1 LINC00641 0.42904780918967 6.12151440230604e-33

TSPAN4 LINC00641 -0.501215650727863 4.09793874159499e-46

PKD1 MEG3 0.406744669358456 1.81151577045307e-29

CPQ CLCA4-AS1 0.443042936127061 2.96733201792794e-35

PIGK CLCA4-AS1 0.447250855818188 5.68981041822125e-36

TSPAN4 LINC01305 0.434493112148708 7.92774689565566e-34

ITGB1 AL021392.1 0.451276858688335 1.14652886868211e-36

EOGT AL021392.1 0.412523186262646 2.41877747379033e-30

EPCIP AL021392.1 0.400096055337938 1.75053577377454e-28

PKD2 AL021392.1 0.414882157631404 1.0511835095246e-30

ITGB1 AC083855.2 0.552053202837638 1.75669542609585e-57

ITGA5 AC083855.2 0.617208441693933 3.01575621534122e-75

EOGT AC083855.2 0.559499966094006 2.57349831765345e-59

CPQ AC083855.2 0.517231359054308 1.72560201674271e-49

TSPAN4 AC083855.2 0.446751925736216 6.92897710983908e-36

EOGT AC009403.1 0.443783345943046 2.22272102745817e-35

EPCIP AC009403.1 0.430941956876102 3.01926338769009e-33

PKD2 AC009403.1 0.538301829230911 3.25674138609019e-54

PIGK AC008074.2 0.455181425737189 2.37529564276172e-37

PKD2 AC008074.2 0.418878587396413 2.52306931506168e-31

CPQ TMEM72-AS1 -0.496805763455882 3.24339404639092e-45

PKD1 LINC00174 0.473605351771951 1.06599401566013e-40

CPQ PSMB8-AS1 0.52085097110269 2.81084036777315e-50

EPCIP PSMB8-AS1 0.417609723205736 3.97744520903645e-31

ITGA5 LINC00460 0.563557061381325 2.46343126331991e-60

TSPAN4 AC011450.1 0.458183056138328 6.98389971721036e-38

ITGB1 CARD8-AS1 0.499077664399132 1.12144871628583e-45

ITGA5 CARD8-AS1 0.473560681149883 1.08672973034986e-40

EOGT CARD8-AS1 0.567088911369045 3.11119336897957e-61

CPQ CARD8-AS1 0.620193074032273 3.66330839063558e-76

PIGK CARD8-AS1 0.444784730400204 1.50204362440207e-35

PKD2 AC078842.1 0.408802097465561 8.88499714851102e-30

PKD1 AC009065.5 0.727929876164153 2.49128163819408e-117

CPQ AC073389.3 -0.519702362058118 5.01133972950357e-50

CPQ AL354892.2 -0.520608521993376 3.17630528372447e-50

ITGB1 SNHG16 0.500985566208426 4.56842034904202e-46

PKD1 MIAT 0.442347779236402 3.88952878057739e-35

ITGB1 LBX2-AS1 0.549997042303076 5.53402491202389e-57

ITGA5 LBX2-AS1 0.538982828433337 2.26190420187556e-54

EOGT LBX2-AS1 0.513958637363719 8.73765943133376e-49

CPQ LBX2-AS1 0.472387197556332 1.80082818043602e-40

TSPAN4 LBX2-AS1 0.565277260990792 9.02031207828284e-61

PKD2 LBX2-AS1 0.429620155266682 4.94665744282067e-33

CPQ AL390755.1 0.464254194420629 5.65640814273286e-39

EPCIP AL390755.1 0.407349236340955 1.47012557220107e-29

PKD1 BX324167.1 0.440728131951589 7.28919390782414e-35

CPQ LINC02636 -0.47302856854945 1.36675588680617e-40

CPQ LINC00324 0.453979901945414 3.86403993842928e-37

ITGB1 WNT5A-AS1 0.412071023339315 2.83555943969078e-30

CPQ AL034550.3 -0.404441620565276 3.9976974465804e-29

ITGB1 AC009961.3 0.576202781987126 1.32807955006181e-63

ITGA5 AC009961.3 0.495642012713752 5.57075217141927e-45

EOGT AC009961.3 0.543244843160619 2.2669056060023e-55

CPQ AC009961.3 0.536115377316513 1.04380858603235e-53

PIGK AC009961.3 0.451003410230165 1.27918449099821e-36

PKD2 AC009961.3 0.426450078545961 1.60207757408339e-32

NDST1 NNT-AS1 0.412517430580989 2.42368089802671e-30

CPQ ARPP21-AS1 -0.447585539475469 4.98444436662266e-36

PKD1 ZNF346-IT1 0.423255550044979 5.17052845698504e-32

ITGB1 MIR22HG 0.486270258017499 4.02796683556621e-43

ITGA5 MIR22HG 0.537641998113346 4.6326212039852e-54

EOGT MIR22HG 0.623851879311232 2.67905077122455e-77

CPQ MIR22HG 0.512167147022745 2.10757004477609e-48

TSPAN4 MIR22HG 0.410300301592947 5.27235444173719e-30

EPCIP MIR22HG 0.408992671620958 8.31554026897579e-30

PKD2 MIR22HG 0.454140834508121 3.62064224894664e-37

CPQ Z80897.2 -0.420179030456584 1.57927069121155e-31

ITGA5 SNHG14 -0.410657995432636 4.65287556031109e-30

CPQ SNHG14 -0.452050760964797 8.4058566351381e-37

NDST1 SNHG14 0.43895774964865 1.44268122971491e-34

CPQ LINC00237 -0.48736126272352 2.4637892844806e-43

NDST1 AC016705.2 0.437283044740922 2.74177784371233e-34

CPQ AC105206.2 -0.441488426932798 5.43016593512166e-35

PKD1 AC105206.2 0.429501673017524 5.16993040572666e-33

PKD1 AC137932.3 0.606405749047161 5.15521062607583e-72

PKD1 AC008147.2 0.419456121836365 2.04963103024397e-31

NDST1 MAGI2-AS3 0.406598798815646 1.90500228469147e-29

PKD2 MAGI2-AS3 0.41406308145095 1.40497763211975e-30

PKD1 AL117344.2 0.484024890806721 1.10162149911976e-42

PKD2 GAS5-AS1 0.411656381996417 3.27989216400881e-30

TSPAN4 AC026774.1 0.447934659806212 4.34098304374188e-36

CPQ LINC01150 0.489727925208453 8.43046038671639e-44

PKD1 IDI2-AS1 0.468300525698929 1.02980239377357e-39

TSPAN4 AC008443.3 0.569281012797172 8.50561827895046e-62

PKD1 AL049840.2 0.616290461744953 5.74125908493245e-75

CPQ AC005081.1 0.415051600204242 9.89854274908241e-31

PKD1 MUC20-OT1 0.461757576071326 1.59984272744082e-38

CPQ AC007879.2 0.435738098454584 4.9420819989643e-34

PKD1 AL121987.2 0.467915552587911 1.21217483865129e-39

EOGT AL137009.1 0.41015560240116 5.54555865712327e-30

PKD2 AL137009.1 0.462702790792467 1.08035211514037e-38

EOGT KIAA2012-AS1 0.429600833540678 4.9824047762996e-33

CPQ KIAA2012-AS1 0.458290854277894 6.68201242571802e-38

PKD2 KIAA2012-AS1 0.404275878794236 4.23102929669365e-29

PKD1 HCG25 0.49669670696972 3.41233953685003e-45

TSPAN4 MNX1-AS2 0.420915067837468 1.21031414624315e-31

PKD1 AL021707.2 0.47769364898073 1.80619048099688e-41

CPQ AC008567.3 -0.409590102588314 6.75443329217369e-30

CPQ AL513534.2 -0.456715294240563 1.27266655650288e-37

PKD2 MIATNB 0.424638376726892 3.11842926583038e-32

CPQ AC079089.1 -0.52885075743582 4.70916988636455e-52

ITGB1 LINC00900 0.420546442259372 1.38295604323394e-31

ITGA5 LINC00900 0.413393233154917 1.78011192716603e-30

EOGT LINC00900 0.498667843381101 1.35904581118721e-45

CPQ LINC00900 0.706195411549395 1.61982225947232e-107

PIGK LINC00900 0.40060405034118 1.47463674950195e-28

EPCIP LINC00900 0.505969793206738 4.25778282374336e-47

PKD2 LINC00900 0.473486533216318 1.12203572816917e-40

CPQ AL023806.5 -0.459176935762813 4.64411550303077e-38

CPQ Z94160.1 -0.408617718897376 9.47264433341012e-30

PKD1 AC074117.1 0.456956732863512 1.15327194026298e-37

PKD1 AC025171.2 0.437675605451676 2.35941885933265e-34

EOGT AC131025.3 0.404250240174154 4.2683080635274e-29

PKD2 AC131025.3 0.412809482480652 2.18688249285444e-30

PKD1 AC007066.2 0.411215005673314 3.82877077070286e-30

PKD1 CTC-338M12.4 0.511554820167631 2.84419987430915e-48

NDST1 BAIAP2-DT 0.457809924833072 8.1371369085168e-38

CPQ AC000068.2 -0.496877306089489 3.13710183720459e-45

PIGK AC000068.2 -0.458929278705913 5.14175568268977e-38

ITGB1 AC015540.1 -0.413437657731564 1.75242015223407e-30

EOGT AC015540.1 -0.4247994865796 2.93957531159858e-32

PKD2 AC015540.1 -0.407105225806931 1.59948238054607e-29

CPQ AF131215.6 -0.463942662464792 6.44300061850388e-39

TSPAN4 AL136981.4 0.483709473822654 1.26809817798398e-42

CPQ AL023806.1 -0.423467485165648 4.78569256323177e-32

PKD1 AL049552.1 0.593476948605781 2.64095847052778e-68

ITGA5 LINC01711 0.401600235630629 1.05254873090439e-28

ITGB1 LINC02256 0.437188391867678 2.84280354588958e-34

EOGT LINC02256 0.528079998668293 7.01645473474037e-52

CPQ LINC02256 0.463703481680241 7.11972629018191e-39

PIGK LINC02256 0.415584944672826 8.19025520511866e-31

PKD2 LINC02256 0.443114242769796 2.88599035912106e-35

ITGA5 DARS-AS1 0.427423587588302 1.11822204362407e-32

EOGT DARS-AS1 0.462023225001462 1.43287883433799e-38

CPQ DARS-AS1 0.402421084740379 7.96527024744435e-29

EOGT AC016738.1 0.429436306853322 5.29735442200108e-33

PKD2 AC016738.1 0.473257296469276 1.23854741936393e-40

PKD1 AC012360.3 0.406974464316414 1.67337693830606e-29

PKD1 ATP1B3-AS1 0.54324173797404 2.27073541631844e-55

CPQ AL359541.1 -0.423408963729626 4.88902655308308e-32

PKD1 CADM3-AS1 0.499250398241604 1.03411896413573e-45

PKD1 AC016026.2 0.50210654250546 2.68821617293287e-46

ITGB1 LINC02773 0.487089326467169 2.78539475003835e-43

ITGA5 LINC02773 0.468165907007527 1.09024902980642e-39

EOGT LINC02773 0.487146537339244 2.71444366657237e-43

CPQ LINC02773 0.480841902237953 4.52801541331803e-42

EPCIP LINC02773 0.515746312658821 3.61033281425596e-49

PKD2 LINC02773 0.438833832070594 1.51306567476809e-34

ITGB1 AL354919.2 0.440843726089134 6.97043283342748e-35

ITGA5 AL354919.2 0.507335077039448 2.20750884151206e-47

EOGT AL365272.1 0.420670070495771 1.32249686637069e-31

PKD2 AL365272.1 0.450252283072046 1.72710981039677e-36

ITGB1 LINC02202 0.430730139520786 3.26831526576085e-33

ITGA5 LINC02202 0.405386718740129 2.8911037808023e-29

EOGT LINC02202 0.419890550492953 1.75254909583979e-31

ITGA5 OTUD6B-AS1 -0.400317206214529 1.62465101429307e-28

NDST1 OTUD6B-AS1 0.419612793374919 1.93714340263215e-31

TSPAN4 OTUD6B-AS1 -0.418968690523158 2.44264285264597e-31

PKD2 CHROMR 0.408443711894818 1.00624714916885e-29

PKD1 CHROMR 0.454137567593279 3.62542861819631e-37

ITGB1 AL627171.2 0.43437045473386 8.30470296372892e-34

CPQ AL627171.2 0.419751344619061 1.84277500909935e-31

PIGK AL627171.2 0.422562282148882 6.65649918737864e-32

CPQ SNHG1 -0.460068176555338 3.21743786352342e-38

CPQ LINC01637 0.472612157304094 1.63488839509629e-40

PKD1 AC009061.2 0.412727205138167 2.25117250366234e-30

CPQ AL512785.1 0.419377584044519 2.10843100094482e-31

ITGB1 AC011462.5 0.40367689880045 5.19234703304707e-29

NDST1 FGD5-AS1 0.418912403049001 2.49258235012391e-31

CPQ AC008915.2 0.431207273122094 2.73369091790131e-33

PKD1 MIR600HG 0.42602090158743 1.87656630164092e-32

TSPAN4 POLR2J4 0.425790317039701 2.0427872108838e-32

PKD2 POLR2J4 0.423312883993792 5.06349385677941e-32

CPQ LINC01506 0.497089986022242 2.84111690842363e-45

ITGB1 AL592295.6 -0.42421953469535 3.63544440718189e-32

ITGA5 AL592295.6 -0.512293046271488 1.98145198294734e-48

EOGT AL592295.6 -0.443457086503348 2.52473603557866e-35

CPQ AL592295.6 -0.536735823438569 7.50683400072996e-54

TSPAN4 AL592295.6 -0.49361374567306 1.42292787148404e-44

NDST1 AF131215.5 0.503862467973506 1.16678329729072e-46

PKD2 AC010538.1 0.406978282969248 1.67117181332696e-29

PKD1 AC245140.3 0.434874466581732 6.8607710378701e-34

PIGK SNHG31 0.466587031268069 2.12441796435969e-39

EPCIP SNHG31 0.418511441086061 2.8788124807669e-31

PKD2 SNHG31 0.409370044700669 7.29242333282737e-30

CPQ AC007375.3 -0.478620397185951 1.20375817235961e-41

PKD1 AC007375.3 0.401089960414282 1.25117079908728e-28

CPQ AC007950.2 -0.467273921494555 1.58995809117002e-39

CPQ AL590666.2 -0.487598521141471 2.21348202105371e-43

PKD1 AL590666.2 0.421543665681485 9.63787052742287e-32

CPQ BX470102.2 0.486078670143231 4.39031235717154e-43

EPCIP BX470102.2 0.544498360753307 1.14516045283227e-55

PKD2 BX470102.2 0.403816784614094 4.95010115893611e-29

CPQ AC010457.1 0.407807486498997 1.25455851663596e-29

PKD1 AC092645.2 0.416392406013197 6.14407233305378e-31

PIGK AC004943.2 0.471310472839267 2.85751913799752e-40

EPCIP AC004943.2 0.400183986160779 1.69936692067129e-28

PKD2 AC004943.2 0.465102642116713 3.96496694879839e-39

TSPAN4 MIR1915HG 0.521301965095939 2.23858493797514e-50

PIGK AC010273.3 0.400027962700046 1.79120556562692e-28

EPCIP AC010273.3 0.416399967510496 6.12753265460029e-31

PKD2 LINC00963 0.406074096010695 2.28251537251257e-29

ITGB1 AL355512.1 0.466098000078168 2.61016350439273e-39

ITGA5 AL355512.1 0.462224851865734 1.31780088064172e-38

ITGA5 AL355916.1 0.434517426833139 7.85506146541557e-34

PKD2 AC007637.1 0.417781372842994 3.74035192257228e-31

ITGA5 CASC8 0.465895282837523 2.84246832663515e-39

EOGT AC010168.2 0.43548793314381 5.43524310178553e-34

PKD2 AC010168.2 0.468435044570194 9.72723552477075e-40

CPQ MIR124-2HG -0.443934720422214 2.09504153949472e-35

PKD2 MKNK1-AS1 0.419863399416103 1.76979652149505e-31

CPQ AC002091.1 0.441460743659734 5.48876396454809e-35

PKD1 Z99716.1 0.505515370064626 5.29481538226185e-47

PKD1 AC026979.4 0.474576192700464 7.00827611030008e-41

CPQ NINJ2-AS1 0.57047093900052 4.18986837059405e-62

PKD1 AC009955.4 0.584932328309147 6.06166260303502e-66

PKD2 GAS8-AS1 0.457628838776891 8.76302686583224e-38

PKD1 GAS8-AS1 0.497583053349053 2.25731246715693e-45

CPQ EPCAM-DT -0.435639336041877 5.13124085225188e-34

ITGB1 AL031602.2 0.407767889287084 1.27187846811398e-29

EOGT AL031602.2 0.454758099050491 2.82012552196001e-37

CPQ AL031602.2 0.400496251863798 1.52933291327887e-28

PKD2 AL031602.2 0.416883186655812 5.15716233517864e-31

PKD1 GABPB1-AS1 0.485251959367619 6.36279446591352e-43

CPQ LINC02828 0.409376504640056 7.27604175911971e-30

PKD1 LINC01126 0.546811148799015 3.22397559668303e-56

CPQ C2orf27A -0.517407150940121 1.58082588434105e-49

ITGA5 AL118505.1 -0.406405597887053 2.03621189652734e-29

EOGT AL118505.1 -0.448665238908075 3.24896381757514e-36

CPQ AL118505.1 -0.592327984667922 5.53768523737295e-68

EPCIP AL118505.1 -0.420899554005772 1.21712937824397e-31

NDST1 AC073476.2 0.492327125274439 2.57109947578546e-44

PKD1 AC073476.2 0.445848923887998 9.88968106999605e-36

CPQ AC010536.1 -0.453998910607091 3.83446621802203e-37

PIGK GNG12-AS1 0.407873122917271 1.22636190835891e-29

CPQ AC021739.2 -0.418081118765462 3.35942206899706e-31

ITGA5 PARTICL -0.416520004324284 5.87080028196028e-31

CPQ PARTICL -0.42262733148795 6.50073644337414e-32

NDST1 AC019080.1 0.43788539520361 2.17726712151015e-34

PKD1 AC019080.1 0.446099204778711 8.96196065178072e-36

PKD1 LINC01534 0.425070603017033 2.66125898601341e-32

ITGA5 AC080038.1 0.417354401721746 4.35791228261101e-31

EOGT AC080038.1 0.497106680206168 2.81909337890207e-45

TSPAN4 AC080038.1 0.508440190210917 1.2943069902359e-47

EPCIP AC080038.1 0.42197272369624 8.2479083366531e-32

PKD2 AC080038.1 0.464635228907059 4.82278939001055e-39

CPQ AC126407.1 -0.450827359528976 1.37253262778329e-36

EOGT AC073593.2 0.432007348310642 2.02484355511105e-33

CPQ AC073593.2 0.561510639758833 8.07757467373408e-60

PIGK AC073593.2 0.43270496766098 1.5575499833082e-33

EPCIP AC073593.2 0.55595931737279 1.94258438414577e-58

PKD2 AC073593.2 0.513704102170688 9.90520500794196e-49

PKD1 TH2LCRR 0.456077163972731 1.65054193434527e-37

NDST1 ZFAS1 -0.404940192466376 3.36990561638965e-29

PIGK AL512353.1 0.423995890624972 3.94541164791035e-32

CPQ LINC01232 0.530461653700394 2.03970119637785e-52

EPCIP LINC01232 0.447371133297753 5.42560049560808e-36

EOGT HSD52 0.421499226056005 9.79447215599158e-32

PIGK HSD52 0.407308863669989 1.49078943028873e-29

ITGB1 ZNNT1 -0.444515000555396 1.66948894575154e-35

ITGA5 ZNNT1 -0.404150886363263 4.41586635851274e-29

EOGT ZNNT1 -0.439639766109124 1.1095790011974e-34

CPQ ZNNT1 -0.420517711555408 1.39739405802254e-31

TSPAN4 ZNNT1 -0.446788061033202 6.83087501607121e-36

EPCIP ZNNT1 -0.40346044175488 5.59051630670168e-29

ITGB1 AL008729.2 -0.425353496533301 2.39867454877082e-32

CPQ AL713852.1 0.490339824955421 6.38024006814165e-44

CPQ AL589826.2 -0.440462671313226 8.07696991883814e-35

NDST1 ZBTB20-AS4 0.403266810434154 5.97222783824215e-29

EOGT AC008105.3 0.451213628289754 1.1759356517309e-36

PKD1 LRP4-AS1 0.411151088014148 3.91545786004375e-30

ITGB1 AL691432.4 -0.407404014824456 1.44254121112736e-29

CPQ AL691432.4 -0.413777660724132 1.55414396980491e-30

NDST1 AC002044.1 -0.409063469827893 8.113325107533e-30

CPQ AC055874.1 0.454641967775754 2.9559812546857e-37

EPCIP AC055874.1 0.426864057000198 1.37512340769038e-32

ITGB1 AC074011.1 0.461343892932117 1.89904786144178e-38

EOGT AC074011.1 0.474592128186798 6.96011795874945e-41

PKD2 AC074011.1 0.403823974349745 4.93795735264413e-29

CPQ AUXG01000058.1 -0.475596692811563 4.50318244184203e-41

EPCIP AF064860.2 0.509447769576442 7.94134046921425e-48

CPQ LINC00092 0.55142499484779 2.49643813262247e-57

CPQ AC008875.3 0.457432520941937 9.49558739285463e-38

EPCIP AC008875.3 0.490914599853242 4.90841870792753e-44

PKD2 AC008875.3 0.445711891638897 1.04373306870978e-35

ITGB1 BX322234.1 0.403744293160446 5.07420673365876e-29

EOGT BX322234.1 0.541408529490372 6.13240905390856e-55

CPQ BX322234.1 0.573228204383715 8.03181430825586e-63

PIGK BX322234.1 0.446717954436744 7.02247779616815e-36

EPCIP BX322234.1 0.473498283206125 1.11636594294804e-40

PKD2 BX322234.1 0.536340563522076 9.26179111120569e-54

CPQ LINC00632 -0.506598184465438 3.14803278460567e-47

CPQ AL158055.1 -0.475869466829188 4.0000428423349e-41

EOGT ZNF433-AS1 0.404653867205056 3.71741451226893e-29

TSPAN4 ZNF433-AS1 0.406140396497061 2.23100225554667e-29

PKD2 ZNF433-AS1 0.417041875620768 4.87298785790839e-31

CPQ AC021739.4 -0.4066358667225 1.88080275227701e-29

PKD1 PITRM1-AS1 0.533015527400293 5.36353147872148e-53

CPQ AC093599.2 -0.424826798578006 2.9102795172403e-32

ITGA5 AC116021.1 0.417888411031037 3.59964834752663e-31

EPCIP SUCLG2-AS1 0.46873131300082 8.57844448567698e-40

PKD2 SUCLG2-AS1 0.493329239099339 1.62215589508528e-44

PKD1 AL590666.1 0.452389975562898 7.33476644714068e-37

CPQ AC007731.5 -0.415995261127799 7.07784632551559e-31

CPQ AL158163.2 -0.417432378098804 4.23804333529182e-31

NDST1 AC005670.3 0.618395150069053 1.30785379357349e-75

EOGT AC060766.4 0.440098186178061 9.29776937043212e-35

CPQ AC060766.4 0.484967975780997 7.22609819035078e-43

PKD2 AC060766.4 0.452386501801419 7.34501736433364e-37

PKD1 AL356019.2 0.443163522082951 2.83107170539919e-35

ITGB1 AC005070.3 -0.415247330565936 9.2341101036667e-31

ITGA5 AC005070.3 -0.47892279615221 1.05419303413178e-41

CPQ AC005070.3 -0.481378575493343 3.57154852260695e-42

TSPAN4 AC005070.3 -0.470418640589638 4.18344433615361e-40

ITGB1 ARHGEF26-AS1 -0.401413505087271 1.12132146441475e-28

ITGA5 ARHGEF26-AS1 -0.406487281272893 1.97968022394816e-29

CPQ LINC00294 -0.421711222584112 9.06940519673305e-32

EPCIP LINC01127 0.414026847898982 1.42309810134651e-30

PKD1 AP000487.1 0.409005889788468 8.27741107781737e-30

CPQ USP30-AS1 0.429266745541135 5.64258759910528e-33

EOGT AC108134.3 0.468082428190974 1.1294876682895e-39

CPQ AC004921.1 0.560724936742813 1.27157270782435e-59

PIGK AC018647.2 0.452874052238219 6.03664347240875e-37

PKD2 AC018647.2 0.414851052951177 1.06284380918229e-30

PKD1 ACAP2-IT1 0.451533591341954 1.03443715134676e-36

CPQ DLGAP1-AS1 0.427471124743782 1.09872703537678e-32

PKD1 THUMPD3-AS1 0.430226382999511 3.94541507366958e-33

EPCIP AC025171.1 0.43801575838368 2.07117467288892e-34

PKD2 AC025171.1 0.517708896439469 1.35990403437478e-49

ITGB1 AC010884.1 -0.452986117303244 5.77023048503418e-37

ITGA5 AC010884.1 -0.404662827823394 3.70601961348426e-29

EOGT AC010884.1 -0.415197641185762 9.39848095081168e-31

EPCIP AC010884.1 -0.404686419146837 3.67618448042441e-29

CPQ ZEB1-AS1 -0.427530193971257 1.07497140506399e-32

CPQ LINC01537 -0.429443695284893 5.28279690102037e-33

ITGB1 AC090337.2 0.461643311022087 1.67746704449797e-38

EOGT AC090337.2 0.448934466644659 2.91939578033359e-36

PKD2 AC090337.2 0.45174895943492 9.48839589089897e-37

PKD1 AL353658.1 0.554621014043661 4.14435405121395e-58

EPCIP AL592182.2 0.439201791652566 1.31342065639808e-34

CPQ AL021368.2 -0.467992645509261 1.17325423659056e-39

TSPAN4 LINC01960 0.402165820633043 8.68715922725234e-29

PKD1 AC105001.1 0.458249014442366 6.79761574153188e-38

PKD1 NPTN-IT1 0.649097480773988 1.44640622967425e-85

EOGT AC079142.1 0.471796108730432 2.32080296951722e-40

CPQ AC079142.1 0.502140193177007 2.64568120625419e-46

PKD2 AC079142.1 0.480089881019943 6.30961501032709e-42

PKD1 AP000941.1 0.438869580481246 1.49241856893636e-34

ITGB1 AC138207.4 0.498965602003771 1.18198156845141e-45

ITGA5 AC138207.4 0.482571660185098 2.10429128253741e-42

EOGT AC138207.4 0.564340768546205 1.55982253132373e-60

CPQ AC138207.4 0.426745944933852 1.4364120441386e-32

PKD2 AC138207.4 0.439706327107162 1.08147726776919e-34

CPQ AC093117.1 0.408730462373059 9.10889462386171e-30

PIGK AC093117.1 0.402183694576932 8.63456838605569e-29

EPCIP AC093117.1 0.481263576707247 3.75797488770745e-42

PKD2 PRR7-AS1 0.465385515393859 3.52126758585877e-39

PKD1 PRR7-AS1 0.44160298555188 5.19419863411654e-35

CPQ AC013391.3 -0.419269708665162 2.19192835219551e-31

CPQ TSC22D1-AS1 -0.453774067155026 4.19913080295341e-37

PKD1 TSC22D1-AS1 0.440802484377164 7.08254253572519e-35

TSPAN4 AL136981.3 0.488526156439258 1.45482697346446e-43

ITGA5 AC107959.4 0.407487499664209 1.40148415316899e-29

ITGB1 AC026356.1 0.486080890026355 4.38593408353428e-43

EPCIP AC026356.1 0.462032405161185 1.42742902395092e-38

PKD2 AC026356.1 0.493188050148799 1.73107013281361e-44

PKD1 AC126773.6 0.41506610471722 9.84772135029682e-31

PIGK AC092675.1 0.404162271096066 4.39870502911466e-29

EPCIP AC092675.1 0.414302570386408 1.29082414194826e-30

PKD1 AC079781.5 0.484237679615352 1.00175810882001e-42

PKD1 AC132872.5 0.549981155507627 5.58313425600509e-57

ITGB1 LINC01587 0.422854285880261 5.98503750832203e-32

ITGA5 LINC01587 0.402048204751045 9.04121929116743e-29

ITGB1 AC083799.1 0.429647161166656 4.89711906541872e-33

EOGT AC083799.1 0.448911713719323 2.94591571744384e-36

CPQ AC083799.1 0.515928635345861 3.29815290332237e-49

EOGT FLJ16779 -0.418537242296714 2.85226611180545e-31

CPQ FLJ16779 -0.427355691155739 1.14666219264886e-32

NDST1 AL162311.3 0.456565880430686 1.35261314076326e-37

PKD1 AC009019.1 0.42489285440864 2.84061626093718e-32

ITGB1 AC120036.4 -0.465918767986465 2.8145382574112e-39

ITGA5 AC120036.4 -0.489355459919238 9.98603901668065e-44

EOGT AC120036.4 -0.466586825380007 2.12460229289792e-39

CPQ AC120036.4 -0.57464735699103 3.41130124854119e-63

TSPAN4 AC120036.4 -0.419945755139949 1.7179927780917e-31

EPCIP AC120036.4 -0.415935216248417 7.23075915148342e-31

ITGB1 LYRM4-AS1 0.569251988062371 8.65348253507614e-62

EOGT LYRM4-AS1 0.516045305588163 3.11261115619469e-49

CPQ LYRM4-AS1 0.42919696790913 5.79105026294487e-33

PIGK LYRM4-AS1 0.506105394033273 3.98939409737115e-47

EPCIP LYRM4-AS1 0.478162975494317 1.47091305570431e-41

PKD2 LYRM4-AS1 0.609491739096693 6.33030442462523e-73

EOGT LEF1-AS1 0.438801468538361 1.53200187215409e-34

CPQ LEF1-AS1 0.483765884263523 1.23659226676129e-42

TSPAN4 LEF1-AS1 0.445268398273314 1.24242925960703e-35

ITGB1 OGFRP1 0.503423724100402 1.43799504023239e-46

ITGA5 OGFRP1 0.404394177813375 4.06315419003075e-29

EOGT OGFRP1 0.418646140679442 2.74286873846515e-31

PKD2 OGFRP1 0.428109004606525 8.67516906709645e-33

ITGB1 AC073115.1 0.41251623372203 2.42470177101625e-30

ITGA5 AC073115.1 0.433955224887081 9.71758994891709e-34

PKD1 AC073896.4 0.439537872798567 1.15400614740953e-34

ITGA5 MAILR -0.406931923856404 1.6981380928608e-29

EOGT MAILR -0.409882245806668 6.10049375102511e-30

CPQ MAILR -0.433366844715296 1.21362582952295e-33

ITGB1 LINC01303 0.439047154634897 1.39392489684739e-34

ITGA5 LINC01303 0.430656322582216 3.35980737603167e-33

EOGT LINC01303 0.408039356032305 1.15772646986461e-29

ITGA5 LINC02285 0.439547153266428 1.14988776609422e-34

CPQ LINC02285 0.462474693906136 1.18785031410079e-38

CPQ LINC02028 0.42174492523433 8.95914187563852e-32

ITGB1 AC092353.2 0.412645100454552 2.31719301398264e-30

ITGA5 AC092353.2 0.406638144741928 1.87932553073283e-29

CPQ LINC00488 0.419520252893038 2.00282433533593e-31

EPCIP LINC00488 0.438382241287853 1.79960109787503e-34

NDST1 AC244517.7 0.443964108147257 2.07111040828436e-35

EPCIP FAM111A-DT 0.489456432987777 9.5381781170723e-44

PKD2 FAM111A-DT 0.491286811651632 4.14066231784611e-44

CPQ ASIC4-AS1 -0.481889588224181 2.84814734835279e-42

PIGK ASIC4-AS1 -0.410755219101502 4.4973381035644e-30

PKD1 AL357874.3 0.409013161533753 8.25650883958553e-30

PKD1 AP002340.1 0.410594227578706 4.75777148213634e-30

CPQ AC011008.1 -0.44189756020412 4.63309261206138e-35

PKD1 AL136379.1 0.418813971759457 2.58235535403165e-31

CPQ ALOX12-AS1 -0.464067649144613 6.1151671965366e-39

ITGA5 PCCA-DT 0.400594693850472 1.47930639929845e-28

EOGT PCCA-DT 0.40879543509942 8.90558860312916e-30

CPQ PCCA-DT 0.469276822270977 6.80409760704282e-40

TSPAN4 PCCA-DT 0.402626260779978 7.42835355625601e-29

NDST1 LINC02035 0.537072603529287 6.27512133645745e-54

PKD1 LINC02035 0.422053621814753 8.009031675792e-32

CPQ AL645608.6 -0.50734047173488 2.2017735326502e-47

ITGB1 AC090409.1 0.454279580136507 3.4230482429594e-37

ITGA5 AC090409.1 0.54858165695194 1.21360348810999e-56

EOGT AC090409.1 0.485467997495019 5.77534094458069e-43

CPQ AC090409.1 0.425526326352113 2.25106161600884e-32

TSPAN4 AC090409.1 0.457920347320198 7.77746230608359e-38

CPQ AC010536.3 -0.503093796863719 1.6823864406528e-46

CPQ AL135999.3 -0.410348733812945 5.18392168740321e-30

ITGB1 AC100861.1 0.410572866261955 4.79343111080544e-30

ITGA5 AC100861.1 0.480013973857369 6.5242043758224e-42

EOGT AC100861.1 0.425055141057157 2.67640466505393e-32

CPQ AC100861.1 0.52766732304339 8.68268913802837e-52

PKD1 AC009065.2 0.755142351225108 4.77268054600366e-131

CPQ AC007608.3 -0.418952788020613 2.45665057462305e-31

ITGB1 AP001267.2 -0.453018682064453 5.69502430763434e-37

CPQ AP001267.2 -0.402221934980262 8.5231091173601e-29

PIGK AL008582.1 -0.44001869745549 9.58739261258042e-35

CPQ AC025211.1 -0.434411148697893 8.17771172696647e-34

TSPAN4 HOXD-AS2 0.40715887092969 1.57011020367464e-29

ITGB1 AC008972.2 0.405017646874239 3.28156122544595e-29

EOGT AC008972.2 0.425133439522848 2.60057679354202e-32

PKD1 AC023906.4 0.426212878342442 1.74845497319445e-32

TSPAN4 ADIRF-AS1 0.564823691623344 1.17629339293554e-60

CPQ AC023593.1 -0.401140482877285 1.22995518294728e-28

PKD1 AC008115.3 0.490810505963928 5.14736004059382e-44

CPQ MIR325HG -0.443920273236186 2.10690654809931e-35

CPQ UBE2D3-AS1 0.445577619728915 1.10030921770545e-35

PIGK UBE2D3-AS1 0.406432661256859 2.01730695113509e-29

CPQ LINC01990 0.428297214493253 8.09021112719898e-33

PIGK LINC01990 0.421712102827995 9.06650831877242e-32

PKD1 AL683813.1 0.493936370666047 1.22626274973304e-44

ITGB1 PCED1B-AS1 0.406072950421896 2.28341572636219e-29

ITGA5 PCED1B-AS1 0.458457536664234 6.2405120183878e-38

CPQ PCED1B-AS1 0.55266431745833 1.24713492206434e-57

PKD1 AC006581.2 0.492140709275015 2.80062680768067e-44

CPQ AL450384.2 -0.4219946228713 8.18255616652806e-32

PKD1 AC096564.1 0.450688026555911 1.45117454370275e-36

NDST1 NORAD 0.52287947877751 1.00692124699387e-50

ITGA5 AC091182.2 0.429073503714219 6.06328427366709e-33

CPQ AC025162.2 -0.443208334632641 2.78203116068045e-35

ITGA5 LINC01605 0.529676713369711 3.06813005295385e-52

TSPAN4 LINC02875 0.425354024541251 2.39820928679354e-32

PKD1 AL162586.2 0.448284494485138 3.77891143978306e-36

ITGB1 AC087645.2 0.435870505728039 4.69928711255704e-34

EOGT AC087645.2 0.430831044382157 3.14723154288375e-33

CPQ AC087645.2 0.64074245866147 9.60776707151056e-83

PIGK AC087645.2 0.444977340535894 1.39277224178602e-35

EPCIP AC087645.2 0.490143129368687 6.97852836941173e-44

PKD2 AC087645.2 0.465207560160811 3.79427452761704e-39

PKD1 BEAN1-AS1 0.506812584508104 2.83943379597981e-47

ITGB1 MANCR 0.547280305950573 2.4900126862753e-56

ITGA5 MANCR 0.601710192182307 1.19981084341935e-70

EOGT MANCR 0.470471040850187 4.09091806488371e-40

CPQ MANCR 0.411078961601083 4.01561380565751e-30

PIGK AC078909.2 -0.405498932443486 2.78177837538216e-29

CPQ AC090945.1 -0.416655849108291 5.59309193116807e-31

CPQ AL138479.2 -0.400754947445037 1.40131109902107e-28

CPQ LINC02175 -0.411963230287796 2.94498171776573e-30

PKD1 LINC02175 0.50663055698591 3.09938659083922e-47

CPQ AP002840.2 0.602843855514217 5.63878281940519e-71

EPCIP AP002840.2 0.433244279936719 1.27106632298018e-33

ITGB1 AC008906.1 0.428688087879977 6.99736139946808e-33

EOGT AC008906.1 0.483295220611726 1.52520690881738e-42

PKD2 AC008906.1 0.558600298818753 4.31107628932548e-59

CPQ LINC00928 -0.424060682804311 3.85301087430627e-32

CPQ AL160412.1 0.430451818676931 3.62674869067445e-33

PIGK AL160412.1 0.501208660963477 4.11149630390897e-46

EPCIP AL160412.1 0.540613416836282 9.41767914056355e-55

PKD2 AL160412.1 0.455324467447973 2.24131743871536e-37

NDST1 AL596244.1 0.462476359606886 1.18702806948876e-38

PKD1 CACNA1C-AS1 0.410863710244155 4.32984799876581e-30

CPQ AC018816.1 0.427399245311208 1.12833712882352e-32

ITGA5 TTLL1-AS1 -0.412735194628704 2.24484847879901e-30

CPQ TTLL1-AS1 -0.448515058421632 3.44856651356087e-36

PKD1 MED8-AS1 0.403947828554267 4.73333259303059e-29

EOGT AC010198.2 0.413034714642294 2.0200448160121e-30

PKD2 AC010198.2 0.467998559776931 1.17032012789736e-39

ITGB1 AL024507.2 0.45858025074973 5.93408393830269e-38

PKD1 COX10-AS1 0.426969781444551 1.32246475913699e-32

PKD1 AL928654.2 0.560527512131124 1.42486585676384e-59

ITGB1 LINC02542 0.438785413334639 1.54148291238344e-34

CPQ LINC02542 0.464002229666317 6.28463986604941e-39

CPQ LINC02609 0.569276707570668 8.52739120075278e-62

ITGB1 AL391422.4 0.402964576634585 6.62020435495279e-29

EPCIP AL391422.4 0.447981936397835 4.26043431590788e-36

CPQ MIR124-1HG -0.40713883931547 1.58101504917198e-29

PKD1 MIR124-1HG 0.478043870486155 1.54964136604324e-41

ITGB1 AC124016.2 -0.499872664006852 7.71906931615134e-46

ITGA5 AC124016.2 -0.404220161800266 4.31245697695165e-29

ITGB1 AL139232.1 -0.426444099348091 1.60561367997848e-32

ITGA5 AL139232.1 -0.438386981383835 1.79633057598661e-34

EOGT AL139232.1 -0.40947795268399 7.02351122577731e-30

CPQ AL139232.1 -0.539229428968598 1.98179486040228e-54

EPCIP AL139232.1 -0.432207868388403 1.87787047251561e-33

CPQ AC098595.1 -0.445583315461135 1.09784867373571e-35

PKD1 AC027601.5 0.429971785523468 4.3387643309055e-33

CPQ C15orf56 0.460425407039368 2.77644775168768e-38

CPQ ZNF503-AS1 0.477248762969937 2.19365599084847e-41

CPQ CPNE8-AS1 0.416399266761505 6.12906358531775e-31

EOGT LINC02084 0.434580596064697 7.66929833843446e-34

TSPAN4 LINC02084 0.653419939912094 4.62309701462656e-87

CPQ AC002456.1 0.429842357281421 4.55338616872748e-33

CPQ AC093297.2 -0.416494323142166 5.92481513536063e-31

TSPAN4 HOXC-AS2 0.620440523685344 3.07273707509988e-76

ITGA5 AL158212.3 -0.446451146472188 7.80166254239921e-36

CPQ AL158212.3 -0.485190592996072 6.54022395164034e-43

NDST1 AL158212.3 0.510954207170105 3.81409116349844e-48

TSPAN4 AL158212.3 -0.450810788776824 1.38165937846178e-36
