# Supplementary material for: Associations between migrasome-related genes and long non-coding rnas in glioma and their prognostic relevance to the tumor microenvironment
Source: IBRO Neurosci Rep. 2026 Jun 24;21:279–90. doi: 10.1016/j.ibneur.2026.06.013 (PMC13356737; doi:10.1016/j.ibneur.2026.06.013)
Supplement: Supplementary file 5 — Supplementary material [file mmc5.docx]

gene lowMean highMean logFC pValue fdr

AC022968.1 0.631968926553672 4.1574715625 2.71778086636824 1.72169276604691e-45 1.70110929780246e-44

RPL22P2 0.748840630885122 5.22116453125 2.80164100167275 5.97995903335393e-33 2.87034946373816e-32

PI15 0.672445809792844 1.92971635416667 1.52089888831124 2.27641753671818e-29 9.14254516096643e-29

AL590867.2 28.0549411487759 78.32185171875 1.48115998770809 1.10604895329562e-21 3.16171872198802e-21

METTL1 13.2280171845574 68.5076051041667 2.37266732726522 1.02167181195596e-49 1.35796907386141e-48

AKAP5 5.34480047080979 2.33032765625 -1.19760327143673 4.58804602065109e-14 9.42079458414389e-14

MYBPHL 0.942910781544256 2.35730989583333 1.32194825581436 3.1389030984157e-35 1.68547222393993e-34

LRRC17 13.4456357344633 27.5342659895833 1.03409017942802 8.34916787126977e-14 1.69563976153628e-13

AC093726.2 1.83888093220339 4.56565708333333 1.31199443784639 1.40958035608497e-45 1.40166560007487e-44

RPL39P36 1.79116407721281 4.02337494791667 1.16750869287827 2.71473501644296e-45 2.63743456796013e-44

NBEAP2 7.17432448210923 2.99447453125 -1.26054013544795 4.0387448355566e-50 5.53430067923176e-49

THNSL2 2.95077871939736 7.63190526041667 1.37094752609355 8.24926389811174e-14 1.67663200552397e-13

CXCL14 44.0908906779661 154.721795364583 1.81112391463087 5.88823494615186e-16 1.30331780530525e-15

PRICKLE3 1.0292168079096 4.38777041666667 2.09194111821158 1.09936104630552e-72 2.34972628230473e-70

RPS19P3 1.3977527306968 5.20463411458333 1.8966875822608 9.1275662791663e-37 5.3592388683643e-36

AC007688.2 0.658127306967985 2.671209375 2.02105447345776 1.45146654771238e-19 3.76639972853917e-19

AC083867.2 3.66272782485876 1.74374734375 -1.07072747992671 1.65080759728213e-20 4.4819341906061e-20

LINC02513 0.656141949152542 2.2697265625 1.79043863920734 6.41083304139581e-20 1.68875818677936e-19

LAIR1 8.56336167608286 21.9609321875 1.35869012998232 1.12471329426452e-45 1.12683425144659e-44

CLDN7 0.803168691148776 1.75073869791667 1.12418883718806 8.69612593479693e-39 5.66192092988617e-38

MACROH2A2 9.9807384180791 4.8247015625 -1.04870685074434 1.20167843641674e-47 1.38446161865981e-46

NDUFA5P11 0.6234184086629 2.5996440625 2.06004144472286 6.48525281744989e-24 2.04084068608023e-23

APOBEC3B 1.11441492467043 3.71111015625 1.73556434063757 3.49741390861918e-39 2.32931273749189e-38

DIRAS2 49.6949676553672 24.4158211458333 -1.02528346604952 8.09312192686034e-16 1.77991250419832e-15

H4C9 5.30157702448211 10.6566 1.00725374063816 2.38707245801423e-43 2.04834390202006e-42

LEFTY2 4.26627099811676 10.6016660416667 1.3132434855082 8.3881428653636e-27 2.99208740804597e-26

CSDC2 119.472126271186 48.7914057291667 -1.29197511160233 8.82003355033429e-47 9.55760628604115e-46

RPS26P3 0.828528436911488 2.39866328125 1.53360752981971 5.25461433809212e-25 1.73029136916634e-24

TMEM71 0.662837994350282 5.10819614583333 2.94608571672163 7.45735948801567e-74 1.84892799572869e-71

TSPOAP1 35.7962949623352 15.2615411979167 -1.22990961025311 1.1686555492146e-55 2.506476348056e-54

CENPI 1.49642688323917 3.69590432291667 1.30440562053594 1.25445681372831e-31 5.64262807239427e-31

COL8A1 0.790858286252354 12.637385 3.99813495185724 7.40981457001732e-65 4.16270398578465e-63

STIL 1.65146167608286 5.15190859375 1.64136350633397 4.10498762183832e-49 5.203288672671e-48

AC025165.4 1.93082429378531 6.37995979166667 1.72433044762812 1.98375003366367e-09 3.31725106798345e-09

IL1RAPL1 3.57951986817326 1.46404864583333 -1.28980259668004 3.43632785617071e-47 3.84467608215971e-46

RPL12P12 1.19760338983051 3.4303590625 1.51820938397873 4.00679499641966e-34 2.04294908029678e-33

SUSD5 36.3560335687382 16.90725421875 -1.10455242915147 2.35177205439195e-30 9.92087144995878e-30

CAV1 22.7929935499058 91.7463888541667 2.00906095903938 1.82635208040322e-48 2.22549259076657e-47

LINC02869 0.184795244821092 2.04043208333333 3.46487515196573 1.61527625599914e-38 1.03823235327701e-37

EHD2 30.1733245762712 85.16448875 1.49697832649543 1.58719213705874e-55 3.35384520325083e-54

HERC5 6.76904435028249 17.7311602604167 1.3892628694129 4.66727283935571e-48 5.54910092377362e-47

AL139421.1 1.03657368173258 3.4781196875 1.7464849091315 1.75958000608144e-43 1.52112460311875e-42

AL033519.5 3.1419747645951 1.24303125 -1.3378090269568 3.93522279667331e-39 2.61340956800501e-38

AC110994.2 0.534586629001883 3.64861796875 2.77085444169655 1.98321717475035e-25 6.68199372431288e-25

HTR1A 5.04816902071563 0.860430416666667 -2.55262978404849 2.23742060116478e-59 6.7870858203359e-58

AL138693.1 0.684405414312618 4.65004317708333 2.7643210340444 3.71942604959039e-42 2.97346205469189e-41

PRSS35 27.2406149246704 11.2777996875 -1.27227364722669 8.33450892205505e-37 4.90443650017765e-36

KCNA1 4.79368248587571 2.11703130208333 -1.17909175291239 5.24879165104841e-09 8.581841268904e-09

NEK2 2.47581078154426 10.6160152083333 2.10026937938475 2.37596032733195e-37 1.44146761131281e-36

VMP1 29.3912362052731 65.4978397916667 1.1560612848755 4.83327328766109e-63 2.14498130749542e-61

AC005046.1 0.654873493408663 1.42269921875 1.11934254244678 3.23779457757225e-18 7.92924933095693e-18

PINLYP 1.27979472693032 8.74588729166667 2.7726923292487 4.24510775153198e-67 3.17019191324246e-65

AC010624.1 0.788634792843691 2.19829916666667 1.47895847058855 5.68829082491697e-24 1.794296317037e-23

TRAC 3.43184971751412 16.3695266666667 2.25395432317525 1.28205055397456e-52 2.15939583796711e-51

KLRC2 38.7970775894539 13.492609375 -1.52377860247038 5.75557661049854e-36 3.2275315763637e-35

SNORD11 0.718255979284369 1.47311411458333 1.03629918991497 9.63641954391257e-06 1.35472307718344e-05

CENPF 5.90317377589454 18.4932719270833 1.64743777937507 8.15574641584858e-28 3.049589877392e-27

HOXD3 1.23776643126177 4.0475828125 1.70932149689635 3.43442952110678e-27 1.24976941183915e-26

RPL19P21 0.794132344632768 2.21435078125 1.47943241926978 7.89093531546408e-25 2.57560017888458e-24

F5 17.1451323446328 7.77411526041667 -1.14104863799187 1.32363346023848e-31 5.94803387944286e-31

TIMP1 42.8662111111111 809.0635953125 4.23834029818218 1.13853147474308e-78 7.05699759094919e-76

PABPC1L2A 1.43961073446328 0.602638958333333 -1.2563129190297 3.46343229628149e-21 9.69333587437602e-21

FTH1P4 0.979190254237288 5.55344651041667 2.50372229248936 2.10204909549546e-42 1.70464905934313e-41

RBMS1 4.72965447269303 12.45872265625 1.39734946639628 2.13032332576847e-64 1.10651849839846e-62

AKAP6 29.9675093691149 14.500288125 -1.04731761764708 8.62267402287964e-55 1.70573003676007e-53

PRRX2 0.579716854990584 2.92057427083333 2.3328317362879 1.63732382618217e-32 7.70785735388796e-32

RPL9P7 2.24364218455744 7.82659088541667 1.80254142005434 4.95422923297068e-25 1.63311279182928e-24

EEF1A1P24 0.88836511299435 2.28451572916667 1.36266373270844 1.39830674577477e-20 3.80862954997537e-20

AL139099.3 1.01225461393597 2.05995645833333 1.02504162428209 2.07980108018276e-19 5.35723799501294e-19

GLRA3 6.55150244821092 1.49037729166667 -2.13614819837554 9.33858922966376e-64 4.51041731754799e-62

MCM10 2.17546996233522 5.22104401041667 1.26301122273679 2.4728812852185e-20 6.65651816714505e-20

MTUS2 1.79583540489642 0.535539322916667 -1.74559071128583 6.39619945400658e-21 1.7662210996028e-20

CES1 0.801019161958569 2.77376744791667 1.79193817720996 1.89116922665915e-11 3.45241427152237e-11

CD276 27.8339213747646 75.9388971354167 1.44799487006977 4.41291756042772e-61 1.58413517444312e-59

ALPK3 2.72737142184557 7.1841434375 1.39730497070411 8.11003685918543e-38 5.01516911860835e-37

PDIA5 3.51236661958569 10.6814014583333 1.60458560308625 2.08416889514742e-58 5.78434635899497e-57

AC062021.1 36.8537410546139 5.78394682291667 -2.67168488250008 1.33913030095049e-63 6.38490460158317e-62

CA10 50.6254028248588 20.1650118229167 -1.32800723080448 2.24235128737026e-38 1.42894181592358e-37

AL356535.1 1.58865480225989 4.479236875 1.49544728559852 5.68466005912422e-21 1.57371227928264e-20

PDE2A 46.3790967514124 15.3454665104167 -1.59566221810939 6.15170252528546e-59 1.77901879405417e-57

C11orf87 8.75020583804143 2.9602546875 -1.56359565057466 0.000789077323190119 0.000997206254228644

TXLNB 1.15320018832392 6.65797583333333 2.52944065634611 9.56472665504404e-58 2.47365913978503e-56

SP100 3.33188596986817 10.97248125 1.71947887288257 6.4967142503564e-70 7.59788688587278e-68

SPC24 5.0525211393597 18.710921875 1.88880528312458 1.29045524167961e-35 7.07429693957322e-35

MDM2 11.8789537193974 48.86455453125 2.04038056822671 3.16768389249149e-44 2.87192013558651e-43

CDC42P6 1.66483554613936 10.0821730729167 2.59835504649613 1.6923552540985e-40 1.2173828220488e-39

H1-9P 0.815885310734463 4.418345 2.43706780237421 8.76973370236111e-44 7.71031669954633e-43

CUL7 6.8568470338983 14.27361625 1.05773364758943 2.54337505974206e-44 2.32860951432317e-43

TCTEX1D1 0.990093738229755 13.38356671875 3.75675371460958 3.04672953307296e-48 3.65509262370914e-47

RIPOR3 1.70391139359699 5.79462244791667 1.76586434981445 2.33254573668725e-40 1.65864963570552e-39

IGLV1-44 1.53551238229755 7.41780640625 2.27227246994684 4.13503966964676e-17 9.62341209725676e-17

SHOX2 0.339877495291902 9.59619588541667 4.81937586411354 7.03419654588019e-70 8.17505529816513e-68

LPAR6 11.6536056967985 27.8753085416667 1.25821137117926 2.76366461308319e-56 6.29783621081886e-55

ANO3 3.3650684086629 1.1662271875 -1.5287869728795 1.79047934229268e-23 5.53791805887099e-23

NPTX2 28.4031830037665 65.0594002604167 1.19570490872541 4.17474111176201e-09 6.86316956707759e-09

HMGCLL1 5.6710027306968 2.16859213541667 -1.38684511173655 2.07431445618388e-39 1.40261372046325e-38

KCNC1 9.02165127118644 3.97502515625 -1.18242752962507 8.33902611273071e-31 3.59944732047882e-30

RPS2P4 0.476374811676083 1.84388729166667 1.95258143534893 2.07347076006525e-26 7.27338026474501e-26

THBD 4.18315320150659 11.2674930208333 1.42950381590443 1.25834895535807e-27 4.66952680600345e-27

OASL 2.12688672316384 8.88717546875 2.0629817748937 4.47056753954533e-46 4.61066019954772e-45

STUM 38.0432595574388 17.7829807291667 -1.09714369742775 3.64889700010445e-35 1.95143053543118e-34

MAP2K3 11.8319572504708 26.5004309375 1.16332707527196 9.35544849936182e-65 5.16215325951433e-63

LY96 17.0431861111111 60.7415729166667 1.83348920481252 3.13583078911103e-51 4.74843432601951e-50

SLC47A2 1.22353474576271 9.5087103125 2.95819460697767 3.84348657589448e-59 1.13626568698871e-57

TRADD 9.75062424670433 19.9104264583333 1.02995763224528 1.28853437772468e-65 7.98676558459681e-64

LINC02381 4.80485404896422 12.1608389583333 1.33967824778044 7.09460066994351e-46 7.23664835203508e-45

AL928646.1 0.51030918079096 15.8531473958333 4.95725388626698 1.2159008298372e-27 4.51381032757491e-27

AC017048.3 0.72891445386064 2.353285625 1.69085502176175 1.82662994774453e-06 2.66046713231844e-06

UBBP2 0.455839924670433 2.1044240625 2.20682625793268 5.07631063365247e-42 4.02361450267552e-41

MIR1244-2 40.1315209039548 107.39195703125 1.42007821125633 1.42557868616155e-45 1.41681644410337e-44

ASCL2 0.629078578154426 1.91792796875 1.60823639796063 2.86337548691643e-57 7.07097837705325e-56

KIFC1 10.1699994350282 29.6769461979167 1.54502304507242 2.07321138910773e-27 7.60832164603478e-27

LINC01159 2.13321850282486 4.42736125 1.0534153481213 2.4908006645387e-33 1.21981665412423e-32

RN7SL689P 1.93655193032015 4.27492234375 1.14240802390562 1.08525097616936e-24 3.5267811782365e-24

AL589674.1 0.605291242937853 1.50572776041667 1.31475956639275 1.65864553229388e-28 6.41349837242768e-28

RARRES2 6.21986468926554 90.0606436458333 3.8559416878353 2.99811576365477e-55 6.19444029168449e-54

GBP5 0.427881261770245 2.97907625 2.79958264467575 5.91012653608831e-58 1.57448141745791e-56

RN7SL128P 0.513423210922787 2.4078478125 2.22952378565116 1.43441521800932e-22 4.28068544036002e-22

MAD2L1 6.53494058380414 17.2535568229167 1.40064778021979 4.31088079301366e-29 1.70737222529135e-28

ITGA5 7.70065291902072 31.97183390625 2.05374882096788 2.74197329455941e-67 2.11564288017976e-65

FGFBP2 2.87978088512241 23.9951922916667 3.05871442438664 2.71258912882034e-28 1.03872724156537e-27

TMSB4XP8 38.5953491996233 575.219213802083 3.89761295025473 1.01523123467907e-67 8.20792382993796e-66

NAP1L3 165.480528672316 71.2693993229167 -1.21530680165189 4.00490301846001e-66 2.69823085609652e-64

ZC3H12B 5.87813596986817 2.47048359375 -1.25056525600344 2.14720006166477e-50 3.0020439959892e-49

SIGLEC14 5.30146534839925 12.7067373958333 1.26113056180315 6.82469082025678e-33 3.27075066501739e-32

PAX8-AS1 1.65786935028249 3.56105703125 1.10297522244151 1.33211894392672e-07 2.04598593890455e-07

WNT4 0.864250094161959 1.84590994791667 1.09481141253279 0.0126532440406863 0.0147942073023492

AC010226.1 0.955429755178908 2.65579802083333 1.47492371796736 1.77516721121817e-40 1.27448781052517e-39

CDK5R2 41.9769307909605 16.1754015104167 -1.37579516192988 3.7317715227799e-28 1.41877512709246e-27

IL7R 0.71490852165725 3.10322348958333 2.11793704709557 1.43322025903688e-36 8.31795590411697e-36

FERMT1 78.1989733050847 26.2818934375 -1.57308045004397 2.22814786073184e-51 3.40727051565038e-50

GAL3ST4 29.2674600282486 65.5832559895833 1.16402998137343 8.18935357681019e-57 1.93741768143493e-55

CDCA2 1.29176798493409 5.61623140625 2.12025541024933 3.73468849264955e-47 4.16096659801189e-46

EFEMP2 11.3529530602637 59.6505453645833 2.39346771676574 1.71757898134322e-63 8.04493228163153e-62

MT1M 39.6777539548023 168.743587760417 2.08843041466534 1.99636693899277e-43 1.72022443144442e-42

MCF2 5.43782424670433 1.97637151041667 -1.46017535900366 8.35284705409712e-44 7.36119388487848e-43

CHRNA9 0.238975329566855 9.09717421875 5.25048488676809 4.83942599323305e-65 2.79469336472573e-63

TMOD2 99.2698603578154 48.9858909895833 -1.01898948168444 1.89532676007166e-57 4.74341872187517e-56

SLC7A4 2.41605951035782 1.01208411458333 -1.2553267925702 1.05253541783382e-19 2.75002052755654e-19

MMRN1 1.80115513182674 4.74340666666667 1.3970011157633 4.93878848647091e-33 2.38104153243263e-32

AC018730.1 2.62497674199623 1.26749796875 -1.05032120495172 1.57158654286034e-50 2.2342241410159e-49

SP8 0.428263983050847 5.392518125 3.65438686352677 6.03919224995918e-15 1.28694453229419e-14

RGS7 14.919711299435 5.9551196875 -1.32501721016995 3.76963572007223e-29 1.49682631250786e-28

ETV7 0.842060028248588 4.26003432291667 2.338870066219 7.0510977131529e-51 1.03403124586812e-49

FTL 4295.4341329096 11466.5456748958 1.41655498656322 1.46093012433257e-60 5.03073993740077e-59

PXDNL 0.464781450094162 1.75125036458333 1.91376095544224 1.64152635817599e-35 8.95399901152318e-35

ARL9 0.639723258003767 3.8155878125 2.57638548890942 5.47027341645571e-51 8.10530205277229e-50

AL390755.3 0.728418691148776 3.33703744791667 2.19572802908865 1.20187951176931e-67 9.63316789713376e-66

AL162430.1 0.427404849340866 1.63984041666667 1.93988024177588 2.38634762688285e-32 1.11408822801623e-31

ESCO2 1.00240640301318 3.351833125 1.74148278931492 7.122110115907e-32 3.24358651984547e-31

MDK 51.5855038606403 201.19383078125 1.96354845510566 2.67633961894837e-47 3.01067968628826e-46

BATF 1.63627796610169 5.80119927083333 1.82593332675873 2.19722212621167e-46 2.30441880636808e-45

DDIT3 39.3169946327684 102.465646770833 1.3819153521858 8.8330793746718e-31 3.80827987414844e-30

UNC13A 43.8263229755179 15.8210658333333 -1.46995084741503 1.96154420841659e-47 2.22137116659601e-46

SNAP91 65.5772177966102 18.5617353645833 -1.82086309821167 9.21594834856623e-68 7.5162526114732e-66

KIF3C 112.893815254237 55.7685025520833 -1.0174440134379 7.03711754874356e-44 6.2312118829031e-43

EFNB1 18.6518259416196 38.14783703125 1.03228438742596 1.68140847604432e-58 4.73007422269957e-57

HAND2 0.492430178907721 2.21282328125 2.16789715420438 5.76766136797469e-35 3.05554595833303e-34

DCST1-AS1 1.45948286252354 3.09239 1.08326500714291 5.48760502510773e-30 2.27823209291981e-29

AC104137.1 0.68826516007533 2.59654036458333 1.91555426524036 5.8126463030626e-25 1.90931210043189e-24

TMEM67 3.36200725047081 7.64157005208333 1.18454625181165 5.8595120956925e-51 8.64057314983363e-50

WEE1 4.37360663841808 18.93145453125 2.1138898841241 7.62638662162165e-69 7.3099308880956e-67

SNX20 1.27091360640301 3.7482790625 1.56036240404632 1.96719170024355e-47 2.22641081351362e-46

C1QTNF2 1.69141520715631 4.22373536458333 1.32028859163377 9.01317528560083e-41 6.61665986718308e-40

AL022718.1 1.01864948210923 5.3812453125 2.40128237112946 1.41765983017625e-27 5.25231809964682e-27

AL008718.2 1.65945117702448 0.728534010416667 -1.1876379554528 1.29384904381728e-39 8.81286555669682e-39

RBP3 1.75941765536723 0.77609828125 -1.18078672949038 2.20580351912609e-51 3.37865868518531e-50

MIR155HG 0.458673446327684 3.21095994791667 2.8074653756614 1.0933072510993e-73 2.64026601742747e-71

PSTPIP1 11.8979717984934 5.64049375 -1.07682230184165 4.17808226768217e-29 1.65653389696269e-28

SETP14 1.47632791902072 5.52949369791667 1.90513418215824 1.87526532390529e-30 7.96131477123718e-30

IRF7 13.2804650659134 31.91373046875 1.2648715893739 2.98904047538802e-51 4.53724143998696e-50

AC015922.2 8.24030715630885 21.09334296875 1.35601773832986 2.32360355989407e-32 1.08670543753094e-31

NCF1B 0.981986676082862 2.98149947916667 1.60226272991389 1.94617299457863e-54 3.74240815244981e-53

AC004854.2 1.25318093220339 2.79970520833333 1.15968020465307 1.02925937935381e-41 8.00463327439736e-41

PRMT8 3.80303582862524 1.48825171875 -1.35353297024319 8.40935962412823e-13 1.63449401286364e-12

EMX1 2.46056271186441 0.860768020833333 -1.51529190147129 7.62967569718113e-10 1.30186571815298e-09

GAPDHP63 0.843448587570621 6.38389442708333 2.92006475656106 1.60165475679491e-42 1.3091327561583e-41

MCM2 9.64355376647834 25.99387765625 1.43053506481682 4.34986517405095e-32 2.00609481427275e-31

SLC27A3 6.27759336158192 21.0001457291667 1.74211585460751 1.67554648451924e-62 7.12969951479068e-61

AC010970.1 2.11056177024482 32.07975609375 3.92596421417213 6.06481329900503e-16 1.34176327537178e-15

GCSHP5 1.72049439736347 4.48997380208333 1.38388383313775 1.18269722874433e-21 3.3746040776908e-21

PITX1 0.60904736346516 5.44776604166667 3.16103841616564 7.42643347927404e-46 7.56267965756302e-45

HOXD11 0.0816295197740113 2.58157739583333 4.9830180706324 1.04941937672706e-61 4.08241701051039e-60

LEMD1 0.724837052730697 2.48364265625 1.77672900404526 2.98861608488739e-09 4.95305847580045e-09

ABRACL 13.6780537664783 36.29686328125 1.40798191261909 2.05792365921308e-62 8.67734477167057e-61

RGR 17.442259039548 4.42648171875 -1.97835453498374 3.16351939744008e-56 7.16512097386094e-55

HCN1 3.01864576271186 0.713598697916667 -2.08071657897146 4.58722160955574e-20 1.216130393922e-19

MARCHF4 13.3242962335217 3.44007213541667 -1.953548611861 3.1857568010351e-35 1.71013705875426e-34

PTPRN2-AS1 0.588147551789077 1.73885703125 1.56388927682864 3.31643291944821e-45 3.20525312563095e-44

PCDHB7 4.37260776836158 12.84694875 1.55485990212572 3.446844447374e-29 1.37187655177482e-28

AC007906.2 0.722166525423729 1.788508125 1.30835321863324 2.52438852295651e-19 6.47551449639623e-19

LAMA4 11.6063289548023 24.264081875 1.06391054674207 1.24629599924193e-28 4.84525906458367e-28

PHBP4 2.18931690207156 0.943635677083333 -1.2141789284232 1.39197097877116e-37 8.52840209233928e-37

SSTR3 2.05547321092279 0.5885990625 -1.80411341979476 2.62172640625154e-13 5.20955359310188e-13

STX1A 32.8168075329567 16.1844738020833 -1.01982443752559 0.000364992908407043 0.000470897324070559

SYN1 85.0896906779661 33.3153800520833 -1.35279599648295 1.13001643633715e-29 4.6191812780148e-29

NSG2 260.630303813559 70.2178660416667 -1.89209477990034 1.85284388344026e-59 5.68541782385671e-58

ZNF217 5.80054439736347 14.6410661979167 1.33576040531235 2.23645617006794e-54 4.2697025135948e-53

AC009220.3 0.770524105461394 1.60350401041667 1.05731596546043 4.61892136224419e-32 2.12701443117709e-31

DIAPH3 1.37617565913371 3.11627208333333 1.17915656969361 5.78344750978845e-35 3.06303635558292e-34

NGFR 9.41925560263653 40.0141042708333 2.08682366012655 7.03713092449396e-44 6.2312118829031e-43

AL138767.3 2.11753620527307 0.80375484375 -1.39755920488217 2.06037311219541e-38 1.31658549901284e-37

SGO1 0.909674011299435 3.58164895833333 1.97720240186063 4.78240013506184e-39 3.15910232722824e-38

LCNL1 13.4138493879473 5.10095817708333 -1.39488313133702 3.29473829028206e-32 1.52820300593153e-31

CRYM 23.9591120056497 10.4172157291667 -1.20160470713972 2.86165381262696e-08 4.53026159082226e-08

MICOS10P2 0.235173540489642 1.884676875 3.00251954084394 9.77196105849714e-36 5.40159381339935e-35

MEOX2 2.60740376647834 39.1061669270833 3.90671022207379 2.94627210735917e-44 2.6764010667486e-43

AL645728.2 2.41265329566855 5.61128932291667 1.21771168977471 8.87060399095722e-47 9.60680729247813e-46

SLC4A10 15.9219106873823 5.69616630208333 -1.48295030279101 2.90542036555715e-13 5.75911861182552e-13

FSIP1 0.886854990583804 2.06696458333333 1.220743534682 3.28475387583274e-41 2.47287442595586e-40

ANGPTL6 0.947420480225989 2.01225072916667 1.08673331423981 5.88321261066522e-41 4.37068871335676e-40

APLN 53.3754397363465 135.88596984375 1.3481485506632 1.49653340932034e-33 7.40304302908e-33

FKBP9 19.5911759887006 91.3459274479167 2.22113640934061 7.82637422868839e-62 3.08983925228154e-60

RPS4XP6 0.902902024482109 2.53087526041667 1.48699505101402 9.54590746366007e-23 2.86901809094487e-22

MYH7 28.2235106403013 9.42629119791667 -1.58213529670019 1.45041701783804e-33 7.182557775419e-33

HOXB2 0.595571374764595 9.81828901041667 4.04312531464312 2.36879349914433e-53 4.25170995333869e-52

PPP1R15A 31.5344347928437 63.5706979166667 1.01143385196711 8.02380967591772e-49 9.94684939491267e-48

RPL15P18 0.497778107344633 2.5104646875 2.33437974601112 4.08575820265987e-23 1.2463037037149e-22

PIPOX 14.9344652542373 55.40401140625 1.89134485402191 3.34890654902503e-37 2.01595717964132e-36

NACA2 0.7250881826742 2.79823973958333 1.94829120449216 8.53387639069763e-23 2.57317060945391e-22

KCNN1 19.6511337099812 8.91034057291667 -1.14106006570258 5.92020467096689e-39 3.8817420964961e-38

ITGB3 0.685999576271186 4.21299375 2.61856618526965 2.59490647484005e-67 2.01895391423109e-65

LOXL2 7.49891544256121 27.6404524479167 1.88202737058154 3.39567360313091e-55 6.98479542590921e-54

HSP90AB3P 1.65950117702448 4.84751994791667 1.54649718247685 9.46524555601947e-21 2.59635193505441e-20

CYP39A1 1.13062707156309 2.6078540625 1.20573999132362 2.11266579883636e-25 7.10398201254288e-25

GPR65 1.38913498116761 6.37181114583333 2.19751671575824 1.53764671631965e-65 9.43648207589567e-64

AL445433.1 0.920409369114878 3.39213328125 1.88184528198379 7.06134545392493e-27 2.52754030251654e-26

RPSAP15 1.22737099811676 5.6810109375 2.21057628140767 2.36100441926069e-20 6.36181382062781e-20

FAM181A-AS1 1.45257048022599 5.90720197916667 2.02386677507373 1.08975836764627e-49 1.44486848860539e-48

PYCARD 8.70291638418079 18.6102030729167 1.09652295772475 7.13223138151998e-42 5.58886820646287e-41

CA12 12.5106847457627 37.788241875 1.59477664322571 4.33132856981107e-21 1.20715116333569e-20

HIP1R 158.663530131827 72.3417715104167 -1.13306972086683 1.56841462902859e-37 9.59048669082099e-37

PPIAP11 2.3345774952919 7.74258140625 1.72965316760103 2.20158632810848e-42 1.78303888661584e-41

AC025176.1 0.543414359698682 2.07867979166667 1.93554294234529 9.55365762534958e-33 4.55046781617253e-32

COL26A1 12.0469956685499 4.74285625 -1.34484535791022 3.90703564079441e-40 2.74570399624233e-39

RFPL1S 7.48506106403013 2.37022098958333 -1.65899250970979 3.10660462313182e-27 1.13158301600659e-26

TLR1 3.97118799435028 9.92927869791667 1.32211825943677 7.64989362594618e-43 6.37892536938833e-42

LGR6 1.30134289077213 16.2219547395833 3.63987462135116 4.44966333450188e-37 2.65537515099687e-36

TRDC 0.698231308851224 18.07313359375 4.69399780891008 7.05237303020992e-43 5.89653221658064e-42

HLA-K 3.93831384180791 8.00878395833333 1.02400511932464 3.76246613361232e-19 9.57610973918986e-19

SEC61G-DT 0.813334698681733 13.0668780208333 4.00592151507495 2.6673384028032e-08 4.22804173558311e-08

ACTBP7 0.417075941619586 2.72649989583333 2.70866810023012 1.03257315813226e-50 1.49421773349956e-49

AC104051.2 65.7823831450094 10.52821359375 -2.64344061113874 3.90944642954552e-16 8.72074812348836e-16

FTH1 108.867327118644 227.87595875 1.06567768375042 2.72983580989528e-31 1.20630458376908e-30

FAM133A 11.9668822504708 3.6410934375 -1.71660366504789 3.41194606628732e-68 2.96472603283237e-66

UNC5A 37.6352177024482 12.4660691145833 -1.59407670032361 1.02916520063483e-57 2.65427557639454e-56

IL18 10.3896217984934 24.6081107291667 1.24399076170262 6.32111493422007e-45 6.01849115216704e-44

RPS3AP25 0.704935263653484 2.33237348958333 1.72623614824785 6.38143262064663e-24 2.00863479011576e-23

G0S2 2.69311064030132 32.02359859375 3.5717900311984 4.8564655541322e-53 8.48740385141807e-52

TMEM132D 4.71680696798493 1.25275328125 -1.91270824426973 2.19857001562454e-23 6.77983572811581e-23

HMGB1P1 3.306165913371 7.03653942708333 1.08970696004279 9.80767007960176e-29 3.83057393678208e-28

AC112191.2 0.42773686440678 1.98646921875 2.21541098234174 6.79770637806139e-25 2.22462777367215e-24

TGFB1I1 8.2464868173258 21.5450793229167 1.38550687415412 2.92965296909654e-49 3.74669167540235e-48

ASF1B 6.92255381355932 33.61625515625 2.27978274883641 3.83244994708811e-47 4.26477598839637e-46

LPIN3 1.00379326741996 4.30926505208333 2.1019796628426 1.21254911272485e-43 1.05558758198121e-42

RPL37AP1 3.59315974576271 15.2053714583333 2.08125607935248 5.53198309027322e-23 1.67973915028789e-22

NCF4 8.50148502824859 17.862236875 1.07112598297634 1.37457244645106e-34 7.145701605188e-34

HOXA6 0.107271327683616 2.09969630208333 4.29084425509173 3.12194429832276e-66 2.11100197190224e-64

HLA-L 1.64594444444444 3.81308682291667 1.21204373989717 1.2673729632438e-33 6.29623303540434e-33

CNPY4 22.9750552730697 47.1190272916667 1.03624142466153 8.14830253385653e-69 7.77013772395191e-67

HMGN2P4 0.866470150659134 4.9950459375 2.52727599054997 1.9107813708173e-46 2.01651416517297e-45

IFI44 25.9265958097928 73.7210005729167 1.50764286077566 3.41192565175041e-58 9.23504475899547e-57

PYGL 11.3131035781544 46.74235765625 2.04673573952109 1.08484321819351e-81 1.55174304940833e-78

CYP27A1 34.7148559322034 74.8916784375 1.10925223881858 2.62110048509341e-46 2.74124654219977e-45

PFN1P1 1.23692782485876 9.37039890625 2.9213491449632 3.89120492023991e-45 3.7432465334641e-44

DDIAS 1.54973418079096 4.73535510416667 1.61145183994507 4.9373895539285e-44 4.41615245878494e-43

BX679664.1 0.75748813559322 2.19329453125 1.53380436322169 2.57042676307626e-22 7.56761964208408e-22

EMP2 4.31819764595104 9.92473197916667 1.2005988645943 2.37418617081074e-22 7.00651216869421e-22

AL121956.5 2.67901845574388 0.9560553125 -1.48653852719903 8.77058970878729e-38 5.41464527340304e-37

FNDC1 0.669347787193974 1.8401190625 1.45897119461612 8.28593626895702e-14 1.68390147454924e-13

CDK5R1 113.457176082863 47.1996238020833 -1.26530059534627 6.18385184675592e-54 1.13850222861808e-52

MR1 6.11705235404896 13.612708125 1.15404557766469 2.72230804261005e-49 3.48871936956126e-48

CRNDE 0.708854661016949 4.55937880208333 2.68527551407866 2.71396476550076e-69 2.85119631720263e-67

H3P14 1.16890979284369 3.53626989583333 1.59706479012221 4.46182405310159e-30 1.86193039199785e-29

AC112220.2 0.871683662900188 1.91728359375 1.13718717215007 2.20785685267369e-38 1.40792517748516e-37

SNAI1 1.75711516007533 6.84901463541667 1.96268769548127 3.45439059703812e-56 7.79543606212668e-55

FTH1P3 3.13001690207156 6.93149947916667 1.14699703409834 1.26646582877284e-41 9.79207155344323e-41

FTH1P5 1.92221101694915 6.54242145833333 1.767057978513 4.04402353979306e-44 3.64335084631262e-43

SYNGR3 28.1495851694915 12.449639375 -1.17700970919361 8.75619182265574e-13 1.69995183694178e-12

H2AC20 1.26562542372881 5.74046713541667 2.18131765632325 3.97896636368021e-48 4.74591914898226e-47

ERVK3-1 2.47954915254237 5.05916276041667 1.02882082899601 2.53628936685503e-44 2.32326604811179e-43

EEF1DP1 1.58108653483992 4.33348505208333 1.45461139784436 1.0724708265837e-23 3.34551166252708e-23

SLC11A1 5.45480037664783 25.6615806770833 2.23401173299231 1.45308548508433e-63 6.87534976975652e-62

RTN1 264.085537806026 108.95060421875 -1.2773310995548 1.4722972253687e-51 2.28144724214425e-50

AC022034.1 0.832124529190207 3.71955255208333 2.16025772909666 1.41470432165176e-30 6.04189868192799e-30

SEMA3E 2.09998695856874 6.91563072916667 1.71948046766548 5.72759460268025e-16 1.26882176817844e-15

KIF14 1.244634086629 4.13661572916667 1.73272928189416 4.01984105388664e-36 2.27338638677074e-35

PLA2G2A 0.712285216572505 77.883325 6.77271561921023 1.69524885537407e-67 1.34714326776414e-65

CXCL13 1.78217335216573 9.42224677083333 2.40243344225466 0.000255343736009665 0.000332315003576408

CALM2P2 3.78603935969868 10.3427661979167 1.44986077504742 8.31105405189653e-22 2.38493904467617e-21

FMOD 8.72861224105461 105.898929791667 3.60079190087065 4.37818062467809e-53 7.67316387520161e-52

SLC6A17 30.0831708568738 9.15150984375 -1.71687495110167 1.1192372740281e-18 2.79058958307221e-18

C8orf48 2.14896016949153 4.93823078125 1.20035551739866 7.74539132846261e-34 3.88209034374022e-33

TMSB4X 1217.6156653484 4109.956273125 1.75506421958573 4.68369874665112e-83 7.91757983581614e-80

IGLV2-11 0.550778483992467 8.03379302083333 3.86653718568372 7.66902812608346e-20 2.01334996476806e-19

HOXA5 0.501072740112994 15.6278160416667 4.96295231535282 9.29979482768313e-72 1.55792508847539e-69

RAB36 4.74356633709981 16.29045875 1.77998320367413 3.94853373947445e-40 2.77277133253502e-39

MIR3605 0.917770527306968 2.15376557291667 1.23065584514613 1.00568268654037e-15 2.2024107356281e-15

NECAB2 39.2611049435028 17.9719072395833 -1.12735725516194 1.6789762982396e-40 1.20822617127575e-39

TMEM230P2 0.413583097928437 2.14238 2.37296526492998 1.47218745561072e-40 1.06477346313035e-39

RSPO3 1.59723973634652 0.5831125 -1.45373471428586 1.07452444340699e-26 3.81458228811626e-26

HSPA12A 9.70323968926554 4.84368744791667 -1.00236073630529 2.6271838019362e-32 1.2237595891033e-31

KRT222 2.1820120527307 0.580398177083333 -1.91054417736287 2.47888817829811e-26 8.68077696336221e-26

KLRC3 5.52269774011299 2.08672541666667 -1.40413239530581 1.28112246905185e-43 1.11372007068813e-42

LEF1 7.13854548022599 14.8344753125 1.05525184848118 2.04150069312995e-35 1.10675525914727e-34

H19 3.88937980225989 38.3194925520833 3.30046642869707 1.21568926740523e-37 7.45817945476749e-37

CHGB 137.674584322034 35.4471883854167 -1.9575191477564 6.14102345243217e-58 1.62899188442191e-56

RBM8B 0.793900564971751 3.44259947916667 2.11646811574683 6.56496195693664e-36 3.66592995763474e-35

SPAG1 5.09196883239171 11.25833921875 1.14469852927727 2.09411455414441e-32 9.81105067632031e-32

BISPR 1.62235983992467 6.26218432291667 1.94857212853531 6.70681060705003e-55 1.33525849291323e-53

TMEM150B 0.674541242937853 1.87258765625 1.47305469260799 7.0302117890032e-24 2.20896904725439e-23

AEN 11.4664274952919 23.1650588541667 1.01453437554593 7.19834465992263e-37 4.25065795335857e-36

NFKBIZ 2.40350028248588 8.04552453125 1.74304950806866 1.04213256688895e-45 1.04578818571506e-44

WWTR1 22.9001007532957 64.58504953125 1.49584629559343 1.60492383120188e-52 2.66698468643422e-51

NKX2-5 0.359443032015066 7.9300065625 4.46348701811169 5.56675818117752e-51 8.24155003017484e-50

CELSR3 20.4200812146893 8.60532541666667 -1.24668694913324 7.61264809093278e-40 5.26040262667715e-39

TLX1 7.62520550847458 3.4312890625 -1.15202555230801 2.02684925172058e-18 5.00056545518697e-18

RPL21P93 0.800065630885122 1.80410125 1.17309005103152 9.07138051124317e-16 1.99058674305602e-15

DYNC2I2 46.2884882297552 96.40225046875 1.05841338059236 1.20649575182402e-52 2.03767379701795e-51

SPI1 37.6272215160075 81.1685253125 1.10914364126498 6.16427187917015e-42 4.85491891542435e-41

IL1A 1.27320974576271 3.05821630208333 1.26422034429297 5.24686907955079e-29 2.07189489348581e-28

MRPS12 28.1377911958569 58.6780977604167 1.06031302006394 1.77004615199296e-63 8.24912486123035e-62

AL358472.1 0.470699058380414 2.10750494791667 2.16265914619982 6.02021157214061e-23 1.82470797365859e-22

MSN 62.552220480226 292.83757515625 2.22696768021357 3.57872863782028e-75 1.03978842219169e-72

AL390786.1 2.29762151600753 0.81600390625 -1.49349320083456 6.01203751459024e-39 3.93719913275635e-38

RPL13P12 38.9015729284369 117.224143645833 1.59136934504264 5.51020115781213e-23 1.67339850611655e-22

FOSL1 3.12751200564972 15.6744304166667 2.32532569116632 9.44463620894641e-62 3.68955904002854e-60

VSTM2L 49.524268079096 22.3535645833333 -1.14763074631026 5.31433040247752e-16 1.17937670168361e-15

NKG7 2.77959783427495 8.91136333333333 1.68077000201619 2.58172640141393e-48 3.11937637649721e-47

PRSS23 5.36569190207156 18.23640953125 1.76498559041401 2.74308971357154e-46 2.86239944806352e-45

MEIS3P2 1.14216760828625 3.02670546875 1.40597391647286 1.61335415644057e-29 6.53317084908807e-29

GLA 5.9774156779661 13.6025414583333 1.18628244707965 1.30975398582352e-79 9.74195014655534e-77

BVES 4.77289138418079 9.81556057291667 1.04020715766145 1.01718581403211e-18 2.54057356775381e-18

MTFP1 0.793158333333333 1.66441994791667 1.06933868800982 2.87874617824996e-34 1.4771049995739e-33

NCF1 1.28557438794727 4.4593371875 1.79441619935421 2.12720973793752e-63 9.76678150048103e-62

PSMC1P1 1.32775753295669 4.30195833333333 1.69600183708727 3.67181515951659e-32 1.69844285799032e-31

LGI2 3.7280172787194 7.52743359375 1.01374952904659 4.37507211135088e-11 7.86641861344996e-11

YWHAZP3 1.83127768361582 4.03590583333333 1.14004194447914 3.09941138766575e-21 8.69285926343891e-21

EEF1A1P12 5.46025767419962 12.6988986458333 1.21766244003419 4.55558953490117e-17 1.0583606621875e-16

ARSL 2.65863347457627 6.88037125 1.37180151414735 2.59334017945959e-44 2.36852458924612e-43

IGHV5-51 0.69222895480226 8.31941609375 3.58716108152905 1.83768026980208e-15 3.9920168945058e-15

STAC 0.394579566854991 8.67241083333333 4.45804494858387 4.40933649728334e-55 8.96139202987406e-54

C1RL 4.82985367231638 24.5735247916667 2.34705342446172 8.47659513544829e-71 1.15898740105633e-68

DGCR5 7.77433507532957 3.28111130208333 -1.24453475556266 7.65053866638577e-50 1.02567964312504e-48

AC138207.9 0.742554378531073 1.98970578125 1.42198652860348 2.44807264377099e-35 1.3213907347147e-34

GRIA4 83.9490643596987 34.6179835416667 -1.27799255413506 8.18935357681019e-57 1.93741768143493e-55

C16orf74 1.29747838983051 2.62557489583333 1.01692283989287 5.41218721934142e-13 1.06036899529716e-12

IGKV3-20 3.13194303201507 29.23439109375 3.22253666626218 2.10222630422786e-22 6.22268356050892e-22

CKAP2L 1.91737707156309 7.04510651041667 1.87748743241831 7.08994776251764e-37 4.19064140635777e-36

TUBB6 11.6209063559322 53.77061609375 2.2100954077782 1.24226195996929e-64 6.67626044671357e-63

RELB 7.95197005649718 17.0756356770833 1.10255505743598 1.31730492161506e-45 1.31271883584182e-44

SLN 12.1505927495292 71.18863828125 2.5506203098002 8.65457130436642e-26 2.96157072883131e-25

SLC25A48 14.6745048493409 5.27277359375 -1.47667786874053 5.42886478762248e-42 4.28661319430319e-41

RPS2P55 1.51686605461394 6.40646036458333 2.07843377765665 2.1156837828269e-33 1.03994554432107e-32

CKS2 42.7652799905838 140.034244010417 1.71126777665658 1.21453830681964e-46 1.29944417809616e-45

MIR221 0.809139406779661 2.09584796875 1.37307387726651 3.42268815071974e-10 5.92817494063278e-10

LINC02883 10.3755997175141 3.84486182291667 -1.43219107124235 1.91829830046725e-37 1.16914968525692e-36

NRGN 555.16742787194 181.681111197917 -1.61151449130346 8.02897337226961e-08 1.2464414748485e-07

GPR179 2.48968615819209 0.980013958333333 -1.34508968948641 1.40160371068051e-40 1.01569840218644e-39

KIF20A 2.80288879472693 14.1880850520833 2.33969347245757 1.67881846314391e-45 1.66139591922092e-44

AC061992.2 0.298178672316384 2.90002421875 3.28181597309458 5.26187972033823e-72 9.23062767921598e-70

COL14A1 3.07161967984934 9.20854651041667 1.58397386104702 4.78004098811939e-18 1.16326216691637e-17

CRTAC1 86.0672732580038 24.8058297916667 -1.79478554446162 5.90866981347988e-66 3.8282827589428e-64

RPS2P5 90.4441253766478 303.177024322917 1.74506172229867 3.95108941110974e-28 1.50031667550716e-27

C9orf64 6.36541464218456 23.3003213541667 1.87202345318194 6.56271540268868e-55 1.30797098513393e-53

AC018659.8 0.709252306967985 2.46297875 1.79603333741838 2.23359002885887e-15 4.83623737617963e-15

IGHV2-5 0.394785687382298 4.74842552083333 3.58830763287817 1.71255698835719e-13 3.4297250617665e-13

HOXC6 0.208431497175141 2.35407255208333 3.49751357225089 1.1593570794891e-71 1.84258503359827e-69

COL8A2 5.37427627118644 16.6922609895833 1.63503699109555 1.40660417483442e-41 1.08305609238286e-40

AC098851.1 0.818105178907721 2.30816942708333 1.49639088743319 2.01852677481252e-34 1.04349472831912e-33

RANBP17 2.15037994350282 0.84454375 -1.34834752116282 7.11750769895313e-49 8.86470567059835e-48

SH3BGR 0.90132434086629 2.16776911458333 1.26609284859319 4.7666715056382e-30 1.98557922597093e-29

GSDMD 7.58024256120527 24.8025928645833 1.71017502868327 1.15536020775983e-62 4.95021268739494e-61

CCL26 0.554825564971751 1.94211703125 1.80752396956509 1.09459604364106e-25 3.72920729782072e-25

CCER2 3.6833115819209 16.4683194791667 2.16061799254561 9.99884695558083e-35 5.2389155605989e-34

AP000547.3 15.0642073917137 3.48518598958333 -2.11181721327794 3.03749126166066e-16 6.81410906147665e-16

IFI6 387.841713182674 1227.02603052083 1.66162597336387 8.7712385017542e-46 8.86419456196301e-45

HMGN1P38 1.04749020715631 7.22208109375 2.78547786337345 5.24515340105296e-39 3.45129608961712e-38

LINC02058 5.84050927495292 1.955088125 -1.57886053524944 1.53290793949838e-39 1.04144768487294e-38

TSPO 69.8376161958569 158.888447239583 1.18593800966153 2.53396519104865e-55 5.25882619727117e-54

PARP9 12.2654178907721 31.87579203125 1.37786480137698 8.98901590410789e-62 3.51896317340813e-60

MTND4LP30 1.18004194915254 5.06577364583333 2.10194446781962 2.37637500647108e-18 5.84506524409123e-18

TEAD4 3.83998639359699 15.6354140625 2.02564432162372 1.01747499835159e-46 1.09617309353116e-45

LOXL3 9.58985527306968 21.23619203125 1.14694414456287 7.42477954096225e-56 1.62427971251992e-54

AP005901.3 1.67156040489642 0.4796128125 -1.80125338541882 7.42862518681601e-26 2.54956229879741e-25

AF279873.1 0.301601836158192 2.68319880208333 3.15323683301938 6.2260656666125e-34 3.14090317608951e-33

ITGB2 34.7346611111111 80.6422225 1.21515937832875 3.20188470558242e-42 2.57077055700799e-41

S100A11 125.344357109228 652.178891458333 2.37937069851535 5.04545727105653e-76 1.77019392368483e-73

JPH4 107.354951177024 36.2066418229167 -1.56806245165221 5.35191338514617e-71 7.53930525733281e-69

Z97653.1 9.25073361581921 4.36986010416667 -1.08198068666073 4.5776950835013e-40 3.2024921022463e-39

FILIP1L 7.23652485875706 18.1054654166667 1.32305630875226 6.92440266713554e-40 4.7955034486177e-39

MIR616 2.17960833333333 6.68691453125 1.61727176572245 9.51628909605861e-15 2.01085676978648e-14

BTN2A3P 1.00542292843691 2.70859833333333 1.42974397271541 1.20102466662865e-60 4.16661449178354e-59

KIAA0513 49.8286025423729 22.73112796875 -1.13230483814267 1.19857082027498e-34 6.24647544927501e-34

TNFRSF4 1.4151827212806 3.5386834375 1.32222436873116 3.18758486568504e-40 2.2511637135364e-39

AFAP1L1 4.27949717514124 13.7142381770833 1.68016128257625 6.24892615892877e-43 5.24599466931289e-42

FKBP9P1 0.569425564971751 3.90479723958333 2.77766846597203 1.89616654308688e-52 3.13415261055116e-51

AC091825.2 1.12386115819209 5.16036666666667 2.19900976307443 6.01548554138853e-25 1.97315141369059e-24

MAN1C1 12.9243024482109 39.0978513541667 1.59700290818585 2.29065176522556e-43 1.96742122745355e-42

PI3 1.50200753295669 67.5570997916667 5.49114344148198 1.42615709438614e-34 7.41179183066246e-34

SLC7A3 1.97459595103578 9.70403453125 2.29702721020251 8.44812400856377e-09 1.36792812555942e-08

COX17P1 1.33565207156309 3.29895848958333 1.30446638041381 1.43260445717121e-29 5.81516696815076e-29

DNM3 33.3325281544256 14.02863171875 -1.24855644221973 3.11693862324899e-61 1.14771235048148e-59

CD248 6.98248333333333 43.1901346875 2.62888968554863 3.94585230004598e-60 1.29405861586164e-58

AC098591.3 0.403610169491525 1.98367833333333 2.29714367144857 5.14974117869673e-24 1.6285618574467e-23

IGLV2-23 2.91182217514124 10.5136420833333 1.85226836907484 3.67430455589896e-21 1.02649779472568e-20

AC068587.2 1.0396274952919 2.8988378125 1.47940792335302 7.01728446119006e-19 1.76691136839308e-18

ZMYND12 2.21639661016949 6.55284536458333 1.56390542145999 1.0770728261041e-39 7.37957597693653e-39

MFAP2 2.59659185499058 6.43447140625 1.30920237351171 5.36791185029858e-52 8.56057640277033e-51

AL359091.1 30.5637450564972 14.08260890625 -1.11790670290494 1.78388232615319e-36 1.02984451582796e-35

CFI 1.019525 5.388685 2.4020361028357 4.11045343195008e-62 1.6835656732844e-60

PLK3 8.67978728813559 22.121464375 1.34971529814458 2.25365746289391e-71 3.46336863822415e-69

SLC32A1 14.8024442090395 5.37290864583333 -1.46206020260166 1.70858650250045e-13 3.42214196617793e-13

AC079944.2 0.995484369114878 3.46411208333333 1.79901504170163 1.56691916044568e-50 2.22929317432957e-49

HOXB3 0.170294444444444 5.302363125 4.9605321990431 4.37893213584061e-62 1.78958775969134e-60

CFAP99 0.576181261770245 1.5217471875 1.4011340515331 2.40571761373131e-13 4.79159372614971e-13

ARHGDIB 97.1317333804143 221.576781510417 1.18979209999965 1.69219569027254e-53 3.05498823889494e-52

BCL7A 43.4259153954802 21.6806406770833 -1.00214887060782 5.7792165605477e-54 1.0671750937774e-52

MARCHF9 22.2709748587571 108.341283385417 2.28234647069791 2.25502734107101e-16 5.08948093302773e-16

ARPC3P3 1.29607255178908 6.4274984375 2.31011087441249 2.37183035946551e-47 2.67622485037992e-46

AC104692.1 3.45264472693032 17.0842864583333 2.30689619815531 2.47313354446216e-11 4.48793971496768e-11

TM4SF1 15.8170916195857 47.61798671875 1.59002227748202 1.3899503737041e-44 1.29619494478574e-43

SSTR2 23.8239782485876 7.55857885416667 -1.6562274288444 1.64776418281768e-55 3.47394274143932e-54

SNORA22 1.44756873822976 3.20403630208333 1.14625863709066 1.03784532629494e-12 2.00673400273626e-12

SIX6 0.135411299435028 3.58869026041667 4.72803737447466 1.72265404338124e-41 1.32202855702328e-40

KCNIP3 49.0446911016949 16.1911665104167 -1.59889004879499 4.03233888350284e-66 2.70690763677745e-64

FAH 5.21595442561205 10.8286428125 1.05384926762507 6.67877297179384e-60 2.15237059636926e-58

BX248409.1 0.629534981167608 1.50053911458333 1.25312247513194 1.71732170958054e-21 4.8686685759491e-21

TSPAN2 0.946042278719397 3.08602307291667 1.70577228408581 3.19546338264506e-19 8.16428161586767e-19

SP140L 2.12907580037665 6.77534307291667 1.67006668568316 3.93099420630866e-63 1.75292175698584e-61

RBM47 2.33344891713748 6.36750927083333 1.44826527021913 2.87573433478549e-47 3.22522798283089e-46

AC135983.2 0.884184274952919 1.78458416666667 1.01316896350009 6.97346114579314e-32 3.17822328446136e-31

NRM 17.2609862052731 39.6825240625 1.20098889812644 1.40462136814436e-49 1.838067159792e-48

FANCD2 2.51663003766478 5.95825333333333 1.24339631971891 6.87971073942758e-34 3.45845420923644e-33

RPL14P1 14.1162975988701 36.4869995833333 1.37002076904676 2.84272104952008e-23 8.72859939164893e-23

SEM1P1 0.616466054613936 2.21180520833333 1.84313097366338 9.90857158049991e-39 6.43555321478854e-38

AL353807.5 0.877183003766478 5.59883666666667 2.6741773294746 4.01374562424774e-70 4.91023683440044e-68

SLC16A10 0.723403531073446 2.22314489583333 1.61972943541431 1.04641743498879e-18 2.61115106914562e-18

TNFAIP2 11.0812176082863 30.3793752083333 1.45497578526941 8.84534178679738e-35 4.65287497950487e-34

LINC01894 0.989696233521657 2.34208046875 1.24273295123411 8.30897015385667e-14 1.68821350536456e-13

STC1 1.41427316384181 10.0301911458333 2.82621639407985 4.65818733174749e-44 4.17843673101035e-43

AL121821.2 1.60932448210923 0.791294322916667 -1.02416892876152 1.08661605712258e-26 3.85603541644931e-26

CCNJL 0.953345856873823 2.2025559375 1.2081070609661 7.63004339896773e-42 5.96638591269154e-41

NSUN7 0.242032438794727 2.13941302083333 3.1439427013484 1.86712382880677e-63 8.65814653283339e-62

C6orf141 0.503367655367232 6.21330364583333 3.62567613881279 6.03100191366259e-42 4.75196951629474e-41

SLC16A4 10.2193110640301 28.0422591666667 1.45630464166532 2.82988866926349e-50 3.92114603613671e-49

TRAF5 2.34829901129944 5.63045791666667 1.26163613932302 3.35710213710114e-53 5.93396523188172e-52

SIGLEC1 4.02474439736346 10.336044375 1.36071510010697 1.48654126212971e-19 3.85472525021642e-19

CCDC152 1.18185710922787 4.02416197916667 1.7676327581271 8.47003190869877e-34 4.23387750920037e-33

EEF1A1P5 62.8274094161959 177.967725208333 1.502149630082 1.73459837192799e-14 3.62454845780436e-14

NPNT 5.04870899246704 32.7482809375 2.69743274896781 4.42017592281074e-46 4.56121927217901e-45

PCDHGA1 6.11415282485876 2.94898114583333 -1.05193601458876 9.43006623564095e-10 1.60065797947735e-09

CACNG2 9.65263804143126 1.71491776041667 -2.49278388812958 8.47931715737111e-63 3.66681168700734e-61

CA2 86.8020421374765 253.686437708333 1.54724550419796 1.3320847549188e-49 1.74806746772866e-48

CTSK 3.97747561205273 11.5896948958333 1.54291759742782 7.57834362363904e-35 3.99884505339296e-34

NEAT1 10.7303306026365 32.16630390625 1.583855645169 3.64704572249561e-39 2.42636190374976e-38

SLFN12 1.45563526365348 3.9463725 1.43887823228392 6.85773292313782e-43 5.7415373122804e-42

TOP2A 17.3915345103578 62.7989390625 1.85235495432321 1.38049692783465e-32 6.52359348744227e-32

STX1B 74.7766722693032 28.0821713541667 -1.41293377821328 3.64143012913944e-54 6.86042484816088e-53

ZBED6CL 0.723829802259887 2.58925765625 1.83881612037102 4.63030585388146e-29 1.83231618116463e-28

LMNA 33.6283712806026 71.8513683854167 1.09533672593337 1.14774063386735e-59 3.6173283197904e-58

PLAT 7.27150579096045 47.1399171354167 2.69662316757834 1.19706430200901e-75 3.77278147387416e-73

AC141557.1 7.14816473634652 16.2824102604167 1.18766948719099 1.13556204729665e-13 2.29469422619878e-13

FBXL16 176.883605367232 65.9081915104167 -1.42427064733216 9.97280581230451e-41 7.2952133784344e-40

MTRNR2L8 5.01878041431262 10.4738978645833 1.06138971099206 0.000118689568476032 0.000157465220163514

SMAGP 0.864890725047081 3.0134671875 1.80083458440587 1.48857214065574e-57 3.78659356436299e-56

FBXO4 8.30531059322034 16.621921875 1.0009811736611 8.18148728687459e-56 1.78771746297806e-54

ROR2 0.751690913370998 1.98079442708333 1.39786769129067 8.84764589671608e-38 5.46040409722654e-37

EFEMP1 105.067375094162 290.6829828125 1.46813185394803 9.42093653603208e-31 4.05608508653662e-30

IGHV3-33 0.276810169491525 4.62882369791667 4.0636767634828 6.56869762169481e-16 1.45151434670725e-15

RPS3AP26 7.02188210922787 33.4039502604167 2.25008904193773 8.88503656658922e-20 2.32798724750918e-19

LCK 0.455386911487759 1.81361848958333 1.99370627240369 1.24986099090305e-43 1.08705168970263e-42

H3P6 20.3898292843691 115.27090421875 2.49910680370003 5.53504315330578e-36 3.10761254334906e-35

CDCA3 3.50153112052731 8.27855625 1.24139327914801 4.83013065722787e-34 2.44999822643e-33

OTOG 1.59825536723164 0.350715052083333 -2.18812668367419 7.37483803713912e-15 1.56600563321459e-14

PTMAP12 0.727762758945386 2.61750432291667 1.84665178719597 6.23351447559868e-32 2.84936582285539e-31

AL034379.1 1.62198126177024 4.94588963541667 1.60847289454795 3.44504923129396e-27 1.25338858258484e-26

AC097450.1 2.02238385122411 0.549480885416667 -1.87991564805025 2.55749444693313e-37 1.54806670705474e-36

SNRPEP4 2.63975037664783 17.6387021875 2.74027099960627 2.28912058841412e-38 1.45824588357522e-37

SLC25A5P3 0.347934133709981 2.0980259375 2.59214638870918 4.65885685823175e-21 1.29648972281981e-20

LRRC32 5.79880762711864 13.5214746875 1.2214243209005 1.55837420160812e-31 6.96920834028451e-31

BTBD19 2.80190828625235 7.099129375 1.34123237245091 1.75251627734817e-51 2.70218345434676e-50

SUSD2 2.16910739171375 7.65808828125 1.81988280936555 7.93170193789899e-59 2.27608020887703e-57

OSBPL3 9.3950459039548 19.7977836458333 1.07536681445046 1.86496846589639e-32 8.7551347193495e-32

TP73-AS1 3.11039811676083 11.6516013020833 1.90535708520575 5.93252302610075e-57 1.42526183036619e-55

CEACAM4 0.52334868173258 1.53207786458333 1.54964525166456 2.18990327624638e-28 8.42218230026917e-28

IQSEC3 16.5575225047081 4.53334744791667 -1.86883817652507 8.56772507398679e-35 4.50811680109746e-34

CD7 0.882427354048964 2.41693770833333 1.45363087272339 2.05622485235321e-45 2.01876985900253e-44

CENPV 11.4606100282486 24.5807328125 1.10084408838749 1.15261208308431e-12 2.22355241051486e-12

USH1C 45.8564467043314 12.4727661458333 -1.8783431144714 2.15245557226101e-44 1.98045083454693e-43

CYBA 31.0397325800377 81.092783125 1.38545739893312 2.05115700399475e-54 3.9402132736862e-53

IGHV3-74 0.277232344632768 3.131291875 3.49759050040515 3.96380461809948e-15 8.50824735929353e-15

SELENOTP1 0.522110593220339 4.17645833333333 2.99985270972545 2.89605480650341e-35 1.55732039117787e-34

AC026401.3 7.7934379472693 29.9226442708333 1.94090587708711 8.09308067552885e-76 2.67909538469317e-73

EIF4A1P2 0.474533474576271 2.4889996875 2.39098428253358 1.512551560528e-35 8.26502975845376e-35

PPP1R3B 5.07684515065913 13.2679285416667 1.3859389851294 8.37238875684436e-57 1.97820290893927e-55

AC064875.1 0.405134745762712 4.78736177083333 3.56275710561916 1.34022592629103e-57 3.43271365005258e-56

MXD3 4.19004274952919 9.78539598958333 1.22366527084935 2.89148576888316e-28 1.10609294121338e-27

SLC10A4 4.19763761770245 13.0564613020833 1.63711440647807 4.58140440147338e-16 1.01830283104707e-15

TSHR 4.8663918079096 2.21833890625 -1.13337269092713 3.64429300077845e-30 1.52625289075395e-29

C15orf65 3.74606101694915 7.59326203125 1.01934539529675 5.32707387315265e-42 4.20804156856783e-41

DUSP5 8.84958177966102 23.6758241666667 1.41973346553837 2.39420048679506e-37 1.4515865031612e-36

IGLV3-21 0.74007368173258 12.1730217708333 4.03987461717455 3.44048205389306e-19 8.77582493719362e-19

CA9 1.50434689265537 24.7149775520833 4.03817640949108 1.36439818660813e-31 6.12086472375831e-31

IGLV6-57 0.45503418079096 5.96252692708333 3.71187705103805 7.03163235768326e-13 1.37129736435365e-12

RIPPLY2 1.76281365348399 0.78712390625 -1.16321731319511 3.04624563846767e-46 3.16806138967038e-45

LINC02100 0.248668549905838 2.15016119791667 3.112148862077 2.71496808275199e-48 3.27610846844732e-47

RAB5IF 1.16828413370998 3.19481348958333 1.45134052137544 7.8441459522414e-37 4.62324918642247e-36

RPL31P17 1.19752321092279 2.62140411458333 1.13028615690321 1.72006510512758e-18 4.25327269013928e-18

AL161909.1 1.50235607344633 3.10126354166667 1.04562934309602 2.91669681639176e-14 6.03091040818467e-14

MAEL 1.78518083804143 4.48483213541667 1.32898376090655 7.61232666199105e-29 2.98694269423346e-28

MKX 9.42810282485876 3.6475309375 -1.37004727821208 9.08589048785257e-43 7.53914027762689e-42

IQGAP1 16.7426900659134 48.6189985416667 1.53798883104891 1.75114940204412e-66 1.2150232511571e-64

RPS26P31 0.822110404896422 2.22926020833333 1.43916096564599 7.13555283551952e-24 2.24131089487307e-23

H3-5 1.07383064971751 3.33842828125 1.63640255885486 1.20918325489748e-29 4.93195056477706e-29

DUSP6 25.5049520715631 67.7911824479167 1.41032024556956 1.42674786131657e-43 1.23742894612576e-42

CXCL8 5.70764435028249 59.8954141666667 3.39147820193725 7.80873341485845e-26 2.67705379515658e-25

MTMR7 6.87696238229755 2.73194291666667 -1.33184411476782 3.04328183514252e-51 4.61580960232261e-50

RHEBP2 1.43646897363465 3.16296010416667 1.13874852624648 6.06853195279421e-27 2.17972477616783e-26

RPL29P12 0.837426177024482 2.24609864583333 1.42338736952382 8.02252247391503e-22 2.30356401177347e-21

SKP1P2 0.27437170433145 2.72897328125 3.31415465893026 9.05649192757856e-33 4.31587563796318e-32

GABBR2 75.9639617702448 36.0111134895833 -1.07687293934866 1.61521564805674e-34 8.38262209757608e-34

MMEL1 0.800776930320151 2.08848963541667 1.38298766672249 2.03628718740929e-30 8.62138069863995e-30

RARRES1 1.46985748587571 15.406800625 3.38981911657538 6.28318784450414e-43 5.27237716464596e-42

ZYX 97.125616007533 282.295827760417 1.53928405760477 2.41512687547177e-63 1.09802650976522e-61

AC015845.2 1.82930056497175 3.76229151041667 1.04031949835295 9.81783232342032e-24 3.0662175353376e-23

CKMT1A 4.87077306967985 1.87923979166667 -1.37400160238027 1.50301997897324e-39 1.02151522328243e-38

AC098614.2 1.13048653483992 3.49205776041667 1.62713361374246 1.28188085800723e-54 2.5091131110152e-53

SPATA18 0.716306355932203 4.52474442708333 2.65918765763992 2.2160668135286e-30 9.36114547877427e-30

AC022432.1 1.28060254237288 4.0297609375 1.65387147525374 3.30159871796741e-40 2.32902989987117e-39

RIBC2 0.613544444444444 3.03864354166667 2.30818768464308 3.06479479361912e-23 9.39496524684265e-23

H1-4 0.75046252354049 3.89001552083333 2.37392397773655 4.09701950963211e-21 1.14304692845625e-20

SLC47A1 1.45954307909605 5.36531791666667 1.87814686571155 1.5713274492155e-47 1.79036972537759e-46

ALDH7A1P1 0.318718455743879 1.82043192708333 2.51392632640661 7.50919151262418e-34 3.76674982943746e-33

OAS2 7.35384213747646 25.33164671875 1.78437075236269 2.31391400000296e-44 2.12690625540632e-43

AC006262.1 1.84220885122411 0.50661078125 -1.86248694437248 9.40225689827436e-44 8.25082430502179e-43

AL645608.2 2.97505729755179 1.0765425 -1.46651217831345 5.13184388014748e-44 4.58640151588347e-43

RN7SKP203 0.155516242937853 10.2525326041667 6.0427712504581 3.68601113278272e-29 1.46455933790801e-28

ANK1 6.85658403954802 2.75384755208333 -1.31604130579815 5.53299625878152e-39 3.63426582239641e-38

ALG6 5.62723846516008 11.825151875 1.07135970596603 1.95303000581195e-60 6.62711550329803e-59

C5orf49 4.1940516007533 15.3293138541667 1.86987660627798 1.80089248523668e-11 3.29278227757877e-11

SMS 67.0886406308851 158.466191770833 1.24003466225597 5.0854253284152e-66 3.32970014020707e-64

RPL29P33 0.665752259887006 1.97454520833333 1.5684630737327 4.50717815662665e-24 1.4290021794113e-23

E2F7 0.773472128060264 4.80969453125 2.63652405938065 4.95499247883513e-54 9.20466898975142e-53

CDK6-AS1 0.298160310734463 3.84221505208333 3.68777813731658 3.47554883747725e-41 2.61017086562558e-40

VWF 35.5183109698682 71.7830090104167 1.0150794218091 3.31069162357109e-29 1.31881556855836e-28

AC034102.1 1.3184733992467 5.18964104166667 1.97676628883918 1.45032292791217e-51 2.24927062923493e-50

CHST1 51.1521649246704 25.20615328125 -1.0210193359079 1.36746579011279e-32 6.46365693115082e-32

KPNA2 21.7396737758945 52.9053859375 1.28308430924385 9.20849037648741e-52 1.4462151904627e-50

AC145098.1 1.38706807909605 3.84836880208333 1.47220846529684 1.93183814321682e-39 1.30817663048495e-38

RGS3 4.07870174199623 9.13628036458333 1.16349691041819 2.17263620666456e-57 5.41557242130395e-56

ANTXR2 3.95117302259887 10.42780078125 1.40008199747245 5.30217212313207e-54 9.82010862845028e-53

AMER3 9.64309185499058 1.9744715625 -2.28802920110389 8.2190809767422e-60 2.61277620383561e-58

CTBS 0.951310451977401 2.22754807291667 1.22746843286941 3.29943790508052e-60 1.09363721648792e-58

PSMB8 30.5618471280603 73.6229027083333 1.26842289047069 2.39367671093538e-54 4.55582583826442e-53

AC004057.1 0.475846186440678 5.76753864583333 3.59938855172114 3.03247035150337e-29 1.21083929968231e-28

ADAMDEC1 1.08031421845574 5.52355 2.35414479557922 3.29791595824039e-34 1.68706319789491e-33

ITGA2 4.09317170433145 11.3919515625 1.47672382449514 3.83940914889245e-42 3.06542778547252e-41

AC026790.1 2.54790564971751 0.647147708333333 -1.97714491175109 6.32585475993083e-42 4.97585741374424e-41

ADGRF5P1 2.83555301318267 1.36606729166667 -1.05360157733721 7.11278127951029e-27 2.54546127583706e-26

CCL20 0.324153389830508 3.71005145833333 3.51669063205618 1.61099174028677e-38 1.03583649414359e-37

FUCA2 15.4052467984934 45.23447546875 1.55400094717349 1.40567138309625e-76 5.68227377579886e-74

NME2P1 1.52062796610169 9.49905770833333 2.64311717819597 1.65021047221866e-47 1.87794759675067e-46

RPL22P1 8.34342316384181 19.1627472395833 1.19959308244724 3.73130756178089e-23 1.14042840419651e-22

NAPB 89.4783044256121 35.5555658333333 -1.33146250263493 1.30027863843217e-20 3.54838292950487e-20

HS3ST3B1 0.739502354048964 7.69635182291667 3.37954810549805 1.15044285091116e-57 2.95884990493679e-56

GLT1D1 8.83407109227872 3.43599015625 -1.36235253921517 1.78115901712982e-13 3.56365955708296e-13

HAMP 4.08843229755179 18.683640625 2.19215594530997 1.53899883910717e-46 1.6343622737406e-45

LINGO1 127.739895574388 63.3174903125 -1.01253319798856 7.92033870247572e-39 5.16767362008898e-38

HOXA3 0.12557895480226 3.08233489583333 4.61735700426155 1.76521064607915e-68 1.56305199827818e-66

ECM2 9.5181606873823 29.8000846875 1.64656171495625 1.68831391974309e-45 1.66990411370334e-44

AFF2 5.62500532015066 1.76015927083333 -1.67614848149382 5.69427893884221e-59 1.65445495105892e-57

ENSAP2 0.633095856873823 3.78534947916667 2.57993064257707 5.97836819266395e-36 3.34641049195022e-35

AC126564.1 1.77371553672316 0.428080677083333 -2.05082003196947 4.16254742766338e-23 1.26931074807151e-22

ZNF236-DT 3.20888921845574 6.4470740625 1.00657057308878 3.69679358109842e-61 1.34524220431556e-59

FP565260.3 0.84670527306968 2.06517208333333 1.28633022227322 5.89569437002623e-28 2.22058814686323e-27

AC243562.1 2.90575188323917 1.41660234375 -1.03647668485535 2.56051457156444e-29 1.02680113129698e-28

AC018450.1 0.704534510357815 1.73053791666667 1.29647827057807 8.11675218652783e-35 4.27566591808739e-34

SLC17A8 10.4680563559322 2.22280692708333 -2.23553904993818 2.77705242507439e-14 5.74822897323818e-14

AC007938.1 5.47703808851224 2.57166776041667 -1.0906916435517 1.91393811377455e-36 1.10015326419676e-35

WDR11-AS1 4.98138064971751 2.47182213541667 -1.01097072288667 6.1834944241947e-26 2.12850941906517e-25

KIF18B 7.18034952919021 14.77628328125 1.04115745089445 2.20354631525775e-16 4.97691530817659e-16

SNRPGP2 17.170902306968 52.1779102604167 1.60347331203696 9.72131114227405e-52 1.52289621474799e-50

TTPA 6.82945037664783 3.39555421875 -1.00812240853463 1.03472193716393e-29 4.23523099748256e-29

MPZL3 0.683522033898305 1.74378369791667 1.35116134654912 2.69985073035329e-54 5.12806172940954e-53

RNF122 16.6845913841808 33.94303328125 1.02459914100076 7.95139931782513e-27 2.83956731928093e-26

MT1A 2.92553418079096 9.59734697916667 1.71393557867847 2.70437753320141e-29 1.08332400322879e-28

SLA 10.2452075329567 23.6547983333333 1.20718365262129 6.12257573066074e-37 3.62808463070862e-36

SLC22A6 8.78024896421846 2.723508125 -1.68879567684631 6.97168431952395e-53 1.19813742995885e-51

AL049873.2 0.53629213747646 1.7234365625 1.68419718849309 2.48379433800289e-22 7.32183825541594e-22

PLOD1 50.4288040960452 115.7149234375 1.19825501914812 1.93860929394157e-64 1.0212022612137e-62

IGLV3-1 0.83945263653484 20.8265327604167 4.63282993866801 2.09649069533107e-18 5.16963857309127e-18

ALDH1L1-AS2 4.44779289077213 11.67245171875 1.39194610473995 2.23281276856702e-16 5.0399791323461e-16

AL596087.2 0.34101313559322 3.23586458333333 3.24625201708509 1.79232795143614e-36 1.03439907687632e-35

CPEB3 12.1792383709981 4.54129197916667 -1.42324921517394 6.52135222605107e-68 5.38953531748532e-66

AC090013.1 0.55946247645951 2.39162307291667 2.09587675732591 8.81745662864869e-10 1.49845189188194e-09

C5orf34 1.74054990583804 3.8041596875 1.12803462732623 1.92730392519745e-40 1.37892329700064e-39

CFAP100 0.296252165725047 2.12891453125 2.84522042574916 2.98610808610282e-20 7.98945033972402e-20

FAS 3.18742876647834 10.070085 1.65961085525275 4.10827989674963e-48 4.89387986419343e-47

FTH1P16 3.98374745762712 14.1205341666667 1.82559656704428 1.12862988213085e-43 9.85299185832073e-43

SPP1 529.231645150659 3654.87680875 2.78785154496738 1.43133912062461e-64 7.62628966991823e-63

SLC6A15 4.52882165725047 1.91278307291667 -1.24346245973468 5.79512661357311e-13 1.13384237562492e-12

GBP2 15.4489939265537 43.94964734375 1.5083386994669 3.02420953196355e-38 1.91536703838087e-37

SNTG2-AS1 1.42947919020716 3.99337 1.48211713107669 0.000761810250508956 0.000963795183576952

FAM177B 0.695353013182674 2.37842682291667 1.7741901497658 3.63011048324201e-50 4.98905428203143e-49

CHGA 103.701428813559 29.56651875 -1.81039948059449 2.12758597259244e-41 1.62274245940756e-40

ATAD3C 1.02268629943503 3.45033140625 1.75437126203874 6.34324776026735e-37 3.75525922006276e-36

TYMP 7.14084030131827 30.5029683333333 2.09478388330504 2.94294171187818e-64 1.49111719706743e-62

IPCEF1 7.78339307909605 3.202858125 -1.28103932498238 2.4762960937973e-17 5.81765329932543e-17

LCP1 20.5007858286252 41.3253810416667 1.01134891033378 1.19857082027498e-34 6.24647544927501e-34

RPL15P3 16.5124279190207 41.5795571354167 1.33232212835595 9.46527010902599e-21 2.59635193505441e-20

PTPRT 22.6815445386064 5.67468630208333 -1.9989063391952 2.9606074707657e-65 1.77017671764914e-63

ATP8A1 40.8599898775895 16.43757515625 -1.31369135592385 5.30254802145498e-58 1.41667931693901e-56

TET1 3.22378898305085 1.45718005208333 -1.14557816293052 5.20957495626615e-50 7.07095228553059e-49

NID1 33.6641187853107 76.6699141145833 1.18744886337753 1.04710606358918e-20 2.86801255743715e-20

HTR1D 0.679669020715631 2.10392828125 1.63018125540175 1.25900839535119e-14 2.64444381696096e-14

CAPG 42.3349215630885 174.4579096875 2.04295888767752 6.40232844304546e-76 2.16456904360782e-73

DCDC2 0.639310075329567 2.08951119791667 1.70857775467755 5.61975217854184e-24 1.77357928988434e-23

TMSB4XP2 1.047606826742 17.7878277604167 4.08572106896646 4.88688581716684e-61 1.74083604923788e-59

AC004453.1 13.0675089453861 47.7364579166667 1.86910737436832 9.45278157036335e-23 2.84286710821456e-22

HOXC11 0.132887711864407 2.32168604166667 4.12689328198748 6.87351348330625e-65 3.89673729335609e-63

IFNGR2 34.6794804143126 81.9863754166667 1.24130189752059 2.94761091033722e-67 2.24488001059927e-65

TENT5B 1.25646092278719 7.26088791666667 2.53078018011198 6.81167945350949e-55 1.3546864111017e-53

CAVIN1 46.2444527306968 174.742970104167 1.91788219512263 4.50657550913039e-65 2.61874286225874e-63

ECT2 6.57010404896422 18.31596375 1.47911349140709 1.39402858643771e-42 1.14496296664352e-41

IGFBP6 12.9842839453861 35.0998668229167 1.43469910185727 1.31450979991322e-16 2.99770784024851e-16

STXBP5L 6.45251073446328 3.02971057291667 -1.09068065622402 4.68805461630795e-17 1.08818344264444e-16

KIF9 3.32099063088512 7.200675625 1.11651862535479 3.11176130639885e-60 1.03511988358652e-58

UBL5P2 1.51587080979284 6.80292723958333 2.1660088539579 2.26946222425758e-24 7.27722884291597e-24

CDCA5 9.83263267419962 21.627615 1.13722492683017 5.31685008465789e-22 1.54118203155438e-21

RNVU1-27 0.70047302259887 3.09869140625 2.14525769222041 2.32390690144332e-18 5.71903769618032e-18

ACADL 0.585782062146893 1.854080625 1.66226806044487 1.67957334846887e-18 4.15591036790135e-18

RPL7P1 7.76981271186441 16.83543734375 1.11554947076867 1.27474182038388e-14 2.67718818048772e-14

PGK1P1 0.307848587570621 2.57154359375 3.06234175649108 4.78165700651119e-25 1.57734454561071e-24

IGLV2-14 1.31712947269303 15.2167505208333 3.53019123633785 8.36451043496656e-25 2.72921690714517e-24

ATF4P3 0.538938559322034 2.14444682291667 1.99241282534987 1.7588593942019e-39 1.19234380004318e-38

Z84468.2 2.73826459510358 1.24436286458333 -1.1378546127596 6.85842357133082e-32 3.12656009582978e-31

ACE 2.4175463747646 6.51014453125 1.42914600834184 9.33999857326122e-29 3.65098325561893e-28

CEROX1 309.330862806026 121.261725625 -1.35102652424241 6.47444102243731e-48 7.61494186035558e-47

AC096533.1 0.727077165725047 2.01748369791667 1.47237662334871 1.3458715897919e-33 6.67650373530104e-33

SOD2 42.9426656308851 231.78360125 2.43229484570114 1.11133872544636e-64 6.11400698215239e-63

DRC1 0.736716760828625 6.58700671875 3.16044105122879 1.08477009773241e-31 4.89357107407428e-31

KIF26A 12.897247645951 5.29613005208333 -1.28405276367067 1.42675324576156e-43 1.23742894612576e-42

CROT 14.4874437853107 31.2079709895833 1.10711149912361 5.16155978362934e-52 8.27406932556789e-51

GGH 4.15264802259887 11.664014375 1.48996090320034 1.20866615611932e-54 2.37079611529945e-53

AC092115.1 1.0181368173258 3.63324375 1.83532671375132 4.20001042331637e-24 1.33297821849408e-23

LITAF 46.244174905838 93.9295441145833 1.02230735746286 3.85568229470824e-56 8.66945734825873e-55

AC148477.1 3.51518644067797 1.28533770833333 -1.45145374843363 2.80673898437652e-34 1.44095282756713e-33

CYTL1 13.8018635122411 52.6103127083333 1.9304825540429 2.4715792840589e-55 5.14082961824108e-54

MT2A 1035.55955894539 2216.48861130208 1.0978654189173 1.62187199844182e-32 7.63898424798015e-32

CLEC2B 3.0015897834275 10.3926955729167 1.79177116973694 1.46527461209465e-52 2.44804864437556e-51

VGF 56.9231981167608 172.45365515625 1.59912008284499 0.00182379536963671 0.00225353677310085

FAM111A 8.45988469868173 20.2967877604167 1.26254151375012 1.57109862758975e-59 4.86099483860755e-58

GPR157 0.451411629001883 1.76223958333333 1.96489458761563 3.73638076138464e-64 1.86768817897708e-62

PABPC1P4 1.67809882297552 3.4612953125 1.04448435695799 2.3172263255243e-14 4.81546977236526e-14

MARVELD1 8.10632744821092 26.040345625 1.68362823893553 1.82903589620613e-59 5.63094743211142e-58

PNPLA4 5.77931836158192 16.2314944270833 1.48982458431992 1.24368912484327e-32 5.89357779726315e-32

SYNPR 18.4122576741996 6.65572921875 -1.46799789263852 1.72657366561531e-14 3.60859135799895e-14

CHAF1B 2.47641120527307 8.41708859375 1.76507041012946 9.93200189936987e-48 1.15428484574239e-46

MTCO2P2 4.78394416195857 19.222968125 2.00655865717736 6.37643309310447e-16 1.40986650851698e-15

TAGLN2 75.5188365819209 557.05351015625 2.88290747415775 1.40292331664753e-78 8.4152771203422e-76

AL592114.1 0.92570918079096 2.12722291666667 1.20034028943298 3.28678576995898e-17 7.6858377002499e-17

SPRY2 51.5705407250471 131.724764166667 1.35290751917736 1.73881362537215e-50 2.45320480757171e-49

PYDC1 1.63230155367232 0.704239947916667 -1.2127686361138 1.45325382059156e-09 2.447536889222e-09

RIN1 3.22529552730697 9.33664526041667 1.53347291207478 5.96223020778409e-38 3.71415982290603e-37

AC005790.1 0.458336299435028 1.70525046875 1.89550520490261 2.30107340241826e-49 2.96524323755839e-48

AC007098.1 0.847932580037665 2.72580442708333 1.6846605900222 8.10158412945307e-32 3.67705533041689e-31

CACNA1B 6.76194100753296 1.98797135416667 -1.76614046151905 3.90976124544161e-37 2.34371406702085e-36

AC011005.1 0.76983290960452 2.23715171875 1.53904584876307 5.63287713042716e-32 2.58052107022156e-31

RBFOX1 8.03311779661017 2.29831833333333 -1.80538139688133 8.56472253684549e-16 1.88185059166539e-15

COL5A3 20.0523994821092 44.208306875 1.14054260110379 1.15061405400256e-29 4.70027863228858e-29

IGHG4 0.520591337099812 7.02133515625 3.75352218533563 5.11848702175566e-20 1.35388714323679e-19

SERINC2 4.02666958568738 14.4618497916667 1.84459309708267 1.40779514132018e-50 2.0136885117576e-49

GPR45 4.28513168549906 1.81431484375 -1.2399147071926 1.13960897402789e-36 6.64712323464511e-36

PRRT1 35.3079186440678 16.8282731770833 -1.06910463560838 6.971157441849e-43 5.83387365576877e-42

MGP 20.0609964689266 89.3269183333333 2.15470172241512 1.60835581552009e-49 2.09582175119804e-48

TRAV8-5 2.28071172316384 0.734664583333333 -1.63432647270767 9.33613819944142e-23 2.80824150466861e-22

TPTE2P1 4.8784329566855 1.81759427083333 -1.42438760924653 5.47037057070676e-51 8.10530205277229e-50

PROM1 4.22017810734463 11.6408338020833 1.46381860589888 1.55443205656014e-16 3.53357751732712e-16

PRKCG 25.4127965630885 7.32563807291667 -1.79452881538177 1.64164731757716e-18 4.06531254099711e-18

RPS3AP6 6.42178874764595 39.1439647916667 2.60774277844808 1.14865946134928e-19 2.99485735891613e-19

MTARC2 11.5507789548023 24.52458125 1.08623835649976 1.39783381704265e-26 4.94628350673798e-26

C16orf54 0.994992467043314 2.42935078125 1.28781331172498 2.50127453983229e-44 2.2934516798906e-43

AC092819.3 4.20467622410546 1.44150989583333 -1.54441397097336 2.87125688295264e-32 1.33544326509516e-31

IL1RN 1.2054713747646 4.32194453125 1.84208316348649 1.36757584922936e-31 6.13364035610708e-31

CTSW 1.10518531073446 3.18484963541667 1.52693696870122 3.99921152018284e-43 3.38794251561731e-42

TRMT112P6 0.687346657250471 4.21604119791667 2.61677916636214 3.52514491598422e-40 2.48201702812293e-39

TMIGD3 16.3552566384181 40.3319948958333 1.30217036826173 6.51509828868764e-32 2.97588436939687e-31

GAS2L3 1.09990098870057 6.59169135416667 2.583275031589 1.62168232809651e-60 5.55344067973381e-59

TPI1P1 4.04578573446328 26.8106752604167 2.7283157342142 2.58825590559719e-33 1.26554348053063e-32

GNG3 17.1844358757062 8.13656015625 -1.07861158309017 0.000402813155487636 0.000518253001196471

RNVU1-3 2.01541487758945 4.06868057291667 1.01348416937799 1.01758392021524e-30 4.37199006386377e-30

ANXA1 20.5437251412429 234.956845208333 3.51562608898023 4.74723425004286e-81 5.88498805863647e-78

C1R 25.9963494350283 153.409701614583 2.56100877101297 4.95087364346326e-71 7.0275950687175e-69

DAPP1 0.956325564971751 2.18474817708333 1.19189325021318 3.60933530566385e-34 1.84383489035218e-33

HMOX1 50.3853694915254 196.720043385417 1.96506717704962 2.2990256137527e-50 3.20467625845063e-49

AC027307.2 5.61798102636535 14.3627434375 1.35420768849032 4.3042534400099e-69 4.30309638263355e-67

FGF13 4.9962711393597 2.05758401041667 -1.27990043517058 7.05355524311591e-41 5.21721796920208e-40

CRISPLD1 30.254534086629 87.41192265625 1.53067870365683 7.09688395255564e-47 7.72637922118104e-46

HOXA11 0.866738983050847 2.60225317708333 1.5860918319875 6.33827129682756e-36 3.5414709965297e-35

ADAM22 49.6156126177024 23.07036453125 -1.10475336701984 3.66668362971662e-57 8.92434320609693e-56

ABCC8 16.1505951977401 4.68871744791667 -1.78432208660633 2.33197564631083e-48 2.82311765254882e-47

NXT1 34.5229298493409 75.373880625 1.12650976518347 9.87239420555548e-53 1.67803629115452e-51

DGKI 9.62868695856874 4.8014703125 -1.00386281710061 5.21649578266628e-43 4.3971323245095e-42

CSAG1 1.26305353107345 2.9494796875 1.22354468852781 7.31348925062279e-29 2.87271509538088e-28

MLLT10P1 3.74693455743879 1.86464786458333 -1.00680757449056 3.06144819626718e-09 5.07195556036958e-09

RPL9P3 0.399906355932203 1.74692375 2.12708252266039 2.77024177666355e-26 9.68282816486066e-26

COL22A1 3.83950084745763 12.50317421875 1.70330373140747 6.09472312863767e-31 2.64916728791532e-30

EMB 3.70094463276836 7.8641584375 1.08739883498341 1.30513308446114e-20 3.56110780712471e-20

AC022018.1 1.52819359698682 3.724058125 1.28504826994047 7.68077325314547e-21 2.11497080767422e-20

SERPINB1 15.4398753295669 42.8425965625 1.47238481669483 7.47316581060792e-62 2.95667060102669e-60

MMP12 0.549141101694915 2.07754442708333 1.91963052634704 4.27529420892844e-13 8.41260273174861e-13

OLFML2A 4.29162495291902 12.5364615625 1.54653429354653 1.92185404549545e-42 1.56466182031471e-41

ACTL6B 52.2855880414313 17.7148266145833 -1.56145599291465 7.87759121410383e-46 7.99584108221947e-45

GDPD2 11.273404519774 31.80624765625 1.49638691087177 1.99645157045775e-28 7.68931585597802e-28

PSTPIP2 1.72362062146893 3.70089145833333 1.10243056002144 4.79693840484346e-35 2.55292128328747e-34

DUSP23 22.9936258003766 55.6834833333333 1.27601548464185 4.94794314214157e-52 7.93848168491135e-51

TESPA1 13.1237870998117 2.165385625 -2.59948821826372 0.000580038755653361 0.000738653654388046

DNAJC19P9 0.458125847457627 3.01421557291667 2.71796673325453 1.00700912092779e-34 5.2717721294066e-34

MELK 3.41871313559322 16.5852764583333 2.27837778412479 1.32077844664937e-43 1.14712168217866e-42

NTAN1 5.19802542372881 11.1905283333333 1.10624255601499 2.28512491295558e-75 6.74474567562048e-73

CD300LG 1.49687547080979 0.624514635416667 -1.2611469179107 2.87141084488467e-20 7.6903189774781e-20

AP000357.2 3.48549971751412 0.56290015625 -2.63041455691592 1.44298994703613e-22 4.30558377168434e-22

C1QTNF1 26.5613664312618 66.15781828125 1.31658229178332 6.98138627650353e-20 1.83697294200627e-19

AC007344.1 0.930916054613936 3.39824234375 1.86806575749104 2.32080335498318e-45 2.2665618900164e-44

LINGO2 1.52136967984934 0.663828854166667 -1.19648751377536 6.28756139289723e-09 1.02442130991785e-08

AC083805.2 0.431051129943503 2.82272416666667 2.71115724461543 1.10552532890267e-35 6.08923089186764e-35

AL049637.1 0.176801224105461 2.720370625 3.94360304998655 8.97481006716911e-43 7.4502943392415e-42

MAB21L1 0.765597222222222 3.25119989583333 2.08631476051112 5.34485130263639e-36 3.00355122310437e-35

LILRA6 0.64263549905838 2.42992859375 1.91884133688573 2.49688180139933e-45 2.43341284575579e-44

AL022069.3 8.19553347457627 3.64689848958333 -1.16816781899456 1.88410590906564e-50 2.64814432192559e-49

Z80897.1 1.56181544256121 0.5464115625 -1.51516406476653 5.92227912473546e-37 3.51275216345952e-36

MTRNR2L2 2.3044013653484 6.7345309375 1.54718544723778 1.03422583314641e-12 2.00056479427416e-12

FHDC1 14.2819102636535 5.99165677083333 -1.25316207142241 9.59591701782353e-31 4.1285533768262e-30

MEIS3P1 4.58515315442561 11.9180144270833 1.37810206999092 4.89246492862109e-32 2.2501950370445e-31

AC148477.7 3.82399486817326 1.17747255208333 -1.69938715746873 1.82350748513065e-40 1.30566506299594e-39

SP5 0.785618549905838 2.784285625 1.82540631729808 3.82172607569573e-32 1.76646772004877e-31

IER5L 5.02659039548023 15.33552171875 1.60922521107375 1.16412961084576e-47 1.3428653916673e-46

KLK7 2.97087514124294 0.982196197916667 -1.59680483045346 5.76281237170681e-15 1.22889330334734e-14

VRK2 2.09193573446328 4.47321432291667 1.09647335133862 2.20383414554748e-33 1.08184519367622e-32

AC090602.1 0.539598352165725 1.80366885416667 1.74097664262932 1.73433329239017e-25 5.85404385042571e-25

CCNP 0.886904190207156 1.976941875 1.15642028660985 2.95869715537055e-16 6.63979031549467e-16

VASP 23.6924371468927 49.2216127604167 1.05486531837251 1.11509621349762e-64 6.11658232743016e-63

THBS1 5.40484952919021 24.83401125 2.19999094889882 8.15999928956873e-30 3.35920271838677e-29

ADAT3 1.14024533898305 2.3041515625 1.01489134464989 7.00625119083851e-32 3.19238522160358e-31

RPL23P8 1.82034712806026 5.48195057291667 1.5904757314221 8.85914839075481e-23 2.66865161714054e-22

UBXN10 1.67436647834275 5.57286776041667 1.73480458633499 1.39751306619066e-08 2.24294454219017e-08

CD247 0.57522302259887 1.47402505208333 1.35756772009707 7.35536972287375e-44 6.49765788809248e-43

HOXA2 0.19355593220339 5.5073140625 4.83052645270076 5.20767176947047e-67 3.82753583214638e-65

MT1DP 0.779401836158192 4.23525619791667 2.44201000789158 1.42787497770432e-29 5.79723476209865e-29

GGN 1.07339482109228 2.49240505208333 1.215357713814 2.12401328778424e-38 1.35538871264063e-37

MPZL2 1.03678474576271 3.53746942708333 1.77060128228228 3.08256062882094e-42 2.47924804900196e-41

HBQ1 4.35511407721281 1.76937802083333 -1.29946820183384 2.46326955047744e-36 1.4067720298258e-35

LINC01436 0.133712005649718 1.96971203125 3.8807838132324 3.83036649757396e-18 9.35579469622853e-18

RNF128 1.56123248587571 3.33452760416667 1.09479700460549 1.28498476459156e-05 1.79588813961519e-05

ADGRB3 43.5564517890772 19.97655890625 -1.12457834269721 3.0819611444642e-53 5.46842246959082e-52

MPP7 1.8629668079096 0.776402447916667 -1.26272539847268 4.78669857830571e-21 1.33087111339107e-20

AC016769.5 0.88259604519774 1.92831432291667 1.1275150478701 1.39166440813998e-10 2.44801813162075e-10

RPS27AP16 22.8447657721281 50.0656396354167 1.13195716167713 1.81769277659898e-20 4.92855018676845e-20

GALNT3 0.515239548022599 3.39010796875 2.71801598215244 2.76085285890736e-67 2.12140739303233e-65

OBI1-AS1 5.98268262711864 2.1475375 -1.47810920836112 2.19130244716473e-41 1.66997004118968e-40

CENPW 7.40445131826742 17.2111455729167 1.21687838770939 5.81122616707528e-38 3.62130531423475e-37

ATP5PDP4 0.941890207156309 2.53903760416667 1.43065095719494 5.15672136284422e-33 2.4828905681535e-32

TNFRSF19 12.9732094161959 40.0100031770833 1.62482531407424 2.5547517277033e-56 5.8432482628097e-55

PPP1R1C 1.71420296610169 5.2571671875 1.61674767755238 8.61304774852085e-28 3.21670260863115e-27

AMER2 81.3185375235405 38.4006811458333 -1.08245236771652 2.49787248563318e-47 2.81502659820297e-46

ITGA3 9.53628083804143 37.2524256770833 1.96583574106906 2.20159036497444e-42 1.78303888661584e-41

TREM2 119.521091525424 287.777814895833 1.26769014873706 1.44034326108539e-42 1.18143727127847e-41

AL669983.1 0.416254708097928 3.52856197916667 3.08354185598091 8.89489541809557e-34 4.44386298494055e-33

C7orf57 0.713752730696798 10.1339798958333 3.82763270168613 3.45904956958883e-45 3.3396171727157e-44

RAB32 17.7034631355932 68.5814583333333 1.95378697580241 1.13922259235279e-71 1.83921357367067e-69

NPM1P27 7.47066878531073 21.5942990104167 1.531341178742 3.11052400276195e-22 9.11587827813957e-22

CGAS 1.30721082862524 3.92218822916667 1.58516693376988 9.76110297881893e-62 3.80519307947878e-60

AC064799.1 3.12119383239171 7.56801677083333 1.27781733142132 1.70776873891317e-17 4.03660349562608e-17

CNN3 415.340876177024 943.419465572917 1.18360350531189 6.44090189575687e-57 1.54540091292386e-55

ATP5MDP1 0.549493926553672 3.32780067708333 2.59839358342936 4.3092017036439e-26 1.495513357209e-25

CD40 5.19903912429379 12.12362265625 1.22150393745122 1.06201942099479e-48 1.31392223109768e-47

AC024619.4 0.365873634651601 2.59447989583333 2.8260279947492 1.08023570537095e-36 6.30872579816985e-36

IGLV2-8 0.240414971751412 2.76414375 3.52323399815314 4.90501187545721e-20 1.29889911455606e-19

PRELID1P5 0.846146421845574 2.02576692708333 1.25948895364676 6.24281876511303e-38 3.88634800593494e-37

MIR9-3HG 54.6897020244821 20.1922305729167 -1.4374689121234 3.35811659891123e-56 7.58738495221802e-55

DNAI3 0.434553013182674 2.31670729166667 2.41447168185398 8.07484832449332e-22 2.31823073327086e-21

TBC1D1 12.4468970809793 26.4089869270833 1.08524282550984 1.50939362753072e-60 5.18801746837962e-59

SKAP2 11.2570763653484 35.6618902083333 1.66355098688014 5.77928386014098e-45 5.51106581432418e-44

FCGR1A 10.3640810263653 25.3943509895833 1.29291540340992 9.46553957149411e-34 4.72261090238618e-33

H3C10 1.27512288135593 4.18923130208333 1.71604925885804 2.42006677691773e-43 2.07474143461435e-42

KNTC1 3.53074708097928 7.19930286458333 1.02788373195278 1.39896002761706e-33 6.93327870829937e-33

ACSL6 14.4212661487759 6.52721671875 -1.14365798888853 1.28017899108647e-41 9.88576758274623e-41

HSPE1P2 0.941796563088512 4.64019307291667 2.30069747251475 4.56125505408923e-37 2.72022250579824e-36

HLA-DMA 33.3641478813559 98.1621109375 1.55686761595925 1.62720981102226e-57 4.11113674401616e-56

POLQ 0.557424011299435 1.67941510416667 1.5911118143238 8.95425357447143e-36 4.96139288490156e-35

FO393411.1 4.19804684557439 10.4265660416667 1.31247391807038 2.74789829494682e-20 7.37545738951156e-20

KIF4A 3.93844792843691 17.2405217708333 2.13010433052145 3.43188792323145e-48 4.09864842212516e-47

GSDMA 0.671879096045198 2.71831541666667 2.01643931733365 2.49613405803506e-22 7.35704752086891e-22

DPYD 3.39537057438795 20.5953965625 2.60068095997561 1.80822024843725e-72 3.6547669043142e-70

IQGAP2 3.66954119585687 14.8181817708333 2.01369683735415 6.26544751030814e-61 2.21073997066755e-59

RBBP8 12.6207860640301 25.6060639583333 1.02068373689033 8.28550654183168e-50 1.10841002982273e-48

IGHGP 2.20712043314501 7.22105005208333 1.71004328900576 5.9572594460371e-14 1.21744410813342e-13

MYO5C 2.13856016949153 5.59054135416667 1.38634819293008 2.90988954604645e-17 6.81564379754802e-17

CD70 0.146356450094162 3.78480572916667 4.69266101635053 7.70506152350317e-52 1.21833009378862e-50

GABBR1 286.194290018832 99.4887977604167 -1.52438889320519 4.0420743366123e-69 4.06283093455707e-67

NDUFA4P1 3.40128163841808 27.1152177604167 2.99495238011213 4.21084464687919e-31 1.84584290921072e-30

ST20 0.828181544256121 1.89499557291667 1.19417551976958 3.58633857186668e-35 1.91907815090247e-34

LRATD2 86.0597706685499 38.5476144791667 -1.15869741415198 1.82290910195416e-40 1.30566506299594e-39

TFRC 22.9921319679849 58.6144028125 1.35011496166767 1.95306841948446e-49 2.5273004356516e-48

PRG4 0.39506252354049 2.40400395833333 2.60528637069107 0.00261276258360882 0.00319801194965677

ART3 2.20454387947269 4.7591953125 1.1102374696469 2.67059646959558e-11 4.8391874246862e-11

BX284668.5 0.930647645951036 2.01128864583333 1.11181318671607 6.42689272135374e-31 2.79031827305575e-30

LINC00641 51.4626430320151 23.6458007291667 -1.12194155598581 2.92513096114153e-60 9.7478154520478e-59

HAS3 1.69908540489642 3.697375 1.12174300212418 2.78871866088499e-16 6.26511556564707e-16

GALM 8.9629945386064 19.8392679166667 1.14630606752808 3.57491231849802e-57 8.74677560032509e-56

PAWR 1.73091228813559 3.93437697916667 1.18460257882255 4.86531414408351e-15 1.0402497011525e-14

DDIT4L 1.80326605461394 21.5334713020833 3.57789673442392 1.57000732511694e-46 1.66634053713182e-45

RAC1P2 11.397518079096 24.7490375520833 1.11865272467821 8.12371930701141e-43 6.76794625958231e-42

RPSAP17 0.537227401129943 1.95781083333333 1.86563658027921 7.19241783993532e-21 1.98314071372475e-20

PAK5 9.98442876647834 3.53508333333333 -1.49793566551041 6.65747444545658e-54 1.22207045718919e-52

NEFL 119.479688276836 38.9321890625 -1.61773000744783 2.55431822036817e-11 4.63164771406593e-11

TEKT3 0.714595291902072 1.52531057291667 1.09390471001615 6.31561411834425e-28 2.37586171415358e-27

CABP4 0.651408615819209 2.31028380208333 1.82643538074901 1.34829336729749e-72 2.78572390721076e-70

CPLX2 140.967216290019 37.5546266145833 -1.90829712524785 3.97218085439425e-41 2.97234217253364e-40

LINC02009 0.562542984934087 2.10093911458333 1.90099910856629 3.30071154768389e-07 4.97460943663332e-07

NEIL3 0.963895998116761 4.56316526041667 2.24308550672924 7.71263594737329e-40 5.32214304505446e-39

ELF4 2.81968535781544 8.05328083333333 1.51404245921833 5.38438686984103e-54 9.96245510892477e-53

VDAC2P5 2.31012325800377 4.8849765625 1.08038181079282 2.60255830116464e-16 5.85961637125033e-16

TEX26 1.30916803201507 7.07092208333333 2.43324808249159 3.39823879017787e-46 3.52034820631518e-45

LINC02768 2.87339854048964 6.00129395833333 1.06251548978128 4.4330193324548e-35 2.3633025942373e-34

WFDC2 3.00949425612053 6.60798369791667 1.13468906435824 2.68880694929592e-28 1.03004460696658e-27

LRRC7 1.63621007532957 0.7746925 -1.07866231208526 1.26366972152896e-22 3.77906890667786e-22

ALDOC 1175.77774571563 539.638821041667 -1.12354933388644 1.34867145091598e-47 1.54805837220881e-46

AL138785.1 0.925417890772128 4.05615916666667 2.13193737047454 2.74103626028605e-28 1.04940434959891e-27

RNU6-353P 4.94048766478343 1.7031259375 -1.53646833520508 2.09778827529139e-34 1.08326500913756e-33

DLL3 419.600857862524 139.549864427083 -1.58823690464479 1.98887029894678e-46 2.09535655574591e-45

CLDN1 1.21756883239171 2.74167588541667 1.17105469514005 1.95789249576174e-31 8.70148445475372e-31

AC008026.1 3.07520480225989 8.21569072916667 1.41769937982847 9.62264554356876e-24 3.00626837840492e-23

SERPINH1 15.7937525894539 76.3256175 2.27281336066281 6.29727573872364e-77 3.00250877850169e-74

LINC00886 0.959067372881356 2.73396114583333 1.5112886691436 9.85026446059917e-40 6.761375697484e-39

VWA3B 0.889525988700565 2.14451229166667 1.26954092356842 3.7788707427419e-13 7.45395898191634e-13

GBGT1 2.81317509416196 5.74033536458333 1.02893567528801 1.70263724036344e-45 1.68317594282606e-44

AC104619.3 1.01693017890772 3.087394375 1.60216915144997 1.00999916844562e-18 2.52364076017822e-18

GCH1 2.34041172316384 5.5515253125 1.2461218646526 6.50044772416243e-45 6.18607090229275e-44

RPS10P2 1.12490809792844 2.51499692708333 1.16074949547672 1.41512880875414e-22 4.22584233158555e-22

RTN3P1 1.72859463276836 4.73771869791667 1.45459295427609 1.76658640155693e-15 3.8389241716666e-15

ECSCR 1.1016918079096 3.64414588541667 1.72586002091815 9.43211973936337e-44 8.27312578082367e-43

FAM181A 7.08722650659134 31.1030054166667 2.1337609284353 2.71199883195921e-56 6.18768322457442e-55

SLC7A7 7.7891247645951 20.0345936979167 1.36296012002019 4.22185354657542e-54 7.87419631155446e-53

AC083855.2 0.730504708097928 8.39558 3.52266451937542 5.02925596807549e-65 2.88638934340629e-63

TNNI2 0.88308225047081 2.37980609375 1.43022430557148 3.34864685959073e-47 3.74883132775976e-46

CMAHP 0.854726129943503 2.00601171875 1.23079590094555 1.57999027029053e-42 1.29256133198647e-41

MT-TE 2.85735814500942 1.26977026041667 -1.17011438315474 2.51399491325813e-19 6.45152296605505e-19

ICAM1 8.45780913370998 27.33610640625 1.69245185987183 3.14698401374163e-41 2.37011614967702e-40

CALU 50.3861202448211 142.638077239583 1.50126088319225 1.03972275620053e-63 4.98289810606929e-62

OSTC 48.46070673258 119.018522708333 1.29629876611269 9.56021283819932e-75 2.61429643715171e-72

MYBL2 8.24254524482109 47.8870439583333 2.53847357528364 2.56587614633031e-40 1.82247772883927e-39

PHYHIP 113.991128954802 38.9757288541667 -1.54827364801349 8.67141302688988e-19 2.17340511167297e-18

NTNG2 23.2772495291902 9.8084025 -1.24683050940715 1.59441710177748e-64 8.44677664032827e-63

AC093388.1 1.06587744821092 2.37152822916667 1.15377547109925 1.09416256327121e-39 7.48838898197576e-39

TGIF1 5.08512956685499 19.5261244791667 1.94104919442916 1.94419378047948e-85 9.03807083700398e-82

HLA-S 0.50083418079096 1.81819338541667 1.86010072322622 3.67509743460862e-20 9.78499954131548e-20

SLCO4A1-AS1 0.641942984934087 2.01173119791667 1.64792047584226 1.50632072841458e-11 2.76396624678006e-11

PSRC1 33.6261394067797 141.417518541667 2.07230579093169 1.53277935440667e-80 1.58344622751067e-77

CHRNB2 19.4487389359699 7.61767619791667 -1.35225374317066 2.55109679508045e-38 1.6206916605576e-37

RPS3AP47 2.96429825800377 6.65753322916667 1.16729711004473 1.81752163930518e-19 4.69661129556418e-19

RET 11.7290205743879 5.41385963541667 -1.11535315837151 2.01581046479252e-26 7.07512185594883e-26

MMP13 0.0580404896421846 4.6793425 6.3331023286768 3.04216899480598e-23 9.32714467574892e-23

CA3 0.96989143126177 42.9514121875 5.46873849347962 5.25811868147109e-81 6.11091980512218e-78

CD53 53.2986061676083 123.402287395833 1.21119942654309 1.49387939085506e-40 1.08004227344284e-39

ATP8B1 0.933583286252354 1.88733348958333 1.01549872984198 6.19242262670102e-28 2.33046141962164e-27

MFAP5 0.469548728813559 2.51174125 2.41934106022301 1.60973413389375e-05 2.23714545738074e-05

STK32B 4.97131986817326 13.6955806770833 1.46200959769042 1.47464129122713e-29 5.98188368463536e-29

AC005480.2 1.05606633709981 5.97839052083333 2.50105667908616 1.02013759626493e-28 3.98183429944298e-28

ECRG4 4.28808573446328 13.5002052604167 1.65457568660151 8.14368184687392e-26 2.78931228481526e-25

HOPX 26.9358492937853 108.995561145833 2.01666992278418 2.69961592856821e-27 9.85847568572778e-27

PIMREG 7.34206558380414 32.5347040625 2.14772152431675 3.35528411195006e-35 1.79958200351057e-34

HPX 0.691046280602637 1.38644682291667 1.00453804413503 2.12221931768727e-24 6.81330597589689e-24

CD3D 0.859832109227872 4.92143890625 2.51695329349448 1.40807333249841e-48 1.72711897215092e-47

FGF12 37.8106647834275 15.7632653645833 -1.26222679446069 1.79823556898231e-55 3.76980726101759e-54

RPSAP9 1.89489576271186 4.95687130208333 1.38731131319562 1.13747240501916e-45 1.13839070889835e-44

AL034348.1 1.87565861581921 0.786519947916667 -1.25384200902804 6.82452360613378e-44 6.05448551794168e-43

CCKBR 6.72655193032015 2.05249026041667 -1.71249178437062 2.36085737958507e-17 5.55557364887173e-17

CHST8 5.01953116760829 16.58323984375 1.72410136611513 0.000699245622544046 0.000886330766953411

NRP1 8.40262594161959 20.8486165625 1.31103948846332 3.53372916668608e-35 1.89256030687004e-34

VPREB3 0.956767702448211 2.16592864583333 1.1787451212101 1.6954199314332e-28 6.55024592250163e-28

ACVR1C 2.35441798493409 0.954744427083333 -1.30218396903251 9.76497149777278e-27 3.46993111621107e-26

PSMB8-AS1 3.8916827212806 9.43830484375 1.27813367345622 1.51504425634586e-54 2.93155545751834e-53

SEPTIN3 190.867389689266 84.6424724479167 -1.17311695967711 6.52135222605107e-68 5.38953531748532e-66

SKA1 1.73140579096045 7.67462916666667 2.14815315211 4.59059873994619e-42 3.65110371112602e-41

SMYD2 12.5071255649718 27.5501027604167 1.13930743802888 8.71928634922283e-34 4.35729991034127e-33

AC124798.3 2.25741252354049 0.60608875 -1.89706911413015 1.07710540236852e-46 1.15840225315458e-45

AP005432.2 0.75684077212806 2.72216734375 1.84669404246537 5.26692945346054e-42 4.16759800796165e-41

EEF1AKMT4 1.56284303201507 4.64636567708333 1.57192981624317 2.59340064950123e-61 9.64485701549507e-60

ITGA11 1.31869439736347 3.00190484375 1.18676798224797 1.66592329866026e-25 5.62824195831896e-25

BCL3 6.14245348399247 21.93453234375 1.83631701523807 4.04183641063694e-58 1.08924562399701e-56

AC092162.3 5.86460503766478 1.88239322916667 -1.63946591422897 5.61171630981424e-42 4.42911140836145e-41

TNFAIP8 0.781149623352166 2.70980729166667 1.79451944039868 3.08537450163456e-68 2.69354642525327e-66

LUZP2 63.6926482580038 24.3214969270833 -1.38889483283451 1.18283122294874e-43 1.0306816584223e-42

RPS2P7 1.65032575329567 7.34180510416667 2.15338399489334 1.56991959105948e-35 8.57094973451293e-35

C14orf132 140.911271421846 67.74362671875 -1.05662988666742 1.07064386573116e-47 1.23656041511e-46

KLHL4 6.36211247645951 24.7123440625 1.95765407965141 1.14975141125772e-44 1.07705931951321e-43

FAM155A 21.8833126647834 6.41001572916667 -1.77143134568106 2.31830364060401e-66 1.58488441900851e-64

ATP2B3 10.242765960452 2.60271317708333 -1.97651711542659 7.49901833711546e-32 3.41273240280622e-31

FZD7 17.1272694915254 42.3918288541667 1.30749103936326 2.09296712427723e-30 8.85522723001936e-30

SHC1 19.898197740113 49.5559720833333 1.31642116379209 2.64466535801255e-56 6.04146834548444e-55

TNFRSF12A 11.4356164312618 122.969238489583 3.42669142094777 3.4974199214146e-86 3.25172617193522e-82

AC011450.1 0.685843549905838 1.67111776041667 1.28486197990273 5.38202128129134e-36 3.02352524850793e-35

RRM2 3.6000370527307 17.0987597395833 2.24780802244096 8.9892683052849e-41 6.60171580319008e-40

CDCP1 1.38250965160075 5.43198869791667 1.97419092557487 6.35621521069191e-52 1.01020360549415e-50

SUB1P3 0.776125894538606 2.81469890625 1.85861800819581 7.62915012052258e-28 2.85613139704283e-27

CLIC3 0.885905979284369 1.77849182291667 1.00542884166169 1.06326359992665e-24 3.45895497561862e-24

ASB5 0.307484698681733 1.84290703125 2.58339676874762 2.7919869985789e-40 1.97703725204016e-39

LCTL 0.614972834274953 3.56363838541667 2.53475636152302 2.78122761678137e-69 2.88921382871785e-67

CARD8-AS1 2.84941963276836 6.91215692708333 1.27846786909063 2.04593959606126e-64 1.06566517615572e-62

ELAVL2 16.8585186440678 6.20075901041667 -1.44296104639355 7.35550946729479e-44 6.49765788809248e-43

LINC01831 0.433439406779661 2.31318822916667 2.4159804367462 3.2509106665868e-18 7.96032181263913e-18

IGHV3-7 0.775829096045198 7.14583484375 3.20329178277035 1.65838691762995e-15 3.60506251266412e-15

APOBEC3G 1.95193140301318 8.15442875 2.06268146101431 1.83656705392859e-70 2.38817932641973e-68

AC110491.1 3.43440320150659 1.11364489583333 -1.62477014402395 1.07592193723639e-31 4.85483339551339e-31

AC023421.1 6.67321614877589 1.77278645833333 -1.91236346789196 2.19584617703197e-30 9.27783677843887e-30

YWHAEP1 0.495023258003766 1.8293165625 1.88573653950234 1.07977522369352e-24 3.5108271174298e-24

LINC02440 12.7592684086629 4.01786239583333 -1.66704554971915 8.70536453191834e-48 1.01489814088415e-46

CHEK2 2.37532848399247 5.99872401041667 1.33652862002623 6.90469224106014e-57 1.65029244501945e-55

AL161787.1 9.30991812617702 22.7481205729167 1.28890697068643 6.7728910690374e-19 1.70676120651512e-18

SQOR 3.80784990583804 13.2701558854167 1.80113680012873 2.10112189532832e-75 6.30167123284357e-73

PTGR1 12.1428827683616 24.6322777083333 1.02043907400693 1.83664516470095e-44 1.69743949538029e-43

GINS1 6.99845654425612 18.7164221354167 1.4191959861654 3.3150091552089e-34 1.69534090322083e-33

AC079780.1 0.740408239171375 2.3562896875 1.67012406366994 1.09526420299503e-18 2.73155014145555e-18

KIF23 1.74801464218456 7.45166895833333 2.09184631415493 6.61054523545575e-45 6.27810227248437e-44

RPS13P2 6.26224905838041 16.38944078125 1.38801383682691 5.43689319737493e-17 1.25823396987881e-16

RIMS1 6.4751709039548 2.13828682291667 -1.59846288690359 5.67274457723069e-32 2.59814496092622e-31

MMP2 31.3017491054614 90.5341752604167 1.53221921468865 9.45575801310662e-41 6.93061963948434e-40

IGFLR1 0.978592043314501 2.61001411458333 1.4152781509637 9.00093300877546e-60 2.85618343512252e-58

PGK1P2 0.674492467043314 2.11717520833333 1.65026642965839 1.30274872134313e-28 5.06155714027904e-28

FAM86B3P 0.777822787193974 1.7107159375 1.13708681591058 9.12750853610145e-37 5.3592388683643e-36

MAL2 10.0181740112994 2.81777953125 -1.8299889340997 9.71460156415541e-16 2.12846725681006e-15

AC008875.2 0.995803672316384 3.00782859375 1.59478911403188 2.07938372069769e-45 2.04042956656325e-44

VAV3 0.953030979284369 9.27869848958333 3.28332743832821 8.19479638900496e-66 5.2365030533865e-64

AC007877.1 0.327266666666667 2.26494963541667 2.79094039902487 1.23674030312327e-53 2.23920400795209e-52

PLCG1-AS1 0.965411346516008 2.00371927083333 1.05346470724214 1.22989257630826e-26 4.35782249551298e-26

FTH1P7 8.38715047080979 25.0398721875 1.5779745539807 1.16266483820394e-44 1.08860788853989e-43

AL136964.1 2.61719430320151 0.7480096875 -1.80689217578703 1.35512044483836e-57 3.46608867562164e-56

H3C2 0.335517467043314 3.2014946875 3.25428583577022 1.64232874157187e-37 1.00358537461482e-36

FRMPD4 4.04564260828625 1.13271489583333 -1.83658409724342 1.27867353781679e-19 3.32777248925167e-19

S100A12 1.01660047080979 3.34656052083333 1.71892630089757 2.60722694026509e-16 5.86941706467668e-16

SFRP4 6.59151026365348 43.5364727604167 2.72354356574975 5.58926615299677e-62 2.24961913668777e-60

LCA5L 0.794425941619586 1.79175984375 1.17339264125818 9.66853293631219e-28 3.60365544098467e-27

SMOC1 670.413160828625 217.964505260417 -1.62095725434388 1.6391795788179e-56 3.8148365792389e-55

AL391845.2 3.40476026365348 1.18581505208333 -1.52167420388182 8.85646403763756e-46 8.94060525406463e-45

HLA-DQA2 9.39142241054614 37.5132605208333 1.99798507449818 1.23201016676875e-28 4.79272574290061e-28

AC005740.1 2.86540555555556 1.15421302083333 -1.31182983401615 2.08815415257249e-10 3.6503926358076e-10

CDKN3 4.36435433145009 17.272045 1.98459876675083 2.10505970558907e-44 1.93876103147245e-43

VAMP8 46.9682202448211 135.72478796875 1.53092739801274 6.21641216927788e-53 1.07629594308866e-51

OCIAD2 6.85404387947269 64.3225385416667 3.23029701167335 3.86969812214088e-55 7.90736665727579e-54

PGAP4 27.9566391713748 12.7383661458333 -1.1340106906099 2.346327846561e-44 2.15456623737293e-43

SUMO1P3 2.23848667608286 5.22278348958333 1.22229516698731 6.65481657635163e-30 2.75481554401733e-29

NIPSNAP2 60.2612596045198 173.446179427083 1.52518532797712 1.16353676768493e-27 4.32200683082327e-27

FRY 21.5101041902072 9.77353572916667 -1.13806203368081 6.56560812885373e-63 2.86590336047031e-61

RNU6-1189P 1.24965513182674 2.5406471875 1.02366603802721 0.00121812920347785 0.00152224429661659

RPS7P11 3.73344402071563 12.4258494791667 1.73476547884028 2.56983020777312e-29 1.03031463375466e-28

FOXJ1 11.3414191148776 60.5585281770833 2.41672897097615 4.19834064730869e-42 3.3476905804762e-41

GDNF-AS1 2.74752622410546 0.836013541666667 -1.71653503497921 2.31737460323018e-56 5.33972499963633e-55

MOXD1 3.17954307909605 48.30977984375 3.92542391725747 1.24612247573284e-46 1.33246966280921e-45

WDR76 6.78769237288136 15.0147220833333 1.14538468477418 8.38345900401595e-33 4.00129415245576e-32

KCNB1 19.2355882297552 7.09321651041667 -1.43926605872737 5.40768347491398e-62 2.18125540598754e-60

PDE6G 0.93238352165725 2.38369666666667 1.35420524718863 1.25410675814645e-48 1.54437848792935e-47

CD72 1.27721643126177 4.22975984375 1.72757273442358 4.24276883784891e-51 6.35732974044143e-50

AL158166.2 0.569467231638418 1.862243125 1.7093567024484 3.18957043231696e-52 5.18898181880436e-51

AC025458.1 1.36291313559322 6.93226817708333 2.34663385089455 4.78420688591643e-34 2.42867395696467e-33

ATCAY 201.943244397363 76.7179519791667 -1.39631377182629 1.31454239045959e-44 1.22710420434719e-43

ZNF888 1.34143587570621 3.14422572916667 1.22892670328957 7.04800473798814e-64 3.45798543807096e-62

AC107464.2 3.09937669491525 6.86849927083333 1.14801680338812 1.44016831520923e-26 5.09317798047083e-26

AC004522.5 1.30494369114878 3.31239697916667 1.34388802988211 2.50773554757062e-23 7.71531146708731e-23

TMEM37 3.83905484934087 11.3610405729167 1.56527190174867 1.93104770597346e-52 3.18897265475812e-51

SEMA3A 1.69301440677966 6.43580427083333 1.92652620074331 3.52516505125879e-21 9.85869215343017e-21

LAP3 74.7324717043314 158.165591666667 1.08162863708347 8.98384349639076e-65 4.97186219688649e-63

F3 57.9201586158192 220.440660052083 1.92825289366766 1.04912016765994e-52 1.78158808380243e-51

LRRC61 3.02104369114878 9.51810078125 1.6556266815183 6.50365652890595e-23 1.96867154737109e-22

UBE2C 16.8404133709981 80.4113466666667 2.25547153950041 1.24695733651533e-40 9.05395223447972e-40

ATP8A2 9.11727005649717 2.70240265625 -1.75435925800067 1.15818680843045e-34 6.04448041054286e-34

LRR1 4.63609482109228 10.9325450520833 1.23764731475104 1.20878103604905e-59 3.79043564339496e-58

FPR3 4.94618677024482 15.7363654166667 1.66971374148619 2.68304868643052e-33 1.31017043918528e-32

PIF1 2.25569698681733 4.55360161458333 1.01343479844785 8.22155261965659e-19 2.06371181104906e-18

CHRNA1 0.466751741996234 8.027775 4.10427286990043 5.50934843062645e-43 4.63767922442276e-42

DOK6 12.2660018361582 3.60433859375 -1.76685862177939 1.42561078020278e-61 5.44337422132868e-60

B9D2 7.03857561205273 14.169835625 1.00946761551111 9.18724152812524e-50 1.22463624527232e-48

VSTM2A-OT1 2.00379877589454 0.778776614583333 -1.36345617048004 7.70523286381301e-49 9.57106246510373e-48

ADH5P4 0.389655320150659 1.8204525 2.22402667848281 1.59253471169698e-36 9.21667692623882e-36

TMEM238 0.806252683615819 2.17304739583333 1.43041567858861 1.28716141020638e-40 9.34009821721021e-40

SLC39A8 7.98426699623352 17.99566625 1.17241764512478 4.12248936977753e-39 2.73289446812881e-38

NRG3 18.3508017890772 8.38925135416667 -1.12922912183609 5.10899502402306e-45 4.88189940759038e-44

PLAC8 0.87521516007533 3.67211114583333 2.06890009310601 3.26886698458678e-54 6.18987592447975e-53

SLC7A14 17.7760917137476 6.31936494791667 -1.49208667526756 1.71788659528794e-44 1.59242777863306e-43

AC008525.1 1.26227424670433 2.54387552083333 1.01100268710326 4.15621260091792e-14 8.54825498441198e-14

LBX2-AS1 1.39915047080979 3.90855598958333 1.48208457971752 1.29250815397726e-58 3.67495246532219e-57

IGHV3-21 0.55776581920904 7.40101166666667 3.7299910590656 3.65797781246278e-19 9.31654532567393e-19

HLA-DQA1 8.93777241054614 40.8211596354167 2.19132995275953 1.33254688191435e-55 2.82861977958874e-54

OTOS 1.55768102636535 21.9151972395833 3.81445992202131 1.24848848927388e-52 2.10668270944172e-51

NKX3-2 0.391713370998117 2.20607432291667 2.49361111619284 8.04739313882185e-41 5.92638714520366e-40

DYNC1I2P1 1.52940155367232 3.58095713541667 1.2273780047056 1.90002260634064e-35 1.0321655840909e-34

COL3A1 27.5791400188324 198.11442515625 2.84468455380954 1.57900013706412e-46 1.67397420460133e-45

HSPD1P1 0.522443926553672 2.00512046875 1.94034080977492 3.03023816985282e-31 1.33682749154005e-30

CTSC 3.70872881355932 12.1414743229167 1.71094693143246 5.90524001583922e-55 1.18073051714549e-53

ROPN1L 0.59786577212806 4.12354395833333 2.78599126330956 8.38746682622834e-27 2.99208740804597e-26

LILRB3 1.07156713747646 4.64129604166667 2.11480547888861 1.61790180353049e-58 4.56523278249613e-57

DOC2A 9.22990263653484 3.93160807291667 -1.23119591734098 5.14430173503864e-10 8.84578238977654e-10

HSPE1P3 1.26094731638418 3.23161786458333 1.35774861129151 2.76163464661193e-31 1.21977663310567e-30

ACTG1P14 1.0037656779661 2.35116619791667 1.22795400142487 6.29287027105453e-47 6.90359425901233e-46

DTHD1 0.712151459510358 1.86697630208333 1.39044760568149 0.0091371287882997 0.010798599421232

CD74 425.083889218456 1460.3791615625 1.78052350129585 2.01530184529771e-59 6.14336685464113e-58

TRAM2 7.53529736346516 15.14347578125 1.00696002477011 1.39364943291116e-32 6.58407296874569e-32

CENPA 1.83266054613936 10.8104484895833 2.56041488384818 3.73740712925522e-49 4.75356262438446e-48

AC104985.2 1.55934708097928 0.736772447916667 -1.08165106319947 3.09958196849739e-34 1.58910192181442e-33

AL390755.1 4.23452419962335 31.639343125 2.90144786843205 2.26748151340809e-83 5.27047734272793e-80

TK1 8.97264896421846 33.5457005208333 1.90252199617752 7.09688395255564e-47 7.72637922118104e-46

EPHA10 4.71187321092279 1.66929489583333 -1.49706187687938 3.22091227620062e-44 2.91733384198493e-43

LINC01871 0.761217702448211 2.28635473958333 1.58666824478886 1.14213509481374e-38 7.40000072754756e-38

TRIM21 15.8619663841808 35.8653015104167 1.17701712832101 3.89385585505293e-55 7.94799666572e-54

PPM1M 12.9303515536723 29.4665053125 1.18831446889587 2.50551975466242e-68 2.19764810556357e-66

AC060766.1 1.32198441619586 3.18796442708333 1.26993036091666 3.40350534332564e-30 1.42704956102533e-29

TEF 72.979763512241 30.918860625 -1.23900931970847 6.49659597919041e-69 6.3581159070024e-67

FLNC 10.1887830508475 63.4578106770833 2.63881600231868 1.12470041171994e-61 4.36613865468315e-60

PDLIM4 4.01152024482109 49.7591983333333 3.6327422593101 4.04703211650929e-59 1.19262380675896e-57

GABRA3 38.3887220338983 12.4319230729167 -1.62663305177674 6.82342718610797e-51 1.00381035226011e-49

GABRA5 15.9376834745763 6.10831447916667 -1.3835957058043 6.20261740404486e-16 1.37176106836601e-15

LILRA4 3.00828451035782 6.2486728125 1.0546087837119 0.000141100567929086 0.000186386663397127

FPGT 5.69428460451977 13.1635461458333 1.208961684086 1.06561098708358e-69 1.20823392102556e-67

PRDX1P1 0.431760357815443 2.15422864583333 2.31886868983504 6.1681196508027e-36 3.44847218600951e-35

CCDC92B 7.27338917137476 2.93440197916667 -1.3095612543855 4.83444953055833e-47 5.34780422490971e-46

PARP4P3 3.77488121468927 0.800407447916667 -2.23762475051565 7.95285872443083e-20 2.08580265135108e-19

PHTF1 5.24996421845574 10.8426215625 1.04633412300347 1.21682714791721e-60 4.21357557086043e-59

PTPN7 0.926564265536723 3.90334760416667 2.07474899591229 3.42514882905998e-65 2.03484480755177e-63

SLC2A9 0.986372645951036 2.24866296875 1.18886274848724 8.17571681498512e-53 1.40246728943403e-51

FREM2 2.30254623352166 7.72907052083333 1.74706480467784 1.82358079650099e-28 7.03370356999293e-28

CRH 2.90125494350282 1.04268864583333 -1.47636865253331 1.65954446142181e-13 3.3271406210392e-13

AC117382.1 0.472837429378531 1.8194103125 1.9440547879745 4.8370088677766e-40 3.37881968055244e-39

GJD2 3.75465715630885 0.808817291666667 -2.21479543299228 1.31388263041245e-33 6.5220628704003e-33

STK40 24.0052731167608 53.2850138541667 1.15037848904916 1.3684890748955e-66 9.63903573775826e-65

FSTL1 21.0748372881356 95.4168994270833 2.17872331405889 1.84532950517917e-76 7.30083024442695e-74

MIR210 0.664778154425612 1.97175723958333 1.56853706079586 3.41887134867825e-10 5.92266747984647e-10

SOCS1 1.4720290960452 7.30998145833333 2.31206155889973 4.46182052810952e-63 1.98486968230135e-61

CDH4 12.8355628531073 25.851968125 1.01012755689801 7.63960041818742e-14 1.55407909174264e-13

RPL19P5 0.559847740112994 1.85993234375 1.73214372255472 1.91857402845404e-24 6.1722982801216e-24

RPL37AP8 0.896758757062147 2.49648364583333 1.477105622152 1.85538057657095e-14 3.87344805448937e-14

KDELR3 2.60581713747646 13.2859291666667 2.35009137605553 3.93304207236608e-54 7.3724715056096e-53

KLRC4 4.07479435028249 1.57988875 -1.36690427752754 4.83677743978024e-42 3.84030215596557e-41

AC109439.1 6.58710164783427 1.20566354166667 -2.44981645511246 3.13762359810372e-59 9.36502581167555e-58

KIF21B 55.2581739642185 21.62222921875 -1.35367261792043 2.47766269107694e-58 6.8255018874927e-57

CTNNA3 8.33848483992467 4.09011609375 -1.02764346670035 1.55344196160206e-36 9.00163704455915e-36

STXBP1 93.6382357344633 39.9718883333333 -1.22811202296893 1.91063427303569e-38 1.22258239184785e-37

HAVCR2 24.6081866760829 54.9016422395833 1.15771095169575 6.381542156955e-40 4.42944294171625e-39

BX537318.1 1.46501520715631 3.448276875 1.23495997824507 1.9994716625486e-50 2.80393480883041e-49

SCGB1D2 1.67828512241055 7.06061265625 2.07280553816959 1.1297584426393e-24 3.66949488923629e-24

CPM 11.4242646892655 32.61900046875 1.51361126405976 1.17532699778593e-34 6.13049243305172e-34

NOX4 0.554212758945386 3.24487182291667 2.54964966115691 5.1844183962493e-60 1.68538916220727e-58

MIR4269 1.69720936911488 0.5637375 -1.59006910355876 6.68010770615073e-15 1.42026758284785e-14

IGLV8-61 0.229448163841808 3.94796635416667 4.10486952799104 1.34786536001647e-16 3.07264390946503e-16

DUSP4 5.42220583804143 11.7658605729167 1.11765505834131 1.90054035909752e-20 5.14868123214137e-20

SEMA3D 8.21931257062147 3.53838598958333 -1.21592630295673 7.14463573710121e-35 3.77427561168741e-34

OSCAR 4.92325400188324 12.1472327604167 1.30294361493105 1.60623864301717e-46 1.70091159264831e-45

INSM2 3.34952033898305 0.738020833333333 -2.18222106494251 1.5733317099064e-42 1.287680596202e-41

AC012213.4 10.5426751412429 2.68048838541667 -1.97567319971696 2.30746955474846e-54 4.40075860210745e-53

FCGBP 19.320288559322 102.813496145833 2.4118411100845 2.73069014185322e-55 5.65447474251232e-54

GIMAP2 8.60333672316384 22.03050484375 1.35653434658706 3.09654601814445e-59 9.28714083990259e-58

Z96811.1 0.289259322033898 2.64742020833333 3.19415184360906 2.24613769745087e-45 2.19594797497891e-44

CACYBPP2 0.950980414312618 3.66568359375 1.94659473011169 2.64764022619684e-28 1.01490512454285e-27

SLITRK4 3.86429868173258 1.7362259375 -1.15425190823472 1.19070491925857e-18 2.96480422785392e-18

H2BC8 1.41579552730697 3.35977057291667 1.24674979684749 1.03088929396558e-16 2.36164086776487e-16

FLNA 95.4985747645951 250.891279114583 1.39351121666863 5.31974164578518e-52 8.49833298138964e-51

ATP5MC2P4 0.373002401129943 2.26592114583333 2.60284083348406 3.14658393055435e-26 1.09790281158066e-25

AC026403.1 19.2298343220339 52.5802651041667 1.45117508294845 3.00372119557743e-22 8.80703179308772e-22

NMNAT3 1.01196873822976 2.67895130208333 1.40450363320076 5.59051713012581e-36 3.13781062585842e-35

SCD 1131.5328452919 487.790096770833 -1.21394608907844 5.03126492717815e-51 7.47852688416289e-50

GABRD 22.4499037193974 7.3022996875 -1.62028647418636 4.94171523536506e-40 3.45066446870497e-39

SNORC 29.5921921374765 14.3434891666667 -1.04482055913914 5.22634093632307e-13 1.02471330357368e-12

MMP1 0.271487947269303 1.86813458333333 2.7826383368018 1.08977069084497e-19 2.84450954467465e-19

MCUB 3.72136737288136 31.4805730208333 3.08055707627881 3.68913356088424e-87 6.85994385646424e-83

MAP3K8 4.67172490583804 9.71406578125 1.0561199322361 3.3721468440012e-27 1.22782593624833e-26

RPS10P3 1.5641356873823 11.0624263020833 2.82223026828835 1.71028770112045e-28 6.60631487377124e-28

VENTX 1.23256906779661 3.22183677083333 1.38621491286891 1.75828258358742e-27 6.48072639084402e-27

AC091042.1 0.759763229755179 1.66343838541667 1.13054663234615 2.24025665896464e-17 5.27511366005413e-17

KCNMB1 3.22771261770245 6.55621520833333 1.02235107912208 5.58911248607664e-26 1.92819619621915e-25

H4C5 1.16372104519774 2.53943317708333 1.12576123900802 1.35779762450605e-17 3.22331760853951e-17

CENPH 8.89157645951036 21.0788720833333 1.24528653745312 1.2037662565978e-35 6.61857881177886e-35

H2AC11 1.12389110169492 3.10863098958333 1.46777711771715 4.73288849252493e-39 3.12750751664894e-38

SMIM3 17.1924298493409 85.7356822395833 2.3181223040426 2.44166507672507e-72 4.7792381159687e-70

FHOD1 7.05756633709981 14.11884859375 1.00037975081784 3.37770864077224e-53 5.96471910495345e-52

EGFR 112.002623399247 473.2296521875 2.07900795018259 0.000192104568030928 0.000251970405765332

CPQ 12.7036307438795 38.7852540625 1.61026736861681 4.53450457624096e-58 1.21848428605781e-56

RPL37P2 2.30722942561205 12.3532328125 2.42065526662253 1.48984448654e-21 4.2334441056252e-21

RPL39P15 1.67933017890772 6.09296911458333 1.8592595158962 3.96835500573047e-25 1.31255000589751e-24

CD44 54.3117882297552 188.966697135417 1.79879473078826 2.36709005145672e-57 5.89237476664494e-56

MX2 1.41378870056497 4.42450645833333 1.64595001886814 5.06169508885282e-48 5.99122980122331e-47

AC079848.2 1.05698436911488 2.367664375 1.16351054618261 1.46357592416275e-24 4.73143155594686e-24

MAP3K7CL 1.81374529190207 11.6330955208333 2.68119126887733 7.5659514088586e-74 1.85116929536481e-71

RSPH1 7.54495353107345 18.2695278125 1.27585542666379 0.000133078685629019 0.000175990196946989

RGS7BP 13.2521296610169 4.63858963541667 -1.51446609889224 1.77874825021218e-32 8.35881316974867e-32

CENPM 5.80707716572505 16.7263865625 1.52624170037243 6.47160365344699e-31 2.80904458300296e-30

UBBP4 3.64869439736347 7.37553015625 1.01536643403015 3.47010397919882e-37 2.08823894800007e-36

CITED1 0.540981403013183 1.55026958333333 1.51886820865647 4.75600607830254e-19 1.20619112146803e-18

HSPA7 1.84289289077213 12.7883822916667 2.794789648471 2.68328931469718e-65 1.61474967012279e-63

ADGRE1 0.704177589453861 3.3156646875 2.23528689567125 1.05891737474732e-46 1.14016031172127e-45

SELL 153.219432862524 45.723164375 -1.74460212918016 7.92752257908692e-28 2.96544522949349e-27

AC131097.3 0.371400753295669 3.59421453125 3.2746278763651 3.33212116688448e-64 1.67461602968154e-62

SH3D21 2.05919039548023 4.14390328125 1.0089131012607 1.23744305045576e-24 4.01294969013339e-24

CD33 2.39593808851224 5.08777348958333 1.08644381339256 1.33609813562857e-35 7.31803971487872e-35

PARP12 6.62662641242938 19.5429241145833 1.56029985465802 1.30618647199962e-65 8.06928154379832e-64

UBE2QL1 21.9878938323917 9.88002520833333 -1.15412279063038 9.71522951295352e-33 4.6238723520187e-32

AVIL 4.55452085687382 18.4433163020833 2.01772689760281 6.1407312089036e-19 1.55103092678026e-18

LHX5-AS1 17.6704224576271 3.6495334375 -2.27555258736671 1.9151853043562e-41 1.46374314568449e-40

NHP2P1 0.712685969868173 3.69526036458333 2.37433759042555 3.78003534134047e-25 1.25137541698818e-24

CDCA4 5.47847292843691 13.3245710416667 1.28224337201875 3.78586975857383e-43 3.21600037280404e-42

TWSG1 26.1031034369115 61.015529375 1.22495513595935 7.53075677952701e-60 2.41855651667193e-58

AEBP1 56.1968558380414 332.159432916667 2.56331456538401 5.65972568872965e-60 1.83349475926704e-58

HTR5A-AS1 1.61835141242938 0.468166302083333 -1.78943191155016 4.42451693759501e-11 7.95377923961516e-11

AC009961.3 1.06024962335217 2.40362796875 1.1808096439376 4.04514290545777e-49 5.13092989952164e-48

COTL1 68.4808862052731 137.535784635417 1.00603375713967 2.07086259920849e-58 5.76462425632962e-57

IGFBP7-AS1 1.41818112052731 6.39786776041667 2.17354937761068 6.97383919981916e-24 2.19273824688261e-23

PDE6B 4.985575 10.29996484375 1.04680760385606 3.55603363826138e-36 2.01660401047485e-35

RIPK3 1.04282518832392 2.32470890625 1.15655274200796 2.76882029683768e-43 2.36827108646259e-42

DENND2D 1.15137923728814 4.32040494791667 1.90780343969756 7.63882834641633e-65 4.26558597902738e-63

RPL37P23 1.4611722693032 4.79137026041667 1.71331202518579 7.35704111702442e-25 2.40386890829501e-24

LINC00836 6.8275709039548 2.69127171875 -1.34308433428954 4.02219187913976e-40 2.82342989779554e-39

FAM180B 1.23152236346516 3.30628083333333 1.42476644514128 1.47661080765995e-12 2.83512322747589e-12

H2AJ 7.99598917137476 17.1804297395833 1.10341770010366 1.65809039005309e-55 3.49175433782981e-54

E2F8 0.630439924670433 2.75863567708333 2.12952413163541 7.25321083162401e-44 6.4164346058063e-43

CR769775.2 1.76542156308851 0.83083453125 -1.08737964019352 1.28805776583043e-28 5.00552437943926e-28

ADA2 9.23874124293785 22.72921421875 1.29877960270831 2.14777732489443e-46 2.25511463587881e-45

AC125616.1 1.74913804143126 0.397006510416667 -2.13940957970485 1.75789222184144e-40 1.26257264832528e-39

ORC6 3.57986167608286 8.39470859375 1.22957640202187 2.99462760141698e-27 1.09122281497842e-26

S100A2 3.00457787193974 8.56660692708333 1.51156157914193 2.5095830068837e-58 6.90320946937905e-57

IGHA1 5.77089896421846 56.1884468229167 3.28340554354774 4.57841376236162e-25 1.51190914422153e-24

PCDHGA3 7.09534882297552 2.38700697916667 -1.57167082749587 8.55756947184761e-15 1.8125982951248e-14

RAD54L 1.40253549905838 4.323739375 1.62424227655829 1.25290328697886e-41 9.69123819524622e-41

WNT16 0.291144397363465 2.92040651041667 3.32636243955523 5.31937430511578e-22 1.54167339781216e-21

ARPP21-AS1 2.09812881355932 0.711847604166667 -1.55946293405153 1.32436215546703e-49 1.73916061305858e-48

C1orf54 20.8030744350283 51.4451578125 1.30623853584344 3.72114452046521e-61 1.35145863980568e-59

AHR 3.8367777306968 13.38721109375 1.80288834549248 4.79000442246657e-45 4.5841550301475e-44

AC007389.3 0.65072829566855 1.46515708333333 1.17092815503114 5.83104292087493e-33 2.79959316069376e-32

PPBP 0.842864124293785 1.93310020833333 1.19754444382807 3.57011899681079e-12 6.73221404986276e-12

SLIT1 124.262415630885 49.1518584895833 -1.33807213259635 8.05787246881933e-46 8.16990940881654e-45

GZMB 0.688154896421846 2.03574234375 1.56474973442715 4.3188745248497e-28 1.63696436586996e-27

PEBP1P2 1.16959769303202 4.349486875 1.8948328401799 1.35196983058193e-25 4.58924406711774e-25

APLNR 111.207745103578 259.571628333333 1.22287543409804 3.33026596794308e-19 8.49702190915225e-19

AC099681.1 0.323408615819209 2.13397848958333 2.72211561514281 5.36681349445661e-05 7.24839460556513e-05

IGFBP3 30.6463962335217 205.416492135417 2.74476267516878 1.85235508940104e-49 2.40199043845274e-48

LGALS3 30.7182381355932 243.210380572917 2.98503742958122 2.44457265669459e-61 9.12787721912367e-60

IGFBP5 134.910121327684 534.013108541667 1.98487656922281 3.4876261355591e-54 6.58400081124076e-53

AL139100.1 2.57662876647834 8.4400821875 1.71177235494735 1.08309174837056e-24 3.52037948976587e-24

IL13RA2 3.10906718455744 45.8695376041667 3.88298266649362 2.76926181848213e-53 4.9229850396439e-52

AC093849.2 0.197672928436911 2.364341875 3.58025144815371 4.79467787637312e-38 3.00495568288366e-37

PWWP3B 3.16118761770245 1.43330994791667 -1.14111604154485 4.35742556687524e-22 1.2684146589863e-21

RPL14P3 0.628626883239171 1.81000963541667 1.52572150193465 5.14128813745604e-21 1.42689929725366e-20

MX1 11.31925913371 43.5296579166667 1.94321914834758 6.2724540989162e-41 4.65613908061264e-40

GNG5P2 0.402919632768362 3.78699739583333 3.23249041968782 1.1874390426084e-46 1.27118186512972e-45

HLA-C 464.931949246704 1174.783418125 1.33730333385723 2.07585393980074e-48 2.51797155972569e-47

PTMAP9 2.52655936911488 8.94325322916667 1.82362564587689 6.51699731336061e-46 6.66209813314681e-45

SBNO2 12.6946177966102 26.3877769791667 1.05565285721569 7.01606176339291e-41 5.19361737620586e-40

TNFSF8 1.93281247645951 4.30129744791667 1.15407022900332 1.94967328829657e-30 8.26588572637363e-30

PLIN2 9.64044929378531 37.1987027604167 1.94808002071496 8.19623592783956e-62 3.2290043872495e-60

MEGF11 33.5622018361582 14.8995510416667 -1.17156850680552 1.39372344366799e-38 8.99246614677525e-38

HPCAL4 81.2290101224105 31.5311316145833 -1.36521811588605 5.52516032253478e-30 2.29331152226639e-29

RPRM 81.4791307438795 31.49372015625 -1.37136640498036 2.42085138613279e-45 2.36055225616881e-44

ST14 4.51822142184557 14.69480953125 1.70147978053297 1.92091553977867e-43 1.65674510492506e-42

MIR22HG 7.46080197740113 16.27147890625 1.12494276049813 2.51162487895507e-46 2.62971084595549e-45

GNG11 18.4678573917137 37.6276309895833 1.02677596406105 1.29487101216787e-32 6.12674973823449e-32

CRYGS 4.77557434086629 11.7079274479167 1.29373955439182 4.0855385130904e-20 1.08591464623951e-19

LAMA2 4.19573220338983 12.7797490625 1.6068650038622 5.0471556602501e-48 5.97782544600959e-47

FAR2P2 0.663119632768362 2.20999270833333 1.73670053544435 7.64954951047837e-20 2.00854795446981e-19

CSMD1 6.50918338041431 2.71304260416667 -1.26256485611694 2.65251535145664e-36 1.5116004584841e-35

HSPB1 178.242585028249 626.768319791667 1.81409019900931 7.85623163632659e-77 3.39736342505798e-74

IMPA2 2.73941916195857 6.17877989583333 1.1734519503073 8.66060124456309e-50 1.15692442631215e-48

UBE2S 16.4141101224105 53.4987971875 1.7045699182953 1.02979639809107e-50 1.4913601263632e-49

RHOH 0.532931779661017 1.52851135416667 1.5201044977432 4.11695814972447e-49 5.21490713856448e-48

LDHAP7 0.61674147834275 1.54672895833333 1.32648262596319 2.39513157827222e-26 8.39379413832867e-26

PHETA2 3.20121732580038 10.9655382291667 1.77628409885291 1.03812474898404e-72 2.2446429892277e-70

HOXD9 1.60779223163842 10.9203319791667 2.76386382485343 9.83750295215196e-46 9.88801985920355e-45

RUNX1 2.39927024482109 9.24415609375 1.94594595432676 4.19591357942291e-54 7.84152735116136e-53

PON1 2.08954161958569 0.721557395833333 -1.53400043194102 4.49544252516645e-46 4.63374466493737e-45

ARAP3 5.91441012241055 25.7314247395833 2.12122514806804 1.32464667737886e-60 4.56990815693134e-59

AC027559.1 0.688505602636535 1.9211134375 1.48040241018181 1.3056467836954e-16 2.97821156089452e-16

EEF1A1P38 0.791967325800377 2.41724213541667 1.60984917989723 4.18441076549417e-20 1.1115588312052e-19

DISP3 27.7139140301318 12.3372840625 -1.1675856430764 3.08234184566728e-48 3.69305068429015e-47

AL133465.1 17.9254846045198 8.18063109375 -1.13172807273388 3.72948627887237e-24 1.18607486498429e-23

HOXB4 0.239030790960452 7.6603296875 5.00213810730253 1.75710766825244e-52 2.90947614346876e-51

RPL9P32 0.647432015065913 2.19484317708333 1.76131724928396 3.84378636445663e-26 1.33673475681824e-25

ZNF468 3.79678799435028 8.88232677083333 1.22615820364108 3.66413653952497e-51 5.51254646722992e-50

MAPT-AS1 5.44770692090396 2.24671994791667 -1.27782878565181 2.47316653146401e-53 4.42623018792813e-52

IL1R1 3.64429618644068 11.8827076041667 1.70515148477353 2.76303647302399e-22 8.11541039581126e-22

AC005099.1 0.769810875706215 1.59107791666667 1.04742852914859 1.23593111548947e-20 3.37525908246831e-20

NEUROD2 6.00099943502825 2.61656484375 -1.19752878174884 5.84671628119207e-11 1.04528111959203e-10

IGKV1-5 2.08402344632768 17.02832 3.03049269305897 5.70601662865186e-25 1.87494927036192e-24

ARPC3P1 0.560866902071563 2.2093021875 1.97786040926658 1.95070830017491e-36 1.12024153309921e-35

DENND2A 47.7578148305085 103.879334427083 1.12109994316973 8.6511639082172e-39 5.63461971535197e-38

SRRM4 6.39801247645951 3.05765177083333 -1.06519969506586 4.37660057909012e-36 2.46839210701185e-35

CCDC8 2.74204708097928 14.3791340104167 2.39065154392629 6.7812319543228e-48 7.96066970900457e-47

DSCAM 40.0661230225989 18.1632716666667 -1.14135882282827 9.76269610719792e-41 7.14995408087221e-40

AC078819.1 0.987514548022599 3.0404421875 1.62240725048752 3.14497821012227e-15 6.77646231949289e-15

S100A13 5.96881016949153 22.6720772395833 1.92540130184598 9.09821473909507e-52 1.43010399893046e-50

AC080023.2 1.05427132768362 5.14051625 2.28566704585232 6.74615021233849e-30 2.79138102355216e-29

HMGA1P7 7.78094218455744 1.40895854166667 -2.4653156986784 2.34229626576363e-58 6.47176806268569e-57

LOXL4 1.25380960451977 2.90592151041667 1.21267744962279 4.92698170938813e-58 1.32013292343044e-56

AC004852.2 0.80839934086629 2.10882739583333 1.38330096715047 6.04043800737047e-18 1.46176398681746e-17

RPL10AP2 0.907909463276836 3.46015057291667 1.93021447569865 2.47670574626668e-21 6.96737418333267e-21

AC073349.2 8.98468606403013 4.02720802083333 -1.15768810010472 1.93758613036228e-42 1.57609274925393e-41

AC022239.1 2.0383945386064 0.446028125 -2.1922267278382 1.07512032130544e-21 3.07472506531447e-21

FGFRL1 13.7299927024482 53.0869477083333 1.95102633584282 6.90417804653841e-62 2.74323057212354e-60

UPK2 5.18498950094162 2.2518190625 -1.20325015866622 4.69690394661596e-45 4.49968721727583e-44

ANKRD55 2.28064006591337 0.937723333333333 -1.2822045395906 3.94381894795292e-10 6.81428297130501e-10

HAPLN1 23.4402033427495 10.2441890625 -1.19417930105709 6.0320487605523e-28 2.27056572272206e-27

MICA 3.82654679849341 8.41530208333333 1.1369720169132 1.60916819986422e-40 1.16068590676785e-39

HLA-DPA1 54.2106506120527 198.540704583333 1.87278659062191 1.45983263308033e-54 2.83949663306786e-53

Z74021.1 3.75569251412429 14.5929301041667 1.95811873338312 3.04860545116429e-26 1.06437886527225e-25

UBE2V1P2 0.592181214689266 2.050848125 1.79211002653962 1.24409532820747e-28 4.83771489502675e-28

HPSE2 28.560525047081 6.76681348958333 -2.07747397173043 4.02491874097578e-61 1.45609657565067e-59

DUSP26 113.288025612053 42.26734046875 -1.42238013477281 1.6746224775839e-69 1.81044214945771e-67

C7orf31 2.83211473634652 6.98068755208333 1.30148942552252 4.55977604736915e-54 8.4958953507845e-53

ASIC1 124.516592937853 55.9938995833333 -1.15299644470686 1.35364123170283e-46 1.44246181682029e-45

TEAD3 3.49635437853107 13.9691500520833 1.99832091915513 2.10656836765126e-66 1.45080143690649e-64

SMIM18 22.8689161487759 8.67669630208333 -1.39817025358586 9.02239025813863e-46 9.09329793225408e-45

AC106865.1 0.583974858757062 2.63326411458333 2.17287406521015 7.20731000384462e-42 5.64240792705153e-41

DLX2 1.23879166666667 2.99971125 1.27589005147091 3.21584150501828e-12 6.07956209697183e-12

ERI1 7.00691534839925 15.3039091666667 1.12704884395718 6.56560812885373e-63 2.86590336047031e-61

SHANK2 8.16222481167608 2.22738645833333 -1.87361055538155 3.05656120820841e-69 3.14015224677544e-67

GABRA4 4.30920084745763 1.37339859375 -1.64966995103606 1.30267878369127e-14 2.73462542139751e-14

PDLIM3 5.88603540489642 18.4500447916667 1.64825619343506 3.31216987448082e-55 6.82057572712855e-54

TIMM8BP2 1.00640927495292 3.23733520833333 1.68558963334907 1.88983215707514e-36 1.08729668814394e-35

MYZAP 0.473063983050847 2.48653890625 2.39403177492742 3.67514403909028e-54 6.91000497882542e-53

GPR26 2.67643766478343 0.461703958333333 -2.53527404591508 4.8968818018149e-17 1.13594706967001e-16

NEGR1 14.1110722222222 5.24239119791667 -1.42853069486527 1.44542003112166e-46 1.53674016459161e-45

CORO6 9.72956487758945 3.71379442708333 -1.38948132799352 3.7886272865265e-27 1.37596727330001e-26

RNU4ATAC 3.68296887947269 11.2240734895833 1.60765524829434 8.11611695502698e-37 4.77743573215343e-36

AL031666.1 0.537448775894539 1.69233104166667 1.65481264225372 1.49171653495141e-54 2.89249876728599e-53

ABCG4 2.54727848399247 1.24415875 -1.03378611273856 8.58405107990371e-07 1.26884534609568e-06

ADAMTSL4 1.30659030131827 4.61528651041667 1.8206133751869 1.23389278686693e-57 3.16909342151803e-56

AP000547.1 1.82373596986817 0.3151975 -2.53256888226316 6.43078849459245e-18 1.55380083234078e-17

CCN1 42.741688606403 111.01519859375 1.37704139187099 1.35597413755224e-22 4.05114702567222e-22

TMEM179 35.8649970809793 13.1776230208333 -1.44448635281704 1.70067557144203e-36 9.82724122155517e-36

CYP1B1 5.0876563559322 13.6926593229167 1.42832953448496 7.87241027526158e-05 0.000105443685852113

LCP2 7.36176657250471 16.0563265104167 1.12501794929818 1.65245218731175e-40 1.19052105474862e-39

MLKL 1.75779595103578 3.5572915625 1.01701161645401 3.16193229245771e-46 3.2828660512703e-45

NID2 3.37102777777778 8.27911307291667 1.29628770717868 2.60782357865066e-27 9.5326281590346e-27

LILRA5 0.350440018832392 2.07667140625 2.56703351579576 7.20068900775635e-36 4.00888658979729e-35

PAX1 2.41946167608286 1.07586395833333 -1.16919042489429 2.00457966968997e-27 7.3637216432013e-27

ADGRE2 0.88162038606403 3.4337328125 1.96154829450286 2.94352129834812e-54 5.57948812872409e-53

ECHDC2 2.70157693032015 10.1490889583333 1.90947655850415 8.0682382922743e-50 1.08012160579439e-48

TFF3 0.578727589453861 2.26188302083333 1.96656799063233 1.21772963991591e-20 3.32701772762803e-20

AC009102.2 13.2138663841808 5.44500921875 -1.27904626504261 3.79205121695854e-46 3.92175708450189e-45

CUX2 13.1963199623352 2.61775640625 -2.33373290397915 1.00475807788506e-67 8.15872334422388e-66

REPS2 20.6590965160075 6.85789963541667 -1.59093846635719 4.17479985236621e-64 2.07567923141042e-62

PRR11 5.48333163841808 12.3929602604167 1.17639620255463 3.5338046160794e-40 2.4871724767599e-39

GBP1 12.1252502824859 70.3975516666667 2.53751072782442 3.4601984828151e-65 2.04912072573079e-63

SLC30A3 7.97263465160075 3.03359479166667 -1.39402816627139 6.16956578124738e-06 8.76083052327568e-06

RAB6C-AS1 1.52435748587571 0.755389322916667 -1.01290898005521 5.22565693145282e-27 1.88462161831585e-26

AC012618.1 0.799407391713748 2.38439380208333 1.57661971053082 9.06243013101402e-37 5.32435665991171e-36

TCAF2 0.788188983050848 3.61809385416667 2.19861634288725 2.12860281620197e-54 4.08055354301811e-53

TACC3 6.25470084745763 26.4242018229167 2.07884710449217 3.65077935151317e-49 4.64974260557448e-48

AP1S2 32.1411858757062 66.0390704166667 1.03889665321416 1.23791434265918e-53 2.23920400795209e-52

RPL7AP6 8.44758742937853 33.4054242708333 1.98347110020353 4.7571429068041e-19 1.20631491002349e-18

AL021807.1 1.17137099811676 3.12261916666667 1.41455854917239 5.91738091627154e-47 6.51086971231179e-46

CCZ1 3.07073775894539 6.42232348958333 1.06451002340826 1.13410310282749e-46 1.21688673958899e-45

ARID5A 21.6135870527307 45.9078223958333 1.08680147461943 1.51504425634586e-54 2.93155545751834e-53

SSC5D 1.74557222222222 4.49966630208333 1.36611796534297 1.21483453806314e-24 3.94169398626489e-24

DNAH9 1.35746280602637 5.32142786458333 1.97090073750524 2.03516420827751e-43 1.7528429112052e-42

P2RY6 1.16891054613936 4.13321411458333 1.82209957399375 5.28583000999685e-54 9.79960209729725e-53

AGAP2-AS1 0.217147740112994 4.31744927083333 4.31343038282237 1.87095461487125e-69 2.0110058418226e-67

CNR1 18.4768574858757 40.5212455729167 1.1329591151081 1.01358973150503e-18 2.53193189915852e-18

KCNV1 3.66475404896422 0.8199415625 -2.16012338148093 1.05645260488351e-13 2.13669090578735e-13

PTCRA 0.655799811676083 2.97294755208333 2.18056661929445 1.61303084486706e-51 2.49536676874401e-50

AL355916.2 23.2810513182674 9.44831442708333 -1.30102732689843 1.60185801968978e-20 4.35094213790994e-20

AL031587.1 2.22051393596987 1.00440541666667 -1.14455191163777 9.33128794311464e-41 6.84478498233597e-40

WDR86 7.34386299435028 3.59260494791667 -1.03150884276732 5.97475764189794e-40 4.15019119727651e-39

DNMBP-AS1 2.8824581920904 0.805262708333333 -1.83976825387623 1.29333759757783e-54 2.52887619631543e-53

CCL2 38.1703041902072 116.221226041667 1.60635099017609 3.3103174846153e-32 1.53389866998309e-31

AL122020.1 2.0986093220339 9.7613615625 2.21764877706204 4.44229985266762e-30 1.85420138374985e-29

GABRB3 25.3468003295669 7.02256854166667 -1.85173293361746 7.38497910766665e-65 4.16132383354731e-63

RNPS1P1 0.96083352165725 4.034725625 2.07011217876749 1.47701006428732e-31 6.6132921130322e-31

LINC01150 1.38752603578154 2.99234171875 1.10876009449011 1.52247240426222e-36 8.8276814335067e-36

AL353691.1 0.443242372881356 3.10282020833333 2.80741239171349 1.61836164504495e-24 5.22274119916884e-24

APOBEC3C 11.6213018361582 44.8138258333333 1.94717220638425 5.33509871457353e-68 4.50937093624976e-66

RNFT1 5.5049209039548 11.0288280208333 1.00248575092958 3.25276304948144e-61 1.19300057012046e-59

RTN4R 19.5886244350282 7.84784317708333 -1.31964797362338 1.91353336696468e-37 1.16662796585929e-36

HOXD4 2.36923102636535 6.03629755208333 1.34924503930426 4.15049550891008e-23 1.26584326698676e-22

CLEC7A 4.28668069679849 11.32207921875 1.40120605927572 1.09030298302839e-45 1.0929479228794e-44

AC022148.1 1.82183719397363 0.893244583333333 -1.02826687500546 3.28618612372246e-32 1.52461654118311e-31

SNORD13 0.727504519774011 1.81156067708333 1.31620501255563 0.008551199403564 0.0101292873556678

NPM1P39 0.969313512241055 2.42550708333333 1.32325112495353 1.02854740545645e-22 3.08630611658265e-22

LINC00906 3.04995235404896 1.00863572916667 -1.59638146898504 2.96115904811532e-37 1.78485421392883e-36

HTR5A 2.00618032015066 0.612768697916667 -1.71103677754192 5.99402151638303e-16 1.32657498330329e-15

TMSB4XP1 2.36093870056497 28.9819171875 3.61772054498739 4.3858764766062e-67 3.24921805109531e-65

STAT1 40.0928852165725 82.8833941666667 1.04773684186245 1.33972299990777e-40 9.71612682655421e-40

TRIM38 2.41325541431262 5.47498973958333 1.18187564532697 2.08795045271883e-48 2.53099339428335e-47

TRIM22 20.0726388888889 48.2214800520833 1.26444563605987 4.8854029661596e-42 3.87725429601954e-41

C1QL3 9.35251219397363 2.87369911458333 -1.70244492683544 4.8903261727609e-10 8.41606804095224e-10

AC012073.1 1.72574312617702 3.67736390625 1.0914542125947 5.29956333449245e-46 5.43848676627412e-45

PDE6B-AS1 0.884058757062147 2.8244484375 1.67575499985912 3.30768744307989e-20 8.83332586587255e-20

MT1E 92.5944627589454 265.730116822917 1.52096391998879 7.50339137161522e-36 4.16867530789319e-35

RPSAP54 1.51340357815443 4.43116453125 1.54988913454814 4.09609570644645e-17 9.53635904111328e-17

GLUD1 565.336615630885 279.3388925 -1.01709368547236 1.9372299082057e-63 8.93865760374317e-62

AL513217.1 3.42698606403013 1.32788822916667 -1.36780660763643 1.35098417297636e-30 5.77639703299504e-30

RAB27A 2.66282989642185 7.44578479166667 1.48346364698485 1.65463015669187e-64 8.74086584195606e-63

TMEM151B 38.2750646892655 10.50301 -1.86560197484545 2.81520848363185e-51 4.28737115095285e-50

FAM110B 91.6199734463277 34.3023792708333 -1.41735349710027 1.84972520452684e-66 1.27864833376121e-64

BMP2 85.755470527307 25.48291375 -1.75069846018284 5.735365286804e-61 2.03141176205944e-59

RAB3A 138.5544326742 51.64275328125 -1.42381504304447 1.87525804316896e-26 6.59051659662196e-26

AC104024.2 2.49951129943503 0.65824453125 -1.92495051356258 2.97814326842398e-14 6.15727975053857e-14

EEF1A1P29 0.956117655367232 2.47187130208333 1.37034356576952 1.17604599964713e-18 2.92908858336973e-18

ANKEF1 0.560165301318267 1.55420895833333 1.47225595693578 9.9225774625092e-19 2.48132501746781e-18

HSPG2 5.11886440677966 15.4101419791667 1.58998445747501 3.89987204029408e-37 2.33853984486515e-36

RPS26P8 0.670569821092279 2.00905838541667 1.58306002956139 2.6960172569004e-24 8.62419420128384e-24

MAP3K20 4.84653093220339 11.62512625 1.26222201987871 1.47266062232972e-63 6.95028534827948e-62

CBR1 52.0325298493409 141.58164109375 1.44414844439703 1.11273940584062e-50 1.60274122785487e-49

SPA17 1.90866572504708 5.54968119791667 1.53984044017997 7.17853874557326e-47 7.81070380186862e-46

IL17RC 9.86108870056497 20.5460428645833 1.05904172051139 1.58895676145348e-44 1.47733254896137e-43

AC008035.1 0.83049279661017 2.41485411458333 1.53989647610181 3.50213552858065e-08 5.52117084815237e-08

SOX8 767.338021421846 264.597643072917 -1.53606203113876 5.7247057332157e-58 1.52727264145116e-56

EEF1A1P17 0.50098615819209 1.71217375 1.7729864640019 5.72106142368949e-21 1.58308239841527e-20

CCNB1 12.5467884180791 57.3857078125 2.19337334445011 8.06541146171509e-52 1.27422537069322e-50

CFAP61 0.690691101694915 2.43450302083333 1.81751474839782 3.24343141302725e-50 4.47415483124939e-49

FREM3 5.98460517890772 2.85403984375 -1.06825059603194 1.02206930881847e-35 5.64292719640126e-35

ZNF850 1.00987697740113 2.16467307291667 1.09996959804954 4.04083144273407e-37 2.4191996444061e-36

AREG 0.506383239171375 3.43491385416667 2.76197235869992 5.04310561881958e-26 1.74403103927748e-25

ALDH3A1 0.208720244821092 1.9857425 3.2500361077481 3.27069135507774e-59 9.74655540828054e-58

ATP5F1EP2 4.90664138418079 23.7327450520833 2.27407124284793 1.04759330932493e-43 9.15413420436893e-43

CCN4 0.979983804143126 13.0635711458333 3.73664761891451 5.46703290380469e-63 2.40899234232816e-61

EEF1A1P25 0.789843361581921 1.95557557291667 1.30795481232819 9.74827205735938e-19 2.43870737127133e-18

CMPK2 5.6279670433145 13.2399814583333 1.23421531628678 1.38132804027127e-29 5.6119280989391e-29

AC093673.1 26.1055078625235 85.7121269791667 1.7151451129898 9.09204269043433e-73 2.01269683129317e-70

HELZ2 6.32565748587571 13.38358484375 1.08117725329806 8.93144761994005e-26 3.05463065096166e-25

CDH22 14.0141164783427 5.71860557291667 -1.29314548469503 4.35026444385968e-41 3.25134463741565e-40

POLR2L 117.332430885122 253.138454114583 1.10932485118717 1.44613817311915e-58 4.09298924340192e-57

AD000090.1 9.85794274952919 59.1833635416667 2.5858331832786 5.31919036516518e-17 1.232067075738e-16

TOM1L1 0.853431073446328 4.23692880208333 2.3116723404958 1.32849435021752e-45 1.32315760269388e-44

GRIA2 107.502692184557 41.01391765625 -1.39018732777649 7.50976506823769e-59 2.15833201613416e-57

GLIPR1 5.97682038606403 18.754598125 1.64979425613695 5.31731613876237e-48 6.28579107439836e-47

GBP4 7.12035061205273 15.5124753645833 1.1234087316378 1.54714421764567e-14 3.23867462874268e-14

IGKV3-15 0.729609651600753 7.14492427083333 3.29172200090156 1.0951179088417e-23 3.41329492371965e-23

SHROOM3 1.21190626177024 4.98729640625 2.04097983426201 3.40785787337841e-42 2.73142746359791e-41

AC092645.1 1.5473 5.00259494791667 1.69292370190049 1.84113128145873e-23 5.69363648407202e-23

COL12A1 2.39624030131827 6.6467125 1.47186835958374 8.63959300284351e-18 2.07054042902275e-17

EDEM2 29.7721551789077 63.8444892708333 1.10059843853642 1.85109951785001e-80 1.72105977672105e-77

AC022613.1 0.755213653483992 3.33810666666667 2.14407330317623 7.94494494159342e-51 1.15964829942686e-49

KCNH3 19.244252306968 9.49225369791667 -1.01960505466491 7.25809406541286e-05 9.73697851138822e-05

MMP9 2.59541572504708 36.9657622395833 3.83215211394112 1.33817073733182e-42 1.10005680197547e-41

CDH9 2.32035009416196 0.583236927083333 -1.99218852492789 4.25145185607134e-30 1.77573556297499e-29

NR2E1 8.30675221280603 23.5794265625 1.50517220811947 1.2461276482162e-25 4.23692514510518e-25

RPS19P1 0.72789736346516 6.58368026041667 3.17708732836823 1.83104920064726e-36 1.05543583031729e-35

HLA-DOA 12.8348125235405 43.6675021875 1.76649778747946 4.13559709571785e-50 5.65451676432893e-49

AC009654.1 0.641236723163842 1.41576338541667 1.14265121485985 1.73983150595155e-06 2.5368279505347e-06

AL031727.1 2.57824435028249 12.6362569791667 2.29310827695033 5.66397818878029e-38 3.53191396446578e-37

CD79A 0.818325800376648 1.6734103125 1.03204398752938 9.51240776232662e-10 1.61389801405532e-09

AC104563.1 1.23191530131827 5.37042166666667 2.12413229912777 5.72763347995172e-23 1.73801149738417e-22

H2BC17 0.686791666666667 2.60912880208333 1.92562372659267 5.5734343908637e-26 1.92349689120472e-25

PALM3 2.54925174199623 6.32899395833333 1.31190234209548 1.13196001114003e-31 5.10397585042407e-31

SLC17A7 114.732401129943 39.9148831770833 -1.52327418105536 3.14001295431845e-15 6.76654779065379e-15

S100A10 158.709396468927 618.6427934375 1.96271908638113 7.92657054598047e-50 1.06192060016215e-48

ETNPPL 202.792584180791 77.61369171875 -1.38562181240723 2.25698102794636e-41 1.71861434130477e-40

RN7SL1 241.053942890772 913.385485364583 1.92186783607046 1.82225555459401e-29 7.35827188657451e-29

AL354714.2 1.28589030131827 4.12162546875 1.68044584113774 3.30624313250631e-53 5.85519914751951e-52

KRT8P30 1.87335706214689 0.74316609375 -1.33386931695027 1.73174648723591e-24 5.58090570713202e-24

GAP43 324.373017372881 834.621798333333 1.36346878536948 9.76152025494817e-33 4.64233936421384e-32

UBE2SP1 1.38085889830508 6.41269296875 2.21536442773285 1.05147242933398e-44 9.87481304215422e-44

TEAD2 6.31866026365348 21.85811875 1.79047863534943 1.23806699284212e-64 6.67626044671357e-63

IGFBP4 64.8522733992467 175.16427078125 1.43347947629995 6.60769764982114e-36 3.68647278123085e-35

HSPB1P1 4.20456139359699 22.6113941666667 2.42702273198913 2.47151056500073e-71 3.76702778329415e-69

AL645924.1 1.69201172316384 5.88572145833333 1.79847970411909 6.85865915359449e-07 1.01964156508706e-06

RPS29P17 0.480058145009416 1.70153119791667 1.82555254179352 7.68209412766073e-22 2.20683671101269e-21

MYOF 7.61664387947269 22.17788890625 1.54189469479566 1.07449158204669e-48 1.32846881437222e-47

NXPH4 5.37111012241055 16.2082038020833 1.59343201373114 9.12647228600914e-17 2.09488646041649e-16

MEDAG 1.19013102636535 5.77053541666667 2.27758477048299 1.19750930694213e-24 3.88615803884623e-24

MXRA8 22.7522743879473 53.9324147395833 1.24514186091747 4.54495076161272e-51 6.78822163953321e-50

PRR32 1.39941770244821 2.95289927083333 1.07730549738156 2.36013339704134e-14 4.90134917556218e-14

PLEKHA4 17.8069833333333 79.9687327083333 2.16699289386003 3.69122552217606e-69 3.73034448830782e-67

ITGB4 39.6828627118644 91.9573991666667 1.2124495577135 1.79136276708721e-30 7.61381347222531e-30

CD180 2.20474515065913 5.14222947916667 1.22178209145147 1.18736187786736e-37 7.28920241629038e-37

BX679664.3 5.3480988700565 13.1278333333333 1.29553078627048 4.64870575334916e-17 1.07918456284054e-16

UNC13C 3.4746606873823 0.924444635416667 -1.91021327560403 4.31340425500839e-25 1.42591559327788e-24

SATB1 35.3026335687382 17.5722106770833 -1.00648011081147 5.42157508975483e-51 8.04582512322355e-50

TIFA 2.12185437853107 6.12459161458333 1.52928800053175 1.11147720893422e-52 1.88232410748013e-51

SH3GL2 150.709898681733 52.0004078125 -1.53517933406289 9.18731615245872e-61 3.20521845881745e-59

SHANK1 25.3704830979284 8.87454307291667 -1.51540624515154 9.9956912234728e-37 5.85232614296211e-36

MRC2 23.7388813559322 94.2041691145833 1.98853895673051 1.69245421758012e-59 5.21910218505843e-58

BCL2A1 4.8317936440678 20.584729375 2.09094373527868 1.09508325058026e-41 8.50587846472011e-41

SRPX2 1.66585480225989 17.2857383854167 3.3752476673238 9.39138074617665e-71 1.2563505393896e-68

OAS1 7.39674952919021 29.6569639583333 2.00340758504529 7.18069351560489e-55 1.42654910173796e-53

GIHCG 15.0965009887006 31.6113564583333 1.06622873753902 1.03558018022388e-08 1.67129087408983e-08

FBLN1 11.7724286723164 36.2396378125 1.6221565552674 6.18740052631874e-32 2.82898236505771e-31

ERRFI1 18.0988899246704 38.58548109375 1.09215687983016 1.01469639055138e-34 5.31052051289133e-34

CDKN2C 22.6695661487759 100.618793072917 2.15007110355607 1.17598368978757e-55 2.51928763958524e-54

SLAMF9 0.201067467043314 2.3979484375 3.57604906131121 5.33637754249316e-39 3.50883806232887e-38

DUSP9 9.09138342749529 3.81140307291667 -1.25417765703872 2.64486067597592e-38 1.67911178797447e-37

DLGAP1-AS4 2.7313684086629 1.01182223958333 -1.43266806416942 1.00465496500316e-07 1.55214016901244e-07

MFAP4 29.1113647363465 78.90058625 1.43845354547371 8.59935662506903e-46 8.69994757579753e-45

FAM83D 2.90778677024482 12.6580975 2.1220672008163 1.06304798129738e-40 7.75799733603798e-40

BIRC3 1.33096073446328 4.87568401041667 1.87313661896251 1.52860153672967e-32 7.20881196436424e-32

PCDHGA5 4.62155141242938 1.7874090625 -1.37050738826776 3.97903971733661e-05 5.41022547118121e-05

LRP1B 17.8862427966102 8.88859046875 -1.00882380194058 3.41936055327867e-41 2.5700488879635e-40

RPS27AP5 1.23304858757062 5.42548703125 2.13752300076021 2.42228684730853e-20 6.52316059749488e-20

SGSM1 12.6051270244821 4.20752213541667 -1.58296789002324 2.85220413083388e-65 1.710862445576e-63

CASP7 7.00733225047081 15.16253609375 1.11357387172112 4.07980778158746e-55 8.30931278188596e-54

ELN-AS1 1.42069439736347 3.33358015625 1.23047616446648 1.82276482787706e-28 7.03201493244272e-28

CD8A 1.25725687382298 3.58410682291667 1.51133419589299 8.09303512248926e-31 3.49732716947915e-30

DTL 4.24459114877589 11.77445921875 1.47196329973686 6.99189483449381e-27 2.50412720430301e-26

RPL15P2 0.713819679849341 2.62821552083333 1.88045200360685 1.07731062954702e-22 3.23002114744064e-22

PIH1D2 1.81898526365348 3.94150848958333 1.11561402677741 2.01093099489571e-41 1.53439728560056e-40

AC061999.1 0.604397740112994 1.97548244791667 1.70863485680019 1.21325116112297e-35 6.66875712121834e-35

PRKCB 36.2011400659134 12.5873403125 -1.52406165635465 1.23429551537065e-34 6.4290546521897e-34

CD151 47.1316419491525 143.63763015625 1.60766590837515 1.13791173100055e-64 6.22337312881036e-63

ITPKC 18.0705820150659 39.9531682291667 1.14466693825145 5.23197955489157e-62 2.11957864538581e-60

CHAD 0.840121233521657 2.71011296875 1.68968355450851 0.000435732742713219 0.000559329721852292

ADAMTS20 1.88070117702448 0.34829359375 -2.43289479607344 1.16110826671404e-55 2.4937331332707e-54

LAMC3 3.95992349340866 9.93568864583333 1.32714740721688 9.5767368745807e-36 5.29525489690241e-35

SLC26A4-AS1 3.51024952919021 1.27502635416667 -1.46104652223904 4.28378323214913e-07 6.4239925448075e-07

IGHV4-34 0.488727777777778 4.31492359375 3.14223200283585 4.50714985924398e-19 1.14463878219942e-18

ZNF367 6.1209695386064 12.6653636458333 1.0490564064558 2.45112660360471e-28 9.41514133320173e-28

CBX3P9 0.824018173258004 4.08789880208333 2.31061142140159 7.99671374931755e-35 4.21362686791045e-34

SAT1 185.528831544256 457.911081979167 1.30342407842003 7.50016521207193e-63 3.25854140463733e-61

IFITM3P2 0.786077024482109 4.5461353125 2.5318980372649 1.95930278282926e-40 1.40020120087279e-39

ANKRD45 0.940120809792844 2.50200677083333 1.41216762699477 1.49193427689504e-19 3.86817036793966e-19

H1-2 43.7516274011299 97.5205570833333 1.15636968639286 4.47232603490766e-37 2.6680430740811e-36

LOX 1.64983017890772 20.4282924479167 3.6301791806199 8.01539225481297e-67 5.77698523171501e-65

TRIM17 3.22291803201507 1.51699666666667 -1.08714958150028 4.36048085212631e-42 3.4754882745516e-41

ALOX15B 4.02514293785311 9.02907692708333 1.16553848763652 2.5332941284669e-13 5.04029577560903e-13

PLA2G5 3.14445070621469 28.1462201041667 3.16206126406438 1.02753939156618e-43 8.99157411114029e-43

PEG13 7.10204759887006 3.29642734375 -1.10733174612352 9.43364894950337e-22 2.70332903776925e-21

AC015967.1 3.64825287193974 1.51266552083333 -1.27011271526414 1.45793498074174e-45 1.44682582170392e-44

LINC02166 1.34346774952919 3.65171442708333 1.44261225817954 6.98100857209398e-50 9.39304300999186e-49

DNAJB1 49.0207642655367 98.345718125 1.00446926342807 3.44151496792906e-57 8.4426082887389e-56

SPATA17 0.681877071563089 2.48097458333333 1.8633233759226 3.29795881297175e-45 3.18969812333862e-44

SPINT1 1.30166647834275 3.55828286458333 1.45082136283651 1.97195228847225e-46 2.07871466575751e-45

SHISA7 32.1923202919021 12.8098123958333 -1.3294672171087 9.59531189499188e-58 2.47812256510242e-56

LINC00900 0.553060781544256 1.709255625 1.62785822620679 7.58245154695402e-44 6.69495187633476e-43

HAUS1 18.7676918079096 37.8273499479167 1.01117848217234 3.19810246316937e-46 3.31856670215594e-45

CFAP77 1.63491756120527 6.58537817708333 2.01004840083867 3.47408219704064e-20 9.26571406396596e-20

PPCS 12.2481365348399 29.825604375 1.28398910212037 1.96490950518032e-64 1.03213254940192e-62

RNASE2 7.43570687382298 36.9605380208333 2.31344395428953 1.30593526671519e-54 2.5508262903959e-53

JAG1 15.9882058851224 44.9996380729167 1.49290534202784 7.48650014343788e-46 7.61550712074548e-45

FAIM2 290.974228389831 120.884004947917 -1.26726801555944 9.09550021332288e-63 3.92414910595682e-61

RASL10A 62.2004554613936 17.0794892708333 -1.86466030990323 3.70282186832504e-65 2.17892318485773e-63

PPP2R2C 35.7901570621469 13.4953553645833 -1.40709990804308 3.19951490625022e-28 1.22116132351648e-27

HNF4G 1.67332255178908 3.93308145833333 1.23294449843243 6.83839870969484e-30 2.82767422286818e-29

STBD1 0.731285922787194 1.80066848958333 1.30002510403923 4.14415322305332e-53 7.27672607957285e-52

RMI2 2.80677109227872 7.47864083333333 1.41386468995133 1.03986590770553e-34 5.43612779133661e-34

CD93 10.6117492467043 34.1587155208333 1.68659123542098 1.1033889044042e-40 8.04608497152788e-40

SSH3 7.06373535781544 16.45743171875 1.22023601340598 6.29900347386705e-52 1.00196723350349e-50

TBX5-AS1 0.433506403013183 1.89634916666667 2.12909941823176 0.00859693960813986 0.0101795779427764

VIM-AS1 0.60553945386064 1.62995088541667 1.42853562628369 1.39768704115335e-61 5.35876093407145e-60

AC091932.1 0.931781073446328 4.2788846875 2.19917186851483 2.30136917231921e-24 7.37445455096945e-24

AL355309.1 0.551373634651601 2.54224203125 2.20499919907933 4.07922835233457e-33 1.97328957366445e-32

COL6A3 1.78752509416196 15.9592494270833 3.15835740194393 1.88976845042304e-33 9.30128224870736e-33

SRP9P1 4.43308201506591 16.2694855208333 1.87578667162874 3.35808636907516e-28 1.27959762174628e-27

IFT22 10.4640133239171 21.9934627083333 1.07163848081326 1.73674476303363e-61 6.57734600175364e-60

S100A8 10.5081085216573 60.8561086979167 2.53389908204893 1.03477824585196e-34 5.41105216018481e-34

LINC01007 2.44048356873823 0.209370208333333 -3.54303896011133 9.32764221394192e-15 1.97233917407607e-14

AL359762.1 1.15322462335217 3.14847161458333 1.44897811429925 7.5368437804482e-64 3.67841496318725e-62

AC026801.2 1.45339482109228 3.11798119791667 1.10118555764398 7.80020598444875e-31 3.37156741703451e-30

RPL29P11 2.62441845574388 6.01421145833333 1.19637782189442 3.55315607084292e-15 7.64002510838623e-15

NEK6 34.1412145480226 91.1086133854167 1.41607307043235 2.02416455977454e-51 3.11326219925621e-50

GAPT 1.28778662900188 4.97672166666667 1.95030212783638 1.63914316635719e-56 3.8148365792389e-55

ECM1 9.74752123352166 21.85474421875 1.16483919592534 8.93308121309949e-30 3.67176492390771e-29

RAPGEF4 54.306652306968 21.28712015625 -1.3511481452159 1.38813330297391e-47 1.59040904305606e-46

CYP11A1 0.753606497175141 1.6294171875 1.11247272306215 1.61407202314012e-05 2.24300644722297e-05

RPS15AP1 7.29280630885122 17.6070992708333 1.27161126517735 1.11531172205041e-25 3.79700136791054e-25

STYK1 2.80827405838041 0.6854828125 -2.03449133650628 7.02431168887889e-16 1.54998310021007e-15

CIITA 1.64811534839925 5.56106234375 1.75454329346023 4.12333152046917e-50 5.64604930950841e-49

ANKRD53 1.04918460451977 4.03427369791667 1.94304042116221 6.33236433157333e-59 1.82558627512568e-57

AC044787.1 2.48332937853107 6.35437765625 1.35547520819896 1.19282213608843e-18 2.96967835326876e-18

HCST 2.50355150659134 6.96539114583333 1.47622823364724 3.8706732763102e-42 3.08906307180207e-41

COX6A1P2 10.1575688323917 42.50317640625 2.06501552179136 2.62604023539207e-36 1.49697174056148e-35

CALM2P3 0.571597551789077 3.59967776041667 2.65479612135707 2.30039020007854e-25 7.71568466278147e-25

SIGLEC7 1.49242377589454 5.15475979166667 1.78824795142941 3.49364804554002e-49 4.45266520951451e-48

AL450998.1 4.33739891713748 11.7556584895833 1.43845331211165 7.8830463613238e-27 2.81623913715305e-26

AC091429.1 0.702699152542373 2.60821973958333 1.89208635634578 2.77203203855792e-28 1.06083424072823e-27

AC093458.1 2.59893399246704 1.20851864583333 -1.1046802606752 5.08165726214036e-31 2.21971850574348e-30

OLFML3 32.8637625235405 89.02569125 1.43772407083562 5.2108863276233e-56 1.15766345593973e-54

YIPF1 27.3333005649718 54.9771758333333 1.00817311835056 9.89953287952393e-70 1.12933628156287e-67

CREG2 24.8259700564972 6.7079396875 -1.88790846860337 4.56801585663e-15 9.77808850627775e-15

AC087385.2 0.585575094161959 1.87782526041667 1.68113672141724 5.51611338186757e-28 2.08014861763998e-27

LRRN4CL 3.52813559322034 18.3344301041667 2.37757751023215 9.40358396346573e-53 1.6012787893832e-51

RN7SKP80 2.64756360640301 16.7119696354167 2.65814452645545 0.0369481960793845 0.0417965510461221

RUNX1T1 4.52380207156309 2.0618475 -1.13359817804571 9.14815762813357e-60 2.89795555528354e-58

AL133163.3 1.97920696798493 0.517345208333333 -1.93572331216974 2.1128067468775e-30 8.9371340896695e-30

RPS26P58 1.06040188323917 2.94973197916667 1.47597273565516 6.0940391283992e-25 1.99821297112649e-24

C1orf158 0.306456732580038 3.83059244791667 3.64381223484753 1.03189155894372e-29 4.22643690276618e-29

MBOAT1 1.27051308851224 3.92128552083333 1.62591545497666 2.22496753345876e-60 7.48160421060861e-59

AK4 17.7389393126177 40.8971329166667 1.20507995965086 4.7993094004896e-30 1.99872694965519e-29

DBIP1 0.384657062146893 1.71521020833333 2.1567406963584 4.59570693487362e-21 1.27949049938576e-20

DLX5 4.43630979284369 13.6699476041667 1.62357569395971 0.0248321176009208 0.0284488464536456

NFAM1 4.53121440677966 10.5003800520833 1.21247188328972 1.1167099325045e-37 6.86907747102917e-37

TYMS 3.67362080979284 14.2989560416667 1.96063519590483 1.09015890804698e-49 1.44486848860539e-48

RPS27P29 0.823577354048964 2.15810375 1.38978815773252 1.37765669555088e-22 4.11460428104218e-22

EIF4EP2 1.05780296610169 4.38004161458333 2.04987365102919 2.75681123485449e-44 2.51288749569212e-43

AC099677.1 1.50799274952919 3.74239078125 1.31133072086524 2.24812672513215e-22 6.6408127805929e-22

AC110611.2 0.763107532956686 1.74152494791667 1.19039286692888 1.98318701218854e-32 9.30072193988547e-32

MRGPRF 2.52104656308851 5.9412678125 1.23674805722162 2.66464454216447e-19 6.82776150772335e-19

VWA5B2 7.86500550847458 3.26845015625 -1.2668410797787 3.64158942808065e-37 2.18930990673003e-36

H3C6 1.79891878531073 4.93118427083333 1.45480410957263 1.79065067802097e-42 1.4604012876228e-41

ENG 39.6211615819209 86.1482838541667 1.12055088040407 2.38523466805327e-49 3.06944212127686e-48

DSG2 0.28835725047081 3.16325526041667 3.45548077636008 5.08388958720982e-42 4.02790485190314e-41

SCRT1 33.3688633239171 7.84788421875 -2.0881268809444 1.00678171895855e-60 3.50582510562439e-59

CFAP161 0.291532485875706 1.9523934375 2.74351524854335 5.35934986158433e-24 1.69340884751335e-23

NNAT 65.0664125706215 195.524631302083 1.58736544492141 0.000240534221356106 0.000313436148992067

GUSB 26.6135224576271 65.500308125 1.29934222111016 2.04066953218136e-75 6.22069671326433e-73

CDH6 3.86235338983051 9.6214075 1.31676778780424 2.48664811304852e-27 9.09684738236267e-27

ST8SIA5 4.13076144067797 8.75774291666667 1.08415135591384 2.46895436028637e-08 3.91925954665572e-08

TRIP6 45.6018708568738 142.303201145833 1.64180319746231 1.47988274818602e-72 3.02400216511198e-70

NIBAN1 4.48406548964218 18.5563204166667 2.04903140896704 2.99655439953861e-61 1.10557398927422e-59

AC092720.2 3.51118145009416 1.27005395833333 -1.46706676213351 2.88160173978625e-43 2.46360387822185e-42

FAAP24 2.15335861581921 5.38684067708333 1.32285079328513 5.60003730234073e-68 4.71188658991067e-66

ELOVL2 28.6988912429379 80.5836598958333 1.48949232966061 1.56640750617452e-14 3.27826084156615e-14

DOK2 1.38793606403013 4.67065770833333 1.75068460935144 1.14998658271152e-32 5.45649413256461e-32

BLACAT1 2.14485602636535 0.858702135416667 -1.32065112537462 6.69104179405733e-43 5.60450099822054e-42

S1PR3 8.18232881355932 22.0372938541667 1.42936365467 1.43831484038926e-48 1.76072840401832e-47

GPR1 0.0714382768361582 2.38808244791667 5.06301155429487 2.67968654573997e-52 4.39020011612641e-51

SERTM1 4.3857843220339 2.00424109375 -1.12977880357566 1.74659782295146e-07 2.66540718241956e-07

SOWAHA 15.7632 7.52523692708333 -1.06675152806757 7.43223000942062e-10 1.26919200133324e-09

NABP1 1.0013647834275 2.03572671875 1.02357627937922 2.36494975664173e-22 6.98146383945912e-22

ALDH5A1 78.2903553201507 36.2269373958333 -1.11177174483539 6.97288919130203e-73 1.60075153718841e-70

MPZ 3.45560306026365 8.97388192708333 1.37679469514098 6.24175415220501e-24 1.96587768394736e-23

DSCAML1 42.1023550847458 14.7675445833333 -1.51147096828838 2.03758192155033e-60 6.90142729166273e-59

DLGAP1-AS2 3.03239161958569 6.20778817708333 1.03362324750221 6.63641158821594e-43 5.56124711504621e-42

BATF2 5.02448907721281 13.42373875 1.41773773750333 1.36896171789588e-33 6.78822483847304e-33

RPS23P8 10.8969071092279 43.09893859375 1.98373363010363 1.94335165400085e-20 5.26159347788961e-20

AC015540.1 12.5672927495292 5.2377046875 -1.26266727237505 4.52408471035127e-49 5.71503771664279e-48

MCF2L2 4.36479458568738 1.59962661458333 -1.44817856986712 3.47785858177559e-59 1.03473248524987e-57

AHNAK2 1.78032288135593 3.58440140625 1.00959329577498 2.42791029462936e-05 3.34199362859079e-05

TRIM61 0.431576883239171 1.75293270833333 2.02208111867503 4.13826717390008e-33 1.99984468629713e-32

ENPEP 1.65051765536723 5.61323671875 1.76591433027264 1.19185065778391e-40 8.6707601649029e-40

ATF5 16.7385626647834 38.2536995833333 1.1924236307279 7.3094565445372e-56 1.60282245808572e-54

CBLN3 1.21915696798493 2.99085869791667 1.29467586573142 2.44753563265875e-31 1.0836172640307e-30

NTS 0.713159039548023 10.92160375 3.93681706597119 0.00942485527132607 0.0111301399574691

DNAI4 1.57722283427495 5.3615946875 1.76527565981535 3.55270839489956e-54 6.70006213013766e-53

GPR4 5.83477664783427 11.6941530208333 1.00303803806951 3.73076797716631e-35 1.99406813841355e-34

OXTR 8.48839166666667 20.5772356770833 1.27748605426766 7.44694073671433e-21 2.05210229696507e-20

C2orf66 0.837050847457627 3.81575401041667 2.18858099991236 2.61619041330366e-58 7.18582876445813e-57

SPON2 2.28523724105461 8.8675915625 1.95619837590732 6.09782018046737e-47 6.69751720353165e-46

AL355922.1 1.51210296610169 5.47968640625 1.85753694970414 1.02121894185207e-26 3.62742430252899e-26

FTLP2 1.72365753295669 15.6521507291667 3.18281584412393 3.40424837213494e-62 1.40048669203206e-60

KRT75 0.0476470338983051 2.13515104166667 5.48580791328013 8.02009846695282e-49 9.94684939491267e-48

CPVL 18.4713327212806 63.4279227083333 1.77983013239812 4.22187347277214e-54 7.87419631155446e-53

KLRC4-KLRK1 2.06059915254237 0.944623385416667 -1.12525272844077 1.09372302753797e-35 6.02600879913142e-35

GPC1 90.9348077683616 192.135615729167 1.07922043681983 2.71717598074189e-27 9.92065332061564e-27

SECTM1 2.97275051789077 14.0926316145833 2.24507074307073 3.07339689039909e-48 3.68470761940497e-47

YWHAZP2 1.16398460451977 2.62914177083333 1.17551996177103 6.2889559146364e-23 1.90461132300755e-22

RANBP1P1 1.93259223163842 4.27882119791667 1.14667612589369 1.47803030112793e-28 5.72463516964671e-28

CADM2 95.1114811205273 40.29615640625 -1.23897726673382 4.13285633662714e-61 1.48935006937174e-59

LRRTM3 27.367920386064 12.5237408854167 -1.1278202476664 7.48213885124056e-48 8.77240680572624e-47

ARHGAP18 6.63208512241055 20.3205654166667 1.61540611647187 2.1155018478012e-61 7.96310867608569e-60

WNK2 15.13138413371 6.70725265625 -1.17375010996972 3.42217375024603e-35 1.83492851458549e-34

IGHV3-11 0.569553201506591 3.60236822916667 2.6610431447769 1.44896412999991e-13 2.91217985271815e-13

DSTNP3 0.674439548022599 2.139020625 1.66518935228492 6.0347171320544e-38 3.75805643236944e-37

ARPP21 25.8443034839925 8.2594715625 -1.64572493481415 1.84744777673925e-72 3.69390230198563e-70

H3C9P 0.933879001883239 1.94308223958333 1.05703941902075 5.00641489636616e-29 1.97862454830879e-28

PARPBP 0.832706026365348 2.69427307291667 1.69401690979947 1.44669823599179e-48 1.76869964822144e-47

ANGPT2 4.84800687382298 20.1521232291667 2.0554681997427 1.20007347010807e-51 1.86427453439094e-50

CHRM1 20.0819745291902 6.16344130208333 -1.70409313022001 6.57209213641338e-35 3.47478115657113e-34

PDZK1IP1 0.726229048964218 3.65297005208333 2.33057338237785 1.87913393980018e-40 1.34497673635814e-39

TCEA3 2.28955221280603 12.7517609895833 2.47755912290326 1.019345446552e-54 2.00155528813458e-53

CD274 1.79162758945386 6.03280848958333 1.75155899731397 8.10877408000147e-40 5.5845427413936e-39

AC093249.4 0.843061676082863 1.7267521875 1.03435096754073 3.7981312086755e-20 1.01067901867946e-19

ITGA4 1.27136487758945 3.71025604166667 1.5451406109005 7.26035187764974e-40 5.02629349087479e-39

OPHN1 62.7267831920904 26.2726919270833 -1.25551755097405 3.75540801580266e-54 7.04660061088299e-53

CPNE9 4.90236600753296 1.73134677083333 -1.50158348839512 3.09405311727458e-08 4.89233994181299e-08

AC026620.2 4.86796304143126 1.99945359375 -1.28371241750282 1.39169728641741e-25 4.71892980323336e-25

GRIP1 2.43872984934087 1.05888260416667 -1.2035873022518 1.45397868509743e-45 1.44426995990314e-44

NRIR 0.718487146892655 1.780614375 1.30934085448129 3.21202404307232e-12 6.07296259084187e-12

SLC2A4RG 18.8815781073446 47.1600866666667 1.32058702053044 3.64263685557409e-72 6.98297240509281e-70

RPL23AP65 2.11658243879473 7.45130984375 1.815757373671 1.83189431038156e-26 6.44542567673512e-26

AC138207.3 10.9322162900188 22.2652741666667 1.02620946825837 1.91865984453122e-32 9.00491666053964e-32

AL353597.1 2.30197052730697 0.481292760416667 -2.2578827347832 4.59152637976308e-59 1.34455800049913e-57

IFITM3 255.829464359699 879.262593020833 1.78111166095616 1.24151061652117e-61 4.80956039879399e-60

AL158847.1 2.34814981167608 0.986546458333333 -1.2510655582631 7.62329311397408e-38 4.72674676406629e-37

RPL7AP30 2.83935287193974 6.73397369791667 1.24589592928123 6.48951034832402e-19 1.6373466068804e-18

CIP2A 3.35676822033898 6.72727036458333 1.00294831569163 2.62814438136177e-29 1.05324018903927e-28

FOXD1 3.14821548964218 7.48030161458333 1.24856214836631 8.13795140073442e-28 3.04354799470347e-27

RPL24P8 2.9043393126177 16.9570079166667 2.54559970949002 3.60780905778652e-24 1.1483603120428e-23

DHRS13 3.12556002824859 6.60976192708333 1.08048359852717 1.72556193963855e-28 6.66393027364046e-28

MXRA7P1 1.82536666666667 5.22371588541667 1.51689014033638 1.04755247197826e-37 6.4522153747717e-37

SLC35D2 2.43512401129944 4.93955979166667 1.02038723111649 4.18301323472699e-65 2.43835658853781e-63

TIMELESS 12.6455388888889 26.3699979166667 1.06026893854922 1.01871880836375e-30 4.37585498764702e-30

MET 2.33400145951036 14.8573928125 2.6703036044634 2.39356075750882e-25 8.02384393111168e-25

GPR156 0.660647881355932 2.17152916666667 1.71675789051391 1.09657374983251e-49 1.45233539018059e-48

GLI3 3.45755946327684 7.22205166666667 1.06265467825654 2.74165376999755e-22 8.05642412343623e-22

SERBP1P5 0.489634133709981 2.17818192708333 2.15334841571298 8.97051786567285e-42 6.9912657355554e-41

NET1 62.3118259416196 30.1030920833333 -1.04959431009988 2.71373111603013e-55 5.62562208501452e-54

AC009902.2 2.5597972693032 10.2334175 1.99918655926765 4.25487822955009e-36 2.40411609475794e-35

PDIA4 84.1046801789077 232.186084739583 1.46502352233919 1.10316518336569e-68 1.01551270221213e-66

PTPRR 3.79642236346516 1.55064015625 -1.29177657411212 2.36112416928433e-12 4.49524971105172e-12

SP6 0.708719444444444 1.96330401041667 1.46999704962166 2.85015300975465e-38 1.80697562960749e-37

PHEX 1.82949830508475 4.49161958333333 1.29578766508334 1.63841278221691e-50 2.32037727513519e-49

AJ011932.1 0.912325329566855 4.20961203125 2.20606699953177 3.07530362687314e-51 4.66057627886765e-50

UPP1 7.78580032956685 32.0876691666667 2.04310174503647 2.42067605050175e-74 6.33978467029296e-72

RPL30P4 0.472695056497175 3.02764354166667 2.67921367822403 1.21834597599381e-24 3.95239766636513e-24

ROR1 0.868404096045198 2.76705583333333 1.67191331886177 1.32924527614617e-51 2.06321501752404e-50

AL162724.2 1.74901807909605 3.89630947916667 1.15556307144032 4.42222485755839e-35 2.35822400993112e-34

IGFBP7 242.860986111111 775.055112604167 1.67416815126904 6.23875053470219e-60 2.01405496862478e-58

COL9A3 12.8033366290019 44.4528008333333 1.79575448715839 4.082625084978e-24 1.29638684178904e-23

RPS29P5 2.44263300376648 12.1734556770833 2.31722973821342 3.45154877923616e-32 1.59774830843655e-31

HOXA9 0.145983003766478 2.52741802083333 4.11379198110371 4.3110140155396e-57 1.04515391941276e-55

GALNT14 5.4591327212806 2.57090510416667 -1.086395412818 9.3972043803934e-34 4.68977497191131e-33

GGCTP1 0.882276930320151 1.92558473958333 1.1259931463729 1.59016397323778e-32 7.49153764437713e-32

S100A9 33.2620005649717 204.405573489583 2.61948768632246 5.67489559286218e-37 3.368167365122e-36

AC007240.3 0.681981403013183 1.8294675 1.42361948284238 3.14159609071489e-42 2.52454534601743e-41

PKIB 2.70167645951036 19.32383984375 2.83845498133424 4.53448155675823e-58 1.21848428605781e-56

CLSTN2 24.0207467514124 11.2969419791667 -1.08834870613342 8.23551766625411e-32 3.73601978541096e-31

EMP1 38.1423983050847 126.228819479167 1.72657386674326 2.06387021016988e-54 3.9605435044488e-53

TPT1P9 2.3629854519774 19.2129936979167 3.02339967963156 3.64570608014071e-33 1.76863826141968e-32

TRBC1 1.42475673258004 6.30625802083333 2.1460685891202 2.57836713953472e-44 2.35656328250627e-43

APOBR 4.40497443502825 10.0870430729167 1.19529777180979 1.90434375241166e-44 1.75825581311295e-43

AC140912.1 4.63611360640301 1.78685619791667 -1.37549238526649 7.09165121518114e-42 5.55941207193479e-41

SNORA63C 0.732941290018832 1.55769984375 1.08764771892862 8.85704174469183e-09 1.43264345200543e-08

USP43 10.6473747645951 4.88426625 -1.12428400943354 1.26988378827093e-41 9.81035792870946e-41

IGLV1-47 2.0044054613936 9.43320802083333 2.23457410853036 2.53198564350298e-23 7.78734254729373e-23

RAB7B 3.7077788606403 11.2476977604167 1.60100262662029 2.19329237507343e-33 1.07695462673595e-32

TNFRSF1A 42.9049670433145 130.51406515625 1.60498870947465 8.90659703381993e-71 1.20013168002813e-68

AL080243.2 4.22116511299435 15.9515740104167 1.91798562068557 1.30855419518021e-29 5.32557786372861e-29

AL365205.4 3.42839199623352 1.43631864583333 -1.25515622896358 6.39073865476663e-28 2.40363643376589e-27

SIX5 1.57938140301318 3.3547165625 1.08683127129541 9.95556172624542e-31 4.28130597362474e-30

DLGAP3 20.7767217984934 7.3368115625 -1.5017429034807 3.82577281239337e-44 3.45508719992495e-43

SNORA23 0.480249670433145 2.66693395833333 2.47332557069997 3.2344635577543e-09 5.35144139660479e-09

P4HA2 3.19889369114878 7.46634625 1.22282936666746 3.53967009722559e-42 2.83463244866106e-41

DLGAP5 2.06881553672316 10.81575265625 2.38625714434648 1.93609838122621e-44 1.78668731508195e-43

SLC25A43 7.04147961393597 17.6798166666667 1.3281527977344 2.55953710155206e-52 4.20446929358309e-51

LINC01765 0.0786053672316384 2.29064973958333 4.86498524045463 2.60085737625325e-34 1.3374707663559e-33

LINC01711 0.778748258003766 3.24838265625 2.06049265451445 1.86193687192496e-47 2.11114122764906e-46

RAB1C 0.350168879472693 2.41606192708333 2.78653465537593 3.34518663310025e-32 1.54928382173099e-31

TAFA3 0.499104001883239 3.65740526041667 2.87340811728029 1.31303455319483e-52 2.20558965823468e-51

PTGER4 2.10387594161959 5.84644328125 1.47450958305853 1.45610839154891e-42 1.19384195506402e-41

CLEC4F 3.96153446327684 1.66433213541667 -1.25111598541701 2.70663837288562e-30 1.13920191362173e-29

AC024940.2 2.46151892655367 7.62292151041667 1.63079518898756 7.06077289966638e-28 2.64814586666592e-27

MADD-AS1 2.53044420903955 0.826037760416667 -1.61511102856198 5.09811549891706e-45 4.87400810809063e-44

CYS1 9.61158728813559 4.34325984375 -1.14599643458939 1.00591032054297e-46 1.08434216872444e-45

SCN2A 9.0812643126177 3.58950541666667 -1.33910809185384 2.51286996007202e-23 7.72982909967563e-23

PTMAP2 4.73693145009416 26.23957375 2.4697195848146 1.22940506284626e-44 1.14936084181127e-43

TCF19 9.46117080979284 25.5045700520833 1.43066514874969 6.81899329769241e-46 6.96316201925263e-45

RPS15P5 0.887873775894539 2.12644484375 1.26001693818112 2.44592650407972e-23 7.52764040770645e-23

PLEKHG1 6.74069354990584 13.74880890625 1.02833769672812 7.02271314384717e-32 3.19910217809501e-31

AC015936.1 3.21894505649718 1.49536536458333 -1.10608992889731 3.80467392041391e-35 2.03240194053711e-34

LINC02308 0.952709510357815 4.93269677083333 2.3722683071388 3.14283660099645e-45 3.0438045101838e-44

HEXB 22.2105349340866 55.6061660416667 1.32400072881938 3.87048477683144e-72 7.26986509345259e-70

B3GALT5-AS1 1.46642302259887 4.01023963541667 1.45138710753264 1.06330908171184e-32 5.05166897660492e-32

ACTA2 15.4362124764595 38.4757540104167 1.31763079182034 2.96888600490482e-28 1.13500072494254e-27

TLCD3B 27.1009756120527 11.4837316666667 -1.23875326282835 1.83606142752338e-57 4.6074982786501e-56

ADGRL2 3.07888945386064 6.89976161458333 1.16413644892301 4.9134593230425e-23 1.49436990696721e-22

NGB 6.07126082862524 1.93416859375 -1.65028260016474 6.70153075646741e-06 9.49809180003899e-06

GPNMB 17.5093188794727 78.5895105208333 2.16621380294277 1.62451849022385e-52 2.6971358326529e-51

AL133240.2 3.70014929378531 1.46669322916667 -1.3350163306915 8.12326598816747e-12 1.5076567626507e-11

KCNAB1 11.5631074387947 5.74763135416667 -1.00848971874559 7.70560718274415e-28 2.88417402502269e-27

KIF24 1.98131920903955 4.03063458333333 1.02454566401743 4.62318277414768e-26 1.60119358698596e-25

KHNYN 4.0531118173258 10.23538 1.33646278288781 1.90203191990721e-49 2.46468874917593e-48

SPRY4 10.8002857344633 49.8104709375 2.20537957004808 1.86853442983114e-45 1.84032826921134e-44

EEF1A1P22 1.19012697740113 2.80560895833333 1.23719843603095 5.27729761478731e-18 1.28092088692038e-17

FABP7 82.4639572504708 392.327891770833 2.25022430506821 1.04066103207968e-40 7.59760184197945e-40

MATK 10.1842806497175 4.06071265625 -1.32653923331688 1.06415826653454e-27 3.95918826854937e-27

MYBPH 0.577798069679849 10.7091565625 4.21213566566268 1.22446747508265e-68 1.11068159508107e-66

IGHV3-23 1.01015663841808 10.38576171875 3.36195610629563 5.6291281823754e-20 1.48599714013729e-19

CASP1 6.37330701506591 21.2603882291667 1.738053876634 2.14985450205436e-59 6.53211510877464e-58

AL606970.4 0.187342372881356 2.53872036458333 3.760352344812 1.00981944348995e-53 1.83733782306219e-52

SLAMF8 0.653178060263653 3.06579489583333 2.23071294491952 4.9573681981098e-47 5.47725856469707e-46

BRSK2 32.5081238700565 16.175484375 -1.00699138229989 2.21240913174422e-45 2.16411087873665e-44

AC024293.1 33.9624255178908 106.7844703125 1.65269044921715 7.71646056826138e-27 2.75831572985045e-26

CLIC4 189.012136864407 411.8927659375 1.12378991252619 2.81628301265253e-56 6.4098877136198e-55

ARPC5 29.4296619114878 59.08190703125 1.005445425144 3.51331712401202e-67 2.63427951294369e-65

AC083864.2 4.59144755178908 2.04890171875 -1.16409828259915 7.02766223761844e-33 3.36623310049467e-32

TAGLN2P1 0.237677966101695 6.58151661458333 4.7913400005402 6.17440000536522e-65 3.51109994188888e-63

TMEM154 0.492737146892655 2.17085020833333 2.13937003743458 2.81296773752681e-70 3.48714233862074e-68

SCIN 6.79610084745763 15.6557735416667 1.20391562689834 5.129629074293e-28 1.93558142525321e-27

AC092809.3 1.37015517890772 5.480910625 2.00007631247792 6.33104607571562e-34 3.19213128465108e-33

DEFB131E 2.50097429378531 0.863725572916667 -1.5338453186516 4.87661639380164e-36 2.74374226453076e-35

PRELID1P6 1.05746313559322 4.52311223958333 2.096708426251 3.97733811243789e-43 3.37248528047344e-42

RCAN3 1.50664081920904 4.29101395833333 1.50998307236053 1.48981801769369e-49 1.9427185160599e-48

IGHG2 2.3385920433145 17.4084668229167 2.89607703211945 4.56285009817107e-28 1.72732486920788e-27

FCGR3B 0.919956308851224 2.86356520833333 1.63817520597786 1.15823754302036e-27 4.30318224025247e-27

GRIN3A 9.21468870056497 1.86059692708333 -2.30816987910802 8.81921396909124e-51 1.28320253329618e-49

SERPINA5 0.452475376647834 10.4508132291667 4.52963211563541 2.03337627933976e-82 3.15088599286024e-79

FBLN7 1.73981676082863 7.20017151041667 2.04909590437887 2.36916562420917e-34 1.22136497871277e-33

RPL13AP7 1.24888935969868 2.5030934375 1.00306647324568 3.29052599928998e-22 9.62821887597123e-22

TMSB4XP4 2.71910602636535 31.8388644270833 3.54958456855637 1.27347003975434e-66 9.00386896928972e-65

IGHV3-49 0.367365489642185 2.357835 2.68217475185903 1.4558715357128e-19 3.77677611698933e-19

C7 2.92926464218456 8.74772270833333 1.57836895122504 4.54651837804277e-05 6.16693480485122e-05

SNCG 88.173829519774 32.2695190104167 -1.4501784453886 1.00932150396979e-29 4.13763963102254e-29

LRIT2 2.1925831920904 0.5287678125 -2.05192531921154 1.66807030000636e-70 2.18434980483227e-68

TOX 49.0948007062147 21.7724825 -1.17306433359219 7.56918129658617e-48 8.8688674360441e-47

ATOH8 39.9111201977401 14.2674855208333 -1.48405967306949 6.76977078231418e-53 1.16451329969595e-51

FOXM1 8.87086873822975 29.88244015625 1.75215065933061 4.1353371615353e-34 2.1067560142123e-33

MCAM 23.7670467043314 66.3811342708333 1.48181063633001 4.00327243470693e-58 1.08041873618832e-56

SPATS2L 12.0794702448211 27.7616947916667 1.20053845856559 4.0877619824148e-66 2.72444998195273e-64

RPSAP12 1.51316257062147 6.45411166666667 2.09265154338211 3.09941688177467e-21 8.69285926343891e-21

ORC1 1.95532627118644 6.92613557291667 1.82464126754683 7.63610408555292e-46 7.75496206831549e-45

LINC01023 10.5429358286252 22.1053847916667 1.06812118608764 6.10971944451298e-51 9.00239564744206e-50

CTF1 4.60760809792844 14.3232496354167 1.63626892795691 3.63709074650828e-62 1.49297356360533e-60

SLC5A9 0.57857829566855 1.58544151041667 1.45430054572828 2.1403990417952e-37 1.30152780190261e-36

BOLA3 2.80735089453861 5.72820807291667 1.02887449888176 2.52516520471351e-66 1.71959146746758e-64

HS6ST3 8.5081395480226 3.36411776041667 -1.33861548836429 8.32035403318551e-05 0.0001112591566569

BIRC5 8.88798964218456 44.6665636979167 2.32926622847904 3.89849762896131e-41 2.91955551391605e-40

ANGPTL4 21.2049012711864 75.8973708333333 1.83965214415237 2.9404210918374e-23 9.02114010934111e-23

HLA-DRB1 251.467872645951 957.7125003125 1.92921853822 1.52697450742082e-57 3.86314162795784e-56

TNFAIP3 6.94669114877589 14.46022453125 1.05769209255093 1.40899191049669e-37 8.62984340437614e-37

TROAP 2.69951073446328 11.5101234895833 2.09213345319835 1.13312108881304e-40 8.25642109971727e-40

CD3E 0.875904472693032 3.86287776041667 2.14083058337145 1.8363884321653e-46 1.94020698273374e-45

TMEM271 9.44718898305085 3.90787713541667 -1.27350000903581 2.54170591661819e-32 1.18513093078022e-31

DDX60L 2.99570998116761 6.38711661458333 1.09226682991155 3.55943408312703e-44 3.21767995992937e-43

AP001269.2 0.34640720338983 1.98264526041667 2.51688573452843 2.58661003302372e-33 1.26507137201673e-32

KCNK7 6.84056770244821 3.28898223958333 -1.05647484244928 3.66157102670681e-29 1.45516131064144e-28

GREB1L 3.14741459510358 1.54852755208333 -1.02327017873084 1.91875349075716e-36 1.10223802322141e-35

FNDC3B 4.38228615819209 12.1620004166667 1.47262494523908 8.95352322447257e-65 4.96987356295724e-63

LTBP1 13.6202173258004 31.9656713020833 1.23077366844665 2.42452043720604e-32 1.13105763998611e-31

FEZF2 6.19883870056497 2.911016875 -1.09047476152504 1.70771236687031e-22 5.08648269453042e-22

EIF5AP4 0.865148728813559 4.34791833333333 2.32930476785856 2.99563756484046e-33 1.4593628639824e-32

BCAT1 5.51269190207156 30.94396 2.48882895676575 2.01482128870688e-53 3.62336575082248e-52

MMP14 39.0129265065913 160.720749270833 2.04253206543583 3.41729506038242e-63 1.53119522042918e-61

NRXN1 29.4211819679849 11.9291690104167 -1.30236165870427 2.09837319388828e-51 3.21675593902330e-50

HOXC9 0.310168455743879 3.78127536458333 3.60774903854078 6.21452618673068e-66 4.01246925146726e-64

SCARNA6 0.849713512241055 3.11348041666667 1.87347979282072 6.92043211994507e-07 1.02857833322979e-06

AC120036.3 19.7884325800377 8.60097625 -1.20208501594176 1.52597615083539e-59 4.74518255244666e-58

MBNL3 1.29244270244821 3.34179083333333 1.37052111291954 4.27247368917033e-50 5.83308724303394e-49

AL354919.2 0.329787806026365 3.85936375 3.54875306523072 2.92052975731465e-60 9.7478154520478e-59

MYT1 34.9449888418079 15.768769375 -1.14801551219827 3.15084085880346e-37 1.89795548329933e-36

TNFRSF10C 0.80210527306968 2.86121760416667 1.83476572100891 4.70794501800126e-48 5.59388099742706e-47

CD63 444.191355131827 1215.08706255208 1.45180646800343 1.8864964448844e-76 7.30820862346363e-74

PSPH 36.5515052259887 104.277636979167 1.51242707157048 3.77893268122444e-33 1.83088205334467e-32

TTC39C-AS1 0.610035075329567 1.60869473958333 1.3989264900034 7.80567717494771e-28 2.9204540657576e-27

CCDC153 0.825027683615819 2.32732692708333 1.49615945039522 3.42738486538721e-55 7.04223442783151e-54

NCF2 9.40663968926554 21.3639457291667 1.18342677476936 3.96652383824666e-43 3.36484994398707e-42

SAMSN1 6.6356 13.29390453125 1.00246607142669 3.65339101037923e-29 1.45252952401116e-28

SNX10 18.1389646421846 45.451104375 1.32522323925857 1.67997046399047e-27 6.198224360695e-27

CASP8 2.01966106403013 5.9296665625 1.55383777856131 4.95809684633967e-73 1.15244763572108e-70

H3P16 1.89050155367232 10.6963026041667 2.50027124565224 1.94881454441105e-31 8.6632097665129e-31

AC010424.1 4.59543634651601 10.3528573958333 1.1717552474052 1.85678659520481e-11 3.39131192788856e-11

CFAP73 0.529917843691149 4.11555416666667 2.95724609262329 3.27256471278251e-25 1.08802683415324e-24

GGT8P 0.125324152542373 2.13051630208333 4.08746670705477 3.64956015703385e-31 1.60547837993954e-30

CMYA5 0.976852306967985 5.41910307291667 2.47184172916777 1.38568502306435e-39 9.4314835299713e-39

BRCA1 3.67597843691149 7.6736828125 1.06179082927775 3.21197435326672e-44 2.91065609644223e-43

HOOK1 1.97364915254237 0.717676510416667 -1.45945994352452 3.46139404880428e-14 7.14061941717383e-14

LINC00643 13.547 6.51036192708333 -1.05716374662799 4.48569702205147e-22 1.30534485328712e-21

ST8SIA3 25.2713601694915 6.92524880208333 -1.86756550729066 1.75253199566668e-51 2.70218345434676e-50

KIF15 4.88291949152542 11.3661930208333 1.21893322412144 5.15222668122512e-24 1.62907082362491e-23

MIR3125 2.47737099811676 5.2492715625 1.0833072997305 1.40272891516744e-12 2.69710931419073e-12

HAR1A 6.00577796610169 1.80242416666667 -1.73641257709497 1.47193638549527e-50 2.10220100524459e-49

SMPD3 15.327011346516 6.123385 -1.32367511093072 4.46412475080852e-51 6.67286171553733e-50

PSORS1C1 0.397087429378531 1.76754666666667 2.1542197102788 2.40897723538135e-52 3.96415324707223e-51

PLXDC1 4.82491224105461 9.88439130208333 1.03464942318052 3.14194930190262e-25 1.04591026260077e-24

KIAA0040 6.35515588512241 34.6820191145833 2.44818847316039 7.51192719600349e-72 1.29337302046005e-69

EXOC3L2 0.465100706214689 2.76055015625 2.56934077801419 9.09790699280255e-52 1.43010399893046e-50

GPR22 3.59421049905838 1.24937359375 -1.52446996128604 1.31872281365776e-10 2.32146650761773e-10

SHISAL1 21.5365411487759 6.074813125 -1.82587463217258 1.528870775222e-52 2.54971767401373e-51

RPS20P14 6.10091304143126 14.51870296875 1.25081550296955 5.22563392785859e-24 1.65200038912837e-23

HSD3B7 3.89164063088512 10.79628328125 1.47208433961028 9.26354613161997e-66 5.83917424804994e-64

MXRA7 21.4015794256121 47.0102157291667 1.13525703034126 3.3754529880413e-46 3.50064407767027e-45

CKS1B 10.1533943032015 23.9261266666667 1.23662475760361 1.26361332377842e-55 2.68843132215786e-54

AC135782.1 1.89887495291902 0.673936458333333 -1.49446042310755 2.66133666803493e-45 2.59232872404974e-44

FCHSD1 2.61413907721281 6.80724 1.38073407780245 3.93305251477221e-60 1.2921397793673e-58

MICB 1.93697683615819 5.9054690625 1.60824495406449 6.9052679528019e-51 1.01504709551266e-49

BACE2 3.08340833333333 10.15613578125 1.71975372599113 1.93074481379417e-63 8.93089547574691e-62

AL139396.1 10.9883882768362 5.13197010416667 -1.0983951230632 1.8608393657838e-32 8.7401636794013e-32

RAB34 9.48160974576271 60.8294729166667 2.68156658399454 3.48921835779716e-67 2.62680224142665e-65

MT2P1 10.0604565442561 47.487406875 2.23884920146561 1.46374586479086e-49 1.91140128902992e-48

SLC2A10 5.31356963276836 22.1010086979167 2.05635892819728 1.65325966219069e-57 4.16563189951706e-56

AC124798.1 7.70606596045198 3.40954942708333 -1.17641343557981 1.49281102757001e-32 7.04360814924731e-32

PTGS1 9.27709708097928 28.58568 1.62354726583822 4.58857166107691e-49 5.78863568776968e-48

AKNAD1 1.0499354519774 2.26539067708333 1.10945923466207 2.55437252587781e-21 7.18477645117197e-21

SMIM5 1.63537711864407 3.59439119791667 1.13612407388645 4.66237437256439e-26 1.61417303232667e-25

ACSS3 2.36131224105461 8.66871651041667 1.87622957928799 1.13978659429126e-37 7.00639065151933e-37

CBLN2 7.50499510357815 1.66840833333333 -2.16937870729792 3.86822883920142e-46 3.99609529249724e-45

IGKV2-30 0.175998917137476 2.99849260416667 4.09059895679404 6.42327675604492e-15 1.36706914591571e-14

FTLP3 7.17733290960452 108.439785989583 3.9173025213836 2.7362568167857e-62 1.14082276924059e-60

MAP3K9 4.7833983992467 2.0123778125 -1.24913476701132 5.6498712732938e-15 1.20563870010211e-14

AL078596.1 1.2853397834275 5.38701505208333 2.06733630567755 1.14876361182215e-35 6.32364101889665e-35

CDRT15P4 1.80149910546139 0.82596640625 -1.12504292481081 2.70842878920773e-34 1.39106580099772e-33

PKMP1 0.230191242937853 2.11762578125 3.20154280846332 1.72163253567762e-38 1.10468450658818e-37

RINL 2.58605338983051 8.14085526041667 1.65442830851591 3.25538697206364e-64 1.64367016453971e-62

BCYRN1 599.875347269303 259.367810677083 -1.20966330076441 2.43597204829518e-21 6.85589529863007e-21

H1-5 0.126976883239171 2.19338880208333 4.11052379176041 1.40389820720962e-40 1.01696482910257e-39

ERHP1 0.323897693032015 1.8061728125 2.47932583713789 5.48703652447475e-21 1.52104120710507e-20

ANGPT1 2.32093375706215 7.74232223958333 1.7380610080322 1.87958196862667e-30 7.97781938064664e-30

PNMA6F 2.64170626177024 0.70601578125 -1.90369772159078 1.29296441914929e-23 4.02051394215402e-23

EEF1A1P16 1.02549693973635 2.37650651041667 1.21251916732696 2.14612312508839e-16 4.85075477221571e-16

KLHDC8A 28.5612256120527 129.020647447917 2.17547216730152 2.46820624950434e-52 4.05802786998525e-51

JAK3 2.57329524482109 6.37290739583333 1.30833470428003 9.66223644225214e-31 4.15612506693682e-30

ATP6V1G2 144.070483286252 54.3082708854167 -1.40753095559163 2.26275648384891e-69 2.41815843776842e-67

GDA 16.3016065442561 6.11342588541667 -1.41496117135493 2.30322794808829e-08 3.66087047565619e-08

GSX2 0.422224011299435 7.88504541666667 4.22303853452898 4.81027488194666e-59 1.40640033694651e-57

SEPTIN14 0.0366264124293785 17.7943525 8.92431932629574 1.05099080991176e-42 8.68199649502851e-42

BATF3 3.54505946327684 12.0543573958333 1.76567301263252 1.06809873598032e-46 1.14938055529827e-45

ERC2 7.59843625235405 3.74928546875 -1.01908686856584 3.3103174846153e-32 1.53389866998309e-31

MMP25 1.76863305084746 3.8055390625 1.1054660746666 9.48993799095923e-23 2.85267661325794e-22

CNGA3 6.95094967043315 30.0870836979167 2.11386227089629 7.30293815791565e-30 3.01171290854827e-29

CD300LF 2.07301144067797 6.45198192708333 1.63801431879193 9.50790947767131e-54 1.733329183699e-52

RPS2P32 1.23121643126177 8.14213848958333 2.72532337001007 2.53384276464704e-66 1.71959146746758e-64

PRAM1 5.72689693973635 12.0808456770833 1.07689590267213 1.73960375225682e-36 1.00459415444769e-35

PHF24 25.0328285781544 9.10681041666667 -1.45880356133041 4.86401548345531e-12 9.12033557677236e-12

CHCHD2P6 3.74162203389831 23.2824394270833 2.63750649028661 2.39866934295446e-53 4.3011819124627e-52

LINC01614 0.143381355932203 2.23061989583333 3.95951534926378 9.05346099519408e-37 5.32076824290878e-36

VEGFA 8.32420216572505 72.3419219791667 3.11944801938184 2.60499155173377e-49 3.34298260210417e-48

CXCL10 3.62209449152542 55.65139375 3.94152173104945 1.45543186482736e-58 4.11303275478188e-57

APOL1 8.98476892655367 34.63988359375 1.94688077635438 2.57897621936018e-44 2.35656328250627e-43

COL15A1 1.21884557438795 5.34449671875 2.13253874786419 5.21571229063361e-35 2.77024193214316e-34

SGO2 2.67647457627119 7.01107619791667 1.38930196584039 4.13474040146915e-44 3.72326865691617e-43

NELL1 6.34286219397363 2.86442786458333 -1.14688699171928 2.29517178836622e-12 4.37284540938468e-12

SYN2 58.5975620998117 21.9932159895833 -1.413782064062 1.97513958472177e-19 5.09329088585513e-19

SYT7 26.7140924199623 12.8007394791667 -1.06137384919558 5.28785746199594e-08 8.27604658747702e-08

PPP4R4 9.37675564971751 3.07271114583333 -1.60957668679587 5.70848545578942e-39 3.74820928850298e-38

SPHKAP 22.6603812617702 3.37359296875 -2.74781430978393 6.06922949718422e-77 2.96992953947738e-74

AL512785.1 0.530867796610169 6.93903864583333 3.7083112688025 2.04358946399526e-64 1.06566517615572e-62

AC084368.1 0.465646139359699 2.61001744791667 2.48675352882064 1.08981483930264e-26 3.86664890990891e-26

AC002075.2 1.434934086629 3.62800708333333 1.33819280403758 1.09914713687808e-21 3.14295571432383e-21

VNN2 0.898536629001883 4.13215375 2.20124471352542 1.06079315731874e-51 1.65481952687433e-50

RAB11FIP4 21.566025094162 10.2063719791667 -1.07929016514116 2.44795598988934e-38 1.55623048314504e-37

RN7SL138P 4.05358154425612 8.65894005208333 1.09499326880973 5.23002850877273e-33 2.51753507948819e-32

ENTPD2 11.7810997645951 34.14587375 1.53523703022782 4.03745334614123e-07 6.06384338676166e-07

GZMA 1.3336209039548 6.44635432291667 2.27313486483655 9.06895339817904e-47 9.81019129954271e-46

MMP19 0.906639736346516 7.6723778125 3.08107246665445 4.32726986460825e-49 5.47012801715774e-48

MDGA2 6.73129910546139 2.42113755208333 -1.47519991879556 1.72932361843561e-56 4.01457836264796e-55

HOXC4 1.59238757062147 8.15800421875 2.35702473755963 9.58085947226625e-67 6.85215699564581e-65

CDH19 1.26529962335217 2.94950635416667 1.22099446117146 7.47699873544009e-11 1.33124082234305e-10

IGLV5-45 0.161454425612053 2.10008583333333 3.70124940179924 1.38481573114922e-16 3.15533004787645e-16

ARNTL2 2.47145630885122 7.03953958333333 1.51011967107902 1.66938603421337e-45 1.65294107061755e-44

TLX1NB 2.46615776836158 0.704858229166667 -1.8068600796936 1.95220468254618e-20 5.28479343018579e-20

TGM5 0.3164315913371 2.49876072916667 2.98124721627269 8.84078097266446e-46 8.92962097700682e-45

C5AR1 9.30410602636535 32.95727828125 1.82465766254303 1.87030628293804e-42 1.52336160014161e-41

AC133637.1 1.89485423728814 0.580359114583333 -1.7070690801231 2.75450685474525e-59 8.28803478381682e-58

AC125807.1 0.862129802259887 3.59121067708333 2.05849328773066 1.48407402477251e-28 5.74684641621092e-28

TLR2 7.6308936440678 19.81272953125 1.37650372481446 9.94059319472596e-44 8.70269917400797e-43

CAPS 11.8839251883239 46.3225694270833 1.96270385090508 3.68651405693787e-42 2.94841844682837e-41

PBK 9.95468893596987 38.71843109375 1.95957235403284 4.83016590378725e-34 2.44999822643e-33

ZFP36 127.477143220339 281.213736197917 1.14142847257274 1.01848702529116e-29 4.17337290332506e-29

AC008760.2 1.37628196798493 5.81475630208333 2.0789426544566 3.51795943894771e-57 8.61876887578823e-56

AL022337.1 1.52422970809793 0.59079125 -1.367359975554 3.84249290448748e-29 1.5244539270097e-28

FKBP5 18.7122798964218 47.5275801041667 1.34477960115578 1.55474820077834e-31 6.95466509345038e-31

ARSI 2.36000207156309 8.22721203125 1.80161549908673 1.58871552048452e-22 4.73583922786304e-22

KCNQ5 11.9656021186441 4.82829052083333 -1.30930860471579 3.77331111718255e-37 2.26557055938035e-36

AC012558.1 5.74550174199623 2.41454875 -1.25067929377578 2.55545039839449e-28 9.80169145176269e-28

RPL7AP50 3.25904510357815 7.39426489583333 1.18195740987457 4.00112526676792e-21 1.11662801043898e-20

CARD16 2.32362490583804 14.3086536458333 2.62243882620256 2.56690705873492e-70 3.22511059170107e-68

LRRTM4 16.2376809322034 6.13917375 -1.40322919485841 1.37896615493354e-52 2.31216191622986e-51

PLSCR1 12.0914857815443 46.11405640625 1.93121504604525 8.32918728276729e-72 1.42092878461521e-69

IDO1 0.136173634651601 2.05002729166667 3.91212380906856 2.84726548114332e-48 3.42463787980983e-47

SLC8A3 15.9354562617702 7.17018114583333 -1.15215885432999 1.06582191217475e-39 7.30518188606321e-39

GRAMD1B 22.0664418079096 10.4044564583333 -1.0846524163421 5.51081795290902e-50 7.47437343795355e-49

ANXA2R 3.02403347457627 9.02332890625 1.57718566431686 1.45086185946645e-49 1.89590838206456e-48

SNORA73B 3.81871440677966 20.1300104166667 2.39818898567986 1.21351349564742e-23 3.7772486527559e-23

MIR23B 0.673631685499058 2.21488 1.71719663219798 3.83905823895212e-13 7.57023202049996e-13

RCN3 15.4362708568738 34.334999375 1.15335567348464 1.75315512657579e-47 1.99387887331357e-46

POLR2J4 2.66785489642185 6.48597572916667 1.28164342412886 1.47708269756988e-62 6.31410408306021e-61

DEDD2 13.9487484463277 28.6374872395833 1.03776922886828 9.97486475764521e-55 1.96070412440183e-53

ATP23 3.05369802259887 10.3667639583333 1.76333631182419 2.02437372133703e-27 7.43059700665093e-27

AL049749.1 12.2425372410546 2.44225635416667 -2.32561603716107 9.32046839267687e-58 2.41721213056941e-56

GSC 3.36072080979284 7.65252036458333 1.18716428254806 1.14933800827302e-20 3.14339465566066e-20

IFI16 37.5366240583804 76.0142063020833 1.01797016521993 7.62643636440861e-38 4.72711947320594e-37

AL355974.1 1.59292048022599 4.37106145833333 1.45630941470521 1.36627297776926e-35 7.47890668873105e-35

H2AC6 43.3859447269303 87.0231634895833 1.00417171894936 4.51357855343175e-30 1.88310507518652e-29

RPL5P4 1.07042057438795 5.94477765625 2.4734451008091 1.02575359809706e-30 4.40505500152767e-30

RPS15AP11 1.00612222222222 5.04381796875 2.32571063755752 3.72174181407963e-32 1.72110890407388e-31

KCNS2 2.61364656308851 0.73042828125 -1.83924953144261 2.53536662022085e-09 4.21767241930638e-09

AL391001.1 0.745338323917138 1.57459598958333 1.07901436148236 1.78797875314965e-31 7.96919101505698e-31

HSPA8P1 1.00128281544256 2.38274557291667 1.25077538485181 3.53602579029322e-27 1.28598473636813e-26

FAM126A 4.92145946327684 12.2392424479167 1.31435614804026 5.54962496326907e-55 1.11321765039901e-53

DSN1 11.9033024011299 26.8475204166667 1.17342696574631 1.6778663400086e-50 2.37262305513726e-49

FOLR1 4.14670004708098 10.335891875 1.3176272847103 3.17374378292177e-26 1.10702993140931e-25

AMZ1 10.2021527306968 3.81379203125 -1.41957552404866 2.35368230365327e-45 2.29746574469462e-44

F2RL1 5.32318700564972 12.54963875 1.23728368109319 3.79545121107644e-20 1.01011042321406e-19

CLEC4GP1 2.56382368173258 0.744431927083333 -1.78408521299365 8.58437254047616e-23 2.58797677351093e-22

RPS7P1 14.8397725047081 47.9607822395833 1.69238621381712 1.11504508033615e-32 5.2934039491577e-32

GLRX 7.61079952919021 21.2420644270833 1.48080405772812 4.09145279181458e-64 2.03969342262177e-62

KSR2 4.41423102636535 1.07735302083333 -2.03467107616442 1.83338730537398e-58 5.13431279268511e-57

PRR36 43.3634021186441 20.6540493229167 -1.07005329271819 1.18891699704574e-44 1.11206798591879e-43

SHCBP1 1.83657645951036 7.02664776041667 1.93581762041981 7.40153116023013e-56 1.62110096495264e-54

CD58 5.7606329566855 30.33202578125 2.3965426108797 2.89050702918693e-85 1.07497956415462e-81

RPS29P3 0.830514406779661 6.11522447916667 2.88032835884853 4.1729565416952e-21 1.16370916156002e-20

MIR6859-1 3.141665913371 6.71700473958333 1.09628827515483 4.5074345589699e-12 8.46794762821229e-12

NMI 6.15863691148776 18.14328703125 1.55875287327714 3.88881838779726e-70 4.7889124451053e-68

GPX1 507.691962052731 1015.43075140625 1.00006653242706 3.10660714568957e-52 5.06287115460978e-51

POM121L9P 0.726731779661017 2.0785878125 1.51610879644334 4.96231819790491e-33 2.39176534188807e-32

AC090543.2 1.19344114877589 2.64378182291667 1.1474756982131 4.90597401622081e-21 1.3628112762418e-20

CMTM3 46.2648849811676 94.9539953645833 1.03731110151467 1.65132979484759e-69 1.79570044065444e-67

BRINP1 58.7143919962335 22.8470397916667 -1.36170692562368 2.69627072541588e-44 2.46011551222317e-43

HJURP 2.94796275894539 12.7707266145833 2.11505040786416 1.50982405337021e-40 1.09114567712472e-39

AC122697.1 2.029243173258 0.375186302083333 -2.43526269838255 7.1175329327435e-48 8.35019084443946e-47

AC018475.1 1.42844891713748 4.47881026041667 1.64866610451447 2.84603903804952e-20 7.62811135082676e-20

EGFLAM 1.31442829566855 3.90437994791667 1.57065801048211 1.56130356788869e-30 6.65728957690672e-30

AL117332.1 1.94910329566855 4.5217834375 1.21408134809755 2.16583099680838e-58 5.99309931334105e-57

AC020916.1 0.834254378531073 2.00398651041667 1.26431353881214 3.06336720795809e-38 1.93950674947159e-37

FRZB 13.3788650659134 45.5185571875 1.76649909210791 4.02044543094224e-37 2.40851104343979e-36

PIRT 7.1955 21.4948502083333 1.57882421124029 4.38342538368904e-34 2.2294801698495e-33

SELENON 85.5233350282486 177.28036265625 1.05164271983995 1.47419588099336e-52 2.46074258591306e-51

GRIN2A 6.95145197740113 3.19765083333333 -1.1203019380567 9.08156957533122e-12 1.6814874664272e-11

PCDHGB4 3.31543380414313 0.988164895833333 -1.74637393983234 4.8253494293168e-05 6.5370717917528e-05

GOLGA2P5 0.810225423728814 1.66063979166667 1.03534391258124 5.71233331226212e-15 1.2185481007401e-14

MDM4 13.481915913371 27.1031128125 1.00743302338532 2.56885804182897e-10 4.47684484028867e-10

AC007326.1 2.5067684086629 0.942565520833333 -1.41116390344802 1.98823744859493e-41 1.51832753004611e-40

PAXIP1-AS2 2.12084274952919 5.76895880208333 1.44367330489281 9.6646827959408e-47 1.04364405323572e-45

CAMSAP3 15.7799203389831 5.90192661458333 -1.41883203496795 5.72468986630516e-55 1.14586230424052e-53

PDCD1LG2 1.79089571563089 8.5721253125 2.25897160912986 4.38409272011048e-61 1.57683180136275e-59

HASPIN 0.82067302259887 2.71524546875 1.72620319466731 2.48501395974882e-36 1.41788384723932e-35

AP006333.2 6.31885988700565 2.78842020833333 -1.18021628784292 1.01963384888933e-44 9.59033455745933e-44

CTSB 179.632811393597 404.644678229167 1.17160472754954 9.23549407001767e-58 2.39851972391032e-56

PLPPR1 111.183834651601 53.49703 -1.05541634098196 3.47322731476746e-35 1.8606932272573e-34

HAGLROS 0.450083945386064 2.17884484375 2.27529745589002 5.33523261478468e-27 1.92302094343712e-26

CNIH3 7.10058451035782 15.4138106770833 1.1182138807274 8.93996177704123e-15 1.89230038980173e-14

AL354710.1 0.458270621468927 1.94620807291667 2.08639425333387 1.89471651762937e-22 5.62545962722627e-22

SYDE1 13.5790061676083 34.9194178645833 1.36265161405743 7.51632222490471e-77 3.32776218505007e-74

UQCRFS1P1 0.500814689265537 2.24921276041667 2.16707135351218 4.95787640901439e-32 2.2797159205149e-31

NEFH 25.9471768832392 9.10464947916667 -1.51090219742791 1.68016433152565e-11 3.07657860607774e-11

HTR2A 8.37833145009416 2.47738192708333 -1.75784665923381 1.02099632840344e-41 7.94369319107195e-41

SLC30A7 5.5956422787194 11.49642625 1.03880981716214 4.08777383686373e-66 2.72444998195273e-64

SETD9 4.20679194915254 8.80198203125 1.06510795652234 1.0768575164351e-50 1.55346512941122e-49

SMIM10L2B 17.7495358286252 7.18095166666667 -1.30553433971635 5.04043342851605e-29 1.9916459754198e-28

HSPB7 1.75536916195857 6.74908817708333 1.94291813508236 9.88795951306012e-43 8.19004931605136e-42

AL606500.1 18.1735346986817 6.38653734375 -1.50873319969405 1.83039083164503e-29 7.38951748033854e-29

LINC01445 0.25598554613936 2.86051822916667 3.48214228032547 7.31011896429596e-05 9.80464960625241e-05

SCN2B 28.4024440207156 9.72943635416667 -1.54558694418054 9.54471254403319e-42 7.43232536667911e-41

IGHG3 1.38015 13.0349775 3.23949111433118 2.2396967234397e-24 7.18302183034861e-24

FCGR2B 0.431586440677966 4.31613885416667 3.32201983347281 4.74286203520404e-52 7.61602068606383e-51

ESPL1 1.55622288135593 4.99946817708333 1.68372593798412 5.26729167535776e-35 2.79683862659273e-34

ZNF474 0.465805696798493 2.28418755208333 2.29388092472571 6.56381394479569e-43 5.50289090637853e-42

CAV2 4.03016614877589 16.07116890625 1.99556364249213 3.43060622571076e-51 5.17373258451675e-50

NALCN 11.0358713276836 4.80026380208333 -1.20101494334583 2.91694942414032e-58 7.96485676092353e-57

CCL5 4.10091064030132 16.6784768229167 2.02397132686597 9.65979360339549e-50 1.28578283504037e-48

BEND3P3 1.63478173258004 0.809394635416667 -1.01418283544214 2.53960099847023e-41 1.92829238736439e-40

STEAP3 6.12575960451977 56.3834248958333 3.20231045719161 1.95148452456403e-80 1.7279930825842e-77

CFAP157 1.16064628060264 2.47546145833333 1.09276912504522 2.32434172680427e-14 4.82868793593284e-14

AOPEP 3.78874905838041 7.69754317708333 1.02267646620637 4.34405618161283e-56 9.69720584598926e-55

BSN 23.2504307909605 7.91537859375 -1.55452718570863 3.03143339416938e-45 2.93744158231264e-44

PDPN 9.55137966101695 206.250491822917 4.43254460927831 3.09114363507023e-80 2.39499232892212e-77

LINC02821 0.948060734463277 3.2851328125 1.79290030869039 4.90718108655782e-26 1.69797231679462e-25

SERPINB8 3.41623290960452 10.0955565104167 1.5632421979001 4.42493369749014e-68 3.79178074215803e-66

DKK1 1.30644199623352 12.34179359375 3.23983709399546 1.08089919001488e-09 1.82987258178502e-09

AC025263.1 2.07130579096045 0.9119390625 -1.18353122809608 3.80438851720513e-36 2.15481585371396e-35

GFRA1 28.1437322033898 9.52608723958333 -1.562857994256 1.82294833396628e-52 3.01581176780276e-51

BANF1P3 3.04070993408663 9.94400322916667 1.70941856660628 3.62195613702345e-35 1.93757981495832e-34

SCIMP 1.79110739171375 4.70234572916667 1.39252877230229 6.7020106491617e-47 7.31796260483457e-46

AL033519.3 1.38754745762712 7.59287666666667 2.45210945811902 1.51666761319116e-23 4.70275708975982e-23

PDLIM7 13.3574670433145 32.1905658333333 1.26899147855025 4.5467617812578e-52 7.31375738083813e-51

PDE1A 11.2741052730697 4.93708213541667 -1.19128239227687 6.58635160012218e-26 2.26383009250041e-25

UBE2SP2 0.391907956685499 3.12485427083333 2.99520214195603 5.27898496888714e-38 3.29737069185275e-37

TRPM8 0.129397269303202 3.61475239583333 4.80401775121536 6.976208877489e-74 1.75300816320146e-71

EHD4 6.45389882297552 14.5711772916667 1.17487457983225 3.00240742208255e-47 3.36323891648343e-46

PTMAP8 0.406926600753296 1.9160034375 2.23525965196939 1.1991030345196e-38 7.76098883637033e-38

KCNAB2 21.1095028248588 10.0386898958333 -1.07232159850243 3.80606556875253e-10 6.58238367289372e-10

HSPB1P2 0.46235856873823 3.0899275 2.74048895709948 6.41300650347907e-58 1.69148731818714e-56

SFRP2 262.617284651601 57.9929822395833 -2.17901163839826 1.49619521081709e-47 1.70790361848642e-46

TMEM159 5.57893149717514 16.0015840104167 1.52015398381014 8.68271534941707e-56 1.89057484686663e-54

LGR5 8.16471577212806 3.71746739583333 -1.13508257482229 8.04792874761712e-38 4.97841766673122e-37

EEF1A1P10 1.10283799435028 2.77170697916667 1.32955386968399 2.01477556771754e-19 5.19190017762024e-19

YWHAEP5 1.92860743879473 4.81389203125 1.3196442667684 1.28112816738854e-22 3.83061236092457e-22

CYB5R1 27.5487052259887 55.93869953125 1.02186219976299 3.50378403327337e-53 6.18148615737365e-52

ENPP1 0.588009604519774 2.01695005208333 1.77826373209869 3.30250897632314e-57 8.12303629824455e-56

RYR2 3.91720856873823 1.04102328125 -1.91182361235425 5.74862184306155e-19 1.45317595393868e-18

AL592078.2 4.04514764595104 1.072046875 -1.91582437261447 5.74306773571405e-48 6.78477411344363e-47

SNORD17 3.24944882297552 14.6934036458333 2.17690169532409 2.65251101209281e-11 4.80782164634621e-11

SELENOV 0.818620998116761 1.64179307291667 1.0040047269023 0.00020857741310222 0.000272999014333482

CIBAR1P1 1.34168629943503 2.7095028125 1.01398075118113 3.32432614642404e-21 9.31663069973701e-21

AL590666.2 37.3176263182674 14.4707447916667 -1.36671804530881 1.35109770947117e-38 8.72349371792236e-38

EEF1A1P14 0.876725423728814 2.7156196875 1.63108445993303 1.06088463499248e-20 2.90489615486455e-20

IGKC 60.3775467514124 344.6425578125 2.51301681834135 4.36737454755558e-25 1.44324381929618e-24

HRH1 8.99165955743879 27.70070171875 1.62326320499617 1.83664870379411e-44 1.69743949538029e-43

LDHA 68.3588495291902 259.558016666667 1.92485702530659 6.28909243204881e-56 1.38561224850649e-54

PRLHR 18.3621738700565 4.40153130208333 -2.06065943449275 1.54849787324779e-67 1.23580763746964e-65

L1CAM 58.4751688323917 17.2998056770833 -1.75706828936065 5.76239671256412e-29 2.27160837121327e-28

PLVAP 19.7825487758945 55.0121013020833 1.47552069644495 4.31089679900128e-29 1.70737222529135e-28

SCYGR1 1.52218615819209 0.649601979166667 -1.22851687317443 8.30802602922996e-28 3.10528128670414e-27

AC138305.3 0.315736911487759 1.8439865625 2.54603330750383 4.04298941522605e-32 1.86781088636344e-31

CD101 1.01790174199623 4.50907625 2.14723360178613 7.13241139735906e-67 5.16059104801135e-65

APOBEC3F 0.933439830508475 3.53435692708333 1.92081880740671 1.31503525981793e-69 1.46425632672541e-67

TAFA2 7.34073625235405 1.82779036458333 -2.00582415567712 2.88923942369903e-50 3.99742612229788e-49

PCDH11X 3.05888935969868 1.233501875 -1.31024801680494 3.77764386303008e-47 4.20630464868529e-46

FAM81B 0.581324529190207 3.81661447916667 2.71487777556176 6.36480563933744e-17 1.46931794988802e-16

PKMYT1 4.08187961393597 8.437138125 1.04752008625658 5.50476502402675e-22 1.5944097448875e-21

RTN4RL1 7.6572620527307 3.28060401041667 -1.2228671691972 5.75742964220799e-15 1.22788627361919e-14

ZMYND10 3.11428145009416 16.8809225520833 2.43842251275615 1.14258699412989e-31 5.14942320552144e-31

RPL31P49 0.93300790960452 5.48969645833333 2.55676516379525 5.26182780488575e-37 3.12899545992486e-36

BICDL1 4.84269919962335 2.39286890625 -1.01707003314102 6.10194578068787e-15 1.29987033786105e-14

PPM1J 0.930580508474576 1.89058864583333 1.02263262145406 4.51044447956114e-35 2.40388979929606e-34

CDC42EP5 2.35611172316384 5.95332015625 1.33728653021482 1.70923944240301e-36 9.87365872366697e-36

RPS7P10 2.63689044256121 11.12399765625 2.07676580931791 1.58655967786705e-30 6.76187880126926e-30

SLC34A2 0.403149952919021 2.35464109375 2.54611871501459 6.3804195067037e-44 5.67674166158638e-43

ERVK9-11 0.618868314500942 1.70586859375 1.46280215317242 6.41833644804071e-48 7.55373204122259e-47

ASB9 0.843890442561205 1.8124971875 1.10285113705236 5.76800263277225e-39 3.78461570064926e-38

KRT18 1.02822288135593 2.21662072916667 1.1082089193376 2.23167182698529e-35 1.20668617687675e-34

AC124854.1 5.80599048964219 2.17530583333333 -1.41632396037923 1.53347750777057e-40 1.10737919444636e-39

TRAM1 65.1087206685499 134.696199427083 1.04878644828885 1.16905324929723e-64 6.37493993275132e-63

CXCR4 37.4420937853107 97.2241861458333 1.37665413946427 5.50037070840477e-47 6.06279747023039e-46

SEMA6B 70.1834970338983 31.1237140625 -1.17311760408597 2.76252364887183e-58 7.56540902073221e-57

RNU6-1160P 0.572647598870056 1.56064463541667 1.44642256943129 9.19058632199318e-22 2.63488980353782e-21

RPS4XP1 0.38537038606403 1.70755203125 2.14761192336194 2.98504800753414e-30 1.25439475028469e-29

TFCP2L1 1.64076120527307 8.01799010416667 2.28887535115836 1.34353808471817e-25 4.56228829169729e-25

FTH1P12 1.51434670433145 6.77628770833333 2.16179958699169 4.90861731121368e-39 3.23787651301945e-38

CRYZ 14.848820527307 33.99150234375 1.19482578767648 4.28878267081352e-59 1.25987225535193e-57

CLEC5A 0.624206920903955 8.92805609375 3.83824983308817 1.75102627987921e-68 1.5579107021222e-66

AL158212.5 2.01663606403013 0.995060416666667 -1.01909471986413 1.06834585457871e-35 5.889933077439e-35

JHY 0.577791619585687 3.33052989583333 2.52713054802625 1.3802983184119e-43 1.1982561732432e-42

PLCXD2 4.78826963276836 2.14345244791667 -1.15956798272923 2.15431670082975e-40 1.53485015045717e-39

TBX5 1.51095583804143 3.88625692708333 1.36291978883429 0.0121775364882284 0.0142505532409444

RPL13AP6 1.03863766478343 3.30172307291667 1.66852667257508 1.19746452450384e-24 3.88615803884623e-24

SKIDA1 0.708637711864407 1.75201463541667 1.3058946774046 1.01763367228591e-33 5.07316303918405e-33

SPHK1 4.65566845574388 12.6709633333333 1.44446598386499 1.88073309528353e-33 9.25926182335114e-33

KCNH1 2.13482791902072 0.87452203125 -1.28755314938219 1.51862462017462e-36 8.80811753342079e-36

IL2RG 0.522784981167608 1.81567921875 1.79621973970959 5.36303908872636e-55 1.07695153191001e-53

SLC66A3 9.76442288135593 24.7169721875 1.33989534251938 2.60338647201576e-70 3.24899137229081e-68

AC025171.5 0.613114030131827 4.1788628125 2.76888307343534 3.14845773568221e-50 4.34677614899869e-49

TMEM158 28.2305143596987 98.3915064583333 1.80127836691486 4.46858307360196e-44 4.01416919099654e-43

DOCK3 27.9313085216573 13.7470722395833 -1.02275876664248 2.06889376788077e-39 1.39945724313361e-38

MIR1915HG 2.61530607344633 5.57678052083333 1.09245269628952 2.35191783169692e-37 1.42781299642195e-36

FHL3 26.1964628531073 60.7894213020833 1.2144482581107 1.03256252925065e-68 9.63370006560979e-67

LINC02525 0.586221563088512 2.30218572916667 1.9734862867806 3.82444311786434e-46 3.9530583533456e-45

GPR82 0.680854519774011 3.95593557291667 2.53860045898996 7.80402887060456e-60 2.49340063314247e-58

HOXC-AS1 0.209141760828625 4.23718802083333 4.34055407919738 4.66961889493259e-71 6.67935102702089e-69

SCNN1B 0.161662617702448 3.86785765625 4.58047668341658 3.80177534091087e-49 4.83212662093217e-48

DYNLT3 17.7343754708098 37.07963484375 1.06407851093665 3.96810113432294e-34 2.02377511225274e-33

IFITM2 43.9559573917137 111.838032447917 1.34728027137047 1.47053750054289e-47 1.68067884588783e-46

LINC00475 0.0824875706214689 3.649221875 5.46726831233711 2.51490645213269e-69 2.65708440212542e-67

SYCE1L 1.96577297551789 4.26274057291667 1.11668454028765 6.93521614804837e-31 3.00466785351723e-30

CHI3L1 29.6415247175141 3036.05206192708 6.67843306021926 4.09817749071796e-78 2.17730315542573e-75

VIP 3.52420654425612 1.18825130208333 -1.56845849666443 1.37673871042106e-13 2.77091355051248e-13

CAMK1G 14.3469510828625 5.32435520833333 -1.43006545032798 3.96666325125466e-07 5.9599307657628e-07

UST-AS2 1.28062335216573 6.23484151041667 2.28350666437476 1.61044148831806e-55 3.39911004259641e-54

HIGD1AP1 0.713757580037665 3.54929296875 2.31402559651767 1.00571252936295e-25 3.43330723031101e-25

SOCS2 4.35733921845574 28.5657822395833 2.71276870051754 4.03226518691207e-57 9.78850798311096e-56

AC006026.3 1.43582438794727 4.16980546875 1.53810077255105 5.06478562165609e-27 1.82802190672933e-26

SHISAL2B 4.21180357815443 0.9915359375 -2.08670118839851 2.56874147046941e-12 4.88053005449869e-12

UNC79 17.6306429849341 4.82648026041667 -1.86904170670101 2.47748570768817e-62 1.03992882019101e-60

IQCH 0.698518738229755 1.400065625 1.00312372939482 2.04842792741454e-40 1.46220795816788e-39

DDN 44.7168043314501 10.5650997395833 -2.08151070317368 5.74426174051337e-26 1.97988039045127e-25

AC106820.1 0.550111534839925 2.84677328125 2.37153154143925 3.23220626212268e-25 1.07499330073638e-24

GPX1P2 0.624551271186441 1.50091276041667 1.26494820694149 1.41783329161333e-41 1.09125041628932e-40

IGF2 0.675011016949153 2.88675979166667 2.0964681114929 8.14027505345177e-07 1.20506659198261e-06

KCNA4 1.82233305084746 0.738627083333333 -1.3028685830706 3.17694481959712e-22 9.30612616893643e-22

AC010273.3 1.27459552730697 4.19553979166667 1.71881693422206 5.39743296368934e-47 5.95639560592304e-46

TTK 2.14412052730697 7.77526828125 1.85850644928424 4.5438013612952e-36 2.55958758901194e-35

PSPHP1 40.9683366760829 174.49692203125 2.090620363448 2.42921335958739e-12 4.62156971777445e-12

IGHV4-39 0.681346186440678 9.90214338541667 3.86128092880359 1.36008334911064e-18 3.37795510574494e-18

PITPNM3 10.211472645951 5.09413921875 -1.0032806462973 1.39528560824904e-05 1.94565698428128e-05

MTND5P11 2.12122015065913 4.813204375 1.18210332471062 2.75098468589284e-11 4.98242526874232e-11

AL591846.1 2.79213672316384 41.8954608854167 3.90735245213843 1.73335011419858e-22 5.16036589393573e-22

RUNDC3A-AS1 2.27731398305085 0.895078177083333 -1.34724761622671 9.01482380070195e-56 1.96059238098307e-54

ADARB2 12.6124553201507 5.56450369791667 -1.18052423515715 3.97301049554261e-38 2.49926836509539e-37

H2AC8 4.47812664783427 10.4002494791667 1.21565090050836 2.82477031644239e-26 9.86971139313157e-26

KCTD14 0.581031544256121 1.62395901041667 1.48282682368221 1.08284255951188e-27 4.02628622158037e-27

AC243960.2 0.293374858757062 2.191855 2.90133521410396 9.79109282588821e-40 6.7232411778948e-39

MTRNR2L10 0.79264274952919 2.03208145833333 1.35821555210997 0.00018527521928603 0.000243201517903694

AC027130.1 6.05699774011299 2.01113005208333 -1.59059649488951 6.18302271685333e-54 1.13850222861808e-52

PLK5 2.45013780602637 1.16660072916667 -1.07055201372981 2.97530326869914e-39 1.99228535403171e-38

SBF2-AS1 0.619528813559322 2.10664208333333 1.76570193524119 2.07580152504344e-48 2.51797155972569e-47

FAM222A 71.3164903954802 31.51399765625 -1.17824293059214 1.25049981119147e-48 1.5409571894702e-47

TOP1P1 0.836404001883239 2.04401166666667 1.28913156099491 1.18213285817171e-53 2.1445619997759e-52

DPEP1 1.25331732580038 9.9106703125 2.98323090300925 2.95303748746216e-51 4.48992085685682e-50

CNTN3 12.8056979755179 4.05428875 -1.65926514169613 7.77725451486312e-47 8.44731587055372e-46

RPS20P10 0.48272368173258 2.75251984375 2.51148345446893 4.80200150204176e-28 1.8152717611398e-27

VSIG4 82.2159615819209 225.039433645833 1.45268741285566 3.3856641068275e-29 1.34810329906761e-28

AC015911.1 0.622453531073446 2.26892098958333 1.86596832834739 2.32468854776571e-21 6.54864165212898e-21

MUC1 3.31195094161959 8.62547177083333 1.38092206422242 1.13882288131038e-52 1.92512831617877e-51

TMEM63C 15.5867618173258 7.06828921875 -1.14088825812711 1.01062150032491e-42 8.35965604917336e-42

HILPDA 15.8382002824859 68.6939929166667 2.11677553599638 1.450827530722e-54 2.82493590929587e-53

AC147067.1 0.978590442561205 2.07203864583333 1.08227381332877 2.93958407186995e-36 1.67263053293824e-35

HAS2 6.1793024952919 13.8984014583333 1.16940305464491 1.27648372360999e-29 5.20074821221029e-29

FBLIM1 4.64928234463277 20.267845625 2.12411279882574 2.57398898462023e-41 1.95360510893931e-40

C1orf162 13.4093744350282 29.8070639583333 1.15241233945683 1.54969853701626e-43 1.3428072831229e-42

SASH3 13.4053183145009 32.518921875 1.2784739503374 1.99109084687521e-44 1.83561399591693e-43

H2BC18 0.694814689265537 1.43376171875 1.0451051181652 3.54897680255425e-22 1.03681419706986e-21

AL136114.1 6.72737024482109 0.88911015625 -2.91960858207591 3.07023622736775e-56 6.97082327813227e-55

EIF1AXP1 2.08333446327684 6.31675859375 1.60028996656336 4.24998545922825e-31 1.86256138615011e-30

RPL34P27 0.719500612052731 3.58375630208333 2.31640471714014 2.25536353221958e-23 6.95266659178102e-23

IL11 0.654053060263654 2.01079984375 1.62028989736055 8.4644076785072e-12 1.56971836822421e-11

MYO1B 6.69748121468927 15.408953125 1.20207831468095 2.85590948663256e-25 9.52396644618588e-25

GRM5 3.50108069679849 1.216210625 -1.52540721669237 1.46050680546854e-25 4.9477362083599e-25

VSX1 0.687819350282486 1.48525640625 1.11061040262793 1.02353462868746e-10 1.81070060940103e-10

BST1 1.78308352165725 5.35573494791667 1.586710282304 2.55472659347556e-56 5.8432482628097e-55

MIR124-2HG 7.49693441619586 2.80855432291667 -1.41647307366367 1.09978511808306e-39 7.51967513101495e-39

NEUROD4 1.76789948210923 0.697858697916667 -1.34102939445803 6.64632138584752e-28 2.49673426605727e-27

IFIH1 7.54163097928437 17.3009301041667 1.19790113516197 1.47205329240375e-42 1.20638303094966e-41

MYOD1 2.27288262711864 0.232292760416667 -3.29050708727696 6.43739829190337e-51 9.47770556119898e-50

TLR8 0.891213653483993 3.30123114583333 1.88916091662441 4.69602229348385e-39 3.10535329115691e-38

STAB1 21.7451845574388 46.5930628645833 1.09941921786848 8.35910155049132e-22 2.39835663217692e-21

GPR27 33.2946920433145 13.53451328125 -1.2986491888299 2.85765002315637e-41 2.16095982840963e-40

H2BC11 1.39819129001883 5.1509259375 1.8812700435406 1.20100235327097e-60 4.16661449178354e-59

TTYH3 80.9612222693032 200.667920729167 1.30950702669488 8.44575170922263e-44 7.43954301435314e-43

PSME2P2 1.81981760828625 5.85618661458333 1.68616766443749 3.80431212654095e-52 6.15140730374165e-51

TNFRSF18 0.898980084745763 2.33881734375 1.37941813398361 2.4537400492706e-22 7.23524254999997e-22

RNF150 14.1129240112994 6.815618125 -1.05010051664401 6.89543869834796e-52 1.09496740047635e-50

COLGALT1 32.8481835216572 75.5861152083333 1.20230765004581 2.59494479968395e-67 2.01895391423109e-65

AC253536.4 0.914040489642185 2.81904453125 1.62487628823933 4.705268569889e-07 7.03671136055058e-07

HGF 1.46255720338983 4.4156634375 1.5941371615413 6.6386424790223e-40 4.60102709271039e-39

AL139246.1 1.95015131826742 0.942267916666667 -1.04937684422948 1.32748060414995e-25 4.50858480989376e-25

CENPN 2.99733780602637 7.51800635416667 1.32666844707687 2.41401772664221e-45 2.35512379994291e-44

ABI3 17.6395148775895 41.6305511979167 1.23883177580996 1.83438517416019e-50 2.58021121887358e-49

ATP5MF 36.1218361111111 78.19678734375 1.11423810831193 7.12648967020059e-57 1.701117784562e-55

AC104695.3 0.586664642184557 1.50169453125 1.35598342573926 1.66440636717783e-12 3.18805484112812e-12

SAMD12 3.0744884180791 1.39725713541667 -1.13774882956948 7.68567122704025e-25 2.50948299327153e-24

RPS27P21 0.867971751412429 2.74879338541667 1.66307847465771 1.68507026024671e-26 5.93669600024395e-26

AC097713.1 0.386934557438795 3.34413385416667 3.11147110678005 6.30281678784048e-27 2.26256521563501e-26

AC133134.1 0.987602354048964 3.25927041666667 1.72254687483143 5.26171852711036e-22 1.52591478495972e-21

IGHA2 0.822015630885122 6.87412052083333 3.06393741346966 5.65585666641217e-24 1.78467087581765e-23

COL1A1 17.6484070621469 156.34342484375 3.1471086693334 9.47346078313277e-51 1.37624221298714e-49

XKR7 7.40750042372881 1.62669114583333 -2.18704644617018 1.01346547743945e-47 1.17489966041063e-46

VTRNA1-3 0.332210875706215 3.56371067708333 3.42320900596684 1.76598392280831e-13 3.53405844216751e-13

IGLV3-25 0.543002401129943 9.878561875 4.18527054629077 2.35916780889021e-15 5.10279462676672e-15

FZD5 2.57633502824859 5.90831307291667 1.19742605980581 1.85204946801988e-44 1.71082264569447e-43

CD52 2.86567424670433 9.53120234375 1.73378359727549 1.66326150191197e-34 8.62474836253572e-34

PLPPR3 14.8811006591337 6.74186317708333 -1.14226198332891 8.66173498102927e-42 6.75891573530169e-41

ACCS 2.30461450094162 6.07099729166667 1.39740808222713 5.952417712464e-46 6.0950004054663e-45

ABCB4 0.996767655367232 2.15413276041667 1.11177800725965 3.50297830997072e-36 1.98712268681835e-35

SNRPGP15 6.03366177024482 13.35908296875 1.14671524721166 2.46739274831457e-28 9.47176370416093e-28

H2AZP3 0.621129237288136 3.42318401041667 2.46237346144905 5.26125544660786e-37 3.12899545992486e-36

PNMA8B 26.6834688323917 11.7007128645833 -1.18934979955074 1.76641469338864e-44 1.63578093742837e-43

LRGUK 0.448281026365348 1.6618371875 1.89030370291779 4.94603904565483e-53 8.6279887638173e-52

RPS11P5 5.4073 23.3267133854167 2.10900274801643 1.02972605141152e-34 5.38614793980231e-34

GAPDHP1 18.9211800376648 61.0965873958333 1.69108973272874 9.13746961380076e-30 3.75328578459521e-29

COX20P1 5.69687184557439 15.6903620833333 1.46163678804651 1.23514009047663e-06 1.81174015795637e-06

DUSP10 7.50183535781544 18.7418506770833 1.32094791421064 2.6265419180577e-59 7.92866022179918e-58

CAPN5 27.9830824858757 75.13172453125 1.42486732563962 9.94405165625572e-55 1.9567157729955e-53

ACP5 3.27321638418079 8.4601946875 1.36998188477097 5.34315714088522e-31 2.32956640175289e-30

LMNB1 21.5720482580038 53.6263132291667 1.31377790615184 1.67886427543342e-26 5.91595247331522e-26

SAA2 0.0441070621468927 4.32584770833333 6.61582939624051 6.11960035495091e-43 5.14671952059304e-42

AC080100.1 6.31383940677966 2.93602119791667 -1.10465518160573 8.59169328962297e-54 1.5693765886104e-52

REXO2 10.4973800847458 22.2303503125 1.08250137510775 1.16136012107933e-83 3.08507020735288e-80

AP000936.3 1.6308397834275 5.31434947916667 1.70427804851245 7.743759302471e-24 2.42947872835243e-23

GAPDHP40 0.655475753295669 4.58384140625 2.80594281015141 6.61603286841199e-41 4.90545563773904e-40

RPS10P16 0.503597457627119 1.77703197916667 1.8191267392425 1.90670154556722e-27 7.02081489897474e-27

PMP22 29.1362332391714 63.3117676041667 1.1196592983663 9.88259155018878e-55 1.94668209614153e-53

UBE2D3P1 1.0383536252354 5.282856875 2.34702046792313 8.94927253939516e-36 4.96011096483019e-35

CAPN6 1.94150258945386 4.63896041666667 1.25662790458488 1.35481510392437e-32 6.40548864924324e-32

IGKV4-1 1.17765729755179 17.4543776041667 3.88959723757283 1.74516924103454e-22 5.19305841527241e-22

FAM20C 31.6404422316384 122.25152265625 1.95001076491412 4.69283779216385e-72 8.47216686847445e-70

LAMC1 18.5309838512241 63.7910228645833 1.78341393247144 1.47918421787962e-57 3.76786719609199e-56

HP 0.16123950094162 8.4913090625 5.71870985589568 3.45593261468305e-52 5.60759746684392e-51

EIF2S2P4 0.818035357815443 3.34074338541667 2.02993406129294 5.92759743189042e-40 4.11897138437976e-39

NPM2 13.7489356403013 4.9159603125 -1.48377476449499 6.17857413735092e-23 1.8717918879772e-22

FRMPD2B 1.73738653483992 0.4806309375 -1.85391734074358 7.29326271964529e-17 1.68052317561096e-16

AC008753.1 0.728739218455744 2.73974421875 1.91056667092015 5.48426220775871e-27 1.97520541842482e-26

HNRNPUP1 1.29805371939736 5.64558333333333 2.120772566154 1.2858197413529e-33 6.38616936176741e-33

CTSL 41.4412511770245 86.6760165625 1.06456529095967 1.53593046411499e-59 4.76010449670304e-58

ARSF 2.80606906779661 10.7944860416667 1.94367212929557 8.30443167346286e-24 2.60230716157806e-23

FES 6.45668361581921 14.5551847916667 1.17266791555862 3.94259438641005e-55 8.03865598851917e-54

HECW1 5.25289821092279 1.66813989583333 -1.65487334552992 4.10566249093524e-47 4.56062090913625e-46

CACNA2D2 6.82427132768362 3.26219291666667 -1.06483290683126 6.6269648594248e-53 1.14524546060413e-51

RPL24P4 19.8613130885122 45.9401952083333 1.20979598026879 4.39704708156051e-19 1.11713472443801e-18

AC005912.1 46.3949890301318 94.1465541666667 1.02093929907286 1.11010974793508e-26 3.93790361748432e-26

HDAC4 25.1669072033898 11.6796972916667 -1.10752504888898 1.57994494917306e-60 5.42049378779946e-59

CHCHD2 643.019617419962 1859.48631911458 1.53196947610568 4.25551745719912e-51 6.37128398684522e-50

AC113346.1 1.56816059322034 0.592039166666667 -1.40530878509692 3.19851504405472e-16 7.16582978845753e-16

AC008080.4 0.748694632768362 6.17197270833333 3.04328236528575 2.5092182843181e-11 4.55208917042879e-11

RPL10AP6 5.9330881826742 18.2040503125 1.61740434810836 1.02202893777882e-23 3.18976638099986e-23

BEST4 0.77488093220339 3.44581947916667 2.15280057434631 1.49174849036153e-54 2.89249876728599e-53

MAP3K4-AS1 17.9182100753296 37.27270203125 1.05669288119858 1.50789955759885e-07 2.30947963706042e-07

DHRS2 2.95783912429379 0.8894171875 -1.73361139759225 2.91520351520898e-24 9.30932669849064e-24

AK2 19.6911733521657 42.1111005729167 1.09665150009871 4.87758736209963e-75 1.37422328785216e-72

RPS3AP49 0.610304143126177 2.30609880208333 1.91785403540801 1.96030086056445e-22 5.81461070381176e-22

FN1 59.9740332391714 258.2164471875 2.10617099447033 4.91237129663976e-51 7.31349433635039e-50

AJUBA 3.33326539548023 6.71831192708333 1.01116259078388 4.83254063166148e-39 3.19108995190857e-38

IL1R2 0.477513983050847 2.73175125 2.51621123417945 1.26900426671991e-31 5.70668303256511e-31

H3C3 0.246896892655367 1.87427036458333 2.92434849307183 6.55896936228277e-31 2.84563778095306e-30

CASP6 5.99107608286252 19.08024640625 1.67119274268293 3.29084204669312e-83 6.11932078582586e-80

LYN 12.6117085687382 27.6470509895833 1.13236186389819 1.76965704088805e-46 1.87183007254342e-45

BTG3 30.7516080979284 68.397363125 1.15327885151722 3.11254772135137e-63 1.40140011812418e-61

HMMR 2.27606059322034 7.10718583333333 1.64273945675194 7.59698303272278e-35 4.00754324804199e-34

PHF11 7.52826676082863 15.8968540625 1.07835163358745 1.11938832552508e-50 1.61107011711601e-49

RUNX2 1.64300969868173 3.45841703125 1.07377084949255 3.87179619354434e-26 1.34622382608371e-25

AC022150.4 0.658645433145009 1.471314375 1.15953160234459 6.98093133645179e-19 1.75799591280229e-18

TTF2 2.7002002354049 5.58303671875 1.04798364913412 2.89998966454175e-57 7.15189758781881e-56

RPS18P12 3.10845880414313 26.3740809375 3.08484945650205 7.4874530106269e-26 2.56833035846905e-25

TFPI2 0.965591760828625 4.56778796875 2.24201041300339 1.76963317507468e-29 7.14888744091107e-29

CCDC18 1.20201468926554 2.73954005208333 1.18847916933805 1.76645691528025e-42 1.44130172617974e-41

SCAMP5 206.261394962335 102.3848859375 -1.010471063274 3.13386426486743e-56 7.10661048844023e-55

DCN 15.5648742937853 45.9114664583333 1.56056058866292 9.43366610706355e-22 2.70332903776925e-21

DEPP1 32.5600848870057 116.828118125 1.84321117707686 6.04386654540917e-61 2.13661023596737e-59

DBX2 7.84288559322034 16.06105703125 1.03411038389598 5.42479771542528e-08 8.48323215190758e-08

ADAMTS14 1.84610626177024 6.01387776041667 1.70380994681246 1.64864327859562e-25 5.57087438951218e-25

HEBP2 4.77026252354049 10.2584801041667 1.1046764272887 5.52510457316065e-71 7.72476086751295e-69

PCDH7 18.2442142655367 7.94303104166667 -1.1996774723237 8.4027295271212e-53 1.43743105388058e-51

HCP5 2.47447655367232 7.50994005208333 1.60167801915888 3.47680047414406e-36 1.97287779623008e-35

EEF1A1P4 1.78774981167608 4.91839802083333 1.46004363826301 3.53094073757091e-18 8.63692347499498e-18

DLGAP2 2.0714700094162 0.58400140625 -1.82661118507119 8.23546168873176e-32 3.73601978541096e-31

MGAT4C 3.91248220338983 1.26479963541667 -1.62917533276263 3.43422990419588e-54 6.48978710045959e-53

GNG12 24.7130005649718 99.5802605208333 2.01058960155043 3.03190476602755e-77 1.52373700335898e-74

JCHAIN 0.983333615819209 14.2379638541667 3.85591807049472 2.11611634417589e-21 5.97195073910315e-21

ORMDL2 4.64900692090395 9.8401765625 1.08176162852891 1.35117384008976e-70 1.78192039407582e-68

GAPDHP65 2.13811337099812 15.4213703125 2.85052070853848 3.77511930320805e-40 2.65400164246328e-39

PTMAP5 2.9834738700565 9.23466427083333 1.63006637102601 6.15982055198688e-53 1.06848752951675e-51

LINC02828 0.692050047080979 1.48757755208333 1.10401660427014 1.36788136523876e-27 5.07092384103165e-27

CDHR1 34.9294902542373 9.4705990625 -1.88291799749317 1.07293640505959e-67 8.63690582341259e-66

GJB2 2.3617052259887 16.9227445833333 2.84106275573831 9.05511500676775e-30 3.72027979564397e-29

EMILIN2 1.9880233992467 13.4570579166667 2.75895638878211 5.69025758188856e-69 5.65830693771218e-67

AC090692.1 5.44370235404896 18.0782110416667 1.73159182891221 4.59022874490769e-34 2.33211211780215e-33

ACTG2 1.90972735404896 8.65453833333333 2.18009017890067 4.49781653651261e-29 1.78102424396192e-28

SRGN 145.429856826742 368.3038540625 1.34057300846824 1.29045524167961e-35 7.07429693957322e-35

STEAP1 2.05234467984934 9.89773630208333 2.2698255621983 1.13813901409172e-39 7.76934470155489e-39

SMIM32 3.30021892655367 1.27373057291667 -1.37350158926593 6.44703534375732e-28 2.42382980625086e-27

ASIC2 3.22215564971751 1.5343578125 -1.07039122787211 1.1844329019323e-20 3.23794910488549e-20

STK17A 20.8433981638418 48.2331596354167 1.21043481675366 2.42078862378385e-53 4.33666324270334e-52

LYZ 10.8290644067797 48.9957875 2.17774911175296 1.02252728124434e-44 9.61268695386173e-44

C2orf27A 8.91656610169492 3.81329723958333 -1.22544922351847 1.34114148926594e-48 1.64719458341481e-47

FP671120.6 0.0861808380414313 8.7248309375 6.66161623842562 2.34622990786247e-32 1.09673567462802e-31

COL4A1 14.0581190207156 91.1583517708333 2.69697126512595 3.00201175429759e-62 1.2432607699591e-60

RPS6KA1 12.3890063088512 26.0507535416667 1.07226462720443 7.73462986361595e-51 1.13070316284543e-49

HTR2C 3.7232290960452 0.530029322916667 -2.81241030867965 2.23066508050039e-49 2.88250293063966e-48

DMRTA2 0.650087193973635 15.3267497395833 4.5592747398453 1.64364506869744e-56 3.82044750655361e-55

FAM201A 2.93877259887006 1.34665979166667 -1.1258283011096 7.16061058898951e-37 4.22971899308323e-36

AL162424.1 0.59598290960452 2.394379375 2.00630889104242 2.76389997625398e-41 2.09261889488773e-40

FPR2 0.293428342749529 1.88941005208333 2.68685570353138 5.94612586205476e-34 3.00293890290354e-33

KCNK3 17.0161026836158 4.12592182291667 -2.04411225365672 1.21809921173545e-48 1.50202618317113e-47

ECE1 34.7052700564972 83.2705083854167 1.26265087674319 9.98387988972578e-66 6.25085005217006e-64

KCNN4 1.1153036252354 4.87818739583333 2.12890866399117 9.02396159494236e-47 9.76720406623709e-46

ST13P4 0.717598964218456 1.44587864583333 1.01069675742008 7.1153419583551e-15 1.51159355324589e-14

HK2 15.0904108757062 36.7772533333333 1.28518165023488 2.84237503823756e-41 2.1502833131012e-40

C21orf62 6.9231700094162 32.2743410416667 2.22088295837602 3.613512841338e-46 3.74127345683074e-45

CRYZL2P 1.71274373822976 4.52443239583333 1.40142750230132 1.25714902126599e-44 1.17470784173071e-43

SIRPB2 2.29719924670433 5.65439458333333 1.29949656921377 1.15665685709295e-38 7.48886986686748e-38

CCDC81 0.768980885122411 1.883190625 1.29215940133051 1.37388099389344e-28 5.33346911930032e-28

S100B 633.691375094162 1280.68320901042 1.01506136855794 2.00257055595449e-27 7.35779480102228e-27

HSPA6 7.76790880414313 25.48546953125 1.7140767643468 2.5307519719418e-56 5.80263044614769e-55

ACTG1P20 1.55086958568738 4.8881984375 1.65622547919341 1.40893438194388e-40 1.02021553085072e-39

HNRNPA1P7 1.83748983050847 6.4570884375 1.813147521193 7.64388024131558e-19 1.92156216151498e-18

POGLUT3 10.058806920904 29.6364983854167 1.55891580360379 8.63779819541682e-58 2.249577835347e-56

AL118505.1 40.4800596986817 11.00359890625 -1.87923595983085 2.47689897739657e-61 9.23004739172129e-60

RNU6-277P 9.30762871939736 3.52178833333333 -1.40210546003718 2.37951544160839e-39 1.60083537035847e-38

H2BC12 19.7414758003766 58.43248578125 1.56554081997905 2.38116293891475e-61 8.90899896360559e-60

OS9 119.538387052731 293.720978645833 1.29697233046138 1.91994908832052e-22 5.69855599318756e-22

EXO1 1.79655103578154 6.74677125 1.90896732844785 1.0552063333013e-35 5.82069468043242e-35

PRECSIT 0.429545668549906 1.6311678125 1.9250217828792 1.67100585558859e-49 2.17289188004684e-48

SYT1 118.948940772128 35.1857008854167 -1.75728126843565 2.11948978898324e-14 4.41194588896713e-14

HNRNPKP4 1.40653206214689 5.99305078125 2.09114815891231 7.73105956800214e-36 4.29259637703792e-35

ZWILCH 4.1739527306968 9.09629895833333 1.12386540821009 7.61463787226421e-46 7.73738749916683e-45

AP001527.2 0.780382391713748 1.76859770833333 1.18035279374397 2.52467990468074e-22 7.43646805441761e-22

AC021739.2 27.8040541431262 13.8822294270833 -1.00205598219607 2.2590858232755e-28 8.68286500285406e-28

KCNK12 2.66622344632768 0.910243541666667 -1.55047318777891 1.16458624230494e-42 9.59480778717783e-42

SAMD11 8.70515367231638 3.68500286458333 -1.24020402521835 1.15040744836069e-45 1.15071686402727e-44

F11R 4.13058893596987 11.8225222395833 1.51711845598671 6.52203694316872e-63 2.86031313580713e-61

RPL13AP5 47.6046723163842 154.93432421875 1.70248171121223 7.47178734442865e-26 2.56342962490131e-25

AL009174.1 4.9871918079096 12.2304769791667 1.29418107357377 5.69972429034333e-16 1.26324640260947e-15

TRHDE 1.6607788606403 0.459337604166667 -1.85423318417694 7.67674979542811e-29 3.01158570561151e-28

OLFM3 6.03045998116761 1.685258125 -1.83929846895471 2.42718502447891e-15 5.24625195050393e-15

PID1 138.2463663371 57.956248125 -1.25420545296655 1.14302800329739e-53 2.07564508997216e-52

HLF 31.9090199623352 12.570893125 -1.34387714644335 3.68988543173478e-56 8.30670939504942e-55

SNORD15B 1.65610306026365 5.23106708333333 1.65931281590288 3.00579200620875e-07 4.53674532105939e-07

OLFML1 1.48897048022599 5.03922473958333 1.758886647255 1.12615929581735e-33 5.6051745464999e-33

IL10RA 7.06565927495292 14.2895413020833 1.01606352090558 3.54340266116974e-25 1.17575968030784e-24

EEF1AKMT3 6.47722937853107 38.36120359375 2.56619924568726 1.25574137632935e-55 2.67474351578972e-54

LINC00463 2.14947565913371 1.042493125 -1.04394690373556 3.37679513260634e-33 1.64117892030358e-32

AC018868.1 1.21329901129944 3.03369447916667 1.32214066124234 1.08723109112655e-20 2.97528508307553e-20

PODNL1 0.798694774011299 5.50025489583333 2.78378229931108 3.75790175531764e-60 1.2367820024802e-58

IGHV4-59 0.690363559322034 4.37920052083333 2.66523929207903 8.89606249942939e-15 1.88322270237807e-14

APCDD1L 0.0528236346516008 3.18175010416667 5.91249314722076 7.64393213940963e-52 1.20969292027508e-50

SLC8A2 28.4743085687382 8.40745395833333 -1.75991993165274 3.99118629699212e-50 5.47316439473219e-49

C9orf24 4.29896887947269 16.1222344270833 1.90698913329537 9.94867269889005e-06 1.3977753595456e-05

OAS3 12.9368144067797 36.9529334895833 1.51420648825754 3.74294420877687e-33 1.81439122946314e-32

MAPT 150.630093644068 71.5455818229167 -1.07407544412855 1.11863057795856e-68 1.01965370574213e-66

KCNE5 3.18931012241055 21.08822875 2.72512163043829 7.551496098418e-18 1.81659182246961e-17

EEF1A1P11 9.22099656308851 18.7548416666667 1.02426849936993 2.67465030190038e-15 5.77576615536379e-15

FAM221A 2.56870574387947 6.35324473958333 1.30645196280466 9.98952013411239e-48 1.15952014290774e-46

SNRPD2 155.760104096045 320.69551515625 1.04187842486522 1.37223097285548e-54 2.67750628963774e-53

IFITM10 28.0594414783427 7.31090635416667 -1.94036411503024 5.30815968800161e-67 3.88603265347992e-65

RTP5 59.9943531073446 14.55352390625 -2.04345819398449 5.33663543479131e-61 1.89379267003711e-59

AL731567.1 0.860660734463277 1.98083208333333 1.20259003102088 2.57515773323805e-28 9.8752439780494e-28

AC080038.1 2.3023738700565 7.40510536458333 1.68539813930127 2.42706130465513e-47 2.73688326016144e-46

TUB 88.3282558851224 34.3810626041667 -1.36126089015524 5.70398900592664e-71 7.91534892277656e-69

BCL2L12 4.18919849340866 12.69394921875 1.59939482735447 3.81824803744576e-76 1.39216318149616e-73

CCNB2 5.49592325800377 25.1088586458333 2.19176268619253 1.46660859101954e-43 1.27140264568803e-42

AC126407.1 8.53706822033898 3.63608734375 -1.23135385255968 2.17154191453423e-35 1.17519854193143e-34

EIF3CL 0.48576120527307 2.14723489583333 2.14416084222517 1.02543069107946e-27 3.81815853036095e-27

S100A16 157.667955414313 433.419992395833 1.45887622706216 1.24847479314316e-49 1.64298575927085e-48

BGN 105.024055414313 401.183518333333 1.93354252719476 2.98220459269982e-62 1.24058376736584e-60

RPL23AP18 0.726322457627119 2.07044817708333 1.51126099931438 3.40771542644502e-30 1.42845961124313e-29

CHI3L2 17.4333480225989 252.47479875 3.85621782324138 7.68942546873096e-70 8.88104761435107e-68

APOL6 9.15013629943503 18.39788875 1.0076750803919 1.16352288661764e-28 4.53198744797969e-28

CDCA7L 8.93491694915254 29.2218865625 1.70952309695364 4.70094334414873e-41 3.50919476051568e-40

GPX1P1 5.95956963276836 43.2308372916667 2.85878072242833 3.98832318831052e-43 3.38025841780465e-42

AC021683.1 2.93552481167608 1.2592534375 -1.22104978144188 5.4198845660028e-13 1.06169030724571e-12

MS4A6A 12.6249088041431 46.7145377083333 1.88759862434599 1.81994256399853e-49 2.36160725593529e-48

SLC43A3 2.72203747645951 12.91045046875 2.24578050491455 1.14734484832373e-71 1.83921357367067e-69

ARHGDIG 33.4226516949153 13.9977665625 -1.25562954498261 1.49024662841976e-35 8.15273199631228e-35

PCSK5 2.19650993408663 4.54345390625 1.0485764183802 3.95977840696047e-29 1.57065015950149e-28

SLC2A4 4.01941718455744 1.94380677083333 -1.04810151458639 2.04095042126635e-30 8.63907878066191e-30

AC015922.3 7.21307066854991 20.3930665625 1.49939327026153 2.57897621936018e-44 2.35656328250627e-43

EIF4A1P10 1.38875357815443 8.76516640625 2.65799085198373 2.62381595261127e-37 1.58717819254413e-36

SLC12A5 26.0625403483992 6.45059020833333 -2.01447463838856 1.46314364071067e-23 4.53982245937175e-23

SPAG4 1.07464952919021 6.40747067708333 2.57588873461855 8.21981220351616e-60 2.61277620383561e-58

CLECL1 0.672580320150659 1.6988234375 1.33675744678987 1.44332424569543e-32 6.81529059134244e-32

FSTL3 10.7583442561205 21.53789203125 1.0014209969676 2.7779538379915e-40 1.96784958542674e-39

RN7SL752P 0.53123352165725 2.01945546875 1.92654824239439 2.14847757714421e-18 5.29502194128517e-18

SOCS3 18.1579264124294 104.432397864583 2.52389798073191 7.59140993116334e-57 1.80745541190758e-55

AC009878.1 2.34818771186441 0.973712552083333 -1.26997989582497 3.02381238016563e-36 1.72003032147996e-35

ETV2 0.74739143126177 1.76839546875 1.24250501408895 5.18930721366759e-41 3.86289702314447e-40

ACTBP11 0.334973775894539 2.13365416666667 2.67120629510785 7.11232914642087e-41 5.25650876302449e-40

MIR27B 1.26399190207156 3.51074588541667 1.47379035420659 3.83714275851525e-13 7.56725735439507e-13

AC098935.1 0.927148116760829 2.2368759375 1.27061350283777 1.78792452506524e-19 4.62269974187822e-19

PTPN5 19.0094330037665 7.85780932291667 -1.27451643622972 1.69582946372436e-12 3.24724012747961e-12

RASGRF1 17.4736597928437 5.31924942708333 -1.715887213341 4.49085545689271e-45 4.30450810417113e-44

CST3 687.350282909605 1607.0422015625 1.2252904069795 3.65445538946674e-33 1.77242039559557e-32

AL390755.2 0.314344303201507 1.78109848958333 2.50234977270465 2.0013942821078e-72 3.95914113572282e-70

TECRP1 7.82741064030132 19.5139115625 1.31789595667704 1.22983907661921e-42 1.01279263196343e-41

PAPPA2 0.572302495291902 2.41281421875 2.07586703327028 2.42863195376164e-25 8.13857318744456e-25

TNFRSF10D 2.86657269303202 6.0786875 1.08443298839232 2.47880679028481e-36 1.41477631262572e-35

ERP27 0.669011064030132 2.99648213541667 2.16316779749366 2.08434497613743e-50 2.92075318999815e-49

MSLN 1.60035376647834 4.24031161458333 1.4057794347614 6.81538648089021e-16 1.50477453825877e-15

BTF3P10 0.271030461393597 1.89704828125 2.80722948505761 1.26844565482144e-29 5.16913148178932e-29

PCSK6 7.71778799435028 3.63516942708333 -1.08616480747422 3.38565990069022e-29 1.34810329906761e-28

PCDH15 19.6398751412429 7.2256228125 -1.44259190670355 2.55190048531672e-48 3.08534392226687e-47

HTATIP2 8.01405866290019 20.0654456770833 1.32410822497674 2.82717621803351e-41 2.13965574987111e-40

AC105053.1 1.59050320150659 0.79312515625 -1.00386282758027 1.03449937448305e-29 4.23523099748256e-29

GPR83 2.93362222222222 1.09168671875 -1.4261241940646 1.78198640101658e-10 3.12252671303115e-10

LHX5 7.07427452919021 1.22149229166667 -2.53393744696538 3.4791381672812e-37 2.09299819542523e-36

FRRS1L 14.5809617702448 5.134648125 -1.5057485661215 3.21928347302224e-42 2.58362434962661e-41

SEL1L3 5.44653978342749 23.05846578125 2.08188465102527 5.66395039496146e-38 3.53191396446578e-37

DEPDC1B 1.60112015065913 6.33182890625 1.98354069852204 2.13160290420819e-40 1.52041258165521e-39

HBA1 2.90865838041431 14.1773873958333 2.285165929586 6.20656344394548e-21 1.71487440178553e-20

SYT4 34.9252157721281 10.98199296875 -1.66912913609766 1.49688152647235e-24 4.83657897215523e-24

PCP4L1 19.6583873822976 9.73804630208333 -1.01344071390283 2.14141370526773e-07 3.25669320761049e-07

EYA2 8.83599985875706 25.03817765625 1.50266426224637 1.61521325630132e-34 8.38262209757608e-34

CD302 1.79485875706215 4.45489302083333 1.31152046907022 4.54117986576334e-42 3.61486470906975e-41

ALOX5AP 27.5841996704331 128.109046354167 2.21545832668872 1.4147175785049e-63 6.71088606436189e-62

AL109766.1 0.411153342749529 1.92560458333333 2.22756301742736 5.85876652047293e-25 1.9237818020165e-24

C6orf15 0.0201616290018832 11.9560890625 9.2119176244835 8.43057517412047e-66 5.33219542050239e-64

OSR1 1.04982189265537 3.4223821875 1.7048562903453 5.8946994145778e-15 1.25658529879714e-14

RNVU1-4 0.685494256120527 1.87191421875 1.44929784176701 4.90244012602115e-21 1.36203308147861e-20

AL365357.1 11.9139471280603 28.8036782291667 1.27360159377232 8.30105958404779e-06 1.17062189417085e-05

CCDC40 3.21223267419962 6.65530052083333 1.05092741614726 5.42666640994991e-26 1.87494064379802e-25

TCIM 21.0215649717514 79.3112359895833 1.91565519021493 2.12692686520484e-45 2.08268589038884e-44

AC110285.1 13.9265002354049 6.72167708333333 -1.05093960980148 9.74773355726477e-39 6.33551574614954e-38

RPL5P1 0.923054378531073 4.7126890625 2.35206295039032 1.1448198644878e-21 3.26957680321772e-21

GRB14 1.07807622410546 5.06398609375 2.23181425723142 3.41440043649131e-40 2.40677695665489e-39

EGFR-AS1 0.342265348399247 2.09616442708333 2.61456474538991 0.0421268582725194 0.0474699387696945

CYP27B1 0.677184133709981 10.99450625 4.02109083415942 2.6906575146996e-50 3.74216727642775e-49

AL109659.2 0.602289924670433 1.88447171875 1.64563011359086 1.74394320611696e-41 1.33781451805878e-40

GBP3 12.2651402071563 34.40508125 1.488057924818 2.41331330255944e-29 9.68816080766252e-29

CMTM7 4.75861516007533 10.03507078125 1.07643710229297 2.1315917485909e-40 1.52041258165521e-39

RPS7P4 0.76455725047081 2.1807453125 1.51212484806938 4.59906231309397e-35 2.44901385200408e-34

RPL34P34 0.973648822975518 2.53492390625 1.38046902286317 5.76548621079929e-18 1.39686275035587e-17

COL4A2 12.5547965160075 66.1605114583333 2.39773174132993 4.89810860447533e-63 2.16342825416197e-61

GASK1A 7.12608239171375 14.8285975520833 1.05720108901084 1.6803907490443e-28 6.49352992071462e-28

AC074286.1 2.19677179849341 0.520268645833333 -2.078056339284 1.02739241249116e-71 1.69693969666901e-69

SYNC 1.68601040489642 5.4308246875 1.68755785273351 1.88596162768305e-35 1.02512296015102e-34

GFOD1 10.8808787664783 5.30885552083333 -1.03532229236027 4.36476182800884e-24 1.38455725335763e-23

TRIB3 13.8530911487759 27.97371109375 1.01386372761092 5.48760860862335e-14 1.12368772246835e-13

ISLR 19.0757026836158 40.9911263020833 1.10357542907097 5.13453018155746e-17 1.19018435210747e-16

AL450345.2 1.10318290960452 5.44150791666667 2.3023344854577 5.8233299614083e-23 1.76647341977793e-22

BTN2A2 4.83093888888889 11.0505685416667 1.19374508888425 5.09058411799732e-65 2.90366293479019e-63

ZBTB42 3.24693691148776 9.46356578125 1.54330452662368 1.28194300486735e-56 2.99845662585011e-55

TRGV7 0.701710687382298 1.556963125 1.14978653552607 7.75136042893165e-21 2.13377567988133e-20

RPL9P18 0.51315263653484 2.1540571875 2.06959662971954 8.23745725302413e-26 2.82039251739981e-25

AC093627.4 0.744675800376648 1.53980463541667 1.04806293847608 1.94289727529585e-39 1.31518656112582e-38

YPEL4 16.2010652071563 7.15894458333333 -1.17826985518432 8.30294989186403e-48 9.70417053671978e-47

SCN8A 9.31883785310735 3.80103973958333 -1.29375593988468 2.58266870737354e-30 1.08751640882271e-29

TNK2 131.115143785311 52.8871483854167 -1.30984523218722 4.01056083715017e-60 1.31296441490858e-58

MYL9 31.5886242937853 70.5622152083333 1.15949074503159 1.43937678326364e-35 7.87675435102631e-35

NT5C1A 4.49244199623352 2.04251734375 -1.13715154776049 1.72654771752209e-33 8.51595618231386e-33

IRX5 0.492880838041431 3.674476875 2.8982280732255 9.44125569593405e-58 2.44512743267261e-56

SOD3 7.29389552730697 32.2190990104167 2.14315470965327 7.88104899717306e-55 1.56234654693425e-53

COX7CP1 1.75806892655367 8.89814734375 2.33951335454129 8.45927151722762e-27 3.01572380872024e-26

CCDC125 3.11999882297552 6.38514583333333 1.03317408586273 4.2427782779714e-51 6.35732974044143e-50

CEBPD 57.9297879001883 191.7669903125 1.72697711638878 7.21714714436126e-57 1.72054937371023e-55

CSMD3 9.69509675141243 2.06873291666667 -2.22850789728231 6.63085615766731e-77 3.08251925629559e-74

RNY1 2.60252495291902 7.7413984375 1.57268220807341 1.19698007443438e-11 2.20549390448943e-11

CSF3 0.677383757062147 1.90966838541667 1.49527683743595 2.73047076857114e-09 4.53412251666194e-09

GNG13 2.82983112052731 1.18406864583333 -1.2569632350873 4.3193173409385e-08 6.78702940297037e-08

AL596325.2 0.747239783427495 1.5519284375 1.05441886150212 2.12971188831144e-16 4.81424660383555e-16

H4C8 3.0729013653484 7.23120640625 1.23463489713796 2.53642913501128e-34 1.305783492955e-33

MIR1250 4.08677754237288 1.570299375 -1.37992408176573 3.43789127021973e-36 1.9531802068358e-35

RPL39P3 51.0886205743879 168.591606458333 1.72245882402858 9.81526212709874e-26 3.35320226443875e-25

TUBAP2 2.20099825800377 14.2216829166667 2.69186228723884 7.57957017017526e-47 8.23741129832899e-46

AL109741.2 0.766623917137476 2.11792848958333 1.46606296537305 4.02027985842272e-27 1.4572534886427e-26

LRRC36 0.472365725047081 1.89080979166667 2.00102804950703 1.18804228222306e-50 1.70591862841219e-49

DLGAP1 40.1080596986817 13.52240578125 -1.56854032940798 1.98486213135161e-64 1.03967637556291e-62

RUNX3 1.76372123352166 4.70892213541667 1.41677431458216 4.4685660072634e-44 4.01416919099654e-43

PTGES 4.17544562146893 9.05552942708333 1.1168688136914 8.83681633370153e-06 1.24419322878155e-05

AL359399.1 2.11912645951036 0.722025625 -1.55334773853927 2.1071390783263e-39 1.4237736613909e-38

TMEM255A 32.9930475517891 71.3311028645833 1.11236923443452 1.14856248826358e-37 7.05800379023836e-37

PCLAF 1.12271398305085 4.99611390625 2.15381592844423 2.59318460836252e-40 1.84117097336774e-39

AC124067.2 0.612470009416196 1.50320119791667 1.29532701450653 1.27974860630393e-29 5.21290806883277e-29

GRIN1 43.7036492467043 9.68632755208333 -2.17373205458911 1.10993166310332e-41 8.61401472262364e-41

KISS1R 0.281954143126177 1.984371875 2.81514996715834 7.85426777666462e-16 1.72942699001869e-15

FBLL1 56.5907421845574 25.8839329166667 -1.1285092151057 9.54345410258158e-43 7.9117489539681e-42

ZBTB20-AS4 3.81568502824859 1.57391197916667 -1.27758722615686 4.88222136370761e-44 4.37096322860583e-43

ABHD17AP1 0.567676365348399 1.77917286458333 1.64806610700977 3.85605866503059e-31 1.69351466405866e-30

TMSB10P1 1.12987278719397 11.6124753125 3.36144327620455 1.23772797136883e-57 3.17455884518668e-56

XAF1 1.69793107344633 6.25846807291667 1.88203166756908 1.3643963452198e-31 6.12086472375831e-31

CBR3 4.7502384180791 9.88600088541667 1.05738711046714 4.69717984992403e-44 4.2113818374801e-43

SH2D2A 0.441700706214689 1.72072708333333 1.96187725209795 2.09816317262541e-60 7.08082471778031e-59

PTMAP4 1.64895767419962 4.61157197916667 1.48370424831372 1.77605997509061e-45 1.75296365375849e-44

DLG2 16.0457287193974 7.66821375 -1.06522685381 1.17228194698399e-37 7.19900356808695e-37

LRRK2 5.27420814500942 11.1340703645833 1.07795469286601 2.2938157832369e-17 5.39917778345445e-17

PART1 2.21327198681733 0.715928645833333 -1.62829304213553 3.44864461708289e-06 4.95652702540241e-06

YBX3 14.2210652071563 36.4187396354167 1.35665146311723 6.03400932330358e-59 1.74770098702228e-57

ADGRL4 7.11766793785311 14.3362140104167 1.0101875460041 6.91098473479983e-36 3.85336615123247e-35

SERPINE1 15.4792624764595 201.153248854167 3.69988639960548 4.70969263412786e-68 4.01728140053246e-66

PDGFA 16.6185232580038 67.5536938020833 2.02324246936412 1.0968318475653e-57 2.82487371266991e-56

DPY19L1 27.4994634180791 79.45080921875 1.53065844532395 1.00071395997752e-68 9.39811923524343e-67

AC099681.2 0.346677354048964 4.02756583333333 3.5382426671803 2.62684446720717e-10 4.57704018625537e-10

AC097468.2 1.72522947269303 0.572367239583333 -1.59177526302974 2.61266875125559e-33 1.27714446450047e-32

PCOLCE2 4.17377179849341 10.0756883333333 1.2714547734171 6.87794130363875e-12 1.27984907976746e-11

INAFM1 31.7288259887006 85.8791934895833 1.43651450210109 3.33735503776103e-62 1.3760114617997e-60

LIF 1.16854849340866 16.95369734375 3.85881042689033 2.40485555696072e-64 1.23873376957575e-62

LMO2 44.4127835687382 106.759135625 1.26531262978129 8.59600019787845e-48 1.00340630056215e-46

GPX7 15.3932018361582 42.4591096875 1.46378077196431 3.57538509478517e-73 8.4157323844975e-71

AC027309.2 9.86463342749529 39.7335206770833 2.01001928618563 4.44895239755368e-38 2.79110222106986e-37

NLRC5 4.6307040960452 10.3636122916667 1.16222347336462 1.10099280506286e-41 8.54820927354651e-41

DYNLL1P1 1.07116887947269 4.26579640625 1.99362915983049 2.94589384057078e-48 3.53868836985876e-47

CCL18 1.94392928436911 20.9888805208333 3.43257757686622 1.43125422120756e-10 2.51575500929715e-10

SLC1A6 5.20707175141243 1.57202197916667 -1.72785089791417 2.10550348328647e-63 9.69104882963166e-62

AC124303.1 2.04334952919021 0.717650416666667 -1.50958285650639 1.8927195746831e-36 1.08862111015256e-35

CAMK2A 133.287495103578 31.48727765625 -2.08170049991826 4.09665237876867e-15 8.7903589872148e-15

COL1A2 20.4905196327684 147.775633541667 2.85037992951192 3.84389990461759e-51 5.77827960601165e-50

HOXD10 0.327015395480226 8.42114145833333 4.68658533643386 7.67323957125336e-54 1.4057526091375e-52

SH2D5 7.69331553672316 2.83027484375 -1.44266332301611 1.31212743771762e-07 2.01594726136984e-07

TBX15 3.93467947269303 8.46811458333333 1.10579467526956 3.67803408794901e-25 1.21826000143077e-24

SOCS2-AS1 0.972209274952919 6.02634807291667 2.63194520053344 4.45448010222627e-56 9.93178147492776e-55

AC010857.1 1.52717688323917 0.568326822916667 -1.42607445809417 5.58486566736505e-12 1.04435415410954e-11

FBXO17 2.05868413370998 13.9823266145833 2.76381004285269 1.64617816501329e-53 2.97479912326746e-52

BZW1P2 1.50923959510358 6.92069208333333 2.19709446246944 4.90858425942319e-39 3.23787651301945e-38

ARHGAP15 1.76143262711864 4.30783338541667 1.29021315730112 1.49010170413045e-51 2.30711417055002e-50

SPN 1.66061101694915 3.37710432291667 1.02407257200123 5.99512351858355e-36 3.35477947120256e-35

CEP112 1.67396285310734 4.47817609375 1.41964374648522 2.26498433044939e-55 4.73229029491083e-54

IL20RA 1.05137396421846 3.33316177083333 1.6646154243718 8.89040476533675e-08 1.37660984770953e-07

RHOV 1.68015 0.8227471875 -1.03006894458595 6.84992821289144e-07 1.01842806702909e-06

SULT4A1 64.101986440678 17.9308646875 -1.83792400329505 1.33562223288738e-18 3.31853225822299e-18

METTL7B 13.1237654425612 274.90823296875 4.38869649399647 3.09484120986797e-83 6.11932078582586e-80

B3GNT7 1.92012645951036 11.66661328125 2.60311258463366 6.79771773348803e-69 6.58351881532343e-67

SLPI 19.1349876647834 91.7805497916667 2.26197547794588 1.76703263499775e-14 3.69149217478746e-14

EEF1B2P3 7.57153333333333 21.2955950520833 1.49189764365383 2.53073892770895e-16 5.69998672005183e-16

VKORC1 5.69794618644068 14.5218739583333 1.34971373476923 1.87696059788741e-59 5.74993118908013e-58

RPL5P23 0.862024529190207 2.19033364583333 1.34534981986483 2.1520713711817e-20 5.8131561805816e-20

RNU6-529P 88.1030114877589 37.3147540104167 -1.23944515788721 4.8560349355515e-44 4.34961318047111e-43

GPRC5A 0.657491713747646 4.03220734375 2.61652520926038 3.97847297602093e-56 8.92396923873452e-55

DTX3L 16.7157683145009 36.568704375 1.12939984533396 7.70213319649579e-49 9.57106246510373e-48

CACNA1E 7.01932212806026 3.19432015625 -1.13582279631191 3.62508696431574e-25 1.20200592192317e-24

NDST3 2.21775936911488 0.939564895833333 -1.2390381217034 4.569209762253e-32 2.10555454205695e-31

RAD51 2.2973434086629 7.55209494791667 1.71691037494297 2.11308462473346e-38 1.34887774105454e-37

AL691432.4 3.1086695386064 1.37573427083333 -1.17609542777583 2.10626235496367e-48 2.55152758896088e-47

TENT5A 5.24205367231638 18.1762872395833 1.79385350870826 1.65780064471951e-66 1.15456190968387e-64

AC015908.5 0.598735734463277 1.84775770833333 1.62578430972787 1.42857998183626e-23 4.4340585481965e-23

RPA3 11.3757565913371 28.3529111458333 1.31753437117784 6.10962037964272e-72 1.06176066317249e-69

PRKAR1B-AS1 1.83421417137476 8.84099401041667 2.26904647923922 8.01040079151158e-71 1.10335853865302e-68

EXOSC10-AS1 0.917674435028249 1.91511598958333 1.0613774499944 2.94707959330959e-38 1.86778953775023e-37

AC092958.1 2.02572904896422 9.65433255208333 2.25273530293465 6.65055366166097e-34 3.34779600659749e-33

AC092670.1 3.60167528248588 9.23797713541667 1.3589088578896 1.62528178980808e-23 5.03450189596556e-23

SERPING1 17.1140641713748 105.358571927083 2.62205338557565 1.49981735473441e-74 3.98415767304091e-72

TTC12 1.26206087570621 4.96593265625 1.97628319642317 4.10528405483652e-45 3.94510372091396e-44

RAB3C 22.1333001412429 5.77567333333333 -1.93815752515564 5.61660190704511e-43 4.72582409328072e-42

HOTAIR 0.127414548022599 3.19517588541667 4.64829343254843 4.42493107582369e-70 5.30848989386719e-68

FSTL5 10.9766812617702 2.68833916666667 -2.02965486125808 1.77094598464324e-60 6.0202450794225e-59

CEBPB 28.2984324387947 62.86287078125 1.15148601898097 1.42495757189288e-46 1.51671929303653e-45

CD14 131.919590348399 341.445828541667 1.37199788467333 1.72485815753634e-32 8.10761815960269e-32

AC023024.2 3.88123540489642 1.81075046875 -1.09992818942093 7.45145901671409e-34 3.74081750582609e-33

PLAU 4.3480720338983 37.3835225520833 3.10395477028713 1.05425285139936e-65 6.57846703750708e-64

NMNAT2 53.3097190677966 16.79991546875 -1.66594460548634 2.1384386085005e-57 5.33748535906937e-56

AC125807.2 1.18432758945386 3.36439786458333 1.50628013386441 1.03828349827655e-21 2.97074652261155e-21

CD24P4 0.503644161958569 2.34022572916667 2.21617099712909 1.45362979022807e-05 2.02488920138519e-05

CTHRC1 5.48464359698682 24.6439452083333 2.1677634535757 5.90259889075308e-53 1.02482564307706e-51

TACSTD2 0.787417467043315 1.60955859375 1.03146447625324 2.7289736812261e-24 8.72811585868582e-24

AL049872.1 1.20162871939736 2.79175020833333 1.21617866292425 5.71202504898341e-35 3.02820568442668e-34

MMP7 0.91277170433145 9.97863859375 3.45051702483939 1.19580342499223e-34 6.23554814574608e-34

PSMB9 18.3648215630885 48.5771959375 1.40333433589873 1.60492383120188e-52 2.66698468643422e-51

EMP3 9.76318752354049 176.236538802083 4.1740170149935 1.20474008273484e-84 3.73369030640906e-81

H3P47 1.41024585687382 3.30390994791667 1.22822766639434 1.3969457593811e-32 6.5979696204449e-32

LINC01224 0.370849058380414 2.17970604166667 2.55522957316717 1.28072322663762e-35 7.02716093222973e-35

KCNJ11 37.1631489171375 11.0308236979167 -1.75233222526172 2.13846817099688e-47 2.41878440630699e-46

C6orf118 1.25046619585687 5.11727473958333 2.03290963444358 4.56638662167591e-35 2.43300742779552e-34

TCTN2 6.88903893596987 14.2983052083333 1.05346951603117 9.4935956026801e-46 9.55784570827485e-45

AUXG01000058.1 120.662005037665 25.07169203125 -2.26684019214104 2.4842033094473e-80 2.00842437126837e-77

PDLIM1 8.00683248587571 44.5832836979167 2.47719935106795 6.07877739164955e-60 1.96582374952562e-58

CALHM6 0.435185875706215 1.86604854166667 2.10028287693808 1.79656674467091e-45 1.77226305661303e-44

FCGR2C 0.436321374764595 3.62067072916667 3.05279392514876 6.38602740867928e-68 5.32503047822382e-66

AL035446.1 2.24534849340866 19.6260508333333 3.12775861840337 1.98324596257376e-45 1.94917857685302e-44

PSME2P1 1.11675466101695 3.44950005208333 1.62707500623002 5.96268587564353e-56 1.31838458808075e-54

C1QTNF6 4.05775296610169 8.81248140625 1.11886727117001 3.42374561760264e-29 1.3629747325909e-28

FAM180A 1.38820348399247 4.66191541666667 1.74770377438111 1.86288323991823e-12 3.56308515184936e-12

C8orf76 2.29536944444444 4.8179540625 1.06969426098216 3.34708573444284e-58 9.07274915917852e-57

AC002428.2 3.96807321092279 1.42065598958333 -1.48188139396352 4.82757077322919e-34 2.44999822643e-33

BLOC1S1 10.054493220339 20.9823355208333 1.06133490477971 6.54978442192326e-48 7.69382446782457e-47

SNAP25 464.019411487759 142.731626770833 -1.70088011378096 5.0513762711289e-23 1.5360644605338e-22

MKI67 6.49671035781544 13.6120546354167 1.06710355484506 2.43041183773122e-17 5.71490998009763e-17

PPIC 5.20151111111111 21.29691796875 2.03364195053328 1.29358091223869e-64 6.93202797206871e-63

CLEC10A 0.80533225047081 2.04196260416667 1.3423004313414 4.07474823425049e-17 9.49022337373345e-17

NAMPT 20.3273040018832 129.379227708333 2.67011521693537 4.07122120865518e-65 2.38815010646508e-63

GALNT12 0.922763983050848 1.96668213541667 1.0917302016323 1.0683917327347e-35 5.889933077439e-35

HROB 2.54073601694915 5.13766453125 1.01586620431846 2.15674125022109e-33 1.05984681680394e-32

RPS4XP11 0.641493832391714 2.59235494791667 2.01475596726241 2.99480418659246e-25 9.97820889619903e-25

UBA52P5 0.873917608286252 5.01882963541667 2.52178179852153 5.33850223067717e-41 3.97236690594006e-40

AL354743.2 0.859437664783428 2.48141651041667 1.52969900502642 1.71184908214657e-35 9.32879269000851e-35

RGN 8.92282080979284 24.9186282291667 1.48165287739998 5.11457627967016e-30 2.12668930949165e-29

AL022344.1 2.35401741996234 0.800038020833333 -1.55698452743423 1.38075958656953e-45 1.37447668695184e-44

TC2N 1.41299425612053 4.53811708333333 1.68333822992529 7.1621936861607e-35 3.78247633042199e-34

ANG 1.27989161958569 5.4115278125 2.08001431232095 4.4046651336454e-75 1.26007304861748e-72

LTF 4.90037438794727 379.152880885417 6.27374389949525 2.62920991135395e-65 1.58734280200087e-63

LINC00092 1.40767933145009 5.13249328125 1.86634110746486 2.86528744014805e-41 2.16585446949402e-40

AC008875.3 2.49423140301318 7.5427559375 1.59649642728945 1.10283237971015e-50 1.58970295354343e-49

PLB1 1.7639629472693 4.16124807291667 1.23819604040951 1.32552416727791e-47 1.52337915997611e-46

AQP5 0.534162711864407 9.81853354166667 4.2001563904356 8.53687035449106e-44 7.51624546599249e-43

PPIA 126.801154190207 294.481411614583 1.21560869302586 3.50819058492257e-45 3.38530378446472e-44

AL157700.1 19.9837365348399 8.263715 -1.27396395739002 1.10634305617982e-49 1.46423125478034e-48

ZDHHC12 7.85112796610169 21.9813927604167 1.48531095461034 1.03121369036622e-71 1.69693969666901e-69

SCARNA5 1.08381501883239 5.30477098958333 2.29117192605742 1.41618156923959e-06 2.07353514015828e-06

LY75 0.743820244821092 1.62759536458333 1.12971615676964 2.2952529257973e-34 1.18424606423976e-33

HLA-DPB1 33.3647951035782 124.356924375 1.89808829189481 4.80546013674189e-61 1.71511576281604e-59

TMEM165 18.4895173728814 44.7916160416667 1.27652115135064 1.98603955342917e-79 1.42040021138521e-76

NUF2 3.92305475517891 13.7452647395833 1.80888531726335 5.95106089773545e-39 3.89923710799495e-38

TMEM100 149.312878154426 53.635239375 -1.47708550702974 6.75785532673533e-50 9.09937145551365e-49

MGST2 10.4807943502825 22.3666648958333 1.09360208710817 7.01900130128991e-62 2.78290680591654e-60

SLCO1A2 25.2449236346516 12.4833985416667 -1.01598255922384 6.15480961408881e-34 3.10664182339798e-33

ANGPTL2 269.036489077213 125.176343072917 -1.10383992212347 2.97435547385574e-33 1.44937473889800e-32

SLC16A3 3.84527547080979 15.9371542708333 2.05123518562002 4.00679238945284e-57 9.73938620678112e-56

RNU4-1 5.36352895480226 27.3321806770833 2.34934612373356 7.60619568985743e-13 1.48210425288587e-12

LILRB2 1.95823615819209 5.76542151041667 1.55787132657341 1.34607219702305e-33 6.67650373530104e-33

RPL34P18 1.39614420903955 9.64400885416667 2.78818500891707 4.35457031433341e-24 1.38156005792578e-23

FTH1P11 1.53766450094162 10.6346122395833 2.78995476571896 1.12113378861531e-40 8.17227863555535e-40

ARHGAP11A 2.53501661958569 7.28762083333333 1.52345269372406 6.33427970799985e-39 4.1415587612608e-38

MT1H 1.92877283427495 6.01201744791667 1.64016595959675 1.33174372276853e-17 3.16348678141043e-17

SMCP 4.15755065913371 0.916364947916667 -2.18173966268758 3.6923139948977e-37 2.21837087997166e-36

MT-TM 10.1854401600753 4.37761265625 -1.21829211571515 1.99532089631393e-28 7.68655315246686e-28

MICB-DT 0.87313615819209 2.06355625 1.24085421238079 4.40502788570379e-18 1.07382660637994e-17

SLC25A15P5 2.85288483992467 0.56068125 -2.34716878210702 3.74582014202088e-26 1.30364075502299e-25

LINC01956 0.678727354048964 2.95702463541667 2.12324219995738 1.80001744039475e-41 1.37855536672736e-40

LINC00632 2.33821770244821 1.13800744791667 -1.0388992602233 5.63823539052861e-42 4.44815388573948e-41

CHODL 1.23626266478343 10.0117978125 3.01764385558142 2.38100171268798e-29 9.56051108776355e-29

MT-TT 19.7325109227872 9.0381046875 -1.12648237500181 1.76291969811651e-41 1.35125687495781e-40

RPS3AP21 1.76985550847458 5.9501475 1.74929384919864 1.50644581867093e-20 4.09777062583176e-20

RPS15AP38 0.915838512241055 4.67467046875 2.351699530108 4.3982326294516e-24 1.39493664923508e-23

RASAL1 7.826934086629 3.81080453125 -1.0383516860296 5.19645718370561e-10 8.93300439896170e-10

ARSJ 1.64233394538606 9.75954739583333 2.57106673501294 1.30435229948437e-46 1.39233243449551e-45

HLA-DRA 506.562949387947 2215.0414296875 2.1285202173057 1.98089676457603e-61 7.47155686354793e-60

VWC2L 4.81427848399247 1.80283848958333 -1.41704944131738 4.91020625386269e-20 1.30008949580773e-19

KCNE3 1.77533474576271 6.00184401041667 1.75731474776682 2.13519334464659e-54 4.08897221871301e-53

CXCL9 1.05185536723164 8.99214927083333 3.09572964062338 9.87594740136943e-46 9.91594178879398e-45

SERTAD1 16.2266092278719 40.4493899479167 1.31775638379148 4.75394446224605e-62 1.93434567342375e-60

WBP1P1 0.483801365348399 1.75097046875 1.85566800608786 1.3927422112686e-24 4.5047906450756e-24

TAS1R1 0.608196939736346 1.62548869791667 1.41826306265782 2.57701567757184e-39 1.73057445014259e-38

MXRA5 2.36332467043314 12.2106888020833 2.36925484112297 2.39180041059493e-35 1.29176673351765e-34

CA3-AS1 1.53734411487759 3.205636875 1.06017088048437 2.72191408693636e-33 1.3284512453171e-32

SLC15A3 8.66139905838041 18.9937856770833 1.13285549512672 1.8264406530539e-44 1.68968477331031e-43

ANPEP 1.97175955743879 7.98640380208333 2.01806238252254 1.95791111824025e-37 1.19212040745506e-36

RGS16 11.4197104990584 47.9502963020833 2.07001365278124 6.29473469155028e-62 2.51721702342747e-60

NOS2 2.45640131826742 17.6617893229167 2.84601332284678 1.51929419112973e-07 2.32654825694287e-07

KDELR2 84.58356586629 187.784383020833 1.15062779931721 5.892274667095e-69 5.82802379971444e-67

TMBIM1 34.3168410546139 110.865034479167 1.69181576766816 6.25607518510536e-68 5.24016748049704e-66

AC007560.1 0.518376600753296 3.37938807291667 2.70468952996806 1.97149480708652e-35 1.06973872009845e-34

AC073073.1 1.07341511299435 3.13282911458333 1.54525797205045 1.297664670392e-52 2.18371715347866e-51

AL031658.2 2.85381563088512 1.05422817708333 -1.43670497574178 2.55531780119696e-32 1.19117910537121e-31

CCDC89 1.57813695856874 4.94685203125 1.64828833165123 1.23382825020707e-49 1.62486092865442e-48

FAUP1 3.00318860640301 15.3785713020833 2.35635449340886 2.28374936539042e-24 7.31925533427006e-24

RN7SL674P 0.891637005649717 2.69850645833333 1.5976327409272 1.95365750943772e-08 3.11456287619979e-08

CNTNAP5 4.63051506591337 1.53221765625 -1.59555142654969 2.9282751511047e-32 1.36162231644891e-31

PABPC3 0.568294491525424 1.74499338541667 1.61851093121944 1.30769900528714e-33 6.49310093546445e-33

ACTN1 21.3015695856874 84.6730502604167 1.99094312365327 2.18618366747666e-61 8.21254248418757e-60

WFIKKN2 0.474475470809793 3.14220130208333 2.72737019787687 1.88863587977057e-20 5.11717677172282e-20

AL353751.1 1.44090263653484 0.616927552083333 -1.22379987041451 5.12839718251552e-49 6.44777184644193e-48

TMEM221 2.43391012241055 6.63378317708333 1.44655596462082 3.52144447279665e-25 1.16889075279639e-24

TUBBP2 0.901172928436911 5.12858703125 2.50868552573747 7.13959917228295e-33 3.41726760897301e-32

CDK2 7.09562570621469 21.7300193229167 1.61468764271191 3.08153865180698e-63 1.39080609782405e-61

FZD2 4.30318253295669 10.9326154166667 1.34516263604625 2.69388570099794e-45 2.61991655910338e-44

AC090114.1 2.40492495291902 8.40328197916667 1.80496102122609 2.52410730285267e-29 1.01263808622536e-28

ARFGEF3 27.8613174199623 11.1363995833333 -1.32298059492817 2.29345241981577e-55 4.78639144180407e-54

SLC17A6 2.99730574387947 1.42952989583333 -1.06812546380859 3.33729897040548e-16 7.46865740217715e-16

C10orf105 0.722799387947269 3.10351057291667 2.10223387117463 1.29061141023952e-16 2.94447569797874e-16

AC007683.1 1.11516760828625 4.60051057291667 2.04453342125005 4.03875276470312e-28 1.53266546244193e-27

RFC2 36.0077565442561 80.90500546875 1.16792124723324 4.78972573521817e-61 1.71278750089196e-59

BX470102.1 0.46783549905838 2.19746822916667 2.23176906540789 2.49844882393953e-64 1.28338828400982e-62

ARC 48.3260240583804 129.409141458333 1.42106732339517 1.70438696202502e-34 8.83307568529968e-34

AC021739.4 11.7036819679849 5.76038526041667 -1.02272526288324 1.74309889194492e-27 6.42730991388376e-27

AC069218.1 0.497610781544256 2.36098838541667 2.24630129518189 1.43396288148307e-25 4.85958443250915e-25

MAP1LC3C 0.429845574387947 8.78876682291667 4.35377039312333 4.35861985179093e-66 2.87406156539193e-64

FLRT1 27.418506873823 10.5871844270833 -1.37283104090358 1.36702650964552e-59 4.27224503308545e-58

CCNA2 4.88889185499058 20.9118377083333 2.09674045229125 6.16356145641108e-48 7.27691589091835e-47

CYP4X1 4.96709915254237 2.24019890625 -1.14877671172864 1.0480367735029e-14 2.20930096398214e-14

AURKA 4.45266261770245 15.7548014583333 1.82305136693949 1.40048126820524e-49 1.8339400832589e-48

OSTM1-AS1 0.455509981167608 1.62642598958333 1.83615060219752 1.11184962880449e-14 2.34041484739354e-14

KCNJ3 11.3074277777778 4.15728598958333 -1.44355687838173 3.24083359118027e-32 1.50432602665994e-31

TRBC2 5.57479821092279 14.0077055729167 1.32922917493805 4.82295510044559e-21 1.34015018070511e-20

HMGN2P3 4.83583634651601 18.1655136458333 1.90936483269234 4.80719218524913e-47 5.32081777885164e-46

AC093599.2 9.29409585687382 3.36606005208333 -1.46525360925394 8.92110688893207e-46 8.99629102329577e-45

LAMP3 0.431601365348399 1.62999432291667 1.91709560588742 8.40683721723632e-46 8.5190810928888e-45

GRM2 2.90584934086629 1.18062765625 -1.29940586323405 2.16558433526016e-12 4.1301580219654e-12

GJC1 3.1051129472693 10.9364036979167 1.81642075158354 5.82405919166273e-59 1.68952231932868e-57

AC012213.1 3.08203309792844 0.870610052083333 -1.82378377230207 4.78848647903128e-49 6.02856506957256e-48

AL117339.3 1.54985922787194 0.741813697916667 -1.06300836973671 3.67517877066864e-54 6.91000497882542e-53

LXN 4.69763017890772 15.4334134375 1.71605213409714 1.60178460327944e-54 3.09617304552819e-53

OPLAH 3.62760776836158 9.72160244791667 1.4221756639555 4.32222598096996e-36 2.43994511585114e-35

MIR4653 1.03951699623352 2.2495259375 1.11370765495117 9.64302133785989e-17 2.21236251421967e-16

PAK3 16.5293574858757 6.08498130208333 -1.44170591164452 1.07064164717572e-47 1.23656041511e-46

TNC 55.115672834275 233.02206828125 2.07993206061037 6.66753549521846e-61 2.34372065280883e-59

ADCYAP1R1 510.963174952919 244.23599296875 -1.06494349464025 2.09786092455106e-35 1.13631587218255e-34

RN7SL3 2.99833526365348 7.25642890625 1.2750980223041 2.01777501081535e-14 4.20303868333275e-14

GAPDHP72 0.252564359698682 2.01135572916667 2.99344528531419 9.51013943774231e-44 8.33762578240539e-43

SOX10 42.6079685499058 90.8167830729167 1.09183566551608 0.00546250150019963 0.00655239423275784

COPZ2 16.3595486346516 39.2438781770833 1.2623346748974 3.83641091038756e-41 2.87653471284906e-40

PPP1R36 0.483946421845574 1.79836875 1.89376963202554 1.11597507118622e-48 1.37792539500052e-47

HK3 1.57463493408663 7.04467385416667 2.16151552529744 9.14404233734252e-45 8.62238677803672e-44

EID3 1.5429604519774 5.02487328125 1.70338613014847 1.35458157735928e-38 8.74295190246297e-38

RANP1 0.973749670433145 7.14382682291667 2.87507426778511 8.17248830694096e-38 5.05210837990582e-37

HIPK4 3.23230291902072 0.918905520833333 -1.81457396768082 5.26450864016935e-11 9.4309767017292e-11

CD36 0.840653013182674 2.88633666666667 1.77965724419905 6.88523761637927e-15 1.46321135401797e-14

DPP10 15.7873138888889 7.52717244791667 -1.06858579750804 1.27412761369151e-39 8.68489845183051e-39

ITPRIPL1 2.49007038606403 7.90986067708333 1.66746576044847 9.18726728816654e-61 3.20521845881745e-59

CKMT1B 5.99128639359699 2.24708510416667 -1.41481103346572 1.31153938751503e-41 1.01195331580257e-40

C2 1.68558017890772 6.45523822916667 1.93722508444968 2.6859468246331e-53 4.78860797737799e-52

DCBLD2 6.97645028248588 16.22072140625 1.21727291952522 1.2150098037093e-38 7.85573967314827e-38

HLA-DQB1 13.1635685499058 50.4096543229167 1.93714941483307 2.81846315658898e-47 3.16481415439445e-46

FABP5P1 0.0755974576271186 2.43322098958333 5.00838582735728 2.75927323220736e-61 1.02208537356366e-59

ISG15 129.65903893597 394.795306875 1.60638205540666 3.59777706433849e-41 2.69978468568903e-40

SUMO2P1 2.38701577212806 10.98128859375 2.20176735247199 2.36591666708245e-27 8.660279611102e-27

CRYBG1 0.646583757062147 3.545700625 2.45516155910849 5.23989020875201e-52 8.39239952039135e-51

LINC02151 1.86186002824859 0.527606458333333 -1.81921048782207 4.37425144334704e-23 1.33277413712991e-22

UNC80 14.836277212806 5.72681583333333 -1.37332401547065 1.40431309332115e-52 2.35254071804566e-51

LINC01842 0.19795720338983 2.55010859375 3.68729821295559 1.44739693015266e-64 7.68981311891106e-63

GPR3 2.82713074387947 9.67202192708333 1.77447891148501 2.8662640078149e-51 4.36155312809477e-50

AC188617.1 0.36695263653484 3.60010177083333 3.2943719224438 2.56921837473259e-62 1.07600485761605e-60

EMILIN1 20.194984180791 72.9509375 1.85292950254036 1.61403171068754e-71 2.54346776781651e-69

ADSS1 2.44984919962335 5.45970947916667 1.1561312379022 1.79162350376131e-41 1.37269217356578e-40

CD300A 12.3478353107345 30.5966578125 1.30911592429556 1.65217241090706e-44 1.5338065891571e-43

SLITRK1 19.3667501412429 8.367806875 -1.21066042086011 2.3370815991006e-41 1.77815189587871e-40

TMCO4 5.91881129943503 13.5038304166667 1.18998932431806 2.70908150241945e-45 2.63331785350181e-44

AL590004.3 0.558115489642185 1.70553390625 1.61158784436157 6.7973025073892e-26 2.33590538024214e-25

TNR 279.701953531073 92.72207546875 -1.59290556535022 4.93796792631678e-57 1.1940378880346e-55

AC060766.4 1.18522693032015 3.06697885416667 1.37165490751408 2.35794613876983e-39 1.5868986047928e-38

NECTIN2 28.1508946327684 57.3718211979167 1.02716154355086 9.15708900632948e-56 1.98688529839786e-54

MYL6 178.849645244821 381.06987125 1.09130829132298 2.36521850839568e-62 9.9505063718592e-61

AC002401.4 0.141221139359699 2.14046067708333 3.92189336510784 3.16426185893594e-38 2.00202277192629e-37

DDOST 119.794021421846 241.843138333333 1.01351569653908 2.73299053460623e-64 1.39999887027556e-62

COL6A1 97.9601959039548 293.97548296875 1.58542827771838 7.88397140556166e-37 4.64372658493567e-36

LAPTM5 102.315604190207 254.17745875 1.31280990550743 2.52613019088135e-41 1.91884766746073e-40

PHYHIPL 295.594968691149 125.29275578125 -1.23831871080137 2.19950039805622e-63 1.00738201728708e-61

CTXN3 2.95926694915254 0.4540665625 -2.70426413923933 2.01198982116113e-11 3.66829598239937e-11

FOXD3-AS1 2.85923201506591 14.0373972916667 2.29557586812918 1.22674154615385e-50 1.76012801317368e-49

HSPB6 6.42854830508475 30.9363921354167 2.26674006656486 3.68536572913147e-60 1.21505985342553e-58

SNHG18 2.40748140301318 10.7953852083333 2.16481816462322 7.17858470135279e-51 1.05189741939839e-49

NUDT16P1 0.876827071563088 2.21271328125 1.33545227531929 3.524201948832e-51 5.31057822030235e-50

IGHG1 14.9418504237288 65.8675383854167 2.14020880949282 7.15059058564664e-25 2.33723381859904e-24

NKD1 36.4474347928437 14.0656403645833 -1.37364204425806 1.37190975364329e-49 1.79779153410831e-48

PIFO 7.90951652542373 30.09748234375 1.9279813937129 2.8915481050121e-48 3.47565203702004e-47

MIR34AHG 0.680581497175141 3.09805171875 2.1865213939166 1.70121429106585e-57 4.27487564086074e-56

SUMO2P21 2.48822156308851 7.74115328125 1.63743356025793 6.88366037711418e-26 2.36470838190353e-25

PDZD4 263.71433559322 114.562892916667 -1.20283616989607 7.569612927944e-55 1.50220867017202e-53

RPLP0P6 18.5250609698682 86.8848128125 2.22962572984496 2.33926602437016e-20 6.30506620135717e-20

LRRC66 0.736200047080979 2.83263447916667 1.94397470244278 4.00372793790121e-38 2.51773151860916e-37

S100A3 4.1055959039548 21.0195238541667 2.3560664476328 1.59437747432135e-61 6.06287303374346e-60

CYP2E1 2.02340776836158 0.918968385416667 -1.13869995358603 2.28131421337674e-41 1.73643216527796e-40

AC005400.1 0.536804566854991 2.4463484375 2.1881610535639 7.56192521221174e-64 3.68099474662506e-62

RAP1B 4.84596374764595 11.2171589583333 1.21085180437258 2.90930888995069e-61 1.07551886299469e-59

TRIP13 3.91071313559322 11.7745086979167 1.59016324496442 3.81415646952365e-36 2.15969060751499e-35

CEP135 2.21497208097928 5.45863151041667 1.3012507958288 4.58851291998734e-49 5.78863568776968e-48

BUB1B 3.74390404896422 8.8258134375 1.23718579041241 4.16818017132377e-30 1.74173730979248e-29

PCNA 118.048872504708 292.319596145833 1.30816228360037 2.65843780886387e-58 7.2910989757852e-57

AC127502.2 1.19795974576271 3.53658203125 1.56177629406845 2.34468361308448e-51 3.57958881652758e-50

C1S 23.2913674199623 111.23936875 2.25580021619044 3.12667569159821e-59 9.34735281113645e-58

IGF2BP2 1.11706854990584 11.2279352604167 3.32930302458059 9.32273358057648e-73 2.03948506977435e-70

TYROBP 145.891324576271 380.836093229167 1.38427611862572 1.43700720599992e-47 1.64336709689843e-46

SLC26A2 5.22261690207156 11.9763777083333 1.19734684084131 2.82218101896695e-46 2.93996952648126e-45

F2RL3 0.601417655367232 1.44992375 1.26953790662253 2.22099610621767e-19 5.71460116163243e-19

GAPDHP38 0.259838983050847 2.41396 3.21571197403845 6.06272737080938e-41 4.50225301358628e-40

AL035461.2 2.27922471751412 4.66948786458333 1.03472115569078 2.20597365626762e-29 8.87304350817573e-29

IGFBP2 11.9842508474576 283.19040140625 4.56256073483719 3.91942295424982e-78 2.14357852453751e-75

CHRDL2 0.611304425612053 2.19493489583333 1.84421523166659 9.94890264270039e-08 1.53718192472799e-07

RPS6P25 2.11894331450094 5.54035375 1.38663310131022 5.46106899559308e-26 1.88646810278754e-25

CCR5 1.83417608286252 6.33002963541667 1.78708010811551 3.44161321137459e-50 4.7369946458557e-49

SLC12A7 11.9754096986817 24.2639425 1.01873897041873 1.06802847751259e-43 9.32831824299982e-43

TWIST1 2.10647782485876 11.1966552083333 2.41016318476821 3.25714183157428e-48 3.89495513557066e-47

AC012085.1 1.43607212806026 6.49201421875 2.17653794798383 4.02068911785693e-17 9.36556993508252e-17

LTBP2 3.84835974576271 10.0516819791667 1.38512135817978 9.2917343299717e-31 4.00138489730949e-30

DSC2 0.887683192090395 1.80358911458333 1.02275392204712 2.51825022796526e-16 5.67323273431233e-16

UQCRHP1 2.53740277777778 1.11246401041667 -1.18959388169744 4.21774210057825e-37 2.52101942655907e-36

KIF18A 0.996894868173258 4.44411421875 2.15638262393302 7.3287100989343e-49 9.12164419609661e-48

TMEM171 0.768621563088512 1.80270182291667 1.22981543079512 1.3976151006211e-05 1.94875920786213e-05

AC136759.1 2.94590913370998 0.775261979166667 -1.92595711295837 7.99039503109404e-54 1.46241531105506e-52

AL355974.2 5.00222834274953 27.3950365625 2.45327170854807 3.91788912488185e-63 1.75127760281678e-61

LINC01353 0.693826883239171 1.41764645833333 1.03085014304692 2.18826066283566e-20 5.90662026788055e-20

GLI1 0.772717843691149 4.51283026041667 2.54601889762839 1.5268896184343e-27 5.64127010824276e-27

HLA-B 425.675268502825 1210.74980296875 1.5080755902779 3.1198533420464e-53 5.53037873168282e-52

YBX1P2 1.47360856873823 6.11764921875 2.05362403070711 1.30802639399596e-41 1.0096617184041e-40

AL139246.5 4.64931487758945 1.84280541666667 -1.33511439287772 6.59905654373186e-18 1.59300865157333e-17

ARL11 1.63446280602637 3.83152223958333 1.22910113227771 9.35549445722919e-41 6.85983515111107e-40

EVI2B 15.7649237758945 32.4596372916667 1.04192868456477 5.60611924995714e-38 3.49818078701185e-37

GABRA1 16.8351031073446 4.04041598958333 -2.05889681558383 3.07574591908498e-20 8.21981824739655e-20

AC015912.3 1.66436064030132 4.03918291666667 1.27909540450771 3.11778985846744e-34 1.59799620777845e-33

GPR21 1.56391360640301 0.440246875 -1.82877614905867 1.20214959019536e-40 8.742265009653e-40

RUBCNL 1.16265197740113 4.67659041666667 2.00803776862847 5.08734332564974e-59 1.48507298493653e-57

FOXD3 0.805588983050847 2.30837713541667 1.51876308742413 2.33713885445946e-31 1.03548003332556e-30

PPFIA2 9.32633314500942 4.371189375 -1.09328408296031 3.5248798634432e-29 1.40263516072601e-28

H3-3A 20.4127860169492 43.0760354166667 1.07741237438466 2.1588519027594e-31 9.58315854185033e-31

AC079250.1 3.7924536252354 11.7488552083333 1.63131674532105 2.68759941419284e-20 7.21778034473077e-20

CHD5 18.2956832862524 5.66919958333333 -1.69028633177767 3.13678723495702e-28 1.19771167626336e-27

WBP1P2 0.53966972693032 1.95081140625 1.85392564691984 4.1305682903583e-24 1.31116280913644e-23

PRR29 1.12133145009416 3.13651291666667 1.48394872199681 1.01252494315244e-22 3.04019075051181e-22

LAMB1 5.37715790960452 17.1921163541667 1.67683140729717 8.68235442290151e-44 7.63710409147841e-43

ZNF816 2.3577327212806 5.02628697916667 1.09209286527275 3.09293169281112e-55 6.38324803860408e-54

AC004921.1 0.662983333333333 1.71120244791667 1.36796594327638 8.58426293704184e-36 4.76064328405288e-35

GLMP 10.2649209039548 27.2724083854167 1.40971959726547 1.08002527800064e-71 1.7616728109142e-69

BRCA2 0.693849905838041 1.8456890625 1.41146401037333 1.46023097036729e-37 8.93500426013696e-37

TNFRSF11B 1.08863785310734 12.596498125 3.53242670347 5.13259293812064e-75 1.42448605499035e-72

KDELR1 110.78713079096 239.385891770833 1.11154782405123 1.80243539029588e-75 5.58604768042532e-73

CDC25C 1.23202080979284 5.15784244791667 2.06574108057821 4.18270446742667e-38 2.62850252016894e-37

THBS4 11.712652259887 41.6364675520833 1.82977987275887 7.96312711020593e-16 1.75298151549993e-15

TUBBP1 1.57977043314501 5.49407140625 1.79816073508423 2.88265357266648e-37 1.73979043115006e-36

MTTP 1.79969152542373 6.93258354166667 1.94564345284041 3.66157557004103e-29 1.45516131064144e-28

AL353597.2 3.62799585687382 0.850378489583333 -2.09299579901282 8.11759571844504e-59 2.32583501362844e-57

PRR16 1.64375423728814 3.47243604166667 1.07895350823987 4.82173527503194e-18 1.17310175898494e-17

GNG5 6.14356643126177 21.8787392708333 1.83238129768886 8.44868033962415e-72 1.42821100832101e-69

VDAC1P8 0.869774246704331 1.771494375 1.02625398695444 6.51965342352643e-49 8.13098292491442e-48

SV2B 16.5033298493409 4.11370786458333 -2.00424589454774 8.88928834002276e-16 1.95131999389356e-15

VEPH1 11.2587484463277 4.0204090625 -1.48563225921544 6.17186287344804e-21 1.70554005248575e-20

VDAC1P1 0.566409039548023 2.69407807291667 2.24987546465983 1.07525826946685e-33 5.35612845452346e-33

ADAMTS2 1.6675643126177 4.00212640625 1.26302433254468 2.32792120079326e-12 4.43385175957704e-12

OTX1 1.03976280602637 7.0854940625 2.76861400019052 1.87210863675229e-29 7.55301803003012e-29

ZDHHC22 166.073942937853 47.6661026041667 -1.80079015759889 1.0932206857083e-70 1.45203133219613e-68

FAM86C1P 1.71575903954802 3.65292322916667 1.09020447834462 1.43030305681225e-61 5.45009945520979e-60

PDK1 2.06354919962335 4.87110505208333 1.23912126138956 1.90474044137675e-35 1.03442314566007e-34

AC026620.1 1.91332707156309 0.8052521875 -1.24857093479558 2.14683196765254e-21 6.05587688690822e-21

B3GNT5 1.93273912429379 7.01076020833333 1.85892397088517 7.20278042282781e-58 1.89174720285993e-56

PRR15 0.897374529190207 2.00188088541667 1.15757399376388 1.29677784183252e-29 5.2788055973896e-29

TMEM200B 0.892765395480226 1.86054005208333 1.05936843488248 7.13233115132751e-21 1.96715659683974e-20

MYL6P5 0.17821115819209 3.0209028125 4.08332019579198 5.57767334435333e-35 2.95911086557062e-34

PTER 12.7656770715631 6.34241036458333 -1.00916692770274 0.000672940233277378 0.000853802104107045

AC092343.1 0.0556618644067797 2.24883661458333 5.33634580525575 2.64972449130507e-15 5.7226047521275e-15

IGSF9 0.850783427495292 1.88947614583333 1.15112246877829 9.73006624393614e-18 2.3273977779559e-17

PLEK2 0.62498549905838 5.04459838541667 3.01284479511657 8.70305530769448e-67 6.24839048056289e-65

PLS3 48.5282525894539 114.948320885417 1.24408857452293 4.55230889297144e-47 5.04170243387754e-46

CBLN1 14.4870896892655 4.7099528125 -1.62098328994024 4.99743045796791e-61 1.77681107774213e-59

RPL12P4 3.10167881355932 17.9353826041667 2.53168731710909 1.51371004737986e-27 5.59479990678364e-27

P2RY1 12.1582572033898 25.9642319791667 1.09458910730664 2.4230373977887e-20 6.52423695509425e-20

BUB1 3.02846883239171 11.01928375 1.86336998320403 3.09417294539633e-39 2.0703902813834e-38

RHBG 0.170199576271186 3.09340484375 4.1838963054731 1.03160103468556e-25 3.51910130984737e-25

ADGRV1 17.6115546610169 7.86032614583333 -1.16386118868759 8.96081238606869e-33 4.27137416864771e-32

RNU4-2 17.5228278719397 73.2311208333333 2.06322125808757 4.65138691277521e-12 8.72956597124092e-12

FEZF1-AS1 0.342533192090395 3.55561026041667 3.37578149558322 2.60451001889973e-25 8.70745483664877e-25

LILRB1 4.97730513182674 10.8667023958333 1.12647746853339 1.56655731319693e-33 7.7432571076281e-33

IGLV7-46 0.244967231638418 4.34504135416667 4.14870922477238 1.11125471160151e-27 4.13027810558267e-27

CNTN6 2.81578267419962 1.09851635416667 -1.357979641127 3.35221942047295e-23 1.02625156607992e-22

RASGRF2 8.77563620527307 3.7099025 -1.24212244808256 8.825910521602e-24 2.76246096867849e-23

RPL39L 5.52483888888889 21.1795180208333 1.93866545904096 6.42895247239001e-34 3.24061727362679e-33

SYTL3 3.47322198681733 7.74140442708333 1.15632069860907 2.56467171828234e-44 2.34695229337894e-43

RHOJ 10.8812869114878 33.7759588020833 1.63414753229173 9.87239420555548e-53 1.67803629115452e-51

BDKRB2 0.450008239171375 2.9637490625 2.71939998034494 5.31428753475709e-47 5.86812213235203e-46

C1orf194 8.36679919962335 20.2937813541667 1.2782899920784 5.43522841220814e-11 9.73212058979397e-11

FAM110C 1.31692919020716 6.53852494791667 2.31178743346558 8.22453804111202e-31 3.55167870121872e-30

ZP3 2.45867838983051 5.24905458333333 1.09417456583554 2.10416691747301e-22 6.22743654789282e-22

CABP1 17.6720946327684 5.12889223958333 -1.78475388430145 6.06037314260669e-39 3.9666539453281e-38

TNFAIP6 1.91074924670433 27.9889269791667 3.8726458121926 2.66030570794042e-72 5.15295673324501e-70

NR2F2 9.39387175141243 19.0673389583333 1.02131171224833 1.88793479956015e-25 6.3655752670573e-25

MFSD4A 15.4606135122411 6.31817078125 -1.29101873079176 4.75051333312707e-20 1.25834466423786e-19

TTC38 13.7854115348399 29.52777203125 1.09893016777623 1.3703969925702e-61 5.26498596628985e-60

TNNT1 8.04217857815443 3.534379375 -1.18612946688985 6.95353703025933e-28 2.608979440631e-27

LILRA2 3.00195117702448 6.78951067708333 1.17740708905084 1.63079628018239e-41 1.25205024070981e-40

CD69 1.63045630885122 5.41645072916667 1.73207201569567 9.98947870718867e-48 1.15952014290774e-46

AL356056.2 1.44185555555556 0.55702 -1.3721256091906 6.35645778847829e-54 1.16796771320903e-52

DLGAP1-AS1 5.36162415254237 10.97264328125 1.03316911339492 5.14147443005897e-54 9.54148872524417e-53

EFNA4 4.38267043314501 9.58143161458333 1.12843103754286 1.57731720971071e-55 3.33677059323898e-54

EPHB4 9.01267989642185 19.3866075520833 1.10503231292768 6.10709268715359e-37 3.62006338914954e-36

AL355032.1 1.11087848399247 4.35577869791667 1.97122964473058 5.71657780554118e-26 1.9710692433532e-25

HSP90AA2P 0.964075329566855 3.90619989583333 2.01854799631982 1.40379211261264e-26 4.96642205746424e-26

SGMS2 0.683799199623352 2.03833958333333 1.57574978242822 1.94911579839157e-45 1.91766181328525e-44

AC013265.1 13.2486781073446 1.82093557291667 -2.86309663743548 1.20907825976127e-64 6.57392112288328e-63

SNCB 157.584440301318 55.10117265625 -1.51597016435722 1.12843488825998e-24 3.66583625911851e-24

RPS29P16 2.1332368173258 8.57973026041667 2.00788815862449 2.53020026633916e-36 1.44322312737965e-35

ADGRE5 7.45820536723164 30.2712665104167 2.02104860846009 2.27341213195373e-68 2.00351178169098e-66

SBSPON 1.86092005649718 5.94146838541667 1.6748034455891 6.77133625119102e-14 1.38032227133191e-13

IGHV1-2 0.640853672316384 5.495873125 3.10028181372064 2.27654504382413e-19 5.85105115271731e-19

ZNF804A 10.044238559322 3.99668453125 -1.32949259270263 1.48544717916238e-49 1.93837826642277e-48

AC006960.1 5.36112655367232 2.22661979166667 -1.26768096091855 1.84769194400449e-30 7.84604514701153e-30

NCMAP 0.435009039548023 2.291481875 2.39716358864292 3.63943788285105e-14 7.50197843161681e-14

MIR222HG 0.944397787193974 2.34144536458333 1.30993280817983 1.98757152687424e-21 5.61514623856373e-21

KCNH7 1.89527217514124 0.758247864583333 -1.32166361000652 9.3212797984683e-12 1.72466863534844e-11

OTP 0.0450178436911488 4.53487682291667 6.65442259951224 3.7118865383178e-71 5.47797858571583e-69

AF201337.1 0.992066101694915 3.93442932291667 1.98764623617293 1.40360289649105e-50 2.00923755660131e-49

HPD 1.45973323917137 4.10496427083333 1.49166491896409 2.12483237436667e-57 5.31065295717046e-56

SLC14A2 2.33521228813559 0.566129791666667 -2.04434895731954 1.09143197601222e-32 5.18395340841564e-32

NDUFA4L2 21.2478178907721 68.1286264583333 1.68094643396897 4.74080263959507e-37 2.82367793348079e-36

GAD2 12.8881380885122 4.21395708333333 -1.61279633048951 7.12668354141961e-15 1.51383002573335e-14

RAC2 6.45251633709981 20.9746123958333 1.70071035617569 1.18850053793987e-56 2.78339641095616e-55

PDGFD 2.61215287193974 11.6321366145833 2.15480488250385 1.69209227096105e-38 1.08681159020395e-37

NPC2 70.2882643126177 175.582248229167 1.32079125765589 3.41691442836014e-61 1.25073865738891e-59

CRB2 11.7507861581921 35.9354005729167 1.61264848783686 5.63920497965666e-30 2.33960322616501e-29

RPL35P1 5.30330692090395 13.3832979166667 1.33546951936363 2.94819190712667e-30 1.23918690128889e-29

HRH3 17.4434018832392 4.6483046875 -1.90790488495306 1.29116276735221e-48 1.58790817849963e-47

NAMPTP1 2.232640913371 10.1555525 2.18544559058452 7.81560908075193e-40 5.38863554709793e-39

SLC25A24 2.26516172316384 6.53118 1.52772961322218 2.41512017265043e-63 1.09802650976522e-61

PCSK2 23.9522389830508 10.9078713020833 -1.13479093740163 1.04648209285641e-29 4.28242396933648e-29

RPSAP19 0.752296468926554 3.40134635416667 2.17673269719107 2.89854615271669e-18 7.11249217600513e-18

GABRB2 7.91170536723164 1.7317671875 -2.19174370856567 7.36369073913008e-19 1.85187759391566e-18

CCK 42.2083646421846 16.5471203125 -1.35094876599613 5.29567640647918e-10 9.0985034443759e-10

SEMA3F 3.79044628060264 8.1211384375 1.09931426265525 4.96514999008197e-35 2.63942149987348e-34

LSP1P4 1.19666516007533 2.71927375 1.18420186817352 8.1348182235089e-11 1.44614670044119e-10

KCNJ12 2.22339806967985 0.854236354166667 -1.38005906483807 9.94617564688439e-41 7.27867329772868e-40

C16orf89 11.7876833804143 26.8763201041667 1.18905540335457 9.17802221242323e-19 2.29821334554282e-18

LYPLA1 18.4450988700565 44.4081016145833 1.26758537715229 1.30959097642934e-52 2.20179423207085e-51

SCG3 828.894366525424 299.399554739583 -1.46911618195884 3.41233302397431e-64 1.71030546039898e-62

ULK4 1.20549515065913 2.76394833333333 1.1971048001575 1.49331650344951e-57 3.79347272973274e-56

AC016168.2 0.631947080979284 1.97794458333333 1.64612634843307 2.38869414559804e-20 6.43455999382813e-20

H4C11 0.595383286252354 1.81302822916667 1.60651076070796 1.46046355292903e-27 5.40121713737377e-27

ERCC6L 0.600239359698682 2.17395421875 1.85671172952383 1.53813886675171e-40 1.11031413925652e-39

HMGN2P5 5.95881111111111 73.8834575 3.63215496005889 1.57899694798658e-46 1.67397420460133e-45

SDC4 62.5498010828625 164.4839375 1.39486950448559 1.30291244680911e-27 4.8339299577844e-27

HCFC1-AS1 1.2307200094162 2.8491103125 1.21100889739624 0.000141415499693625 0.000186762870511574

ADPRH 3.51693140301318 9.45410651041667 1.42662392247682 2.95776070232224e-67 2.24488001059927e-65

CAMKV 49.0719378531073 17.99027625 -1.44768090453704 9.18801144198501e-24 2.87434510033162e-23

LDHBP2 0.585168314500942 1.96068614583333 1.74443505843295 1.8717686640305e-20 5.07221485101241e-20

PROS1 16.1798142184557 43.5192309375 1.42745801941662 6.6106610645466e-45 6.27810227248437e-44

LGALS1 224.33592405838 1037.54475802083 2.20944100318898 5.56628711417089e-70 6.59268209477756e-68

AC009362.1 0.266711629001883 1.82548078125 2.77492384518522 3.58946860464299e-36 2.03494416778465e-35

SFN 1.57861638418079 4.08969411458333 1.37333231408224 1.92816814036654e-37 1.17439523649249e-36

TSPAN31 26.3743241996234 119.71890828125 2.18244499542253 2.05175559477757e-34 1.06008479027554e-33

LGALS3BP 182.036746045198 421.50637109375 1.21132473465788 6.37328888960729e-64 3.14353599210206e-62

HNRNPA3P5 0.736201412429379 2.924153125 1.98984643814312 1.19774905282091e-35 6.58939160864048e-35

IL1RAP 4.76883921845574 21.3644121875 2.16349957562747 4.71390562800148e-43 3.97889583080742e-42

AURKB 5.13168860640301 20.7253820833333 2.01389316411606 4.03480736789445e-38 2.53641795152121e-37

H3P36 0.832662146892655 3.08290015625 1.88848502148564 6.52416764869782e-30 2.70193535473354e-29

TWIST2 0.670003154425612 2.27133140625 1.76129842966008 8.01782596561653e-13 1.55986057575475e-12

F2RL2 0.492270338983051 5.2114553125 3.40416358526188 4.24731677441736e-59 1.24966543386536e-57

KIF2C 4.50446878531073 20.54092953125 2.18907258559325 4.20243037378401e-49 5.31955022467758e-48

OIP5 0.994200047080979 3.42725661458333 1.78544614045102 2.85921707389039e-47 3.20863859317995e-46

MIR124-2 3.48137015065913 1.20976052083333 -1.52493372842465 9.92030848234871e-27 3.52441987446072e-26

AC092535.5 1.10416384180791 2.35780567708333 1.09449055793628 4.64366919782604e-28 1.75613237204749e-27

HOXB-AS1 0.206169632768362 2.11230947916667 3.3569174672756 1.75907212721031e-45 1.73711875759297e-44

YBX1P1 3.2305950094162 17.8443730208333 2.46559740242821 3.79251199223844e-37 2.27562957391655e-36

FBLN5 9.7192934086629 33.3219698958333 1.77755035204934 1.71315336815137e-50 2.4206752948917e-49

RPSAP58 8.8976777306968 52.3850568229167 2.55765458143325 6.12063464614399e-22 1.76701135297388e-21

RNASE3 2.04492132768362 5.86943916666667 1.52117731754152 1.5611917515713e-30 6.65728957690672e-30

FAM27B 0.332355743879473 1.91209302083333 2.52435251791327 2.60457995367532e-35 1.40423787296586e-34

MDFIC 8.92585014124294 21.4449947395833 1.26457947201423 4.36713694806483e-26 1.51364234015406e-25

E2F2 2.09429868173258 6.03737833333333 1.52745500152548 6.87711285119118e-25 2.24903119007914e-24

NRXN3 8.04801106403013 3.44866041666667 -1.22259621226494 1.65602865561246e-17 3.91580866896914e-17

COL6A2 15.809411393597 165.503413385417 3.38800541159357 3.59763163859165e-57 8.76777554649378e-56

RCAN1 39.34223173258 116.6528059375 1.56807030640834 1.6623375853143e-54 3.20988238825747e-53

MREG 9.79307269303201 26.3764000520833 1.429414175011 2.09130121818827e-45 2.04996026105487e-44

PVALB 10.9448860640301 3.90858588541667 -1.48553829184964 8.18044947177138e-17 1.8819183215092e-16

TUBA1C 6.63689962335217 41.4730827083333 2.64359392709235 1.87371253678759e-71 2.92787265727439e-69

SPRED3 4.20810536723164 11.1091308333333 1.40050321220441 5.21290017620884e-32 2.39283828132815e-31

TPM4 34.2480241996234 89.791831875 1.39056345273562 3.8684312188202e-59 1.14180124625336e-57

TDH 3.93047325800377 1.86345776041667 -1.07672091666388 1.33808017277737e-34 6.96378416255113e-34

PI4K2B 6.74979778719397 15.8287528645833 1.22963140394601 1.03616026519062e-68 9.63370006560979e-67

TMEFF2 31.9199480696799 14.0481884375 -1.18407420252881 1.17006062575671e-45 1.1684896528435e-44

PAMR1 17.5076285781544 43.6278386979167 1.31726532222375 1.93851480523669e-27 7.12948631395891e-27

CASP3 30.4850548964218 67.2809169270833 1.14209522331127 2.21757856660325e-56 5.12246875105434e-55

SYT14 3.6989697740113 1.82924364583333 -1.01587626414834 2.18856295623537e-27 8.02056132660558e-27

PIWIL4 1.55475032956685 3.52681953125 1.18168483139462 7.23434835173646e-44 6.4027942694212e-43

HOXA10 0.436238935969868 5.13534109375 3.55726965795507 6.32313189197396e-54 1.16299344739125e-52

CST7 1.75616426553672 5.92018541666667 1.7532145650142 4.15483653897136e-51 6.23567757132842e-50

SYP 143.690638229755 60.2177410416667 -1.25470557588391 1.33291597502137e-41 1.02759421871983e-40

MSC 2.41892631826742 4.92959130208333 1.02710121719991 3.89706545561612e-07 5.85914716584587e-07

ZNF90 0.722531120527307 1.46683640625 1.02157634551839 2.53700058577882e-25 8.49244390505079e-25

SRP14P2 2.13085739171375 5.53240197916667 1.37647194163053 8.51366378784895e-31 3.67227042762819e-30

NKX6-1 0.51611972693032 2.2745728125 2.13981793928379 7.33684020287871e-36 4.08224247673637e-35

LSP1 1.97709905838041 11.1627690104167 2.49723788193981 1.32429632096574e-69 1.46579107668797e-67

FOXD2-AS1 1.36981445386064 4.04985744791667 1.56389063870731 2.04455388572167e-37 1.24407822767512e-36

NAT2 0.643347787193974 1.52289869791667 1.24314921838005 8.67561203090494e-23 2.61505925943714e-22

LZTS1 13.8416367231638 33.15266703125 1.26011038893633 4.6895720842813e-19 1.18999171543683e-18

TMEM176A 16.7969467043315 61.1913823958333 1.86512948365679 4.26170766313359e-47 4.72830871097668e-46

AC007182.2 1.12945612052731 2.94596890625 1.38311398028751 4.96454876850462e-19 1.25822249353064e-18

HLA-DOB 0.823536911487759 1.71967911458333 1.06223416967944 1.75908152149827e-24 5.66605246704665e-24

HOXC10 0.520161723163842 9.42452854166667 4.17938830439025 2.33718676089009e-69 2.48342787535721e-67

NCAPG 2.5336963747646 10.3666315625 2.03263163955187 4.15170684899232e-40 2.91216106665282e-39

LINC01579 0.609792325800377 6.62058510416667 3.44056882366654 1.37042895559958e-57 3.50042945458437e-56

FAM222A-AS1 4.63464910546139 1.82903442708333 -1.34137788437326 2.35527144738388e-41 1.79125859157886e-40

AC020915.2 2.61958997175141 5.55490182291667 1.08442040042873 9.64967043636415e-66 6.06201424879025e-64

SAMD9L 7.609565913371 24.6561202604167 1.69605974088789 9.06665811612249e-53 1.54532087689549e-51

OTOGL 1.55392245762712 0.692707291666667 -1.16559674822239 2.25694873984132e-41 1.71861434130477e-40

PIK3CD-AS2 0.75851972693032 4.66157833333333 2.61955990382727 1.11029883580448e-59 3.50526432118579e-58

CLSPN 1.05188634651601 3.707171875 1.81734017039966 1.03441698757577e-35 5.70940453664929e-35

TRIM67 30.6473683145009 5.22537677083333 -2.55215622498722 8.48305584774277e-37 4.98869144493285e-36

AC010343.1 6.79846949152542 37.51417234375 2.46415382847369 3.04682563002771e-27 1.11002591282064e-26

PDGFRL 2.02574016007533 6.25555807291667 1.62668946354896 1.31176570682598e-37 8.04229585177352e-37

DNALI1 20.8580745291902 54.6772546875 1.39033482336057 4.56298648462074e-55 9.25286081586943e-54

HAPLN3 3.99935014124294 8.95013932291667 1.16214454639978 3.47680588235869e-36 1.97287779623008e-35

TCF7 1.44540018832392 3.87100125 1.42123778611489 1.62834489359938e-58 4.58773837825462e-57

RUNDC3A 98.0626435028249 37.0827408854167 -1.40295577190524 2.6952686050065e-59 8.12293674393774e-58

IFI35 36.1980742937853 77.2402603125 1.09344007719581 1.02964310788107e-40 7.5230701733e-40

AC002398.1 0.829500612052731 2.15098979166667 1.37468572712715 3.43881049627024e-50 4.73664305023297e-49

NAPSB 16.693699905838 61.9008003645833 1.8906543216566 9.70212180329015e-51 1.40836030392022e-49

ATP5MFP2 0.935724576271186 5.217298125 2.47914702308553 1.12964057862215e-34 5.90046813468508e-34

POLE2 2.05242763653484 5.35373458333333 1.38321426064257 5.76154376194595e-42 4.54158144355171e-41

GINS4 1.23509896421846 3.25742765625 1.39910649392189 4.56041216697051e-52 7.32937461061509e-51

CAPZA1 53.2706760828625 110.427503854167 1.05168604864999 3.44214482602035e-69 3.49763295299718e-67

RBBP4P1 0.857729331450094 2.7206265625 1.6653445816259 1.44123469324483e-44 1.343346321849e-43

TRIM6 0.725858192090395 2.4209671875 1.73782389844345 3.7243236051773e-53 6.55192028744294e-52

GBP1P1 1.34921501883239 9.54399375 2.82247281530065 1.54378844395351e-50 2.19806631817117e-49

TRIM5 5.4498049905838 12.8475831770833 1.23722048016431 1.29903310352167e-47 1.49477231188029e-46

SDC1 4.13785593220339 21.064729375 2.34787406042771 1.10568303171058e-44 1.03629919227108e-43

CDC45 3.43394731638418 12.41666265625 1.85433764808564 9.52919174162556e-36 5.2721011733272e-35

CA13 1.79578126177024 4.27550979166667 1.25148481959573 7.95757398374889e-60 2.53809756823003e-58

YBX1P10 10.1285196798493 23.0316525 1.18519459249816 4.58632956281659e-33 2.21283856306628e-32

SEC61G 50.8190707627119 780.186867604167 3.94037781382085 3.80204275619988e-72 7.214182148116e-70

METTL24 0.65765527306968 1.44273213541667 1.13340000551489 1.94363155875952e-12 3.71447367267557e-12

HLA-A 423.155110546139 1147.21029114583 1.43887137538917 1.87139533752637e-57 4.68983777645591e-56

P3H2 3.30173625235405 12.01986671875 1.86412411361696 8.92986605936347e-62 3.50318268721231e-60

DGKB 18.0548251883239 7.51870333333333 -1.26382866880218 6.58791282168248e-47 7.21450170313226e-46

RNA5SP311 1.72877161016949 0.790417552083333 -1.12906039660717 4.84523565921154e-17 1.12424703123332e-16

RPL41P2 4.49774053672316 16.13963703125 1.84333579061326 1.34230633286599e-31 6.02903049749833e-31

PHLDA2 1.02428097928437 9.85398625 3.26609593089439 1.64064273169532e-45 1.62534638230551e-44

AL603825.1 4.70389891713748 10.926421875 1.21589206914461 5.15967928227699e-22 1.49749081089341e-21

GMFG 22.6962202448211 54.27910421875 1.25794485605631 1.09139606723047e-52 1.85000089974025e-51

CYP2S1 1.5532266007533 3.85901052083333 1.3129626567623 2.56728698883812e-52 4.21347763084244e-51

AC099560.2 4.88783945386064 27.1274981770833 2.47248719753088 7.73296786166112e-27 2.76368513141627e-26

CELF4 23.9205555084746 7.57241317708333 -1.65942585697842 8.36835560062106e-34 4.18417780031053e-33

ARHGEF19 1.34391812617702 2.88980739583333 1.10452809174277 2.91172708089858e-41 2.1991699865682e-40

TAFA1 7.94017391713748 2.4527546875 -1.69476765787616 3.83402414704824e-24 1.21911215824832e-23

TPT1P4 2.0695777306968 11.2509341145833 2.44263644642983 8.53299498437411e-43 7.09302824025197e-42

APOC1 82.3866881355932 208.371037708333 1.33867161204002 2.4726150301261e-36 1.41167566733788e-35

FABP5 3.36016676082863 73.4755161458333 4.45065884943416 3.56121695765522e-71 5.2976663462079e-69

LRMDA 1.58629185499058 3.36846192708333 1.0864317622859 2.16619182069983e-46 2.27315671026599e-45

PLBD1 2.72173766478343 11.1716634375 2.03724409190078 2.05988838889826e-67 1.62303494032047e-65

FP671120.7 1.53474745762712 11.3678667708333 2.88888836661914 1.9017154734444e-17 4.48875339282795e-17

ADAM9 18.5583201977401 37.3038720833333 1.00725925658068 6.27519919921029e-34 3.16483127500177e-33

RPL10P9 25.3966082862524 51.6976257291667 1.02546218656478 8.31157394300235e-14 1.68855804075307e-13

MIR4530 1.11596393596987 2.801175 1.32774171175539 8.59736133972575e-13 1.66998782108221e-12

DCAF12L2 2.19848370998117 0.926859166666667 -1.24608679506395 2.25084792174744e-34 1.16165742727987e-33

S100A4 10.3582939265537 91.5889519270833 3.14438717925568 3.32131346037182e-76 1.23519647591228e-73

TPTEP1 30.6834266007533 6.47256588541667 -2.24504995540813 4.627648456332e-25 1.5276251206372e-24

FANCI 3.64752881355932 9.09980854166667 1.31891681686648 3.80352957033747e-29 1.50931967705531e-28

ZNF530 2.06660324858757 4.87569953125 1.23834777818345 7.61531959079756e-57 1.81082951139234e-55

DCTD 28.2712587570621 71.7105914583333 1.34285009724063 3.25500076176013e-69 3.32564500906207e-67

SLC35G2 7.9439299905838 17.74245296875 1.15928066844257 4.17656991233299e-39 2.76677297897513e-38

E2F1 15.4611845574388 35.60444453125 1.20340648974322 4.64878434515653e-27 1.68048493192429e-26

CXCR2 0.745957015065913 2.00620244791667 1.42730279280175 6.56000623345202e-33 3.14552129734503e-32

FBXO41 27.174479566855 12.99541171875 -1.06425006389541 4.19541226054718e-26 1.45656629919482e-25

MAST1 33.9645784839925 15.7270108333333 -1.11078645994689 7.5255135627413e-43 6.28365176017847e-42

TWF1P1 0.496018173258004 1.63600390625 1.72171130840115 3.32817284048064e-35 1.7855560868072e-34

LENG9 1.61152650659134 3.74887692708333 1.21803054554653 2.79243784142638e-48 3.36522240190042e-47

AC138207.4 1.37100979284369 2.94404161458333 1.10255918827473 3.22533122982308e-38 2.03996715029116e-37

MYCBP 5.68246209981168 13.45715296875 1.24378515993393 6.33895098715646e-70 7.46030339279585e-68

LRRC2 2.32815131826742 6.04441505208333 1.37641790019796 4.79668938219613e-18 1.1671609403551e-17

CALN1 27.5999947740113 7.73282796875 -1.83559997077034 7.94510316466697e-51 1.15964829942686e-49

BRIP1 0.774001035781544 2.40567369791667 1.63603356860284 7.54982968413459e-33 3.61175927389973e-32

RSAD2 7.84180277777778 25.2961261979167 1.68965920669745 5.64828508577448e-26 1.94788318193577e-25

ADAMTS3 0.739124717514124 4.23933859375 2.51994947257163 5.0339688889362e-33 2.42443183640929e-32

ZDHHC23 2.26667678907721 5.89639869791667 1.37925539034651 5.09957227177574e-25 1.6801301628928e-24

DRAXIN 4.99718055555556 18.0025929166667 1.84901846230165 1.83851695875675e-36 1.05941192587796e-35

VIM 191.127388841808 911.11696078125 2.25310173228599 3.2815860102246e-74 8.47515164723978e-72

FAM111B 2.33567217514124 8.39567296875 1.84580817337039 1.19734182882531e-32 5.6782890351968e-32

TGFBI 18.0284255178908 80.34456859375 2.15592709203978 1.15749770053339e-40 8.42743529421237e-40

INSYN2B 11.0564965630885 4.24429286458333 -1.38129820218857 4.35358328139697e-48 5.17945496593581e-47

GABRG2 7.54506614877589 1.7749721875 -2.08773713030934 2.23077311703159e-38 1.42205094656162e-37

RPL26P19 7.29871633709981 23.8005896354167 1.70528265808422 5.93949169047605e-23 1.80082908828309e-22

VSNL1 129.535550047081 34.7190408333333 -1.89954909215446 2.15127969455408e-11 3.9138094041907e-11

AC009041.1 2.72864693973635 0.7769040625 -1.81237737528147 1.40145601235421e-58 3.97257233989734e-57

MIR320E 1.16045701506591 3.08619541666667 1.41113633142639 6.55041765210958e-11 1.16973990435972e-10

GPR84 1.47202678907721 5.43532145833333 1.88456143541392 1.34041991918385e-44 1.25063263408047e-43

PRPS2 10.7168639830508 28.1090663541667 1.39115273559526 4.85251321284402e-50 6.60076687584744e-49

FGL2 6.75027622410546 17.1802421875 1.34773192992832 1.16019032897552e-45 1.15925519437398e-44

LINC00601 0.32948615819209 2.0244521875 2.61924180697928 1.91628817336141e-65 1.16448949619789e-63

WARS1 35.6611094161959 77.6113710416667 1.12191645499419 4.26456533540379e-39 2.82405955882598e-38

HLA-DQB1-AS1 0.753218220338983 2.18740739583333 1.53808213730091 1.07499682101489e-27 3.99871291993836e-27

RPL5P34 2.12228601694915 16.624516875 2.96962141099581 2.89003502106043e-33 1.40865533988516e-32

VAMP5 78.7649223634652 250.107665625 1.66692409631667 9.65855226327107e-64 4.65286993097217e-62

RPS15P4 27.8930148775895 67.6010585416667 1.27714195770179 5.20837786961688e-30 2.16472477616285e-29

LAT2 13.4132556497175 27.8681279166667 1.05495664149345 1.68317905908371e-43 1.45643157764828e-42

MYL12A 23.3693676082863 82.1127825520833 1.8129881304981 2.37430959621664e-80 2.00683122462038e-77

SCPEP1 24.805277165725 50.370780625 1.02194001194132 7.3374701158643e-64 3.59053307380254e-62

FSTL4 4.10786177024482 1.65611088541667 -1.31058836354823 7.00199040538292e-24 2.20085199391064e-23

ETNK2 5.45959896421846 14.7256770833333 1.43146708363839 4.13091075359515e-20 1.09781742837076e-19

NNMT 2.94917641242938 106.477285572917 5.17408976453367 2.44380003798801e-78 1.37704429413294e-75

AL158050.1 0.361258286252354 2.44651421875 2.7596250830232 4.92243989565522e-42 3.90498164930498e-41

SDHDP6 0.992116290018832 2.03828276041667 1.03877306316407 9.76652652008298e-23 2.9329547907129e-22

TMEM130 63.4472236346516 20.036836875 -1.66290226029508 5.98894855500048e-21 1.65573146565914e-20

AC068522.1 0.734653389830508 1.78464489583333 1.28050138828324 1.07883276662309e-23 3.36479290428654e-23

ITGB3BP 4.01062532956685 9.74720390625 1.2811612284439 4.18303711612563e-65 2.43835658853781e-63

MILR1 7.11717198681733 15.7590797916667 1.14680729072587 8.97680063999576e-36 4.97240416743286e-35

RBP1 9.28378639359699 95.2790081770833 3.35937316143736 2.83632383745724e-57 7.01348959541455e-56

STRA6 1.16531774952919 2.5411596875 1.12476364670501 1.13641164568384e-32 5.39345955882874e-32

SMC4 4.537409086629 14.1466047916667 1.64051520333968 3.91021899603516e-47 4.34871544445417e-46

CDT1 10.3837009887006 21.29782515625 1.03638537117257 7.34576914401766e-18 1.76798572654683e-17

PRF1 1.55029693973635 6.53718447916667 2.07612483864132 1.95847888202293e-48 2.3818126102823e-47

AL441992.1 3.05482518832392 7.61817734375 1.31835604831707 1.02588569987422e-63 4.92928800753517e-62

RTP4 7.69165889830508 15.53439953125 1.01409978634236 9.15727760586513e-36 5.07086292677374e-35

OLFML2B 15.9197195386064 37.3994628125 1.23220262828723 5.45039361807908e-30 2.26329162213985e-29

KCNC2 7.59616257062147 1.76292416666667 -2.10730036438753 7.96763536998059e-27 2.84481911875555e-26

SEC24D 4.25662184557439 11.6375067708333 1.45100117395502 3.84356610441462e-59 1.13626568698871e-57

NPTX1 46.841013747646 23.0216866666667 -1.02477876411127 1.26646505341001e-08 2.03507757242993e-08

CNTNAP2 15.7194686440678 4.59259364583333 -1.77517140765468 4.15280849314086e-52 6.70907679669455e-51

TMEM155 8.78960244821092 2.97789130208333 -1.56150681991846 2.10583048394779e-09 3.51696765304555e-09

ULBP2 1.6239868173258 5.2933465625 1.70464019071646 7.64596543011705e-38 4.73764502409285e-37

PTP4A2P1 1.09586087570621 2.59057380208333 1.24120703115407 2.40958374497718e-35 1.30099331410716e-34

RN7SL417P 3.50015687382298 1.71622932291667 -1.02817724484887 6.47934138924326e-25 2.12230673124852e-24

CSF2RB 2.11353959510358 4.86626104166667 1.20315257298019 5.10295083130474e-30 2.122804713828e-29

MIR4754 0.738306403013183 1.69489390625 1.19890339354723 7.14268337677575e-12 1.32804916899455e-11

PACSIN1 77.3368616290019 19.5302061458333 -1.98544904299193 4.64690450943382e-20 1.23142638382388e-19

AC092675.1 2.29445560263654 5.33081901041667 1.21620531008948 1.21740104087642e-29 4.96437990243356e-29

MT-TL2 2.69443163841808 1.24501026041667 -1.11382335203182 3.55792870946976e-19 9.06918222790818e-19

SERPINA1 9.27180823917137 49.97749578125 2.43035598047413 2.12320275282449e-65 1.28602459898278e-63

CHST6 5.36062730696799 18.0640209895833 1.75264532601151 3.43142751063543e-50 4.7299773580627e-49

FAM183A 0.503614406779661 14.2824195833333 4.82577704218802 8.96772960881274e-50 1.19692300379021e-48

GDF10 14.7234569679849 6.39026609375 -1.20416853414251 2.10713284022486e-39 1.4237736613909e-38

ELN 34.5329509887006 92.2578206770833 1.41769758939304 2.95102437065285e-41 2.22704132192734e-40

LTBR 5.87981478342749 15.2441002083333 1.37440838156145 6.41073280933116e-61 2.25771925358926e-59

TCERG1L 1.48905583804143 0.512903020833333 -1.53763988103297 3.81158648400677e-22 1.11196188688588e-21

NDUFB1P1 0.514053813559322 3.58707729166667 2.80281753124013 1.86671068572118e-33 9.19266027568468e-33

MT-TG 1.58931483050847 0.7226440625 -1.13704780984718 1.59851337847534e-16 3.63234371386585e-16

PEAK3 1.39238314500942 3.47403536458333 1.31905618504625 1.22266265949103e-47 1.40776545840469e-46

SYN3 8.07090428436912 3.26843619791667 -1.30412979066416 1.59852998533604e-38 1.02817935238062e-37

RAP2A 188.219442843691 92.1115909375 -1.03096104887444 8.02733234363766e-58 2.09352377180845e-56

PHACTR1 7.90361186440678 3.81860578125 -1.04946611073447 3.12088348174409e-32 1.44973340851939e-31

NAT1 1.15851520715631 3.00820536458333 1.37662607873684 8.33637805712855e-56 1.81942429544959e-54

FBP1 2.96150131826742 9.164036875 1.62965453556151 5.08060831310969e-57 1.22693391665292e-55

AC091057.1 1.09441209981168 2.5455940625 1.21784628990518 1.6867166881937e-39 1.14427204731711e-38

THEMIS2 13.0887184557439 28.04614375 1.09947857191787 5.84746388623136e-36 3.27510816158049e-35

BARX1 0.983696892655367 2.03545151041667 1.04906310293255 6.58777182395238e-28 2.47523978715689e-27

ACAA2 11.8539151129944 33.2532028125 1.48812967041249 3.28867877551896e-65 1.9600314689351e-63

PGK1 171.760797551789 349.439617291667 1.02464238600741 4.14926637712952e-30 1.73422360716393e-29

ZNF600 0.733826600753296 1.86062505208333 1.34227624941186 2.7991503185032e-67 2.14198354619617e-65

CFAP43 1.22183771186441 2.67713630208333 1.13163791852762 2.545287455933e-07 3.8542036028562e-07

CFAP45 0.249273116760829 3.97315921875 3.99448740186674 1.53055454938056e-59 4.75136257858623e-58

AC051618.1 1.48656384180791 0.562028958333333 -1.40326505059913 3.96898267928264e-37 2.37844772546763e-36

ACTBP2 1.70947419962335 9.92391770833333 2.53735712313841 6.27336227727254e-53 1.08514578182217e-51

RASSF9 0.56871247645951 4.54913453125 2.99982074027557 1.58593593483785e-10 2.78321313871262e-10

MARCHF11 2.20709618644068 0.49704390625 -2.15070430177728 4.38868052250118e-45 4.21308798739853e-44

KHDRBS2 5.49652594161959 1.47797234375 -1.89490078314537 8.81935791527891e-59 2.5230147759171e-57

ZDHHC1 7.20911614877589 16.0990105729167 1.15907772635975 2.77823607644947e-47 3.12152869133401e-46

SYT5 18.4081016007533 8.87337036458333 -1.0527867600927 2.73882524466044e-10 4.76813551394634e-10

PLAAT4 76.8079588512241 170.591045052083 1.15121420024161 3.43749485129861e-32 1.59163886354327e-31

IGHV1-3 0.31893615819209 4.95101692708333 3.95638530963316 5.66893048335576e-18 1.37418540396298e-17

RAD51AP1 5.1595327212806 14.382015 1.47895350208379 2.52865808270192e-42 2.04436508903662e-41

UBBP1 1.87625404896422 6.94409786458333 1.8879320949289 1.05389660177137e-45 1.057023048001e-44

LUM 5.27113950094162 46.23852078125 3.13290846657631 6.70271155220138e-42 5.26783268441186e-41

P3H1 7.52784472693032 17.0074438020833 1.17585754626135 4.33386336008398e-68 3.73093468429452e-66

CENPS 0.923475988700565 2.25619739583333 1.28874694007211 2.73178524401392e-60 9.15271110134033e-59

LINC02774 1.69511949152542 0.805275052083333 -1.07383343107257 5.49286284830281e-31 2.39223489798695e-30

ELFN2 3.84312890772128 1.24903447916667 -1.62146806605392 1.64124440086073e-57 4.14096874274156e-56

AL049839.2 0.872670574387947 2.66416510416667 1.61017443535844 2.33808046826093e-39 1.5740987077231e-38

TAP1 30.5509021186441 71.559914375 1.22793668001419 6.03030719946181e-37 3.57568757570129e-36

HLA-DRB5 89.0581443032015 296.737061875 1.73636567194724 1.0844664024331e-35 5.9767791207005e-35

BNIP3P1 0.600052354048964 1.94622213541667 1.69751609878107 9.32356216949175e-23 2.80491245011647e-22

HOXA7 0.175269679849341 3.40981458333333 4.28204494252314 3.44410846017725e-60 1.13955866222413e-58

LYPD6 4.68901666666667 10.350066875 1.14228277735224 2.03560056603607e-22 6.03315150230168e-22

LINC01952 0.79120983992467 1.82643203125 1.20689579284058 1.98979056050114e-19 5.12964861673627e-19

AUNIP 1.53803747645951 3.32204557291667 1.11098120776819 1.89528910955198e-26 6.65839807143757e-26

RRAS 40.7965184086629 84.58419109375 1.05194200950116 1.23866702520567e-50 1.77586841431761e-49

RPL26P36 0.349991713747646 2.38149067708333 2.76647222998597 1.91021525307802e-29 7.70007644287574e-29

EPHA2 5.19920188323917 14.2534708854167 1.4549511942511 1.63246010130514e-42 1.33313990266882e-41

EFNB2 6.47845668549906 16.6441227604167 1.36129075762378 2.01165254261758e-46 2.11815849546851e-45

TOMM20P4 0.471277730696799 1.93015260416667 2.03406549982599 1.41504164358528e-18 3.51116885007583e-18

MEST 35.0913626647834 108.511391458333 1.62865862729695 7.09129807307393e-41 5.24304921148349e-40

GNS 52.0191496233522 111.039489479167 1.09395811972894 2.71073495557459e-53 4.82355181807746e-52

C2orf50 0.86458950094162 2.67915307291667 1.63168978942407 1.17826884491887e-19 3.07119556647973e-19

CRY2 69.9060879943503 29.8020608333333 -1.23000600525792 1.0548755492498e-76 4.3589801862889e-74

BTBD17 98.9104648775894 44.1123210416667 -1.16494149621065 4.51295487592954e-34 2.2941059572966e-33

FLJ16779 112.663063747646 44.2501384375 -1.34826073590507 1.64299430187723e-44 1.52604790426609e-43

SPTBN2 44.0246782015066 16.0394918229167 -1.45668402491002 2.44760389877251e-63 1.11007791457743e-61

AC012462.3 0.633129425612053 1.623161875 1.35823453084435 1.67361065310433e-13 3.35461788234074e-13

COL5A2 8.75813822975518 51.04846 2.5431713142332 2.82224717260624e-60 9.43879247744839e-59

AC107373.2 2.45112683615819 1.04074322916667 -1.23583096725123 1.70789133979915e-08 2.72954357228751e-08

SUB1P1 2.33994256120527 5.59476489583333 1.25760439102822 7.98829305554414e-42 6.23603313886832e-41

ST8SIA4 3.90836935028249 11.31396859375 1.53346635434277 2.12066868371412e-49 2.74226941402393e-48

RPL7AP66 2.2733854519774 5.39716963541667 1.24736071905799 8.10555746677628e-19 2.03541986623504e-18

DTYMK 30.0376030602637 67.6523416666667 1.17137017810399 5.40812635493999e-60 1.75504554223576e-58

CHIC2 23.6777300847458 64.3570058333333 1.4425664265904 3.18270905660173e-52 5.1823533193966e-51

CD48 0.548351506591337 2.69658401041667 2.29796008354102 1.13865558069138e-52 1.92512831617877e-51

AL160270.1 1.54071040489642 0.667201666666667 -1.20740091765557 3.28510979002601e-39 2.19420318051486e-38

CCDC85A 3.83034948210923 1.43238098958333 -1.41906075344189 1.07370399392835e-47 1.23932500106131e-46

RHOC 68.5112872881356 162.929551510417 1.24983469995002 4.35826894443468e-71 6.33140711107522e-69

SLC37A2 5.36979270244821 10.7831341145833 1.00583825786384 1.31654786910111e-32 6.22773025335414e-32

IGLV3-10 0.437432297551789 3.21354989583333 2.87703622288935 1.12194438023898e-16 2.5676991692977e-16

AC120036.4 13.7289149246704 6.39625994791667 -1.10191712834955 1.48661755314221e-69 1.63571913613488e-67

HOXD8 2.86630579096045 6.91437802083333 1.27040694906189 5.76064088303391e-16 1.27598710208476e-15

AL512625.2 10.5376349340866 5.2125109375 -1.01550069404607 4.96191669068291e-32 2.28100966287389e-31

TRIP10 11.3359366290019 25.2196248958333 1.15364321956695 7.60336252939834e-66 4.87532849083318e-64

TMEM14DP 0.802793220338983 3.88897182291667 2.27628844375935 2.2077656780553e-29 8.87833105178164e-29

TP53I13 12.7320348399247 27.6805213020833 1.12040810335567 2.88903297559302e-71 4.36760716919937e-69

AC008429.2 0.753867702448211 1.59757880208333 1.08350382546055 3.73958716336214e-22 1.09147109249284e-21

SHISAL2A 0.439051789077213 1.69953140625 1.95267399182325 2.9003253793008e-69 2.99619724600547e-67

WIPF3 6.35924510357815 13.8108739583333 1.11887719624955 6.4102916219838e-22 1.84977300916804e-21

OSM 3.1242897834275 9.60411041666667 1.62012371784571 1.14129740602698e-23 3.55544065422545e-23

REEP4 14.5886769303202 32.67142515625 1.16318033882956 3.84816521962348e-74 9.80227839162995e-72

AC073325.2 1.65622048022599 5.72162057291667 1.78852908825692 6.8074616140585e-27 2.44042314851393e-26

TMEM196 7.2299040960452 2.60530229166667 -1.47252573283925 1.80022908554627e-22 5.35261590114053e-22

CFD 7.12031497175141 25.7065647916667 1.8521238671973 1.17135679936244e-41 9.08314415518956e-41

SCN3B 44.1567463276836 13.1838661458333 -1.74386037141792 2.22638023614243e-47 2.51363330243282e-46

PCED1B 3.66607165725047 8.2686290625 1.17341316544043 5.45328406410149e-47 6.01446127947611e-46

GCK 1.22130885122411 4.35730822916667 1.83500908782104 2.11497394795704e-45 2.07207273773768e-44

SNORD99 7.09987537664783 14.5584945833333 1.03599557533029 8.25576919293539e-20 2.16433142735984e-19

NDE1 6.9322668079096 14.5717180208333 1.071771895807 9.83520179321122e-57 2.31500730816155e-55

OR7E14P 0.894094161958569 1.81958854166667 1.0251135721678 1.20831820930952e-17 2.87654296531949e-17

WDR45P1 1.17321822033898 2.97534994791667 1.34258798025614 1.24318480271189e-35 6.82723609167974e-35

ADRA1D 1.84215969868173 4.71271708333333 1.35516093967786 2.55324037441226e-17 5.9961486186153e-17

MT1XP1 8.64667777777778 18.7454049479167 1.11631915905134 3.61477735571257e-23 1.10517568118177e-22

MTMR11 7.60259260828625 19.3555857291667 1.34818657626107 1.41625310199826e-61 5.41877087071145e-60

SPTB 2.25123728813559 1.06506270833333 -1.07977975444525 5.44377903895509e-13 1.06599695902875e-12

GRIK1 4.59236756120527 16.98585515625 1.88702383149189 7.53507078441106e-22 2.1652703019027e-21

LRRC46 1.71138870056497 4.68527994791667 1.45296778189861 2.66582724840875e-36 1.51872113002943e-35

IGHM 4.29669298493409 25.0485536458333 2.54342870183884 5.11539520507588e-22 1.48510185540025e-21

FCER1G 74.8614258474576 236.152864479167 1.65742660325897 4.40961210397137e-55 8.96139202987406e-54

C4orf47 3.31585842749529 11.65294796875 1.81324065822678 3.10029657048102e-48 3.71217094192496e-47

CDH8 2.88905936911488 1.30275947916667 -1.14902909888685 1.01338600645819e-13 2.05159638433207e-13

CD2 0.885757768361582 5.55843864583333 2.64969557135738 3.32630727930369e-53 5.88512691328755e-52

AK4P1 0.87466647834275 2.14945447916667 1.2971656488506 6.02663953252414e-32 2.75751383138008e-31

DNAJC22 1.38755131826742 5.44030223958333 1.9711456733829 1.74934283263298e-62 7.4098018161299e-61

IGHV3-15 0.722711629001883 5.61923635416667 2.95888206996288 2.16395566288621e-10 3.78041671846759e-10

ITGB1P1 0.725130037664783 5.0085590625 2.78808396480313 4.23965067244979e-50 5.79252786584892e-49

RASGEF1C 25.2371093691149 12.598305625 -1.00231695966322 7.03475881335251e-41 5.2053856002503e-40

MYT1L 4.4756354519774 1.05034380208333 -2.09123089815544 2.00677422500584e-33 9.86933793017286e-33

RPL39P40 2.7769790960452 5.85586723958333 1.07636652896466 1.12221415955643e-18 2.79763672033139e-18

AOX1 0.445801412429379 3.06277317708333 2.78036543515726 3.18925596054547e-61 1.1720200511135e-59

OSMR 5.95932867231638 33.016631875 2.46997123103377 4.25867734506866e-66 2.81815321108725e-64

CFAP94 2.04528728813559 4.35621765625 1.09077253605412 1.7534907078827e-09 2.94014064139574e-09

VOPP1 42.6028530131827 152.655037239583 1.84125324300358 1.04330642193803e-32 4.95917252452394e-32

RYR3 1.70374943502825 5.27524619791667 1.63052524793814 2.23280977759076e-16 5.0399791323461e-16

NHLH1 1.31503719397363 5.71503770833333 2.11965941281237 1.92328216119922e-18 4.74883993018528e-18

CCRL2 2.25589048964218 4.90116442708333 1.1194275129363 8.77632064885061e-41 6.45042223183309e-40

S100A6 413.780485640301 1678.62678078125 2.02034399006542 3.13189683183076e-76 1.18852288954884e-73

AC079944.1 0.640230555555556 2.066748125 1.69069914044355 4.72519861559641e-25 1.55927361591864e-24

ESM1 1.60112895480226 12.1600333854167 2.92498577751822 9.60876422641724e-39 6.24737660105694e-38

AMIGO2 2.99086219397363 10.3657205208333 1.79318705767137 6.90088636898342e-38 4.28314516203782e-37

LINC00634 79.4414170433145 30.495146875 -1.38131169286584 9.47238520433101e-76 3.03687935990578e-73

ELAVL4 20.1985095574388 10.0135497916667 -1.01229534227939 1.35109770947117e-38 8.72349371792236e-38

TBR1 3.72075823917137 1.17250828125 -1.66599854031468 3.94322830773251e-12 7.42677424274422e-12

HS3ST3A1 0.566221798493409 2.31102005208333 2.02909058120685 2.58481046649978e-13 5.1400439123691e-13

AC034243.1 0.471547080979284 1.6782971875 1.83152447908856 1.24777410303002e-30 5.34124296635433e-30

CA15P1 2.95830983992467 0.677025 -2.12749214844451 3.77144515383259e-55 7.72357077483668e-54

ANXA5 235.583396374765 730.140233333333 1.63193571635625 2.3987736127681e-78 1.37704429413294e-75

TPT1P6 0.850171657250471 3.61489838541667 2.08812902488498 6.23376973438722e-26 2.14541825302481e-25

CSTA 1.37489256120527 7.69607182291667 2.48480337706585 6.41224199671179e-58 1.69148731818714e-56

ASIC4 94.6919424670433 40.1148785416667 -1.23910423988954 9.55186181622499e-37 5.60305585087393e-36

PRKG2 3.76964595103578 1.00569166666667 -1.90624097101424 3.23493007237254e-51 4.89053046307052e-50

DRC7 0.677624293785311 2.2917984375 1.75792266202442 2.19883870664953e-15 4.76325599227659e-15

RPS2P46 12.6410234934087 34.4138136979167 1.44487450115834 1.63933048784956e-29 6.63547027025742e-29

LINC01778 1.99897966101695 4.78950796875 1.26061366078365 1.39227614001947e-28 5.40375178953497e-28

AC091825.1 0.88507368173258 3.81025333333333 2.10601745315075 7.05961810066837e-16 1.55758897225829e-15

SPRN 3.73662966101695 1.4410565625 -1.37461062123849 4.60684556118228e-30 1.92115481520934e-29

SELENOF 141.470487758945 305.7784615625 1.11198566666732 6.54205402560489e-69 6.36908348723157e-67

RPS26P47 0.870161723163842 3.42000515625 1.97464303837711 1.48548378441191e-31 6.6480315213332e-31

ADAM19 3.83860108286252 13.39483328125 1.8030240802245 7.37364353871145e-36 4.10149271918455e-35

LRRC55 18.7753865348399 39.2542211979167 1.06400519311678 1.99161617651253e-12 3.80344077254293e-12

MPPED1 11.3836296610169 2.82354041666667 -2.01138344555423 3.00277178178203e-20 8.03172342955076e-20

PYROXD2 2.16054293785311 4.35544182291667 1.01142517270268 2.16410189939722e-20 5.84396501897462e-20

UBE2T 11.7639428907721 35.4877509375 1.59294945978085 2.38476869050457e-32 1.11363068307214e-31

CASKIN1 28.1654376177024 10.7981181770833 -1.38314597631136 5.19445396289888e-57 1.25117709119307e-55

RN7SL5P 0.663981873822975 4.346250625 2.71055560662019 3.2390251587142e-31 1.42758172141006e-30

VASN 4.65714312617702 29.6235291145833 2.66922639648047 2.24826015918056e-64 1.16128882388785e-62

PABPC1L 6.87714006591337 19.572023125 1.50891225826973 1.0563577373401e-38 6.851414335105e-38

GAD1 35.1560483992467 15.9864456770833 -1.13692370318391 6.65058265575216e-34 3.34779600659749e-33

MIR6071 15.6329873822976 4.75483848958333 -1.71712525402182 1.16999643923782e-44 1.09492117703207e-43

AL136090.2 2.93952349340866 1.37364359375 -1.09757457798695 1.0828209305442e-27 4.02628622158037e-27

AL365259.1 2.92395466101695 1.46196848958333 -1.00000872434692 2.032158286025e-31 9.02507363951155e-31

LOXL1 1.41704679849341 13.30198359375 3.23068208697381 5.41870022347259e-67 3.9514012021754e-65

IGFN1 8.33460508474576 2.5533796875 -1.70670575977414 5.14297851201254e-42 4.07125097619724e-41

SHD 260.850533333333 80.75758765625 -1.69155366201633 6.02457997461379e-58 1.60038663754205e-56

GOLT1A 1.06537589453861 8.10310973958333 2.92711313536893 2.19756275953801e-08 3.49650718863774e-08

EIF1P3 0.727095856873823 4.42049588541667 2.60399073845103 5.19332408098291e-36 2.92016514320766e-35

EIF1P5 0.531971563088512 2.78865427083333 2.39014805172929 1.78800580357996e-40 1.28271481163462e-39

LRFN5 6.85630230696798 2.30094692708333 -1.57520301473166 4.565582643714e-40 3.19522052163575e-39

FST 2.33842820150659 7.71651635416667 1.72241055190318 7.57834000816087e-13 1.47683119316445e-12

P2RX6 11.9950086158192 5.65935421875 -1.0837248503129 6.90482569027114e-42 5.41980724823098e-41

SNAI2 3.37608639359699 10.3499400520833 1.61619868350855 1.52631548458618e-36 8.84720587153367e-36

MYO5B 1.28584967043315 2.70635109375 1.07362702559776 1.67669716811045e-18 4.14934573343277e-18

PTTG1 10.4670307909605 51.6360538541667 2.30252650342766 4.15488277003276e-51 6.23567757132842e-50

GRM1 3.93273884180791 1.72012765625 -1.19301874960793 2.18277884069414e-33 1.07207534449835e-32

RXFP1 2.6631447740113 0.752168333333333 -1.82400338396174 1.23807954536191e-21 3.52991247255515e-21

FAP 0.703531120527307 2.47547296875 1.81501804844062 3.41329126456597e-38 2.15445183518684e-37

IQGAP3 2.58288601694915 8.93886052083333 1.79110695734842 2.40150881588179e-40 1.70638350903026e-39

HOXB8 0.0886834745762712 2.88338317708333 5.022953465823 1.42697273434408e-48 1.74799459783453e-47

NPY2R 0.760412806026365 2.82951739583333 1.89570127354858 1.03849845150158e-11 1.91804516345569e-11

DBF4 6.22463540489642 13.3148666666667 1.09697673976199 1.28709098184575e-31 5.78522040305094e-31

AC110285.3 1.52435946327684 0.558620104166667 -1.44826374793624 1.82021952092731e-38 1.16593117435905e-37

UQCRHL 4.68145188323917 12.1722461458333 1.37856747838036 2.63838118148193e-33 1.28937445649557e-32

C16orf92 1.65893479284369 0.814051041666667 -1.0270660193103 1.25975141256761e-30 5.39127215574101e-30

IL9 2.53608201506591 0.8054 -1.65482402455405 3.03832291294483e-42 2.44578418035537e-41

MMD2 69.2675538135593 23.6583686458333 -1.54982912930168 4.41163201118613e-40 3.09096824596858e-39

LINC01602 24.5079470338983 3.15719588541667 -2.95653395923273 1.80560191014045e-27 6.65250000377683e-27

AL121820.2 4.2103172787194 13.7427753125 1.70667252123886 2.54218152796674e-37 1.53929877930777e-36

MYO16 13.0420375706215 5.53214963541667 -1.23725719624964 1.24786096499297e-36 7.26713894270099e-36

CISTR 2.6062604519774 0.952847447916667 -1.45166410382693 3.55132496242165e-32 1.64352632345024e-31

TPST1 55.4730769774011 118.042875520833 1.08945131419784 9.62708702540207e-56 2.08642987456121e-54

AK5 35.807327354049 15.34257171875 -1.22271451308173 1.20197658275909e-13 2.425738501889e-13

AL078590.2 0.808445291902072 1.66435302083333 1.04173941771149 2.0622162993106e-20 5.57692147842941e-20

STXBP6 10.3784180320151 4.09387057291667 -1.34204915481867 2.73860296733086e-47 3.07885865643998e-46

PCBP3 9.54135762711864 3.98168510416667 -1.26081543151344 8.27589820138521e-53 1.41704126410341e-51

CP 6.49711228813559 29.4107219791667 2.17847165588371 7.88324403416047e-43 6.57054786262725e-42

AC016931.1 1.5657225047081 0.698306510416667 -1.16489621596972 9.40925168787878e-46 9.47806257508699e-45

LINC02587 1.55909406779661 23.5424286979167 3.91648327956424 1.25919072226833e-42 1.03604652568936e-41

MT-TW 1.76615061205273 0.7706396875 -1.19647998597259 7.19950595387062e-16 1.58788771453237e-15

RNU6-882P 1.05579223163842 2.58448796875 1.29155253027274 6.09988012867908e-22 1.76156656301891e-21

POU4F1 0.678721374764595 1.65092046875 1.2823792683964 0.00668682469715688 0.00797776884663366

SLC16A11 1.20855018832392 3.10315140625 1.36045670312744 7.84212790413494e-23 2.36727870742515e-22

GALNT13 103.778997316384 32.6803834895833 -1.6670176840689 5.72437502370896e-64 2.83097748845394e-62

HPCA 74.3987713276836 33.6720194270833 -1.14372854871979 1.45723075326113e-06 2.13229507844592e-06

LINC01770 2.32117970809793 4.69513385416667 1.01630806651495 5.53504393784222e-40 3.85195142306048e-39

CNTN4 2.83070037664783 1.06357692708333 -1.4122346659997 1.15836122112447e-28 4.51282776174513e-28

CARD19 11.0583253295669 25.73184078125 1.21842174629732 2.91641163714727e-54 5.53374228497485e-53

LINC01117 4.37654905838041 1.90537140625 -1.19972149849929 1.04342335846963e-27 3.88437584599455e-27

POSTN 3.89403239171375 107.747821770833 4.79025000899925 1.14231073696312e-55 2.4584801103969e-54

ALPK1 1.53324416195857 3.79476536458333 1.30742322690888 1.21307635686458e-41 9.3949168957503e-41

CTTNBP2 44.6576120998117 22.0352691145833 -1.01909159423973 3.64678177070741e-42 2.91789617152772e-41

ASIC4-AS1 43.9934215630885 9.41361932291667 -2.22446639217381 5.76874299021458e-66 3.75069146514126e-64

ADGRA1 31.3427650188324 15.3611902083333 -1.0288424555534 1.76869461472344e-43 1.52829351118877e-42

RBM24 1.3076645480226 2.61960572916667 1.00235719411019 1.74313493546998e-17 4.11915035265781e-17

ERFE 2.00735564971751 4.77319989583333 1.24966050966631 1.53241860334275e-07 2.34510113810867e-07

PCDHGB5 11.1964463747646 5.27140052083333 -1.08678269260607 1.87449845301044e-06 2.72912334330032e-06

DAAM2 106.583683050847 49.6307805729167 -1.10267954204629 2.2354757010818e-31 9.91855658831211e-31

MT-TL1 29.6951493879473 12.5259247916667 -1.24531016860631 4.28028470683962e-32 1.97498496584821e-31

DOK3 4.44946624293785 11.8097727604167 1.40827701899193 1.66847700530825e-55 3.50965270517046e-54

FAM163B 56.1807933615819 18.8112347916667 -1.57848244556638 2.09786092455106e-35 1.13631587218255e-34

TMEM255B 1.20127066854991 3.31827989583333 1.46587432867821 4.93298847163581e-72 8.73608767905409e-70

CYTIP 2.13229585687382 6.11072380208333 1.51893564780823 4.65626768772695e-49 5.86608959106541e-48

ITGA7 34.800318079096 110.533515052083 1.66731148027343 8.27591725096154e-53 1.41704126410341e-51

NEDD8P1 1.02049618644068 2.74258354166667 1.42626477606298 4.31841912594629e-27 1.56288446179391e-26

ALOX5 9.34204138418079 24.96178890625 1.41791158854101 1.36431757490176e-47 1.56505153024665e-46

ULBP3 0.611771516007533 3.42782239583333 2.48622751979805 1.56744647874668e-55 3.31966597634334e-54

CCT7P2 2.1084854519774 0.586933072916667 -1.84493915790529 2.91437019387512e-42 2.35007431722064e-41

ASPN 2.03934237288136 6.12469104166667 1.58653306855114 5.89136634428251e-36 3.29870391966074e-35

AL139095.2 1.32602170433145 9.5985659375 2.85571448808606 4.16137497492337e-20 1.10575546811518e-19

PLK1 4.84922457627119 15.26063703125 1.65398921227687 2.20854992125015e-36 1.26440842936104e-35

HHIP-AS1 1.47696426553672 2.97752567708333 1.01147902822338 1.81916559219983e-17 4.29717786927793e-17

SPEF1 4.60997956685499 13.8265883333333 1.58461295876978 1.00176435461512e-14 2.11415495203649e-14

MN1 23.2624397834275 8.91168947916667 -1.38423154667609 3.59764062488559e-57 8.76777554649378e-56

STMN2 241.104490348399 115.723113802083 -1.05898147205321 3.24120133256841e-15 6.98055811664461e-15

CDC6 2.3406038606403 8.26316833333333 1.81981427415053 3.00008421580738e-42 2.41709558028329e-41

ICAM5 15.4731798493409 6.47983098958333 -1.2557416218555 1.23659564120171e-13 2.49425056385137e-13

PCLO 3.83712509416196 1.68459942708333 -1.18762021845776 1.05590833918145e-12 2.04080818699502e-12

CENPU 4.54701836158192 19.4379630729167 2.09588431011053 1.25011289278099e-42 1.02903272427014e-41

GLP1R 4.20000470809793 1.04328302083333 -2.00926036121444 1.68735953600768e-38 1.08418972260065e-37

FOSL2 21.4288875235405 45.2124714583333 1.07716382740811 1.52602163894333e-31 6.82780856019038e-31

FO681492.1 2.01733163841808 0.850385 -1.2462602203069 4.47863659005014e-55 9.09173006462689e-54

UNC93B1 13.3306596516008 36.1924121875 1.44093909285773 1.79167799433417e-65 1.09592935212644e-63

HDHD3 7.42620922787194 21.8009919791667 1.55369591364415 2.75243475310313e-52 4.50541586566485e-51

SNRPEP2 5.05500918079096 10.9740919270833 1.11831594861543 1.62809921262653e-15 3.53963578379403e-15

MCOLN2 0.530194350282486 1.58288135416667 1.57795991931213 2.28796729250209e-31 1.01441945169472e-30

CENPE 1.01554096045198 4.09814671875 2.01272320561709 1.31712466542852e-43 1.14448285764688e-42

HNRNPKP2 0.610756120527307 2.51759119791667 2.04337571850514 3.54846283244237e-34 1.81323622888887e-33

GOLGA6L2 1.52569223163842 0.584835052083333 -1.38336227875707 1.65514477057924e-48 2.01951555176647e-47

PLK4 2.80574952919021 6.86884536458333 1.29168138153787 4.74506374024579e-30 1.97702382341555e-29

RPSAP47 1.08546544256121 2.36795270833333 1.12532647211089 3.59032642238628e-14 7.40320690000808e-14

PLAAT5 2.361975 6.18948098958333 1.38982474449433 1.03629556631687e-21 2.9655149362361e-21

TMEM51 14.1799156779661 31.6616302083333 1.15888658602382 1.11148426365362e-58 3.16994630101826e-57

AL732437.3 7.34559712806026 2.69552625 -1.44631281398792 8.15013916314715e-36 4.52258542938589e-35

EPHB6 27.6146331450094 12.6617859375 -1.12495205120496 0.000393250827221696 0.000506300569977667

AP000757.1 7.32964425612053 3.47898203125 -1.07507795139722 3.53948702425718e-31 1.5585309309984e-30

RPS27AP11 2.14912316384181 4.41895614583333 1.03995744934271 2.35760952341525e-18 5.8004431182729e-18

CDCA8 4.89386351224105 23.27143890625 2.24951464644893 1.00184098061022e-51 1.56548176760059e-50

RNF135 10.2171716101695 30.3971269791667 1.5729390975819 4.29695234848769e-71 6.29148259213611e-69

OSBPL10 1.23716807909605 3.34797265625 1.43624622988295 2.45910591569825e-32 1.14690430154023e-31

GADD45A 45.897520386064 162.97743984375 1.82818415393451 8.30737666737114e-66 5.29026264143035e-64

MYO1F 8.37004105461394 17.0412471875 1.0257243208318 2.2203176698677e-39 1.49644099569373e-38

BTBD8 11.6542601694915 5.55316072916667 -1.06947636423754 2.30942185600746e-31 1.02368770947458e-30

AC092131.1 2.72778799435028 6.51685171875 1.25644364753152 9.18116012931501e-18 2.19891386662304e-17

EIF4HP1 2.53276370056497 8.78214072916667 1.79386016896863 7.68041307121507e-29 3.01238728241393e-28

WSCD2 7.59094896421846 2.01032286458333 -1.91685303029749 3.11034966036751e-39 2.0797213164064e-38

LINC02732 3.38458088512241 12.335689375 1.86578924222131 1.72406091966278e-40 1.23880008190498e-39

FABP5P7 0.33488488700565 15.1349967708333 5.49807928689025 2.64238813595616e-63 1.19550382939428e-61

NEFM 62.7284711864407 13.6002833333333 -2.20548369451338 1.24671261940098e-18 3.10176895340664e-18

GATA4 0.0726894538606403 2.11588375 4.86337048933949 2.86419497723105e-50 3.96572640369407e-49

MAP3K6 7.33109938794727 17.3449668229167 1.24241561162239 2.44095650553922e-55 5.08282040543133e-54

TBX1 0.665007532956685 1.75295197916667 1.39834388693761 2.22782948832482e-21 6.28149952015163e-21

MYOZ3 0.723348446327684 1.70794786458333 1.23950125246308 7.48888034016508e-21 2.06304785074622e-20

GREM2 3.21318790018832 1.3892065625 -1.20974421789723 1.2768987659032e-11 2.34972118277783e-11

NEXN 2.84470630885122 6.35289625 1.15913474396203 4.71681053757624e-37 2.81028811106152e-36

L3MBTL4-AS1 0.898445103578154 1.85730859375 1.04771127988319 2.17481218651574e-32 1.01815691967709e-31

RPL31P63 1.03103554613936 3.3159796875 1.68534109729076 8.32025853746144e-19 2.08820633694285e-18

TUNAR 5.67250715630885 1.7968575 -1.65851052368996 3.34822598462678e-22 9.79088884795329e-22

JPH3 41.3464081450094 11.19286140625 -1.88518310252676 1.41718605972272e-68 1.27925120293903e-66

HLA-DRB6 16.0693479755179 53.86833796875 1.74512616166239 3.77373935477166e-38 2.37712341808872e-37

HLA-DQB2 2.95707598870057 10.7536853645833 1.86258794409085 2.29431118673549e-40 1.63208555919458e-39

EEF1A1P13 11.4854422316384 26.5402680208333 1.2083765332174 3.36083195334587e-17 7.85503647215516e-17

TXNP6 0.391255320150659 2.37167661458333 2.59972503283322 2.53386536394116e-35 1.36730198614294e-34

MTFR2 1.07685696798493 3.08681255208333 1.51929123872077 5.70980919992104e-50 7.7273582294419e-49

DEPDC1 0.71485922787194 4.26513052083333 2.57685881600866 3.24076488843851e-51 4.8953714947615e-50

SAMD9 5.22895814500942 14.5475430208333 1.47618008516885 7.99592150691692e-45 7.57047659985337e-44

GABRG1 22.7347480696798 9.9574553125 -1.19105001212271 3.62903597544362e-47 4.04811410863331e-46

IL32 7.35619129001883 17.9816456770833 1.28949416097605 4.4477152463691e-34 2.26156043221858e-33

PRDX4 74.6803057438795 179.511161510417 1.26527381051316 2.82924408329788e-53 5.02481315462503e-52

RPS4XP3 0.558916337099812 3.47740604166667 2.63730728352092 3.83086712876717e-22 1.11741136093217e-21

GAPDHP71 0.5423631826742 2.0344678125 1.90732030336801 5.48871741659398e-34 2.77948530396419e-33

AC108463.1 0.789970433145009 4.89160588541667 2.6304376087163 1.50060462809173e-57 3.80678622910856e-56

LINC02520 2.25445838041431 0.722395833333333 -1.64191939989414 1.60462040920244e-29 6.49921945308634e-29

SARDH 1.76246511299435 3.7719753125 1.09772553317772 2.02576370887859e-21 5.7221747176967e-21

C3 164.382812335217 478.934184791667 1.54276795372574 1.74932516116877e-43 1.51296285450852e-42

ALDH1A3 0.871685075329567 3.40022890625 1.96375295946989 5.915842535614e-23 1.79395127119606e-22

TDO2 0.451647881355932 2.38439682291667 2.40035400976214 1.82796941493845e-26 6.43283332149517e-26

AC092117.1 8.92877024482109 4.29333510416667 -1.05636270466886 2.69142842036676e-37 1.62754834070634e-36

AGTRAP 29.2515152542373 64.8526384895833 1.1486539113239 4.55948473216386e-66 2.9958875828476e-64

AC131235.1 3.6082395480226 14.2072208854167 1.97725734632014 6.24352310102324e-18 1.50973097611869e-17

CILP 0.703237947269303 1.96113052083333 1.47960072902266 1.25631282809322e-05 1.75660854488258e-05

PYY2 0.661174387947269 1.65500520833333 1.3237330123878 6.40083217146455e-30 2.65203819581959e-29

IGHV3-48 0.96427104519774 3.72472046875 1.94962152576579 1.15751286732879e-16 2.64682141760684e-16

PLEK 15.0979967984934 31.4500369791667 1.05870456789867 6.73042518882122e-31 2.91798219599279e-30

FAM86B1 0.802629661016949 1.79236369791667 1.1590570351972 2.57375013778449e-34 1.32426352551474e-33

AP003355.2 3.06773493408663 0.781701979166667 -1.9724832373683 4.69766381316617e-13 9.22516196069542e-13

NDC80 3.33557518832392 13.5904981770833 2.02659087384598 9.30311753678147e-44 8.16768038699015e-43

COL27A1 2.3458672787194 5.31055765625 1.17874197207866 1.86636087321465e-24 6.00640021416172e-24

C1QB 354.027708474576 1013.78719166667 1.51782065754996 3.69209074694557e-47 4.11597286807271e-46

MAK 0.567563700564972 1.59627192708333 1.49185220993253 1.12149925869901e-24 3.64394176402378e-24

HLA-DMB 12.2239780602637 40.1909370833333 1.71715635623629 2.46789546087697e-56 5.67249889925924e-55

CDK4 60.7927179378531 328.742492291667 2.43498752125356 3.8498590200058e-22 1.12277491337842e-21

PTPRC 6.52504929378531 15.12656125 1.21302334847225 6.66860306545957e-31 2.89185340490254e-30

H2BC4 2.90680282485876 6.8109471875 1.2284222320143 3.13475197068022e-22 9.18542592102091e-22

ITGB1 17.2881737288136 36.9665354166667 1.09643436235217 4.7641624978848e-41 3.55353396101756e-40

RPL3P7 1.36908959510358 3.7606809375 1.45777704926471 4.33661787279551e-18 1.057288702565e-17

AL645608.6 10.2894253766478 3.88359713541667 -1.40569695992259 1.90746705310453e-45 1.87767865815134e-44

RNASE6 39.905102212806 89.44812296875 1.16447798895345 4.49390861631408e-40 3.14624362651959e-39

MS4A4E 0.505530838041431 1.82356317708333 1.85088917629434 1.80822582344585e-40 1.29672036972524e-39

IFI27 27.3516264124294 57.6483999479167 1.07565394322831 1.57169823180325e-15 3.41821387372882e-15

THCAT158 0.490621186440678 1.59539411458333 1.7012314208357 3.09302130862301e-34 1.58617570970339e-33

NXPH1 66.3219175141243 29.7039578125 -1.15883054766154 9.4771068424119e-32 4.28984424865261e-31

HLA-DPB2 0.686843314500942 1.9841128125 1.53044112846496 3.83040074742627e-21 1.06962459676215e-20

AC079140.2 2.01396092278719 6.803995 1.75634639005067 2.0190853445423e-30 8.5523671940237e-30

DES 3.1725497645951 9.12449234375 1.52410150003485 1.70700754964707e-38 1.09567847378969e-37

UBE2MP1 2.40489571563089 6.03302088541667 1.32690624152669 1.78384831772889e-33 8.79158745512025e-33

ZIC1 13.5876001883239 28.8621374479167 1.08688747266224 5.35851619727559e-26 1.85207451093568e-25

TUBB2BP1 0.875243644067797 2.83694505208333 1.6965816226694 2.50563544857567e-34 1.2910028031661e-33

DGKK 4.32488940677966 0.7917940625 -2.44946608752536 1.7588780041245e-60 6.00116265810919e-59

SLC22A18 2.28215739171375 6.2724134375 1.45862236359896 1.8201139618658e-42 1.48312967225655e-41

MSR1 5.14278860640301 28.9199328645833 2.49144144493997 2.91338984209764e-64 1.48018133073605e-62

AC092597.1 1.11344910546139 3.22912390625 1.53610718472873 3.45177005102288e-18 8.44770519857469e-18

CACNA2D3 8.41103573446328 3.368629375 -1.32012175651829 3.2892075591918e-30 1.38033885270078e-29

TRH 7.35254270244821 29.0097038541667 1.98022040464287 2.61685589154271e-13 5.20154305753465e-13

H4C3 0.567156355932203 2.260395 1.99475648000752 1.93759351648508e-20 5.24676735678463e-20

ADORA1 40.7727945856874 83.944039375 1.04182104398767 3.96042940485928e-24 1.25844471605192e-23

HMGB2 73.5863582862523 164.805052552083 1.16325022899167 8.35839751405956e-51 1.21805957503086e-49

LRAT 1.68839806967985 4.86224989583333 1.52596895773447 1.90234276986045e-16 4.30918063169144e-16

AP000843.1 3.76946487758945 1.2469465625 -1.59596008927669 2.26674798839531e-29 9.10567700242186e-29

SLC17A9 1.15043700564972 2.77994145833333 1.27287251390134 4.96561809455627e-43 4.18755866069269e-42

FAM20A 1.31835437853107 7.26897244791667 2.46301321293771 2.39222782999658e-49 3.07631234431441e-48

SSTR1 18.0662541902072 4.602811875 -1.97271002919862 8.66210990296025e-61 3.03336974850369e-59

SCN3A 31.8248524952919 13.8748256770833 -1.19768418181152 1.53891565731231e-39 1.0451474305231e-38

PPIAP6 0.634388606403013 2.43736895833333 1.9418858908703 8.65468388758075e-38 5.34663943154698e-37

RPSAP61 0.905490583804143 2.6915134375 1.57164608431268 4.54676709359839e-22 1.32228861597532e-21

SYT16 6.63315329566855 2.76430182291667 -1.26277972135038 2.36237555434459e-15 5.10913857095111e-15

MORN3 1.75042914312618 5.62973494791667 1.68535833769181 2.80996778647014e-36 1.59936795192569e-35

DPP4 0.213855508474576 1.97542630208333 3.20745575055445 1.98011047902979e-42 1.60927248066254e-41

RPL13AP25 17.7928286723164 37.4778722395833 1.07474316213851 7.57576038233633e-25 2.47402993167447e-24

LINC02328 0.793805555555556 1.79674515625 1.1785282323973 1.10337145349338e-40 8.04608497152788e-40

LINC01503 1.96575484934087 5.65731348958333 1.52503370408803 1.85709542638255e-47 2.1069365133364e-46

SMIM10 8.54937443502825 20.7335140625 1.27807388956851 8.5563254517513e-43 7.10924360032687e-42

SLC49A3 2.66733385122411 6.0184346875 1.1739899024584 1.46017529712286e-38 9.41142448873469e-38

SLC25A27 31.1587212335217 15.1524952083333 -1.04008063950491 2.7744736183109e-46 2.89189108365982e-45

TSTD1 3.93017057438795 24.42867671875 2.63591188237241 1.66932247968234e-43 1.44511412987398e-42

RPL10P16 9.32013813559322 26.7144473958333 1.51919693185146 3.89673692241007e-24 1.2384177588825e-23

TMSB10 1720.25025833333 5649.7957371875 1.71558024886086 8.25075613151967e-68 6.7587141086171e-66

COLEC11 1.12507490583804 2.5253828125 1.16648103954862 3.22106847285643e-26 1.12311584947994e-25

MAPK8IP2 84.4691251883239 37.0178794791667 -1.19020185503502 1.674553601256e-52 2.77525171259851e-51

CLDN23 0.747319915254237 2.91099348958333 1.96171373872512 1.23017881102729e-49 1.62120304684993e-48

HOXC13 0.134475376647834 3.07235619791667 4.51393155178006 2.86084321183191e-64 1.46146647043996e-62

CARD6 3.75268469868173 7.6335875 1.02443814923232 6.15112752931485e-33 2.95174752019638e-32

GAS1 20.0377614877589 51.6160865625 1.36509941576182 4.47252683159551e-21 1.24556891468502e-20

RPS26P6 0.568839030131827 2.31527744791667 2.02509272505047 4.25538282797125e-29 1.68682250450065e-28

CAVIN3 1.01793667608286 4.25200729166667 2.06249625404211 3.2750053865229e-44 2.96488437986336e-43

FBN3 1.39530809792844 4.65933666666667 1.73954085942435 2.64020586128795e-14 5.47137278397965e-14

C17orf102 1.46057989642185 0.6162721875 -1.2449016898705 2.58473052236653e-25 8.64507337801166e-25

SH2D4A 0.679705178907721 6.12698536458333 3.17219638482105 1.90240385199779e-70 2.42295887862321e-68

CLEC18B 1.1052920433145 7.28777083333333 2.72104998091932 8.7666455103797e-69 8.27491234850307e-67

RNU5B-1 0.389873163841808 1.90199401041667 2.28643594483526 1.08908895007768e-11 2.01028479518508e-11

LINC00672 39.8739097928437 15.6041407291667 -1.35351615938675 1.18849759699144e-56 2.78339641095616e-55

LRTM2 9.24784110169491 2.44421348958333 -1.91974630665447 5.33115531429055e-43 4.4897116426283e-42

RN7SL4P 2.1575713747646 12.1183429166667 2.48971224446449 1.98761585823349e-24 6.38888796609365e-24

AL049871.1 0.3844815913371 3.87621114583333 3.33366072979841 5.04822230090831e-55 1.01702810060011e-53

FRMPD1 4.708456826742 1.87597119791667 -1.32761662309917 2.96809642562727e-47 3.32680850117776e-46

AL512306.2 0.671797316384181 1.74325130208333 1.37568262162411 1.81679299747651e-14 3.79374124515168e-14

RPS26P15 0.60181972693032 2.01451567708333 1.74302973029501 1.24284838073227e-23 3.86791056731658e-23

C1orf115 41.8635769303201 17.6244965104167 -1.24811354062401 1.65311843041922e-10 2.89888129136603e-10

CELF5 27.5882095103578 11.65711578125 -1.24284094883855 1.22263539753482e-43 1.06387015522508e-42

TOB2P1 1.52201501883239 3.05836953125 1.00678013759241 3.60048512570866e-24 1.14642159096837e-23

BAALC-AS1 2.35395155367232 6.44584291666667 1.45328440197437 6.72621471264857e-38 4.18027949805148e-37

AC009227.1 4.45067881355932 1.16088546875 -1.9387997463634 7.05018270164434e-69 6.79265012109205e-67

AC005336.1 1.45586459510358 3.09702708333333 1.08900781762339 5.18797235060654e-11 9.29491154405446e-11

IGF2BP3 0.314706403013183 4.05744067708333 3.68849156450582 4.59158584673615e-67 3.3881166198436e-65

RCOR2 85.0150842749529 34.7418775520833 -1.29104311857703 8.97289952827938e-50 1.19692300379021e-48

NPIPB5 0.69461577212806 2.00108385416667 1.52649454932637 1.19755729185445e-25 4.07401716831934e-25

AC010173.1 1.01243229755179 2.10765151041667 1.05781090768314 2.86254483821341e-46 2.98034833519476e-45

AP001025.1 0.680562429378531 2.5536196875 1.90774426511332 6.70829829843216e-50 9.03918890285116e-49

AC007608.3 2.2883577212806 1.0850340625 -1.07657226117338 1.44422805489611e-30 6.16374126251851e-30

RDH10 8.33419124293785 43.2720261458333 2.37632056355649 3.56734345996798e-65 2.1058651313684e-63

AC025211.1 6.41208375706215 2.42750145833333 -1.40132110829278 1.69260896221061e-38 1.08681159020395e-37

MRPS17 13.4871649246704 37.9134564583333 1.49112287094762 9.17643151202567e-33 4.37191247671323e-32

PTHLH 1.97953606403013 4.32784114583333 1.12848519452316 3.60464556809696e-29 1.43345561032427e-28

AC093503.2 2.7807049905838 1.18139708333333 -1.2349567396799 1.08625942181619e-41 8.44086667307649e-41

ADAM33 1.69141798493409 10.06017828125 2.57235074287495 2.32733049842253e-39 1.56742885252325e-38

TRDJ1 0.420243785310734 14.4302183854167 5.10172283874634 8.20071976652674e-27 2.9269171604331e-26

RSPH9 0.939455838041431 2.71936015625 1.53336998665641 2.12393457606538e-35 1.15010376942154e-34

NUAK2 2.15949034839925 8.3173575 1.94543437525371 4.89802293329361e-72 8.73608767905409e-70

RPL18AP3 42.0975759416196 110.05325296875 1.38639272155707 2.58854763470524e-21 7.2787000253053e-21

RPS2P17 0.775277919020716 3.75458229166667 2.27586693191215 5.58757648654994e-35 2.96351924607519e-34

FZD1 7.95637179849341 18.9762942708333 1.25401568795727 4.79538719543011e-50 6.53261720871963e-49

CDK1 7.18703347457627 23.6015105729167 1.71541088895118 3.00247444886861e-35 1.61361307447144e-34

FXYD2 1.59239350282486 0.52011265625 -1.61430084062856 4.16799926659762e-48 4.96184035610645e-47

WNT10B 5.52512048022599 2.10718703125 -1.3906875524896 7.39849938406406e-11 1.31764290821445e-10

AJAP1 7.88256016949153 3.12408348958333 -1.33523126808619 4.2515303534064e-35 2.26915060050494e-34

AC008972.2 1.33029971751412 3.01972197916667 1.18266440649443 7.60942291310547e-42 5.9527647904584e-41

SMAD9 52.7931267419962 26.15822546875 -1.01308543995752 5.81178306100724e-45 5.53921609530649e-44

ANXA2P2 1.24738855932203 21.9199857291667 4.13526402181125 8.67037840853684e-79 5.77961437868553e-76

PTGFRN 18.8635581920904 67.1676661458333 1.83216506678292 8.22839592301184e-65 4.58104856851512e-63

TFEC 1.70948469868173 3.78078375 1.14512382415991 4.72915063297404e-36 2.66157857203851e-35

CARMIL2 3.99960051789077 1.59372260416667 -1.32745536759958 5.8385237264192e-27 2.0987380768737e-26

ATP2B2 45.0400566854991 21.4421386979167 -1.07075983178803 3.8745298362064e-23 1.18342447937349e-22

RBFOX3 9.54896497175141 2.88361197916667 -1.72746731790899 1.42746473417191e-27 5.28759098245551e-27

COL5A1 3.49429571563088 21.2141334895833 2.60195214056813 3.59762265231965e-57 8.76777554649378e-56

FKBP10 57.6293596986817 161.551901302083 1.48712183415635 1.11087105448741e-68 1.01756883045288e-66

CNMD 1.21154844632768 6.32432583333333 2.38405960303124 1.89021013194587e-22 5.61387276849281e-22

CXCL6 0.210689736346516 3.4304 4.02518487015325 6.88204753968643e-10 1.17642649384509e-09

EPHA3 3.43658413370998 7.8503390625 1.19177968697254 3.95862072117734e-05 5.38325315608455e-05

AL133260.1 3.03577434086629 11.0851904166667 1.86849709285326 4.30126241844403e-29 1.70428454428746e-28

PNMA5 3.55367537664783 1.05169 -1.75660238596689 5.72714766070901e-40 3.98414929857404e-39

WDR62 1.65154129001883 5.75084302083333 1.79996041866943 2.80915972672541e-39 1.88306867766615e-38

NCAPH 2.78764661016949 8.39479552083333 1.59044750222652 4.29913103156957e-34 2.18780354493804e-33

PRKCE 20.4662808851224 8.25873505208333 -1.30925622737415 5.4988625534773e-57 1.32278588851113e-55

SEPTIN2P1 0.325616666666667 3.35485536458333 3.36500411906083 1.12504605608312e-30 4.82477661735831e-30

CHRNA4 8.77157236346516 3.00550635416667 -1.54522740848877 5.94415901147328e-56 1.31585281926602e-54

TMEM220-AS1 0.342205225988701 1.79233067708333 2.3889031355747 5.15182649720367e-58 1.37839156425183e-56

TRPV2 5.51859548022599 11.0940966145833 1.00741915134078 4.52889775557593e-42 3.60663185288798e-41

AC016739.1 19.5569541902072 77.2458459895833 1.98177565028849 5.93972576919194e-27 2.13428407107486e-26

AL513477.1 2.68332212806026 5.71231473958333 1.09005521381631 4.09901452254916e-33 1.98234525479328e-32

AL512308.1 4.47028446327684 0.995863385416667 -2.16634689005895 1.26158648724078e-60 4.36044623238705e-59

HOXB13 0.283888512241055 3.09791239583333 3.44789997188944 2.93877369957183e-44 2.67089427876531e-43

F13A1 7.93561440677966 72.8304568229167 3.19812806268342 4.25034585061108e-21 1.18493524875732e-20

IGHV1-18 0.87391972693032 9.067865 3.37519023923628 2.80937257446019e-17 6.58601651816531e-17

TMEM185B 12.4800216101695 24.99855765625 1.00222442565342 1.62598548334927e-39 1.10347445484962e-38

JPT2 18.8101380414313 38.3815330208333 1.02890189990423 5.70852087991911e-54 1.05516844693932e-52

AL731533.2 9.70946040489642 4.414969375 -1.1369876908379 5.25586208970371e-52 8.41073627866097e-51

AC092718.4 8.15981224105461 21.3452903125 1.38730992350632 6.05211052857737e-54 1.11645828649699e-52

HTRA3 1.36256732580038 8.1717365625 2.58431517909685 3.43935455531854e-61 1.25647933116205e-59

PALLD 26.2435761770245 54.1572101041667 1.04518909325867 1.00579995765468e-55 2.16970420099638e-54

WIPI1 7.40800701506591 15.5278009895833 1.06769616325257 7.8752965122607e-58 2.05675756524561e-56

DACH2 6.96652452919021 1.74939776041667 -1.99358074957136 3.65759858892358e-52 5.92448133806916e-51

MT-TF 30.2383229755179 12.5362271875 -1.2702748992034 9.3295890641317e-42 7.26785541045366e-41

B2M 604.103503248588 1408.632885 1.22142801040033 9.92553027610777e-54 1.8076908470541e-52

AQP9 0.80113483992467 5.84712177083333 2.86760964702753 6.42633151702053e-29 2.52905046685707e-28

GDNF 2.39839533898305 1.19422572916667 -1.00599392817187 7.52260756800278e-35 3.97056167263729e-34

CXCL11 0.89933154425612 9.17330567708333 3.3505167371311 1.29096430879114e-45 1.28784771040618e-44

CPNE8 3.08147132768362 7.07013635416667 1.19811867176682 3.90026691628774e-17 9.09068228984338e-17

NTRK1 0.771303248587571 2.39884479166667 1.63696972404768 7.401971726623e-12 1.37598384741132e-11

ARHGAP44 6.76730258945386 2.24992994791667 -1.58870081436234 5.5832149340335e-49 6.99125271466907e-48

IL6 1.72539938794727 5.07229307291667 1.55570775629532 3.46222434381533e-35 1.85533318943072e-34

CDC25A 4.27391007532957 8.633533125 1.01439452491192 9.95032694352742e-18 2.37853618093447e-17

CRYGD 0.415685310734463 1.78763822916667 2.10449112984239 2.48580350773744e-06 3.5980007960129e-06

PPARG 1.38250715630885 2.84140203125 1.03931602519906 8.91420390228607e-29 3.48894172938349e-28

OR52K3P 0.476962664783427 1.7860503125 1.9048244754239 3.46124606756082e-56 7.80143886379314e-55

EEF1A1P7 0.953375564971751 2.63901125 1.46888094612764 8.16330072122143e-19 2.04936650345771e-18

PIK3CG 2.13320936911488 4.65409307291667 1.12547449265562 1.52977111815772e-38 9.84982097052271e-38

PARVG 3.76214585687382 8.4789346875 1.17232723006325 9.04246550916935e-45 8.53096391665134e-44

AL590682.1 0.867808286252354 1.79932125 1.05200452082281 2.29454508062967e-22 6.7768528866437e-22

RIBC1 2.87722942561205 7.37539484375 1.35804002266831 2.99347733848783e-23 9.18240038092728e-23

AL162151.2 35.6515490112994 78.4349145833333 1.13753123862501 1.45603019453283e-11 2.67300636463007e-11

CEP55 2.04916661958569 8.35906541666667 2.02830435519116 2.35043671864059e-49 3.02675697944057e-48

AC026367.1 1.50310936911488 3.03675458333333 1.01458033485142 2.69936085051449e-22 7.93465302170676e-22

FTH1P8 8.44384081920904 39.4150896354167 2.22277676863671 1.46846068638424e-41 1.1288146532995e-40

RHBDF1 10.6278870998117 22.2447423958333 1.06560958429654 1.10849949975076e-46 1.19147677444309e-45

SIGLEC9 4.36658888888889 13.1396816145833 1.58935170442824 5.4054104239157e-51 8.0282433572454e-50

STOX1 30.341722834275 12.2809294270833 -1.3048832568753 7.01424008757642e-34 3.5251295791482e-33

KATNAL2 2.7451722693032 5.66247338541667 1.04453567795836 1.57641162482988e-32 7.42863004655642e-32

IGLV3-19 0.800694020715631 9.11717854166667 3.50926449030323 2.00373592251127e-23 6.18517089626445e-23

SVOP 24.2248027306968 5.075658125 -2.25481811373207 1.47877170203516e-46 1.5713005599625e-45

AC005840.1 1.9675563559322 3.93689661458333 1.00065386803886 2.86562576060695e-17 6.71365894147489e-17

AL021395.1 3.02769816384181 0.996548177083333 -1.60320993042375 1.27500121269085e-28 4.95582097616772e-28

TPPP3 39.9060885122411 135.15484515625 1.75993245007628 5.31459287827285e-25 1.74911247029175e-24

TEKT1 1.35640941619586 7.96664239583333 2.55417911242416 1.73067449871845e-18 4.27837235887319e-18

FTH1P15 1.70443323917137 3.43012119791667 1.00896746088862 3.02325336806205e-31 1.33406256238998e-30

STAR 1.82391822033898 0.622230208333333 -1.55152070198687 4.53293612317385e-52 7.29783092730889e-51

VWA1 24.6599379943503 62.863316875 1.3500492232803 4.8250405960172e-51 7.20077286379934e-50

RNF165 13.4709757532957 6.55033541666667 -1.04021366605898 2.18826709985543e-47 2.47210368905296e-46

TAGLN 16.6904761299435 45.9366247916667 1.46061974701398 3.06789581915295e-29 1.22393312072837e-28

C1QC 373.513305320151 939.64120640625 1.33095034600962 3.43936320975457e-43 2.92700040665383e-42

NAP1L2 58.5174296610169 22.2700304166667 -1.39376287287604 4.13559709571785e-50 5.65451676432893e-49

SYNGR1 85.3996649717514 39.1718171875 -1.12441435335544 6.72317490961334e-46 6.86908996946484e-45

GTSE1 3.50591177024482 12.3095382291667 1.81191504784011 3.05482982861879e-35 1.64127595097274e-34

FGFBP3 38.7333048022599 18.1909376041667 -1.09035469537764 7.17720348078009e-23 2.16902484519918e-22

LBP 0.575946092278719 2.18396328125 1.92294291164836 2.49301374312123e-09 4.1490728142253e-09

FTH1P10 0.259286111111111 2.15612583333333 3.05582454331116 4.6563677017422e-40 3.25508110578557e-39

FUCA1 24.2985238700565 52.72518203125 1.1176234977765 4.19592348179917e-54 7.84152735116136e-53

FTH1P23 4.78018305084746 14.2968325 1.58055777911744 2.95894162163619e-41 2.23211032269067e-40

ABCC3 0.690037806026365 21.7898077083333 4.98083424780167 6.64875591546881e-81 7.27256566165544e-78

CHRM4 8.47651539548023 3.0588178125 -1.47049712970356 3.79627198526835e-43 3.22336427242306e-42

PCED1B-AS1 3.32821770244821 8.32359729166667 1.32245736211077 4.29845479060546e-44 3.86881736840796e-43

LHX6 3.32002401129943 1.53920270833333 -1.10901043282112 0.000107336698675115 0.0001426887299296

LINC00689 35.1300670903955 12.0016740104167 -1.5494706827727 2.44449693678286e-22 7.2094243520186e-22

ITGA1 2.81540442561205 6.71362203125 1.25374914071915 2.44789806090569e-36 1.39885262576955e-35

DYRK3 2.28264486817326 6.204904375 1.44270255304073 8.62765757889223e-71 1.17103133342701e-68

IQCG 7.19659849340866 16.10029859375 1.16170036851844 5.254748237829e-46 5.39547451587136e-45

CYTH4 8.30301826741996 16.7309213541667 1.01080911779312 7.22306121415948e-32 3.28875669141272e-31

TPX2 16.1163047551789 54.1613488020833 1.74874267553192 7.27149965821736e-37 4.29249321093815e-36

LMO1 8.95385282485876 20.56449375 1.19957504661559 1.5046591574287e-07 2.30470651008127e-07

AC005544.1 2.47400527306968 1.08489630208333 -1.18929142348711 2.12814495225144e-21 6.00407455425816e-21

CCT6A 143.192745809793 360.065693229167 1.33030174018621 1.01153783436205e-35 5.58810042482541e-35

PLA2G4A 8.10166831450094 18.6096515104167 1.19976011249423 1.84778786554321e-36 1.06442426765105e-35

CALY 24.0015833804143 9.24578213541667 -1.3762623104288 3.79622040400053e-17 8.85483171254263e-17

C1QA 283.164069161959 768.417410208333 1.44025199414393 1.53759703142699e-42 1.25898797003016e-41

CHST2 20.9781063559322 53.88801140625 1.36107989376442 1.63842719131622e-50 2.32037727513519e-49

ARL4C 31.5855213747646 109.446066354167 1.79288481120328 5.27326026783622e-53 9.17271044718564e-52

FKBP1C 0.911879990583804 3.94892140625 2.11454278090056 2.32078052687336e-45 2.2665618900164e-44

DIRAS3 6.58510381355932 44.9358613541667 2.77058916699234 1.37789381605777e-66 9.66865490928084e-65

AL162231.2 1.66481638418079 6.941018125 2.05978422781151 1.33650266278595e-41 1.02993232550786e-40

CTSZ 91.2299033898305 239.36019640625 1.39160456971191 1.42146825222761e-52 2.37699659623853e-51

AL358780.1 0.478606261770245 1.872123125 1.9677641432097 1.81819623298959e-47 2.06406342810998e-46

AC021242.3 1.88052255178908 6.11564213541667 1.70137038031956 6.85716560410633e-22 1.97504638178992e-21

FCGR3A 47.7560093220339 222.449590677083 2.21972425431485 3.67392176938404e-59 1.08957855345608e-57

NEUROD6 7.55758498116761 1.56569880208333 -2.27111859232392 3.83518572441532e-18 9.36633550637022e-18

XKR8 2.41137655367232 10.31787296875 2.09721672880687 1.20140067491766e-66 8.52673494278393e-65

MICALL2 8.4505538606403 24.48334546875 1.53468289937162 1.16258850282303e-58 3.31061764318442e-57

MAP7D2 14.7139893596987 5.00249005208333 -1.55647015488431 8.93919609215459e-08 1.38370431557725e-07

HOTAIRM1 0.342566619585687 4.36495848958333 3.67151144986797 3.26170632274888e-64 1.64367016453971e-62

IGHV1-46 0.217318738229755 2.61504703125 3.58895241251701 1.40636150042116e-14 2.94762084088497e-14

TBX19 1.35420583804143 2.80517286458333 1.05064263359891 2.07744943363398e-58 5.77431572771657e-57

CD109 2.51404642184557 8.59472682291667 1.77344049607325 6.01325385158808e-39 3.93719913275635e-38

AL512329.2 2.10494237288136 9.72225505208333 2.2075102449599 6.96315211386653e-37 4.11700520055161e-36

RASEF 0.269857815442561 2.36513510416667 3.13165122192295 5.33509599378616e-44 4.76318187476196e-43

RPS3AP5 2.76079392655367 10.6080294270833 1.94200157243042 2.09248546537413e-18 5.16044658204668e-18

PLOD2 14.9841089453861 41.09942140625 1.4556847897507 1.17359739000972e-56 2.755434781216e-55

CELSR1 0.618029001883239 3.55013197916667 2.52212621369384 6.63981267148089e-44 5.89342800124998e-43

IL2RA 0.21296384180791 3.4306196875 4.00978879258703 5.66092687577682e-36 3.17637100950724e-35

TMEM220 3.58041854990584 8.43993625 1.23710385377143 1.94786932442282e-40 1.39310115721701e-39

HNRNPA3P6 2.42101257062147 5.90624765625 1.28663128146242 4.40414736072276e-26 1.52590125158636e-25

AC011228.2 0.0964887947269303 15.9740555208333 7.37115350633102 2.63480630605671e-17 6.18223637364347e-17

MARCO 1.49445696798493 10.5893930208333 2.82492663591391 1.320713823835e-12 2.54204259954578e-12

RPL17P34 0.752377071563089 1.53269307291667 1.02654103225344 1.26010710083101e-17 2.99714652595966e-17

HACD4 1.69265282485876 3.68790088541667 1.12351378642505 6.53915713920955e-38 4.06674337804688e-37

SLC35F3 3.64740291902072 1.62889572916667 -1.16297532392097 1.85880833827789e-16 4.2115926709245e-16

KCNJ9 60.7441191619586 20.9424432291667 -1.53631498076571 9.95827553459653e-52 1.55739389037698e-50

SLC25A21-AS1 8.28667029190207 2.98498348958333 -1.47306956996671 1.4825296767253e-54 2.88063106987533e-53

RSPH4A 1.71357434086629 7.63061244791667 2.1547900728529 3.66940884238983e-37 2.20532183013054e-36

EEF1A1P8 1.2685536252354 3.12007260416667 1.2983950936666 2.97035188106656e-15 6.40316406543388e-15

TGFB2 8.24944858757062 38.398825 2.21869257072103 5.16997222776702e-59 1.50682811246595e-57

RNU4-62P 1.54669877589454 3.38384609375 1.12947169730349 1.45446935777848e-13 2.92261267645244e-13

PPIAP29 8.01906073446328 20.1031505729167 1.32591644871774 4.42205955747797e-32 2.03888414260607e-31

AC011495.1 2.34356563088512 9.19703541666667 1.97246369842458 1.84722903860765e-31 8.2234196727099e-31

MAGED4B 0.532067231638418 1.63496307291667 1.61957759127031 3.87225271968531e-22 1.12912873330012e-21

SEZ6L 235.788382250471 82.65253 -1.51236174938745 2.37257657848546e-44 2.17759434733155e-43

PRRT3-AS1 1.99272801318267 4.05267380208333 1.02412924755047 2.20854646630463e-36 1.26440842936104e-35

ODF3B 4.1591081920904 11.03421640625 1.40763806058384 9.47135077819893e-23 2.84799106922072e-22

TFPI 3.37842015065913 10.1721355729167 1.5902019329475 1.94285724526499e-35 1.05481548834168e-34

LINC02593 9.71982396421846 3.141960625 -1.62926508449604 8.65558726634491e-56 1.88687743514283e-54

PLAUR 3.66505131826742 20.389875 2.47594762663351 1.90051959502037e-71 2.94501348911698e-69

EYA4 0.4734881826742 4.76025604166667 3.3296388499471 7.51914517360972e-39 4.91108199870997e-38

ARHGEF39 0.779698917137476 1.68654916666667 1.11308534074498 1.15067320449929e-28 4.48381564075111e-28

ATRNL1 18.901190960452 8.00262104166667 -1.23993264307507 3.38321103414598e-43 2.88185108474322e-42

SYT2 2.31807547080979 0.83555484375 -1.47212110625946 8.24370784031754e-10 1.40389914177768e-09

SERPINA3 0.809732156308851 4.69469052083333 2.53551338048513 1.76948791246104e-58 4.96283977861433e-57

AL133342.1 1.92173483992467 4.5062675 1.22952366958578 9.41689963719721e-51 1.36909498634622e-49

RPS15AP24 0.741153813559322 4.83237119791667 2.70488639440407 3.26955546598423e-25 1.08722074195237e-24

EFHC2 1.78226671374765 7.14033776041667 2.00227906954556 2.94221418390328e-32 1.36776181874204e-31

FAM114A1 6.02637467043315 23.3090680208333 1.95152904580771 8.4491938782699e-86 5.23709200554763e-82

PROCR 7.8880815913371 17.3807629166667 1.13974503005313 3.66416102903801e-51 5.51254646722992e-50

GNAL 13.0588920433145 4.02576546875 -1.69769746744335 4.04366875694042e-68 3.49730328071196e-66

CLEC2L 8.75744764595104 3.04469505208333 -1.52421271911502 4.76327948631082e-18 1.15933484355955e-17

CALD1 34.9239039077213 87.09681984375 1.31840520569436 4.87587296244929e-57 1.18055804344719e-55

LHFPL2 17.3542852165725 35.6816194791667 1.03988915117118 7.15510374845094e-46 7.29436152425687e-45

GMPR 20.9759011299435 56.3645661458333 1.42605569991135 1.75407690432808e-33 8.64715271367461e-33

CELF3 34.3249597928437 14.231260625 -1.27019456697428 7.86821198720427e-41 5.79902504566244e-40

SPINK8 4.72458060263653 22.6218189583333 2.2594567639297 8.36239403383403e-52 1.31890345257968e-50

SNORD14E 6.75522434086629 16.4928071354167 1.28776138425491 7.42922928898425e-25 2.42702949101655e-24

CLEC17A 1.15143281544256 3.74133760416667 1.70012392084153 8.0120209309158e-34 4.01248395395581e-33

ZC3HAV1L 1.71385654425612 4.96695015625 1.53511391336576 1.49394356929262e-35 8.17055313852831e-35

SPRY1 7.90579712806026 36.8695560416667 2.22144720506122 1.84522506271739e-58 5.15969323928269e-57

PNMA3 22.4058433145009 8.02896666666667 -1.48058879889107 3.82902973523784e-42 3.05845394874346e-41

LINC02885 1.58989934086629 0.392685364583333 -2.01748969425975 6.23710511545117e-43 5.23843584561041e-42

KNL1 1.65061209981168 4.23220036458333 1.35840680921458 1.63999627449516e-26 5.78230330066209e-26

MTCO1P2 1.56514218455744 4.5677415625 1.54518730298458 7.87710543748144e-12 1.46269997613309e-11

KIAA1549L 20.8332956214689 8.996334375 -1.21148188833572 1.09678466546865e-42 9.04421767378694e-42

GAPDHP60 0.465166525423729 4.92055880208333 3.40300297856195 2.38209948441926e-40 1.69323929330184e-39

TUBA1A 1308.33641699623 2667.76940635417 1.02790041623888 4.93744606712229e-44 4.41615245878494e-43

PTX3 2.72018413370998 45.8190034375 4.07416986211361 2.73252130849376e-67 2.11564288017976e-65

RGS4 48.1267315913371 16.5879855208333 -1.53669975647172 2.62046977370453e-05 3.59985486421659e-05

RIMS3 41.2642978813559 16.5860279166667 -1.31492566718481 2.6432343812117e-20 7.1017112149446e-20

CSRNP3 12.9030469397363 6.10971942708333 -1.07853375080344 3.01485820765617e-43 2.57516253428417e-42

ADAMTS7 1.45738564030132 4.55998125 1.64564521131742 6.01105469614064e-47 6.60612068999617e-46

ARHGAP9 3.52803879472693 7.46584015625 1.08143819582318 1.63677137647018e-45 1.62237546617607e-44

RIMBP2 6.109640913371 2.92768067708333 -1.06132938284171 8.27522294919669e-11 1.47040765483997e-10

ZWINT 14.4816782485876 29.1402268229167 1.00878330441628 3.56493887633684e-23 1.09029668430072e-22

AC009245.1 1.82832617702448 4.40850197916667 1.26976503437813 9.73013730886215e-18 2.3273977779559e-17

ELK3 15.0330491054614 34.19910796875 1.18582103894277 1.69766830340906e-58 4.76860152596548e-57

SOWAHB 2.5091804613936 0.579505572916667 -2.11432179341808 4.41054500468182e-09 7.24058306365838e-09

MT1L 10.9861061676083 41.9377863020833 1.9325705714157 6.92128030342998e-44 6.13447126988944e-43

PPM1L 31.8770167137476 14.0689865104167 -1.17999821291748 5.05628621740606e-65 2.89297360654356e-63

RHOD 1.83292923728814 7.17752411458333 1.9693351827546 4.86948950929653e-32 2.24018202437825e-31

AL031729.1 6.38146064030132 13.5259825520833 1.08377481525817 1.12202637338453e-26 3.97941644346468e-26

HOXC8 0.140127966101695 2.33329869791667 4.0575541900463 1.75203666872053e-63 8.18570900875836e-62

NUP107 10.911811440678 24.0012532291667 1.13721911885225 1.52612884400587e-25 5.16345812487066e-25

RNU6-644P 1.43247052730697 0.622843541666667 -1.20156374743241 3.53327581704001e-19 9.00881171230755e-19

GNLY 1.70975527306968 5.63033677083333 1.71943137890379 3.32793359233207e-43 2.83606439731507e-42

ZC3H12A 2.88710211864407 6.22358286458333 1.10812322946101 2.61386682442168e-40 1.85514708397409e-39

EPSTI1 3.29097467043314 10.5305095833333 1.67798842404558 9.4899617618416e-52 1.48790757977609e-50

RGS1 41.8083930320151 99.5161778125 1.25113848362369 1.47476176933098e-27 5.45301155313374e-27

PIK3R6 1.67807401129944 3.83986557291667 1.19424945844808 1.21307854082799e-41 9.3949168957503e-41

SYT13 27.2286688794727 8.58329692708333 -1.66552264310196 1.62587244975925e-20 4.41487999463687e-20

INA 125.096497740113 28.8606486458333 -2.11586576969109 4.87418800513169e-60 1.58731218836119e-58

MIR4648 2.47903596986817 5.22204135416667 1.07483467809257 1.90022447596881e-10 3.32530341903256e-10

LRRC25 6.01975098870057 17.2994686979167 1.52295201520447 1.00766395391082e-48 1.24750407609665e-47

FADD 3.33994053672316 7.12519984375 1.09310806103413 6.51878401144958e-45 6.20034724771892e-44

CHMP4BP1 0.76791450094162 1.74970447916667 1.18809367859399 1.83003820114086e-31 8.15079289825492e-31

ISG20 1.00718945386064 4.49766932291667 2.15884251337835 8.21233863552087e-76 2.67909538469317e-73

AC016717.2 2.72051242937853 0.850588802083333 -1.67734465248046 2.67286193298981e-39 1.792996668252e-38

CNNM1 6.81520838041431 1.81200614583333 -1.91116992063 7.20963306832445e-42 5.64240792705153e-41

ITGAL 2.23349011299435 5.13472515625 1.20098718795913 2.55031551813745e-39 1.71326289955802e-38

SLC25A45 1.30392391713748 3.2943821875 1.33714824256604 1.62863782061079e-68 1.455986551647e-66

MIR6503 0.640688276836158 2.00251901041667 1.64412144080512 2.44983385147276e-15 5.29397564998675e-15

KCNT1 3.55079712806026 1.09655739583333 -1.6951616081891 2.29290985929871e-23 7.06489790118633e-23

AC020899.1 0.62140593220339 1.81494526041667 1.54631811731748 8.29799703992538e-26 2.84059747712468e-25

SWAP70 10.5633037664783 26.6973772395833 1.33763689709056 2.6265282922417e-59 7.92866022179918e-58

TMEM106C 27.801927259887 82.8840990625 1.57591045817615 4.98829444450724e-65 2.87174412370316e-63

PPIAP48 0.635078860640301 1.94129854166667 1.61201434513101 6.53625378601308e-24 2.05654211761274e-23

ANK3 8.71466129943503 4.03198234375 -1.11195527300931 2.68366959659698e-22 7.89102405893752e-22

SNRPGP10 5.25377961393597 55.0042289583333 3.38811495487315 2.77747896298301e-52 4.54241172530071e-51

NEURL1 15.2004492467043 5.92649604166667 -1.35886267433947 4.1553019246167e-15 8.9131202316585e-15

IRAK2 4.85225207156309 10.8928316666667 1.16665263667586 1.6060312561283e-42 1.3121331813579e-41

AF106564.1 6.78450762711864 2.40075421875 -1.49875640576633 2.66423898154229e-46 2.78479616985828e-45

CASP4 2.41709717514124 11.48823828125 2.24881019758731 7.49142905337998e-73 1.67835088250121e-70

TNFRSF14 4.79318615819209 13.98632515625 1.54496007301139 1.24225844026979e-64 6.67626044671357e-63

SLITRK5 7.47293983050847 2.98206604166667 -1.32536369796991 7.87259625737499e-64 3.8222174257412e-62

CFH 4.40481031073446 16.5989663541667 1.91394160808989 2.02182953528381e-45 1.98604966764936e-44

XRCC2 1.9056774952919 4.97998994791667 1.3858388428287 3.78596261716122e-32 1.75037232387153e-31

CLIC1 54.8696634180791 324.566105885417 2.56443171739204 3.92811925526188e-81 5.21738411082819e-78

GPR61 2.28529072504708 0.8217796875 -1.47555413457424 9.62855399651215e-46 9.68847194616577e-45

OSR2 0.473514171374765 2.45478067708333 2.37411462383424 6.31590256770072e-59 1.82366782991296e-57

FANCB 0.637798540489642 1.44883291666667 1.18371852768446 4.43146188324124e-44 3.98467281039027e-43

EVC2 0.418426506591337 3.11310182291667 2.89530661324096 5.68988269819358e-63 2.50126167311843e-61

IRF1 3.61922010357815 11.1482320833333 1.62306418854755 3.74563521974994e-61 1.35770149924464e-59

IGLC2 3.94183771186441 34.24164171875 3.11881158756535 7.7574678965578e-25 2.5324809609637e-24

NCF1C 1.69805258945386 5.95218770833333 1.80953888353015 1.72058901460428e-62 7.30464674122525e-61

RPSAP4 0.497098210922787 1.70619046875 1.77917589328302 2.38718807877976e-19 6.1311826415621e-19

MMP11 1.26908724105461 4.91123916666667 1.95229583236528 9.15695524138349e-56 1.98688529839786e-54

POC1A 6.02502928436911 17.0980123958333 1.50478846629283 1.89559941100303e-41 1.44996590076517e-40

AL139156.2 1.02245579096045 2.56054817708333 1.32441423872564 5.70567084126923e-38 3.55671972153541e-37

OR2L13 3.40437419962335 1.49122333333333 -1.19089328359733 1.58410657484308e-19 4.10199996646805e-19

EYA1 11.100072834275 5.33817255208333 -1.05615129770972 2.25698815634513e-30 9.52967637766523e-30

RAMP3 21.9918224576271 56.8120998958333 1.36923106406026 2.85667215866559e-20 7.65304981852567e-20

AC004067.1 1.30509425612053 5.7806246875 2.14707140262122 5.59064359237467e-76 1.92514847407791e-73

GAL 0.950856308851224 5.47218239583333 2.52481707213933 3.07555499411346e-21 8.62853728357571e-21

TMEM45A 4.69650061205273 12.2973638020833 1.38869097443714 1.14259317805762e-31 5.14942320552144e-31

GSAP 2.51100381355932 8.97244442708333 1.83723686271426 1.20472959092802e-69 1.35769374201858e-67

COL21A1 0.837975282485876 2.90471036458333 1.79341472142093 1.2492251154058e-15 2.72708863829195e-15

VIPR2 37.2477936911488 15.65510765625 -1.25052154298904 8.08272235247538e-32 3.66939018906933e-31

AC073861.1 21.8767792843691 102.279781614583 2.22504872104173 1.01389398669948e-29 4.15546808081923e-29

SUMF2 66.2096017419962 168.110966770833 1.34430148479175 2.79812042071262e-58 7.65162488575752e-57

RAB38 0.733028625235405 2.90516296875 1.98667765294901 1.88705265098175e-58 5.26083119115527e-57

CENPL 3.15456134651601 7.22620348958333 1.19579847452842 2.52281240518345e-56 5.79156749066497e-55

ADAM12 0.993788088512241 10.2353113020833 3.36447292175036 4.90946987935578e-68 4.16856586331601e-66

HFE 1.55731096986817 6.64251411458333 2.09267233218071 7.83150181504026e-63 3.39456354896675e-61

SPCS2P4 6.20404868173258 13.6118409895833 1.13358029125535 5.99710111749763e-31 2.60734382230228e-30

MORN5 5.65078959510358 14.31985125 1.34149212930181 1.19622450465367e-05 1.67410210461617e-05

ANXA2 11.2597799905838 97.5867751041667 3.11550700965908 1.79605901393568e-80 1.72105977672105e-77

WDR38 1.15688050847458 10.48484609375 3.17999391977795 1.51943583808992e-35 8.30020840460695e-35

AL590135.1 0.605054990583804 3.0552590625 2.33615654150693 8.65789506032147e-29 3.39076576762169e-28

MYO1G 0.577099576271186 2.92440161458333 2.34124927657796 4.6056034602633e-55 9.32910635551155e-54

AC105052.5 0.798297598870056 2.4630340625 1.62543800286512 1.38336977462014e-25 4.69240440697948e-25

EVA1C 5.60305291902072 21.3049302604167 1.9269023054716 4.57982726265602e-71 6.60169674023943e-69

EVC 2.09597052730697 7.65598442708333 1.86896946484216 5.76153330554702e-42 4.54158144355171e-41

AL049873.1 3.22474241996234 15.03102375 2.22068743999706 9.41225653819322e-24 2.94253168062665e-23

AL596087.1 0.706378013182674 1.66380442708333 1.23597351769004 5.24965027801137e-32 2.40857103076053e-31

SAA1 0.440053107344633 131.50848625 8.22326253999087 9.91081458883137e-63 4.26600919628054e-61

AL356653.1 0.495422410546139 3.26733546875 2.72138355310866 1.35071851865292e-31 6.06534915584425e-31

AP002840.2 0.489649435028249 2.03219729166667 2.05321934492003 7.72850377480465e-38 4.78719279455338e-37

HOXA1 0.159610216572505 2.17882296875 3.7709240751583 4.58853963615606e-72 8.44791034993286e-70

TCIRG1 9.61494482109228 25.3546994791667 1.39890269145677 8.30167011426438e-61 2.9126331278254e-59

MRPS15 25.3993939736347 52.6883103125 1.05268883906721 4.654512432297e-76 1.66443574381851e-73

PTGES3P1 19.0369032485876 51.3081544791667 1.43038932052525 2.29582888983582e-49 2.96053663013156e-48

CENPK 1.62590437853107 7.35362838541667 2.17721385878187 5.15147977872588e-46 5.29242619072165e-45

SKA3 3.27301798493409 10.8594505208333 1.73025767082538 5.8485316649747e-32 2.6773374276269e-31

MYD88 15.6283119585687 39.5690145833333 1.3402091785213 1.91682962446138e-61 7.24460302171938e-60

GPX8 1.43665466101695 12.7024311458333 3.14431942632677 2.3754734610483e-70 3.00489312980906e-68

PNCK 14.113111299435 6.3600721875 -1.14992102620875 4.5700554620822e-32 2.10555454205695e-31

PLOD3 33.5794311205273 74.20513984375 1.14394132971855 1.82856322964611e-65 1.11482404115637e-63

PSMA6P1 0.949357438794727 3.4062675 1.84316845875467 5.25740821081671e-31 2.29379412670429e-30

RPL35P5 4.93177758945386 17.7356867708333 1.84647555170388 4.70535030679068e-31 2.05727695637838e-30

SERPINI1 99.0297053672316 44.842585625 -1.14299188060128 8.17211372677633e-16 1.79707254907055e-15

MCHR2 2.15901365348399 0.401763958333333 -2.42595231402144 4.5424737178829e-17 1.05544544275937e-16

CFAP300 2.78071600753296 6.40638734375 1.20405461831642 5.80607135358598e-09 9.47550437247071e-09

KCNE4 5.17111247645951 22.42114484375 2.11631335510464 6.33181600169216e-49 7.902021379293e-48

APOBEC3H 0.708457015065913 1.43617484375 1.01947917058364 5.02635104019652e-25 1.65629979784608e-24

OR2I1P 0.662053531073446 2.0021121875 1.59650303966747 1.3755576241751e-21 3.9158747736583e-21

TMEM183B 0.977864406779661 3.79188953125 1.95521059863949 2.82144440261054e-50 3.9182045307351e-49

AC134684.10 5.00158210922787 2.2708565625 -1.13914794108265 1.19621231953733e-17 2.84881763342683e-17

SNORA7B 0.641953907721281 2.31753161458333 1.85204739879292 2.05498203830095e-21 5.80206362013455e-21

SERTAD3 15.1197551318267 32.6740768229167 1.11171169958151 1.19159828956221e-65 7.41062548308003e-64

GNGT2 2.22797504708098 5.03278182291667 1.17562298179897 9.80372835329081e-47 1.05804021317146e-45

CA7 2.76575193032015 1.09796291666667 -1.33284243345586 4.51568465185405e-07 6.76134601024447e-07

IGLV1-40 1.13748502824859 14.1457676041667 3.63645100350503 1.60955019847791e-21 4.56661366199218e-21

NOG 39.1208152071563 10.7032202604167 -1.86989151052298 8.42130384689014e-66 5.33219542050239e-64

ASPM 1.34354213747646 5.65383984375 2.07318944727668 4.95465702510789e-40 3.45840267950005e-39

COL2A1 1.18804995291902 4.92391963541667 2.05121171823357 3.89456477664372e-09 6.411068698804e-09

CLVS2 9.85848333333333 3.50060588541667 -1.49376106841443 1.76176813283198e-29 7.11866111038911e-29

PKMP4 0.207651224105461 2.12554671875 3.355599687644 1.10959712593178e-36 6.47613262922205e-36

SNHG4 1.65021299435028 3.32451026041667 1.01048958393953 1.00233656624205e-34 5.25026716880871e-34

CACNA1I 4.25099241996234 1.41847458333333 -1.58345938629072 7.19210835041273e-36 4.00530861862608e-35

C8orf88 1.6335299905838 5.69391354166667 1.80142764487768 3.31213795117792e-55 6.82057572712855e-54

GJD3 0.29988352165725 1.90446182291667 2.66690921310327 5.08470075853076e-38 3.1792202624371e-37

FTH1P20 11.1144917608286 30.5102430208333 1.45685169247385 1.1097841209948e-51 1.72689838743919e-50

FCGR2A 9.54438013182674 35.8269877083333 1.90832333983389 1.93862936357334e-59 5.91934532276622e-58

AQP1 201.146522693032 1105.370298125 2.45821104942082 9.55488414843027e-47 1.03298296941896e-45

AC123912.4 0.802839783427495 3.550105 2.14467768124322 9.82287073196353e-30 4.02947896008961e-29

NFE2L3 1.69623780602637 5.75970760416667 1.76365712965668 5.40836945548083e-59 1.57384397534689e-57

CDH18 8.41219538606403 2.32170697916667 -1.85729645697798 6.64686959892138e-53 1.1455496848311e-51

MEIS1 3.57657038606403 8.00447630208333 1.16223018289106 1.12535065368896e-36 6.56601675724701e-36

NMUR2 2.13029755178908 0.925298385416667 -1.20306437606083 1.02680061876171e-30 4.40853325002863e-30

AL450405.1 27.3714688323917 71.6402346875 1.38809720750182 2.43869773572134e-17 5.73294366570649e-17

UPK3A 0.642640583804143 2.33686067708333 1.86248772629812 7.01951554303726e-17 1.6180474962536e-16

UGCG 18.4458873822976 39.8731556770833 1.11211859401855 5.08732369187084e-55 1.02379636418115e-53

AC097658.1 0.762242514124294 3.52379697916667 2.20880882498175 7.76311669766732e-34 3.88992603053419e-33

SIGLEC10 12.9443079566855 28.7453069270833 1.15100859886659 1.90842562297202e-31 8.48772409929795e-31

HCLS1 28.1109331920904 62.41081265625 1.1506646499396 1.89051848892845e-41 1.4466745391615e-40

SLC29A1 22.2817484463277 63.7972352604167 1.51763145896192 2.38127180879043e-36 1.36161590665615e-35

TREM1 0.510740725047081 8.04526958333333 3.97747775877594 8.78224519695171e-62 3.45255495639148e-60

GRIN2B 4.16032467043314 1.06905333333333 -1.96036229166384 2.76727212291654e-29 1.10804102337711e-28

LINC01116 1.88710047080979 11.65362375 2.62653549717283 2.39347368163855e-17 5.62876477931818e-17

ISLR2 6.17101826741996 2.88504239583333 -1.09691604575174 4.13642266792442e-08 6.50459023340842e-08

C1orf53 9.04105263653484 19.0691461979167 1.07667759145336 9.94628063970345e-41 7.27867329772868e-40

HMGN1P36 1.88891812617702 4.84657322916667 1.35940487936751 5.28386928617108e-42 4.17744682722582e-41

SHC3 59.9294049435028 28.7226461979167 -1.06107537924088 0.000373630508922911 0.000481706948167616

RBPMS 2.48198540489642 6.17239770833333 1.31433639074676 1.1342675020335e-45 1.1357945180567e-44

IGKV1-9 0.326929990583804 5.64239208333333 4.10925328820259 2.83991172954821e-22 8.33462099288967e-22

AL391416.1 0.291532015065913 1.85259776041667 2.66782344549018 5.15022576515897e-37 3.06655293317743e-36

Z97353.1 0.943694962335217 2.85534791666667 1.59727403809659 4.17204141948606e-17 9.70831062386976e-17

SPATA6 10.6182945386064 25.81810484375 1.28183103897756 1.44672877921205e-48 1.76869964822144e-47

AL035411.1 0.493118502824859 4.03695411458333 3.03326089687543 1.41531030447452e-52 2.36882944299763e-51

CPLX1 86.5550381826742 32.0397692708333 -1.43375403976123 9.62191843833392e-43 7.97324301964435e-42

APOL4 4.70944529190207 33.3159833854167 2.82258543289895 2.99207552793034e-62 1.24191170629162e-60

PIPSL 0.672342608286252 1.53118828125 1.1873832081839 3.10137045545446e-31 1.36723526835409e-30

NRSN1 48.5872539077213 16.4557080729167 -1.56198978985175 1.53465526113138e-50 2.18673674948184e-49

MS4A4A 10.9638585216573 32.3399966145833 1.56056391102144 1.44971967420109e-31 6.4942272565091e-31

EN2 1.30823356873823 6.29761979166667 2.26718652080183 9.69623931372795e-40 6.66302919581564e-39

ADM 7.79909806967985 61.9059458333333 2.98869878393288 1.88971766796768e-50 2.65402568246669e-49

IBSP 1.25061468926554 28.8871758854167 4.52971989477652 1.27507562456803e-46 1.36186279373018e-45

AL359091.4 1.23586073446328 2.57767463541667 1.0605539930409 5.72309568101088e-39 3.75647596852797e-38

GPR158 34.1486582862524 15.5766498958333 -1.13244391240989 5.58322682510544e-49 6.99125271466907e-48

AC011474.1 1.40831346516008 0.677190416666667 -1.05633502556041 1.13642582952253e-37 6.98804176586357e-37

C20orf204 2.80653130885122 1.39901489583333 -1.00437683018149 2.49359362832721e-18 6.13013928063782e-18

ZFR2 5.97419449152542 2.20279213541667 -1.43941084295614 8.23849885414253e-16 1.81124244730173e-15

BNC2 0.689987711864407 1.41141609375 1.03250079163316 3.37792365748163e-17 7.89299954899107e-17

CUBN 0.826728625235405 2.07798578125 1.32970003688179 8.35903476509088e-50 1.11744249789263e-48

IL4I1 1.68388229755179 6.95332104166667 2.04591090337857 1.25573527723893e-55 2.67474351578972e-54

TMEM176B 48.7949893126177 186.105770416667 1.93131787623213 3.70172330659648e-53 6.51832811421984e-52

GRP 8.43404811676083 1.86996994791667 -2.17321016739968 5.0492559107952e-05 6.83240530208388e-05

FTH1P2 5.44395296610169 30.12953125 2.46845172029387 4.35027989130957e-41 3.25134463741565e-40

GZMH 0.752472457627119 3.157840625 2.06922767906586 3.29862301219692e-45 3.18969812333862e-44

GDAP1L1 76.1612345574388 27.1315683333333 -1.48908441996948 6.71473783325482e-58 1.76856303129424e-56

FERMT3 16.1698206685499 35.7724938541667 1.14554701961973 5.5259862589264e-45 5.27493400845669e-44

PSD 85.2677175612053 26.68921125 -1.67574296926158 6.84450079515452e-58 1.80019083855585e-56

ARHGAP29 2.97186308851224 6.00217010416667 1.01411655259722 4.02137570098108e-33 1.94581007441434e-32

EZH2 11.6817212806026 26.7962077604167 1.19777597436095 2.67574353345146e-29 1.07208470167054e-28

EFCAB10 0.637638888888889 1.66488979166667 1.38461515493737 1.30306107526942e-17 3.09614371257793e-17

GPD1 18.137758992467 39.3047191145833 1.11570632487168 7.83535897555691e-07 1.16085172616111e-06

KCTD16 4.0218934086629 1.403311875 -1.51903917453782 3.66440926365079e-36 2.07679641138636e-35

AC073072.1 0.705441807909605 1.59017229166667 1.17258410228022 2.43368894809463e-18 5.98366335975402e-18

NTNG1 2.26987622410546 4.68147572916667 1.04434974864051 1.8210645941943e-20 4.93625308003542e-20

EPHA5-AS1 0.675087099811676 2.1255571875 1.65469551883387 2.47032787899295e-28 9.48106231369947e-28

RASSF10 0.45982038606403 2.48220791666667 2.43248163045212 7.73073007421856e-16 1.70262851747121e-15

FPR1 20.6689617231638 64.7080580729167 1.64647946295356 4.90531414996457e-38 3.07015538938375e-37

NEU4 69.621938700565 28.9575797916667 -1.26560095776853 1.21499013026516e-37 7.4563503208847e-37

CYP4F3 1.24584279661017 3.00990677083333 1.27259676431538 0.000230659192829593 0.000301011136968649

ZMAT4 4.40361530131827 2.15692630208333 -1.02971155764838 1.82630536891102e-10 3.19895896146387e-10

NDST4 2.33081770244821 0.385747239583333 -2.5951084350542 7.47130720096842e-65 4.18460715066288e-63

THSD4 5.60720875706215 2.57345859375 -1.12357421235575 3.6756690444251e-39 2.44453025325768e-38

FAM81A 13.1283145480226 6.14752369791667 -1.09460441429954 3.74687603022691e-22 1.09325529235948e-21

ANKRD62P1-PARP4P3 2.89882532956686 0.55526203125 -2.38422775247205 2.8209726080539e-29 1.12881397991741e-28

SPAG5 6.6851200094162 14.5903645833333 1.1259905691769 8.56365215382971e-24 2.68172973729309e-23

PTN 745.835847975518 1699.96385166667 1.1885740231457 8.11964458136516e-29 3.18264736489218e-28

MIR210HG 3.38924143126177 9.38567953125 1.46949879090782 2.09190234039722e-22 6.19311001746319e-22

SLC6A7 7.11616789077213 1.68999723958333 -2.07407965828015 1.17349695682026e-13 2.36903440582703e-13

CCR5AS 2.64002570621469 5.95334432291667 1.17314836044336 3.1804327591339e-33 1.54776621711842e-32

IL2RB 0.609392467043315 2.26101895833333 1.89152951745302 6.16396259527188e-43 5.17934407858475e-42

GALNT9 13.8294093691149 4.53953630208333 -1.6071226988668 5.009745573825e-45 4.79198657125905e-44

AC004486.1 0.745974717514124 1.737514375 1.21982627297894 2.44658638791077e-12 4.65366958707045e-12

FAM13C 22.5919201035782 9.55066244791667 -1.24213418246379 5.59497426112282e-66 3.6504753117747e-64

AL049597.1 0.595869303201507 1.49642046875 1.32844777239267 4.61927092348659e-20 1.22427797637162e-19

CCR1 13.7903753295669 28.1883219270833 1.03143587280201 1.49118244491687e-28 5.7731704274889e-28

CLCF1 1.70522697740113 14.5522653645833 3.09320806654075 8.70283423518122e-79 5.77961437868553e-76

FOXS1 3.45997716572505 7.95028109375 1.20024335311741 4.22667708391581e-34 2.15269954465666e-33

UCP2 41.1602379001883 86.8375271875 1.0770673230807 2.43949090419051e-39 1.6405907183878e-38

ADRA2B 0.672999858757062 1.55731130208333 1.21037925669609 2.66698824535156e-11 4.83312020488376e-11

HSPE1P4 0.366781826741996 1.89094234375 2.36611131175351 3.45602172531913e-40 2.43426984781474e-39

AC004947.2 10.7593377589454 4.71851708333333 -1.18918385072809 2.09511473238476e-36 1.20168594844832e-35

LATS2 3.28266911487759 10.0658905729167 1.61653357720754 5.68574911116323e-67 4.12994159070626e-65

AC133435.1 1.06674628060264 2.80522484375 1.39489932969716 6.67125027232662e-24 2.0986617968857e-23

BX322639.1 2.59304392655367 1.17660786458333 -1.140013061332 4.92851863744431e-35 2.62076879987978e-34

PLCB1 21.5865314971751 8.92013395833333 -1.27499417002055 1.32602045156872e-61 5.10504146934169e-60

TTC26 3.54564985875706 10.3438617708333 1.54465292221512 4.64286171005785e-72 8.4641189704437e-70

ADAM11 15.6601735875706 6.92014442708333 -1.17822615139042 1.40176555212592e-13 2.82067205300092e-13

MGARP 1.35816506591337 5.23194994791667 1.94568990960035 2.41431515474776e-33 1.18298261666758e-32

AC138466.1 1.66266958568738 0.6854984375 -1.27827621458231 5.24920951207483e-15 1.12129868899519e-14

EN1 0.4225 9.10268963541667 4.42926964499594 1.30884785163818e-72 2.73460964058561e-70

IGLC3 2.72901285310734 18.9610975520833 2.79659138166784 3.61171881740716e-27 1.31299924554616e-26

MAOB 86.0292630414313 321.05497265625 1.89992095976719 2.65167783367615e-44 2.42061606859146e-43

AC021074.1 0.838771374764595 4.62543614583333 2.46323987625641 6.88631992215601e-27 2.46774174123128e-26

CACNG5 1.11340094161959 2.69078322916667 1.27305296300525 1.22932983690908e-20 3.35772448844365e-20

WNT7B 23.9649219397363 6.84904005208333 -1.80695053864534 7.73668927558177e-57 1.83734019258548e-55

CACNG3 27.8257187382298 7.31670005208333 -1.92715393092024 1.12379335473403e-20 3.07488779153609e-20

AC009902.3 2.27105748587571 7.42645057291667 1.70930862357433 1.60277904127107e-29 6.49317565848269e-29

HOXD13 0.248824905838041 7.866788125 4.98257192556054 3.41341394345739e-57 8.38473345820214e-56

LINC02609 0.536558427495292 1.63432125 1.60688441025143 3.54240460762357e-35 1.89666034203168e-34

SLFN13 0.823986534839925 2.40716817708333 1.54664427244904 1.22411269053401e-19 3.18890101996076e-19

DLL4 3.70975870998117 7.48346338541667 1.01238075719567 5.28003472988124e-26 1.82528807960851e-25

GPR183 10.5118342278719 24.6353786458333 1.22871721646446 2.51482717146428e-33 1.23125885343281e-32

MAGEE1 28.0917905367232 12.7095259895833 -1.14423835797714 3.62840161254573e-45 3.49586155364186e-44

CHEK1 4.16350941619586 11.6769604166667 1.48779278897599 1.04563228123531e-44 9.82492787749903e-44

CDK6 12.1848256120527 30.327701875 1.31555057631432 2.1710599599902e-18 5.34996818924169e-18

FZD6 3.73632175141243 12.3130388020833 1.72049625390661 6.05442524175538e-46 6.19603948103694e-45

PLP2 12.7229019303202 102.936586875 3.01625617876325 9.20408194241695e-79 5.90172081790494e-76

OPCML 34.495270338983 17.1204572916667 -1.01067732993026 2.88127055631848e-29 1.15195354661642e-28

AC005696.4 28.6133777306968 9.38654630208333 -1.60802348034751 2.02837111595365e-59 6.17308689053324e-58

EME1 2.15672834274953 5.04153677083333 1.22501909689333 2.32018196320464e-31 1.02821219270234e-30

SDHAF3 27.7451192561205 58.0895865104167 1.06604555649464 4.12170568886001e-36 2.32957803295902e-35

GGACT 1.23975456685499 4.04966708333333 1.70774877143235 8.86070944546009e-58 2.30440408585077e-56

NODAL 0.852241619585687 2.79881276041667 1.71548056136998 3.49335617355371e-20 9.31578345722519e-20

MIR124-1HG 12.5455219868173 5.08816020833333 -1.30195649695518 1.3424215460646e-36 7.79585529327646e-36

SLC44A3-AS1 0.888448870056497 2.80074479166667 1.6564498716027 4.75110302180954e-55 9.60290877071178e-54

TES 2.4273893126177 6.55110015625 1.43233169419708 1.09665866714783e-48 1.35497461233315e-47

GDF15 1.20355767419962 20.90742234375 4.11863802220208 6.58916930285314e-68 5.42148686666169e-66

MT3 611.450284274953 1392.03203052083 1.18688530167868 1.49012685006064e-26 5.26785337963452e-26

TGFB2-AS1 1.27262438794727 2.68467307291667 1.07693974010548 6.80424862811837e-23 2.05832118496602e-22

GABRE 0.609912711864407 2.45420895833333 2.00858339926043 1.86927598813408e-10 3.27237685928763e-10

HOXB7 1.14675051789077 11.41903390625 3.31581713429307 9.15979547266237e-48 1.0672080000887e-46

IKBIP 5.48638968926554 16.3862240104167 1.57855443945621 7.01197141163464e-60 2.25584097576723e-58

ATF3 13.2086822504708 37.5875279166667 1.50876748941132 1.55780271670963e-47 1.77605158373323e-46

CISH 0.951211958568738 4.20302671875 2.14358986999975 1.32855837969583e-65 8.18031227498144e-64

GLIS3 7.77495202448211 16.68680265625 1.10180186943732 3.81187093836504e-31 1.6748993407112e-30

CD300C 3.57576633709981 8.66572515625 1.27706801565234 9.2865147901533e-43 7.70217406435774e-42

GALNT17 27.6190503295669 13.7405644270833 -1.00722244635535 9.90439927805503e-17 2.27008880285262e-16

GCNT1 0.825586064030132 2.55570286458333 1.6302295897498 2.96457858389051e-25 9.87945120042613e-25

AF228730.4 1.73396224105461 0.815108697916667 -1.08900811633881 4.21118662092335e-13 8.29172122152369e-13

FUT9 21.521252306968 8.55724057291667 -1.33054447448215 1.33895178792509e-58 3.8011921368652e-57

AC107075.1 2.36067984934087 6.35204723958333 1.4280192422802 9.01999287114472e-17 2.07070083257946e-16

FXYD5 18.348825094162 45.8665946354167 1.32175611066672 7.26382138165358e-56 1.59469608727094e-54

AL353807.2 0.812928672316384 1.62698119791667 1.00099690015516 7.47706140237474e-14 1.52168060388703e-13

EMILIN3 3.53680927495292 36.4399760416667 3.36500168275034 1.14979118487785e-50 1.65354733819054e-49

MIR7158 9.03642401129943 3.99492604166667 -1.17758317243784 2.58677116281517e-26 9.04834645834238e-26

HLA-F 12.1641437853107 26.1055020833333 1.10171913125095 5.25976501745682e-37 3.12899545992486e-36

RHBDL3 46.7209040489642 22.7262472395833 -1.03970871745614 1.67169854158893e-33 8.25198682793898e-33

RPL7AP11 1.23145786252354 3.11422041666667 1.3385037951617 1.38082797955219e-21 3.93027648550022e-21

SOHLH1 3.83447693032015 1.09513229166667 -1.80792463235238 1.47176571108493e-20 4.00460687703018e-20

PPIAP31 1.58370442561205 13.8712529166667 3.13072309600787 7.12708855243436e-35 3.76607592021929e-34

LINC02574 0.644485169491525 1.60143354166667 1.31314486291107 2.12398126625142e-20 5.74061506481761e-20

RAB42 1.73557518832392 10.9117265104167 2.65239361862669 1.54004981461891e-73 3.6714392695947e-71

RPS15AP12 1.73193488700565 3.85908072916667 1.15587253211499 4.00070997500335e-24 1.27080973667898e-23

EIF1P7 0.337277024482109 2.24661869791667 2.73574933772182 2.42945017689545e-33 1.19008498523106e-32

RDH5 0.860097080979284 2.63250213541667 1.61386328736471 4.85381589951321e-51 7.23234899433372e-50

ST13P6 1.22000254237288 2.54704119791667 1.06193814088941 1.60997293936419e-13 3.22880142444749e-13

CTSS 25.6743230225989 68.9757208854167 1.42576239344577 1.46557239009449e-44 1.36466292407647e-43

ADGRA1-AS1 4.39961605461394 1.49632958333333 -1.55594964828118 3.03649204454705e-56 6.90263686654675e-55

PFN1 301.854155367232 615.317330885417 1.02747896609309 1.21326075889219e-64 6.5774296826823e-63

CPNE8-AS1 0.938833097928437 2.89408510416667 1.62416673762069 2.81587695564034e-22 8.26799810360526e-22

XPNPEP2 0.662853248587571 1.57707348958333 1.25048848194428 1.39832260258254e-18 3.47066046031998e-18

SNORD89 1.53918578154426 4.2733996875 1.47321688021671 1.21168608376289e-12 2.33460809528245e-12

MAP3K19 0.435872504708098 1.83756942708333 2.0758206539425 2.42540164512489e-12 4.61479009424919e-12

XRCC6P2 0.516971798493409 2.30582182291667 2.1571235495068 1.9039353145311e-31 8.46977922815928e-31

AC002456.1 0.957157297551789 2.572879375 1.42655588111457 1.52694762994928e-57 3.86314162795784e-56

RIMS2 8.32280329566855 2.31169364583333 -1.84811932109591 1.18396595274573e-49 1.56473680819523e-48

SPOCD1 3.30587801318267 36.5017580208333 3.46486055434652 8.33434531901877e-64 4.0358633126863e-62

GINS2 5.66184830508475 17.3754652604167 1.61770660769099 2.38778258178246e-41 1.81524190957665e-40

FAM192BP 0.587525753295669 1.64972364583333 1.48950037451616 4.27773547049046e-20 1.13570090053926e-19

AC078817.1 1.32595541431262 4.52044421875 1.76943228638558 8.71860372389885e-23 2.62759215957697e-22

KCNJ4 18.7778808851224 7.97109494791667 -1.23618444319291 6.98938193749341e-14 1.42336687944858e-13

ARSD 4.03072024482109 14.849985 1.88135191331879 4.77511553222178e-64 2.36782062191104e-62

PCOLCE 9.92537829566855 47.81334609375 2.26821937724547 1.92930639186669e-67 1.52661499390473e-65

ELDR 0.0450779190207156 13.9743159895833 8.27614103647429 1.82366406277288e-39 1.23582482679525e-38

NCR3LG1 5.00230889830508 1.83995869791667 -1.4429207662869 4.88908322002608e-34 2.47853060186437e-33

COLEC12 8.57459001883239 20.7061658854167 1.27192083991682 5.43071382992249e-21 1.50565397454956e-20

CAMK4 9.11571313559322 3.33028401041667 -1.45271030760527 1.97626738054965e-21 5.58406394664357e-21

WDR93 0.762716713747646 1.5346190625 1.00866136114718 1.34007886480552e-09 2.25938584559422e-09

CDCA7 8.05081958568738 20.1889269791667 1.32635667057308 5.55940774447613e-34 2.81221945072181e-33

TP73 1.23690913370998 4.10429786458333 1.73039591624352 6.02210429716354e-23 1.82498418197125e-22

TRAF1 6.64976676082863 14.0248038020833 1.07660494421796 1.14473319711331e-38 7.41425071414908e-38

SLC1A5 13.0059559322034 27.9191514583333 1.10208265400025 3.40181998733795e-43 2.89637557987863e-42

IGKV3-11 1.0751918079096 11.42707375 3.40979004866016 9.9595837828051e-19 2.48989594570128e-18

C5orf63 0.948886676082862 2.47210526041667 1.38143246940068 1.58428456186887e-42 1.29550446033209e-41

NIPSNAP3A 18.2796546610169 37.8603638541667 1.05044946074127 1.05872718553112e-52 1.79626204515978e-51

CDC20 8.18228813559322 47.84755828125 2.54786905702797 1.42414281200029e-54 2.77588423366304e-53

RGS22 0.304010734463277 2.81847322916667 3.21271969382422 6.37327689434796e-38 3.96508879699266e-37

BST2 96.4906266949152 327.3582496875 1.76240962861417 2.23590282920112e-49 2.88726479923575e-48

EOLA1 3.54452410546139 7.13653989583333 1.00963282113007 1.62205564226764e-41 1.2458539722415e-40

FANCA 1.80795103578154 4.31013723958333 1.25337820088017 6.67540037665569e-29 2.626514388572e-28

AC113404.3 1.22923290960452 5.98099046875 2.28262612146772 1.85433874732746e-41 1.41957303444027e-40

IGHV3-30 0.861739124293785 4.85957927083333 2.49550832354809 1.36564829115784e-19 3.54915862670581e-19

AL121894.2 9.80665889830508 24.3213263541667 1.31038830470666 2.20561455698695e-18 5.43151935997515e-18

KCNIP2 64.9826278248588 17.7904071875 -1.86895455438185 2.41501178764227e-59 7.31386713211857e-58

CD163 13.2376168079096 93.76922265625 2.82447105824342 2.73770008081379e-41 2.0736265988893e-40

AC104407.1 0.636691148775894 3.37545807291667 2.40641768585139 9.16710509369975e-17 2.10395358204575e-16
