# Supplementary material for: Associations between migrasome-related genes and long non-coding rnas in glioma and their prognostic relevance to the tumor microenvironment
Source: IBRO Neurosci Rep. 2026 Jun 24;21:279–90. doi: 10.1016/j.ibneur.2026.06.013 (PMC13356737; doi:10.1016/j.ibneur.2026.06.013)
Supplement: Supplementary file 6 — Supplementary material [file mmc6.docx]

ONTOLOGY ID Description GeneRatio BgRatio pvalue p.adjust qvalue geneID Count

BP GO:0002443 leukocyte mediated immunity 143/2475 466/18870 9.75195191292671e-24 5.8550719285212e-20 4.25287755529004e-20 KLRC2/IL7R/BATF/IRF7/IL18/EMP2/MR1/KLRC3/NFKBIZ/SPI1/NKG7/IGHV5-51/C1RL/IGHV2-5/ITGB2/CFI/IGHV3-33/IGHV3-74/CHGA/ACE/IGHG4/C1R/TREM2/CD40/FCGR1A/CD70/TFRC/CPLX2/ICAM1/HPX/BCL3/IGHV3-7/VAMP8/FOXJ1/IGHV3-21/CTSC/CD74/KLRC4/STXBP1/HAVCR2/RIPK3/IGHA1/AHR/GZMB/IL13RA2/HLA-C/IL1R1/S100A13/MICA/ADGRE2/ARID5A/CLEC7A/RAB27A/SLAMF9/CD8A/IGHV4-34/JAG1/GAPT/SPON2/KLRC4-KLRK1/IGHV3-11/IGHV3-23/IGHG2/IGHV3-49/C7/HLA-DRB1/MICB/JAK3/PRAM1/F2RL1/IGHG3/FCGR2B/SCIMP/IGKC/SCNN1B/IGHV4-39/SASH3/TLR8/IGHA2/FZD5/FES/LYN/EXO1/IGHV4-59/SPN/SERPING1/HLA-DRA/NOS2/C2/CD300A/NECTIN2/IGHG1/C1S/TYROBP/HLA-B/LILRB1/ZP3/RAC2/HLA-A/RSAD2/LAT2/MILR1/PRF1/ULBP2/CSF2RB/IGHV1-3/IGHM/FCER1G/CD2/IGHV3-15/IL9/ULBP3/UNC93B1/C3/IGHV3-48/C1QB/PTPRC/CXCL6/IGHV1-18/B2M/IL6/PIK3CG/C1QC/C1QA/FCGR3A/PIK3R6/FADD/SWAP70/IGLC2/HFE/MYO1G/TCIRG1/MYD88/TREM1/IL4I1/GRP/IL2RB/CLCF1/IGLC3/HLA-F/NCR3LG1/BST2/IGHV3-30 143

BP GO:0030198 extracellular matrix organization 109/2475 321/18870 3.81390826518502e-22 9.97928648331984e-19 7.24853324106632e-19 COL8A1/CAV1/FERMT1/EFEMP2/ITGB3/LOXL2/FMOD/COL14A1/COL8A2/LOXL3/NPNT/CTSK/ECM2/MPZL3/LCP1/NID1/KIF9/COL5A3/COL22A1/MMP12/OLFML2A/KLK7/HAS3/SERPINH1/MMP13/BCL3/MMP2/SMOC1/SLC39A8/COL3A1/MMP1/AEBP1/LAMA2/RUNX1/LOXL4/HPSE2/ADAMTSL4/CCN1/CYP1B1/NID2/BMP2/COL12A1/MMP9/NR2E1/FBLN1/MFAP4/ADAMTS20/LOX/SPINT1/ENG/COL9A3/TNFRSF1A/MMP14/SMPD3/SLC2A10/MMP25/COL15A1/MMP19/EGFLAM/CAV2/PDPN/SFRP2/HAS2/COLGALT1/COL1A1/LAMC1/ADAMTS14/COL4A1/MIR27B/CST3/C6orf15/COL4A2/COL1A2/MMP7/EMILIN1/TNR/LAMB1/TNFRSF11B/ADAMTS2/FBLN5/ADAMTS3/TGFBI/OLFML2B/ELN/LUM/COL5A2/LOXL1/RXFP1/FAP/POSTN/PRDX4/COL27A1/ITGB1/DPP4/CARMIL2/COL5A1/FKBP10/IL6/VWA1/TGFB2/PTX3/ADAMTS7/MMP11/ANXA2/PLOD3/COL2A1/IBSP/THSD4/CTSS 109

BP GO:0043062 extracellular structure organization 109/2475 322/18870 5.039520613921e-22 9.97928648331984e-19 7.24853324106632e-19 COL8A1/CAV1/FERMT1/EFEMP2/ITGB3/LOXL2/FMOD/COL14A1/COL8A2/LOXL3/NPNT/CTSK/ECM2/MPZL3/LCP1/NID1/KIF9/COL5A3/COL22A1/MMP12/OLFML2A/KLK7/HAS3/SERPINH1/MMP13/BCL3/MMP2/SMOC1/SLC39A8/COL3A1/MMP1/AEBP1/LAMA2/RUNX1/LOXL4/HPSE2/ADAMTSL4/CCN1/CYP1B1/NID2/BMP2/COL12A1/MMP9/NR2E1/FBLN1/MFAP4/ADAMTS20/LOX/SPINT1/ENG/COL9A3/TNFRSF1A/MMP14/SMPD3/SLC2A10/MMP25/COL15A1/MMP19/EGFLAM/CAV2/PDPN/SFRP2/HAS2/COLGALT1/COL1A1/LAMC1/ADAMTS14/COL4A1/MIR27B/CST3/C6orf15/COL4A2/COL1A2/MMP7/EMILIN1/TNR/LAMB1/TNFRSF11B/ADAMTS2/FBLN5/ADAMTS3/TGFBI/OLFML2B/ELN/LUM/COL5A2/LOXL1/RXFP1/FAP/POSTN/PRDX4/COL27A1/ITGB1/DPP4/CARMIL2/COL5A1/FKBP10/IL6/VWA1/TGFB2/PTX3/ADAMTS7/MMP11/ANXA2/PLOD3/COL2A1/IBSP/THSD4/CTSS 109

BP GO:0045229 external encapsulating structure organization 109/2475 323/18870 6.64842537196525e-22 9.97928648331984e-19 7.24853324106632e-19 COL8A1/CAV1/FERMT1/EFEMP2/ITGB3/LOXL2/FMOD/COL14A1/COL8A2/LOXL3/NPNT/CTSK/ECM2/MPZL3/LCP1/NID1/KIF9/COL5A3/COL22A1/MMP12/OLFML2A/KLK7/HAS3/SERPINH1/MMP13/BCL3/MMP2/SMOC1/SLC39A8/COL3A1/MMP1/AEBP1/LAMA2/RUNX1/LOXL4/HPSE2/ADAMTSL4/CCN1/CYP1B1/NID2/BMP2/COL12A1/MMP9/NR2E1/FBLN1/MFAP4/ADAMTS20/LOX/SPINT1/ENG/COL9A3/TNFRSF1A/MMP14/SMPD3/SLC2A10/MMP25/COL15A1/MMP19/EGFLAM/CAV2/PDPN/SFRP2/HAS2/COLGALT1/COL1A1/LAMC1/ADAMTS14/COL4A1/MIR27B/CST3/C6orf15/COL4A2/COL1A2/MMP7/EMILIN1/TNR/LAMB1/TNFRSF11B/ADAMTS2/FBLN5/ADAMTS3/TGFBI/OLFML2B/ELN/LUM/COL5A2/LOXL1/RXFP1/FAP/POSTN/PRDX4/COL27A1/ITGB1/DPP4/CARMIL2/COL5A1/FKBP10/IL6/VWA1/TGFB2/PTX3/ADAMTS7/MMP11/ANXA2/PLOD3/COL2A1/IBSP/THSD4/CTSS 109

BP GO:0002460 adaptive immune response based on somatic recombination of immune receptors built from immunoglobulin superfamily domains 119/2475 380/18870 9.73401978738797e-21 1.16886109606955e-17 8.49011452192597e-18 ASCL2/IL7R/BATF/IRF7/IL18/EMP2/MR1/SLC11A1/CXCL13/LEF1/NFKBIZ/IGHV5-51/C1RL/RELB/IGHV2-5/LOXL3/CFI/IGHV3-33/IGHV3-74/IGHG4/ANXA1/C1R/TREM2/CD40/FCGR1A/CD70/TFRC/ICAM1/HPX/BCL3/IGHV3-7/FOXJ1/IGHV3-21/CTSC/CD74/HAVCR2/RIPK3/IGHA1/AHR/IL13RA2/HLA-C/IL1R1/MICA/ARID5A/CLEC7A/RAB27A/CD8A/IGHV4-34/JAG1/GAPT/IGHV3-11/CD274/IGHV3-23/IGHG2/IGHV3-49/C7/HLA-DRB1/TNFAIP3/MICB/JAK3/IGHG3/FCGR2B/IGKC/IGHV4-39/SASH3/TLR8/IGHA2/FZD5/EXO1/IGHV4-59/SOCS3/SPN/SERPING1/HLA-DRA/C2/NECTIN2/IGHG1/C1S/HLA-B/LILRB1/ZP3/HLA-A/RSAD2/PRF1/ULBP2/CSF2RB/IGHV1-3/IGHM/FCER1G/IGHV3-15/IL9/ULBP3/UNC93B1/C3/IGHV3-48/C1QB/PTPRC/IGHV1-18/B2M/IL6/C1QC/PLA2G4A/C1QA/FCGR3A/ZC3H12A/FADD/SWAP70/IGLC2/HFE/MYO1G/TCIRG1/MYD88/IL4I1/IL2RB/CLCF1/IGLC3/HLA-F/NCR3LG1/IGHV3-30 119

BP GO:0002449 lymphocyte mediated immunity 113/2475 368/18870 5.08978106238635e-19 5.09317424976127e-16 3.69946718271344e-16 KLRC2/IL7R/BATF/IRF7/IL18/EMP2/MR1/KLRC3/NFKBIZ/NKG7/IGHV5-51/C1RL/IGHV2-5/CFI/IGHV3-33/IGHV3-74/IGHG4/C1R/TREM2/CD40/FCGR1A/CD70/TFRC/ICAM1/HPX/BCL3/IGHV3-7/FOXJ1/IGHV3-21/CTSC/CD74/KLRC4/HAVCR2/RIPK3/IGHA1/AHR/GZMB/IL13RA2/HLA-C/IL1R1/MICA/ARID5A/RAB27A/CD8A/IGHV4-34/JAG1/GAPT/KLRC4-KLRK1/IGHV3-11/IGHV3-23/IGHG2/IGHV3-49/C7/HLA-DRB1/MICB/IGHG3/FCGR2B/IGKC/IGHV4-39/SASH3/TLR8/IGHA2/FZD5/EXO1/IGHV4-59/SPN/SERPING1/HLA-DRA/C2/NECTIN2/IGHG1/C1S/HLA-B/LILRB1/ZP3/HLA-A/RSAD2/PRF1/ULBP2/CSF2RB/IGHV1-3/IGHM/FCER1G/CD2/IGHV3-15/IL9/ULBP3/UNC93B1/C3/IGHV3-48/C1QB/PTPRC/IGHV1-18/B2M/IL6/C1QC/C1QA/FCGR3A/PIK3R6/FADD/SWAP70/IGLC2/HFE/MYO1G/TCIRG1/MYD88/IL4I1/IL2RB/CLCF1/IGLC3/HLA-F/NCR3LG1/IGHV3-30 113

BP GO:0002274 myeloid leukocyte activation 81/2475 241/18870 1.69342597502697e-16 1.45247565058028e-13 1.05501711496793e-13 BATF/PYCARD/TLR1/IL18/SLC11A1/PLA2G2A/SPI1/RELB/ITGB2/CXCL8/HAMP/CHGA/IFNGR2/ANXA1/THBS1/TREM2/CPLX2/S100A12/VAMP8/CTSC/CD74/STXBP1/HAVCR2/CD33/IL13RA2/SBNO2/RHOH/S100A13/LCP2/ADGRE2/CNR1/CD93/BATF2/TAFA3/BATF3/CD300LF/PRAM1/C5AR1/TLR2/PLSCR1/F2RL1/NMI/FCGR2B/CCL5/SPHK1/SCNN1B/VSIG4/FES/DHRS2/FN1/LYN/MAPT/LRRK2/CD300A/NECTIN2/TYROBP/LILRA2/RAC2/CST7/IFI35/ADAM9/CXCR2/LAT2/MILR1/LTBR/FCER1G/CD2/LRFN5/PTPRC/CXCL6/PRKCE/IL6/PIK3CG/LBP/PLA2G4A/C1QA/FCGR3A/ZC3H12A/MYD88/GRP/CAMK4 81

BP GO:0050808 synapse organization 127/2475 483/18870 3.14804564019281e-15 2.36260825296471e-12 1.71609909043669e-12 IL1RAPL1/UNC13A/SYN1/ITGB3/HIP1R/CDK5R1/SEMA3E/LGI2/DNM3/CACNG2/ROR2/PTPRT/PRRT1/FGF13/LINGO2/ADGRB3/ITGA3/TREM2/NEFL/NTNG2/CHRNB2/NRP1/FRMPD4/CHRNA1/NRG3/SLITRK4/FLNA/SLIT1/NEUROD2/DSCAM/SHANK2/NEGR1/CUX2/GABRB3/C1QL3/RAB3A/CDH9/GAP43/UNC13C/SHANK1/SRPX2/SHISA7/CDH6/CBLN3/OXTR/LRRTM3/OPHN1/CLSTN2/DLGAP3/SNCG/NRXN1/CBLN2/ERC2/C5AR1/TLR2/SLC8A3/NEFH/FCGR2B/BSN/DKK1/L1CAM/GRM5/ABI3/FZD5/SRGN/ASIC2/COL4A1/IL10RA/SLC8A2/MAPT/FRRS1L/LRRK2/SNAP25/ZDHHC12/LILRB2/ACTN1/ARC/PAK3/TNC/SLITRK1/TNR/RAP1B/GABRA1/PPFIA2/IGSF9/CBLN1/SNCB/ZNF804A/GABRB2/SEMA3F/CAMKV/IL1RAP/LZTS1/GABRG2/NPTX1/RAP2A/SPTBN2/SPTB/CDH8/AMIGO2/LRFN5/POU4F1/CTTNBP2/ICAM5/PCLO/C3/C1QB/ITGB1/LRTM2/FZD1/GDNF/NTRK1/ARHGAP44/C1QC/C1QA/SEZ6L/TUBA1A/INA/ANK3/NEURL1/SLITRK5/GRIN2B/GPR158/NTNG1/WNT7B/PFN1/CDC20 127

BP GO:0045785 positive regulation of cell adhesion 126/2475 482/18870 6.51569195051459e-15 4.34669049676551e-12 3.15725283637216e-12 COL8A1/CAV1/CD276/ITGA5/WNT4/IL7R/FERMT1/EFEMP2/MMRN1/MDK/EFNB1/ITGB3/PYCARD/COL26A1/IL18/EMP2/CXCL13/LEF1/NFKBIZ/IL1A/ITGB2/NPNT/LCK/ITGA2/ECM2/NID1/ANXA1/ACTL6B/TESPA1/ITGA3/HLA-DMA/CD70/TFRC/RET/NRP1/VAV3/HLA-DQA2/HLA-DQA1/CD74/SOCS1/HAVCR2/FLNA/MAP3K8/CD44/LAMA2/RHOH/RUNX1/HLA-DPA1/DUSP26/CCN1/CCL2/HLA-DOA/S100A10/KLRC4-KLRK1/CD274/ITGA4/GLI3/HLA-DRB1/CD3E/ANGPT1/VEGFA/EGFLAM/PDCD1LG2/CCL5/PDPN/SFRP2/IL2RG/IGF2/HAS2/SASH3/TNFRSF18/DUSP10/LAMC1/FN1/LYN/EMILIN2/F11R/FSTL3/MIR27B/BTN2A2/RUNX3/LIF/SPN/HLA-DPB1/LILRB2/HLA-DRA/CD36/HLA-DQB1/EMILIN1/IGFBP2/LAMB1/LILRB1/ZP3/SDC4/LGALS1/HLA-DOB/HLA-A/ADAM9/HLA-DRB5/RRAS/EFNB2/ADAM19/ALOX5/HLA-DQB2/HLA-DMB/PTPRC/DPP4/WNT10B/PRKCE/B2M/IL6/CHST2/IL2RA/TGFB2/PLAUR/RHOD/PIK3R6/FADD/ANK3/TNFRSF14/SAA1/IBSP/IL4I1/CDK6/NODAL/GCNT1 126

BP GO:0042742 defense response to bacterium 96/2475 330/18870 1.06932056768784e-14 6.42020068839781e-12 4.66336327571656e-12 RARRES2/GBP5/IL7R/PYCARD/IL18/MR1/SLC11A1/PLA2G2A/CXCL13/NFKBIZ/IGKV3-20/GSDMD/PI3/CYBA/OAS2/CCL20/HAMP/GBP2/CHGA/VGF/IGHG4/KLK7/TREM2/FCGR1A/GSDMA/WFDC2/BCL3/SHC1/S100A12/PLAC8/HAVCR2/H2BC8/IGHA1/PPBP/TNFSF8/MICA/GBP1/SSC5D/GBP4/OAS1/CASP7/S100A8/SPON2/KLRC4-KLRK1/S100A9/TNFRSF1A/CASP1/SLAMF8/IGHG2/C5AR1/TLR2/F2RL1/IGHG3/SCNN1B/STAB1/H2BC11/IGHA2/ACP5/HP/JCHAIN/EMILIN2/LYZ/FPR2/H2BC12/OAS3/GBP3/SERPINE1/SPN/SLPI/CEBPB/ANG/LTF/NOS2/CD36/ISG15/EMILIN1/IGHG1/RNASE3/HLA-A/EPHA2/IGHM/FCER1G/H2BC4/RNASE6/HMGB2/CXCL6/IL6/LBP/GNLY/CASP4/TNFRSF14/MYD88/AQP1/TREM1/NCR3LG1/COLEC12 96

BP GO:0042060 wound healing 114/2475 423/18870 1.34491725339647e-14 7.34080289944763e-12 5.33204993380054e-12 CAV1/F5/TIMP1/THBD/ITGA5/WNT4/FERMT1/MMRN1/ITGB3/SLC11A1/IL1A/MIR221/PLAT/LCK/VWF/ITGA2/ANXA1/HMOX1/DGKI/MMP12/C1QTNF1/THBS1/CD40/CLDN1/FZD7/TNFRSF12A/VAV3/F3/COL3A1/STXBP1/FLNA/CD44/HSPB1/CCN1/CLEC7A/RAB27A/RAB3A/CCN4/S100A10/MYOF/FBLN1/CASP7/CD151/GPR4/MCAM/TNFAIP3/VEGFA/SYT7/ENTPD2/PLSCR1/F2RL1/GPX1/PDPN/CXCR4/FN1/AJUBA/LYN/TFPI2/EMILIN2/F11R/MYL9/SERPINE1/PDGFA/VKORC1/SERPING1/PLAU/PPIA/CD36/DCBLD2/EMILIN1/F2RL3/P2RY1/SDC4/PROS1/F2RL2/TMEFF2/DGKB/ARHGEF19/MYL12A/SERPINA1/RHOC/FCER1G/ANXA5/FAP/ALOX5/GATA4/PLEK/ITGB1/DGKK/AJAP1/CARMIL2/COL5A1/FKBP10/PRKCE/F13A1/IL6/PPARG/PIK3CG/PLA2G4A/CD109/TGFB2/TFPI/PLAUR/PROCR/ELK3/CHMP4BP1/CLIC1/ANXA2/SAA1/NOG/AQP1/FERMT3/ADRA2B/FZD6 114

BP GO:0048002 antigen processing and presentation of peptide antigen 36/2475 71/18870 2.78293631779914e-14 1.39239580433883e-11 1.01137764602121e-11 PYCARD/MR1/SLC11A1/ACE/TREM2/FCGR1A/HLA-DMA/HLA-DQA2/HLA-DQA1/CD74/HLA-C/MICA/HLA-DPA1/HLA-DOA/HLA-DRB1/MICB/FCGR2B/CTSL/HLA-DPB1/HLA-DRA/HLA-DQB1/HLA-B/HLA-DOB/HLA-A/ULBP2/TAP1/HLA-DRB5/FCER1G/ULBP3/UNC93B1/HLA-DQB2/HLA-DMB/B2M/HFE/HLA-F/CTSS 36

BP GO:0099177 regulation of trans-synaptic signaling 125/2475 490/18870 5.79032742729796e-14 2.67424045180746e-11 1.94245558957858e-11 AKAP5/UNC13A/STX1A/SYN1/NGFR/SLC4A10/GRIA4/JPH4/CACNG2/CA2/STX1B/PLAT/ROR2/CPEB3/ACE/VGF/CACNA1B/PRRT1/PRKCG/DGKI/GABBR1/NEFL/CPLX2/NTNG2/CHRNB2/RIMS1/KCNB1/NRG3/STXBP1/LAMA2/NEUROD2/ASIC1/SHANK2/CUX2/CNR1/CCL2/RAB3A/GRIA2/NR2E1/MYOF/UNC13C/SHANK1/PRKCB/SHISA7/OXTR/CHRM1/OPHN1/CLSTN2/DLGAP3/GRIN3A/SNCG/NRXN1/CBLN2/ERC2/PHF24/SYT7/SLC8A3/GRIN2A/NEFH/HTR2A/NALCN/DKK1/HRH1/GRM5/DLGAP2/SRGN/S100B/SYT1/SLC8A2/MAPT/RASGRF1/FRRS1L/SYT4/DLGAP1/GRIN1/LRRK2/CAMK2A/SNAP25/ZDHHC12/LILRB2/ARC/GRM2/TNR/RAP1B/TYROBP/PPFIA2/SV2B/CBLN1/P2RY1/RASGRF2/CAMKV/SYP/LZTS1/CELF4/FABP5/MIR320E/SYN3/GRIK1/GRM1/CNTN4/JPH3/ITGB1/ADORA1/MAPK8IP2/PRKCE/NTRK1/ARHGAP44/SYNGR1/RGS4/RIMS3/INA/NEURL1/CA7/NOG/GRIN2B/CPLX1/GPR158/NTNG1/PTN/PLCB1/CACNG5/CACNG3/PFN1/RIMS2/CDC20 125

BP GO:0007159 leukocyte cell-cell adhesion 111/2475 419/18870 1.05332195948821e-13 4.51724646054803e-11 3.28113750237569e-11 CAV1/CD276/ASCL2/ITGA5/IL7R/MDK/EFNB1/PYCARD/IL18/PLA2G2A/LEF1/NFKBIZ/IL1A/MSN/MIR221/LOXL3/ITGB2/LCK/LRRC32/ANXA1/ACTL6B/TESPA1/HLA-DMA/CD70/TFRC/PAWR/ICAM1/HLA-DQA2/FOXJ1/SLC39A8/HLA-DQA1/CD74/SOCS1/HAVCR2/MAP3K8/CD44/SELL/TWSG1/LGALS3/RHOH/RUNX1/HLA-DPA1/CCL2/HLA-DOA/PLA2G5/S100A8/KLRC4-KLRK1/CD274/ITGA4/GLI3/S100A9/GPNMB/HLA-DRB1/CD3E/JAK3/IDO1/PDCD1LG2/FCGR2B/CCL5/IL2RG/IGF2/VSIG4/HAS2/SASH3/DUSP10/LYN/F11R/BTN2A2/RUNX3/SPN/CEBPB/HLA-DPB1/LILRB2/HLA-DRA/HLA-DQB1/CD300A/LAPTM5/IGFBP2/LILRB1/ZP3/RAC2/SDC4/LGALS1/CASP3/HLA-DOB/HLA-A/FGL2/HLA-DRB5/EFNB2/ALOX5/HLA-DQB2/HLA-DMB/PTPRC/ITGB1/DPP4/WNT10B/B2M/IL6/CHST2/IL2RA/ZC3H12A/PIK3R6/FADD/ITGAL/TNFRSF14/IRF1/HFE/IL4I1/FERMT3/GCNT1/FUT9 111

BP GO:0050804 modulation of chemical synaptic transmission 124/2475 489/18870 1.16186911452405e-13 4.65057477573491e-11 3.37798157296359e-11 AKAP5/UNC13A/STX1A/SYN1/NGFR/SLC4A10/GRIA4/JPH4/CACNG2/CA2/STX1B/PLAT/ROR2/CPEB3/ACE/VGF/CACNA1B/PRRT1/PRKCG/DGKI/GABBR1/NEFL/CPLX2/NTNG2/CHRNB2/RIMS1/KCNB1/NRG3/STXBP1/LAMA2/NEUROD2/ASIC1/SHANK2/CUX2/CNR1/CCL2/RAB3A/GRIA2/NR2E1/MYOF/UNC13C/SHANK1/PRKCB/SHISA7/OXTR/CHRM1/OPHN1/CLSTN2/DLGAP3/GRIN3A/SNCG/NRXN1/CBLN2/ERC2/PHF24/SYT7/SLC8A3/GRIN2A/NEFH/HTR2A/NALCN/DKK1/HRH1/GRM5/DLGAP2/SRGN/S100B/SYT1/SLC8A2/MAPT/RASGRF1/FRRS1L/SYT4/DLGAP1/GRIN1/LRRK2/CAMK2A/SNAP25/ZDHHC12/LILRB2/ARC/GRM2/TNR/RAP1B/TYROBP/PPFIA2/SV2B/CBLN1/P2RY1/RASGRF2/CAMKV/SYP/LZTS1/CELF4/MIR320E/SYN3/GRIK1/GRM1/CNTN4/JPH3/ITGB1/ADORA1/MAPK8IP2/PRKCE/NTRK1/ARHGAP44/SYNGR1/RGS4/RIMS3/INA/NEURL1/CA7/NOG/GRIN2B/CPLX1/GPR158/NTNG1/PTN/PLCB1/CACNG5/CACNG3/PFN1/RIMS2/CDC20 124

BP GO:0019882 antigen processing and presentation 47/2475 116/18870 1.63033929983833e-13 6.11784822264333e-11 4.44374718370407e-11 PYCARD/MR1/SLC11A1/RELB/PSMB8/ACE/THBS1/TREM2/RAB32/FCGR1A/HLA-DMA/ICAM1/HLA-DQA2/HLA-DQA1/CD74/HLA-C/MICA/HLA-DPA1/RAB27A/HLA-DOA/CD8A/HLA-DRB1/MICB/RAB34/FCGR2B/CTSL/RAB3C/HLA-DPB1/LILRB2/HLA-DRA/HLA-DQB1/HLA-B/HLA-DOB/HLA-A/FGL2/ULBP2/TAP1/HLA-DRB5/FCER1G/ULBP3/UNC93B1/HLA-DQB2/HLA-DMB/B2M/HFE/HLA-F/CTSS 47

BP GO:0019724 B cell mediated immunity 68/2475 208/18870 2.04034226116455e-13 7.20600878590116e-11 5.23414116904318e-11 BATF/IRF7/NFKBIZ/IGHV5-51/C1RL/IGHV2-5/CFI/IGHV3-33/IGHV3-74/IGHG4/C1R/TREM2/CD40/FCGR1A/CD70/TFRC/HPX/BCL3/IGHV3-7/FOXJ1/IGHV3-21/CD74/IGHA1/IL13RA2/IGHV4-34/GAPT/IGHV3-11/IGHV3-23/IGHG2/IGHV3-49/C7/IGHG3/FCGR2B/IGKC/IGHV4-39/TLR8/IGHA2/EXO1/IGHV4-59/SERPING1/C2/NECTIN2/IGHG1/C1S/ZP3/CSF2RB/IGHV1-3/IGHM/FCER1G/IGHV3-15/IL9/C3/IGHV3-48/C1QB/PTPRC/IGHV1-18/C1QC/C1QA/FCGR3A/SWAP70/IGLC2/TCIRG1/MYD88/IL2RB/CLCF1/IGLC3/NCR3LG1/IGHV3-30 68

BP GO:0016064 immunoglobulin mediated immune response 67/2475 205/18870 3.11047002884324e-13 9.94298572089924e-11 7.22216589671544e-11 BATF/IRF7/NFKBIZ/IGHV5-51/C1RL/IGHV2-5/CFI/IGHV3-33/IGHV3-74/IGHG4/C1R/TREM2/CD40/FCGR1A/TFRC/HPX/BCL3/IGHV3-7/FOXJ1/IGHV3-21/CD74/IGHA1/IL13RA2/IGHV4-34/GAPT/IGHV3-11/IGHV3-23/IGHG2/IGHV3-49/C7/IGHG3/FCGR2B/IGKC/IGHV4-39/TLR8/IGHA2/EXO1/IGHV4-59/SERPING1/C2/NECTIN2/IGHG1/C1S/ZP3/CSF2RB/IGHV1-3/IGHM/FCER1G/IGHV3-15/IL9/C3/IGHV3-48/C1QB/PTPRC/IGHV1-18/C1QC/C1QA/FCGR3A/SWAP70/IGLC2/TCIRG1/MYD88/IL2RB/CLCF1/IGLC3/NCR3LG1/IGHV3-30 67

BP GO:0030199 collagen fibril organization 33/2475 65/18870 3.146514468639e-13 9.94298572089924e-11 7.22216589671544e-11 EFEMP2/LOXL2/FMOD/COL14A1/LOXL3/COL5A3/SERPINH1/COL3A1/AEBP1/LOXL4/CYP1B1/COL12A1/LOX/SFRP2/COLGALT1/COL1A1/ADAMTS14/COL1A2/EMILIN1/ADAMTS2/ADAMTS3/LUM/COL5A2/LOXL1/COL27A1/COL5A1/FKBP10/TGFB2/ADAMTS7/MMP11/ANXA2/PLOD3/COL2A1 33

BP GO:0050900 leukocyte migration 105/2475 396/18870 4.63280524957606e-13 1.39076813592273e-10 1.01019537626282e-10 ASCL2/RARRES2/MDK/ITGB3/PYCARD/EMP2/CXCL13/SPI1/IL1A/MSN/CCL26/ITGB2/ROR2/LCK/CXCL8/ITGA2/CCL20/CHGA/ANXA1/HMOX1/THBS1/ITGA3/TREM2/ICAM1/RET/MMP2/VAV3/S100A12/FOXJ1/CD74/SELL/RIPK3/PPBP/LGALS3/IL1R1/ADGRE2/CCL2/IL17RC/S100A8/ECM1/KLRC4-KLRK1/ITGA4/S100A9/PTGER4/SLAMF8/MMP14/SMPD3/HSD3B7/VEGFA/CXCL10/C5AR1/F2RL1/CCL5/PLVAP/CXCR4/DPEP1/BST1/TNFRSF18/LYN/FPR2/F11R/SERPINE1/CCL18/SPN/PPIA/CXCL9/EMILIN1/CD300A/CCR5/THBS4/ZP3/TNFAIP6/RAC2/PDGFD/CXCR2/HOXA7/FCER1G/ITGA7/ALOX5/MCOLN2/ITGB1/ADORA1/DPP4/CXCL6/CXCL11/IL6/PIK3CG/LBP/ITGA1/CHST2/TGFB2/FADD/ITGAL/SWAP70/TNFRSF14/MYO1G/SAA1/MYD88/TREM1/PTN/CCR1/PLCB1/GPR183/GCNT1/FUT9 105

BP GO:0050863 regulation of T cell activation 102/2475 381/18870 5.27006159196907e-13 1.50673570467535e-10 1.09442933210666e-10 CAV1/CD276/ASCL2/IL7R/MDK/BATF/EFNB1/PYCARD/IL18/PLA2G2A/LEF1/NFKBIZ/IL1A/LOXL3/FANCD2/LCK/LRRC32/ANXA1/ACTL6B/TESPA1/CGAS/HLA-DMA/CD70/TFRC/PAWR/HLA-DQA2/FOXJ1/HLA-DQA1/CD74/SOCS1/HAVCR2/MAP3K8/TWSG1/RIPK3/LGALS3/TNFSF8/RHOH/RUNX1/HLA-DPA1/CCL2/HLA-DOA/PLA2G5/KLRC4-KLRK1/CD274/GLI3/GPNMB/TOX/HLA-DRB1/CD3E/JAK3/IDO1/PDCD1LG2/FCGR2B/CCL5/IL2RG/IGF2/VSIG4/SASH3/DUSP10/LYN/BTN2A2/RUNX3/SPN/CEBPB/HLA-DPB1/LILRB2/HLA-DRA/HLA-DQB1/CD300A/LAPTM5/IGFBP2/LILRB1/ZP3/RAC2/SDC4/LGALS1/CASP3/HLA-DOB/TCF7/HLA-A/FGL2/HLA-DRB5/EFNB2/CD2/HLA-DQB2/HLA-DMB/PTPRC/DPP4/WNT10B/B2M/IL6/LMO1/IL2RA/ZC3H12A/PIK3R6/FADD/TNFRSF14/IRF1/HFE/IL4I1/CAMK4/FANCA 102

BP GO:0002237 response to molecule of bacterial origin 98/2475 369/18870 2.52400878575179e-12 6.68394164418627e-10 4.85493359372063e-10 THBD/MAP2K3/LY96/GBP5/PYCARD/TLR1/SLC11A1/CXCL13/NFKBIZ/SPI1/IL1A/TSPO/CXCL8/GBP2/ACE/SOD2/LITAF/ABCC8/TREM2/CD40/CLDN1/GCH1/HAVCR2/CITED1/AHR/PPBP/SBNO2/ARID5A/SSC5D/CNR1/CCL2/CMPK2/MMP9/PALM3/CD180/CASP7/S100A8/SPON2/KLRC4-KLRK1/CD274/S100A9/PTGER4/CASP1/TNFAIP3/CASP8/CXCL10/C5AR1/TLR2/ZFP36/CARD16/IDO1/PDCD1LG2/FCGR2B/SCIMP/FZD5/DUSP10/ACP5/LYN/GJB2/IL10RA/CYP27B1/GBP3/CSF3/SERPINE1/SLPI/CEBPB/CD14/LTF/MGST2/LILRB2/CXCL9/NOS2/CD36/C2/CCR5/LILRB1/LILRA2/CASP3/TRIM6/TRIM5/ADAM9/VIM/CSF2RB/LOXL1/FOSL2/CDK4/HMGB2/CXCL6/PRKCE/B2M/CXCL11/IL6/LBP/ZC3H12A/IRAK2/MYD88/ADM/MAOB 98

BP GO:0022407 regulation of cell-cell adhesion 121/2475 493/18870 2.56047064983818e-12 6.68394164418627e-10 4.85493359372063e-10 CAV1/CD276/ASCL2/WNT4/IL7R/MMRN1/MDK/EFNB1/PYCARD/IL18/PLA2G2A/CXCL13/LEF1/NFKBIZ/SPI1/IL1A/MIR221/LOXL3/ITGB2/LCK/LRRC32/IL1RN/ANXA1/ACTL6B/TESPA1/C1QTNF1/HLA-DMA/CD70/TFRC/PAWR/HLA-DQA2/FOXJ1/HLA-DQA1/CD74/SOCS1/HAVCR2/MAP3K8/CD44/TWSG1/LGALS3/RHOH/RUNX1/HLA-DPA1/CCL2/BMP2/HLA-DOA/PLA2G5/JAG1/KLRC4-KLRK1/CD274/ITGA4/GLI3/GPNMB/HLA-DRB1/CD3E/JAK3/VEGFA/IDO1/PDCD1LG2/FCGR2B/CCL5/PDPN/IL2RG/IGF2/VSIG4/HAS2/SASH3/DUSP10/LYN/EMILIN2/F11R/FSTL3/BTN2A2/RUNX3/SPN/CEBPB/HLA-DPB1/LILRB2/HLA-DRA/HLA-DQB1/EMILIN1/CD300A/TNR/LAPTM5/IGFBP2/LILRB1/ZP3/SDC4/LGALS1/CASP3/HLA-DOB/HLA-A/FGL2/HLA-DRB5/EFNB2/ADAM19/ALOX5/HLA-DQB2/HLA-DMB/PTPRC/DPP4/WNT10B/B2M/IL6/CHST2/IL2RA/PLAUR/ZC3H12A/PIK3R6/FADD/SWAP70/ANK3/TNFRSF14/IRF1/HFE/IL4I1/FERMT3/NODAL/GCNT1/FUT9/FXYD5 121

BP GO:0050867 positive regulation of cell activation 103/2475 396/18870 2.7761119843817e-12 6.94490681426155e-10 5.04448769793569e-10 CAV1/KLRC2/CD276/IL7R/MDK/EFNB1/PYCARD/IL18/LEF1/NFKBIZ/SPI1/IL1A/ITGB2/TNFRSF4/LCK/HAMP/ANXA1/ACTL6B/TESPA1/THBS1/TREM2/CD40/HLA-DMA/CD70/TFRC/CHRNB2/VAV3/HLA-DQA2/VAMP8/HLA-DQA1/CTSC/CD74/SOCS1/STXBP1/HAVCR2/FLNA/MAP3K8/RHOH/RUNX1/HLA-DPA1/LILRA5/CCL2/CLEC7A/HLA-DOA/KLRC4-KLRK1/CD274/GLI3/TAFA3/ACTA2/TOX/HLA-DRB1/CD3E/MMP14/F2RL1/PDCD1LG2/CCL5/IL2RG/IGF2/SASH3/BST1/DUSP10/LYN/RPS6KA1/BTN2A2/RUNX3/LRRK2/SPN/HLA-DPB1/LILRB2/HLA-DRA/HLA-DQB1/NECTIN2/TYROBP/IGFBP2/LILRB1/ZP3/LILRA2/LGALS1/HLA-DOB/HLA-A/HLA-DRB5/EFNB2/CD2/HLA-DQB2/PLEK/HLA-DMB/PTPRC/DPP4/WNT10B/B2M/IL6/LBP/PLA2G4A/FCGR3A/IL2RA/PIK3R6/FADD/TNFRSF14/MYD88/IL4I1/CLCF1/GPR183/HLA-F 103

BP GO:0002504 antigen processing and presentation of peptide or polysaccharide antigen via MHC class II 23/2475 37/18870 4.44750942943737e-12 1.06811386457368e-09 7.75832908048801e-10 PYCARD/THBS1/TREM2/HLA-DMA/HLA-DQA2/HLA-DQA1/CD74/HLA-DPA1/HLA-DOA/HLA-DRB1/FCGR2B/CTSL/HLA-DPB1/HLA-DRA/HLA-DQB1/HLA-DOB/HLA-DRB5/FCER1G/UNC93B1/HLA-DQB2/HLA-DMB/B2M/CTSS 23

BP GO:0032963 collagen metabolic process 42/2475 106/18870 7.56034011616217e-12 1.74585700220914e-09 1.26811696766234e-09 WNT4/CTSK/ITGA2/MMP12/SERPINH1/MMP13/MMP2/TRAM2/MMP1/RUNX1/MMP9/MRC2/MFAP4/CIITA/ENG/MMP14/SMPD3/MMP25/MMP19/RCN3/CTSB/COL1A1/CTSL/ADAMTS14/CST3/COL1A2/MMP7/EMILIN1/ADAMTS2/P3H2/ADAMTS3/VIM/P3H1/FAP/FOSL2/ITGB1/COL5A1/IL6/MMP11/PLOD3/CTSS/PCOLCE 42

BP GO:1903037 regulation of leukocyte cell-cell adhesion 99/2475 382/18870 9.26785954413237e-12 2.02919549624055e-09 1.47392211173684e-09 CAV1/CD276/ASCL2/IL7R/MDK/EFNB1/PYCARD/IL18/PLA2G2A/LEF1/NFKBIZ/IL1A/MIR221/LOXL3/ITGB2/LCK/LRRC32/ANXA1/ACTL6B/TESPA1/HLA-DMA/CD70/TFRC/PAWR/HLA-DQA2/FOXJ1/HLA-DQA1/CD74/SOCS1/HAVCR2/MAP3K8/CD44/TWSG1/LGALS3/RHOH/RUNX1/HLA-DPA1/CCL2/HLA-DOA/PLA2G5/KLRC4-KLRK1/CD274/ITGA4/GLI3/GPNMB/HLA-DRB1/CD3E/JAK3/IDO1/PDCD1LG2/FCGR2B/CCL5/IL2RG/IGF2/VSIG4/HAS2/SASH3/DUSP10/LYN/BTN2A2/RUNX3/SPN/CEBPB/HLA-DPB1/LILRB2/HLA-DRA/HLA-DQB1/CD300A/LAPTM5/IGFBP2/LILRB1/ZP3/SDC4/LGALS1/CASP3/HLA-DOB/HLA-A/FGL2/HLA-DRB5/EFNB2/ALOX5/HLA-DQB2/HLA-DMB/PTPRC/DPP4/WNT10B/B2M/IL6/CHST2/IL2RA/ZC3H12A/PIK3R6/FADD/TNFRSF14/IRF1/HFE/IL4I1/GCNT1/FUT9 99

BP GO:0002495 antigen processing and presentation of peptide antigen via MHC class II 22/2475 35/18870 9.46327013569876e-12 2.02919549624055e-09 1.47392211173684e-09 PYCARD/TREM2/HLA-DMA/HLA-DQA2/HLA-DQA1/CD74/HLA-DPA1/HLA-DOA/HLA-DRB1/FCGR2B/CTSL/HLA-DPB1/HLA-DRA/HLA-DQB1/HLA-DOB/HLA-DRB5/FCER1G/UNC93B1/HLA-DQB2/HLA-DMB/B2M/CTSS 22

BP GO:0033627 cell adhesion mediated by integrin 37/2475 88/18870 1.64579101885916e-11 3.31789199350794e-09 2.40997695029689e-09 ITGA5/FERMT1/MMRN1/ITGB3/CXCL13/ITGB2/NPNT/ITGA2/ITGA3/ICAM1/RET/ITGA11/CYP1B1/ITGB4/ITGA4/CD3E/CCL5/SFRP2/MUC1/LYN/EMILIN2/SERPINE1/LIF/PLAU/EMILIN1/ADAM9/EPHA2/SNAI2/ITGA7/ITGB1/DPP4/PIK3CG/ITGA1/TGFB2/ITGAL/SWAP70/FERMT3 37

BP GO:0050890 cognition 86/2475 317/18870 1.65784076957425e-11 3.31789199350794e-09 2.40997695029689e-09 NPTX2/ITGA5/TMOD2/MDK/FOSL1/JPH4/AFF2/CPEB3/ATP8A1/PRRT1/PRKCG/FGF13/ABCC8/NTAN1/ADGRB3/ITGA3/TREM2/PAK5/CHRNB2/GABRA5/CRH/EGFR/CSMD1/NEUROD2/ASIC1/SHANK2/CUX2/CNR1/SHANK1/SHISA7/OXTR/CHRM1/CLSTN2/SCN2A/NRXN1/C5AR1/TLR2/SLC8A3/BRINP1/GRIN2A/HTR2A/DKK1/HRH1/TAFA2/VIP/GRM5/S100B/SLC8A2/MAPT/SLC2A4/SLC12A5/RASGRF1/SYT4/GRIN1/CBR3/SNAP25/LILRB2/HLA-DRA/ARC/TNR/LRRC66/HRH3/NRXN3/RCAN1/CASP3/STRA6/CNTNAP2/ELAVL4/TBR1/GLP1R/JPH3/ITGB1/ADORA1/MAPK8IP2/CHRNA4/B2M/NTRK1/TUBA1A/HOXA1/NOG/GRIN2B/GPR158/PTN/PLCB1/EN1/CAMK4 86

BP GO:0002697 regulation of immune effector process 99/2475 389/18870 2.86655402318796e-11 5.42718672046326e-09 3.94207976837885e-09 KLRC2/ASCL2/IL7R/BATF/PYCARD/IL18/MR1/KLRC3/NFKBIZ/SPI1/NCF1/LOXL3/ITGB2/TNFRSF4/LITAF/ANXA1/KLK7/TREM2/CGAS/CD40/FCGR1A/TFRC/ICAM1/HPX/VAMP8/FOXJ1/CD74/KLRC4/STXBP1/HAVCR2/RIPK3/AHR/LGALS3/IL13RA2/HLA-C/IL1R1/MICA/ADGRE2/ARID5A/CLEC7A/PLA2G5/SPON2/KLRC4-KLRK1/SLAMF8/HLA-DRB1/MICB/ANGPT1/JAK3/PRAM1/F2RL1/FCGR2B/SCIMP/VSIG4/SASH3/FZD5/DUSP10/ACP5/FES/LYN/SPN/SERPING1/HLA-DRA/NOS2/CD36/CD300A/NECTIN2/LAPTM5/TYROBP/TWIST1/HLA-B/LILRB1/ZP3/RAC2/TRIM6/HLA-A/RSAD2/FGL2/ULBP2/ULBP3/C3/HLA-DMB/PTPRC/COLEC11/CXCL6/B2M/IL6/LBP/TGFB2/ZC3H12A/PIK3R6/FADD/TNFRSF14/CFH/HFE/MYD88/IL4I1/CLCF1/HLA-F/BST2 99

BP GO:0001819 positive regulation of cytokine production 119/2475 499/18870 2.89257120344477e-11 5.42718672046326e-09 3.94207976837885e-09 CD276/LY96/GBP5/FERMT1/MDK/BATF/DDIT3/IRF7/PYCARD/TLR1/IL18/SLC11A1/H19/IL1A/GSDMD/RBM47/CYBA/OAS2/PYDC1/ANXA1/HMOX1/ABCC8/MMP12/THBS1/TREM2/CGAS/CD40/BCL3/F3/CD74/HAVCR2/HSPB1/RUNX1/IL1R1/S100A13/HLA-DPA1/CYP1B1/LILRA5/ARID5A/CLEC7A/STAT1/IL17RC/OAS1/ALOX15B/NFAM1/SPON2/KLRC4-KLRK1/CD274/RAB7B/PTGER4/CASP1/CD3E/CASP8/BRCA1/C5AR1/TLR2/IDO1/IFI16/F2RL1/CD58/SCIMP/CLEC5A/SPHK1/CHI3L1/HILPDA/SASH3/IFIH1/TLR8/HGF/FZD5/SCAMP5/OAS3/MIR27B/LRRK2/SERPINE1/SPN/CEBPB/CD14/HLA-DPB1/LILRB2/NOS2/CD36/ISG15/LAPTM5/TYROBP/TWIST1/LILRB1/ZP3/LILRA2/IL1RAP/TRIM6/HLA-A/RSAD2/LUM/OSM/FCER1G/CD2/IL9/POSTN/UNC93B1/MCOLN2/RNF135/GATA4/C3/PTPRC/HMGB2/B2M/IL6/PIK3CG/LBP/FCGR3A/FADD/TNFRSF14/IRF1/SAA1/MYD88/PLCB1/NODAL/HLA-F 119

BP GO:0007611 learning or memory 77/2475 274/18870 3.00694308278474e-11 5.47081402092109e-09 3.97376880126864e-09 NPTX2/ITGA5/TMOD2/MDK/FOSL1/JPH4/AFF2/CPEB3/ATP8A1/PRRT1/PRKCG/FGF13/ABCC8/NTAN1/ADGRB3/ITGA3/TREM2/PAK5/CHRNB2/GABRA5/CRH/EGFR/CSMD1/NEUROD2/ASIC1/SHANK2/CUX2/CNR1/SHANK1/SHISA7/OXTR/CLSTN2/SCN2A/NRXN1/TLR2/SLC8A3/BRINP1/GRIN2A/HTR2A/DKK1/HRH1/TAFA2/VIP/GRM5/S100B/SLC8A2/MAPT/SLC2A4/SLC12A5/RASGRF1/SYT4/GRIN1/SNAP25/LILRB2/ARC/TNR/LRRC66/NRXN3/RCAN1/CASP3/STRA6/CNTNAP2/ELAVL4/TBR1/GLP1R/JPH3/ITGB1/MAPK8IP2/B2M/NTRK1/TUBA1A/NOG/GRIN2B/PTN/PLCB1/EN1/CAMK4 77

BP GO:0002696 positive regulation of leukocyte activation 97/2475 380/18870 3.79200849870177e-11 6.4859129730851e-09 4.71109391063704e-09 CAV1/KLRC2/CD276/IL7R/MDK/EFNB1/PYCARD/IL18/LEF1/NFKBIZ/SPI1/IL1A/ITGB2/TNFRSF4/LCK/HAMP/ANXA1/ACTL6B/TESPA1/THBS1/TREM2/CD40/HLA-DMA/CD70/TFRC/CHRNB2/VAV3/HLA-DQA2/VAMP8/HLA-DQA1/CTSC/CD74/SOCS1/STXBP1/HAVCR2/MAP3K8/RHOH/RUNX1/HLA-DPA1/CCL2/CLEC7A/HLA-DOA/KLRC4-KLRK1/CD274/GLI3/TAFA3/TOX/HLA-DRB1/CD3E/MMP14/F2RL1/PDCD1LG2/CCL5/IL2RG/IGF2/SASH3/BST1/DUSP10/LYN/BTN2A2/RUNX3/LRRK2/SPN/HLA-DPB1/LILRB2/HLA-DRA/HLA-DQB1/NECTIN2/TYROBP/IGFBP2/LILRB1/ZP3/LGALS1/HLA-DOB/HLA-A/HLA-DRB5/EFNB2/CD2/HLA-DQB2/HLA-DMB/PTPRC/DPP4/WNT10B/B2M/IL6/LBP/PLA2G4A/FCGR3A/IL2RA/PIK3R6/FADD/TNFRSF14/MYD88/IL4I1/CLCF1/GPR183/HLA-F 97

BP GO:0032496 response to lipopolysaccharide 91/2475 348/18870 3.81166964552343e-11 6.4859129730851e-09 4.71109391063704e-09 THBD/MAP2K3/LY96/GBP5/PYCARD/SLC11A1/CXCL13/NFKBIZ/SPI1/IL1A/TSPO/CXCL8/GBP2/ACE/SOD2/LITAF/ABCC8/TREM2/CD40/CLDN1/GCH1/HAVCR2/CITED1/PPBP/SBNO2/ARID5A/CNR1/CCL2/CMPK2/MMP9/PALM3/CD180/CASP7/S100A8/SPON2/KLRC4-KLRK1/CD274/S100A9/PTGER4/CASP1/TNFAIP3/CASP8/CXCL10/TLR2/ZFP36/CARD16/IDO1/PDCD1LG2/SCIMP/DUSP10/ACP5/LYN/GJB2/IL10RA/CYP27B1/GBP3/CSF3/SERPINE1/SLPI/CEBPB/CD14/LTF/MGST2/LILRB2/CXCL9/NOS2/CD36/C2/CCR5/LILRB1/LILRA2/CASP3/TRIM6/TRIM5/ADAM9/VIM/CSF2RB/LOXL1/FOSL2/CDK4/HMGB2/CXCL6/PRKCE/CXCL11/IL6/LBP/ZC3H12A/IRAK2/MYD88/ADM/MAOB 91

BP GO:0002399 MHC class II protein complex assembly 14/2475 16/18870 3.99698167894984e-11 6.4859129730851e-09 4.71109391063704e-09 HLA-DMA/HLA-DQA2/HLA-DQA1/HLA-DPA1/HLA-DOA/HLA-DRB1/HLA-DPB1/HLA-DRA/HLA-DQB1/HLA-DOB/HLA-DRB5/HLA-DQB2/HLA-DMB/B2M 14

BP GO:0002503 peptide antigen assembly with MHC class II protein complex 14/2475 16/18870 3.99698167894984e-11 6.4859129730851e-09 4.71109391063704e-09 HLA-DMA/HLA-DQA2/HLA-DQA1/HLA-DPA1/HLA-DOA/HLA-DRB1/HLA-DPB1/HLA-DRA/HLA-DQB1/HLA-DOB/HLA-DRB5/HLA-DQB2/HLA-DMB/B2M 14

BP GO:0019886 antigen processing and presentation of exogenous peptide antigen via MHC class II 20/2475 31/18870 4.19813722450713e-11 6.63305681472127e-09 4.81797299754378e-09 HLA-DMA/HLA-DQA2/HLA-DQA1/CD74/HLA-DPA1/HLA-DOA/HLA-DRB1/FCGR2B/CTSL/HLA-DPB1/HLA-DRA/HLA-DQB1/HLA-DOB/HLA-DRB5/FCER1G/UNC93B1/HLA-DQB2/HLA-DMB/B2M/CTSS 20

BP GO:0002478 antigen processing and presentation of exogenous peptide antigen 23/2475 40/18870 4.33980397962243e-11 6.68107258811617e-09 4.85284963227415e-09 FCGR1A/HLA-DMA/HLA-DQA2/HLA-DQA1/CD74/HLA-DPA1/HLA-DOA/HLA-DRB1/FCGR2B/CTSL/HLA-DPB1/HLA-DRA/HLA-DQB1/HLA-DOB/HLA-A/HLA-DRB5/FCER1G/UNC93B1/HLA-DQB2/HLA-DMB/B2M/HLA-F/CTSS 23

BP GO:0031349 positive regulation of defense response 115/2475 480/18870 4.59725562421809e-11 6.90048069195136e-09 5.01221843450936e-09 CAV1/KLRC2/PDE2A/OASL/LY96/TRADD/GBP5/MDK/IRF7/PYCARD/TLR1/IL18/PLA2G2A/KLRC3/NFKBIZ/SPI1/FOSL1/NKG7/GSDMD/PARP9/RBM47/NEAT1/CYBA/GBP2/MMP12/KLK7/TREM2/CGAS/CD40/FCGR1A/HPX/S100A12/VAMP8/CTSC/LILRA4/KLRC4/HAVCR2/LILRA5/CNR1/CLEC7A/CCN4/TIFA/OAS1/BIRC3/PLA2G5/S100A8/GPR4/KLRC4-KLRK1/S100A9/RAB7B/TNFRSF1A/TAFA3/PTGER4/CASP1/TNFAIP3/CD300LF/TLR2/PLSCR1/IDO1/IFI16/F2RL1/NMI/CCL5/SCIMP/IFIH1/TLR8/CASP6/LYN/EMILIN2/FPR2/OAS3/ALOX5AP/LRRK2/SERPINE1/NLRC5/CEBPB/CD14/LTF/ZDHHC12/MGST2/SLC15A3/PAK3/CD36/EMILIN1/CD300A/NECTIN2/TYROBP/ZP3/LILRA2/LGALS1/IFI35/TRIM6/TRIM5/RSAD2/OSM/OSMR/ALPK1/UNC93B1/RNF135/C3/HMGB2/COLEC11/PRKCE/IL6/PIK3CG/LBP/FADD/IRAK2/CASP4/IRF1/MYD88/GRP/HLA-F/CTSS/COLEC12 115

BP GO:0001906 cell killing 66/2475 223/18870 6.77380481172e-11 9.91949368038216e-09 7.20510226828137e-09 KLRC2/GBP5/IL7R/IL18/EMP2/MR1/PLA2G2A/KLRC3/SPI1/NKG7/HAMP/GBP2/CHGA/FCGR1A/ICAM1/S100A12/CTSC/KLRC4/HAVCR2/RIPK3/GZMB/HLA-C/MICA/GBP1/CLEC7A/RAB27A/KLRC4-KLRK1/C7/HLA-DRB1/MICB/APOL1/GZMA/F2RL1/FCGR2B/SCNN1B/H2BC11/LYZ/H2BC12/GBP3/LTF/HLA-DRA/NOS2/NECTIN2/IGHG1/TYROBP/HLA-B/LILRB1/HLA-A/PRF1/ULBP2/CD2/ULBP3/C3/PTPRC/CXCL6/B2M/FCGR3A/GNLY/PIK3R6/FADD/CFH/HFE/MYD88/TREM1/GZMH/HLA-F 66

BP GO:0042391 regulation of membrane potential 107/2475 440/18870 8.6152322963079e-11 1.23156796921506e-08 8.94559082797083e-09 CAV1/KCNA1/AKAP6/GLRA3/KCNC1/CHRNA9/HCN1/STX1A/BVES/GRIA4/IFI6/TSPO/CACNG2/STX1B/RNF122/SOD2/FGF13/TREM2/GABBR1/CHRNB2/FGF12/RIMS1/KCNB1/CHRNA1/SLC39A8/GABRA3/GABRA5/GJD2/CTNNA3/FLNA/GABRD/RGS7BP/ASIC1/GABRA4/CUX2/CNR1/GABRB3/GRIA2/KCNH3/SHANK1/DSG2/CHRM1/GRIN3A/KCNK7/NRXN1/KCNQ5/SLC8A3/GLRX/GRIN2A/SCN2B/NALCN/KCNAB2/RYR2/TBX5/KCNH1/KCNA4/GRM5/DCN/ASIC2/KCNK3/KCNN4/KCNK12/PID1/SLC8A2/MAPT/KCNE5/SCN8A/GRIN1/LRRK2/SLC1A6/KCNJ11/KCNE3/KCNJ3/GJC1/CD36/DSC2/GABRA1/CBLN1/KCNH7/GABRB2/CELF4/INSYN2B/GABRG2/KCNC2/SCN3B/GRIK1/P2RX6/GRM1/GABRG1/ADORA1/SLC25A27/MAPK8IP2/CHRNA4/STOX1/KCNJ9/RGS4/RIMS3/ANK3/CLIC1/KCNE4/CACNA1I/SLC29A1/GRIN2B/UCP2/GABRE/RIMS2/KCNIP2 107

BP GO:0006935 chemotaxis 112/2475 468/18870 8.87011681712201e-11 1.23851584581397e-08 8.99605727621456e-09 CXCL14/RARRES2/MDK/ITGB3/LGR6/SEMA3E/CXCL13/TMSB4X/LEF1/SPI1/FOSL1/CCL26/ITGB2/CXCL8/ITGA2/CCL20/CHGA/TYMP/ANXA1/THBS1/TREM2/ECSCR/NRP1/MMP2/VAV3/S100A12/SEMA3A/F3/FPR3/NRG3/CD74/SEMA3D/PPBP/SLIT1/LGALS3/HSPB1/DSCAM/CCN1/ADGRE2/CCL2/IL17RC/LOX/RNASE2/S100A8/KLRC4-KLRK1/ANGPT2/MET/S100A9/GPNMB/SLAMF8/FEZF2/HSD3B7/ANGPT1/VEGFA/CXCL10/C5AR1/F2RL1/CMTM3/CCL5/L1CAM/CXCR4/SEMA6B/DPEP1/BST1/HGF/FES/LYN/FPR2/CMTM7/SERPINE1/PDGFA/CCL18/SPN/PLAU/PPIA/CXCL9/CCR5/THBS4/TNFAIP6/RAC2/PDGFD/SEMA3F/RNASE3/LSP1/S100A4/CXCR2/EPHA2/EFNB2/FCER1G/CCRL2/TBR1/ALOX5/HMGB2/DPP4/LRTM2/CXCL6/GDNF/CXCL11/IL6/PIK3CG/LBP/ITGA1/TGFB2/PLAUR/SWAP70/SAA1/TREM1/FPR1/PTN/CCR1/GPR183/PLP2 112

BP GO:0022409 positive regulation of cell-cell adhesion 85/2475 322/18870 1.00062322058078e-10 1.36539586735614e-08 9.91766029393824e-09 CAV1/CD276/IL7R/MMRN1/MDK/EFNB1/PYCARD/IL18/CXCL13/LEF1/NFKBIZ/IL1A/ITGB2/LCK/ANXA1/ACTL6B/TESPA1/HLA-DMA/CD70/TFRC/HLA-DQA2/HLA-DQA1/CD74/SOCS1/HAVCR2/MAP3K8/CD44/RHOH/RUNX1/HLA-DPA1/CCL2/HLA-DOA/KLRC4-KLRK1/CD274/ITGA4/GLI3/HLA-DRB1/CD3E/PDCD1LG2/CCL5/PDPN/IL2RG/IGF2/HAS2/SASH3/DUSP10/LYN/EMILIN2/F11R/FSTL3/BTN2A2/RUNX3/SPN/HLA-DPB1/LILRB2/HLA-DRA/HLA-DQB1/EMILIN1/IGFBP2/LILRB1/ZP3/LGALS1/HLA-DOB/HLA-A/HLA-DRB5/EFNB2/ADAM19/ALOX5/HLA-DQB2/HLA-DMB/PTPRC/DPP4/WNT10B/B2M/IL6/CHST2/IL2RA/PLAUR/PIK3R6/FADD/ANK3/TNFRSF14/IL4I1/NODAL/GCNT1 85

BP GO:0046651 lymphocyte proliferation 82/2475 307/18870 1.1392423689598e-10 1.52000248516326e-08 1.10406576247964e-08 CD276/WNT4/IL7R/EFNB1/PYCARD/IL18/EMP2/SLC11A1/PLA2G2A/LEF1/NFKBIZ/IL1A/MSN/TNFRSF4/LRRC32/ANXA1/CD40/CD70/TFRC/PAWR/ELF4/CHRNB2/VAV3/FOXJ1/CD74/HAVCR2/TWSG1/RIPK3/AHR/LGALS3/TNFSF8/HLA-DPA1/CD79A/CD180/CD151/PLA2G5/GAPT/CD274/GPNMB/HLA-DRB1/CD3E/IDO1/PDCD1LG2/FCGR2B/CCL5/IGF2/VSIG4/SASH3/BST1/LYN/BTN2A2/SH2D2A/SPN/CEBPB/HLA-DPB1/LILRB2/CD300A/TYROBP/IGFBP2/LILRB1/ZP3/RAC2/SDC4/CASP3/HLA-A/IL9/FOSL2/HLA-DMB/PTPRC/IL6/PIK3CG/LMO1/FCGR3A/IL2RA/FADD/TNFRSF14/IRF1/GAL/MYD88/IL4I1/CLCF1/GPR183 82

BP GO:0042330 taxis 112/2475 470/18870 1.16778451052288e-10 1.52421265243029e-08 1.10712385059411e-08 CXCL14/RARRES2/MDK/ITGB3/LGR6/SEMA3E/CXCL13/TMSB4X/LEF1/SPI1/FOSL1/CCL26/ITGB2/CXCL8/ITGA2/CCL20/CHGA/TYMP/ANXA1/THBS1/TREM2/ECSCR/NRP1/MMP2/VAV3/S100A12/SEMA3A/F3/FPR3/NRG3/CD74/SEMA3D/PPBP/SLIT1/LGALS3/HSPB1/DSCAM/CCN1/ADGRE2/CCL2/IL17RC/LOX/RNASE2/S100A8/KLRC4-KLRK1/ANGPT2/MET/S100A9/GPNMB/SLAMF8/FEZF2/HSD3B7/ANGPT1/VEGFA/CXCL10/C5AR1/F2RL1/CMTM3/CCL5/L1CAM/CXCR4/SEMA6B/DPEP1/BST1/HGF/FES/LYN/FPR2/CMTM7/SERPINE1/PDGFA/CCL18/SPN/PLAU/PPIA/CXCL9/CCR5/THBS4/TNFAIP6/RAC2/PDGFD/SEMA3F/RNASE3/LSP1/S100A4/CXCR2/EPHA2/EFNB2/FCER1G/CCRL2/TBR1/ALOX5/HMGB2/DPP4/LRTM2/CXCL6/GDNF/CXCL11/IL6/PIK3CG/LBP/ITGA1/TGFB2/PLAUR/SWAP70/SAA1/TREM1/FPR1/PTN/CCR1/GPR183/PLP2 112

BP GO:0002455 humoral immune response mediated by circulating immunoglobulin 27/2475 55/18870 1.21728537475614e-10 1.55501731702891e-08 1.12949906105592e-08 C1RL/CFI/IGHG4/C1R/TREM2/HPX/BCL3/FOXJ1/IGHA1/IGHG2/C7/IGHG3/FCGR2B/IGHA2/EXO1/SERPING1/C2/IGHG1/C1S/ZP3/IGHM/C3/C1QB/PTPRC/C1QC/C1QA/NCR3LG1 27

BP GO:0032943 mononuclear cell proliferation 83/2475 314/18870 1.54317778640649e-10 1.93025821449678e-08 1.40205823883379e-08 CD276/WNT4/IL7R/EFNB1/PYCARD/IL18/EMP2/SLC11A1/PLA2G2A/LEF1/NFKBIZ/IL1A/MSN/TNFRSF4/LRRC32/ACE/ANXA1/CD40/CD70/TFRC/PAWR/ELF4/CHRNB2/VAV3/FOXJ1/CD74/HAVCR2/TWSG1/RIPK3/AHR/LGALS3/TNFSF8/HLA-DPA1/CD79A/CD180/CD151/PLA2G5/GAPT/CD274/GPNMB/HLA-DRB1/CD3E/IDO1/PDCD1LG2/FCGR2B/CCL5/IGF2/VSIG4/SASH3/BST1/LYN/BTN2A2/SH2D2A/SPN/CEBPB/HLA-DPB1/LILRB2/CD300A/TYROBP/IGFBP2/LILRB1/ZP3/RAC2/SDC4/CASP3/HLA-A/IL9/FOSL2/HLA-DMB/PTPRC/IL6/PIK3CG/LMO1/FCGR3A/IL2RA/FADD/TNFRSF14/IRF1/GAL/MYD88/IL4I1/CLCF1/GPR183 83

BP GO:0002768 immune response-regulating cell surface receptor signaling pathway 89/2475 346/18870 1.58338663020194e-10 1.9401333321903e-08 1.40923110825492e-08 TRAC/KLRC2/CD276/LY96/TLR1/TRDC/KLRC3/NFKBIZ/BTN2A3P/LCK/CYBA/IGHG4/TESPA1/CD247/TREM2/CD40/FCGR1A/PAWR/CD3D/VAV3/FPR3/LILRA4/KLRC4/IGHA1/LGALS3/LCP2/GBP1/CLEC7A/CD79A/OAS1/CD8A/PRKCB/NFAM1/KLRC4-KLRK1/TRBC1/RAB7B/IGHG2/HLA-DRB1/TNFAIP3/CD3E/MICB/PRAM1/C5AR1/TLR2/PLSCR1/F2RL1/NMI/CMTM3/IGHG3/FCGR2B/SCIMP/IGKC/IGHA2/LYN/FPR2/KCNN4/BTN2A2/CD14/LTF/HLA-DPB1/LILRB2/TRBC2/PAK3/HLA-DQB1/CD300A/NECTIN2/LAPTM5/IGHG1/TYROBP/LILRB1/LILRA2/IFI35/HLA-A/LAT2/THEMIS2/IGHM/FCER1G/FOSL2/PTPRC/COLEC11/PRKCE/LBP/FCGR3A/ZC3H12A/IRAK2/MYO1G/MYD88/FPR1/IGLC3 89

BP GO:0048704 embryonic skeletal system morphogenesis 37/2475 94/18870 1.65087184460958e-10 1.98236691100718e-08 1.43990780046684e-08 HOXD3/SHOX2/HOXB2/HOXA5/HOXB3/HOXA3/HOXC11/HOXA11/HOXA2/HOXD9/HOXB4/DLX2/HOXD4/DSCAML1/GLI3/HOXA9/MMP14/HOXC9/HOXC4/GSC/RUNX2/OSR1/IRX5/HOXD10/TBX15/TWIST1/HOXA7/HOXB8/TBX1/RDH10/OSR2/EYA1/HOXA1/NOG/COL2A1/NODAL/HOXB7 37

BP GO:0070661 leukocyte proliferation 89/2475 350/18870 3.00036133817366e-10 3.50380652624189e-08 2.54501743367933e-08 CD276/WNT4/IL7R/EFNB1/PYCARD/IL18/EMP2/SLC11A1/PLA2G2A/LEF1/NFKBIZ/IL1A/MSN/TNFRSF4/LRRC32/ACE/ANXA1/TREM2/CD40/CD70/TFRC/PAWR/ELF4/CHRNB2/VAV3/FOXJ1/CD74/HAVCR2/TWSG1/RIPK3/AHR/LGALS3/TNFSF8/HLA-DPA1/CD79A/CD180/CD151/PLA2G5/GAPT/CD274/GPNMB/HLA-DRB1/TNFAIP3/CD3E/IDO1/F2RL1/PDCD1LG2/FCGR2B/CCL5/IGF2/VSIG4/SASH3/BST1/LYN/BTN2A2/SH2D2A/SPN/CEBPB/HLA-DPB1/LILRB2/CD300A/TYROBP/IGFBP2/LILRB1/ZP3/RAC2/SDC4/CASP3/HLA-A/CSF2RB/IL9/FOSL2/HLA-DMB/PTPRC/IL6/PIK3CG/LMO1/FCGR3A/IL2RA/FADD/TNFRSF14/IRF1/GAL/TCIRG1/MYD88/IL4I1/CLCF1/GPR183/BST2 89

BP GO:0002429 immune response-activating cell surface receptor signaling pathway 83/2475 318/18870 3.0346092499097e-10 3.50380652624189e-08 2.54501743367933e-08 TRAC/KLRC2/CD276/LY96/TLR1/TRDC/KLRC3/NFKBIZ/BTN2A3P/LCK/CYBA/IGHG4/TESPA1/CD247/TREM2/PAWR/CD3D/VAV3/FPR3/KLRC4/IGHA1/LGALS3/LCP2/GBP1/CLEC7A/CD79A/OAS1/CD8A/PRKCB/NFAM1/KLRC4-KLRK1/TRBC1/RAB7B/IGHG2/HLA-DRB1/TNFAIP3/CD3E/MICB/PRAM1/C5AR1/TLR2/PLSCR1/F2RL1/NMI/CMTM3/IGHG3/FCGR2B/SCIMP/IGKC/IGHA2/LYN/FPR2/KCNN4/BTN2A2/CD14/LTF/HLA-DPB1/TRBC2/PAK3/HLA-DQB1/CD300A/NECTIN2/LAPTM5/IGHG1/TYROBP/LILRA2/IFI35/HLA-A/LAT2/THEMIS2/IGHM/FCER1G/FOSL2/PTPRC/COLEC11/PRKCE/LBP/ZC3H12A/IRAK2/MYO1G/MYD88/FPR1/IGLC3 83

BP GO:0002764 immune response-regulating signaling pathway 116/2475 500/18870 3.13032304194952e-10 3.54612444223866e-08 2.57575538486531e-08 CAV1/TRAC/KLRC2/CD276/OASL/LY96/GBP5/IRF7/PYCARD/TLR1/TRDC/KLRC3/NFKBIZ/FOSL1/BTN2A3P/LCK/CYBA/GBP2/IGHG4/TESPA1/CD247/TREM2/CGAS/CD40/FCGR1A/PAWR/CD3D/VAV3/FPR3/LILRA4/KLRC4/HAVCR2/CD33/IGHA1/LGALS3/LCP2/GBP1/CLEC7A/CD79A/TIFA/OAS1/BIRC3/CD8A/PRKCB/NFAM1/KLRC4-KLRK1/TRBC1/RAB7B/CASP1/IGHG2/HLA-DRB1/TNFAIP3/CD3E/MICB/CD300LF/PRAM1/C5AR1/TLR2/PLSCR1/F2RL1/NMI/CMTM3/IGHG3/FCGR2B/SCIMP/IGKC/IFIH1/TLR8/IGHA2/LYN/FPR2/KCNN4/OAS3/BTN2A2/CD14/LTF/ZDHHC12/HLA-DPB1/LILRB2/SLC15A3/TRBC2/PAK3/CD36/HLA-DQB1/CD300A/NECTIN2/LAPTM5/IGHG1/TYROBP/LILRB1/LILRA2/IFI35/HLA-A/RSAD2/LAT2/THEMIS2/IGHM/FCER1G/ALPK1/FOSL2/UNC93B1/RNF135/PTPRC/COLEC11/PRKCE/LBP/FCGR3A/ZC3H12A/IRAK2/IRF1/MYO1G/MYD88/FPR1/IGLC3/CTSS/COLEC12 116

BP GO:0001503 ossification 106/2475 444/18870 3.29391820386194e-10 3.66234905481242e-08 2.66017604651072e-08 SHOX2/WNT4/MDK/HAND2/SNAI1/LEF1/WWTR1/STC1/NPNT/CTSK/ROR2/RIPPLY2/SPP1/MGP/GDPD2/HOXA2/DNAI3/GABBR1/MMP13/ITGA11/MMP2/SMOC1/MIR210/EGFR/CITED1/TWSG1/IGFBP3/IGFBP5/SBNO2/RUNX1/CCN1/BMP2/SOX8/AREG/CCN4/MRC2/LOX/JAG1/ECM1/DLX5/GLI3/PHEX/PTGER4/MMP14/SNX10/SMPD3/NELL1/DKK1/PDLIM7/SFRP2/ENPP1/CLEC5A/IGF2/HGF/COL1A1/FAM20C/RUNX2/SRGN/FSTL3/OSR1/CYP27B1/CEBPD/RUNX3/COL1A2/TENT5A/CEBPB/CTHRC1/LTF/TNC/ISG15/COL6A1/SLC26A2/CHRDL2/TWIST1/GLI1/H3-3A/ADGRV1/TNFAIP6/SGMS2/TWIST2/TPM4/GDF10/EPHA2/COL5A2/SNAI2/ERFE/ALOX5/ASPN/MN1/FOSL2/PTHLH/FZD1/WNT10B/IL6/PPARG/ADAMTS7/CLIC1/OSR2/TCIRG1/NOG/COL2A1/IBSP/PTN/CCR1/WNT7B/CDK6 106

BP GO:0002757 immune response-activating signaling pathway 111/2475 473/18870 3.72216541433619e-10 4.063251117759e-08 2.95137441370236e-08 CAV1/TRAC/KLRC2/CD276/OASL/LY96/GBP5/IRF7/PYCARD/TLR1/TRDC/KLRC3/NFKBIZ/FOSL1/BTN2A3P/LCK/CYBA/GBP2/IGHG4/TESPA1/CD247/TREM2/CGAS/CD40/PAWR/CD3D/VAV3/FPR3/LILRA4/KLRC4/HAVCR2/IGHA1/LGALS3/LCP2/GBP1/CLEC7A/CD79A/TIFA/OAS1/BIRC3/CD8A/PRKCB/NFAM1/KLRC4-KLRK1/TRBC1/RAB7B/CASP1/IGHG2/HLA-DRB1/TNFAIP3/CD3E/MICB/CD300LF/PRAM1/C5AR1/TLR2/PLSCR1/F2RL1/NMI/CMTM3/IGHG3/FCGR2B/SCIMP/IGKC/IFIH1/TLR8/IGHA2/LYN/FPR2/KCNN4/OAS3/BTN2A2/CD14/LTF/ZDHHC12/HLA-DPB1/SLC15A3/TRBC2/PAK3/CD36/HLA-DQB1/CD300A/NECTIN2/LAPTM5/IGHG1/TYROBP/LILRA2/IFI35/HLA-A/RSAD2/LAT2/THEMIS2/IGHM/FCER1G/ALPK1/FOSL2/UNC93B1/RNF135/PTPRC/COLEC11/PRKCE/LBP/ZC3H12A/IRAK2/IRF1/MYO1G/MYD88/FPR1/IGLC3/CTSS/COLEC12 111

BP GO:0043410 positive regulation of MAPK cascade 111/2475 474/18870 4.24632485339424e-10 4.55266686067483e-08 3.30686538864894e-08 MAP2K3/HAND2/ITGB3/PYCARD/PLA2G2A/NECAB2/SPI1/IL1A/NCF1/MIR221/CCL26/NPNT/IQGAP1/CCL20/IGFBP6/SPRY2/C1QTNF1/THBS1/TNFRSF19/TREM2/CD40/ICAM1/RET/NRP1/FZD7/SHC1/S100A12/PDE6G/SEMA3A/DOK6/CD74/HAVCR2/NOX4/EGFR/CD44/WNT16/IGFBP3/LILRA5/P2RY6/CCL2/BMP2/IGFBP4/PLA2G5/DOK2/ACTA2/GPNMB/HLA-DRB1/ANGPT1/VEGFA/C5AR1/F2RL1/HTR2A/FCGR2B/CAV2/CCL5/DKK1/SCIMP/SPHK1/CHI3L1/IGF2/IL11/GRM5/HGF/HTR2C/FPR2/MIR27B/LRRK2/PDGFA/LIF/CCL18/PPIA/CD36/LAPTM5/RAP1B/P2RY1/PDGFD/TRIM5/ADAM9/LTBR/OSM/GRM1/IQGAP3/DOK3/GADD45A/GATA4/TBX1/PTPRC/ADORA1/MAPK8IP2/CAVIN3/PRKCE/NTRK1/IL6/PIK3CG/ITGA1/MARCO/TGFB2/ZC3H12A/PIK3R6/RAMP3/MYD88/EZH2/CCR1/ADRA2B/PLCB1/WNT7B/GPR183/NODAL/GDF15/MT3/TP73 111

BP GO:0050866 negative regulation of cell activation 64/2475 223/18870 5.37248131267067e-10 5.65901364934644e-08 4.11046908188266e-08 THBD/ASCL2/MDK/PLA2G2A/SPI1/LOXL3/LRRC32/ANXA1/C1QTNF1/TREM2/PAWR/FOXJ1/CD74/SOCS1/HAVCR2/CD33/TWSG1/LGALS3/IL13RA2/RUNX1/MICA/CNR1/PLA2G5/CD274/GLI3/TAFA3/GPNMB/HLA-DRB1/TNFAIP3/SAMSN1/JAK3/CD300LF/IDO1/PDCD1LG2/FCGR2B/VSIG4/FN1/LYN/BTN2A2/RUNX3/PDGFA/SPN/CEBPB/LILRB2/EMILIN1/CD300A/LAPTM5/TYROBP/LILRB1/SDC4/CASP3/CST7/FGL2/MILR1/LRFN5/PTPRC/IL2RA/ZC3H12A/TNFRSF14/IRF1/GAL/HFE/IL4I1/HLA-F 64

BP GO:0048706 embryonic skeletal system development 44/2475 128/18870 5.79372049619135e-10 5.99749963088498e-08 4.35633103733589e-08 HOXD3/SHOX2/HAND2/HOXB2/HOXA6/HOXA5/HOXC6/HOXB3/HOXA3/HOXC11/HOXA11/HOXA2/NKX3-2/HOXD9/HOXB4/DLX2/HOXD4/DSCAML1/GLI3/HOXA9/MMP14/HOXC9/SLC2A10/HOXC4/GSC/COL1A1/RUNX2/PCSK5/OSR1/IRX5/HOXD10/TBX15/TWIST1/HOXA7/HOXB8/TBX1/RDH10/OSR2/EYA1/HOXA1/NOG/COL2A1/NODAL/HOXB7 44

BP GO:0034765 regulation of monoatomic ion transmembrane transport 107/2475 454/18870 5.96638965684531e-10 6.07155991520326e-08 4.41012530745943e-08 AKAP5/CAV1/KCNA1/VMP1/AKAP6/KCNC1/RGS7/HCN1/ITGB3/TMSB4X/STAC/SLN/JPH4/KCNIP3/CACNG2/CYBA/HAMP/CACNA1B/FGF13/TREM2/CABP4/FGF12/CLIC3/NIPSNAP2/KCNB1/TMEM37/MIR210/FLNA/KCNMB1/APLNR/TCAF2/P2RY6/KCNV1/CCL2/KCNH3/MMP9/WNK2/SCN2A/KCNAB1/CLIC4/KCNK7/CD63/CXCL10/KCNQ5/KCNS2/GLRX/PIRT/SELENON/GRIN2A/SCN2B/NALCN/KCNAB2/RYR2/KCNH1/KCNA4/GRM5/HECW1/CACNA2D2/LYN/ASIC2/KCNN4/KCNK12/KCNE5/OSR1/SCN8A/GRIN1/CACNA1E/KCNJ11/KCNE3/CXCL9/ARC/KCNJ3/ADCYAP1R1/DPP10/F2RL3/CABP1/KCNH7/KCNJ12/KCNC2/SCN3B/LRRC55/HPCA/JPH3/ITGB1/CACNA2D3/SCN3A/FXYD2/PRKCE/CXCL11/PIK3CG/FKBP1C/KCNJ9/RGS4/ANK3/CLIC1/RAMP3/GAL/TCIRG1/KCNE4/CACNA1I/GRIN2B/GRP/CACNG3/FXYD5/CTSS/KCNJ4/KCNIP2 107

BP GO:1903039 positive regulation of leukocyte cell-cell adhesion 74/2475 275/18870 6.36288500669994e-10 6.36712693003774e-08 4.62481273381717e-08 CAV1/CD276/IL7R/MDK/EFNB1/PYCARD/IL18/LEF1/NFKBIZ/IL1A/ITGB2/LCK/ANXA1/ACTL6B/TESPA1/HLA-DMA/CD70/TFRC/HLA-DQA2/HLA-DQA1/CD74/SOCS1/HAVCR2/MAP3K8/CD44/RHOH/RUNX1/HLA-DPA1/CCL2/HLA-DOA/KLRC4-KLRK1/CD274/ITGA4/GLI3/HLA-DRB1/CD3E/PDCD1LG2/CCL5/IL2RG/IGF2/HAS2/SASH3/DUSP10/LYN/BTN2A2/RUNX3/SPN/HLA-DPB1/LILRB2/HLA-DRA/HLA-DQB1/IGFBP2/LILRB1/ZP3/LGALS1/HLA-DOB/HLA-A/HLA-DRB5/EFNB2/ALOX5/HLA-DQB2/HLA-DMB/PTPRC/DPP4/WNT10B/B2M/IL6/CHST2/IL2RA/PIK3R6/FADD/TNFRSF14/IL4I1/GCNT1 74

BP GO:0045055 regulated exocytosis 64/2475 227/18870 1.19036544712436e-09 1.15324620698098e-07 8.376694546657e-08 KLRC2/CDK5R2/UNC13A/STX1A/SYN1/SYNGR3/SPI1/NKG7/ITGB2/STX1B/NAPB/CHGA/PRKCG/CPLX2/RIMS1/VAMP8/KCNB1/DOC2A/STXBP1/IL13RA2/S100A13/ADGRE2/RAB27A/RAB3A/UNC13C/PRKCB/RAPGEF4/SCIN/ERC2/PRAM1/SYN2/SYT7/F2RL1/FCGR2B/FES/LYN/SCAMP5/SYT1/SYT4/LRRK2/SNAP25/CD300A/RAP1B/PPFIA2/SV2B/P2RY1/ZP3/RAC2/SYP/LAT2/MILR1/SYT5/PCLO/PLEK/PIK3CG/SYNGR1/FCGR3A/SYT2/RIMS3/SYT13/CPLX1/GRP/HLA-F/RIMS2 64

BP GO:0002695 negative regulation of leukocyte activation 59/2475 202/18870 1.20164011404868e-09 1.15324620698098e-07 8.376694546657e-08 ASCL2/MDK/PLA2G2A/SPI1/LOXL3/LRRC32/ANXA1/PAWR/FOXJ1/CD74/SOCS1/HAVCR2/CD33/TWSG1/LGALS3/IL13RA2/RUNX1/MICA/CNR1/PLA2G5/CD274/GLI3/TAFA3/GPNMB/HLA-DRB1/TNFAIP3/SAMSN1/JAK3/CD300LF/IDO1/PDCD1LG2/FCGR2B/VSIG4/FN1/LYN/BTN2A2/RUNX3/SPN/CEBPB/LILRB2/CD300A/LAPTM5/TYROBP/LILRB1/SDC4/CASP3/CST7/FGL2/MILR1/LRFN5/PTPRC/IL2RA/ZC3H12A/TNFRSF14/IRF1/GAL/HFE/IL4I1/HLA-F 59

BP GO:0051216 cartilage development 60/2475 207/18870 1.21010178280816e-09 1.15324620698098e-07 8.376694546657e-08 HOXD3/TIMP1/SHOX2/MDK/HAND2/PITX1/LOXL2/SNAI1/HOXA5/HOXB3/STC1/CTSK/EFEMP1/HOXA3/CYTL1/MGP/HOXA11/SERPINH1/MMP13/NKX3-2/COL3A1/SNORC/CD44/TWSG1/RUNX1/DLX2/CCN1/ARID5A/BMP2/CCN4/ECM1/TYMS/GLI3/SCIN/SMPD3/HOXC4/FRZB/SFRP2/CHI3L1/COL1A1/RUNX2/BGN/OSR1/RUNX3/SLC26A2/CHRDL2/TGFBI/PRKG2/SNAI2/FOSL2/COL27A1/PTHLH/WNT10B/CNMD/ADAMTS7/OSR2/EVC/NOG/COL2A1/WNT7B 60

BP GO:0001909 leukocyte mediated cytotoxicity 46/2475 140/18870 1.28378093865997e-09 1.20434699308038e-07 8.74786912642805e-08 KLRC2/IL7R/IL18/EMP2/MR1/KLRC3/SPI1/NKG7/FCGR1A/ICAM1/CTSC/KLRC4/HAVCR2/RIPK3/GZMB/HLA-C/MICA/RAB27A/KLRC4-KLRK1/HLA-DRB1/MICB/F2RL1/FCGR2B/SCNN1B/HLA-DRA/NOS2/NECTIN2/IGHG1/TYROBP/HLA-B/LILRB1/HLA-A/PRF1/ULBP2/CD2/ULBP3/PTPRC/CXCL6/B2M/FCGR3A/PIK3R6/FADD/HFE/MYD88/TREM1/HLA-F 46

BP GO:0019884 antigen processing and presentation of exogenous antigen 24/2475 49/18870 1.38520081081943e-09 1.26894221816021e-07 9.21706162529846e-08 MR1/FCGR1A/HLA-DMA/HLA-DQA2/HLA-DQA1/CD74/HLA-DPA1/HLA-DOA/HLA-DRB1/FCGR2B/CTSL/HLA-DPB1/HLA-DRA/HLA-DQB1/HLA-DOB/HLA-A/HLA-DRB5/FCER1G/UNC93B1/HLA-DQB2/HLA-DMB/B2M/HLA-F/CTSS 24

BP GO:0050670 regulation of lymphocyte proliferation 66/2475 238/18870 1.39490650230802e-09 1.26894221816021e-07 9.21706162529846e-08 CD276/EFNB1/PYCARD/IL18/PLA2G2A/IL1A/TNFRSF4/LRRC32/ANXA1/CD40/CD70/TFRC/PAWR/CHRNB2/VAV3/FOXJ1/CD74/HAVCR2/TWSG1/RIPK3/AHR/LGALS3/TNFSF8/HLA-DPA1/PLA2G5/CD274/GPNMB/HLA-DRB1/CD3E/IDO1/PDCD1LG2/FCGR2B/CCL5/IGF2/VSIG4/SASH3/BST1/LYN/BTN2A2/SPN/CEBPB/HLA-DPB1/LILRB2/CD300A/TYROBP/IGFBP2/LILRB1/ZP3/RAC2/SDC4/CASP3/HLA-A/HLA-DMB/PTPRC/IL6/LMO1/FCGR3A/IL2RA/FADD/TNFRSF14/IRF1/GAL/MYD88/IL4I1/CLCF1/GPR183 66

BP GO:0007162 negative regulation of cell adhesion 80/2475 312/18870 1.55761365408228e-09 1.39580781777762e-07 1.01385598882371e-07 CLDN7/ASCL2/MDK/SEMA3E/PLA2G2A/SPI1/MIR221/LOXL3/LRRC32/ADAMDEC1/IL1RN/ANXA1/ADAM22/MMP12/C1QTNF1/THBS1/PAWR/FZD7/MMP2/FOXJ1/CD74/SOCS1/HAVCR2/TWSG1/LGALS3/RUNX1/DSCAM/CYP1B1/GBP1/BMP2/HSPG2/FBLN1/PLA2G5/JAG1/ANGPT2/CD274/GLI3/GPNMB/SPRY4/HLA-DRB1/MMP14/ANGPT1/JAK3/VEGFA/IDO1/PDCD1LG2/FCGR2B/VSIG4/MUC1/COL1A1/ARHGDIG/BTN2A2/RUNX3/SERPINE1/SPN/CEBPB/TACSTD2/LILRB2/TNC/CD300A/TNR/LAPTM5/LILRB1/SDC4/CASP3/TGFBI/FGL2/HOXA7/SNAI2/POSTN/PTPRC/AJAP1/IL2RA/ZC3H12A/SWAP70/TNFRSF14/IRF1/HFE/IL4I1/FXYD5 80

BP GO:0007596 blood coagulation 63/2475 224/18870 1.7805233406238e-09 1.57209737310372e-07 1.14190529414929e-07 CAV1/F5/THBD/MMRN1/ITGB3/PLAT/LCK/VWF/ITGA2/DGKI/C1QTNF1/THBS1/CD40/VAV3/F3/COL3A1/STXBP1/FLNA/HSPB1/RAB27A/FBLN1/ENTPD2/PLSCR1/F2RL1/PDPN/LYN/TFPI2/EMILIN2/F11R/MYL9/SERPINE1/PDGFA/VKORC1/SERPING1/PLAU/PPIA/CD36/EMILIN1/F2RL3/P2RY1/PROS1/F2RL2/DGKB/MYL12A/SERPINA1/FCER1G/ANXA5/FAP/PLEK/DGKK/F13A1/IL6/PIK3CG/PLA2G4A/TFPI/PLAUR/PROCR/CLIC1/ANXA2/SAA1/FERMT3/ADRA2B/FZD6 63

BP GO:0051251 positive regulation of lymphocyte activation 83/2475 330/18870 2.07934489191282e-09 1.79529258131042e-07 1.3040248894367e-07 CAV1/KLRC2/CD276/IL7R/MDK/EFNB1/PYCARD/IL18/LEF1/NFKBIZ/SPI1/IL1A/TNFRSF4/LCK/ANXA1/ACTL6B/TESPA1/CD40/HLA-DMA/CD70/TFRC/CHRNB2/VAV3/HLA-DQA2/HLA-DQA1/CD74/SOCS1/HAVCR2/MAP3K8/RHOH/RUNX1/HLA-DPA1/CCL2/CLEC7A/HLA-DOA/KLRC4-KLRK1/CD274/GLI3/TOX/HLA-DRB1/CD3E/MMP14/PDCD1LG2/CCL5/IL2RG/IGF2/SASH3/BST1/DUSP10/LYN/BTN2A2/RUNX3/SPN/HLA-DPB1/LILRB2/HLA-DRA/HLA-DQB1/TYROBP/IGFBP2/LILRB1/ZP3/LGALS1/HLA-DOB/HLA-A/HLA-DRB5/EFNB2/HLA-DQB2/HLA-DMB/PTPRC/DPP4/WNT10B/B2M/IL6/FCGR3A/IL2RA/PIK3R6/FADD/TNFRSF14/MYD88/IL4I1/CLCF1/GPR183/HLA-F 83

BP GO:0097530 granulocyte migration 49/2475 156/18870 2.11007352776588e-09 1.79529258131042e-07 1.3040248894367e-07 RARRES2/MDK/EMP2/CXCL13/IL1A/CCL26/ITGB2/CXCL8/CCL20/ANXA1/THBS1/VAV3/S100A12/CD74/PPBP/LGALS3/IL1R1/ADGRE2/CCL2/IL17RC/S100A8/S100A9/PTGER4/SLAMF8/CXCL10/C5AR1/CCL5/DPEP1/BST1/CCL18/PPIA/CXCL9/CD300A/THBS4/TNFAIP6/RAC2/CXCR2/FCER1G/MCOLN2/DPP4/CXCL6/CXCL11/PIK3CG/LBP/ITGA1/TGFB2/SAA1/MYD88/TREM1 49

BP GO:0002685 regulation of leukocyte migration 64/2475 230/18870 2.12301421174283e-09 1.79529258131042e-07 1.3040248894367e-07 ASCL2/RARRES2/MDK/ITGB3/PYCARD/CXCL13/SPI1/IL1A/MSN/CXCL8/ITGA2/CCL20/ANXA1/HMOX1/THBS1/TREM2/ICAM1/CD74/RIPK3/LGALS3/IL1R1/CCL2/ECM1/KLRC4-KLRK1/ITGA4/PTGER4/SLAMF8/MMP14/SMPD3/VEGFA/CXCL10/C5AR1/F2RL1/CCL5/PLVAP/BST1/TNFRSF18/LYN/FPR2/SERPINE1/SPN/EMILIN1/CD300A/THBS4/ZP3/TNFAIP6/RAC2/PDGFD/CXCR2/HOXA7/ADORA1/DPP4/IL6/LBP/CHST2/FADD/SWAP70/TNFRSF14/MYD88/PTN/CCR1/PLCB1/GCNT1/FUT9 64

BP GO:0017157 regulation of exocytosis 56/2475 190/18870 2.18267827945893e-09 1.82011116525991e-07 1.32205206312841e-07 IL1RAPL1/KLRC2/CDK5R2/STX1A/SYN1/SPI1/ITGB2/STXBP5L/PRKCG/ANXA1/CPLX2/RIMS1/VAMP8/KCNB1/DOC2A/STXBP1/IL13RA2/ADGRE2/RAB27A/RAB3A/S100A10/PRKCB/RAPGEF4/SMPD3/PRAM1/SYT7/F2RL1/FCGR2B/FES/LYN/SCAMP5/SYT1/SYT4/LRRK2/RAB3C/CD300A/RAP1B/PPFIA2/SV2B/P2RY1/ZP3/RAC2/SDC4/SYP/SDC1/VSNL1/SYT5/STXBP6/PCLO/SYT2/RIMS3/SYT13/ANXA2/CPLX1/HLA-F/RIMS2 56

BP GO:0048568 embryonic organ development 105/2475 453/18870 2.29968786959686e-09 1.89141451630953e-07 1.37384381308433e-07 STIL/HOXD3/SHOX2/ASCL2/RARRES2/CHRNA9/HAND2/HOXB2/SNAI1/HOXA5/RSPO3/LEF1/NKX2-5/TEAD4/MFAP2/HOXB3/EFEMP1/USH1C/CXCL8/E2F7/HOXA3/TTPA/HOXC11/SPRY2/HOXA11/HOXA2/MFAP5/ATP8A2/NKX3-2/EGFR/CITED1/E2F8/WNT16/APLNR/ST14/HOXD9/HOXB4/TEAD3/DLX2/CCN1/HOXD4/TEAD2/SPINT1/DLX5/ENG/DSCAML1/GLI3/HOXA9/MMP14/HOXC9/CASP8/VEGFA/HOXC4/GSC/FOLR1/FRZB/RYR2/IGF2/FZD5/RUNX2/ECE1/PCSK5/SOCS3/OSR1/CCDC40/IRX5/PDGFA/LIF/HOXD10/TBX15/CEBPB/CTHRC1/FZD2/TWIST1/GLI1/OTX1/NR2F2/CRB2/PHLDA2/STRA6/HOXA7/EPHA2/HOXB8/PLK4/GATA4/TBX1/ALDH1A3/COL27A1/ZIC1/RDH10/GDNF/STOX1/TGFB2/OSR2/EYA1/HOXA1/NOG/COL2A1/EN2/ADM/EN1/WNT7B/FZD6/NODAL/HOXB7 105

BP GO:0032944 regulation of mononuclear cell proliferation 66/2475 242/18870 2.93370705022331e-09 2.38026717966767e-07 1.7289257907646e-07 CD276/EFNB1/PYCARD/IL18/PLA2G2A/IL1A/TNFRSF4/LRRC32/ANXA1/CD40/CD70/TFRC/PAWR/CHRNB2/VAV3/FOXJ1/CD74/HAVCR2/TWSG1/RIPK3/AHR/LGALS3/TNFSF8/HLA-DPA1/PLA2G5/CD274/GPNMB/HLA-DRB1/CD3E/IDO1/PDCD1LG2/FCGR2B/CCL5/IGF2/VSIG4/SASH3/BST1/LYN/BTN2A2/SPN/CEBPB/HLA-DPB1/LILRB2/CD300A/TYROBP/IGFBP2/LILRB1/ZP3/RAC2/SDC4/CASP3/HLA-A/HLA-DMB/PTPRC/IL6/LMO1/FCGR3A/IL2RA/FADD/TNFRSF14/IRF1/GAL/MYD88/IL4I1/CLCF1/GPR183 66

BP GO:0006959 humoral immune response 69/2475 258/18870 3.16166384218424e-09 2.53101729446322e-07 1.83842432255008e-07 CXCL14/RARRES2/SLC11A1/CXCL13/C1RL/IGKV3-20/PI3/CFI/CXCL8/IGHG4/C1R/KLK7/TREM2/WFDC2/HPX/BCL3/S100A12/FOXJ1/H2BC8/IGHA1/PPBP/CCL2/SPON2/S100A9/IGHG2/C7/HLA-DRB1/CXCL10/IGHG3/FCGR2B/VSIG4/BST1/H2BC11/IGHA2/JCHAIN/LYZ/H2BC12/EXO1/SLPI/SERPING1/ANG/LTF/CXCL9/C2/HLA-DQB1/IGHG1/C1S/ZP3/RNASE3/HLA-A/CFD/IGHM/ALOX5/C3/C1QB/PTPRC/H2BC4/RNASE6/COLEC11/CXCL6/CXCL11/IL6/C1QC/C1QA/GNLY/CFH/TREM1/GPR183/NCR3LG1 69

BP GO:1903706 regulation of hemopoiesis 98/2475 417/18870 3.68828679086908e-09 2.91374656478657e-07 2.11642273886019e-07 LRRC17/H4C9/ASCL2/IL7R/MDK/BATF/IRF7/IL18/HOXA5/LEF1/NFKBIZ/SPI1/MIR221/LOXL3/FANCD2/ROR2/ANXA1/ACTL6B/TESPA1/TREM2/FOXJ1/LILRB3/CD74/SOCS1/H4C5/RHOH/RUNX1/STAT1/HLA-DOA/LOX/JAG1/NFAM1/GLI3/HOXA9/RAB7B/SLAMF8/SCIN/TOX/HLA-DRB1/MMP14/CASP8/JAK3/ZFP36/FCGR2B/CD101/IL2RG/SASH3/DUSP10/FES/LYN/FSTL3/TCIM/BTN2A2/CSF3/H4C8/RUNX3/LIF/CEBPB/LTF/LILRB2/HLA-DRA/ISG15/TYROBP/HLA-B/EVI2B/TNFRSF11B/LILRB1/TNFAIP6/H4C11/TMEM176A/TCF7/FGL2/HOXA7/CD2/SNAI2/HOXB8/POU4F1/ERFE/PTPRC/H4C3/HMGB2/WNT10B/B2M/C1QC/IL2RA/ZC3H12A/PIK3R6/FADD/IRF1/MEIS1/HCLS1/IL4I1/TMEM176B/PTN/CCR1/CDK6/CAMK4/FANCA 98

BP GO:0045071 negative regulation of viral genome replication 25/2475 55/18870 4.50406711678914e-09 3.51200246353273e-07 2.55097061720539e-07 APOBEC3B/OASL/OAS2/FAM111A/APOBEC3G/MX1/APOBEC3C/OAS1/IFITM3/PLSCR1/IFI16/CCL5/APOBEC3F/IFITM2/IFIH1/OAS3/SLPI/LTF/ISG15/TRIM6/RSAD2/BTBD17/ISG20/APOBEC3H/BST2 25

BP GO:0050817 coagulation 63/2475 229/18870 4.6086678780671e-09 3.54749255639934e-07 2.57674912534844e-07 CAV1/F5/THBD/MMRN1/ITGB3/PLAT/LCK/VWF/ITGA2/DGKI/C1QTNF1/THBS1/CD40/VAV3/F3/COL3A1/STXBP1/FLNA/HSPB1/RAB27A/FBLN1/ENTPD2/PLSCR1/F2RL1/PDPN/LYN/TFPI2/EMILIN2/F11R/MYL9/SERPINE1/PDGFA/VKORC1/SERPING1/PLAU/PPIA/CD36/EMILIN1/F2RL3/P2RY1/PROS1/F2RL2/DGKB/MYL12A/SERPINA1/FCER1G/ANXA5/FAP/PLEK/DGKK/F13A1/IL6/PIK3CG/PLA2G4A/TFPI/PLAUR/PROCR/CLIC1/ANXA2/SAA1/FERMT3/ADRA2B/FZD6 63

BP GO:0050870 positive regulation of T cell activation 67/2475 251/18870 5.79004921978758e-09 4.40043740703856e-07 3.19629232745902e-07 CAV1/CD276/IL7R/MDK/EFNB1/PYCARD/IL18/LEF1/NFKBIZ/IL1A/LCK/ANXA1/ACTL6B/TESPA1/HLA-DMA/CD70/TFRC/HLA-DQA2/HLA-DQA1/CD74/SOCS1/HAVCR2/MAP3K8/RHOH/RUNX1/HLA-DPA1/CCL2/HLA-DOA/KLRC4-KLRK1/CD274/GLI3/HLA-DRB1/CD3E/PDCD1LG2/CCL5/IL2RG/IGF2/SASH3/DUSP10/LYN/BTN2A2/RUNX3/SPN/HLA-DPB1/LILRB2/HLA-DRA/HLA-DQB1/IGFBP2/LILRB1/ZP3/LGALS1/HLA-DOB/HLA-A/HLA-DRB5/EFNB2/HLA-DQB2/HLA-DMB/PTPRC/DPP4/WNT10B/B2M/IL6/IL2RA/PIK3R6/FADD/TNFRSF14/IL4I1 67

BP GO:0007599 hemostasis 63/2475 231/18870 6.66401060002024e-09 5.00133995531519e-07 3.63276262051103e-07 CAV1/F5/THBD/MMRN1/ITGB3/PLAT/LCK/VWF/ITGA2/DGKI/C1QTNF1/THBS1/CD40/VAV3/F3/COL3A1/STXBP1/FLNA/HSPB1/RAB27A/FBLN1/ENTPD2/PLSCR1/F2RL1/PDPN/LYN/TFPI2/EMILIN2/F11R/MYL9/SERPINE1/PDGFA/VKORC1/SERPING1/PLAU/PPIA/CD36/EMILIN1/F2RL3/P2RY1/PROS1/F2RL2/DGKB/MYL12A/SERPINA1/FCER1G/ANXA5/FAP/PLEK/DGKK/F13A1/IL6/PIK3CG/PLA2G4A/TFPI/PLAUR/PROCR/CLIC1/ANXA2/SAA1/FERMT3/ADRA2B/FZD6 63

BP GO:0006887 exocytosis 85/2475 349/18870 6.8980288112307e-09 5.11305740526286e-07 3.71390946912655e-07 IL1RAPL1/KLRC2/CDK5R2/UNC13A/STX1A/SYN1/SYNGR3/SPI1/NKG7/ITGB2/TNFAIP2/STX1B/ANK1/NAPB/CHGA/STXBP5L/PRKCG/ANXA1/CPLX2/RIMS1/VAMP8/KCNB1/DOC2A/STXBP1/IL13RA2/S100A13/ADGRE2/RAB27A/RAB3A/S100A10/UNC13C/PRKCB/RAPGEF4/BRSK2/SCIN/SMPD3/EXOC3L2/ERC2/PRAM1/SYN2/SYT7/F2RL1/FCGR2B/CCL5/FES/LYN/SCAMP5/SYT1/SYT4/LRRK2/RAB3C/SNAP25/CD300A/RAP1B/PPFIA2/SV2B/P2RY1/SYTL3/ZP3/RAC2/SDC4/SYP/SDC1/VSNL1/LAT2/MILR1/SYT5/WIPF3/STXBP6/PCLO/PLEK/ARHGAP44/PIK3CG/SYNGR1/FCGR3A/SYT2/RIMS3/SYT13/ANXA2/MYO1G/CPLX1/GRP/CCR1/HLA-F/RIMS2 85

BP GO:0097529 myeloid leukocyte migration 65/2475 242/18870 7.45950800550934e-09 5.46181537379001e-07 3.96723256313545e-07 RARRES2/MDK/EMP2/CXCL13/SPI1/IL1A/CCL26/ITGB2/ROR2/CXCL8/CCL20/CHGA/ANXA1/THBS1/TREM2/MMP2/VAV3/S100A12/CD74/PPBP/LGALS3/IL1R1/ADGRE2/CCL2/IL17RC/S100A8/S100A9/PTGER4/SLAMF8/MMP14/VEGFA/CXCL10/C5AR1/CCL5/DPEP1/BST1/LYN/FPR2/SERPINE1/CCL18/PPIA/CXCL9/EMILIN1/CD300A/THBS4/TNFAIP6/RAC2/PDGFD/CXCR2/FCER1G/MCOLN2/DPP4/CXCL6/CXCL11/IL6/PIK3CG/LBP/ITGA1/TGFB2/SWAP70/SAA1/MYD88/TREM1/CCR1/PLCB1 65

BP GO:0002396 MHC protein complex assembly 14/2475 20/18870 7.6872685252049e-09 5.49456669349169e-07 3.9910217418451e-07 HLA-DMA/HLA-DQA2/HLA-DQA1/HLA-DPA1/HLA-DOA/HLA-DRB1/HLA-DPB1/HLA-DRA/HLA-DQB1/HLA-DOB/HLA-DRB5/HLA-DQB2/HLA-DMB/B2M 14

BP GO:0002501 peptide antigen assembly with MHC protein complex 14/2475 20/18870 7.6872685252049e-09 5.49456669349169e-07 3.9910217418451e-07 HLA-DMA/HLA-DQA2/HLA-DQA1/HLA-DPA1/HLA-DOA/HLA-DRB1/HLA-DPB1/HLA-DRA/HLA-DQB1/HLA-DOB/HLA-DRB5/HLA-DQB2/HLA-DMB/B2M 14

BP GO:0031589 cell-substrate adhesion 86/2475 356/18870 8.49915069918351e-09 6.00340009387033e-07 4.36061688504239e-07 COL8A1/HOXD3/ITGA5/WNT4/FERMT1/EFEMP2/MDK/ITGB3/COL26A1/EMP2/SEMA3E/KIF14/BVES/ITGB2/SIGLEC1/NPNT/ZYX/VWF/ITGA2/ECM2/NID1/COL5A3/MMP12/THBS1/ITGA3/NRP1/ITGA11/FZD7/COL3A1/FLNA/CD44/CCN1/NID2/GBP1/S100A10/ITGB4/FBLN1/JAG1/TRIP6/ANGPT2/ITGA4/SPRY4/CD3E/MMP14/CD63/ANGPT1/VEGFA/EGFLAM/CAMSAP3/PDPN/L1CAM/HAS2/BST1/COL1A1/LAMC1/FN1/AJUBA/MSLN/SERPINE1/PLAU/TACSTD2/ACTN1/CD36/EMILIN1/PPFIA2/LAMB1/RAC2/SDC4/FBLN5/ADAM9/HOXA7/RRAS/POSTN/ITGA7/ITGB1/AJAP1/EPHA3/PRKCE/PARVG/ITGA1/MICALL2/RHOD/ITGAL/MYO1G/FERMT3/CDK6 86

BP GO:0002456 T cell mediated immunity 42/2475 129/18870 8.98207532665064e-09 6.19866439784028e-07 4.50244864831381e-07 IL7R/IL18/EMP2/MR1/NFKBIZ/CD70/ICAM1/CTSC/RIPK3/AHR/HLA-C/IL1R1/MICA/ARID5A/RAB27A/CD8A/JAG1/HLA-DRB1/MICB/FCGR2B/SASH3/FZD5/SPN/HLA-DRA/NECTIN2/HLA-B/LILRB1/ZP3/HLA-A/RSAD2/PRF1/ULBP2/ULBP3/UNC93B1/PTPRC/B2M/IL6/FADD/HFE/MYO1G/IL4I1/HLA-F 42

BP GO:1990266 neutrophil migration 42/2475 129/18870 8.98207532665064e-09 6.19866439784028e-07 4.50244864831381e-07 MDK/EMP2/CXCL13/IL1A/CCL26/ITGB2/CXCL8/CCL20/VAV3/S100A12/CD74/PPBP/LGALS3/IL1R1/CCL2/S100A8/S100A9/SLAMF8/CXCL10/C5AR1/CCL5/DPEP1/BST1/CCL18/PPIA/CXCL9/THBS4/TNFAIP6/RAC2/CXCR2/FCER1G/MCOLN2/DPP4/CXCL6/CXCL11/PIK3CG/LBP/ITGA1/TGFB2/SAA1/MYD88/TREM1 42

BP GO:0060078 regulation of postsynaptic membrane potential 46/2475 148/18870 9.40392817919873e-09 6.41604372589877e-07 4.66034383330387e-07 KCNA1/GLRA3/CHRNA9/HCN1/STX1A/GRIA4/STX1B/GABBR1/CHRNB2/RIMS1/CHRNA1/GABRA3/GABRA5/GABRD/RGS7BP/GABRA4/CUX2/GRIA2/SHANK1/CHRM1/GRIN3A/NRXN1/SLC8A3/GRIN2A/GRM5/SLC8A2/GRIN1/LRRK2/GABRA1/CBLN1/GABRB2/CELF4/INSYN2B/GABRG2/GRIK1/P2RX6/GRM1/GABRG1/ADORA1/MAPK8IP2/CHRNA4/RGS4/SLC29A1/GRIN2B/GABRE/RIMS2 46

BP GO:0035249 synaptic transmission, glutamatergic 38/2475 111/18870 9.62809212795861e-09 6.49517585800714e-07 4.71782207996837e-07 UNC13A/GRIA4/CACNG2/ROR2/NAPB/SLC17A8/ABCC8/DGKI/STXBP1/CNR1/CCL2/GRIA2/SLC17A7/UNC13C/OXTR/OPHN1/GRIN3A/NRXN1/GRIN2A/HTR2A/DKK1/GRM5/SYT1/FRRS1L/GRIN1/LRRK2/SLC17A6/GRM2/TNR/GRIK1/CDH8/GRM1/ADORA1/MAPK8IP2/NTRK1/GRIN2B/CACNG5/CACNG3 38

BP GO:0002819 regulation of adaptive immune response 58/2475 208/18870 1.09275856599817e-08 7.27684731583177e-07 5.28559529252271e-07 ASCL2/IL7R/BATF/IRF7/PYCARD/IL18/MR1/SLC11A1/NFKBIZ/LOXL3/ANXA1/TREM2/CD40/FCGR1A/TFRC/HPX/FOXJ1/HAVCR2/RIPK3/AHR/HLA-C/IL1R1/MICA/ARID5A/CLEC7A/CD274/HLA-DRB1/TNFAIP3/SAMSN1/MICB/JAK3/FCGR2B/SASH3/FZD5/DUSP10/SPN/HLA-DRA/NECTIN2/HLA-B/LILRB1/ZP3/HLA-A/RSAD2/ULBP2/ULBP3/C3/PTPRC/B2M/IL6/PLA2G4A/ZC3H12A/FADD/TNFRSF14/IRF1/HFE/IL4I1/CLCF1/HLA-F 58

BP GO:0071219 cellular response to molecule of bacterial origin 64/2475 239/18870 1.10291989630362e-08 7.27684731583177e-07 5.28559529252271e-07 MAP2K3/LY96/GBP5/PYCARD/TLR1/CXCL13/NFKBIZ/SPI1/IL1A/TSPO/CXCL8/GBP2/LITAF/TREM2/CD40/HAVCR2/AHR/PPBP/SBNO2/ARID5A/CCL2/CMPK2/MMP9/CD180/CASP7/SPON2/KLRC4-KLRK1/CD274/CASP1/TNFAIP3/CXCL10/TLR2/ZFP36/CARD16/PDCD1LG2/FCGR2B/SCIMP/FZD5/LYN/GBP3/CSF3/SERPINE1/CD14/LTF/LILRB2/CXCL9/NOS2/CD36/CCR5/LILRB1/LILRA2/TRIM5/ADAM9/VIM/CDK4/HMGB2/CXCL6/PRKCE/CXCL11/IL6/LBP/ZC3H12A/IRAK2/MYD88 64

BP GO:0050803 regulation of synapse structure or activity 66/2475 250/18870 1.2064290974725e-08 7.87326119698355e-07 5.71880520689765e-07 IL1RAPL1/CDK5R1/DNM3/ROR2/SLC17A8/PTPRT/LINGO2/ADGRB3/TREM2/NEFL/NTNG2/CHRNB2/FRMPD4/SLITRK4/SLIT1/NEUROD2/SHANK2/NEGR1/CUX2/C1QL3/SLC17A7/SRPX2/OXTR/LRRTM3/CLSTN2/NRXN1/CBLN2/TLR2/FCGR2B/DKK1/ABI3/SRGN/ASIC2/IL10RA/LRRK2/SNAP25/LILRB2/SLC17A6/ARC/PAK3/SLITRK1/PPFIA2/IGSF9/CBLN1/ZNF804A/SEMA3F/CAMKV/IL1RAP/LZTS1/RAP2A/CDH8/AMIGO2/LRFN5/CTTNBP2/ICAM5/ITGB1/LRTM2/FZD1/NTRK1/ARHGAP44/TUBA1A/NEURL1/SLITRK5/GRIN2B/GPR158/CDC20 66

BP GO:0023061 signal release 108/2475 484/18870 1.24230307251652e-08 8.02020177138622e-07 5.8255366490503e-07 HTR1A/UNC13A/STX1A/SYN1/IL1A/TSPO/SLC32A1/SLC16A10/STX1B/NAPB/SPP1/CHGA/VGF/PRKCG/IL1RN/ANXA1/ABCC8/C1QTNF1/GABBR1/CPLX2/CHRNB2/ACVR1C/RIMS1/VAMP8/KCNB1/DOC2A/CRH/STXBP1/ASIC1/CNR1/GLUD1/RAB3A/MYOF/UNC13C/PRKCB/RAPGEF4/NNAT/OXTR/FOXD1/BRSK2/SNCG/HLA-DRB1/NRXN1/SMPD3/ERC2/SYN2/SYT7/F2RL1/HTR2A/CCL5/VIP/IL11/GPR27/LYN/HTR2C/SYT1/PCSK5/SYT4/PTGES/LRRK2/LIF/CAMK2A/KCNJ11/SNAP25/NOS2/GRM2/RAP1B/PPFIA2/SV2B/P2RY1/LILRB1/HRH3/F2RL2/SYP/NKX6-1/VSNL1/SYN3/SYT5/CRY2/OSM/GCK/ALOX5/PCLO/GLP1R/TUNAR/TRH/ADORA1/PRKCE/CHRNA4/GDNF/IL6/PPARG/PLA2G4A/SYT2/RIMS3/SYT13/GAL/HFE/TCIRG1/AQP1/CPLX1/GPR158/GRP/UCP2/ADRA2B/PLCB1/MAOB/RIMS2 108

BP GO:0050878 regulation of body fluid levels 87/2475 365/18870 1.36893111971001e-08 8.74368344972226e-07 6.35104325751241e-07 CAV1/F5/THBD/MMRN1/APLN/ITGB3/EMP2/PLAT/LCK/CYBA/OAS2/VWF/ITGA2/DGKI/C1QTNF1/THBS1/CD40/VAV3/VAMP8/F3/COL3A1/STXBP1/FLNA/APLNR/HSPB1/RAB27A/FBLN1/OXTR/CHRM1/VEGFA/ENTPD2/PLSCR1/F2RL1/PDPN/SCNN1B/VIP/SOCS2/HAS2/LYN/TFPI2/EMILIN2/KCNN4/HK2/F11R/MYL9/SERPINE1/PDGFA/VKORC1/SERPING1/PLAU/AQP5/PPIA/CD36/EMILIN1/F2RL3/P2RY1/PROS1/F2RL2/DGKB/MYL12A/SERPINA1/FCER1G/ANXA5/MYO5B/FAP/FOSL2/PLEK/DGKK/ADORA1/PRKCE/F13A1/IL6/PIK3CG/PLA2G4A/TFPI/PLAUR/PROCR/NEURL1/CLIC1/ANXA2/SAA1/AQP1/SLC29A1/ADM/FERMT3/ADRA2B/FZD6 87

BP GO:0070663 regulation of leukocyte proliferation 69/2475 267/18870 1.45199262556008e-08 9.17659339353971e-07 6.66549080077054e-07 CD276/EFNB1/PYCARD/IL18/PLA2G2A/IL1A/TNFRSF4/LRRC32/ANXA1/CD40/CD70/TFRC/PAWR/CHRNB2/VAV3/FOXJ1/CD74/HAVCR2/TWSG1/RIPK3/AHR/LGALS3/TNFSF8/HLA-DPA1/PLA2G5/CD274/GPNMB/HLA-DRB1/TNFAIP3/CD3E/IDO1/PDCD1LG2/FCGR2B/CCL5/IGF2/VSIG4/SASH3/BST1/LYN/BTN2A2/SPN/CEBPB/HLA-DPB1/LILRB2/CD300A/TYROBP/IGFBP2/LILRB1/ZP3/RAC2/SDC4/CASP3/HLA-A/CSF2RB/HLA-DMB/PTPRC/IL6/LMO1/FCGR3A/IL2RA/FADD/TNFRSF14/IRF1/GAL/MYD88/IL4I1/CLCF1/GPR183/BST2 69

BP GO:0002263 cell activation involved in immune response 76/2475 305/18870 1.51644388768415e-08 9.48409281422463e-07 6.88884542398623e-07 KLRC2/ASCL2/MDK/BATF/PYCARD/IL18/SLC11A1/LEF1/NFKBIZ/SPI1/NKG7/RELB/LOXL3/ITGB2/CHGA/LCP1/ANXA1/TREM2/CD40/TFRC/CPLX2/ICAM1/BCL3/VAMP8/CD74/STXBP1/HAVCR2/LGALS3/IL13RA2/SBNO2/S100A13/ADGRE2/RAB27A/CD180/GAPT/PTGER4/HLA-DRB1/JAK3/PRAM1/F2RL1/NMI/FCGR2B/SCNN1B/FES/LYN/EXO1/SOCS3/TCIM/SPN/HLA-DRA/CD300A/TYROBP/LILRB1/LILRA2/RAC2/LGALS1/IFI35/FGL2/LAT2/MILR1/FCER1G/HLA-DMB/PTPRC/PRKCE/IL6/PIK3CG/LBP/FCGR3A/ZC3H12A/ITGAL/SWAP70/MYD88/GRP/CLCF1/GPR183/HLA-F 76

BP GO:0034329 cell junction assembly 101/2475 446/18870 1.66862425991704e-08 1.03282680995277e-06 7.50201878332751e-07 CLDN7/IL1RAPL1/CAV1/VMP1/ITGA5/WNT4/ITGB3/SNAI1/LGI2/DNM3/ITGA2/ECT2/ACE/FGF13/LINGO2/THBS1/ADGRB3/MPP7/CLDN1/NTNG2/CHRNB2/HOPX/NRP1/NRG3/CDH4/SLITRK4/SLIT1/APLNR/DSCAM/SHANK2/NEGR1/CUX2/GABRB3/C1QL3/CDH22/CDH9/S100A10/GAP43/ITGB4/SRPX2/CDH6/TRIP6/OXTR/LRRTM3/OPHN1/CLSTN2/MMP14/NRXN1/CBLN2/VEGFA/CDH19/TLR2/CAMSAP3/BSN/DKK1/TBX5/ABI3/FZD5/LAMC1/FN1/AJUBA/GJB2/ASIC2/F11R/SNAP25/ZDHHC12/ACTN1/GJC1/SLITRK1/RAP1B/GABRA1/CBLN1/GABRB2/SDC4/IL1RAP/LZTS1/CAPZA1/GABRG2/NPTX1/CNTNAP2/RAP2A/EPHA2/SPTBN2/RHOC/CDH8/AMIGO2/LRFN5/SNAI2/POU4F1/ICAM5/PCLO/CLDN23/LRTM2/FZD1/EPHA3/NTRK1/MICALL2/RHOD/SLITRK5/GJD3/CDH18 101

BP GO:1903305 regulation of regulated secretory pathway 41/2475 127/18870 1.76785371525642e-08 1.08308099044893e-06 7.86704397669963e-07 KLRC2/CDK5R2/STX1A/SYN1/SPI1/ITGB2/PRKCG/RIMS1/VAMP8/KCNB1/DOC2A/STXBP1/IL13RA2/ADGRE2/RAB27A/RAB3A/PRKCB/PRAM1/SYT7/F2RL1/FCGR2B/FES/LYN/SCAMP5/SYT1/SYT4/LRRK2/CD300A/RAP1B/PPFIA2/SV2B/P2RY1/ZP3/RAC2/SYP/SYT5/SYT2/RIMS3/SYT13/HLA-F/RIMS2 41

BP GO:0002366 leukocyte activation involved in immune response 75/2475 301/18870 1.88639250927096e-08 1.14403036622857e-06 8.30975456237063e-07 KLRC2/ASCL2/MDK/BATF/PYCARD/IL18/SLC11A1/LEF1/NFKBIZ/SPI1/NKG7/RELB/LOXL3/ITGB2/CHGA/LCP1/ANXA1/TREM2/CD40/TFRC/CPLX2/ICAM1/BCL3/VAMP8/CD74/STXBP1/HAVCR2/LGALS3/IL13RA2/SBNO2/S100A13/ADGRE2/RAB27A/CD180/GAPT/PTGER4/HLA-DRB1/JAK3/PRAM1/F2RL1/NMI/FCGR2B/SCNN1B/FES/LYN/EXO1/SOCS3/SPN/HLA-DRA/CD300A/TYROBP/LILRB1/LILRA2/RAC2/LGALS1/IFI35/FGL2/LAT2/MILR1/FCER1G/HLA-DMB/PTPRC/PRKCE/IL6/PIK3CG/LBP/FCGR3A/ZC3H12A/ITGAL/SWAP70/MYD88/GRP/CLCF1/GPR183/HLA-F 75

BP GO:0048791 calcium ion-regulated exocytosis of neurotransmitter 14/2475 21/18870 2.02625613698706e-08 1.21656418464703e-06 8.83660965846041e-07 UNC13A/STX1B/RIMS1/STXBP1/RAB3A/UNC13C/SYT7/SYT1/SYT4/SNAP25/SYT5/SYT2/RIMS3/RIMS2 14

BP GO:0002703 regulation of leukocyte mediated immunity 65/2475 249/18870 2.48618696925306e-08 1.47792738251439e-06 1.07350418067904e-06 KLRC2/IL7R/IL18/MR1/KLRC3/SPI1/ITGB2/TREM2/CD40/FCGR1A/TFRC/ICAM1/HPX/VAMP8/FOXJ1/KLRC4/STXBP1/HAVCR2/RIPK3/AHR/IL13RA2/HLA-C/IL1R1/MICA/ADGRE2/ARID5A/CLEC7A/KLRC4-KLRK1/HLA-DRB1/MICB/JAK3/PRAM1/F2RL1/FCGR2B/SCIMP/SASH3/FZD5/FES/LYN/SPN/HLA-DRA/NOS2/CD300A/NECTIN2/TYROBP/HLA-B/LILRB1/ZP3/RAC2/HLA-A/RSAD2/ULBP2/ULBP3/C3/PTPRC/CXCL6/B2M/IL6/PIK3R6/FADD/HFE/IL4I1/CLCF1/HLA-F/BST2 65

BP GO:0001666 response to hypoxia 74/2475 298/18870 2.72754248455474e-08 1.60447305092504e-06 1.16542162242408e-06 CAV1/MDM2/ASCL2/LOXL2/IL1A/PLK3/STC1/LMNA/PLAT/CA9/ITGA2/PLOD1/ACE/SOD2/HMOX1/THBS1/TREM2/TFRC/CHRNB2/MMP2/MIR210/KCNMB1/BMP2/HSPG2/AK4/ANGPT2/SCN2A/NGB/ANGPTL4/MMP14/VEGFA/TLR2/SLC8A3/MDM4/RYR2/CXCR4/HILPDA/CHCHD2/AJUBA/KCNK3/HK2/SLC2A4/SOD3/LIF/PLAU/KCNJ11/PDLIM1/ANG/NOS2/CCNA2/TWIST1/PDK1/CASP3/BRIP1/ACAA2/PGK1/VASN/POSTN/FOSL2/AGTRAP/ADORA1/DPP4/PRKCE/CHRNA4/PPARG/STOX1/PLOD2/TGFB2/AQP1/SLC29A1/ADM/UCP2/MGARP/MT3 74

BP GO:0010038 response to metal ion 85/2475 359/18870 2.77205806839779e-08 1.60447305092504e-06 1.16542162242408e-06 CAV1/KCNA1/MDM2/KCNC1/MT1M/LOXL2/SLC11A1/IL1A/NCF1/TSPO/CACNG2/IQGAP1/HAMP/MT1A/ECT2/SOD2/MT2A/HMOX1/ABCC8/THBS1/MT1DP/TFRC/CLDN1/KCNB1/SLC39A8/KCNMB1/EGFR/IMPA2/NEUROD2/SLC30A3/MT1E/MMP9/ITPKC/S100A8/CPNE9/CLIC4/CASP8/SMPD3/CNGA3/SYT7/PLSCR1/RYR2/KCNH1/DPEP1/KCNK3/SYT1/MAPT/S100A16/SYT4/ALOX5AP/SOD3/DLG2/LRRK2/CD14/MT1H/RASAL1/PCNA/TNFRSF11B/MTTP/ADGRV1/SLC25A24/CASP3/SDC1/ADAM9/KCNC2/NPTX1/FBP1/SYT5/DTYMK/RYR3/HPCA/CDK1/B2M/CPNE8/SYT2/TUBA1A/SYT13/ANK3/HFE/TCIRG1/AQP1/UCP2/MAOB/MT3/KCNIP2 85

BP GO:0042098 T cell proliferation 58/2475 213/18870 2.77923379907069e-08 1.60447305092504e-06 1.16542162242408e-06 CD276/WNT4/EFNB1/PYCARD/IL18/SLC11A1/PLA2G2A/IL1A/MSN/TNFRSF4/LRRC32/ANXA1/CD70/TFRC/PAWR/ELF4/FOXJ1/HAVCR2/TWSG1/RIPK3/LGALS3/TNFSF8/HLA-DPA1/CD151/PLA2G5/CD274/GPNMB/HLA-DRB1/CD3E/IDO1/PDCD1LG2/CCL5/IGF2/VSIG4/SASH3/BTN2A2/SH2D2A/SPN/CEBPB/HLA-DPB1/LILRB2/IGFBP2/LILRB1/ZP3/RAC2/SDC4/CASP3/HLA-A/HLA-DMB/PTPRC/IL6/PIK3CG/LMO1/IL2RA/FADD/TNFRSF14/IRF1/IL4I1 58

BP GO:0001763 morphogenesis of a branching structure 56/2475 203/18870 2.93445818143317e-08 1.67795113536426e-06 1.21879300708548e-06 SHOX2/WNT4/MDK/HOXD11/SEMA3E/HOXA5/RSPO3/LEF1/NPNT/HS3ST3B1/SPRY2/FGF13/HOXA11/NRP1/CITED1/ST14/CSMD1/DLX2/BMP2/SOX8/AREG/SPINT1/ENG/FOXD1/TIMELESS/MET/GLI3/CLIC4/GREB1L/MMP14/VEGFA/SFRP2/HGF/FZD5/COL4A1/SOCS3/LRRK2/PDGFA/TACSTD2/TNC/SOX10/EPHA2/HS3ST3A1/SNAI2/RDH10/HOXB13/GDNF/CTSZ/CELSR1/SPRY1/EYA1/NOG/ADM/HOXD13/DLL4/HOXB7 56

BP GO:0002573 myeloid leukocyte differentiation 62/2475 235/18870 3.39430880029323e-08 1.9225877393359e-06 1.39648672885947e-06 LRRC17/BATF/IRF7/LEF1/SPI1/RELB/TSPAN2/ROR2/TREM2/TFRC/LILRB3/CD74/SOCS1/OSCAR/SBNO2/RUNX1/BMP2/CCN4/MMP9/BATF2/HLA-DRB1/SNX10/CASP8/BATF3/VEGFA/TLR2/IFI16/F2RL1/CD101/FAM20C/FES/DHRS2/LYN/FSTL3/CSF3/LIF/CEBPB/LTF/TYROBP/EVI2B/TNFRSF11B/LILRB1/TNFAIP6/LTBR/HOXA7/EPHA2/POU4F1/ERFE/FOSL2/PPARG/C1QC/CD109/FADD/ANXA2/TCIRG1/MYD88/HCLS1/CCR1/UCP2/GPR183/CDK6/CAMK4 62

BP GO:0006968 cellular defense response 23/2475 52/18870 3.52243224807195e-08 1.97651245022654e-06 1.43565536682362e-06 KLRC2/LY96/KLRC3/FOSL1/NCF1/DCDC2/NCF2/C5AR1/CLEC5A/SPN/LILRB2/CXCL9/TYROBP/CCR5/LGALS3BP/LSP1/CXCR2/PRF1/ITGB1/LBP/GNLY/TCIRG1/CD300C 23

BP GO:0071621 granulocyte chemotaxis 41/2475 130/18870 3.72441839269499e-08 2.07050074349451e-06 1.50392450301514e-06 RARRES2/MDK/CXCL13/CCL26/ITGB2/CXCL8/CCL20/ANXA1/THBS1/VAV3/S100A12/CD74/PPBP/LGALS3/ADGRE2/CCL2/IL17RC/S100A8/S100A9/CXCL10/C5AR1/CCL5/DPEP1/BST1/CCL18/PPIA/CXCL9/THBS4/TNFAIP6/RAC2/CXCR2/FCER1G/DPP4/CXCL6/CXCL11/PIK3CG/LBP/ITGA1/TGFB2/SAA1/TREM1 41

BP GO:0050433 regulation of catecholamine secretion 24/2475 56/18870 3.7750643213385e-08 2.0794024023226e-06 1.51039029293147e-06 STX1A/CHGA/GABBR1/CHRNB2/KCNB1/CRH/CNR1/PRKCB/OXTR/SNCG/SYT7/HTR2A/VIP/SYT1/SYT4/GRM2/P2RY1/HRH3/SYT5/CHRNA4/GDNF/SYT2/SYT13/ADRA2B 24

BP GO:0001649 osteoblast differentiation 66/2475 257/18870 3.85698080483335e-08 2.10521025020177e-06 1.52913602626072e-06 SHOX2/WNT4/HAND2/SNAI1/LEF1/WWTR1/NPNT/SPP1/GDPD2/HOXA2/DNAI3/GABBR1/ITGA11/SMOC1/MIR210/CITED1/TWSG1/IGFBP3/IGFBP5/CCN1/BMP2/SOX8/AREG/CCN4/MRC2/LOX/JAG1/DLX5/GLI3/NELL1/SFRP2/CLEC5A/IGF2/HGF/COL1A1/FAM20C/RUNX2/CEBPD/TENT5A/CEBPB/CTHRC1/LTF/TNC/COL6A1/TWIST1/GLI1/H3-3A/TNFAIP6/TWIST2/TPM4/GDF10/EPHA2/SNAI2/ERFE/FOSL2/PTHLH/FZD1/WNT10B/IL6/PPARG/CLIC1/TCIRG1/NOG/IBSP/WNT7B/CDK6 66

BP GO:0030593 neutrophil chemotaxis 36/2475 107/18870 3.90770844184637e-08 2.11368301665276e-06 1.53529028682499e-06 MDK/CXCL13/CCL26/ITGB2/CXCL8/CCL20/VAV3/S100A12/CD74/PPBP/LGALS3/CCL2/S100A8/S100A9/CXCL10/C5AR1/CCL5/DPEP1/BST1/CCL18/PPIA/CXCL9/THBS4/TNFAIP6/RAC2/CXCR2/FCER1G/DPP4/CXCL6/CXCL11/PIK3CG/LBP/ITGA1/TGFB2/SAA1/TREM1 36

BP GO:0030574 collagen catabolic process 21/2475 45/18870 4.36455767177324e-08 2.33971466618987e-06 1.69947015358614e-06 CTSK/MMP12/MMP13/MMP2/MMP1/MMP9/MRC2/MMP14/MMP25/MMP19/CTSB/CTSL/ADAMTS14/CST3/MMP7/ADAMTS2/ADAMTS3/FAP/ITGB1/MMP11/CTSS 21

BP GO:0051960 regulation of nervous system development 103/2475 466/18870 4.49229961860387e-08 2.37834506531684e-06 1.72752964788521e-06 IL1RAPL1/CUL7/SHOX2/ASCL2/MCF2/MDK/SEMA3E/KIF14/LEF1/MIR221/TSPO/HOXB3/SPP1/TYMP/ACE/FGF13/ABCC8/LINGO2/ADGRB3/TREM2/NEFL/DLL3/NRP1/SEMA3A/CTSC/CDH4/SEMA3D/SLITRK4/SLIT1/DSCAM/HAPLN1/DLX2/CUX2/RTN4R/BMP2/SOX8/NR2E1/S100A10/SRPX2/SPINT1/OXTR/LRRTM3/GLI3/CLSTN2/FEZF2/NRXN1/CBLN2/GSX2/VEGFA/TLR2/BRINP1/DKK1/L1CAM/CXCR4/SEMA6B/GRM5/DUSP10/FN1/LYN/ASIC2/DMRTA2/MAPT/SYT4/LIF/CHODL/SOX10/SLITRK1/TNR/CBLN1/NCMAP/OTP/SEMA3F/IL1RAP/CST7/NKX6-1/HAPLN3/DRAXIN/FSTL4/AMIGO2/POU4F1/DAAM2/ITGB1/HMGB2/LRTM2/WDR62/TRPV2/B2M/NTRK1/IL6/NEURL1/SLITRK5/XRCC2/ANXA2/NOG/ASPM/ISLR2/EZH2/RASSF10/PTN/CLCF1/DLL4/MT3/TP73 103

BP GO:0061448 connective tissue development 71/2475 285/18870 4.51584506072819e-08 2.37834506531684e-06 1.72752964788521e-06 HOXD3/TIMP1/SHOX2/MDK/HAND2/PITX1/ITGB3/LOXL2/SNAI1/HOXA5/SPI1/HOXB3/STC1/CTSK/EFEMP1/HOXA3/CYTL1/MGP/HOXA11/SERPINH1/MMP13/NKX3-2/COL3A1/SNORC/CD44/TWSG1/RUNX1/DLX2/CCN1/ARID5A/BMP2/SOX8/CCN4/LOX/ECM1/TYMS/GPR4/FOXD1/GLI3/ACTA2/SCIN/SMPD3/HOXC4/FRZB/SFRP2/CHI3L1/COL1A1/RUNX2/BGN/OSR1/RUNX3/SLC26A2/CHRDL2/PDGFD/TGFBI/PRKG2/SNAI2/RXFP1/FOSL2/COL27A1/PTHLH/WNT10B/COL5A1/CNMD/PPARG/ADAMTS7/OSR2/EVC/NOG/COL2A1/WNT7B 71

BP GO:0051783 regulation of nuclear division 44/2475 146/18870 5.42145310050358e-08 2.83046994916726e-06 2.05593411399417e-06 NEK2/CENPF/SPC24/CUL7/MAD2L1/WNT4/CDCA2/IL1A/KNTC1/UBE2C/CCDC8/CCNB1/TOM1L1/DLGAP5/BIRC5/SMPD3/HASPIN/ESPL1/CAV2/PKMYT1/SPHK1/IGF2/TTK/NPM2/ZWILCH/LIF/MKI67/NUF2/AURKA/GPR3/TRIP13/BUB1B/CDC25C/BUB1/AURKB/RAD51AP1/PLK1/CDCA8/NDC80/KNL1/ZWINT/PLCB1/CHEK1/CDC20 44

BP GO:0007565 female pregnancy 52/2475 186/18870 5.62213805600063e-08 2.90994111105412e-06 2.11365861760532e-06 VMP1/TIMP1/THBD/ITGA5/WNT4/ITGB3/EMP2/FOSL1/TEAD4/ARHGDIB/STC1/ITGA2/SPP1/ACE/ABCC8/ITGA3/ACVR1C/MMP2/CRH/HAVCR2/IGFBP5/CSMD1/TEAD3/CNR1/HSPG2/MMP9/ITGB4/FBLN1/DSG2/OXTR/ANGPT2/IGFBP7/VEGFA/IDO1/CTSB/SYDE1/PRLHR/GJB2/PCSK5/CYP27B1/LIF/IGFBP2/H3-3A/NR2F2/PTHLH/TPPP3/HFE/ADM/PTN/UCP2/ADRA2B/NODAL 52

BP GO:0071216 cellular response to biotic stimulus 67/2475 265/18870 5.8497818616703e-08 3.00188805961269e-06 2.18044500700855e-06 MAP2K3/LY96/GBP5/DDIT3/PYCARD/TLR1/CXCL13/NFKBIZ/SPI1/IL1A/TSPO/CXCL8/GBP2/LITAF/TREM2/CD40/HAVCR2/AHR/PPBP/SBNO2/ARID5A/CCL2/CLEC7A/CMPK2/MMP9/CD180/CASP7/SPON2/KLRC4-KLRK1/CD274/CASP1/TNFAIP3/CXCL10/TLR2/ZFP36/CARD16/PDCD1LG2/FCGR2B/SCIMP/FZD5/LYN/GBP3/CSF3/SERPINE1/CD14/LTF/LILRB2/CXCL9/NOS2/CD36/CCR5/LILRB1/LILRA2/TRIM5/ADAM9/VIM/CDK4/HMGB2/CXCL6/PRKCE/CXCL11/IL6/LBP/ZC3H12A/IRAK2/MYD88/EME1 67

BP GO:0006958 complement activation, classical pathway 20/2475 42/18870 5.98047671501984e-08 3.04294764381179e-06 2.2102689589944e-06 C1RL/CFI/IGHG4/C1R/TREM2/IGHA1/IGHG2/C7/IGHG3/IGHA2/SERPING1/C2/IGHG1/C1S/IGHM/C3/C1QB/C1QC/C1QA/NCR3LG1 20

BP GO:0007088 regulation of mitotic nuclear division 38/2475 118/18870 6.25717490974949e-08 3.15007313456611e-06 2.28808040192633e-06 NEK2/CENPF/SPC24/CUL7/MAD2L1/CDCA2/IL1A/KNTC1/UBE2C/CCDC8/CCNB1/TOM1L1/DLGAP5/BIRC5/SMPD3/HASPIN/ESPL1/CAV2/PKMYT1/SPHK1/IGF2/TTK/ZWILCH/MKI67/NUF2/AURKA/TRIP13/BUB1B/CDC25C/BUB1/AURKB/PLK1/CDCA8/NDC80/KNL1/ZWINT/CHEK1/CDC20 38

BP GO:0050807 regulation of synapse organization 63/2475 244/18870 6.295948969819e-08 3.15007313456611e-06 2.28808040192633e-06 IL1RAPL1/CDK5R1/DNM3/ROR2/PTPRT/LINGO2/ADGRB3/TREM2/NEFL/NTNG2/CHRNB2/FRMPD4/SLITRK4/SLIT1/NEUROD2/SHANK2/NEGR1/CUX2/C1QL3/SRPX2/OXTR/LRRTM3/CLSTN2/NRXN1/CBLN2/TLR2/FCGR2B/DKK1/ABI3/SRGN/ASIC2/IL10RA/LRRK2/SNAP25/LILRB2/ARC/PAK3/SLITRK1/PPFIA2/IGSF9/CBLN1/ZNF804A/SEMA3F/CAMKV/IL1RAP/LZTS1/RAP2A/CDH8/AMIGO2/LRFN5/CTTNBP2/ICAM5/ITGB1/LRTM2/FZD1/NTRK1/ARHGAP44/TUBA1A/NEURL1/SLITRK5/GRIN2B/GPR158/CDC20 63

BP GO:1903131 mononuclear cell differentiation 105/2475 481/18870 6.50457165330851e-08 3.22755770301358e-06 2.34436192776487e-06 ASCL2/WNT4/IL7R/MDK/BATF/IRF7/IL18/MR1/LEF1/NFKBIZ/SPI1/IL1A/RELB/LOXL3/FANCD2/CTSK/LCK/ANXA1/ACTL6B/TESPA1/TREM2/CD3D/BCL3/FZD7/FOXJ1/CD74/SOCS1/RIPK3/TNFSF8/RHOH/RUNX1/HLA-DOA/CD79A/CD8A/NFAM1/BATF2/ITGA4/GLI3/PTGER4/SLAMF8/TOX/HLA-DRB1/CD3E/MMP14/JAK3/BATF3/VEGFA/IFI16/F2RL1/FCGR2B/IL2RG/SASH3/IL11/FZD5/DUSP10/CTSL/FES/HDAC4/DHRS2/LYN/RUNX2/SOCS3/CMTM7/BTN2A2/RUNX3/SPN/CEBPB/LILRB2/HLA-DRA/HLA-B/KDELR1/LILRB1/LGALS1/TMEM176A/TCF7/RSAD2/FGL2/LTBR/HOXA7/FCER1G/CD2/IL9/FOSL2/PTPRC/ITGB1/WNT10B/B2M/NTRK1/IL6/PPARG/IL2RA/ZC3H12A/PIK3R6/FADD/IRF1/TCIRG1/IL4I1/TMEM176B/EZH2/CLCF1/DLL4/GPR183/CDK6/CAMK4/FANCA 105

BP GO:0007613 memory 39/2475 123/18870 6.75951165762692e-08 3.32291281119959e-06 2.41362386072441e-06 ITGA5/MDK/CPEB3/FGF13/ABCC8/NTAN1/ITGA3/TREM2/PAK5/CHRNB2/CSMD1/ASIC1/CUX2/CNR1/SHANK1/SHISA7/OXTR/SCN2A/SLC8A3/BRINP1/GRIN2A/HTR2A/HRH1/TAFA2/S100B/SLC8A2/MAPT/SLC2A4/RASGRF1/SYT4/SNAP25/ARC/LRRC66/RCAN1/JPH3/TUBA1A/PTN/PLCB1/CAMK4 39

BP GO:0061138 morphogenesis of a branching epithelium 52/2475 187/18870 6.80743297430963e-08 3.32291281119959e-06 2.41362386072441e-06 WNT4/MDK/HOXD11/SEMA3E/HOXA5/RSPO3/LEF1/NPNT/HS3ST3B1/SPRY2/HOXA11/NRP1/CITED1/ST14/CSMD1/BMP2/SOX8/AREG/SPINT1/ENG/FOXD1/TIMELESS/MET/GLI3/CLIC4/GREB1L/MMP14/VEGFA/SFRP2/HGF/FZD5/COL4A1/SOCS3/PDGFA/TACSTD2/TNC/SOX10/EPHA2/HS3ST3A1/SNAI2/RDH10/HOXB13/GDNF/CTSZ/CELSR1/SPRY1/EYA1/NOG/ADM/HOXD13/DLL4/HOXB7 52

BP GO:0050868 negative regulation of T cell activation 41/2475 133/18870 7.60334699736091e-08 3.68149156227056e-06 2.67408035739102e-06 ASCL2/MDK/PLA2G2A/LOXL3/LRRC32/ANXA1/PAWR/FOXJ1/CD74/SOCS1/HAVCR2/TWSG1/LGALS3/RUNX1/PLA2G5/CD274/GLI3/GPNMB/HLA-DRB1/JAK3/IDO1/PDCD1LG2/FCGR2B/VSIG4/BTN2A2/RUNX3/SPN/CEBPB/LILRB2/CD300A/LAPTM5/LILRB1/SDC4/CASP3/FGL2/IL2RA/ZC3H12A/TNFRSF14/IRF1/HFE/IL4I1 41

BP GO:0051932 synaptic transmission, GABAergic 24/2475 58/18870 8.53666057133479e-08 4.10032880562353e-06 2.97830608396127e-06 CA2/GABBR2/GABBR1/GABRA3/GABRA5/STXBP1/GABRD/GABRA4/CNR1/GABRB3/OXTR/PHF24/NALCN/ZDHHC12/GABRA1/GABRB2/GABRG2/SYN3/GABRG1/ADORA1/PRKCE/CA7/EZH2/GABRE 24

BP GO:0048705 skeletal system morphogenesis 60/2475 230/18870 8.68010926162156e-08 4.13614095291872e-06 3.00431851887202e-06 HOXD3/SHOX2/HOXB2/HOXA5/HOXB3/STC1/RIPPLY2/HOXA3/HOXC11/MGP/HOXA11/SP5/HOXA2/SERPINH1/MMP13/MMP2/SFRP4/NKX3-2/COL3A1/HOXD9/HOXB4/DLX2/HOXD4/CHAD/DLX5/DSCAML1/GLI3/HOXA9/MMP14/HOXC9/SMPD3/HOXC4/GSC/HAS2/COL1A1/ACP5/RUNX2/PAPPA2/OSR1/IRX5/HOXD10/TBX15/LTF/TWIST1/HOXA7/HOXD8/HOXB8/FOSL2/TBX1/COL27A1/RDH10/WNT10B/HOXC8/OSR2/EYA1/HOXA1/NOG/COL2A1/NODAL/HOXB7 60

BP GO:0007214 gamma-aminobutyric acid signaling pathway 16/2475 29/18870 9.13986076252698e-08 4.32092315104032e-06 3.13853652210106e-06 HTR1A/GABBR2/GABBR1/GABRA3/GABRA5/GABRD/GABRA4/GABRB3/SHISA7/GPR156/PHF24/GABRA1/GABRB2/GABRG2/GABRG1/GABRE 16

BP GO:0032760 positive regulation of tumor necrosis factor production 35/2475 106/18870 1.02204846807915e-07 4.79404609558377e-06 3.48219309477954e-06 LY96/PYCARD/TLR1/IL1A/CYBA/OAS2/ABCC8/THBS1/HAVCR2/HSPB1/LILRA5/ARID5A/CLEC7A/OAS1/SPON2/TLR2/SASH3/IFIH1/FZD5/OAS3/MIR27B/LRRK2/SPN/CD14/CD36/TYROBP/TWIST1/LILRA2/CD2/PTPRC/IL6/LBP/FCGR3A/FADD/MYD88 35

BP GO:0051250 negative regulation of lymphocyte activation 48/2475 169/18870 1.06101191996316e-07 4.93822912206109e-06 3.58692157030385e-06 ASCL2/MDK/PLA2G2A/LOXL3/LRRC32/ANXA1/PAWR/FOXJ1/CD74/SOCS1/HAVCR2/TWSG1/LGALS3/RUNX1/MICA/PLA2G5/CD274/GLI3/GPNMB/HLA-DRB1/TNFAIP3/SAMSN1/JAK3/IDO1/PDCD1LG2/FCGR2B/VSIG4/LYN/BTN2A2/RUNX3/SPN/CEBPB/LILRB2/CD300A/LAPTM5/TYROBP/LILRB1/SDC4/CASP3/FGL2/IL2RA/ZC3H12A/TNFRSF14/IRF1/GAL/HFE/IL4I1/HLA-F 48

BP GO:0071222 cellular response to lipopolysaccharide 59/2475 226/18870 1.07802059732639e-07 4.97879666642125e-06 3.61638812528196e-06 MAP2K3/LY96/GBP5/PYCARD/CXCL13/NFKBIZ/SPI1/IL1A/TSPO/CXCL8/GBP2/LITAF/CD40/HAVCR2/PPBP/SBNO2/ARID5A/CCL2/CMPK2/MMP9/CD180/CASP7/SPON2/KLRC4-KLRK1/CD274/CASP1/TNFAIP3/CXCL10/TLR2/ZFP36/CARD16/PDCD1LG2/SCIMP/LYN/GBP3/CSF3/SERPINE1/CD14/LTF/LILRB2/CXCL9/NOS2/CD36/CCR5/LILRB1/LILRA2/TRIM5/ADAM9/VIM/CDK4/HMGB2/CXCL6/PRKCE/CXCL11/IL6/LBP/ZC3H12A/IRAK2/MYD88 59

BP GO:0007229 integrin-mediated signaling pathway 36/2475 111/18870 1.12779683929356e-07 5.1689253611592e-06 3.75448959838749e-06 TIMP1/ITGA5/FERMT1/ITGB3/EMP2/LOXL3/ITGB2/ZYX/ITGA2/NID1/ITGA3/NRP1/ITGA11/VAV3/COL3A1/FLNA/LAMA2/ITGB4/ITGA4/CD63/SLC2A10/PRAM1/BST1/LAMC1/FN1/ISG15/LAMB1/ADAM9/ITGA7/PLEK/ITGB1/ITGA1/ITGAL/FERMT3/PTN/ADAM11 36

BP GO:0006909 phagocytosis 61/2475 237/18870 1.14412577638815e-07 5.20403875866246e-06 3.77999449089004e-06 ITGB3/PYCARD/NCF4/SLC11A1/ITGB2/CEACAM4/CYBA/ITGA2/PRKCG/ANXA1/THBS1/TREM2/FCGR1A/VAV3/P2RY6/CCL2/CLEC7A/RAB27A/PLA2G5/CD93/SPON2/MET/RAB7B/NCF2/RAB34/CD300LF/SYT7/TLR2/PLSCR1/F2RL1/FCGR2B/SPHK1/IL2RG/XKR7/LYN/FPR2/TUB/CD302/CD14/CD36/C2/CD300A/TYROBP/FCER1G/ICAM5/C3/PTPRC/ITGB1/MSR1/ADORA1/COLEC11/PRKCE/LBP/XKR8/MARCO/PTX3/ITGAL/MYO1G/MYD88/IL2RB/COLEC12 61

BP GO:0070371 ERK1 and ERK2 cascade 79/2475 336/18870 1.16109304472988e-07 5.24150574478058e-06 3.80720893099792e-06 HAND2/ITGB3/PYCARD/PLA2G2A/NECAB2/IL1A/MIR221/CCL26/NPNT/DUSP5/DUSP6/CCL20/SPRY2/TREM2/ICAM1/NRP1/SHC1/CD74/HAVCR2/NOX4/DUSP4/EGFR/CD44/DUSP26/CCN1/GBP1/P2RY6/CCL2/BMP2/FBLN1/DUSP9/FAM83D/PLA2G5/OXTR/WNK2/PTPRR/PTGER4/ACTA2/GPNMB/HLA-DRB1/ANGPT1/C5AR1/F2RL1/HTR2A/CCL5/SCIMP/CHI3L1/DUSP10/FN1/LYN/HTR2C/FPR2/MIR27B/BTN2A2/PDGFA/LIF/CCL18/CD36/EMILIN1/RAP1B/P2RY1/PDGFD/SPRED3/RRAS/EPHA2/IQGAP3/GATA4/PTPRC/CAVIN3/CDK1/NTRK1/MARCO/SPRY1/RAMP3/CCR1/GPR183/NODAL/MT3/ATF3 79

BP GO:0048525 negative regulation of viral process 32/2475 93/18870 1.21083363271897e-07 5.42525756033187e-06 3.94067850774132e-06 APOBEC3B/OASL/OAS2/FAM111A/APOBEC3G/TRIM21/MX1/APOBEC3C/STAT1/OAS1/CIITA/IFITM3/ZFP36/PLSCR1/IFI16/CCL5/APOBEC3F/IFITM2/IFIH1/OAS3/SLPI/LTF/PPIA/ISG15/TRIM6/TRIM5/RSAD2/BTBD17/PTX3/ISG20/APOBEC3H/BST2 32

BP GO:0002699 positive regulation of immune effector process 67/2475 270/18870 1.25607770902269e-07 5.58628930738684e-06 4.05764518400078e-06 KLRC2/PYCARD/IL18/MR1/KLRC3/NFKBIZ/SPI1/ITGB2/TNFRSF4/ANXA1/KLK7/TREM2/CD40/FCGR1A/TFRC/HPX/VAMP8/CD74/KLRC4/STXBP1/IL13RA2/HLA-C/IL1R1/MICA/ADGRE2/ARID5A/CLEC7A/PLA2G5/SPON2/KLRC4-KLRK1/HLA-DRB1/MICB/F2RL1/SCIMP/SASH3/FZD5/FES/LYN/HLA-DRA/NOS2/CD36/CD300A/NECTIN2/LAPTM5/TYROBP/HLA-B/LILRB1/ZP3/RAC2/TRIM6/HLA-A/RSAD2/ULBP2/ULBP3/C3/HLA-DMB/PTPRC/COLEC11/B2M/IL6/LBP/FADD/TNFRSF14/HFE/MYD88/CLCF1/HLA-F 67

BP GO:0036293 response to decreased oxygen levels 75/2475 315/18870 1.40900674696376e-07 6.22035037409588e-06 4.51820042776381e-06 CAV1/MDM2/ASCL2/LOXL2/IL1A/PLK3/STC1/LMNA/PLAT/CA9/ITGA2/PLOD1/ACE/SOD2/HMOX1/THBS1/TREM2/TFRC/CHRNB2/MMP2/MIR210/KCNMB1/BMP2/HSPG2/AK4/OXTR/ANGPT2/SCN2A/NGB/ANGPTL4/MMP14/VEGFA/TLR2/SLC8A3/MDM4/RYR2/CXCR4/HILPDA/CHCHD2/AJUBA/KCNK3/HK2/SLC2A4/SOD3/LIF/PLAU/KCNJ11/PDLIM1/ANG/NOS2/CCNA2/TWIST1/PDK1/CASP3/BRIP1/ACAA2/PGK1/VASN/POSTN/FOSL2/AGTRAP/ADORA1/DPP4/PRKCE/CHRNA4/PPARG/STOX1/PLOD2/TGFB2/AQP1/SLC29A1/ADM/UCP2/MGARP/MT3 75

BP GO:0007160 cell-matrix adhesion 61/2475 239/18870 1.57772373900899e-07 6.91434549562774e-06 5.02228924372974e-06 HOXD3/ITGA5/WNT4/FERMT1/EFEMP2/ITGB3/EMP2/SEMA3E/ITGB2/SIGLEC1/NPNT/ZYX/ITGA2/ECM2/NID1/COL5A3/MMP12/THBS1/ITGA3/NRP1/ITGA11/COL3A1/CD44/NID2/S100A10/ITGB4/JAG1/TRIP6/ITGA4/CD3E/MMP14/CD63/VEGFA/CAMSAP3/L1CAM/BST1/FN1/AJUBA/MSLN/SERPINE1/PLAU/ACTN1/CD36/EMILIN1/PPFIA2/SDC4/FBLN5/ADAM9/HOXA7/RRAS/POSTN/ITGA7/ITGB1/AJAP1/EPHA3/PARVG/ITGA1/RHOD/ITGAL/FERMT3/CDK6 61

BP GO:0070269 pyroptosis 18/2475 37/18870 1.82312271609064e-07 7.87831765486919e-06 5.72247800486045e-06 GBP5/PYCARD/GSDMD/GBP2/TREM2/GSDMA/TRIM21/GZMB/GBP1/MAP3K20/CASP1/CASP8/GZMA/CASP6/GBP3/CASP3/IFI27/CASP4 18

BP GO:0042129 regulation of T cell proliferation 50/2475 182/18870 1.82392763828584e-07 7.87831765486919e-06 5.72247800486045e-06 CD276/EFNB1/PYCARD/IL18/PLA2G2A/IL1A/LRRC32/ANXA1/CD70/TFRC/PAWR/FOXJ1/HAVCR2/TWSG1/RIPK3/LGALS3/TNFSF8/HLA-DPA1/PLA2G5/CD274/GPNMB/HLA-DRB1/CD3E/IDO1/PDCD1LG2/CCL5/IGF2/VSIG4/SASH3/BTN2A2/SPN/CEBPB/HLA-DPB1/LILRB2/IGFBP2/LILRB1/ZP3/RAC2/SDC4/CASP3/HLA-A/HLA-DMB/PTPRC/IL6/LMO1/IL2RA/FADD/TNFRSF14/IRF1/IL4I1 50

BP GO:0001913 T cell mediated cytotoxicity 24/2475 60/18870 1.8409542006005e-07 7.89506358600384e-06 5.73464154367508e-06 IL7R/EMP2/MR1/CTSC/RIPK3/HLA-C/MICA/RAB27A/HLA-DRB1/MICB/FCGR2B/HLA-DRA/NECTIN2/HLA-B/LILRB1/HLA-A/PRF1/ULBP2/ULBP3/PTPRC/B2M/FADD/HFE/HLA-F 24

BP GO:0002822 regulation of adaptive immune response based on somatic recombination of immune receptors built from immunoglobulin superfamily domains 52/2475 193/18870 2.05723664077661e-07 8.7600346037041e-06 6.36292004683651e-06 ASCL2/IL7R/BATF/IL18/MR1/SLC11A1/NFKBIZ/LOXL3/ANXA1/TREM2/CD40/FCGR1A/TFRC/HPX/FOXJ1/HAVCR2/RIPK3/AHR/HLA-C/IL1R1/MICA/ARID5A/CLEC7A/CD274/HLA-DRB1/TNFAIP3/MICB/JAK3/FCGR2B/SASH3/FZD5/SPN/HLA-DRA/NECTIN2/HLA-B/LILRB1/ZP3/HLA-A/RSAD2/ULBP2/ULBP3/C3/PTPRC/B2M/IL6/PLA2G4A/ZC3H12A/FADD/HFE/IL4I1/CLCF1/HLA-F 52

BP GO:0140014 mitotic nuclear division 67/2475 274/18870 2.26473403272462e-07 9.5756782623089e-06 6.95536923467614e-06 CENPI/NEK2/CENPF/SPC24/CUL7/KIFC1/MAD2L1/CDCA2/KIF14/IL1A/KIF18B/CDCA5/KNTC1/KIF4A/MYBL2/PSRC1/CHEK2/KIF23/UBE2C/FLNA/UBE2S/CCDC8/CCNB1/TOM1L1/ANKRD53/DLGAP5/BIRC5/SMPD3/DSN1/HASPIN/ESPL1/CAV2/PKMYT1/SPHK1/IGF2/TTK/NPM2/ZWILCH/MKI67/NUF2/AURKA/TRIP13/BUB1B/KIF18A/CDC25C/BUB1/AURKB/KIF2C/NCAPG/SMC4/CDT1/REEP4/NDE1/PLK1/CENPE/CDCA8/NDC80/CDK1/NCAPH/TPX2/KNL1/ZWINT/CHMP4BP1/CENPK/SPAG5/CHEK1/CDC20 67

BP GO:0019730 antimicrobial humoral response 40/2475 133/18870 2.28216873578166e-07 9.58191684589726e-06 6.95990067894252e-06 CXCL14/RARRES2/SLC11A1/CXCL13/IGKV3-20/PI3/CXCL8/IGHG4/KLK7/WFDC2/BCL3/S100A12/H2BC8/IGHA1/PPBP/SPON2/S100A9/IGHG2/CXCL10/IGHG3/H2BC11/IGHA2/JCHAIN/LYZ/H2BC12/SLPI/ANG/LTF/CXCL9/IGHG1/RNASE3/HLA-A/IGHM/H2BC4/RNASE6/COLEC11/CXCL6/CXCL11/GNLY/NCR3LG1 40

BP GO:0050432 catecholamine secretion 24/2475 61/18870 2.65924611527524e-07 1.10450185752253e-05 8.0226361298009e-06 STX1A/CHGA/GABBR1/CHRNB2/KCNB1/CRH/CNR1/PRKCB/OXTR/SNCG/SYT7/HTR2A/VIP/SYT1/SYT4/GRM2/P2RY1/HRH3/SYT5/CHRNA4/GDNF/SYT2/SYT13/ADRA2B 24

BP GO:0033628 regulation of cell adhesion mediated by integrin 21/2475 49/18870 2.66743453265767e-07 1.10450185752253e-05 8.0226361298009e-06 FERMT1/ITGB3/CXCL13/RET/CYP1B1/CD3E/CCL5/SFRP2/MUC1/LYN/SERPINE1/LIF/PLAU/ADAM9/EPHA2/SNAI2/DPP4/PIK3CG/TGFB2/SWAP70/FERMT3 21

BP GO:0001910 regulation of leukocyte mediated cytotoxicity 32/2475 96/18870 2.78373722326973e-07 1.1447642663364e-05 8.31508530353748e-06 KLRC2/IL7R/MR1/KLRC3/SPI1/ICAM1/KLRC4/HAVCR2/RIPK3/HLA-C/MICA/KLRC4-KLRK1/HLA-DRB1/MICB/F2RL1/FCGR2B/HLA-DRA/NOS2/NECTIN2/TYROBP/HLA-B/LILRB1/HLA-A/ULBP2/ULBP3/PTPRC/CXCL6/B2M/PIK3R6/FADD/HFE/HLA-F 32

BP GO:1903557 positive regulation of tumor necrosis factor superfamily cytokine production 35/2475 110/18870 2.84518639718357e-07 1.16028915795257e-05 8.4278515750859e-06 LY96/PYCARD/TLR1/IL1A/CYBA/OAS2/ABCC8/THBS1/HAVCR2/HSPB1/LILRA5/ARID5A/CLEC7A/OAS1/SPON2/TLR2/SASH3/IFIH1/FZD5/OAS3/MIR27B/LRRK2/SPN/CD14/CD36/TYROBP/TWIST1/LILRA2/CD2/PTPRC/IL6/LBP/FCGR3A/FADD/MYD88 35

BP GO:0070482 response to oxygen levels 79/2475 343/18870 2.87352545192709e-07 1.16028915795257e-05 8.4278515750859e-06 CAV1/MDM2/ASCL2/LOXL2/IL1A/PLK3/STC1/LMNA/PLAT/CA9/ITGA2/PLOD1/FAS/ACE/SOD2/HMOX1/THBS1/TREM2/TFRC/CHRNB2/MMP2/MIR210/KCNMB1/BMP2/HSPG2/AK4/OXTR/ANGPT2/SCN2A/NGB/ANGPTL4/MMP14/VEGFA/TLR2/SLC8A3/MDM4/PDPN/RYR2/CXCR4/HILPDA/MYOD1/COL1A1/CHCHD2/AJUBA/KCNK3/HK2/SLC2A4/SOD3/LIF/PLAU/KCNJ11/PDLIM1/ANG/NOS2/CCNA2/TWIST1/PDK1/CASP3/BRIP1/ACAA2/PGK1/VASN/POSTN/FOSL2/AGTRAP/ADORA1/DPP4/PRKCE/CHRNA4/PPARG/STOX1/PLOD2/TGFB2/AQP1/SLC29A1/ADM/UCP2/MGARP/MT3 79

BP GO:0044706 multi-multicellular organism process 56/2475 216/18870 2.87946509884965e-07 1.16028915795257e-05 8.4278515750859e-06 VMP1/TIMP1/THBD/ITGA5/WNT4/ITGB3/EMP2/FOSL1/TEAD4/ARHGDIB/STC1/ITGA2/SPP1/ACE/ABCC8/ITGA3/TEKT3/ACVR1C/MMP2/CRH/HAVCR2/IGFBP5/CSMD1/TEAD3/CNR1/HSPG2/MMP9/ITGB4/FBLN1/DSG2/OXTR/ANGPT2/IGFBP7/HEXB/VEGFA/IDO1/CTSB/SYDE1/PRLHR/GJB2/PCSK5/CYP27B1/LIF/SMCP/IGFBP2/H3-3A/NR2F2/RXFP1/PTHLH/TPPP3/HFE/ADM/PTN/UCP2/ADRA2B/NODAL 56

BP GO:0010721 negative regulation of cell development 69/2475 287/18870 2.98456202526374e-07 1.18893804574318e-05 8.6359450252709e-06 LRRC17/ASCL2/MCF2/MDK/SEMA3E/IL1A/MIR221/TSPO/LOXL3/TTPA/SPP1/ANXA1/FGF13/ABCC8/TREM2/DLL3/NRP1/FOXJ1/SEMA3A/LILRB3/CD74/SOCS1/SEMA3D/SLIT1/RUNX1/DLX2/RTN4R/NR2E1/S1PR3/GLI3/JAK3/VEGFA/BRINP1/FRZB/FCGR2B/SEMA6B/DUSP10/LYN/FSTL3/SYT4/RUNX3/LTF/SOX10/TNR/TNFRSF11B/LILRB1/TNFAIP6/SEMA3F/NKX6-1/TMEM176A/DRAXIN/FGL2/FSTL4/HOXA7/SNAI2/ERFE/DAAM2/PTHLH/B2M/IL6/C1QC/ZC3H12A/IRF1/NOG/TMEM176B/PTN/CDK6/NODAL/MT3 69

BP GO:1905818 regulation of chromosome separation 27/2475 74/18870 2.99016730358463e-07 1.18893804574318e-05 8.6359450252709e-06 CENPF/SPC24/MAD2L1/KNTC1/UBE2C/CCNB1/DLGAP5/BIRC5/PLSCR1/HASPIN/ESPL1/TTK/ZWILCH/NUF2/TRIP13/BUB1B/BUB1/AURKB/NCAPG/SMC4/PLK1/CDCA8/NDC80/NCAPH/KNL1/ZWINT/CDC20 27

BP GO:0019058 viral life cycle 75/2475 321/18870 3.14042186039671e-07 1.23235900979228e-05 8.95133661343211e-06 APOBEC3B/CAV1/OASL/ITGA5/ITGB3/MIR221/TNFRSF4/SIGLEC1/TOP2A/CXCL8/OAS2/KPNA2/ITGA2/FAM111A/TFRC/CLDN1/ICAM1/NRP1/APOBEC3G/VAMP8/CD74/TRIM21/EGFR/MX1/CCL2/APOBEC3C/TRIM38/TRIM22/OAS1/CIITA/IFITM3/HLA-DRB1/PLSCR1/IFI16/CTSB/HTR2A/CAV2/CCL5/APOBEC3F/CXCR4/CLEC5A/IFITM2/IFIH1/CTSL/F11R/OAS3/PCSK5/SLPI/LTF/PPIA/ANPEP/LAMP3/ISG15/NECTIN2/CCR5/LGALS1/TRIM6/TRIM5/RSAD2/EPHA2/EFNB2/BTBD17/ITGB1/IFI27/DPP4/CDK1/JPT2/PTX3/ZC3H12A/CHMP4BP1/ISG20/TNFRSF14/APOBEC3H/SLC1A5/BST2 75

BP GO:1902105 regulation of leukocyte differentiation 75/2475 321/18870 3.14042186039671e-07 1.23235900979228e-05 8.95133661343211e-06 LRRC17/ASCL2/IL7R/MDK/BATF/IRF7/IL18/LEF1/NFKBIZ/SPI1/LOXL3/FANCD2/ROR2/ANXA1/ACTL6B/TESPA1/TREM2/FOXJ1/LILRB3/CD74/SOCS1/RHOH/RUNX1/HLA-DOA/NFAM1/GLI3/SLAMF8/TOX/HLA-DRB1/MMP14/CASP8/JAK3/FCGR2B/CD101/IL2RG/SASH3/DUSP10/FES/LYN/FSTL3/BTN2A2/RUNX3/LIF/CEBPB/LTF/LILRB2/HLA-DRA/TYROBP/HLA-B/EVI2B/TNFRSF11B/LILRB1/TNFAIP6/TMEM176A/TCF7/FGL2/HOXA7/CD2/POU4F1/ERFE/PTPRC/WNT10B/C1QC/IL2RA/ZC3H12A/PIK3R6/FADD/IRF1/HCLS1/IL4I1/TMEM176B/CCR1/CDK6/CAMK4/FANCA 75

BP GO:0045088 regulation of innate immune response 93/2475 425/18870 3.24509935161882e-07 1.2651673056571e-05 9.18964225137168e-06 CAV1/KLRC2/OASL/LY96/GBP5/IRF7/PYCARD/TLR1/KLRC3/NFKBIZ/SPI1/FOSL1/NCF1/PARP9/RBM47/CYBA/GBP2/MMP12/TREM2/CGAS/CD40/HPX/TRIM21/LILRA4/KLRC4/HAVCR2/MICA/CLEC7A/TIFA/OAS1/BIRC3/PLA2G5/KLRC4-KLRK1/RAB7B/CASP1/SLAMF8/TNFAIP3/CASP8/CD300LF/TLR2/PLSCR1/IFI16/F2RL1/NMI/FCGR2B/CCL5/SCIMP/VSIG4/IFIH1/TLR8/DUSP10/CASP6/LYN/FPR2/OAS3/NLRC5/SERPING1/CD14/LTF/ZDHHC12/SLC15A3/PAK3/CD36/ISG15/CD300A/NECTIN2/TYROBP/HLA-B/LILRB1/LILRA2/AURKB/IFI35/TRIM6/TRIM5/HLA-A/RSAD2/ALPK1/UNC93B1/RNF135/HMGB2/COLEC11/PRKCE/PPARG/LBP/PIK3R6/FADD/IRAK2/CFH/IRF1/MYD88/HLA-F/CTSS/COLEC12 93

BP GO:0017156 calcium-ion regulated exocytosis 25/2475 66/18870 3.56400198903024e-07 1.3768877932894e-05 1.00011328019881e-05 CDK5R2/UNC13A/STX1A/STX1B/RIMS1/KCNB1/DOC2A/STXBP1/RAB3A/UNC13C/RAPGEF4/SCIN/SYN2/SYT7/SCAMP5/SYT1/SYT4/SNAP25/RAP1B/ZP3/SYT5/SYT2/RIMS3/SYT13/RIMS2 25

BP GO:0007612 learning 43/2475 150/18870 3.6212983189816e-07 1.3768877932894e-05 1.00011328019881e-05 NPTX2/FOSL1/JPH4/ATP8A1/FGF13/ABCC8/ADGRB3/PAK5/CHRNB2/GABRA5/CSMD1/NEUROD2/ASIC1/SHANK2/SHANK1/CLSTN2/NRXN1/TLR2/SLC8A3/GRIN2A/DKK1/HRH1/TAFA2/GRM5/SLC8A2/SLC12A5/GRIN1/SNAP25/ARC/TNR/NRXN3/STRA6/CNTNAP2/ELAVL4/TBR1/JPH3/ITGB1/MAPK8IP2/TUBA1A/NOG/PTN/PLCB1/EN1 43

BP GO:0045667 regulation of osteoblast differentiation 43/2475 150/18870 3.6212983189816e-07 1.3768877932894e-05 1.00011328019881e-05 WNT4/HAND2/WWTR1/NPNT/GDPD2/HOXA2/DNAI3/SMOC1/MIR210/CITED1/TWSG1/IGFBP5/CCN1/BMP2/AREG/CCN4/JAG1/GLI3/NELL1/SFRP2/HGF/FAM20C/RUNX2/CEBPD/TENT5A/CEBPB/CTHRC1/LTF/TWIST1/GLI1/TNFAIP6/TWIST2/GDF10/SNAI2/ERFE/WNT10B/IL6/PPARG/CLIC1/TCIRG1/NOG/WNT7B/CDK6 43

BP GO:0031341 regulation of cell killing 35/2475 111/18870 3.63608948305402e-07 1.3768877932894e-05 1.00011328019881e-05 KLRC2/IL7R/MR1/KLRC3/SPI1/ICAM1/KLRC4/HAVCR2/RIPK3/HLA-C/MICA/CLEC7A/KLRC4-KLRK1/HLA-DRB1/MICB/F2RL1/FCGR2B/HLA-DRA/NOS2/NECTIN2/TYROBP/HLA-B/LILRB1/HLA-A/PRF1/ULBP2/ULBP3/PTPRC/CXCL6/B2M/PIK3R6/FADD/CFH/HFE/HLA-F 35

BP GO:0051966 regulation of synaptic transmission, glutamatergic 28/2475 79/18870 3.64632177103623e-07 1.3768877932894e-05 1.00011328019881e-05 UNC13A/CACNG2/ROR2/DGKI/STXBP1/CNR1/CCL2/OXTR/OPHN1/NRXN1/GRIN2A/HTR2A/DKK1/GRM5/SYT1/FRRS1L/GRIN1/LRRK2/GRM2/TNR/GRIK1/GRM1/ADORA1/MAPK8IP2/NTRK1/GRIN2B/CACNG5/CACNG3 28

BP GO:0007094 mitotic spindle assembly checkpoint signaling 20/2475 46/18870 3.85982707832028e-07 1.43051862828611e-05 1.03906845909558e-05 CENPF/SPC24/MAD2L1/KNTC1/CCNB1/BIRC5/HASPIN/TTK/ZWILCH/NUF2/TRIP13/BUB1B/BUB1/AURKB/PLK1/CDCA8/NDC80/KNL1/ZWINT/CDC20 20

BP GO:0071173 spindle assembly checkpoint signaling 20/2475 46/18870 3.85982707832028e-07 1.43051862828611e-05 1.03906845909558e-05 CENPF/SPC24/MAD2L1/KNTC1/CCNB1/BIRC5/HASPIN/TTK/ZWILCH/NUF2/TRIP13/BUB1B/BUB1/AURKB/PLK1/CDCA8/NDC80/KNL1/ZWINT/CDC20 20

BP GO:0071174 mitotic spindle checkpoint signaling 20/2475 46/18870 3.85982707832028e-07 1.43051862828611e-05 1.03906845909558e-05 CENPF/SPC24/MAD2L1/KNTC1/CCNB1/BIRC5/HASPIN/TTK/ZWILCH/NUF2/TRIP13/BUB1B/BUB1/AURKB/PLK1/CDCA8/NDC80/KNL1/ZWINT/CDC20 20

BP GO:0051607 defense response to virus 77/2475 334/18870 3.88733849107051e-07 1.4318760920483e-05 1.04005446357799e-05 APOBEC3B/HERC5/OASL/GBP5/IRF7/PYCARD/IFI6/PARP9/OAS2/GBP2/IFNGR2/MMP12/TREM2/CGAS/CD40/APOBEC3G/VAMP8/TRIM21/LILRA4/MX2/RIPK3/MX1/MICA/MLKL/GBP1/APOBEC3C/STAT1/TRIM38/TRIM22/OAS1/RNASE2/SPON2/IFITM3/RAB7B/CASP1/TNFAIP3/DDX60L/MICB/CXCL10/TLR2/PLSCR1/IFI16/F2RL1/APOBEC3F/IFITM2/IFIH1/TLR8/OAS3/GBP3/NLRC5/SPN/DTX3L/ZDHHC12/CXCL9/ISG15/LILRB1/TRIM6/TRIM5/RSAD2/FGL2/PRF1/RTP4/ZDHHC1/UNC93B1/RNF135/PTPRC/RNASE6/IFI27/IL6/ZC3H12A/FADD/ISG20/IRF1/MYD88/APOBEC3H/SLFN13/BST2 77

BP GO:0048562 embryonic organ morphogenesis 70/2475 295/18870 4.16446012021589e-07 1.52260913814572e-05 1.10595912537917e-05 STIL/HOXD3/SHOX2/CHRNA9/HAND2/HOXB2/HOXA5/NKX2-5/MFAP2/HOXB3/EFEMP1/USH1C/HOXA3/HOXC11/SPRY2/HOXA11/HOXA2/MFAP5/ATP8A2/NKX3-2/WNT16/APLNR/HOXD9/HOXB4/DLX2/HOXD4/TEAD2/DLX5/ENG/DSCAML1/GLI3/HOXA9/MMP14/HOXC9/HOXC4/GSC/FOLR1/FRZB/RYR2/FZD5/RUNX2/OSR1/CCDC40/IRX5/HOXD10/TBX15/CTHRC1/FZD2/TWIST1/GLI1/OTX1/CRB2/STRA6/HOXA7/EPHA2/HOXB8/GATA4/TBX1/ALDH1A3/ZIC1/RDH10/STOX1/OSR2/EYA1/HOXA1/NOG/COL2A1/FZD6/NODAL/HOXB7 70

BP GO:0002833 positive regulation of response to biotic stimulus 83/2475 369/18870 4.18438553954104e-07 1.52260913814572e-05 1.10595912537917e-05 CAV1/KLRC2/OASL/LY96/GBP5/IRF7/PYCARD/TLR1/MR1/KLRC3/NFKBIZ/SPI1/FOSL1/PARP9/RBM47/CYBA/GBP2/MMP12/KLK7/TREM2/CGAS/CD40/HPX/LILRA4/KLRC4/HAVCR2/CLEC7A/CD180/TIFA/OAS1/BIRC3/PLA2G5/KLRC4-KLRK1/CD274/RAB7B/CASP1/HLA-DRB1/TNFAIP3/CD300LF/TLR2/PLSCR1/IFI16/F2RL1/NMI/CCL5/SCIMP/IFIH1/TLR8/CASP6/LYN/EMILIN2/FPR2/OAS3/NLRC5/CD14/LTF/ZDHHC12/SLC15A3/PAK3/CD36/EMILIN1/CD300A/NECTIN2/TYROBP/LILRA2/IFI35/TRIM6/TRIM5/RSAD2/ALPK1/UNC93B1/RNF135/HMGB2/COLEC11/PRKCE/LBP/FADD/IRAK2/IRF1/MYD88/HLA-F/CTSS/COLEC12 83

BP GO:0030595 leukocyte chemotaxis 60/2475 240/18870 4.2753784610999e-07 1.54634772773758e-05 1.12320183667323e-05 RARRES2/MDK/CXCL13/SPI1/CCL26/ITGB2/CXCL8/CCL20/CHGA/ANXA1/THBS1/MMP2/VAV3/S100A12/CD74/PPBP/LGALS3/ADGRE2/CCL2/IL17RC/S100A8/KLRC4-KLRK1/S100A9/SLAMF8/HSD3B7/VEGFA/CXCL10/C5AR1/F2RL1/CCL5/CXCR4/DPEP1/BST1/LYN/FPR2/SERPINE1/CCL18/PPIA/CXCL9/CCR5/THBS4/TNFAIP6/RAC2/CXCR2/FCER1G/ALOX5/DPP4/CXCL6/CXCL11/IL6/PIK3CG/LBP/ITGA1/TGFB2/SWAP70/SAA1/TREM1/PTN/CCR1/GPR183 60

BP GO:0051983 regulation of chromosome segregation 39/2475 131/18870 4.3049250552374e-07 1.54771078033804e-05 1.12419190065229e-05 CENPF/SPC24/MAD2L1/CDCA2/BCL7A/ACTL6B/KNTC1/UBE2C/TACC3/MAP3K20/CCNB1/RMI2/NEK6/DLGAP5/BIRC5/PLSCR1/HASPIN/ESPL1/TTK/ZWILCH/MKI67/NUF2/TRIP13/BUB1B/BUB1/AURKB/KIF2C/NCAPG/SMC4/PLK1/CDC6/CENPE/CDCA8/NDC80/CDK1/NCAPH/KNL1/ZWINT/CDC20 39

BP GO:0140546 defense response to symbiont 77/2475 335/18870 4.41036310049675e-07 1.57617976520134e-05 1.14487057176429e-05 APOBEC3B/HERC5/OASL/GBP5/IRF7/PYCARD/IFI6/PARP9/OAS2/GBP2/IFNGR2/MMP12/TREM2/CGAS/CD40/APOBEC3G/VAMP8/TRIM21/LILRA4/MX2/RIPK3/MX1/MICA/MLKL/GBP1/APOBEC3C/STAT1/TRIM38/TRIM22/OAS1/RNASE2/SPON2/IFITM3/RAB7B/CASP1/TNFAIP3/DDX60L/MICB/CXCL10/TLR2/PLSCR1/IFI16/F2RL1/APOBEC3F/IFITM2/IFIH1/TLR8/OAS3/GBP3/NLRC5/SPN/DTX3L/ZDHHC12/CXCL9/ISG15/LILRB1/TRIM6/TRIM5/RSAD2/FGL2/PRF1/RTP4/ZDHHC1/UNC93B1/RNF135/PTPRC/RNASE6/IFI27/IL6/ZC3H12A/FADD/ISG20/IRF1/MYD88/APOBEC3H/SLFN13/BST2 77

BP GO:0002275 myeloid cell activation involved in immune response 32/2475 98/18870 4.72549771732692e-07 1.67880995827401e-05 1.21941681986206e-05 PYCARD/SPI1/ITGB2/CHGA/TREM2/CPLX2/VAMP8/STXBP1/HAVCR2/IL13RA2/SBNO2/S100A13/ADGRE2/PRAM1/F2RL1/NMI/SCNN1B/FES/LYN/CD300A/TYROBP/LILRA2/RAC2/IFI35/LAT2/MILR1/FCER1G/PRKCE/PIK3CG/LBP/MYD88/GRP 32

BP GO:0070372 regulation of ERK1 and ERK2 cascade 73/2475 313/18870 4.8321709225861e-07 1.70660907171806e-05 1.23960892459902e-05 HAND2/ITGB3/PYCARD/PLA2G2A/NECAB2/IL1A/MIR221/CCL26/NPNT/DUSP6/CCL20/SPRY2/TREM2/ICAM1/NRP1/SHC1/CD74/HAVCR2/NOX4/DUSP4/EGFR/CD44/DUSP26/CCN1/GBP1/P2RY6/CCL2/BMP2/FBLN1/FAM83D/PLA2G5/WNK2/PTPRR/ACTA2/GPNMB/HLA-DRB1/ANGPT1/C5AR1/F2RL1/HTR2A/CCL5/SCIMP/CHI3L1/DUSP10/FN1/LYN/HTR2C/FPR2/MIR27B/BTN2A2/PDGFA/LIF/CCL18/CD36/EMILIN1/RAP1B/P2RY1/PDGFD/SPRED3/RRAS/EPHA2/GATA4/PTPRC/CAVIN3/NTRK1/MARCO/SPRY1/RAMP3/CCR1/GPR183/NODAL/MT3/ATF3 73

BP GO:0000819 sister chromatid segregation 57/2475 225/18870 5.17356532588389e-07 1.81649626997701e-05 1.31942635550243e-05 CENPI/NEK2/CENPF/SPC24/KIFC1/MAD2L1/KIF14/BCL7A/TOP2A/KIF18B/CDCA5/ACTL6B/KNTC1/KIF4A/MYBL2/PSRC1/CHEK2/KIF23/UBE2C/FLNA/TACC3/MAP3K20/CCNB1/RMI2/NEK6/ANKRD53/DLGAP5/BIRC5/DSN1/HASPIN/ESPL1/TTK/ZWILCH/NUF2/TRIP13/BUB1B/KIF18A/BUB1/AURKB/KIF2C/NCAPG/SMC4/CDT1/PLK1/CDC6/CENPE/CDCA8/NDC80/CDK1/NCAPH/TPX2/KNL1/ZWINT/CHMP4BP1/CENPK/SPAG5/CDC20 57

BP GO:0010975 regulation of neuron projection development 97/2475 453/18870 5.22750468604338e-07 1.8247638450584e-05 1.32543157370121e-05 IL1RAPL1/MDM2/CUL7/SHOX2/MCF2/MDK/CDK5R1/SEMA3E/MIR221/DNM3/STX1B/ROR2/SPP1/KIF26A/FGF13/ADGRB3/ITGA3/NEFL/LRRC7/NTNG2/CHRNB2/RET/NRP1/SEMA3A/ATP8A2/MIR210/CDH4/SEMA3D/FLNA/AVIL/SLIT1/DSCAM/NEGR1/CUX2/CNR1/RTN4R/NR2E1/MBOAT1/ROR1/S100A9/BRSK2/TOX/FEZF2/VEGFA/DKK1/SFRP2/L1CAM/RTN4RL1/SEMA6B/PLK5/ABI3/PMP22/FES/HECW1/FN1/LYN/MAPT/CSMD3/LRRK2/SNAP25/CHODL/ARC/PAK3/SLITRK1/TNR/PPFIA2/ZNF804A/SEMA3F/ULK4/LZTS1/TRIM67/DRAXIN/VIM/FSTL4/PACSIN1/RAP2A/P3H1/EFNB2/ELAVL4/TBR1/STMN2/FZD1/EPHA3/TRPV2/B2M/NTRK1/ARHGAP44/EFHC2/SERPINI1/ISLR2/EZH2/NTNG1/NEU4/PTN/MGARP/MT3/FUT9 97

BP GO:0050729 positive regulation of inflammatory response 43/2475 152/18870 5.40033742431376e-07 1.87419802864623e-05 1.3613384818334e-05 PDE2A/TRADD/MDK/PYCARD/IL18/PLA2G2A/NFKBIZ/NKG7/GSDMD/NEAT1/TREM2/FCGR1A/S100A12/VAMP8/CTSC/LILRA5/CNR1/CCN4/S100A8/GPR4/S100A9/TNFRSF1A/TAFA3/PTGER4/CASP1/TLR2/IDO1/NMI/ALOX5AP/LRRK2/SERPINE1/CEBPB/MGST2/ZP3/LGALS1/IFI35/OSM/OSMR/C3/IL6/PIK3CG/LBP/CASP4 43

BP GO:1901342 regulation of vasculature development 80/2475 354/18870 5.51287266126443e-07 1.90225789989837e-05 1.38171999005557e-05 SP100/ITGA5/WNT4/MDK/ITGB3/EMP2/SEMA3E/CXCL13/HOXA5/IL1A/MIR221/CXCL8/SPRY2/HMOX1/ABCC8/THBS1/ADGRB3/CD40/ECSCR/NRP1/F3/MIR210/APLNR/HSPB1/RUNX1/CYP1B1/STAT1/HSPG2/NR2E1/PRKCB/ECM1/ENG/GPR4/ANGPT2/GPNMB/ANGPTL4/TNFAIP3/BRCA1/VEGFA/CXCL10/C5AR1/SFRP2/CXCR4/SPHK1/CHI3L1/STAB1/DCN/EMILIN2/HK2/MIR27B/HTATIP2/COL4A2/SERPINE1/EMILIN1/HSPB6/TWIST1/THBS4/RHOJ/E2F2/CXCR2/WARS1/PGK1/RRAS/EPHA2/ALOX5/GADD45A/GATA4/C3/ITGB1/CNMD/IL6/PPARG/PIK3CG/TGFB2/ZC3H12A/PIK3R6/ADAM12/AQP1/ADM/NODAL 80

BP GO:0050730 regulation of peptidyl-tyrosine phosphorylation 58/2475 231/18870 5.67512163780696e-07 1.9408483483362e-05 1.4097504658573e-05 CAV1/ITGA5/ITGB3/IL18/NCF1/MIR221/PARP9/ITGB2/ACE/TREM2/CD40/FCGR1A/HPX/NRP1/CD74/SOCS1/NOX4/EGFR/CD44/LILRA5/AREG/ERRFI1/TNFRSF1A/CTF1/CD3E/SAMSN1/ANGPT1/VEGFA/HTR2A/CCL5/GFRA1/EHD4/SFRP2/IGF2/IL11/GRM5/BST1/TNFRSF18/HGF/LYN/SOCS3/TNK2/CSF3/PDGFA/LIF/GPRC5A/CD36/CD300A/THBS4/PDGFD/OSM/IL9/PTPRC/PRKCE/IL6/TNFRSF14/HCLS1/CLCF1 58

BP GO:0010965 regulation of mitotic sister chromatid separation 23/2475 59/18870 5.71198889239064e-07 1.9408483483362e-05 1.4097504658573e-05 CENPF/SPC24/MAD2L1/KNTC1/UBE2C/CCNB1/DLGAP5/BIRC5/HASPIN/ESPL1/TTK/ZWILCH/NUF2/TRIP13/BUB1B/BUB1/AURKB/PLK1/CDCA8/NDC80/KNL1/ZWINT/CDC20 23

BP GO:0045069 regulation of viral genome replication 29/2475 85/18870 5.72168816881257e-07 1.9408483483362e-05 1.4097504658573e-05 APOBEC3B/OASL/TOP2A/CXCL8/OAS2/FAM111A/APOBEC3G/MX1/APOBEC3C/TRIM38/OAS1/IFITM3/PLSCR1/IFI16/CCL5/APOBEC3F/IFITM2/IFIH1/OAS3/SLPI/LTF/PPIA/ISG15/TRIM6/RSAD2/BTBD17/ISG20/APOBEC3H/BST2 29

BP GO:0031577 spindle checkpoint signaling 20/2475 47/18870 5.89262716508718e-07 1.9876030055721e-05 1.44371107894478e-05 CENPF/SPC24/MAD2L1/KNTC1/CCNB1/BIRC5/HASPIN/TTK/ZWILCH/NUF2/TRIP13/BUB1B/BUB1/AURKB/PLK1/CDCA8/NDC80/KNL1/ZWINT/CDC20 20

BP GO:0045765 regulation of angiogenesis 79/2475 349/18870 6.04002808355542e-07 2.0259401460149e-05 1.47155756249163e-05 SP100/ITGA5/MDK/ITGB3/EMP2/SEMA3E/CXCL13/HOXA5/IL1A/MIR221/CXCL8/SPRY2/HMOX1/ABCC8/THBS1/ADGRB3/CD40/ECSCR/NRP1/F3/MIR210/APLNR/HSPB1/RUNX1/CYP1B1/STAT1/HSPG2/NR2E1/PRKCB/ECM1/ENG/GPR4/ANGPT2/GPNMB/ANGPTL4/TNFAIP3/BRCA1/VEGFA/CXCL10/C5AR1/SFRP2/CXCR4/SPHK1/CHI3L1/STAB1/DCN/EMILIN2/HK2/MIR27B/HTATIP2/COL4A2/SERPINE1/EMILIN1/HSPB6/TWIST1/THBS4/RHOJ/E2F2/CXCR2/WARS1/PGK1/RRAS/EPHA2/ALOX5/GADD45A/GATA4/C3/ITGB1/CNMD/IL6/PPARG/PIK3CG/TGFB2/ZC3H12A/PIK3R6/ADAM12/AQP1/ADM/NODAL 79

BP GO:0034612 response to tumor necrosis factor 63/2475 259/18870 6.25264911708517e-07 2.0856058499433e-05 1.51489621591134e-05 TRADD/PYCARD/TMSB4X/CCL26/TNFRSF4/TRAF5/NPNT/CTSK/CXCL8/CCL20/FAS/GBP2/PYDC1/THBS1/TNFRSF19/CD40/CD70/CLDN1/GCH1/CYP1B1/GBP1/CCL2/STAT1/BIRC3/TNFRSF1A/CASP1/TNFAIP3/CASP8/BRCA1/SMPD3/ZFP36/CARD16/F2RL1/CD58/CCL5/PLVAP/KRT18/SPHK1/CHI3L1/HAS2/MYOD1/H2BC11/TNFRSF18/COL1A1/PID1/SLC2A4/MIR27B/GBP3/LRRK2/YBX3/CCL18/CD14/LAPTM5/CASP3/ADAM9/POSTN/TUBA1A/ADAMTS7/ZC3H12A/CASP4/TNFRSF14/GPD1/TRAF1 63

BP GO:0044409 entry into host 45/2475 163/18870 6.32769625150713e-07 2.09897725381485e-05 1.52460864030207e-05 CAV1/ITGA5/ITGB3/TNFRSF4/SIGLEC1/CXCL8/ITGA2/FUCA2/TFRC/CLDN1/ICAM1/NRP1/VAMP8/CD74/TRIM21/EGFR/TRIM38/TRIM22/CIITA/IFITM3/HLA-DRB1/PLSCR1/CTSB/HTR2A/CAV2/CXCR4/CLEC5A/IFITM2/CTSL/F11R/ANPEP/NECTIN2/CCR5/LGALS1/TRIM6/TRIM5/EPHA2/EFNB2/ITGB1/DPP4/CDK1/JPT2/PTX3/TNFRSF14/SLC1A5 45

BP GO:0001704 formation of primary germ layer 38/2475 128/18870 6.51121085645293e-07 2.14798406495293e-05 1.56020512309338e-05 COL8A1/ITGA5/ITGB3/SNAI1/LEF1/ITGB2/DUSP5/ITGA2/HOXA11/ITGA3/FZD7/MMP2/DUSP4/TWSG1/COL12A1/MMP9/ITGB4/ITGA4/ATOH8/MMP14/DKK1/SFRP2/FN1/ETV2/EYA2/COL4A2/COL6A1/LAMB1/CRB2/EPHA2/COL5A2/ITGA7/ITGB1/COL5A1/TBX19/EYA1/NOG/NODAL 38

BP GO:0030168 platelet activation 39/2475 133/18870 6.61591056151898e-07 2.17057511704398e-05 1.57661431149641e-05 THBD/MMRN1/ITGB3/LCK/VWF/DGKI/C1QTNF1/CD40/VAV3/COL3A1/STXBP1/FLNA/HSPB1/ENTPD2/PLSCR1/PDPN/LYN/EMILIN2/F11R/MYL9/PDGFA/PPIA/EMILIN1/F2RL3/P2RY1/F2RL2/DGKB/MYL12A/FCER1G/PLEK/DGKK/IL6/PIK3CG/PLA2G4A/CLIC1/SAA1/FERMT3/ADRA2B/FZD6 39

BP GO:0000070 mitotic sister chromatid segregation 49/2475 184/18870 6.65199569513812e-07 2.17057511704398e-05 1.57661431149641e-05 CENPI/NEK2/CENPF/SPC24/KIFC1/MAD2L1/KIF14/KIF18B/CDCA5/KNTC1/KIF4A/MYBL2/PSRC1/CHEK2/KIF23/UBE2C/FLNA/CCNB1/ANKRD53/DLGAP5/BIRC5/DSN1/HASPIN/ESPL1/TTK/ZWILCH/NUF2/TRIP13/BUB1B/KIF18A/BUB1/AURKB/KIF2C/NCAPG/SMC4/CDT1/PLK1/CENPE/CDCA8/NDC80/CDK1/NCAPH/TPX2/KNL1/ZWINT/CHMP4BP1/CENPK/SPAG5/CDC20 49

BP GO:0007416 synapse assembly 52/2475 200/18870 6.86186138824318e-07 2.22695220405471e-05 1.61756425214745e-05 IL1RAPL1/LGI2/DNM3/FGF13/LINGO2/ADGRB3/NTNG2/CHRNB2/NRG3/SLITRK4/SLIT1/DSCAM/SHANK2/NEGR1/CUX2/GABRB3/C1QL3/CDH9/GAP43/SRPX2/OXTR/LRRTM3/CLSTN2/NRXN1/CBLN2/TLR2/BSN/DKK1/ABI3/FZD5/ASIC2/SNAP25/ZDHHC12/SLITRK1/GABRA1/CBLN1/GABRB2/IL1RAP/LZTS1/GABRG2/NPTX1/RAP2A/SPTBN2/AMIGO2/LRFN5/POU4F1/ICAM5/PCLO/LRTM2/FZD1/NTRK1/SLITRK5 52

BP GO:0044703 multi-organism reproductive process 53/2475 206/18870 7.6646343647292e-07 2.47411100676528e-05 1.79708999281681e-05 VMP1/TIMP1/THBD/ITGA5/WNT4/ITGB3/EMP2/FOSL1/TEAD4/ARHGDIB/STC1/ITGA2/SPP1/ACE/ABCC8/ITGA3/ACVR1C/MMP2/CRH/HAVCR2/IGFBP5/CSMD1/TEAD3/CNR1/HSPG2/MMP9/ITGB4/FBLN1/DSG2/OXTR/ANGPT2/IGFBP7/VEGFA/IDO1/CTSB/SYDE1/PRLHR/GJB2/PCSK5/CYP27B1/LIF/IGFBP2/H3-3A/NR2F2/RXFP1/PTHLH/TPPP3/HFE/ADM/PTN/UCP2/ADRA2B/NODAL 53

BP GO:0045730 respiratory burst 18/2475 40/18870 7.90309765625091e-07 2.53744376086259e-05 1.84309223697425e-05 NCF1B/NCF4/SLC11A1/NCF1/CYBA/TREM2/IGHA1/CLEC7A/SLAMF8/NCF2/IGHA2/CD52/DUSP10/JCHAIN/RAC2/PIK3CG/LBP/NCF1C 18

BP GO:0010959 regulation of metal ion transport 87/2475 398/18870 8.08812800513228e-07 2.58038473848932e-05 1.87428275387658e-05 AKAP5/CAV1/VMP1/AKAP6/KCNC1/RGS7/ITGB3/NKX2-5/STAC/SLN/TSPO/JPH4/KCNIP3/STC1/CYBA/HAMP/ACE/FGF13/ABCC8/TREM2/CABP4/FGF12/NIPSNAP2/MIR210/FLNA/KCNMB1/CD33/APLNR/LGALS3/LILRA5/P2RY6/CCL2/WNK2/KCNAB1/CD63/CXCL10/KCNS2/GLRX/SELENON/HTR2A/SCN2B/CCL5/KCNAB2/RYR2/CXCR4/VIP/HECW1/LYN/KCNN4/KCNE5/OSR1/GRIN1/CAMK2A/LILRB2/KCNE3/CXCL9/ADCYAP1R1/DPP10/F2RL3/LILRB1/CABP1/LILRA2/KCNC2/SCN3B/GCK/LRRC55/HPCA/JPH3/ITGB1/ADORA1/FXYD2/PRKCE/TRPV2/B2M/CXCL11/PIK3CG/FKBP1C/RGS4/ANK3/RAMP3/GAL/HFE/KCNE4/GRP/CCR1/FXYD5/KCNIP2 87

BP GO:0007269 neurotransmitter secretion 41/2475 144/18870 8.16577448889025e-07 2.58038473848932e-05 1.87428275387658e-05 UNC13A/STX1A/SYN1/SLC32A1/STX1B/NAPB/PRKCG/ABCC8/CPLX2/RIMS1/DOC2A/STXBP1/ASIC1/RAB3A/MYOF/UNC13C/PRKCB/SNCG/NRXN1/ERC2/SYN2/SYT7/SYT1/SYT4/LRRK2/CAMK2A/SNAP25/RAP1B/PPFIA2/SV2B/P2RY1/HRH3/SYP/SYN3/SYT5/PCLO/SYT2/RIMS3/CPLX1/GPR158/RIMS2 41

BP GO:0099643 signal release from synapse 41/2475 144/18870 8.16577448889025e-07 2.58038473848932e-05 1.87428275387658e-05 UNC13A/STX1A/SYN1/SLC32A1/STX1B/NAPB/PRKCG/ABCC8/CPLX2/RIMS1/DOC2A/STXBP1/ASIC1/RAB3A/MYOF/UNC13C/PRKCB/SNCG/NRXN1/ERC2/SYN2/SYT7/SYT1/SYT4/LRRK2/CAMK2A/SNAP25/RAP1B/PPFIA2/SV2B/P2RY1/HRH3/SYP/SYN3/SYT5/PCLO/SYT2/RIMS3/CPLX1/GPR158/RIMS2 41

BP GO:0045839 negative regulation of mitotic nuclear division 22/2475 56/18870 8.57056235027533e-07 2.69411813356299e-05 1.95689390009318e-05 CENPF/SPC24/MAD2L1/KNTC1/CCNB1/TOM1L1/BIRC5/HASPIN/TTK/ZWILCH/NUF2/TRIP13/BUB1B/BUB1/AURKB/PLK1/CDCA8/NDC80/KNL1/ZWINT/CHEK1/CDC20 22

BP GO:0033046 negative regulation of sister chromatid segregation 20/2475 48/18870 8.85978925316609e-07 2.7279063936415e-05 1.98143626860281e-05 CENPF/SPC24/MAD2L1/KNTC1/CCNB1/BIRC5/HASPIN/TTK/ZWILCH/NUF2/TRIP13/BUB1B/BUB1/AURKB/PLK1/CDCA8/NDC80/KNL1/ZWINT/CDC20 20

BP GO:0033048 negative regulation of mitotic sister chromatid segregation 20/2475 48/18870 8.85978925316609e-07 2.7279063936415e-05 1.98143626860281e-05 CENPF/SPC24/MAD2L1/KNTC1/CCNB1/BIRC5/HASPIN/TTK/ZWILCH/NUF2/TRIP13/BUB1B/BUB1/AURKB/PLK1/CDCA8/NDC80/KNL1/ZWINT/CDC20 20

BP GO:0045841 negative regulation of mitotic metaphase/anaphase transition 20/2475 48/18870 8.85978925316609e-07 2.7279063936415e-05 1.98143626860281e-05 CENPF/SPC24/MAD2L1/KNTC1/CCNB1/BIRC5/HASPIN/TTK/ZWILCH/NUF2/TRIP13/BUB1B/BUB1/AURKB/PLK1/CDCA8/NDC80/KNL1/ZWINT/CDC20 20

BP GO:2000816 negative regulation of mitotic sister chromatid separation 20/2475 48/18870 8.85978925316609e-07 2.7279063936415e-05 1.98143626860281e-05 CENPF/SPC24/MAD2L1/KNTC1/CCNB1/BIRC5/HASPIN/TTK/ZWILCH/NUF2/TRIP13/BUB1B/BUB1/AURKB/PLK1/CDCA8/NDC80/KNL1/ZWINT/CDC20 20

BP GO:0071706 tumor necrosis factor superfamily cytokine production 50/2475 191/18870 8.95983137340693e-07 2.73070190690026e-05 1.98346681164974e-05 LY96/PYCARD/TLR1/IL1A/TSPO/CYBA/OAS2/ABCC8/THBS1/TREM2/ELF4/BCL3/LILRA4/HAVCR2/CD33/HSPB1/LILRA5/ARID5A/CLEC7A/OAS1/SPON2/CD274/GPNMB/TNFAIP3/ANGPT1/TLR2/ZFP36/SASH3/IFIH1/FZD5/ACP5/OAS3/MIR27B/LRRK2/SPN/CD14/LTF/CD36/TYROBP/TWIST1/LILRB1/LILRA2/CD2/PTPRC/IL6/LBP/FCGR3A/ZC3H12A/FADD/MYD88 50

BP GO:1903555 regulation of tumor necrosis factor superfamily cytokine production 50/2475 191/18870 8.95983137340693e-07 2.73070190690026e-05 1.98346681164974e-05 LY96/PYCARD/TLR1/IL1A/TSPO/CYBA/OAS2/ABCC8/THBS1/TREM2/ELF4/BCL3/LILRA4/HAVCR2/CD33/HSPB1/LILRA5/ARID5A/CLEC7A/OAS1/SPON2/CD274/GPNMB/TNFAIP3/ANGPT1/TLR2/ZFP36/SASH3/IFIH1/FZD5/ACP5/OAS3/MIR27B/LRRK2/SPN/CD14/LTF/CD36/TYROBP/TWIST1/LILRB1/LILRA2/CD2/PTPRC/IL6/LBP/FCGR3A/ZC3H12A/FADD/MYD88 50

BP GO:0006836 neurotransmitter transport 53/2475 207/18870 9.00676412974545e-07 2.73114201186827e-05 1.98378648535542e-05 UNC13A/STX1A/SYN1/ITGB3/SYNGR3/SLC6A17/SLC32A1/STX1B/NAPB/SLC17A8/SLC6A15/PRKCG/ABCC8/CPLX2/RIMS1/DOC2A/STXBP1/ASIC1/RAB3A/SLC17A7/MYOF/UNC13C/PRKCB/SNCG/NRXN1/ERC2/SYN2/SYT7/SYT1/SYT4/LRRK2/SLC1A6/CAMK2A/SNAP25/SLC17A6/RAP1B/PPFIA2/SV2B/P2RY1/HRH3/SYP/SYN3/SYT5/PCLO/ITGB1/GDNF/SYT2/RIMS3/SLC29A1/CPLX1/GPR158/SLC6A7/RIMS2 53

BP GO:0032640 tumor necrosis factor production 49/2475 186/18870 9.39201918696897e-07 2.81948415992809e-05 2.04795449955855e-05 LY96/PYCARD/TLR1/IL1A/TSPO/CYBA/OAS2/ABCC8/THBS1/TREM2/ELF4/BCL3/LILRA4/HAVCR2/CD33/HSPB1/LILRA5/ARID5A/CLEC7A/OAS1/SPON2/GPNMB/TNFAIP3/ANGPT1/TLR2/ZFP36/SASH3/IFIH1/FZD5/ACP5/OAS3/MIR27B/LRRK2/SPN/CD14/LTF/CD36/TYROBP/TWIST1/LILRB1/LILRA2/CD2/PTPRC/IL6/LBP/FCGR3A/ZC3H12A/FADD/MYD88 49

BP GO:0032680 regulation of tumor necrosis factor production 49/2475 186/18870 9.39201918696897e-07 2.81948415992809e-05 2.04795449955855e-05 LY96/PYCARD/TLR1/IL1A/TSPO/CYBA/OAS2/ABCC8/THBS1/TREM2/ELF4/BCL3/LILRA4/HAVCR2/CD33/HSPB1/LILRA5/ARID5A/CLEC7A/OAS1/SPON2/GPNMB/TNFAIP3/ANGPT1/TLR2/ZFP36/SASH3/IFIH1/FZD5/ACP5/OAS3/MIR27B/LRRK2/SPN/CD14/LTF/CD36/TYROBP/TWIST1/LILRB1/LILRA2/CD2/PTPRC/IL6/LBP/FCGR3A/ZC3H12A/FADD/MYD88 49

BP GO:0030217 T cell differentiation 71/2475 307/18870 9.61440788794996e-07 2.87188581886824e-05 2.08601685675709e-05 ASCL2/WNT4/IL7R/MDK/BATF/IL18/MR1/LEF1/NFKBIZ/SPI1/IL1A/RELB/LOXL3/FANCD2/LCK/ANXA1/ACTL6B/TESPA1/CD3D/BCL3/FZD7/FOXJ1/CD74/SOCS1/RIPK3/TNFSF8/RHOH/RUNX1/HLA-DOA/CD8A/GLI3/PTGER4/TOX/HLA-DRB1/CD3E/JAK3/IL2RG/SASH3/FZD5/DUSP10/CTSL/RUNX2/SOCS3/BTN2A2/RUNX3/SPN/LILRB2/HLA-DRA/KDELR1/TCF7/RSAD2/FGL2/FCER1G/CD2/FOSL2/PTPRC/WNT10B/B2M/IL6/IL2RA/ZC3H12A/PIK3R6/FADD/IRF1/TCIRG1/IL4I1/DLL4/GPR183/CDK6/CAMK4/FANCA 71

BP GO:0046631 alpha-beta T cell activation 48/2475 181/18870 9.81766766273414e-07 2.91808300232949e-05 2.11957254438288e-05 TRAC/ASCL2/BATF/IL18/LEF1/NFKBIZ/NKG7/RELB/LOXL3/ANXA1/CD247/ELF4/CD3D/BCL3/SOCS1/TWSG1/TNFSF8/RUNX1/CD274/GLI3/TRBC1/PTGER4/TOX/HLA-DRB1/CD3E/JAK3/IL2RG/SASH3/CTSL/SOCS3/RUNX3/SPN/HLA-DRA/TRBC2/CD300A/LILRB1/HLA-A/RSAD2/FOSL2/PTPRC/IL6/IL2RA/ZC3H12A/TNFRSF14/IRF1/HFE/TCIRG1/GPR183 48

BP GO:1901888 regulation of cell junction assembly 54/2475 213/18870 9.95113338554309e-07 2.94318250476851e-05 2.13780376542935e-05 IL1RAPL1/CAV1/WNT4/SNAI1/ACE/LINGO2/THBS1/ADGRB3/CLDN1/NTNG2/CHRNB2/HOPX/NRP1/SLITRK4/SLIT1/APLNR/NEGR1/CUX2/S100A10/SRPX2/OXTR/LRRTM3/CLSTN2/MMP14/NRXN1/CBLN2/VEGFA/TLR2/CAMSAP3/DKK1/TBX5/ABI3/FZD5/ASIC2/F11R/SNAP25/SLITRK1/RAP1B/CBLN1/SDC4/IL1RAP/LZTS1/CNTNAP2/RAP2A/EPHA2/AMIGO2/LRFN5/SNAI2/ICAM5/LRTM2/FZD1/EPHA3/NTRK1/SLITRK5 54

BP GO:0000075 cell cycle checkpoint signaling 50/2475 192/18870 1.05923714029028e-06 3.11748028936413e-05 2.26440633241621e-05 CENPF/SPC24/MDM2/MAD2L1/PARP9/PLK3/FANCD2/FBXO4/KNTC1/RBBP8/CHEK2/WDR76/MAP3K20/CCNB1/DTL/NABP1/TIMELESS/ORC1/BIRC5/BRCA1/CAMSAP3/HASPIN/TTK/MUC1/ZWILCH/DTX3L/RAD51/NUF2/CDK2/TRIP13/BUB1B/BRCA2/BUB1/AURKB/CLSPN/CDC45/E2F1/BRIP1/CDT1/PLK1/CDC6/CDCA8/NDC80/CDK1/GTSE1/KNL1/ZWINT/CHEK1/EME1/CDC20 50

BP GO:0033631 cell-cell adhesion mediated by integrin 11/2475 17/18870 1.11796307122866e-06 3.2742684291009e-05 2.37829063111698e-05 ITGA5/CXCL13/NPNT/ITGA4/CD3E/CCL5/ADAM9/ITGB1/DPP4/SWAP70/FERMT3 11

BP GO:0030900 forebrain development 88/2475 407/18870 1.13856079482494e-06 3.31840728744125e-05 2.410351238099e-05 STIL/KCNA1/KCNC1/WNT4/CDK5R2/MDK/EMX1/PITX1/SSTR3/SLC4A10/CDK5R1/SEMA3E/KIF14/LEF1/SLC32A1/SSTR2/KIF26A/FGF13/NEFL/CHRNB2/NRP1/SEMA3A/NRG3/COL3A1/FLNA/EGFR/TWSG1/SLIT1/DLX2/TACC3/RTN4R/HTR5A/BMP2/NR2E1/DNAJB1/DLX5/ATF5/OXTR/OPHN1/GLI3/SCN2A/TOX/FEZF2/GSX2/GSC/DKK1/RTN4RL1/CXCR4/SEMA6B/DMRTA2/LHX5/LRRK2/TNR/TYROBP/GLI1/CHD5/LAMB1/OTX1/NR2F2/OTP/CASP3/E2F1/DRAXIN/KCNC2/CNTNAP2/PHACTR1/NDE1/ELAVL4/TBR1/POU4F1/ALDH1A3/ZIC1/SSTR1/WDR62/B2M/LHX6/NEUROD6/TBX19/TUBA1A/SLITRK5/NOG/ASPM/AQP1/EZH2/PLCB1/MGARP/WNT7B/CDK6 88

BP GO:0045669 positive regulation of osteoblast differentiation 26/2475 74/18870 1.14452075999268e-06 3.31966311255848e-05 2.41126341655208e-05 WNT4/WWTR1/NPNT/GDPD2/DNAI3/MIR210/CCN1/BMP2/CCN4/JAG1/GLI3/NELL1/SFRP2/HGF/FAM20C/RUNX2/CEBPD/TENT5A/CEBPB/CTHRC1/LTF/GDF10/WNT10B/IL6/CLIC1/WNT7B 26

BP GO:0043270 positive regulation of monoatomic ion transport 54/2475 214/18870 1.16355666150455e-06 3.34680606856992e-05 2.43097891617609e-05 AKAP5/CAV1/VMP1/AKAP6/KCNC1/RGS7/TMSB4X/NKX2-5/STAC/TSPO/STC1/FGF13/ABCC8/TREM2/FGF12/NIPSNAP2/MIR210/FLNA/KCNMB1/APLNR/LGALS3/LILRA5/P2RY6/CCL2/WNK2/CHRM1/CXCL10/GLRX/PIRT/CCL5/RYR2/KCNN4/KCNE5/GRIN1/CAMK2A/CXCL9/ARC/ADCYAP1R1/F2RL3/P2RY1/LILRA2/KCNC2/SCN3B/LRRC55/ADORA1/FXYD2/TRPV2/CXCL11/ANK3/RAMP3/GAL/CCR1/CTSS/KCNIP2 54

BP GO:0046718 viral entry into host cell 43/2475 156/18870 1.16502742893257e-06 3.34680606856992e-05 2.43097891617609e-05 CAV1/ITGA5/ITGB3/TNFRSF4/SIGLEC1/ITGA2/TFRC/CLDN1/ICAM1/NRP1/VAMP8/CD74/TRIM21/EGFR/TRIM38/TRIM22/CIITA/IFITM3/HLA-DRB1/PLSCR1/CTSB/HTR2A/CAV2/CXCR4/CLEC5A/IFITM2/CTSL/F11R/ANPEP/NECTIN2/CCR5/LGALS1/TRIM6/TRIM5/EPHA2/EFNB2/ITGB1/DPP4/CDK1/JPT2/PTX3/TNFRSF14/SLC1A5 43

BP GO:0002705 positive regulation of leukocyte mediated immunity 42/2475 151/18870 1.19089965593474e-06 3.40483882582485e-05 2.47313146593365e-05 KLRC2/IL18/MR1/KLRC3/SPI1/ITGB2/TREM2/CD40/FCGR1A/TFRC/HPX/KLRC4/HLA-C/IL1R1/MICA/ARID5A/CLEC7A/KLRC4-KLRK1/HLA-DRB1/MICB/F2RL1/SCIMP/SASH3/FZD5/HLA-DRA/NOS2/NECTIN2/TYROBP/HLA-B/ZP3/HLA-A/RSAD2/ULBP2/ULBP3/C3/PTPRC/B2M/IL6/FADD/HFE/CLCF1/HLA-F 42

BP GO:2000027 regulation of animal organ morphogenesis 37/2475 126/18870 1.21965167680665e-06 3.47051595618347e-05 2.52083656623095e-05 WNT4/NGFR/RSPO3/CELSR3/HOXC11/HOXA11/FZD7/BMP2/SOX8/PLEKHA4/PRKCB/SP6/VEGFA/DKK1/SFRP2/JHY/HGF/RUNX2/PDGFA/CTHRC1/TACSTD2/FZD2/NKD1/TNFRSF11B/ARHGEF19/SNAI2/SPEF1/TBX1/FZD1/AJAP1/GDNF/STOX1/CELSR1/SPRY1/NOG/FZD6/HOXB7 37

BP GO:0034508 centromere complex assembly 15/2475 30/18870 1.24490680403738e-06 3.52567002426434e-05 2.56089815746119e-05 CENPI/CENPF/CENPV/CENPW/KNTC1/CENPA/CENPH/DLGAP5/HJURP/CENPN/H3-3A/OIP5/ITGB3BP/CENPE/CENPK 15

BP GO:0008038 neuron recognition 19/2475 45/18870 1.3004836447559e-06 3.66577643338706e-05 2.66266554990052e-05 CDK5R1/CRTAC1/EMB/NRP1/DSCAM/CNR1/GAP43/DSCAML1/FEZF2/CXCR4/IGSF9/CNTN6/CASP3/CNTNAP2/CNTN4/NEXN/EPHA3/PALLD/OPCML 19

BP GO:1903038 negative regulation of leukocyte cell-cell adhesion 42/2475 152/18870 1.43941209563602e-06 4.03842533747601e-05 2.93334201289721e-05 ASCL2/MDK/PLA2G2A/MIR221/LOXL3/LRRC32/ANXA1/PAWR/FOXJ1/CD74/SOCS1/HAVCR2/TWSG1/LGALS3/RUNX1/PLA2G5/CD274/GLI3/GPNMB/HLA-DRB1/JAK3/IDO1/PDCD1LG2/FCGR2B/VSIG4/BTN2A2/RUNX3/SPN/CEBPB/LILRB2/CD300A/LAPTM5/LILRB1/SDC4/CASP3/FGL2/IL2RA/ZC3H12A/TNFRSF14/IRF1/HFE/IL4I1 42

BP GO:0051306 mitotic sister chromatid separation 23/2475 62/18870 1.59168113503637e-06 4.44486210918995e-05 3.22856055934184e-05 CENPF/SPC24/MAD2L1/KNTC1/UBE2C/CCNB1/DLGAP5/BIRC5/HASPIN/ESPL1/TTK/ZWILCH/NUF2/TRIP13/BUB1B/BUB1/AURKB/PLK1/CDCA8/NDC80/KNL1/ZWINT/CDC20 23

BP GO:0071356 cellular response to tumor necrosis factor 58/2475 238/18870 1.60946630559905e-06 4.47372023093365e-05 3.24952188308813e-05 TRADD/PYCARD/TMSB4X/CCL26/TNFRSF4/TRAF5/NPNT/CTSK/CXCL8/CCL20/FAS/GBP2/PYDC1/THBS1/TNFRSF19/CD40/CD70/CLDN1/CYP1B1/GBP1/CCL2/STAT1/BIRC3/TNFRSF1A/CASP1/TNFAIP3/CASP8/BRCA1/SMPD3/ZFP36/CARD16/F2RL1/CD58/CCL5/PLVAP/KRT18/SPHK1/CHI3L1/HAS2/MYOD1/H2BC11/TNFRSF18/COL1A1/PID1/SLC2A4/MIR27B/GBP3/LRRK2/YBX3/CCL18/LAPTM5/POSTN/ADAMTS7/ZC3H12A/CASP4/TNFRSF14/GPD1/TRAF1 58

BP GO:0034341 response to type II interferon 40/2475 143/18870 1.81238392051336e-06 4.99153810034963e-05 3.62564296604869e-05 SP100/GBP5/SLC11A1/CCL26/PARP9/ZYX/CCL20/GBP2/IFNGR2/CD40/CLDN1/GCH1/HPX/CD74/TRIM21/STXBP1/CITED1/HLA-DPA1/GBP1/CCL2/STAT1/GBP4/CIITA/IFITM3/RAB7B/CASP1/TLR2/CD58/CCL5/IFITM2/CYP27B1/GBP3/NLRC5/CCL18/CAMK2A/NOS2/VIM/PPARG/IRF1/BST2 40

BP GO:1903900 regulation of viral life cycle 40/2475 143/18870 1.81238392051336e-06 4.99153810034963e-05 3.62564296604869e-05 APOBEC3B/OASL/TOP2A/CXCL8/OAS2/KPNA2/FAM111A/APOBEC3G/CD74/TRIM21/MX1/APOBEC3C/TRIM38/TRIM22/OAS1/CIITA/IFITM3/HLA-DRB1/PLSCR1/IFI16/CCL5/APOBEC3F/IFITM2/IFIH1/OAS3/SLPI/LTF/PPIA/LAMP3/ISG15/NECTIN2/LGALS1/TRIM6/TRIM5/RSAD2/BTBD17/PTX3/ISG20/APOBEC3H/BST2 40

BP GO:0018212 peptidyl-tyrosine modification 65/2475 278/18870 1.83078352231557e-06 5.00058535391079e-05 3.63221450984474e-05 CAV1/ITGA5/ITGB3/IL18/NCF1/MIR221/PARP9/ITGB2/EFEMP1/LCK/ACE/TREM2/CD40/FCGR1A/HPX/NRP1/CD74/SOCS1/NOX4/EGFR/CD44/LILRA5/AREG/ERRFI1/TNFRSF1A/CTF1/CD3E/SAMSN1/ANGPT1/JAK3/VEGFA/HTR2A/CCL5/GFRA1/EHD4/SFRP2/IGF2/IL11/GRM5/BST1/TNFRSF18/HGF/FES/LYN/SOCS3/TNK2/CSF3/PDGFA/LIF/GPRC5A/CD36/CD300A/THBS4/PDGFD/OSM/IL9/TPST1/PTPRC/EPHA3/PRKCE/NTRK1/IL6/TNFRSF14/HCLS1/CLCF1 65

BP GO:0007091 metaphase/anaphase transition of mitotic cell cycle 30/2475 94/18870 1.83232641215918e-06 5.00058535391079e-05 3.63221450984474e-05 CENPF/SPC24/MAD2L1/BCL7A/ACTL6B/KNTC1/UBE2C/TACC3/MAP3K20/CCNB1/NEK6/DLGAP5/BIRC5/HASPIN/ESPL1/TTK/ZWILCH/NUF2/TRIP13/BUB1B/BUB1/AURKB/PLK1/CDC6/CENPE/CDCA8/NDC80/KNL1/ZWINT/CDC20 30

BP GO:0033047 regulation of mitotic sister chromatid segregation 21/2475 54/18870 1.84236947163e-06 5.00524267315226e-05 3.63559739031344e-05 CENPF/SPC24/MAD2L1/KNTC1/CCNB1/BIRC5/HASPIN/TTK/ZWILCH/NUF2/TRIP13/BUB1B/BUB1/AURKB/PLK1/CDCA8/NDC80/CDK1/KNL1/ZWINT/CDC20 21

BP GO:0042119 neutrophil activation 18/2475 42/18870 1.89902300864259e-06 5.09963325433412e-05 3.70415873149589e-05 IL18/PLA2G2A/SPI1/ITGB2/CXCL8/ANXA1/PRAM1/F2RL1/FCGR2B/CCL5/SCNN1B/CD300A/TYROBP/LILRA2/CXCR2/FCER1G/CXCL6/MYD88 18

BP GO:0001914 regulation of T cell mediated cytotoxicity 20/2475 50/18870 1.91958213770738e-06 5.09963325433412e-05 3.70415873149589e-05 IL7R/MR1/RIPK3/HLA-C/MICA/HLA-DRB1/MICB/FCGR2B/HLA-DRA/NECTIN2/HLA-B/LILRB1/HLA-A/ULBP2/ULBP3/PTPRC/B2M/FADD/HFE/HLA-F 20

BP GO:0051985 negative regulation of chromosome segregation 20/2475 50/18870 1.91958213770738e-06 5.09963325433412e-05 3.70415873149589e-05 CENPF/SPC24/MAD2L1/KNTC1/CCNB1/BIRC5/HASPIN/TTK/ZWILCH/NUF2/TRIP13/BUB1B/BUB1/AURKB/PLK1/CDCA8/NDC80/KNL1/ZWINT/CDC20 20

BP GO:1902100 negative regulation of metaphase/anaphase transition of cell cycle 20/2475 50/18870 1.91958213770738e-06 5.09963325433412e-05 3.70415873149589e-05 CENPF/SPC24/MAD2L1/KNTC1/CCNB1/BIRC5/HASPIN/TTK/ZWILCH/NUF2/TRIP13/BUB1B/BUB1/AURKB/PLK1/CDCA8/NDC80/KNL1/ZWINT/CDC20 20

BP GO:1905819 negative regulation of chromosome separation 20/2475 50/18870 1.91958213770738e-06 5.09963325433412e-05 3.70415873149589e-05 CENPF/SPC24/MAD2L1/KNTC1/CCNB1/BIRC5/HASPIN/TTK/ZWILCH/NUF2/TRIP13/BUB1B/BUB1/AURKB/PLK1/CDCA8/NDC80/KNL1/ZWINT/CDC20 20

BP GO:0002709 regulation of T cell mediated immunity 31/2475 99/18870 1.95950567936749e-06 5.18276303917287e-05 3.76454070466938e-05 IL7R/IL18/MR1/RIPK3/AHR/HLA-C/IL1R1/MICA/ARID5A/HLA-DRB1/MICB/FCGR2B/SASH3/FZD5/SPN/HLA-DRA/NECTIN2/HLA-B/LILRB1/ZP3/HLA-A/RSAD2/ULBP2/ULBP3/PTPRC/B2M/IL6/FADD/HFE/IL4I1/HLA-F 31

BP GO:0006956 complement activation 24/2475 67/18870 1.97106776216656e-06 5.19047844037194e-05 3.77014484702496e-05 C1RL/CFI/IGHG4/C1R/TREM2/IGHA1/IGHG2/C7/IGHG3/VSIG4/IGHA2/SERPING1/C2/IGHG1/C1S/CFD/IGHM/C3/C1QB/COLEC11/C1QC/C1QA/CFH/NCR3LG1 24

BP GO:0031343 positive regulation of cell killing 26/2475 76/18870 2.04371090434168e-06 5.35827086011679e-05 3.8920221910768e-05 KLRC2/MR1/KLRC3/SPI1/KLRC4/HLA-C/MICA/CLEC7A/KLRC4-KLRK1/HLA-DRB1/MICB/F2RL1/HLA-DRA/NOS2/NECTIN2/TYROBP/HLA-B/HLA-A/PRF1/ULBP2/ULBP3/PTPRC/B2M/FADD/HFE/HLA-F 26

BP GO:0033045 regulation of sister chromatid segregation 32/2475 104/18870 2.0647827828631e-06 5.38998079491741e-05 3.91505495167131e-05 CENPF/SPC24/MAD2L1/BCL7A/ACTL6B/KNTC1/UBE2C/TACC3/MAP3K20/CCNB1/RMI2/NEK6/DLGAP5/BIRC5/HASPIN/ESPL1/TTK/ZWILCH/NUF2/TRIP13/BUB1B/BUB1/AURKB/PLK1/CDC6/CENPE/CDCA8/NDC80/CDK1/KNL1/ZWINT/CDC20 32

BP GO:0002687 positive regulation of leukocyte migration 41/2475 149/18870 2.14574619523354e-06 5.57708231869358e-05 4.05095761533495e-05 ASCL2/RARRES2/MDK/ITGB3/PYCARD/CXCL13/SPI1/IL1A/CXCL8/ITGA2/CCL20/THBS1/TREM2/ICAM1/CD74/LGALS3/IL1R1/ITGA4/MMP14/VEGFA/CXCL10/C5AR1/F2RL1/CCL5/PLVAP/TNFRSF18/FPR2/SERPINE1/SPN/THBS4/ZP3/RAC2/PDGFD/CXCR2/IL6/LBP/FADD/SWAP70/TNFRSF14/PTN/CCR1 41

BP GO:0030071 regulation of mitotic metaphase/anaphase transition 29/2475 90/18870 2.17636452688129e-06 5.63228130146347e-05 4.09105183070289e-05 CENPF/SPC24/MAD2L1/BCL7A/ACTL6B/KNTC1/UBE2C/MAP3K20/CCNB1/NEK6/DLGAP5/BIRC5/HASPIN/ESPL1/TTK/ZWILCH/NUF2/TRIP13/BUB1B/BUB1/AURKB/PLK1/CDC6/CENPE/CDCA8/NDC80/KNL1/ZWINT/CDC20 29

BP GO:0051784 negative regulation of nuclear division 23/2475 63/18870 2.19748438184779e-06 5.66253057022067e-05 4.11302362502616e-05 CENPF/SPC24/MAD2L1/KNTC1/CCNB1/TOM1L1/BIRC5/HASPIN/TTK/ZWILCH/LIF/NUF2/TRIP13/BUB1B/BUB1/AURKB/PLK1/CDCA8/NDC80/KNL1/ZWINT/CHEK1/CDC20 23

BP GO:0002824 positive regulation of adaptive immune response based on somatic recombination of immune receptors built from immunoglobulin superfamily domains 36/2475 124/18870 2.27084262476911e-06 5.82655517910844e-05 4.23216419002179e-05 IL18/MR1/SLC11A1/NFKBIZ/TREM2/CD40/FCGR1A/TFRC/HPX/HLA-C/IL1R1/MICA/ARID5A/CLEC7A/CD274/HLA-DRB1/MICB/SASH3/FZD5/HLA-DRA/NECTIN2/HLA-B/ZP3/HLA-A/RSAD2/ULBP2/ULBP3/C3/PTPRC/B2M/IL6/PLA2G4A/FADD/HFE/CLCF1/HLA-F 36

BP GO:0002440 production of molecular mediator of immune response 71/2475 314/18870 2.29615063634393e-06 5.86642060451444e-05 4.26112075537419e-05 IGLV1-44/BATF/PYCARD/IL18/IGLV2-11/NFKBIZ/IGKV3-20/TNFRSF4/IGLV2-23/IGLV3-21/IGLV6-57/IGLV3-1/IGLV2-14/LITAF/KLK7/CGAS/CD40/IGLV2-8/POLQ/TFRC/HPX/VPREB3/CD74/IGLV8-61/IL13RA2/IL1R1/IGKV1-5/ARID5A/CLEC7A/IGKV3-15/SLAMF9/GAPT/SPON2/IGLV1-47/IGKV2-30/ANGPT1/JAK3/IGLV5-45/F2RL1/FCGR2B/SCIMP/SASH3/IGLV3-25/FZD5/ACP5/IGKV4-1/EXO1/CD36/LAPTM5/TWIST1/LILRB1/IGLV7-46/TRIM6/HLA-A/RSAD2/IGLV3-10/PTPRC/B2M/IL6/IGLV3-19/TGFB2/SWAP70/TNFRSF14/HFE/MYD88/IGLV1-40/TREM1/IGKV1-9/CLCF1/HLA-F/BST2 71

BP GO:0045089 positive regulation of innate immune response 76/2475 343/18870 2.31344765052365e-06 5.88195092924351e-05 4.27240132891333e-05 CAV1/KLRC2/OASL/LY96/GBP5/IRF7/PYCARD/TLR1/KLRC3/NFKBIZ/SPI1/FOSL1/PARP9/RBM47/CYBA/GBP2/MMP12/TREM2/CGAS/CD40/HPX/LILRA4/KLRC4/HAVCR2/CLEC7A/TIFA/OAS1/BIRC3/PLA2G5/KLRC4-KLRK1/RAB7B/CASP1/TNFAIP3/CD300LF/TLR2/PLSCR1/IFI16/F2RL1/NMI/CCL5/SCIMP/IFIH1/TLR8/CASP6/LYN/FPR2/OAS3/NLRC5/CD14/LTF/ZDHHC12/SLC15A3/PAK3/CD36/CD300A/NECTIN2/TYROBP/LILRA2/IFI35/TRIM6/TRIM5/RSAD2/ALPK1/UNC93B1/RNF135/HMGB2/COLEC11/PRKCE/LBP/FADD/IRAK2/IRF1/MYD88/HLA-F/CTSS/COLEC12 76

BP GO:0051304 chromosome separation 27/2475 81/18870 2.3218227352277e-06 5.88195092924351e-05 4.27240132891333e-05 CENPF/SPC24/MAD2L1/KNTC1/UBE2C/CCNB1/DLGAP5/BIRC5/PLSCR1/HASPIN/ESPL1/TTK/ZWILCH/NUF2/TRIP13/BUB1B/BUB1/AURKB/NCAPG/SMC4/PLK1/CDCA8/NDC80/NCAPH/KNL1/ZWINT/CDC20 27

BP GO:0006813 potassium ion transport 58/2475 241/18870 2.46529367195221e-06 6.21916941445424e-05 4.51734262843787e-05 CAV1/KCNA1/AKAP6/KCNC1/RGS7/HCN1/KCNN1/KCNIP3/ABCC8/TREM2/KCNB1/FLNA/KCNMB1/KCNV1/KCNH3/SLC17A7/KCNAB1/KCNK7/CD63/KCNQ5/KCNS2/HTR2A/NALCN/KCNAB2/KCNH1/VIP/KCNA4/KCNK3/KCNN4/KCNK12/KCNE5/SLC12A5/KCNJ11/SNAP25/KCNE3/CDK2/SLC17A6/KCNJ3/DPP10/SLC12A7/KCNH7/KCNJ12/KCNC2/GCK/LRRC55/ITGB1/ADORA1/FXYD2/KCNJ9/RGS4/KCNT1/ANK3/GAL/KCNE4/AQP1/GRP/KCNJ4/KCNIP2 58

BP GO:0009952 anterior/posterior pattern specification 54/2475 219/18870 2.48521875097785e-06 6.24320225141046e-05 4.53479906861979e-05 LEFTY2/HOXD3/DDIT3/MEOX2/HOXB2/HOXA6/HOXA5/LEF1/HOXC6/HOXB3/RIPPLY2/HOXA3/HOXC11/HOXA11/HOXA2/DLL3/HOXD9/HOXB4/HOXD4/BMP2/GLI3/HOXA9/FEZF2/HOXC9/HOXC4/DKK1/SFRP2/FZD5/PCSK5/PCSK6/OSR1/HOXD10/ARC/AURKA/NKD1/OTX1/NR2F2/CRB2/HOXA10/HOXC10/BARX1/HOXA7/HOXD8/HOXB8/GATA4/TBX1/HOXC13/HOXC8/XRCC2/NOG/EN1/HOXD13/NODAL/HOXB7 54

BP GO:1904062 regulation of monoatomic cation transmembrane transport 71/2475 315/18870 2.59052649932252e-06 6.48063379247183e-05 4.70725933626894e-05 AKAP5/CAV1/VMP1/AKAP6/KCNC1/RGS7/ITGB3/TMSB4X/STAC/SLN/JPH4/KCNIP3/CYBA/HAMP/FGF13/TREM2/CABP4/FGF12/NIPSNAP2/MIR210/FLNA/KCNMB1/APLNR/P2RY6/MMP9/WNK2/KCNAB1/CD63/CXCL10/KCNS2/GLRX/PIRT/SELENON/GRIN2A/SCN2B/KCNAB2/RYR2/HECW1/LYN/KCNN4/KCNE5/OSR1/GRIN1/KCNE3/CXCL9/ADCYAP1R1/DPP10/F2RL3/CABP1/KCNC2/SCN3B/LRRC55/HPCA/JPH3/ITGB1/FXYD2/PRKCE/CXCL11/PIK3CG/FKBP1C/RGS4/ANK3/RAMP3/GAL/TCIRG1/KCNE4/GRIN2B/GRP/FXYD5/CTSS/KCNIP2 71

BP GO:0048167 regulation of synaptic plasticity 53/2475 214/18870 2.66434640072943e-06 6.63764970538568e-05 4.8213090798087e-05 AKAP5/SLC4A10/JPH4/CPEB3/ACE/VGF/PRRT1/PRKCG/CPLX2/RIMS1/KCNB1/STXBP1/NEUROD2/SHANK2/RAB3A/NR2E1/UNC13C/SHISA7/ERC2/SYT7/SLC8A3/GRIN2A/HRH1/GRM5/S100B/SLC8A2/MAPT/RASGRF1/SYT4/GRIN1/CAMK2A/SNAP25/LILRB2/ARC/GRM2/TNR/TYROBP/CBLN1/RASGRF2/SYP/LZTS1/MIR320E/CNTN4/JPH3/ADORA1/SYNGR1/RIMS3/NEURL1/NOG/GRIN2B/PTN/RIMS2/CDC20 53

BP GO:0042116 macrophage activation 33/2475 110/18870 2.68595236892422e-06 6.66382562934753e-05 4.84032216809615e-05 TLR1/SLC11A1/ITGB2/HAMP/IFNGR2/THBS1/TREM2/CTSC/CD74/HAVCR2/SBNO2/CD93/TAFA3/C5AR1/TLR2/NMI/FCGR2B/SPHK1/VSIG4/MAPT/LRRK2/TYROBP/CST7/IFI35/LRFN5/PTPRC/PRKCE/IL6/LBP/PLA2G4A/C1QA/FCGR3A/ZC3H12A 33

BP GO:0060485 mesenchyme development 73/2475 327/18870 2.72981314996403e-06 6.74477290221565e-05 4.89911885653063e-05 MDM2/CUL7/WNT4/MDK/EFNB1/HAND2/LOXL2/TGFB1I1/EMP2/SNAI1/SEMA3E/HOXA5/LEF1/WWTR1/NKX2-5/MIR221/LOXL3/SPRY2/DLL3/RET/NRP1/SEMA3A/SEMA3D/FLNA/WNT16/APLNR/STAT1/BMP2/SOX8/TEAD2/FAM83D/JAG1/ENG/FOXD1/SP6/ACTA2/GSC/FOLR1/FRZB/MDM4/PDPN/DKK1/SFRP2/SEMA6B/TBX5/HAS2/HGF/COL1A1/FN1/ACTG2/OSR1/TMEM100/SOX10/TWIST1/CRB2/SEMA3F/SPRED3/S100A4/VASN/SNAI2/RBM24/GATA4/TBX1/RDH10/EPHA3/GDNF/IL6/TGFB2/SPRY1/NOG/BNC2/EZH2/NODAL 73

BP GO:0002821 positive regulation of adaptive immune response 37/2475 130/18870 2.78172846837021e-06 6.84487611643227e-05 4.97182961365736e-05 PYCARD/IL18/MR1/SLC11A1/NFKBIZ/TREM2/CD40/FCGR1A/TFRC/HPX/HLA-C/IL1R1/MICA/ARID5A/CLEC7A/CD274/HLA-DRB1/MICB/SASH3/FZD5/HLA-DRA/NECTIN2/HLA-B/ZP3/HLA-A/RSAD2/ULBP2/ULBP3/C3/PTPRC/B2M/IL6/PLA2G4A/FADD/HFE/CLCF1/HLA-F 37

BP GO:0033555 multicellular organismal response to stress 29/2475 91/18870 2.79747845221265e-06 6.85553494983051e-05 4.97957174114586e-05 HTR1A/MDK/HCN1/TSPO/PRKCG/THBS1/GCH1/RET/GABRA5/NEUROD2/ASIC1/NR2E1/IDO1/BRINP1/PIRT/SELENON/HTR2C/ADCYAP1R1/THBS4/ASIC4/SCN3A/DPP4/MAPK8IP2/AQP9/NTRK1/VWA1/NMUR2/GRP/ADAM11 29

BP GO:0099003 vesicle-mediated transport in synapse 54/2475 220/18870 2.87959089238547e-06 7.028074682066e-05 5.1048973329709e-05 UNC13A/SNAP91/STX1A/SYN1/ITGB3/CDK5R1/DNM3/SLC32A1/STX1B/NAPB/SLC17A8/PRKCG/CPLX2/RIMS1/DOC2A/STXBP1/RAB3A/SLC17A7/UNC13C/SH3GL2/PRKCB/RAPGEF4/OPHN1/BRSK2/SNCG/ATP6V1G2/ERC2/SYN2/SYT7/BSN/SYT1/SLC2A4/SYT4/LRRK2/SNAP25/SLC17A6/ARC/RAP1B/PPFIA2/SV2B/P2RY1/SNCB/SYP/PACSIN1/SYN3/SYT5/EFNB2/HPCA/PCLO/CALY/SYT2/RIMS3/CPLX1/RIMS2 54

BP GO:0018108 peptidyl-tyrosine phosphorylation 64/2475 276/18870 2.94408978730856e-06 7.15640286761159e-05 5.1981095200594e-05 CAV1/ITGA5/ITGB3/IL18/NCF1/MIR221/PARP9/ITGB2/EFEMP1/LCK/ACE/TREM2/CD40/FCGR1A/HPX/NRP1/CD74/SOCS1/NOX4/EGFR/CD44/LILRA5/AREG/ERRFI1/TNFRSF1A/CTF1/CD3E/SAMSN1/ANGPT1/JAK3/VEGFA/HTR2A/CCL5/GFRA1/EHD4/SFRP2/IGF2/IL11/GRM5/BST1/TNFRSF18/HGF/FES/LYN/SOCS3/TNK2/CSF3/PDGFA/LIF/GPRC5A/CD36/CD300A/THBS4/PDGFD/OSM/IL9/PTPRC/EPHA3/PRKCE/NTRK1/IL6/TNFRSF14/HCLS1/CLCF1 64

BP GO:0016079 synaptic vesicle exocytosis 30/2475 96/18870 2.98673896616781e-06 7.23079869067401e-05 5.25214751138932e-05 UNC13A/STX1A/SYN1/STX1B/NAPB/PRKCG/CPLX2/RIMS1/DOC2A/STXBP1/RAB3A/UNC13C/PRKCB/ERC2/SYT7/SYT1/SYT4/LRRK2/SNAP25/RAP1B/PPFIA2/SV2B/P2RY1/SYP/SYT5/PCLO/SYT2/RIMS3/CPLX1/RIMS2 30

BP GO:0001505 regulation of neurotransmitter levels 48/2475 188/18870 3.1439937074351e-06 7.58093904395194e-05 5.50647471143674e-05 UNC13A/STX1A/SYN1/ITGB3/SYNGR3/SLC32A1/STX1B/NAPB/SLC17A8/PRKCG/ABCC8/CPLX2/RIMS1/DOC2A/STXBP1/ASIC1/RAB3A/MYOF/UNC13C/PRKCB/SNCG/NRXN1/ERC2/SYN2/SYT7/SYT1/SYT4/LRRK2/SLC1A6/CAMK2A/SNAP25/RAP1B/PPFIA2/SV2B/P2RY1/HRH3/SYP/SYN3/SYT5/PCLO/ITGB1/GDNF/SYT2/RIMS3/SLC29A1/CPLX1/GPR158/RIMS2 48

BP GO:0007566 embryo implantation 22/2475 60/18870 3.34552081896748e-06 8.0346027988323e-05 5.83599694862411e-05 VMP1/TIMP1/ITGB3/EMP2/TEAD4/ARHGDIB/STC1/SPP1/ACVR1C/MMP2/HSPG2/MMP9/ITGB4/FBLN1/IGFBP7/VEGFA/SYDE1/PCSK5/LIF/H3-3A/TPPP3/NODAL 22

BP GO:0022408 negative regulation of cell-cell adhesion 51/2475 205/18870 3.54877129135485e-06 8.41392609129703e-05 6.11152140612286e-05 ASCL2/MDK/PLA2G2A/SPI1/MIR221/LOXL3/LRRC32/IL1RN/ANXA1/C1QTNF1/PAWR/FOXJ1/CD74/SOCS1/HAVCR2/TWSG1/LGALS3/RUNX1/BMP2/PLA2G5/JAG1/CD274/GLI3/GPNMB/HLA-DRB1/JAK3/VEGFA/IDO1/PDCD1LG2/FCGR2B/VSIG4/BTN2A2/RUNX3/SPN/CEBPB/LILRB2/CD300A/TNR/LAPTM5/LILRB1/SDC4/CASP3/FGL2/IL2RA/ZC3H12A/SWAP70/TNFRSF14/IRF1/HFE/IL4I1/FXYD5 51

BP GO:0031640 killing of cells of another organism 29/2475 92/18870 3.57778564317464e-06 8.41392609129703e-05 6.11152140612286e-05 GBP5/PLA2G2A/NKG7/HAMP/GBP2/CHGA/S100A12/GZMB/MICA/GBP1/CLEC7A/C7/MICB/APOL1/GZMA/F2RL1/SCNN1B/H2BC11/LYZ/H2BC12/GBP3/LTF/NOS2/PRF1/CXCL6/GNLY/MYD88/TREM1/GZMH 29

BP GO:0141061 disruption of cell in another organism 29/2475 92/18870 3.57778564317464e-06 8.41392609129703e-05 6.11152140612286e-05 GBP5/PLA2G2A/NKG7/HAMP/GBP2/CHGA/S100A12/GZMB/MICA/GBP1/CLEC7A/C7/MICB/APOL1/GZMA/F2RL1/SCNN1B/H2BC11/LYZ/H2BC12/GBP3/LTF/NOS2/PRF1/CXCL6/GNLY/MYD88/TREM1/GZMH 29

BP GO:0001912 positive regulation of leukocyte mediated cytotoxicity 24/2475 69/18870 3.58797665431538e-06 8.41392609129703e-05 6.11152140612286e-05 KLRC2/MR1/KLRC3/SPI1/KLRC4/HLA-C/MICA/KLRC4-KLRK1/HLA-DRB1/MICB/F2RL1/HLA-DRA/NOS2/NECTIN2/TYROBP/HLA-B/HLA-A/ULBP2/ULBP3/PTPRC/B2M/FADD/HFE/HLA-F 24

BP GO:0002711 positive regulation of T cell mediated immunity 24/2475 69/18870 3.58797665431538e-06 8.41392609129703e-05 6.11152140612286e-05 IL18/MR1/HLA-C/IL1R1/MICA/ARID5A/HLA-DRB1/MICB/SASH3/FZD5/HLA-DRA/NECTIN2/HLA-B/ZP3/HLA-A/RSAD2/ULBP2/ULBP3/PTPRC/B2M/IL6/FADD/HFE/HLA-F 24

BP GO:0030099 myeloid cell differentiation 90/2475 430/18870 3.59979075909986e-06 8.41392609129703e-05 6.11152140612286e-05 LRRC17/H4C9/BATF/IRF7/HOXA5/LEF1/SPI1/RELB/MIR221/TSPAN2/ROR2/TREM2/TFRC/LILRB3/CD74/SOCS1/OSCAR/FLNA/H4C5/SBNO2/RUNX1/STAT1/BMP2/CCN4/MMP9/LOX/JAG1/BATF2/HOXA9/RAB7B/SCIN/HLA-DRB1/SNX10/CASP8/BATF3/VEGFA/TLR2/ZFP36/IFI16/F2RL1/CD101/CLEC5A/IL11/FAM20C/FES/DHRS2/LYN/FSTL3/ETV2/CSF3/H4C8/LIF/CEBPB/LTF/ACTN1/ISG15/TYROBP/EVI2B/TNFRSF11B/LILRB1/TNFAIP6/H4C11/CASP3/LTBR/HOXA7/EPHA2/HOXB8/POU4F1/ERFE/FOSL2/H4C3/HMGB2/B2M/PPARG/C1QC/DYRK3/CD109/FADD/ANXA2/TCIRG1/MYD88/MEIS1/HCLS1/CCR1/UCP2/GPR183/CDK6/HOXB7/CAMK4/SLC1A5 90

BP GO:0007059 chromosome segregation 89/2475 424/18870 3.60156396646125e-06 8.41392609129703e-05 6.11152140612286e-05 CENPI/STIL/NEK2/CENPF/SPC24/KIFC1/MAD2L1/CDCA2/ESCO2/SGO1/KIF14/BCL7A/FANCD2/TOP2A/MAEL/KIF18B/ECT2/CDCA5/ACTL6B/CENPW/KNTC1/KIF4A/MYBL2/PSRC1/SKA1/CHEK2/KIF23/UBE2C/FLNA/CENPH/CENPM/TACC3/MAP3K20/CCNB1/FAM83D/HAUS1/RMI2/NEK6/ANKRD53/DLGAP5/BIRC5/BRCA1/SGO2/PLSCR1/DSN1/HJURP/HASPIN/ESPL1/SYCE1L/TTK/CENPN/ZWILCH/CCNB2/MKI67/NUF2/AURKA/TRIP13/BUB1B/KIF18A/BUB1/AURKB/KIF2C/OIP5/NCAPG/BRIP1/ITGB3BP/SMC4/CDT1/CENPS/NDE1/PTTG1/PLK1/CDC6/CENPU/CENPE/CDCA8/NDC80/CDK1/NCAPH/TPX2/KNL1/ZWINT/CHMP4BP1/CENPL/CENPK/SKA3/ASPM/SPAG5/CDC20 89

BP GO:0002483 antigen processing and presentation of endogenous peptide antigen 13/2475 25/18870 3.67100322966265e-06 8.5429082910444e-05 6.20520864157175e-05 HLA-C/MICA/HLA-DRB1/MICB/HLA-DRA/HLA-B/HLA-A/ULBP2/TAP1/ULBP3/B2M/HFE/HLA-F 13

BP GO:0044784 metaphase/anaphase transition of cell cycle 30/2475 97/18870 3.7865511396526e-06 8.72640133730732e-05 6.33849025920689e-05 CENPF/SPC24/MAD2L1/BCL7A/ACTL6B/KNTC1/UBE2C/TACC3/MAP3K20/CCNB1/NEK6/DLGAP5/BIRC5/HASPIN/ESPL1/TTK/ZWILCH/NUF2/TRIP13/BUB1B/BUB1/AURKB/PLK1/CDC6/CENPE/CDCA8/NDC80/KNL1/ZWINT/CDC20 30

BP GO:0051952 regulation of amine transport 30/2475 97/18870 3.7865511396526e-06 8.72640133730732e-05 6.33849025920689e-05 STX1A/SLC17A8/CHGA/GABBR1/CHRNB2/KCNB1/CRH/STXBP1/CNR1/PRKCB/OXTR/SNCG/SYT7/HTR2A/VIP/SYT1/SYT4/GRM2/P2RY1/HRH3/SYT5/ITGB1/TRH/ADORA1/CHRNA4/GDNF/SYT2/RGS4/SYT13/ADRA2B 30

BP GO:0071248 cellular response to metal ion 50/2475 200/18870 3.79345561132114e-06 8.72640133730732e-05 6.33849025920689e-05 KCNA1/MT1M/SLC11A1/NCF1/TSPO/IQGAP1/MT1A/ECT2/MT2A/HMOX1/MT1DP/CLDN1/KCNB1/SLC39A8/EGFR/NEUROD2/MT1E/MMP9/ITPKC/CPNE9/CLIC4/SMPD3/SYT7/KCNH1/DPEP1/KCNK3/SYT1/SYT4/ALOX5AP/DLG2/LRRK2/MT1H/RASAL1/ADGRV1/SLC25A24/NPTX1/FBP1/SYT5/RYR3/HPCA/B2M/CPNE8/SYT2/TUBA1A/SYT13/ANK3/HFE/AQP1/UCP2/MT3 50

BP GO:0050792 regulation of viral process 44/2475 168/18870 3.89684581210036e-06 8.93002376177501e-05 6.48639300905254e-05 APOBEC3B/OASL/TOP2A/CXCL8/OAS2/KPNA2/FAM111A/APOBEC3G/CD74/TRIM21/MX1/APOBEC3C/STAT1/TRIM38/TRIM22/OAS1/CIITA/IFITM3/HLA-DRB1/ZFP36/PLSCR1/IFI16/CCL5/APOBEC3F/CXCR4/IFITM2/IFIH1/OAS3/SLPI/LTF/PPIA/LAMP3/ISG15/NECTIN2/LGALS1/MDFIC/TRIM6/TRIM5/RSAD2/BTBD17/PTX3/ISG20/APOBEC3H/BST2 44

BP GO:0043266 regulation of potassium ion transport 31/2475 102/18870 3.95117815244645e-06 9.0201040407941e-05 6.55182352835126e-05 CAV1/AKAP6/KCNC1/RGS7/KCNIP3/ABCC8/TREM2/FLNA/KCNMB1/KCNAB1/CD63/KCNS2/HTR2A/KCNAB2/VIP/KCNN4/KCNE5/KCNE3/DPP10/KCNC2/GCK/LRRC55/ITGB1/ADORA1/FXYD2/RGS4/ANK3/GAL/KCNE4/GRP/KCNIP2 31

BP GO:0050727 regulation of inflammatory response 89/2475 425/18870 3.9803261670649e-06 9.05222663146124e-05 6.57515602478066e-05 PDE2A/TRADD/MDK/PYCARD/IL18/PLA2G2A/TMSB4X/NFKBIZ/NKG7/NCF1/MIR221/GSDMD/FANCD2/NEAT1/HAMP/ANXA1/TREM2/FCGR1A/ELF4/S100A12/VAMP8/SLC39A8/CTSC/AHR/SBNO2/IL1R1/LILRA5/CNR1/CCN4/MMP9/BIRC3/S100A8/GPR4/S100A9/TNFRSF1A/TAFA3/PTGER4/CASP1/SLAMF8/HLA-DRB1/TNFAIP3/TLR2/PBK/ZFP36/CARD16/IDO1/NMI/GPX1/FCGR2B/CCL5/SPHK1/BST1/HGF/DUSP10/ACP5/LYN/FPR2/IL10RA/SOCS3/ALOX5AP/PTGES/LRRK2/SERPINE1/SPN/CEBPB/MGST2/TNC/ZP3/TNFAIP6/LGALS1/CST7/IFI35/OSM/OSMR/LRFN5/ALOX5/C3/PTPRC/ADORA1/IL6/PPARG/PIK3CG/LBP/IL2RA/CASP4/SAA1/MYD88/SIGLEC10/FANCA 89

BP GO:0048144 fibroblast proliferation 32/2475 107/18870 4.07254546827845e-06 9.22700490246937e-05 6.70210759685308e-05 CAV1/NGFR/ITGB3/FTH1/CKS2/CD248/FBXO4/SOD2/PAWR/COL3A1/CD74/EGFR/E2F8/CCNB1/CKS1B/GPX1/SPHK1/FN1/PDGFA/LIF/CCNA2/CD300A/PDGFD/E2F1/S100A6/CDC6/FOSL2/CDK4/CDK1/AQP1/WNT7B/CDK6 32

BP GO:0071805 potassium ion transmembrane transport 53/2475 217/18870 4.14401290724026e-06 9.35362913348516e-05 6.79408210316438e-05 CAV1/KCNA1/AKAP6/KCNC1/RGS7/HCN1/KCNN1/KCNIP3/ABCC8/TREM2/KCNB1/FLNA/KCNMB1/KCNV1/KCNH3/SLC17A7/KCNAB1/KCNK7/CD63/KCNQ5/KCNS2/NALCN/KCNAB2/KCNH1/KCNA4/KCNK3/KCNN4/KCNK12/KCNE5/SLC12A5/KCNJ11/SNAP25/KCNE3/SLC17A6/KCNJ3/DPP10/SLC12A7/KCNH7/KCNJ12/KCNC2/LRRC55/ITGB1/FXYD2/KCNJ9/RGS4/KCNT1/ANK3/GAL/KCNE4/AQP1/GRP/KCNJ4/KCNIP2 53

BP GO:0060326 cell chemotaxis 72/2475 325/18870 4.25064315037245e-06 9.55837508420831e-05 6.94280093514412e-05 CXCL14/RARRES2/MDK/CXCL13/TMSB4X/LEF1/SPI1/CCL26/ITGB2/CXCL8/CCL20/CHGA/ANXA1/THBS1/NRP1/MMP2/VAV3/S100A12/CD74/PPBP/LGALS3/HSPB1/ADGRE2/CCL2/IL17RC/LOX/S100A8/KLRC4-KLRK1/MET/S100A9/SLAMF8/HSD3B7/VEGFA/CXCL10/C5AR1/F2RL1/CCL5/CXCR4/DPEP1/BST1/HGF/LYN/FPR2/SERPINE1/CCL18/PPIA/CXCL9/CCR5/THBS4/TNFAIP6/RAC2/PDGFD/CXCR2/EPHA2/FCER1G/CCRL2/ALOX5/HMGB2/DPP4/CXCL6/CXCL11/IL6/PIK3CG/LBP/ITGA1/TGFB2/SWAP70/SAA1/TREM1/PTN/CCR1/GPR183 72

BP GO:0050672 negative regulation of lymphocyte proliferation 28/2475 88/18870 4.27231503568515e-06 9.57126099785583e-05 6.95216071989142e-05 PLA2G2A/LRRC32/PAWR/FOXJ1/HAVCR2/TWSG1/PLA2G5/CD274/GPNMB/HLA-DRB1/IDO1/PDCD1LG2/FCGR2B/VSIG4/LYN/BTN2A2/SPN/CEBPB/LILRB2/CD300A/TYROBP/LILRB1/SDC4/CASP3/IL2RA/TNFRSF14/GAL/IL4I1 28

BP GO:1902099 regulation of metaphase/anaphase transition of cell cycle 29/2475 93/18870 4.55336701494019e-06 0.000101581942145253 7.37848427903825e-05 CENPF/SPC24/MAD2L1/BCL7A/ACTL6B/KNTC1/UBE2C/MAP3K20/CCNB1/NEK6/DLGAP5/BIRC5/HASPIN/ESPL1/TTK/ZWILCH/NUF2/TRIP13/BUB1B/BUB1/AURKB/PLK1/CDC6/CENPE/CDCA8/NDC80/KNL1/ZWINT/CDC20 29

BP GO:0045766 positive regulation of angiogenesis 47/2475 185/18870 4.58506101288535e-06 0.000101581942145253 7.37848427903825e-05 ITGA5/MDK/ITGB3/EMP2/IL1A/CXCL8/HMOX1/THBS1/CD40/NRP1/F3/MIR210/APLNR/HSPB1/RUNX1/CYP1B1/NR2E1/PRKCB/ECM1/ENG/ANGPT2/ANGPTL4/BRCA1/VEGFA/C5AR1/SFRP2/CXCR4/SPHK1/CHI3L1/EMILIN2/HK2/MIR27B/SERPINE1/EMILIN1/HSPB6/TWIST1/CXCR2/RRAS/GATA4/C3/ITGB1/ZC3H12A/PIK3R6/ADAM12/AQP1/ADM/NODAL 47

BP GO:1904018 positive regulation of vasculature development 47/2475 185/18870 4.58506101288535e-06 0.000101581942145253 7.37848427903825e-05 ITGA5/MDK/ITGB3/EMP2/IL1A/CXCL8/HMOX1/THBS1/CD40/NRP1/F3/MIR210/APLNR/HSPB1/RUNX1/CYP1B1/NR2E1/PRKCB/ECM1/ENG/ANGPT2/ANGPTL4/BRCA1/VEGFA/C5AR1/SFRP2/CXCR4/SPHK1/CHI3L1/EMILIN2/HK2/MIR27B/SERPINE1/EMILIN1/HSPB6/TWIST1/CXCR2/RRAS/GATA4/C3/ITGB1/ZC3H12A/PIK3R6/ADAM12/AQP1/ADM/NODAL 47

BP GO:0007389 pattern specification process 97/2475 475/18870 4.63883535125752e-06 0.000102395468562317 7.4375754103173e-05 LEFTY2/STIL/HOXD3/SP8/DDIT3/EFNB1/HAND2/EMX1/HOXD11/MEOX2/HOXB2/HOXA6/SNAI1/HOXA5/LEF1/NKX2-5/HOXC6/HOXB3/STC1/RIPPLY2/HOXA3/HOXC11/DRC1/HOXA11/MEIS3P1/HOXA2/DLL3/NRP1/FOXJ1/NRG3/NKX3-2/APLNR/HOXD9/HOXB4/DLX2/HOXD4/BMP2/SHROOM3/ENG/DSCAML1/FOXD1/GLI3/HOXA9/FEZF2/HOXC9/GSX2/HOXC4/GSC/FOLR1/DKK1/SFRP2/TBX5/FZD5/DMRTA2/PCSK5/PCSK6/OSR1/CCDC40/HOXD10/ARC/AURKA/NKD1/GLI1/OTX1/NR2F2/CRB2/SEMA3F/HOXA10/HOXC10/BARX1/CFAP45/HOXA7/HOXD8/TBR1/FST/HOXB8/DAAM2/GATA4/TBX1/GREM2/C3/ZIC1/HOXC13/GDNF/C1QA/SPRY1/HOXC8/XRCC2/EYA1/NOG/MEIS1/EN1/WNT7B/HOXD13/DLL4/NODAL/HOXB7 97

BP GO:0071241 cellular response to inorganic substance 55/2475 229/18870 4.73476674573174e-06 0.000104130181470232 7.56357764702779e-05 KCNA1/MT1M/SLC11A1/NCF1/TSPO/IQGAP1/MT1A/ECT2/MT2A/HMOX1/MT1DP/CLDN1/KCNB1/SLC39A8/EGFR/NEUROD2/MT1E/MMP9/ITPKC/CPNE9/TIMELESS/CLIC4/SMPD3/SYT7/KCNH1/DPEP1/KCNK3/SYT1/SYT4/ALOX5AP/DLG2/LRRK2/RAD51/MT1H/RASAL1/CDK2/CCNA2/ADGRV1/SLC25A24/KCNC2/NPTX1/FBP1/SYT5/RYR3/HPCA/B2M/CPNE8/SYT2/TUBA1A/SYT13/ANK3/HFE/AQP1/UCP2/MT3 55

BP GO:0019731 antibacterial humoral response 24/2475 70/18870 4.78571919755495e-06 0.000104517215785177 7.59169018896152e-05 IGKV3-20/PI3/IGHG4/KLK7/WFDC2/H2BC8/IGHA1/SPON2/IGHG2/IGHG3/H2BC11/IGHA2/JCHAIN/H2BC12/SLPI/ANG/LTF/IGHG1/RNASE3/HLA-A/IGHM/H2BC4/RNASE6/NCR3LG1 24

BP GO:0070374 positive regulation of ERK1 and ERK2 cascade 53/2475 218/18870 4.78718093619647e-06 0.000104517215785177 7.59169018896152e-05 HAND2/ITGB3/PYCARD/PLA2G2A/NECAB2/IL1A/MIR221/CCL26/NPNT/CCL20/SPRY2/TREM2/ICAM1/NRP1/SHC1/CD74/HAVCR2/NOX4/EGFR/CD44/P2RY6/CCL2/BMP2/PLA2G5/ACTA2/GPNMB/HLA-DRB1/ANGPT1/C5AR1/F2RL1/HTR2A/CCL5/SCIMP/CHI3L1/HTR2C/FPR2/MIR27B/PDGFA/CCL18/CD36/RAP1B/P2RY1/PDGFD/GATA4/PTPRC/CAVIN3/NTRK1/MARCO/RAMP3/CCR1/GPR183/NODAL/MT3 53

BP GO:0045956 positive regulation of calcium ion-dependent exocytosis 9/2475 13/18870 4.92061473278501e-06 0.000107041198752323 7.77502167731819e-05 CDK5R2/STX1A/KCNB1/STXBP1/SYT7/SCAMP5/SYT1/SYT4/ZP3 9

BP GO:0031099 regeneration 48/2475 191/18870 5.03732486168669e-06 0.000109184471009267 7.93069994374613e-05 MDK/LGR6/MIR221/TSPO/SPP1/LCP1/ACE/ANXA1/NEFL/CLDN1/HOPX/FZD7/MMP2/FLNA/CPQ/RUNX1/RTN4R/HSPG2/GAP43/TYMS/ANGPT2/FOLR1/GPX1/SELENON/RTN4RL1/MYOD1/DUSP10/CEBPB/RGN/CCNA2/AURKA/TNC/TNR/PCNA/GLI1/NNMT/ELAVL4/POSTN/GATA4/CDK1/WNT10B/PTGFRN/IL6/HFE/ADM/EZH2/PTN/UCP2 48

BP GO:0009612 response to mechanical stimulus 52/2475 213/18870 5.15466020266756e-06 0.000110597369519704 8.03332693853453e-05 KCNA1/PDE2A/ANO3/KCNC1/CHRNA9/MDK/ITGB3/FOSL1/ITGA2/FAS/THBS1/CD40/MMP2/ATP8A2/STAT1/ENG/ANGPT2/TNFRSF1A/PTGER4/CASP1/MMP14/NRXN1/PSPH/CASP8/PHF24/CXCL10/HTR2A/RYR2/CXCR4/CHI3L1/TLR8/COL1A1/ASIC2/F11R/TNC/IGFBP2/P2RY1/ADGRV1/STRA6/CNTNAP2/LTBR/POSTN/GADD45A/GATA4/NTRK1/TUBA1A/FADD/IRF1/MYD88/AQP1/PTN/CHEK1 52

BP GO:0045637 regulation of myeloid cell differentiation 52/2475 213/18870 5.15466020266756e-06 0.000110597369519704 8.03332693853453e-05 LRRC17/H4C9/IRF7/HOXA5/LEF1/SPI1/MIR221/ROR2/TREM2/LILRB3/CD74/H4C5/RUNX1/STAT1/LOX/JAG1/HOXA9/RAB7B/SCIN/HLA-DRB1/CASP8/ZFP36/CD101/FES/LYN/FSTL3/CSF3/H4C8/LIF/CEBPB/LTF/ISG15/TYROBP/EVI2B/TNFRSF11B/LILRB1/TNFAIP6/H4C11/HOXA7/HOXB8/POU4F1/ERFE/H4C3/HMGB2/B2M/C1QC/FADD/MEIS1/HCLS1/CCR1/CDK6/CAMK4 52

BP GO:0050830 defense response to Gram-positive bacterium 34/2475 118/18870 5.15777206287759e-06 0.000110597369519704 8.03332693853453e-05 RARRES2/IL7R/PYCARD/IL18/MR1/PLA2G2A/GSDMD/GBP2/CHGA/HAVCR2/H2BC8/TNFSF8/SSC5D/GBP4/KLRC4-KLRK1/C5AR1/TLR2/H2BC11/ACP5/LYZ/H2BC12/ANG/CD36/RNASE3/HLA-A/EPHA2/H2BC4/RNASE6/HMGB2/IL6/LBP/CASP4/TNFRSF14/MYD88 34

BP GO:0032026 response to magnesium ion 11/2475 19/18870 5.29898023474701e-06 0.000113220915763064 8.22389028378231e-05 KCNA1/MDM2/THBS1/SMPD3/CNGA3/CD14/TNFRSF11B/KCNC2/FBP1/RYR3/ANK3 11

BP GO:0009410 response to xenobiotic stimulus 90/2475 434/18870 5.33443560619536e-06 0.00011357429567233 8.24955831148465e-05 CENPF/MDM2/KCNC1/EMX1/ITGB3/NFKBIZ/FOSL1/TSPO/LCK/CA9/DUSP6/ITGA2/ACE/SOD2/ABCC8/THBS1/ITGA3/TFRC/TGIF1/RET/MMP2/VAV3/S100A12/RAD54L/AHR/PLIN2/SHANK2/CYP1B1/STAT1/HSPG2/CBR1/ALDH3A1/PRKCB/LOX/TYMS/ENG/OXTR/GPX1/GRIN2A/HTR2A/SFRP2/CXCR4/CRYZ/SCNN1B/DPEP1/COL1A1/LYN/ASIC2/KCNK3/SLC12A5/GRIN1/CBR3/VKORC1/KCNJ11/SLCO1A2/NOS2/GRM2/ADCYAP1R1/SOX10/ADSS1/CYP2E1/PCNA/IGFBP2/TNFRSF11B/CD69/GAD2/CASP3/NKX6-1/NAT2/CYP2S1/E2F1/NNMT/RAP2A/NAT1/FBP1/TP53I13/AOX1/FOSL2/GATA4/CDK4/C1orf115/FZD1/CDK1/NTRK1/ABCC3/SLITRK5/GAL/SLC29A1/MAOB/TP73 90

BP GO:0015844 monoamine transport 28/2475 89/18870 5.46020510309164e-06 0.000115433350137191 8.3845921949995e-05 HTR1A/STX1A/ITGB3/CHGA/GABBR1/CHRNB2/KCNB1/CRH/CNR1/PRKCB/OXTR/SNCG/SYT7/HTR2A/VIP/SYT1/SYT4/GRM2/P2RY1/LILRB1/HRH3/SYT5/CHRNA4/GDNF/SYT2/SYT13/ADRA2B/MAOB 28

BP GO:0032945 negative regulation of mononuclear cell proliferation 28/2475 89/18870 5.46020510309164e-06 0.000115433350137191 8.3845921949995e-05 PLA2G2A/LRRC32/PAWR/FOXJ1/HAVCR2/TWSG1/PLA2G5/CD274/GPNMB/HLA-DRB1/IDO1/PDCD1LG2/FCGR2B/VSIG4/LYN/BTN2A2/SPN/CEBPB/LILRB2/CD300A/TYROBP/LILRB1/SDC4/CASP3/IL2RA/TNFRSF14/GAL/IL4I1 28

BP GO:0051047 positive regulation of secretion 69/2475 310/18870 5.63744797381661e-06 0.00011876223731507 8.62638853389555e-05 KLRC2/CDK5R2/STX1A/IL1A/ITGB2/STX1B/CYBA/SPP1/ABCC8/C1QTNF1/TREM2/GABBR1/CHRNB2/VAMP8/KCNB1/CRH/STXBP1/CD33/GLUD1/RAB27A/BMP2/RAB3A/S100A10/PRKCB/S100A8/RAPGEF4/NNAT/OXTR/SCIN/HLA-DRB1/SMPD3/SYT7/TLR2/F2RL1/VIP/GPR27/SCAMP5/KCNN4/SYT1/SYT4/PTGES/SNAP25/ANG/PPIA/P2RY1/ZP3/SDC4/F2RL2/NKX6-1/SDC1/ADAM9/VSNL1/GCK/TUNAR/TRH/ADORA1/PRKCE/GDNF/PPARG/PLA2G4A/TGFB2/GAL/HFE/ANXA2/AQP1/GPR158/GRP/PLCB1/HLA-F 69

BP GO:0051588 regulation of neurotransmitter transport 30/2475 99/18870 6.00467695927845e-06 0.000126056225396881 9.1561930961688e-05 STX1A/SYN1/ITGB3/SYNGR3/STX1B/SLC17A8/PRKCG/CPLX2/RIMS1/STXBP1/ASIC1/RAB3A/MYOF/PRKCB/SNCG/SYT1/SYT4/LRRK2/CAMK2A/RAP1B/PPFIA2/SV2B/P2RY1/SYP/ITGB1/GDNF/RIMS3/CPLX1/GPR158/RIMS2 30

BP GO:0032412 regulation of monoatomic ion transmembrane transporter activity 55/2475 231/18870 6.24329742072368e-06 0.000130608911895557 9.48688106145541e-05 CAV1/VMP1/AKAP6/TMSB4X/STAC/SLN/JPH4/CACNG2/PRRT1/FGF13/TREM2/CABP4/FGF12/NIPSNAP2/CRH/TCAF2/P2RY6/CCL2/MMP9/SHANK1/SHISA7/WNK2/KCNAB1/KCNS2/GLRX/PIRT/SELENON/CNIH3/SCN2B/RYR2/GRM5/HECW1/KCNE5/OSR1/KCNE3/ARC/CABP1/SCN3B/LRRC55/HPCA/JPH3/ITGB1/MAPK8IP2/FXYD2/PRKCE/FKBP1C/ANK3/GAL/KCNE4/GRP/CACNG5/CACNG3/FXYD5/CTSS/KCNIP2 55

BP GO:0099504 synaptic vesicle cycle 49/2475 198/18870 6.38523356172022e-06 0.000133114383001973 9.6688679262452e-05 UNC13A/SNAP91/STX1A/SYN1/CDK5R1/DNM3/SLC32A1/STX1B/NAPB/SLC17A8/PRKCG/CPLX2/RIMS1/DOC2A/STXBP1/RAB3A/SLC17A7/UNC13C/SH3GL2/PRKCB/RAPGEF4/OPHN1/BRSK2/SNCG/ATP6V1G2/ERC2/SYN2/SYT7/BSN/SYT1/SLC2A4/SYT4/LRRK2/SNAP25/SLC17A6/RAP1B/PPFIA2/SV2B/P2RY1/SNCB/SYP/PACSIN1/SYN3/SYT5/PCLO/SYT2/RIMS3/CPLX1/RIMS2 49

BP GO:0009615 response to virus 90/2475 436/18870 6.4704028397185e-06 0.000134423178718581 9.76393333270943e-05 APOBEC3B/HERC5/OASL/GBP5/IRF7/PYCARD/IFI44/H19/FOSL1/IFI6/PARP9/OAS2/GBP2/IFNGR2/MMP12/TREM2/CGAS/CD40/BCL3/APOBEC3G/VAMP8/TRIM21/LILRA4/MX2/RIPK3/MX1/HSPB1/MICA/MLKL/GBP1/APOBEC3C/STAT1/TRIM38/TRIM22/OAS1/RNASE2/SPON2/IFITM3/RAB7B/ACTA2/CASP1/TNFAIP3/DDX60L/MICB/BATF3/CXCL10/TLR2/PLSCR1/IFI16/F2RL1/NMI/CCL5/APOBEC3F/CXCR4/IFITM2/IFIH1/TLR8/OAS3/GBP3/NLRC5/SPN/DTX3L/ZDHHC12/CXCL9/ISG15/LILRB1/NPC2/TRIM6/TRIM5/RSAD2/FGL2/PRF1/RTP4/ZDHHC1/BTBD17/UNC93B1/RNF135/PTPRC/RNASE6/IFI27/IL6/ZC3H12A/FADD/ISG20/IRF1/MYD88/APOBEC3H/SLFN13/CDK6/BST2 90

BP GO:0044000 movement in host 47/2475 188/18870 7.31005063182071e-06 0.000151343255149833 0.000109929363947852 CAV1/ITGA5/ITGB3/TNFRSF4/SIGLEC1/CXCL8/ITGA2/FUCA2/TFRC/CLDN1/ICAM1/NRP1/VAMP8/CD74/TRIM21/EGFR/TRIM38/TRIM22/CIITA/IFITM3/HLA-DRB1/PLSCR1/CTSB/HTR2A/CAV2/CXCR4/CLEC5A/IFITM2/CTSL/F11R/PPIA/ANPEP/NECTIN2/CCR5/LGALS1/TRIM6/TRIM5/EPHA2/EFNB2/ITGB1/DPP4/CDK1/JPT2/PTX3/CHMP4BP1/TNFRSF14/SLC1A5 47

BP GO:0050764 regulation of phagocytosis 30/2475 100/18870 7.51250741923954e-06 0.0001550003248973 0.000112585705327942 PYCARD/SLC11A1/CYBA/ITGA2/PRKCG/TREM2/FCGR1A/CCL2/CLEC7A/RAB27A/PLA2G5/CD300LF/SYT7/TLR2/PLSCR1/F2RL1/FCGR2B/SPHK1/IL2RG/FPR2/TUB/CD36/C2/CD300A/FCER1G/C3/PTPRC/COLEC11/PTX3/IL2RB 30

BP GO:0002444 myeloid leukocyte mediated immunity 32/2475 110/18870 7.75454278832033e-06 0.000159311504661584 0.000115717164664424 SPI1/ITGB2/CHGA/ACE/FCGR1A/CPLX2/VAMP8/STXBP1/IL13RA2/S100A13/ADGRE2/SPON2/PRAM1/F2RL1/FCGR2B/SCNN1B/FES/LYN/CD300A/IGHG1/TYROBP/RAC2/LAT2/MILR1/C3/CXCL6/IL6/PIK3CG/FCGR3A/MYD88/TREM1/GRP 32

BP GO:0010720 positive regulation of cell development 91/2475 444/18870 7.77452879177949e-06 0.000159311504661584 0.000115717164664424 IL1RAPL1/CUL7/SHOX2/IL7R/MDK/IL18/LEF1/NFKBIZ/SPI1/MIR221/TSPO/ROR2/ACE/ANXA1/HOXA11/ACTL6B/TESPA1/TREM2/NEFL/RET/NRP1/CD74/SOCS1/CDH4/RHOH/RUNX1/DSCAM/HAPLN1/CUX2/CLEC7A/BMP2/SOX8/NR2E1/SPINT1/GLI3/TOX/HLA-DRB1/MMP14/CASP8/GSX2/VEGFA/TLR2/L1CAM/CD101/CXCR4/IL2RG/SASH3/GRM5/MYOD1/DUSP10/FES/FN1/LYN/DMRTA2/F11R/MAPT/BTN2A2/RUNX3/LIF/LILRB2/CHODL/HLA-DRA/AURKA/SOX10/SLITRK1/TYROBP/EVI2B/OTP/NKX6-1/HAPLN3/POU4F1/PTPRC/ITGB1/WNT10B/WDR62/TRPV2/IL6/IL2RA/PIK3R6/FADD/NEURL1/XRCC2/ASPM/HCLS1/ISLR2/IL4I1/RASSF10/PTN/CCR1/CLCF1/TP73 91

BP GO:0032102 negative regulation of response to external stimulus 97/2475 481/18870 8.03322187161274e-06 0.000164052599037969 0.000119160895861409 THBD/WNT4/MMRN1/MDK/SEMA3E/CXCL13/MIR221/PLAT/HAMP/SPP1/MMP12/C1QTNF1/THBS1/TREM2/CGAS/ELF4/NRP1/SEMA3A/SLC39A8/TRIM21/LILRA4/HAVCR2/SEMA3D/AHR/SLIT1/MICA/CCL2/RTN4R/TRIM38/OAS1/KLRC4-KLRK1/ANGPT2/RAB7B/TNFRSF1A/TAFA3/PTGER4/SLAMF8/GRIN3A/HLA-DRB1/TNFAIP3/MICB/PBK/ZFP36/CARD16/IFI16/F2RL1/NMI/GPX1/FCGR2B/RTN4RL1/SEMA6B/VSIG4/HGF/DUSP10/ACP5/LYN/FPR2/IL10RA/OAS3/SOCS3/SERPINE1/PDGFA/NLRC5/SPN/SERPING1/PLAU/LTF/ZDHHC12/ISG15/TNR/HLA-B/LILRB1/TNFAIP6/LILRA2/SEMA3F/PROS1/AURKB/CST7/HLA-A/FGL2/CNTNAP2/LRFN5/FAP/ALOX5/PTPRC/ADORA1/DPP4/AJAP1/PPARG/CD109/IL2RA/TFPI/PLAUR/ANXA2/SAA1/SIGLEC10/HLA-F 97

BP GO:0006801 superoxide metabolic process 24/2475 72/18870 8.33248796869161e-06 0.000168505624935861 0.000122395386252896 NCF1B/NCF4/H19/NCF1/ITGB2/CYBA/SOD2/GCH1/NOX4/CLEC7A/NCF2/F2RL1/BST1/ACP5/FPR2/MAPT/MIR27B/SOD3/NOS2/CD36/TYROBP/FBLN5/NCF1C/MT3 24

BP GO:0050766 positive regulation of phagocytosis 24/2475 72/18870 8.33248796869161e-06 0.000168505624935861 0.000122395386252896 PYCARD/SLC11A1/CYBA/ITGA2/TREM2/FCGR1A/CCL2/CLEC7A/RAB27A/PLA2G5/CD300LF/F2RL1/FCGR2B/IL2RG/FPR2/TUB/CD36/C2/FCER1G/C3/PTPRC/COLEC11/PTX3/IL2RB 24

BP GO:0033273 response to vitamin 27/2475 86/18870 8.3354714533562e-06 0.000168505624935861 0.000122395386252896 F5/MDM2/NFKBIZ/IL1A/TSPO/STC1/ITGA2/SPP1/SOD2/CD40/PRKCB/TYMS/PHEX/CXCL10/FOLR1/GPX1/COL1A1/FES/RUNX2/CYP27B1/TNC/BRIP1/SNAI2/POSTN/MN1/GATA4/LRAT 27

BP GO:0098742 cell-cell adhesion via plasma-membrane adhesion molecules 63/2475 279/18870 8.72971410911063e-06 0.000175883233258726 0.000127754170095533 CLDN7/IL1RAPL1/ITGA5/PCDHB7/CELSR3/MIR221/ITGB2/PCDHGA1/PTPRT/IL1RN/EMB/MPZL2/ITGA3/CLDN1/ICAM1/NTNG2/RET/CDH4/SELL/PCDHGA3/DSCAM/FGFRL1/BMP2/CDH22/CDH9/PCDHGA5/DSG2/CDH6/MPZ/DSCAML1/CADM2/CLSTN2/CDH19/PCDHGB4/L1CAM/PCDH11X/AJUBA/PCDH7/CDHR1/PCDH15/SLITRK1/NECTIN2/DSC2/IGSF9/CBLN1/CNTN6/CRB2/IL1RAP/CDH8/AMIGO2/LRFN5/CNTN4/PCDHGB5/NEXN/ITGB1/CLDN23/PALLD/CELSR1/TGFB2/ITGAL/CDH18/NTNG1/FXYD5 63

BP GO:0002220 innate immune response activating cell surface receptor signaling pathway 28/2475 91/18870 8.78578606932562e-06 0.00017642093498405 0.000128144733973653 KLRC2/LY96/TLR1/KLRC3/CYBA/TREM2/KLRC4/CLEC7A/OAS1/KLRC4-KLRK1/RAB7B/TNFAIP3/TLR2/F2RL1/NMI/SCIMP/LYN/CD14/LTF/PAK3/TYROBP/LILRA2/IFI35/COLEC11/PRKCE/LBP/IRAK2/MYD88 28

BP GO:1903034 regulation of response to wounding 44/2475 173/18870 8.83329298165715e-06 0.000176783636872898 0.000128408185343879 CAV1/THBD/WNT4/FERMT1/MMRN1/MDK/MIR221/PLAT/SPP1/ANXA1/C1QTNF1/THBS1/CLDN1/TNFRSF12A/F3/FLNA/RTN4R/CLEC7A/CCN4/TNFAIP3/F2RL1/RTN4RL1/CXCR4/EMILIN2/SERPINE1/PDGFA/VKORC1/SERPING1/PLAU/CD36/EMILIN1/TNR/PROS1/FAP/ALOX5/ITGB1/AJAP1/PRKCE/CD109/TFPI/PLAUR/ANXA2/SIGLEC10/PTN 44

BP GO:0009954 proximal/distal pattern formation 15/2475 34/18870 8.88499941283384e-06 0.000177227695929084 0.000128730731132613 SP8/HOXC11/HOXA11/HOXD9/DLX2/GLI3/HOXA9/HOXC9/OSR1/HOXD10/GLI1/HOXA10/HOXC10/EN1/NODAL 15

BP GO:0097028 dendritic cell differentiation 18/2475 46/18870 8.98712778102346e-06 0.000178671242375049 0.000129779262449565 BATF/SPI1/RELB/TREM2/BATF2/BATF3/F2RL1/FCGR2B/DHRS2/LYN/CEBPB/LILRB2/HLA-B/LILRB1/TMEM176A/LTBR/TMEM176B/CAMK4 18

BP GO:0070664 negative regulation of leukocyte proliferation 29/2475 96/18870 9.1256080360793e-06 0.000180825579698416 0.000131344082311887 PLA2G2A/LRRC32/PAWR/FOXJ1/HAVCR2/TWSG1/PLA2G5/CD274/GPNMB/HLA-DRB1/TNFAIP3/IDO1/PDCD1LG2/FCGR2B/VSIG4/LYN/BTN2A2/SPN/CEBPB/LILRB2/CD300A/TYROBP/LILRB1/SDC4/CASP3/IL2RA/TNFRSF14/GAL/IL4I1 29

BP GO:0030100 regulation of endocytosis 65/2475 291/18870 9.21271254183711e-06 0.000181951072701283 0.000132161593008418 CAV1/SNAP91/APLN/ITGB3/PYCARD/HIP1R/SLC11A1/NECAB2/STX1B/CYBA/ITGA2/PRKCG/TREM2/FCGR1A/SFRP4/LGALS3/CCL2/CLEC7A/RAB27A/SLC17A7/CD151/PLA2G5/OPHN1/CD63/ANGPT1/CD300LF/VEGFA/SYT7/TLR2/PLSCR1/F2RL1/FCGR2B/DKK1/EHD4/SPHK1/IL2RG/SCAMP5/FPR2/TUB/MIR27B/SYT4/TNK2/LRRK2/SERPINE1/CD14/ARC/CD36/C2/CD300A/LILRB1/APOC1/PACSIN1/EFNB2/FCER1G/HPCA/C3/PTPRC/COLEC11/EPHA3/B2M/CALY/PTX3/HFE/ANXA2/IL2RB 65

BP GO:0050731 positive regulation of peptidyl-tyrosine phosphorylation 43/2475 168/18870 9.36876461757986e-06 0.000184426435291638 0.000133959592098821 ITGA5/ITGB3/IL18/NCF1/MIR221/PARP9/ACE/TREM2/CD40/FCGR1A/HPX/NRP1/CD74/NOX4/CD44/LILRA5/AREG/TNFRSF1A/CTF1/CD3E/ANGPT1/VEGFA/HTR2A/CCL5/GFRA1/EHD4/IGF2/IL11/GRM5/TNFRSF18/HGF/LYN/TNK2/CSF3/LIF/CD36/THBS4/OSM/PTPRC/IL6/TNFRSF14/HCLS1/CLCF1 43

BP GO:0002062 chondrocyte differentiation 33/2475 116/18870 9.5055352172079e-06 0.000186507298836981 0.00013547104370448 SHOX2/MDK/LOXL2/EFEMP1/CYTL1/HOXA11/SERPINH1/NKX3-2/COL3A1/TWSG1/RUNX1/ARID5A/BMP2/CCN4/ECM1/GLI3/SCIN/SMPD3/SFRP2/RUNX2/OSR1/RUNX3/SLC26A2/TGFBI/PRKG2/SNAI2/FOSL2/COL27A1/PTHLH/WNT10B/ADAMTS7/OSR2/COL2A1 33

BP GO:0007093 mitotic cell cycle checkpoint signaling 38/2475 142/18870 1.01247647479487e-05 0.000198010057155323 0.000143826162697588 CENPF/SPC24/MDM2/MAD2L1/PLK3/FANCD2/KNTC1/RBBP8/CHEK2/MAP3K20/CCNB1/DTL/NABP1/ORC1/BIRC5/BRCA1/HASPIN/TTK/MUC1/ZWILCH/NUF2/CDK2/TRIP13/BUB1B/BUB1/AURKB/CLSPN/PLK1/CDC6/CDCA8/NDC80/CDK1/GTSE1/KNL1/ZWINT/CHEK1/EME1/CDC20 38

BP GO:0050806 positive regulation of synaptic transmission 44/2475 174/18870 1.03426190018143e-05 0.000201611781693762 0.000146442303649717 AKAP5/STX1A/CACNG2/CA2/STX1B/ROR2/PRRT1/PRKCG/RIMS1/STXBP1/LAMA2/SHANK2/CCL2/NR2E1/SHISA7/OXTR/CLSTN2/NRXN1/SLC8A3/GRIN2A/NALCN/SYT1/SLC8A2/GRIN1/SNAP25/ZDHHC12/LILRB2/ARC/TNR/TYROBP/RASGRF2/MIR320E/ADORA1/PRKCE/NTRK1/RIMS3/CA7/NOG/GRIN2B/GPR158/PTN/CACNG5/CACNG3/RIMS2 44

BP GO:0002577 regulation of antigen processing and presentation 11/2475 20/18870 1.03760893643192e-05 0.000201611781693762 0.000146442303649717 PYCARD/SLC11A1/THBS1/TREM2/CD74/HLA-DOA/FCGR2B/LILRB2/HLA-DOB/FGL2/HFE 11

BP GO:1903532 positive regulation of secretion by cell 64/2475 288/18870 1.27846103303613e-05 0.00024760903362416 0.00017985276943527 KLRC2/CDK5R2/STX1A/IL1A/ITGB2/STX1B/SPP1/ABCC8/C1QTNF1/TREM2/GABBR1/CHRNB2/VAMP8/KCNB1/CRH/STXBP1/CD33/GLUD1/RAB27A/BMP2/RAB3A/S100A10/PRKCB/RAPGEF4/NNAT/OXTR/HLA-DRB1/SMPD3/SYT7/TLR2/F2RL1/VIP/GPR27/SCAMP5/KCNN4/SYT1/SYT4/PTGES/SNAP25/ANG/PPIA/P2RY1/ZP3/SDC4/F2RL2/NKX6-1/SDC1/ADAM9/VSNL1/GCK/TUNAR/TRH/PRKCE/GDNF/PPARG/PLA2G4A/TGFB2/GAL/HFE/ANXA2/GPR158/GRP/PLCB1/HLA-F 64

BP GO:0061041 regulation of wound healing 36/2475 133/18870 1.30290579575917e-05 0.000251532038512477 0.000182702274896944 CAV1/THBD/WNT4/FERMT1/MMRN1/MIR221/PLAT/ANXA1/C1QTNF1/THBS1/CLDN1/TNFRSF12A/F3/CLEC7A/CCN4/TNFAIP3/F2RL1/CXCR4/EMILIN2/SERPINE1/PDGFA/VKORC1/SERPING1/PLAU/CD36/EMILIN1/PROS1/FAP/ALOX5/ITGB1/AJAP1/PRKCE/CD109/TFPI/PLAUR/ANXA2 36

BP GO:0098813 nuclear chromosome segregation 68/2475 312/18870 1.37430799318232e-05 0.000264466192021366 0.000192097099047042 CENPI/NEK2/CENPF/SPC24/KIFC1/MAD2L1/SGO1/KIF14/BCL7A/FANCD2/TOP2A/MAEL/KIF18B/ECT2/CDCA5/ACTL6B/KNTC1/KIF4A/MYBL2/PSRC1/CHEK2/KIF23/UBE2C/FLNA/TACC3/MAP3K20/CCNB1/FAM83D/RMI2/NEK6/ANKRD53/DLGAP5/BIRC5/DSN1/HASPIN/ESPL1/SYCE1L/TTK/ZWILCH/CCNB2/NUF2/AURKA/TRIP13/BUB1B/KIF18A/BUB1/AURKB/KIF2C/NCAPG/BRIP1/SMC4/CDT1/PTTG1/PLK1/CDC6/CENPE/CDCA8/NDC80/CDK1/NCAPH/TPX2/KNL1/ZWINT/CHMP4BP1/CENPK/ASPM/SPAG5/CDC20 68

BP GO:0030282 bone mineralization 34/2475 123/18870 1.38047441229962e-05 0.000264804101324183 0.00019234254212737 WNT4/ROR2/MGP/MMP13/SBNO2/CCN1/BMP2/LOX/ECM1/PHEX/SNX10/SMPD3/NELL1/ENPP1/FAM20C/SRGN/OSR1/CYP27B1/COL1A2/TENT5A/LTF/ISG15/TWIST1/ADGRV1/SGMS2/ALOX5/ASPN/FOSL2/PTHLH/WNT10B/OSR2/IBSP/PTN/CCR1 34

BP GO:0015837 amine transport 31/2475 108/18870 1.4379714747584e-05 0.000274954800460174 0.000199715582297153 STX1A/SLC17A8/CHGA/GABBR1/CHRNB2/KCNB1/CRH/STXBP1/CNR1/PRKCB/OXTR/SNCG/SYT7/HTR2A/VIP/SYT1/SYT4/GRM2/P2RY1/HRH3/SYT5/ITGB1/TRH/ADORA1/CHRNA4/AQP9/GDNF/SYT2/RGS4/SYT13/ADRA2B 31

BP GO:0050767 regulation of neurogenesis 81/2475 390/18870 1.4585366767417e-05 0.000278001720862132 0.000201928736900279 IL1RAPL1/CUL7/SHOX2/ASCL2/MCF2/MDK/SEMA3E/LEF1/MIR221/TSPO/HOXB3/SPP1/ACE/FGF13/ABCC8/TREM2/NEFL/DLL3/NRP1/SEMA3A/CDH4/SEMA3D/SLIT1/DSCAM/HAPLN1/DLX2/CUX2/RTN4R/BMP2/SOX8/NR2E1/S100A10/SPINT1/GLI3/FEZF2/GSX2/VEGFA/TLR2/BRINP1/L1CAM/CXCR4/SEMA6B/GRM5/DUSP10/FN1/LYN/DMRTA2/MAPT/SYT4/LIF/CHODL/SOX10/SLITRK1/TNR/OTP/SEMA3F/NKX6-1/HAPLN3/DRAXIN/FSTL4/POU4F1/DAAM2/ITGB1/HMGB2/WDR62/TRPV2/B2M/IL6/NEURL1/XRCC2/ANXA2/NOG/ASPM/ISLR2/EZH2/RASSF10/PTN/CLCF1/DLL4/MT3/TP73 81

BP GO:0006816 calcium ion transport 90/2475 445/18870 1.49854337461031e-05 0.00028409405702367 0.000206353953197704 AKAP5/CAV1/VMP1/AKAP6/CHRNA9/DDIT3/ITGB3/STAC/SLN/TSPO/JPH4/STC1/CACNG2/LCK/CYBA/ACE/CACNA1B/CABP4/CHRNB2/ATP2B3/NIPSNAP2/TMEM37/FLNA/MCUB/CD33/APLNR/LGALS3/ASIC1/LILRA5/P2RY6/CCL2/PRKCB/SCN2A/GRIN3A/CXCL10/SLC8A3/SELENON/GRIN2A/HTR2A/NALCN/CCL5/TRPM8/RYR2/CXCR4/CACNA2D2/LYN/HTR2C/KCNN4/SLC8A2/CYP27B1/SCN8A/GRIN1/CAMK2A/CACNA1E/TMEM165/LILRB2/CXCL9/ADCYAP1R1/F2RL3/CCR5/LILRB1/CABP1/LILRA2/GCK/RYR3/P2RX6/HPCA/MCOLN2/JPH3/PTPRC/CACNA2D3/SCN3A/ANXA2P2/ATP2B2/PRKCE/CHRNA4/TRPV2/CXCL11/PIK3CG/FKBP1C/RGS4/RAMP3/ANXA2/CACNA1I/NMUR2/GRIN2B/CCR1/PLCB1/CACNG5/CACNG3 90

BP GO:0030856 regulation of epithelial cell differentiation 43/2475 171/18870 1.50469537197747e-05 0.00028409405702367 0.000206353953197704 MACROH2A2/CAV1/WWTR1/IL1A/SPRY2/PROM1/SFRP4/FOXJ1/STAT1/BMP2/MMP9/ERRFI1/ALOX15B/JAG1/S1PR3/TNFRSF1A/ATOH8/VEGFA/ZFP36/FRZB/F11R/ETV2/OSR1/CYP27B1/SERPINE1/LIF/CEBPB/TMEM100/SFN/SPRED3/NKX6-1/PLAAT4/HOXA7/FST/WNT10B/AJAP1/GDNF/CD109/SPRY1/EZH2/PLCB1/NODAL/TP73 43

BP GO:0051897 positive regulation of phosphatidylinositol 3-kinase/protein kinase B signal transduction 43/2475 171/18870 1.50469537197747e-05 0.00028409405702367 0.000206353953197704 IL18/SEMA3E/H19/NCF1/MIR221/ROR2/SPRY2/C1QTNF1/THBS1/TREM2/RET/F3/NOX4/EGFR/WNT16/IGFBP5/HCST/ENG/NTS/ROR1/ANGPT1/F2RL1/GPX1/CCL5/CHI3L1/IGF2/HGF/FN1/DCN/CSF3/PDGFA/FAM110C/PDGFD/OSM/ERFE/ITGB1/NTRK1/PIK3CG/STOX1/TGFB2/RAMP3/HCLS1/GDF15 43

BP GO:0002758 innate immune response-activating signaling pathway 58/2475 255/18870 1.5533036394856e-05 0.000292352195970895 0.000212352317386202 CAV1/KLRC2/OASL/LY96/GBP5/IRF7/PYCARD/TLR1/KLRC3/NFKBIZ/FOSL1/CYBA/GBP2/TREM2/CGAS/CD40/LILRA4/KLRC4/HAVCR2/CLEC7A/TIFA/OAS1/BIRC3/KLRC4-KLRK1/RAB7B/CASP1/TNFAIP3/CD300LF/TLR2/F2RL1/NMI/SCIMP/IFIH1/TLR8/LYN/OAS3/CD14/LTF/ZDHHC12/SLC15A3/PAK3/CD36/CD300A/TYROBP/LILRA2/IFI35/RSAD2/ALPK1/UNC93B1/RNF135/COLEC11/PRKCE/LBP/IRAK2/IRF1/MYD88/CTSS/COLEC12 58

BP GO:0002708 positive regulation of lymphocyte mediated immunity 35/2475 129/18870 1.615405101308e-05 0.000303090382132914 0.000220152083378916 KLRC2/IL18/MR1/KLRC3/TREM2/CD40/FCGR1A/TFRC/HPX/KLRC4/HLA-C/IL1R1/MICA/ARID5A/KLRC4-KLRK1/HLA-DRB1/MICB/SASH3/FZD5/HLA-DRA/NECTIN2/HLA-B/ZP3/HLA-A/RSAD2/ULBP2/ULBP3/C3/PTPRC/B2M/IL6/FADD/HFE/CLCF1/HLA-F 35

BP GO:0051963 regulation of synapse assembly 32/2475 114/18870 1.73933822425456e-05 0.000325326688424435 0.000236303599379788 IL1RAPL1/LINGO2/ADGRB3/NTNG2/CHRNB2/SLITRK4/SLIT1/NEGR1/CUX2/SRPX2/OXTR/LRRTM3/CLSTN2/NRXN1/CBLN2/TLR2/DKK1/ABI3/ASIC2/SNAP25/SLITRK1/CBLN1/IL1RAP/LZTS1/RAP2A/AMIGO2/LRFN5/ICAM5/LRTM2/FZD1/NTRK1/SLITRK5 32

BP GO:0141060 disruption of anatomical structure in another organism 29/2475 99/18870 1.75760120222081e-05 0.000327721665159433 0.000238043209571782 GBP5/PLA2G2A/NKG7/HAMP/GBP2/CHGA/S100A12/GZMB/MICA/GBP1/CLEC7A/C7/MICB/APOL1/GZMA/F2RL1/SCNN1B/H2BC11/LYZ/H2BC12/GBP3/LTF/NOS2/PRF1/CXCL6/GNLY/MYD88/TREM1/GZMH 29

BP GO:0022898 regulation of transmembrane transporter activity 55/2475 239/18870 1.7913021876134e-05 0.000332971465462256 0.000241856443320265 CAV1/VMP1/AKAP6/TMSB4X/STAC/SLN/JPH4/CACNG2/PRRT1/FGF13/TREM2/CABP4/FGF12/NIPSNAP2/CRH/TCAF2/P2RY6/CCL2/MMP9/SHANK1/SHISA7/WNK2/KCNAB1/KCNS2/GLRX/PIRT/SELENON/CNIH3/SCN2B/RYR2/GRM5/HECW1/KCNE5/OSR1/KCNE3/ARC/CABP1/SCN3B/LRRC55/HPCA/JPH3/ITGB1/MAPK8IP2/FXYD2/PRKCE/FKBP1C/ANK3/GAL/KCNE4/GRP/CACNG5/CACNG3/FXYD5/CTSS/KCNIP2 55

BP GO:0048754 branching morphogenesis of an epithelial tube 40/2475 156/18870 1.8058279951721e-05 0.000333605885631177 0.000242317259400745 WNT4/MDK/HOXD11/SEMA3E/HOXA5/LEF1/NPNT/HS3ST3B1/SPRY2/HOXA11/NRP1/CITED1/CSMD1/BMP2/SOX8/AREG/ENG/FOXD1/TIMELESS/MET/GLI3/CLIC4/GREB1L/MMP14/VEGFA/SFRP2/COL4A1/TACSTD2/TNC/EPHA2/HS3ST3A1/RDH10/GDNF/CTSZ/CELSR1/SPRY1/EYA1/NOG/DLL4/HOXB7 40

BP GO:0072006 nephron development 40/2475 156/18870 1.8058279951721e-05 0.000333605885631177 0.000242317259400745 WNT4/HOXD11/ITGB3/WWTR1/NPNT/IQGAP1/HS3ST3B1/SLC22A6/NID1/PROM1/HOXA11/ITGA3/RET/FOXJ1/CITED1/STAT1/BMP2/SOX8/JAG1/ENPEP/GPR4/ANGPT2/FOXD1/GLI3/ACTA2/GREB1L/ANGPT1/VEGFA/OSR1/LIF/TACSTD2/PDGFD/HS3ST3A1/GDNF/NUP107/EYA1/NOG/AQP1/WNT7B/HOXB7 40

BP GO:0051383 kinetochore organization 11/2475 21/18870 1.92029869979713e-05 0.0003525832842074 0.000256101642145808 CENPF/CENPW/KNTC1/CENPA/CENPH/DLGAP5/NUF2/SMC4/CENPE/NDC80/CENPK 11

BP GO:2001044 regulation of integrin-mediated signaling pathway 11/2475 21/18870 1.92029869979713e-05 0.0003525832842074 0.000256101642145808 TIMP1/EMP2/LOXL3/NID1/FLNA/LAMA2/CD63/SLC2A10/BST1/LAMC1/LAMB1 11

BP GO:0019883 antigen processing and presentation of endogenous antigen 14/2475 32/18870 1.99099860066179e-05 0.000364449865804067 0.000264721027039211 CD74/HLA-C/MICA/HLA-DRB1/MICB/HLA-DRA/HLA-B/HLA-A/ULBP2/TAP1/ULBP3/B2M/HFE/HLA-F 14

BP GO:0006821 chloride transport 34/2475 125/18870 2.00264513626659e-05 0.000365467519700443 0.000265460207952407 GLRA3/ANO3/SLC4A10/TSPO/CA2/SLC17A8/ATP8B1/CLIC3/GABRA3/GABRA5/GABRD/GABRA4/P2RY6/GABRB3/SLC17A7/CLIC4/APOL1/TTYH3/BEST4/SLC12A5/SLC17A6/SLC26A2/SLC12A7/GABRA1/GABRB2/GABRG2/PRKG2/GABRG1/SLC25A27/CLIC1/CA7/NMUR2/UCP2/GABRE 34

BP GO:0001916 positive regulation of T cell mediated cytotoxicity 16/2475 40/18870 2.03241331022351e-05 0.000367893766208777 0.000267222531190253 MR1/HLA-C/MICA/HLA-DRB1/MICB/HLA-DRA/NECTIN2/HLA-B/HLA-A/ULBP2/ULBP3/PTPRC/B2M/FADD/HFE/HLA-F 16

BP GO:0071459 protein localization to chromosome, centromeric region 16/2475 40/18870 2.03241331022351e-05 0.000367893766208777 0.000267222531190253 H4C9/KNTC1/CENPA/H4C5/HASPIN/TTK/H2AC8/H2BC11/ZWILCH/H4C8/BUB1B/H4C11/AURKB/H4C3/CDK1/KNL1 16

BP GO:0003002 regionalization 87/2475 430/18870 2.0343226246055e-05 0.000367893766208777 0.000267222531190253 LEFTY2/STIL/HOXD3/SP8/DDIT3/HAND2/EMX1/HOXD11/MEOX2/HOXB2/HOXA6/SNAI1/HOXA5/LEF1/NKX2-5/HOXC6/HOXB3/RIPPLY2/HOXA3/HOXC11/DRC1/HOXA11/HOXA2/DLL3/NRP1/FOXJ1/NKX3-2/APLNR/HOXD9/HOXB4/DLX2/HOXD4/BMP2/ENG/DSCAML1/FOXD1/GLI3/HOXA9/FEZF2/HOXC9/GSX2/HOXC4/GSC/FOLR1/DKK1/SFRP2/FZD5/DMRTA2/PCSK5/PCSK6/OSR1/CCDC40/HOXD10/ARC/AURKA/NKD1/GLI1/OTX1/NR2F2/CRB2/SEMA3F/HOXA10/HOXC10/BARX1/CFAP45/HOXA7/HOXD8/TBR1/HOXB8/DAAM2/GATA4/TBX1/GREM2/C3/HOXC13/GDNF/C1QA/SPRY1/HOXC8/XRCC2/NOG/EN1/WNT7B/HOXD13/DLL4/NODAL/HOXB7 87

BP GO:0002474 antigen processing and presentation of peptide antigen via MHC class I 15/2475 36/18870 2.05979059144094e-05 0.000371380862192535 0.000269755410789942 MR1/ACE/FCGR1A/HLA-C/MICA/MICB/HLA-B/HLA-A/ULBP2/TAP1/FCER1G/ULBP3/B2M/HFE/HLA-F 15

BP GO:0050673 epithelial cell proliferation 95/2475 480/18870 2.16814088232971e-05 0.000388995183239986 0.00028254971144908 COL8A1/CAV1/FERMT1/MDK/NGFR/APLN/ITGB3/LOXL2/HOXA5/NFKBIZ/NKX2-5/COL8A2/CCL26/NEAT1/HMOX1/SERPINB1/MMP12/THBS1/CLDN1/NRP1/FZD7/F3/EGFR/WNT16/APLNR/IGFBP3/IGFBP5/CCL2/STAT1/HSPG2/AREG/IGFBP4/JAG1/ECM1/DLX5/ITGA4/FABP7/ATOH8/TNFAIP3/MMP14/VEGFA/C5AR1/ZFP36/GPX1/CAV2/SFRP2/LGR5/VIP/IGF2/HAS2/HGF/DUSP10/LAMC1/RUNX2/RPS6KA1/MIR27B/RASGRF1/OSR1/RUNX3/CEBPB/TACSTD2/RGN/ANG/TWIST1/GLI1/LAMB1/BRCA2/THBS4/NR2F2/SFN/NKX6-1/EPHA2/EFNB2/FST/SNAI2/FAP/IQGAP3/ALOX5/TBX1/CDK4/HMGB2/WNT10B/CNMD/B2M/IL6/PPARG/CD109/TGFB2/OSR2/EYA1/NOG/PTN/DLL4/CDK6/NODAL 95

BP GO:0060562 epithelial tube morphogenesis 71/2475 334/18870 2.17044281121578e-05 0.000388995183239986 0.00028254971144908 STIL/WNT4/MDK/HAND2/HOXD11/SEMA3E/HOXA5/LEF1/NKX2-5/NPNT/HS3ST3B1/SPRY2/HOXA11/VASP/RET/NRP1/CITED1/APLNR/ST14/CSMD1/BMP2/SOX8/AREG/TEAD2/SPINT1/ENG/FOXD1/TIMELESS/MET/GLI3/CLIC4/GREB1L/MMP14/VEGFA/CXCL10/FOLR1/SFRP2/LGR5/RYR2/CXCR4/COL4A1/OSR1/CCDC40/CTHRC1/TACSTD2/FZD2/TNC/TWIST1/SDC4/CASP3/CXCR2/EPHA2/EFNB2/HS3ST3A1/GATA4/RDH10/FZD1/GDNF/STOX1/CTSZ/CELSR1/TGFB2/SPRY1/EYA1/NOG/ADM/DLL4/FZD6/NODAL/HOXB7/PFN1 71

BP GO:0048146 positive regulation of fibroblast proliferation 19/2475 53/18870 2.23395591943571e-05 0.000399186647032501 0.000289952361347812 NGFR/ITGB3/CD248/CD74/EGFR/CCNB1/SPHK1/FN1/PDGFA/LIF/CCNA2/PDGFD/E2F1/S100A6/CDC6/FOSL2/CDK4/AQP1/CDK6 19

BP GO:0022617 extracellular matrix disassembly 21/2475 62/18870 2.28474198520206e-05 0.000405845883998614 0.00029478935050427 CTSK/LCP1/KIF9/MMP12/KLK7/MMP13/MMP2/MMP1/MMP9/MMP14/MMP19/PDPN/LAMC1/CST3/MMP7/FAP/DPP4/CARMIL2/IL6/MMP11/CTSS 21

BP GO:1903053 regulation of extracellular matrix organization 21/2475 62/18870 2.28474198520206e-05 0.000405845883998614 0.00029478935050427 EFEMP2/ITGB3/NID1/AEBP1/LAMA2/RUNX1/BMP2/TNFRSF1A/SLC2A10/PDPN/HAS2/COLGALT1/LAMC1/MIR27B/CST3/EMILIN1/LAMB1/FAP/DPP4/CARMIL2/IL6 21

BP GO:1901987 regulation of cell cycle phase transition 91/2475 456/18870 2.30900435816749e-05 0.000408945786620579 0.000297040989159693 STIL/CENPF/SPC24/MDM2/MAD2L1/WEE1/KIF14/MIR221/PARP9/PLK3/BCL7A/FANCD2/FBXO4/SUSD2/E2F7/CDCA5/ANXA1/ACTL6B/KNTC1/RBBP8/RRM2/CHEK2/WDR76/UBE2C/EGFR/CCL2/MAP3K20/CCNB1/CDKN2C/FAM83D/DTL/NEK6/NABP1/ATF5/TIMELESS/DLGAP5/GPNMB/ORC1/BIRC5/BRCA1/CAMSAP3/HASPIN/ESPL1/PKMYT1/TTK/PLK5/MUC1/NPM2/ZWILCH/TCIM/BTN2A2/DTX3L/RAD51/NUF2/CDK2/AURKA/TRIP13/BUB1B/GLI1/BRCA2/CDC25C/BUB1/AURKB/CLSPN/CDC45/E2F1/BRIP1/CDT1/DBF4/MYO16/PLK1/MN1/CDC6/CENPE/CDCA8/NDC80/CDK4/CDK1/WNT10B/CDC25A/STOX1/GTSE1/DYRK3/KNL1/ZWINT/EZH2/PLCB1/CHEK1/CDK6/EME1/CDC20 91

BP GO:0051937 catecholamine transport 24/2475 76/18870 2.33274791879465e-05 0.000411935838365974 0.000299212836766757 STX1A/CHGA/GABBR1/CHRNB2/KCNB1/CRH/CNR1/PRKCB/OXTR/SNCG/SYT7/HTR2A/VIP/SYT1/SYT4/GRM2/P2RY1/HRH3/SYT5/CHRNA4/GDNF/SYT2/SYT13/ADRA2B 24

BP GO:0002428 antigen processing and presentation of peptide antigen via MHC class Ib 10/2475 18/18870 2.36907226883414e-05 0.000417123457539008 0.000302980904762458 HLA-C/MICA/MICB/HLA-B/HLA-A/ULBP2/ULBP3/B2M/HFE/HLA-F 10

BP GO:0002218 activation of innate immune response 61/2475 276/18870 2.40067235874163e-05 0.000421451369645753 0.000306124517767515 CAV1/KLRC2/OASL/LY96/GBP5/IRF7/PYCARD/TLR1/KLRC3/NFKBIZ/FOSL1/CYBA/GBP2/TREM2/CGAS/CD40/LILRA4/KLRC4/HAVCR2/CLEC7A/TIFA/OAS1/BIRC3/KLRC4-KLRK1/RAB7B/CASP1/TNFAIP3/CD300LF/TLR2/IFI16/F2RL1/NMI/SCIMP/IFIH1/TLR8/CASP6/LYN/OAS3/CD14/LTF/ZDHHC12/SLC15A3/PAK3/CD36/CD300A/TYROBP/LILRA2/IFI35/TRIM5/RSAD2/ALPK1/UNC93B1/RNF135/COLEC11/PRKCE/LBP/IRAK2/IRF1/MYD88/CTSS/COLEC12 61

BP GO:0043299 leukocyte degranulation 25/2475 81/18870 2.46552218257818e-05 0.000431574203620974 0.000313477317858567 KLRC2/SPI1/NKG7/ITGB2/CHGA/CPLX2/VAMP8/STXBP1/IL13RA2/S100A13/ADGRE2/RAB27A/PRAM1/F2RL1/FCGR2B/FES/LYN/CD300A/RAC2/LAT2/MILR1/PIK3CG/FCGR3A/GRP/HLA-F 25

BP GO:0008608 attachment of spindle microtubules to kinetochore 18/2475 49/18870 2.49360568373085e-05 0.000433959667394783 0.000315210018236366 NEK2/SPC24/SGO1/ECT2/KNTC1/CCNB1/BIRC5/DSN1/ZWILCH/NUF2/AURKB/KIF2C/CDT1/CENPE/CDCA8/NDC80/KNL1/SPAG5 18

BP GO:0036230 granulocyte activation 18/2475 49/18870 2.49360568373085e-05 0.000433959667394783 0.000315210018236366 IL18/PLA2G2A/SPI1/ITGB2/CXCL8/ANXA1/PRAM1/F2RL1/FCGR2B/CCL5/SCNN1B/CD300A/TYROBP/LILRA2/CXCR2/FCER1G/CXCL6/MYD88 18

BP GO:0015698 inorganic anion transport 42/2475 169/18870 2.54490182425231e-05 0.000441606663376036 0.000320764473923861 GLRA3/ANO3/SLC4A10/SLC11A1/TSPO/CA2/SLC17A8/SLC22A6/ATP8B1/CLIC3/SFRP4/GABRA3/GABRA5/GABRD/GABRA4/P2RY6/GABRB3/SLC17A7/CLIC4/APOL1/ENPP1/SLC34A2/TTYH3/BEST4/SLC12A5/CEBPB/SLC17A6/SLC26A2/SLC12A7/GABRA1/GABRB2/GABRG2/CRY2/SLC37A2/PRKG2/GABRG1/SLC25A27/CLIC1/CA7/NMUR2/UCP2/GABRE 42

BP GO:1901379 regulation of potassium ion transmembrane transport 26/2475 86/18870 2.56274011931866e-05 0.00044342050940603 0.000322081975256703 CAV1/AKAP6/KCNC1/RGS7/KCNIP3/TREM2/FLNA/KCNMB1/KCNAB1/CD63/KCNS2/KCNAB2/KCNN4/KCNE5/KCNE3/DPP10/KCNC2/LRRC55/ITGB1/FXYD2/RGS4/ANK3/GAL/KCNE4/GRP/KCNIP2 26

BP GO:2001046 positive regulation of integrin-mediated signaling pathway 8/2475 12/18870 2.62187548980202e-05 0.000452348863240555 0.00032856715530096 EMP2/LOXL3/NID1/FLNA/LAMA2/CD63/LAMC1/LAMB1 8

BP GO:0001706 endoderm formation 20/2475 58/18870 2.64259842063421e-05 0.000454051498796672 0.000329803878031245 COL8A1/ITGA5/ITGB2/DUSP5/MMP2/DUSP4/COL12A1/MMP9/ITGA4/MMP14/DKK1/FN1/COL4A2/COL6A1/LAMB1/COL5A2/ITGA7/COL5A1/NOG/NODAL 20

BP GO:1901890 positive regulation of cell junction assembly 30/2475 106/18870 2.64686916353823e-05 0.000454051498796672 0.000329803878031245 IL1RAPL1/CAV1/WNT4/LINGO2/ADGRB3/CLDN1/HOPX/NRP1/SLITRK4/CUX2/S100A10/SRPX2/OXTR/LRRTM3/CLSTN2/NRXN1/CBLN2/VEGFA/TBX5/ASIC2/SLITRK1/CBLN1/SDC4/IL1RAP/CNTNAP2/EPHA2/AMIGO2/LRTM2/NTRK1/SLITRK5 30

BP GO:0007188 adenylate cyclase-modulating G protein-coupled receptor signaling pathway 56/2475 248/18870 2.68757170101098e-05 0.000459720241962106 0.000333921414223676 AKAP5/HTR1A/PDE2A/ITGB3/GPR65/SSTR2/TSHR/GABBR2/CHGA/HTR1D/GPR157/ADGRB3/GABBR1/FLNA/ADGRE1/GPR26/ADGRE2/CNR1/HTR5A/RAPGEF4/S1PR3/GPR4/CHRM1/PTGER4/ADGRL2/CXCL10/LGR5/VIP/GRM5/FPR2/ADGRL4/CXCL9/GRM2/ADCYAP1R1/GPR3/P2RY1/ADGRE5/HRH3/ADRA1D/GRM1/RXFP1/NPY2R/GLP1R/ADORA1/PTHLH/CXCL11/CHRM4/MARCO/GNAL/RGS1/RAMP3/VIPR2/ADM/FPR1/ADRA2B/RIMS2 56

BP GO:0060541 respiratory system development 50/2475 214/18870 2.76112952011214e-05 0.000470960842010037 0.000342086112494755 HOXA5/LEF1/LOXL3/ACE/SPRY2/MMP12/ITGA3/HOPX/FOXJ1/COL3A1/CRH/STK40/IGFBP5/FGFRL1/BMP2/RAB3A/ERRFI1/LOX/DLX5/TIMELESS/GLI3/PHEX/MMP14/SMPD3/VEGFA/RCN3/SELENON/PDPN/TBX5/CHI3L1/CCDC40/PDGFA/LIF/TNC/GLI1/ADAMTS2/MSC/STRA6/RXFP1/FOSL2/ALDH1A3/RDH10/CTSZ/CELSR1/SPRY1/PLOD3/NOG/WNT7B/NODAL/TP73 50

BP GO:0042130 negative regulation of T cell proliferation 23/2475 72/18870 2.78802934204287e-05 0.00047420193115086 0.00034444030308882 PLA2G2A/LRRC32/PAWR/FOXJ1/HAVCR2/TWSG1/PLA2G5/CD274/GPNMB/HLA-DRB1/IDO1/PDCD1LG2/VSIG4/BTN2A2/SPN/CEBPB/LILRB2/LILRB1/SDC4/CASP3/IL2RA/TNFRSF14/IL4I1 23

BP GO:0030323 respiratory tube development 46/2475 192/18870 2.92545620777956e-05 0.000496170595240353 0.000360397415070791 HOXA5/LOXL3/ACE/SPRY2/MMP12/ITGA3/HOPX/FOXJ1/COL3A1/CRH/STK40/IGFBP5/BMP2/RAB3A/ERRFI1/LOX/TIMELESS/GLI3/PHEX/MMP14/SMPD3/VEGFA/RCN3/SELENON/PDPN/TBX5/CHI3L1/PCSK5/CCDC40/PDGFA/LIF/TNC/GLI1/ADAMTS2/STRA6/RXFP1/FOSL2/RDH10/CTSZ/CELSR1/SPRY1/PLOD3/NOG/WNT7B/NODAL/TP73 46

BP GO:0032729 positive regulation of type II interferon production 24/2475 77/18870 2.97158173040181e-05 0.000502573991812182 0.000365048572544246 CD276/PYCARD/IL18/SLC11A1/BCL3/HAVCR2/IL1R1/HLA-DPA1/ARID5A/CLEC7A/KLRC4-KLRK1/CD3E/F2RL1/SASH3/TLR8/FZD5/CD14/HLA-DPB1/ISG15/LILRB1/ZP3/HLA-A/CD2/FADD 24

BP GO:0014059 regulation of dopamine secretion 15/2475 37/18870 3.04569476068574e-05 0.000510791936959698 0.000371017741650133 GABBR1/CHRNB2/CNR1/PRKCB/SNCG/SYT7/HTR2A/SYT1/SYT4/GRM2/SYT5/CHRNA4/GDNF/SYT2/SYT13 15

BP GO:0044319 wound healing, spreading of cells 15/2475 37/18870 3.04569476068574e-05 0.000510791936959698 0.000371017741650133 ITGA5/FERMT1/MIR221/MMP12/FLNA/CD44/CCN1/CD151/PDPN/AJUBA/TMEFF2/RHOC/ITGB1/CARMIL2/COL5A1 15

BP GO:0090505 epiboly involved in wound healing 15/2475 37/18870 3.04569476068574e-05 0.000510791936959698 0.000371017741650133 ITGA5/FERMT1/MIR221/MMP12/FLNA/CD44/CCN1/CD151/PDPN/AJUBA/TMEFF2/RHOC/ITGB1/CARMIL2/COL5A1 15

BP GO:0051962 positive regulation of nervous system development 63/2475 290/18870 3.1062254833693e-05 0.000518577956281507 0.000376673178034693 IL1RAPL1/CUL7/SHOX2/MDK/MIR221/TSPO/ACE/LINGO2/ADGRB3/NEFL/NRP1/CDH4/SLITRK4/DSCAM/HAPLN1/CUX2/BMP2/SOX8/NR2E1/SRPX2/SPINT1/OXTR/LRRTM3/GLI3/CLSTN2/NRXN1/CBLN2/GSX2/VEGFA/TLR2/L1CAM/CXCR4/GRM5/FN1/LYN/ASIC2/DMRTA2/MAPT/LIF/CHODL/SOX10/SLITRK1/CBLN1/OTP/IL1RAP/NKX6-1/HAPLN3/AMIGO2/ITGB1/LRTM2/WDR62/TRPV2/NTRK1/IL6/NEURL1/SLITRK5/XRCC2/ASPM/ISLR2/RASSF10/PTN/CLCF1/TP73 63

BP GO:0046928 regulation of neurotransmitter secretion 25/2475 82/18870 3.10939480781716e-05 0.000518577956281507 0.000376673178034693 STX1A/SYN1/STX1B/PRKCG/CPLX2/RIMS1/STXBP1/ASIC1/RAB3A/MYOF/PRKCB/SNCG/SYT1/SYT4/LRRK2/CAMK2A/RAP1B/PPFIA2/SV2B/P2RY1/SYP/RIMS3/CPLX1/GPR158/RIMS2 25

BP GO:0000280 nuclear division 88/2475 441/18870 3.13611392645461e-05 0.000521585263557713 0.000378857559332306 CENPI/NEK2/CENPF/SPC24/CUL7/KIFC1/MAD2L1/WNT4/CDCA2/SGO1/KIF14/IL1A/CKS2/FANCD2/TOP2A/MAEL/KIF18B/CDCA5/KNTC1/KIF4A/MYBL2/PSRC1/CHEK2/KIF23/UBE2C/FLNA/RAD54L/UBE2S/CCDC8/CCNB1/TOM1L1/ANKRD53/DLGAP5/BIRC5/SMPD3/DSN1/HASPIN/ESPL1/CAV2/PKMYT1/SPHK1/SYCE1L/IGF2/TTK/NPM2/ZWILCH/CCNB2/LIF/RAD51/MKI67/NUF2/AURKA/GPR3/TRIP13/BUB1B/KIF18A/BRCA2/CDC25C/BUB1/AURKB/KIF2C/NCAPG/BRIP1/SMC4/CDT1/RAD51AP1/CENPS/REEP4/NDE1/PTTG1/PLK1/CENPE/CDCA8/NDC80/CDK1/NCAPH/TPX2/KNL1/ZWINT/CHMP4BP1/CENPK/ASPM/SPAG5/PLCB1/CHEK1/EME1/CDC20/FANCA 88

BP GO:0002526 acute inflammatory response 30/2475 107/18870 3.22201557108331e-05 0.000533473101440826 0.000387492383896585 IL1A/FCGR1A/TFRC/F3/CNR1/PLSCR1/FCGR2B/SAA2/HP/FN1/ALOX5AP/PTGES/SPN/CEBPB/IGHG1/ZP3/CXCR2/SERPINA1/OSM/OSMR/C3/ADORA1/IL6/PIK3CG/LBP/FCGR3A/SERPINA3/HFE/SAA1/CD163 30

BP GO:0030098 lymphocyte differentiation 86/2475 429/18870 3.22536202236875e-05 0.000533473101440826 0.000387492383896585 ASCL2/WNT4/IL7R/MDK/BATF/IL18/MR1/LEF1/NFKBIZ/SPI1/IL1A/RELB/LOXL3/FANCD2/LCK/ANXA1/ACTL6B/TESPA1/CD3D/BCL3/FZD7/FOXJ1/CD74/SOCS1/RIPK3/TNFSF8/RHOH/RUNX1/HLA-DOA/CD79A/CD8A/NFAM1/ITGA4/GLI3/PTGER4/SLAMF8/TOX/HLA-DRB1/CD3E/MMP14/JAK3/FCGR2B/IL2RG/SASH3/IL11/FZD5/DUSP10/CTSL/HDAC4/RUNX2/SOCS3/CMTM7/BTN2A2/RUNX3/SPN/LILRB2/HLA-DRA/KDELR1/LGALS1/TCF7/RSAD2/FGL2/FCER1G/CD2/IL9/FOSL2/PTPRC/ITGB1/WNT10B/B2M/NTRK1/IL6/IL2RA/ZC3H12A/PIK3R6/FADD/IRF1/TCIRG1/IL4I1/EZH2/CLCF1/DLL4/GPR183/CDK6/CAMK4/FANCA 86

BP GO:0021675 nerve development 28/2475 97/18870 3.27599744857109e-05 0.000540359579154418 0.000392494431157606 KCNA1/HOXD3/KCNC1/NGFR/HOXB2/HOXB3/HOXA3/PRKCG/CHRNB2/RET/ATP8B1/NRP1/SEMA3A/GABRA5/ITGA4/GLI3/SCN2A/ECE1/SCN8A/GABRB2/SEMA3F/KCNC2/NPTX1/POU4F1/TBX1/NTRK1/HOXA1/TCIRG1 28

BP GO:0002286 T cell activation involved in immune response 33/2475 123/18870 3.56624231274886e-05 0.000585593325012567 0.000425350318301319 ASCL2/MDK/BATF/IL18/SLC11A1/LEF1/NFKBIZ/RELB/LOXL3/LCP1/ANXA1/ICAM1/BCL3/CD74/HAVCR2/LGALS3/RAB27A/PTGER4/HLA-DRB1/JAK3/F2RL1/FCGR2B/SOCS3/SPN/HLA-DRA/LILRB1/FGL2/FCER1G/HLA-DMB/IL6/ZC3H12A/ITGAL/GPR183 33

BP GO:0072678 T cell migration 23/2475 73/18870 3.56993740056228e-05 0.000585593325012567 0.000425350318301319 ASCL2/ITGB3/PYCARD/CXCL13/MSN/CCL26/CCL20/ICAM1/RIPK3/CCL2/ECM1/ITGA4/CXCL10/CCL5/F11R/SPN/CXCL11/PIK3CG/FADD/ITGAL/TNFRSF14/MYO1G/GPR183 23

BP GO:0048762 mesenchymal cell differentiation 58/2475 262/18870 3.57949284276502e-05 0.000585593325012567 0.000425350318301319 CUL7/WNT4/MDK/EFNB1/HAND2/LOXL2/TGFB1I1/EMP2/SNAI1/SEMA3E/LEF1/WWTR1/MIR221/LOXL3/SPRY2/RET/NRP1/SEMA3A/SEMA3D/FLNA/WNT16/STAT1/BMP2/SOX8/FAM83D/JAG1/ENG/SP6/GSC/FOLR1/FRZB/PDPN/SFRP2/SEMA6B/TBX5/HAS2/HGF/COL1A1/FN1/OSR1/TMEM100/SOX10/TWIST1/CRB2/SEMA3F/SPRED3/S100A4/VASN/SNAI2/TBX1/RDH10/EPHA3/GDNF/IL6/TGFB2/SPRY1/NOG/EZH2 58

BP GO:0002706 regulation of lymphocyte mediated immunity 45/2475 188/18870 3.63897576035121e-05 0.000592097844583975 0.00043007492726102 KLRC2/IL7R/IL18/MR1/KLRC3/TREM2/CD40/FCGR1A/TFRC/HPX/FOXJ1/KLRC4/HAVCR2/RIPK3/AHR/HLA-C/IL1R1/MICA/ARID5A/KLRC4-KLRK1/HLA-DRB1/MICB/FCGR2B/SASH3/FZD5/SPN/HLA-DRA/NECTIN2/HLA-B/LILRB1/ZP3/HLA-A/RSAD2/ULBP2/ULBP3/C3/PTPRC/B2M/IL6/PIK3R6/FADD/HFE/IL4I1/CLCF1/HLA-F 45

BP GO:0030324 lung development 45/2475 188/18870 3.63897576035121e-05 0.000592097844583975 0.00043007492726102 HOXA5/LOXL3/ACE/SPRY2/MMP12/ITGA3/HOPX/FOXJ1/COL3A1/CRH/STK40/IGFBP5/BMP2/RAB3A/ERRFI1/LOX/TIMELESS/GLI3/PHEX/MMP14/SMPD3/VEGFA/RCN3/SELENON/PDPN/TBX5/CHI3L1/CCDC40/PDGFA/LIF/TNC/GLI1/ADAMTS2/STRA6/RXFP1/FOSL2/RDH10/CTSZ/CELSR1/SPRY1/PLOD3/NOG/WNT7B/NODAL/TP73 45

BP GO:0070665 positive regulation of leukocyte proliferation 41/2475 166/18870 3.68341014860502e-05 0.000597707960330393 0.000434149878966446 CD276/EFNB1/PYCARD/IL18/IL1A/TNFRSF4/ANXA1/CD40/CD70/TFRC/CHRNB2/VAV3/CD74/HAVCR2/HLA-DPA1/CD274/CD3E/PDCD1LG2/CCL5/IGF2/SASH3/BST1/LYN/SPN/HLA-DPB1/LILRB2/IGFBP2/ZP3/RAC2/HLA-A/CSF2RB/HLA-DMB/PTPRC/IL6/FCGR3A/IL2RA/FADD/MYD88/CLCF1/GPR183/BST2 41

BP GO:0050851 antigen receptor-mediated signaling pathway 48/2475 205/18870 3.73176844308211e-05 0.00060392284992628 0.000438664112915 TRAC/CD276/TRDC/NFKBIZ/BTN2A3P/LCK/IGHG4/TESPA1/CD247/PAWR/CD3D/VAV3/IGHA1/LGALS3/LCP2/GBP1/CD79A/CD8A/PRKCB/NFAM1/TRBC1/IGHG2/HLA-DRB1/CD3E/PRAM1/CMTM3/IGHG3/FCGR2B/IGKC/IGHA2/LYN/KCNN4/BTN2A2/HLA-DPB1/TRBC2/HLA-DQB1/CD300A/NECTIN2/LAPTM5/IGHG1/HLA-A/LAT2/THEMIS2/IGHM/FOSL2/PTPRC/ZC3H12A/IGLC3 48

BP GO:0007200 phospholipase C-activating G protein-coupled receptor signaling pathway 31/2475 113/18870 3.81273503212705e-05 0.000615367234755129 0.000446976831864809 LPAR6/GPR65/CHGA/GPR157/CCKBR/FPR3/P2RY6/PRKCB/GPR4/CHRM1/C5AR1/F2RL1/HTR2A/HRH1/VIP/GRM5/GPR27/HTR2C/FPR2/GPR83/GNG13/F2RL3/P2RY1/F2RL2/CXCR2/ADRA1D/GRM1/NMUR2/GRP/FPR1/PLCB1 31

BP GO:0099558 maintenance of synapse structure 12/2475 26/18870 4.09056977912713e-05 0.000657463135164399 0.000477553520282286 ITGB3/ADGRB3/SHANK2/RAB3A/SHANK1/CBLN3/OPHN1/CBLN2/ERC2/BSN/CBLN1/PCLO 12

BP GO:0050671 positive regulation of lymphocyte proliferation 37/2475 145/18870 4.0954565714771e-05 0.000657463135164399 0.000477553520282286 CD276/EFNB1/PYCARD/IL18/IL1A/TNFRSF4/ANXA1/CD40/CD70/TFRC/CHRNB2/VAV3/CD74/HAVCR2/HLA-DPA1/CD274/CD3E/PDCD1LG2/CCL5/IGF2/SASH3/BST1/SPN/HLA-DPB1/LILRB2/IGFBP2/ZP3/HLA-A/HLA-DMB/PTPRC/IL6/FCGR3A/IL2RA/FADD/MYD88/CLCF1/GPR183 37

BP GO:0048285 organelle fission 95/2475 488/18870 4.2222655374018e-05 0.000676012860974944 0.000491027259549632 CENPI/NEK2/CENPF/SPC24/CUL7/KIFC1/MAD2L1/WNT4/CDCA2/SGO1/KIF14/IL1A/MTFP1/CKS2/FANCD2/TOP2A/MAEL/KIF18B/CDCA5/KNTC1/KIF4A/MYBL2/PSRC1/CHEK2/KIF23/UBE2C/FLNA/RAD54L/UBE2S/CCDC8/CCNB1/TOM1L1/ANKRD53/DLGAP5/BIRC5/SMPD3/DSN1/HASPIN/ESPL1/CAV2/PKMYT1/SPHK1/SYCE1L/IGF2/TTK/NPM2/DCN/ZWILCH/MAPT/CCNB2/LRRK2/LIF/RAD51/MKI67/NUF2/AURKA/GPR3/TRIP13/BUB1B/KIF18A/BRCA2/CDC25C/BUB1/AURKB/KIF2C/NCAPG/BRIP1/SMC4/CDT1/RAD51AP1/CENPS/REEP4/NDE1/PTTG1/PLK1/CENPE/CDCA8/MTFR2/NDC80/CDK1/NCAPH/PPARG/TPX2/KNL1/ZWINT/CHMP4BP1/CENPK/ASPM/SPAG5/UCP2/PLCB1/CHEK1/EME1/CDC20/FANCA 95

BP GO:0045576 mast cell activation 22/2475 69/18870 4.27253097455692e-05 0.000682241382213823 0.000495551394949309 CHGA/CPLX2/S100A12/VAMP8/STXBP1/IL13RA2/RHOH/S100A13/LCP2/ADGRE2/CNR1/CD300LF/PLSCR1/FES/LYN/CD300A/NECTIN2/RAC2/LAT2/MILR1/PIK3CG/GRP 22

BP GO:0006898 receptor-mediated endocytosis 57/2475 258/18870 4.41610852660133e-05 0.000699826885997451 0.000508324763962172 CAV1/SNAP91/APLN/ITGB3/HIP1R/NECAB2/ITGB2/DNM3/SIGLEC1/CACNG2/CXCL8/FCGR1A/TFRC/SFRP4/HSPG2/LRP1B/ITGA4/OPHN1/CD63/ANGPT1/VEGFA/FOLR1/FCGR2B/CAV2/DKK1/PDLIM7/STAB1/CTSL/HMMR/MIR27B/TNK2/SERPINE1/CD14/SNAP25/ARC/CD36/LILRB1/SDC1/APOC1/CXCR2/EFNB2/FCER1G/HPCA/C3/ITGB1/MSR1/DPP4/B2M/CALY/MARCO/RAMP3/HFE/ANXA2/ADM/CUBN/CACNG5/CACNG3 57

BP GO:0090504 epiboly 15/2475 38/18870 4.4245457868278e-05 0.000699826885997451 0.000508324763962172 ITGA5/FERMT1/MIR221/MMP12/FLNA/CD44/CCN1/CD151/PDPN/AJUBA/TMEFF2/RHOC/ITGB1/CARMIL2/COL5A1 15

BP GO:0031644 regulation of nervous system process 32/2475 119/18870 4.42928408859146e-05 0.000699826885997451 0.000508324763962172 STX1A/STX1B/ITGA2/TYMP/FGF12/RIMS1/CTSC/CUX2/SHANK1/DLGAP3/NRXN1/SLC8A3/GRIN2A/DLGAP2/HTR2C/SLC8A2/DLGAP1/GRIN1/LRRK2/TMEM100/SOX10/TNR/CBLN1/NCMAP/CST7/CELF4/FABP5/GRM1/ADORA1/RGS4/GRIN2B/RIMS2 32

BP GO:0035710 CD4-positive, alpha-beta T cell activation 32/2475 119/18870 4.42928408859146e-05 0.000699826885997451 0.000508324763962172 ASCL2/BATF/IL18/LEF1/NFKBIZ/NKG7/RELB/LOXL3/ANXA1/BCL3/SOCS1/TWSG1/RUNX1/CD274/PTGER4/TOX/HLA-DRB1/CD3E/JAK3/IL2RG/SASH3/CTSL/SOCS3/RUNX3/SPN/HLA-DRA/RSAD2/IL6/IL2RA/ZC3H12A/TCIRG1/GPR183 32

BP GO:0001890 placenta development 38/2475 151/18870 4.50876604679823e-05 0.000710515258398335 0.000516088347337617 CUL7/ASCL2/SNAI1/RSPO3/LEF1/FOSL1/STC1/E2F7/TTPA/SPP1/EGFR/CITED1/E2F8/ST14/CCN1/SPINT1/CASP8/CTSB/IGF2/FZD5/GJB2/SOCS3/ETV2/CYP27B1/LIF/CEBPB/ANG/NR2F2/PHLDA2/ETNK2/ADAM19/PLK4/PPARG/TPPP3/ADM/PTN/WNT7B/NODAL 38

BP GO:0048771 tissue remodeling 44/2475 184/18870 4.52692311774506e-05 0.000711509068035114 0.00051681020878528 CAV1/TIMP1/MDM2/MDK/HAND2/ITGB3/IL18/RSPO3/IL1A/CTSK/HAMP/HOXA3/SPP1/ACE/TFRC/MMP2/NOX4/FLNA/WNT16/IGFBP5/RUNX1/JAG1/GPNMB/TNFAIP3/MMP14/SNX10/SYT7/ACP5/MIR27B/CST3/LIF/IL20RA/CTHRC1/TMBIM1/TNFRSF11B/THBS4/RAC2/EPHA2/FOSL2/IL6/PPARG/TCIRG1/PTN/DLL4 44

BP GO:1902074 response to salt 75/2475 366/18870 4.76162047515934e-05 0.000746443063521062 0.000542184791221249 CAV1/MDM2/KCNC1/CDK5R1/IL1A/TSPO/CACNG2/IQGAP1/ITGA2/ECT2/SOD2/PRKCG/THBS1/NEFL/CLDN1/CHRNB2/KCNB1/KCNMB1/IMPA2/NEUROD2/CNR1/PRKCB/ITPKC/OXTR/CHRM1/CPNE9/PHEX/CLIC4/SYT7/HTR2A/ENPP1/RYR2/KCNH1/DPEP1/COL1A1/RUNX2/SYT1/S100A16/SYT4/ALOX5AP/DLG2/KCNJ11/RASAL1/CCNA2/GRM2/CD36/PCNA/MTTP/P2RY1/ADGRV1/HRH3/SLC25A24/SDC1/ADAM9/NPTX1/SYT5/RYR3/ELAVL4/P2RX6/HPCA/ASPN/PRKCE/B2M/CPNE8/NTRK1/CHRM4/SYT2/TUBA1A/RGS4/ZC3H12A/SYT13/FADD/PLCB1/EN1/KCNIP2 75

BP GO:0007249 canonical NF-kappaB signal transduction 64/2475 300/18870 4.82156353774683e-05 0.000749960363377486 0.000544739609641454 TRADD/PYCARD/IL1A/RELB/TRAF5/ECT2/LITAF/HMOX1/TNFRSF19/TREM2/CD40/TFRC/BCL3/S100A12/SLC39A8/CD74/TRIM21/FLNA/RIPK3/HSPB1/RHOH/S100A13/CLEC7A/STAT1/TRIM38/TRIM22/TIFA/BIRC3/PRKCB/NEK6/ECM1/SECTM1/ROR1/TNFRSF1A/CASP1/HLA-DRB1/TNFAIP3/CASP8/ANGPT1/TLR2/CARD16/F2RL1/TLR8/AJUBA/S100B/MIR27B/LTF/CD36/LGALS1/TRIM6/TRIM5/S100A4/LTBR/RHOC/CARD19/ALPK1/PRDX4/PRKCE/ZC3H12A/FADD/IRAK2/MYD88/TRAF1/BST2 64

BP GO:0032612 interleukin-1 production 34/2475 130/18870 4.82704786177452e-05 0.000749960363377486 0.000544739609641454 GBP5/PYCARD/GSDMD/PYDC1/ANXA1/SERPINB1/TREM2/ELF4/HAVCR2/CD33/HSPB1/S100A13/LILRA5/CLEC7A/S1PR3/CASP1/TNFAIP3/CASP8/CARD16/IFI16/F2RL1/SPHK1/TLR8/FZD5/ACP5/IL1R2/MIR27B/CD36/TYROBP/LILRA2/IL6/ZC3H12A/SAA1/MYD88 34

BP GO:0032652 regulation of interleukin-1 production 34/2475 130/18870 4.82704786177452e-05 0.000749960363377486 0.000544739609641454 GBP5/PYCARD/GSDMD/PYDC1/ANXA1/SERPINB1/TREM2/ELF4/HAVCR2/CD33/HSPB1/S100A13/LILRA5/CLEC7A/S1PR3/CASP1/TNFAIP3/CASP8/CARD16/IFI16/F2RL1/SPHK1/TLR8/FZD5/ACP5/IL1R2/MIR27B/CD36/TYROBP/LILRA2/IL6/ZC3H12A/SAA1/MYD88 34

BP GO:0061326 renal tubule development 29/2475 104/18870 4.83402166267633e-05 0.000749960363377486 0.000544739609641454 WNT4/HOXD11/WWTR1/NPNT/HS3ST3B1/SLC22A6/PROM1/HOXA11/CITED1/STAT1/BMP2/SOX8/JAG1/FOXD1/GLI3/GREB1L/VEGFA/LGR5/COL4A1/OSR1/LIF/TACSTD2/HS3ST3A1/GDNF/EYA1/NOG/AQP1/WNT7B/HOXB7 29

BP GO:0071674 mononuclear cell migration 48/2475 207/18870 4.84715294866e-05 0.000750059440818419 0.000544811575320087 ASCL2/RARRES2/MDK/ITGB3/PYCARD/CXCL13/SPI1/MSN/CCL26/CCL20/ANXA1/THBS1/ICAM1/RET/S100A12/RIPK3/LGALS3/CCL2/ECM1/KLRC4-KLRK1/ITGA4/SLAMF8/HSD3B7/CXCL10/C5AR1/CCL5/CXCR4/LYN/FPR2/F11R/SERPINE1/CCL18/SPN/CCR5/PDGFD/CXCR2/ALOX5/CXCL11/IL6/PIK3CG/FADD/ITGAL/TNFRSF14/MYO1G/SAA1/CCR1/PLCB1/GPR183 48

BP GO:0072001 renal system development 68/2475 324/18870 4.88313253800493e-05 0.000753684518205183 0.000547444678797306 CENPF/WNT4/HOXD11/ITGB3/WWTR1/NPNT/IQGAP1/HS3ST3B1/SLC22A6/NID1/HOXC11/ACE/PROM1/HOXA11/ITGA3/RET/NRP1/FOXJ1/FREM2/CITED1/STAT1/BMP2/SOX8/MMP9/JAG1/ENPEP/GPR4/ANGPT2/FOXD1/GLI3/ACTA2/GREB1L/ANGPT1/VEGFA/GFRA1/LGR5/HAS2/COL4A1/PCSK5/OSR1/LRRK2/PDGFA/LIF/TACSTD2/RGN/PDGFD/SDC4/SDC1/CXCR2/STRA6/EFNB2/HS3ST3A1/RDH10/GDNF/TGFB2/SPRY1/NUP107/FADD/OSR2/EYA1/NOG/AQP1/UPK3A/EZH2/WNT7B/HOXB7/GCNT1/TP73 68

BP GO:0050829 defense response to Gram-negative bacterium 26/2475 89/18870 4.93583536445281e-05 0.000759865526363454 0.000551934302697112 RARRES2/PYCARD/MR1/SLC11A1/NFKBIZ/GSDMD/CHGA/TREM2/SSC5D/F2RL1/H2BC11/LYZ/H2BC12/SERPINE1/LTF/NOS2/RNASE3/IGHM/RNASE6/HMGB2/CXCL6/IL6/LBP/TNFRSF14/AQP1/TREM1 26

BP GO:0002698 negative regulation of immune effector process 33/2475 125/18870 5.0637703882761e-05 0.00077756719721764 0.000564792050575526 ASCL2/IL7R/SPI1/LOXL3/ANXA1/FOXJ1/HAVCR2/AHR/LGALS3/IL13RA2/MICA/SLAMF8/ANGPT1/JAK3/FCGR2B/VSIG4/DUSP10/ACP5/SPN/SERPING1/CD300A/TWIST1/HLA-B/LILRB1/HLA-A/FGL2/PTPRC/TGFB2/ZC3H12A/HFE/IL4I1/HLA-F/BST2 33

BP GO:1904064 positive regulation of cation transmembrane transport 36/2475 141/18870 5.10190375362519e-05 0.000781424238182797 0.000567593642622695 AKAP5/CAV1/VMP1/AKAP6/KCNC1/RGS7/TMSB4X/STAC/FGF13/TREM2/NIPSNAP2/MIR210/FLNA/KCNMB1/APLNR/P2RY6/WNK2/CXCL10/GLRX/PIRT/RYR2/KCNN4/KCNE5/GRIN1/CXCL9/ADCYAP1R1/F2RL3/KCNC2/LRRC55/FXYD2/CXCL11/ANK3/RAMP3/GAL/CTSS/KCNIP2 36

BP GO:1901988 negative regulation of cell cycle phase transition 60/2475 277/18870 5.17914525998498e-05 0.000791236339464372 0.00057472073957728 CENPF/SPC24/MDM2/MAD2L1/WEE1/PARP9/PLK3/FANCD2/FBXO4/SUSD2/E2F7/KNTC1/RBBP8/CHEK2/WDR76/CCL2/MAP3K20/CCNB1/DTL/NABP1/ATF5/TIMELESS/GPNMB/ORC1/BIRC5/BRCA1/CAMSAP3/HASPIN/PKMYT1/TTK/MUC1/ZWILCH/BTN2A2/DTX3L/RAD51/NUF2/CDK2/TRIP13/BUB1B/BRCA2/BUB1/AURKB/CLSPN/CDC45/E2F1/BRIP1/CDT1/MYO16/PLK1/CDC6/CDCA8/NDC80/CDK1/GTSE1/KNL1/ZWINT/EZH2/CHEK1/EME1/CDC20 60

BP GO:1903531 negative regulation of secretion by cell 38/2475 152/18870 5.26238977793025e-05 0.000801913406768863 0.000582476111407027 IL1RAPL1/SPI1/TSPO/CHGA/ANXA1/ABCC8/GABBR1/ACVR1C/VAMP8/KCNB1/CRH/IL13RA2/ASIC1/CNR1/TNFRSF1A/F2RL1/FCGR2B/IL11/SYT4/LIF/KCNJ11/CD300A/RAP1B/P2RY1/LILRB1/HRH3/RSAD2/VSNL1/CRY2/OSM/STXBP6/TRH/ADORA1/RHBDF1/UCP2/ADRA2B/MAOB/HLA-F 38

BP GO:0048168 regulation of neuronal synaptic plasticity 19/2475 56/18870 5.39306011196909e-05 0.000819745137019302 0.000595428328951044 SLC4A10/VGF/RAB3A/SHISA7/GRIN2A/GRM5/S100B/SLC8A2/RASGRF1/SYT4/GRIN1/CAMK2A/ARC/SYP/JPH3/SYNGR1/NEURL1/NOG/GRIN2B 19

BP GO:0002486 antigen processing and presentation of endogenous peptide antigen via MHC class I via ER pathway, TAP-independent 9/2475 16/18870 5.42260240871414e-05 0.000820083245892184 0.000595673916990659 HLA-C/MICA/MICB/HLA-B/HLA-A/ULBP2/ULBP3/HFE/HLA-F 9

BP GO:0034112 positive regulation of homotypic cell-cell adhesion 9/2475 16/18870 5.42260240871414e-05 0.000820083245892184 0.000595673916990659 MMRN1/CCL5/PDPN/EMILIN2/F11R/EMILIN1/IL6/PLAUR/ANK3 9

BP GO:0002291 T cell activation via T cell receptor contact with antigen bound to MHC molecule on antigen presenting cell 7/2475 10/18870 5.52751666032489e-05 0.000827611222658121 0.000601141922134822 ICAM1/HAVCR2/LGALS3/LILRB1/FGL2/HLA-DMB/ITGAL 7

BP GO:0031055 chromatin remodeling at centromere 7/2475 10/18870 5.52751666032489e-05 0.000827611222658121 0.000601141922134822 CENPI/CENPW/CENPA/HJURP/CENPN/OIP5/ITGB3BP 7

BP GO:0034080 CENP-A containing chromatin assembly 7/2475 10/18870 5.52751666032489e-05 0.000827611222658121 0.000601141922134822 CENPI/CENPW/CENPA/HJURP/CENPN/OIP5/ITGB3BP 7

BP GO:0048251 elastic fiber assembly 7/2475 10/18870 5.52751666032489e-05 0.000827611222658121 0.000601141922134822 EFEMP2/COL3A1/MFAP4/LOX/EMILIN1/FBLN5/THSD4 7

BP GO:0019885 antigen processing and presentation of endogenous peptide antigen via MHC class I 11/2475 23/18870 5.71962101679131e-05 0.000854243895144652 0.000620486773306268 HLA-C/MICA/MICB/HLA-B/HLA-A/ULBP2/TAP1/ULBP3/B2M/HFE/HLA-F 11

BP GO:0010810 regulation of cell-substrate adhesion 50/2475 220/18870 5.9279310159403e-05 0.000883158754831404 0.000641489309103844 COL8A1/ITGA5/WNT4/FERMT1/EFEMP2/MDK/ITGB3/COL26A1/EMP2/SEMA3E/NPNT/ECM2/NID1/MMP12/THBS1/ITGA3/NRP1/FZD7/FLNA/CCN1/GBP1/S100A10/FBLN1/JAG1/ANGPT2/SPRY4/CD3E/MMP14/VEGFA/EGFLAM/CAMSAP3/PDPN/HAS2/BST1/COL1A1/FN1/SERPINE1/PLAU/TACSTD2/CD36/EMILIN1/RAC2/SDC4/HOXA7/RRAS/POSTN/AJAP1/EPHA3/PRKCE/CDK6 50

BP GO:0001822 kidney development 66/2475 314/18870 5.96690126004746e-05 0.00088676423676547 0.000644108179269845 CENPF/WNT4/HOXD11/ITGB3/WWTR1/NPNT/IQGAP1/HS3ST3B1/SLC22A6/NID1/HOXC11/ACE/PROM1/HOXA11/ITGA3/RET/NRP1/FOXJ1/FREM2/CITED1/STAT1/BMP2/SOX8/MMP9/JAG1/ENPEP/GPR4/ANGPT2/FOXD1/GLI3/ACTA2/GREB1L/ANGPT1/VEGFA/GFRA1/HAS2/PCSK5/OSR1/LRRK2/PDGFA/LIF/TACSTD2/RGN/PDGFD/SDC4/SDC1/CXCR2/STRA6/EFNB2/HS3ST3A1/RDH10/GDNF/TGFB2/SPRY1/NUP107/FADD/OSR2/EYA1/NOG/AQP1/UPK3A/EZH2/WNT7B/HOXB7/GCNT1/TP73 66

BP GO:0048732 gland development 87/2475 443/18870 6.317976181748e-05 0.000934988497429602 0.000679136250368323 CAV1/HOXD3/WNT4/MDK/HAND2/PITX1/APLN/HOXA5/LEF1/NKX2-5/MSN/TSPO/HOXB3/OAS2/E2F7/ITGA2/HOXA3/SOD2/ANXA1/HOXA11/CLDN1/CCKBR/NRP1/MMP2/NRG3/CRH/EGFR/TWSG1/RIPK3/E2F8/IGFBP5/CSMD1/HOXD9/CYP1B1/BMP2/AREG/ALOX15B/TYMS/OXTR/MET/GLI3/HOXA9/TNFAIP3/VEGFA/GPX1/FRZB/TFCP2L1/SOCS2/IGF2/HGF/RPS6KA1/HK2/CCNB2/CCDC40/PDGFA/CEBPB/RGN/AURKA/TNC/SOX10/PCNA/GLI1/BRCA2/OTP/HOXA10/STRA6/EPHA2/SNAI2/RXFP1/IQGAP3/TBX1/ALDH1A3/HOXB13/IL6/TBX19/TGFB2/FADD/NEURL1/HFE/NOG/SLC29A1/EZH2/PTN/UCP2/WNT7B/HOXD13/NODAL 87

BP GO:0017158 regulation of calcium ion-dependent exocytosis 15/2475 39/18870 6.32254047229211e-05 0.000934988497429602 0.000679136250368323 CDK5R2/STX1A/KCNB1/DOC2A/STXBP1/RAB3A/SYT7/SCAMP5/SYT1/SYT4/RAP1B/ZP3/SYT5/SYT2/SYT13 15

BP GO:0032946 positive regulation of mononuclear cell proliferation 37/2475 148/18870 6.55808991133338e-05 0.000967439111244365 0.000702707008991445 CD276/EFNB1/PYCARD/IL18/IL1A/TNFRSF4/ANXA1/CD40/CD70/TFRC/CHRNB2/VAV3/CD74/HAVCR2/HLA-DPA1/CD274/CD3E/PDCD1LG2/CCL5/IGF2/SASH3/BST1/SPN/HLA-DPB1/LILRB2/IGFBP2/ZP3/HLA-A/HLA-DMB/PTPRC/IL6/FCGR3A/IL2RA/FADD/MYD88/CLCF1/GPR183 37

BP GO:0043583 ear development 50/2475 221/18870 6.70155733984046e-05 0.000986180153637306 0.000716319712563442 CHRNA9/MCM2/USH1C/SLC17A8/CYTL1/SPRY2/HOXA2/ATP8B1/ATP8A2/NKX3-2/GABRA5/FREM2/BMP2/JAG1/DLX5/GLI3/ROR1/GSC/FRZB/LGR5/GJB2/KCNK3/ECE1/PCDH15/OSR1/CEBPD/CTHRC1/FZD2/CCNA2/TWIST1/OTX1/ADGRV1/GABRB2/SDC4/STRA6/HPCA/TBX1/ALDH1A3/ZIC1/SLC25A27/RDH10/STOX1/TGFB2/EYA4/OSR2/EYA1/HOXA1/NOG/COL2A1/FZD6 50

BP GO:0090322 regulation of superoxide metabolic process 13/2475 31/18870 6.76491431138915e-05 0.000993069572752579 0.000721323896334713 H19/ITGB2/GCH1/CLEC7A/F2RL1/BST1/ACP5/FPR2/MAPT/MIR27B/CD36/TYROBP/FBLN5 13

BP GO:0045786 negative regulation of cell cycle 81/2475 407/18870 6.9901968875794e-05 0.00102363761251285 0.000743527232483734 NEK2/CENPF/SPC24/MDM2/MAD2L1/TMEM67/WEE1/PARP9/PLK3/FANCD2/FBXO4/SUSD2/E2F7/TENT5B/KNTC1/RBBP8/CHEK2/WDR76/E2F8/CCL2/BMP2/MAP3K20/CCNB1/TOM1L1/DTL/ALOX15B/NABP1/ATF5/TIMELESS/GPNMB/ORC1/BIRC5/HOXC9/BRCA1/BRINP1/CAMSAP3/HASPIN/ESPL1/PKMYT1/TTK/MUC1/BTG3/ZWILCH/BTN2A2/RUNX3/LIF/HOXD10/DTX3L/RAD51/NUF2/CDK2/TRIP13/BUB1B/BRCA2/BUB1/LILRB1/NR2F2/AURKB/CASP3/CLSPN/CDC45/E2F1/BRIP1/CDT1/TP53I13/MYO16/PLK1/CDC6/CDCA8/NDC80/GAS1/CDK1/GTSE1/KNL1/ZWINT/EZH2/LATS2/CHEK1/CDK6/EME1/CDC20 81

BP GO:0045165 cell fate commitment 61/2475 286/18870 7.20883947373845e-05 0.00105308691484977 0.000764917964904557 WNT4/BATF/PITX1/TGFB1I1/NKX2-5/TEAD4/LOXL3/SPRY2/FGF13/HOXA11/HOXA2/NRP1/FZD7/WNT16/TEAD3/DLX2/BMP2/SOX8/NR2E1/GAP43/JAG1/DSCAML1/GLI3/SLAMF8/TOX/FEZF2/GSX2/GSC/PDPN/DKK1/SFRP2/TBX5/NEUROD4/MYOD1/CTSL/RUNX2/DMRTA2/SOCS3/EYA2/HOXD10/TBX15/SPN/NR2F2/CASP3/HOXC10/MYT1L/TBR1/POU4F1/GATA4/TBX1/ITGB1/GAS1/WNT10B/IL6/PPARG/TBX19/EYA1/LATS2/WNT7B/DLL4/NODAL 61

BP GO:0042542 response to hydrogen peroxide 28/2475 101/18870 7.24744220922723e-05 0.00105615638408253 0.000767147498028319 MDM2/FOSL1/ECT2/SOD2/ANXA1/HMOX1/MMP2/RIPK3/CYP1B1/STAT1/AREG/NET1/TNFAIP3/SMPD3/GPX1/SPHK1/COL1A1/HP/HBA1/PCNA/PDGFD/CASP3/SDC1/ADAM9/CDK1/IL6/AQP1/EZH2 28

BP GO:0034109 homotypic cell-cell adhesion 27/2475 96/18870 7.38752662900361e-05 0.00107136980387772 0.000778197885175743 MMRN1/ITGB3/C1QTNF1/CTNNA3/STXBP1/FLNA/MEGF11/HSPB1/DSG2/CCL5/PDPN/LYN/EMILIN2/F11R/MYL9/PPIA/EMILIN1/F2RL3/DSC2/MYL12A/PLEK/IL6/PIK3CG/PLAUR/ANK3/CLIC1/FERMT3 27

BP GO:0051310 metaphase chromosome alignment 27/2475 96/18870 7.38752662900361e-05 0.00107136980387772 0.000778197885175743 NEK2/CENPF/SPC24/KIFC1/SGO1/KIF14/ECT2/CDCA5/KNTC1/PSRC1/CCNB1/FAM83D/ANKRD53/BIRC5/DSN1/ZWILCH/NUF2/KIF18A/AURKB/KIF2C/CDT1/CENPE/CDCA8/NDC80/KNL1/CHMP4BP1/SPAG5 27

BP GO:0002367 cytokine production involved in immune response 32/2475 122/18870 7.48014666650293e-05 0.00108218796592009 0.000786055742278292 PYCARD/IL18/LITAF/CD74/IL1R1/ARID5A/CLEC7A/SLAMF9/SPON2/ANGPT1/JAK3/F2RL1/SCIMP/SASH3/FZD5/ACP5/CD36/LAPTM5/TWIST1/LILRB1/TRIM6/HLA-A/RSAD2/B2M/IL6/TGFB2/TNFRSF14/HFE/MYD88/TREM1/HLA-F/BST2 32

BP GO:0001959 regulation of cytokine-mediated signaling pathway 41/2475 171/18870 7.5885409216777e-05 0.00109523076186906 0.000795529479719401 CAV1/IRF7/PYCARD/PARP9/RBM47/PYDC1/IL1RN/MMP12/VRK2/TREM2/HPX/CD74/IL1R1/PALM3/OAS1/ECM1/TNFRSF1A/CASP1/TNFAIP3/CASP8/ANGPT1/CD300LF/CARD16/F2RL1/CCL5/CXCR4/SPHK1/H2BC11/IL1R2/OAS3/MIR27B/NLRC5/ISG15/LAPTM5/TRIM6/PTPRC/IL6/PPARG/FADD/IRAK2/CASP4 41

BP GO:0000302 response to reactive oxygen species 47/2475 205/18870 7.76275542480309e-05 0.00111501396101717 0.000809899162048834 MDM2/H19/IL1A/FOSL1/NCF1/PLK3/ECT2/SOD2/ANXA1/HMOX1/PAWR/GCH1/MMP2/EGFR/RIPK3/CYP1B1/STAT1/AREG/MMP9/MET/NET1/TNFAIP3/SMPD3/GPX1/SPHK1/HGF/COL1A1/HP/MAPT/HBA1/SOD3/LRRK2/ADCYAP1R1/CD36/PCNA/PDGFD/FBLN5/CASP3/SDC1/ADAM9/CDK1/IL6/CRYGD/AQP1/EZH2/UCP2/MT3 47

BP GO:0050777 negative regulation of immune response 47/2475 205/18870 7.76275542480309e-05 0.00111501396101717 0.000809899162048834 ASCL2/IL7R/SPI1/LOXL3/ANXA1/MMP12/TREM2/CGAS/FOXJ1/COL3A1/TRIM21/HAVCR2/AHR/LGALS3/IL13RA2/MICA/OAS1/SLAMF8/HLA-DRB1/TNFAIP3/SAMSN1/JAK3/IFI16/NMI/GPX1/FCGR2B/VSIG4/DUSP10/LYN/OAS3/NLRC5/SPN/SERPING1/ISG15/CD300A/HLA-B/LILRB1/AURKB/HLA-A/FGL2/PTPRC/PPARG/ZC3H12A/TNFRSF14/HFE/IL4I1/HLA-F 47

BP GO:0051701 biological process involved in interaction with host 48/2475 211/18870 8.03948058581831e-05 0.00114950943531141 0.000834955221167494 CAV1/ITGA5/ITGB3/TNFRSF4/SIGLEC1/CXCL8/ITGA2/FUCA2/TFRC/CLDN1/ICAM1/NRP1/VAMP8/CD74/TRIM21/EGFR/TRIM38/TRIM22/CIITA/IFITM3/HLA-DRB1/PLSCR1/CTSB/HTR2A/CAV2/CXCR4/CLEC5A/IFITM2/CTSL/F11R/LTF/PPIA/ANPEP/NECTIN2/CCR5/LGALS1/TRIM6/TRIM5/EPHA2/EFNB2/ITGB1/DPP4/CDK1/JPT2/PTX3/CHMP4BP1/TNFRSF14/SLC1A5 48

BP GO:1903307 positive regulation of regulated secretory pathway 16/2475 44/18870 8.04120524368405e-05 0.00114950943531141 0.000834955221167494 KLRC2/CDK5R2/STX1A/ITGB2/VAMP8/KCNB1/STXBP1/RAB27A/RAB3A/SYT7/F2RL1/SCAMP5/SYT1/SYT4/ZP3/HLA-F 16

BP GO:0060759 regulation of response to cytokine stimulus 43/2475 183/18870 8.47246667475661e-05 0.00120828242078952 0.000877645441518106 CAV1/IRF7/PYCARD/PARP9/RBM47/PYDC1/IL1RN/MMP12/VRK2/TREM2/HPX/CD74/IL1R1/PALM3/OAS1/ECM1/TNFRSF1A/CASP1/TNFAIP3/CASP8/ANGPT1/CD300LF/TLR2/CARD16/F2RL1/CCL5/CXCR4/SPHK1/IFIH1/H2BC11/IL1R2/OAS3/MIR27B/NLRC5/ISG15/LAPTM5/TRIM6/PTPRC/IL6/PPARG/FADD/IRAK2/CASP4 43

BP GO:0031338 regulation of vesicle fusion 11/2475 24/18870 9.3098704002616e-05 0.00132198023015627 0.000960230739776541 ANXA1/CPLX2/DOC2A/STXBP1/RAB3A/ERC2/SPHK1/SYT1/SYT4/ANXA2/CPLX1 11

BP GO:1903707 negative regulation of hemopoiesis 31/2475 118/18870 9.31375145496508e-05 0.00132198023015627 0.000960230739776541 LRRC17/ASCL2/MDK/MIR221/LOXL3/ANXA1/FOXJ1/LILRB3/CD74/SOCS1/RUNX1/GLI3/JAK3/FCGR2B/LYN/FSTL3/RUNX3/LTF/TNFRSF11B/LILRB1/TNFAIP6/TMEM176A/FGL2/HOXA7/SNAI2/ERFE/C1QC/ZC3H12A/IRF1/TMEM176B/CDK6 31

BP GO:0001701 in utero embryonic development 78/2475 392/18870 9.49480216777188e-05 0.00134449981639864 0.000976588018398185 MYBPHL/STIL/NEK2/LPAR6/ASCL2/HAND2/EMX1/ASF1B/SNAI1/RSPO3/LEF1/FOSL1/TEAD4/TM4SF1/E2F7/TTPA/TET1/RBBP8/HOPX/ACVR1C/RRM2/GINS1/COL3A1/EGFR/CITED1/E2F8/ST14/CCN1/BMP2/SOX8/CCNB1/SPINT1/GLI3/PTPRR/PSPH/CASP8/ANGPT1/VEGFA/PPP4R4/CMTM3/CAMSAP3/SLC34A2/IGF2/FZD5/NPM2/CCNB2/ARHGDIG/SOCS3/ETV2/FBLL1/YBX3/LIF/CEBPB/TMEM100/SOX10/TWIST1/FOXD3/BRCA2/ZP3/NR2F2/GINS4/PHLDA2/CELF4/ADAMTS3/ETNK2/SEC24D/PLK4/ITGB1/RDH10/FKBP10/XRCC2/PLOD3/NOG/ADM/LATS2/WNT7B/CHEK1/NODAL 78

BP GO:0035987 endodermal cell differentiation 17/2475 49/18870 9.59935223794409e-05 0.001353704964294 0.000983274249985984 COL8A1/ITGA5/ITGB2/MMP2/COL12A1/MMP9/ITGA4/MMP14/DKK1/FN1/COL4A2/COL6A1/LAMB1/COL5A2/ITGA7/COL5A1/NODAL 17

BP GO:0071711 basement membrane organization 14/2475 36/18870 9.60490197850176e-05 0.001353704964294 0.000983274249985984 CAV1/FERMT1/NID1/COL3A1/LAMA2/NID2/CAV2/LAMC1/COL4A1/LAMB1/ITGB1/MMP11/PLOD3/CTSS 14

BP GO:0032409 regulation of transporter activity 58/2475 271/18870 9.7005038581823e-05 0.00136397717012943 0.000990735547502755 CAV1/VMP1/AKAP6/SYNGR3/TMSB4X/STAC/SLN/JPH4/CACNG2/PRRT1/FGF13/TREM2/CABP4/FGF12/NIPSNAP2/CRH/PON1/TCAF2/P2RY6/CCL2/MMP9/SHANK1/SHISA7/WNK2/KCNAB1/KCNS2/GLRX/PIRT/SELENON/CNIH3/SCN2B/RYR2/GRM5/HECW1/KCNE5/OSR1/KCNE3/ARC/CABP1/SCN3B/LRRC55/HPCA/JPH3/ITGB1/MAPK8IP2/FXYD2/PRKCE/PPARG/FKBP1C/ANK3/GAL/KCNE4/GRP/CACNG5/CACNG3/FXYD5/CTSS/KCNIP2 58

BP GO:0048499 synaptic vesicle membrane organization 12/2475 28/18870 0.000100000954851124 0.0014006783852799 0.00101739376384421 STX1A/STX1B/CPLX2/DOC2A/STXBP1/RAB3A/ERC2/SYT1/SNAP25/SYP/SYNGR1/CPLX1 12

BP GO:0032757 positive regulation of interleukin-8 production 20/2475 63/18870 0.000100548365099207 0.0014006783852799 0.00101739376384421 DDIT3/PYCARD/TLR1/F3/CD74/CLEC7A/TLR2/F2RL1/CD58/CHI3L1/TLR8/SERPINE1/CD14/NOS2/LILRA2/CD2/IL6/LBP/FADD/MYD88 20

BP GO:0140895 cell surface toll-like receptor signaling pathway 20/2475 63/18870 0.000100548365099207 0.0014006783852799 0.00101739376384421 LY96/TLR1/CYBA/TREM2/OAS1/RAB7B/TNFAIP3/TLR2/F2RL1/NMI/SCIMP/LYN/CD14/LTF/LILRA2/IFI35/PRKCE/LBP/IRAK2/MYD88 20

BP GO:2000179 positive regulation of neural precursor cell proliferation 20/2475 63/18870 0.000100548365099207 0.0014006783852799 0.00101739376384421 MDK/FLNA/DISP3/HAPLN1/NR2E1/GLI3/TOX/VEGFA/LYN/DMRTA2/LHX5/SOX10/GLI1/OTP/HAPLN3/ITGB1/WDR62/ASPM/RASSF10/DLL4 20

BP GO:0002476 antigen processing and presentation of endogenous peptide antigen via MHC class Ib 9/2475 17/18870 0.000101802178202256 0.00141159417534952 0.00102532253383237 HLA-C/MICA/MICB/HLA-B/HLA-A/ULBP2/ULBP3/HFE/HLA-F 9

BP GO:0002484 antigen processing and presentation of endogenous peptide antigen via MHC class I via ER pathway 9/2475 17/18870 0.000101802178202256 0.00141159417534952 0.00102532253383237 HLA-C/MICA/MICB/HLA-B/HLA-A/ULBP2/ULBP3/HFE/HLA-F 9

BP GO:0043123 positive regulation of canonical NF-kappaB signal transduction 45/2475 196/18870 0.00010524536058013 0.00145597498830208 0.00105755888645035 TRADD/IL1A/TRAF5/ECT2/LITAF/HMOX1/TNFRSF19/CD40/TFRC/S100A12/CD74/TRIM21/FLNA/S100A13/CLEC7A/TRIM38/TRIM22/TIFA/BIRC3/PRKCB/NEK6/ECM1/SECTM1/ROR1/TNFRSF1A/CASP1/HLA-DRB1/CASP8/CARD16/F2RL1/AJUBA/S100B/LTF/CD36/LGALS1/TRIM6/TRIM5/S100A4/LTBR/RHOC/ALPK1/PRKCE/FADD/MYD88/BST2 45

BP GO:0042063 gliogenesis 69/2475 338/18870 0.000105708716679867 0.00145902329872626 0.00105977305070705 ASCL2/CDK5R2/MDK/EMX1/CDK5R1/LEF1/MIR221/TSPO/TSPAN2/ROR2/GPR157/ANXA1/ABCC8/TREM2/COL3A1/LAMA2/HAPLN1/DLX2/CCL2/BMP2/SOX8/AREG/NR2E1/GAP43/MXRA8/ITGB4/CDKN2C/LAMC3/SPINT1/S100A8/GPC1/CHRM1/GLI3/ROR1/S100A9/HEXB/MMP14/GSX2/C5AR1/TLR2/SLC8A3/CXCR4/NEUROD4/DUSP10/LYN/MAPT/LIF/SOX10/LAMB1/P2RY1/NCMAP/NKX6-1/HAPLN3/VIM/RRAS/DAAM2/CDK1/IL6/C1QA/TGFB2/TUBA1A/MYD88/NOG/EZH2/PTN/CLCF1/GPR183/CDK6/TP73 69

BP GO:0010948 negative regulation of cell cycle process 66/2475 320/18870 0.000107855521049831 0.0014852397898697 0.00107881560528598 NEK2/CENPF/SPC24/MDM2/MAD2L1/TMEM67/WEE1/PARP9/PLK3/FANCD2/FBXO4/SUSD2/E2F7/KNTC1/RBBP8/CHEK2/WDR76/E2F8/CCL2/MAP3K20/CCNB1/TOM1L1/DTL/NABP1/ATF5/TIMELESS/GPNMB/ORC1/BIRC5/BRCA1/CAMSAP3/HASPIN/ESPL1/PKMYT1/TTK/MUC1/ZWILCH/BTN2A2/LIF/DTX3L/RAD51/NUF2/CDK2/TRIP13/BUB1B/BRCA2/BUB1/AURKB/CLSPN/CDC45/E2F1/BRIP1/CDT1/MYO16/PLK1/CDC6/CDCA8/NDC80/CDK1/GTSE1/KNL1/ZWINT/EZH2/CHEK1/EME1/CDC20 66

BP GO:0042113 B cell activation 59/2475 278/18870 0.000108383644451204 0.00148909702811219 0.00108161734062709 IL7R/BATF/LEF1/NFKBIZ/SPI1/TNFRSF4/CD40/SKAP2/CD70/TFRC/PAWR/CHRNB2/BCL3/VAV3/FOXJ1/CD74/AHR/CD79A/CD180/PRKCB/GAPT/NFAM1/ITGA4/SLAMF8/TNFAIP3/MMP14/SAMSN1/CASP8/JAK3/FCGR2B/IL2RG/SASH3/IL11/BST1/HDAC4/LYN/EXO1/CMTM7/CD300A/LAPTM5/TYROBP/LGALS1/CASP3/LAT2/THEMIS2/IL9/FOSL2/PTPRC/ITGB1/CHRNA4/NTRK1/IL6/SWAP70/TCIRG1/IL4I1/EZH2/CLCF1/GPR183/BST2 59

BP GO:0003170 heart valve development 22/2475 73/18870 0.000110274801197977 0.0015022819554815 0.00109119431634346 PDE2A/MDM2/SHOX2/SNAI1/NKX2-5/APLNR/FGFRL1/CCN1/BMP2/JAG1/TNFRSF1A/MDM4/DKK1/TBX5/EMILIN1/TWIST1/STRA6/ELN/SNAI2/GATA4/TGFB2/DLL4 22

BP GO:0050818 regulation of coagulation 22/2475 73/18870 0.000110274801197977 0.0015022819554815 0.00109119431634346 CAV1/THBD/PLAT/C1QTNF1/THBS1/F3/F2RL1/EMILIN2/SERPINE1/PDGFA/VKORC1/SERPING1/PLAU/CD36/EMILIN1/PROS1/ANXA5/FAP/TFPI/PLAUR/PROCR/ANXA2 22

BP GO:0032609 type II interferon production 31/2475 119/18870 0.000110344160953921 0.0015022819554815 0.00109119431634346 CD276/DDIT3/PYCARD/IL18/SLC11A1/BCL3/HAVCR2/RIPK3/IL1R1/HLA-DPA1/ARID5A/CLEC7A/KLRC4-KLRK1/CD274/HLA-DRB1/CD3E/F2RL1/PDCD1LG2/SASH3/TLR8/FZD5/CD14/HLA-DPB1/ISG15/LAPTM5/LILRB1/ZP3/HLA-A/CD2/ZC3H12A/FADD 31

BP GO:0032649 regulation of type II interferon production 31/2475 119/18870 0.000110344160953921 0.0015022819554815 0.00109119431634346 CD276/DDIT3/PYCARD/IL18/SLC11A1/BCL3/HAVCR2/RIPK3/IL1R1/HLA-DPA1/ARID5A/CLEC7A/KLRC4-KLRK1/CD274/HLA-DRB1/CD3E/F2RL1/PDCD1LG2/SASH3/TLR8/FZD5/CD14/HLA-DPB1/ISG15/LAPTM5/LILRB1/ZP3/HLA-A/CD2/ZC3H12A/FADD 31

BP GO:0043367 CD4-positive, alpha-beta T cell differentiation 26/2475 93/18870 0.000111069411164898 0.00150873471636662 0.00109588132997421 ASCL2/BATF/IL18/LEF1/NFKBIZ/RELB/LOXL3/ANXA1/BCL3/SOCS1/RUNX1/PTGER4/TOX/HLA-DRB1/JAK3/IL2RG/SASH3/CTSL/SOCS3/RUNX3/SPN/HLA-DRA/RSAD2/IL6/ZC3H12A/GPR183 26

BP GO:0051051 negative regulation of transport 90/2475 469/18870 0.000113174399332322 0.00153124261033081 0.00111223011581762 IL1RAPL1/CAV1/SP100/FERMT1/ITGB3/NECAB2/SPI1/SLN/TSPO/STC1/STX1B/HAMP/CHGA/ACE/ANXA1/ABCC8/THBS1/GABBR1/ACVR1C/SFRP4/VAMP8/KCNB1/CD74/CRH/CD33/LGALS3/IL13RA2/ASIC1/TCAF2/CNR1/MMP9/PRKCB/TNFRSF1A/KCNAB1/ANGPT1/CD300LF/TLR2/PLSCR1/F2RL1/HTR2A/FCGR2B/ENPP1/VIP/IL11/HECW1/SCAMP5/OS9/PID1/KCNE5/MIR27B/SYT4/OSR1/LRRK2/LIF/KCNJ11/LILRB2/KCNE3/CD36/CD300A/RAP1B/P2RY1/LILRB1/CABP1/HRH3/LYPLA1/MDFIC/APOC1/FABP5/RSAD2/VSNL1/PACSIN1/CRY2/OSM/PRKG2/STXBP6/TRH/ADORA1/EPHA3/PRKCE/RHBDF1/RGS4/ANK3/ANXA2/KCNE4/GRP/UCP2/ADRA2B/MAOB/GABRE/HLA-F 90

BP GO:0007492 endoderm development 24/2475 83/18870 0.000113236462189687 0.00153124261033081 0.00111223011581762 COL8A1/ITGA5/ITGB2/DUSP5/HOXC11/MMP2/DUSP4/COL12A1/MMP9/ITGA4/MMP14/DKK1/LAMC1/FN1/COL4A2/ARC/COL6A1/LAMB1/COL5A2/ITGA7/GATA4/COL5A1/NOG/NODAL 24

BP GO:0031638 zymogen activation 19/2475 59/18870 0.000120158798347954 0.00162119870849689 0.00117757043537688 THBD/C1RL/PLAT/C1R/THBS1/RUNX1/S100A10/MMP14/CASP8/IFI16/GRIN2A/CTSL/SERPINE1/PLAU/PGK1/CTSZ/PLAUR/FADD/ANXA2 19

BP GO:0044772 mitotic cell cycle phase transition 90/2475 470/18870 0.000122326171464685 0.00164674065801339 0.00119612303133866 STIL/CENPF/SPC24/MDM2/MAD2L1/WEE1/KIF14/CKS2/MIR221/PLK3/BCL7A/CCNJL/USH1C/E2F7/CDCA5/ANXA1/ACTL6B/MELK/CCNP/KNTC1/RBBP8/RRM2/CHEK2/CDKN3/UBE2C/EGFR/UBE2S/TACC3/CCL2/MAP3K20/CCNB1/CDKN2C/DTL/NEK6/NABP1/DLGAP5/GPNMB/BRSK2/FOXM1/ORC1/BIRC5/BCAT1/BRCA1/CKS1B/HASPIN/ESPL1/PKMYT1/TTK/PLK5/MUC1/NPM2/ZWILCH/CCNB2/BTN2A2/CAMK2A/NUF2/CDK2/CCNA2/AURKA/TRIP13/BUB1B/CDC25C/BUB1/AURKB/E2F2/CLSPN/E2F1/IQGAP3/DBF4/MYO16/PLK1/CDC6/CENPE/CDCA8/NDC80/CDK4/ITGB1/CDK1/WNT10B/CDC25A/STOX1/GTSE1/KNL1/ZWINT/EZH2/LATS2/PLCB1/CHEK1/CDK6/CDC20 90

BP GO:0014046 dopamine secretion 15/2475 41/18870 0.000123393741522562 0.00165369648236933 0.00120117544907888 GABBR1/CHRNB2/CNR1/PRKCB/SNCG/SYT7/HTR2A/SYT1/SYT4/GRM2/SYT5/CHRNA4/GDNF/SYT2/SYT13 15

BP GO:0042554 superoxide anion generation 15/2475 41/18870 0.000123393741522562 0.00165369648236933 0.00120117544907888 NCF1B/NCF4/NCF1/ITGB2/CYBA/SOD2/NOX4/CLEC7A/NCF2/F2RL1/ACP5/FPR2/MAPT/TYROBP/NCF1C 15

BP GO:0050920 regulation of chemotaxis 51/2475 232/18870 0.000123751484842773 0.00165479713807574 0.00120197491900975 RARRES2/MDK/SEMA3E/CXCL13/TMSB4X/SPI1/CCL26/CXCL8/ITGA2/THBS1/TREM2/NRP1/SEMA3A/F3/CD74/SEMA3D/SLIT1/HSPB1/DSCAM/CCL2/KLRC4-KLRK1/ANGPT2/MET/SLAMF8/FEZF2/VEGFA/CXCL10/C5AR1/F2RL1/CCL5/CXCR4/SEMA6B/BST1/LYN/FPR2/SERPINE1/THBS4/TNFAIP6/RAC2/PDGFD/SEMA3F/CXCR2/EFNB2/TBR1/DPP4/IL6/LBP/SWAP70/PTN/CCR1/GPR183 51

BP GO:0070593 dendrite self-avoidance 8/2475 14/18870 0.000124469928108063 0.00165702316709714 0.00120359181270091 EMB/DSCAM/DSCAML1/IGSF9/CNTN6/CNTN4/NEXN/PALLD 8

BP GO:0098883 synapse pruning 8/2475 14/18870 0.000124469928108063 0.00165702316709714 0.00120359181270091 ADGRB3/TREM2/DKK1/C3/C1QB/ITGB1/C1QC/C1QA 8

BP GO:0034767 positive regulation of monoatomic ion transmembrane transport 38/2475 158/18870 0.000127301628802859 0.0016872383649721 0.00122553885937084 AKAP5/CAV1/VMP1/AKAP6/KCNC1/RGS7/TMSB4X/STAC/FGF13/TREM2/NIPSNAP2/MIR210/FLNA/KCNMB1/APLNR/P2RY6/CCL2/WNK2/CXCL10/GLRX/PIRT/RYR2/KCNN4/KCNE5/GRIN1/CXCL9/ARC/ADCYAP1R1/F2RL3/KCNC2/LRRC55/FXYD2/CXCL11/ANK3/RAMP3/GAL/CTSS/KCNIP2 38

BP GO:2001236 regulation of extrinsic apoptotic signaling pathway 38/2475 158/18870 0.000127301628802859 0.0016872383649721 0.00122553885937084 CAV1/SP100/PYCARD/G0S2/IL1A/MIR221/IFI6/LMNA/HMOX1/THBS1/PAK5/ICAM1/RET/NRP1/TNFRSF12A/LGALS3/FAIM2/TNFAIP3/BRCA1/DEDD2/GPX1/SFRP2/HGF/BCL2L12/EYA2/SERPINE1/TMBIM1/LTBR/SNAI2/PTPRC/HMGB2/GDNF/EYA4/FADD/EYA1/COL2A1/ATF3/TRAF1 38

BP GO:0060348 bone development 48/2475 215/18870 0.000130455356851363 0.00172522899236912 0.00125313365044098 LRRC17/SHOX2/STC1/RIPPLY2/HOXA11/SP5/SERPINH1/MMP13/SFRP4/COL3A1/HOXB4/BMP2/CCN4/CHAD/LOX/ECM1/DLX5/ENG/GLI3/PHEX/PTGER4/MMP14/SNX10/SMPD3/HAS2/COL1A1/ACP5/FAM20C/RUNX2/BGN/PAPPA2/VKORC1/RGN/LTF/TYROBP/TWIST1/PLS3/LILRB1/P3H1/FOSL2/COL27A1/PTPRC/ADAMTS7/OSR2/ANXA2/EVC/COL2A1/BNC2 48

BP GO:0060272 embryonic skeletal joint morphogenesis 7/2475 11/18870 0.000134711549937459 0.00177760032049342 0.00129117397661283 SHOX2/HOXC11/HOXA11/OSR1/OSR2/NOG/COL2A1 7

BP GO:0019079 viral genome replication 33/2475 131/18870 0.000135737943698603 0.00178721625869827 0.00129815858897348 APOBEC3B/OASL/MIR221/TOP2A/CXCL8/OAS2/FAM111A/APOBEC3G/MX1/CCL2/APOBEC3C/TRIM38/OAS1/IFITM3/PLSCR1/IFI16/CCL5/APOBEC3F/IFITM2/IFIH1/OAS3/SLPI/LTF/PPIA/ISG15/TRIM6/RSAD2/BTBD17/IFI27/ZC3H12A/ISG20/APOBEC3H/BST2 33

BP GO:0048145 regulation of fibroblast proliferation 25/2475 89/18870 0.000137427291459589 0.00180524462045612 0.00131125363135974 CAV1/NGFR/ITGB3/FTH1/CD248/FBXO4/SOD2/PAWR/CD74/EGFR/CCNB1/SPHK1/FN1/PDGFA/LIF/CCNA2/CD300A/PDGFD/E2F1/S100A6/CDC6/FOSL2/CDK4/AQP1/CDK6 25

BP GO:0045123 cellular extravasation 22/2475 74/18870 0.000137708533672369 0.00180524462045612 0.00131125363135974 MDK/ICAM1/SELL/RIPK3/IL1R1/CCL2/ITGA4/PTGER4/PLVAP/BST1/F11R/SPN/PDGFD/ITGB1/PIK3CG/ITGA1/CHST2/FADD/ITGAL/PLCB1/GCNT1/FUT9 22

BP GO:0010469 regulation of signaling receptor activity 29/2475 110/18870 0.000144470215086635 0.00188975854331189 0.00137264098407047 NCF1/CACNG2/PRRT1/NRP1/CRH/CCL2/SHANK1/ERRFI1/SHISA7/DLGAP3/CNIH3/FCGR2B/DLGAP2/DLGAP1/SERPINE1/GPRC5A/PLAU/ARC/TAFA1/GREM2/ITGB1/MAPK8IP2/PPARG/RAMP3/HFE/NOG/ADRA2B/CACNG5/CACNG3 29

BP GO:0099505 regulation of presynaptic membrane potential 12/2475 29/18870 0.000150313231557695 0.00196191443972261 0.00142505198705613 KCNA1/KCNC1/GRIA4/GABBR1/GABRA5/KCNQ5/KCNA4/KCNJ11/KCNJ3/KCNC2/KCNJ9/GRIN2B 12

BP GO:0002011 morphogenesis of an epithelial sheet 19/2475 60/18870 0.000154435280104763 0.00201134364804555 0.00146095528136553 ITGA5/FERMT1/HOXB2/MIR221/MMP12/FLNA/CD44/HOXB4/CCN1/CD151/JAG1/PDPN/AJUBA/TMEFF2/RHOC/ITGB1/CARMIL2/COL5A1/DLL4 19

BP GO:0072593 reactive oxygen species metabolic process 51/2475 234/18870 0.000155031810937296 0.00201474240880416 0.00146342399797953 PXDNL/NCF1B/NCF4/H19/NCF1/IFI6/TSPO/ITGB2/CYBA/SOD2/THBS1/HBQ1/GCH1/NOX4/RIPK3/CCN1/CYP1B1/CLEC7A/RAB27A/CBR1/FOXM1/NCF2/BRCA1/F2RL1/GPX1/BST1/ACP5/HP/STK17A/FPR2/HK2/PID1/MAPT/MIR27B/HBA1/SOD3/GRIN1/LRRK2/NOS2/CD36/TYROBP/RAC2/FBLN5/ALOX5/GADD45A/PRDX4/ZC3H12A/NCF1C/UCP2/MAOB/MT3 51

BP GO:0072080 nephron tubule development 27/2475 100/18870 0.000157174119410648 0.00203377890720157 0.00147725130834463 WNT4/HOXD11/WWTR1/NPNT/HS3ST3B1/SLC22A6/PROM1/HOXA11/CITED1/STAT1/BMP2/SOX8/JAG1/FOXD1/GLI3/GREB1L/VEGFA/OSR1/LIF/TACSTD2/HS3ST3A1/GDNF/EYA1/NOG/AQP1/WNT7B/HOXB7 27

BP GO:2000177 regulation of neural precursor cell proliferation 27/2475 100/18870 0.000157174119410648 0.00203377890720157 0.00147725130834463 MDK/EMX1/FLNA/DISP3/HAPLN1/NR2E1/SPINT1/GLI3/TAFA3/TOX/VEGFA/SHCBP1/LYN/DMRTA2/LHX5/LRRK2/SOX10/GLI1/OTP/HAPLN3/TAFA1/ITGB1/WDR62/ASPM/RASSF10/PTN/DLL4 27

BP GO:0008037 cell recognition 37/2475 154/18870 0.000158585622943797 0.00204763027990227 0.00148731236187018 CDK5R1/MSN/CRTAC1/EMB/TREM2/FCGR1A/NRP1/HAVCR2/LGALS3/DSCAM/CNR1/CLEC7A/SPA17/GAP43/PLA2G5/DSCAML1/SPON2/FEZF2/FOLR1/CXCR4/TUB/CD36/IGSF9/CNTN6/ZP3/CASP3/PRF1/CNTNAP2/CNTN4/NEXN/COLEC11/EPHA3/PALLD/LBP/PTX3/OPCML/COLEC12 37

BP GO:0009636 response to toxic substance 55/2475 258/18870 0.00016135148536108 0.00207887192727022 0.0015100049782041 CES1/MDM2/KCNC1/SLC47A2/MT1M/PXDNL/SLC11A1/H19/SLC47A1/TTPA/MT1A/IPCEF1/SOD2/MT2A/PRKCG/MTARC2/MT1DP/HBQ1/NEFL/CLDN1/GCH1/SLC39A8/AHR/PON1/CYP1B1/MT1E/TYMS/S100A9/PTGS1/TLR2/GPX1/SCN2B/CCL5/HP/DHRS2/LYN/HBA1/ALOX5AP/SOD3/PTGES/GPX7/MGST2/MT1H/CD36/FBLN5/SDC1/BRIP1/KCNC2/SELENOF/PRDX4/SLC22A18/CDK1/GPX8/MAOB/MT3 55

BP GO:0050000 chromosome localization 30/2475 116/18870 0.000162535256090186 0.00208470409008524 0.00151424121554458 NEK2/CENPF/SPC24/KIFC1/SGO1/KIF14/LMNA/ECT2/CDCA5/KNTC1/PSRC1/CCNB1/FAM83D/ANKRD53/DLGAP5/BIRC5/DSN1/ZWILCH/NUF2/KIF18A/AURKB/KIF2C/CDT1/NDE1/CENPE/CDCA8/NDC80/KNL1/CHMP4BP1/SPAG5 30

BP GO:0032613 interleukin-10 production 20/2475 65/18870 0.000162845805837771 0.00208470409008524 0.00151424121554458 PYCARD/RBM47/TREM2/BCL3/LILRA5/CLEC7A/CD274/HLA-DRB1/JAK3/TLR2/IDO1/F2RL1/PDCD1LG2/FCGR2B/SASH3/HGF/ISG15/TYROBP/LILRB1/IL6 20

BP GO:0032653 regulation of interleukin-10 production 20/2475 65/18870 0.000162845805837771 0.00208470409008524 0.00151424121554458 PYCARD/RBM47/TREM2/BCL3/LILRA5/CLEC7A/CD274/HLA-DRB1/JAK3/TLR2/IDO1/F2RL1/PDCD1LG2/FCGR2B/SASH3/HGF/ISG15/TYROBP/LILRB1/IL6 20

BP GO:0002752 cell surface pattern recognition receptor signaling pathway 21/2475 70/18870 0.000168262748969485 0.00214946711662295 0.00156128234934059 LY96/TLR1/CYBA/TREM2/OAS1/RAB7B/TNFAIP3/TLR2/F2RL1/NMI/SCIMP/LYN/CD14/LTF/LILRA2/IFI35/COLEC11/PRKCE/LBP/IRAK2/MYD88 21

BP GO:0097191 extrinsic apoptotic signaling pathway 50/2475 229/18870 0.000170827620770253 0.00216725122190383 0.00157419997411332 CAV1/SP100/TRADD/PYCARD/G0S2/NFKBIZ/SPI1/IL1A/MIR221/IFI6/LMNA/FAS/HMOX1/THBS1/CD70/PAK5/ICAM1/RET/NRP1/TNFRSF12A/LGALS3/BCL2A1/FAIM2/TNFRSF1A/TNFAIP3/TNFRSF10C/CASP8/BRCA1/DEDD2/GPX1/SFRP2/KRT18/HGF/BCL2L12/EYA2/SERPINE1/TMBIM1/LTBR/SNAI2/PTPRC/IFI27/HMGB2/GDNF/TGFB2/EYA4/FADD/EYA1/COL2A1/ATF3/TRAF1 50

BP GO:0032611 interleukin-1 beta production 29/2475 111/18870 0.000171400091979133 0.00216725122190383 0.00157419997411332 GBP5/PYCARD/GSDMD/PYDC1/SERPINB1/TREM2/ELF4/CD33/HSPB1/LILRA5/CLEC7A/S1PR3/CASP1/TNFAIP3/CASP8/CARD16/IFI16/F2RL1/SPHK1/TLR8/FZD5/ACP5/MIR27B/CD36/TYROBP/LILRA2/IL6/ZC3H12A/MYD88 29

BP GO:0032651 regulation of interleukin-1 beta production 29/2475 111/18870 0.000171400091979133 0.00216725122190383 0.00157419997411332 GBP5/PYCARD/GSDMD/PYDC1/SERPINB1/TREM2/ELF4/CD33/HSPB1/LILRA5/CLEC7A/S1PR3/CASP1/TNFAIP3/CASP8/CARD16/IFI16/F2RL1/SPHK1/TLR8/FZD5/ACP5/MIR27B/CD36/TYROBP/LILRA2/IL6/ZC3H12A/MYD88 29

BP GO:1902106 negative regulation of leukocyte differentiation 29/2475 111/18870 0.000171400091979133 0.00216725122190383 0.00157419997411332 LRRC17/ASCL2/MDK/LOXL3/ANXA1/FOXJ1/LILRB3/CD74/SOCS1/RUNX1/GLI3/JAK3/FCGR2B/LYN/FSTL3/RUNX3/LTF/TNFRSF11B/LILRB1/TNFAIP6/TMEM176A/FGL2/HOXA7/ERFE/C1QC/ZC3H12A/IRF1/TMEM176B/CDK6 29

BP GO:0007193 adenylate cyclase-inhibiting G protein-coupled receptor signaling pathway 23/2475 80/18870 0.000171459748568341 0.00216725122190383 0.00157419997411332 AKAP5/HTR1A/PDE2A/ITGB3/SSTR2/GABBR2/HTR1D/GABBR1/FLNA/HTR5A/S1PR3/CHRM1/GRM5/FPR2/GRM2/P2RY1/HRH3/GRM1/NPY2R/ADORA1/CHRM4/MARCO/RGS1 23

BP GO:0043122 regulation of canonical NF-kappaB signal transduction 56/2475 265/18870 0.000179846670213146 0.00225511786394966 0.00163802260078254 TRADD/PYCARD/IL1A/TRAF5/ECT2/LITAF/HMOX1/TNFRSF19/TREM2/CD40/TFRC/S100A12/SLC39A8/CD74/TRIM21/FLNA/HSPB1/RHOH/S100A13/CLEC7A/STAT1/TRIM38/TRIM22/TIFA/BIRC3/PRKCB/NEK6/ECM1/SECTM1/ROR1/TNFRSF1A/CASP1/HLA-DRB1/TNFAIP3/CASP8/ANGPT1/CARD16/F2RL1/AJUBA/S100B/LTF/CD36/LGALS1/TRIM6/TRIM5/S100A4/LTBR/RHOC/CARD19/ALPK1/PRKCE/ZC3H12A/FADD/MYD88/TRAF1/BST2 56

BP GO:0010755 regulation of plasminogen activation 9/2475 18/18870 0.00017991363371617 0.00225511786394966 0.00163802260078254 PLAT/THBS1/RUNX1/S100A10/SERPINE1/PLAU/CTSZ/PLAUR/ANXA2 9

BP GO:0016264 gap junction assembly 9/2475 18/18870 0.00017991363371617 0.00225511786394966 0.00163802260078254 CAV1/ACE/HOPX/APLNR/TBX5/GJB2/GJC1/CNTNAP2/GJD3 9

BP GO:0060263 regulation of respiratory burst 9/2475 18/18870 0.00017991363371617 0.00225511786394966 0.00163802260078254 NCF1/IGHA1/CLEC7A/SLAMF8/IGHA2/DUSP10/JCHAIN/RAC2/LBP 9

BP GO:0007584 response to nutrient 37/2475 155/18870 0.00018240937462792 0.00227689373236181 0.00165383967410761 F5/MDM2/NFKBIZ/IL1A/TSPO/STC1/ITGA2/SPP1/SOD2/CD40/TFRC/SHANK2/CYP1B1/CNR1/STAT1/PRKCB/TYMS/PHEX/CXCL10/FOLR1/GPX1/SFRP2/COL1A1/FES/RUNX2/CYP27B1/TNC/C2/IGFBP2/TNFRSF11B/BRIP1/SNAI2/POSTN/MN1/GATA4/LRAT/PPARG 37

BP GO:0045807 positive regulation of endocytosis 37/2475 155/18870 0.00018240937462792 0.00227689373236181 0.00165383967410761 APLN/PYCARD/SLC11A1/CYBA/ITGA2/TREM2/FCGR1A/SFRP4/CCL2/CLEC7A/RAB27A/CD151/PLA2G5/CD63/ANGPT1/CD300LF/VEGFA/F2RL1/FCGR2B/IL2RG/FPR2/TUB/LRRK2/SERPINE1/CD14/CD36/C2/FCER1G/C3/PTPRC/COLEC11/B2M/CALY/PTX3/HFE/ANXA2/IL2RB 37

BP GO:0032755 positive regulation of interleukin-6 production 27/2475 101/18870 0.000188078063026812 0.00234278151537963 0.00170169778362106 PYCARD/TLR1/IL1A/CYBA/CD74/LILRA5/ARID5A/CLEC7A/IL17RC/SPON2/RAB7B/TLR2/F2RL1/SCIMP/IFIH1/TLR8/LILRB2/NOS2/CD36/TYROBP/TWIST1/LILRA2/IL1RAP/UNC93B1/IL6/LBP/MYD88 27

BP GO:0002221 pattern recognition receptor signaling pathway 51/2475 236/18870 0.000193315337728757 0.00240303372199473 0.00174546244788109 CAV1/OASL/LY96/GBP5/IRF7/PYCARD/TLR1/NFKBIZ/FOSL1/CYBA/GBP2/TREM2/CGAS/CD40/LILRA4/HAVCR2/TIFA/OAS1/BIRC3/RAB7B/CASP1/TNFAIP3/CD300LF/TLR2/F2RL1/NMI/SCIMP/IFIH1/TLR8/LYN/OAS3/CD14/LTF/ZDHHC12/SLC15A3/CD36/CD300A/LILRA2/IFI35/RSAD2/ALPK1/UNC93B1/RNF135/COLEC11/PRKCE/LBP/IRAK2/IRF1/MYD88/CTSS/COLEC12 51

BP GO:0046879 hormone secretion 63/2475 308/18870 0.000194677566544617 0.00241496716845843 0.00175413040059667 HTR1A/STX1A/TSPO/SLC16A10/SPP1/CHGA/VGF/IL1RN/ANXA1/ABCC8/C1QTNF1/GABBR1/ACVR1C/VAMP8/KCNB1/CRH/CNR1/GLUD1/RAB3A/PRKCB/RAPGEF4/NNAT/FOXD1/BRSK2/HLA-DRB1/SMPD3/SYT7/F2RL1/CCL5/VIP/IL11/GPR27/LYN/HTR2C/LIF/KCNJ11/SNAP25/NOS2/P2RY1/F2RL2/NKX6-1/VSNL1/CRY2/OSM/GCK/ALOX5/PCLO/GLP1R/TUNAR/TRH/ADORA1/PRKCE/IL6/PPARG/GAL/HFE/TCIRG1/AQP1/CPLX1/GRP/UCP2/PLCB1/RIMS2 63

BP GO:0016032 viral process 83/2475 432/18870 0.000196762314505555 0.00243579574493062 0.0017692594009691 APOBEC3B/CAV1/OASL/ITGA5/IRF7/ITGB3/LEF1/MIR221/PARP9/TNFRSF4/SIGLEC1/TOP2A/CXCL8/OAS2/KPNA2/ITGA2/FAM111A/TFRC/CLDN1/ICAM1/NRP1/APOBEC3G/VAMP8/CD74/TRIM21/EGFR/MX1/CCL2/APOBEC3C/STAT1/TRIM38/TRIM22/EDEM2/OAS1/CIITA/IFITM3/HLA-DRB1/ZFP36/PLSCR1/IFI16/CTSB/HTR2A/CAV2/CCL5/APOBEC3F/CXCR4/CLEC5A/IFITM2/IFIH1/CTSL/MGAT4C/F11R/OAS3/PCSK5/SLPI/LTF/PPIA/ANPEP/LAMP3/ISG15/NECTIN2/CCR5/LGALS1/MDFIC/TRIM6/TRIM5/RSAD2/EPHA2/EFNB2/BTBD17/ITGB1/IFI27/DPP4/CDK1/JPT2/PTX3/ZC3H12A/CHMP4BP1/ISG20/TNFRSF14/APOBEC3H/SLC1A5/BST2 83

BP GO:0043491 phosphatidylinositol 3-kinase/protein kinase B signal transduction 60/2475 290/18870 0.000198150678878811 0.0024479355473012 0.00177807724192097 DDIT3/IL18/SEMA3E/H19/NCF1/MIR221/PLK3/ROR2/SPRY2/C1QTNF1/THBS1/TREM2/CD40/RET/F3/NOX4/EGFR/WNT16/IGFBP5/LOX/HCST/ENG/NTS/ROR1/SMPD3/ANGPT1/RCN3/F2RL1/GPX1/HTR2A/CCL5/CHI3L1/IGF2/HGF/FN1/DCN/BTN2A2/CSF3/PDGFA/TMEM100/GRM2/TWIST1/FAM110C/PDGFD/RRAS/EPHA2/OSM/MYO16/ERFE/GATA4/ITGB1/CAVIN3/NTRK1/PIK3CG/STOX1/FCGR3A/TGFB2/RAMP3/HCLS1/GDF15 60

BP GO:0002285 lymphocyte activation involved in immune response 47/2475 213/18870 0.000203591520663658 0.00250998663257619 0.00182314853584683 KLRC2/ASCL2/MDK/BATF/IL18/SLC11A1/LEF1/NFKBIZ/SPI1/NKG7/RELB/LOXL3/LCP1/ANXA1/CD40/TFRC/ICAM1/BCL3/CD74/HAVCR2/LGALS3/RAB27A/CD180/GAPT/PTGER4/HLA-DRB1/JAK3/F2RL1/FCGR2B/EXO1/SOCS3/SPN/HLA-DRA/LILRB1/LGALS1/FGL2/FCER1G/HLA-DMB/PTPRC/IL6/FCGR3A/ZC3H12A/ITGAL/SWAP70/CLCF1/GPR183/HLA-F 47

BP GO:0048265 response to pain 13/2475 34/18870 0.000207805746801597 0.0025462565383608 0.00184949346723742 TSPO/PRKCG/THBS1/GCH1/RET/PIRT/THBS4/SCN3A/AQP9/NTRK1/VWA1/NMUR2/ADAM11 13

BP GO:0060317 cardiac epithelial to mesenchymal transition 13/2475 34/18870 0.000207805746801597 0.0025462565383608 0.00184949346723742 EMP2/SNAI1/WNT16/BMP2/JAG1/ENG/HAS2/TMEM100/TWIST1/SNAI2/TGFB2/SPRY1/NOG 13

BP GO:1901658 glycosyl compound catabolic process 13/2475 34/18870 0.000207805746801597 0.0025462565383608 0.00184949346723742 APOBEC3B/GLA/FUCA2/DPYD/APOBEC3G/ADA2/APOBEC3C/GUSB/UPP1/GDA/APOBEC3F/FUCA1/APOBEC3H 13

BP GO:0007405 neuroblast proliferation 23/2475 81/18870 0.000210102212251475 0.00256915210256182 0.00186612384040703 KCNA1/LEF1/FGF13/ACSL6/HAPLN1/TEAD3/NR2E1/GLI3/TAFA3/VEGFA/NEUROD4/DMRTA2/LHX5/LRRK2/SOX10/OTP/HAPLN3/TAFA1/NDE1/ITGB1/WDR62/ASPM/PTN 23

BP GO:0033630 positive regulation of cell adhesion mediated by integrin 10/2475 22/18870 0.00021235109008562 0.00259137387169525 0.00188226479722876 FERMT1/ITGB3/CXCL13/RET/CD3E/CCL5/SFRP2/LIF/ADAM9/TGFB2 10

BP GO:0002269 leukocyte activation involved in inflammatory response 17/2475 52/18870 0.000220052069989877 0.00267245134647758 0.00194115605884789 ITGB2/IFNGR2/TREM2/CTSC/TAFA3/C5AR1/TLR2/SPHK1/SCNN1B/MAPT/LRRK2/TYROBP/CST7/PTPRC/IL6/C1QA/MYD88 17

BP GO:0010955 negative regulation of protein processing 12/2475 30/18870 0.000220775461001479 0.00267245134647758 0.00194115605884789 MDM2/PLAT/THBS1/CARD16/IL1R2/BCL2L12/LRRK2/SERPINE1/PLAU/CST7/GAS1/CTSZ 12

BP GO:0035640 exploration behavior 12/2475 30/18870 0.000220775461001479 0.00267245134647758 0.00194115605884789 HTR1A/SLC4A10/ITGA3/CRH/BRINP1/LRRK2/TNR/GAD1/JPH3/DPP4/PRKCE/NOG 12

BP GO:1903318 negative regulation of protein maturation 12/2475 30/18870 0.000220775461001479 0.00267245134647758 0.00194115605884789 MDM2/PLAT/THBS1/CARD16/IL1R2/BCL2L12/LRRK2/SERPINE1/PLAU/CST7/GAS1/CTSZ 12

BP GO:0099084 postsynaptic specialization organization 15/2475 43/18870 0.000228108226761663 0.00275565753214693 0.00200159352636571 ITGB3/NTNG2/SHANK2/C1QL3/GAP43/SHANK1/OPHN1/NRXN1/ABI3/ZDHHC12/LILRB2/CBLN1/IL1RAP/NPTX1/SPTBN2 15

BP GO:0072498 embryonic skeletal joint development 8/2475 15/18870 0.000236025944980203 0.00284558187482156 0.00206691078007394 SHOX2/HOXC11/HOXA11/SLC2A10/OSR1/OSR2/NOG/COL2A1 8

BP GO:0032355 response to estradiol 29/2475 113/18870 0.000239031827092819 0.00287604627227512 0.00208903883481816 SSTR3/SSTR2/ITGA2/ANXA1/MMP2/STXBP1/EGFR/CYP1B1/HTR5A/AREG/OXTR/CASP8/GPX1/SOCS2/MYOD1/COL1A1/GJB2/CCNA2/ADCYAP1R1/PCNA/IGFBP2/KIF18A/NR2F2/CASP3/POU4F1/POSTN/SSTR1/RAMP3/EZH2 29

BP GO:0007369 gastrulation 44/2475 197/18870 0.000240797560716739 0.0028914971090866 0.00210026167168305 COL8A1/ITGA5/APLN/ITGB3/SNAI1/LEF1/MIR221/ITGB2/DUSP5/ITGA2/IL1RN/HOXA11/ITGA3/FZD7/MMP2/DUSP4/TWSG1/APLNR/COL12A1/MMP9/ITGB4/ITGA4/ATOH8/MMP14/GSC/DKK1/SFRP2/FN1/ETV2/EYA2/OSR1/COL4A2/COL6A1/LAMB1/CRB2/EPHA2/COL5A2/ITGA7/ITGB1/COL5A1/TBX19/EYA1/NOG/NODAL 44

BP GO:0002761 regulation of myeloid leukocyte differentiation 31/2475 124/18870 0.000246899858805862 0.00295885579295488 0.00214918818160035 LRRC17/IRF7/LEF1/ROR2/TREM2/LILRB3/CD74/RUNX1/HLA-DRB1/CASP8/CD101/FES/LYN/FSTL3/LIF/CEBPB/LTF/TYROBP/EVI2B/TNFRSF11B/LILRB1/TNFAIP6/HOXA7/POU4F1/ERFE/C1QC/FADD/HCLS1/CCR1/CDK6/CAMK4 31

BP GO:0051303 establishment of chromosome localization 28/2475 108/18870 0.000252910360840197 0.00302484822008873 0.00219712230019068 NEK2/CENPF/SPC24/KIFC1/SGO1/KIF14/ECT2/CDCA5/KNTC1/PSRC1/CCNB1/FAM83D/ANKRD53/BIRC5/DSN1/ZWILCH/NUF2/KIF18A/AURKB/KIF2C/CDT1/NDE1/CENPE/CDCA8/NDC80/KNL1/CHMP4BP1/SPAG5 28

BP GO:0007409 axonogenesis 85/2475 448/18870 0.000255023125188135 0.00304405336705678 0.00221107210977178 SHOX2/CDK5R2/MCF2/EFNB1/NGFR/LGR6/UNC5A/CDK5R1/SEMA3E/SPP1/FGF13/EMB/VASP/HOXA2/NEFL/NTNG2/CHRNB2/RET/NRP1/SEMA3A/ATP8A2/EPHA10/CDH4/STXBP1/SEMA3D/SLITRK4/SLIT1/LAMA2/DSCAM/RTN4R/RAB3A/NR2E1/GAP43/DLX5/DSCAML1/ITGA4/OPHN1/FOXD1/GLI3/BRSK2/FEZF2/NRXN1/VEGFA/NEFH/L1CAM/SEMA6B/FN1/ECE1/S100B/MAPT/SNAP25/CHODL/PAK3/SLITRK1/TNR/IGSF9/CNTN6/EFNA4/CCK/SEMA3F/NRXN3/NKX6-1/DRAXIN/FSTL4/NPTX1/EFNB2/S100A6/TBR1/POU4F1/CNTN4/EPHB6/NEXN/ITGB1/LRTM2/EPHA3/TRPV2/PALLD/GDNF/NTRK1/ANK3/SLITRK5/NOG/ISLR2/NTNG1/MT3 85

BP GO:0043086 negative regulation of catalytic activity 90/2475 480/18870 0.000259151855072511 0.00308553412106089 0.0022412019817587 AKAP5/CAV1/TIMP1/MAD2L1/PPP1R15A/PYCARD/H19/WWTR1/GLA/SLN/IFI6/PARP9/GABBR2/PTPRT/SPRY2/PYDC1/ANXA1/SERPINB1/THBS1/SERPINH1/PIF1/MIR210/CD44/LGALS3/HSPB1/BMP2/MMP9/ERRFI1/ECM1/KLRC4-KLRK1/PKIB/SPRY4/SERPINA5/ANGPTL4/VEGFA/PPP4R4/GZMA/ZFP36/CARD16/SLC8A3/IFI16/GPX1/SERPINB8/DPEP1/HGF/DUSP10/HP/LYN/RPS6KA1/MAPT/BCL2L12/MIR27B/CST3/CYP27B1/TRIB3/LRRK2/SERPINE1/CAMK2A/GPRC5A/DTX3L/SERPING1/LTF/PPIA/TMBIM1/LAMP3/GRM2/CD300A/ADGRV1/NR2F2/CRB2/SFN/CST7/APOC1/WARS1/SERPINA1/CRY2/CSTA/PLK1/GADD45A/PTPRC/PPARG/TGFB2/PLAUR/SERPINA3/SPRY1/PTX3/SERPINI1/AQP1/LATS2/MT3 90

BP GO:0006953 acute-phase response 16/2475 48/18870 0.000260040017531114 0.00308553412106089 0.0022412019817587 IL1A/TFRC/CNR1/PLSCR1/SAA2/HP/FN1/PTGES/CEBPB/SERPINA1/IL6/LBP/SERPINA3/HFE/SAA1/CD163 16

BP GO:0032964 collagen biosynthetic process 16/2475 48/18870 0.000260040017531114 0.00308553412106089 0.0022412019817587 WNT4/ITGA2/SERPINH1/TRAM2/RUNX1/CIITA/ENG/RCN3/COL1A1/EMILIN1/ADAMTS3/VIM/FOSL2/COL5A1/IL6/PCOLCE 16

BP GO:0016485 protein processing 52/2475 245/18870 0.000268318163766343 0.00317747979339866 0.00230798744416891 MDM2/THBD/C1RL/PLAT/MMEL1/ACE/FAM111A/C1R/THBS1/F3/CPM/AEBP1/RUNX1/S100A10/CASP7/ENPEP/GLI3/PHEX/CASP1/MMP14/CASP8/BACE2/CARD16/ATP23/IFI16/GRIN2A/CTSL/IL1R2/CASP6/SRGN/ECE1/BCL2L12/PCSK5/PCSK6/LRRK2/SERPINE1/PLAU/ADAMTS2/PCSK2/CASP3/CST7/ADAMTS3/PGK1/ADAM19/DPP4/GAS1/CTSZ/PLAUR/FADD/CASP4/ANXA2/CTSS 52

BP GO:0031214 biomineral tissue development 40/2475 175/18870 0.000272873144780712 0.00322505976626653 0.00234254753175817 WNT4/ROR2/SPP1/MGP/MMP13/SBNO2/CCN1/BMP2/LOX/ECM1/PHEX/SNX10/SMPD3/NELL1/ENPP1/COL1A1/FAM20C/SRGN/OSR1/CYP27B1/COL1A2/TENT5A/CEBPB/LTF/ISG15/TWIST1/ADGRV1/SGMS2/ALOX5/ASPN/FOSL2/TBX1/FAM20A/PTHLH/WNT10B/OSR2/TCIRG1/IBSP/PTN/CCR1 40

BP GO:0060993 kidney morphogenesis 26/2475 98/18870 0.000279510873837484 0.00329702020927358 0.00239481656562651 WNT4/HOXD11/WWTR1/NPNT/HS3ST3B1/HOXA11/FOXJ1/CITED1/STAT1/BMP2/SOX8/FOXD1/GLI3/GREB1L/VEGFA/OSR1/LRRK2/LIF/TACSTD2/HS3ST3A1/GDNF/EYA1/NOG/WNT7B/HOXB7/GCNT1 26

BP GO:0052547 regulation of peptidase activity 63/2475 312/18870 0.00028199119601438 0.00331450594500004 0.0024075174673262 CAV1/TIMP1/NGFR/PYCARD/HIP1R/IFI6/CTSK/LCK/PSMB8/FAS/SERPINB1/THBS1/ST20/SERPINH1/ACVR1C/F3/CD44/CLEC7A/MMP9/BIRC3/S100A8/ECM1/S100A9/CASP1/SERPINA5/CASP8/VEGFA/CARD16/RCN3/IFI16/GPX1/GRIN2A/SERPINB8/DPEP1/HGF/CTSL/LYN/RPS6KA1/MAPT/BCL2L12/MIR27B/CST3/GRIN1/SERPINE1/SERPING1/PSMB9/LTF/LAMP3/LAPTM5/CRB2/SFN/CST7/SERPINA1/CSTA/PPARG/PLAUR/SERPINA3/FADD/SERPINI1/AQP1/GRIN2B/NODAL/CTSS 63

BP GO:0045580 regulation of T cell differentiation 41/2475 181/18870 0.000282097358077119 0.00331450594500004 0.0024075174673262 ASCL2/IL7R/MDK/BATF/IL18/LEF1/NFKBIZ/LOXL3/FANCD2/ANXA1/ACTL6B/TESPA1/FOXJ1/CD74/SOCS1/RHOH/RUNX1/HLA-DOA/GLI3/TOX/HLA-DRB1/JAK3/IL2RG/SASH3/DUSP10/BTN2A2/RUNX3/LILRB2/HLA-DRA/TCF7/FGL2/CD2/PTPRC/WNT10B/IL2RA/ZC3H12A/PIK3R6/IRF1/IL4I1/CAMK4/FANCA 41

BP GO:0031630 regulation of synaptic vesicle fusion to presynaptic active zone membrane 7/2475 12/18870 0.000286601462992674 0.00334127220156896 0.00242695934834675 CPLX2/DOC2A/STXBP1/RAB3A/ERC2/SYT1/CPLX1 7

BP GO:0033605 positive regulation of catecholamine secretion 7/2475 12/18870 0.000286601462992674 0.00334127220156896 0.00242695934834675 STX1A/CHRNB2/KCNB1/OXTR/VIP/SYT1/GDNF 7

BP GO:0044341 sodium-dependent phosphate transport 7/2475 12/18870 0.000286601462992674 0.00334127220156896 0.00242695934834675 SLC17A8/SFRP4/SLC17A7/SLC34A2/CEBPB/SLC17A6/CRY2 7

BP GO:1901632 regulation of synaptic vesicle membrane organization 7/2475 12/18870 0.000286601462992674 0.00334127220156896 0.00242695934834675 CPLX2/DOC2A/STXBP1/RAB3A/ERC2/SYT1/CPLX1 7

BP GO:0051968 positive regulation of synaptic transmission, glutamatergic 13/2475 35/18870 0.000291182326306913 0.00338809823090446 0.00246097180312023 CACNG2/ROR2/STXBP1/CCL2/OXTR/NRXN1/GRIN2A/GRIN1/TNR/NTRK1/GRIN2B/CACNG5/CACNG3 13

BP GO:0021537 telencephalon development 57/2475 276/18870 0.000296099869123352 0.00343865302556404 0.00249769267591988 KCNA1/KCNC1/CDK5R2/MDK/EMX1/CDK5R1/KIF14/LEF1/SLC32A1/KIF26A/FGF13/NEFL/SEMA3A/NRG3/COL3A1/FLNA/EGFR/DLX2/TACC3/RTN4R/HTR5A/BMP2/NR2E1/DLX5/ATF5/OXTR/GLI3/SCN2A/FEZF2/GSX2/RTN4RL1/CXCR4/SEMA6B/DMRTA2/LHX5/LRRK2/TNR/LAMB1/CASP3/DRAXIN/CNTNAP2/PHACTR1/NDE1/TBR1/ALDH1A3/ZIC1/WDR62/LHX6/NEUROD6/TUBA1A/SLITRK5/ASPM/AQP1/EZH2/PLCB1/MGARP/CDK6 57

BP GO:1903522 regulation of blood circulation 54/2475 258/18870 0.000298413563482773 0.00345883211419029 0.0025123499156861 CAV1/HTR1A/MDM2/SHOX2/APLN/MYH7/NKX2-5/BVES/STC1/CHGA/ACE/TMIGD3/FGF13/GCH1/HOPX/ATP2B3/MMP2/CTNNA3/FLNA/DSG2/OXTR/SLC8A3/HTR2A/HSPB7/SCN2B/RYR2/HRH1/TBX5/ASIC2/ECE1/SLC8A2/KCNE5/KCNE3/KCNJ3/GJC1/DSC2/BDKRB2/KCNJ12/SCN3B/ADRA1D/GLP1R/GATA4/DES/ADORA1/ATP2B2/PIK3CG/TGFB2/RGS4/ZC3H12A/KCNE4/GJD3/ADM/ADRA2B/KCNIP2 54

BP GO:0001892 embryonic placenta development 24/2475 88/18870 0.000302259557062914 0.00349665969288195 0.00253982627504645 ASCL2/SNAI1/RSPO3/LEF1/E2F7/TTPA/EGFR/CITED1/E2F8/ST14/CCN1/SPINT1/CASP8/IGF2/FZD5/SOCS3/LIF/CEBPB/NR2F2/PHLDA2/PLK4/ADM/WNT7B/NODAL 24

BP GO:0043268 positive regulation of potassium ion transport 15/2475 44/18870 0.000304502799688293 0.00350021904278224 0.00254241163684681 AKAP6/KCNC1/RGS7/ABCC8/TREM2/FLNA/KCNMB1/KCNN4/KCNE5/KCNC2/LRRC55/ADORA1/FXYD2/GAL/KCNIP2 15

BP GO:0050691 regulation of defense response to virus by host 15/2475 44/18870 0.000304502799688293 0.00350021904278224 0.00254241163684681 PYCARD/PARP9/MMP12/CGAS/APOBEC3G/STAT1/TRIM22/TNFAIP3/MICB/APOBEC3F/DTX3L/LILRB1/TRIM6/ZDHHC1/ZC3H12A 15

BP GO:2000300 regulation of synaptic vesicle exocytosis 15/2475 44/18870 0.000304502799688293 0.00350021904278224 0.00254241163684681 SYN1/PRKCG/RIMS1/RAB3A/PRKCB/SYT1/SYT4/LRRK2/RAP1B/PPFIA2/SV2B/P2RY1/SYP/RIMS3/RIMS2 15

BP GO:0009914 hormone transport 64/2475 319/18870 0.000304899160455548 0.00350021904278224 0.00254241163684681 HTR1A/STX1A/CRYM/TSPO/SLC16A10/SPP1/CHGA/VGF/IL1RN/ANXA1/ABCC8/C1QTNF1/GABBR1/ACVR1C/VAMP8/KCNB1/CRH/CNR1/GLUD1/RAB3A/PRKCB/RAPGEF4/NNAT/FOXD1/BRSK2/HLA-DRB1/SMPD3/SYT7/F2RL1/CCL5/VIP/IL11/GPR27/LYN/HTR2C/LIF/KCNJ11/SNAP25/NOS2/P2RY1/F2RL2/NKX6-1/VSNL1/CRY2/OSM/GCK/ALOX5/PCLO/GLP1R/TUNAR/TRH/ADORA1/PRKCE/IL6/PPARG/GAL/HFE/TCIRG1/AQP1/CPLX1/GRP/UCP2/PLCB1/RIMS2 64

BP GO:0002718 regulation of cytokine production involved in immune response 30/2475 120/18870 0.000308266127167789 0.00351868788501028 0.00255582662568771 PYCARD/IL18/LITAF/CD74/IL1R1/ARID5A/CLEC7A/SPON2/ANGPT1/JAK3/F2RL1/SCIMP/SASH3/FZD5/ACP5/CD36/LAPTM5/TWIST1/LILRB1/TRIM6/HLA-A/RSAD2/B2M/IL6/TGFB2/TNFRSF14/HFE/MYD88/HLA-F/BST2 30

BP GO:0030326 embryonic limb morphogenesis 30/2475 120/18870 0.000308266127167789 0.00351868788501028 0.00255582662568771 SHOX2/SP8/HAND2/PITX1/LEF1/HOXC11/HOXA11/FREM2/HOXD9/MAP3K20/DLX5/GLI3/HOXA9/DKK1/SFRP2/TBX5/RUNX2/ECE1/OSR1/HOXD10/TWIST1/HOXA10/HOXC10/RDH10/TGFB2/OSR2/NOG/EN1/HOXD13/FZD6 30

BP GO:0035113 embryonic appendage morphogenesis 30/2475 120/18870 0.000308266127167789 0.00351868788501028 0.00255582662568771 SHOX2/SP8/HAND2/PITX1/LEF1/HOXC11/HOXA11/FREM2/HOXD9/MAP3K20/DLX5/GLI3/HOXA9/DKK1/SFRP2/TBX5/RUNX2/ECE1/OSR1/HOXD10/TWIST1/HOXA10/HOXC10/RDH10/TGFB2/OSR2/NOG/EN1/HOXD13/FZD6 30

BP GO:0051965 positive regulation of synapse assembly 19/2475 63/18870 0.000313843391960126 0.00357555166096508 0.00259713007668192 IL1RAPL1/LINGO2/ADGRB3/SLITRK4/CUX2/SRPX2/OXTR/LRRTM3/CLSTN2/NRXN1/CBLN2/ASIC2/SLITRK1/CBLN1/IL1RAP/AMIGO2/LRTM2/NTRK1/SLITRK5 19

BP GO:0042100 B cell proliferation 27/2475 104/18870 0.000315628162954151 0.00358907479238015 0.00260695270956748 IL7R/LEF1/NFKBIZ/TNFRSF4/CD40/CD70/TFRC/PAWR/CHRNB2/VAV3/CD74/AHR/CD79A/CD180/GAPT/FCGR2B/SASH3/BST1/LYN/CD300A/TYROBP/CASP3/IL9/FOSL2/PTPRC/CLCF1/GPR183 27

BP GO:0045921 positive regulation of exocytosis 22/2475 78/18870 0.000317325788382631 0.00358981330561697 0.00260748913446669 KLRC2/CDK5R2/STX1A/ITGB2/VAMP8/KCNB1/STXBP1/RAB27A/RAB3A/S100A10/SMPD3/SYT7/F2RL1/SCAMP5/SYT1/SYT4/ZP3/SDC4/SDC1/VSNL1/ANXA2/HLA-F 22

BP GO:0001562 response to protozoan 12/2475 31/18870 0.000317486819667324 0.00358981330561697 0.00260748913446669 BATF/SLC11A1/NKG7/GBP2/CD40/BCL3/GBP1/CLEC7A/GBP4/BATF2/SPN/MYD88 12

BP GO:1902042 negative regulation of extrinsic apoptotic signaling pathway via death domain receptors 12/2475 31/18870 0.000317486819667324 0.00358981330561697 0.00260748913446669 MIR221/HMOX1/ICAM1/FAIM2/TNFAIP3/BRCA1/GPX1/SFRP2/HGF/SERPINE1/TMBIM1/HMGB2 12

BP GO:0030193 regulation of blood coagulation 20/2475 68/18870 0.000319525624603999 0.00359930928728408 0.00261438661545249 CAV1/THBD/PLAT/C1QTNF1/THBS1/F3/F2RL1/EMILIN2/SERPINE1/PDGFA/VKORC1/SERPING1/PLAU/CD36/EMILIN1/PROS1/FAP/TFPI/PLAUR/ANXA2 20

BP GO:2000379 positive regulation of reactive oxygen species metabolic process 20/2475 68/18870 0.000319525624603999 0.00359930928728408 0.00261438661545249 H19/TSPO/ITGB2/CYBA/SOD2/THBS1/RIPK3/CYP1B1/CLEC7A/RAB27A/CBR1/F2RL1/FPR2/PID1/MAPT/GRIN1/CD36/TYROBP/GADD45A/ZC3H12A 20

BP GO:0032732 positive regulation of interleukin-1 production 21/2475 73/18870 0.000320410037270169 0.00360251285350205 0.00261671355097636 GBP5/PYCARD/GSDMD/PYDC1/HAVCR2/HSPB1/S100A13/LILRA5/CLEC7A/CASP1/CASP8/IFI16/F2RL1/TLR8/FZD5/CD36/TYROBP/LILRA2/IL6/SAA1/MYD88 21

BP GO:0031629 synaptic vesicle fusion to presynaptic active zone membrane 10/2475 23/18870 0.000331700997714625 0.00372249119678245 0.00270386076444996 STX1A/STX1B/CPLX2/DOC2A/STXBP1/RAB3A/ERC2/SYT1/SNAP25/CPLX1 10

BP GO:0060384 innervation 11/2475 27/18870 0.000333589292643746 0.00373669797207658 0.00271417996744509 PRKCG/RET/NRP1/SEMA3A/GABRA5/ITGA4/ECE1/GABRB2/NPTX1/POU4F1/NTRK1 11

BP GO:0098815 modulation of excitatory postsynaptic potential 16/2475 49/18870 0.000339577712150107 0.00379669382448649 0.00275775842681152 STX1A/STX1B/RIMS1/CUX2/SHANK1/NRXN1/SLC8A3/GRIN2A/SLC8A2/GRIN1/LRRK2/CBLN1/CELF4/RGS4/GRIN2B/RIMS2 16

BP GO:0001667 ameboidal-type cell migration 92/2475 497/18870 0.000349529176268161 0.00390069363255398 0.00283329950553511 TIMP1/SP100/MAP2K3/FERMT1/EFNB1/HAND2/MEOX2/ITGB3/LOXL2/EMP2/SNAI1/SEMA3E/CXCL13/TMSB4X/MIR221/CD248/ARHGDIB/STC1/CENPV/IQGAP1/ITGA2/ANXA1/HMOX1/THBS1/ITGA3/CD40/RET/NRP1/ACVR1C/S100A12/SEMA3A/FSTL1/MIR210/SEMA3D/S100A2/HSPB1/CYP1B1/SOX8/MMP9/NR2E1/ITGB4/SRPX2/ANGPT2/ITGA4/MET/PTPRR/S100A9/ACTA2/ATOH8/ANGPT1/VEGFA/FOLR1/GPX1/SYDE1/SEMA6B/HAS2/DUSP10/FN1/DCN/MIR27B/TACSTD2/PDLIM1/SOX10/TWIST1/RHOJ/NR2F2/EPHB4/CRB2/SEMA3F/SDC4/ADAM9/RRAS/EPHA2/EFNB2/SNAI2/FAP/DAAM2/GADD45A/TBX1/ITGB1/DPP4/PRKCE/GDNF/PPARG/PIK3CG/TGFB2/ZC3H12A/AQP1/PTN/DLL4/NODAL/PFN1 92

BP GO:0051896 regulation of phosphatidylinositol 3-kinase/protein kinase B signal transduction 52/2475 248/18870 0.000365180098022166 0.00406779463548254 0.00295467463354328 DDIT3/IL18/SEMA3E/H19/NCF1/MIR221/ROR2/SPRY2/C1QTNF1/THBS1/TREM2/RET/F3/NOX4/EGFR/WNT16/IGFBP5/HCST/ENG/NTS/ROR1/ANGPT1/RCN3/F2RL1/GPX1/CCL5/CHI3L1/IGF2/HGF/FN1/DCN/BTN2A2/CSF3/PDGFA/GRM2/TWIST1/FAM110C/PDGFD/RRAS/EPHA2/OSM/ERFE/GATA4/ITGB1/CAVIN3/NTRK1/PIK3CG/STOX1/TGFB2/RAMP3/HCLS1/GDF15 52

BP GO:0061351 neural precursor cell proliferation 38/2475 166/18870 0.00037078239310154 0.00412255090404009 0.00299444727996039 KCNA1/MDK/EMX1/KIF14/LEF1/FGF13/MELK/ACSL6/FLNA/DISP3/HAPLN1/TEAD3/NR2E1/SPINT1/ATF5/GLI3/TAFA3/TOX/VEGFA/SHCBP1/NEUROD4/LYN/DMRTA2/LHX5/LRRK2/SOX10/GLI1/OTP/HAPLN3/TAFA1/NDE1/ITGB1/WDR62/ASPM/RASSF10/PTN/DLL4/FZD6 38

BP GO:0042102 positive regulation of T cell proliferation 27/2475 105/18870 0.000372585217742818 0.00413493834995911 0.00300344499875181 CD276/EFNB1/PYCARD/IL18/IL1A/ANXA1/CD70/TFRC/HAVCR2/HLA-DPA1/CD274/CD3E/PDCD1LG2/CCL5/IGF2/SASH3/SPN/HLA-DPB1/LILRB2/IGFBP2/ZP3/HLA-A/HLA-DMB/PTPRC/IL6/IL2RA/FADD 27

BP GO:2000116 regulation of cysteine-type endopeptidase activity 43/2475 195/18870 0.000376246646599641 0.0041678687567975 0.00302736425881202 NGFR/PYCARD/HIP1R/IFI6/LCK/FAS/THBS1/ST20/ACVR1C/F3/CD44/CLEC7A/MMP9/BIRC3/S100A8/S100A9/CASP1/CASP8/VEGFA/CARD16/IFI16/GPX1/GRIN2A/DPEP1/HGF/RPS6KA1/MAPT/BCL2L12/MIR27B/CST3/GRIN1/PSMB9/LTF/LAMP3/LAPTM5/SFN/CST7/PPARG/PLAUR/FADD/AQP1/GRIN2B/NODAL 43

BP GO:0010718 positive regulation of epithelial to mesenchymal transition 18/2475 59/18870 0.000382582444390828 0.0042252006139008 0.00306900770423069 MDK/LOXL2/TGFB1I1/EMP2/SNAI1/LEF1/WWTR1/MIR221/BMP2/JAG1/ENG/PDPN/COL1A1/TWIST1/CRB2/IL6/TGFB2/EZH2 18

BP GO:0006820 monoatomic anion transport 39/2475 172/18870 0.000383533366851422 0.0042252006139008 0.00306900770423069 GLRA3/ANO3/SLC4A10/TSPO/STC1/CA2/SLC17A8/SLC22A6/ATP8B1/CLIC3/GABRA3/GABRA5/GABRD/GABRA4/TCAF2/P2RY6/GABRB3/SLC17A7/CLIC4/APOL1/GRM5/TTYH3/BEST4/SLC12A5/SLC17A6/SLC26A2/SLC12A7/GABRA1/GABRB2/GABRG2/PRKG2/GABRG1/SLC25A27/ABCC3/CLIC1/CA7/NMUR2/UCP2/GABRE 39

BP GO:0051048 negative regulation of secretion 39/2475 172/18870 0.000383533366851422 0.0042252006139008 0.00306900770423069 IL1RAPL1/SPI1/TSPO/CHGA/ANXA1/ABCC8/GABBR1/ACVR1C/VAMP8/KCNB1/CD74/CRH/IL13RA2/ASIC1/CNR1/TNFRSF1A/F2RL1/FCGR2B/IL11/SYT4/LIF/KCNJ11/CD300A/RAP1B/P2RY1/LILRB1/HRH3/RSAD2/VSNL1/CRY2/OSM/STXBP6/TRH/ADORA1/RHBDF1/UCP2/ADRA2B/MAOB/HLA-F 39

BP GO:0044839 cell cycle G2/M phase transition 36/2475 155/18870 0.00038949857620585 0.00428305760355297 0.00311103258380728 CENPF/WEE1/KIF14/PLK3/USH1C/MELK/RBBP8/CHEK2/CCNB1/DTL/NABP1/ATF5/BRSK2/FOXM1/ORC1/BRCA1/PKMYT1/CCNB2/CDK2/CCNA2/AURKA/CDC25C/AURKB/CLSPN/PLK1/CDC6/NDC80/CDK4/CDK1/WNT10B/CDC25A/STOX1/DYRK3/PLCB1/CHEK1/CDK6 36

BP GO:0031100 animal organ regeneration 19/2475 64/18870 0.000392140660352927 0.00430422765038204 0.00312640961385967 LCP1/ACE/CLDN1/HSPG2/TYMS/ANGPT2/CEBPB/RGN/CCNA2/AURKA/PCNA/GLI1/NNMT/CDK1/IL6/HFE/ADM/EZH2/UCP2 19

BP GO:0050931 pigment cell differentiation 13/2475 36/18870 0.000401475064775808 0.00438736476317179 0.00318679690974801 SOD2/RAB32/CITED1/ADAMTSL4/RAB27A/ADAMTS20/GLI3/CD63/ENPP1/SOX10/MREG/LRMDA/RAB38 13

BP GO:0002347 response to tumor cell 15/2475 45/18870 0.000401907165180627 0.00438736476317179 0.00318679690974801 MR1/SPI1/NKG7/HAVCR2/AHR/MICA/CD274/HLA-DRB1/PLK5/ABI3/NECTIN2/LAPTM5/HLA-A/PRF1/IL4I1 15

BP GO:0034331 cell junction maintenance 15/2475 45/18870 0.000401907165180627 0.00438736476317179 0.00318679690974801 ITGB3/ADGRB3/CLDN1/SHANK2/RAB3A/SHANK1/CBLN3/OPHN1/CBLN2/ERC2/F2RL1/CAMSAP3/BSN/CBLN1/PCLO 15

BP GO:0003015 heart process 53/2475 255/18870 0.000403074055954574 0.00439211729936708 0.00319024895180017 CAV1/MDM2/SHOX2/MAP2K3/APLN/MYH7/WWTR1/NKX2-5/BVES/STC1/CHGA/ACE/TMIGD3/FGF13/GCH1/TNNI2/HOPX/FGF12/ATP2B3/CTNNA3/NOX4/FLNA/PPCS/DSG2/SLC8A3/GPX1/HSPB7/SCN2B/RYR2/CXCR4/TBX5/SLC8A2/KCNE5/KCNE3/KCNJ3/GJC1/DSC2/KCNJ12/SCN3B/GLP1R/GATA4/DES/ADORA1/ATP2B2/PIK3CG/TGFB2/RGS4/ZC3H12A/RAMP3/KCNE4/GJD3/ADM/KCNIP2 53

BP GO:0002832 negative regulation of response to biotic stimulus 35/2475 150/18870 0.000424905818849313 0.00461326317607826 0.00335087999903437 MMP12/TREM2/CGAS/TRIM21/LILRA4/HAVCR2/AHR/MICA/TRIM38/OAS1/RAB7B/SLAMF8/TNFAIP3/MICB/CARD16/IFI16/F2RL1/NMI/VSIG4/DUSP10/OAS3/NLRC5/SERPING1/LTF/ZDHHC12/ISG15/HLA-B/LILRB1/LILRA2/AURKB/HLA-A/FGL2/PPARG/IL4I1/HLA-F 35

BP GO:0072073 kidney epithelium development 35/2475 150/18870 0.000424905818849313 0.00461326317607826 0.00335087999903437 WNT4/HOXD11/WWTR1/NPNT/IQGAP1/HS3ST3B1/SLC22A6/PROM1/HOXA11/RET/FOXJ1/CITED1/STAT1/BMP2/SOX8/JAG1/FOXD1/GLI3/GREB1L/VEGFA/OSR1/LIF/TACSTD2/SDC4/SDC1/CXCR2/EFNB2/HS3ST3A1/GDNF/SPRY1/EYA1/NOG/AQP1/WNT7B/HOXB7 35

BP GO:0042886 amide transport 69/2475 354/18870 0.000430605269638045 0.00466670404134806 0.00338969719192556 STX1A/SLC16A10/CA2/SLC17A8/CHGA/VGF/IL1RN/ANXA1/ABCC8/GABBR1/ACVR1C/KCNB1/CD74/CRH/STXBP1/CNR1/GLUD1/RAB3A/SLC17A7/PRKCB/S100A8/RAPGEF4/NNAT/BRSK2/HLA-DRB1/SMPD3/SYT7/FOLR1/F2RL1/CCL5/VIP/GPR27/HTR2C/SYT4/SLC1A6/KCNJ11/SNAP25/SLC15A3/NOS2/SLC17A6/GRM2/MTTP/SLC14A2/HRH3/F2RL2/NKX6-1/VSNL1/TAP1/GCK/GRM1/ALOX5/PCLO/TUNAR/ITGB1/TRH/ADORA1/PRKCE/AQP9/IL6/GAL/HFE/TCIRG1/AQP1/UPK3A/CPLX1/GRP/UCP2/PLCB1/RIMS2 69

BP GO:0030316 osteoclast differentiation 27/2475 106/18870 0.000438411825731432 0.00472604338842663 0.00343279879348006 LRRC17/TREM2/TFRC/LILRB3/OSCAR/SBNO2/BMP2/CCN4/SNX10/FAM20C/FSTL3/CEBPB/LTF/TYROBP/TNFRSF11B/LILRB1/TNFAIP6/EPHA2/POU4F1/ERFE/FOSL2/CD109/ANXA2/TCIRG1/CCR1/GPR183/CAMK4 27

BP GO:0002064 epithelial cell development 46/2475 214/18870 0.000438523532917571 0.00472604338842663 0.00343279879348006 PDE2A/HOXA5/IL1A/MSN/STC1/IQGAP1/CLDN1/ICAM1/FOXJ1/NKX3-2/FLNA/ST14/ADAMTSL4/SOX8/JAG1/TYMS/S1PR3/GPR4/MET/TNFRSF1A/ACTA2/CLIC4/VEGFA/F2RL1/GPX1/FRZB/CXCR4/TFCP2L1/FZD5/F11R/RAP1B/SFN/NKX6-1/SDC1/VIM/EPHA2/FOSL2/COL27A1/HOXB13/PALLD/NTRK1/MYD88/PLOD3/PLCB1/WNT7B/CDK6 46

BP GO:0007215 glutamate receptor signaling pathway 16/2475 50/18870 0.000439229215646579 0.00472604338842663 0.00343279879348006 CDK5R1/NECAB2/GRIA4/KCNB1/GRIA2/GRIN3A/GRIN2A/GRM5/FRRS1L/GRIN1/GRM2/GRIK1/GRM1/SSTR1/GRIN2B/PLCB1 16

BP GO:1902041 regulation of extrinsic apoptotic signaling pathway via death domain receptors 16/2475 50/18870 0.000439229215646579 0.00472604338842663 0.00343279879348006 SP100/MIR221/HMOX1/THBS1/ICAM1/LGALS3/FAIM2/TNFAIP3/BRCA1/GPX1/SFRP2/HGF/SERPINE1/TMBIM1/HMGB2/ATF3 16

BP GO:0062197 cellular response to chemical stress 63/2475 317/18870 0.000440204289014005 0.00472806180901626 0.00343426488915361 CAV1/MDM2/DDIT3/PYCARD/H19/NCF1/RELB/TSPO/FANCD2/FAS/ECT2/SOD2/ANXA1/HMOX1/VRK2/PAWR/GCH1/MMP2/TRIM21/EGFR/RIPK3/WNT16/CYP1B1/MMP9/MET/NET1/SCN2A/CASP1/TNFAIP3/SMPD3/GPX1/SELENON/SPHK1/HGF/CHCHD2/DHRS2/GJB2/MAPT/SLC2A4/SOD3/LRRK2/YBX3/GPX7/AQP5/PPIA/CD36/PCNA/BDKRB2/PDGFD/SLC25A24/FBLN5/CASP3/FBP1/ALOX5/CDK1/IL6/CRYGD/STOX1/ZC3H12A/GPX8/AQP1/EZH2/MT3 63

BP GO:0015833 peptide transport 54/2475 262/18870 0.00044267749537922 0.00473767501293554 0.00344124751544443 STX1A/SLC16A10/CA2/CHGA/VGF/IL1RN/ANXA1/ABCC8/GABBR1/ACVR1C/KCNB1/CD74/CRH/CNR1/GLUD1/RAB3A/PRKCB/S100A8/RAPGEF4/NNAT/BRSK2/HLA-DRB1/SMPD3/SYT7/F2RL1/CCL5/VIP/GPR27/HTR2C/KCNJ11/SNAP25/SLC15A3/NOS2/F2RL2/NKX6-1/VSNL1/TAP1/GCK/ALOX5/PCLO/TUNAR/TRH/ADORA1/PRKCE/IL6/GAL/HFE/TCIRG1/AQP1/CPLX1/GRP/UCP2/PLCB1/RIMS2 54

BP GO:0090068 positive regulation of cell cycle process 54/2475 262/18870 0.00044267749537922 0.00473767501293554 0.00344124751544443 STIL/MDM2/MAD2L1/WNT4/KIF14/IL1A/MIR221/CENPV/E2F7/ECT2/CDCA5/ANXA1/RRM2/KIF23/UBE2C/EGFR/E2F8/MAP3K20/CCNB1/FAM83D/DTL/DLGAP5/BIRC5/SMPD3/PLSCR1/ESPL1/SPHK1/IGF2/NPM2/AURKA/GLI1/CDC25C/BUB1/AURKB/NCAPG/SMC4/RAD51AP1/DBF4/CDC6/PLK4/CDCA8/NDC80/CDK4/CDK1/WNT10B/NCAPH/PRKCE/CDC25A/STOX1/DYRK3/EZH2/SPAG5/PLCB1/CDC20 54

BP GO:0002230 positive regulation of defense response to virus by host 12/2475 32/18870 0.000447791245313242 0.00478387657804396 0.00347480638571411 PYCARD/PARP9/CGAS/APOBEC3G/STAT1/TRIM22/APOBEC3F/DTX3L/LILRB1/TRIM6/ZDHHC1/ZC3H12A 12

BP GO:0051591 response to cAMP 23/2475 85/18870 0.000452286265105859 0.0048147637157723 0.00349724150118248 AKAP6/PDE2A/THBD/HCN1/FOSL1/STC1/VGF/CITED1/AHR/IGFBP5/CYP1B1/STAT1/AREG/CNGA3/COL1A1/RAP1B/SDC1/FBP1/WNT10B/AQP9/PIK3CG/AQP1/GPD1 23

BP GO:1904029 regulation of cyclin-dependent protein kinase activity 23/2475 85/18870 0.000452286265105859 0.0048147637157723 0.00349724150118248 HERC5/CDK5R2/CDK5R1/CCNJL/CCNP/PSRC1/CDKN3/EGFR/CCNB1/CDKN2C/PKMYT1/CCNB2/SERTAD1/CCNA2/CDC25C/NR2F2/PLK1/CDC6/GADD45A/CDC25A/STOX1/DIRAS3/LATS2 23

BP GO:1902107 positive regulation of leukocyte differentiation 41/2475 185/18870 0.000455787162062246 0.00483488713961435 0.0035118583083948 IL7R/MDK/IL18/LEF1/NFKBIZ/SPI1/ROR2/ANXA1/ACTL6B/TESPA1/TREM2/CD74/SOCS1/RHOH/RUNX1/GLI3/TOX/HLA-DRB1/MMP14/CASP8/CD101/IL2RG/SASH3/DUSP10/FES/BTN2A2/RUNX3/LIF/LILRB2/HLA-DRA/TYROBP/EVI2B/POU4F1/PTPRC/WNT10B/IL2RA/PIK3R6/FADD/HCLS1/IL4I1/CCR1 41

BP GO:1903708 positive regulation of hemopoiesis 41/2475 185/18870 0.000455787162062246 0.00483488713961435 0.0035118583083948 IL7R/MDK/IL18/LEF1/NFKBIZ/SPI1/ROR2/ANXA1/ACTL6B/TESPA1/TREM2/CD74/SOCS1/RHOH/RUNX1/GLI3/TOX/HLA-DRB1/MMP14/CASP8/CD101/IL2RG/SASH3/DUSP10/FES/BTN2A2/RUNX3/LIF/LILRB2/HLA-DRA/TYROBP/EVI2B/POU4F1/PTPRC/WNT10B/IL2RA/PIK3R6/FADD/HCLS1/IL4I1/CCR1 41

BP GO:0034142 toll-like receptor 4 signaling pathway 17/2475 55/18870 0.000464973972025883 0.00492363973199895 0.00357632445206207 LY96/TREM2/OAS1/RAB7B/TNFAIP3/F2RL1/NMI/SCIMP/LYN/CD14/LTF/LILRA2/IFI35/PRKCE/LBP/IRAK2/MYD88 17

BP GO:1901990 regulation of mitotic cell cycle phase transition 69/2475 355/18870 0.000467358650343428 0.0049401784096161 0.00358833745065386 STIL/CENPF/SPC24/MDM2/MAD2L1/WEE1/KIF14/MIR221/PLK3/BCL7A/E2F7/CDCA5/ANXA1/ACTL6B/KNTC1/RBBP8/RRM2/CHEK2/UBE2C/EGFR/CCL2/MAP3K20/CCNB1/CDKN2C/DTL/NEK6/NABP1/DLGAP5/GPNMB/ORC1/BIRC5/BRCA1/HASPIN/ESPL1/TTK/PLK5/MUC1/NPM2/ZWILCH/BTN2A2/NUF2/CDK2/AURKA/TRIP13/BUB1B/CDC25C/BUB1/AURKB/CLSPN/E2F1/MYO16/PLK1/CDC6/CENPE/CDCA8/NDC80/CDK4/CDK1/WNT10B/CDC25A/STOX1/GTSE1/KNL1/ZWINT/EZH2/PLCB1/CHEK1/CDK6/CDC20 69

BP GO:0002691 regulation of cellular extravasation 14/2475 41/18870 0.00047111964630524 0.00497118164572348 0.00361085689509316 MDK/ICAM1/RIPK3/IL1R1/ITGA4/PTGER4/PLVAP/BST1/PDGFD/CHST2/FADD/PLCB1/GCNT1/FUT9 14

BP GO:0007156 homophilic cell adhesion via plasma membrane adhesion molecules 38/2475 168/18870 0.000475766763940169 0.00501140991350311 0.00364007701385802 PCDHB7/CELSR3/PCDHGA1/PTPRT/EMB/MPZL2/RET/CDH4/PCDHGA3/DSCAM/CDH22/CDH9/PCDHGA5/DSG2/CDH6/DSCAML1/CADM2/CLSTN2/CDH19/PCDHGB4/L1CAM/PCDH11X/PCDH7/CDHR1/PCDH15/NECTIN2/DSC2/IGSF9/CNTN6/CDH8/AMIGO2/CNTN4/PCDHGB5/NEXN/ITGB1/PALLD/CELSR1/CDH18 38

BP GO:0001658 branching involved in ureteric bud morphogenesis 18/2475 60/18870 0.000480359800513429 0.00505092862045995 0.00366878173753735 WNT4/HOXD11/NPNT/HS3ST3B1/HOXA11/CITED1/BMP2/SOX8/FOXD1/GLI3/GREB1L/VEGFA/TACSTD2/HS3ST3A1/GDNF/EYA1/NOG/HOXB7 18

BP GO:2001257 regulation of cation channel activity 30/2475 123/18870 0.00048414949947211 0.00506593383929562 0.00367968089627998 CAV1/AKAP6/TMSB4X/STAC/JPH4/FGF13/TREM2/CABP4/FGF12/NIPSNAP2/P2RY6/MMP9/KCNAB1/KCNS2/PIRT/SELENON/KCNE5/KCNE3/CABP1/LRRC55/HPCA/JPH3/ITGB1/FKBP1C/ANK3/GAL/KCNE4/GRP/CTSS/KCNIP2 30

BP GO:0042832 defense response to protozoan 11/2475 28/18870 0.000484734687686031 0.00506593383929562 0.00367968089627998 BATF/SLC11A1/NKG7/GBP2/CD40/BCL3/GBP1/CLEC7A/GBP4/BATF2/MYD88 11

BP GO:0051953 negative regulation of amine transport 11/2475 28/18870 0.000484734687686031 0.00506593383929562 0.00367968089627998 CHGA/GABBR1/CRH/CNR1/SYT4/P2RY1/HRH3/TRH/ADORA1/RGS4/ADRA2B 11

BP GO:1900046 regulation of hemostasis 20/2475 70/18870 0.000485940982284415 0.00506593383929562 0.00367968089627998 CAV1/THBD/PLAT/C1QTNF1/THBS1/F3/F2RL1/EMILIN2/SERPINE1/PDGFA/VKORC1/SERPING1/PLAU/CD36/EMILIN1/PROS1/FAP/TFPI/PLAUR/ANXA2 20

BP GO:0043903 regulation of biological process involved in symbiotic interaction 19/2475 65/18870 0.00048684940460919 0.00506593383929562 0.00367968089627998 CAV1/CXCL8/FUCA2/CD74/TRIM21/TRIM38/TRIM22/CIITA/IFITM3/HLA-DRB1/F2RL1/IFITM2/LTF/NECTIN2/LGALS1/TRIM6/TRIM5/CXCL6/PTX3 19

BP GO:2000117 negative regulation of cysteine-type endopeptidase activity 19/2475 65/18870 0.00048684940460919 0.00506593383929562 0.00367968089627998 IFI6/THBS1/CD44/MMP9/VEGFA/CARD16/IFI16/GPX1/DPEP1/HGF/RPS6KA1/BCL2L12/CST3/LTF/LAMP3/SFN/CST7/PLAUR/AQP1 19

BP GO:0007411 axon guidance 49/2475 233/18870 0.000494351933733713 0.00512623317812991 0.00372347979539819 CDK5R2/EFNB1/NGFR/LGR6/UNC5A/CDK5R1/SEMA3E/EMB/VASP/HOXA2/RET/NRP1/SEMA3A/EPHA10/CDH4/SEMA3D/SLIT1/LAMA2/DSCAM/GAP43/DLX5/DSCAML1/OPHN1/FOXD1/GLI3/FEZF2/NRXN1/VEGFA/L1CAM/SEMA6B/ECE1/TNR/IGSF9/CNTN6/EFNA4/SEMA3F/NRXN3/DRAXIN/EFNB2/TBR1/CNTN4/EPHB6/NEXN/LRTM2/EPHA3/PALLD/GDNF/NTRK1/NOG 49

BP GO:0097485 neuron projection guidance 49/2475 233/18870 0.000494351933733713 0.00512623317812991 0.00372347979539819 CDK5R2/EFNB1/NGFR/LGR6/UNC5A/CDK5R1/SEMA3E/EMB/VASP/HOXA2/RET/NRP1/SEMA3A/EPHA10/CDH4/SEMA3D/SLIT1/LAMA2/DSCAM/GAP43/DLX5/DSCAML1/OPHN1/FOXD1/GLI3/FEZF2/NRXN1/VEGFA/L1CAM/SEMA6B/ECE1/TNR/IGSF9/CNTN6/EFNA4/SEMA3F/NRXN3/DRAXIN/EFNB2/TBR1/CNTN4/EPHB6/NEXN/LRTM2/EPHA3/PALLD/GDNF/NTRK1/NOG 49

BP GO:0006213 pyrimidine nucleoside metabolic process 10/2475 24/18870 0.000502131207508917 0.00518897722871521 0.00376905443012853 APOBEC3B/TYMP/DPYD/APOBEC3G/TK1/APOBEC3C/UPP1/APOBEC3F/DTYMK/APOBEC3H 10

BP GO:0099500 vesicle fusion to plasma membrane 10/2475 24/18870 0.000502131207508917 0.00518897722871521 0.00376905443012853 STX1A/STX1B/CPLX2/DOC2A/STXBP1/RAB3A/ERC2/SYT1/SNAP25/CPLX1 10

BP GO:0007015 actin filament organization 86/2475 464/18870 0.000513834757465527 0.00530079705124231 0.00385027563787245 WNT4/TMOD2/PSTPIP1/DIAPH3/GPR65/PYCARD/HIP1R/EMP2/TMSB4X/CCL26/USH1C/IQGAP1/ZYX/LCP1/CAPG/TAGLN2/GAS2L3/GDPD2/PSTPIP2/FHDC1/VASP/CNN3/DNAI3/IQGAP2/MYO5C/PAWR/NRP1/FLNA/FHOD1/SH3D21/AVIL/COTL1/RHOH/CORO6/SHROOM3/S100A10/SHANK1/SSH3/ARHGAP18/MET/PTGER4/ARPC5/SCIN/FCHSD1/F2RL1/CDC42EP5/MYO1B/F11R/CSF3/RHOV/TACSTD2/PDLIM1/ANG/ACTN1/PAK3/PLS3/RHOJ/RAC2/SDC4/TPM4/TMEFF2/CAPZA1/GMFG/PACSIN1/ELN/PHACTR1/SPTBN2/RHOC/WIPF3/SPTB/MYO5B/IQGAP3/DAAM2/MYO1F/PLEK/TMSB10/CARMIL2/PRKCE/MICALL2/CALD1/RGS4/RHOD/SWAP70/MYO1G/HCLS1/PFN1 86

BP GO:1902850 microtubule cytoskeleton organization involved in mitosis 37/2475 163/18870 0.000521769194009458 0.00537341722269774 0.00390302387068915 STIL/NEK2/KIFC1/MAD2L1/PLK3/SPRY2/KIF4A/MYBL2/PSRC1/CHEK2/KIF23/CENPA/FLNA/CENPH/TACC3/CCNB1/NEK6/ANKRD53/DLGAP5/BIRC5/ESPL1/TTK/NUF2/AURKA/AURKB/NDE1/PLK1/CENPE/CDCA8/NDC80/ITGB1/CDK1/WDR62/TPX2/SPRY1/CHMP4BP1/CDC20 37

BP GO:0085029 extracellular matrix assembly 15/2475 46/18870 0.000524823688091462 0.00538639559538656 0.00391245081378844 EFEMP2/HAS3/COL3A1/MFAP4/LOX/SMPD3/HAS2/MIR27B/COL1A2/EMILIN1/FBLN5/ELN/FKBP10/PLOD3/THSD4 15

BP GO:1902622 regulation of neutrophil migration 15/2475 46/18870 0.000524823688091462 0.00538639559538656 0.00391245081378844 MDK/IL1A/CXCL8/CD74/IL1R1/SLAMF8/C5AR1/BST1/THBS4/TNFAIP6/RAC2/CXCR2/DPP4/LBP/MYD88 15

BP GO:0001508 action potential 34/2475 146/18870 0.000528921202270023 0.00541918583349696 0.00393626826119042 CAV1/KCNA1/AKAP6/HCN1/FGF13/CHRNB2/FGF12/KCNB1/CHRNA1/GJD2/CTNNA3/FLNA/CNR1/DSG2/SCN2B/KCNAB2/RYR2/SLC8A2/KCNE5/SCN8A/KCNJ11/KCNE3/KCNJ3/GJC1/CD36/DSC2/KCNH7/KCNC2/SCN3B/CHRNA4/ANK3/KCNE4/CACNA1I/KCNIP2 34

BP GO:0008625 extrinsic apoptotic signaling pathway via death domain receptors 23/2475 86/18870 0.000541901771265361 0.00553960737112451 0.00402373739236454 SP100/TRADD/SPI1/MIR221/HMOX1/THBS1/ICAM1/LGALS3/FAIM2/TNFRSF1A/TNFAIP3/TNFRSF10C/CASP8/BRCA1/DEDD2/GPX1/SFRP2/HGF/SERPINE1/TMBIM1/HMGB2/FADD/ATF3 23

BP GO:0002790 peptide secretion 51/2475 246/18870 0.00054511847248421 0.00553960737112451 0.00402373739236454 STX1A/SLC16A10/CHGA/VGF/IL1RN/ANXA1/ABCC8/GABBR1/ACVR1C/KCNB1/CD74/CRH/CNR1/GLUD1/RAB3A/PRKCB/S100A8/RAPGEF4/NNAT/BRSK2/HLA-DRB1/SMPD3/SYT7/F2RL1/CCL5/VIP/GPR27/HTR2C/KCNJ11/SNAP25/NOS2/F2RL2/NKX6-1/VSNL1/GCK/ALOX5/PCLO/TUNAR/TRH/ADORA1/PRKCE/IL6/GAL/HFE/TCIRG1/AQP1/CPLX1/GRP/UCP2/PLCB1/RIMS2 51

BP GO:0046688 response to copper ion 13/2475 37/18870 0.000545287800855194 0.00553960737112451 0.00402373739236454 MT1M/LOXL2/IL1A/MT1A/MT2A/MT1DP/TFRC/MT1E/SOD3/MT1H/CDK1/AQP1/MT3 13

BP GO:0071276 cellular response to cadmium ion 13/2475 37/18870 0.000545287800855194 0.00553960737112451 0.00402373739236454 MT1M/SLC11A1/NCF1/MT1A/MT2A/HMOX1/MT1DP/SLC39A8/EGFR/MT1E/MMP9/MT1H/MT3 13

BP GO:1901381 positive regulation of potassium ion transmembrane transport 13/2475 37/18870 0.000545287800855194 0.00553960737112451 0.00402373739236454 AKAP6/KCNC1/RGS7/TREM2/FLNA/KCNMB1/KCNN4/KCNE5/KCNC2/LRRC55/FXYD2/GAL/KCNIP2 13

BP GO:0033604 negative regulation of catecholamine secretion 7/2475 13/18870 0.000550624760794665 0.00556557418150029 0.00404259858935371 CHGA/GABBR1/CRH/CNR1/SYT4/P2RY1/ADRA2B 7

BP GO:0044771 meiotic cell cycle phase transition 7/2475 13/18870 0.000550624760794665 0.00556557418150029 0.00404259858935371 PKMYT1/TTK/CCNB2/CDC25C/NDC80/CDC25A/CDC20 7

BP GO:2001198 regulation of dendritic cell differentiation 7/2475 13/18870 0.000550624760794665 0.00556557418150029 0.00404259858935371 FCGR2B/CEBPB/LILRB2/HLA-B/LILRB1/TMEM176A/TMEM176B 7

BP GO:0045446 endothelial cell differentiation 30/2475 124/18870 0.000559867469897507 0.00564000719675274 0.00409666359552344 PDE2A/MSN/STC1/CLDN1/ICAM1/NRP1/FSTL1/JAG1/S1PR3/MET/TNFRSF1A/CLIC4/ATOH8/VEGFA/F2RL1/GPX1/PDPN/CXCR4/F11R/ETV2/TMEM100/FZD2/RAP1B/NR2F2/BARX1/COL27A1/FZD1/MYD88/PLOD3/PLCB1 30

BP GO:0046632 alpha-beta T cell differentiation 30/2475 124/18870 0.000559867469897507 0.00564000719675274 0.00409666359552344 ASCL2/BATF/IL18/LEF1/NFKBIZ/RELB/LOXL3/ANXA1/BCL3/SOCS1/TNFSF8/RUNX1/GLI3/PTGER4/TOX/HLA-DRB1/JAK3/IL2RG/SASH3/CTSL/SOCS3/RUNX3/SPN/HLA-DRA/RSAD2/FOSL2/IL6/ZC3H12A/IRF1/GPR183 30

BP GO:0002720 positive regulation of cytokine production involved in immune response 22/2475 81/18870 0.0005634257542616 0.00565686994746931 0.00410891198716037 PYCARD/IL18/CD74/IL1R1/ARID5A/CLEC7A/SPON2/F2RL1/SCIMP/SASH3/FZD5/CD36/LAPTM5/LILRB1/TRIM6/HLA-A/RSAD2/B2M/IL6/TNFRSF14/MYD88/HLA-F 22

BP GO:0030500 regulation of bone mineralization 22/2475 81/18870 0.0005634257542616 0.00565686994746931 0.00410891198716037 WNT4/MGP/CCN1/BMP2/ECM1/NELL1/ENPP1/FAM20C/SRGN/OSR1/CYP27B1/TENT5A/LTF/ISG15/TWIST1/ADGRV1/SGMS2/ALOX5/WNT10B/OSR2/PTN/CCR1 22

BP GO:0007187 G protein-coupled receptor signaling pathway, coupled to cyclic nucleotide second messenger 17/2475 56/18870 0.000587066753609074 0.00588438862882952 0.00427417197118424 HTR1A/SSTR3/SSTR2/TSHR/HTR1D/ANXA1/CNR1/CCL2/HTR5A/CHRM1/HTR2A/HRH1/HTR2C/HRH3/SSTR1/CHRM4/CCR1 17

BP GO:2000377 regulation of reactive oxygen species metabolic process 34/2475 147/18870 0.000602883676459763 0.00603285598910736 0.00438201240626807 H19/TSPO/ITGB2/CYBA/SOD2/THBS1/GCH1/RIPK3/CYP1B1/CLEC7A/RAB27A/CBR1/FOXM1/BRCA1/F2RL1/BST1/ACP5/HP/STK17A/FPR2/HK2/PID1/MAPT/MIR27B/GRIN1/LRRK2/CD36/TYROBP/RAC2/FBLN5/ALOX5/GADD45A/ZC3H12A/MT3 34

BP GO:0070588 calcium ion transmembrane transport 68/2475 352/18870 0.000608448172082095 0.00607840736302978 0.00441509900505494 AKAP5/CAV1/VMP1/AKAP6/CHRNA9/DDIT3/ITGB3/STAC/SLN/JPH4/CACNG2/LCK/CYBA/CACNA1B/CABP4/ATP2B3/NIPSNAP2/TMEM37/FLNA/MCUB/APLNR/ASIC1/P2RY6/SCN2A/GRIN3A/CXCL10/SLC8A3/SELENON/GRIN2A/HTR2A/NALCN/TRPM8/RYR2/CACNA2D2/LYN/HTR2C/SLC8A2/SCN8A/GRIN1/CACNA1E/TMEM165/CXCL9/ADCYAP1R1/F2RL3/CCR5/CABP1/RYR3/P2RX6/HPCA/MCOLN2/JPH3/PTPRC/CACNA2D3/SCN3A/ANXA2P2/ATP2B2/PRKCE/TRPV2/CXCL11/PIK3CG/FKBP1C/RAMP3/ANXA2/CACNA1I/GRIN2B/PLCB1/CACNG5/CACNG3 68

BP GO:0099174 regulation of presynapse organization 12/2475 33/18870 0.000620389486554249 0.00617714507010234 0.00448681791532557 IL1RAPL1/NTNG2/SLITRK4/LRRTM3/CBLN2/DKK1/SLITRK1/CBLN1/IL1RAP/LRFN5/FZD1/SLITRK5 12

BP GO:1905606 regulation of presynapse assembly 12/2475 33/18870 0.000620389486554249 0.00617714507010234 0.00448681791532557 IL1RAPL1/NTNG2/SLITRK4/LRRTM3/CBLN2/DKK1/SLITRK1/CBLN1/IL1RAP/LRFN5/FZD1/SLITRK5 12

BP GO:0032677 regulation of interleukin-8 production 26/2475 103/18870 0.000642962612779823 0.00639130385286433 0.00464237383190451 DDIT3/PYCARD/TLR1/TMSB4X/ANXA1/BCL3/F3/CD74/CD33/SSC5D/CLEC7A/TLR2/F2RL1/CD58/CHI3L1/TLR8/SERPINE1/CD14/NOS2/LILRA2/CD2/PTPRC/IL6/LBP/FADD/MYD88 26

BP GO:0071277 cellular response to calcium ion 23/2475 87/18870 0.000646603277872827 0.00641686955429496 0.00466094368025598 IQGAP1/ECT2/KCNB1/NEUROD2/ITPKC/CPNE9/CLIC4/SYT7/KCNH1/DPEP1/SYT1/SYT4/ALOX5AP/RASAL1/ADGRV1/SLC25A24/SYT5/RYR3/HPCA/CPNE8/SYT2/TUBA1A/SYT13 23

BP GO:0060536 cartilage morphogenesis 6/2475 10/18870 0.000662067467001472 0.00652718074199809 0.00474106907922755 HAND2/SNAI1/HOXA5/STC1/SNAI2/WNT7B 6

BP GO:0062042 regulation of cardiac epithelial to mesenchymal transition 6/2475 10/18870 0.000662067467001472 0.00652718074199809 0.00474106907922755 EMP2/JAG1/ENG/TWIST1/TGFB2/NOG 6

BP GO:1903596 regulation of gap junction assembly 6/2475 10/18870 0.000662067467001472 0.00652718074199809 0.00474106907922755 CAV1/ACE/HOPX/APLNR/TBX5/CNTNAP2 6

BP GO:1990504 dense core granule exocytosis 6/2475 10/18870 0.000662067467001472 0.00652718074199809 0.00474106907922755 UNC13A/STXBP1/RAB3A/UNC13C/SYT4/SNAP25 6

BP GO:0045619 regulation of lymphocyte differentiation 45/2475 212/18870 0.000666988143085338 0.00656491280505635 0.00476847606005618 ASCL2/IL7R/MDK/BATF/IL18/LEF1/NFKBIZ/SPI1/LOXL3/FANCD2/ANXA1/ACTL6B/TESPA1/FOXJ1/CD74/SOCS1/RHOH/RUNX1/HLA-DOA/NFAM1/GLI3/SLAMF8/TOX/HLA-DRB1/MMP14/JAK3/IL2RG/SASH3/DUSP10/BTN2A2/RUNX3/LILRB2/HLA-DRA/TCF7/FGL2/CD2/PTPRC/WNT10B/IL2RA/ZC3H12A/PIK3R6/IRF1/IL4I1/CAMK4/FANCA 45

BP GO:0000079 regulation of cyclin-dependent protein serine/threonine kinase activity 22/2475 82/18870 0.000676064048307854 0.00664335277584346 0.00482545154989997 HERC5/CDK5R2/CDK5R1/CCNJL/CCNP/PSRC1/CDKN3/EGFR/CCNB1/CDKN2C/PKMYT1/CCNB2/SERTAD1/CCNA2/CDC25C/NR2F2/PLK1/CDC6/GADD45A/CDC25A/DIRAS3/LATS2 22

BP GO:0043300 regulation of leukocyte degranulation 15/2475 47/18870 0.00067842526573502 0.00664480472344708 0.00482650618346387 KLRC2/SPI1/ITGB2/VAMP8/STXBP1/IL13RA2/ADGRE2/PRAM1/F2RL1/FCGR2B/FES/LYN/CD300A/RAC2/HLA-F 15

BP GO:0046636 negative regulation of alpha-beta T cell activation 15/2475 47/18870 0.00067842526573502 0.00664480472344708 0.00482650618346387 ASCL2/LOXL3/ANXA1/SOCS1/TWSG1/RUNX1/CD274/GLI3/JAK3/RUNX3/CD300A/LILRB1/ZC3H12A/TNFRSF14/HFE 15

BP GO:0001773 myeloid dendritic cell activation 11/2475 29/18870 0.000689103355320013 0.00669478405395042 0.00486280906334664 BATF/PYCARD/SPI1/RELB/HAVCR2/BATF2/BATF3/DHRS2/LTBR/CD2/CAMK4 11

BP GO:0002418 immune response to tumor cell 11/2475 29/18870 0.000689103355320013 0.00669478405395042 0.00486280906334664 MR1/NKG7/HAVCR2/AHR/MICA/CD274/HLA-DRB1/NECTIN2/HLA-A/PRF1/IL4I1 11

BP GO:0006910 phagocytosis, recognition 11/2475 29/18870 0.000689103355320013 0.00669478405395042 0.00486280906334664 TREM2/FCGR1A/CLEC7A/PLA2G5/SPON2/TUB/CD36/COLEC11/LBP/PTX3/COLEC12 11

BP GO:0097421 liver regeneration 11/2475 29/18870 0.000689103355320013 0.00669478405395042 0.00486280906334664 CLDN1/TYMS/CEBPB/RGN/AURKA/PCNA/GLI1/IL6/HFE/EZH2/UCP2 11

BP GO:1905820 positive regulation of chromosome separation 11/2475 29/18870 0.000689103355320013 0.00669478405395042 0.00486280906334664 UBE2C/DLGAP5/BIRC5/PLSCR1/ESPL1/AURKB/NCAPG/SMC4/CDCA8/NCAPH/CDC20 11

BP GO:2001233 regulation of apoptotic signaling pathway 75/2475 398/18870 0.000698487932509803 0.00673261653985186 0.00489028898709742 CAV1/SP100/MDM2/WNT4/DDIT3/NGFR/PYCARD/G0S2/DDIAS/SNAI1/IL1A/MIR221/IFI6/CTSK/LMNA/LCK/FAS/SOD2/HMOX1/THBS1/TREM2/PAK5/ICAM1/RET/NRP1/TNFRSF12A/CTSC/CD74/MIR210/CD44/RIPK3/WNT16/LGALS3/HSPB1/MMP9/FAIM2/S100A8/S100A9/TNFAIP3/BRCA1/DEDD2/GPX1/FCGR2B/SFRP2/MUC1/HGF/CTSL/BCL2L12/MIR27B/EYA2/LRRK2/YBX3/SERPINE1/IL20RA/PPIA/TMBIM1/BDKRB2/LTBR/ACAA2/SNAI2/GATA4/PTPRC/HMGB2/MAPK8IP2/FZD1/GDNF/PLAUR/EYA4/FADD/EYA1/NOG/COL2A1/ATF3/CTSS/TRAF1 75

BP GO:0051382 kinetochore assembly 8/2475 17/18870 0.000698735810469716 0.00673261653985186 0.00489028898709742 CENPF/CENPW/KNTC1/CENPA/CENPH/DLGAP5/CENPE/CENPK 8

BP GO:1900120 regulation of receptor binding 8/2475 17/18870 0.000698735810469716 0.00673261653985186 0.00489028898709742 NRP1/MMP9/MIR27B/GREM2/B2M/HFE/ANXA2/NOG 8

BP GO:0071346 cellular response to type II interferon 29/2475 120/18870 0.000699245954224884 0.00673261653985186 0.00489028898709742 SP100/GBP5/CCL26/PARP9/ZYX/CCL20/GBP2/IFNGR2/CLDN1/HPX/STXBP1/HLA-DPA1/GBP1/CCL2/STAT1/GBP4/RAB7B/CASP1/TLR2/CD58/CCL5/GBP3/NLRC5/CCL18/CAMK2A/NOS2/VIM/PPARG/IRF1 29

BP GO:0072009 nephron epithelium development 29/2475 120/18870 0.000699245954224884 0.00673261653985186 0.00489028898709742 WNT4/HOXD11/WWTR1/NPNT/IQGAP1/HS3ST3B1/SLC22A6/PROM1/HOXA11/FOXJ1/CITED1/STAT1/BMP2/SOX8/JAG1/FOXD1/GLI3/GREB1L/VEGFA/OSR1/LIF/TACSTD2/HS3ST3A1/GDNF/EYA1/NOG/AQP1/WNT7B/HOXB7 29

BP GO:0032970 regulation of actin filament-based process 72/2475 379/18870 0.000700065450959833 0.00673261653985186 0.00489028898709742 CAV1/WNT4/TMOD2/ABRACL/ITGB3/GPR65/PYCARD/HIP1R/CDK5R1/TMSB4X/IL1A/CCL26/STC1/CAPG/ECT2/FGF13/VASP/DNAI3/IQGAP2/NRP1/CTNNA3/FLNA/FHOD1/AVIL/COTL1/S100A10/SHANK1/SSH3/DSG2/ARHGAP18/MET/PTGER4/ACTA2/ARPC5/SCIN/FCHSD1/F2RL1/PDPN/RYR2/CDC42EP5/BST1/F11R/CSF3/PDGFA/TACSTD2/PAK3/DSC2/SDC4/TMEFF2/CAPZA1/GMFG/ARHGEF19/ELN/PEAK3/SPTBN2/RHOC/SPTB/DAAM2/GATA4/PLEK/ADORA1/TMSB10/CARMIL2/EPHA3/PRKCE/ARHGAP44/CELSR1/RGS4/SWAP70/HCLS1/PFN1/BST2 72

BP GO:0050853 B cell receptor signaling pathway 21/2475 77/18870 0.000701968346760037 0.00673261653985186 0.00489028898709742 NFKBIZ/LCK/IGHG4/VAV3/IGHA1/CD79A/PRKCB/NFAM1/IGHG2/CMTM3/IGHG3/FCGR2B/IGKC/IGHA2/LYN/CD300A/IGHG1/LAT2/IGHM/PTPRC/IGLC3 21

BP GO:1903036 positive regulation of response to wounding 21/2475 77/18870 0.000701968346760037 0.00673261653985186 0.00489028898709742 THBD/FERMT1/MDK/MIR221/PLAT/ANXA1/THBS1/CLDN1/F3/FLNA/CLEC7A/CCN4/CXCR4/EMILIN2/SERPINE1/PLAU/CD36/EMILIN1/ITGB1/PRKCE/PTN 21

BP GO:0003197 endocardial cushion development 16/2475 52/18870 0.000715403971484692 0.00683962650444919 0.00496801651669641 MDM2/SNAI1/APLNR/BMP2/JAG1/ENG/MDM4/DKK1/TBX5/TMEM100/TWIST1/SNAI2/RBM24/GATA4/TGFB2/NOG 16

BP GO:0099172 presynapse organization 16/2475 52/18870 0.000715403971484692 0.00683962650444919 0.00496801651669641 IL1RAPL1/NTNG2/SLITRK4/LRRTM3/CBLN2/C5AR1/BSN/DKK1/SLITRK1/PPFIA2/CBLN1/IL1RAP/LRFN5/PCLO/FZD1/SLITRK5 16

BP GO:0010466 negative regulation of peptidase activity 32/2475 137/18870 0.000718612424218719 0.00685600242873633 0.00497991129812662 TIMP1/IFI6/SERPINB1/THBS1/SERPINH1/CD44/MMP9/ECM1/SERPINA5/VEGFA/CARD16/IFI16/GPX1/SERPINB8/DPEP1/HGF/RPS6KA1/BCL2L12/CST3/SERPINE1/SERPING1/LTF/LAMP3/CRB2/SFN/CST7/SERPINA1/CSTA/PLAUR/SERPINA3/SERPINI1/AQP1 32

BP GO:0043954 cellular component maintenance 20/2475 72/18870 0.000722826371983694 0.00685600242873633 0.00497991129812662 ITGB3/ADGRB3/ITGA3/TREM2/CLDN1/SHANK2/RAB3A/SHANK1/CBLN3/OPHN1/CBLN2/ERC2/F2RL1/CAMSAP3/FCGR2B/BSN/CBLN1/ZNF804A/PCLO/GRIN2B 20

BP GO:0061035 regulation of cartilage development 20/2475 72/18870 0.000722826371983694 0.00685600242873633 0.00497991129812662 SHOX2/MDK/LOXL2/CTSK/EFEMP1/HOXA11/NKX3-2/CCN1/BMP2/CCN4/GLI3/SCIN/SMPD3/FRZB/RUNX2/PRKG2/SNAI2/PTHLH/ADAMTS7/NOG 20

BP GO:0070527 platelet aggregation 20/2475 72/18870 0.000722826371983694 0.00685600242873633 0.00497991129812662 MMRN1/ITGB3/C1QTNF1/STXBP1/FLNA/HSPB1/PDPN/LYN/EMILIN2/F11R/MYL9/PPIA/EMILIN1/F2RL3/MYL12A/PLEK/IL6/PIK3CG/CLIC1/FERMT3 20

BP GO:0071260 cellular response to mechanical stimulus 20/2475 72/18870 0.000722826371983694 0.00685600242873633 0.00497991129812662 PDE2A/ITGB3/ITGA2/FAS/CD40/ENG/TNFRSF1A/PTGER4/CASP1/CASP8/TLR8/COL1A1/F11R/LTBR/GADD45A/FADD/IRF1/MYD88/AQP1/CHEK1 20

BP GO:0002475 antigen processing and presentation via MHC class Ib 10/2475 25/18870 0.000739152730729999 0.00698206178918354 0.00507147550625678 HLA-C/MICA/MICB/HLA-B/HLA-A/ULBP2/ULBP3/B2M/HFE/HLA-F 10

BP GO:0072170 metanephric tubule development 10/2475 25/18870 0.000739152730729999 0.00698206178918354 0.00507147550625678 WNT4/WWTR1/SLC22A6/STAT1/SOX8/OSR1/LIF/CXCR2/AQP1/WNT7B 10

BP GO:0032731 positive regulation of interleukin-1 beta production 18/2475 62/18870 0.000741931282728031 0.00698206178918354 0.00507147550625678 GBP5/PYCARD/GSDMD/PYDC1/HSPB1/LILRA5/CLEC7A/CASP1/CASP8/IFI16/F2RL1/TLR8/FZD5/CD36/TYROBP/LILRA2/IL6/MYD88 18

BP GO:0043030 regulation of macrophage activation 18/2475 62/18870 0.000741931282728031 0.00698206178918354 0.00507147550625678 HAMP/THBS1/TREM2/CTSC/CD74/HAVCR2/TAFA3/FCGR2B/SPHK1/VSIG4/LRRK2/CST7/LRFN5/PTPRC/IL6/LBP/PLA2G4A/ZC3H12A 18

BP GO:0090303 positive regulation of wound healing 18/2475 62/18870 0.000741931282728031 0.00698206178918354 0.00507147550625678 THBD/FERMT1/MIR221/PLAT/ANXA1/THBS1/CLDN1/F3/CLEC7A/CCN4/CXCR4/EMILIN2/SERPINE1/PLAU/CD36/EMILIN1/ITGB1/PRKCE 18

BP GO:0014074 response to purine-containing compound 33/2475 143/18870 0.000750225957166939 0.00703341568958642 0.00510877681579939 AKAP6/PDE2A/THBD/HCN1/FOSL1/STC1/VGF/CITED1/AHR/IGFBP5/CYP1B1/P2RY6/STAT1/AREG/CNGA3/SELENON/ENPP1/RYR2/COL1A1/KCNJ11/RAP1B/P2RY1/SDC1/FBP1/RYR3/P2RX6/ADORA1/WNT10B/AQP9/PIK3CG/AQP1/GPD1/CHEK1 33

BP GO:0051988 regulation of attachment of spindle microtubules to kinetochore 9/2475 21/18870 0.000751383600719133 0.00703341568958642 0.00510877681579939 NEK2/ECT2/KNTC1/CCNB1/BIRC5/ZWILCH/AURKB/CDCA8/SPAG5 9

BP GO:0097501 stress response to metal ion 9/2475 21/18870 0.000751383600719133 0.00703341568958642 0.00510877681579939 MT1M/SLC11A1/MT1A/MT2A/MT1DP/SLC39A8/MT1E/MT1H/MT3 9

BP GO:0032637 interleukin-8 production 26/2475 104/18870 0.000752074096055044 0.00703341568958642 0.00510877681579939 DDIT3/PYCARD/TLR1/TMSB4X/ANXA1/BCL3/F3/CD74/CD33/SSC5D/CLEC7A/TLR2/F2RL1/CD58/CHI3L1/TLR8/SERPINE1/CD14/NOS2/LILRA2/CD2/PTPRC/IL6/LBP/FADD/MYD88 26

BP GO:0001837 epithelial to mesenchymal transition 39/2475 178/18870 0.000781736431886858 0.00729944873569004 0.00530201201163502 CUL7/WNT4/MDK/LOXL2/TGFB1I1/EMP2/SNAI1/LEF1/WWTR1/MIR221/LOXL3/SPRY2/FLNA/WNT16/BMP2/FAM83D/JAG1/ENG/SP6/GSC/PDPN/SFRP2/TBX5/HAS2/HGF/COL1A1/TMEM100/TWIST1/CRB2/SPRED3/S100A4/VASN/SNAI2/EPHA3/IL6/TGFB2/SPRY1/NOG/EZH2 39

BP GO:0051961 negative regulation of nervous system development 35/2475 155/18870 0.000803914622707779 0.00748664322483829 0.005437982201428 ASCL2/MCF2/SEMA3E/TSPO/SPP1/FGF13/ABCC8/TREM2/DLL3/NRP1/SEMA3A/SEMA3D/SLIT1/DLX2/RTN4R/NR2E1/TLR2/BRINP1/DKK1/SEMA6B/DUSP10/SYT4/SOX10/TNR/CBLN1/SEMA3F/NKX6-1/DRAXIN/FSTL4/DAAM2/B2M/IL6/NOG/PTN/MT3 35

BP GO:2001237 negative regulation of extrinsic apoptotic signaling pathway 25/2475 99/18870 0.000804277961362541 0.00748664322483829 0.005437982201428 IL1A/MIR221/IFI6/LMNA/HMOX1/THBS1/PAK5/ICAM1/NRP1/LGALS3/FAIM2/TNFAIP3/BRCA1/GPX1/SFRP2/HGF/EYA2/SERPINE1/TMBIM1/SNAI2/HMGB2/GDNF/EYA4/EYA1/COL2A1 25

BP GO:0071887 leukocyte apoptotic process 29/2475 121/18870 0.000806412051563912 0.00749265575432796 0.0054423494495215 IL7R/IRF7/NFKBIZ/FAS/ANXA1/BCL3/CHEK2/CD74/RIPK3/LGALS3/PTCRA/CASP7/CD274/GLI3/JAK3/IDO1/CCL5/CTSL/LYN/CCR5/KDELR1/LILRB1/AURKB/CASP3/CXCR2/IL6/IL2RA/FADD/HCLS1 29

BP GO:0060560 developmental growth involved in morphogenesis 50/2475 244/18870 0.000807419765664588 0.00749265575432796 0.0054423494495215 UNC13A/EMX1/SEMA3E/IQGAP1/SPP1/SPRY2/FGF13/NRP1/RIMS1/SEMA3A/CDH4/SEMA3D/SLIT1/DSCAM/RTN4R/AREG/SH3GL2/ITGA4/CPNE9/VEGFA/SFRP2/L1CAM/CXCR4/SEMA6B/FN1/S100B/SYT1/MAPT/SYT4/RASAL1/FLRT1/AURKA/TNC/TNR/NKD1/SEMA3F/NKX6-1/DRAXIN/FSTL4/POSTN/ITGB1/RDH10/TRPV2/SYT2/SPRY1/ISLR2/WNT7B/HOXD13/MT3/RIMS2 50

BP GO:0099173 postsynapse organization 42/2475 196/18870 0.000810136858908292 0.0074947021585291 0.00544383587131142 IL1RAPL1/ITGB3/HIP1R/CDK5R1/DNM3/ROR2/ITGA3/TREM2/NEFL/NTNG2/NRP1/SHANK2/CUX2/C1QL3/GAP43/SHANK1/SHISA7/OPHN1/NRXN1/NEFH/FCGR2B/ABI3/SRGN/FRRS1L/LRRK2/ZDHHC12/LILRB2/ACTN1/ARC/PAK3/PPFIA2/CBLN1/ZNF804A/SEMA3F/IL1RAP/LZTS1/NPTX1/SPTBN2/GDNF/ARHGAP44/INA/GRIN2B 42

BP GO:1901991 negative regulation of mitotic cell cycle phase transition 42/2475 196/18870 0.000810136858908292 0.0074947021585291 0.00544383587131142 CENPF/SPC24/MDM2/MAD2L1/WEE1/PLK3/E2F7/KNTC1/RBBP8/CHEK2/CCL2/CCNB1/DTL/NABP1/GPNMB/ORC1/BIRC5/BRCA1/HASPIN/TTK/MUC1/ZWILCH/BTN2A2/NUF2/CDK2/TRIP13/BUB1B/BUB1/AURKB/CLSPN/MYO16/PLK1/CDC6/CDCA8/NDC80/CDK1/GTSE1/KNL1/ZWINT/EZH2/CHEK1/CDC20 42

BP GO:1902476 chloride transmembrane transport 27/2475 110/18870 0.000815054611814857 0.00752859675282523 0.00546845547651652 GLRA3/ANO3/SLC17A8/CLIC3/GABRA3/GABRA5/GABRD/GABRA4/GABRB3/SLC17A7/CLIC4/APOL1/TTYH3/BEST4/SLC12A5/SLC17A6/SLC26A2/SLC12A7/GABRA1/GABRB2/GABRG2/GABRG1/SLC25A27/CLIC1/NMUR2/UCP2/GABRE 27

BP GO:0090183 regulation of kidney development 12/2475 34/18870 0.000845435162782971 0.00779722383617351 0.00566357487171129 WNT4/RET/STAT1/SOX8/FOXD1/VEGFA/OSR1/TACSTD2/GDNF/NOG/EZH2/HOXB7 12

BP GO:0051592 response to calcium ion 33/2475 144/18870 0.000852971452056097 0.00784314385210379 0.00569692923652057 CAV1/CACNG2/IQGAP1/ECT2/THBS1/KCNB1/KCNMB1/NEUROD2/ITPKC/CPNE9/CLIC4/SYT7/RYR2/KCNH1/DPEP1/SYT1/S100A16/SYT4/ALOX5AP/RASAL1/MTTP/ADGRV1/SLC25A24/SDC1/ADAM9/SYT5/RYR3/HPCA/CPNE8/SYT2/TUBA1A/SYT13/KCNIP2 33

BP GO:0046683 response to organophosphorus 30/2475 127/18870 0.000853026804700829 0.00784314385210379 0.00569692923652057 AKAP6/PDE2A/THBD/HCN1/FOSL1/STC1/VGF/CITED1/AHR/IGFBP5/CYP1B1/P2RY6/STAT1/AREG/TYMS/CNGA3/ENPP1/COL1A1/KCNJ11/RAP1B/P2RY1/SDC1/FBP1/RYR3/P2RX6/WNT10B/AQP9/PIK3CG/AQP1/GPD1 30

BP GO:0001656 metanephros development 24/2475 94/18870 0.000857104833065678 0.00785657621026921 0.00570668593554215 WNT4/WWTR1/SLC22A6/HOXC11/HOXA11/RET/FOXJ1/CITED1/STAT1/SOX8/FOXD1/GLI3/GREB1L/OSR1/PDGFA/LIF/CXCR2/RDH10/GDNF/SPRY1/OSR2/EYA1/AQP1/WNT7B 24

BP GO:2001251 negative regulation of chromosome organization 24/2475 94/18870 0.000857104833065678 0.00785657621026921 0.00570668593554215 CENPF/SPC24/MAD2L1/TOP2A/KNTC1/PIF1/CCNB1/BIRC5/HASPIN/ESPL1/TTK/ZWILCH/NUF2/TRIP13/BUB1B/H3-3A/BUB1/AURKB/PLK1/CDCA8/NDC80/KNL1/ZWINT/CDC20 24

BP GO:0031295 T cell costimulation 15/2475 48/18870 0.000868600378554976 0.0079377118308129 0.00576561943179246 CAV1/EFNB1/LCK/MAP3K8/KLRC4-KLRK1/CD274/CD3E/PDCD1LG2/LYN/SPN/LILRB2/LGALS1/EFNB2/DPP4/TNFRSF14 15

BP GO:0042088 T-helper 1 type immune response 15/2475 48/18870 0.000868600378554976 0.0079377118308129 0.00576561943179246 ASCL2/IL18/SLC11A1/LEF1/NFKBIZ/RELB/ANXA1/BCL3/HAVCR2/IL1R1/ARID5A/HLA-DRB1/JAK3/SPN/PLA2G4A 15

BP GO:0035567 non-canonical Wnt signaling pathway 19/2475 68/18870 0.000898668167657483 0.00818756248651825 0.00594710042106054 TMEM67/RSPO3/CELSR3/FZD7/SFRP4/PLEKHA4/FRZB/DKK1/SFRP2/FZD5/CTHRC1/FZD2/NKD1/ARHGEF19/DAAM2/SPEF1/FZD1/CELSR1/FZD6 19

BP GO:0072171 mesonephric tubule morphogenesis 19/2475 68/18870 0.000898668167657483 0.00818756248651825 0.00594710042106054 WNT4/HOXD11/NPNT/HS3ST3B1/HOXA11/CITED1/BMP2/SOX8/FOXD1/GLI3/GREB1L/VEGFA/OSR1/TACSTD2/HS3ST3A1/GDNF/EYA1/NOG/HOXB7 19

BP GO:1902692 regulation of neuroblast proliferation 16/2475 53/18870 0.000901619280152442 0.0082020032697504 0.00595758959756231 HAPLN1/NR2E1/GLI3/TAFA3/VEGFA/DMRTA2/LHX5/LRRK2/SOX10/OTP/HAPLN3/TAFA1/ITGB1/WDR62/ASPM/PTN 16

BP GO:0021545 cranial nerve development 18/2475 63/18870 0.000913166439035189 0.00829448003020768 0.00602476082000603 KCNA1/HOXD3/KCNC1/HOXB2/HOXB3/HOXA3/CHRNB2/ATP8B1/NRP1/GLI3/SCN8A/SEMA3F/KCNC2/POU4F1/TBX1/NTRK1/HOXA1/TCIRG1 18

BP GO:0031663 lipopolysaccharide-mediated signaling pathway 17/2475 58/18870 0.000915261441597689 0.00830095120143886 0.00602946120613647 LY96/SPI1/CD180/TNFAIP3/TLR2/CARD16/SCIMP/LYN/CD14/LTF/CD36/LILRA2/TRIM5/PRKCE/LBP/IRAK2/MYD88 17

BP GO:0022612 gland morphogenesis 29/2475 122/18870 0.000927652980872315 0.0084006463003882 0.0061018755255283 CAV1/WNT4/MDK/MSN/NRP1/MMP2/NRG3/EGFR/TWSG1/IGFBP5/CSMD1/AREG/GLI3/TNFAIP3/HGF/RPS6KA1/PDGFA/CEBPB/TNC/GLI1/EPHA2/SNAI2/RXFP1/HOXB13/IL6/TGFB2/NOG/PTN/HOXD13 29

BP GO:0033209 tumor necrosis factor-mediated signaling pathway 27/2475 111/18870 0.000944770881593518 0.00854277767031247 0.00620511376417556 TRADD/PYCARD/TMSB4X/TNFRSF4/TRAF5/FAS/PYDC1/TNFRSF19/CD70/STAT1/BIRC3/TNFRSF1A/CASP1/TNFAIP3/CASP8/CARD16/F2RL1/PLVAP/KRT18/SPHK1/H2BC11/TNFRSF18/MIR27B/LAPTM5/CASP4/TNFRSF14/TRAF1 27

BP GO:0002507 tolerance induction 11/2475 30/18870 0.000960240557293902 0.00863066512873142 0.00626895151098115 FOXJ1/HAVCR2/CD274/TNFAIP3/CD3E/IDO1/LYN/LILRB2/HLA-B/XKR8/IL2RA 11

BP GO:0021602 cranial nerve morphogenesis 11/2475 30/18870 0.000960240557293902 0.00863066512873142 0.00626895151098115 HOXD3/HOXB2/HOXB3/HOXA3/CHRNB2/ATP8B1/NRP1/GLI3/SEMA3F/TBX1/HOXA1 11

BP GO:0071295 cellular response to vitamin 11/2475 30/18870 0.000960240557293902 0.00863066512873142 0.00626895151098115 MDM2/PHEX/FOLR1/COL1A1/FES/CYP27B1/TNC/BRIP1/SNAI2/POSTN/MN1 11

BP GO:0097205 renal filtration 11/2475 30/18870 0.000960240557293902 0.00863066512873142 0.00626895151098115 EMP2/IGKV3-20/ITGA3/IGHA1/MCAM/F2RL1/TMEM63C/IGHA2/JCHAIN/ADORA1/AQP1 11

BP GO:0050921 positive regulation of chemotaxis 33/2475 145/18870 0.000967845872258523 0.00868601885955182 0.00630915812881293 RARRES2/MDK/CXCL13/TMSB4X/SPI1/CCL26/CXCL8/ITGA2/THBS1/TREM2/NRP1/F3/CD74/HSPB1/DSCAM/MET/VEGFA/CXCL10/C5AR1/F2RL1/CCL5/CXCR4/FPR2/SERPINE1/THBS4/RAC2/PDGFD/CXCR2/IL6/LBP/SWAP70/PTN/CCR1 33

BP GO:0002524 hypersensitivity 7/2475 14/18870 0.000976779593481267 0.00871500606344037 0.00633021321239059 FCGR1A/FCGR2B/SPN/IGHG1/ZP3/C3/FCGR3A 7

BP GO:0035641 locomotory exploration behavior 7/2475 14/18870 0.000976779593481267 0.00871500606344037 0.00633021321239059 SLC4A10/CRH/LRRK2/TNR/GAD1/DPP4/PRKCE 7

BP GO:0010951 negative regulation of endopeptidase activity 30/2475 128/18870 0.000976881925498896 0.00871500606344037 0.00633021321239059 TIMP1/IFI6/SERPINB1/THBS1/SERPINH1/CD44/MMP9/SERPINA5/VEGFA/CARD16/IFI16/GPX1/SERPINB8/DPEP1/HGF/RPS6KA1/BCL2L12/CST3/SERPINE1/SERPING1/LTF/LAMP3/CRB2/SFN/CST7/SERPINA1/PLAUR/SERPINA3/SERPINI1/AQP1 30

BP GO:0031623 receptor internalization 30/2475 128/18870 0.000976881925498896 0.00871500606344037 0.00633021321239059 CAV1/APLN/ITGB3/NECAB2/ITGB2/DNM3/CACNG2/CXCL8/TFRC/SFRP4/OPHN1/CD63/ANGPT1/VEGFA/DKK1/SNAP25/ARC/CD36/LILRB1/CXCR2/EFNB2/FCER1G/HPCA/ITGB1/CALY/RAMP3/ANXA2/ADM/CACNG5/CACNG3 30

BP GO:0072088 nephron epithelium morphogenesis 21/2475 79/18870 0.00100936798960734 0.00899146203205118 0.00653101917998317 WNT4/HOXD11/NPNT/HS3ST3B1/HOXA11/CITED1/STAT1/BMP2/SOX8/FOXD1/GLI3/GREB1L/VEGFA/OSR1/LIF/TACSTD2/HS3ST3A1/GDNF/EYA1/NOG/HOXB7 21

BP GO:0030902 hindbrain development 35/2475 157/18870 0.00102407432204861 0.00910895145122942 0.00661635854385559 KCNC1/CDK5R2/MDK/SSTR3/HOXB2/CDK5R1/KIF14/LEF1/HOXB3/SSTR2/HOXA2/FLNA/NEUROD2/FAIM2/ATF5/OPHN1/CD3E/GSX2/FCGR2B/LHX5/GLI1/OTX1/CBLN1/GDF10/SPTBN2/TBR1/MYO16/POU4F1/ITGB1/SSTR1/TUBA1A/NOG/EN2/EZH2/EN1 35

BP GO:0030501 positive regulation of bone mineralization 14/2475 44/18870 0.00104962046923671 0.009322368782984 0.00677137590166252 WNT4/CCN1/BMP2/NELL1/FAM20C/OSR1/TENT5A/LTF/ISG15/ADGRV1/ALOX5/WNT10B/OSR2/PTN 14

BP GO:0000086 G2/M transition of mitotic cell cycle 32/2475 140/18870 0.00106099860981139 0.00939613719863098 0.00682495817068063 CENPF/WEE1/KIF14/PLK3/USH1C/MELK/RBBP8/CHEK2/CCNB1/DTL/NABP1/BRSK2/FOXM1/ORC1/BRCA1/PKMYT1/CDK2/CCNA2/AURKA/CDC25C/AURKB/CLSPN/PLK1/CDC6/CDK4/CDK1/WNT10B/CDC25A/STOX1/PLCB1/CHEK1/CDK6 32

BP GO:0098581 detection of external biotic stimulus 10/2475 26/18870 0.00106105613269017 0.00939613719863098 0.00682495817068063 LY96/TLR1/TREM2/SSC5D/CLEC7A/HLA-DRB1/TLR2/HLA-B/HLA-A/LBP 10

BP GO:0021761 limbic system development 29/2475 123/18870 0.00106447852300643 0.00939871919430968 0.00682683362355359 KCNA1/CDK5R2/MDK/CDK5R1/SEMA3E/KIF14/LEF1/SLC32A1/FGF13/NEFL/NRP1/DLX2/HTR5A/NR2E1/GLI3/SCN2A/FEZF2/SEMA6B/LHX5/OTP/CASP3/CNTNAP2/TBR1/ALDH1A3/ZIC1/NEUROD6/TUBA1A/EZH2/CDK6 29

BP GO:0071774 response to fibroblast growth factor 29/2475 123/18870 0.00106447852300643 0.00939871919430968 0.00682683362355359 KCNC1/WNT4/NGFR/APLN/CXCL13/IQGAP1/CXCL8/SPRY2/THBS1/GALNT3/CD44/FGFRL1/CCL2/GPC1/SPRY4/ZFP36/SHCBP1/CCL5/COL1A1/FAM20C/RUNX2/FLRT1/TNC/POSTN/TBX1/FGFBP3/SPRY1/NOG/DLL4 29

BP GO:0002704 negative regulation of leukocyte mediated immunity 19/2475 69/18870 0.00108999527771631 0.00960988494479986 0.00698021552759666 IL7R/SPI1/FOXJ1/HAVCR2/AHR/IL13RA2/MICA/JAK3/FCGR2B/SPN/CD300A/HLA-B/LILRB1/HLA-A/PTPRC/HFE/IL4I1/HLA-F/BST2 19

BP GO:0035107 appendage morphogenesis 33/2475 146/18870 0.00109602741305163 0.00963477099262369 0.0069982917042042 SHOX2/SP8/HAND2/PITX1/LEF1/HOXC11/HOXA11/COL3A1/FREM2/HOXD9/MAP3K20/DLX5/GLI3/HOXA9/DKK1/SFRP2/TBX5/RUNX2/ECE1/PCSK5/OSR1/HOXD10/TWIST1/HOXA10/HOXC10/RDH10/TGFB2/OSR2/NOG/COL2A1/EN1/HOXD13/FZD6 33

BP GO:0035108 limb morphogenesis 33/2475 146/18870 0.00109602741305163 0.00963477099262369 0.0069982917042042 SHOX2/SP8/HAND2/PITX1/LEF1/HOXC11/HOXA11/COL3A1/FREM2/HOXD9/MAP3K20/DLX5/GLI3/HOXA9/DKK1/SFRP2/TBX5/RUNX2/ECE1/PCSK5/OSR1/HOXD10/TWIST1/HOXA10/HOXC10/RDH10/TGFB2/OSR2/NOG/COL2A1/EN1/HOXD13/FZD6 33

BP GO:0001774 microglial cell activation 15/2475 49/18870 0.00110199273138065 0.00965892607183857 0.00701583693601234 ITGB2/IFNGR2/TREM2/CTSC/TAFA3/C5AR1/TLR2/SPHK1/MAPT/LRRK2/TYROBP/CST7/PTPRC/IL6/C1QA 15

BP GO:0014911 positive regulation of smooth muscle cell migration 15/2475 49/18870 0.00110199273138065 0.00965892607183857 0.00701583693601234 MDM2/MDK/ITGB3/MIR221/S100A11/ITGA2/NRP1/IGFBP5/CYP1B1/P2RY6/CCN4/CCL5/HAS2/PDGFD/POSTN 15

BP GO:0016082 synaptic vesicle priming 8/2475 18/18870 0.00111379355187255 0.00969182764991972 0.00703973525607795 UNC13A/STX1A/STX1B/NAPB/STXBP1/UNC13C/ERC2/SNAP25 8

BP GO:0044070 regulation of monoatomic anion transport 8/2475 18/18870 0.00111379355187255 0.00969182764991972 0.00703973525607795 STC1/CA2/ATP8B1/TCAF2/GRM5/PRKG2/CA7/GABRE 8

BP GO:0061450 trophoblast cell migration 8/2475 18/18870 0.00111379355187255 0.00969182764991972 0.00703973525607795 TIMP1/ITGB3/ARHGDIB/ACVR1C/ITGB4/VEGFA/SYDE1/NODAL 8

BP GO:0061644 protein localization to CENP-A containing chromatin 8/2475 18/18870 0.00111379355187255 0.00969182764991972 0.00703973525607795 H4C9/CENPA/H4C5/H2AC8/H2BC11/H4C8/H4C11/H4C3 8

BP GO:0001885 endothelial cell development 18/2475 64/18870 0.00111704609156303 0.00969182764991972 0.00703973525607795 PDE2A/MSN/STC1/CLDN1/ICAM1/S1PR3/MET/TNFRSF1A/CLIC4/VEGFA/F2RL1/GPX1/F11R/RAP1B/COL27A1/MYD88/PLOD3/PLCB1 18

BP GO:0032615 interleukin-12 production 18/2475 64/18870 0.00111704609156303 0.00969182764991972 0.00703973525607795 MDK/THBS1/CD40/LILRA5/CLEC7A/JAK3/TLR2/IDO1/SCIMP/TLR8/ACP5/CD36/LAPTM5/HLA-B/LILRB1/UNC93B1/IRF1/PLCB1 18

BP GO:0032655 regulation of interleukin-12 production 18/2475 64/18870 0.00111704609156303 0.00969182764991972 0.00703973525607795 MDK/THBS1/CD40/LILRA5/CLEC7A/JAK3/TLR2/IDO1/SCIMP/TLR8/ACP5/CD36/LAPTM5/HLA-B/LILRB1/UNC93B1/IRF1/PLCB1 18

BP GO:0044403 biological process involved in symbiotic interaction 62/2475 322/18870 0.00112279750216446 0.00971350661120363 0.00705548194014808 CAV1/ITGA5/ITGB3/LEF1/MIR221/TNFRSF4/SIGLEC1/CXCL8/ITGA2/FUCA2/TFRC/CLDN1/ICAM1/NRP1/VAMP8/CD74/TRIM21/EGFR/TRIM38/TRIM22/CIITA/IFITM3/HLA-DRB1/APOL1/PLSCR1/F2RL1/GPX1/CTSB/HTR2A/CAV2/CCL5/CXCR4/CLEC5A/SCNN1B/IFITM2/CTSL/FN1/F11R/LTF/PPIA/ANPEP/NECTIN2/CCR5/LGALS1/TRIM6/TRIM5/EPHA2/EFNB2/ITGB1/IFI27/DPP4/CDK1/CXCL6/JPT2/PTX3/ZC3H12A/CHMP4BP1/TNFRSF14/MYD88/APOBEC3H/TREM1/SLC1A5 62

BP GO:0060713 labyrinthine layer morphogenesis 9/2475 22/18870 0.00112455995423464 0.00971350661120363 0.00705548194014808 RSPO3/LEF1/ST14/CCN1/SPINT1/FZD5/SOCS3/ADM/WNT7B 9

BP GO:0061687 detoxification of inorganic compound 9/2475 22/18870 0.00112455995423464 0.00971350661120363 0.00705548194014808 MT1M/SLC11A1/MT1A/MT2A/MT1DP/SLC39A8/MT1E/MT1H/MT3 9

BP GO:0051703 biological process involved in intraspecies interaction between organisms 16/2475 54/18870 0.00112740032223492 0.00971350661120363 0.00705548194014808 TREM2/CHRNB2/SHANK2/NR2E1/SHANK1/OXTR/NRXN1/BRINP1/LTF/NRXN3/CNTNAP2/GAD1/TBX1/MAPK8IP2/GRP/EN1 16

BP GO:0002886 regulation of myeloid leukocyte mediated immunity 17/2475 59/18870 0.0011308696071338 0.00971350661120363 0.00705548194014808 SPI1/ITGB2/FCGR1A/VAMP8/STXBP1/IL13RA2/ADGRE2/PRAM1/F2RL1/FCGR2B/FES/LYN/CD300A/TYROBP/RAC2/C3/CXCL6 17

BP GO:0046686 response to cadmium ion 17/2475 59/18870 0.0011308696071338 0.00971350661120363 0.00705548194014808 MT1M/SLC11A1/NCF1/MT1A/SOD2/MT2A/HMOX1/MT1DP/SLC39A8/EGFR/MT1E/MMP9/MT1H/PCNA/DTYMK/CDK1/MT3 17

BP GO:0060135 maternal process involved in female pregnancy 17/2475 59/18870 0.0011308696071338 0.00971350661120363 0.00705548194014808 WNT4/STC1/SPP1/ITGA3/HAVCR2/CSMD1/CNR1/DSG2/ANGPT2/CTSB/GJB2/CYP27B1/LIF/NR2F2/TPPP3/PTN/NODAL 17

BP GO:0007517 muscle organ development 67/2475 354/18870 0.00116146656115365 0.00996206461880935 0.00723602400430014 CAV1/CENPF/SHOX2/EFEMP2/PITX1/MEOX2/MYH7/LEF1/NKX2-5/BVES/TEAD4/MKX/RBFOX1/ITGA11/CHRNA1/COL3A1/LAMA2/HOXD9/FGFRL1/BMP2/SOX8/LOX/COL6A3/ENG/GPC1/CTF1/H1-5/CXCL10/GSC/GPX1/SELENON/CAV2/RYR2/FHL3/IGF2/MYOD1/DMRTA2/HLF/ZBTB42/YBX3/LIF/HOXD10/CHODL/FZD2/MYL6/TWIST1/NR2F2/RCAN1/MSC/VAMP5/STRA6/ELN/POU4F1/RBM24/ITGA7/TBX1/ITGB1/DES/FZD1/WNT10B/TAGLN/TGFB2/NEURL1/EVC/NOG/DLL4/ATF3 67

BP GO:0051346 negative regulation of hydrolase activity 45/2475 218/18870 0.00122451758876117 0.010487879604739 0.00761795385575123 TIMP1/PPP1R15A/IFI6/ANXA1/SERPINB1/THBS1/SERPINH1/CD44/LGALS3/MMP9/ECM1/KLRC4-KLRK1/SERPINA5/ANGPTL4/VEGFA/PPP4R4/GZMA/CARD16/IFI16/GPX1/SERPINB8/DPEP1/HGF/RPS6KA1/BCL2L12/CST3/LRRK2/SERPINE1/CAMK2A/SERPING1/LTF/LAMP3/CRB2/SFN/CST7/APOC1/SERPINA1/CRY2/CSTA/TGFB2/PLAUR/SERPINA3/PTX3/SERPINI1/AQP1 45

BP GO:0050864 regulation of B cell activation 30/2475 130/18870 0.0012723190783035 0.0108817717181399 0.00790406049094528 SPI1/TNFRSF4/CD40/TFRC/PAWR/CHRNB2/VAV3/FOXJ1/CD74/AHR/NFAM1/SLAMF8/TNFAIP3/MMP14/SAMSN1/FCGR2B/IL2RG/SASH3/BST1/LYN/CD300A/LAPTM5/TYROBP/CASP3/THEMIS2/PTPRC/IL6/IL4I1/CLCF1/GPR183 30

BP GO:0009306 protein secretion 69/2475 368/18870 0.00127798031231743 0.0109146426673596 0.00792793656349644 STX1A/IL1A/GSDMD/CHGA/CAVIN1/STXBP5L/VGF/IL1RN/ANXA1/ABCC8/TREM2/ACVR1C/KCNB1/CD33/CNR1/GLUD1/RAB3A/PRKCB/RAPGEF4/NNAT/PDIA4/BRSK2/SNCG/HLA-DRB1/SYT7/TLR2/RCN3/F2RL1/CCL5/STEAP3/VIP/GPR27/KCNN4/PCSK5/SYT4/RAB3C/KCNJ11/SNAP25/ANG/PPIA/NOS2/LTBP2/MTTP/CBLN1/F2RL2/NKX6-1/ADAM9/RSAD2/VSNL1/PRF1/P3H1/GCK/ALOX5/PCLO/TUNAR/PLEK/TRH/PRKCE/IL6/PPARG/RHBDF1/TGFB2/GAL/SAA1/TCIRG1/CPLX1/UCP2/PLCB1/RIMS2 69

BP GO:0033632 regulation of cell-cell adhesion mediated by integrin 6/2475 11/18870 0.00129489456747828 0.011012106208413 0.00799872997325559 CXCL13/CD3E/CCL5/DPP4/SWAP70/FERMT3 6

BP GO:1901731 positive regulation of platelet aggregation 6/2475 11/18870 0.00129489456747828 0.011012106208413 0.00799872997325559 MMRN1/PDPN/EMILIN2/F11R/EMILIN1/IL6 6

BP GO:1904238 pericyte cell differentiation 6/2475 11/18870 0.00129489456747828 0.011012106208413 0.00799872997325559 WNT4/SPI1/GPR4/ACTA2/OSR1/EPHA2 6

BP GO:0060047 heart contraction 49/2475 243/18870 0.00130080741635086 0.0110467436036359 0.00802388911775717 CAV1/MDM2/SHOX2/MAP2K3/APLN/MYH7/NKX2-5/BVES/STC1/CHGA/ACE/TMIGD3/FGF13/GCH1/TNNI2/HOPX/FGF12/ATP2B3/CTNNA3/FLNA/DSG2/SLC8A3/GPX1/HSPB7/SCN2B/RYR2/CXCR4/TBX5/SLC8A2/KCNE5/KCNE3/KCNJ3/GJC1/DSC2/KCNJ12/SCN3B/GLP1R/GATA4/DES/ADORA1/ATP2B2/PIK3CG/TGFB2/RGS4/ZC3H12A/KCNE4/GJD3/ADM/KCNIP2 49

BP GO:0099590 neurotransmitter receptor internalization 11/2475 31/18870 0.00131375511298851 0.0111409402519534 0.00809230959427805 ITGB3/DNM3/CACNG2/OPHN1/SNAP25/ARC/EFNB2/HPCA/CALY/CACNG5/CACNG3 11

BP GO:0030888 regulation of B cell proliferation 18/2475 65/18870 0.00135839801422621 0.0115032745802738 0.00835549398402373 TNFRSF4/CD40/TFRC/PAWR/CHRNB2/VAV3/CD74/AHR/FCGR2B/SASH3/BST1/LYN/CD300A/TYROBP/CASP3/PTPRC/CLCF1/GPR183 18

BP GO:0048880 sensory system development 74/2475 401/18870 0.00137195369425243 0.0115765588084683 0.00840872455967676 COL8A1/HCN1/COL8A2/MFAP2/SIX6/EFEMP1/USH1C/SLC17A8/SPRY2/PROM1/MAB21L1/MEIS3P1/CABP4/RET/MFAP5/NRP1/LCTL/SMOC1/SEMA3A/ATP8A2/FREM2/EGFR/TWSG1/PDE6B/WNT16/MEGF11/CRYGS/DSCAM/DLX2/CYP1B1/SOX8/NR2E1/SLC17A7/LAMC3/JAG1/GLI3/SIX5/CLIC4/VEGFA/VSX1/NEUROD4/FZD5/COL4A1/OLFM3/TUB/AQP5/SLC17A6/NKD1/TWIST1/RHOJ/CRB2/SPRED3/CELF4/VIM/STRA6/EPHA2/COL5A2/POU4F1/HPCA/C3/ALDH1A3/RDH10/ATP2B2/COL5A1/CRYGD/C1QA/TGFB2/SPRY1/OSR2/TCIRG1/AQP1/MEIS1/WNT7B/DLL4 74

BP GO:0050688 regulation of defense response to virus 24/2475 97/18870 0.00137419686229323 0.0115765588084683 0.00840872455967676 HERC5/PYCARD/PARP9/MMP12/TREM2/CGAS/APOBEC3G/LILRA4/STAT1/TRIM38/TRIM22/RAB7B/TNFAIP3/MICB/F2RL1/APOBEC3F/SPN/DTX3L/ZDHHC12/LILRB1/TRIM6/FGL2/ZDHHC1/ZC3H12A 24

BP GO:1903035 negative regulation of response to wounding 24/2475 97/18870 0.00137419686229323 0.0115765588084683 0.00840872455967676 THBD/WNT4/MMRN1/MDK/PLAT/SPP1/C1QTNF1/THBS1/RTN4R/RTN4RL1/SERPINE1/PDGFA/SERPING1/PLAU/TNR/PROS1/FAP/ALOX5/AJAP1/CD109/TFPI/PLAUR/ANXA2/SIGLEC10 24

BP GO:0035592 establishment of protein localization to extracellular region 69/2475 369/18870 0.00137476456203163 0.0115765588084683 0.00840872455967676 STX1A/IL1A/GSDMD/CHGA/CAVIN1/STXBP5L/VGF/IL1RN/ANXA1/ABCC8/TREM2/ACVR1C/KCNB1/CD33/CNR1/GLUD1/RAB3A/PRKCB/RAPGEF4/NNAT/PDIA4/BRSK2/SNCG/HLA-DRB1/SYT7/TLR2/RCN3/F2RL1/CCL5/STEAP3/VIP/GPR27/KCNN4/PCSK5/SYT4/RAB3C/KCNJ11/SNAP25/ANG/PPIA/NOS2/LTBP2/MTTP/CBLN1/F2RL2/NKX6-1/ADAM9/RSAD2/VSNL1/PRF1/P3H1/GCK/ALOX5/PCLO/TUNAR/PLEK/TRH/PRKCE/IL6/PPARG/RHBDF1/TGFB2/GAL/SAA1/TCIRG1/CPLX1/UCP2/PLCB1/RIMS2 69

BP GO:0031294 lymphocyte costimulation 15/2475 50/18870 0.00138603423812383 0.0116551114365483 0.00846578187903145 CAV1/EFNB1/LCK/MAP3K8/KLRC4-KLRK1/CD274/CD3E/PDCD1LG2/LYN/SPN/LILRB2/LGALS1/EFNB2/DPP4/TNFRSF14 15

BP GO:0071675 regulation of mononuclear cell migration 29/2475 125/18870 0.00139152345972373 0.0116849046883654 0.00848742244186295 ASCL2/RARRES2/MDK/ITGB3/PYCARD/CXCL13/SPI1/MSN/CCL20/THBS1/RIPK3/LGALS3/CCL2/ECM1/KLRC4-KLRK1/ITGA4/SLAMF8/CXCL10/C5AR1/CCL5/LYN/FPR2/SERPINE1/SPN/PDGFD/FADD/TNFRSF14/CCR1/PLCB1 29

BP GO:0061333 renal tubule morphogenesis 21/2475 81/18870 0.0014258385117331 0.0119396574957399 0.00867246414755959 WNT4/HOXD11/NPNT/HS3ST3B1/HOXA11/CITED1/BMP2/SOX8/FOXD1/GLI3/GREB1L/VEGFA/LGR5/COL4A1/OSR1/TACSTD2/HS3ST3A1/GDNF/EYA1/NOG/HOXB7 21

BP GO:0072028 nephron morphogenesis 21/2475 81/18870 0.0014258385117331 0.0119396574957399 0.00867246414755959 WNT4/HOXD11/NPNT/HS3ST3B1/HOXA11/CITED1/STAT1/BMP2/SOX8/FOXD1/GLI3/GREB1L/VEGFA/OSR1/LIF/TACSTD2/HS3ST3A1/GDNF/EYA1/NOG/HOXB7 21

BP GO:0090287 regulation of cellular response to growth factor stimulus 67/2475 357/18870 0.00145258117471654 0.0121466537228386 0.0088228174854869 ITGA5/WNT4/FERMT1/NGFR/APLN/ITGB3/TGFB1I1/CXCL13/NPNT/TET1/SPRY2/THBS1/ITGA3/NRP1/SFRP4/FSTL1/MIR210/CITED1/TWSG1/CCN1/BMP2/PRKCB/LOX/ENG/GPC1/FOXD1/SPRY4/LTBP1/CD63/SLC2A10/VEGFA/FOLR1/CAV2/DKK1/SFRP2/FAM20C/RUNX2/DCN/FSTL3/MIR27B/PCSK6/FSTL5/VWC2L/WFIKKN2/EMILIN1/CHRDL2/VEPH1/TNFAIP6/CRB2/SPRED3/ADAMTS3/FSTL4/VASN/FST/ASPN/GATA4/GREM2/CILP/FZD1/HTRA3/PPARG/FGFBP3/CD109/SPRY1/NOG/LATS2/MT3 67

BP GO:0001823 mesonephros development 25/2475 103/18870 0.00147875657466804 0.0123307899738587 0.00895656629995732 WNT4/HOXD11/NPNT/HS3ST3B1/HOXA11/RET/FOXJ1/CITED1/BMP2/SOX8/FOXD1/GLI3/GREB1L/VEGFA/OSR1/TACSTD2/SDC4/SDC1/HS3ST3A1/GDNF/SPRY1/OSR2/EYA1/NOG/HOXB7 25

BP GO:0045639 positive regulation of myeloid cell differentiation 25/2475 103/18870 0.00147875657466804 0.0123307899738587 0.00895656629995732 HOXA5/LEF1/MIR221/ROR2/TREM2/CD74/RUNX1/STAT1/JAG1/RAB7B/SCIN/HLA-DRB1/CASP8/CD101/FES/CSF3/LIF/ISG15/TYROBP/EVI2B/POU4F1/HMGB2/FADD/HCLS1/CCR1 25

BP GO:0031639 plasminogen activation 10/2475 27/18870 0.00148897780330572 0.0123307899738587 0.00895656629995732 PLAT/THBS1/RUNX1/S100A10/SERPINE1/PLAU/PGK1/CTSZ/PLAUR/ANXA2 10

BP GO:0042730 fibrinolysis 10/2475 27/18870 0.00148897780330572 0.0123307899738587 0.00895656629995732 THBD/PLAT/THBS1/SERPINE1/SERPING1/PLAU/PROS1/FAP/PLAUR/ANXA2 10

BP GO:0060669 embryonic placenta morphogenesis 10/2475 27/18870 0.00148897780330572 0.0123307899738587 0.00895656629995732 RSPO3/LEF1/ST14/CCN1/SPINT1/IGF2/FZD5/SOCS3/ADM/WNT7B 10

BP GO:0098698 postsynaptic specialization assembly 10/2475 27/18870 0.00148897780330572 0.0123307899738587 0.00895656629995732 NTNG2/C1QL3/GAP43/NRXN1/ABI3/ZDHHC12/CBLN1/IL1RAP/NPTX1/SPTBN2 10

BP GO:0150146 cell junction disassembly 10/2475 27/18870 0.00148897780330572 0.0123307899738587 0.00895656629995732 ABCC8/ADGRB3/TREM2/DKK1/SNAI2/C3/C1QB/ITGB1/C1QC/C1QA 10

BP GO:0006882 intracellular zinc ion homeostasis 12/2475 36/18870 0.00150117830120259 0.0123780770019809 0.00899091360482603 MT1M/LCK/MT1A/MT2A/MT1DP/SLC39A8/MT1E/S100A8/S100A9/SLC30A7/MT1H/MT3 12

BP GO:0045589 regulation of regulatory T cell differentiation 12/2475 36/18870 0.00150117830120259 0.0123780770019809 0.00899091360482603 MDK/FANCD2/SOCS1/HLA-DRB1/IL2RG/DUSP10/BTN2A2/LILRB2/HLA-DRA/IRF1/IL4I1/FANCA 12

BP GO:0052548 regulation of endopeptidase activity 56/2475 288/18870 0.00150275561967488 0.0123780770019809 0.00899091360482603 TIMP1/NGFR/PYCARD/HIP1R/IFI6/LCK/PSMB8/FAS/SERPINB1/THBS1/ST20/SERPINH1/ACVR1C/F3/CD44/CLEC7A/MMP9/BIRC3/S100A8/S100A9/CASP1/SERPINA5/CASP8/VEGFA/CARD16/IFI16/GPX1/GRIN2A/SERPINB8/DPEP1/HGF/LYN/RPS6KA1/MAPT/BCL2L12/MIR27B/CST3/GRIN1/SERPINE1/SERPING1/PSMB9/LTF/LAMP3/LAPTM5/CRB2/SFN/CST7/SERPINA1/PPARG/PLAUR/SERPINA3/FADD/SERPINI1/AQP1/GRIN2B/NODAL 56

BP GO:0008016 regulation of heart contraction 43/2475 208/18870 0.00150383957306667 0.0123780770019809 0.00899091360482603 CAV1/MDM2/SHOX2/APLN/MYH7/NKX2-5/BVES/STC1/CHGA/TMIGD3/FGF13/GCH1/HOPX/ATP2B3/CTNNA3/FLNA/DSG2/SLC8A3/HSPB7/SCN2B/RYR2/TBX5/SLC8A2/KCNE5/KCNE3/KCNJ3/GJC1/DSC2/KCNJ12/SCN3B/GLP1R/GATA4/DES/ADORA1/ATP2B2/PIK3CG/TGFB2/RGS4/ZC3H12A/KCNE4/GJD3/ADM/KCNIP2 43

BP GO:0042267 natural killer cell mediated cytotoxicity 20/2475 76/18870 0.00150499603788242 0.0123780770019809 0.00899091360482603 KLRC2/IL18/KLRC3/NKG7/KLRC4/HAVCR2/GZMB/MICA/RAB27A/KLRC4-KLRK1/NECTIN2/HLA-B/LILRB1/HLA-A/ULBP2/CD2/ULBP3/FCGR3A/PIK3R6/HLA-F 20

BP GO:0046883 regulation of hormone secretion 50/2475 251/18870 0.00153668869853902 0.0126135613543283 0.00916195951663491 HTR1A/STX1A/TSPO/SPP1/CHGA/ANXA1/ABCC8/C1QTNF1/GABBR1/ACVR1C/VAMP8/KCNB1/CRH/CNR1/GLUD1/PRKCB/RAPGEF4/NNAT/BRSK2/HLA-DRB1/SYT7/F2RL1/CCL5/IL11/GPR27/HTR2C/LIF/KCNJ11/SNAP25/NOS2/P2RY1/F2RL2/NKX6-1/VSNL1/CRY2/OSM/GCK/ALOX5/TUNAR/TRH/ADORA1/PRKCE/IL6/PPARG/GAL/HFE/TCIRG1/GRP/UCP2/PLCB1 50

BP GO:0003158 endothelium development 32/2475 143/18870 0.00153782926571758 0.0126135613543283 0.00916195951663491 PDE2A/MSN/STC1/CLDN1/ICAM1/NRP1/FSTL1/JAG1/S1PR3/MET/TNFRSF1A/CLIC4/ATOH8/VEGFA/CXCL10/F2RL1/GPX1/PDPN/CXCR4/F11R/ETV2/TMEM100/FZD2/RAP1B/NR2F2/BARX1/COL27A1/FZD1/MYD88/PLOD3/PLCB1/DLL4 32

BP GO:0071692 protein localization to extracellular region 70/2475 377/18870 0.00154292733786137 0.0126381115095767 0.0091797917150279 STX1A/IL1A/GSDMD/CHGA/CAVIN1/STXBP5L/VGF/IL1RN/ANXA1/ABCC8/TREM2/ACVR1C/KCNB1/CD33/CNR1/GLUD1/RAB3A/PRKCB/RAPGEF4/NNAT/PDIA4/BRSK2/SNCG/HLA-DRB1/LTBP1/SYT7/TLR2/RCN3/F2RL1/CCL5/STEAP3/VIP/GPR27/KCNN4/PCSK5/SYT4/RAB3C/KCNJ11/SNAP25/ANG/PPIA/NOS2/LTBP2/MTTP/CBLN1/F2RL2/NKX6-1/ADAM9/RSAD2/VSNL1/PRF1/P3H1/GCK/ALOX5/PCLO/TUNAR/PLEK/TRH/PRKCE/IL6/PPARG/RHBDF1/TGFB2/GAL/SAA1/TCIRG1/CPLX1/UCP2/PLCB1/RIMS2 70

BP GO:0002688 regulation of leukocyte chemotaxis 29/2475 126/18870 0.00158537729128532 0.012968127052966 0.0094195011010972 RARRES2/MDK/CXCL13/SPI1/CXCL8/THBS1/CD74/CCL2/KLRC4-KLRK1/SLAMF8/VEGFA/CXCL10/C5AR1/F2RL1/CCL5/BST1/LYN/FPR2/SERPINE1/THBS4/TNFAIP6/RAC2/CXCR2/DPP4/IL6/LBP/SWAP70/PTN/CCR1 29

BP GO:0001657 ureteric bud development 24/2475 98/18870 0.00159767820806581 0.0130509659336424 0.00947967177374389 WNT4/HOXD11/NPNT/HS3ST3B1/HOXA11/RET/FOXJ1/CITED1/BMP2/SOX8/FOXD1/GLI3/GREB1L/VEGFA/OSR1/TACSTD2/SDC4/SDC1/HS3ST3A1/GDNF/SPRY1/EYA1/NOG/HOXB7 24

BP GO:0002468 dendritic cell antigen processing and presentation 7/2475 15/18870 0.0016249390359197 0.013147423603328 0.00954973456092217 SLC11A1/THBS1/CD74/HLA-DRB1/FCGR2B/HLA-DRA/FGL2 7

BP GO:0043518 negative regulation of DNA damage response, signal transduction by p53 class mediator 7/2475 15/18870 0.0016249390359197 0.013147423603328 0.00954973456092217 MDM2/SNAI1/CD74/CD44/TWIST1/SNAI2/DYRK3 7

BP GO:0044849 estrous cycle 7/2475 15/18870 0.0016249390359197 0.013147423603328 0.00954973456092217 MDK/CA12/CYP1B1/OXTR/HAS2/PCNA/PTN 7

BP GO:0048934 peripheral nervous system neuron differentiation 7/2475 15/18870 0.0016249390359197 0.013147423603328 0.00954973456092217 HAND2/HOXD9/RUNX1/NEFH/RUNX3/HOXD10/POU4F1 7

BP GO:0048935 peripheral nervous system neuron development 7/2475 15/18870 0.0016249390359197 0.013147423603328 0.00954973456092217 HAND2/HOXD9/RUNX1/NEFH/RUNX3/HOXD10/POU4F1 7

BP GO:0099509 regulation of presynaptic cytosolic calcium ion concentration 7/2475 15/18870 0.0016249390359197 0.013147423603328 0.00954973456092217 TSPOAP1/ATP2B3/CNR1/ERC2/SV2B/P2RY1/ADORA1 7

BP GO:0045066 regulatory T cell differentiation 13/2475 41/18870 0.00162700461979892 0.013147423603328 0.00954973456092217 MDK/FANCD2/SOCS1/TOX/HLA-DRB1/IL2RG/DUSP10/BTN2A2/LILRB2/HLA-DRA/IRF1/IL4I1/FANCA 13

BP GO:0051954 positive regulation of amine transport 13/2475 41/18870 0.00162700461979892 0.013147423603328 0.00954973456092217 STX1A/SLC17A8/GABBR1/CHRNB2/KCNB1/STXBP1/OXTR/VIP/SYT1/SYT4/ITGB1/TRH/GDNF 13

BP GO:0098664 G protein-coupled serotonin receptor signaling pathway 9/2475 23/18870 0.00163425499771179 0.0131758553211168 0.00957038616280145 HTR1A/HTR1D/HTR5A/CHRM1/HTR2A/HRH1/HTR2C/HRH3/CHRM4 9

BP GO:0007178 transmembrane receptor protein serine/threonine kinase signaling pathway 75/2475 410/18870 0.00163491209430913 0.0131758553211168 0.00957038616280145 FERMT1/TGFB1I1/FMOD/LEF1/WWTR1/SPI1/NPNT/LRRC32/ZYX/TET1/SPRY2/THBS1/ITGA3/ACVR1C/SFRP4/COL3A1/FSTL1/MIR210/CITED1/TWSG1/CCN1/BMP2/LOX/DLX5/ENG/FOXD1/ATOH8/LTBP1/SMPD3/SLC2A10/FOLR1/CAV2/DKK1/SFRP2/RUNX2/FSTL3/ETV2/MIR27B/PCSK6/COL1A2/FSTL5/TMEM100/VWC2L/WFIKKN2/EMILIN1/CHRDL2/LTBP2/VEPH1/TNFAIP6/CRB2/SPRED3/ADAM9/VIM/FSTL4/GDF10/VASN/FST/ASPN/GATA4/GREM2/CILP/FZD1/SMAD9/HTRA3/PPARG/CD109/TGFB2/SPRY1/PPM1L/HFE/NOG/RBPMS/LATS2/NODAL/GDF15 75

BP GO:0042476 odontogenesis 30/2475 132/18870 0.00164234563925172 0.0132180203995541 0.00960101309922377 HAND2/NGFR/LEF1/DLX2/BMP2/PRKCB/GLI3/SP6/PHEX/SNX10/SMPD3/COL1A1/FAM20C/RUNX2/OSR1/SERPINE1/COL1A2/AQP5/TNC/LAMB1/TNFRSF11B/FST/ASPN/TBX1/FAM20A/TGFB2/OSR2/TCIRG1/AQP1/ADM 30

BP GO:0015850 organic hydroxy compound transport 59/2475 308/18870 0.00165167770306488 0.0132753318998682 0.00964264175832845 CAV1/HTR1A/CES1/STX1A/ITGB3/TSPO/OSBPL3/SLC10A4/SPP1/CHGA/EMB/C1QTNF1/TREM2/GABBR1/CHRNB2/ATP8B1/KCNB1/CRH/PON1/ABCG4/CNR1/PRKCB/OXTR/SNCG/SYT7/GRAMD1B/HTR2A/VIP/ABCB4/SYT1/MIR27B/SYT4/SLCO1A2/SLC16A3/GRM2/CD36/MTTP/P2RY1/LILRB1/NPC2/HRH3/APOC1/STRA6/SYT5/OSBPL10/MSR1/CHRNA4/AQP9/GDNF/PPARG/STAR/ABCC3/SYT2/SYT13/GAL/ANXA2/AQP1/ADRA2B/MAOB 59

BP GO:0044344 cellular response to fibroblast growth factor stimulus 27/2475 115/18870 0.00165935976145092 0.0133192459996675 0.00967453911017613 WNT4/NGFR/APLN/CXCL13/IQGAP1/CXCL8/SPRY2/THBS1/GALNT3/CD44/FGFRL1/CCL2/GPC1/SPRY4/ZFP36/SHCBP1/CCL5/COL1A1/FAM20C/RUNX2/FLRT1/POSTN/TBX1/FGFBP3/SPRY1/NOG/DLL4 27

BP GO:1990138 neuron projection extension 38/2475 179/18870 0.00166880105764553 0.0133771449267073 0.00971659445130409 UNC13A/EMX1/SEMA3E/IQGAP1/NRP1/RIMS1/SEMA3A/CDH4/SEMA3D/SLIT1/DSCAM/RTN4R/SH3GL2/ITGA4/CPNE9/VEGFA/L1CAM/CXCR4/SEMA6B/FN1/S100B/SYT1/MAPT/SYT4/RASAL1/FLRT1/AURKA/TNR/SEMA3F/NKX6-1/DRAXIN/POSTN/ITGB1/TRPV2/SYT2/ISLR2/MT3/RIMS2 38

BP GO:0002700 regulation of production of molecular mediator of immune response 40/2475 191/18870 0.00167488988564985 0.0133901982336108 0.0097260758234597 PYCARD/IL18/TNFRSF4/LITAF/KLK7/CGAS/CD40/TFRC/HPX/CD74/IL13RA2/IL1R1/ARID5A/CLEC7A/SPON2/ANGPT1/JAK3/F2RL1/FCGR2B/SCIMP/SASH3/FZD5/ACP5/CD36/LAPTM5/TWIST1/LILRB1/TRIM6/HLA-A/RSAD2/PTPRC/B2M/IL6/TGFB2/TNFRSF14/HFE/MYD88/CLCF1/HLA-F/BST2 40

BP GO:0021543 pallium development 40/2475 191/18870 0.00167488988564985 0.0133901982336108 0.0097260758234597 KCNA1/CDK5R2/MDK/EMX1/CDK5R1/KIF14/LEF1/SLC32A1/KIF26A/FGF13/NEFL/COL3A1/FLNA/EGFR/DLX2/TACC3/HTR5A/NR2E1/GLI3/SCN2A/FEZF2/SEMA6B/DMRTA2/LHX5/LAMB1/CASP3/CNTNAP2/PHACTR1/NDE1/TBR1/ZIC1/WDR62/LHX6/NEUROD6/TUBA1A/ASPM/EZH2/PLCB1/MGARP/CDK6 40

BP GO:0010001 glial cell differentiation 50/2475 252/18870 0.00167807828281406 0.013397848417574 0.00973163259476295 MDK/EMX1/LEF1/TSPAN2/ROR2/GPR157/TREM2/LAMA2/HAPLN1/DLX2/BMP2/SOX8/NR2E1/GAP43/MXRA8/ITGB4/CDKN2C/LAMC3/SPINT1/S100A8/GPC1/GLI3/ROR1/S100A9/GSX2/C5AR1/TLR2/SLC8A3/CXCR4/NEUROD4/DUSP10/LYN/MAPT/LIF/SOX10/NCMAP/NKX6-1/HAPLN3/VIM/DAAM2/CDK1/IL6/C1QA/TUBA1A/MYD88/NOG/PTN/CLCF1/CDK6/TP73 50

BP GO:0003179 heart valve morphogenesis 17/2475 61/18870 0.0016929180992595 0.0134623881299102 0.00977851152253201 MDM2/SNAI1/NKX2-5/FGFRL1/CCN1/BMP2/JAG1/MDM4/TBX5/EMILIN1/TWIST1/STRA6/ELN/SNAI2/GATA4/TGFB2/DLL4 17

BP GO:0034113 heterotypic cell-cell adhesion 17/2475 61/18870 0.0016929180992595 0.0134623881299102 0.00977851152253201 ITGA5/ITGB3/MIR221/ITGB2/LCK/IL1RN/CTNNA3/CD44/DSG2/ITGA4/CD58/LILRB2/DSC2/CD2/ITGA7/PTPRC/ITGB1 17

BP GO:0002281 macrophage activation involved in immune response 8/2475 19/18870 0.00170409976327978 0.0134623881299102 0.00977851152253201 TREM2/HAVCR2/SBNO2/NMI/TYROBP/IFI35/PRKCE/LBP 8

BP GO:0002283 neutrophil activation involved in immune response 8/2475 19/18870 0.00170409976327978 0.0134623881299102 0.00977851152253201 SPI1/ITGB2/PRAM1/SCNN1B/TYROBP/LILRA2/FCER1G/MYD88 8

BP GO:0002544 chronic inflammatory response 8/2475 19/18870 0.00170409976327978 0.0134623881299102 0.00977851152253201 CXCL13/NFKBIZ/THBS1/S100A8/S100A9/TNFAIP3/IDO1/CCL5 8

BP GO:0030903 notochord development 8/2475 19/18870 0.00170409976327978 0.0134623881299102 0.00977851152253201 STIL/TEAD2/GLI1/CRB2/EPHA2/COL27A1/NOG/COL2A1 8

BP GO:0048266 behavioral response to pain 8/2475 19/18870 0.00170409976327978 0.0134623881299102 0.00977851152253201 TSPO/THBS1/PIRT/THBS4/SCN3A/NTRK1/VWA1/ADAM11 8

BP GO:0071625 vocalization behavior 8/2475 19/18870 0.00170409976327978 0.0134623881299102 0.00977851152253201 SHANK2/SHANK1/SRPX2/GLI3/NRXN1/BRINP1/NRXN3/CNTNAP2 8

BP GO:0030857 negative regulation of epithelial cell differentiation 16/2475 56/18870 0.00172400426877969 0.0135838866532195 0.00986676293072835 CAV1/IL1A/SPRY2/STAT1/MMP9/JAG1/S1PR3/VEGFA/FRZB/OSR1/SPRED3/HOXA7/FST/SPRY1/EZH2/NODAL 16

BP GO:0052372 modulation by symbiont of entry into host 16/2475 56/18870 0.00172400426877969 0.0135838866532195 0.00986676293072835 CAV1/CXCL8/FUCA2/CD74/TRIM21/TRIM38/TRIM22/CIITA/IFITM3/HLA-DRB1/IFITM2/NECTIN2/LGALS1/TRIM6/TRIM5/PTX3 16

BP GO:0045471 response to ethanol 28/2475 121/18870 0.00173536431294652 0.013655474881954 0.00991876160383174 CLDN7/RGS7/CTSK/SOD2/GABBR1/CLDN1/CHRNB2/KCNMB1/CNR1/PRKCB/S100A8/TYMS/GRIN3A/CASP8/GRIN2A/SLC2A4/CSF3/GRIN1/CD14/TNC/ADCYAP1R1/KCNC2/CDK1/PRKCE/RGS4/MYD88/GRIN2B/MAOB 28

BP GO:2000463 positive regulation of excitatory postsynaptic potential 11/2475 32/18870 0.00176735523415175 0.013879368036542 0.0100813881579637 STX1A/STX1B/RIMS1/CUX2/SHANK1/NRXN1/GRIN2A/GRIN1/RGS4/GRIN2B/RIMS2 11

BP GO:0051092 positive regulation of NF-kappaB transcription factor activity 33/2475 150/18870 0.00176844046434954 0.013879368036542 0.0100813881579637 CAV1/TRADD/PYCARD/IL18/TRAF5/CD40/TFRC/S100A12/TRIM21/RIPK3/CLEC7A/TRIM38/TRIM22/PRKCB/S100A8/NTS/ROR1/S100A9/RAB7B/TLR2/CARD16/SPHK1/CAMK2A/LTF/PPIA/CD36/IL1RAP/TRIM6/TRIM5/NTRK1/IRAK2/MYD88/TRAF1 33

BP GO:0014013 regulation of gliogenesis 26/2475 110/18870 0.00180871619656279 0.0141769347835026 0.0102975281054825 ASCL2/MDK/MIR221/TSPO/ABCC8/TREM2/DLX2/BMP2/SOX8/NR2E1/SPINT1/GSX2/TLR2/CXCR4/DUSP10/LYN/LIF/SOX10/NKX6-1/DAAM2/IL6/NOG/EZH2/PTN/CLCF1/TP73 26

BP GO:0072163 mesonephric epithelium development 24/2475 99/18870 0.00185156628701086 0.0144750051916839 0.0105140338912911 WNT4/HOXD11/NPNT/HS3ST3B1/HOXA11/RET/FOXJ1/CITED1/BMP2/SOX8/FOXD1/GLI3/GREB1L/VEGFA/OSR1/TACSTD2/SDC4/SDC1/HS3ST3A1/GDNF/SPRY1/EYA1/NOG/HOXB7 24

BP GO:0072164 mesonephric tubule development 24/2475 99/18870 0.00185156628701086 0.0144750051916839 0.0105140338912911 WNT4/HOXD11/NPNT/HS3ST3B1/HOXA11/RET/FOXJ1/CITED1/BMP2/SOX8/FOXD1/GLI3/GREB1L/VEGFA/OSR1/TACSTD2/SDC4/SDC1/HS3ST3A1/GDNF/SPRY1/EYA1/NOG/HOXB7 24

BP GO:0030072 peptide hormone secretion 48/2475 241/18870 0.00189024038427837 0.0147581316868756 0.0107196850483407 STX1A/SLC16A10/CHGA/VGF/IL1RN/ANXA1/ABCC8/GABBR1/ACVR1C/KCNB1/CRH/CNR1/GLUD1/RAB3A/PRKCB/RAPGEF4/NNAT/BRSK2/HLA-DRB1/SMPD3/SYT7/F2RL1/CCL5/VIP/GPR27/HTR2C/KCNJ11/SNAP25/NOS2/F2RL2/NKX6-1/VSNL1/GCK/ALOX5/PCLO/TUNAR/TRH/PRKCE/IL6/GAL/HFE/TCIRG1/AQP1/CPLX1/GRP/UCP2/PLCB1/RIMS2 48

BP GO:0046634 regulation of alpha-beta T cell activation 27/2475 116/18870 0.0018977162885867 0.0147972579177591 0.0107481046939367 ASCL2/BATF/IL18/NFKBIZ/LOXL3/ANXA1/SOCS1/TWSG1/RUNX1/CD274/GLI3/HLA-DRB1/CD3E/JAK3/IL2RG/SASH3/RUNX3/HLA-DRA/CD300A/LILRB1/HLA-A/PTPRC/IL2RA/ZC3H12A/TNFRSF14/IRF1/HFE 27

BP GO:0019932 second-messenger-mediated signaling 59/2475 310/18870 0.00193025920965082 0.0150314867636102 0.0109182386587253 AKAP5/AKAP6/PDE2A/CXCL8/CCL20/THBS1/TREM2/CGAS/EGFR/AHR/APLNR/NEUROD2/GBP1/CLEC7A/RCAN3/CD3E/VEGFA/KSR2/GRIN2A/TRPM8/RYR2/CXCR4/SPHK1/GRM5/BST1/HTR2C/FPR2/SLC8A2/MAPT/GRIN1/LRRK2/KCNJ11/RGN/TMEM100/NOS2/ADCYAP1R1/CD36/GPR3/CCR5/RCAN1/RUNDC3A/CXCR2/LAT2/KCNC2/CCRL2/HPCA/GLP1R/PLEK/PTPRC/JPT2/FCGR3A/GPR61/GAL/AQP1/NMUR2/GRIN2B/ADM/FPR1/CCR1 59

BP GO:0003230 cardiac atrium development 12/2475 37/18870 0.00196000574136934 0.0152236409717742 0.0110578113794419 MDM2/SHOX2/NKX2-5/CCN1/BMP2/ENG/MDM4/TBX5/GATA4/TGFB2/NOG/DLL4 12

BP GO:0097106 postsynaptic density organization 12/2475 37/18870 0.00196000574136934 0.0152236409717742 0.0110578113794419 ITGB3/SHANK2/C1QL3/SHANK1/OPHN1/NRXN1/ABI3/ZDHHC12/LILRB2/CBLN1/IL1RAP/NPTX1 12

BP GO:0060675 ureteric bud morphogenesis 18/2475 67/18870 0.00197522384391436 0.0153022502694991 0.011114909861239 WNT4/HOXD11/NPNT/HS3ST3B1/HOXA11/CITED1/BMP2/SOX8/FOXD1/GLI3/GREB1L/VEGFA/TACSTD2/HS3ST3A1/GDNF/EYA1/NOG/HOXB7 18

BP GO:0060760 positive regulation of response to cytokine stimulus 18/2475 67/18870 0.00197522384391436 0.0153022502694991 0.011114909861239 IRF7/PARP9/RBM47/MMP12/TREM2/HPX/CD74/IL1R1/CASP1/CD300LF/TLR2/CXCR4/IFIH1/NLRC5/LAPTM5/TRIM6/FADD/CASP4 18

BP GO:0021766 hippocampus development 23/2475 94/18870 0.00200186600741954 0.0154633186168866 0.0112319031224379 KCNA1/CDK5R2/MDK/CDK5R1/KIF14/LEF1/SLC32A1/FGF13/NEFL/DLX2/HTR5A/NR2E1/GLI3/SCN2A/FEZF2/SEMA6B/LHX5/CASP3/ZIC1/NEUROD6/TUBA1A/EZH2/CDK6 23

BP GO:0001894 tissue homeostasis 54/2475 279/18870 0.00200374115322081 0.0154633186168866 0.0112319031224379 ITGB3/NFKBIZ/WWTR1/CTSK/USH1C/HAMP/SPP1/CYTL1/PROM1/ABCC8/TFRC/CLDN1/SLC39A8/COL3A1/NOX4/IGHA1/LAMA2/HSPB1/TFF3/SH3GL2/TNFAIP3/SNX10/ANGPT1/VEGFA/IGHG3/IGKC/SASH3/IGHA2/ACP5/LAMC1/JCHAIN/CDHR1/LYZ/F11R/IL10RA/TUB/PCDH15/IL20RA/LTF/TNFRSF11B/ADGRV1/RAC2/CRB2/PIWIL4/FOSL2/MAK/ITGB1/B2M/IL6/SERPINA3/TUBA1A/TCIRG1/COL2A1/CUBN 54

BP GO:0060249 anatomical structure homeostasis 54/2475 279/18870 0.00200374115322081 0.0154633186168866 0.0112319031224379 ITGB3/NFKBIZ/WWTR1/CTSK/USH1C/HAMP/SPP1/CYTL1/PROM1/ABCC8/TFRC/CLDN1/SLC39A8/COL3A1/NOX4/IGHA1/LAMA2/HSPB1/TFF3/SH3GL2/TNFAIP3/SNX10/ANGPT1/VEGFA/IGHG3/IGKC/SASH3/IGHA2/ACP5/LAMC1/JCHAIN/CDHR1/LYZ/F11R/IL10RA/TUB/PCDH15/IL20RA/LTF/TNFRSF11B/ADGRV1/RAC2/CRB2/PIWIL4/FOSL2/MAK/ITGB1/B2M/IL6/SERPINA3/TUBA1A/TCIRG1/COL2A1/CUBN 54

BP GO:0097553 calcium ion transmembrane import into cytosol 42/2475 205/18870 0.00202503543517754 0.0156075901833195 0.0113366959096555 AKAP5/CAV1/AKAP6/DDIT3/ITGB3/JPH4/LCK/CYBA/CACNA1B/FLNA/APLNR/P2RY6/SCN2A/CXCL10/SLC8A3/SELENON/GRIN2A/HTR2A/RYR2/LYN/HTR2C/SLC8A2/SCN8A/GRIN1/CACNA1E/CXCL9/ADCYAP1R1/F2RL3/CCR5/RYR3/MCOLN2/JPH3/PTPRC/SCN3A/PRKCE/TRPV2/CXCL11/FKBP1C/RAMP3/CACNA1I/GRIN2B/PLCB1 42

BP GO:0045930 negative regulation of mitotic cell cycle 49/2475 248/18870 0.00203050784541336 0.01562970397931 0.0113527584393354 CENPF/SPC24/MDM2/MAD2L1/WEE1/PLK3/FANCD2/E2F7/KNTC1/RBBP8/CHEK2/CCL2/MAP3K20/CCNB1/TOM1L1/DTL/NABP1/GPNMB/ORC1/BIRC5/BRCA1/BRINP1/HASPIN/TTK/MUC1/BTG3/ZWILCH/BTN2A2/NUF2/CDK2/TRIP13/BUB1B/BUB1/AURKB/CLSPN/MYO16/PLK1/CDC6/CDCA8/NDC80/GAS1/CDK1/GTSE1/KNL1/ZWINT/EZH2/CHEK1/EME1/CDC20 49

BP GO:0003094 glomerular filtration 10/2475 28/18870 0.00204689064531666 0.0157155133433263 0.0114150867459239 EMP2/IGKV3-20/IGHA1/MCAM/F2RL1/TMEM63C/IGHA2/JCHAIN/ADORA1/AQP1 10

BP GO:0031664 regulation of lipopolysaccharide-mediated signaling pathway 10/2475 28/18870 0.00204689064531666 0.0157155133433263 0.0114150867459239 LY96/CD180/TNFAIP3/CARD16/SCIMP/CD14/LTF/CD36/LILRA2/TRIM5 10

BP GO:0010717 regulation of epithelial to mesenchymal transition 26/2475 111/18870 0.00207298182231811 0.0158686496397369 0.0115263184994968 MDK/LOXL2/TGFB1I1/EMP2/SNAI1/LEF1/WWTR1/MIR221/SPRY2/BMP2/JAG1/ENG/PDPN/SFRP2/TBX5/COL1A1/TWIST1/CRB2/SPRED3/VASN/EPHA3/IL6/TGFB2/SPRY1/NOG/EZH2 26

BP GO:0060840 artery development 26/2475 111/18870 0.00207298182231811 0.0158686496397369 0.0115263184994968 PDE2A/EFEMP2/MDK/DDIT3/HAND2/NGFR/NRP1/COL3A1/APLNR/LOX/JAG1/ENG/GLI3/SLC2A10/VEGFA/FOLR1/STRA6/EFNB2/LOXL1/TBX1/FKBP10/TGFB2/EYA1/HOXA1/NOG/DLL4 26

BP GO:0021700 developmental maturation 62/2475 330/18870 0.0020767016205688 0.0158686496397369 0.0115263184994968 CDK5R2/FERMT1/DDIT3/UNC13A/CDK5R1/KIF14/HOXA5/ACTL6B/ADGRB3/RAB32/NEFL/RET/MMP2/FOXJ1/KCNB1/ROPN1L/STXBP1/NEUROD2/SRRM4/CLEC7A/BMP2/RAB3A/SOX8/UNC13C/SHANK1/TYMS/NRXN1/CD63/SNX10/ATP6V1G2/VEGFA/TFCP2L1/VSX1/FZD5/FAM20C/RUNX2/LRRK2/ANG/LTF/AURKA/SOX10/TRIP13/H3-3A/BRCA2/SYP/NKX6-1/CNTNAP2/C3/WNT10B/HOXB13/PPARG/LHX6/C1QA/TGFB2/SEZ6L/ADAMTS7/NEURL1/GAL/RAB38/PFN1/CDC20/KCNIP2 62

BP GO:0032733 positive regulation of interleukin-10 production 13/2475 42/18870 0.00207740816402952 0.0158686496397369 0.0115263184994968 PYCARD/RBM47/TREM2/BCL3/LILRA5/CLEC7A/CD274/TLR2/F2RL1/SASH3/HGF/ISG15/IL6 13

BP GO:0002448 mast cell mediated immunity 16/2475 57/18870 0.00210969760167638 0.0160712440198518 0.0116734745212395 CHGA/CPLX2/VAMP8/STXBP1/IL13RA2/S100A13/ADGRE2/SPON2/FES/LYN/CD300A/RAC2/LAT2/MILR1/PIK3CG/GRP 16

BP GO:0007080 mitotic metaphase chromosome alignment 16/2475 57/18870 0.00210969760167638 0.0160712440198518 0.0116734745212395 KIFC1/KIF14/CDCA5/PSRC1/CCNB1/ANKRD53/BIRC5/NUF2/KIF18A/AURKB/KIF2C/CDT1/CENPE/CDCA8/NDC80/CHMP4BP1 16

BP GO:0035418 protein localization to synapse 20/2475 78/18870 0.00211196061486727 0.0160712440198518 0.0116734745212395 CACNG2/STX1B/LRRC7/HSPB1/C1QL3/SHANK1/GRIP1/NRXN1/GRIN2A/BSN/ASIC2/MAPT/DLG2/SNAP25/ZDHHC12/NPTX1/PCLO/ARHGAP44/CPLX1/CACNG3 20

BP GO:0099054 presynapse assembly 14/2475 47/18870 0.00213298282307352 0.0161811231922169 0.0117532861224718 IL1RAPL1/NTNG2/SLITRK4/LRRTM3/CBLN2/BSN/DKK1/SLITRK1/CBLN1/IL1RAP/LRFN5/PCLO/FZD1/SLITRK5 14

BP GO:0060349 bone morphogenesis 24/2475 100/18870 0.00213910521982011 0.0161811231922169 0.0117532861224718 SHOX2/STC1/RIPPLY2/HOXA11/SP5/SERPINH1/MMP13/SFRP4/COL3A1/DLX5/GLI3/MMP14/SMPD3/HAS2/COL1A1/ACP5/RUNX2/PAPPA2/LTF/TWIST1/FOSL2/COL27A1/OSR2/COL2A1 24

BP GO:0035176 social behavior 15/2475 52/18870 0.00213987538551303 0.0161811231922169 0.0117532861224718 TREM2/CHRNB2/SHANK2/NR2E1/SHANK1/OXTR/NRXN1/BRINP1/NRXN3/CNTNAP2/GAD1/TBX1/MAPK8IP2/GRP/EN1 15

BP GO:0043154 negative regulation of cysteine-type endopeptidase activity involved in apoptotic process 15/2475 52/18870 0.00213987538551303 0.0161811231922169 0.0117532861224718 IFI6/THBS1/CD44/MMP9/VEGFA/CARD16/GPX1/DPEP1/HGF/RPS6KA1/BCL2L12/LAMP3/SFN/PLAUR/AQP1 15

BP GO:0050819 negative regulation of coagulation 15/2475 52/18870 0.00213987538551303 0.0161811231922169 0.0117532861224718 THBD/PLAT/C1QTNF1/THBS1/SERPINE1/PDGFA/SERPING1/PLAU/PROS1/ANXA5/FAP/TFPI/PLAUR/PROCR/ANXA2 15

BP GO:0150063 visual system development 72/2475 395/18870 0.00219709142773254 0.0165928766441587 0.0120523664814246 COL8A1/HCN1/COL8A2/MFAP2/SIX6/EFEMP1/USH1C/SLC17A8/SPRY2/PROM1/MAB21L1/MEIS3P1/CABP4/RET/MFAP5/NRP1/LCTL/SMOC1/ATP8A2/FREM2/EGFR/TWSG1/PDE6B/WNT16/MEGF11/CRYGS/DSCAM/DLX2/CYP1B1/SOX8/NR2E1/SLC17A7/LAMC3/JAG1/GLI3/SIX5/CLIC4/VEGFA/VSX1/NEUROD4/FZD5/COL4A1/OLFM3/TUB/AQP5/SLC17A6/NKD1/TWIST1/RHOJ/CRB2/SPRED3/CELF4/VIM/STRA6/EPHA2/COL5A2/HPCA/C3/ALDH1A3/RDH10/ATP2B2/COL5A1/CRYGD/C1QA/TGFB2/SPRY1/OSR2/TCIRG1/AQP1/MEIS1/WNT7B/DLL4 72

BP GO:0030330 DNA damage response, signal transduction by p53 class mediator 19/2475 73/18870 0.00224063432481986 0.0168955708386679 0.0122722307908063 SP100/MDM2/BATF/SNAI1/PLK3/E2F7/SMYD2/BCL3/CHEK2/CD74/CD44/FOXM1/MDM4/MUC1/TWIST1/BRCA2/SNAI2/GTSE1/DYRK3 19

BP GO:0010811 positive regulation of cell-substrate adhesion 28/2475 123/18870 0.0022446061076594 0.0168955708386679 0.0122722307908063 COL8A1/ITGA5/WNT4/FERMT1/EFEMP2/MDK/ITGB3/COL26A1/EMP2/NPNT/ECM2/NID1/ITGA3/NRP1/FLNA/CCN1/S100A10/CD3E/VEGFA/EGFLAM/HAS2/FN1/CD36/EMILIN1/SDC4/RRAS/PRKCE/CDK6 28

BP GO:0098754 detoxification 34/2475 158/18870 0.00224561384564573 0.0168955708386679 0.0122722307908063 SLC47A2/MT1M/PXDNL/SLC11A1/H19/SLC47A1/MT1A/IPCEF1/SOD2/MT2A/MTARC2/MT1DP/HBQ1/GCH1/SLC39A8/MT1E/S100A9/PTGS1/GPX1/HP/HBA1/ALOX5AP/SOD3/PTGES/GPX7/MGST2/MT1H/CD36/FBLN5/SELENOF/PRDX4/SLC22A18/GPX8/MT3 34

BP GO:0048839 inner ear development 40/2475 194/18870 0.00226025763773416 0.0169844641513841 0.0123367991477935 CHRNA9/MCM2/USH1C/SLC17A8/CYTL1/SPRY2/ATP8B1/ATP8A2/GABRA5/FREM2/BMP2/JAG1/DLX5/GLI3/ROR1/FRZB/LGR5/GJB2/KCNK3/PCDH15/CEBPD/CTHRC1/FZD2/CCNA2/OTX1/ADGRV1/GABRB2/SDC4/HPCA/TBX1/ALDH1A3/ZIC1/SLC25A27/STOX1/TGFB2/EYA4/EYA1/HOXA1/COL2A1/FZD6 40

BP GO:0051651 maintenance of location in cell 47/2475 237/18870 0.00229010438897858 0.0171872334392842 0.0124840822151819 CAV1/AKAP6/SP100/FTL/DDIT3/ITGB3/FTH1/TMSB4X/TSPO/JPH4/LCK/CYBA/KDELR3/FLNA/APLNR/MYZAP/P2RY6/S100A8/S100A9/HEXB/SCIN/CXCL10/SELENON/HTR2A/SLC30A7/RYR2/LYN/SRGN/HTR2C/HK2/OS9/CXCL9/KDELR2/F2RL3/CCR5/KDELR1/RYR3/MCOLN2/JPH3/PTPRC/TMSB10/PRKCE/CXCL11/FKBP1C/ANK3/ASPM/PLCB1 47

BP GO:0007501 mesodermal cell fate specification 6/2475 12/18870 0.0023031876719639 0.0171994263463573 0.0124929386291522 HOXA11/DKK1/SFRP2/EYA2/EYA1/NODAL 6

BP GO:0016554 cytidine to uridine editing 6/2475 12/18870 0.0023031876719639 0.0171994263463573 0.0124929386291522 APOBEC3B/RBM47/APOBEC3G/APOBEC3C/APOBEC3F/APOBEC3H 6

BP GO:0034135 regulation of toll-like receptor 2 signaling pathway 6/2475 12/18870 0.0023031876719639 0.0171994263463573 0.0124929386291522 TLR1/CYBA/TREM2/TNFAIP3/F2RL1/LYN 6

BP GO:0060707 trophoblast giant cell differentiation 6/2475 12/18870 0.0023031876719639 0.0171994263463573 0.0124929386291522 SNAI1/E2F7/E2F8/LIF/NR2F2/PLK4 6

BP GO:0060706 cell differentiation involved in embryonic placenta development 9/2475 24/18870 0.00231352927183353 0.017212428436293 0.0125023827994604 SNAI1/E2F7/E2F8/ST14/CASP8/FZD5/LIF/NR2F2/PLK4 9

BP GO:0071294 cellular response to zinc ion 9/2475 24/18870 0.00231352927183353 0.017212428436293 0.0125023827994604 MT1M/TSPO/MT1A/MT2A/MT1DP/MT1E/KCNK3/MT1H/MT3 9

BP GO:1903861 positive regulation of dendrite extension 9/2475 24/18870 0.00231352927183353 0.017212428436293 0.0125023827994604 UNC13A/RIMS1/CPNE9/CXCR4/SYT1/SYT4/RASAL1/SYT2/RIMS2 9

BP GO:0032535 regulation of cellular component size 68/2475 370/18870 0.00232354098808044 0.0172655199163799 0.0125409462136754 IL7R/TMOD2/PPP1R15A/PYCARD/HIP1R/SEMA3E/TMSB4X/MSN/CCL26/USH1C/SPP1/CAPG/FGF13/VASP/NEFL/RET/NRP1/VAV3/SEMA3A/CDH4/SEMA3D/AVIL/COTL1/SLIT1/DSCAM/RTN4R/SSH3/ARHGAP18/ARPC5/SCIN/FCHSD1/VEGFA/F2RL1/L1CAM/CDC42EP5/SEMA6B/FN1/KCNN4/MAPT/SLC12A5/CSF3/BLOC1S1/PAK3/TNR/SLC12A7/PRR16/SEMA3F/NKX6-1/CAPZA1/DRAXIN/FSTL4/ELN/SPTBN2/SPTB/IQGAP3/DAAM2/PLEK/TMSB10/CARMIL2/PRKCE/TRPV2/SWAP70/AQP1/HCLS1/ISLR2/WNT7B/MT3/PFN1 68

BP GO:0035115 embryonic forelimb morphogenesis 11/2475 33/18870 0.00234083810795051 0.0173511012347344 0.0126031088775035 SHOX2/HOXA11/HOXD9/HOXA9/TBX5/RUNX2/OSR1/TWIST1/RDH10/OSR2/EN1 11

BP GO:0038094 Fc-gamma receptor signaling pathway 11/2475 33/18870 0.00234083810795051 0.0173511012347344 0.0126031088775035 LCK/CD247/FCGR1A/VAV3/FCGR2B/LYN/FCER1G/PTPRC/PRKCE/FCGR3A/MYO1G 11

BP GO:0046942 carboxylic acid transport 65/2475 351/18870 0.00237036127209709 0.0175482725988544 0.0127463258489172 CES1/SLC7A4/SLC27A3/SLC11A1/PLA2G2A/SLC6A17/IL1A/SLC7A3/SLC32A1/SLC16A10/SLC16A4/SLC10A4/SLC47A1/SLC17A8/SLC22A6/SLC6A15/ACE/CROT/ANXA1/ABCC8/EMB/THBS1/GABBR1/SLC7A7/ATP8B1/SLC7A14/STXBP1/PLIN2/SLC17A7/PLA2G5/SLC35D2/FABP7/FOLR1/ABCB4/SLC43A3/SYT4/PTGES/SLC1A6/SLCO1A2/SLC16A3/NOS2/SLC17A6/GRM2/CD36/SLC26A2/BDKRB2/HRH3/LYPLA1/FABP5/RBP1/GRM1/SLC16A11/ERFE/ITGB1/TRH/ADORA1/AQP9/PPARG/ABCC3/PLA2G4A/RGS4/NMUR2/SLC6A7/UCP2/SLC1A5 65

BP GO:0071456 cellular response to hypoxia 32/2475 147/18870 0.00245485273684613 0.0181513988079115 0.0131844113154699 MDM2/PLK3/STC1/LMNA/HMOX1/TREM2/MIR210/KCNMB1/AK4/SCN2A/VEGFA/SLC8A3/MDM4/HILPDA/CHCHD2/AJUBA/KCNK3/SLC2A4/CCNA2/TWIST1/PDK1/BRIP1/ACAA2/PGK1/VASN/PRKCE/PPARG/STOX1/AQP1/SLC29A1/MGARP/MT3 32

BP GO:0002228 natural killer cell mediated immunity 20/2475 79/18870 0.00248572082681979 0.0183570330187282 0.0133337753421562 KLRC2/IL18/KLRC3/NKG7/KLRC4/HAVCR2/GZMB/MICA/RAB27A/KLRC4-KLRK1/NECTIN2/HLA-B/LILRB1/HLA-A/ULBP2/CD2/ULBP3/FCGR3A/PIK3R6/HLA-F 20

BP GO:0002643 regulation of tolerance induction 8/2475 20/18870 0.00251643492846209 0.0184702632157535 0.0134160209865119 FOXJ1/HAVCR2/CD274/CD3E/IDO1/LILRB2/HLA-B/IL2RA 8

BP GO:0042474 middle ear morphogenesis 8/2475 20/18870 0.00251643492846209 0.0184702632157535 0.0134160209865119 HOXA2/NKX3-2/GSC/OSR1/TBX1/OSR2/EYA1/NOG 8

BP GO:0043011 myeloid dendritic cell differentiation 8/2475 20/18870 0.00251643492846209 0.0184702632157535 0.0134160209865119 BATF/SPI1/RELB/BATF2/BATF3/DHRS2/LTBR/CAMK4 8

BP GO:1903978 regulation of microglial cell activation 8/2475 20/18870 0.00251643492846209 0.0184702632157535 0.0134160209865119 TREM2/CTSC/TAFA3/SPHK1/LRRK2/CST7/PTPRC/IL6 8

BP GO:1904862 inhibitory synapse assembly 8/2475 20/18870 0.00251643492846209 0.0184702632157535 0.0134160209865119 LGI2/FGF13/GABRB3/CLSTN2/GABRA1/CBLN1/GABRB2/GABRG2 8

BP GO:0032635 interleukin-6 production 37/2475 177/18870 0.0025232637041813 0.0184752137559811 0.0134196168503506 PYCARD/TLR1/IL1A/CYBA/TREM2/ELF4/FOXJ1/CD74/HAVCR2/LILRA5/ARID5A/CLEC7A/IL17RC/SPON2/RAB7B/TNFAIP3/TLR2/F2RL1/SCIMP/IFIH1/TLR8/HGF/CEBPB/LILRB2/NOS2/CD36/TYROBP/TWIST1/HLA-B/LILRA2/IL1RAP/UNC93B1/IL6/LBP/ZC3H12A/MYD88/EOLA1 37

BP GO:0032675 regulation of interleukin-6 production 37/2475 177/18870 0.0025232637041813 0.0184752137559811 0.0134196168503506 PYCARD/TLR1/IL1A/CYBA/TREM2/ELF4/FOXJ1/CD74/HAVCR2/LILRA5/ARID5A/CLEC7A/IL17RC/SPON2/RAB7B/TNFAIP3/TLR2/F2RL1/SCIMP/IFIH1/TLR8/HGF/CEBPB/LILRB2/NOS2/CD36/TYROBP/TWIST1/HLA-B/LILRA2/IL1RAP/UNC93B1/IL6/LBP/ZC3H12A/MYD88/EOLA1 37

BP GO:0040036 regulation of fibroblast growth factor receptor signaling pathway 12/2475 38/18870 0.00252755940583433 0.0184841250580138 0.0134260896446845 WNT4/NGFR/APLN/SPRY2/THBS1/GPC1/SPRY4/FAM20C/RUNX2/FGFBP3/SPRY1/NOG 12

BP GO:0015849 organic acid transport 65/2475 352/18870 0.002543314956313 0.0185458846125393 0.013470949182957 CES1/SLC7A4/SLC27A3/SLC11A1/PLA2G2A/SLC6A17/IL1A/SLC7A3/SLC32A1/SLC16A10/SLC16A4/SLC10A4/SLC47A1/SLC17A8/SLC22A6/SLC6A15/ACE/CROT/ANXA1/ABCC8/EMB/THBS1/GABBR1/SLC7A7/ATP8B1/SLC7A14/STXBP1/PLIN2/SLC17A7/PLA2G5/SLC35D2/FABP7/FOLR1/ABCB4/SLC43A3/SYT4/PTGES/SLC1A6/SLCO1A2/SLC16A3/NOS2/SLC17A6/GRM2/CD36/SLC26A2/BDKRB2/HRH3/LYPLA1/FABP5/RBP1/GRM1/SLC16A11/ERFE/ITGB1/TRH/ADORA1/AQP9/PPARG/ABCC3/PLA2G4A/RGS4/NMUR2/SLC6A7/UCP2/SLC1A5 65

BP GO:0072676 lymphocyte migration 28/2475 124/18870 0.00254388189713883 0.0185458846125393 0.013470949182957 ASCL2/ITGB3/PYCARD/CXCL13/MSN/CCL26/CCL20/ICAM1/RET/RIPK3/CCL2/ECM1/KLRC4-KLRK1/ITGA4/HSD3B7/CXCL10/CCL5/F11R/CCL18/SPN/CXCL11/PIK3CG/FADD/ITGAL/TNFRSF14/MYO1G/SAA1/GPR183 28

BP GO:0010273 detoxification of copper ion 7/2475 16/18870 0.00256380483484471 0.0185458846125393 0.013470949182957 MT1M/MT1A/MT2A/MT1DP/MT1E/MT1H/MT3 7

BP GO:0023035 CD40 signaling pathway 7/2475 16/18870 0.00256380483484471 0.0185458846125393 0.013470949182957 ITGA5/FANCD2/TREM2/CD40/TNFAIP3/ITGB1/FANCA 7

BP GO:0046135 pyrimidine nucleoside catabolic process 7/2475 16/18870 0.00256380483484471 0.0185458846125393 0.013470949182957 APOBEC3B/DPYD/APOBEC3G/APOBEC3C/UPP1/APOBEC3F/APOBEC3H 7

BP GO:0048172 regulation of short-term neuronal synaptic plasticity 7/2475 16/18870 0.00256380483484471 0.0185458846125393 0.013470949182957 SLC4A10/RAB3A/SHISA7/SLC8A2/SYT4/SYP/SYNGR1 7

BP GO:0097091 synaptic vesicle clustering 7/2475 16/18870 0.00256380483484471 0.0185458846125393 0.013470949182957 SYN1/RAB3A/BRSK2/SYN2/BSN/SYN3/PCLO 7

BP GO:1990169 stress response to copper ion 7/2475 16/18870 0.00256380483484471 0.0185458846125393 0.013470949182957 MT1M/MT1A/MT2A/MT1DP/MT1E/MT1H/MT3 7

BP GO:2000696 regulation of epithelial cell differentiation involved in kidney development 7/2475 16/18870 0.00256380483484471 0.0185458846125393 0.013470949182957 WWTR1/PROM1/STAT1/MMP9/OSR1/LIF/GDNF 7

BP GO:0060079 excitatory postsynaptic potential 25/2475 107/18870 0.00259082622673157 0.0187187974311628 0.013596545769015 GLRA3/CHRNA9/STX1A/STX1B/CHRNB2/RIMS1/CHRNA1/CUX2/SHANK1/NRXN1/SLC8A3/GRIN2A/SLC8A2/GRIN1/LRRK2/CBLN1/CELF4/P2RX6/ADORA1/MAPK8IP2/CHRNA4/RGS4/SLC29A1/GRIN2B/RIMS2 25

BP GO:0007052 mitotic spindle organization 29/2475 130/18870 0.00261162050211015 0.0188237328867579 0.0136727664626807 STIL/NEK2/KIFC1/PLK3/KIF4A/MYBL2/PSRC1/CHEK2/KIF23/FLNA/CENPH/TACC3/CCNB1/NEK6/ANKRD53/DLGAP5/BIRC5/TTK/NUF2/AURKA/AURKB/PLK1/CENPE/CDCA8/NDC80/WDR62/TPX2/CHMP4BP1/CDC20 29

BP GO:0045621 positive regulation of lymphocyte differentiation 29/2475 130/18870 0.00261162050211015 0.0188237328867579 0.0136727664626807 IL7R/MDK/IL18/LEF1/NFKBIZ/SPI1/ANXA1/ACTL6B/TESPA1/CD74/SOCS1/RHOH/RUNX1/GLI3/TOX/HLA-DRB1/MMP14/IL2RG/SASH3/DUSP10/BTN2A2/RUNX3/LILRB2/HLA-DRA/PTPRC/WNT10B/IL2RA/PIK3R6/IL4I1 29

BP GO:0014002 astrocyte development 13/2475 43/18870 0.00262551875287748 0.0188560081055254 0.0136962098217314 TSPAN2/ROR2/TREM2/LAMC3/S100A8/ROR1/S100A9/C5AR1/MAPT/VIM/IL6/C1QA/CDK6 13

BP GO:0033003 regulation of mast cell activation 13/2475 43/18870 0.00262551875287748 0.0188560081055254 0.0136962098217314 VAMP8/STXBP1/IL13RA2/ADGRE2/CNR1/CD300LF/PLSCR1/FES/LYN/CD300A/NECTIN2/RAC2/MILR1 13

BP GO:0090175 regulation of establishment of planar polarity 15/2475 53/18870 0.00262866069026062 0.0188560081055254 0.0136962098217314 RSPO3/CELSR3/FZD7/PLEKHA4/DKK1/SFRP2/JHY/CTHRC1/FZD2/NKD1/ARHGEF19/SPEF1/FZD1/CELSR1/FZD6 15

BP GO:1904645 response to amyloid-beta 15/2475 53/18870 0.00262866069026062 0.0188560081055254 0.0136962098217314 NGFR/CACNA1B/MMP12/TREM2/ICAM1/MMP13/MMP2/MMP9/ITGA4/FCGR2B/GRM5/FPR2/CD36/CASP4/RAMP3 15

BP GO:0002224 toll-like receptor signaling pathway 19/2475 74/18870 0.00265085417454199 0.018966999731351 0.013776829462286 LY96/IRF7/TLR1/NFKBIZ/FOSL1/CD40/BIRC3/CD300LF/TLR2/SCIMP/TLR8/CD36/CD300A/UNC93B1/PRKCE/IRAK2/IRF1/MYD88/CTSS 19

BP GO:0099601 regulation of neurotransmitter receptor activity 14/2475 48/18870 0.0026536108884635 0.018966999731351 0.013776829462286 CACNG2/PRRT1/CRH/CCL2/SHANK1/SHISA7/DLGAP3/CNIH3/DLGAP2/DLGAP1/ARC/MAPK8IP2/CACNG5/CACNG3 14

BP GO:2001222 regulation of neuron migration 14/2475 48/18870 0.0026536108884635 0.018966999731351 0.013776829462286 MDK/KIF26A/NTNG2/SEMA3A/NRG3/COL3A1/FLNA/SCRT1/CAMK2A/ULK4/NKX6-1/PHACTR1/WDR62/NTNG1 14

BP GO:1901654 response to ketone 42/2475 208/18870 0.00268736838488011 0.0191854456395008 0.0139354993661159 CAV1/F5/MSN/FOSL1/NCF1/TSPO/CA9/SPP1/ACE/HOXA11/THBS1/NEFL/CLDN1/AHR/CYP1B1/P2RY6/TYMS/DSG2/OXTR/IGFBP7/HOXA9/PTGER4/PSPH/TLR2/SCNN1B/ABCB4/GJB2/TNC/SOX10/PCNA/HOXA10/FBP1/HPCA/POSTN/CDK4/PTPRC/PRKCE/HOXB13/TGFB2/AQP1/MAOB/HOXD13 42

BP GO:0008306 associative learning 21/2475 85/18870 0.00270857602843777 0.019313884174276 0.0140287917062354 NPTX2/ABCC8/CHRNB2/GABRA5/CSMD1/NEUROD2/ASIC1/SHANK2/SHANK1/CLSTN2/GRIN2A/HRH1/TAFA2/GRIN1/SNAP25/TNR/ELAVL4/TBR1/ITGB1/TUBA1A/NOG 21

BP GO:0031348 negative regulation of defense response 60/2475 321/18870 0.00273655749563605 0.0194902624007104 0.014156905418518 MDK/MIR221/HAMP/MMP12/TREM2/CGAS/ELF4/SLC39A8/TRIM21/LILRA4/HAVCR2/AHR/MICA/TRIM38/OAS1/RAB7B/TNFRSF1A/TAFA3/PTGER4/SLAMF8/HLA-DRB1/TNFAIP3/MICB/PBK/ZFP36/IFI16/F2RL1/NMI/GPX1/FCGR2B/VSIG4/HGF/DUSP10/ACP5/LYN/FPR2/IL10RA/OAS3/SOCS3/NLRC5/SPN/SERPING1/ZDHHC12/ISG15/HLA-B/LILRB1/TNFAIP6/AURKB/CST7/HLA-A/FGL2/LRFN5/ALOX5/PTPRC/ADORA1/PPARG/IL2RA/SAA1/SIGLEC10/HLA-F 60

BP GO:0002377 immunoglobulin production 40/2475 196/18870 0.00274232272627426 0.0195081820480458 0.0141699214953283 IGLV1-44/BATF/IGLV2-11/NFKBIZ/IGKV3-20/TNFRSF4/IGLV2-23/IGLV3-21/IGLV6-57/IGLV3-1/IGLV2-14/CGAS/CD40/IGLV2-8/POLQ/TFRC/HPX/VPREB3/IGLV8-61/IL13RA2/IGKV1-5/IGKV3-15/GAPT/IGLV1-47/IGKV2-30/IGLV5-45/FCGR2B/SASH3/IGLV3-25/IGKV4-1/EXO1/IGLV7-46/IGLV3-10/PTPRC/IL6/IGLV3-19/SWAP70/IGLV1-40/IGKV1-9/CLCF1 40

BP GO:0003209 cardiac atrium morphogenesis 10/2475 29/18870 0.00276151553478857 0.01957513491248 0.0142185532351072 SHOX2/NKX2-5/CCN1/BMP2/ENG/TBX5/GATA4/TGFB2/NOG/DLL4 10

BP GO:0036037 CD8-positive, alpha-beta T cell activation 10/2475 29/18870 0.00276151553478857 0.01957513491248 0.0142185532351072 SOCS1/TNFSF8/RUNX1/CD274/TOX/RUNX3/LILRB1/HLA-A/IRF1/HFE 10

BP GO:0072207 metanephric epithelium development 10/2475 29/18870 0.00276151553478857 0.01957513491248 0.0142185532351072 WNT4/WWTR1/SLC22A6/STAT1/SOX8/OSR1/LIF/CXCR2/AQP1/WNT7B 10

BP GO:0045787 positive regulation of cell cycle 62/2475 334/18870 0.002778239101374 0.01962417360547 0.0142541728755325 STIL/MDM2/MAD2L1/WNT4/KIF14/IL1A/FOSL1/MIR221/CENPV/E2F7/ECT2/CDCA5/ANXA1/PSRC1/RRM2/KIF23/UBE2C/TRIM21/EGFR/E2F8/MAP3K20/CCNB1/NR2E1/FAM83D/DTL/DLGAP5/BIRC5/SMPD3/PLSCR1/ESPL1/SPHK1/DYNLT3/IGF2/NPM2/AURKA/GLI1/BRCA2/CDC25C/BUB1/AURKB/NCAPG/SMC4/RAD51AP1/DBF4/CDC6/PLK4/CDCA8/NDC80/CDK4/CDK1/WNT10B/NCAPH/PRKCE/CDC25A/STOX1/DYRK3/TGFB2/EZH2/SPAG5/PLCB1/CHEK1/CDC20 62

BP GO:0071214 cellular response to abiotic stimulus 62/2475 334/18870 0.002778239101374 0.01962417360547 0.0142541728755325 TIMP1/PDE2A/MDM2/RGR/ITGB3/PYCARD/RELB/TSPO/FBXO4/ITGA2/FAS/ECT2/CD40/ST20/CHEK2/MMP2/MMP1/NEUROD2/ASIC1/MAP3K20/MMP9/MFAP4/ENG/NET1/TNFRSF1A/PTGER4/SCN2A/CASP1/CASP8/BRCA1/PBK/IFI16/SFRP2/SCNN1B/TLR8/COL1A1/ASIC2/F11R/SLC2A4/YBX3/RAD51/AQP5/ADSS1/PCNA/BRCA2/BDKRB2/AURKB/CASP3/LTBR/FBP1/RAD51AP1/CRY2/SNAI2/GRM1/HPCA/GADD45A/CDC25A/FADD/IRF1/MYD88/AQP1/CHEK1 62

BP GO:0104004 cellular response to environmental stimulus 62/2475 334/18870 0.002778239101374 0.01962417360547 0.0142541728755325 TIMP1/PDE2A/MDM2/RGR/ITGB3/PYCARD/RELB/TSPO/FBXO4/ITGA2/FAS/ECT2/CD40/ST20/CHEK2/MMP2/MMP1/NEUROD2/ASIC1/MAP3K20/MMP9/MFAP4/ENG/NET1/TNFRSF1A/PTGER4/SCN2A/CASP1/CASP8/BRCA1/PBK/IFI16/SFRP2/SCNN1B/TLR8/COL1A1/ASIC2/F11R/SLC2A4/YBX3/RAD51/AQP5/ADSS1/PCNA/BRCA2/BDKRB2/AURKB/CASP3/LTBR/FBP1/RAD51AP1/CRY2/SNAI2/GRM1/HPCA/GADD45A/CDC25A/FADD/IRF1/MYD88/AQP1/CHEK1 62

BP GO:0050918 positive chemotaxis 18/2475 69/18870 0.00281200339665526 0.0198393283120073 0.0144104521891802 CXCL8/ITGA2/NRP1/F3/LGALS3/ANGPT2/MET/GPNMB/ANGPT1/VEGFA/CXCL10/F2RL1/CCL5/HGF/FPR2/S100A4/HMGB2/GDNF 18

BP GO:0090596 sensory organ morphogenesis 53/2475 277/18870 0.00282416607541339 0.0199017524844859 0.0144557944779314 COL8A1/CHRNA9/HCN1/COL8A2/MFAP2/EFEMP1/USH1C/SPRY2/PROM1/HOXA2/CABP4/MFAP5/LCTL/ATP8A2/NKX3-2/WNT16/MEGF11/DSCAM/SOX8/JAG1/DLX5/GLI3/VEGFA/GSC/FRZB/VSX1/FZD5/OLFM3/OSR1/CTHRC1/AQP5/FZD2/NKD1/TWIST1/OTX1/CRB2/STRA6/EPHA2/COL5A2/TBX1/ALDH1A3/ZIC1/HOXC13/COL5A1/STOX1/OSR2/EYA1/HOXA1/NOG/COL2A1/AQP1/MEIS1/FZD6 53

BP GO:0070167 regulation of biomineral tissue development 24/2475 102/18870 0.00282927034120378 0.0199143483336313 0.0144649435720457 WNT4/MGP/CCN1/BMP2/ECM1/NELL1/ENPP1/FAM20C/SRGN/OSR1/CYP27B1/TENT5A/CEBPB/LTF/ISG15/TWIST1/ADGRV1/SGMS2/ALOX5/ASPN/WNT10B/OSR2/PTN/CCR1 24

BP GO:0001578 microtubule bundle formation 28/2475 125/18870 0.00287652144608341 0.0202232257169611 0.0146892990892685 SPAG1/KIF20A/CFAP100/DRC1/DNAI3/PSRC1/FOXJ1/LRRC61/RSPH1/DNAI4/CFAP73/CFAP157/ZMYND10/JHY/LRGUK/CAPN6/FES/CCDC40/TTC12/CFAP43/DRC7/PLK1/SPEF1/RSPH9/TPPP3/IQCG/RSPH4A/NEURL1 28

BP GO:0045824 negative regulation of innate immune response 22/2475 91/18870 0.00290840667589843 0.0203996187874932 0.0148174235836783 MMP12/CGAS/TRIM21/HAVCR2/MICA/OAS1/SLAMF8/TNFAIP3/IFI16/NMI/VSIG4/DUSP10/OAS3/NLRC5/SERPING1/ISG15/HLA-B/LILRB1/AURKB/HLA-A/PPARG/HLA-F 22

BP GO:0060021 roof of mouth development 22/2475 91/18870 0.00290840667589843 0.0203996187874932 0.0148174235836783 HAND2/MEOX2/SNAI1/LEF1/LOXL3/LRRC32/GABRB3/DLX5/GLI3/MMP25/OSR1/WFIKKN2/FZD2/TWIST1/MSC/SNAI2/TBX1/FZD1/TGFB2/OSR2/COL2A1/BNC2 22

BP GO:0002820 negative regulation of adaptive immune response 17/2475 64/18870 0.00296361413778096 0.0207625895953756 0.0150810702853608 ASCL2/IL7R/LOXL3/FOXJ1/HAVCR2/AHR/SAMSN1/JAK3/FCGR2B/SPN/LILRB1/PTPRC/ZC3H12A/TNFRSF14/HFE/IL4I1/HLA-F 17

BP GO:0006979 response to oxidative stress 72/2475 400/18870 0.00305015945461925 0.0212858573647608 0.0154611499460367 MDM2/PXDNL/H19/IL1A/FOSL1/NCF1/PLK3/FANCD2/IPCEF1/ECT2/SOD2/ANXA1/HMOX1/VRK2/PAWR/GCH1/MMP2/EGFR/RIPK3/WNT16/CYP1B1/STAT1/AREG/MMP9/MET/NET1/TNFAIP3/MMP14/SMPD3/MICB/PTGS1/GPX1/SELENON/SPHK1/HGF/COL1A1/HP/CHCHD2/DHRS2/GJB2/MAPT/HBA1/SOD3/LRRK2/GPX7/PDLIM1/PPIA/ADCYAP1R1/CD36/PCNA/PDGFD/SLC25A24/FBLN5/RCAN1/CASP3/SDC1/ADAM9/ALOX5/PRDX4/DGKK/CDK1/CHRNA4/IL6/CRYGD/STOX1/ZC3H12A/GPX8/AQP1/RBPMS/EZH2/UCP2/MT3 72

BP GO:0034110 regulation of homotypic cell-cell adhesion 11/2475 34/18870 0.00305603082085673 0.0212858573647608 0.0154611499460367 MMRN1/C1QTNF1/CCL5/PDPN/LYN/EMILIN2/F11R/EMILIN1/IL6/PLAUR/ANK3 11

BP GO:0035456 response to interferon-beta 11/2475 34/18870 0.00305603082085673 0.0212858573647608 0.0154611499460367 STAT1/OAS1/IFITM3/PLSCR1/IFI16/IFITM2/XAF1/CAMK2A/TRIM6/IRF1/BST2 11

BP GO:0048333 mesodermal cell differentiation 11/2475 34/18870 0.00305603082085673 0.0212858573647608 0.0154611499460367 ITGB3/ITGA2/HOXA11/ITGA3/ITGB4/DKK1/SFRP2/EYA2/ITGB1/EYA1/NODAL 11

BP GO:0090022 regulation of neutrophil chemotaxis 11/2475 34/18870 0.00305603082085673 0.0212858573647608 0.0154611499460367 MDK/CXCL8/CD74/C5AR1/BST1/THBS4/TNFAIP6/RAC2/CXCR2/DPP4/LBP 11

BP GO:0050768 negative regulation of neurogenesis 32/2475 149/18870 0.00306738406001671 0.0213401783271615 0.0155006064044023 ASCL2/MCF2/SEMA3E/TSPO/SPP1/FGF13/ABCC8/TREM2/DLL3/NRP1/SEMA3A/SEMA3D/SLIT1/DLX2/RTN4R/NR2E1/BRINP1/SEMA6B/DUSP10/SYT4/SOX10/TNR/SEMA3F/NKX6-1/DRAXIN/FSTL4/DAAM2/B2M/IL6/NOG/PTN/MT3 32

BP GO:0071772 response to BMP 37/2475 179/18870 0.00308426246121173 0.0213814652515311 0.0155305954867095 LEF1/ITGA3/SFRP4/FSTL1/MIR210/TWSG1/CCN1/BMP2/SPINT1/DLX5/ENG/FOXD1/SMPD3/DKK1/SFRP2/RUNX2/FSTL3/ETV2/PCSK6/FSTL5/TMEM100/VWC2L/CHRDL2/TNFAIP6/CRB2/FSTL4/FST/GATA4/GREM2/FZD1/SMAD9/HTRA3/PPARG/ADAMTS7/HFE/NOG/COL2A1 37

BP GO:0071773 cellular response to BMP stimulus 37/2475 179/18870 0.00308426246121173 0.0213814652515311 0.0155305954867095 LEF1/ITGA3/SFRP4/FSTL1/MIR210/TWSG1/CCN1/BMP2/SPINT1/DLX5/ENG/FOXD1/SMPD3/DKK1/SFRP2/RUNX2/FSTL3/ETV2/PCSK6/FSTL5/TMEM100/VWC2L/CHRDL2/TNFAIP6/CRB2/FSTL4/FST/GATA4/GREM2/FZD1/SMAD9/HTRA3/PPARG/ADAMTS7/HFE/NOG/COL2A1 37

BP GO:0007204 positive regulation of cytosolic calcium ion concentration 36/2475 173/18870 0.00309603619262315 0.0213814652515311 0.0155305954867095 CAV1/CHRNA9/CXCL13/JPH4/GNG3/C1QTNF1/CCKBR/FPR3/S1PR3/OXTR/PTGER4/C5AR1/F2RL1/HTR2A/RYR2/CXCR4/CD52/FPR2/GRIN1/ADCYAP1R1/CD36/CCR5/P2RY1/BDKRB2/CXCR2/ADRA1D/CCRL2/GLP1R/JPH3/PIK3CG/SWAP70/SAA1/NMUR2/ADM/FPR1/CCR1 36

BP GO:0001755 neural crest cell migration 16/2475 59/18870 0.00309824696349635 0.0213814652515311 0.0155305954867095 EFNB1/HAND2/SEMA3E/RET/NRP1/SEMA3A/SEMA3D/SOX8/FOLR1/SEMA6B/FN1/SOX10/TWIST1/SEMA3F/TBX1/GDNF 16

BP GO:0032608 interferon-beta production 16/2475 59/18870 0.00309824696349635 0.0213814652515311 0.0155305954867095 IRF7/PYCARD/RELB/OAS2/TRIM38/OAS1/TLR2/NMI/IFIH1/TLR8/OAS3/ISG15/LILRB1/RNF135/HMGB2/IRF1 16

BP GO:0032648 regulation of interferon-beta production 16/2475 59/18870 0.00309824696349635 0.0213814652515311 0.0155305954867095 IRF7/PYCARD/RELB/OAS2/TRIM38/OAS1/TLR2/NMI/IFIH1/TLR8/OAS3/ISG15/LILRB1/RNF135/HMGB2/IRF1 16

BP GO:0051480 regulation of cytosolic calcium ion concentration 16/2475 59/18870 0.00309824696349635 0.0213814652515311 0.0155305954867095 TSPOAP1/CAV1/ATP2B3/CNR1/ERC2/CAV2/RYR2/GRM5/SLC8A2/GPR3/SV2B/P2RY1/GRM1/TUNAR/ADORA1/ATP2B2 16

BP GO:0001707 mesoderm formation 19/2475 75/18870 0.00312209941810637 0.0215213374354887 0.0156321927478575 ITGB3/SNAI1/LEF1/ITGA2/HOXA11/ITGA3/TWSG1/ITGB4/DKK1/SFRP2/ETV2/EYA2/CRB2/EPHA2/ITGB1/TBX19/EYA1/NOG/NODAL 19

BP GO:0050678 regulation of epithelial cell proliferation 73/2475 407/18870 0.00314526929639943 0.0216561890545667 0.0157301432822101 CAV1/MDK/NGFR/APLN/ITGB3/HOXA5/NKX2-5/CCL26/NEAT1/HMOX1/MMP12/THBS1/CLDN1/NRP1/FZD7/F3/EGFR/APLNR/CCL2/STAT1/HSPG2/AREG/JAG1/ECM1/DLX5/ITGA4/ATOH8/TNFAIP3/VEGFA/C5AR1/ZFP36/GPX1/CAV2/SFRP2/VIP/IGF2/HAS2/DUSP10/LAMC1/RUNX2/MIR27B/OSR1/RUNX3/TACSTD2/RGN/ANG/TWIST1/GLI1/LAMB1/BRCA2/THBS4/NR2F2/SFN/EFNB2/SNAI2/IQGAP3/ALOX5/TBX1/CDK4/HMGB2/WNT10B/CNMD/B2M/PPARG/CD109/TGFB2/OSR2/EYA1/NOG/PTN/DLL4/CDK6/NODAL 73

BP GO:0001818 negative regulation of cytokine production 69/2475 381/18870 0.00315249251965886 0.0216810596655576 0.0157482082461526 DDIT3/PYCARD/SLC11A1/TMSB4X/LEF1/RELB/MIR221/TSPO/SIGLEC1/LRRC32/ANXA1/SERPINB1/THBS1/RNF128/TREM2/ELF4/BCL3/FOXJ1/LILRA4/MIR210/HAVCR2/CD33/TWSG1/LILRA5/GBP1/SSC5D/OAS1/CD274/PTGER4/GPNMB/HLA-DRB1/TNFAIP3/SLC2A10/ANGPT1/JAK3/ZFP36/CARD16/IDO1/F2RL1/NMI/PDCD1LG2/FCGR2B/VSIG4/TLR8/HGF/ACP5/FN1/IL1R2/SRGN/OAS3/MIR27B/BTN2A2/XAF1/LTF/LAPTM5/TYROBP/TWIST1/LILRB1/CASP3/EPHA2/PTPRC/IL6/LBP/TGFB2/ZC3H12A/HFE/EZH2/HLA-F/BST2 69

BP GO:0033002 muscle cell proliferation 48/2475 247/18870 0.00316081337666511 0.0217134136310038 0.0157717088034729 MDM2/EFEMP2/DDIT3/APLN/ITGB3/IL18/NKX2-5/MIR221/ITGA2/SOD2/HMOX1/THBS1/MMP2/IGFBP3/IGFBP5/RUNX1/P2RY6/STAT1/BMP2/CCN4/MMP9/SIX5/TNFAIP3/SMPD3/ANGPT1/DSN1/SELENON/CAV2/CCL5/PDE1A/TBX5/VIP/HGF/FES/MIR27B/ANG/GLI1/PDGFD/ELN/NDC80/CDK1/IL6/PPARG/TGFB2/VIPR2/MYD88/NOG/TP73 48

BP GO:0009164 nucleoside catabolic process 9/2475 25/18870 0.00319911607620426 0.0218990164036364 0.0159065228374532 APOBEC3B/DPYD/APOBEC3G/ADA2/APOBEC3C/UPP1/GDA/APOBEC3F/APOBEC3H 9

BP GO:0046629 gamma-delta T cell activation 9/2475 25/18870 0.00319911607620426 0.0218990164036364 0.0159065228374532 TRDC/LEF1/CD247/MICA/CD3E/MICB/LILRB1/TCF7/PTPRC 9

BP GO:2000311 regulation of AMPA receptor activity 9/2475 25/18870 0.00319911607620426 0.0218990164036364 0.0159065228374532 CACNG2/PRRT1/SHANK1/SHISA7/CNIH3/ARC/MAPK8IP2/CACNG5/CACNG3 9

BP GO:0043303 mast cell degranulation 15/2475 54/18870 0.00320606852411666 0.0218990164036364 0.0159065228374532 CHGA/CPLX2/VAMP8/STXBP1/IL13RA2/S100A13/ADGRE2/FES/LYN/CD300A/RAC2/LAT2/MILR1/PIK3CG/GRP 15

BP GO:0070169 positive regulation of biomineral tissue development 15/2475 54/18870 0.00320606852411666 0.0218990164036364 0.0159065228374532 WNT4/CCN1/BMP2/NELL1/FAM20C/OSR1/TENT5A/CEBPB/LTF/ISG15/ADGRV1/ALOX5/WNT10B/OSR2/PTN 15

BP GO:0001662 behavioral fear response 12/2475 39/18870 0.00322186788289043 0.0219819258737206 0.0159667447832716 HTR1A/MDK/GABRA5/NEUROD2/ASIC1/NR2E1/BRINP1/HTR2C/ASIC4/DPP4/MAPK8IP2/GRP 12

BP GO:0043588 skin development 59/2475 317/18870 0.00325551253733598 0.0221862625132409 0.0161151663088392 MACROH2A2/TRADD/FERMT1/NGFR/IL18/SNAI1/NFKBIZ/IL1A/ITGA2/COL5A3/ANXA1/TNFRSF19/ITGA3/CLDN1/ATP8A2/COL3A1/EGFR/WNT16/IGFBP5/ST14/ITGB4/ERRFI1/ALOX15B/JAG1/KRT75/MET/CLIC4/SLC2A10/ZFP36/DKK1/LGR5/COL1A1/CYP27B1/PDGFA/COL1A2/ADAMTS2/SFN/CASP3/ADAM9/PLAAT4/HOXA7/EPHA2/COL5A2/CSTA/FST/FOSL2/HOXC13/WNT10B/COL5A1/PALLD/CD109/TGFB2/SLITRK5/GAL/MYD88/UGCG/EZH2/LATS2/FZD6 59

BP GO:0006911 phagocytosis, engulfment 14/2475 49/18870 0.00327425815129664 0.022238287262879 0.0161529548950012 ITGB2/ITGA2/THBS1/TREM2/FCGR1A/F2RL1/FCGR2B/XKR7/CD36/CD300A/C3/MSR1/XKR8/MARCO 14

BP GO:0010543 regulation of platelet activation 14/2475 49/18870 0.00327425815129664 0.022238287262879 0.0161529548950012 THBD/MMRN1/C1QTNF1/FLNA/PDPN/LYN/EMILIN2/F11R/PDGFA/EMILIN1/FCER1G/PLEK/IL6/PLA2G4A 14

BP GO:0060071 Wnt signaling pathway, planar cell polarity pathway 14/2475 49/18870 0.00327425815129664 0.022238287262879 0.0161529548950012 RSPO3/CELSR3/FZD7/PLEKHA4/DKK1/SFRP2/CTHRC1/FZD2/NKD1/ARHGEF19/SPEF1/FZD1/CELSR1/FZD6 14

BP GO:0050852 T cell receptor signaling pathway 31/2475 144/18870 0.00339480895375145 0.0230309976930211 0.0167287463519384 TRAC/CD276/TRDC/NFKBIZ/BTN2A3P/LCK/TESPA1/CD247/PAWR/CD3D/LGALS3/LCP2/GBP1/CD8A/TRBC1/HLA-DRB1/CD3E/PRAM1/KCNN4/BTN2A2/HLA-DPB1/TRBC2/HLA-DQB1/CD300A/NECTIN2/LAPTM5/HLA-A/THEMIS2/FOSL2/PTPRC/ZC3H12A 31

BP GO:0007422 peripheral nervous system development 20/2475 81/18870 0.0034013168614325 0.023049104329617 0.0167418982498691 HAND2/CLDN1/LAMA2/HOXD9/RUNX1/SOX8/ITGB4/GPC1/NEFH/PMP22/ASIC2/SCN8A/RUNX3/HOXD10/SOX10/NCMAP/POU4F1/CDK1/GDNF/SERPINI1 20

BP GO:0007259 receptor signaling pathway via JAK-STAT 36/2475 174/18870 0.00342187615329618 0.0231622823273847 0.0168241059788834 CAV1/IL7R/IL18/MIR221/PARP9/HAMP/CD40/RET/HPX/BCL3/OCIAD2/SOCS1/CYP1B1/CCL2/STAT1/TNFRSF1A/CTF1/JAK3/NMI/CCL5/SOCS2/TNFRSF18/LYN/IL10RA/SOCS3/LIF/CAMK2A/CD300A/CSF2RB/OSM/IL9/GADD45A/PTPRC/IL6/HCLS1/CLCF1 36

BP GO:0010639 negative regulation of organelle organization 66/2475 363/18870 0.003471326925276 0.0234552346808585 0.017036894225393 CENPF/SPC24/MAD2L1/TMOD2/TMEM67/HIP1R/TMSB4X/TSPO/TOP2A/LMNA/CAPG/FGF13/DNAI3/KNTC1/TFRC/PIF1/AVIL/CCNB1/TOM1L1/SHANK1/SSH3/MET/KIF24/SCIN/BIRC5/BRCA1/GPX1/HASPIN/ESPL1/TTK/HGF/ZWILCH/F11R/MAPT/LRRK2/LIF/TACSTD2/NUF2/LILRB2/TRIP13/BUB1B/H3-3A/BUB1/AURKB/TMEFF2/CAPZA1/GMFG/ACAA2/SPTBN2/SPTB/PLK1/SPEF1/STMN2/CDCA8/NDC80/MAK/TMSB10/CARMIL2/PPARG/TPX2/KNL1/ZWINT/SWAP70/CHEK1/PFN1/CDC20 66

BP GO:0034764 positive regulation of transmembrane transport 43/2475 217/18870 0.00347296862612978 0.0234552346808585 0.017036894225393 AKAP5/CAV1/VMP1/AKAP6/KCNC1/RGS7/TMSB4X/STAC/CA2/SLC17A8/FGF13/TREM2/NIPSNAP2/MIR210/FLNA/KCNMB1/APLNR/P2RY6/CCL2/WNK2/CXCL10/GLRX/PIRT/RYR2/KCNN4/KCNE5/GRIN1/CXCL9/ARC/ADCYAP1R1/F2RL3/KCNC2/LRRC55/ERFE/C3/ITGB1/FXYD2/CXCL11/ANK3/RAMP3/GAL/CTSS/KCNIP2 43

BP GO:0099565 chemical synaptic transmission, postsynaptic 26/2475 115/18870 0.00348474835533886 0.0235083473319714 0.0170754730173494 GLRA3/CHRNA9/STX1A/STX1B/CHRNB2/RIMS1/CHRNA1/CUX2/SHANK1/NRXN1/SLC8A3/GRIN2A/SLC8A2/GRIN1/LRRK2/CBLN1/CELF4/INSYN2B/P2RX6/ADORA1/MAPK8IP2/CHRNA4/RGS4/SLC29A1/GRIN2B/RIMS2 26

BP GO:0050769 positive regulation of neurogenesis 47/2475 242/18870 0.00350641521309495 0.0236279651396432 0.0171623583529474 IL1RAPL1/CUL7/SHOX2/MDK/MIR221/TSPO/ACE/NEFL/NRP1/CDH4/DSCAM/HAPLN1/CUX2/BMP2/SOX8/NR2E1/SPINT1/GLI3/GSX2/VEGFA/TLR2/L1CAM/CXCR4/GRM5/FN1/LYN/DMRTA2/MAPT/LIF/CHODL/SOX10/SLITRK1/OTP/NKX6-1/HAPLN3/ITGB1/WDR62/TRPV2/IL6/NEURL1/XRCC2/ASPM/ISLR2/RASSF10/PTN/CLCF1/TP73 47

BP GO:0030278 regulation of ossification 27/2475 121/18870 0.00357805758153599 0.0240836969950024 0.0174933827711867 WNT4/MDK/MGP/CCN1/BMP2/ECM1/PTGER4/NELL1/DKK1/ENPP1/FAM20C/RUNX2/SRGN/OSR1/CYP27B1/TENT5A/LTF/ISG15/TWIST1/ADGRV1/SGMS2/GDF10/ALOX5/WNT10B/OSR2/PTN/CCR1 27

BP GO:0007413 axonal fasciculation 8/2475 21/18870 0.0036026203609193 0.0241407730434816 0.0175348404079989 CDK5R1/CRTAC1/NRP1/CNR1/FEZF2/CASP3/CNTN4/EPHA3 8

BP GO:0051315 attachment of mitotic spindle microtubules to kinetochore 8/2475 21/18870 0.0036026203609193 0.0241407730434816 0.0175348404079989 BIRC5/NUF2/AURKB/KIF2C/CDT1/CENPE/CDCA8/NDC80 8

BP GO:0106030 neuron projection fasciculation 8/2475 21/18870 0.0036026203609193 0.0241407730434816 0.0175348404079989 CDK5R1/CRTAC1/NRP1/CNR1/FEZF2/CASP3/CNTN4/EPHA3 8

BP GO:2001185 regulation of CD8-positive, alpha-beta T cell activation 8/2475 21/18870 0.0036026203609193 0.0241407730434816 0.0175348404079989 SOCS1/RUNX1/CD274/RUNX3/LILRB1/HLA-A/IRF1/HFE 8

BP GO:0002520 immune system development 40/2475 199/18870 0.00363048647442923 0.0243003799247192 0.017650772121763 LRRC17/IL7R/BATF/HAND2/LEF1/NFKBIZ/NKX2-5/CD248/HOXA3/CD40/POLQ/TFRC/RET/BCL3/FOXJ1/NKX3-2/HAVCR2/RIPK3/HOXB4/CD274/TOX/TNFAIP3/CD3E/IDO1/PDPN/LYN/EXO1/CCNB2/LILRB2/HLA-B/LTBR/BARX1/TBX1/PTPRC/HMGB2/XKR8/IL2RA/FADD/SWAP70/CLCF1 40

BP GO:0051259 protein complex oligomerization 49/2475 255/18870 0.00363870741592629 0.0243282843265272 0.0176710407035314 KCNA1/GLRA3/KCNC1/GBP5/PRMT8/HCN1/PYCARD/GSDMD/ECT2/SOD2/VASP/CD247/POLQ/CLDN1/RRM2/KCNB1/CD74/TK1/ASIC1/MLKL/KCNV1/TIFA/HLA-DRB1/KCNS2/EHD4/CRYZ/KCNA4/KCTD14/IFIH1/SYT1/ALOX5AP/GRIN1/COL1A2/AQP5/ARC/ISG15/KCNJ12/PRF1/KCNC2/RYR3/RNF135/ALDH1A3/TDO2/B2M/ZC3H12A/GRIN2B/KCTD16/TP73/SLC1A5 49

BP GO:0033077 T cell differentiation in thymus 21/2475 87/18870 0.00364908220823342 0.0243705112104933 0.0177017125328857 WNT4/IL7R/MR1/IL1A/TESPA1/CD3D/FZD7/FOXJ1/CD74/RIPK3/GLI3/TOX/CD3E/IL2RG/FZD5/SPN/PTPRC/B2M/FADD/CDK6/CAMK4 21

BP GO:0051384 response to glucocorticoid 28/2475 127/18870 0.00365363333848925 0.0243737939603216 0.0177040969840479 SSTR3/FOSL1/SSTR2/STC1/ACE/IL1RN/ANXA1/NEFL/CLDN1/CYP1B1/AREG/TYMS/IGFBP7/ZFP36/MYOD1/GJB2/GPR83/PCNA/IGFBP2/CASP3/SDC1/ADAM9/FOSL2/IL6/AQP1/ADM/MAOB/WNT7B 28

BP GO:0050820 positive regulation of coagulation 10/2475 30/18870 0.00366215302353591 0.0244035147095556 0.0177256848840578 THBD/PLAT/THBS1/F3/EMILIN2/SERPINE1/VKORC1/PLAU/CD36/EMILIN1 10

BP GO:0001961 positive regulation of cytokine-mediated signaling pathway 16/2475 60/18870 0.00372013753103161 0.0247076390888427 0.0179465880194038 IRF7/PARP9/RBM47/MMP12/TREM2/HPX/CD74/IL1R1/CASP1/CD300LF/CXCR4/NLRC5/LAPTM5/TRIM6/FADD/CASP4 16

BP GO:0002763 positive regulation of myeloid leukocyte differentiation 16/2475 60/18870 0.00372013753103161 0.0247076390888427 0.0179465880194038 LEF1/ROR2/TREM2/CD74/RUNX1/HLA-DRB1/CASP8/CD101/FES/LIF/TYROBP/EVI2B/POU4F1/FADD/HCLS1/CCR1 16

BP GO:0008347 glial cell migration 16/2475 60/18870 0.00372013753103161 0.0247076390888427 0.0179465880194038 CDK5R2/CDK5R1/MIR221/TSPO/TREM2/COL3A1/CCL2/NR2E1/GLI3/HEXB/MMP14/LAMB1/P2RY1/RRAS/TGFB2/GPR183 16

BP GO:0051656 establishment of organelle localization 82/2475 469/18870 0.0037397608726326 0.0248105240655095 0.0180213193315695 NEK2/CENPF/SPC24/KIFC1/MAD2L1/SNAP91/SGO1/KIF14/LMNA/CHGA/ECT2/CDCA5/SPRY2/KNTC1/MYO5C/NEFL/CPLX2/PSRC1/VAMP8/CENPA/STXBP1/FHOD1/SLIT1/IL13RA2/S100A13/ADGRE2/RAB27A/RAB3A/CCNB1/FAM83D/SPATA17/ANKRD53/ITGA4/BIRC5/SMPD3/DSN1/NEFH/ESPL1/BICDL1/MYO1B/LMNB1/FES/LYN/ZWILCH/MAPT/SLC2A4/SYT4/LRRK2/BLOC1S1/NUF2/CD300A/NECTIN2/KIF18A/PPFIA2/RAC2/SDC4/AURKB/KIF2C/MREG/SDC1/LAT2/MILR1/CDT1/NDE1/MYO5B/PLK1/CENPE/CDCA8/MYO1F/NDC80/ITGB1/WIPI1/PIK3CG/SPRY1/KNL1/TUBA1A/CHMP4BP1/MYO1G/TCIRG1/GRP/SPAG5/MGARP 82

BP GO:0014061 regulation of norepinephrine secretion 6/2475 13/18870 0.0038053844529661 0.0249542085165365 0.0181256856628932 STX1A/KCNB1/OXTR/P2RY1/HRH3/ADRA2B 6

BP GO:0032490 detection of molecule of bacterial origin 6/2475 13/18870 0.0038053844529661 0.0249542085165365 0.0181256856628932 LY96/TLR1/TREM2/SSC5D/TLR2/LBP 6

BP GO:0033690 positive regulation of osteoblast proliferation 6/2475 13/18870 0.0038053844529661 0.0249542085165365 0.0181256856628932 ITGB3/CCN1/BMP2/SOX8/CTHRC1/LTF 6

BP GO:0036462 TRAIL-activated apoptotic signaling pathway 6/2475 13/18870 0.0038053844529661 0.0249542085165365 0.0181256856628932 SPI1/MIR221/TNFRSF10C/CASP8/FADD/ATF3 6

BP GO:0048243 norepinephrine secretion 6/2475 13/18870 0.0038053844529661 0.0249542085165365 0.0181256856628932 STX1A/KCNB1/OXTR/P2RY1/HRH3/ADRA2B 6

BP GO:0060670 branching involved in labyrinthine layer morphogenesis 6/2475 13/18870 0.0038053844529661 0.0249542085165365 0.0181256856628932 RSPO3/ST14/SPINT1/FZD5/SOCS3/ADM 6

BP GO:0070486 leukocyte aggregation 6/2475 13/18870 0.0038053844529661 0.0249542085165365 0.0181256856628932 MSN/CD44/S100A8/S100A9/HAS2/RAC2 6

BP GO:0070493 thrombin-activated receptor signaling pathway 6/2475 13/18870 0.0038053844529661 0.0249542085165365 0.0181256856628932 IQGAP2/MET/F2RL1/F2RL3/F2RL2/PLEK 6

BP GO:0072683 T cell extravasation 6/2475 13/18870 0.0038053844529661 0.0249542085165365 0.0181256856628932 ICAM1/RIPK3/CCL2/F11R/FADD/ITGAL 6

BP GO:0075509 endocytosis involved in viral entry into host cell 6/2475 13/18870 0.0038053844529661 0.0249542085165365 0.0181256856628932 CAV1/SIGLEC1/CAV2/CTSL/DPP4/JPT2 6

BP GO:2001234 negative regulation of apoptotic signaling pathway 47/2475 243/18870 0.00380713774169677 0.0249542085165365 0.0181256856628932 MDM2/WNT4/DDIAS/SNAI1/IL1A/MIR221/IFI6/LMNA/SOD2/HMOX1/THBS1/PAK5/ICAM1/NRP1/CD74/MIR210/CD44/WNT16/LGALS3/HSPB1/MMP9/FAIM2/TNFAIP3/BRCA1/GPX1/SFRP2/MUC1/HGF/EYA2/LRRK2/YBX3/SERPINE1/PPIA/TMBIM1/BDKRB2/ACAA2/SNAI2/GATA4/HMGB2/MAPK8IP2/FZD1/GDNF/PLAUR/EYA4/EYA1/NOG/COL2A1 47

BP GO:0014812 muscle cell migration 25/2475 110/18870 0.00383223584520342 0.0250913238981476 0.0182252804989701 MDM2/MDK/DDIT3/ITGB3/MIR221/S100A11/PLAT/ITGA2/ACE/ANXA1/NRP1/IGFBP3/IGFBP5/CYP1B1/P2RY6/CCN4/NET1/CCL5/HAS2/SERPINE1/PDGFA/PLAU/THBS4/PDGFD/POSTN 25

BP GO:0033623 regulation of integrin activation 7/2475 17/18870 0.00386935315190718 0.0252517351348377 0.0183417964626447 FERMT1/KIF14/CXCL13/PTGER4/FBLIM1/RAP1B/PLEK 7

BP GO:0034134 toll-like receptor 2 signaling pathway 7/2475 17/18870 0.00386935315190718 0.0252517351348377 0.0183417964626447 TLR1/CYBA/TREM2/TNFAIP3/TLR2/F2RL1/LYN 7

BP GO:1901163 regulation of trophoblast cell migration 7/2475 17/18870 0.00386935315190718 0.0252517351348377 0.0183417964626447 TIMP1/ITGB3/ARHGDIB/ACVR1C/VEGFA/SYDE1/NODAL 7

BP GO:0015872 dopamine transport 15/2475 55/18870 0.00388365735363631 0.0253175665051383 0.0183896135963372 GABBR1/CHRNB2/CNR1/PRKCB/SNCG/SYT7/HTR2A/SYT1/SYT4/GRM2/SYT5/CHRNA4/GDNF/SYT2/SYT13 15

BP GO:0032228 regulation of synaptic transmission, GABAergic 11/2475 35/18870 0.00393668177570784 0.0255522566284864 0.0185600826136644 CA2/STXBP1/CNR1/OXTR/PHF24/NALCN/ZDHHC12/SYN3/ADORA1/PRKCE/CA7 11

BP GO:0086005 ventricular cardiac muscle cell action potential 11/2475 35/18870 0.00393668177570784 0.0255522566284864 0.0185600826136644 CAV1/CTNNA3/DSG2/SCN2B/RYR2/KCNE5/KCNE3/KCNJ3/DSC2/SCN3B/KCNE4 11

BP GO:0110110 positive regulation of animal organ morphogenesis 11/2475 35/18870 0.00393668177570784 0.0255522566284864 0.0185600826136644 WNT4/NGFR/HOXC11/HOXA11/BMP2/SOX8/PRKCB/DKK1/GDNF/STOX1/SPRY1 11

BP GO:2000406 positive regulation of T cell migration 11/2475 35/18870 0.00393668177570784 0.0255522566284864 0.0185600826136644 ASCL2/ITGB3/PYCARD/CXCL13/CCL20/ITGA4/CXCL10/CCL5/SPN/FADD/TNFRSF14 11

BP GO:0045582 positive regulation of T cell differentiation 26/2475 116/18870 0.00394322428573393 0.0255659124342338 0.018570001615595 IL7R/MDK/IL18/LEF1/NFKBIZ/ANXA1/ACTL6B/TESPA1/CD74/SOCS1/RHOH/RUNX1/GLI3/HLA-DRB1/IL2RG/SASH3/DUSP10/BTN2A2/RUNX3/LILRB2/HLA-DRA/PTPRC/WNT10B/IL2RA/PIK3R6/IL4I1 26

BP GO:0001654 eye development 70/2475 391/18870 0.00394730193646481 0.0255659124342338 0.018570001615595 COL8A1/HCN1/COL8A2/MFAP2/SIX6/EFEMP1/USH1C/SLC17A8/SPRY2/PROM1/MAB21L1/MEIS3P1/CABP4/RET/MFAP5/NRP1/LCTL/SMOC1/ATP8A2/FREM2/EGFR/TWSG1/PDE6B/WNT16/MEGF11/CRYGS/DSCAM/DLX2/CYP1B1/SOX8/NR2E1/SLC17A7/LAMC3/JAG1/GLI3/SIX5/CLIC4/VEGFA/VSX1/NEUROD4/FZD5/COL4A1/OLFM3/TUB/AQP5/SLC17A6/NKD1/TWIST1/RHOJ/CRB2/SPRED3/CELF4/VIM/STRA6/EPHA2/COL5A2/HPCA/ALDH1A3/RDH10/ATP2B2/COL5A1/CRYGD/TGFB2/SPRY1/OSR2/TCIRG1/AQP1/MEIS1/WNT7B/DLL4 70

BP GO:0070227 lymphocyte apoptotic process 20/2475 82/18870 0.00395530372340889 0.0255901331415377 0.0185875945168818 IL7R/NFKBIZ/FAS/BCL3/CHEK2/CD74/RIPK3/LGALS3/PTCRA/CASP7/CD274/GLI3/JAK3/IDO1/CCL5/LYN/KDELR1/AURKB/IL2RA/FADD 20

BP GO:0090087 regulation of peptide transport 40/2475 200/18870 0.00397662432741346 0.0257003793991286 0.0186676727533556 STX1A/CA2/CHGA/ABCC8/GABBR1/ACVR1C/KCNB1/CD74/CRH/CNR1/GLUD1/PRKCB/S100A8/RAPGEF4/NNAT/BRSK2/HLA-DRB1/SYT7/F2RL1/CCL5/GPR27/HTR2C/KCNJ11/SNAP25/NOS2/F2RL2/NKX6-1/VSNL1/GCK/ALOX5/TUNAR/TRH/ADORA1/PRKCE/IL6/HFE/TCIRG1/GRP/UCP2/PLCB1 40

BP GO:0071320 cellular response to cAMP 14/2475 50/18870 0.00400856745086386 0.0258789666397705 0.0187973910004855 AKAP6/PDE2A/HCN1/STC1/AHR/IGFBP5/CYP1B1/RAP1B/FBP1/WNT10B/AQP9/PIK3CG/AQP1/GPD1 14

BP GO:0002209 behavioral defense response 12/2475 40/18870 0.00406245218716396 0.0261425111808493 0.0189888186511201 HTR1A/MDK/GABRA5/NEUROD2/ASIC1/NR2E1/BRINP1/HTR2C/ASIC4/DPP4/MAPK8IP2/GRP 12

BP GO:0009595 detection of biotic stimulus 12/2475 40/18870 0.00406245218716396 0.0261425111808493 0.0189888186511201 LY96/TLR1/TSPO/TREM2/SSC5D/CLEC7A/HLA-DRB1/TLR2/HLA-B/HLA-A/FAP/LBP 12

BP GO:0032570 response to progesterone 12/2475 40/18870 0.00406245218716396 0.0261425111808493 0.0189888186511201 CAV1/FOSL1/TSPO/THBS1/CYP1B1/TYMS/DSG2/OXTR/TLR2/GJB2/SOX10/TGFB2 12

BP GO:0140894 endolysosomal toll-like receptor signaling pathway 13/2475 45/18870 0.00407622811063844 0.0262030766341255 0.0190328108445566 CAV1/LILRA4/HAVCR2/OAS1/RAB7B/TNFAIP3/F2RL1/SCIMP/TLR8/RSAD2/UNC93B1/MYD88/COLEC12 13

BP GO:0097696 receptor signaling pathway via STAT 38/2475 188/18870 0.00408070473261023 0.0262037980904725 0.0190333348800497 CAV1/IL7R/IL18/MIR221/PARP9/HAMP/PTPRT/CD40/RET/HPX/BCL3/OCIAD2/SOCS1/CYP1B1/CCL2/STAT1/TNFRSF1A/CTF1/JAK3/NMI/CCL5/SOCS2/TNFRSF18/LYN/IL10RA/SOCS3/LIF/CAMK2A/CD300A/CSF2RB/OSM/IL9/GADD45A/PTPRC/IL6/PPARG/HCLS1/CLCF1 38

BP GO:0048736 appendage development 37/2475 182/18870 0.00412461680433188 0.0264292415082269 0.0191970874800281 SHOX2/SP8/HAND2/PITX1/MEOX2/LEF1/HOXC11/HOXA11/SMOC1/COL3A1/FREM2/HOXD9/MAP3K20/ITGB4/DLX5/GLI3/HOXA9/DKK1/SFRP2/TBX5/RUNX2/ECE1/PCSK5/OSR1/HOXD10/TWIST1/HOXA10/HOXC10/HOXC13/RDH10/TGFB2/OSR2/NOG/COL2A1/EN1/HOXD13/FZD6 37

BP GO:0060173 limb development 37/2475 182/18870 0.00412461680433188 0.0264292415082269 0.0191970874800281 SHOX2/SP8/HAND2/PITX1/MEOX2/LEF1/HOXC11/HOXA11/SMOC1/COL3A1/FREM2/HOXD9/MAP3K20/ITGB4/DLX5/GLI3/HOXA9/DKK1/SFRP2/TBX5/RUNX2/ECE1/PCSK5/OSR1/HOXD10/TWIST1/HOXA10/HOXC10/HOXC13/RDH10/TGFB2/OSR2/NOG/COL2A1/EN1/HOXD13/FZD6 37

BP GO:0010977 negative regulation of neuron projection development 30/2475 140/18870 0.00419526718984668 0.0268532880680591 0.0195050970346031 MDM2/MCF2/SEMA3E/DNM3/STX1B/SPP1/FGF13/NRP1/SEMA3A/MIR210/SEMA3D/FLNA/SLIT1/RTN4R/DKK1/RTN4RL1/SEMA6B/PMP22/LRRK2/TNR/SEMA3F/DRAXIN/VIM/FSTL4/EFNB2/STMN2/B2M/NEU4/MGARP/MT3 30

BP GO:0010976 positive regulation of neuron projection development 33/2475 158/18870 0.00422626345472871 0.0270228815571791 0.019628282599564 MDK/MIR221/ROR2/ITGA3/LRRC7/RET/NRP1/ATP8A2/FLNA/AVIL/NEGR1/CUX2/CNR1/ROR1/S100A9/TOX/VEGFA/PLK5/FES/LYN/ZNF804A/TRIM67/PACSIN1/P3H1/ELAVL4/STMN2/FZD1/EPHA3/NTRK1/SERPINI1/EZH2/PTN/FUT9 33

BP GO:0048332 mesoderm morphogenesis 19/2475 77/18870 0.00427515286136821 0.0272773834002707 0.0198131420153795 ITGB3/SNAI1/LEF1/ITGA2/HOXA11/ITGA3/TWSG1/ITGB4/DKK1/SFRP2/ETV2/EYA2/CRB2/EPHA2/ITGB1/TBX19/EYA1/NOG/NODAL 19

BP GO:0072078 nephron tubule morphogenesis 19/2475 77/18870 0.00427515286136821 0.0272773834002707 0.0198131420153795 WNT4/HOXD11/NPNT/HS3ST3B1/HOXA11/CITED1/BMP2/SOX8/FOXD1/GLI3/GREB1L/VEGFA/OSR1/TACSTD2/HS3ST3A1/GDNF/EYA1/NOG/HOXB7 19

BP GO:0001911 negative regulation of leukocyte mediated cytotoxicity 9/2475 26/18870 0.004331030163947 0.0274588227078541 0.0199449318837686 IL7R/HAVCR2/MICA/FCGR2B/HLA-B/LILRB1/HLA-A/PTPRC/HLA-F 9

BP GO:0002407 dendritic cell chemotaxis 9/2475 26/18870 0.004331030163947 0.0274588227078541 0.0199449318837686 SPI1/SLAMF8/CCL5/CXCR4/CCR5/CXCR2/PIK3CG/CCR1/GPR183 9

BP GO:0002438 acute inflammatory response to antigenic stimulus 9/2475 26/18870 0.004331030163947 0.0274588227078541 0.0199449318837686 FCGR1A/CNR1/FCGR2B/SPN/IGHG1/ZP3/CXCR2/C3/FCGR3A 9

BP GO:0002726 positive regulation of T cell cytokine production 9/2475 26/18870 0.004331030163947 0.0274588227078541 0.0199449318837686 IL18/IL1R1/ARID5A/SASH3/FZD5/HLA-A/RSAD2/B2M/IL6 9

BP GO:0033622 integrin activation 9/2475 26/18870 0.004331030163947 0.0274588227078541 0.0199449318837686 FERMT1/KIF14/CXCL13/PTGER4/FBLIM1/FN1/RAP1B/PLEK/FERMT3 9

BP GO:0099637 neurotransmitter receptor transport 9/2475 26/18870 0.004331030163947 0.0274588227078541 0.0199449318837686 CACNG2/STX1B/LRRC7/GRIP1/SNAP25/ARHGAP44/CPLX1/CACNG5/CACNG3 9

BP GO:1905954 positive regulation of lipid localization 25/2475 111/18870 0.00434364689836778 0.0275097636896626 0.0199819332666419 CAV1/CES1/IL1A/SPP1/ATP8A1/C1QTNF1/TREM2/ATP8A2/CRH/PLIN2/PON1/ABCG4/HILPDA/ABCB4/PTGES/CD36/ERFE/C3/MSR1/LRAT/PPARG/PLA2G4A/ZC3H12A/GAL/ANXA2 25

BP GO:0043254 regulation of protein-containing complex assembly 74/2475 419/18870 0.00440495153060532 0.0278686290724492 0.02024259796051 CLDN7/GBP5/FERMT1/TMOD2/STX1A/PYCARD/HIP1R/CDK5R1/KIF14/CXCL13/TMSB4X/MSN/CCL26/STX1B/NAPB/GBP2/LCP1/CAPG/KIF9/VASP/MPP7/TREM2/TFRC/PSRC1/STXBP1/MMP1/AVIL/COTL1/LGALS3/CLEC7A/HAUS1/SSH3/ANKRD53/ARHGAP18/MET/PTGER4/ARPC5/SCIN/FCHSD1/VEGFA/HJURP/CAMSAP3/DKK1/CDC42EP5/FBLIM1/FES/AJUBA/MAPT/CSF3/ZDHHC12/PAK3/CD36/ISG15/RAP1B/H3-3A/CAPZA1/WARS1/ELN/SPTBN2/RHOC/SPTB/STXBP6/DAAM2/STMN2/PLEK/TMSB10/WNT10B/CARMIL2/PRKCE/TPPP3/CALY/MYD88/HCLS1/PFN1 74

BP GO:0032411 positive regulation of transporter activity 22/2475 94/18870 0.00443541037731526 0.0280076929418272 0.0203436080960044 VMP1/AKAP6/SYNGR3/TMSB4X/STAC/FGF13/TREM2/NIPSNAP2/PON1/P2RY6/CCL2/WNK2/GLRX/PIRT/RYR2/ARC/LRRC55/FXYD2/ANK3/GAL/CTSS/KCNIP2 22

BP GO:0045599 negative regulation of fat cell differentiation 16/2475 61/18870 0.00444092666232836 0.0280076929418272 0.0203436080960044 DDIT3/TGFB1I1/WWTR1/SOD2/BMP2/CCN4/JAG1/RUNX1T1/TLCD3B/ENPP1/MIR27B/TRIB3/E2F1/WNT10B/IL6/MMP11 16

BP GO:0090497 mesenchymal cell migration 16/2475 61/18870 0.00444092666232836 0.0280076929418272 0.0203436080960044 EFNB1/HAND2/SEMA3E/RET/NRP1/SEMA3A/SEMA3D/SOX8/FOLR1/SEMA6B/FN1/SOX10/TWIST1/SEMA3F/TBX1/GDNF 16

BP GO:0048638 regulation of developmental growth 60/2475 328/18870 0.00448875006337165 0.0282795964118398 0.0205411073204272 AKAP6/UNC13A/SEMA3E/NKX2-5/MAEL/DUSP6/SPP1/FGF13/HOPX/NRP1/RIMS1/SEMA3A/ATP8A2/PLAC8/CDH4/SEMA3D/SLIT1/RUNX1/DSCAM/RTN4R/CPNE9/VEGFA/L1CAM/CXCR4/SEMA6B/TBX5/SOCS2/IGF2/SASH3/MYOD1/DUSP10/FN1/SYT1/MAPT/SYT4/YBX3/RASAL1/TNR/NKD1/GLI1/GPR21/ZP3/SEMA3F/NKX6-1/DRAXIN/FSTL4/FOSL2/CDK1/TRPV2/SYT2/RGS4/NOG/ISLR2/FOXS1/LATS2/PLCB1/GDF15/MT3/RIMS2/TP73 60

BP GO:0032479 regulation of type I interferon production 27/2475 123/18870 0.00453648651018022 0.0285204869184524 0.020716080034915 IRF7/PYCARD/RELB/SIGLEC1/OAS2/MMP12/CGAS/TRIM21/LILRA4/HAVCR2/STAT1/TRIM38/OAS1/TLR2/NMI/IFIH1/TLR8/OAS3/XAF1/CD14/ISG15/TYROBP/LILRB1/RNF135/HMGB2/IRF1/MYD88 27

BP GO:0032606 type I interferon production 27/2475 123/18870 0.00453648651018022 0.0285204869184524 0.020716080034915 IRF7/PYCARD/RELB/SIGLEC1/OAS2/MMP12/CGAS/TRIM21/LILRA4/HAVCR2/STAT1/TRIM38/OAS1/TLR2/NMI/IFIH1/TLR8/OAS3/XAF1/CD14/ISG15/TYROBP/LILRB1/RNF135/HMGB2/IRF1/MYD88 27

BP GO:0061844 antimicrobial humoral immune response mediated by antimicrobial peptide 20/2475 83/18870 0.0045821458551361 0.0287774097429259 0.0209026979496024 CXCL14/CXCL13/CXCL8/KLK7/S100A12/H2BC8/PPBP/S100A9/CXCL10/H2BC11/H2BC12/ANG/LTF/CXCL9/RNASE3/H2BC4/RNASE6/CXCL6/CXCL11/GNLY 20

BP GO:0030858 positive regulation of epithelial cell differentiation 18/2475 72/18870 0.00460513741265658 0.0288915830988403 0.0209856286648366 MACROH2A2/PROM1/SFRP4/FOXJ1/BMP2/ALOX15B/ATOH8/ETV2/CYP27B1/SERPINE1/LIF/TMEM100/SFN/NKX6-1/PLAAT4/WNT10B/GDNF/TP73 18

BP GO:0009266 response to temperature stimulus 35/2475 171/18870 0.00462160704076858 0.0289646437085329 0.0210386968134317 ANO3/NGFR/HSP90AB3P/IL1A/STAC/VGF/HMOX1/THBS1/PLAC8/HSPB1/MICA/DNAJB1/MICB/CXCL10/PIRT/HTR2A/SCN2B/TRPM8/CXCR4/LYN/HSPA6/MAPT/SLC12A5/TCIM/LXN/HSPB6/HSP90AA2P/CDH8/ADORA1/SLC25A27/TFEC/TRPV2/NTRK1/GMPR/UCP2 35

BP GO:0031570 DNA integrity checkpoint signaling 29/2475 135/18870 0.00464688262339433 0.0290926832855679 0.0211316993674581 MDM2/PARP9/PLK3/FANCD2/FBXO4/CHEK2/WDR76/MAP3K20/DTL/TIMELESS/ORC1/BRCA1/CAMSAP3/MUC1/DTX3L/RAD51/CDK2/BRCA2/CLSPN/CDC45/E2F1/BRIP1/CDT1/PLK1/CDC6/CDK1/GTSE1/CHEK1/EME1 29

BP GO:0002279 mast cell activation involved in immune response 15/2475 56/18870 0.00467377433590092 0.0292001468394892 0.0212097563652308 CHGA/CPLX2/VAMP8/STXBP1/IL13RA2/S100A13/ADGRE2/FES/LYN/CD300A/RAC2/LAT2/MILR1/PIK3CG/GRP 15

BP GO:0043370 regulation of CD4-positive, alpha-beta T cell differentiation 15/2475 56/18870 0.00467377433590092 0.0292001468394892 0.0212097563652308 ASCL2/BATF/IL18/NFKBIZ/LOXL3/ANXA1/SOCS1/RUNX1/HLA-DRB1/JAK3/IL2RG/SASH3/RUNX3/HLA-DRA/ZC3H12A 15

BP GO:0016358 dendrite development 45/2475 233/18870 0.00468649485002773 0.0292374164232397 0.0212368274205761 IL1RAPL1/CUL7/MCF2/CDK5R1/DNM3/CPEB3/DCDC2/ACTL6B/ADGRB3/CHRNB2/NRP1/SEMA3A/DSCAM/CUX2/NR2E1/SHANK1/GRIN3A/CD3E/FEZF2/ABI3/HECW1/SLC12A5/CSMD3/LRRK2/CAMK2A/SULT4A1/ARC/FLRT1/PAK3/PPFIA2/IGSF9/LZTS1/FSTL4/PACSIN1/RAP2A/PHACTR1/ELAVL4/ITGB1/MAPK8IP2/ARHGAP44/NEURL1/SLITRK5/EZH2/PTN/MGARP 45

BP GO:0098656 monoatomic anion transmembrane transport 31/2475 147/18870 0.00468947901658558 0.0292374164232397 0.0212368274205761 GLRA3/ANO3/SLC4A10/SLC17A8/CLIC3/GABRA3/GABRA5/GABRD/GABRA4/TCAF2/GABRB3/SLC17A7/CLIC4/APOL1/GRM5/TTYH3/BEST4/SLC12A5/SLC17A6/SLC26A2/SLC12A7/GABRA1/GABRB2/GABRG2/GABRG1/SLC25A27/ABCC3/CLIC1/NMUR2/UCP2/GABRE 31

BP GO:0034143 regulation of toll-like receptor 4 signaling pathway 10/2475 31/18870 0.00478043774284421 0.0297119546667046 0.0215815120067599 TREM2/RAB7B/TNFAIP3/F2RL1/LYN/CD14/LTF/LILRA2/IFI35/LBP 10

BP GO:0060325 face morphogenesis 10/2475 31/18870 0.00478043774284421 0.0297119546667046 0.0215815120067599 LEF1/CRISPLD1/MMP2/DLX5/DKK1/COL1A1/STRA6/RRAS/TBX1/NOG 10

BP GO:0086011 membrane repolarization during action potential 10/2475 31/18870 0.00478043774284421 0.0297119546667046 0.0215815120067599 CAV1/KCNA1/FLNA/SCN2B/KCNE5/KCNE3/KCNJ3/KCNH7/KCNE4/KCNIP2 10

BP GO:0051347 positive regulation of transferase activity 73/2475 414/18870 0.00484388922615354 0.0300546422614069 0.0218304258369874 NEK2/MAP2K3/ITGB3/IL18/EMP2/SLC11A1/KIF14/NCF1/ROR2/IQGAP1/ECT2/ACE/THBS1/TREM2/CD40/FCGR1A/PSRC1/RET/S100A12/UBE2C/CD74/NOX4/EGFR/RIPK3/UBE2S/CCN1/LCP2/LILRA5/TOM1L1/MET/ROR1/PKIB/HLA-DRB1/ANGPT1/VEGFA/CCL5/DKK1/CHI3L1/IGF2/GRM5/SLC8A2/MAPT/TCIM/TRIB3/LRRK2/PDGFA/ANG/LTF/PPIA/RFC2/CD300A/PCNA/EPHB4/PDGFD/AURKB/CLSPN/ADAM9/PLAAT4/EPHA2/MMD2/PLK1/CENPE/PTPRC/NTRK1/PIK3CG/STOX1/TPX2/TGFB2/PIK3R6/EZH2/ADRA2B/MT3/CDC20 73

BP GO:0048863 stem cell differentiation 47/2475 246/18870 0.00484558522802163 0.0300546422614069 0.0218304258369874 BATF/EFNB1/HAND2/SEMA3E/NKX2-5/ACE/RET/NRP1/SEMA3A/SEMA3D/HOXB4/HOXD4/SOX8/TEAD2/JAG1/ZFP36/GSC/FOLR1/FRZB/SFRP2/SEMA6B/TBX5/FN1/RUNX2/DMRTA2/OSR1/LIF/TACSTD2/SOX10/TWIST1/SEMA3F/TRIM6/HOXA7/OSM/SNAI2/RBM24/TBX1/PTPRC/ITGB1/RDH10/FZD1/GDNF/TGFB2/NOG/EZH2/PTN/CDK6 47

BP GO:0045581 negative regulation of T cell differentiation 14/2475 51/18870 0.00487111777338015 0.030150712485953 0.0219002072003407 ASCL2/MDK/LOXL3/ANXA1/FOXJ1/CD74/SOCS1/RUNX1/GLI3/JAK3/RUNX3/FGL2/ZC3H12A/IRF1 14

BP GO:0048013 ephrin receptor signaling pathway 14/2475 51/18870 0.00487111777338015 0.030150712485953 0.0219002072003407 EFNB1/CDK5R1/MMP2/EPHA10/MMP9/LYN/PAK3/EFNA4/EPHB4/EPHA2/EFNB2/EPHB6/EPHA3/NTRK1 14

BP GO:0009620 response to fungus 17/2475 67/18870 0.00493994780528005 0.0304824734048319 0.0221411843536272 RARRES2/SPI1/HAMP/CHGA/S100A12/COTL1/CLEC7A/IL17RC/PLA2G5/S100A8/SPON2/S100A9/SCIMP/LTF/PTX3/GNLY/MYD88 17

BP GO:0048645 animal organ formation 17/2475 67/18870 0.00493994780528005 0.0304824734048319 0.0221411843536272 HAND2/EMP2/HOXA3/HOXC11/HOXA11/NKX3-2/BMP2/GLI3/FOLR1/DKK1/TBX5/TBR1/RDH10/GDNF/SPRY1/EYA1/NOG 17

BP GO:0070613 regulation of protein processing 17/2475 67/18870 0.00493994780528005 0.0304824734048319 0.0221411843536272 MDM2/PLAT/THBS1/RUNX1/S100A10/MMP14/CARD16/IL1R2/BCL2L12/LRRK2/SERPINE1/PLAU/CST7/GAS1/CTSZ/PLAUR/ANXA2 17

BP GO:2000514 regulation of CD4-positive, alpha-beta T cell activation 19/2475 78/18870 0.00497177409329226 0.0306473630966393 0.0222609532784068 ASCL2/BATF/IL18/NFKBIZ/LOXL3/ANXA1/SOCS1/TWSG1/RUNX1/CD274/HLA-DRB1/CD3E/JAK3/IL2RG/SASH3/RUNX3/HLA-DRA/IL2RA/ZC3H12A 19

BP GO:0048483 autonomic nervous system development 13/2475 46/18870 0.00501243207333203 0.0307783492274414 0.0223560960849415 HAND2/HOXB2/KIF26A/RET/NRP1/SEMA3A/SOX8/FN1/SOX10/SEMA3F/TBX1/GDNF/NTRK1 13

BP GO:1901976 regulation of cell cycle checkpoint 13/2475 46/18870 0.00501243207333203 0.0307783492274414 0.0223560960849415 MAD2L1/FBXO4/WDR76/MAP3K20/CCNB1/BIRC5/BRCA1/RAD51/BRCA2/AURKB/CDCA8/NDC80/KNL1 13

BP GO:0051928 positive regulation of calcium ion transport 26/2475 118/18870 0.00501313662657574 0.0307783492274414 0.0223560960849415 AKAP5/CAV1/VMP1/AKAP6/STAC/TSPO/STC1/NIPSNAP2/APLNR/LGALS3/LILRA5/P2RY6/CCL2/CXCL10/CCL5/RYR2/GRIN1/CAMK2A/CXCL9/ADCYAP1R1/F2RL3/LILRA2/TRPV2/CXCL11/RAMP3/CCR1 26

BP GO:0015669 gas transport 8/2475 22/18870 0.00501865487902483 0.0307783492274414 0.0223560960849415 CA2/IPCEF1/HBQ1/NGB/HBA1/AQP5/RHBG/AQP1 8

BP GO:0072234 metanephric nephron tubule development 8/2475 22/18870 0.00501865487902483 0.0307783492274414 0.0223560960849415 WWTR1/SLC22A6/STAT1/SOX8/OSR1/LIF/AQP1/WNT7B 8

BP GO:0031952 regulation of protein autophosphorylation 12/2475 41/18870 0.00507022359434948 0.0310165435412547 0.0225291103985796 CAV1/ACE/TOM1L1/ERRFI1/ENG/GPNMB/VEGFA/ENPP1/PDGFA/PDGFD/RAP2A/PTPRC 12

BP GO:0035136 forelimb morphogenesis 12/2475 41/18870 0.00507022359434948 0.0310165435412547 0.0225291103985796 SHOX2/HOXA11/HOXD9/HOXA9/TBX5/RUNX2/OSR1/HOXD10/TWIST1/RDH10/OSR2/EN1 12

BP GO:0045638 negative regulation of myeloid cell differentiation 22/2475 95/18870 0.00507299229805331 0.0310165435412547 0.0225291103985796 LRRC17/H4C9/HOXA5/LILRB3/H4C5/HOXA9/ZFP36/LYN/FSTL3/H4C8/LTF/TNFRSF11B/LILRB1/TNFAIP6/H4C11/HOXA7/HOXB8/ERFE/H4C3/C1QC/MEIS1/CDK6 22

BP GO:0002702 positive regulation of production of molecular mediator of immune response 29/2475 136/18870 0.00518336893221584 0.0316591526643173 0.0229958745903199 PYCARD/IL18/TNFRSF4/KLK7/CD40/TFRC/HPX/CD74/IL1R1/ARID5A/CLEC7A/SPON2/F2RL1/SCIMP/SASH3/FZD5/CD36/LAPTM5/LILRB1/TRIM6/HLA-A/RSAD2/PTPRC/B2M/IL6/TNFRSF14/MYD88/CLCF1/HLA-F 29

BP GO:0034614 cellular response to reactive oxygen species 32/2475 154/18870 0.00519257936604378 0.0316831773513484 0.0230133251107396 MDM2/H19/NCF1/ECT2/SOD2/ANXA1/PAWR/GCH1/MMP2/EGFR/RIPK3/CYP1B1/MMP9/MET/NET1/TNFAIP3/SMPD3/SPHK1/HGF/MAPT/SOD3/LRRK2/CD36/PCNA/PDGFD/FBLN5/CDK1/IL6/CRYGD/AQP1/EZH2/MT3 32

BP GO:0002292 T cell differentiation involved in immune response 20/2475 84/18870 0.00528885096764377 0.0322051330727517 0.0233924517550423 ASCL2/BATF/IL18/LEF1/NFKBIZ/RELB/LOXL3/ANXA1/BCL3/PTGER4/HLA-DRB1/JAK3/SOCS3/SPN/HLA-DRA/FGL2/FCER1G/IL6/ZC3H12A/GPR183 20

BP GO:0048678 response to axon injury 20/2475 84/18870 0.00528885096764377 0.0322051330727517 0.0233924517550423 MIR221/TSPO/SPP1/SOD2/TREM2/NEFL/MMP2/KCNB1/FLNA/RTN4R/GAP43/FOLR1/RTN4RL1/LYN/TNC/TNR/TYROBP/CDK1/NTRK1/PTN 20

BP GO:0033674 positive regulation of kinase activity 61/2475 337/18870 0.00531860279131004 0.0323534864833085 0.0235002094218498 MAP2K3/ITGB3/IL18/EMP2/SLC11A1/KIF14/NCF1/ROR2/IQGAP1/ECT2/ACE/THBS1/TREM2/CD40/FCGR1A/PSRC1/RET/S100A12/CD74/NOX4/EGFR/RIPK3/CCN1/LCP2/LILRA5/TOM1L1/MET/ROR1/HLA-DRB1/ANGPT1/VEGFA/CCL5/DKK1/CHI3L1/GRM5/SLC8A2/MAPT/TCIM/LRRK2/PDGFA/ANG/LTF/PPIA/CD300A/EPHB4/PDGFD/CLSPN/ADAM9/EPHA2/MMD2/CENPE/PTPRC/NTRK1/PIK3CG/STOX1/TPX2/TGFB2/PIK3R6/EZH2/ADRA2B/MT3 61

BP GO:0042093 T-helper cell differentiation 18/2475 73/18870 0.00537897478685769 0.0326876160124429 0.0237429070338285 ASCL2/BATF/IL18/LEF1/NFKBIZ/RELB/LOXL3/ANXA1/BCL3/PTGER4/HLA-DRB1/JAK3/SOCS3/SPN/HLA-DRA/IL6/ZC3H12A/GPR183 18

BP GO:0001820 serotonin secretion 5/2475 10/18870 0.0054940994973629 0.033019592975142 0.0239840411122433 HTR1A/CNR1/LILRB1/HRH3/MAOB 5

BP GO:0001886 endothelial cell morphogenesis 5/2475 10/18870 0.0054940994973629 0.033019592975142 0.0239840411122433 STC1/MET/CLIC4/COL27A1/PLOD3 5

BP GO:0002883 regulation of hypersensitivity 5/2475 10/18870 0.0054940994973629 0.033019592975142 0.0239840411122433 FCGR1A/FCGR2B/SPN/ZP3/C3 5

BP GO:0014012 peripheral nervous system axon regeneration 5/2475 10/18870 0.0054940994973629 0.033019592975142 0.0239840411122433 MIR221/TSPO/NEFL/MMP2/TNC 5

BP GO:0061299 retina vasculature morphogenesis in camera-type eye 5/2475 10/18870 0.0054940994973629 0.033019592975142 0.0239840411122433 NRP1/CYP1B1/CLIC4/COL4A1/RHOJ 5

BP GO:0070391 response to lipoteichoic acid 5/2475 10/18870 0.0054940994973629 0.033019592975142 0.0239840411122433 TREM2/TLR2/CD14/CD36/LBP 5

BP GO:0071223 cellular response to lipoteichoic acid 5/2475 10/18870 0.0054940994973629 0.033019592975142 0.0239840411122433 TREM2/TLR2/CD14/CD36/LBP 5

BP GO:0090184 positive regulation of kidney development 5/2475 10/18870 0.0054940994973629 0.033019592975142 0.0239840411122433 WNT4/RET/SOX8/FOXD1/NOG 5

BP GO:0140059 dendrite arborization 5/2475 10/18870 0.0054940994973629 0.033019592975142 0.0239840411122433 NRP1/SEMA3A/SULT4A1/PHACTR1/PTN 5

BP GO:2000425 regulation of apoptotic cell clearance 5/2475 10/18870 0.0054940994973629 0.033019592975142 0.0239840411122433 TREM2/CCL2/CD300LF/C2/C3 5

BP GO:2000659 regulation of interleukin-1-mediated signaling pathway 5/2475 10/18870 0.0054940994973629 0.033019592975142 0.0239840411122433 IL1RN/VRK2/IL1R1/IL1R2/IL6 5

BP GO:0038066 p38MAPK cascade 15/2475 57/18870 0.00558951602669539 0.0334590769932992 0.0243032637861143 MAP2K3/HAND2/SPI1/NCF1/TREM2/BMP2/MAP3K20/VEGFA/ZFP36/SPHK1/HGF/DUSP10/ULK4/GADD45A/ZC3H12A 15

BP GO:0061900 glial cell activation 15/2475 57/18870 0.00558951602669539 0.0334590769932992 0.0243032637861143 ITGB2/IFNGR2/TREM2/CTSC/TAFA3/C5AR1/TLR2/SPHK1/MAPT/LRRK2/TYROBP/CST7/PTPRC/IL6/C1QA 15

BP GO:0072132 mesenchyme morphogenesis 15/2475 57/18870 0.00558951602669539 0.0334590769932992 0.0243032637861143 MDM2/SNAI1/LEF1/APLNR/BMP2/ENG/ACTA2/MDM4/ACTG2/OSR1/TMEM100/TWIST1/SNAI2/TGFB2/NOG 15

BP GO:1901016 regulation of potassium ion transmembrane transporter activity 15/2475 57/18870 0.00558951602669539 0.0334590769932992 0.0243032637861143 CAV1/AKAP6/TREM2/KCNAB1/KCNS2/KCNE5/KCNE3/LRRC55/ITGB1/FXYD2/ANK3/GAL/KCNE4/GRP/KCNIP2 15

BP GO:0000076 DNA replication checkpoint signaling 7/2475 18/18870 0.00562289107920197 0.0335584871168276 0.0243754711113673 TIMELESS/ORC1/CAMSAP3/CLSPN/CDC45/CDT1/CDC6 7

BP GO:0051917 regulation of fibrinolysis 7/2475 18/18870 0.00562289107920197 0.0335584871168276 0.0243754711113673 THBD/PLAT/THBS1/SERPINE1/PLAU/FAP/PLAUR 7

BP GO:1901970 positive regulation of mitotic sister chromatid separation 7/2475 18/18870 0.00562289107920197 0.0335584871168276 0.0243754711113673 UBE2C/DLGAP5/BIRC5/ESPL1/AURKB/CDCA8/CDC20 7

BP GO:0030509 BMP signaling pathway 34/2475 167/18870 0.00565233023808063 0.0337006859478015 0.0244787583508786 LEF1/ITGA3/SFRP4/FSTL1/MIR210/TWSG1/CCN1/BMP2/DLX5/ENG/FOXD1/SMPD3/DKK1/SFRP2/RUNX2/FSTL3/ETV2/PCSK6/FSTL5/TMEM100/VWC2L/CHRDL2/TNFAIP6/CRB2/FSTL4/FST/GATA4/GREM2/FZD1/SMAD9/HTRA3/PPARG/HFE/NOG 34

BP GO:0098661 inorganic anion transmembrane transport 28/2475 131/18870 0.00574642669504286 0.0340930326460095 0.02476374246159 GLRA3/ANO3/SLC17A8/CLIC3/GABRA3/GABRA5/GABRD/GABRA4/GABRB3/SLC17A7/CLIC4/APOL1/TTYH3/BEST4/SLC12A5/SLC17A6/SLC26A2/SLC12A7/GABRA1/GABRB2/GABRG2/SLC37A2/GABRG1/SLC25A27/CLIC1/NMUR2/UCP2/GABRE 28

BP GO:0006817 phosphate ion transport 9/2475 27/18870 0.00575205634426766 0.0340930326460095 0.02476374246159 SLC17A8/SFRP4/SLC17A7/SLC34A2/CEBPB/SLC17A6/CRY2/SLC37A2/UCP2 9

BP GO:0021895 cerebral cortex neuron differentiation 9/2475 27/18870 0.00575205634426766 0.0340930326460095 0.02476374246159 EMX1/DLX2/NR2E1/OPHN1/TOX/FEZF2/CHD5/ELAVL4/LHX6 9

BP GO:0051984 positive regulation of chromosome segregation 9/2475 27/18870 0.00575205634426766 0.0340930326460095 0.02476374246159 CCNB1/BIRC5/AURKB/NCAPG/SMC4/CDC6/CDCA8/CDK1/NCAPH 9

BP GO:1903859 regulation of dendrite extension 9/2475 27/18870 0.00575205634426766 0.0340930326460095 0.02476374246159 UNC13A/RIMS1/CPNE9/CXCR4/SYT1/SYT4/RASAL1/SYT2/RIMS2 9

BP GO:0045685 regulation of glial cell differentiation 19/2475 79/18870 0.00575902429434185 0.0340930326460095 0.02476374246159 MDK/TREM2/DLX2/BMP2/NR2E1/SPINT1/GSX2/TLR2/CXCR4/DUSP10/LIF/NKX6-1/DAAM2/CDK1/IL6/NOG/PTN/CLCF1/TP73 19

BP GO:0002791 regulation of peptide secretion 39/2475 198/18870 0.005763562314407 0.0340930326460095 0.02476374246159 STX1A/CHGA/ABCC8/GABBR1/ACVR1C/KCNB1/CD74/CRH/CNR1/GLUD1/PRKCB/S100A8/RAPGEF4/NNAT/BRSK2/HLA-DRB1/SYT7/F2RL1/CCL5/GPR27/HTR2C/KCNJ11/SNAP25/NOS2/F2RL2/NKX6-1/VSNL1/GCK/ALOX5/TUNAR/TRH/ADORA1/PRKCE/IL6/HFE/TCIRG1/GRP/UCP2/PLCB1 39

BP GO:0031334 positive regulation of protein-containing complex assembly 39/2475 198/18870 0.005763562314407 0.0340930326460095 0.02476374246159 GBP5/FERMT1/PYCARD/HIP1R/CDK5R1/CXCL13/MSN/CCL26/GBP2/LCP1/VASP/MPP7/TFRC/PSRC1/MMP1/LGALS3/CLEC7A/ANKRD53/MET/FCHSD1/VEGFA/CDC42EP5/FES/AJUBA/MAPT/CSF3/CD36/ISG15/RAP1B/WARS1/RHOC/PLEK/WNT10B/CARMIL2/PRKCE/TPPP3/CALY/MYD88/PFN1 39

BP GO:0002690 positive regulation of leukocyte chemotaxis 22/2475 96/18870 0.00578456832315659 0.0341109668933194 0.0247767691432067 RARRES2/MDK/CXCL13/SPI1/CXCL8/THBS1/CD74/VEGFA/CXCL10/C5AR1/F2RL1/CCL5/FPR2/SERPINE1/THBS4/RAC2/CXCR2/IL6/LBP/SWAP70/PTN/CCR1 22

BP GO:0014909 smooth muscle cell migration 22/2475 96/18870 0.00578456832315659 0.0341109668933194 0.0247767691432067 MDM2/MDK/DDIT3/ITGB3/MIR221/S100A11/PLAT/ITGA2/ACE/NRP1/IGFBP3/IGFBP5/CYP1B1/P2RY6/CCN4/CCL5/HAS2/SERPINE1/PDGFA/PLAU/PDGFD/POSTN 22

BP GO:0035082 axoneme assembly 22/2475 96/18870 0.00578456832315659 0.0341109668933194 0.0247767691432067 SPAG1/CFAP100/DRC1/DNAI3/FOXJ1/LRRC61/RSPH1/DNAI4/CFAP73/CFAP157/ZMYND10/JHY/LRGUK/CCDC40/TTC12/CFAP43/DRC7/SPEF1/RSPH9/IQCG/RSPH4A/NEURL1 22

BP GO:0071559 response to transforming growth factor beta 54/2475 293/18870 0.00578931966427256 0.0341109668933194 0.0247767691432067 CAV1/PDE2A/WNT4/FERMT1/TGFB1I1/FMOD/NFKBIZ/SPI1/NPNT/CTSK/LRRC32/ZYX/TET1/SPRY2/THBS1/ITGA3/CLDN1/COL3A1/CITED1/TWSG1/BMP2/LOX/ENG/ACTA2/LTBP1/SLC2A10/FOLR1/CAV2/COL1A1/MIR27B/COL4A2/RUNX3/COL1A2/MXRA5/WFIKKN2/EMILIN1/LTBP2/VEPH1/PDGFD/SPRED3/ADAM9/GDF10/VASN/POSTN/ASPN/CILP/SMAD9/HTRA3/PPARG/CD109/TGFB2/SPRY1/LATS2/GDF15 54

BP GO:0031345 negative regulation of cell projection organization 38/2475 192/18870 0.00586629585522913 0.0345306277596036 0.0250815931147722 MDM2/MCF2/SEMA3E/DNM3/STX1B/SPP1/SPRY2/FGF13/ITGA3/NRP1/SEMA3A/MIR210/SEMA3D/FLNA/SLIT1/RTN4R/KIF24/DKK1/RTN4RL1/SEMA6B/ABI3/PMP22/LRRK2/TACSTD2/TNR/SEMA3F/DRAXIN/VIM/FSTL4/EFNB2/STMN2/MAK/B2M/ARHGAP44/GRIN2B/NEU4/MGARP/MT3 38

BP GO:0046131 pyrimidine ribonucleoside metabolic process 6/2475 14/18870 0.00592683809757972 0.034682978496948 0.0251922542713376 APOBEC3B/APOBEC3G/APOBEC3C/UPP1/APOBEC3F/APOBEC3H 6

BP GO:0046133 pyrimidine ribonucleoside catabolic process 6/2475 14/18870 0.00592683809757972 0.034682978496948 0.0251922542713376 APOBEC3B/APOBEC3G/APOBEC3C/UPP1/APOBEC3F/APOBEC3H 6

BP GO:0071415 cellular response to purine-containing compound 6/2475 14/18870 0.00592683809757972 0.034682978496948 0.0251922542713376 P2RY6/SELENON/RYR2/P2RY1/RYR3/CHEK1 6

BP GO:0098814 spontaneous synaptic transmission 6/2475 14/18870 0.00592683809757972 0.034682978496948 0.0251922542713376 STX1B/DOC2A/CBLN2/SYT1/ITGB1/RIMS2 6

BP GO:1900426 positive regulation of defense response to bacterium 6/2475 14/18870 0.00592683809757972 0.034682978496948 0.0251922542713376 CYBA/KLK7/HAVCR2/F2RL1/EMILIN2/EMILIN1 6

BP GO:1900452 regulation of long-term synaptic depression 6/2475 14/18870 0.00592683809757972 0.034682978496948 0.0251922542713376 KCNB1/MAPT/LILRB2/ARC/CBLN1/ADORA1 6

BP GO:0032414 positive regulation of ion transmembrane transporter activity 20/2475 85/18870 0.00608278171816869 0.0355608777369862 0.0258299232904965 VMP1/AKAP6/TMSB4X/STAC/FGF13/TREM2/NIPSNAP2/P2RY6/CCL2/WNK2/GLRX/PIRT/RYR2/ARC/LRRC55/FXYD2/ANK3/GAL/CTSS/KCNIP2 20

BP GO:1905314 semi-lunar valve development 13/2475 47/18870 0.00611339796313677 0.0357050986096042 0.0259346792558628 SNAI1/NKX2-5/BMP2/JAG1/TNFRSF1A/EMILIN1/TWIST1/STRA6/ELN/SNAI2/GATA4/TGFB2/DLL4 13

BP GO:0071453 cellular response to oxygen levels 35/2475 174/18870 0.00613950017109031 0.0357797715996609 0.0259889185696194 CAV1/MDM2/PLK3/STC1/LMNA/FAS/HMOX1/TREM2/MIR210/KCNMB1/AK4/SCN2A/VEGFA/SLC8A3/MDM4/HILPDA/MYOD1/CHCHD2/AJUBA/KCNK3/SLC2A4/CCNA2/TWIST1/PDK1/BRIP1/ACAA2/PGK1/VASN/PRKCE/PPARG/STOX1/AQP1/SLC29A1/MGARP/MT3 35

BP GO:0060292 long-term synaptic depression 10/2475 32/18870 0.00615002070134078 0.0357797715996609 0.0259889185696194 PRRT1/KCNB1/STXBP1/SHANK2/MAPT/LILRB2/ARC/GRM2/CBLN1/ADORA1 10

BP GO:0061037 negative regulation of cartilage development 10/2475 32/18870 0.00615002070134078 0.0357797715996609 0.0259889185696194 CTSK/EFEMP1/NKX3-2/CCN4/GLI3/FRZB/SNAI2/PTHLH/ADAMTS7/NOG 10

BP GO:0140058 neuron projection arborization 10/2475 32/18870 0.00615002070134078 0.0357797715996609 0.0259889185696194 NTNG2/NRP1/SEMA3A/GRIP1/LRRK2/SULT4A1/PHACTR1/TUBA1A/NTNG1/PTN 10

BP GO:0000768 syncytium formation by plasma membrane fusion 16/2475 63/18870 0.00622453578487264 0.0361432426038446 0.0262529285928203 EHD2/ADGRB3/TREM2/CD53/SBNO2/CXCL10/KCNH1/MYOD1/CXCL9/TYROBP/ADAM9/ITGB1/PTGFRN/CD109/ADAM12/GDF15 16

BP GO:0140253 cell-cell fusion 16/2475 63/18870 0.00622453578487264 0.0361432426038446 0.0262529285928203 EHD2/ADGRB3/TREM2/CD53/SBNO2/CXCL10/KCNH1/MYOD1/CXCL9/TYROBP/ADAM9/ITGB1/PTGFRN/CD109/ADAM12/GDF15 16

BP GO:0030073 insulin secretion 39/2475 199/18870 0.00628349955040159 0.0363936456394049 0.0264348108075414 STX1A/CHGA/VGF/IL1RN/ANXA1/ABCC8/ACVR1C/KCNB1/CNR1/GLUD1/RAB3A/PRKCB/RAPGEF4/NNAT/BRSK2/HLA-DRB1/SYT7/F2RL1/CCL5/GPR27/KCNJ11/SNAP25/NOS2/F2RL2/NKX6-1/VSNL1/GCK/ALOX5/PCLO/TUNAR/TRH/PRKCE/IL6/GAL/TCIRG1/CPLX1/UCP2/PLCB1/RIMS2 39

BP GO:1902075 cellular response to salt 39/2475 199/18870 0.00628349955040159 0.0363936456394049 0.0264348108075414 MDM2/CDK5R1/IQGAP1/ECT2/CLDN1/CHRNB2/KCNB1/NEUROD2/PRKCB/ITPKC/CHRM1/CPNE9/CLIC4/SYT7/KCNH1/DPEP1/COL1A1/SYT1/SYT4/ALOX5AP/DLG2/RASAL1/CCNA2/P2RY1/ADGRV1/HRH3/SLC25A24/NPTX1/SYT5/RYR3/HPCA/B2M/CPNE8/CHRM4/SYT2/TUBA1A/ZC3H12A/SYT13/PLCB1 39

BP GO:0002052 positive regulation of neuroblast proliferation 11/2475 37/18870 0.0062979676581182 0.0363936456394049 0.0264348108075414 HAPLN1/NR2E1/GLI3/VEGFA/DMRTA2/SOX10/OTP/HAPLN3/ITGB1/WDR62/ASPM 11

BP GO:0010934 macrophage cytokine production 11/2475 37/18870 0.0062979676581182 0.0363936456394049 0.0264348108075414 PYCARD/LITAF/CD74/SPON2/ACP5/CD36/LAPTM5/TWIST1/LILRB1/TGFB2/MYD88 11

BP GO:0010935 regulation of macrophage cytokine production 11/2475 37/18870 0.0062979676581182 0.0363936456394049 0.0264348108075414 PYCARD/LITAF/CD74/SPON2/ACP5/CD36/LAPTM5/TWIST1/LILRB1/TGFB2/MYD88 11

BP GO:0050679 positive regulation of epithelial cell proliferation 42/2475 218/18870 0.00640761617055413 0.0369848466630991 0.0268642343218941 MDK/APLN/ITGB3/NKX2-5/CCL26/NEAT1/HMOX1/MMP12/CLDN1/NRP1/FZD7/F3/EGFR/APLNR/HSPG2/AREG/ECM1/DLX5/ITGA4/TNFAIP3/VEGFA/C5AR1/CAV2/VIP/IGF2/HAS2/LAMC1/RUNX2/MIR27B/OSR1/ANG/TWIST1/LAMB1/THBS4/IQGAP3/TBX1/HMGB2/OSR2/EYA1/NOG/PTN/NODAL 42

BP GO:0010212 response to ionizing radiation 29/2475 138/18870 0.00641259583215959 0.0369848466630991 0.0268642343218941 MDM2/THBD/AEN/IL1A/FANCD2/FBXO4/ECT2/SOD2/ANXA1/CHEK2/RAD54L/MAP3K20/NABP1/NET1/BRCA1/CXCL10/IFI16/GPX1/SFRP2/RAD51/BRCA2/CASP3/RAD51AP1/SNAI2/GADD45A/PTPRC/XRCC2/EYA1/IKBIP 29

BP GO:1990778 protein localization to cell periphery 63/2475 353/18870 0.00643877278024079 0.0371001840427694 0.0269480105349405 AKAP5/CAV1/EHD2/CNPY4/EMP2/STAC/KCNIP3/CACNG2/STX1B/ANK1/FGF13/ITGA3/TREM2/LRRC7/VAMP8/KCNB1/STXBP1/FLNA/EGFR/LGALS3/GBP1/C1QL3/RAB3A/S100A10/GRIP1/TNFRSF1A/MMP14/RAB34/PRAM1/GRIN2A/EHD4/KRT18/ABI3/F11R/PID1/TUB/RAB3C/SNAP25/TMBIM1/DPP10/ZDHHC22/P2RY1/LYPLA1/ZDHHC23/VAMP5/MYL12A/NPTX1/PACSIN1/RAP2A/EPHA2/SCN3B/PRKG2/PLK1/ITGB1/EPHA3/PRKCE/ARHGAP44/ANK3/RAMP3/RAB38/CPLX1/GPR158/CACNG3 63

BP GO:0070231 T cell apoptotic process 15/2475 58/18870 0.00664467711714008 0.0382132580568094 0.0277565006012415 IL7R/FAS/BCL3/CHEK2/RIPK3/LGALS3/PTCRA/CD274/GLI3/JAK3/IDO1/CCL5/KDELR1/IL2RA/FADD 15

BP GO:0099024 plasma membrane invagination 15/2475 58/18870 0.00664467711714008 0.0382132580568094 0.0277565006012415 ITGB2/ITGA2/THBS1/TREM2/FCGR1A/F2RL1/FCGR2B/XKR7/CD36/CD300A/AURKB/C3/MSR1/XKR8/MARCO 15

BP GO:0032835 glomerulus development 17/2475 69/18870 0.00677552136409979 0.0389211228360961 0.0282706637522259 ITGB3/WWTR1/IQGAP1/NID1/PROM1/RET/FOXJ1/JAG1/ENPEP/GPR4/ANGPT2/ACTA2/ANGPT1/OSR1/PDGFD/NOG/AQP1 17

BP GO:0051091 positive regulation of DNA-binding transcription factor activity 46/2475 244/18870 0.00678072859536251 0.0389211228360961 0.0282706637522259 CAV1/TRADD/DDIT3/PYCARD/IL18/TRAF5/CD40/TFRC/S100A12/TRIM21/RIPK3/NEUROD2/CLEC7A/TRIM38/TRIM22/BMP2/PRKCB/S100A8/NFAM1/NTS/ROR1/S100A9/RAB7B/TLR2/CARD16/SPHK1/HDAC4/CSF3/CAMK2A/LTF/PPIA/FZD2/CD36/IL1RAP/NKX6-1/TRIM6/TRIM5/FZD1/NTRK1/IL6/PPARG/IRAK2/MYD88/HCLS1/NODAL/TRAF1 46

BP GO:0002223 stimulatory C-type lectin receptor signaling pathway 8/2475 23/18870 0.00682366814905845 0.0389442049115465 0.0282874295993088 KLRC2/KLRC3/KLRC4/CLEC7A/KLRC4-KLRK1/LYN/PAK3/TYROBP 8

BP GO:0021542 dentate gyrus development 8/2475 23/18870 0.00682366814905845 0.0389442049115465 0.0282874295993088 MDK/LEF1/NR2E1/SCN2A/FEZF2/NEUROD6/TUBA1A/CDK6 8

BP GO:0045591 positive regulation of regulatory T cell differentiation 8/2475 23/18870 0.00682366814905845 0.0389442049115465 0.0282874295993088 SOCS1/HLA-DRB1/IL2RG/DUSP10/BTN2A2/LILRB2/HLA-DRA/IL4I1 8

BP GO:0046794 transport of virus 8/2475 23/18870 0.00682366814905845 0.0389442049115465 0.0282874295993088 CAV1/SIGLEC1/KPNA2/CAV2/CTSL/DPP4/JPT2/BST2 8

BP GO:1990840 response to lectin 8/2475 23/18870 0.00682366814905845 0.0389442049115465 0.0282874295993088 KLRC2/KLRC3/KLRC4/CLEC7A/KLRC4-KLRK1/LYN/PAK3/TYROBP 8

BP GO:1990858 cellular response to lectin 8/2475 23/18870 0.00682366814905845 0.0389442049115465 0.0282874295993088 KLRC2/KLRC3/KLRC4/CLEC7A/KLRC4-KLRK1/LYN/PAK3/TYROBP 8

BP GO:0043281 regulation of cysteine-type endopeptidase activity involved in apoptotic process 33/2475 163/18870 0.00690844656502543 0.0393532383077919 0.0285845342244086 NGFR/PYCARD/HIP1R/IFI6/LCK/FAS/THBS1/ST20/ACVR1C/F3/CD44/CLEC7A/MMP9/S100A8/S100A9/CASP1/CASP8/VEGFA/CARD16/GPX1/DPEP1/HGF/RPS6KA1/MAPT/BCL2L12/MIR27B/LAMP3/LAPTM5/SFN/PPARG/PLAUR/AQP1/NODAL 33

BP GO:0050905 neuromuscular process 33/2475 163/18870 0.00690844656502543 0.0393532383077919 0.0285845342244086 KCNA1/MYH7/STAC/JPH4/USH1C/RBFOX1/ABCC8/NEFL/GCH1/TNNI2/FGF12/ATP8A2/CHRNA1/CSMD1/SHANK1/HEXB/GRIN3A/NRXN1/SLC8A3/GRIN2A/PCDH15/HOXD10/TNR/TNNT1/HOXC10/STRA6/CNTNAP2/POU4F1/JPH3/ALDH1A3/TUBA1A/HOXA1/FOXS1 33

BP GO:0021872 forebrain generation of neurons 14/2475 53/18870 0.0070435665048356 0.039933496973591 0.0290060096710242 SLC4A10/SEMA3E/LEF1/NRP1/DLX2/NR2E1/GLI3/FEZF2/LHX5/OTP/TBR1/B2M/LHX6/ASPM 14

BP GO:0032330 regulation of chondrocyte differentiation 14/2475 53/18870 0.0070435665048356 0.039933496973591 0.0290060096710242 SHOX2/MDK/LOXL2/EFEMP1/HOXA11/NKX3-2/CCN4/GLI3/SCIN/RUNX2/PRKG2/SNAI2/PTHLH/ADAMTS7 14

BP GO:0045778 positive regulation of ossification 14/2475 53/18870 0.0070435665048356 0.039933496973591 0.0290060096710242 WNT4/CCN1/BMP2/NELL1/FAM20C/OSR1/TENT5A/LTF/ISG15/ADGRV1/ALOX5/WNT10B/OSR2/PTN 14

BP GO:0071622 regulation of granulocyte chemotaxis 14/2475 53/18870 0.0070435665048356 0.039933496973591 0.0290060096710242 RARRES2/MDK/CXCL8/THBS1/CD74/C5AR1/CCL5/BST1/THBS4/TNFAIP6/RAC2/CXCR2/DPP4/LBP 14

BP GO:0086002 cardiac muscle cell action potential involved in contraction 14/2475 53/18870 0.0070435665048356 0.039933496973591 0.0290060096710242 CAV1/FGF12/CTNNA3/FLNA/DSG2/SCN2B/RYR2/KCNE5/KCNE3/KCNJ3/GJC1/DSC2/SCN3B/KCNE4 14

BP GO:0021987 cerebral cortex development 27/2475 127/18870 0.00710475884161828 0.0402114708211311 0.0292079181619177 KCNA1/CDK5R2/MDK/EMX1/CDK5R1/KIF14/KIF26A/FGF13/NEFL/COL3A1/FLNA/EGFR/TACC3/NR2E1/GLI3/DMRTA2/LAMB1/CNTNAP2/PHACTR1/NDE1/TBR1/WDR62/LHX6/TUBA1A/ASPM/PLCB1/MGARP 27

BP GO:0008277 regulation of G protein-coupled receptor signaling pathway 29/2475 139/18870 0.00711268854297823 0.0402114708211311 0.0292079181619177 TMOD2/RGS7/APLN/ITGB3/NECAB2/CXCL8/RGS3/CHGA/PDE6G/APLNR/MET/PHF24/CCL5/GRM5/GPR27/TUB/CAMK2A/RGS16/SYP/C3/PLEK/FRMPD1/RGS4/RAMP3/ADM/GPR158/GRP/KCTD16/PLCB1 29

BP GO:0048813 dendrite morphogenesis 29/2475 139/18870 0.00711268854297823 0.0402114708211311 0.0292079181619177 IL1RAPL1/CUL7/CDK5R1/DNM3/DCDC2/ADGRB3/CHRNB2/NRP1/SEMA3A/DSCAM/CUX2/NR2E1/SHANK1/ABI3/HECW1/LRRK2/SULT4A1/ARC/PAK3/PPFIA2/LZTS1/RAP2A/PHACTR1/ELAVL4/ITGB1/MAPK8IP2/ARHGAP44/SLITRK5/PTN 29

BP GO:0030048 actin filament-based movement 28/2475 133/18870 0.00711999922442106 0.0402149344717066 0.0292104340117606 CAV1/MYH7/EMP2/STC1/FGF13/MYO5C/FGF12/CTNNA3/FLNA/DSG2/ACTA2/SCN2B/PDPN/RYR2/MYO1B/KCNE5/KCNE3/KCNJ3/GJC1/MYL6/DSC2/SCN3B/MYO5B/MYO1F/GATA4/ADORA1/MYO1G/KCNE4 28

BP GO:0002294 CD4-positive, alpha-beta T cell differentiation involved in immune response 18/2475 75/18870 0.00724548268107055 0.0408085159635531 0.0296415865978822 ASCL2/BATF/IL18/LEF1/NFKBIZ/RELB/LOXL3/ANXA1/BCL3/PTGER4/HLA-DRB1/JAK3/SOCS3/SPN/HLA-DRA/IL6/ZC3H12A/GPR183 18

BP GO:0042246 tissue regeneration 18/2475 75/18870 0.00724548268107055 0.0408085159635531 0.0296415865978822 MDK/LGR6/ANXA1/HOPX/FZD7/CPQ/RUNX1/GAP43/GPX1/SELENON/MYOD1/DUSP10/POSTN/GATA4/WNT10B/PTGFRN/EZH2/PTN 18

BP GO:0061045 negative regulation of wound healing 18/2475 75/18870 0.00724548268107055 0.0408085159635531 0.0296415865978822 THBD/WNT4/MMRN1/PLAT/C1QTNF1/THBS1/SERPINE1/PDGFA/SERPING1/PLAU/PROS1/FAP/ALOX5/AJAP1/CD109/TFPI/PLAUR/ANXA2 18

BP GO:2000241 regulation of reproductive process 40/2475 207/18870 0.00727918368608404 0.0409599052026697 0.0297515493626461 TIMP1/WNT4/ITGB3/SNAI1/IL1A/ARHGDIB/PLAT/MAEL/KIF9/ACVR1C/STXBP1/CNR1/JAG1/OXTR/VEGFA/SYDE1/PKMYT1/VIP/TTK/NPM2/YBX3/LIF/RGN/PLB1/AURKA/GPR3/TRIP13/CDC25C/P2RY1/ZP3/RAD51AP1/PRDX4/CDC25A/LHFPL2/ASPM/PLCB1/HOXD13/NODAL/CDC20/FANCA 40

BP GO:0030225 macrophage differentiation 16/2475 64/18870 0.00731150966723655 0.0411032809382849 0.0298556914561019 SPI1/TSPAN2/ROR2/SOCS1/MMP9/HLA-DRB1/CASP8/VEGFA/TLR2/LIF/FOSL2/C1QC/FADD/MYD88/HCLS1/UCP2 16

BP GO:0030195 negative regulation of blood coagulation 13/2475 48/18870 0.00739846173372969 0.0415143591115076 0.0301542813210449 THBD/PLAT/C1QTNF1/THBS1/SERPINE1/PDGFA/SERPING1/PLAU/PROS1/FAP/TFPI/PLAUR/ANXA2 13

BP GO:0043277 apoptotic cell clearance 13/2475 48/18870 0.00739846173372969 0.0415143591115076 0.0301542813210449 ITGB3/ANXA1/THBS1/TREM2/CCL2/CD300LF/XKR7/CD36/C2/TYROBP/C3/XKR8/MARCO 13

BP GO:0002675 positive regulation of acute inflammatory response 9/2475 28/18870 0.00750713483324441 0.0418892542182151 0.0304265893309838 FCGR1A/CNR1/ALOX5AP/ZP3/OSM/OSMR/C3/IL6/PIK3CG 9

BP GO:0007210 serotonin receptor signaling pathway 9/2475 28/18870 0.00750713483324441 0.0418892542182151 0.0304265893309838 HTR1A/HTR1D/HTR5A/CHRM1/HTR2A/HRH1/HTR2C/HRH3/CHRM4 9

BP GO:0030194 positive regulation of blood coagulation 9/2475 28/18870 0.00750713483324441 0.0418892542182151 0.0304265893309838 THBD/PLAT/THBS1/F3/EMILIN2/SERPINE1/PLAU/CD36/EMILIN1 9

BP GO:0046639 negative regulation of alpha-beta T cell differentiation 9/2475 28/18870 0.00750713483324441 0.0418892542182151 0.0304265893309838 ASCL2/LOXL3/ANXA1/SOCS1/RUNX1/GLI3/JAK3/RUNX3/ZC3H12A 9

BP GO:0090594 inflammatory response to wounding 9/2475 28/18870 0.00750713483324441 0.0418892542182151 0.0304265893309838 TIMP1/MDK/NFKBIZ/IL1A/HMOX1/ALOX5/IL6/PPARG/SIGLEC10 9

BP GO:1900048 positive regulation of hemostasis 9/2475 28/18870 0.00750713483324441 0.0418892542182151 0.0304265893309838 THBD/PLAT/THBS1/F3/EMILIN2/SERPINE1/PLAU/CD36/EMILIN1 9

BP GO:0048588 developmental cell growth 45/2475 239/18870 0.00751812381008693 0.0419116205717381 0.0304428353078142 AKAP6/UNC13A/EMX1/SEMA3E/IQGAP1/SPP1/FGF13/NRP1/RIMS1/SEMA3A/CDH4/SEMA3D/SLIT1/DSCAM/RTN4R/SH3GL2/ITGA4/CPNE9/VEGFA/L1CAM/CXCR4/SEMA6B/FN1/S100B/SYT1/MAPT/SYT4/RASAL1/FLRT1/AURKA/TNR/SEMA3F/NKX6-1/DRAXIN/FSTL4/POSTN/GATA4/ITGB1/TRPV2/SYT2/RGS4/MEIS1/ISLR2/MT3/RIMS2 45

BP GO:1905477 positive regulation of protein localization to membrane 23/2475 104/18870 0.00762221746611177 0.0424524987630196 0.0308357064369701 AKAP5/CNPY4/CDK5R1/NECAB2/STAC/ITGB2/CACNG2/ITGA3/TREM2/KCNB1/EGFR/GZMB/LGALS3/TCAF2/DPP10/TNFAIP6/EPHA2/HPCA/ITGB1/EPHA3/PRKCE/ANK3/RAMP3 23

BP GO:0010712 regulation of collagen metabolic process 12/2475 43/18870 0.00767709080400008 0.0427006791787058 0.031015974234261 WNT4/ITGA2/RUNX1/MFAP4/CIITA/ENG/CST3/EMILIN1/VIM/FAP/ITGB1/IL6 12

BP GO:0036294 cellular response to decreased oxygen levels 32/2475 158/18870 0.00768100158444408 0.0427006791787058 0.031015974234261 MDM2/PLK3/STC1/LMNA/HMOX1/TREM2/MIR210/KCNMB1/AK4/SCN2A/VEGFA/SLC8A3/MDM4/HILPDA/CHCHD2/AJUBA/KCNK3/SLC2A4/CCNA2/TWIST1/PDK1/BRIP1/ACAA2/PGK1/VASN/PRKCE/PPARG/STOX1/AQP1/SLC29A1/MGARP/MT3 32

BP GO:0042770 signal transduction in response to DNA damage 37/2475 189/18870 0.00775846092995444 0.0430913963214121 0.0312997747045146 SP100/MDM2/BATF/SNAI1/PARP9/PLK3/FANCD2/FBXO4/E2F7/SMYD2/BCL3/CHEK2/WDR76/CD74/CD44/MAP3K20/DTL/FOXM1/BRCA1/MDM4/MUC1/DTX3L/RAD51/CDK2/TWIST1/BRCA2/CLSPN/E2F1/BRIP1/SNAI2/PLK1/GADD45A/CDK1/GTSE1/DYRK3/CHEK1/EME1 37

BP GO:0036336 dendritic cell migration 10/2475 33/18870 0.00780618704629306 0.0430941978720137 0.031301809632833 SPI1/SLAMF8/CCL5/CXCR4/CCR5/CXCR2/ALOX5/PIK3CG/CCR1/GPR183 10

BP GO:0050869 negative regulation of B cell activation 10/2475 33/18870 0.00780618704629306 0.0430941978720137 0.031301809632833 PAWR/FOXJ1/TNFAIP3/SAMSN1/FCGR2B/LYN/CD300A/LAPTM5/TYROBP/CASP3 10

BP GO:1990806 ligand-gated ion channel signaling pathway 10/2475 33/18870 0.00780618704629306 0.0430941978720137 0.031301809632833 CDK5R1/GRIA4/GRIA2/GRIN3A/GRIN2A/GRIN1/F2RL2/GRIK1/GRIN2B/PLCB1 10

BP GO:1902749 regulation of cell cycle G2/M phase transition 25/2475 116/18870 0.00783267127039702 0.0430941978720137 0.031301809632833 CENPF/KIF14/RBBP8/CCNB1/DTL/NABP1/ATF5/ORC1/BRCA1/PKMYT1/CDK2/AURKA/CDC25C/AURKB/CLSPN/PLK1/CDC6/CDK4/CDK1/WNT10B/CDC25A/STOX1/DYRK3/CHEK1/CDK6 25

BP GO:0002369 T cell cytokine production 11/2475 38/18870 0.00783405990904867 0.0430941978720137 0.031301809632833 IL18/IL1R1/ARID5A/SASH3/FZD5/HLA-A/RSAD2/B2M/IL6/HFE/HLA-F 11

BP GO:0002724 regulation of T cell cytokine production 11/2475 38/18870 0.00783405990904867 0.0430941978720137 0.031301809632833 IL18/IL1R1/ARID5A/SASH3/FZD5/HLA-A/RSAD2/B2M/IL6/HFE/HLA-F 11

BP GO:0071392 cellular response to estradiol stimulus 11/2475 38/18870 0.00783405990904867 0.0430941978720137 0.031301809632833 SSTR3/SSTR2/ITGA2/MMP2/EGFR/MYOD1/CCNA2/KIF18A/POU4F1/SSTR1/RAMP3 11

BP GO:0002823 negative regulation of adaptive immune response based on somatic recombination of immune receptors built from immunoglobulin superfamily domains 15/2475 59/18870 0.00785368739679135 0.0430941978720137 0.031301809632833 ASCL2/IL7R/LOXL3/FOXJ1/HAVCR2/AHR/JAK3/FCGR2B/SPN/LILRB1/PTPRC/ZC3H12A/HFE/IL4I1/HLA-F 15

BP GO:0071168 protein localization to chromatin 15/2475 59/18870 0.00785368739679135 0.0430941978720137 0.031301809632833 MACROH2A2/H4C9/ESCO2/LEF1/SPI1/CENPA/H4C5/H1-5/H2AC8/H2BC11/H4C8/H4C11/PLK1/H4C3/EZH2 15

BP GO:0098900 regulation of action potential 15/2475 59/18870 0.00785368739679135 0.0430941978720137 0.031301809632833 CAV1/FGF13/CHRNB2/FGF12/KCNB1/CTNNA3/FLNA/CNR1/DSG2/KCNAB2/RYR2/SLC8A2/KCNE3/CD36/DSC2 15

BP GO:0002523 leukocyte migration involved in inflammatory response 7/2475 19/18870 0.00790885268015657 0.0430941978720137 0.031301809632833 MDK/S100A8/S100A9/SLAMF8/ALOX5/LBP/PTN 7

BP GO:0016553 base conversion or substitution editing 7/2475 19/18870 0.00790885268015657 0.0430941978720137 0.031301809632833 APOBEC3B/RBM47/APOBEC3G/APOBEC3C/APOBEC3F/ADARB2/APOBEC3H 7

BP GO:0032928 regulation of superoxide anion generation 7/2475 19/18870 0.00790885268015657 0.0430941978720137 0.031301809632833 ITGB2/CLEC7A/F2RL1/ACP5/FPR2/MAPT/TYROBP 7

BP GO:0035821 modulation of process of another organism 7/2475 19/18870 0.00790885268015657 0.0430941978720137 0.031301809632833 CLEC7A/S100A9/F2RL1/SLPI/NOS2/PRF1/CXCL6 7

BP GO:0043031 negative regulation of macrophage activation 7/2475 19/18870 0.00790885268015657 0.0430941978720137 0.031301809632833 TAFA3/FCGR2B/VSIG4/CST7/LRFN5/PTPRC/ZC3H12A 7

BP GO:0045091 regulation of single stranded viral RNA replication via double stranded DNA intermediate 7/2475 19/18870 0.00790885268015657 0.0430941978720137 0.031301809632833 APOBEC3B/TOP2A/CXCL8/APOBEC3G/APOBEC3C/APOBEC3F/APOBEC3H 7

BP GO:0051797 regulation of hair follicle development 7/2475 19/18870 0.00790885268015657 0.0430941978720137 0.031301809632833 TRADD/FERMT1/NGFR/FST/WNT10B/TGFB2/GAL 7

BP GO:0098543 detection of other organism 7/2475 19/18870 0.00790885268015657 0.0430941978720137 0.031301809632833 TLR1/SSC5D/CLEC7A/HLA-DRB1/TLR2/HLA-B/HLA-A 7

BP GO:1902259 regulation of delayed rectifier potassium channel activity 7/2475 19/18870 0.00790885268015657 0.0430941978720137 0.031301809632833 AKAP6/KCNAB1/KCNS2/KCNE5/KCNE3/ANK3/KCNE4 7

BP GO:1903817 negative regulation of voltage-gated potassium channel activity 7/2475 19/18870 0.00790885268015657 0.0430941978720137 0.031301809632833 CAV1/KCNAB1/KCNE5/KCNE3/ANK3/KCNE4/GRP 7

BP GO:0046578 regulation of Ras protein signal transduction 36/2475 183/18870 0.0079096945461291 0.0430941978720137 0.031301809632833 LPAR6/GPR65/KIF14/ARHGDIB/IQSEC3/SPRY2/ITGA3/NRP1/COL3A1/RTN4R/GPR4/OPHN1/MET/NET1/SPRY4/FOXM1/F2RL1/SYDE1/F11R/RASGRF1/CCDC125/RASAL1/ARFGEF3/F2RL3/F2RL2/TRIM67/FBP1/MMD2/ITGB1/NTRK1/ARHGAP44/PIK3CG/CYTH4/TGFB2/SPRY1/PSD 36

BP GO:0048708 astrocyte differentiation 20/2475 87/18870 0.00796344849700243 0.043347728718044 0.0314859637572945 TSPAN2/ROR2/TREM2/BMP2/SOX8/NR2E1/GAP43/LAMC3/S100A8/ROR1/S100A9/C5AR1/MAPT/LIF/VIM/IL6/C1QA/NOG/CLCF1/CDK6 20

BP GO:0051235 maintenance of location 62/2475 350/18870 0.00803241949038638 0.0436835567212679 0.0317298950692894 CAV1/AKAP6/CES1/SP100/FTL/DDIT3/ITGB3/FTH1/TMSB4X/TSPO/JPH4/LCK/CYBA/TREM2/ACVR1C/KDELR3/FLNA/APLNR/PLIN2/MYZAP/P2RY6/S100A8/S100A9/HEXB/SCIN/LTBP1/CXCL10/SELENON/HTR2A/SLC30A7/ENPP1/RYR2/HILPDA/LYN/SRGN/HTR2C/HK2/OS9/CXCL9/KDELR2/CD36/F2RL3/CCR5/KDELR1/NPC2/CRY2/RYR3/MCOLN2/JPH3/C3/PTPRC/MSR1/TMSB10/PRKCE/CXCL11/IL6/PPARG/FKBP1C/ZC3H12A/ANK3/ASPM/PLCB1 62

BP GO:0001764 neuron migration 35/2475 177/18870 0.00805473020677118 0.0437652490148906 0.0317892329094098 CDK5R2/MDK/CDK5R1/SEMA3E/DCDC2/KIF26A/FGF13/ITGA3/NTNG2/NRP1/SEMA3A/NRG3/COL3A1/FLNA/SCRT1/FEZF2/SEPTIN14/VEGFA/CXCR4/NEUROD4/CAMK2A/TWIST1/NR2F2/CCK/ULK4/NKX6-1/PHACTR1/NDE1/POU4F1/WDR62/LHX6/CELSR1/TUBA1A/ASPM/NTNG1 35

BP GO:0032956 regulation of actin cytoskeleton organization 60/2475 337/18870 0.00809153174907476 0.0439254580664058 0.0319056020142921 WNT4/TMOD2/ITGB3/GPR65/PYCARD/HIP1R/CDK5R1/TMSB4X/IL1A/CCL26/CAPG/ECT2/VASP/DNAI3/IQGAP2/NRP1/FLNA/FHOD1/AVIL/COTL1/S100A10/SHANK1/SSH3/ARHGAP18/MET/PTGER4/ARPC5/SCIN/FCHSD1/F2RL1/CDC42EP5/BST1/F11R/CSF3/PDGFA/TACSTD2/PAK3/SDC4/TMEFF2/CAPZA1/GMFG/ARHGEF19/ELN/PEAK3/SPTBN2/RHOC/SPTB/DAAM2/PLEK/TMSB10/CARMIL2/EPHA3/PRKCE/ARHGAP44/CELSR1/RGS4/SWAP70/HCLS1/PFN1/BST2 60

BP GO:0043279 response to alkaloid 21/2475 93/18870 0.00822384257006952 0.0446033882481458 0.0323980219348624 MDM2/PRKCG/CHRNB2/CNR1/CASP7/OXTR/SELENON/HTR2A/RYR2/CASP6/RAD51/CCNA2/GRM2/CASP3/RYR3/ELAVL4/PRKCE/RGS4/FADD/EN1/CHEK1 21

BP GO:0002287 alpha-beta T cell activation involved in immune response 18/2475 76/18870 0.00835786679231213 0.0452077767757135 0.0328370242963956 ASCL2/BATF/IL18/LEF1/NFKBIZ/RELB/LOXL3/ANXA1/BCL3/PTGER4/HLA-DRB1/JAK3/SOCS3/SPN/HLA-DRA/IL6/ZC3H12A/GPR183 18

BP GO:0002293 alpha-beta T cell differentiation involved in immune response 18/2475 76/18870 0.00835786679231213 0.0452077767757135 0.0328370242963956 ASCL2/BATF/IL18/LEF1/NFKBIZ/RELB/LOXL3/ANXA1/BCL3/PTGER4/HLA-DRB1/JAK3/SOCS3/SPN/HLA-DRA/IL6/ZC3H12A/GPR183 18

BP GO:0086003 cardiac muscle cell contraction 18/2475 76/18870 0.00835786679231213 0.0452077767757135 0.0328370242963956 CAV1/STC1/FGF13/FGF12/CTNNA3/FLNA/DSG2/SCN2B/RYR2/KCNE5/KCNE3/KCNJ3/GJC1/DSC2/SCN3B/GATA4/ADORA1/KCNE4 18

BP GO:0098869 cellular oxidant detoxification 22/2475 99/18870 0.00842696760181678 0.0455405161847957 0.0330787121837386 PXDNL/H19/IPCEF1/SOD2/HBQ1/GCH1/S100A9/PTGS1/GPX1/HP/HBA1/ALOX5AP/SOD3/PTGES/GPX7/MGST2/CD36/FBLN5/SELENOF/PRDX4/GPX8/MT3 22

BP GO:0033044 regulation of chromosome organization 46/2475 247/18870 0.00848537550925104 0.0458149231632583 0.0332780298512184 NEK2/CENPF/SPC24/MAD2L1/BCL7A/TOP2A/FBXO4/CENPV/ACTL6B/KNTC1/UBE2C/PIF1/TACC3/MAP3K20/CCNB1/RMI2/NEK6/PKIB/DLGAP5/BIRC5/HASPIN/ESPL1/TTK/ZWILCH/MAPT/NUF2/CDK2/EID3/TRIP13/BUB1B/H3-3A/BUB1/AURKB/NCAPG/SMC4/PLK1/CDC6/CENPE/CDCA8/NDC80/CDK1/NCAPH/CCT6A/KNL1/ZWINT/CDC20 46

BP GO:0010812 negative regulation of cell-substrate adhesion 16/2475 65/18870 0.00854559341766809 0.0460572198201788 0.0334540239340441 SEMA3E/MMP12/THBS1/FZD7/GBP1/FBLN1/JAG1/ANGPT2/SPRY4/MMP14/COL1A1/SERPINE1/TACSTD2/HOXA7/POSTN/AJAP1 16

BP GO:1905330 regulation of morphogenesis of an epithelium 16/2475 65/18870 0.00854559341766809 0.0460572198201788 0.0334540239340441 WNT4/MDK/SOX8/VEGFA/CXCL10/HGF/PDGFA/LIF/TACSTD2/NKD1/SNAI2/GDNF/STOX1/NOG/HOXD13/HOXB7 16

BP GO:0070252 actin-mediated cell contraction 23/2475 105/18870 0.00857889793705391 0.0461952495193468 0.033554282891871 CAV1/MYH7/EMP2/STC1/FGF13/FGF12/CTNNA3/FLNA/DSG2/ACTA2/SCN2B/PDPN/RYR2/KCNE5/KCNE3/KCNJ3/GJC1/MYL6/DSC2/SCN3B/GATA4/ADORA1/KCNE4 23

BP GO:0071347 cellular response to interleukin-1 24/2475 111/18870 0.00868532736719988 0.046726438631423 0.0339401162821252 PYCARD/CCL26/CXCL8/CCL20/GBP2/IL1RN/VRK2/CD40/MMP2/IL1R1/GBP1/CCL2/CCL5/HAS2/IL1R2/GBP3/CCL18/IL1RAP/IL6/ADAMTS7/ZC3H12A/IRAK2/MYD88/PLCB1 24

BP GO:0051258 protein polymerization 52/2475 286/18870 0.00870611356693213 0.0467290300135749 0.0339419985529368 TMOD2/PSTPIP1/DIAPH3/PYCARD/HIP1R/CDK5R1/TMSB4X/CCL26/CAPG/FGF13/PSTPIP2/FHDC1/VASP/NEFL/PSRC1/UBE2C/AVIL/COTL1/UBE2S/HAUS1/SSH3/ANKRD53/ARHGAP18/MET/ARPC5/SCIN/FCHSD1/CAMSAP3/CDC42EP5/FES/MAPT/CSF3/ANG/PAK3/CAPZA1/TRIM6/ELN/SPTBN2/NDE1/SPTB/DAAM2/STMN2/RNF135/TMSB10/CARMIL2/PRKCE/TPPP3/TPX2/MICALL2/TUBA1A/HCLS1/PFN1 52

BP GO:0007260 tyrosine phosphorylation of STAT protein 19/2475 82/18870 0.00875054393956977 0.0467290300135749 0.0339419985529368 CAV1/IL18/MIR221/PARP9/CD40/HPX/SOCS1/TNFRSF1A/CTF1/JAK3/CCL5/TNFRSF18/LYN/SOCS3/LIF/OSM/IL6/HCLS1/CLCF1 19

BP GO:0014032 neural crest cell development 19/2475 82/18870 0.00875054393956977 0.0467290300135749 0.0339419985529368 EFNB1/HAND2/SEMA3E/RET/NRP1/SEMA3A/SEMA3D/SOX8/JAG1/FOLR1/SEMA6B/FN1/SOX10/TWIST1/SEMA3F/SNAI2/TBX1/RDH10/GDNF 19

BP GO:0010763 positive regulation of fibroblast migration 6/2475 15/18870 0.00879477080535304 0.0467290300135749 0.0339419985529368 ITGB3/THBS1/ACTA2/ITGB1/PRKCE/AQP1 6

BP GO:0032230 positive regulation of synaptic transmission, GABAergic 6/2475 15/18870 0.00879477080535304 0.0467290300135749 0.0339419985529368 CA2/OXTR/NALCN/ZDHHC12/PRKCE/CA7 6

BP GO:0034501 protein localization to kinetochore 6/2475 15/18870 0.00879477080535304 0.0467290300135749 0.0339419985529368 KNTC1/TTK/ZWILCH/AURKB/CDK1/KNL1 6

BP GO:0038065 collagen-activated signaling pathway 6/2475 15/18870 0.00879477080535304 0.0467290300135749 0.0339419985529368 ITGA2/ITGA11/OSCAR/COL1A1/COL4A1/COL4A2 6

BP GO:0043374 CD8-positive, alpha-beta T cell differentiation 6/2475 15/18870 0.00879477080535304 0.0467290300135749 0.0339419985529368 SOCS1/TNFSF8/RUNX1/TOX/RUNX3/IRF1 6

BP GO:0050966 detection of mechanical stimulus involved in sensory perception of pain 6/2475 15/18870 0.00879477080535304 0.0467290300135749 0.0339419985529368 KCNA1/ITGA2/PHF24/HTR2A/CXCR4/NTRK1 6

BP GO:0060099 regulation of phagocytosis, engulfment 6/2475 15/18870 0.00879477080535304 0.0467290300135749 0.0339419985529368 ITGA2/TREM2/F2RL1/CD36/CD300A/C3 6

BP GO:1902563 regulation of neutrophil activation 6/2475 15/18870 0.00879477080535304 0.0467290300135749 0.0339419985529368 PLA2G2A/SPI1/ITGB2/PRAM1/FCGR2B/CD300A 6

BP GO:1903054 negative regulation of extracellular matrix organization 6/2475 15/18870 0.00879477080535304 0.0467290300135749 0.0339419985529368 TNFRSF1A/MIR27B/CST3/EMILIN1/FAP/DPP4 6

BP GO:1903083 protein localization to condensed chromosome 6/2475 15/18870 0.00879477080535304 0.0467290300135749 0.0339419985529368 KNTC1/TTK/ZWILCH/AURKB/CDK1/KNL1 6

BP GO:1905153 regulation of membrane invagination 6/2475 15/18870 0.00879477080535304 0.0467290300135749 0.0339419985529368 ITGA2/TREM2/F2RL1/CD36/CD300A/C3 6

BP GO:0097305 response to alcohol 47/2475 254/18870 0.00885806539632355 0.0467908105557678 0.0339868733357667 CLDN7/CES1/MDM2/RGS7/FOSL1/TSPO/CTSK/ACE/SOD2/GABBR1/NEFL/CLDN1/CHRNB2/KCNMB1/AHR/CYP1B1/P2RY6/CNR1/PRKCB/S100A8/TYMS/IGFBP7/PTGER4/GRIN3A/CASP8/BRCA1/GRAMD1B/GRIN2A/SCNN1B/SLC2A4/CSF3/GRIN1/RAD51/CD14/TNC/ADCYAP1R1/CCR5/KCNC2/FBP1/CDK4/PTPRC/CDK1/PRKCE/RGS4/MYD88/GRIN2B/MAOB 47

BP GO:0002673 regulation of acute inflammatory response 13/2475 49/18870 0.00888775747183054 0.0467908105557678 0.0339868733357667 FCGR1A/CNR1/FCGR2B/ALOX5AP/PTGES/SPN/ZP3/OSM/OSMR/C3/ADORA1/IL6/PIK3CG 13

BP GO:0046596 regulation of viral entry into host cell 13/2475 49/18870 0.00888775747183054 0.0467908105557678 0.0339868733357667 CD74/TRIM21/TRIM38/TRIM22/CIITA/IFITM3/HLA-DRB1/IFITM2/NECTIN2/LGALS1/TRIM6/TRIM5/PTX3 13

BP GO:0048286 lung alveolus development 13/2475 49/18870 0.00888775747183054 0.0467908105557678 0.0339868733357667 HOXA5/ACE/MMP12/HOPX/STK40/IGFBP5/ERRFI1/SMPD3/SELENON/PDGFA/LIF/STRA6/FOSL2 13

BP GO:1900047 negative regulation of hemostasis 13/2475 49/18870 0.00888775747183054 0.0467908105557678 0.0339868733357667 THBD/PLAT/C1QTNF1/THBS1/SERPINE1/PDGFA/SERPING1/PLAU/PROS1/FAP/TFPI/PLAUR/ANXA2 13

BP GO:2000404 regulation of T cell migration 13/2475 49/18870 0.00888775747183054 0.0467908105557678 0.0339868733357667 ASCL2/ITGB3/PYCARD/CXCL13/CCL20/RIPK3/ECM1/ITGA4/CXCL10/CCL5/SPN/FADD/TNFRSF14 13

BP GO:2001259 positive regulation of cation channel activity 13/2475 49/18870 0.00888775747183054 0.0467908105557678 0.0339868733357667 AKAP6/TMSB4X/STAC/FGF13/TREM2/NIPSNAP2/P2RY6/PIRT/LRRC55/ANK3/GAL/CTSS/KCNIP2 13

BP GO:0097193 intrinsic apoptotic signaling pathway 57/2475 319/18870 0.0089698881398476 0.0467908105557678 0.0339868733357667 CAV1/MDM2/DDIT3/PPP1R15A/PYCARD/DDIAS/SNAI1/AEN/IFI6/LCK/MAEL/SOD2/MELK/TREM2/BCL3/CHEK2/CD74/CD44/RIPK3/HSPB1/CYP1B1/MMP9/BCL2A1/S100A8/S100A9/TNFRSF1A/SCN2A/BRSK2/BRCA1/IFI16/GPX1/FCGR2B/SFRP2/MUC1/CASP6/MIR27B/TRIB3/LRRK2/YBX3/IL20RA/CEBPB/PPIA/BRCA2/PDK1/BDKRB2/SFN/E2F2/CASP3/E2F1/EPHA2/SNAI2/POU4F1/GATA4/FZD1/PLAUR/CASP4/TP73 57

BP GO:0045861 negative regulation of proteolysis 44/2475 235/18870 0.0089884686338145 0.0467908105557678 0.0339868733357667 TIMP1/MDM2/IFI6/PLAT/PRKCG/SERPINB1/THBS1/SERPINH1/TRIM21/CD44/MMP9/ECM1/OPHN1/SERPINA5/VEGFA/PBK/CARD16/IFI16/GPX1/SERPINB8/DPEP1/HGF/IL1R2/RPS6KA1/BCL2L12/CST3/LRRK2/SERPINE1/SERPING1/PLAU/LTF/LAMP3/CRB2/SFN/CST7/SERPINA1/CSTA/GAS1/CTSZ/PLAUR/SERPINA3/HFE/SERPINI1/AQP1 44

BP GO:0001765 membrane raft assembly 5/2475 11/18870 0.00899343693893339 0.0467908105557678 0.0339868733357667 CAV1/EMP2/S100A10/CAV2/ANXA2 5

BP GO:0002604 regulation of dendritic cell antigen processing and presentation 5/2475 11/18870 0.00899343693893339 0.0467908105557678 0.0339868733357667 SLC11A1/THBS1/CD74/FCGR2B/FGL2 5

BP GO:0007614 short-term memory 5/2475 11/18870 0.00899343693893339 0.0467908105557678 0.0339868733357667 MDK/CUX2/BRINP1/SLC2A4/RCAN1 5

BP GO:0019065 receptor-mediated endocytosis of virus by host cell 5/2475 11/18870 0.00899343693893339 0.0467908105557678 0.0339868733357667 CAV1/SIGLEC1/CAV2/CTSL/DPP4 5

BP GO:0031340 positive regulation of vesicle fusion 5/2475 11/18870 0.00899343693893339 0.0467908105557678 0.0339868733357667 ANXA1/DOC2A/ERC2/SYT1/ANXA2 5

BP GO:0031953 negative regulation of protein autophosphorylation 5/2475 11/18870 0.00899343693893339 0.0467908105557678 0.0339868733357667 CAV1/ERRFI1/ENG/ENPP1/PTPRC 5

BP GO:0032493 response to bacterial lipoprotein 5/2475 11/18870 0.00899343693893339 0.0467908105557678 0.0339868733357667 TLR1/SSC5D/TLR2/CD14/CD36 5

BP GO:0043320 natural killer cell degranulation 5/2475 11/18870 0.00899343693893339 0.0467908105557678 0.0339868733357667 KLRC2/NKG7/RAB27A/FCGR3A/HLA-F 5

BP GO:0046940 nucleoside monophosphate phosphorylation 5/2475 11/18870 0.00899343693893339 0.0467908105557678 0.0339868733357667 CMPK2/AK4/AK2/DTYMK/AK5 5

BP GO:0051256 mitotic spindle midzone assembly 5/2475 11/18870 0.00899343693893339 0.0467908105557678 0.0339868733357667 KIF4A/KIF23/BIRC5/AURKB/CDCA8 5

BP GO:0051709 regulation of killing of cells of another organism 5/2475 11/18870 0.00899343693893339 0.0467908105557678 0.0339868733357667 CLEC7A/F2RL1/NOS2/PRF1/CXCL6 5

BP GO:0070669 response to interleukin-2 5/2475 11/18870 0.00899343693893339 0.0467908105557678 0.0339868733357667 CITED1/JAK3/IL2RG/IL2RA/IL2RB 5

BP GO:0070944 neutrophil-mediated killing of bacterium 5/2475 11/18870 0.00899343693893339 0.0467908105557678 0.0339868733357667 F2RL1/SCNN1B/CXCL6/MYD88/TREM1 5

BP GO:1902260 negative regulation of delayed rectifier potassium channel activity 5/2475 11/18870 0.00899343693893339 0.0467908105557678 0.0339868733357667 KCNAB1/KCNE5/KCNE3/ANK3/KCNE4 5

BP GO:1905809 negative regulation of synapse organization 5/2475 11/18870 0.00899343693893339 0.0467908105557678 0.0339868733357667 SLIT1/NEUROD2/TLR2/DKK1/CBLN1 5

BP GO:0032147 activation of protein kinase activity 20/2475 88/18870 0.00906652626073464 0.0469498195317624 0.0341023707563539 ITGB3/IL18/EMP2/SLC11A1/KIF14/ECT2/RIPK3/TOM1L1/ANGPT1/VEGFA/CCL5/CHI3L1/ANG/PPIA/CD300A/CLSPN/TPX2/TGFB2/ADRA2B/MT3 20

BP GO:0048864 stem cell development 20/2475 88/18870 0.00906652626073464 0.0469498195317624 0.0341023707563539 EFNB1/HAND2/SEMA3E/RET/NRP1/SEMA3A/SEMA3D/SOX8/JAG1/FOLR1/SEMA6B/FN1/SOX10/TWIST1/SEMA3F/SNAI2/TBX1/PTPRC/RDH10/GDNF 20

BP GO:0099072 regulation of postsynaptic membrane neurotransmitter receptor levels 20/2475 88/18870 0.00906652626073464 0.0469498195317624 0.0341023707563539 ITGB3/DNM3/CACNG2/STX1B/LRRC7/C1QL3/OPHN1/GRIP1/FRRS1L/SNAP25/ARC/NPTX1/RAP2A/EFNB2/HPCA/ARHGAP44/CALY/CPLX1/CACNG5/CACNG3 20

BP GO:0034138 toll-like receptor 3 signaling pathway 8/2475 24/18870 0.00907873758767091 0.0469498195317624 0.0341023707563539 CAV1/HAVCR2/OAS1/TNFAIP3/F2RL1/SCIMP/UNC93B1/COLEC12 8

BP GO:0035235 ionotropic glutamate receptor signaling pathway 8/2475 24/18870 0.00907873758767091 0.0469498195317624 0.0341023707563539 CDK5R1/GRIA4/GRIA2/GRIN3A/GRIN2A/GRIN1/GRIK1/GRIN2B 8

BP GO:0040037 negative regulation of fibroblast growth factor receptor signaling pathway 8/2475 24/18870 0.00907873758767091 0.0469498195317624 0.0341023707563539 WNT4/NGFR/APLN/SPRY2/THBS1/GPC1/SPRY4/SPRY1 8

BP GO:0060143 positive regulation of syncytium formation by plasma membrane fusion 8/2475 24/18870 0.00907873758767091 0.0469498195317624 0.0341023707563539 EHD2/TREM2/CD53/MYOD1/CXCL9/TYROBP/ADAM9/GDF15 8

BP GO:1903317 regulation of protein maturation 17/2475 71/18870 0.00912587877200916 0.0471529915207771 0.0342499463288649 MDM2/PLAT/THBS1/RUNX1/S100A10/MMP14/CARD16/IL1R2/BCL2L12/LRRK2/SERPINE1/PLAU/CST7/GAS1/CTSZ/PLAUR/ANXA2 17

BP GO:0071560 cellular response to transforming growth factor beta stimulus 52/2475 287/18870 0.00931215381203775 0.0480740941422826 0.0349189964640199 CAV1/PDE2A/WNT4/FERMT1/TGFB1I1/FMOD/NFKBIZ/SPI1/NPNT/CTSK/LRRC32/ZYX/TET1/SPRY2/THBS1/ITGA3/CLDN1/COL3A1/CITED1/TWSG1/BMP2/LOX/ENG/ACTA2/LTBP1/SLC2A10/FOLR1/CAV2/COL1A1/MIR27B/COL4A2/COL1A2/WFIKKN2/EMILIN1/LTBP2/VEPH1/PDGFD/SPRED3/ADAM9/GDF10/VASN/POSTN/ASPN/CILP/SMAD9/HTRA3/PPARG/CD109/TGFB2/SPRY1/LATS2/GDF15 52

BP GO:0046006 regulation of activated T cell proliferation 12/2475 44/18870 0.00932360760921137 0.0480918729258634 0.034931910223334 PYCARD/IL18/LRRC32/RIPK3/CD274/PDCD1LG2/IGF2/BTN2A2/IGFBP2/CASP3/IL2RA/FADD 12

BP GO:0034446 substrate adhesion-dependent cell spreading 22/2475 100/18870 0.00949948489310488 0.0489570019727053 0.0355603035122056 MDK/ITGB3/KIF14/BVES/NRP1/FZD7/FLNA/GBP1/S100A10/FBLN1/ITGA4/SPRY4/PDPN/HAS2/LAMC1/FN1/TACSTD2/LAMB1/POSTN/PARVG/MICALL2/FERMT3 22

BP GO:0034599 cellular response to oxidative stress 47/2475 255/18870 0.009514436729883 0.0489920052540459 0.0355857284209671 MDM2/H19/NCF1/FANCD2/ECT2/SOD2/ANXA1/HMOX1/VRK2/PAWR/GCH1/MMP2/EGFR/RIPK3/WNT16/CYP1B1/MMP9/MET/NET1/TNFAIP3/SMPD3/GPX1/SELENON/SPHK1/HGF/CHCHD2/DHRS2/GJB2/MAPT/SOD3/LRRK2/GPX7/PPIA/CD36/PCNA/PDGFD/SLC25A24/FBLN5/ALOX5/CDK1/IL6/CRYGD/ZC3H12A/GPX8/AQP1/EZH2/MT3 47

BP GO:0015718 monocarboxylic acid transport 35/2475 179/18870 0.0095886945090251 0.0492470569000724 0.0357709871904695 CES1/SLC27A3/PLA2G2A/IL1A/SLC16A10/SLC16A4/SLC10A4/SLC22A6/ACE/CROT/ANXA1/EMB/THBS1/ATP8B1/PLIN2/PLA2G5/FABP7/ABCB4/SLC43A3/PTGES/SLCO1A2/SLC16A3/NOS2/CD36/BDKRB2/LYPLA1/FABP5/RBP1/SLC16A11/ERFE/AQP9/PPARG/ABCC3/PLA2G4A/NMUR2 35

BP GO:0070373 negative regulation of ERK1 and ERK2 cascade 18/2475 77/18870 0.00960344053395601 0.0492470569000724 0.0357709871904695 MIR221/DUSP6/SPRY2/DUSP4/DUSP26/GBP1/FBLN1/WNK2/PTPRR/DUSP10/LYN/BTN2A2/LIF/EMILIN1/SPRED3/PTPRC/SPRY1/ATF3 18

BP GO:0002532 production of molecular mediator involved in inflammatory response 23/2475 106/18870 0.00963101334985486 0.0492470569000724 0.0357709871904695 GBP5/PYCARD/H19/NCF1/MIR221/TREM2/VAMP8/CLEC7A/IL17RC/SLAMF8/DUSP10/IL1R2/LYN/ALOX5AP/SERPINE1/NOS2/CD36/ALOX5/IL6/LBP/ZC3H12A/MYD88/EZH2 23

BP GO:0032526 response to retinoic acid 23/2475 106/18870 0.00963101334985486 0.0492470569000724 0.0357709871904695 HAND2/HOXA2/TFRC/RET/FZD7/MMP2/TEAD2/IGFBP7/MICB/BRINP1/DKK1/COL1A1/LYN/GJB2/OSR1/TNC/IGFBP2/TBX1/LRAT/WNT10B/EPHA3/AQP1/WNT7B 23

BP GO:0002710 negative regulation of T cell mediated immunity 9/2475 29/18870 0.00964266351754956 0.0492470569000724 0.0357709871904695 IL7R/AHR/FCGR2B/SPN/LILRB1/PTPRC/HFE/IL4I1/HLA-F 9

BP GO:0003171 atrioventricular valve development 9/2475 29/18870 0.00964266351754956 0.0492470569000724 0.0357709871904695 MDM2/APLNR/CCN1/BMP2/MDM4/TBX5/TWIST1/GATA4/TGFB2 9

BP GO:0031342 negative regulation of cell killing 9/2475 29/18870 0.00964266351754956 0.0492470569000724 0.0357709871904695 IL7R/HAVCR2/MICA/FCGR2B/HLA-B/LILRB1/HLA-A/PTPRC/HLA-F 9

BP GO:0034656 nucleobase-containing small molecule catabolic process 9/2475 29/18870 0.00964266351754956 0.0492470569000724 0.0357709871904695 APOBEC3B/DPYD/APOBEC3G/ADA2/APOBEC3C/UPP1/GDA/APOBEC3F/APOBEC3H 9

BP GO:0099068 postsynapse assembly 11/2475 39/18870 0.0096459924907537 0.0492470569000724 0.0357709871904695 NTNG2/C1QL3/GAP43/NRXN1/ABI3/ZDHHC12/CBLN1/IL1RAP/LZTS1/NPTX1/SPTBN2 11

BP GO:0140448 signaling receptor ligand precursor processing 11/2475 39/18870 0.0096459924907537 0.0492470569000724 0.0357709871904695 ACE/ENPEP/CASP1/BACE2/CTSL/ECE1/PCSK5/PCSK6/PCSK2/DPP4/CTSZ 11

BP GO:0006334 nucleosome assembly 25/2475 118/18870 0.0097564517628507 0.049768680020523 0.0361498722474538 MACROH2A2/H4C9/H1-9P/NAP1L3/MCM2/ASF1B/H1-4/CHAF1B/H3C10/H3C2/H2BC8/H4C5/H2BC17/H1-2/H3C6/H1-5/H2BC11/H3C3/H4C8/H3-3A/H4C11/H2BC4/H4C3/HMGB2/NAP1L2 25

BP GO:0035025 positive regulation of Rho protein signal transduction 10/2475 34/18870 0.00978541880689155 0.0497895377259126 0.0361650224058445 LPAR6/GPR65/COL3A1/RTN4R/GPR4/NET1/F2RL1/F11R/F2RL3/F2RL2 10

BP GO:0060351 cartilage development involved in endochondral bone morphogenesis 10/2475 34/18870 0.00978541880689155 0.0497895377259126 0.0361650224058445 SHOX2/STC1/HOXA11/SERPINH1/MMP13/SMPD3/COL1A1/FOSL2/COL27A1/COL2A1 10

BP GO:1902624 positive regulation of neutrophil migration 10/2475 34/18870 0.00978541880689155 0.0497895377259126 0.0361650224058445 MDK/IL1A/CXCL8/CD74/IL1R1/C5AR1/THBS4/RAC2/CXCR2/LBP 10

CC GO:0062023 collagen-containing extracellular matrix 164/2620 429/19886 7.83537085739882e-40 5.17918013674062e-37 3.76097801155143e-37 LEFTY2/COL8A1/TIMP1/RARRES2/EFEMP2/MMRN1/MDK/RBP3/LAMA4/ANGPTL6/LOXL2/COL26A1/TGFB1I1/FMOD/COL14A1/COL8A2/LOXL3/MFAP2/NPNT/EFEMP1/VWF/ADAMDEC1/ECM2/NID1/COL5A3/MGP/ANXA1/COL22A1/SERPINB1/THBS1/PRG4/SERPINH1/ICAM1/MFAP5/HPX/MMP2/SMOC1/F3/COL3A1/CTSC/FREM2/SNORC/AEBP1/LGALS3/LAMA2/LOXL4/HAPLN1/ADAMTSL4/CCN1/NID2/SSC5D/HSPG2/FREM3/COL12A1/MMP9/S100A10/ITGB4/SRPX2/FBLN1/MFAP4/CD151/ADAMTS20/LAMC3/LOX/COL6A3/S100A8/ECM1/GPC1/ANGPT2/IGFBP7/COL9A3/S100A9/ACTA2/SERPINA5/ANGPTL4/LTBP1/MXRA7/ANGPT1/COL15A1/ENTPD2/PLSCR1/EGFLAM/CTSB/SERPINB8/SFRP2/L1CAM/COL1A1/LAMC1/CTSL/FN1/DCN/EMILIN2/COL4A1/BGN/PCSK6/COL4A2/SOD3/SERPINE1/COL1A2/SPN/SLPI/SERPING1/CTHRC1/ANG/ANGPTL2/MXRA5/TNC/EMILIN1/TNR/COL6A1/LTBP2/LAMB1/THBS4/ADAMTS2/CBLN1/ZP3/SBSPON/LGALS1/LGALS3BP/FBLN5/COL6A2/HAPLN3/P3H2/S100A4/ADAMTS3/TGFBI/FGL2/SERPINA1/GDF10/ELN/LUM/P3H1/COL5A2/S100A6/ANXA5/ADAM19/LOXL1/POSTN/ASPN/CILP/COL27A1/C1QB/ANXA2P2/COL5A1/F13A1/VWA1/C1QC/FGFBP3/C1QA/CTSZ/MARCO/TGFB2/SERPINA3/COL21A1/ANXA2/PLOD3/COL2A1/THSD4/ADAM11/GDF15/EMILIN3/CTSS/PCOLCE/COLEC12 164

CC GO:0097060 synaptic membrane 123/2620 393/19886 3.53551277268028e-21 1.16848697137083e-18 8.48523065443268e-19 IL1RAPL1/KCNA1/GLRA3/PDE2A/KCNC1/ITGA5/CHRNA9/UNC13A/SNAP91/HCN1/STX1A/ITGB3/HIP1R/FOSL1/GRIA4/GPR179/DNM3/CACNG2/IQSEC3/GABBR2/ANK1/PTPRT/PRRT1/PRKCG/DGKI/ADGRB3/ITGA3/GABBR1/LRRC7/NTNG2/CHRNB2/NRP1/ATP2B3/RIMS1/KCNB1/CHRNA1/PDLIM4/GABRA3/GABRA5/STXBP1/GABRD/RGS7BP/SHANK2/GABRA4/CNR1/GABRB3/HTR5A/GRIA2/CDH9/UNC13C/SHANK1/SRPX2/SHISA7/FAIM2/LRRTM3/CHRM1/GRIP1/CLSTN2/SCN2A/GRIN3A/NRXN1/ERC2/SYT7/KCNQ5/LRRTM4/GRIN2A/CNIH3/SYDE1/HTR2A/KCNH1/DDN/GRM5/SYT1/SCN8A/GRIN1/DLG2/SLC1A6/SNAP25/ARC/KCNJ3/GRM2/SLITRK1/GABRA1/PPFIA2/CBLN1/P2RY1/CNTN6/GAD2/GABRB2/NRXN3/SYP/DGKB/GABRG2/KCNC2/CNTNAP2/RAP2A/EFNB2/GRIK1/CDH8/LRFN5/P2RX6/GRM1/GABRG1/ITGB1/ADORA1/ATP2B2/CHRNA4/CHRM4/KCNJ9/RIMS3/ANK3/GRIN2B/GPR158/KCTD16/NTNG1/SLC6A7/ADAM11/CACNG5/CACNG3/MAGEE1/GABRE/RIMS2/KCNJ4 123

CC GO:0009897 external side of plasma membrane 114/2620 387/19886 1.73569207201273e-17 3.82430819866804e-15 2.77710731522037e-15 KLRC2/CD276/THBD/ITGA5/IL7R/ITGB3/MR1/KLRC3/ITGB2/BTN2A3P/TNFRSF4/CD248/CTSK/ITGA2/FAS/ACE/CLEC2B/THBS1/ITGA3/CD40/FCGR1A/TFRC/ICAM1/CHRNB2/CD3D/ITGA11/F3/CD74/KLRC4/ADGRE1/SELL/CD33/IL13RA2/HLA-C/ST14/IL1R1/MICA/RTN4R/SPA17/GRIA2/CD79A/SLAMF9/CD8A/ENG/ENPEP/KLRC4-KLRK1/CD274/ITGA4/CLEC4F/SERPINA5/MCAM/HLA-DRB1/CD3E/MICB/CXCL10/FOLR1/PDCD1LG2/CTSB/FCGR2B/GFRA1/TRPM8/CXCR4/IL2RG/SCNN1B/TLR8/TNFRSF18/ECE1/SLC2A4/CD302/BTN2A2/SPN/CD14/PLAU/CLEC10A/LY75/CXCL9/ANPEP/KCNJ3/CD36/CCR5/HLA-B/LILRB1/CD69/SDC1/HLA-A/ADAM9/CXCR2/ULBP2/CSF2RB/FCER1G/CD2/OSMR/CCRL2/ANXA5/ITGA7/ULBP3/PTPRC/ITGB1/COLEC11/CHRNA4/B2M/ITGA1/FCGR3A/IL2RA/PLAUR/CLEC17A/ITGAL/TNFRSF14/HFE/CUBN/IL2RB/CCR1/HLA-F/CD163 114

CC GO:0005788 endoplasmic reticulum lumen 92/2620 313/19886 2.63442566990223e-14 4.35338841951343e-12 3.16131080388267e-12 COL8A1/F5/TIMP1/CES1/PDIA5/WNT4/COL26A1/CHGB/PRSS23/ARSL/COL14A1/COL8A2/FUCA2/SPP1/VGF/COL5A3/COL22A1/THBS1/SERPINH1/CALU/COL3A1/CTSC/FSTL1/IGFBP3/IGFBP5/ADAMTSL4/CCN1/COL12A1/EDEM2/IGFBP4/MXRA8/COL6A3/PDIA4/IGFBP7/COL9A3/P4HA2/LTBP1/APOL1/COL15A1/ARSI/RCN3/COLGALT1/COL1A1/FAM20C/LAMC1/ARSF/FN1/COL4A1/POGLUT3/OS9/FSTL3/CST3/ERP27/MSLN/COL4A2/PDGFA/GPX7/COL1A2/SERPING1/ARSJ/TNC/COL6A1/LAMB1/MTTP/PDGFD/SCG3/LGALS1/COL6A2/P3H2/SERPINA1/P3H1/COL5A2/SELENOF/CP/C3/COL27A1/COL5A1/FKBP10/B2M/IL6/VWA1/CTSZ/PLAUR/ADAMTS7/COL21A1/SUMF2/GPX8/PLOD3/COL2A1/WNT7B/RDH5/ARSD 92

CC GO:0042611 MHC protein complex 20/2620 25/19886 6.36927027385973e-14 8.42017530204257e-12 6.11449946290535e-12 MR1/HLA-DMA/HLA-DQA2/HLA-DQA1/CD74/HLA-C/HLA-DPA1/HLA-DOA/HLA-DRB1/HLA-DPB1/HLA-DRA/HLA-DQB1/HLA-B/HLA-DOB/HLA-A/HLA-DRB5/HLA-DQB2/HLA-DMB/B2M/HLA-F 20

CC GO:0045211 postsynaptic membrane 82/2620 275/19886 2.84737366147871e-13 3.13685665039572e-11 2.27789892918297e-11 IL1RAPL1/KCNA1/GLRA3/KCNC1/ITGA5/CHRNA9/HCN1/ITGB3/GRIA4/GPR179/DNM3/CACNG2/IQSEC3/GABBR2/ANK1/PTPRT/PRRT1/ADGRB3/ITGA3/GABBR1/LRRC7/CHRNB2/NRP1/KCNB1/CHRNA1/PDLIM4/GABRA3/GABRA5/GABRD/RGS7BP/SHANK2/GABRA4/GABRB3/HTR5A/GRIA2/CDH9/SHANK1/SHISA7/FAIM2/LRRTM3/CHRM1/GRIP1/CLSTN2/GRIN3A/LRRTM4/GRIN2A/CNIH3/HTR2A/KCNH1/DDN/GRM5/SCN8A/GRIN1/DLG2/ARC/SLITRK1/GABRA1/CBLN1/P2RY1/GABRB2/DGKB/GABRG2/KCNC2/EFNB2/GRIK1/LRFN5/P2RX6/GRM1/GABRG1/ADORA1/ATP2B2/CHRNA4/CHRM4/ANK3/GRIN2B/GPR158/KCTD16/CACNG5/CACNG3/MAGEE1/GABRE/KCNJ4 82

CC GO:0005581 collagen trimer 38/2620 86/19886 1.58312884458743e-12 1.26498988750619e-10 9.18600826025676e-11 COL8A1/C1QTNF2/COL26A1/COL14A1/COL8A2/COL5A3/COL22A1/C1QTNF1/COL3A1/C1QL3/COL12A1/LOX/COL6A3/COL9A3/COL15A1/COL1A1/EMILIN2/COL4A1/COL4A2/COL1A2/CTHRC1/C1QTNF6/EMILIN1/COL6A1/COL6A2/LUM/COL5A2/COL27A1/C1QB/MSR1/COLEC11/COL5A1/C1QC/C1QA/MARCO/COL21A1/COL2A1/COLEC12 38

CC GO:0098978 glutamatergic synapse 106/2620 407/19886 1.67522665890603e-12 1.26498988750619e-10 9.18600826025676e-11 TSPOAP1/IL1RAPL1/KCNA1/NPTX2/ITGA5/HCN1/ITGB3/HIP1R/SLC6A17/DNM3/CACNG2/PLAT/ROR2/NAPB/PTPRT/VGF/ADGRB3/ITGA3/CNN3/GABBR1/CPLX2/NTNG2/NRP1/ATP2B3/MAL2/SEMA3A/NRG3/DOC2A/STXBP1/SLITRK4/FLNA/RGS7BP/SHANK2/SLC30A3/CNR1/RTN4R/C1QL3/SH3GL2/SHANK1/DNAJB1/CDH6/LRRTM3/CHRM1/OPHN1/CLSTN2/DLGAP3/SCN2A/GRIN3A/CBLN2/ERC2/SYN2/GRIN2A/HTR2A/BSN/GRM5/LYN/DLGAP2/SRGN/SYT1/SYT4/SCN8A/DLGAP1/LRRK2/SLC1A6/SNAP25/ACTN1/ARC/AURKA/GRM2/PAK3/SLITRK1/TNR/RAP1B/PPFIA2/CBLN1/P2RY1/SEMA3F/CAMKV/IL1RAP/LZTS1/NPTX1/SYN3/EFNB2/SPTBN2/SPTB/CDH8/ELAVL4/LRFN5/P2RX6/GRM1/HPCA/CTTNBP2/ADGRA1/ICAM5/PCLO/ITGB1/ATP2B2/ARHGAP44/CALY/CPLX1/NTNG1/FAM81A/PLCB1/CACNG5/CACNG3/PFN1 106

CC GO:0005604 basement membrane 39/2620 90/19886 1.72237654879814e-12 1.26498988750619e-10 9.18600826025676e-11 COL8A1/TIMP1/EFEMP2/LAMA4/LOXL2/COL8A2/NPNT/NID1/SMOC1/FREM2/LAMA2/NID2/HSPG2/FREM3/ITGB4/FBLN1/CD151/LAMC3/ACTA2/COL15A1/ENTPD2/EGFLAM/LAMC1/FN1/COL4A1/COL4A2/SPN/ANG/TNC/LAMB1/THBS4/P3H2/TGFBI/LOXL1/ANXA2P2/COL5A1/VWA1/ANXA2/COL2A1 39

CC GO:0042613 MHC class II protein complex 15/2620 17/19886 6.32164391996659e-12 4.17860663109792e-10 3.03438908158396e-10 HLA-DMA/HLA-DQA2/HLA-DQA1/CD74/HLA-DPA1/HLA-DOA/HLA-DRB1/HLA-DPB1/HLA-DRA/HLA-DQB1/HLA-DOB/HLA-DRB5/HLA-DQB2/HLA-DMB/B2M 15

CC GO:0072562 blood microparticle 49/2620 144/19886 1.11014571521562e-10 6.67096652506839e-09 4.84427221184996e-09 MSN/IGKV3-20/IGLV3-21/IGHG4/C1R/TFRC/HPX/IGHV3-7/HSPA7/IGHA1/PON1/IGKV1-5/IGKV3-15/ENG/IGLV1-47/IGHV3-23/IGHG2/ANGPTL4/IGKV2-30/APOL1/IGHG3/IGKC/IGHA2/IGLV3-25/IGKV4-1/HP/FN1/JCHAIN/ACTG2/HSPA6/HBA1/SERPING1/IGHG1/C1S/PROS1/LGALS3BP/IGHM/ANXA5/CP/C3/C1QB/F13A1/C1QC/SERPINA3/CFH/CLIC1/IGLC2/IGLC3/PFN1 49

CC GO:0034702 monoatomic ion channel complex 79/2620 300/19886 6.50902438778374e-10 3.36671245914763e-08 2.44481388863973e-08 KCNA1/AKAP6/GLRA3/KCNC1/CHRNA9/HCN1/KCNN1/GRIA4/KCNIP3/CACNG2/SLC17A8/CACNA1B/ABCC8/CHRNB2/CLIC3/KCNB1/CHRNA1/GABRA3/GABRA5/GABRD/MCUB/KCNMB1/GABRA4/KCNV1/GABRB3/GRIA2/SLC17A7/SHISA7/SCN2A/KCNAB1/CLIC4/GRIN3A/CNGA3/KCNQ5/KCNS2/GRIN2A/CNIH3/SCN2B/KCNAB2/RYR2/KCNH1/SCNN1B/KCNA4/TTYH3/CACNA2D2/BEST4/KCNN4/OLFM3/KCNE5/SCN8A/GRIN1/CACNA1E/KCNJ11/VWC2L/SLC17A6/KCNJ3/DPP10/UNC80/GABRA1/GABRB2/GABRG2/KCNC2/CNTNAP2/SCN3B/GRIK1/RYR3/LRRC55/GABRG1/CACNA2D3/SCN3A/CHRNA4/CLIC1/CACNA1I/GRIN2B/CACNG5/CACNG3/GABRE/KCNJ4/KCNIP2 79

CC GO:0019814 immunoglobulin complex 52/2620 165/19886 7.13070717519921e-10 3.36671245914763e-08 2.44481388863973e-08 IGLV1-44/IGLV2-11/IGHV5-51/IGKV3-20/IGHV2-5/IGLV2-23/IGHV3-33/IGHV3-74/IGLV3-21/IGLV6-57/IGLV3-1/IGLV2-14/IGHG4/IGLV2-8/IGHV3-7/IGHV3-21/IGLV8-61/IGHA1/IGKV1-5/IGKV3-15/CD79A/IGHV4-34/IGHV3-11/IGLV1-47/IGHV3-23/IGHG2/IGHV3-49/IGKV2-30/IGLV5-45/IGHG3/IGKC/IGHV4-39/IGHA2/IGLV3-25/IGKV4-1/JCHAIN/IGHV4-59/IGHG1/IGLV7-46/IGHV1-3/IGLV3-10/IGHM/IGHV3-15/IGHV3-48/IGHV1-18/IGLV3-19/IGLC2/IGLV1-40/IGKV1-9/IGLC3/NCR3LG1/IGHV3-30 52

CC GO:0042734 presynaptic membrane 52/2620 165/19886 7.13070717519921e-10 3.36671245914763e-08 2.44481388863973e-08 KCNA1/PDE2A/KCNC1/UNC13A/SNAP91/HCN1/STX1A/FOSL1/ITGA3/GABBR1/NTNG2/CHRNB2/ATP2B3/RIMS1/GABRA5/STXBP1/RGS7BP/CNR1/CDH9/UNC13C/CHRM1/SCN2A/NRXN1/ERC2/SYT7/KCNQ5/GRIN2A/HTR2A/KCNH1/SYT1/SCN8A/SLC1A6/SNAP25/KCNJ3/GRM2/PPFIA2/P2RY1/CNTN6/GAD2/NRXN3/SYP/KCNC2/GRIK1/ADORA1/ATP2B2/KCNJ9/RIMS3/GPR158/KCTD16/NTNG1/ADAM11/RIMS2 52

CC GO:0060076 excitatory synapse 27/2620 63/19886 5.95176757515908e-09 2.6227455781201e-07 1.90456562405091e-07 AKAP5/SLC17A8/ADGRB3/ITGA3/ATP2B3/STXBP1/GRIA2/SLC17A7/UNC13C/SHANK1/SRPX2/BSN/SYT1/SCN8A/GRIN1/SLC17A6/KCNJ3/CBLN1/CNTN6/SYP/SPTBN2/P2RX6/ITGB1/ATP2B2/PALLD/KCNJ9/CACNG3 27

CC GO:0031983 vesicle lumen 81/2620 326/19886 7.39964483657174e-09 3.0569782731087e-07 2.21989345097152e-07 LEFTY2/F5/TIMP1/RARRES2/MMRN1/FTL/PYCARD/PYGL/TMSB4X/GLA/GSDMD/S100A11/VWF/FUCA2/GGH/CTSW/SERPINB1/THBS1/LRRC7/ALDOC/S100A12/PLAC8/CTSC/OSCAR/EGFR/COTL1/ADA2/PPBP/RAB27A/RNASE2/S100A8/ECM1/GUSB/S100A9/HEXB/ARPC5/VEGFA/DSN1/CHI3L1/IGF2/HGF/HP/FN1/HEBP2/SRGN/LYZ/HSPA6/ISLR/SERPINE1/PDGFA/SLPI/SERPING1/LTF/PPIA/ACTN1/HK3/NPC2/SCG3/PROS1/LGALS3BP/RNASE3/GMFG/FABP5/SERPINA1/GNS/CFD/ALOX5/PRDX4/C3/F13A1/B2M/FUCA1/CTSZ/TGFB2/SERPINA3/PTX3/ARHGAP9/ANXA2/SERPINI1/GRP/FERMT3 81

CC GO:0034774 secretory granule lumen 80/2620 322/19886 9.17851658353013e-09 3.5688232127726e-07 2.59158115299674e-07 LEFTY2/F5/TIMP1/RARRES2/MMRN1/FTL/PYCARD/PYGL/TMSB4X/GLA/GSDMD/S100A11/VWF/FUCA2/GGH/CTSW/SERPINB1/THBS1/LRRC7/ALDOC/S100A12/PLAC8/CTSC/OSCAR/COTL1/ADA2/PPBP/RAB27A/RNASE2/S100A8/ECM1/GUSB/S100A9/HEXB/ARPC5/VEGFA/DSN1/CHI3L1/IGF2/HGF/HP/FN1/HEBP2/SRGN/LYZ/HSPA6/ISLR/SERPINE1/PDGFA/SLPI/SERPING1/LTF/PPIA/ACTN1/HK3/NPC2/SCG3/PROS1/LGALS3BP/RNASE3/GMFG/FABP5/SERPINA1/GNS/CFD/ALOX5/PRDX4/C3/F13A1/B2M/FUCA1/CTSZ/TGFB2/SERPINA3/PTX3/ARHGAP9/ANXA2/SERPINI1/GRP/FERMT3 80
[truncated: 86,520 more chars]
