# Supplementary material for: Associations between migrasome-related genes and long non-coding rnas in glioma and their prognostic relevance to the tumor microenvironment
Source: IBRO Neurosci Rep. 2026 Jun 24;21:279–90. doi: 10.1016/j.ibneur.2026.06.013 (PMC13356737; doi:10.1016/j.ibneur.2026.06.013)
Supplement: Supplementary file 7 — Supplementary material [file mmc7.docx]

ID Description GeneRatio BgRatio pvalue p.adjust qvalue geneID Count

hsa04512 ECM-receptor interaction 42/1228 89/8865 2.89114853587502e-14 9.65643610982257e-12 7.36482048086058e-12 ITGA5/LAMA4/ITGB3/NPNT/VWF/ITGA2/SPP1/THBS1/ITGA3/ITGA11/FREM2/CD44/LAMA2/HSPG2/ITGB4/CHAD/LAMC3/COL6A3/ITGA4/COL9A3/COL1A1/LAMC1/FN1/HMMR/COL4A1/COL4A2/COL1A2/TNC/CD36/TNR/COL6A1/LAMB1/THBS4/SV2B/SDC4/COL6A2/SDC1/ITGA7/ITGB1/ITGA1/COL2A1/IBSP 42

hsa05322 Systemic lupus erythematosus 53/1228 141/8865 1.20181068836408e-12 2.00702384956801e-10 1.53072729781109e-10 MACROH2A2/H4C9/H2AC20/H3-5/C1R/CD40/FCGR1A/H3C10/HLA-DMA/H3C2/HLA-DQA2/HLA-DQA1/TRIM21/H2BC8/H4C5/H2AC11/H2AJ/HLA-DPA1/HLA-DOA/H2BC17/H3C6/FCGR3B/C7/HLA-DRB1/H2AC6/GRIN2A/H2AC8/H2BC18/H2BC11/H3C3/H2BC12/H4C8/HLA-DPB1/HLA-DRA/ACTN1/C2/HLA-DQB1/C1S/H3-3A/H4C11/HLA-DOB/HLA-DRB5/HLA-DQB2/C3/C1QB/HLA-DMB/H2BC4/H4C3/C1QC/C1QA/FCGR3A/FCGR2A/GRIN2B 53

hsa04145 Phagosome 56/1228 159/8865 6.08763210917604e-12 6.777563748216e-10 5.16914726463369e-10 ITGA5/ITGB3/NCF4/NCF1/TUBB6/ITGB2/CYBA/ITGA2/C1R/THBS1/FCGR1A/HLA-DMA/TFRC/HLA-DQA2/HLA-DQA1/HLA-C/HLA-DPA1/CLEC7A/HLA-DOA/MRC2/RAB7B/FCGR3B/HLA-DRB1/NCF2/ATP6V1G2/TLR2/FCGR2B/CTSL/CD14/FCGR2C/HLA-DPB1/HLA-DRA/CD36/HLA-DQB1/HLA-B/THBS4/TUBA1C/HLA-DOB/SEC61G/HLA-A/TAP1/HLA-DRB5/HLA-DQB2/C3/HLA-DMB/ITGB1/MSR1/COLEC11/FCGR3A/MARCO/TUBA1A/TCIRG1/FCGR2A/HLA-F/CTSS/COLEC12 56

hsa05140 Leishmaniasis 34/1228 79/8865 1.96248976024666e-10 1.63867894980596e-08 1.24979611047287e-08 NCF4/IL1A/NCF1/ITGB2/CYBA/IFNGR2/FCGR1A/HLA-DMA/HLA-DQA2/HLA-DQA1/HLA-DPA1/STAT1/HLA-DOA/PRKCB/ITGA4/FCGR3B/HLA-DRB1/NCF2/TLR2/FCGR2C/HLA-DPB1/HLA-DRA/NOS2/HLA-DQB1/HLA-DOB/HLA-DRB5/HLA-DQB2/C3/HLA-DMB/ITGB1/FCGR3A/TGFB2/MYD88/FCGR2A 34

hsa05033 Nicotine addiction 23/1228 41/8865 2.45782172611051e-10 1.64182491304182e-08 1.2521954899342e-08 GRIA4/SLC32A1/SLC17A8/CACNA1B/CHRNB2/GABRA3/GABRA5/GABRD/GABRA4/GABRB3/GRIA2/SLC17A7/GRIN3A/GRIN2A/GRIN1/SLC17A6/GABRA1/GABRB2/GABRG2/GABRG1/CHRNA4/GRIN2B/GABRE 23

hsa04640 Hematopoietic cell lineage 39/1228 100/8865 3.27633447467924e-10 1.82382619090478e-08 1.39100516293399e-08 ITGA5/IL7R/ITGB3/IL1A/CD7/ITGA2/ITGA3/FCGR1A/HLA-DMA/TFRC/CD3D/HLA-DQA2/HLA-DQA1/CD44/CD33/IL1R1/HLA-DPA1/HLA-DOA/CD8A/ITGA4/HLA-DRB1/CD3E/IL11/IL1R2/CSF3/CD14/HLA-DPB1/HLA-DRA/ANPEP/CD36/HLA-DQB1/HLA-DOB/HLA-DRB5/CD2/HLA-DQB2/HLA-DMB/IL6/ITGA1/IL2RA 39

hsa04514 Cell adhesion molecules 52/1228 158/8865 5.95250176620888e-10 2.84019369987681e-08 2.1661735750715e-08 CLDN7/CD276/ITGB2/SIGLEC1/CD40/HLA-DMA/CLDN1/ICAM1/NTNG2/HLA-DQA2/HLA-DQA1/CDH4/SLITRK4/SELL/HLA-C/HLA-DPA1/NEGR1/HLA-DOA/CD8A/MPZ/CD274/ITGA4/HLA-DRB1/NRXN1/CD58/PDCD1LG2/L1CAM/F11R/SPN/HLA-DPB1/HLA-DRA/HLA-DQB1/SLITRK1/NECTIN2/HLA-B/SDC4/NRXN3/HLA-DOB/SDC1/HLA-A/CNTNAP2/HLA-DRB5/CD2/HLA-DQB2/HLA-DMB/PTPRC/ITGB1/CLDN23/ITGAL/SLITRK5/NTNG1/HLA-F 52

hsa04510 Focal adhesion 61/1228 203/8865 1.21309782038444e-09 5.06468340010503e-08 3.86275884911887e-08 CAV1/ITGA5/LAMA4/ITGB3/EMP2/ZYX/VWF/ITGA2/SPP1/PRKCG/VASP/THBS1/ITGA3/PAK5/ITGA11/SHC1/VAV3/FLNA/EGFR/LAMA2/ITGB4/BIRC3/PRKCB/CHAD/LAMC3/COL6A3/ITGA4/MET/COL9A3/EMP1/VEGFA/CAV2/HGF/COL1A1/LAMC1/FN1/COL4A1/RASGRF1/COL4A2/MYL9/PDGFA/COL1A2/ACTN1/PAK3/TNC/TNR/COL6A1/RAP1B/LAMB1/THBS4/RAC2/PDGFD/COL6A2/MYL12A/ITGA7/ITGB1/PARVG/ITGA1/COL2A1/SHC3/IBSP 61

hsa04940 Type I diabetes mellitus 23/1228 44/8865 1.61084421201725e-09 5.97802185348624e-08 4.55934852992017e-08 IL1A/FAS/HLA-DMA/HLA-DQA2/HLA-DQA1/GZMB/HLA-C/HLA-DPA1/HLA-DOA/HLA-DRB1/HLA-DPB1/HLA-DRA/HLA-DQB1/HLA-B/GAD2/HLA-DOB/HLA-A/PRF1/HLA-DRB5/GAD1/HLA-DQB2/HLA-DMB/HLA-F 23

hsa05330 Allograft rejection 21/1228 39/8865 4.10722537593749e-09 1.24879090834491e-07 9.52434288747141e-08 FAS/CD40/HLA-DMA/HLA-DQA2/HLA-DQA1/GZMB/HLA-C/HLA-DPA1/HLA-DOA/HLA-DRB1/HLA-DPB1/HLA-DRA/HLA-DQB1/HLA-B/HLA-DOB/HLA-A/PRF1/HLA-DRB5/HLA-DQB2/HLA-DMB/HLA-F 21

hsa05145 Toxoplasmosis 40/1228 112/8865 4.11278442868084e-09 1.24879090834491e-07 9.52434288747141e-08 MAP2K3/LY96/LAMA4/IFNGR2/CD40/HLA-DMA/HLA-DQA2/HLA-DQA1/SOCS1/LAMA2/HLA-DPA1/STAT1/HLA-DOA/BIRC3/LAMC3/CIITA/TNFRSF1A/HLA-DRB1/CASP8/TLR2/LAMC1/HSPA6/IL10RA/HLA-DPB1/HLA-DRA/NOS2/HLA-DQB1/CCR5/LAMB1/CASP3/HLA-DOB/HLA-DRB5/ALOX5/HLA-DQB2/HLA-DMB/ITGB1/PIK3CG/TGFB2/PIK3R6/MYD88 40

hsa04820 Cytoskeleton in muscle cells 65/1228 232/8865 8.0368920166388e-09 2.23693494463113e-07 1.70607707721631e-07 MYBPHL/ITGA5/TMOD2/DIAPH3/MYH7/ITGB3/LMNA/ZYX/ITGA2/NID1/COL5A3/THBS1/ITGA3/TNNI2/ITGA11/COL3A1/FLNC/PDLIM4/LAMA2/PDLIM3/NID2/HSPG2/ITGB4/FBLN1/COL6A3/DSG2/ITGA4/COL9A3/MYBPH/PDLIM7/FHL3/COL1A1/LMNB1/FN1/DCN/ACTG2/COL4A1/BGN/COL4A2/MYL9/COL1A2/PDLIM1/COL6A1/DSC2/THBS4/TNNT1/SDC4/COL6A2/TPM4/CAPZA1/SDC1/VIM/ELN/SPTBN2/COL5A2/DAAM2/ITGA7/MYOZ3/COL27A1/ITGB1/DES/FBN3/COL5A1/ITGA1/ANK3 65

hsa05150 Staphylococcus aureus infection 37/1228 102/8865 9.67211956404611e-09 2.48499071876262e-07 1.89526553400742e-07 ITGB2/CFI/C1R/FCGR1A/HLA-DMA/ICAM1/HLA-DQA2/FPR3/HLA-DQA1/HLA-DPA1/HLA-DOA/FCGR3B/HLA-DRB1/C5AR1/FCGR2B/KRT18/FPR2/FCGR2C/HLA-DPB1/HLA-DRA/C2/HLA-DQB1/C1S/HLA-DOB/HLA-DRB5/CFD/HLA-DQB2/C3/C1QB/HLA-DMB/C1QC/C1QA/FCGR3A/ITGAL/CFH/FCGR2A/FPR1 37

hsa05323 Rheumatoid arthritis 35/1228 95/8865 1.52608659981341e-08 3.64080660241199e-07 2.77678915154019e-07 IL18/IL1A/ITGB2/CTSK/CXCL8/CCL20/HLA-DMA/ICAM1/HLA-DQA2/HLA-DQA1/MMP1/HLA-DPA1/CCL2/HLA-DOA/HLA-DRB1/ANGPT1/ATP6V1G2/VEGFA/TLR2/CCL5/IL11/ACP5/CTSL/HLA-DPB1/HLA-DRA/HLA-DQB1/HLA-DOB/HLA-DRB5/HLA-DQB2/HLA-DMB/CXCL6/IL6/TGFB2/ITGAL/TCIRG1 35

hsa05332 Graft-versus-host disease 22/1228 45/8865 1.83758167990917e-08 4.09168187393109e-07 3.12066502833698e-07 IL1A/FAS/HLA-DMA/HLA-DQA2/HLA-DQA1/GZMB/HLA-C/HLA-DPA1/HLA-DOA/HLA-DRB1/HLA-DPB1/HLA-DRA/HLA-DQB1/HLA-B/HLA-DOB/HLA-A/PRF1/HLA-DRB5/HLA-DQB2/HLA-DMB/IL6/HLA-F 22

hsa05169 Epstein-Barr virus infection 58/1228 204/8865 2.92310559433054e-08 6.10198292816501e-07 4.65389180149994e-07 MDM2/MAP2K3/TRADD/IRF7/RELB/TRAF5/OAS2/FAS/CD247/CD40/HLA-DMA/ICAM1/CD3D/HLA-DQA2/HLA-DQA1/CD44/HLA-C/HLA-DPA1/STAT1/HLA-DOA/OAS1/HLA-DRB1/TNFAIP3/CD3E/CASP8/JAK3/CXCL10/TLR2/CD58/LYN/OAS3/RUNX3/HLA-DPB1/HLA-DRA/CDK2/CCNA2/HLA-DQB1/ISG15/HLA-B/E2F2/CASP3/HLA-DOB/HLA-A/E2F1/VIM/TAP1/HLA-DRB5/GADD45A/HLA-DQB2/HLA-DMB/CDK4/B2M/IL6/FADD/ITGAL/MYD88/CDK6/HLA-F 58

hsa05152 Tuberculosis 53/1228 182/8865 4.84604914306448e-08 9.52106125755021e-07 7.26157209053625e-07 TRADD/TLR1/IL18/IL1A/ITGB2/PLK3/IFNGR2/FCGR1A/HLA-DMA/HLA-DQA2/HLA-DQA1/CD74/HLA-DPA1/CLEC7A/STAT1/HLA-DOA/MRC2/CIITA/TNFRSF1A/FCGR3B/HLA-DRB1/CASP8/TLR2/FCGR2B/SPHK1/IL10RA/CYP27B1/CAMK2A/CEBPB/CD14/FCGR2C/HLA-DPB1/HLA-DRA/NOS2/HLA-DQB1/CASP3/LSP1/HLA-DOB/HLA-DRB5/FCER1G/HLA-DQB2/C3/HLA-DMB/IL6/LBP/FCGR3A/TGFB2/FADD/IRAK2/TCIRG1/MYD88/FCGR2A/CTSS 53

hsa05416 Viral myocarditis 28/1228 70/8865 5.43289736425704e-08 1.00810428870103e-06 7.68866176696026e-07 CAV1/MYH7/ITGB2/CD40/HLA-DMA/ICAM1/HLA-DQA2/HLA-DQA1/HLA-C/LAMA2/HLA-DPA1/HLA-DOA/HLA-DRB1/CASP8/HLA-DPB1/HLA-DRA/HLA-DQB1/HLA-B/RAC2/CASP3/HLA-DOB/HLA-A/PRF1/HLA-DRB5/HLA-DQB2/HLA-DMB/ITGAL/HLA-F 28

hsa05205 Proteoglycans in cancer 57/1228 204/8865 7.49092891680721e-08 1.31682645169137e-06 1.00432398773814e-06 CAV1/MDM2/ITGA5/WNT4/ITGB3/MSN/IQGAP1/ANK1/ITGA2/FAS/PRKCG/THBS1/FZD7/MMP2/VAV3/FLNA/EGFR/CD44/WNT16/HPSE2/HSPG2/MMP9/PRKCB/GPC1/MET/CD63/VEGFA/TLR2/CAV2/IGF2/HGF/COL1A1/FZD5/CTSL/FN1/DCN/CAMK2A/COL1A2/HOXD10/PLAU/FZD2/TWIST1/SDC4/TWIST2/CASP3/SDC1/LUM/RRAS/ITGB1/FZD1/WNT10B/TGFB2/PLAUR/ANK3/HCLS1/WNT7B/FZD6 57

hsa04610 Complement and coagulation cascades 32/1228 88/8865 9.0094322729203e-08 1.50457518957769e-06 1.14751716318248e-06 F5/THBD/ITGB2/CFI/PLAT/VWF/C1R/F3/SERPINA5/C7/C5AR1/VSIG4/SERPINE1/SERPING1/PLAU/C2/C1S/F2RL3/BDKRB2/PROS1/F2RL2/SERPINA1/CFD/C3/C1QB/F13A1/C1QC/C1QA/TFPI/PLAUR/PROCR/CFH 32

hsa05310 Asthma 17/1228 32/8865 1.65078294694424e-07 2.62553097275893e-06 2.00245349955141e-06 CD40/HLA-DMA/HLA-DQA2/HLA-DQA1/HLA-DPA1/HLA-DOA/HLA-DRB1/HLA-DPB1/HLA-DRA/HLA-DQB1/RNASE3/HLA-DOB/HLA-DRB5/FCER1G/IL9/HLA-DQB2/HLA-DMB 17

hsa04080 Neuroactive ligand-receptor interaction 86/1228 368/8865 3.48783322963293e-07 5.29516499407909e-06 4.03854373957498e-06 HTR1A/GLRA3/LPAR6/CHRNA9/SSTR3/APLN/GRIA4/TSPO/SSTR2/TSHR/GABBR2/HTR1D/VGF/GABBR1/CHRNB2/CCKBR/FPR3/CHRNA1/GABRA3/GABRA5/CRH/GABRD/APLNR/GABRA4/P2RY6/CNR1/GABRB3/HTR5A/GRIA2/S1PR3/NTS/OXTR/CHRM1/GPR156/PTGER4/GRIN3A/GZMA/C5AR1/F2RL1/GRIN2A/HTR2A/HRH1/PRLHR/VIP/GRM5/HTR2C/FPR2/GPR83/GRIN1/KISS1R/GRM2/ADCYAP1R1/F2RL3/GABRA1/P2RY1/BDKRB2/HRH3/GABRB2/CCK/F2RL2/GABRG2/LYPD6/ADRA1D/GRIK1/P2RX6/GRM1/RXFP1/NPY2R/GLP1R/GABRG1/C3/TRH/ADORA1/SSTR1/CHRNA4/CHRM4/GAL/VIPR2/MCHR2/NMUR2/GRIN2B/ADM/GRP/FPR1/ADRA2B/GABRE 86

hsa04613 Neutrophil extracellular trap formation 53/1228 193/8865 3.93675350548448e-07 5.71685074274703e-06 4.36015720058236e-06 MACROH2A2/H4C9/ITGB3/NCF4/H2AC20/NCF1/GSDMD/ITGB2/CYBA/H3-5/VWF/PRKCG/FCGR1A/H3C10/H3C2/FPR3/H2BC8/H4C5/H2AC11/H2AJ/CLEC7A/H2BC17/PRKCB/H3C6/CASP1/FCGR3B/NCF2/C5AR1/TLR2/H2AC6/H2AC8/H2BC18/TLR8/H2BC11/HDAC4/H3C3/FPR2/H2BC12/H4C8/H3-3A/RAC2/H4C11/C3/H2BC4/H4C3/AQP9/SIGLEC9/FCGR3A/ITGAL/CASP4/FCGR2A/FPR1/PLCB1 53

hsa04612 Antigen processing and presentation 29/1228 81/8865 5.26953598493166e-07 7.3334375790299e-06 5.59310398400641e-06 KLRC2/KLRC3/HLA-DMA/HLA-DQA2/HLA-DQA1/CD74/KLRC4/HLA-C/HLA-DPA1/HLA-DOA/CD8A/CIITA/HLA-DRB1/CTSB/CTSL/HSPA6/HLA-DPB1/HLA-DRA/HLA-DQB1/HLA-B/HLA-DOB/HLA-A/TAP1/HLA-DRB5/HLA-DQB2/HLA-DMB/B2M/HLA-F/CTSS 29

hsa05320 Autoimmune thyroid disease 22/1228 54/8865 9.9847162535808e-07 1.28655872762821e-05 9.81239243889146e-06 TSHR/FAS/CD40/HLA-DMA/HLA-DQA2/HLA-DQA1/GZMB/HLA-C/HLA-DPA1/HLA-DOA/HLA-DRB1/HLA-DPB1/HLA-DRA/HLA-DQB1/HLA-B/HLA-DOB/HLA-A/PRF1/HLA-DRB5/HLA-DQB2/HLA-DMB/HLA-F 22

hsa05164 Influenza A 48/1228 173/8865 1.00151278198603e-06 1.28655872762821e-05 9.81239243889146e-06 TRADD/IRF7/PYCARD/IL18/IL1A/CXCL8/OAS2/KPNA2/FAS/IFNGR2/NXT1/HLA-DMA/ICAM1/HLA-DQA2/HLA-DQA1/MX2/MX1/HLA-DPA1/CCL2/STAT1/HLA-DOA/OAS1/PRKCB/DNAJB1/CIITA/TNFRSF1A/CASP1/HLA-DRB1/CASP8/CXCL10/CCL5/IFIH1/OAS3/SOCS3/HLA-DPB1/HLA-DRA/HLA-DQB1/CASP3/HLA-DOB/RSAD2/HLA-DRB5/HLA-DQB2/HLA-DMB/CDK4/IL6/FADD/MYD88/CDK6 48

hsa04974 Protein digestion and absorption 33/1228 105/8865 2.64766725601129e-06 3.27526245743619e-05 2.49799405830305e-05 COL8A1/COL26A1/COL14A1/COL8A2/SLC16A10/COL5A3/COL22A1/SLC7A7/COL3A1/COL12A1/COL6A3/COL9A3/COL15A1/SLC8A3/COL1A1/COL4A1/KCNN4/SLC8A2/COL4A2/COL1A2/KCNE3/COL6A1/COL6A2/ELN/COL5A2/COL27A1/DPP4/FXYD2/COL5A1/COL21A1/COL2A1/XPNPEP2/SLC1A5 33

hsa05144 Malaria 20/1228 50/8865 4.34338850248764e-06 5.18104199939597e-05 3.95150382557146e-05 IL18/ITGB2/CXCL8/THBS1/CD40/ICAM1/CCL2/KLRC4-KLRK1/MET/TLR2/HGF/HBA1/CSF3/CD36/THBS4/SDC1/IL6/TGFB2/ITGAL/MYD88 20

hsa05166 Human T-cell leukemia virus 1 infection 56/1228 224/8865 4.78240144080917e-06 5.50800717665608e-05 4.20087531279789e-05 MAD2L1/SPI1/FOSL1/RELB/TSPO/ITGB2/LCK/CD40/HLA-DMA/ICAM1/CD3D/NRP1/CHEK2/HLA-DQA2/HLA-DQA1/HLA-C/IL1R1/HLA-DPA1/HLA-DOA/CDKN2C/TNFRSF1A/HLA-DRB1/CD3E/JAK3/ZFP36/ESPL1/IL2RG/IL1R2/CCNB2/MMP7/HLA-DPB1/HLA-DRA/CDK2/CCNA2/HLA-DQB1/BUB1B/HLA-B/E2F2/HLA-DOB/HLA-A/E2F1/LTBR/HLA-DRB5/PTTG1/HLA-DQB2/HLA-DMB/CDK4/B2M/IL6/IL2RA/TGFB2/ITGAL/IL2RB/CHEK1/HLA-F/CDC20 56

hsa04110 Cell cycle 43/1228 158/8865 6.26419337214265e-06 6.85852969192615e-05 5.23089878804327e-05 MDM2/MAD2L1/ESCO2/WEE1/MCM2/SGO1/CDCA5/CHEK2/ORC6/CCNB1/CDKN2C/ORC1/ESPL1/PKMYT1/TTK/CCNB2/CDK2/CCNA2/TRIP13/BUB1B/PCNA/CDC25C/BUB1/SFN/AURKB/E2F2/CDC45/E2F1/CDT1/PTTG1/DBF4/PLK1/CDC6/GADD45A/NDC80/CDK4/CDK1/CDC25A/TGFB2/KNL1/CHEK1/CDK6/CDC20 43

hsa04380 Osteoclast differentiation 40/1228 143/8865 6.36570121106918e-06 6.85852969192615e-05 5.23089878804327e-05 ITGB3/NCF4/SPI1/IL1A/FOSL1/NCF1/RELB/CTSK/LCK/CYBA/IFNGR2/LILRA6/TREM2/FCGR1A/LILRB3/LILRA4/SOCS1/OSCAR/IL1R1/LCP2/LILRA5/STAT1/TNFRSF1A/FCGR3B/NCF2/FCGR2B/ACP5/SOCS3/FCGR2C/LILRB2/TYROBP/TNFRSF11B/LILRB1/LILRA2/FOSL2/PPARG/FCGR3A/TGFB2/FCGR2A/CAMK4 40

hsa05202 Transcriptional misregulation in cancer 50/1228 198/8865 1.13919557765132e-05 0.000118903538417357 9.06859637472433e-05 MDM2/ETV7/DDIT3/NGFR/NFKBIZ/TLX1/SPI1/PLAT/H3-5/DUSP6/CXCL8/PROM1/HOXA11/CD40/FCGR1A/H3C10/H3C2/WNT16/GZMB/IGFBP3/RUNX1/PTCRA/MMP9/BCL2A1/CDKN2C/BIRC3/SPINT1/RUNX1T1/H3C6/MET/HOXA9/IL1R2/H3C3/RUNX2/PDGFA/LMO2/CEBPB/CD14/PLAU/CCNA2/H3-3A/HOXA10/GADD45A/NTRK1/IL6/PPARG/EYA1/MEIS1/IL2RB/TRAF1 50

hsa05165 Human papillomavirus infection 73/1228 333/8865 2.94490233250871e-05 0.00029805981183573 0.000227325794088391 MDM2/OASL/TRADD/ITGA5/WNT4/LAMA4/ITGB3/VWF/ITGA2/FAS/SPP1/THBS1/ITGA3/ITGA11/FZD7/EGFR/MX2/WNT16/MX1/HLA-C/LAMA2/STAT1/ITGB4/CHAD/LAMC3/JAG1/PPP2R2C/COL6A3/ITGA4/COL9A3/TNFRSF1A/PTGER4/CASP8/ATP6V1G2/VEGFA/COL1A1/FZD5/LAMC1/FN1/COL4A1/COL4A2/DLG2/COL1A2/CDK2/FZD2/CCNA2/TNC/ISG15/TNR/COL6A1/HLA-B/LAMB1/THBS4/COL6A2/CASP3/TCF7/HLA-A/E2F1/ITGA7/CDK4/ITGB1/FZD1/WNT10B/ITGA1/FADD/IRF1/TCIRG1/COL2A1/IBSP/WNT7B/CDK6/FZD6/HLA-F 73

hsa04061 Viral protein interaction with cytokine and cytokine receptor 29/1228 100/8865 5.6852888962891e-05 0.000558496026870753 0.000425956629381413 CXCL14/IL18/CXCL13/CCL26/CXCL8/CCL20/PPBP/CCL2/TNFRSF1A/TNFRSF10C/CXCL10/CCL5/CXCR4/IL2RG/IL10RA/TNFRSF10D/CCL18/IL20RA/CXCL9/CCR5/CXCR2/LTBR/CXCL6/CXCL11/IL6/IL2RA/TNFRSF14/IL2RB/CCR1 29

hsa05412 Arrhythmogenic right ventricular cardiomyopathy 26/1228 86/8865 6.28544923015083e-05 0.000599811440820107 0.000457467282314737 ITGA5/ITGB3/LEF1/CACNG2/LMNA/ITGA2/ITGA3/ITGA11/CTNNA3/LAMA2/ITGB4/DSG2/ITGA4/SLC8A3/RYR2/CACNA2D2/SLC8A2/DSC2/TCF7/ITGA7/ITGB1/DES/CACNA2D3/ITGA1/CACNG5/CACNG3 26

hsa05032 Morphine addiction 27/1228 91/8865 6.5720010324274e-05 0.000609735651341876 0.000465036330364746 PDE2A/SLC32A1/GABBR2/CACNA1B/PRKCG/GNG3/GABBR1/GABRA3/GABRA5/GABRD/GNG11/GABRA4/GABRB3/PRKCB/PDE1A/GNG12/GNG13/KCNJ3/GABRA1/GNG5/GABRB2/GABRG2/GABRG1/ADORA1/KCNJ9/GNGT2/GABRE 27

hsa04672 Intestinal immune network for IgA production 18/1228 50/8865 6.92564092950043e-05 0.000625179478500849 0.000476815108090783 CD40/HLA-DMA/HLA-DQA2/HLA-DQA1/HLA-DPA1/HLA-DOA/ITGA4/HLA-DRB1/CXCR4/HLA-DPB1/HLA-DRA/HLA-DQB1/HLA-DOB/LTBR/HLA-DRB5/HLA-DQB2/HLA-DMB/IL6 18

hsa05133 Pertussis 24/1228 78/8865 8.74453246563321e-05 0.000768598379874077 0.000586198575258514 LY96/ITGA5/PYCARD/IL1A/ITGB2/CXCL8/C1R/CASP7/CASP1/SERPING1/CD14/NOS2/C2/C1S/CASP3/C3/C1QB/ITGB1/CXCL6/IL6/C1QC/C1QA/IRF1/MYD88 24

hsa04658 Th1 and Th2 cell differentiation 27/1228 93/8865 9.9300179786275e-05 0.000850416924323483 0.000648600364595912 LCK/IFNGR2/CD247/HLA-DMA/DLL3/CD3D/HLA-DQA2/HLA-DQA1/HLA-DPA1/STAT1/HLA-DOA/JAG1/HLA-DRB1/CD3E/JAK3/IL2RG/RUNX3/HLA-DPB1/HLA-DRA/HLA-DQB1/HLA-DOB/HLA-DRB5/HLA-DQB2/HLA-DMB/IL2RA/IL2RB/DLL4 27

hsa04010 MAPK signaling pathway 65/1228 300/8865 0.000115210945365544 0.000962011393802292 0.000733711809959517 MAP2K3/TRADD/DDIT3/NGFR/IL1A/RELB/CACNG2/DUSP5/DUSP6/FAS/CACNA1B/PRKCG/RET/PTPN7/DUSP4/FLNA/MAP3K8/EGFR/HSPB1/IL1R1/MAP3K20/AREG/DUSP9/PRKCB/ANGPT2/MET/PTPRR/TNFRSF1A/MAP3K9/ANGPT1/VEGFA/IGF2/HGF/DUSP10/CACNA2D2/GNG12/RPS6KA1/HSPA6/MAPT/PTPN5/RASGRF1/PDGFA/CACNA1E/CD14/RAP1B/RASGRF2/EFNA4/RAC2/PDGFD/IL1RAP/CASP3/RRAS/EPHA2/GADD45A/MAP3K6/CACNA2D3/MAPK8IP2/GDNF/NTRK1/PLA2G4A/TGFB2/MYD88/CACNA1I/CACNG5/CACNG3 65

hsa04115 p53 signaling pathway 23/1228 75/8865 0.000129048732027963 0.00105127503652048 0.000801791865231501 MDM2/FAS/THBS1/RRM2/CHEK2/IGFBP3/RPRM/CCNB1/CASP8/MDM4/STEAP3/CCNB2/SERPINE1/CDK2/SFN/CASP3/GADD45A/CDK4/CDK1/GTSE1/CHEK1/CDK6/TP73 23

hsa05321 Inflammatory bowel disease 21/1228 66/8865 0.000140211934608738 0.00111501871807901 0.00085040822494523 IL18/IL1A/IFNGR2/HLA-DMA/HLA-DQA2/HLA-DQA1/HLA-DPA1/STAT1/HLA-DOA/HLA-DRB1/TLR2/IL2RG/HLA-DPB1/HLA-DRA/HLA-DQB1/HLA-DOB/HLA-DRB5/HLA-DQB2/HLA-DMB/IL6/TGFB2 21

hsa04151 PI3K-Akt signaling pathway 75/1228 362/8865 0.000161797062538881 0.0012567492764648 0.000958504017978194 MDM2/LPAR6/ITGA5/IL7R/LAMA4/NGFR/ITGB3/VWF/ITGA2/SPP1/GNG3/THBS1/ITGA3/RET/ITGA11/EGFR/GNG11/LAMA2/AREG/ITGB4/CHAD/LAMC3/PPP2R2C/COL6A3/ANGPT2/CHRM1/ITGA4/MET/COL9A3/BRCA1/ANGPT1/JAK3/VEGFA/TLR2/IL2RG/IGF2/HGF/COL1A1/LAMC1/FN1/GNG12/COL4A1/COL4A2/CSF3/GNG13/PDGFA/COL1A2/CDK2/TNC/TNR/COL6A1/LAMB1/THBS4/GNG5/EFNA4/PDGFD/COL6A2/EPHA2/OSM/OSMR/ITGA7/CDK4/ITGB1/GDNF/NTRK1/IL6/PIK3CG/ITGA1/IL2RA/PIK3R6/GNGT2/COL2A1/IBSP/IL2RB/CDK6 75

hsa04060 Cytokine-cytokine receptor interaction 64/1228 298/8865 0.00016933102408087 0.00128537641006842 0.000980337507836614 CXCL14/IL7R/NGFR/IL18/CXCL13/IL1A/CCL26/TNFRSF4/CXCL8/CCL20/FAS/IFNGR2/IL1RN/TNFRSF19/CD40/CD70/ACVR1C/TNFRSF12A/PPBP/IL13RA2/TNFSF8/IL1R1/CCL2/BMP2/IL17RC/TNFRSF1A/CTF1/TNFRSF10C/CXCL10/CCL5/CXCR4/IL2RG/IL11/TNFRSF18/IL1R2/IL10RA/TNFRSF10D/CSF3/LIF/CCL18/IL20RA/CXCL9/CCR5/TNFRSF11B/IL1RAP/CXCR2/CSF2RB/GDF10/LTBR/OSM/OSMR/IL9/IL32/CXCL6/CXCL11/IL6/IL2RA/TGFB2/TNFRSF14/IL2RB/CCR1/CLCF1/NODAL/GDF15 64

hsa05146 Amoebiasis 28/1228 103/8865 0.000256428107749329 0.00190326639973947 0.00145159303100205 LAMA4/ITGB2/CXCL8/PRKCG/COL3A1/LAMA2/HSPB1/IL1R1/PRKCB/LAMC3/RAB7B/TLR2/COL1A1/LAMC1/FN1/IL1R2/COL4A1/COL4A2/COL1A2/CD14/NOS2/ACTN1/LAMB1/CASP3/IL6/TGFB2/GNAL/PLCB1 28

hsa04668 TNF signaling pathway 31/1228 119/8865 0.00028303381498604 0.00205507161315951 0.00156737261388151 MAP2K3/TRADD/TRAF5/CCL20/FAS/ICAM1/BCL3/MAP3K8/RIPK3/MLKL/CCL2/MMP9/BIRC3/CASP7/JAG1/TNFRSF1A/TNFAIP3/MMP14/CASP8/CXCL10/CCL5/SOCS3/LIF/CEBPB/CASP3/CXCL6/IL6/RHBDF1/FADD/IRF1/TRAF1 31

hsa04727 GABAergic synapse 25/1228 89/8865 0.000312656309452417 0.00222185547568313 0.00169457619008925 SLC32A1/GABBR2/CACNA1B/PRKCG/GNG3/GABBR1/GABRA3/GABRA5/GABRD/GNG11/GABRA4/GABRB3/PRKCB/GNG12/SLC12A5/GNG13/GABRA1/GNG5/GAD2/GABRB2/GABRG2/GAD1/GABRG1/GNGT2/GABRE 25

hsa04650 Natural killer cell mediated cytotoxicity 34/1228 136/8865 0.000341111909945354 0.00237357037336975 0.00181028689049946 KLRC2/KLRC3/ITGB2/LCK/FAS/IFNGR2/PRKCG/CD247/ICAM1/SHC1/VAV3/GZMB/HLA-C/MICA/LCP2/PRKCB/HCST/KLRC4-KLRK1/FCGR3B/MICB/TYROBP/HLA-B/RAC2/CASP3/HLA-A/LAT2/PRF1/ULBP2/CD48/FCER1G/ULBP3/FCGR3A/ITGAL/SHC3 34

hsa05170 Human immunodeficiency virus 1 infection 47/1228 213/8865 0.000646816010480921 0.00440890913266587 0.00336260954965377 APOBEC3B/MAP2K3/TRADD/WEE1/TRAF5/FAS/PRKCG/GNG3/CD247/CGAS/PAK5/CD3D/APOBEC3G/HLA-C/GNG11/AP1S2/APOBEC3C/CCNB1/PRKCB/TNFRSF1A/CD3E/CASP8/TLR2/APOBEC3F/CXCR4/GNG12/CCNB2/GNG13/PAK3/CCR5/HLA-B/CDC25C/GNG5/RAC2/CASP3/TRIM5/HLA-A/TAP1/CDK1/B2M/FADD/MYD88/APOBEC3H/GNGT2/CHEK1/HLA-F/BST2 47

hsa04659 Th17 cell differentiation 28/1228 109/8865 0.000696845506597745 0.00465492798407293 0.00355024447571904 LCK/IFNGR2/CD247/HLA-DMA/CD3D/HLA-DQA2/HLA-DQA1/AHR/RUNX1/IL1R1/HLA-DPA1/STAT1/HLA-DOA/HLA-DRB1/CD3E/JAK3/IL2RG/HLA-DPB1/HLA-DRA/HLA-DQB1/IL1RAP/HLA-DOB/HLA-DRB5/HLA-DQB2/HLA-DMB/IL6/IL2RA/IL2RB 28

hsa05410 Hypertrophic cardiomyopathy 26/1228 99/8865 0.000745783260783136 0.00488414919806995 0.00372506809307573 ITGA5/MYH7/ITGB3/CACNG2/LMNA/ITGA2/ACE/ITGA3/ITGA11/LAMA2/ITGB4/ITGA4/SLC8A3/RYR2/CACNA2D2/SLC8A2/TPM4/ITGA7/ITGB1/DES/CACNA2D3/IL6/ITGA1/TGFB2/CACNG5/CACNG3 26

hsa04670 Leukocyte transendothelial migration 29/1228 116/8865 0.000904941601761291 0.00574368992301138 0.00438062704496928 CLDN7/NCF4/MSN/NCF1/ITGB2/CYBA/PRKCG/VASP/CLDN1/ICAM1/MMP2/VAV3/CTNNA3/RHOH/MMP9/PRKCB/RAPGEF4/ITGA4/NCF2/CXCR4/F11R/MYL9/ACTN1/RAP1B/RAC2/MYL12A/ITGB1/CLDN23/ITGAL 29

hsa05143 African trypanosomiasis 13/1228 37/8865 0.000911423850058691 0.00574368992301138 0.00438062704496928 LAMA4/IL18/FAS/PRKCG/ICAM1/PRKCB/APOL1/IDO1/F2RL1/HBA1/IL6/MYD88/PLCB1 13

hsa05142 Chagas disease 26/1228 103/8865 0.00140339633264528 0.0086802662056208 0.00662031018518826 CXCL8/FAS/IFNGR2/ACE/CD247/CD3D/CCL2/PPP2R2C/TNFRSF1A/CD3E/CASP8/TLR2/CCL5/SERPINE1/NOS2/BDKRB2/C3/C1QB/IL6/C1QC/C1QA/TGFB2/GNAL/FADD/MYD88/PLCB1 26

hsa05222 Small cell lung cancer 24/1228 93/8865 0.00152400866375635 0.00925488897626582 0.00705856644266098 LAMA4/CKS2/TRAF5/ITGA2/ITGA3/LAMA2/BIRC3/LAMC3/CKS1B/LAMC1/FN1/COL4A1/COL4A2/NOS2/CDK2/LAMB1/E2F2/CASP3/E2F1/GADD45A/CDK4/ITGB1/CDK6/TRAF1 24

hsa04724 Glutamatergic synapse 28/1228 116/8865 0.00194522971581979 0.0115713426767276 0.0088252912945732 GRIA4/SLC17A8/PRKCG/GNG3/GNG11/SHANK2/GRIA2/SLC17A7/SHANK1/PRKCB/GRIN3A/GRIN2A/GRM5/GNG12/GNG13/DLGAP1/GRIN1/SLC1A6/SLC17A6/KCNJ3/GRM2/GNG5/GRIK1/GRM1/PLA2G4A/GNGT2/GRIN2B/PLCB1 28

hsa04721 Synaptic vesicle cycle 21/1228 79/8865 0.00197475009752537 0.0115713426767276 0.0088252912945732 UNC13A/STX1A/DNM3/SLC32A1/STX1B/SLC17A8/CACNA1B/CPLX2/RIMS1/STXBP1/RAB3A/SLC17A7/UNC13C/ATP6V1G2/SYT1/SLC1A6/SNAP25/SLC17A6/TCIRG1/CPLX1/SLC6A7 21

hsa05219 Bladder cancer 13/1228 41/8865 0.00264555656434854 0.0152347567671106 0.0116193228416034 MDM2/CXCL8/TYMP/THBS1/MMP2/MMP1/EGFR/MMP9/VEGFA/E2F2/E2F1/CDK4/UPK3A 13

hsa05034 Alcoholism 40/1228 188/8865 0.00318841347350538 0.0180496627144203 0.0137662098231633 MACROH2A2/H4C9/H2AC20/H3-5/GNG3/H3C10/SHC1/H3C2/CRH/H2BC8/H4C5/H2AC11/H2AJ/GNG11/H2BC17/H3C6/GRIN3A/H2AC6/GRIN2A/H2AC8/H2BC18/H2BC11/HDAC4/H3C3/GNG12/H2BC12/GNG13/H4C8/GRIN1/H3-3A/GNG5/H4C11/H2BC4/H4C3/GNGT2/SLC29A1/GRIN2B/SHC3/MAOB/CAMK4 40

hsa04723 Retrograde endocannabinoid signaling 33/1228 149/8865 0.00366581796326115 0.0204063866621537 0.0155636481948982 GRIA4/SLC32A1/SLC17A8/CACNA1B/PRKCG/GNG3/RIMS1/GABRA3/GABRA5/GABRD/GNG11/GABRA4/CNR1/GABRB3/GRIA2/SLC17A7/PRKCB/GRM5/GNG12/GNG13/SLC17A6/KCNJ3/GABRA1/GNG5/NDUFA4L2/GABRB2/GABRG2/GRM1/GABRG1/KCNJ9/GNGT2/PLCB1/GABRE 33

hsa05167 Kaposi sarcoma-associated herpesvirus infection 41/1228 196/8865 0.00392844552884285 0.0213582213694095 0.0162895983970914 TRADD/IRF7/LEF1/CXCL8/FAS/CLEC2B/GNG3/ICAM1/HLA-C/GNG11/MICA/STAT1/ANGPT2/TNFRSF1A/CASP8/MICB/VEGFA/ZFP36/LYN/GNG12/GNG13/MAP1LC3C/CCR5/HLA-B/GNG5/E2F2/RCAN1/CASP3/TCF7/HLA-A/E2F1/C3/CDK4/IL6/PIK3CG/PIK3R6/FADD/GNGT2/CCR1/CDK6/HLA-F 41

hsa05340 Primary immunodeficiency 12/1228 38/8865 0.00396469977515984 0.0213582213694095 0.0162895983970914 IL7R/LCK/CD40/CD3D/CD79A/CD8A/CIITA/CD3E/JAK3/IL2RG/TAP1/PTPRC 12

hsa05162 Measles 31/1228 139/8865 0.00427484018272639 0.0226634384290573 0.0172850680738477 TRADD/IRF7/IL1A/MSN/OAS2/FAS/CD3D/MX2/MX1/STAT1/OAS1/TNFAIP3/CD3E/CASP8/JAK3/TLR2/FCGR2B/IL2RG/IFIH1/HSPA6/OAS3/CDK2/CASP3/CDK4/IL6/IL2RA/FADD/MYD88/IL2RB/CDK6/TP73 31

hsa04933 AGE-RAGE signaling pathway in diabetic complications 24/1228 101/8865 0.00488852260250162 0.0255119773318053 0.0194576064112729 THBD/IL1A/CXCL8/ICAM1/MMP2/F3/COL3A1/NOX4/CCL2/STAT1/PRKCB/VEGFA/COL1A1/FN1/COL4A1/COL4A2/SERPINE1/COL1A2/CASP3/CDK4/PRKCE/IL6/TGFB2/PLCB1 24

hsa00910 Nitrogen metabolism 7/1228 17/8865 0.00525843562072828 0.0270202691895884 0.0206079582221254 CA12/CA2/CA9/CA3/GLUD1/CA13/CA7 7

hsa04911 Insulin secretion 21/1228 86/8865 0.00587385113455299 0.0297252466506166 0.0226710043789764 STX1A/KCNN1/PRKCG/ABCC8/KCNMB1/RAB3A/PRKCB/RAPGEF4/RYR2/KCNN4/CAMK2A/KCNJ11/SNAP25/ADCYAP1R1/CCK/GCK/PCLO/GLP1R/FXYD2/PLCB1/RIMS2 21

hsa04621 NOD-like receptor signaling pathway 39/1228 189/8865 0.00614059241428063 0.0306113114383542 0.0233467928398415 GBP5/PSTPIP1/IRF7/PYCARD/IL18/GSDMD/TRAF5/CYBA/CXCL8/OAS2/ANTXR2/GBP2/PYDC1/RIPK3/GBP1/CCL2/STAT1/GBP4/OAS1/BIRC3/TRIP6/CASP1/TNFAIP3/CASP8/CARD16/IFI16/CTSB/CCL5/OAS3/GBP3/NAMPT/MAP1LC3C/CARD6/TRPV2/IL6/FADD/CASP4/MYD88/PLCB1 39

hsa04726 Serotonergic synapse 26/1228 115/8865 0.00700916715729376 0.0344273798608252 0.026257251579955 HTR1A/HTR1D/CACNA1B/PRKCG/GNG3/GNG11/GABRB3/HTR5A/PRKCB/ALOX15B/PTGS1/HTR2A/GNG12/HTR2C/GNG13/CYP4X1/KCNJ3/GNG5/GABRB2/CASP3/ALOX5/PLA2G4A/KCNJ9/GNGT2/PLCB1/MAOB 26

hsa04814 Motor proteins 40/1228 197/8865 0.00738043049108277 0.0357255620872702 0.0272473558938525 KIFC1/KIF3C/MYH7/KIF14/TUBB6/KIF20A/KIF18B/KIF9/KIF26A/DNAI3/KIF4A/MYO5C/TNNI2/KIF23/KIF21B/DNAH9/DNAI4/ACTA2/KIF24/KIF15/BICDL1/DYNLT3/MYO1B/ACTG2/MYL9/MYL6/KIF18A/TNNT1/KIF2C/TUBA1C/TPM4/DNALI1/CAPZA1/MYL12A/MYO5B/MYO16/CENPE/MYO1F/TUBA1A/MYO1G 40

hsa05414 Dilated cardiomyopathy 24/1228 105/8865 0.00813221657906497 0.0388022905343957 0.0295939310095297 ITGA5/MYH7/ITGB3/CACNG2/LMNA/ITGA2/ITGA3/ITGA11/LAMA2/ITGB4/ITGA4/SLC8A3/RYR2/CACNA2D2/SLC8A2/TPM4/ITGA7/ITGB1/DES/CACNA2D3/ITGA1/TGFB2/CACNG5/CACNG3 24

hsa04210 Apoptosis 29/1228 136/8865 0.0106363612343521 0.0500358401728678 0.038161592568024 TRADD/DDIT3/CTSK/LMNA/FAS/CTSW/CTSC/GZMB/BCL2A1/BIRC3/CASP7/TNFRSF1A/BIRC5/CASP8/CTSB/LMNB1/CTSL/CASP6/TUBA1C/CASP3/PRF1/CSF2RB/GADD45A/NTRK1/CTSZ/TUBA1A/FADD/CTSS/TRAF1 29

hsa04713 Circadian entrainment 22/1228 97/8865 0.0120228214928036 0.0557725330360613 0.0425368830593345 GRIA4/PRKCG/GNG3/GNG11/GRIA2/PRKCB/GRIN2A/RYR2/GNG12/GNG13/GRIN1/CAMK2A/KCNJ3/ADCYAP1R1/GNG5/RYR3/PRKG2/KCNJ9/GNGT2/CACNA1I/GRIN2B/PLCB1 22

hsa04020 Calcium signaling pathway 48/1228 254/8865 0.0140652743321114 0.0643534469441808 0.0490814187220036 SLN/CACNA1B/PRKCG/RET/CCKBR/ATP2B3/EGFR/HTR5A/PRKCB/ITPKC/OXTR/CHRM1/MET/GRIN3A/VEGFA/SLC8A3/GRIN2A/HTR2A/PDE1A/RYR2/HRH1/CXCR4/SPHK1/CAMK1G/GRM5/HGF/HTR2C/SLC8A2/GRIN1/PDGFA/CAMK2A/CACNA1E/NOS2/BDKRB2/PDGFD/ADRA1D/RYR3/P2RX6/GRM1/MCOLN2/ATP2B2/GDNF/NTRK1/GNAL/CACNA1I/GRIN2B/PLCB1/CAMK4 48
