# Supplementary material for: Associations between migrasome-related genes and long non-coding rnas in glioma and their prognostic relevance to the tumor microenvironment
Source: IBRO Neurosci Rep. 2026 Jun 24;21:279–90. doi: 10.1016/j.ibneur.2026.06.013 (PMC13356737; doi:10.1016/j.ibneur.2026.06.013)
Supplement: Supplementary file 8 — Supplementary material [file mmc8.docx]

id TCGA-06-0675-11A-32R-A36H-07 TCGA-06-0680-11A-32R-A36H-07 TCGA-06-AABW-11A-31R-A36H-07 TCGA-06-0681-11A-41R-A36H-07 TCGA-06-0678-11A-32R-A36H-07 TCGA-06-2559-01A-01R-1849-01 TCGA-DU-8161-01A-11R-2256-07 TCGA-DB-A64S-01A-11R-A29R-07 TCGA-DH-A66G-01A-21R-A31N-07 TCGA-S9-A6U0-01A-12R-A32Q-07 TCGA-E1-A7YL-01A-11R-A34F-07 TCGA-HT-A74J-01A-12R-A32Q-07 TCGA-EZ-7264-01A-11R-2027-07 TCGA-TQ-A7RM-01A-11R-A33Z-07 TCGA-QH-A6X4-01A-51R-A32Q-07 TCGA-CS-5397-01A-01R-1896-07 TCGA-12-5295-01A-01R-1849-01 TCGA-06-2569-01A-01R-1849-01 TCGA-P5-A77W-01A-11R-A32Q-07 TCGA-DU-7304-02A-12R-A36H-07 TCGA-HT-A619-01A-11R-A29R-07 TCGA-S9-A6UA-01A-12R-A33Z-07 TCGA-FG-8187-01A-11R-2256-07 TCGA-FG-A6J3-01A-11R-A31N-07 TCGA-E1-A7YK-01A-11R-A34F-07 TCGA-HT-8104-01A-11R-2404-07 TCGA-S9-A6WG-01A-11R-A33Z-07 TCGA-DU-A6S6-01A-21R-A32Q-07 TCGA-DU-7298-01A-11R-2027-07 TCGA-HT-A61B-01A-11R-A29R-07 TCGA-26-5136-01B-01R-1850-01 TCGA-HT-7693-01A-11R-2256-07 TCGA-RY-A843-01A-11R-A36H-07 TCGA-06-5414-01A-01R-1849-01 TCGA-06-0138-01A-02R-1849-01 TCGA-QH-A65Z-01A-11R-A29R-07 TCGA-HT-7603-01A-21R-2090-07 TCGA-RY-A83Y-01A-11R-A36H-07 TCGA-S9-A6TU-01A-12R-A32Q-07 TCGA-E1-A7Z6-01A-11R-A34R-07 TCGA-DH-5141-01A-01R-1470-07 TCGA-QH-A6CU-01A-11R-A31N-07 TCGA-DU-6393-01A-11R-1708-07 TCGA-76-4932-01A-01R-1850-01 TCGA-R8-A6MO-01A-11R-A33Z-07 TCGA-CS-6667-01A-12R-2027-07 TCGA-FG-A4MU-01B-11R-A28M-07 TCGA-FG-A4MT-01A-11R-A26U-07 TCGA-06-5858-01A-01R-1849-01 TCGA-HT-7902-01A-12R-2403-07 TCGA-DH-A66D-01A-11R-A31N-07 TCGA-TM-A7CF-02A-11R-A32Q-07 TCGA-FG-6689-01A-11R-1896-07 TCGA-28-2513-01A-01R-1850-01 TCGA-DU-5870-02A-12R-A36H-07 TCGA-DB-5270-01A-02R-1470-07 TCGA-DU-5874-01A-11R-1708-07 TCGA-19-1787-01B-01R-1850-01 TCGA-E1-5311-01A-01R-1470-07 TCGA-DH-5140-01A-01R-1470-07 TCGA-16-1045-01B-01R-1850-01 TCGA-FG-7643-01A-11R-2090-07 TCGA-DB-A64O-01A-11R-A29R-07 TCGA-HT-A74H-01A-11R-A32Q-07 TCGA-06-0644-01A-02R-1849-01 TCGA-27-1837-01A-01R-1850-01 TCGA-P5-A5ET-01A-11R-A27Q-07 TCGA-06-5413-01A-01R-1849-01 TCGA-DU-7018-01A-11R-2027-07 TCGA-76-4928-01B-01R-1850-01 TCGA-FG-7638-01B-12R-2090-07 TCGA-TQ-A7RF-01A-11R-A33Z-07 TCGA-DU-6407-02A-12R-A36H-07 TCGA-DB-A64R-01A-11R-A29R-07 TCGA-27-1835-01A-01R-1850-01 TCGA-HT-7874-01A-11R-2403-07 TCGA-WY-A85A-01A-21R-A36H-07 TCGA-15-1444-01A-02R-1850-01 TCGA-E1-5318-01A-01R-1470-07 TCGA-HT-A74O-01A-11R-A32Q-07 TCGA-06-0747-01A-01R-1849-01 TCGA-06-0749-01A-01R-1849-01 TCGA-QH-A6CW-01A-11R-A32Q-07 TCGA-DU-A5TU-01A-11R-A28M-07 TCGA-06-0750-01A-01R-1849-01 TCGA-DH-A7UR-01A-11R-A33Z-07 TCGA-19-5960-01A-11R-1850-01 TCGA-QH-A870-01A-11R-A36H-07 TCGA-R8-A6YH-01A-21R-A32Q-07 TCGA-06-0125-02A-11R-2005-01 TCGA-E1-A7YV-01A-11R-A34R-07 TCGA-DH-5144-01A-01R-1470-07 TCGA-TM-A7C3-01A-11R-A32Q-07 TCGA-S9-A7IX-01A-12R-A34F-07 TCGA-HT-A61C-01A-11R-A29R-07 TCGA-WY-A859-01A-12R-A36H-07 TCGA-HT-8107-01A-13R-2404-07 TCGA-28-5204-01A-01R-1850-01 TCGA-HT-A5RC-01A-11R-A28M-07 TCGA-32-2616-01A-01R-1850-01 TCGA-DU-A5TP-01A-11R-A28M-07 TCGA-DU-8158-01A-11R-2256-07 TCGA-P5-A72X-01A-11R-A32Q-07 TCGA-E1-A7YE-01A-11R-A34F-07 TCGA-DB-A4XD-01A-11R-A27Q-07 TCGA-CS-5393-01A-01R-1470-07 TCGA-27-2523-01A-01R-1850-01 TCGA-HT-A618-01A-11R-A29R-07 TCGA-28-5207-01A-01R-1850-01 TCGA-14-0787-01A-01R-1849-01 TCGA-TM-A84C-01A-11R-A36H-07 TCGA-S9-A7R8-01A-11R-A34R-07 TCGA-P5-A72W-01A-11R-A32Q-07 TCGA-12-3653-01A-01R-1849-01 TCGA-S9-A89V-01A-11R-A36H-07 TCGA-02-2483-01A-01R-1849-01 TCGA-28-5215-01A-01R-1850-01 TCGA-06-0158-01A-01R-1849-01 TCGA-QH-A65S-01A-11R-A29R-07 TCGA-06-0882-01A-01R-1849-01 TCGA-DU-5871-01A-12R-1708-07 TCGA-32-1982-01A-01R-1850-01 TCGA-DU-5870-01A-11R-1708-07 TCGA-14-1402-02A-01R-2005-01 TCGA-E1-5303-01A-01R-1470-07 TCGA-DU-A76L-01A-11R-A32Q-07 TCGA-DH-A66B-01A-11R-A29R-07 TCGA-P5-A735-01A-11R-A32Q-07 TCGA-DU-6399-01A-12R-1708-07 TCGA-E1-A7YH-01A-11R-A34F-07 TCGA-FG-8188-01A-11R-2256-07 TCGA-HW-7490-01A-11R-2027-07 TCGA-HT-7478-01A-11R-2027-07 TCGA-27-2524-01A-01R-1850-01 TCGA-32-1980-01A-01R-1850-01 TCGA-CS-5396-01A-02R-1470-07 TCGA-E1-A7YM-01A-11R-A34F-07 TCGA-28-2514-01A-02R-1850-01 TCGA-QH-A6CY-01A-11R-A32Q-07 TCGA-12-1597-01B-01R-1849-01 TCGA-FG-A4MY-01A-11R-A26U-07 TCGA-32-4213-01A-01R-1850-01 TCGA-DU-5852-01A-11R-1708-07 TCGA-19-2619-01A-01R-1850-01 TCGA-HT-7611-01A-11R-2403-07 TCGA-S9-A6WD-01A-12R-A33Z-07 TCGA-VV-A86M-01A-11R-A36H-07 TCGA-HT-7468-01A-11R-2027-07 TCGA-DB-A4XF-01A-11R-A27Q-07 TCGA-HT-8108-01A-11R-2404-07 TCGA-FG-7637-01A-11R-2090-07 TCGA-14-0790-01B-01R-1849-01 TCGA-P5-A780-01A-12R-A32Q-07 TCGA-DU-6401-01A-11R-1708-07 TCGA-HT-7605-01A-11R-2090-07 TCGA-14-1034-01A-01R-1849-01 TCGA-CS-6188-01A-11R-1896-07 TCGA-DU-A7TI-01A-11R-A33Z-07 TCGA-DU-7301-01A-11R-2090-07 TCGA-P5-A737-01A-11R-A32Q-07 TCGA-S9-A7J0-01A-11R-A34F-07 TCGA-DB-A4X9-01A-11R-A26U-07 TCGA-HT-7610-01A-21R-2090-07 TCGA-HT-8558-01A-21R-2404-07 TCGA-HT-8015-01B-11R-A28M-07 TCGA-06-0744-01A-01R-1849-01 TCGA-HT-A616-01A-11R-A29R-07 TCGA-DU-6408-01A-11R-1708-07 TCGA-DU-6407-02B-11R-A36H-07 TCGA-CS-6670-01A-11R-1896-07 TCGA-FG-5962-01B-11R-1896-07 TCGA-06-0645-01A-01R-1849-01 TCGA-DU-A7TB-01A-11R-A33Z-07 TCGA-DB-A64P-01A-11R-A29R-07 TCGA-27-1834-01A-01R-1850-01 TCGA-28-5209-01A-01R-1850-01 TCGA-S9-A6U1-01A-21R-A33Z-07 TCGA-TM-A84O-01A-11R-A36H-07 TCGA-HT-7884-01B-11R-2403-07 TCGA-HT-7467-01A-11R-2027-07 TCGA-DU-5872-01A-11R-1708-07 TCGA-S9-A6WE-01A-12R-A33Z-07 TCGA-HT-7692-01A-12R-2256-07 TCGA-HT-7485-01A-11R-2027-07 TCGA-06-0238-01A-02R-1849-01 TCGA-R8-A6MK-01A-11R-A32Q-07 TCGA-28-2509-01A-01R-1850-01 TCGA-DB-A4XE-01A-11R-A27Q-07 TCGA-TM-A7C5-01A-11R-A32Q-07 TCGA-HT-7684-01A-11R-2256-07 TCGA-S9-A6UB-01A-21R-A33Z-07 TCGA-06-0156-01A-02R-1849-01 TCGA-TQ-A7RK-02B-11R-A40A-07 TCGA-32-5222-01A-01R-1850-01 TCGA-28-5208-01A-01R-1850-01 TCGA-HT-8018-01A-11R-2404-07 TCGA-DB-5275-01A-01R-1470-07 TCGA-RY-A83Z-01A-11R-A36H-07 TCGA-S9-A7IS-01A-11R-A34F-07 TCGA-DU-6397-01A-11R-1708-07 TCGA-HT-8110-01A-11R-2404-07 TCGA-P5-A5F6-01A-11R-A28M-07 TCGA-HT-7472-01A-11R-2027-07 TCGA-19-1389-02A-21R-2005-01 TCGA-CS-6669-01A-11R-1896-07 TCGA-HT-7474-01A-11R-2027-07 TCGA-DH-A7UV-01A-12R-A34F-07 TCGA-FG-6691-01A-11R-1896-07 TCGA-TQ-A8XE-02A-11R-A36H-07 TCGA-28-5218-01A-01R-1850-01 TCGA-CS-5394-01A-01R-1470-07 TCGA-28-1753-01A-01R-1850-01 TCGA-HT-7677-01A-11R-2256-07 TCGA-HT-7686-01A-11R-2256-07 TCGA-HT-7616-01A-11R-2256-07 TCGA-WH-A86K-01A-11R-A36H-07 TCGA-FG-8189-01B-11R-A28M-07 TCGA-W9-A837-01A-11R-A36H-07 TCGA-HT-7479-01A-11R-2027-07 TCGA-QH-A6CZ-01A-11R-A32Q-07 TCGA-S9-A7R2-01A-21R-A34R-07 TCGA-DU-8164-01A-11R-2256-07 TCGA-06-0745-01A-01R-1849-01 TCGA-HT-7681-01A-11R-2403-07 TCGA-QH-A65R-01A-21R-A31N-07 TCGA-HT-7857-01A-11R-2403-07 TCGA-S9-A7R4-01A-12R-A34R-07 TCGA-06-2561-01A-02R-1849-01 TCGA-FG-6692-01A-11R-1896-07 TCGA-27-1830-01A-01R-1850-01 TCGA-HT-8013-01A-11R-2403-07 TCGA-TQ-A7RO-01A-11R-A33Z-07 TCGA-P5-A730-01A-11R-A32Q-07 TCGA-06-0184-01A-01R-1849-01 TCGA-FG-5965-01B-11R-1896-07 TCGA-19-4065-01A-01R-2005-01 TCGA-S9-A7QZ-01A-12R-A34R-07 TCGA-HT-7480-01A-11R-2090-07 TCGA-CS-6668-01A-11R-1896-07 TCGA-DU-A6S7-01A-21R-A32Q-07 TCGA-02-2485-01A-01R-1849-01 TCGA-FG-A87N-01A-11R-A36H-07 TCGA-FG-8191-01A-11R-2256-07 TCGA-E1-A7YI-01A-11R-A34F-07 TCGA-DU-A7TA-01A-11R-A33Z-07 TCGA-06-0132-01A-02R-1849-01 TCGA-32-1970-01A-01R-1850-01 TCGA-06-5416-01A-01R-1849-01 TCGA-DU-A76O-01A-11R-A32Q-07 TCGA-DU-5872-02A-21R-A36H-07 TCGA-HT-A617-01A-11R-A29R-07 TCGA-DB-5276-01A-01R-1470-07 TCGA-06-0210-01A-01R-1849-01 TCGA-06-0210-02A-01R-2005-01 TCGA-DU-7304-01A-12R-2090-07 TCGA-HT-7860-01A-11R-2403-07 TCGA-06-0139-01A-01R-1849-01 TCGA-06-5411-01A-01R-1849-01 TCGA-DH-A66F-01A-11R-A29R-07 TCGA-CS-4942-01A-01R-1470-07 TCGA-76-4931-01A-01R-1850-01 TCGA-HT-7676-01A-11R-2403-07 TCGA-FG-5965-02B-11R-A29R-07 TCGA-HT-7880-01A-11R-2403-07 TCGA-TM-A84B-01A-11R-A36H-07 TCGA-HT-7608-01A-11R-2090-07 TCGA-14-1034-02B-01R-2005-01 TCGA-FG-7641-01B-11R-2256-07 TCGA-19-4065-02A-11R-2005-01 TCGA-06-5410-01A-01R-1849-01 TCGA-FG-A70Z-01A-12R-A33Z-07 TCGA-E1-5319-01A-01R-1896-07 TCGA-F6-A8O4-01A-11R-A36H-07 TCGA-CS-6186-01A-12R-2027-07 TCGA-VM-A8CB-01A-11R-A36H-07 TCGA-VM-A8C9-01A-11R-A36H-07 TCGA-HT-7858-01A-11R-2403-07 TCGA-HT-7690-01A-11R-2256-07 TCGA-CS-5390-01A-02R-1470-07 TCGA-HT-7601-01A-11R-2090-07 TCGA-28-5213-01A-01R-1850-01 TCGA-E1-5302-01A-01R-1470-07 TCGA-HT-7694-01A-11R-2256-07 TCGA-06-2563-01A-01R-1849-01 TCGA-DU-7011-01A-11R-2027-07 TCGA-06-2557-01A-01R-1849-01 TCGA-TQ-A7RV-02A-11R-A36H-07 TCGA-E1-A7YO-01A-11R-A34F-07 TCGA-76-4927-01A-01R-1850-01 TCGA-S9-A6WO-01A-21R-A34F-07 TCGA-HT-A5RB-01A-11R-A28M-07 TCGA-QH-A6XC-01A-12R-A32Q-07 TCGA-FG-A6J1-01A-11R-A31N-07 TCGA-F6-A8O3-01A-11R-A36H-07 TCGA-S9-A6WM-01A-12R-A33Z-07 TCGA-CS-6666-01A-11R-1896-07 TCGA-HT-7481-01A-11R-2027-07 TCGA-DU-7306-01A-11R-2090-07 TCGA-HW-7489-01A-11R-2027-07 TCGA-HT-7695-01A-11R-2256-07 TCGA-06-0178-01A-01R-1849-01 TCGA-41-3915-01A-01R-1850-01 TCGA-DU-A5TW-01A-11R-A28M-07 TCGA-06-1804-01A-01R-1849-01 TCGA-VW-A8FI-01A-11R-A36H-07 TCGA-HT-A5R7-01A-11R-A28M-07 TCGA-12-0619-01A-01R-1849-01 TCGA-S9-A7QY-01A-11R-A34F-07 TCGA-14-0781-01B-01R-1849-01 TCGA-HT-7856-01A-11R-2403-07 TCGA-32-2632-01A-01R-1850-01 TCGA-HT-7879-01A-11R-2403-07 TCGA-DB-A4XC-01A-11R-A26U-07 TCGA-FG-A4MX-01A-11R-A26U-07 TCGA-06-0129-01A-01R-1849-01 TCGA-WY-A858-01A-11R-A36H-07 TCGA-HT-7875-01A-11R-2403-07 TCGA-DU-A7TG-01A-21R-A34R-07 TCGA-HT-7469-01A-11R-2256-07 TCGA-06-2562-01A-01R-1849-01 TCGA-DU-7010-01A-11R-2027-07 TCGA-FG-A87Q-01A-11R-A36H-07 TCGA-HT-7606-01A-11R-2090-07 TCGA-DB-5278-01A-01R-1470-07 TCGA-DU-5847-01A-11R-1708-07 TCGA-S9-A7R1-01A-12R-A34R-07 TCGA-RY-A847-01A-11R-A36H-07 TCGA-S9-A7R7-01A-11R-A34R-07 TCGA-19-0957-02A-11R-2005-01 TCGA-27-2519-01A-01R-1850-01 TCGA-HT-7687-01A-11R-2256-07 TCGA-41-4097-01A-01R-1850-01 TCGA-DU-6542-01A-11R-1896-07 TCGA-HT-7882-01A-11R-2403-07 TCGA-HT-7483-01A-11R-2027-07 TCGA-DB-A75P-01A-11R-A32Q-07 TCGA-VM-A8C8-01A-11R-A36H-07 TCGA-E1-A7YN-01A-11R-A34F-07 TCGA-HT-A5RA-01A-11R-A28M-07 TCGA-DU-A6S8-01A-12R-A32Q-07 TCGA-DH-A7UT-01A-12R-A34F-07 TCGA-27-2528-01A-01R-1850-01 TCGA-RY-A845-01A-11R-A36H-07 TCGA-16-0846-01A-01R-1850-01 TCGA-S9-A6WN-01A-12R-A33Z-07 TCGA-06-0171-02A-11R-2005-01 TCGA-DU-7292-01A-11R-2027-07 TCGA-DU-5851-01A-13R-1896-07 TCGA-06-0125-01A-01R-1849-01 TCGA-02-0047-01A-01R-1849-01 TCGA-P5-A5EV-01A-11R-A27Q-07 TCGA-TM-A84G-01A-11R-A36H-07 TCGA-QH-A65V-01A-11R-A29R-07 TCGA-DU-8166-01A-11R-2256-07 TCGA-12-3652-01A-01R-1849-01 TCGA-DB-A75M-01A-11R-A32Q-07 TCGA-06-2565-01A-01R-1849-01 TCGA-14-2554-01A-01R-1850-01 TCGA-S9-A6TV-01A-12R-A34R-07 TCGA-DU-5855-01A-11R-1708-07 TCGA-DU-A76K-01A-11R-A33Z-07 TCGA-14-0871-01A-01R-1849-01 TCGA-06-5408-01A-01R-1849-01 TCGA-TM-A84F-01A-11R-A36H-07 TCGA-DU-5853-01A-11R-1896-07 TCGA-19-2625-01A-01R-1850-01 TCGA-TM-A84T-01A-11R-A36H-07 TCGA-E1-A7YD-01A-11R-A34F-07 TCGA-27-1832-01A-01R-1850-01 TCGA-WY-A85C-01A-11R-A36H-07 TCGA-DU-7006-01A-11R-2027-07 TCGA-06-0190-02A-01R-2005-01 TCGA-26-5132-01A-01R-1850-01 TCGA-HW-7487-01A-11R-2027-07 TCGA-26-5133-01A-01R-1850-01 TCGA-TQ-A7RG-01A-11R-A33Z-07 TCGA-DB-A4XA-01A-11R-A26U-07 TCGA-32-2615-01A-01R-1850-01 TCGA-DU-7013-01A-11R-2027-07 TCGA-HT-7602-01A-21R-2090-07 TCGA-P5-A5EY-01A-11R-A27Q-07 TCGA-HT-8563-01A-11R-2404-07 TCGA-E1-5304-01A-01R-1470-07 TCGA-DU-A7TC-01A-21R-A34R-07 TCGA-DU-7299-01A-21R-2027-07 TCGA-DU-A5TT-01A-11R-A28M-07 TCGA-TM-A84S-01A-11R-A36H-07 TCGA-HT-7691-01A-11R-2256-07 TCGA-DB-A4XG-01A-11R-A27Q-07 TCGA-06-2567-01A-01R-1849-01 TCGA-27-2521-01A-01R-1850-01 TCGA-S9-A6TZ-01A-21R-A32Q-07 TCGA-S9-A6U5-01A-12R-A33Z-07 TCGA-HT-7855-01A-11R-2403-07 TCGA-06-0646-01A-01R-1849-01 TCGA-06-0211-02A-02R-2005-01 TCGA-E1-A7Z4-01A-11R-A34R-07 TCGA-S9-A6U9-01A-11R-A32Q-07 TCGA-DH-5143-01A-01R-1470-07 TCGA-FN-7833-01A-11R-2090-07 TCGA-HW-8322-01A-11R-2404-07 TCGA-IK-7675-01A-11R-2090-07 TCGA-FG-7634-01A-11R-2090-07 TCGA-DU-6396-01A-11R-1708-07 TCGA-RY-A840-01A-11R-A36H-07 TCGA-KT-A74X-01A-11R-A32Q-07 TCGA-P5-A5EW-01A-11R-A27Q-07 TCGA-06-2558-01A-01R-1849-01 TCGA-41-5651-01A-01R-1850-01 TCGA-06-0211-01B-01R-1849-01 TCGA-TQ-A7RP-01A-21R-A34F-07 TCGA-S9-A7IQ-01A-21R-A34F-07 TCGA-DU-6410-01A-11R-1896-07 TCGA-HT-8564-01A-11R-2404-07 TCGA-TM-A7C4-01A-11R-A32Q-07 TCGA-DH-A669-02A-11R-A31N-07 TCGA-S9-A6WH-01A-12R-A33Z-07 TCGA-S9-A6WP-01A-12R-A34F-07 TCGA-E1-A7YQ-01A-11R-A34R-07 TCGA-S9-A6TW-01A-12R-A32Q-07 TCGA-DU-6404-02A-21R-A36H-07 TCGA-S9-A7IY-01A-11R-A34F-07 TCGA-DB-A75O-01A-11R-A32Q-07 TCGA-DB-5279-01A-03R-2347-07 TCGA-06-0219-01A-01R-1849-01 TCGA-QH-A6CS-01A-11R-A31N-07 TCGA-S9-A6WI-01A-21R-A33Z-07 TCGA-S9-A6WL-01A-21R-A33Z-07 TCGA-HT-8105-01A-11R-2404-07 TCGA-TM-A84R-01A-21R-A36H-07 TCGA-DU-6406-01A-11R-1708-07 TCGA-QH-A6X3-01A-21R-A32Q-07 TCGA-06-0686-01A-01R-1849-01 TCGA-06-5859-01A-01R-1849-01 TCGA-FG-8185-01A-11R-2256-07 TCGA-HW-8319-01A-11R-2404-07 TCGA-CS-4941-01A-01R-1470-07 TCGA-28-1747-01C-01R-1850-01 TCGA-S9-A6TX-01A-21R-A32Q-07 TCGA-TM-A84I-01A-11R-A36H-07 TCGA-CS-6665-01A-11R-1896-07 TCGA-VV-A829-01A-21R-A36H-07 TCGA-QH-A65X-01A-11R-A32Q-07 TCGA-06-5415-01A-01R-1849-01 TCGA-FG-A4MT-02A-11R-A29R-07 TCGA-P5-A5F4-01A-11R-A28M-07 TCGA-27-1831-01A-01R-1850-01 TCGA-S9-A6U2-01A-21R-A33Z-07 TCGA-FG-A60L-01A-12R-A31N-07 TCGA-VM-A8CF-01A-11R-A36H-07 TCGA-19-2624-01A-01R-1850-01 TCGA-26-1442-01A-01R-1850-01 TCGA-15-0742-01A-01R-1850-01 TCGA-06-0168-01A-01R-1849-01 TCGA-FG-A4MW-01A-11R-A26U-07 TCGA-19-2629-01A-01R-1850-01 TCGA-TQ-A7RH-01A-12R-A34F-07 TCGA-TQ-A7RQ-01A-11R-A33Z-07 TCGA-CS-6290-01A-11R-1708-07 TCGA-HT-A74K-01A-11R-A32Q-07 TCGA-S9-A6U6-01A-12R-A33Z-07 TCGA-CS-4938-01B-11R-1896-07 TCGA-HT-7680-01A-11R-2256-07 TCGA-RY-A83X-01A-11R-A36H-07 TCGA-28-5216-01A-01R-1850-01 TCGA-HW-7486-01A-11R-2027-07 TCGA-DU-6392-01A-11R-1708-07 TCGA-DU-7302-01A-11R-2090-07 TCGA-14-1825-01A-01R-1850-01 TCGA-DB-5274-01A-01R-1470-07 TCGA-TM-A7CF-01A-11R-A32Q-07 TCGA-HT-A614-01A-11R-A29R-07 TCGA-HT-7881-01A-11R-2403-07 TCGA-12-5299-01A-02R-1849-01 TCGA-DU-6405-01A-11R-1708-07 TCGA-FG-5963-02A-12R-A29R-07 TCGA-DU-5849-01A-11R-1708-07 TCGA-HT-7471-01A-11R-2256-07 TCGA-DB-A75L-01A-11R-A32Q-07 TCGA-DU-8165-01A-11R-2256-07 TCGA-TQ-A7RK-02A-11R-A36H-07 TCGA-DU-6403-01A-11R-1708-07 TCGA-VM-A8CA-01A-11R-A36H-07 TCGA-E1-A7Z3-01A-11R-A34R-07 TCGA-DB-A4XB-01A-11R-A26U-07 TCGA-FG-8186-01A-11R-2256-07 TCGA-DU-6404-01A-11R-1708-07 TCGA-P5-A5EX-01A-12R-A28M-07 TCGA-FG-A710-01A-12R-A33Z-07 TCGA-HT-8012-01A-11R-2403-07 TCGA-DB-5277-01A-01R-1470-07 TCGA-TM-A84L-01A-11R-A36H-07 TCGA-HT-A74L-01A-11R-A32Q-07 TCGA-FG-A711-01A-21R-A33Z-07 TCGA-P5-A731-01A-11R-A32Q-07 TCGA-E1-5305-01A-01R-1896-07 TCGA-P5-A736-01A-11R-A32Q-07 TCGA-DU-6395-01A-13R-1708-07 TCGA-S9-A7J1-01A-21R-A34R-07 TCGA-P5-A77X-01A-11R-A32Q-07 TCGA-06-0130-01A-01R-1849-01 TCGA-TQ-A7RV-01A-21R-A34F-07 TCGA-06-5412-01A-01R-1849-01 TCGA-76-4926-01B-01R-1850-01 TCGA-HT-7620-01A-11R-2256-07 TCGA-TQ-A7RW-01A-11R-A33Z-07 TCGA-DU-7300-01A-21R-2090-07 TCGA-E1-5322-01A-01R-1470-07 TCGA-14-0789-01A-01R-1849-01 TCGA-TM-A84J-01A-11R-A36H-07 TCGA-06-5417-01A-01R-1849-01 TCGA-06-6698-01A-13T-1900-53 TCGA-E1-A7YU-01A-11R-A34R-07 TCGA-DU-7008-01A-11R-2027-07 TCGA-VM-A8CH-01A-12R-A36H-07 TCGA-DU-7309-01A-11R-2090-07 TCGA-HT-7475-01A-11R-2027-07 TCGA-DU-6400-01A-12R-1708-07 TCGA-P5-A5F0-01A-11R-A28M-07 TCGA-76-4929-01A-01R-1850-01 TCGA-12-0618-01A-01R-1849-01 TCGA-HW-A5KJ-01A-12R-A27Q-07 TCGA-P5-A5EU-01A-11R-A27Q-07 TCGA-06-0190-01A-01R-1849-01 TCGA-E1-5307-01A-01R-1896-07 TCGA-P5-A72Z-01A-11R-A32Q-07 TCGA-QH-A6XA-01A-12R-A32Q-07 TCGA-QH-A6X9-01A-12R-A32Q-07 TCGA-TM-A84M-01A-11R-A36H-07 TCGA-DU-A7T6-01A-11R-A33Z-07 TCGA-CS-4943-01A-01R-1470-07 TCGA-06-0211-01A-01R-1849-01 TCGA-P5-A733-01A-11R-A32Q-07 TCGA-TQ-A7RI-01A-11R-A33Z-07 TCGA-76-4925-01A-01R-1850-01 TCGA-HT-8019-01A-21R-2404-07 TCGA-14-0817-01A-01R-1849-01 TCGA-DU-7009-01A-11R-2027-07 TCGA-06-0187-01A-01R-1849-01 TCGA-HT-7482-01A-11R-2027-07 TCGA-DU-6397-02A-12R-A36H-07 TCGA-FG-A60J-01A-11R-A28M-07 TCGA-26-5135-01A-01R-1850-01 TCGA-HT-A5R5-01A-11R-A28M-07 TCGA-FG-8181-01A-11R-2256-07 TCGA-06-0649-01B-01R-1849-01 TCGA-HT-7607-01A-11R-2090-07 TCGA-DU-7007-01A-11R-2027-07 TCGA-28-2510-01A-01R-1850-01 TCGA-DB-A64U-01A-11R-A29R-07 TCGA-DH-A669-01A-12R-A31N-07 TCGA-S9-A6WQ-01A-12R-A34F-07 TCGA-VM-A8CE-01A-11R-A36H-07 TCGA-FG-7636-01A-11R-2090-07 TCGA-06-5418-01A-01R-1849-01 TCGA-DB-A64V-01A-11R-A29R-07 TCGA-QH-A86X-01A-11R-A36H-07 TCGA-HT-A5R9-01A-11R-A28M-07 TCGA-HW-A5KK-01A-11R-A27Q-07 TCGA-14-0736-02A-01R-2005-01 TCGA-06-2564-01A-01R-1849-01 TCGA-DU-6404-02B-11R-A36H-07 TCGA-DU-7290-01A-11R-2027-07 TCGA-DU-5854-01A-11R-1708-07 TCGA-DU-7019-01A-11R-2027-07 TCGA-HT-7604-01A-11R-2090-07 TCGA-VM-A8CD-01A-11R-A36H-07 TCGA-FG-5964-01A-11R-1708-07 TCGA-DB-A75K-01A-11R-A32Q-07 TCGA-06-0743-01A-01R-1849-01 TCGA-DB-A64Q-01A-11R-A29R-07 TCGA-P5-A5F2-01A-11R-A28M-07 TCGA-QH-A6CV-01A-11R-A31N-07 TCGA-S9-A7QW-01A-11R-A34F-07 TCGA-DU-A7T8-01A-21R-A34R-07 TCGA-06-2570-01A-01R-1849-01 TCGA-HT-7609-01A-11R-2090-07 TCGA-12-0821-01A-01R-1849-01 TCGA-06-0157-01A-01R-1849-01 TCGA-DH-A7US-01A-11R-A33Z-07 TCGA-DU-7014-01A-11R-2027-07 TCGA-HT-7688-01A-11R-2256-07 TCGA-HT-A4DS-01A-11R-A26U-07 TCGA-E1-A7YY-01A-11R-A34R-07 TCGA-DU-A6S2-01A-21R-A32Q-07 TCGA-TQ-A8XE-01A-11R-A36H-07 TCGA-E1-A7YW-01A-11R-A34R-07 TCGA-27-2526-01A-01R-1850-01 TCGA-DU-6407-01A-13R-1708-07 TCGA-DB-A4XH-01A-11R-A27Q-07 TCGA-DB-5280-01A-01R-1470-07 TCGA-S9-A7J3-01A-21R-A34R-07 TCGA-DU-6394-01A-11R-1708-07 TCGA-06-0221-02A-11R-2005-01 TCGA-E1-A7Z2-01A-21R-A34R-07 TCGA-TQ-A7RS-01A-12R-A33Z-07 TCGA-28-5220-01A-01R-1850-01 TCGA-DU-7294-01A-11R-2027-07 TCGA-S9-A6TS-01A-12R-A33Z-07 TCGA-QH-A6CX-01A-11R-A32Q-07 TCGA-P5-A5EZ-01A-11R-A27Q-07 TCGA-HT-8010-01A-11R-2403-07 TCGA-WY-A85B-01A-11R-A36H-07 TCGA-FG-6690-01A-11R-1896-07 TCGA-TQ-A7RK-01A-11R-A33Z-07 TCGA-06-0174-01A-01R-1849-01 TCGA-DH-A7UU-01A-12R-A34F-07 TCGA-DB-A64L-01A-11R-A29R-07 TCGA-FG-A60K-01A-11R-A29R-07 TCGA-HT-8106-01A-11R-2404-07 TCGA-WY-A85D-01A-11R-A36H-07 TCGA-06-0878-01A-01R-1849-01 TCGA-HT-A61A-01A-11R-A29R-07 TCGA-12-3650-01A-01R-1849-01 TCGA-E1-A7YJ-01A-11R-A34F-07 TCGA-P5-A781-01A-11R-A32Q-07 TCGA-P5-A72U-01A-31R-A32Q-07 TCGA-FG-A6IZ-01A-11R-A31N-07 TCGA-TM-A84Q-01A-11R-A36H-07 TCGA-06-0156-01A-03R-1849-01 TCGA-08-0386-01A-01R-1849-01 TCGA-CS-4944-01A-01R-1470-07 TCGA-HT-7877-01A-11R-2403-07 TCGA-S9-A7IZ-01A-11R-A34F-07 TCGA-QH-A6X5-01A-12R-A32Q-07 TCGA-32-2634-01A-01R-1850-01 TCGA-DU-7015-01A-11R-2027-07 TCGA-WY-A85E-01A-11R-A36H-07 TCGA-02-0055-01A-01R-1849-01 TCGA-32-2638-01A-01R-1850-01 TCGA-FG-A70Y-01A-12R-A34R-07 TCGA-DB-5273-01A-01R-1470-07 TCGA-HW-7491-01A-11R-2027-07 TCGA-DU-8168-01A-11R-2256-07 TCGA-HW-8320-01A-11R-2404-07 TCGA-HW-A5KL-01A-11R-A27Q-07 TCGA-FG-8182-01A-11R-2256-07 TCGA-FG-A713-01A-11R-A32Q-07 TCGA-HT-8114-01A-11R-2404-07 TCGA-HW-7493-01A-11R-2027-07 TCGA-DB-A64W-01A-11R-A29R-07 TCGA-DU-A6S3-01A-12R-A32Q-07 TCGA-28-2499-01A-01R-1850-01 TCGA-IK-8125-01A-11R-2256-07 TCGA-HW-8321-01A-11R-2404-07 TCGA-KT-A7W1-01A-11R-A34F-07 TCGA-19-1390-01A-01R-1850-01 TCGA-14-1823-01A-01R-1849-01 TCGA-FG-5963-01A-11R-1708-07 TCGA-DU-A7TJ-01A-11R-A34R-07 TCGA-FG-5965-02A-11R-A29R-07 TCGA-HT-8113-01A-11R-2404-07 TCGA-DU-8162-01A-21R-2256-07 TCGA-HT-7873-01B-11R-2403-07 TCGA-DU-A5TR-01A-11R-A28M-07 TCGA-12-0616-01A-01R-1849-01 TCGA-S9-A6U8-01A-21R-A33Z-07 TCGA-HT-7854-01A-11R-2256-07 TCGA-HT-7476-01A-11R-2027-07 TCGA-06-0152-02A-01R-2005-01 TCGA-HT-8011-01A-11R-2403-07 TCGA-DU-A5TS-01A-11R-A28M-07 TCGA-TQ-A7RR-01A-21R-A34F-07 TCGA-S9-A7J2-01A-11R-A34F-07 TCGA-19-2620-01A-01R-1850-01 TCGA-14-1829-01A-01R-1850-01 TCGA-HT-7689-01A-11R-2256-07 TCGA-FG-6688-01A-11R-1896-07 TCGA-R8-A6ML-01A-11R-A32Q-07 TCGA-DB-A64X-01A-11R-A29R-07 TCGA-S9-A7QX-01A-11R-A34F-07 TCGA-HT-A615-01A-11R-A29R-07 TCGA-41-2572-01A-01R-1850-01 TCGA-HT-A4DV-01A-11R-A26U-07 TCGA-TQ-A7RJ-01A-11R-A33Z-07 TCGA-E1-A7YS-01A-11R-A34F-07 TCGA-26-5134-01A-01R-1850-01 TCGA-02-2486-01A-01R-1849-01 TCGA-HW-7495-01A-11R-2027-07 TCGA-TM-A7CA-01A-21R-A33Z-07 TCGA-DH-5142-01A-01R-1470-07 TCGA-TQ-A7RN-01A-11R-A33Z-07 TCGA-VW-A7QS-01A-12R-A33Z-07 TCGA-DU-A76R-01A-11R-A32Q-07 TCGA-DB-5281-01A-01R-1470-07 TCGA-TM-A84H-01A-11R-A36H-07 TCGA-41-2571-01A-01R-1850-01 TCGA-CS-5395-01A-01R-1470-07 TCGA-P5-A5F1-01A-11R-A28M-07 TCGA-HT-7477-01B-11R-A28M-07 TCGA-DU-7012-01A-11R-2027-07 TCGA-HT-8109-01A-11R-2404-07 TCGA-R8-A73M-01A-11R-A32Q-07 TCGA-06-0141-01A-01R-1849-01 TCGA-26-5139-01A-01R-1850-01 TCGA-HW-A5KM-01A-11R-A27Q-07 TCGA-S9-A7R3-01A-11R-A34R-07 TCGA-TQ-A7RU-01A-21R-A34F-07 TCGA-S9-A6TY-01A-12R-A32Q-07 TCGA-HT-7473-01A-11R-2027-07 TCGA-DU-8167-01A-11R-2256-07 TCGA-06-5856-01A-01R-1849-01 TCGA-HT-8111-01A-11R-2404-07 TCGA-S9-A89Z-01A-11R-A36H-07 TCGA-DU-6402-01A-11R-1708-07 TCGA-HT-7470-01A-12R-2090-07 TCGA-QH-A6X8-01A-12R-A32Q-07 TCGA-DU-A5TY-01A-11R-A28M-07 TCGA-DU-8163-01A-11R-2256-07 TCGA-DU-A7TD-01A-12R-A34F-07

aDCs 0.670059035508684 0.637079450817983 0.592028999267237 0.696522670807231 0.713991494598922 0.699581280671095 0.670591711230102 0.687895662176161 0.670968106880711 0.679935024979891 0.72313308572879 0.656502602290301 0.61499477974811 0.553192609916601 0.648080725112415 0.626173842198106 0.662788016802723 0.626894171749395 0.632983006321845 0.599043517442573 0.542220835171761 0.641404309222485 0.611503747734802 0.640764934513667 0.567403803308979 0.628962912697575 0.653866579320432 0.577802729909189 0.59187132524614 0.680567693511086 0.6690435186874 0.637992872984382 0.618870727879235 0.628250371768774 0.683247578523492 0.677285468904437 0.674199431157397 0.685004704476156 0.615553915120319 0.656254172503204 0.623700564521235 0.653735449678516 0.646804845368447 0.658195482855957 0.621624499000353 0.566416828545129 0.624483452784007 0.589251681445544 0.667686318965588 0.554692608479858 0.634235879221837 0.512101235148495 0.649029972861983 0.677833511812705 0.677448345448645 0.649261519193386 0.49722728145396 0.686615537689004 0.715697844150621 0.604282482639083 0.641455362211388 0.601798534537766 0.613368374586216 0.608044308685542 0.67320679486742 0.692917902906131 0.559326009071217 0.669594104192866 0.646953210566604 0.651199396426739 0.706955537231476 0.641492603326261 0.618914302084117 0.56590755395619 0.620722573689522 0.721782407831437 0.592058304647518 0.676891798627135 0.578530794029843 0.715495357288905 0.625602394529387 0.679857000262451 0.696236997851644 0.644356793350141 0.634470250773681 0.578571248724 0.622082640120528 0.600417107262769 0.613502943927827 0.716857254512229 0.578207994179362 0.605532783781328 0.561244320642585 0.657397641721224 0.660170587137532 0.581925688943064 0.68503201764264 0.684443228151275 0.582491387547792 0.684915263060535 0.6373776771203 0.625939999932702 0.627660491815035 0.622640874181907 0.524153947722513 0.616939644244477 0.601462653719923 0.643508022208226 0.681784939811866 0.65851300305244 0.5985844231524 0.586851220974312 0.576127672296624 0.688389468210752 0.615622532920945 0.635402206155578 0.671894079836891 0.645758096538017 0.562396749166502 0.675003903264605 0.66686166623053 0.664050842899385 0.671868571975471 0.63221841301649 0.714630770040316 0.726334054368921 0.635266449318679 0.593168002703075 0.593206673565354 0.53974257082928 0.616386289318933 0.645364148874985 0.69442562751029 0.737770446578104 0.742105469747154 0.711434437762429 0.571832040694563 0.696523750313484 0.655621961737212 0.592260158978759 0.64958357432626 0.666816034207005 0.656542421016588 0.650759004212944 0.598704906537178 0.633471529989173 0.582316777922071 0.682658065650803 0.597612164474485 0.621947937042832 0.672331370593993 0.590573856398964 0.606615367206673 0.706782338234934 0.669380413810971 0.68553638088861 0.662045433498769 0.722848419182902 0.657661953407233 0.602495308403798 0.557204529106012 0.603760737885557 0.616230488710492 0.586690215989586 0.644893634156553 0.568190236254463 0.623862911163677 0.684809468039476 0.626225644816914 0.621235808841569 0.706867841481678 0.720612688424899 0.561253581069913 0.589257007032396 0.706373055275284 0.631154655340242 0.604021108520214 0.621459043953099 0.581852183108175 0.611021544442787 0.633827331956732 0.583535088619576 0.680643072990573 0.619880223636173 0.670593350504485 0.754315136906004 0.626854817391753 0.557625897122915 0.625731580902664 0.74851757341467 0.562438343575555 0.640706670968418 0.537688689986804 0.614781850869433 0.703878638857181 0.742994080458669 0.70014693766162 0.574566479599798 0.584256821333131 0.727784854144628 0.665322833642662 0.652022683232621 0.621131399401055 0.720872174078436 0.642326837503028 0.59022319227753 0.567870870226873 0.659266710953573 0.621948536204796 0.613545274343533 0.619942199784729 0.69835336166586 0.646940675183441 0.649371461562199 0.763762810571847 0.635226194026758 0.641353779967402 0.604348053761695 0.633094413418649 0.627290191301485 0.596392153364761 0.613144607867974 0.703565930530386 0.663019424566704 0.647732328576168 0.718087652455121 0.630839804813534 0.62553086568507 0.606408464469301 0.65331545397321 0.630676678126876 0.625660487298303 0.588936043887303 0.628227764557231 0.696970164194899 0.630713764465023 0.655599769606584 0.593505140326281 0.645292013263847 0.554877868235422 0.709106760550854 0.594024834401802 0.725618782827919 0.547115347810511 0.735938278859495 0.709516775764031 0.661162285176299 0.664330977036773 0.564379197206493 0.787458353736147 0.625478763159599 0.647577521171471 0.693923088992711 0.717294491755621 0.692429227106856 0.726853369346179 0.712476666703856 0.674565850942695 0.695668564212148 0.707082728296358 0.589155000017611 0.597585648198687 0.653410645869995 0.649325298711468 0.645772360858616 0.74185295068118 0.688972627614286 0.589348500262627 0.703838645858348 0.659358013512938 0.706045527532837 0.723509210438369 0.592073686918356 0.572935385295366 0.668011085466026 0.635234647211385 0.631853399717959 0.692768450636818 0.652063920023211 0.624852365982184 0.657215703772201 0.660114300099322 0.693531704439467 0.671705331672487 0.660721497753301 0.680207549786014 0.630071462945777 0.629880739951237 0.642257104183947 0.589744437493717 0.564534758410778 0.594511498961251 0.671504493519924 0.664749269490117 0.653029623054157 0.60590149219168 0.587229530650324 0.623068237855139 0.601525930862435 0.640632683042924 0.648128881314702 0.666750924948578 0.571732855830913 0.650084198011431 0.6364505581337 0.612672068265428 0.659314121430329 0.57275791191421 0.669529769360252 0.626527323852195 0.653197472407418 0.566554799332378 0.591588147464475 0.577187298584564 0.655567585438168 0.595902282982194 0.548050231108564 0.695301114019444 0.572577830856747 0.67743053370128 0.6000274350009 0.626406064989831 0.667925125334652 0.623808120914726 0.667752066045381 0.736356263413163 0.661368064118582 0.585016409924383 0.69107941417652 0.71388375135926 0.706059447437909 0.634578074069622 0.699076231120601 0.751543863834938 0.719228502489203 0.673521164878852 0.620664875422907 0.708894665581576 0.675994235067735 0.579039450039917 0.591699621821659 0.669512585676487 0.617285416951907 0.708442604185148 0.768172659087959 0.732555849462042 0.564916760077294 0.596072749041825 0.623383413067478 0.664826664904914 0.609505212607043 0.57878592658945 0.56510247474752 0.717760835753083 0.604579410369719 0.598115366831815 0.67929637666615 0.638535619204216 0.616927995170187 0.7076334555764 0.686652639147462 0.609379448793118 0.660008948124966 0.696612319491625 0.68433252879743 0.600147265787423 0.531543477983602 0.679632325472735 0.666661389005558 0.579133355975209 0.654210298165295 0.712333133535591 0.671423380780598 0.653646965392716 0.65505978033461 0.654499969625621 0.655651440985764 0.682246368671466 0.695082133281295 0.630518305002353 0.606921635077521 0.66071079372998 0.618226576865852 0.630999035271947 0.628331225075924 0.64861175264568 0.616476469249513 0.766103390753578 0.642066402600297 0.656599190639694 0.617466672292407 0.648722504407517 0.732520179470361 0.620909270611797 0.702462754461491 0.699168332675819 0.586731397542047 0.633389959283307 0.678044338392036 0.714509450267275 0.565879423493945 0.527399460022038 0.644581614824987 0.74312986827395 0.614319819486036 0.591125860674557 0.700857953184422 0.634963259743648 0.621367858734396 0.636402089532044 0.598223392930629 0.566178439885574 0.598790466691013 0.646706460384833 0.582818251682871 0.653659396778888 0.625772167229287 0.6715014301176 0.670761161463157 0.634369789805107 0.686775378019715 0.69772969132588 0.622083808667428 0.812939423880994 0.594431702753533 0.659269265944667 0.652915059834589 0.675251861156309 0.707254723222278 0.627956369625196 0.610185345386394 0.5590008622287 0.617188638058765 0.648036695740401 0.644985362170772 0.626414464419605 0.6633550884906 0.667112857534651 0.631548382822428 0.57179664763737 0.703496278074608 0.722959363031864 0.642612821787486 0.600507497008464 0.613186375968957 0.594701869926312 0.657703822558936 0.578903286509827 0.61621098758787 0.621609711422202 0.579134521496302 0.605590317520327 0.653115859264359 0.710392950691307 0.546758834476528 0.684570015936662 0.608252726051517 0.71668272300025 0.708168745864046 0.712393729183888 0.65755731857148 0.635169739977356 0.670998041588806 0.5708305527515 0.66609239668721 0.622803508806411 0.645472442840259 0.738688408058201 0.625665621098594 0.658873708877606 0.546969782920673 0.591167965167407 0.557868322094435 0.681511326563926 0.671409066204429 0.717409563525133 0.633253421249747 0.697762542498869 0.604991406894375 0.686934124091275 0.596281122182504 0.647521894191168 0.743661810780746 0.730669580848223 0.688991436930239 0.70226083819402 0.687927158678973 0.797723781890687 0.744254420798701 0.710234371003452 0.582056248052351 0.67695716149279 0.58660521959284 0.718044175880224 0.64230557803892 0.582818998790653 0.6649009167369 0.722474307107479 0.76089242930874 0.629598967952253 0.728122680379799 0.539202564012487 0.690623137423303 0.661059585016329 0.635136334008781 0.68995913543096 0.670704551583708 0.680803619650647 0.703498078180223 0.629704791649692 0.627775858898533 0.538494032601629 0.620111746144579 0.633761217359557 0.578282482749519 0.636599756800942 0.537159928377129 0.683905709561149 0.675664228707204 0.630566277441048 0.628941107607015 0.66876294887351 0.643588101400957 0.689488887935298 0.619607798680333 0.646530095270432 0.621514486337166 0.605862499959644 0.588554498703064 0.633506523699186 0.644067457145205 0.662755297160468 0.59644523074884 0.640525532930727 0.647240812482221 0.596184503749473 0.703378793723964 0.577750963207645 0.63139522440654 0.637838181523643 0.573214248984493 0.64911883062685 0.67824906908276 0.710129445259483 0.701324320454089 0.622423186816766 0.678114754134421 0.592199411702668 0.640980760721171 0.60684890837609 0.744543521024173 0.629021680207387 0.616258104780582 0.635601031733549 0.655653012147667 0.658769827942553 0.634379216668117 0.585403207128885 0.646924224245066 0.720349605450712 0.646428676201319 0.634043820701441 0.668464812564286 0.656028814430432 0.730417484333549 0.612798395755753 0.755834812682334 0.650096099922139 0.698555911683191 0.668500307347896 0.632382312514253 0.666535066930638 0.616642909899738 0.580080254988425 0.653067269987946 0.604217667605275 0.697685940701732 0.669656354048968 0.65401104854082 0.612484633377434 0.572461818740489 0.604230255753467 0.621967940044196 0.632833627315512 0.684271224702139 0.594736422731924 0.624858202405473 0.769363751276097 0.639295399944744 0.604530820985428 0.581615386777556 0.664598746184733 0.613016234052064 0.606210056487383 0.582796859691175 0.614323098498484 0.663619885599079 0.590293645075426 0.615367319226035 0.619980110091178 0.580393190512156 0.654576953513863 0.598680001021682 0.613212318626043 0.581008178675159 0.677831333266107 0.55662116185654 0.751500738687174 0.517040766727926 0.718399066767335 0.624848130499371 0.696990847912298 0.693920411524377 0.685382366765708 0.59791363267585 0.616133909458641 0.65331415413894 0.571539126245149 0.660542154792682 0.648491490190875 0.654893145059688 0.66387613037263 0.546672815552931 0.640070133479341 0.59042158669296 0.63833441692034 0.670702357073164 0.658966474466034 0.741720081242971 0.638357141521964 0.57957709557109 0.637152377383334 0.568508790827665 0.692040793196123 0.669905345198448 0.560504170586764 0.619515104002848 0.60167981746519 0.670950701424822 0.707847571849771 0.531384541503015 0.524917320907047 0.672789965127046 0.71080464961048 0.673090415171059 0.628508986205153 0.599651792170165 0.701568339177644 0.768727207663672 0.65142854368167 0.660789023571319 0.66794046933125 0.666624692339352 0.580656682910969 0.639255534714636 0.635500479358593 0.591821695022001 0.671841015829306 0.637317229338111 0.790656195956713 0.6102492061481 0.596753125935039 0.607721469649966 0.599912871164143 0.596974775772643 0.695222645276481 0.572097533538946 0.66336975149304 0.660069794108609 0.643228662960869 0.554102875631347 0.670613470433837 0.607165219594126 0.643684827781196 0.661425430870642 0.699851975623676 0.586658785055097 0.747619196324514 0.572942529152042 0.545098217577293 0.654437562365789 0.522276790868228 0.712949860522404 0.696521127188732 0.667294119675339 0.595640437494 0.635855882399368 0.643128154634524 0.627231269273682 0.598711941144912 0.718422117382217 0.56149173874538 0.670736104434554 0.630390955008669 0.692662967139384 0.693064415042533 0.619217356454848 0.536097905369975 0.571261807747061 0.648067560886934 0.653306671198597 0.703857358152756 0.606800293954245 0.650143590230313 0.640407053684722 0.62997437388945 0.578907651066467 0.717066250235838 0.612196001185375 0.662104142896721

APC_co_inhibition 0.71686803731616 0.681249941344952 0.721558639579984 0.682132911045493 0.687645709190378 0.802821889713027 0.837388990925996 0.778892893280536 0.756571505924973 0.81641580923709 0.78129451978646 0.743480105006963 0.653311820418081 0.634593768062539 0.652936531242109 0.803778864198837 0.830309184504155 0.680298732004582 0.640643064015914 0.667714015549631 0.591026585004919 0.838671603808619 0.733073560704174 0.79816636089257 0.802156220144492 0.802845830670179 0.760302515216679 0.636891678848782 0.752744637430179 0.801827700255942 0.838422116607055 0.758244485214909 0.717142198053905 0.841330671195927 0.79647008397943 0.698271207633345 0.742056248752334 0.716327716791165 0.780082084164986 0.800467866826194 0.718060762192391 0.748045675790116 0.590155414322722 0.820079120796666 0.695149058859876 0.741249162077491 0.807598082437363 0.74001671589754 0.823819016994447 0.733489047799005 0.780966298878731 0.675917252821401 0.781899040121262 0.856458220306796 0.640216583547468 0.766699351919858 0.662053291739734 0.844696541712422 0.664104266064605 0.713490723573277 0.844204291976993 0.791766352446153 0.792973145313106 0.807815548309222 0.890493869978194 0.853837799384761 0.670747989843587 0.799306431423336 0.639332653845844 0.841624251677859 0.716087348157036 0.739642561156051 0.754057928051566 0.633264590714957 0.855588826581245 0.690844671614138 0.738784422351634 0.825398018528139 0.725751100155304 0.807778334262818 0.743527231507108 0.821747021651811 0.7543618612094 0.777608819838734 0.822160424407195 0.643019384121268 0.748120516822799 0.75037238323133 0.691008977696625 0.801893226636258 0.690472987213083 0.687094965456737 0.730105095067069 0.825314388029788 0.813412346902316 0.653266225426548 0.70923792430282 0.849274340912431 0.837667296249568 0.833295922448298 0.838010229218077 0.845828081872959 0.73162129180888 0.764991963971975 0.737187326624964 0.780659602519639 0.835674351807338 0.819172137707238 0.800521755766286 0.830993131558599 0.782007443319565 0.748614986813151 0.741449025124961 0.864691535726833 0.765444281374937 0.759318590093534 0.796109164432228 0.822817821636232 0.771901582334729 0.818727256210028 0.77793300620966 0.868302280407967 0.730386551512961 0.808897340526195 0.814208700128521 0.835367279617139 0.788684433316202 0.734477082176808 0.788157806554041 0.738270884581653 0.770221444555958 0.791065362037516 0.809186527979836 0.838911165989656 0.835657290320986 0.743362864570921 0.752345600955211 0.787324164724816 0.694490897655725 0.766516948190067 0.715924681202925 0.838608865632293 0.790480647874876 0.758954975444171 0.790980343974503 0.708797042921377 0.766272334197346 0.71222971329969 0.794135046557694 0.796492831495495 0.645796566723363 0.760183658063374 0.704360717642609 0.729358570012181 0.714192357012768 0.906488885110498 0.796275992344275 0.80529914710968 0.778483175301836 0.698559348747101 0.669032974370959 0.729415822099996 0.700198409184766 0.674875358177393 0.76551307933334 0.737464516758642 0.713229667688786 0.785102441972044 0.759428349819728 0.693512235261447 0.714576403880528 0.847345077252309 0.666128615466084 0.712173134321699 0.86878956583141 0.779377806362308 0.75112561919095 0.706082161991265 0.694368744291117 0.705444985258169 0.784863329680328 0.693036080295626 0.681396696460027 0.807010788548585 0.802367159724735 0.737533918185169 0.835586724487575 0.735240276857888 0.650598748131359 0.770254582249101 0.659649842783082 0.775889160877549 0.715980494100669 0.849258013760575 0.799867315198965 0.789781356548659 0.702620468482488 0.688640807345783 0.723731633486211 0.688387534114288 0.843061045712688 0.66667026716716 0.789437826357446 0.888903723780411 0.664451258712912 0.724365908374409 0.758800265373685 0.818219640469112 0.780095379088668 0.759055768990695 0.638487516933888 0.827703362726104 0.691861377381694 0.826751045387084 0.801608029557177 0.745269513650085 0.656115410708772 0.658140731601503 0.797665724474788 0.674928682124394 0.835715780651859 0.688772552565108 0.73487332093413 0.72672129584656 0.640293843863156 0.932129804830804 0.762932305492255 0.825964882026072 0.792844447694217 0.802296753568415 0.788317363611785 0.668656308630402 0.665702065566577 0.869957788543601 0.739045689518824 0.91149172558336 0.661265474081968 0.695518920036361 0.647500164036424 0.793570654284891 0.818149724630165 0.696021342817853 0.799707116885688 0.68317231248244 0.727035500522964 0.833221474489783 0.811610822723725 0.674601804837346 0.743242134909622 0.875132122630076 0.842703096716388 0.808623817163849 0.816753086606091 0.83191732059307 0.764441833447974 0.87896834329354 0.892154728249706 0.776260686756591 0.668290458617065 0.832633896340215 0.760862459615826 0.804492969562445 0.616802287563845 0.729457802074549 0.757931139212276 0.741933959896781 0.795643613749265 0.620496254400973 0.897058998671598 0.864732034482041 0.792022664343903 0.721277474967631 0.683759979197786 0.817024646602424 0.660476005862018 0.807481180805655 0.771146962500825 0.782480862899593 0.693793752577294 0.827194873649213 0.834406766587601 0.798908175299069 0.693105199024075 0.841709848776121 0.808209689662889 0.823775731408042 0.7296742945829 0.683915774991853 0.8027608118351 0.763364179152495 0.697067549126038 0.78454528399595 0.733083142174105 0.634742185025621 0.835985555534279 0.736376560462407 0.662656730445426 0.792912015075693 0.734558412513496 0.707350127879149 0.771832128372326 0.801104535300438 0.732277121949451 0.784304157379062 0.830250821097212 0.727590268129409 0.846891756058242 0.668517149630473 0.884917769758558 0.709223630635292 0.839632754117548 0.752636901514188 0.745003611118708 0.6791336414987 0.836352103423282 0.812450205360705 0.666216314177064 0.702100382314408 0.74651047191873 0.891605667428106 0.722577667536846 0.864674420793874 0.698849732348587 0.724688115556226 0.826384122809591 0.667214631654758 0.752064833904537 0.808550862740642 0.793245848674301 0.866288910613235 0.724134854596285 0.82443826031847 0.802068600503071 0.873914289597463 0.73596008075861 0.802561821758829 0.760145024685411 0.862493775599278 0.788514597759735 0.651150717394619 0.735123138671039 0.79895026732672 0.733495408797148 0.855993102956978 0.863430920794399 0.874488462191177 0.728750035822793 0.736552500082086 0.755335755010102 0.750448395999983 0.743823596593097 0.637278271633621 0.709057014387384 0.74152213707062 0.769889584550908 0.75033138815912 0.764846616507708 0.841271943570979 0.877504886628067 0.78618491682766 0.728725595310906 0.69120739418481 0.801094750004433 0.840606124720785 0.782082097476938 0.803807192498289 0.735575200227075 0.713120240997788 0.853496008756739 0.727709497816942 0.858288658681103 0.850133818808492 0.817826192515471 0.70282002963804 0.760757996816151 0.69047554266923 0.743136644450417 0.857179885830636 0.807139509005115 0.758723919864443 0.764038540561147 0.913597036040491 0.634234176153612 0.728552507693018 0.766542163238867 0.796748901068457 0.742783247616293 0.793545962946569 0.681828078328167 0.846617074135378 0.687392123209938 0.76242350368788 0.753149305666404 0.715245899135052 0.817460759441519 0.80867681250048 0.762914582635029 0.76340574062255 0.790275438393131 0.848674396524729 0.662479047676675 0.711098658154731 0.750619209418617 0.824947790771225 0.670462556374415 0.640800472612839 0.785558730389194 0.756782435575631 0.676252615464714 0.781076391422225 0.746005562110796 0.682394576295636 0.583326474034914 0.78309489536086 0.697343295528892 0.755846459819558 0.668504525332436 0.757325726144192 0.818560136036381 0.675243046290715 0.755634171331574 0.763882105438306 0.782795354460648 0.704298702476225 0.820731532176941 0.83668580198783 0.693962866237809 0.710307210452478 0.670912515361847 0.677155382210493 0.851742752406949 0.697150163730815 0.743856903053748 0.798318301431533 0.702221975255182 0.736991884924868 0.882336889544828 0.868517830248582 0.705212553961998 0.718768719470678 0.803806021100248 0.690304139649815 0.668687777019091 0.780372703312444 0.729510451268616 0.709433935378792 0.912779505378016 0.728847863336529 0.793181329531357 0.806927421091244 0.685688190453628 0.746254202624171 0.764144492615578 0.851966394055799 0.766272487624815 0.813644039204012 0.774298174003474 0.615814240909683 0.831329813547731 0.693019472985686 0.715333109585788 0.782076600699013 0.797151570754531 0.676844024518015 0.77862302171812 0.75731718996894 0.746656533889038 0.662451716428234 0.7484253677361 0.74363784766193 0.68255760126432 0.728876335386995 0.691660758044859 0.855984230912259 0.822684635419528 0.615086026911479 0.718047986189459 0.765368215403081 0.720604020382225 0.772464072156369 0.776631210946246 0.786509523714097 0.741729055077418 0.755846319947977 0.724101106698054 0.77847473538045 0.723040317359001 0.878321682603133 0.785985641415406 0.692355532432359 0.695904184594343 0.789203911897322 0.732850771751689 0.725660754228395 0.792162583942855 0.751531238024405 0.82459812276651 0.768870878175491 0.71444720394243 0.668922593435549 0.924729118675193 0.687849410253162 0.914199863465492 0.803242508755065 0.720351260877761 0.771351311932473 0.751893505554957 0.785798983088317 0.91279419536717 0.673070559641554 0.743906312136487 0.694554477935456 0.723325168619797 0.695055184263695 0.759338795910432 0.740578784932527 0.650596415418304 0.691381348819317 0.629665316537282 0.773461843390516 0.710134481716715 0.659381633388158 0.81917620282591 0.856455153934032 0.76742487121089 0.633138582521694 0.725905700964142 0.707428794686566 0.604393278957463 0.663134919179468 0.734511712879132 0.793260017974874 0.747700087476937 0.715197907461975 0.747706816533582 0.665177036173443 0.844242766496815 0.677726868168417 0.848910342030445 0.792192514081707 0.675563318571551 0.756771299896115 0.778879612218266 0.819871424775601 0.661169674737106 0.780580361916378 0.728452124175721 0.7737323264427 0.766850116300814 0.719621761598223 0.783952132262179 0.770938986233543 0.71247243858318 0.737162779569864 0.816354215736256 0.78721763311075 0.687321207556561 0.64304536517361 0.836991447880833 0.838737272338395 0.850962661711195 0.592417482139658 0.835223805620696 0.830484090057117 0.770576691691359 0.734791510288619 0.855878239102723 0.755673816859314 0.767275397108261 0.79434858472088 0.688814161752369 0.720852165367557 0.817493305147414 0.696303298564227 0.773241923673733 0.779583603728935 0.751778371128928 0.725770664730079 0.785594321260163 0.705173834848259 0.744375045625396 0.688855953746907 0.831452029986447 0.713279864468744 0.641303556988783 0.704814946004652 0.721062124794655 0.839595593630751 0.764228589340664 0.662667214113365 0.73620170597743 0.714077144061763 0.726199363384797 0.715426795931904 0.697479615384754 0.651329721937697 0.818388103244699 0.680025519997222 0.751890997504487 0.832553732323651 0.767751814823601 0.722134781444376 0.767282598336644 0.776283046003875 0.706247337741993 0.740468297448208 0.728591284768248 0.710726347457328 0.680503723759029 0.874700335677154 0.766938003469996 0.812311268373431 0.678698209570483 0.779532863462281 0.81155728969827 0.718776398940683 0.862792927150645 0.718543126673196 0.759904528744058 0.762825304444059 0.797899015764772 0.780745169791174 0.658916807132066 0.775189290623367 0.709351618604751 0.720959162015983 0.803525460181937 0.65950775216703 0.918389241442717 0.889874557250774 0.765484559739696 0.801169132591065 0.662716381817463 0.645833216788981 0.751155817726411 0.757662955818281 0.763882649619702 0.690241000361574 0.811198613154045 0.770003081953766 0.632661023282095 0.62679571122151 0.873648853131952 0.701381741670997 0.716806183847938 0.784817477052875 0.716922398222839 0.841574668851085 0.824495475124926 0.785662921806474 0.72208788449039 0.646653378045839 0.766370172073449 0.741119174229175 0.806958662495289 0.779021995876551 0.792345453724666 0.81637623624533 0.73475502998642 0.896871905409917 0.756271041062956 0.728538141288963 0.732392105321338 0.670108038093103 0.799882664462361 0.826594443826952 0.770667736647562 0.831381241906183 0.646754665345896 0.711826247908336 0.764634926575649 0.803212651970394 0.84619235612994 0.72785255639313 0.769383930532208 0.632126728420062 0.71758502948406 0.918770419035979 0.706101798833901 0.704264824429398 0.796242319534706 0.675346039204857 0.698194175238939 0.717326005205171 0.736044204221055 0.711546596112283 0.711238360601874 0.737270003163554 0.78685151173429 0.727147977501178 0.874429866308262 0.747800501064188 0.681629782407632 0.846057614747741 0.820571985995927 0.889349014629772 0.798570334929068 0.630072393934173 0.648039740125402 0.841212789958811 0.786928992463756 0.806972689399777 0.765261879440382 0.781412016270088 0.888499779837409 0.731395491754179 0.737598638086514 0.815940874634909 0.791181741901053 0.833029234608164

APC_co_stimulation 0.607149469536835 0.604857539610155 0.601073127676971 0.6228301142382 0.671158264072181 0.652368487746598 0.675065440411248 0.658333745498699 0.654866076830272 0.687945368015599 0.647397766212571 0.551756134947581 0.540716650800519 0.536925615622086 0.509675910966017 0.666821987583086 0.668991687622832 0.546410966187785 0.533896311933413 0.560644323589507 0.538473409288343 0.711364656663672 0.605748775040989 0.693058314362151 0.666162180517071 0.66787639599601 0.656414387497234 0.535846958760353 0.608862344423642 0.634686936285445 0.67348471434496 0.591512516454883 0.538754785861811 0.695621246538327 0.696097815645846 0.584822884040649 0.588004832144168 0.58528710624854 0.606656485359416 0.552447132635532 0.593557224535297 0.626583894429998 0.536606892606851 0.648999503696933 0.567589264660889 0.564510107631753 0.661529094764368 0.572021259959042 0.648902349242243 0.595718099268601 0.609598950753978 0.563357271447841 0.585807553452344 0.721752321381764 0.557058006432356 0.658198959283311 0.546815033541789 0.644060588565268 0.58355412526245 0.582301035022987 0.732502822730363 0.627903559988143 0.616113664368672 0.633377032800577 0.753095783536264 0.661023313306727 0.541669347025054 0.686712615571759 0.564470546527987 0.695843291835046 0.574410876330425 0.60881227145987 0.609704449330416 0.540852443361596 0.600304769933324 0.590012529955009 0.556768876996857 0.636515652587653 0.608057862903943 0.655862774479719 0.585553424096505 0.684621465059059 0.644557058060872 0.626273334690495 0.699986061681715 0.565739106612795 0.584155322452665 0.583902662447699 0.596110184908495 0.660457770390247 0.530580190645883 0.561751725200111 0.58436907512146 0.670206384882082 0.666960974561953 0.546138039575957 0.607878073825299 0.688092090300409 0.619198003739856 0.666788557672848 0.647900668779872 0.697518683868037 0.583524948087111 0.61803641762152 0.559755055311509 0.583845366225246 0.629583725402017 0.649780329660142 0.657636024528274 0.669831291828118 0.624674390143333 0.541595381420711 0.601006311798564 0.670416980420254 0.584546775763283 0.677020300199292 0.70733702080542 0.66388530508857 0.596054313082449 0.702158108064232 0.623349276042437 0.684964636108993 0.600086828840484 0.687039192618595 0.640646804817145 0.706985050116369 0.620327761842828 0.573668965294489 0.584008835785439 0.562205103290948 0.601463388014028 0.613488440592411 0.674123212050891 0.747920380524471 0.71129567712851 0.586715371527041 0.594371718342785 0.617011260048415 0.594264848033678 0.630071025218562 0.624510283332747 0.725602445722511 0.673047725103272 0.675674653314869 0.63958433590145 0.595282751999065 0.598428680925946 0.570116685328664 0.555589313954718 0.591068063942042 0.539598027211254 0.579788981987252 0.613731795350093 0.630482602946888 0.612591383262001 0.707580163196197 0.672244793370225 0.623373595424429 0.666547375301431 0.535896592976352 0.526555218464341 0.567969165882419 0.575010531322931 0.621100581505973 0.678716412107488 0.627966382231397 0.596405132063539 0.593696074735731 0.648320567969066 0.556678249505682 0.5572128371787 0.747199703356148 0.569145018402255 0.576751717369883 0.661929821470198 0.649221461877634 0.589968033554395 0.597275854179384 0.525371557135024 0.598418353307735 0.633657879131314 0.552325164701534 0.541830485921893 0.603971450195074 0.680348812366224 0.55573002160318 0.68466682507305 0.566326300154613 0.576096942444357 0.631680011420242 0.477496185244583 0.683355314381817 0.579110581296538 0.670651094072593 0.700258452020176 0.676666769746741 0.610210964176865 0.553246336792672 0.646457070940656 0.615436681003588 0.663468129460115 0.657111402121436 0.633695476022164 0.782626705661143 0.560863878992787 0.598294744600068 0.543527777475042 0.658233220319792 0.612274368357043 0.743314459977984 0.548955652493851 0.710212888343091 0.583712469895385 0.659708036844891 0.663445564987946 0.607799679928081 0.555908078165291 0.521402517666775 0.668053009947015 0.557212225406889 0.661246848608941 0.570950966504824 0.678559608561031 0.632799216447675 0.592742399588961 0.754274768185963 0.634893740811926 0.684876120238041 0.629923424965622 0.706510969516434 0.619320615624183 0.577104997317467 0.575849394512344 0.721496877870531 0.623146104304117 0.716205492779941 0.529219671030532 0.514903234299291 0.553600499402535 0.563750635068128 0.630319032058826 0.629311544629442 0.668049148956716 0.600256953021542 0.615454402975042 0.715154841109256 0.639146723868612 0.617427885598967 0.556411049846273 0.737016930838229 0.675052872109674 0.651586399221113 0.69791902234695 0.714508596424463 0.609597424767624 0.651782533010519 0.753283920248946 0.668621883223823 0.546402261870924 0.650390737470324 0.616556602654104 0.605537723225028 0.568473213311944 0.588595435141223 0.57839480821295 0.584972681627797 0.618888139786812 0.505482865352622 0.755319079207802 0.760808205382193 0.671076187193269 0.621339038576958 0.548866759421391 0.590227928610435 0.539591563676141 0.651729648784543 0.654834966018736 0.622019638965787 0.577418863213035 0.668719306840073 0.751328945481379 0.592979936791632 0.560487362439189 0.672217258698533 0.639374462098852 0.716025169360368 0.648360605575352 0.546977010597591 0.696208556499089 0.604951617240912 0.568243243489417 0.617212205512559 0.600756428035834 0.559130446373061 0.610360031408346 0.644539967276688 0.594617699037129 0.629295039156084 0.592228939746797 0.578963668267052 0.675583330911377 0.67591675402037 0.569789964976592 0.598484052764248 0.631377197869448 0.596185088490897 0.703720446098143 0.550309932415827 0.726506023121358 0.567380290397577 0.724480525429647 0.558269931305097 0.574925648093167 0.546212401116461 0.628411281216291 0.647064302482046 0.514106077259138 0.602943542142079 0.561828820991861 0.693270803750817 0.588464017836653 0.660691284023149 0.554758063565245 0.605207587714826 0.682174629254181 0.57626375212812 0.594962023249652 0.634364866903081 0.667709429334256 0.699181171988595 0.606253289816779 0.721132504649619 0.61075840902262 0.774415973996578 0.610081689662462 0.632912423510438 0.565347617516177 0.752146654576437 0.645301705417901 0.562610835282361 0.570611327355693 0.620447368591045 0.56469089415534 0.697827617911901 0.66301699292679 0.789038775783037 0.592524267002135 0.576738127706235 0.628619107372744 0.674842467137722 0.593767040472932 0.514452211880616 0.537364728702972 0.668700451904911 0.644690419645769 0.575856907596046 0.639031096871617 0.689611930671862 0.634302946257052 0.618754355191513 0.556720923064005 0.61807837755148 0.670262863616076 0.691958282978446 0.628859662283044 0.618102461094322 0.566384982067122 0.582099312869353 0.738592477983318 0.557388888583211 0.680690653865983 0.769368258286051 0.639359237492754 0.577170084323561 0.598280849439423 0.592119232458132 0.609129147088827 0.703892147425568 0.622035917906471 0.617967324469844 0.637792180995929 0.715016423196534 0.547300992227072 0.554528746366453 0.595973163800427 0.592742878305233 0.592155627403222 0.697532899066438 0.537627093861852 0.683866005483227 0.579279627707789 0.585151214755978 0.611293661664524 0.557714512949316 0.710234443507951 0.730756693196893 0.572387392905431 0.644741326530488 0.638591246060298 0.687846376081317 0.547573858318934 0.562040703521471 0.638549001609104 0.662486872661655 0.455850388977614 0.594753469771792 0.632662595437626 0.665912936182161 0.59060629526287 0.637806993674578 0.636608912725652 0.574473457866087 0.516918222410964 0.625119073201347 0.563821361931257 0.622533057237419 0.590342915986863 0.612114374579877 0.659904721279781 0.591630929860662 0.693391453241188 0.578251829075031 0.57076331824361 0.610900713486241 0.69287272723517 0.648722594286353 0.620071560097467 0.569776452772021 0.57425458703983 0.580254525813627 0.61732517886692 0.582982931838884 0.646101575612602 0.661143782137659 0.560246229661441 0.545138421192472 0.672999079953613 0.702015771937997 0.583653429664509 0.562018931755813 0.723550735319999 0.583451699118696 0.600904016842321 0.64262591883 0.626810174590495 0.589597510329862 0.693192968605034 0.584016287756271 0.627218298142446 0.626853265514438 0.54667022719267 0.559373946101224 0.634196480130279 0.703289322953567 0.645202735743745 0.653091433557981 0.608020112721301 0.536887411049311 0.64472270807011 0.627925404444334 0.572698374923588 0.61136963313108 0.655967841980836 0.52282541751536 0.645614713793054 0.632867086219754 0.632286467923008 0.556884688708178 0.636017991369077 0.597221790467349 0.5332939735419 0.563675800568766 0.570043771909178 0.635512622764069 0.608096987506436 0.577999349041844 0.587192172098107 0.591753435125563 0.538328120367527 0.672546582144917 0.616623595912217 0.665109925000583 0.623154362574417 0.595349874605108 0.583703396982873 0.62224842473765 0.667243093292281 0.733629938079414 0.659586738608007 0.553696732606028 0.591333386310678 0.626592743583434 0.568682849409803 0.521340908674101 0.624555597202495 0.617974727204779 0.63729229843314 0.66598611051962 0.543110293194394 0.533751904973396 0.799869800850464 0.540221685570395 0.733883539377011 0.643544630649841 0.595206698663003 0.617957204910443 0.650401419914153 0.62541597764384 0.709247166459689 0.556082447596882 0.569277960891542 0.51538960228573 0.523116377974174 0.575837801508394 0.580001235751016 0.589454704927154 0.564943874286839 0.601608790339656 0.53843391336379 0.619398715972366 0.544303064474667 0.562296586772689 0.646968597477039 0.758451112163948 0.56336878905018 0.530867886604619 0.604848303815184 0.559695184184881 0.532454569179655 0.62329470142057 0.598449509575572 0.704499325725048 0.563992829561121 0.525935939851618 0.597682523877604 0.566834059138231 0.699226477156554 0.55425312985038 0.669311070841294 0.640885655646459 0.609038073829151 0.635341259404688 0.664465263295548 0.665065438423643 0.621916698421174 0.695412856938478 0.604100098506703 0.631025052273269 0.639443218175082 0.572124609247738 0.651234628389426 0.627104456709344 0.600578204549205 0.581798664473063 0.668541121979361 0.645960022124274 0.539592643391109 0.563159232991435 0.702342306308862 0.706680805444 0.676954867447808 0.556221785703432 0.705306257682487 0.672436905353021 0.663871825682055 0.58151391607793 0.681231434730623 0.644489856773302 0.568011551429059 0.673504513566698 0.507258271909672 0.585403061865573 0.650342421290037 0.553756106850033 0.598471976943922 0.643329258814852 0.596870123355482 0.616808638822096 0.649842834478462 0.576619015803684 0.585047610822306 0.601802819127925 0.648983574137225 0.606743770209028 0.584390700957762 0.605251572238879 0.537365687248361 0.688398982239588 0.595811088948085 0.574706687014067 0.584954714284179 0.588071335531971 0.582098883246501 0.577781875517996 0.584670090871217 0.543240589536286 0.61837028929234 0.557249351182839 0.564909424741568 0.621012797344905 0.633113038236391 0.594762668675218 0.583935285907121 0.597248894395617 0.572119847129211 0.61567353693508 0.5420989356574 0.604244274894405 0.539668658133155 0.726938282584265 0.569347989086413 0.701430449850473 0.59643243373183 0.653029805117468 0.641144510830003 0.581374051846302 0.658932271475383 0.554814571371019 0.627776630510717 0.690218626868717 0.603890587982852 0.61270523500223 0.561506099205885 0.583607815238115 0.58190326727253 0.644398579366068 0.618713882993186 0.526249866876425 0.778523945425481 0.690956181423938 0.559402067378109 0.625263764979114 0.558612748781525 0.525551990652213 0.600401113011006 0.578319629246446 0.616088047631049 0.564883287876929 0.586930801753375 0.65555735917156 0.522820678840448 0.534697701085665 0.642689647275935 0.571389243342027 0.656156189214249 0.626400713156891 0.57449514530277 0.712023247542401 0.696495939791021 0.60433208431104 0.664041477409295 0.580172983782706 0.640847186091707 0.550226190945814 0.657549323786098 0.673896099318215 0.589777473829005 0.688698820903231 0.610198101905924 0.705320319662529 0.584188995405634 0.535168683103287 0.590785483929155 0.562759547718175 0.647933118377304 0.701059006637279 0.584878021921546 0.649684965030529 0.536714826879972 0.553308721393684 0.567127713479101 0.65381207820581 0.659687016067841 0.622423034914741 0.584025454088336 0.4793002560282 0.642182248083473 0.738419872003807 0.56556860109461 0.542325733719302 0.637946464422778 0.530821141402904 0.514383414103421 0.548527922256264 0.586979292269262 0.516805705689658 0.609262630210604 0.595228038476675 0.628459246448799 0.594321530887881 0.710815300903146 0.594990366731142 0.53173027639647 0.739594458073555 0.665978616491477 0.665429855886203 0.596874725447208 0.51906449170757 0.519292779408795 0.635165113569138 0.656029550530489 0.656249158022602 0.604309937861506 0.615196089202243 0.720753711675414 0.570067491681975 0.587568352617325 0.680504396536447 0.630874689784051 0.65968466913548

B_cells 0.505001588331171 0.536869926742165 0.50657859606011 0.56512014292575 0.543864349336847 0.519431642664012 0.64038154722682 0.563910322576852 0.547509249985277 0.591762106275404 0.572757113075006 0.520153306814645 0.51258536603082 0.505751380316183 0.475947398422404 0.535623777751399 0.563541748793394 0.532430625561186 0.539747938306216 0.514253763612752 0.604271606035689 0.609404613410489 0.499602812210626 0.578671709742323 0.528400991009097 0.568403359848248 0.608488136917816 0.525939688035271 0.552851000392625 0.554031571328547 0.562281762321087 0.519301564526985 0.507680009269209 0.554624364958049 0.558306218348289 0.53804480654152 0.538299341842241 0.527805465105093 0.551024682515599 0.590225307080375 0.518982015104458 0.536030572465862 0.511637647451094 0.532332562389454 0.544093716342983 0.569200700974832 0.550447359137254 0.510105077666012 0.548863875930001 0.572833321396793 0.54525000134373 0.522397493844463 0.528465381317848 0.593983054182182 0.54811040957146 0.569213872713476 0.532885116746847 0.575577278375947 0.575407674275561 0.540151672878859 0.566086718764625 0.58914916336322 0.532911102501495 0.554577249381172 0.598084124543878 0.557818619948974 0.50281426148285 0.665326635268657 0.523462500093692 0.573170592080413 0.534566212081349 0.534600545932768 0.550077850375508 0.542624814133199 0.580329658450778 0.523678216277264 0.498009939759185 0.540059622359563 0.549466840743717 0.552341996355131 0.546349313480258 0.547994519483438 0.5684163392264 0.587593718759188 0.576370051309999 0.52487083534037 0.549286236015052 0.526967203936894 0.513454281495466 0.530010985894645 0.58178137745679 0.550315173528541 0.549430984820566 0.574595725948908 0.542276709725679 0.504858134328348 0.517886717017338 0.584656883964345 0.546879861436289 0.565467530313484 0.572261836427367 0.62032961507628 0.580473372143661 0.483284859774443 0.534750889937031 0.542347705967701 0.517871191150462 0.569921565644004 0.60992901843744 0.575470223193893 0.570366268807852 0.49369078646932 0.562267070499231 0.547461246461211 0.543701696385567 0.537947298833822 0.560051313579761 0.530074556806666 0.542478472280168 0.568420093977966 0.53010992391508 0.606523362995946 0.505443679684642 0.568770954131219 0.5566469266479 0.570617313997836 0.557265273548931 0.510462689725416 0.568023495424443 0.528621313957173 0.509593394150485 0.545563967988103 0.59415440262463 0.535080729754158 0.522457940727384 0.592362188498798 0.52062307676228 0.579687147845201 0.547939620671171 0.547618885255794 0.550685667264214 0.542641036874639 0.576981553123458 0.547521485873739 0.573890798982812 0.508518366536172 0.525027781838941 0.587328537148545 0.576242150222274 0.563937034868648 0.673029592683896 0.542117783157695 0.553691965354132 0.554518366836857 0.502527714424448 0.550682432608692 0.555025255615453 0.525232991686085 0.580284083690064 0.478902502268625 0.509479757662447 0.527499371269713 0.517788700619493 0.504196437603904 0.591782662906806 0.544172219862985 0.532895826554617 0.518393135658874 0.550191168786318 0.523330395518466 0.548069833647208 0.549229120195835 0.554958701936383 0.501157969931236 0.600205926682846 0.55793695113006 0.495754666111042 0.578128589390425 0.545612782320457 0.559523409794507 0.590660231238719 0.521997389443162 0.540860836890477 0.611809972584616 0.563184037265156 0.540909031852141 0.530998057053638 0.497906939314002 0.574638046316384 0.593192739709586 0.522255943379188 0.553037685660297 0.554754207958837 0.585397322765789 0.588164429197259 0.583054387971807 0.568200117836139 0.556563222443879 0.547867079198365 0.607936148352873 0.624855772160323 0.508416853058633 0.590540239875122 0.581722198595029 0.528242656444657 0.505448571486048 0.518271375565171 0.572539733972608 0.492084585829216 0.695837303768193 0.566291338951822 0.568941344958829 0.580191952220086 0.556748085664901 0.678735322972045 0.57259273037573 0.521417618252788 0.557380073788873 0.570948070161222 0.485274270175371 0.568707780653539 0.523760818970789 0.567906889045224 0.594403180640093 0.51737739255881 0.617346676699806 0.540492603697434 0.530627975885027 0.574588343011421 0.58302369059089 0.546730487600714 0.524258724907764 0.559416496256957 0.538913284267728 0.596525842860502 0.544337577968615 0.536054584926211 0.563309807808375 0.513828774714012 0.516217268471604 0.572235534869437 0.511799498595993 0.57674276877216 0.522386116526869 0.533465638089177 0.562095314074779 0.465334493469267 0.533186825019487 0.505317126969064 0.660774099288026 0.648177310055002 0.575363492153557 0.556499268296698 0.548135664324324 0.554572638760086 0.596845234587469 0.595384142495829 0.564648149595965 0.545057070907738 0.587287339802189 0.518143562647243 0.581298460696057 0.496547343666869 0.548840667227515 0.553871930375581 0.549822731189927 0.43501298172369 0.536018613867188 0.558241712990443 0.585331036803849 0.6372271403991 0.602553440828589 0.550784099926104 0.57940066205814 0.569713505607271 0.594035207683085 0.656663947785268 0.563179902737216 0.541516630833573 0.548900055365475 0.576046472338062 0.565556108417033 0.516122978660165 0.570572478736876 0.598928566045726 0.635038323243719 0.575619015849738 0.524757644738974 0.535777391960318 0.518071840242745 0.536425147699318 0.531097429531931 0.538596649814345 0.55464530669001 0.547764077819905 0.597901067979906 0.529821107368416 0.62109923312122 0.542354934004369 0.543658583136928 0.522378243337784 0.584138822221091 0.549175983698854 0.536885707671856 0.571011268241227 0.554246332530664 0.575673911952193 0.544289230633582 0.550164505145031 0.545107133880783 0.586323840897309 0.536130000569863 0.528244442431971 0.524076714516334 0.521409698970538 0.568646181712175 0.528345703995169 0.561742502099364 0.541014194742116 0.545616011513419 0.553698024859301 0.513953880297108 0.540720770006223 0.531168288998509 0.585394935400093 0.546301502657389 0.535124311228506 0.560520489482372 0.529326655731261 0.559621145902143 0.595786474954996 0.571256342642749 0.518174229455384 0.647841016537143 0.550265562638284 0.572940423815395 0.514147865992191 0.665303337794994 0.627196778039691 0.54176503606639 0.523942703427466 0.533184513547771 0.510839521893747 0.602847604571588 0.585492336047006 0.650560093522703 0.547922970694838 0.552114848570925 0.544985161736891 0.519380582041932 0.519208454806564 0.500226237161824 0.528017304087151 0.541727351012526 0.540875958061803 0.544303803120595 0.51173155803612 0.563790094882765 0.579171232840752 0.537672302232983 0.573245575674318 0.518756768752056 0.563041251886102 0.600691833445157 0.605318564831237 0.516402967038597 0.49714900479038 0.59278403584037 0.533065308204994 0.553416023982875 0.589576702563215 0.608599022065359 0.523238398209318 0.511065411257863 0.57087617543297 0.539717482375038 0.549836816437323 0.546124280183778 0.582958786905965 0.556169058789132 0.589899315010978 0.610704357673871 0.513047854767469 0.464057568146177 0.553192786804659 0.549396885546147 0.510504298720178 0.611625783436656 0.509273131838806 0.580627822671757 0.532882121151344 0.600585810411164 0.598609784654031 0.560835423739253 0.558085594197945 0.581404765726326 0.527712006289468 0.579820227060706 0.557713314532923 0.609939490625469 0.573986223775601 0.519135442902657 0.55587015481895 0.587201662051285 0.518932387911262 0.557610683732039 0.59727995898914 0.58769322622487 0.561879718498395 0.538670554522335 0.589666276523833 0.550947052146452 0.50701348766673 0.557322019665105 0.504117068064214 0.581401743991978 0.527999998160742 0.572781535072425 0.593778356850427 0.614812887934649 0.54618331919168 0.561581768508233 0.562726957621381 0.560165592849105 0.54242282339196 0.56609306045177 0.5493080195148 0.512882712418054 0.564196710590702 0.544455000781376 0.577131990677715 0.505494070305857 0.537472054048534 0.601764283159945 0.531340538004044 0.485436722112059 0.684982847495569 0.581347784246284 0.556623080130898 0.528358660649558 0.579006885577597 0.528854733701865 0.544681310263087 0.53584610195013 0.526858907055746 0.510180295671873 0.561490132912878 0.524067659749406 0.586332347312188 0.566268990598783 0.479034997251676 0.466235556040327 0.533361854987037 0.60540556264359 0.515932218686317 0.545131007902738 0.520236978553801 0.545152086046334 0.586468299743508 0.527459599643982 0.55801790446476 0.558788308457646 0.581895426357201 0.527069047232414 0.527801844385639 0.566835298892966 0.585669825491591 0.50322811368614 0.524417444374084 0.552539529080833 0.513387291958097 0.571100607767474 0.523296973903339 0.506079547766825 0.537786925725494 0.548987748904611 0.55819447310401 0.57133694137944 0.478078151223908 0.60293584437541 0.534935100472186 0.562610201245199 0.534761327247864 0.533789749201434 0.525711519679301 0.565002224869262 0.516184646805062 0.642209079459089 0.562904211865858 0.558866961264884 0.565363529190188 0.575875517237624 0.502915561845141 0.525665072630366 0.531404781205423 0.525181976027838 0.607687260117636 0.573028115773988 0.539445273532101 0.509017200628204 0.612759381696362 0.49906776016634 0.549987234233153 0.541771940650209 0.512991261209456 0.513643292339288 0.532827628720852 0.565856371661168 0.597333862135172 0.50453028908843 0.546414599686866 0.497921779599036 0.515101233927931 0.559833198952361 0.569615066422442 0.509738725428571 0.536770594612586 0.561931689842319 0.564743791463788 0.533973593569589 0.577922375883808 0.517293271528577 0.570337701762781 0.553737282040973 0.541235619976359 0.565384019686529 0.504952301632098 0.527540338205098 0.488273815682549 0.574208626442048 0.530596087841133 0.570152580709473 0.528519550476084 0.531253626431483 0.48162644927358 0.496759908266861 0.594631252492333 0.512215642410824 0.53738524976887 0.536452904168652 0.553967323620961 0.583856967364022 0.573713416642713 0.586190940904226 0.538969499865635 0.577708296803967 0.562857814380204 0.612878983922841 0.511863407894055 0.532382251724986 0.569061265661718 0.54556060606485 0.536194420756951 0.533251038205992 0.548385702907768 0.594235497042104 0.560712104276437 0.59650312437983 0.56611673905103 0.660497390051329 0.540646132929589 0.527354764977171 0.560504802997168 0.584664861112457 0.558860302781184 0.511474716907726 0.539481579506289 0.558257804452224 0.561939099084613 0.543864185389682 0.514563525014507 0.483078374447087 0.527107092028758 0.477097371413149 0.549352831083428 0.540121655363244 0.515395024217111 0.559745506322154 0.588107258249743 0.49610063128062 0.553157969658834 0.541699281548482 0.54585776185091 0.53510576693765 0.571831395866945 0.509574577994622 0.537412026872316 0.580494346025219 0.489277921445535 0.524019619021415 0.572108844666791 0.559620772211936 0.560335365285008 0.544101181797546 0.519991146177503 0.509520921702159 0.566445341593237 0.584981954039313 0.547107696401035 0.534361327872742 0.557564421564008 0.544476127817108 0.571484843145067 0.575830315839533 0.494261752021828 0.548066853497907 0.523437043841555 0.599740821837004 0.556390938647418 0.805463787104341 0.594936567580424 0.595508234393158 0.541857515557083 0.54281828566333 0.54116761173412 0.509391712446964 0.724245247351191 0.525039668149259 0.567381954626668 0.54793923136061 0.538576412352117 0.595343179680562 0.579557456171424 0.526064875078508 0.552940544370968 0.541614192152354 0.601585386987288 0.512289444292746 0.608088914519865 0.546581899799472 0.516867697276912 0.606794472601753 0.571343458855442 0.541658998970127 0.550733478971106 0.5144066798629 0.523345221084411 0.517320384432799 0.586875990195953 0.588751025718051 0.491493168031674 0.563552183363724 0.548306991913094 0.597377002140695 0.567606277620581 0.557060233053724 0.498393138097402 0.576918724895176 0.594326207723125 0.565017525062327 0.551226554046837 0.571517894481818 0.561087611875989 0.533967739175203 0.612570084361968 0.541850672980658 0.523661657051324 0.567396417684473 0.575927597551237 0.631623441338239 0.527944525521014 0.571774719124272 0.534808791761975 0.562207418208308 0.508002375184077 0.615152286096258 0.527712755267237 0.563535281184862 0.527670670247391 0.537516592377095 0.557742207671993 0.55630274866231 0.531848058810933 0.542361251636062 0.569189326927699 0.502710958639028 0.522345207246338 0.589080773497643 0.511337012329577 0.49100548665357 0.590813181646309 0.489338970149235 0.522818687050091 0.513985859095004 0.51894200431849 0.501839604906749 0.495734464729372 0.536013097095079 0.60449534190359 0.532820328405098 0.719623679024572 0.541956684739337 0.523904248724771 0.599483824292744 0.53799883409466 0.607214262175356 0.559187050109293 0.561165810846547 0.49284400110998 0.638199028073054 0.525765749861788 0.548903597025211 0.603367078608847 0.546862129374541 0.591990491877272 0.515422655981235 0.562132668007218 0.594635408797481 0.562099636531484 0.58465472953852

CCR 0.595016545036879 0.590501995525412 0.583221794433688 0.603851765405692 0.617050108585366 0.605221344331434 0.639207533017557 0.611369125834277 0.625225826720123 0.636469684685757 0.627865083546005 0.581217178822387 0.564046546549872 0.534038414296092 0.539765500345385 0.600538286536984 0.614139283412143 0.582318071489422 0.55283070944724 0.539394123835326 0.546707756123525 0.642549788623603 0.590415049344515 0.641595648501936 0.61773660988361 0.607609856182721 0.661140096563351 0.562912850937829 0.597537795641444 0.619372923896751 0.635823632850533 0.596995293304925 0.573473401429697 0.638782870716107 0.644159288166536 0.571763381921207 0.574201847222064 0.591458862602611 0.601666931407758 0.575644646923479 0.596176710902588 0.589797408106099 0.558038261924296 0.605048140393475 0.577746344822857 0.585467458228995 0.61708880306426 0.555199074858634 0.617600529559573 0.583860144690856 0.605228921736338 0.546953870286641 0.593698004292026 0.669322203926525 0.578246590610854 0.61039499234972 0.563562056761561 0.619851084568276 0.585284742987707 0.594158325962477 0.650589078981034 0.595684192205396 0.592002109064302 0.611949469309984 0.681814891194144 0.614907906323569 0.562067614379564 0.646612471212369 0.566051815312897 0.63990285419967 0.582441918813403 0.577707809543656 0.599113406942908 0.550524285348924 0.590444616448333 0.580571669461431 0.572197568950893 0.609132551978072 0.56580390383879 0.62260904754707 0.594878819215845 0.612034846084504 0.607158021182641 0.618003216707449 0.647729995596861 0.567977239693603 0.582991895418286 0.561678135943393 0.578396448531184 0.637329477061418 0.583760991205051 0.585225810435197 0.574731057384288 0.628310735377679 0.614074887178113 0.557945157988313 0.593492632086429 0.610630012166706 0.594970472651576 0.602146194509455 0.629708712820479 0.638125190868798 0.597086459119158 0.613506118921013 0.566986519595132 0.588697638714066 0.592405718537337 0.614636721164378 0.623317197858303 0.621891118698185 0.604840505689535 0.561060320263792 0.576959030067813 0.605150830352296 0.588265540255623 0.618673986607421 0.636704909483428 0.62051134394628 0.580059387853967 0.641350460389441 0.606320260339297 0.643477364911231 0.584382393563789 0.637277671277808 0.596953035525409 0.643109085191259 0.609806955747317 0.558710985087509 0.592802287763137 0.567317737895543 0.586175999996593 0.60452284052039 0.633989840721358 0.656816332128313 0.652056824325758 0.588751437768269 0.570390460463439 0.602873110300931 0.582252645926762 0.585351115042028 0.595625908264034 0.658471955851237 0.621346122186041 0.607718478228468 0.594889418336352 0.573293442150503 0.579788364058756 0.584995186344162 0.581070417091723 0.586028230822596 0.583830460031981 0.583234157022115 0.595706243880178 0.619849149176458 0.591787374564923 0.643174777928807 0.611631094956165 0.618170006408289 0.614142989899664 0.563889322589659 0.554282139929978 0.561248654745772 0.569471080389597 0.563305059640128 0.618666059903251 0.595064656887065 0.581010942597021 0.59825821617506 0.597984859984705 0.58482523242719 0.588559769630036 0.676183652903848 0.561949538466455 0.593937208602601 0.627843742767552 0.604203687767824 0.578629613767103 0.57698476464853 0.558581625230969 0.580662999690152 0.621022678398208 0.574211609718772 0.578058662743521 0.586369218460009 0.64181853498654 0.560346832265166 0.620645596621638 0.562461685616061 0.572872820942409 0.607580826658958 0.542656855024146 0.664326384697711 0.573917649488141 0.624297454426448 0.631743991451107 0.652617850192228 0.608128520281435 0.580787520261441 0.584648738534341 0.601524053156914 0.631445189495804 0.607268695214985 0.614472172726969 0.68351544032404 0.544047000501872 0.5819288321653 0.574473199381702 0.602782258386007 0.583858604948137 0.675860438420742 0.54487856789255 0.641129781122169 0.587505615756007 0.620657753323669 0.642962823559735 0.584935813133786 0.548829500099985 0.55871203794331 0.608421967733707 0.553909684680886 0.618905802742882 0.563103177895937 0.6306578482661 0.615210763408875 0.591727650671777 0.670305022246082 0.604182568847028 0.625757346425034 0.619679853181136 0.63404651482318 0.599482851561764 0.585618508395048 0.591151922243814 0.63855832863101 0.629304621760091 0.649103381629396 0.558403212047319 0.559633616549328 0.560966250940187 0.570103484211531 0.6222614740521 0.561578896381668 0.62356212305593 0.569524280330848 0.617722133721072 0.665243815573545 0.59370631166059 0.589269509562676 0.577634862023471 0.703462844841679 0.625638664812494 0.616884654809856 0.654318079688348 0.661162079004631 0.598264480284834 0.624740245446278 0.69206550201942 0.625228146604139 0.582741526698366 0.607876095610524 0.597240209040208 0.593782563469733 0.566192821792448 0.583245782834575 0.58561845279836 0.587709598685483 0.579962480022824 0.541691854777811 0.641965990347512 0.696939759998883 0.62858380567747 0.599362451067881 0.566915120460719 0.572965695378545 0.556182843265902 0.635007353079007 0.627727842344972 0.624045171801179 0.565544766182047 0.627059385245585 0.666423864207307 0.602460390346501 0.5735048544247 0.617440079499049 0.625493427948181 0.649420654339561 0.599900472177474 0.581820691911707 0.634625689455742 0.579279440344261 0.566965713315027 0.590842445117355 0.599681562942419 0.559385614928428 0.591724185354588 0.60932802668528 0.567514037392525 0.596432114172395 0.580705109978355 0.578800689851115 0.621992187192625 0.645724175960358 0.563225852964157 0.592807443293143 0.608486237027411 0.577040823397067 0.652374935586642 0.563958761692181 0.649057827317544 0.572823656333449 0.639722761521007 0.577115823294909 0.591836657412555 0.574499183711635 0.600451675061607 0.610861298822955 0.561389008290999 0.599693586224411 0.573662928531783 0.632471454126155 0.597537853825412 0.622653567966287 0.568916807137344 0.585084522276501 0.636679918702762 0.597162957135432 0.595169323304006 0.616992904283606 0.614275213183307 0.649160012722739 0.608410369203597 0.64692768856783 0.609847739353504 0.714341384514619 0.599183408101049 0.600966932290605 0.570417556255913 0.656433886098961 0.628535200132366 0.55979342256784 0.583432457625918 0.60565382835896 0.572979185246115 0.643005378541657 0.62247025994669 0.705387494996878 0.573074786828042 0.575048774458999 0.597893331101575 0.622055637529364 0.58057545011332 0.546880878094177 0.580800592015773 0.616315611501339 0.605819703571733 0.577787410034203 0.60619572492121 0.641458688626107 0.630833340067169 0.611946283930119 0.586684328249558 0.580418781686931 0.632548447657704 0.65080103725542 0.616111008993747 0.608245838108586 0.557751308120668 0.581399718714734 0.664107540556119 0.591708663915217 0.625195656941053 0.704253357723083 0.617624242918504 0.576984490609962 0.571896986659096 0.589673342794619 0.599734248406691 0.650074499448212 0.598553141944716 0.582884837588157 0.603422682573681 0.661268487462188 0.539220831148601 0.562428343932604 0.569336629495586 0.599420312089802 0.58401674289249 0.629010903273526 0.552618592684844 0.649167063232985 0.582860925121787 0.592981564581856 0.617707452116417 0.576074901318391 0.636914154564673 0.680079769952843 0.591200198807739 0.620402417847239 0.603473864996828 0.628065803996964 0.580683348095682 0.560535660231267 0.622840071981852 0.624788877599763 0.55143058362608 0.589089110961069 0.590908750780603 0.610511558027423 0.577185441207738 0.605051255544628 0.613091405726241 0.565763870324947 0.532184161324206 0.602510758128484 0.555196568888539 0.597751275116981 0.5612435045452 0.598389531301529 0.641594529972222 0.603792237159063 0.615038988180251 0.599594397037906 0.588051601393796 0.602476728458335 0.62250576899706 0.609169730734528 0.584824810277396 0.568645368998468 0.597302455054781 0.564802072583738 0.598616096364534 0.582177151109588 0.619280536148853 0.627697473166002 0.577642705937022 0.567986005202908 0.632059404047576 0.625870463439664 0.58221900873306 0.562808439403961 0.627946401834411 0.601829471139044 0.605814942165336 0.597804389491279 0.589909412185139 0.584988622735425 0.629961901460353 0.592593942234786 0.604422536334891 0.603415897457757 0.573073436511307 0.573048844596164 0.600691207749058 0.655151709413801 0.59334565365535 0.610751515969308 0.605768890035344 0.567664573558466 0.617126484403405 0.598715262047807 0.586965596211838 0.591046148300477 0.619483077371298 0.548738947387682 0.629517893026495 0.595216500634211 0.618865122754673 0.566995054003138 0.580609479653597 0.617308531630358 0.550692128381128 0.583409657730674 0.561932793669284 0.627932192781963 0.587340552557784 0.590843292597374 0.598935755774313 0.605047615287899 0.56961496001192 0.629149796033023 0.581422053712753 0.596588605505792 0.619096875707104 0.596184979991621 0.585693592610029 0.623746046338337 0.610821856177366 0.659416260182384 0.637226811648129 0.584383095359315 0.585258818517884 0.607425434115285 0.57357500197299 0.56842756875281 0.610451974775204 0.588664842148748 0.626239433347679 0.611473511228756 0.584122820985909 0.541401403051961 0.690997061555681 0.549111817336282 0.666324659278163 0.599515893563265 0.577216811034231 0.598319562059759 0.610397217859386 0.598976387673208 0.675929216384292 0.558235068024451 0.571802633171227 0.536835288468259 0.572976125055656 0.568425273895904 0.586501988625837 0.601471932832716 0.564677757061652 0.58622051405626 0.552039728294831 0.601954429199117 0.586050618702781 0.583202165600676 0.619083251988439 0.686631483418982 0.572010445264023 0.547041106824759 0.579407840578558 0.560166162922134 0.555901184545913 0.590507787922808 0.586563677293244 0.66048469930743 0.564162360286087 0.569748664008222 0.604862131100737 0.571917933924666 0.647623501370616 0.570244599496081 0.645879624242055 0.603854394448667 0.595146564855083 0.602298412302359 0.612035942577895 0.637762311541938 0.608020291670008 0.63909704019496 0.596168078651156 0.632159869636571 0.623450039827162 0.572899507700529 0.610491374773045 0.584602952693451 0.588181089182709 0.565024291337267 0.632421907303271 0.622173049640021 0.592402684907276 0.576141491635211 0.625888465209544 0.663600034438155 0.607753094641091 0.551985530771098 0.640395581369292 0.61251070220791 0.62861522011864 0.579971369632771 0.62820732047352 0.614528791303396 0.604659202858399 0.621308335290566 0.562120323974827 0.56191332210223 0.611290934981074 0.546672754766614 0.622147707133784 0.61528653564142 0.585240405930105 0.600469463140911 0.6187000030813 0.570431402049742 0.59395298815513 0.588594092843331 0.612798923575348 0.58062505192664 0.601345257278757 0.569212831586334 0.574045553347865 0.627330721232948 0.579247614344654 0.568947551699944 0.583904192813445 0.585251486653475 0.583603173898479 0.577943290348007 0.56399393218665 0.553974653431632 0.603834598519724 0.564613891473084 0.581494271671709 0.593023370325297 0.60963616202239 0.57652197681015 0.591782789010111 0.593331359441966 0.569396610332978 0.59710480036606 0.571518037449306 0.600639062873597 0.567088924876406 0.681204514586595 0.580703287532493 0.64310816096925 0.569947697910512 0.600066468243134 0.609990358776623 0.576453457079579 0.651019339217729 0.575058004222797 0.607891441149571 0.669311979733331 0.592619073881773 0.600168174303101 0.578539724572167 0.58468761806276 0.560524120780883 0.599276821926076 0.609174047169476 0.571381192223544 0.694402030794526 0.638573596691564 0.586911208602826 0.597287938415968 0.562740672147344 0.567905657470233 0.597341656435887 0.560003236562213 0.585278039177324 0.57577733523788 0.626095001278404 0.622824771861781 0.533277843357134 0.556750655348714 0.601840032645928 0.583873673398017 0.61668984753507 0.596503541570083 0.580190038817267 0.648775553807143 0.627020626015517 0.595309197255869 0.615719135913471 0.580748618373202 0.600517930098902 0.568742336186933 0.639047801905802 0.616205477739397 0.584100152688381 0.642050481970859 0.600749490861894 0.682275719778025 0.578551922181386 0.57430711773268 0.577711764985999 0.559856564306259 0.60890118815275 0.637651734265112 0.592660682410917 0.614071142118338 0.567919561738544 0.57084355423705 0.562372480484073 0.608538281153443 0.617121807174804 0.58042266039583 0.577085826195517 0.542405432009241 0.582315448779852 0.658816815129363 0.570614758269839 0.558235938402145 0.613088365257575 0.55638917107486 0.573598579876591 0.571287189430714 0.596802043733143 0.550811442359928 0.590000355098095 0.57215058447456 0.597322455105851 0.589757019599981 0.664922126499545 0.579888689827414 0.551595324444504 0.679182292860585 0.633984781550889 0.633807749878056 0.593948557069706 0.556790405556198 0.538946125952239 0.62842312511428 0.622390892927851 0.625170746085202 0.605022659778022 0.626518854309942 0.649887724889122 0.583624156267124 0.567207540922914 0.642784314407339 0.60426802811406 0.621632888231197

CD8+_T_cells 0.775299132558547 0.771488954451204 0.650859912487297 0.754133692915136 0.731611750071205 0.66713795089354 0.781084219737236 0.610982708107299 0.703603948695759 0.775857692699938 0.740688352368804 0.597856544784618 0.55073800714302 0.495699884639561 0.449179804292309 0.710865230533838 0.78613120958623 0.831534169650701 0.477686320079712 0.479022874703754 0.611321833907429 0.808573358124248 0.615630726426729 0.792774085553484 0.743979867487713 0.686069149971388 0.697639324328766 0.691455265620512 0.619999464675463 0.544474154128853 0.69283171739751 0.660255692008547 0.591133874511452 0.679506068310047 0.694926317927725 0.623929334241676 0.756727007857307 0.666998310858192 0.531347990806172 0.484568578964704 0.583254186802548 0.562368027229833 0.560831986841008 0.621016842075853 0.67471840995527 0.54235960502216 0.596460144431142 0.213188144554751 0.537452255208513 0.649742792204516 0.588879685369411 0.495999113286734 0.681261543040132 0.706396749402712 0.619161624463377 0.746672925312275 0.587024467756935 0.721617513255608 0.666659185058062 0.74491745058219 0.695026060810117 0.724250725350736 0.627559975160715 0.740588609486413 0.766761141825861 0.632427427821405 0.625265888865718 0.772266948933855 0.463842008003814 0.647588345944866 0.672424323660273 0.630911336009059 0.633883673904317 0.529632413229044 0.630652004514842 0.705698549225974 0.523368560214877 0.61674784670951 0.487880042660091 0.715054431594268 0.591991663300016 0.660754406420503 0.5792644715069 0.603362351892612 0.703324668625064 0.612478851343167 0.603980757763438 0.5378312781616 0.411377251866048 0.722016484785173 0.642720893284176 0.596180864360446 0.635080588493011 0.683715217946956 0.787407918480838 0.709987493168795 0.750961869255097 0.694906369351247 0.624986608795022 0.716590471983092 0.715652888888615 0.791158250858746 0.675815581661573 0.632985987962796 0.421012414305037 0.650839963910819 0.793512182883179 0.707254538191277 0.72024106147861 0.637534263399834 0.616029697956293 0.315245061817418 0.571544372409822 0.710067287474708 0.710067287474708 0.68762513893669 0.754891738821309 0.684553058159042 0.57451671030508 0.697360044258071 0.562368027229833 0.789502519011053 0.582855215272983 0.796185292131262 0.551256670131454 0.73598048831994 0.668015688258582 0.573978098740167 0.648685517651169 0.488518397107395 0.429410765002375 0.617306406850901 0.730374938329555 0.735202493837288 0.747510765524361 0.626323163419064 0.611361731060386 0.720839518772957 0.695544723798551 0.60904769618891 0.631629484762276 0.759140785611174 0.684852286806215 0.645433899685216 0.651458369781644 0.51993740506062 0.640566447024526 0.654031736147337 0.639529121047658 0.554608030979798 0.599851402432442 0.673122523837011 0.596998755996054 0.535297808948863 0.699235210447025 0.715453403123833 0.604419626445959 0.63067195309132 0.526221206651265 0.612438954190211 0.469607146606025 0.554787568168102 0.70687551523819 0.762691632224301 0.765284947166472 0.688024110466255 0.713318905440661 0.586485856192023 0.596260658666359 0.670609003200753 0.698596855999721 0.701050530906544 0.55107713294315 0.591054080205539 0.798998041414694 0.537113129408383 0.57507527044647 0.654470604829858 0.507988207750155 0.689181127901993 0.685809818477171 0.653413330276512 0.576651207988251 0.583074649614244 0.710825333380881 0.5185809018601 0.685550486982953 0.579583648730552 0.620956996346418 0.505793864337549 0.447723558209398 0.772745714769332 0.470764164041763 0.669591625800363 0.7058581378378 0.767738622073295 0.64369837353161 0.585767707438806 0.614473708990991 0.652874718711599 0.661891475279763 0.667676562458452 0.680144422757351 0.751081560713966 0.779588076501368 0.697659272905244 0.473477170442803 0.539447112856337 0.552333893261279 0.901972593195359 0.445249934726096 0.706117469332017 0.625964089042456 0.704980400472757 0.758542328316827 0.577967814035815 0.775797846970503 0.65263533579386 0.642142384566307 0.67370103255488 0.790300462070182 0.617565738345118 0.741705729769194 0.747809994171535 0.611301885330951 0.830456946520876 0.572721338422038 0.779448436466021 0.753555184197267 0.779867356572064 0.49175006649687 0.67302278095462 0.622453139582286 0.712281579463793 0.65229620999373 0.746473439547493 0.633045833692231 0.566936251243349 0.552573276179018 0.397592785519585 0.736100179778809 0.754492767291745 0.676693319026616 0.713638082664313 0.521154268225793 0.759539757140739 0.668634094129408 0.70653638943806 0.493705026991737 0.862973126180404 0.746194159476798 0.768656256591294 0.684732595347346 0.79225542256505 0.651977032770079 0.681381234499002 0.847512979409769 0.754253384374006 0.575733573470252 0.643937756449349 0.640626292753961 0.650640478146037 0.744558376205582 0.710605899039621 0.726085994386733 0.598614590690791 0.589897062769801 0.662769212644805 0.719981729984393 0.685730024171258 0.69317084319764 0.666120573493149 0.669751214412189 0.465836865651638 0.585129352991502 0.786869306915925 0.680643137169307 0.71004733889823 0.672244786471969 0.764387261224951 0.748328657159969 0.681500925957871 0.712939882487575 0.741007529592456 0.679007353898091 0.786390541080447 0.748747577266012 0.633265268033491 0.665182990398672 0.538908501291425 0.645513693991129 0.679206839662874 0.608209855976824 0.619700236028289 0.682179177558131 0.636078017316923 0.664584533104325 0.597238138913793 0.713598185511356 0.698716547458591 0.700930839447675 0.793372542847831 0.397732425554933 0.572741286998517 0.674678512802314 0.70665608089693 0.738972774791675 0.587583027898326 0.612758131413862 0.720739775890566 0.809870015595334 0.531387887959129 0.656724793971899 0.481855572563663 0.607072787117564 0.637893337776443 0.573519281481168 0.741625935463281 0.43784901285267 0.626063831924847 0.601028368444658 0.706516440861582 0.533203208418649 0.69917536471759 0.730095658258859 0.622173859511591 0.664843864598542 0.701768679659761 0.706975258120581 0.73586079686107 0.695285392304334 0.741625935463281 0.623570259865067 0.849906808587158 0.643758219261044 0.772406588969203 0.507748824832417 0.857048398966367 0.668634094129408 0.543357033846072 0.491111712049566 0.676972599097311 0.580301797483769 0.782440722937756 0.712401270922662 0.83448655896948 0.715034483017789 0.671347100530448 0.645493745414651 0.645234413920434 0.63452202835162 0.47317794179563 0.462226173309077 0.629814164302756 0.733646504871986 0.518720541895447 0.691235831279251 0.711723019322402 0.591692434652843 0.641404287236612 0.69396878625677 0.749745006089924 0.621236276417114 0.792016039647311 0.734244962166333 0.526141412345352 0.535297808948863 0.664145664421804 0.743979867487713 0.560792089688052 0.743840227452365 0.80470333428747 0.712461116652097 0.670908231847927 0.859681611061495 0.655667519418553 0.685869664206605 0.715074380170746 0.862753691839143 0.561709724206051 0.799416961520737 0.819186000810671 0.546528857506112 0.71876486681922 0.578386734141858 0.580581077554464 0.612957617178645 0.686687555842213 0.586805033415674 0.685670178441823 0.665282733281063 0.623610157018024 0.763768855354125 0.385823125397425 0.622911956841286 0.737975345967763 0.521273959684662 0.631609536185797 0.650660426722515 0.547187160529893 0.623151339759024 0.599631968091182 0.570726480774215 0.732110464483161 0.472878713148456 0.6481070089333 0.620159053287289 0.68525125833578 0.730115606835338 0.604539317904828 0.712959831064053 0.752657498255747 0.588700148181107 0.729457303811556 0.725866560045473 0.552533379026061 0.518082187448144 0.659617337561244 0.68965989373747 0.642142384566307 0.751221200749314 0.684014446594129 0.553351270661669 0.731113035659249 0.70835170989758 0.486064722200572 0.76903527954438 0.602404820221657 0.628058689572671 0.74605451944145 0.670668848930188 0.551276618707932 0.604659009363698 0.732250104518509 0.547127314800459 0.644875339543826 0.741625935463281 0.720739775890566 0.537272718020209 0.683455886452738 0.729138126587904 0.500587285876729 0.63520027995188 0.752557755373356 0.692392848714989 0.715732683194528 0.787368021327881 0.612339211307819 0.613715663084818 0.545950348788243 0.682219074711088 0.57507527044647 0.608389393165128 0.69600354105755 0.612837925719775 0.68309681207613 0.58776256508663 0.621415813605418 0.704002920225324 0.555645356956667 0.636377245964096 0.565719388078177 0.716171551877049 0.498891656876079 0.704282200296019 0.619401007381116 0.70755376683845 0.668873477047146 0.691036345514469 0.671506689142274 0.689619996584514 0.558617694851924 0.716650317712527 0.589218811169541 0.626921620713411 0.788385398728271 0.70573844637893 0.641144955742395 0.597238138913793 0.76949409680338 0.55412926514432 0.657722222795811 0.695943695328116 0.674818152837661 0.657223508383855 0.670948129000883 0.701090428059501 0.79847937842626 0.637035548987878 0.512536483187194 0.617146818239075 0.646770454309258 0.395777465060066 0.617525841192161 0.672603860848577 0.604260037834133 0.696522204045985 0.610025176436344 0.580640923283899 0.660614766385156 0.916495156871517 0.625066403100935 0.714276437111616 0.611002656683777 0.585448530215154 0.611441525366299 0.726684451681081 0.591193720240887 0.679625759768917 0.557041757310143 0.615670623579685 0.605955666834783 0.579783134495335 0.651019501099123 0.589099119710672 0.711044767722142 0.684752543923824 0.665522116198802 0.698257730199591 0.652715130099773 0.771089982921639 0.651558112664036 0.61991967036955 0.744458633323191 0.642621150401785 0.516466352753407 0.569489669032564 0.563824273312744 0.503100806512987 0.628677095443496 0.58628637042724 0.622931905417764 0.565639593772264 0.634422285469229 0.678329102297831 0.75963950002313 0.746493388123971 0.6601160519732 0.687365807442473 0.536315186349254 0.655168805006597 0.603661580539786 0.684393469547216 0.67471840995527 0.766342221719818 0.790340359223139 0.729257818046773 0.751201252172835 0.709488778756839 0.488199219883743 0.649762740780994 0.623709899900415 0.564303039148222 0.586386113309632 0.570507046432954 0.752358269608573 0.682219074711088 0.641623721577873 0.716690214865483 0.825489750977794 0.67755110781518 0.641763361613221 0.739232106285892 0.727322806128384 0.695165700845464 0.517942547412796 0.681381234499002 0.662110909621024 0.425540741165597 0.764247621189603 0.435854155204846 0.598814076455574 0.628018792419714 0.557859648945751 0.653373433123555 0.579842980224769 0.387678343009901 0.653393381700034 0.720360752937479 0.545491531529243 0.647348963027127 0.724470159691996 0.67619460461466 0.747490816947883 0.674558821343444 0.534579660195647 0.480239737868926 0.84637591055051 0.448880575645136 0.633983416786708 0.589657679852063 0.576571413682338 0.541940684916117 0.663247978480283 0.635060639916533 0.420653339928429 0.660275640585026 0.607172529999956 0.532006293829954 0.647947420321475 0.792774085553484 0.694726832162943 0.607531604376564 0.64323955627261 0.46378216227438 0.697000969881462 0.486782870953788 0.710107184627665 0.536774003608253 0.917771865766124 0.48676292237731 0.702267394071717 0.755749527609874 0.747630456983231 0.714854945829485 0.628038740996193 0.754073847185702 0.504616898325333 0.634901051304707 0.765763713001949 0.472958507454369 0.742703158593106 0.661552349479633 0.630193187255843 0.54485317708194 0.79666405796674 0.677750593579962 0.629714421420365 0.776356407111894 0.70982790455697 0.55640340286284 0.648007266050909 0.59749747040801 0.604738803669611 0.504796435513637 0.559475483640488 0.600589499762137 0.701230068094849 0.690637373984904 0.720181215749175 0.674858049990618 0.6408656756717 0.697360044258071 0.63430259401036 0.625206043136283 0.745136884923451 0.536135649160949 0.69578410671629 0.793113211353614 0.629275552737844 0.725886508621951 0.731013292776858 0.733786144907333 0.368068892331793 0.760676825999998 0.834287073204697 0.552513430449583 0.681461028804915 0.713019676793487 0.774860263876026 0.619261367345768 0.57655146510586 0.611242039601516 0.586485856192023 0.640386909836222 0.679107096780483 0.481396755304664 0.612738182837384 0.621355967875983 0.604599163634263 0.46298421921525 0.689420510819732 0.71978224421961 0.671007974730318 0.558617694851924 0.530769482088303 0.63101107889145 0.839892623195082 0.672364477930838 0.618303835674813 0.691215882702773 0.543097702351855 0.462505453379772 0.586306319003719 0.684094240900042 0.54054428456264 0.76167425482391 0.624547740112501 0.688403133419341 0.60180636292731 0.809650581254073 0.721757153290956 0.533163311265692 0.758542328316827 0.680583291439872 0.682099383252218 0.577309511012033 0.516605992788754 0.608050267364998 0.706456595132147 0.620498179087419 0.653592867464816 0.653293638817642 0.703584000119281 0.809929861324769 0.696502255469506 0.503400035160161 0.722335662008825 0.616628155250641 0.703883228766454

Check-point 0.606856617833377 0.591035738422782 0.575805097702254 0.604685340320573 0.619655469965611 0.628264747518403 0.692296352437186 0.62320588926194 0.656058565260665 0.696539122449183 0.648287183596439 0.559068649556502 0.548104635685328 0.552143295354509 0.524168364437727 0.616950736301529 0.673217250810699 0.597758075687087 0.547671769935595 0.583011380732544 0.578564558000571 0.696616623065948 0.5700292513093 0.683265205638352 0.652721089603915 0.626612831779405 0.647578565247651 0.545840408651908 0.59000779309642 0.648148606628025 0.657453228370734 0.602728656922292 0.56121080996195 0.661457504267086 0.649434934534386 0.566113655887252 0.572830222820283 0.597331926492357 0.616017654231716 0.573194231081878 0.598694915560461 0.588992433741422 0.550209243427802 0.625505223504029 0.584682164664192 0.582903400537841 0.659217889989673 0.570383598389312 0.634010136758634 0.599365248566391 0.618454952750137 0.556018945978393 0.605184365041062 0.708520891915384 0.580352317124006 0.631235618883397 0.573989006001815 0.656363810763777 0.575723241709962 0.601583522374236 0.698848780457347 0.606409375211728 0.58167987544825 0.616575696842105 0.722032489188586 0.642671709059611 0.564406246849252 0.676857862008166 0.563260313748699 0.655536621479565 0.584624068041133 0.592147633848999 0.623827493965656 0.5366691778156 0.600214298104967 0.570172851331376 0.574368077661804 0.635106101328645 0.588926770329804 0.670830266238355 0.599483757840562 0.650012993138958 0.629612935854509 0.645588088112326 0.67090171904423 0.585866945682557 0.584679410361681 0.570497485429193 0.583282938337128 0.649262439657421 0.558385957712596 0.578804803498582 0.59449084964577 0.647989402255225 0.654343989581716 0.560474817127588 0.574042316296144 0.66271056517356 0.616313376510347 0.643730482792691 0.651939248874552 0.676948487028401 0.601982499400753 0.647862297916665 0.579193688391006 0.595651642748567 0.632447788463038 0.639597304103734 0.642537212466225 0.640423003084203 0.623068927436839 0.583101037673894 0.594998899054807 0.664742672606576 0.604269490259766 0.649894530148832 0.664466116590447 0.636112004664332 0.597776340459303 0.67128919321819 0.616535932112036 0.686947710756564 0.593913275245815 0.645900162551185 0.62750622125625 0.667808604923829 0.646131624556459 0.572086223713889 0.614951619021249 0.571912769131414 0.60481264466415 0.625964686526416 0.671054044409283 0.683313843151361 0.671246759019263 0.60907850145782 0.57405053492658 0.618032280047903 0.571880214152572 0.606275808409889 0.59316668865395 0.688706501066566 0.658597347735447 0.61014133574749 0.618717375987442 0.581554991888524 0.607173675306991 0.577652765175072 0.587267622246498 0.612657792579462 0.584787115430701 0.605783037750424 0.609194599233537 0.636503519387584 0.587213789950959 0.665119238880368 0.634690046544447 0.634987079849127 0.659364659732196 0.567288817539064 0.564654643956918 0.575628117948536 0.570577440361912 0.580522820952391 0.639398430058129 0.622593125494902 0.583275073560633 0.605383594643447 0.632737358243415 0.565285861310113 0.573957297631983 0.702026301442677 0.566744319711881 0.599764219406651 0.671681974552738 0.630745156008668 0.58416269999474 0.608731100063528 0.556602290224317 0.572507040981163 0.66024737303702 0.562196295884435 0.564516108005731 0.60915693862441 0.662415384311706 0.557090711328143 0.653526108050597 0.571143437071789 0.586500654332656 0.613181606210877 0.52945614498868 0.657434679245751 0.583305748087506 0.652057312873228 0.66011319648221 0.672853346053832 0.606588395106834 0.578784522651999 0.60067652062181 0.616393787991938 0.667235756701617 0.619089903794978 0.636310646520386 0.68853670058722 0.558880390138213 0.587614000092352 0.583097773255681 0.616121917675992 0.61110071031473 0.742981720978979 0.559264070833124 0.692104551587144 0.587579234982956 0.65514986684236 0.686744819639024 0.613668233975773 0.564899247210908 0.540618304995043 0.635757730479546 0.539124649143279 0.652611424409772 0.564771752641678 0.645808179726831 0.624560063141124 0.587547347217545 0.740935318719274 0.620561167096037 0.64762107301747 0.646744840218812 0.656699995771225 0.623736402015283 0.585515616454833 0.581374825670717 0.677865086456751 0.619356287864544 0.669411265790184 0.556387358347194 0.56990846889033 0.55500159147803 0.593600168550278 0.645787916522595 0.600797410877022 0.625135189163126 0.607558477486947 0.625081290203394 0.685862620672955 0.616073246862066 0.587563353129687 0.59802337798775 0.750158411379385 0.662515518575314 0.653245248800087 0.659353309781048 0.689201889770059 0.598834284665301 0.658752932677087 0.737284095434288 0.630395007450929 0.579684850209239 0.653059273067921 0.603260360691146 0.617426495044282 0.547247547852501 0.595047693848809 0.615016824160291 0.582252657461809 0.579243645639063 0.523424686877079 0.686104625872828 0.712403144259067 0.660578933504529 0.606800301090133 0.583550440240121 0.603535634250628 0.547844122166408 0.664179676837264 0.649653465419669 0.654491832272978 0.574211676017727 0.678645593953058 0.696631341346731 0.622985053389272 0.553173825470108 0.647700750464844 0.647220317631449 0.700884504556925 0.633034210011422 0.55862740897131 0.647852122951392 0.597179790129614 0.580808501159219 0.605681885693137 0.611463505420411 0.553067087413647 0.614468464815352 0.637516833500128 0.571199884089296 0.634085340874506 0.599145379759924 0.575253183496939 0.64447357879033 0.663364603645846 0.576056281491129 0.606204420071194 0.631869773710418 0.592818859115235 0.678291233271749 0.566032974384835 0.661870350374388 0.569639331619889 0.692394949828717 0.592999396159847 0.598266006620272 0.577840191517031 0.628027411338632 0.656306240463542 0.544474769618069 0.58833429948059 0.574528859909094 0.652924235412212 0.58971282804401 0.647770972148634 0.570204309599007 0.596812222860269 0.671588489378046 0.593547102458495 0.602039258223921 0.641839947344806 0.64187893581906 0.688747540231612 0.619243222450468 0.682434896032255 0.624629083473715 0.740676755907909 0.614153222283389 0.625374103682375 0.581939208596243 0.723809323619301 0.648159748074771 0.579600656363565 0.575717585030275 0.594318784721887 0.579353429661852 0.68388629422788 0.682436202783382 0.757282540801261 0.568598304600173 0.57148633077861 0.610598679779034 0.639599550352832 0.618355895121946 0.532521757672211 0.559260552262298 0.625863071229003 0.62636588133609 0.593197296191785 0.634474950422065 0.660223106425776 0.672382712933169 0.620814807095919 0.57012857303179 0.596846121480195 0.633305163749289 0.700675547610531 0.65433799877485 0.600560614372636 0.58489853538678 0.593742279931351 0.686872040101245 0.587191019755047 0.669131890045517 0.729571071705132 0.640124530977683 0.54966013720687 0.613856210915803 0.585854027265959 0.61545103646046 0.663213582489321 0.640282197037987 0.60724101352412 0.623476120235232 0.743623013378983 0.550290281638379 0.558796326524352 0.597050136563148 0.6193088376047 0.587624533222578 0.669474845178157 0.559220803882546 0.678108169779411 0.58692949143398 0.597899926848009 0.644991604951736 0.569772486483409 0.665643842792421 0.698685021376585 0.60432989144014 0.639991718307907 0.618794629876743 0.673394512819628 0.590769502346297 0.578290630893892 0.639790498615752 0.680315825732763 0.537073476452088 0.596563051073921 0.626346867054087 0.635417153137059 0.596554086712794 0.624888638910796 0.617304055844924 0.56219848767753 0.55281134573851 0.626993370820454 0.557363427926662 0.651165352747366 0.569227712512703 0.623019297278356 0.672564237712924 0.609463616665773 0.673230503392577 0.594934037347795 0.600894837284307 0.603110861875025 0.638255740586799 0.633139939951932 0.580627695069103 0.591032974666945 0.580784324102578 0.557099121035824 0.637384578539389 0.583773550643488 0.626363536749117 0.650454685352891 0.584694769506964 0.558965556990009 0.679343553919544 0.668006507893404 0.576770483469703 0.583945063516579 0.678004242047555 0.615394339846775 0.630419837802771 0.627612689738976 0.605577386252705 0.584651104296678 0.670640511428151 0.581640286590625 0.62751409984499 0.629332378070838 0.578324076975679 0.584640825133883 0.618409007905636 0.689350928799184 0.618974735773703 0.644585645679363 0.60355446152528 0.546937928208695 0.645072779485019 0.614676911375593 0.584319207193838 0.595759414806661 0.640061521624678 0.540404476629438 0.64043964276066 0.611667159123775 0.626267839619497 0.554297487747308 0.612910024677007 0.630136836193782 0.539745037148611 0.594612034405239 0.557563443543371 0.637868341618599 0.615200307883986 0.57690070833712 0.593297858521468 0.623651163869995 0.569709566756263 0.672721836708846 0.607993821349364 0.632574291104302 0.612234818125292 0.596629016198672 0.581361601795861 0.626035710270751 0.633999323151463 0.704551621065459 0.645307666543134 0.584322992133847 0.604261273635849 0.616321627227722 0.565788510152271 0.565764143699605 0.621722579355346 0.602535170255283 0.652705071034607 0.643185325578756 0.574507086048715 0.554195137032001 0.768794464147471 0.563801342963028 0.69610127268152 0.619438276152527 0.582119712166307 0.61598261037968 0.624288444737907 0.6207049623005 0.688311662033227 0.575268718932296 0.586167310681561 0.543703964747885 0.566999722004165 0.568881945007643 0.605008360629535 0.589633304595688 0.559278420924178 0.600686481618818 0.550104223725939 0.614252099138819 0.576758333436837 0.58372720977662 0.660238166317773 0.705103664424599 0.589762710410256 0.564802500832082 0.587126439966386 0.557002269952693 0.553267613954995 0.602818175704979 0.602744473458598 0.679550433627666 0.572982023904891 0.558935335445582 0.580104305029392 0.569927877359927 0.684992920079468 0.559607430542137 0.649713614404323 0.613438607122296 0.624239778962889 0.622568162768624 0.64629049821071 0.638942056912737 0.594287929714531 0.67062107681202 0.591617387302665 0.654099761078187 0.614673186102575 0.57386767759353 0.65421114233473 0.616808438477059 0.595616158057336 0.586467118311327 0.632845449802911 0.642877813512747 0.580222898043459 0.578506673435767 0.652723871892324 0.689529928177437 0.637455714294519 0.548041440065579 0.676504746519854 0.650110905172354 0.645836408890259 0.588457959601476 0.644046956479298 0.635844574654114 0.601430573891108 0.654004558491222 0.551122053909494 0.581585769580927 0.625475273031287 0.559971874265818 0.628958117329035 0.630288167714753 0.580263430969675 0.600530625231309 0.649294761686759 0.585331723203956 0.588109701968497 0.578537312954068 0.654472608524034 0.591482822989691 0.572953640072144 0.567731006876593 0.563701934316676 0.666635243678411 0.586797750779236 0.562784553373678 0.598560277059851 0.570658679387849 0.595768100695205 0.588754552733843 0.568118967335467 0.543563572342288 0.624400336367403 0.562332419675373 0.599211377242293 0.618899636448501 0.646175537448035 0.58028697877586 0.62014218657152 0.613600756889885 0.573511509573643 0.615836475971862 0.579336457299804 0.59972394329554 0.555938109643536 0.751885428554402 0.594514876855919 0.658653343376744 0.597578069300045 0.653855426531119 0.645929454807207 0.589268620020921 0.67160710547077 0.59288626735537 0.635920370561771 0.665186015427527 0.599366005395007 0.608788941355656 0.559892437409573 0.607844891299213 0.565859511605947 0.607304387379059 0.636442925028711 0.556255606261113 0.720426884834999 0.677157205184953 0.592303406531323 0.632569966045519 0.562285133833601 0.562547724698912 0.611956692404624 0.578149138549269 0.58804436296425 0.56423652410961 0.639104029682559 0.635419097703885 0.518301411768572 0.536892974694918 0.640457606676677 0.581518294842258 0.631417833976134 0.634099921491091 0.585281539114769 0.675621016243409 0.673731985545057 0.609302787423168 0.644445972234983 0.580203382400728 0.602010210764038 0.566885898625171 0.677112093575884 0.664650780207666 0.591772959223043 0.622640402312103 0.616474535011769 0.725404830215393 0.598451236494564 0.567627915093504 0.589448800361376 0.569598548248872 0.613559151220332 0.668222511053595 0.596074609549975 0.62593489656915 0.556170169622361 0.581800356912758 0.58514710429819 0.64582898563786 0.653663294173581 0.59099079697188 0.590717569166437 0.5402635708891 0.607169345620069 0.721632243962155 0.575660317257052 0.550176736187733 0.670082184084222 0.552834644431962 0.574588596418672 0.547436740868024 0.607267030827193 0.53871286395371 0.586674696401166 0.593901022685121 0.64226347179586 0.592433574033972 0.727369878820483 0.58390387515177 0.555329251564175 0.697562772256619 0.64169482311477 0.691383740564063 0.598210575948176 0.556988909751822 0.540042490835888 0.656824191490249 0.639946846718416 0.640495632978608 0.611926131323464 0.656342708884817 0.693363032858253 0.58735953722004 0.584691383431035 0.664653042621757 0.639668024501915 0.661525956471428

Cytolytic_activity 0.585641210814697 0.64274064069465 0.584430249346381 0.684356376032428 0.619514284962496 0.682831644292417 0.786002360265813 0.638733836935848 0.683596585806023 0.865986716179507 0.74969150555909 0.498691909787198 0.536747365302032 0.650959198145332 0.520269140005779 0.648559858575513 0.832129659712505 0.673398749284054 0.590722931700448 0.607437377059665 0.730427391045437 0.811828257945523 0.606016757508741 0.844621639238599 0.762679815098408 0.686848689330851 0.751033125885767 0.546061061681718 0.589103743933094 0.693522994476711 0.801098846867152 0.694803589258618 0.519980380101972 0.78306879495726 0.765349634105535 0.597207800974109 0.563096230258671 0.664119332514829 0.592795448549337 0.521617079505575 0.666814059881052 0.654756628637772 0.616590577477827 0.706846190823331 0.672957346843937 0.652261853744085 0.762850425027086 0.416593198860886 0.639704211177075 0.716703566370511 0.629705584273344 0.287376986642852 0.642428958500849 0.879952812872942 0.668692446096373 0.687430526073757 0.614916756436667 0.793169982180698 0.625459875420585 0.682761650443825 0.825064247141877 0.725741488849408 0.598384441333581 0.70334415348223 0.845520663564944 0.72388891919365 0.626165017832 0.890793738949055 0.631529289101558 0.868554425975739 0.692675210693436 0.64964960920233 0.668758056655141 0.480879971919233 0.722680026253798 0.531059542032698 0.603664027213763 0.676386307896796 0.666213768043317 0.762135356690036 0.593139210397722 0.686237064452089 0.584736070602136 0.643917821173095 0.77209356279141 0.686436084981754 0.664687064120808 0.547610224588642 0.561051112660263 0.708130939283467 0.640403301134809 0.674813286570715 0.681645396779619 0.716828295785052 0.725935101325981 0.415110111545328 0.592034344691959 0.761731322592605 0.672647898766155 0.736767091894446 0.725154229610159 0.781775563921682 0.688846941069579 0.671792786312377 0.523133844997565 0.55701048577685 0.769242862788078 0.765607796644477 0.752184746152229 0.755983685329896 0.667657183863213 0.47509081191355 0.663415286823765 0.723753810133733 0.654799939026237 0.799183838669256 0.806687210420892 0.699225259759041 0.564978942107669 0.735228212558515 0.603617738107776 0.842895673643183 0.643560763577801 0.817336915431295 0.577227125703982 0.720035120076382 0.729715550947562 0.540125875326722 0.672685495711962 0.330735091059061 0.566894834730687 0.744606329058312 0.817822963829876 0.816715351990622 0.842939325155778 0.713399009237617 0.536036059551533 0.678270617846267 0.598160890112016 0.64049524929669 0.588746164079773 0.866271561097403 0.704201616095764 0.695955794040626 0.63982648640427 0.589731015873587 0.745325825940316 0.668185069926311 0.6371934780395 0.644028525078762 0.643382882601165 0.727343444249647 0.651326726253404 0.679747314831603 0.65359672127593 0.791479772357926 0.666832014239682 0.55132369430679 0.676465193576145 0.571866923166621 0.550822759691775 0.475233440429782 0.571237354000516 0.494152029643805 0.660099669557812 0.728544856759524 0.587469182541552 0.553100329555933 0.679160087653759 0.361990004480969 0.645608064859737 0.777831109361683 0.531489594860479 0.639503775517226 0.800904344078437 0.715795721072999 0.59361234222572 0.647561386935121 0.44681860338754 0.638592666732095 0.751169553818772 0.625530109831034 0.577858235531981 0.559146417433429 0.814854792268467 0.279187905192331 0.77887476339905 0.468846192871276 0.645003550756048 0.604134589119996 0.612982115528104 0.805209141836587 0.56893534432763 0.75274975550642 0.774489560267671 0.732917604228853 0.626927761412126 0.532407171267626 0.66967265766947 0.689075954812063 0.741768947317216 0.725393507181971 0.699535338279051 0.74750153572084 0.466241146703674 0.669522984879934 0.520972720112939 0.621918303669041 0.619514266892887 0.928248064200428 0.637075157202335 0.802816174370769 0.670519628314007 0.69793863712867 0.833131785307746 0.656892380633953 0.495846033122759 0.490556110154881 0.688119185112089 0.557965102682508 0.782549557091832 0.530332625976799 0.760159786394431 0.7517626495846 0.513358671311463 0.817812031255575 0.500391986246547 0.761291316033303 0.807145791969362 0.828450232694273 0.584572812410891 0.677784831927672 0.650116153700407 0.820649794391709 0.703384867460231 0.816563113988309 0.532408972822208 0.582126111222458 0.594936931724832 0.497399622973035 0.758917087055255 0.599361238763438 0.637648219723278 0.591468481567605 0.704324497678055 0.788226444770327 0.679395599976541 0.665661613689006 0.541585141754148 0.902364268728314 0.785556095487543 0.754802957192279 0.74676442373291 0.804804787818411 0.610939133559608 0.73508822423174 0.941580089647821 0.70091589271925 0.581103509005646 0.675779598638877 0.666612449612835 0.66078844441949 0.551607836866631 0.666722694947771 0.733407368419527 0.580128746395658 0.618035011490787 0.410990072326932 0.868577714857361 0.849498892358648 0.73990565011567 0.693079598811338 0.643679247358558 0.588690170584543 0.670121166969699 0.814083869459694 0.741223657087836 0.849976971376176 0.653068092460706 0.783640898707747 0.872582660957212 0.629181178281778 0.560290548045311 0.774038701036329 0.690132712061442 0.829586964443884 0.684686741112319 0.595666396677186 0.76883275342969 0.570830998367126 0.489827445990398 0.637216145439756 0.704616163369131 0.658540095445187 0.728830117579648 0.713254675044308 0.597432047449673 0.680305517627913 0.578957653071998 0.57616610769101 0.749416494993806 0.812353369331813 0.501439804744819 0.652340222825 0.679882243556423 0.578186226044705 0.788515092117097 0.60462451200383 0.779618497965214 0.597015036521957 0.795242756687525 0.56172316149652 0.609899540639812 0.380630526488737 0.676581700388986 0.687750178817636 0.530028873102863 0.565651176634284 0.580214324493913 0.806408975436678 0.667403883031933 0.716968189537581 0.597750744707765 0.63165959011248 0.790650603320406 0.514478582427422 0.631485872582275 0.70987270870969 0.707942426071875 0.811875094200394 0.70969717285001 0.812898707946609 0.681399679213107 0.879397948142069 0.651595769884294 0.743555421328687 0.596504298267935 0.839995346220837 0.757820405885103 0.692649894294224 0.519796287222262 0.637312502158556 0.527001443153788 0.826606195305837 0.728012731242679 0.864880188352234 0.657504882229128 0.370360839737643 0.65020648090684 0.640092897611317 0.760424187927947 0.498649663094357 0.5338298683423 0.599580869783157 0.716907198885482 0.609135277407354 0.719674448585019 0.779885599578657 0.815212971537701 0.641212448507182 0.642896626136095 0.694039866794995 0.730628377169573 0.824870930840146 0.723032400003458 0.643641968565361 0.629148552786149 0.677243718293434 0.831529695658287 0.554068308877724 0.778475505606885 0.851679804831066 0.751573495348354 0.54229035003176 0.701883925502518 0.649676797646199 0.702939646586398 0.791663490924646 0.708061903947889 0.624359208143455 0.771878210038465 0.875034208536728 0.561283819439782 0.528497518807832 0.577309883461494 0.619454093393142 0.646744637007598 0.693352218707944 0.0522462437022742 0.826936666658944 0.650108935450433 0.618204034056569 0.788142568935527 0.480802864070381 0.702685150305278 0.870812869678245 0.579353199965019 0.68721073304336 0.641592480491838 0.619949415218347 0.664459834437627 0.382374075993901 0.746483551348688 0.801717505096252 0.592469459805102 0.711055032462309 0.615145130776321 0.762339533548911 0.728611257756066 0.778188204625583 0.706654252418629 0.522256851878627 0.568753160126084 0.726103078212234 0.492046403020663 0.725969558347554 0.561449384151893 0.733778656480528 0.797559494360403 0.738439493961254 0.76781370232291 0.667621736609472 0.634791021588606 0.767553766763657 0.733606438602539 0.57950761931334 0.608821363988654 0.590021330675627 0.630845227376217 0.579932318490126 0.735464760643375 0.613339758036002 0.725355233991922 0.745052791347688 0.53094640216402 0.516929020904359 0.777410971082217 0.76076027337153 0.598735880296039 0.666494471669864 0.784177146849595 0.713982484081889 0.745117006013597 0.765194173517429 0.597529579024326 0.689116310381709 0.839908944213143 0.62660253097625 0.675521719305231 0.676355257638093 0.636914675071296 0.588976595909504 0.688781939046769 0.842711819714072 0.569475233995521 0.753618504198544 0.642489751891284 0.509280272567212 0.719250895632582 0.655774887022846 0.470330672511838 0.631143739890471 0.673640738837483 0.375316359701097 0.682314248965264 0.621862095122855 0.815813668754048 0.577246625709063 0.657906788493298 0.770212210015793 0.443777107303426 0.500748331695484 0.578591719166238 0.689135465029753 0.657000196864796 0.67269881630824 0.681711680524663 0.720439168640874 0.513275972469196 0.778757409857796 0.556425540094465 0.655842436036745 0.725604574305835 0.572568797622692 0.637240293025977 0.688165611339532 0.720544826652618 0.833922433945785 0.664508216252191 0.541929765694235 0.659956546082447 0.651513072130497 0.445087568150342 0.553312926812036 0.655982746487209 0.558444005541298 0.740762124282194 0.601852953120896 0.559869586962842 0.592957037128719 0.907544724347622 0.589617259885206 0.871503183327085 0.618068387803139 0.579934732278184 0.672055045284272 0.717682847551308 0.52279809062533 0.79478512984894 0.696076654822648 0.621769539705066 0.534484747311623 0.541879058207076 0.576893385346053 0.556991031874771 0.584250705857972 0.568661862631827 0.694386453829967 0.600704639532757 0.639493711218641 0.576541590273525 0.784704195113218 0.75142426359369 0.867845504452767 0.369029314783639 0.684206840570347 0.526510181699706 0.582040313832997 0.473806898199215 0.701593680276035 0.668504190109833 0.824637021739935 0.551853665858628 0.609590123121655 0.659434188906007 0.587253335746955 0.82416610663336 0.554961112830296 0.751034933318546 0.588274590390173 0.779016422593586 0.660170304421799 0.782356288449723 0.596330250498256 0.646450813007316 0.767657250831952 0.641373546904089 0.777149290269903 0.694542839220411 0.559735625860228 0.722015849687178 0.640192061726348 0.578124523772205 0.489474366019777 0.699725961925497 0.783005407609694 0.625187259726392 0.730542442092371 0.725022103069244 0.84639714903531 0.722579436248097 0.644887583606744 0.775905261333286 0.746133815572867 0.744843006694523 0.555793467778389 0.695856293681583 0.71910292877756 0.638711450448907 0.801239197479334 0.530458757880925 0.562304822158018 0.692511705749344 0.488634418218423 0.709400610385132 0.722288198494458 0.573593728433014 0.693282111423295 0.795932960242349 0.572269121266526 0.653211817926162 0.644104485397108 0.781198133216809 0.511202266782332 0.673027819035292 0.539283838179284 0.461114713457412 0.867424341955991 0.565418199913005 0.594228335229363 0.579776112902115 0.641350222297931 0.612306640346989 0.645631543335446 0.305640678151371 0.499257315209958 0.731803131794566 0.648705759202967 0.580430683742962 0.681196184652026 0.796665963457269 0.597585571390807 0.662740862538172 0.686083677231244 0.424565381972861 0.680254552508825 0.469677957362234 0.768868555543241 0.611428989491725 0.914566893728588 0.522466687680799 0.777676327680679 0.601965166126533 0.692026233353251 0.716461276141878 0.583100552726068 0.829915740767174 0.653028917566112 0.690467557935522 0.821350954383268 0.60383678689679 0.712955282207304 0.589026748715556 0.608915150430877 0.575588290701509 0.618343607522602 0.721303666560635 0.524709886671561 0.842090892947501 0.745761606187083 0.46898139155527 0.701775622547474 0.63130522718811 0.562953035164713 0.563707036225644 0.563997501694005 0.572375307737979 0.560014482512547 0.74432028284278 0.733114613739841 0.276422737933077 0.650846744008463 0.703497899405258 0.609174102970196 0.65819884440246 0.73656480479906 0.460899177829994 0.81386634481895 0.768419057896624 0.677583293542226 0.700696777299301 0.67940266696402 0.60442738121911 0.447520475059345 0.880574865369435 0.786263629204887 0.514556351444041 0.678410220746491 0.697523644289012 0.857543986056636 0.683518181745401 0.556431074761442 0.487292672429317 0.559151729787494 0.579513122972868 0.854690594976623 0.576125288627169 0.663665716014316 0.67461750897441 0.608122915207017 0.542686461526371 0.722402786142413 0.762571342419867 0.700383010410061 0.541987120196345 0.526986481128515 0.659789242540941 0.818466123942906 0.322509802276355 0.459761426880763 0.853762736376938 0.441689915695864 0.554926987037758 0.548785358329112 0.678149607301593 0.53643709274707 0.588514237173087 0.663463559299232 0.741152675294798 0.627805608739999 0.824494128421181 0.600655506813013 0.375249799027163 0.835186797834993 0.733217514790823 0.729269794342684 0.623133576931411 0.678107076389956 0.463343213372986 0.72426341785693 0.652119561654987 0.766308584820901 0.666880820728309 0.745041058345366 0.872180744294977 0.551307020615338 0.663441292945378 0.738371895105497 0.694270814477714 0.734220621200557

DCs 0.593519321539431 0.375445388599981 0.432810501680704 0.475523315902714 0.548222137516808 0.518506779073019 0.600045994170309 0.554712151183489 0.53582780722202 0.623849143325311 0.465745202603359 0.533473550695149 0.52049843250513 0.374940134709244 0.470122738785304 0.495832331787345 0.573330508021999 0.36403359213122 0.512767237518299 0.563121344067225 0.520799985165956 0.53423094878798 0.462789293662724 0.594434316726741 0.525218062520034 0.45565907234487 0.555342827068116 0.481766466369453 0.501971439057547 0.605495748242909 0.505168602138086 0.468415824129643 0.527768372981101 0.644431731488602 0.583572225079383 0.453018570540527 0.454259897008677 0.63492080930689 0.52763658051062 0.507669979284562 0.585012659055438 0.609669873952801 0.576537227787584 0.512273731391298 0.515134845870324 0.525387745555847 0.300830566074361 0.533275544908494 0.484121336091037 0.467114107034181 0.429248565069607 0.341119629851097 0.545830410299579 0.683024485884255 0.540450154071087 0.45205437815472 0.496227634533558 0.469947274991216 0.501777325483468 0.55345109837413 0.704663097921094 0.46472438436463 0.495788364827541 0.593488258770219 0.793513843078795 0.483615932956645 0.500371512899678 0.660500516400124 0.5394315143347 0.608898390290839 0.475562524171646 0.484203253511121 0.510182820750746 0.438972471262067 0.448780662924113 0.48716068520873 0.275031069552733 0.574027729814745 0.542856788964477 0.600536588857044 0.446099276732512 0.513827087818678 0.577887433357148 0.604023043818346 0.667450024946041 0.447654475219971 0.452976166951075 0.446500277330764 0.53093559410146 0.582877636179288 0.493821506040248 0.557726761363213 0.392024657459969 0.530889662117384 0.435203911567547 0.434762953635015 0.500196343576 0.496455749717532 0.463395382902152 0.444511953863658 0.460111406984594 0.61061624075727 0.443606605900926 0.520005544641403 0.515535591955998 0.565421480012622 0.355291038349197 0.474825079022318 0.601132160036485 0.622341278291201 0.266117348708012 0.516057039544769 0.501361943456467 0.534931828866301 0.424985903867175 0.509289562848052 0.51119208575206 0.522316592244999 0.543602168414209 0.538062812572108 0.615355461536486 0.513067677293096 0.582299158497329 0.537615601109372 0.572147268223691 0.49996418309095 0.47413768968062 0.472612792673353 0.534152677543202 0.553877095400265 0.538384752533601 0.515215498958945 0.600306897962898 0.553308730042446 0.550952715864064 0.560995444073719 0.309750169904907 0.489061001271971 0.411038549602737 0.39453994547331 0.56140533327182 0.675714632444701 0.523348943468198 0.504564216605409 0.529168989640615 0.529744591946287 0.576794863624875 0.536727914879608 0.523861634998229 0.533473581772113 0.546383046257642 0.43252480041072 0.504343148646102 0.598150880210496 0.459707714830719 0.624410715145689 0.435967760623057 0.497849021613533 0.603549480605424 0.533076581758889 0.498268822719224 0.505471328025708 0.461963583549218 0.390461820190123 0.443630697196217 0.492649625421475 0.482474672100922 0.51900076692611 0.582881475598184 0.528129914716705 0.516621351426769 0.69907176617127 0.451498992787252 0.562249799315683 0.545980170503792 0.375626865259118 0.528533124879604 0.485244067924105 0.471512407011603 0.453137449386421 0.607983008555719 0.58135839143931 0.572105378556348 0.574624781966345 0.734529888630641 0.551381679697914 0.536367702132406 0.501006953360653 0.525829580670387 0.61340400262532 0.44736819880286 0.752865335686789 0.549845158219776 0.478264004243613 0.50476314207481 0.623252862036114 0.503404277750509 0.527863864233008 0.523493034625075 0.624321721000556 0.564867342652566 0.492993375995788 0.691167782045478 0.723965647430065 0.324832441522724 0.440934836469544 0.559915938070511 0.641324276835362 0.562536940393497 0.658363254422588 0.458899656052351 0.632977331886543 0.484039924668652 0.575433891582918 0.63185142323033 0.480651202741674 0.434986257218606 0.479026025599944 0.58677579793253 0.476405048776613 0.582153653506823 0.500931178968308 0.524766578966651 0.565919729423471 0.606089975184994 0.593528095748262 0.489179314571555 0.5518820206969 0.586732013980328 0.330583901113336 0.492206738087729 0.483804819670496 0.542306002711469 0.548103939554493 0.591387699123633 0.566433754136848 0.470533735246392 0.392073490809299 0.564910696405081 0.544194233187977 0.60239121159739 0.443136836152572 0.589283719186117 0.405578252503921 0.581148200982699 0.617028208918466 0.432400394824196 0.514929201881956 0.543881547195414 0.929956309386525 0.634950033236166 0.422814111391403 0.558088579094801 0.6564262273149 0.526769267165056 0.590102648559561 0.681212969958611 0.594621470886937 0.555626720270417 0.578331090621993 0.444104695273867 0.590653340243567 0.433657790451756 0.454266475762456 0.470650188955726 0.46149511996147 0.413126146806026 0.450299879115288 0.520031416693665 0.838262685769462 0.543127344985094 0.60471669289966 0.558895687812476 0.402374803284295 0.548543227413942 0.601550271956451 0.628611352261363 0.567798397116134 0.578129995128533 0.533179684088244 0.545633061892283 0.528763530685865 0.415809198962523 0.505545406389893 0.59188951061031 0.609984835626885 0.578660416332049 0.509883924557228 0.543005139554324 0.561096182994265 0.431623289046742 0.491291705492214 0.579821133645608 0.488890510728664 0.474492450401247 0.529163533611271 0.501365335417691 0.609447955055151 0.49761524485138 0.429822597010383 0.596527563841761 0.510996664204307 0.509148793063996 0.367071032609703 0.47002501040827 0.451625826532973 0.630681552166345 0.450314186375842 0.62525750079678 0.470551211180752 0.574779100452685 0.547378700826521 0.587340609637183 0.534754117078037 0.5739721225093 0.542698040230263 0.447772314357055 0.498833342343335 0.407610756389113 0.450819147644533 0.451964464589035 0.488582038915132 0.436641458695348 0.514989488384992 0.557363060591317 0.465317567753324 0.57807333821251 0.540136939396962 0.527699373794851 0.69821043687594 0.623112338698099 0.625404731966466 0.499988481257009 0.776659944676473 0.51849398141753 0.446922368708268 0.565245105341365 0.583442347573712 0.557890855949324 0.486253374352952 0.524868768171014 0.571274426743158 0.494159722303298 0.538674219076065 0.53301755596603 0.807374944709016 0.429181991607983 0.471156764625977 0.498675260415906 0.52228649350346 0.494064109028652 0.498975630557511 0.511858234986642 0.447461070835283 0.507818913640798 0.576222583728219 0.518911951781263 0.497028847602596 0.594773695817007 0.576935297595793 0.446312042734567 0.440722534287385 0.51695408028724 0.665630581430914 0.554963905912578 0.45123585537612 0.532551971802611 0.377849328788748 0.489068972019682 0.5077781098674 0.521168975625467 0.804490727250554 0.539022740357382 0.578188785873364 0.502324497487703 0.499117681005546 0.550022084898298 0.613203399920939 0.561146217382042 0.436126343532527 0.501572615528639 0.654611139226822 0.460403784034129 0.517397265183865 0.487481745688847 0.542565821510176 0.515465685823087 0.616440572724128 0.506002820963913 0.575912991001884 0.482560568803752 0.526950558496838 0.630719213609605 0.575107497741688 0.367363301457699 0.759305610423787 0.547342817720161 0.586267047026264 0.549450607515369 0.612628528558685 0.609795630928677 0.527579375904827 0.538945332638505 0.571171906305564 0.609726818039867 0.518983380385491 0.467349167279875 0.458678685439754 0.465932398021013 0.551564611858649 0.545254990979504 0.484241757326035 0.530468604928188 0.410744585428349 0.379001702892364 0.60324410961215 0.485431385588019 0.581997485917949 0.543865585571805 0.534247383981443 0.530033133542136 0.605877852776139 0.533379280251115 0.575079397564744 0.482439048141496 0.446797619455014 0.443896088881399 0.512935857131298 0.534936745125006 0.403042707529811 0.478316580346795 0.641291357849633 0.599248481021826 0.566688329639194 0.53485758838365 0.449949566230276 0.503247125108587 0.557904538756278 0.528631844973733 0.443207033478085 0.541036286529492 0.618389590656768 0.654360810564141 0.482746197130928 0.534898057452778 0.412114228686237 0.519530477083719 0.524394636929597 0.646322642048793 0.639177623038381 0.356972856404662 0.464548605733047 0.548950512671564 0.716069957267137 0.455734422198536 0.525349520523022 0.609697674717441 0.542630916853603 0.60882988150296 0.626696570627146 0.508457065084535 0.581205031086304 0.523701631833909 0.47905212705983 0.518918712985468 0.573921369413186 0.600316861540702 0.445428482602592 0.465775864503097 0.703689058095952 0.486989896134713 0.445291819631956 0.444786885423774 0.588341926857672 0.41312237344033 0.417958781259472 0.521256904124422 0.539959358697132 0.502351284574876 0.495933367630227 0.490492115340397 0.469613849462565 0.626976837408498 0.547120401646955 0.425544986569071 0.622675770282667 0.552386178256532 0.611794329512647 0.520649770517849 0.573568692829863 0.565653789129227 0.577985857302708 0.444132073089906 0.445580445558321 0.45789349843472 0.545789105297478 0.585402008621318 0.545526275147664 0.584397977054087 0.439758317572637 0.744366020083861 0.469077691087792 0.540866356998716 0.513875762300063 0.492179721635227 0.565199469831841 0.550191075553523 0.57653230818704 0.660467030620058 0.433303244054538 0.411115799418879 0.160180906472019 0.493554483723812 0.482069022087081 0.578017964823346 0.37599337400753 0.442738223763527 0.611591044059732 0.421945395770659 0.513287733408266 0.549381552912526 0.570789632252274 0.558839878428545 0.616597965988074 0.538915928783785 0.479582237302045 0.563307185759679 0.488835701966715 0.51583877268637 0.574091153956554 0.44749917614033 0.776186402165344 0.448986550927459 0.449542681463658 0.487566204572837 0.439817581542224 0.54152655300303 0.534336283841094 0.670638394837821 0.486268109681059 0.559769821466407 0.547181440114809 0.36897274760758 0.636491902385779 0.420526642753205 0.602241509524799 0.517288359762089 0.606902536998596 0.602723128537679 0.425985642663305 0.59945527948521 0.535709976269259 0.525196340562189 0.569300723074887 0.53435856549351 0.546604043712123 0.598979351060779 0.46453206583343 0.571760771074811 0.554586206887629 0.468823353841241 0.41360948839691 0.359563184762762 0.515021641186438 0.53704486381524 0.537108343045795 0.497851118383895 0.645898417475923 0.558506397614019 0.614511309885885 0.514249246698866 0.431441845553622 0.573081750487245 0.447100828033433 0.562338830792028 0.447192643448306 0.503928662038704 0.402980499284021 0.454418015020052 0.5417252153452 0.629665416445403 0.462510797238939 0.448516471279193 0.408407867067964 0.461995463270129 0.47547138973428 0.50336936755525 0.596239451753966 0.586135782654366 0.478432248786057 0.528346763881668 0.494586857420668 0.569575611060812 0.528037661825204 0.426804898580653 0.505480022410699 0.508002231556966 0.492072851996247 0.537820628527823 0.532426945023039 0.563157862353876 0.537364984757711 0.586203202347048 0.535725239870948 0.569084643995232 0.411802019603796 0.514990425957464 0.55442465335471 0.452584110128701 0.713086484210282 0.560538280375573 0.543059520382713 0.488973837838748 0.503420332197908 0.504217088060058 0.486077181920395 0.682403830434278 0.613909372608052 0.524860796000381 0.751464401402553 0.489788375174996 0.626368731280026 0.445738297460929 0.512192585966949 0.432634808420316 0.503376460609735 0.545739327371365 0.422485669310856 0.718202084157271 0.543568566938481 0.621338537803401 0.451330265634588 0.52770112113955 0.597658188437683 0.575763417293645 0.520797129441528 0.573704030740984 0.442684211405 0.613313395464204 0.544428364106834 0.417512418906673 0.429751818000134 0.490903245826291 0.548552111040044 0.593659505728264 0.402044793785841 0.523813290374901 0.558733429492704 0.554105902739755 0.343233083702042 0.608910368357353 0.379022457122236 0.481471144421814 0.438515293887734 0.371018447388465 0.626903536011659 0.493187423333238 0.560417857963307 0.502196253744337 0.827600978221559 0.521331944319288 0.542954200519151 0.463726592661905 0.298310566175603 0.521171977766206 0.582468713747145 0.57140093429597 0.518117654973724 0.469460787662144 0.614029090201212 0.527257823410263 0.534808349601718 0.506258043338743 0.538792094101439 0.503598639853661 0.535007316802477 0.230735408000086 0.538958179461141 0.423041193337131 0.411955735503962 0.365574333029101 0.501045427142319 0.55820847263952 0.524516819196614 0.491686486399526 0.543807027295266 0.347667130338545 0.485403124752168 0.605234182838146 0.438897226565671 0.672014296900745 0.516980218854506 0.489545523763886 0.688611498731715 0.484607134898625 0.586710165719215 0.562981041269437 0.47237958506254 0.444655920518239 0.596239028693836 0.516732970124867 0.572289667473511 0.512595964528933 0.628411573946211 0.6183745119856 0.510666082554332 0.472101425785336 0.566737078174806 0.561791663729296 0.52960454088302

HLA 0.81998806212498 0.808145432437375 0.8326691723221 0.79657056332529 0.799171032135972 0.861415303690914 0.907133825448753 0.87623305009316 0.872809140013796 0.929801643934699 0.916368529645847 0.830180675292656 0.741724182802488 0.753672630136695 0.807740777953841 0.866716339441412 0.905476617287674 0.770599816334526 0.792946251040595 0.834533657314428 0.805351047460605 0.935852226538886 0.827019058088197 0.90731835423575 0.913741395989316 0.864479321031272 0.848510278489799 0.747161106330006 0.837751627821148 0.914506823053439 0.910094617347535 0.870842855181347 0.832513500248549 0.86980511564553 0.878133508415977 0.798975848117426 0.776187696493013 0.834699589721052 0.871117447966477 0.797263429056088 0.849531813518583 0.833888515847955 0.828897799065948 0.868363010719709 0.834702673935515 0.773805883004771 0.898119440232807 0.800597972618052 0.899491787884163 0.809183987237864 0.880016939309493 0.766875202463654 0.850485049135649 0.928077746018681 0.817549988023791 0.871342252828635 0.787625385424561 0.894830202740176 0.768498108261253 0.849955115496079 0.917323553287206 0.83301394293382 0.853702479707662 0.867180005208577 0.903247852671231 0.896934788048512 0.727531933566426 0.894054794046319 0.795449100380023 0.864366731626149 0.845147361816724 0.871818187015164 0.876279684155029 0.773127961167995 0.829495711784622 0.756824723047433 0.825657912988072 0.869489056929708 0.835751201164377 0.918311178400289 0.847550630327251 0.890681605046507 0.83523773046682 0.848681702570213 0.909941044722351 0.794379286116469 0.823327112968816 0.810364349159035 0.82175173335317 0.861341088131641 0.777560882572676 0.829720684163642 0.860250810632735 0.895726012608217 0.885092254817236 0.782620859238431 0.786257204507589 0.879003737062932 0.874876684023109 0.891777565025328 0.905976722453132 0.891530363198914 0.848498464968108 0.903278017935906 0.745014560991938 0.843594221460605 0.862042104739636 0.915228572072299 0.861936744404504 0.882089959054764 0.880251555332946 0.868817878720114 0.856265708270272 0.896190759668019 0.859863327319325 0.895418433432757 0.899815212588294 0.898469571490622 0.79170643743799 0.863248996094726 0.816650087997673 0.897526346394563 0.815820064598766 0.88998569492096 0.84043326210941 0.845925639828957 0.903378916770339 0.76017224599288 0.868896698413027 0.822024303195769 0.75422169397354 0.858299386388236 0.913615099268822 0.86757342471108 0.89182255286681 0.87311911598077 0.80127192587483 0.878110382777402 0.824014351299005 0.86139722057943 0.836767072773746 0.924673683848068 0.895089718278427 0.855057622428805 0.845043791899883 0.791588729694885 0.873680566663921 0.810633242976344 0.877408876748632 0.878272913645444 0.828900955408474 0.856016911635551 0.839762506690143 0.858233080260547 0.826609909483834 0.883711178725086 0.854689133471589 0.852517397893525 0.899181554601673 0.79079399575855 0.812763349196939 0.76302556671077 0.763825412123036 0.775873057283464 0.864788063256545 0.870725999182767 0.795965309570798 0.853740001508656 0.852729063252331 0.708854466285339 0.796497812750093 0.909076444030164 0.819436933174643 0.825098150815246 0.907044653467314 0.852669624887952 0.788030484533997 0.872673355392418 0.730481626692449 0.798209249819024 0.89883506583933 0.738987507137506 0.802466056961488 0.859963566673032 0.905113788723995 0.829610092927483 0.843948391958181 0.787313983237512 0.811713835719626 0.85624108136626 0.759610101419017 0.885912631025042 0.806470836449583 0.888811724994883 0.890200676663988 0.830762165505822 0.807080895098624 0.850099703391795 0.855712794617108 0.873056313058014 0.92524454546844 0.833271590404197 0.891373550266628 0.896643936994751 0.768753167230277 0.811655460499107 0.776897176417327 0.848580929665079 0.888636693351352 0.863885711359902 0.793106768213401 0.91727661022531 0.782266750685642 0.907114985032344 0.892507720333545 0.87414082630974 0.750359608984089 0.738757744531509 0.836510078240199 0.720637072998163 0.909036547933541 0.77723644106513 0.902802524939822 0.854333813621073 0.830573680939767 0.948038117015346 0.886619168266092 0.881739872957916 0.859417607075374 0.884276326445863 0.88562237593819 0.825808884584625 0.855604475908429 0.915376270689352 0.840486840194581 0.906658282970568 0.75781427418456 0.781154482670551 0.775852416060414 0.724184437829127 0.86717501843705 0.839873458386433 0.858726047417905 0.81622609386802 0.893828771001164 0.900945434786557 0.880564289525718 0.851825783825728 0.873450627748555 0.951510639826677 0.91187568500775 0.886351796519562 0.87595405315351 0.905987231708951 0.854125531743607 0.887145029225181 0.911320509923529 0.842256932962477 0.80528225709904 0.901838088301427 0.84916868056899 0.830443389140179 0.770156195715245 0.812503226490481 0.838519392217997 0.871137987932079 0.816874809158229 0.733161912990492 0.870569967913637 0.906402204671117 0.893364961201558 0.834537560965095 0.811011184592379 0.858896388378906 0.778462153127985 0.928683635355175 0.894361987867469 0.911419953590523 0.775787418972807 0.925066710309769 0.90688242822896 0.898250405272329 0.783050687463492 0.88058076266334 0.887315332133937 0.924975724044192 0.855384032820842 0.778957241095406 0.882248103607032 0.814477923041706 0.803092290009972 0.879703278897562 0.841036565570423 0.791656237964559 0.885768690905254 0.841853626292514 0.784440109583547 0.853177206262087 0.824941220823722 0.777313332922359 0.887705008692588 0.907375998450842 0.739121993178824 0.85342016613714 0.857924078725893 0.845480958944821 0.905819088873065 0.789161784675501 0.917581872839451 0.785624361487098 0.926453873261879 0.812143200248087 0.80468519491769 0.817159411753512 0.8903829765985 0.913432500697336 0.758287231559152 0.825542484516626 0.794210386892905 0.865501626389678 0.845889312624079 0.866056046132661 0.824601882799925 0.776850494217483 0.898913872760416 0.758655856264225 0.832218552849062 0.926039769589942 0.884477419289054 0.892090145338827 0.866989481084034 0.885887673856639 0.890206510363347 0.919976425437965 0.822816505901088 0.878868426373624 0.76766728344402 0.930831778470634 0.88619258283897 0.801897020103025 0.824194783130469 0.821588333791577 0.796900987057746 0.89715925607242 0.935443429706467 0.933023889102464 0.821954728219261 0.815695845865781 0.820357787446421 0.897055102871188 0.834215530977744 0.799258378563354 0.798300547450973 0.836454812279308 0.871472463255472 0.831378689366292 0.896338039862372 0.873474436212931 0.851131801129661 0.874866044883722 0.790424548742636 0.834354325299103 0.894335884289827 0.926183764491996 0.881534071964488 0.818341612017513 0.777187953678204 0.853163463021403 0.913065648468881 0.83236371848289 0.917359041396072 0.912505538297345 0.889510719575326 0.779740973241687 0.853602715534576 0.814842859300697 0.875722636842011 0.903724873090027 0.875360340733212 0.839947059268547 0.859731527737765 0.971212289139948 0.791479468748326 0.792615682656279 0.821548955687125 0.862975527906888 0.82682759230124 0.86865423884376 0.762521204090607 0.889130563868001 0.822731380709202 0.817041468104402 0.893468825020367 0.845630021778281 0.870601715959672 0.909232700498539 0.861224914527013 0.819506817052372 0.856132245144998 0.870801238316633 0.826848797270172 0.80976768832897 0.865039159177294 0.91447234958269 0.828013599938574 0.836970516289491 0.771007031807736 0.876707961571706 0.832696479090487 0.886529336208392 0.854632885098194 0.729064566010606 0.779922541513179 0.885695996126366 0.803887697413389 0.888988251904787 0.791534168841876 0.873163298470163 0.908568347134007 0.845890973740014 0.882905423803177 0.827222251096542 0.848961544555926 0.819406550512759 0.874516268828908 0.835245264466663 0.78558850773637 0.82615968146668 0.811889300834955 0.763742130965538 0.892214786484159 0.883472186529455 0.869504633033799 0.897097864521394 0.841672467172516 0.816019667552781 0.911956979893568 0.891681262044244 0.835466854220874 0.855937318715531 0.911953770373937 0.872793660455735 0.857309016429309 0.860404970686243 0.846760232488769 0.77647028369643 0.923074295958922 0.830442562589304 0.856188642401631 0.910295540606801 0.811143994397387 0.837410441509972 0.833133333315997 0.906685668411872 0.863388219491404 0.898648107103804 0.834116635612725 0.74431920289267 0.875782113002247 0.858805029943338 0.784065554150989 0.800267902112644 0.886274768907052 0.733134687844766 0.869934727880598 0.835959986548777 0.884324537951868 0.770582920934041 0.852470480477285 0.853543845103475 0.765524207033532 0.817730789057043 0.760262619256776 0.894472764586427 0.885665217362434 0.843234531875198 0.830201248168616 0.86138867603507 0.790640549884953 0.905003958485311 0.828070121294348 0.864529051359107 0.873361739852713 0.860943107354547 0.827534460440821 0.86624399081247 0.861555169758872 0.925539417755756 0.901062974859068 0.80218397825143 0.832281632551693 0.838275593019179 0.77417109128351 0.801974475929943 0.851663277793665 0.818770131769248 0.862744136371053 0.800254086076628 0.82736980884693 0.765215177978797 0.926465309301456 0.784608827410863 0.918087382190081 0.863347643037136 0.832830056281571 0.889345901339124 0.874131077430969 0.876502865098596 0.896174695726504 0.753987142150042 0.840348762176655 0.809864172651169 0.782191026303295 0.794883217603145 0.869827206921681 0.803795566712143 0.777666524539691 0.834520388744521 0.768137361514744 0.822443789382171 0.82499445216736 0.812136843159503 0.902373779062955 0.920935571892805 0.821580466928344 0.825499422992559 0.792519412696121 0.771293202336524 0.814252335515044 0.844016473107984 0.843819570966209 0.909709306812506 0.776140517125485 0.796523555647992 0.805281367527616 0.777165600008531 0.911395770689646 0.749122198300827 0.887736420406884 0.816613057362351 0.869591237196274 0.863458636902418 0.875930593140113 0.890487606023974 0.837789750269207 0.913612320498319 0.844038825469059 0.894036480384915 0.845492542743014 0.759284657679982 0.894828077124749 0.859430378364404 0.798158368039842 0.854902003357698 0.858719578066549 0.916167364980162 0.78575410690456 0.782626138521887 0.872125316491348 0.917323420609456 0.85915905736573 0.771019226392004 0.912498370983015 0.878884798194613 0.865941588561472 0.783584215964669 0.86073967543295 0.887492413355281 0.822754938329298 0.899505462001668 0.780202957575371 0.800290095337058 0.91301723395127 0.764484936913544 0.855983082145168 0.83163334957459 0.847629017200282 0.839370094562272 0.894950884674081 0.839355508668736 0.838138569691671 0.790054117854295 0.921256203385534 0.809823526676736 0.735967029914545 0.827065493223586 0.768024233108828 0.915148270267773 0.801766256180505 0.76068659260391 0.781727069221315 0.798038816974932 0.843700828638734 0.824133674152483 0.780285995823414 0.739871131371802 0.878562487155302 0.748803310355038 0.835604541467239 0.868459204281809 0.875932589545233 0.832234252936576 0.867238939741875 0.832287571110879 0.795831219540001 0.847857057356634 0.758340297729913 0.833754983692093 0.806902480009489 0.930761695761438 0.775893419396188 0.874665969744616 0.825236209305875 0.890059560158986 0.884535504090525 0.806059247157154 0.89183636484966 0.870161352465592 0.888109958999235 0.894016299924165 0.82824750163659 0.881435235864187 0.751943627897573 0.894504490128444 0.830562298446685 0.844815166301835 0.821902695410175 0.813041130023706 0.914160852163221 0.895868621535682 0.816522952584195 0.882496978054675 0.78498798498565 0.812820391147433 0.804877703172904 0.771591089567541 0.798851697416504 0.784933042992279 0.887177111140172 0.850832564134934 0.733206923024602 0.754932059352957 0.896476929489855 0.820304808489679 0.836714393618278 0.859942011178032 0.819709010584438 0.892262156757325 0.914394544120769 0.892115965113195 0.889790778146607 0.781982149840293 0.828316570931734 0.688575272392842 0.925022293079492 0.902534103980975 0.766701148619715 0.865100031749863 0.827528278886108 0.91748702142669 0.874348301611506 0.834723599946119 0.863256966602859 0.737780813567382 0.875512177080251 0.900664831467894 0.847998349259148 0.859159054576286 0.810886632829355 0.839933150615246 0.814881899076389 0.899155551687679 0.895543518165686 0.85284115200363 0.839908292422292 0.814825620116952 0.863368321970558 0.869631171769261 0.755496329164579 0.807728308796927 0.916114685728247 0.805063419185437 0.811721948526245 0.813597863454068 0.819941293600324 0.793436359842468 0.847862685757311 0.821915703053622 0.841295882265169 0.840651688583706 0.936725568873106 0.808439036367007 0.795232759540238 0.899869388161591 0.880399376977779 0.945517441274314 0.863956370976603 0.779594499323981 0.771447297795073 0.90259575809945 0.861469764025475 0.863489220608284 0.863053140955268 0.899918890057362 0.875082003670806 0.811686685309923 0.770157210726703 0.908343954991014 0.832166895977015 0.911997886117132

iDCs 0.377890518108556 0.353844352062729 0.234230343806893 0.262740356483312 0.217533090664929 0.334719869285613 0.606486352773185 0.375594772838398 0.530347001074313 0.586423291440716 0.221504097409262 0.268945360568799 0.312303273360608 0.188996148467104 0.281180636580298 0.329612552561549 0.565222374565711 0.282297788654821 0.293090975897233 0.396609814910743 0.480484934266423 0.450393396133659 0.286677370642115 0.405361466704494 0.299639830626428 0.291728207930854 0.444944998027204 0.294658630221291 0.306234656161346 0.421928265086303 0.3447217235569 0.342708284854489 0.309825240492571 0.555657680500633 0.389516795724852 0.237721406291772 0.24518230554517 0.317665068238288 0.416309540219261 0.213104428541254 0.380300733362234 0.336320165554768 0.371998656682896 0.38426405218059 0.340740832274447 0.326889253510712 0.422831536700444 0.275893680947862 0.313267398006728 0.179231147973685 0.47068075162596 0.274606078250947 0.302804617159846 0.594986609556024 0.476955851118031 0.439484659142093 0.373809454030317 0.311820181245952 0.31312624177598 0.314054681450498 0.459609437969986 0.322721814908941 0.239516809855424 0.290667149878044 0.70841391700898 0.258228904774375 0.227417784729257 0.485170702199172 0.228714465080784 0.49055351330299 0.539256012658069 0.349527501780214 0.382089158579073 0.224146160457714 0.295215978402243 0.20578317178725 0.304318891160384 0.267578354187783 0.265550161528378 0.592005773194081 0.196437098792017 0.420555889932819 0.440513400795528 0.377363158747747 0.553704490395389 0.2875639865314 0.29320851350174 0.220974280828595 0.394988298150373 0.629223305929476 0.35326722387584 0.238998137714813 0.286455757345834 0.324259109854239 0.281090819354354 0.257042355795174 0.334250448390621 0.361769809432188 0.385038431621388 0.415297962932829 0.223398075639525 0.367036379116433 0.320681378479036 0.356871876868195 0.242788434126967 0.385788149582669 0.240763617885737 0.216376052812797 0.404013574823778 0.446343476021322 0.369649821608258 0.238300539069785 0.469716457432594 0.4430306404925 0.344041075937252 0.357617601260114 0.414742167764979 0.44212165978084 0.240185098959672 0.30960161911012 0.298299112461454 0.36540800739333 0.287383336690502 0.295681566250298 0.387497828754342 0.448881502699681 0.396746478994359 0.263625089929573 0.315723416474226 0.303976605327788 0.251206881947647 0.341602654112834 0.424048289909425 0.535236623113574 0.421611388551351 0.422915545702087 0.21408192603702 0.367760156372626 0.211598284440634 0.389731426010115 0.321588149992802 0.377104319398525 0.448622524415273 0.310272517172775 0.362118086933962 0.206501333212711 0.490702406442472 0.510243510438656 0.458223000051068 0.313544823650208 0.506769294315648 0.239237524856633 0.297273338123405 0.420813699914235 0.31610102525665 0.520233015806098 0.381033708177079 0.319682997799714 0.524670286523739 0.322284392310538 0.520662596868013 0.307508670644882 0.168797858376019 0.22285945457043 0.329358421982013 0.365777071813162 0.2381503082542 0.21452080246369 0.362403443309242 0.241790987702716 0.272037606641018 0.439404024390442 0.279405199007609 0.419131025629926 0.417102895447035 0.549816072403588 0.208745587667276 0.389847138505984 0.226649533204093 0.307105240888927 0.459593047146234 0.37389951405516 0.356981387030819 0.368460296966118 0.556364315557388 0.210910046407901 0.338861234244431 0.22079474047223 0.428479084145353 0.372804451160115 0.293492589430732 0.480592067386917 0.366968329969783 0.577002430856053 0.554944613684965 0.343687541374585 0.255278683642205 0.235337509337811 0.271364782666652 0.411767844389703 0.394357961978586 0.45752789055053 0.681754731700262 0.344090557250306 0.235357458266296 0.297414334841521 0.195339907725341 0.247396636607007 0.319683457680508 0.553860570247398 0.373241284508992 0.516109213973699 0.37928811755953 0.242977948947575 0.386448623862557 0.374158997443337 0.215478351030971 0.199997982526594 0.241471804846956 0.216036921028552 0.295636336794387 0.207977553920603 0.412088963989264 0.595569850588019 0.412273419445968 0.544657323024064 0.448800382071012 0.465497105171944 0.405491715046224 0.254860280640295 0.346339173583078 0.229632115791095 0.40589738798053 0.352382321932977 0.395901881556649 0.594753854111239 0.373459302080314 0.187330412938604 0.485042990940607 0.226609853125614 0.41745313789985 0.285153521433178 0.424057424971449 0.299214836376833 0.364213598075159 0.451586128443621 0.259334905407521 0.670471872774624 0.214022079251565 0.816986711484461 0.530093216564067 0.301649881372525 0.475850135745823 0.379951733074185 0.291428370908617 0.507589955429693 0.470321429594346 0.479408006182886 0.344902770996739 0.426286338674899 0.319718731136144 0.338921090558704 0.220804714936472 0.199938135741139 0.565907379636276 0.25209460926523 0.309761353456646 0.347624215525658 0.192786444879258 0.671160555451144 0.415450799513898 0.433391591914914 0.26740832925542 0.260612801728335 0.430280281228915 0.441879272753476 0.484968003575209 0.45606491876708 0.519757461267005 0.422105834246695 0.532569598369818 0.414427389912837 0.211109535692751 0.502689601091959 0.617300809817003 0.464341861632196 0.363740730636201 0.276729549481359 0.334355507382283 0.403548021840637 0.330466528289214 0.313932497357643 0.308391411525861 0.279727591129918 0.489317928520608 0.447358315645957 0.308969450361253 0.444670692681729 0.21674510798977 0.297108801758581 0.410750172134573 0.436421645702374 0.328876739895343 0.231873606316828 0.314958603794732 0.38499578231285 0.383136124877488 0.207897758206663 0.484210220371453 0.289484608966396 0.475196367607975 0.195300009868371 0.329026398484183 0.440162000697969 0.289291813634501 0.35673650988479 0.271770260381817 0.221413157255266 0.298458989968327 0.299131915901374 0.416293860705763 0.301345292341947 0.462036002265787 0.391415941298814 0.390533779669296 0.217433346022504 0.44845267859463 0.351169291556089 0.320117823996604 0.483637994011674 0.553010883534331 0.532252588650242 0.448382116078369 0.537933676357233 0.423139807798874 0.237960793433592 0.248364159638531 0.321777730946845 0.568774048928132 0.2232983309971 0.202371905016311 0.356875108042211 0.43419750984425 0.340455789983026 0.502508098850722 0.456225577969704 0.288241331563022 0.205324346432095 0.527264620033377 0.367683390358034 0.422106948296325 0.220136425832224 0.392183818239428 0.343401777036053 0.402125146469034 0.233063331490519 0.571909283911325 0.420977231553831 0.290903343438846 0.374574412045119 0.198052961999304 0.300091731200195 0.460796865749451 0.469025978190127 0.602914077899342 0.188228114720431 0.359436604620044 0.275228343356787 0.431715415393277 0.250696119199286 0.410536192517028 0.542706810607013 0.542596818897569 0.301587121153124 0.357266974697811 0.408196957589693 0.402525128961762 0.47217711911968 0.521734555867257 0.384734090525661 0.257500768884671 0.425019618879292 0.343422070088769 0.224704730455294 0.318161323854009 0.28425409065214 0.314971825511051 0.391808253433334 0.22778683990623 0.403959441834532 0.214660444963085 0.36982324346105 0.840645380694054 0.408690097483358 0.183350601705842 0.605220623872172 0.371819064617489 0.388574290211509 0.24959101874036 0.22504386223954 0.489036941573587 0.21896941351585 0.610607524765915 0.432407644466987 0.309708339347164 0.445779477012134 0.252124532657958 0.341841332345384 0.486031936534599 0.398342778230403 0.21249598622246 0.209144566236977 0.233063331490519 0.14775173882432 0.20780798802848 0.375626901188123 0.368873246395366 0.410208281085414 0.484491129686375 0.450891255767388 0.533811772844092 0.262777408374594 0.363535429727097 0.229362805256547 0.322991586073909 0.269719487581748 0.291274152022966 0.292776012135364 0.358182420645304 0.203499019475715 0.446318673445912 0.360098768338045 0.595739948660715 0.50109180703892 0.441231152813506 0.313373329660245 0.359546679814632 0.40186088248289 0.437780433860623 0.201693641447821 0.475095269619 0.565636044294227 0.589596098327577 0.472174868983084 0.363257605176939 0.325230353056937 0.368673837723417 0.385149887342098 0.215608019066124 0.453715338918343 0.151552009700717 0.268079640800367 0.315634221407517 0.489024711997326 0.26766474794779 0.30124721368982 0.359897776855372 0.173575626748181 0.375644891782647 0.543355159719878 0.20327958126238 0.259076734234988 0.343332279853368 0.283493662093279 0.672110266878895 0.240683822171797 0.350543876883668 0.289118314175144 0.506268822878388 0.432360085924828 0.270253430042902 0.349549500622042 0.262062171594946 0.2476061003561 0.343679885475146 0.429097321175089 0.297390459887385 0.544955792547766 0.254629780561764 0.555826785933864 0.214869908712178 0.358507977121268 0.250309180165821 0.389218218329776 0.366524589761368 0.343422552843671 0.525376718667595 0.466182333369544 0.512761062649284 0.335701890061989 0.416457462953892 0.202950423942377 0.262545405003714 0.307648556293252 0.291617587713792 0.44235259844185 0.365124616652836 0.436691785282588 0.302278105181617 0.254053428108239 0.5037875811363 0.276558471730302 0.43513845688167 0.328419439014231 0.237561814863891 0.485385479660429 0.409859347265215 0.354141855998854 0.722478103017981 0.331027916189513 0.296131868895918 0.170842623545733 0.29523248333477 0.275895060012865 0.447183642551572 0.370681070518843 0.327219098897535 0.387191388730579 0.278522880945049 0.383026262906478 0.437212180375325 0.308589305437845 0.379350274593246 0.509021659591934 0.402417696700866 0.353258956325701 0.34583384473341 0.203379325904805 0.362667770797906 0.535145279838711 0.413076682646192 0.743546492630526 0.208765536595761 0.248543529295435 0.258433002384566 0.213244071040649 0.533913629162791 0.218839745480698 0.424181429388301 0.438992826851676 0.422414953720983 0.45305857488945 0.220854587257685 0.32499967171471 0.289180107871877 0.405516233457325 0.285947311571838 0.43680505527761 0.31711772482191 0.223078892783765 0.230320353823828 0.406275133092527 0.339517062659901 0.245391769294263 0.399456902037563 0.371729556490519 0.184727077771309 0.253141928010694 0.30962041989707 0.368156420079713 0.313139211850713 0.386246997578207 0.500542857157214 0.336284917698322 0.552757698473568 0.377600451215409 0.219508034584946 0.510062161882573 0.336926084776662 0.457164570207571 0.229283009542607 0.224934143132872 0.510873250445541 0.229302958471092 0.34934750244073 0.346330189010218 0.315460338015654 0.645919740729452 0.509124290945504 0.358627647062845 0.306574435151284 0.302335735752731 0.393950698829641 0.203329453583593 0.201933028589641 0.387983829187877 0.225890331498701 0.532722241691192 0.342341549902141 0.326276612280815 0.397820864326522 0.185345494554345 0.426422960786359 0.291771660684035 0.36369313179352 0.285057442473998 0.391899254870271 0.235756436835997 0.467439227501112 0.257959594239827 0.362350959746511 0.278305343752879 0.45561106835659 0.339044622019027 0.372711432693689 0.362630644841486 0.201883156268428 0.217363524772806 0.292675283069753 0.674959231386604 0.241382034668773 0.42300873521397 0.305485524946311 0.4099711834324 0.318055404054852 0.201244790556908 0.481933178590494 0.464982503915516 0.387144550392179 0.526131581104981 0.18556493276768 0.459862411798945 0.256674054617065 0.312651516776753 0.313946156281412 0.642744300425015 0.371284209584003 0.317026202509444 0.519382150344276 0.468393220198428 0.328433933411612 0.430835948699496 0.244484093048194 0.380819354644143 0.36045623862763 0.318455229167194 0.197294902716873 0.289007072819689 0.49649655326308 0.393405811506617 0.222041548502544 0.298330402222594 0.320754966291691 0.378614006756565 0.374558866104963 0.272736006961284 0.178203778156707 0.475041514531438 0.42594680409961 0.391610454691926 0.577633773719678 0.300423287499597 0.27969945357157 0.176418349057297 0.320382663871413 0.507978833071982 0.260420188547485 0.366644188136912 0.214889857640663 0.723905136231861 0.469144145831031 0.276611842373323 0.251408579112477 0.288157117649436 0.375348447221651 0.512218990795273 0.217722605485536 0.442990454645916 0.364451569342603 0.336055329610698 0.310753107155111 0.408069533591019 0.454306392637393 0.380865239437302 0.328177125166391 0.357086044989433 0.219657651548584 0.309669568845866 0.391981228242398 0.308609907257257 0.410643870113246 0.176687659591845 0.283226539212103 0.316915457830744 0.360069946573928 0.261974062911275 0.179121428867018 0.247925283211861 0.435890158613647 0.26703509082347 0.579440016954323 0.201224841628423 0.271225631682367 0.605029154979099 0.293473096936019 0.524493334066772 0.210620786944868 0.266386234347693 0.262844948452536 0.476818830112086 0.363498581636719 0.345595325794928 0.470668623928375 0.465259215233892 0.509050384445675 0.28016103119156 0.318742228730835 0.576959093215462 0.392340506764827 0.315604095386208

Inflammation-promoting 0.577392582112727 0.557880542917875 0.570948903031609 0.581667053669853 0.566757051400615 0.636324215422463 0.754829523879056 0.591099499135564 0.600900979304677 0.747622958535996 0.672398753618641 0.517344122013785 0.527441699673562 0.513093437549799 0.501331770578135 0.595457624127982 0.711627151839321 0.510223879446392 0.559550268742806 0.563857903834346 0.672310304829084 0.691016971554118 0.556433416257277 0.704891555024112 0.653334100260835 0.637202203283194 0.680589580151991 0.549063481641646 0.523129926816497 0.638016787966455 0.717499073537113 0.580585697441547 0.518933604283426 0.650737059662467 0.668255161233067 0.545724383301803 0.532142537875411 0.614554054136431 0.563682216454039 0.520048555480047 0.592905242325642 0.570123123016384 0.58092020597686 0.616145129475127 0.605830060360914 0.540535269256863 0.675836604696929 0.498922000403865 0.603870394011211 0.615437307933696 0.589743830921146 0.509500862648972 0.55829213097571 0.735692044128826 0.606782861382744 0.613760460112595 0.593622931905058 0.693264570161494 0.552504090837321 0.590228940385627 0.693945761872699 0.592518420987319 0.572655435254051 0.638828064479764 0.698585014131548 0.711344765699659 0.56303999559468 0.726809415953696 0.556025281462553 0.646308921835208 0.604163496506384 0.537829779438088 0.613171988551994 0.483931141279299 0.686652759076742 0.541394346033581 0.588610287077218 0.598902616071259 0.591375554285174 0.655821398715549 0.606554432848494 0.625391571352945 0.507717241448079 0.596119940739568 0.680638729870346 0.617101907997254 0.600141654336116 0.478594736657541 0.49757416501594 0.645141614735103 0.599503954304489 0.600303422973758 0.60512272162678 0.626666306035731 0.686822062176997 0.482914346496582 0.566175623070984 0.684200108780005 0.607407425070732 0.631377785616872 0.638891720594048 0.688925208135778 0.604448939223886 0.582127164267341 0.557330733277044 0.528716126376719 0.619622789801087 0.653337372896277 0.671090646490782 0.659183219259641 0.578922344304319 0.48024660098643 0.57828206645133 0.661940284873766 0.554034949902669 0.670520186789512 0.688982440622915 0.626781947654383 0.558064919304723 0.647795344025495 0.569871262880208 0.730913338303535 0.596036784812445 0.703465698850823 0.586146416848271 0.654994518882115 0.654567128420644 0.542879326276542 0.565959038069082 0.483982373652876 0.562378892732474 0.638408303600865 0.711977337696723 0.722805591671524 0.717026765807887 0.615198472650745 0.577240957166438 0.665374466050644 0.597929844294692 0.624999376394053 0.55159620265762 0.745010376711764 0.634816136544727 0.611685093919198 0.599461757345304 0.528252553386471 0.588111731978309 0.595527486622512 0.547756600774465 0.586236744988303 0.587828200714356 0.658462767287354 0.586457334249501 0.623315239246403 0.56727234285865 0.66500087434876 0.615793572493474 0.637219814424468 0.58343590802713 0.564269962265904 0.519897962338085 0.46038284688875 0.515580136138041 0.493114430799012 0.629327235122535 0.646804893609269 0.544806395700757 0.554937944441116 0.60710215645624 0.555856433626894 0.59461145851159 0.709233291592782 0.536060895960811 0.585759783782531 0.715475493236842 0.626154215588066 0.538071098193889 0.584790520233638 0.479962848236107 0.606942599611661 0.664109253614195 0.583518232089634 0.555279880632 0.58820195918794 0.690029463791157 0.51733298112008 0.633854699623336 0.537795265773407 0.625915525226069 0.574261903949852 0.570046117975297 0.685848449051037 0.552727431932032 0.685035761542958 0.691610133143751 0.66905783819452 0.614280052440316 0.54953607063465 0.661082164332841 0.581326831975158 0.66502533340876 0.597393651886462 0.629421685004841 0.705839151403492 0.489044963490145 0.634918708489333 0.523155118593346 0.577854102357391 0.616816401916945 0.789204606902713 0.569117698182735 0.697105949576744 0.594022791851979 0.596041738248158 0.722402677523287 0.585769022765677 0.508159268633578 0.491253712531735 0.620380202300536 0.522083590182935 0.642334265058604 0.543657859140693 0.646965926175048 0.647664854128227 0.554312075308427 0.752359167520719 0.546624656039003 0.629007119843896 0.704473979684361 0.6861005428949 0.58609945098287 0.590177916842034 0.566226041182933 0.675153686133442 0.643141910218473 0.68491317441471 0.558559587018921 0.544768371768924 0.534543679576454 0.513369573834548 0.732170605714947 0.575307165865481 0.5964142228574 0.602446343859548 0.636005206020217 0.736278866205132 0.651983511665837 0.624028556099232 0.578676981767079 0.809793963499947 0.665662082988302 0.642759558121571 0.627246396297754 0.715694680035237 0.557950080000718 0.652573592640913 0.80634140404697 0.616417711377256 0.559256076473444 0.647921575902353 0.606947699440249 0.610919765269449 0.547843531021319 0.579350605580419 0.657179583281532 0.544448848030598 0.578996790997728 0.450755142079549 0.716112949530117 0.699428898518051 0.652478900174866 0.597712693904664 0.560582001614067 0.645869616428304 0.594564726939815 0.678714499175782 0.661374463336546 0.706865446696593 0.599783256536418 0.720702653311089 0.690292469006679 0.577880663816573 0.504878461184432 0.703877989763605 0.627857436640019 0.755859255840237 0.592941789370138 0.565654206031866 0.655768484361702 0.538330546638174 0.520539048548243 0.594203943274299 0.599760426688466 0.543177601797106 0.62851230126955 0.649016671034298 0.579843884207677 0.593437428144868 0.546370092238488 0.566725120812901 0.681532979276704 0.666577295568274 0.485338332134965 0.618484364673144 0.67300118083018 0.563855113130181 0.670969040894771 0.557300096583309 0.625410797565162 0.507594258633098 0.717479878596268 0.513055090069244 0.586052654041959 0.525705930431397 0.618554656121998 0.567376575957994 0.560232443150959 0.541269944732037 0.588217037059436 0.62165410374412 0.599706132643249 0.686223151848775 0.54839361078327 0.568528825430692 0.69094033092532 0.555272520786771 0.570349046279341 0.59728322574049 0.645708377798021 0.674895523510505 0.63227398783011 0.678455206750232 0.632780233547829 0.766364075758144 0.5842179220833 0.597792585249869 0.53281653326359 0.727447833051038 0.697438396451848 0.578244634681798 0.529497089955346 0.578769008979765 0.502836389079288 0.736265655043385 0.63254646313157 0.766799546364277 0.580496327003094 0.539718974269923 0.600416113686695 0.62355718640061 0.657909785886829 0.563835740655492 0.540617249794843 0.557541996654258 0.673572995421764 0.568833103233828 0.66544927908395 0.689440307913447 0.68780457689859 0.566605100892906 0.562758404563919 0.558766479047807 0.667095444642093 0.789611867652932 0.622634323372039 0.597043572784964 0.530209654714038 0.647735328484443 0.691259490745536 0.540065895171429 0.652555610107226 0.745623831770711 0.689885342817405 0.541777938447538 0.573426694936742 0.571374330546635 0.624100669291671 0.681649405214297 0.668333337807388 0.614387249729882 0.652567442840111 0.779479366144132 0.510516422781282 0.555905272793074 0.541537781843344 0.599795488429976 0.61131175856226 0.61681274984575 0.4437919206713 0.733240977748848 0.579480238125034 0.555786182093819 0.657997749003624 0.489354393165315 0.64382813987637 0.748287659319293 0.565160716436199 0.620172383444107 0.582964276895256 0.6240553634746 0.593934729903212 0.499269438306895 0.623387301114811 0.676783811237668 0.543588080736005 0.61411764058012 0.568082585609212 0.635568947180263 0.618800331210904 0.637550276875896 0.611329592129637 0.50675736115547 0.501613336646696 0.650717492473432 0.50805002116419 0.674734095737587 0.479526132350706 0.633001035903622 0.678286523564586 0.622243152064388 0.671628047260374 0.581533266266376 0.594426115286242 0.626139562327671 0.667041224626142 0.572078333032489 0.571050412584899 0.540800997600587 0.5587735395675 0.538047454274916 0.65869170120043 0.524100945824751 0.631339407725523 0.672081708727338 0.550833354278521 0.523792415832603 0.722037418532887 0.703379315629103 0.550140837656416 0.549175873382063 0.660550160080934 0.582441024074905 0.664760947657996 0.645764572813319 0.523100467680078 0.60845735344396 0.727643653697377 0.563945728366061 0.572061447944423 0.553399220475133 0.592547204248475 0.557844618095892 0.608810391758467 0.684301951404175 0.613351134966844 0.673956924454309 0.576696328891139 0.530930122996127 0.625481376094126 0.592906239214967 0.536792805362964 0.581439497296912 0.602730668763112 0.499321006426188 0.645079815047972 0.623999984779386 0.641931484871747 0.527647690588952 0.612886710428051 0.659010498840527 0.498757129344657 0.552631088887856 0.549493944173349 0.662239971022763 0.619696154259822 0.60742005935068 0.587329218310309 0.648178237692869 0.511386138400103 0.69173816310348 0.511877678871453 0.634972252583109 0.657468597598907 0.580513612117562 0.550078864605271 0.665184128111866 0.660753900710797 0.732966478028317 0.659101168251874 0.548291030376726 0.585623619289405 0.59955553434935 0.506992053890761 0.530542705025551 0.611259089929961 0.554086230743392 0.635724116367824 0.5881512852041 0.564896056110548 0.529855966225752 0.815170906814367 0.527969505087839 0.739576012699169 0.622307092054383 0.556993958761375 0.582370483366327 0.651492288009039 0.586579901317271 0.700709152269235 0.562443235953031 0.5882664537067 0.507069266764853 0.54498954795428 0.566822576945084 0.533364455587424 0.536127354583556 0.550106524400677 0.627085442025007 0.575859658172066 0.587989603699209 0.58865059545215 0.641986608752171 0.692803792505638 0.714745900243012 0.533456749405624 0.584878836966895 0.519056600118704 0.495326640558128 0.518923189566819 0.581572362447713 0.585005793258373 0.681869841370561 0.506971712530316 0.547007031547855 0.585611154925121 0.530840832993868 0.714406340251976 0.526617335994728 0.683812611677647 0.518037070300398 0.649598980633599 0.599554860569129 0.653005085699415 0.624772918863958 0.599535179406715 0.639332065030305 0.599670165523803 0.669315061118912 0.605038791398681 0.565738307346954 0.703634622608551 0.558678508667515 0.542441217802927 0.51868284223734 0.640170724853732 0.671659108185977 0.553223845838618 0.594647146017081 0.615128616289059 0.725267808296412 0.642156412565476 0.561269838112846 0.655167494395591 0.651275378588167 0.645653850934929 0.544661842719875 0.655343793586841 0.614266119370328 0.634194959110424 0.704023334191084 0.570664232945319 0.485611114188636 0.637478095240337 0.500210717083515 0.633860519396048 0.64717764159139 0.521301622667378 0.606392354097414 0.667917193359198 0.54072998328899 0.558783072818095 0.538456896497561 0.713970500761643 0.577342945270713 0.580445684935146 0.498172468785474 0.493340173891732 0.790961001753517 0.536959964258625 0.564005410240454 0.603421589814267 0.568306891971416 0.591400751225493 0.558161402538745 0.536938062378298 0.497058796148169 0.669968654588047 0.592715524544175 0.568246490116237 0.605804119648048 0.64469199802947 0.522722621502868 0.577863428509688 0.551465236625868 0.530676711305206 0.588972619647732 0.550129717207673 0.635007766249726 0.590258604284106 0.810719591173693 0.54430666417971 0.643063368483283 0.556113890159265 0.648165112444192 0.670586341689543 0.578774353584969 0.711541309223052 0.604679489300517 0.597875755264616 0.715641229060471 0.585179451269967 0.592060289409277 0.550076119299568 0.528804657771995 0.563934836595688 0.589874432551415 0.639738134544145 0.557777680187334 0.731583037384139 0.675439901086361 0.558915033429256 0.618309599091887 0.534266161899482 0.561564048189602 0.557318764838805 0.534504326269029 0.532779229256927 0.541017766934223 0.631199318046789 0.602406056156804 0.466044276883504 0.528487437561299 0.633607790741458 0.554249765585991 0.60896722494037 0.674421950927144 0.620193332781634 0.694341980820826 0.700106671165984 0.623779256711171 0.642581729889173 0.560853922144223 0.562572825182867 0.4782497532909 0.723756498531997 0.700298573959138 0.518687287708304 0.597111489588301 0.592205691473765 0.791268447783372 0.617509632910029 0.509668007557353 0.507686359501391 0.502964611320852 0.570562737061842 0.70520893111518 0.582589874266575 0.59479488476501 0.573353491165963 0.572379227229742 0.489457633303464 0.629997523539493 0.674264663926929 0.571553017681285 0.519081763725759 0.561708683019827 0.63301902146192 0.733135909803062 0.515152311757758 0.505293517469643 0.713989001558545 0.525836362328336 0.529443861350215 0.504863379031168 0.592445567026393 0.448730397429913 0.544955746385095 0.584803919696243 0.629121611142439 0.600972211420023 0.77486252345923 0.573257518216539 0.524537729499493 0.728642434068256 0.664022275428248 0.646050591064313 0.565398697035572 0.582071776261749 0.487897027977865 0.624870589663555 0.636938262332724 0.675451772493473 0.613175348486036 0.647047686221734 0.709142142983603 0.535035573940988 0.528659576566407 0.668736392482348 0.574514543797296 0.706349607133629

Macrophages 0.728029717555884 0.723266211337006 0.69312357186518 0.721088206689318 0.73119230977285 0.789291279951463 0.73310392810736 0.7047969814021 0.722713610271537 0.758564072624259 0.721395795283916 0.683030194172015 0.675215766217426 0.67135644564912 0.669454668607046 0.727619696935838 0.772097171043074 0.687024079897531 0.673414367818998 0.66407949933064 0.676002193859345 0.726107237683455 0.674122616522492 0.767951456379613 0.684960207360585 0.750347330294514 0.739635585904156 0.660732768917596 0.753583949459699 0.710574957405492 0.801344148305212 0.774921336817179 0.69016942200551 0.79291970939686 0.805528998105766 0.67551676898751 0.715933693096889 0.708326293643793 0.685209692300133 0.672613873832101 0.717720178614053 0.673640404165567 0.648015552228015 0.772325989477358 0.700031884953 0.702824245149168 0.788070828280458 0.675682336274485 0.777317643735239 0.653768843246569 0.725722884662266 0.652852732684262 0.722098440263375 0.798640536832587 0.685147241761968 0.752225374972675 0.695464141645839 0.778846009654337 0.674798196403172 0.716291304956904 0.834240416333977 0.713162597684053 0.704557101089724 0.737848448277592 0.832597253524598 0.751317595749229 0.645912348069933 0.801217423397115 0.626855782203111 0.801332491799554 0.679155580313521 0.719819225403412 0.74801592733186 0.639001162802772 0.72862346766828 0.70032317764646 0.634982437765326 0.73459436608703 0.678350903967768 0.753724219764892 0.746510252131194 0.808023894262138 0.704934490771944 0.772355456361066 0.814021496786475 0.650857757698905 0.700614585036568 0.683581401477817 0.669765805594876 0.783547669926945 0.667016800913426 0.669588517906136 0.687701799194104 0.762670301571757 0.789242172097271 0.642804073930211 0.672233651650413 0.764959136032092 0.716160949045832 0.811424918892393 0.716143161728537 0.775458888383177 0.686909747679575 0.728230146474621 0.674536179741583 0.682045138713515 0.754740873596613 0.731573358023379 0.769769071432507 0.813437425726503 0.632741468975514 0.686111554640059 0.715227217691212 0.759565366111629 0.68869650308643 0.77026596651736 0.786341517199442 0.789484756692419 0.712959448279718 0.798760017334428 0.731344069828456 0.763093239702321 0.71759914401785 0.769771213693371 0.741836370798136 0.815895668105928 0.720703155033981 0.677283728572692 0.677154452593187 0.652092743294754 0.6776200499162 0.669037146644506 0.719191393673411 0.794344890958456 0.804768773422303 0.721819588650221 0.702043877946586 0.780661359607085 0.680629216323869 0.763140698318566 0.695309378400777 0.77160240009459 0.799163269407391 0.75560296951884 0.703380502766483 0.686021058704565 0.710110401791743 0.686613660730361 0.697872938472792 0.730989730530286 0.634736872215271 0.697520472978475 0.702385764275006 0.700395870315039 0.675960407893993 0.763553531647491 0.767525427501559 0.707934344633715 0.71466116333508 0.677512114924269 0.68041483161238 0.681153767317475 0.682017755137865 0.699698560738941 0.754033079784659 0.75360961596706 0.660734539075872 0.682880054508029 0.751457206697457 0.644942409545654 0.68192191183755 0.813957164422501 0.702521849638901 0.734402324959446 0.747543257334132 0.763918910658783 0.688110751794852 0.712821584239413 0.668228190311946 0.662651682704636 0.705821777683583 0.69577837827858 0.648883650335448 0.696967414558999 0.776696837232872 0.680691953025116 0.764027020520294 0.701537245892997 0.703846146390764 0.708403890444085 0.638962100509156 0.794536836841354 0.664446130382848 0.795195608096883 0.799399963025816 0.749331862200865 0.663049876923913 0.729011198677625 0.657199065903899 0.702012713264358 0.784462160432018 0.767866663919397 0.736893246236847 0.836772812109118 0.671228054048927 0.697388578817179 0.687953028446691 0.729392498849462 0.736653015130364 0.811339294426832 0.666952792950162 0.870417195218383 0.669711438683476 0.760538381682631 0.759412318983739 0.688967480329385 0.685130330203359 0.644005703472019 0.702522814518584 0.649646357097467 0.761572476971732 0.660328311009989 0.786242798120544 0.712585699091056 0.691395138266468 0.754006434987129 0.73518196116499 0.806599014310324 0.76096395697589 0.796433313977541 0.729867677721018 0.649822622871355 0.687899663010793 0.797128332443535 0.726326491353903 0.837520110455338 0.621078781061554 0.654188492511073 0.66146170490952 0.708555725855011 0.743034343011316 0.707467951162839 0.705886380971511 0.671906565739842 0.708758755013875 0.806817029059812 0.747069505577557 0.724157247459052 0.72982128117713 0.850912541881157 0.805476871103424 0.729239478194471 0.818486852372582 0.816665511005813 0.764511985351086 0.777456757893763 0.878177098662115 0.781754855600443 0.71671479272154 0.682922935021941 0.778446995387418 0.701145324827396 0.671012207964708 0.695068391573745 0.74150375720057 0.718016042913547 0.763561466369543 0.661656685812373 0.820269223476951 0.844263007268246 0.766982677721873 0.747240768154736 0.637947808023206 0.728404860308411 0.667937338726113 0.771505184468712 0.762405076514603 0.746945592651221 0.662368550744443 0.738785410701461 0.812081715006183 0.754378262944568 0.66783270217503 0.790196546509394 0.702925343878237 0.792920365790451 0.708570937615696 0.667992765790284 0.776632682351799 0.719329117809357 0.679567082329249 0.734599493031034 0.679921596062657 0.676491319604396 0.725634658753143 0.744693559356124 0.671730360549358 0.734182010641563 0.674885256336342 0.674788286341421 0.797107799061368 0.800045780086501 0.681872865281622 0.764531986374075 0.705381531348923 0.676928254867261 0.792361352164072 0.639999547545765 0.785422313115919 0.700797844464014 0.799006983931657 0.683224757551153 0.71708290742779 0.666596547642264 0.723828520606938 0.738490247423754 0.664650143386983 0.696031316685391 0.632051135446501 0.821966878484715 0.660669488921689 0.74918447244302 0.672767228512294 0.706725913804108 0.752195116494631 0.662602830130168 0.711545582339893 0.737232083737896 0.791041372313011 0.787847427361136 0.722032842090546 0.814841630445472 0.749816036498531 0.865060331016497 0.701995686618663 0.718850594642999 0.705637415075273 0.788390383752402 0.757615711646064 0.697188413425223 0.67263090636435 0.768117040962135 0.691517280241125 0.739437292009632 0.763034715254128 0.834762158929703 0.749538461717422 0.701184482475061 0.770082626913037 0.814219081332591 0.707292452154802 0.627061130681408 0.708406806227702 0.699699014327751 0.739814623091772 0.71214836568673 0.769358655425427 0.767203098591672 0.777381404638107 0.721646744890065 0.669067250071334 0.736781832955086 0.769762197772434 0.714870969413853 0.724064157715822 0.753671100199759 0.65625716431005 0.693190309568639 0.764472224847559 0.677138833086634 0.768917510069997 0.84687748447253 0.766416089980519 0.683275502753588 0.714743957115745 0.706952662366949 0.738784815672696 0.769797723781672 0.7786622375912 0.669295009471423 0.707410029408942 0.799049410801907 0.634330746341136 0.657906020824757 0.691329108741636 0.719273096674101 0.696886894709536 0.751215704713171 0.635305871340674 0.783739182811992 0.749826869222497 0.73438585556101 0.785058834891937 0.665604438110075 0.785007539927905 0.815033128432801 0.721423569990078 0.724091687325228 0.703632724955846 0.733534111691145 0.692655030334491 0.657644298917539 0.774690232302555 0.727945480674405 0.658702273826731 0.714701473669727 0.701319675518059 0.779747784782072 0.734254611496061 0.761198475345202 0.686061414778747 0.664595619470185 0.680258992244994 0.729889078326972 0.676481241354007 0.720222312458957 0.710291054381039 0.73061043596549 0.765388744129212 0.691330046711954 0.774663338744411 0.761786870619277 0.7334288253131 0.733960370427248 0.796314579626431 0.697793313403444 0.702318504452951 0.740095264037339 0.720800736218299 0.685374953341067 0.751732055443158 0.617714905766178 0.78429733269872 0.727826953040844 0.670140001501968 0.676180963462706 0.723058646256574 0.7607520891445 0.682919750297617 0.664630404742679 0.715456653547725 0.698145776551718 0.692166456434242 0.742581023564732 0.720903408594802 0.656593505275183 0.764839805796582 0.678990413984936 0.701038437976513 0.756047988273738 0.72576168455885 0.70141460845259 0.758251757765055 0.820474920724773 0.710939645372037 0.722397466695827 0.719669540811572 0.633611797022453 0.739909213085417 0.767908761570668 0.681029829963639 0.710105045957704 0.741277292941043 0.664743573584799 0.759976350933469 0.697404181792113 0.746077647105296 0.701392779296755 0.768749636835186 0.765538129350203 0.653277914940514 0.672983915528526 0.62046120165735 0.759086796332646 0.737807710094057 0.713790921218674 0.706977509368242 0.734985710307591 0.715439158021037 0.773214604278901 0.687305258152657 0.77234055948031 0.76631297203671 0.731105907001936 0.662240834069057 0.686558379821542 0.723968066090762 0.791153888173332 0.74674131820216 0.695257489840717 0.706810867446132 0.705776721457316 0.692880807448466 0.698246007929817 0.762718962984677 0.730487009571797 0.773685320740634 0.712162914467889 0.692447680679989 0.631450683406856 0.859906199094572 0.647517622449476 0.830808496391879 0.785554756679577 0.666083143181996 0.735167915794706 0.718305928245065 0.750446066662799 0.818437520356532 0.738355214698612 0.740502342864118 0.723899833391226 0.665177972178191 0.664479250628336 0.695224198421976 0.684068464296163 0.6783202171554 0.710607970118988 0.657873446539318 0.809404929150668 0.741194465408877 0.619672063102306 0.738712411542877 0.828172992434469 0.68599956692909 0.657803348114899 0.668499826688929 0.637955122622099 0.657388118993083 0.726271455825512 0.697883370702405 0.801486518382934 0.641312083559319 0.713405216871573 0.762996022784307 0.661172084916189 0.809100753955119 0.662191358256522 0.784155700833354 0.69414342909988 0.731015721451502 0.731293769743964 0.783464696228293 0.729322598702079 0.694847008915774 0.807705641668344 0.702458054421838 0.753268704335866 0.778991378555357 0.616910057763704 0.704940275093497 0.726177574792265 0.675492491364975 0.685190591197417 0.797752456770016 0.744531834661011 0.68143493390494 0.653024382824022 0.753798464773367 0.793910104924991 0.79242948012752 0.636530284000997 0.757669084274877 0.725486255622054 0.737176879206006 0.680126368035039 0.751938395274918 0.745939823787732 0.710958932277838 0.780420165270034 0.670848466970134 0.663376399750607 0.745702195439355 0.655220913316072 0.709792584794768 0.794813362759629 0.688118284907618 0.760383091127497 0.778152735042585 0.688836061811282 0.708969987124769 0.675736861821457 0.772145700439931 0.716356737989357 0.663684547114694 0.690042678953547 0.684239010737215 0.774735656124847 0.682391100931782 0.655133106694118 0.701980451797835 0.685339876446852 0.692579351912482 0.725604276286354 0.665009857610092 0.650170457929649 0.70568502364875 0.694359776143368 0.723904802791159 0.757343493336169 0.700345224513043 0.673286603442371 0.716814159320697 0.725893367790067 0.623693390345586 0.791535787449679 0.674803333634053 0.685161985764367 0.661210339994958 0.771656630794613 0.678094169985235 0.806598120857623 0.681806684629246 0.793645024336245 0.729811576340228 0.668844010879785 0.770350390396613 0.691190429602682 0.705179049372594 0.791366843034947 0.77170255431123 0.734614803100031 0.665410088868931 0.695870611329362 0.657546140676007 0.767983208886979 0.725068592844808 0.632122075837495 0.835021876376752 0.786317181468334 0.701176278280015 0.715082410225355 0.655077395757176 0.684698225128936 0.69156929885882 0.663854977222301 0.684947823104813 0.697788748801775 0.709280836421249 0.742480819838672 0.63156236355996 0.655152374019568 0.778228453023454 0.691705565305565 0.7055955986556 0.709773280649489 0.742333882949826 0.773661830171642 0.779173748605948 0.712494640890385 0.755234673956537 0.663755859445932 0.723586790329059 0.666589638841786 0.717319532058937 0.726812274273132 0.724067935614436 0.761931485813056 0.683786312775633 0.800811335450927 0.754410525075655 0.658624077487677 0.681506335561661 0.614517845896208 0.764032650697018 0.776157539755047 0.70000733003437 0.752326894250666 0.682575241455854 0.638749587193675 0.694451152661671 0.740677141778895 0.751641652841883 0.671276034167512 0.732277421669128 0.662357530054284 0.755325397920698 0.78188280809492 0.66122580747526 0.662718094408652 0.700488492250975 0.657249621401294 0.690260291427808 0.682634068798244 0.731265897612113 0.690361051122611 0.752851977899841 0.735561038092812 0.72365368357595 0.677270135650967 0.775129011600362 0.713179477856395 0.63801761064252 0.859762637378611 0.796382994182676 0.725088873227126 0.725462426272323 0.675770594744509 0.665855225197521 0.787591565078354 0.736123016079197 0.787981893030212 0.728570004364741 0.728637071625425 0.780081431623386 0.660991611298645 0.72065800013553 0.785814268678184 0.69433618632824 0.79364326490569

Mast_cells 0.357667948204122 0.273283025021555 0.295790414774051 0.261851226336213 0.380209842536991 0.358426066350823 0.361592291189582 0.502844212029688 0.493146328591006 0.399655519035179 0.260396071023953 0.271659837398903 0.362315613204393 0.254757058122734 0.316945052580485 0.311875451218847 0.377088973636892 0.370594376386552 0.564285545611837 0.234905070286387 0.470015084004432 0.319373300169662 0.320131054172391 0.358724446427592 0.265041187666159 0.328426702568829 0.334427508569452 0.220363448868059 0.270380312325024 0.277133608964204 0.362082442346397 0.253766148258221 0.265243863512795 0.383738069876961 0.321461751079499 0.387805739264213 0.246030184325195 0.338433731507689 0.55718073327027 0.171586053965961 0.261855560050758 0.292106905035379 0.402519659130784 0.323137215159005 0.352997697253341 0.341762958221024 0.402273059115858 0.27804668782827 0.410276657921308 0.310097416919221 0.312683313975867 0.385171318241169 0.334027386636831 0.397448251002855 0.177617386438407 0.636460495166724 0.440437817628736 0.407229763732207 0.393101264893595 0.176746267856389 0.272662930324275 0.399994581216382 0.194933361916078 0.343215908517781 0.592899673095618 0.292515637415556 0.332405319623519 0.552927790091291 0.230054117363197 0.244633028910116 0.504805887203049 0.474381755534844 0.397146621941405 0.227384057460917 0.329940120825481 0.304875934043239 0.18158064349858 0.230402427171375 0.348006065669069 0.486366836434347 0.300535373282315 0.392051066255994 0.366646393100084 0.199182558663479 0.489961699817596 0.501527918592868 0.251469844818505 0.179086983435552 0.361948277969976 0.450920237927161 0.454194507643401 0.291796687910073 0.251564817225508 0.254117858213626 0.30693520412188 0.335347543411649 0.29823441606757 0.246489185194561 0.245053095578431 0.312066680592389 0.368719234425557 0.353747478516999 0.392994561584996 0.310698170941703 0.314445494823305 0.350289228857046 0.371951710319921 0.259807090925717 0.361166146668822 0.354433303821321 0.298963236164917 0.294379352229308 0.452493014171089 0.376742939868083 0.265257175567158 0.327990840086563 0.440619962950099 0.410438822360223 0.195558439371877 0.311335178234324 0.442610928122556 0.295935634140382 0.238473660152009 0.515465712500994 0.209702479249377 0.416861839704381 0.551395245053304 0.216624879584344 0.279370715829365 0.23963649665878 0.205499830823152 0.600251441263864 0.358739676192688 0.372103296087754 0.268495716383167 0.488770178087701 0.172709863434366 0.403594524554127 0.214774943146225 0.284820518538356 0.320081437076156 0.40326215658224 0.229404554374089 0.370944561581912 0.326472057659564 0.271313886221671 0.451408297765223 0.280700960555855 0.493405058231435 0.208857980297831 0.474137005895784 0.244338527552728 0.559265478218886 0.500102014392939 0.223463038607612 0.503645845532766 0.344954863931596 0.287319360827623 0.412085853962422 0.439223218303043 0.34428142999936 0.280186025822716 0.352590812274133 0.350992810480312 0.400499526300118 0.297514677468166 0.383315960466879 0.243309761549592 0.203795441079021 0.242423899754978 0.225304965148687 0.521693571882744 0.240815069013449 0.309555115667403 0.309097115695492 0.191821274157418 0.167356806499064 0.234169164775351 0.386066951654196 0.323091704712781 0.478796635549896 0.263889576992331 0.310844603461911 0.289913087375874 0.530441493021269 0.269411178957612 0.610563368835304 0.178847592069501 0.392164715369073 0.342526675329141 0.353687886542855 0.399234029737013 0.27201907836753 0.284990579973423 0.392987977836573 0.408392188092173 0.408589642308458 0.281921360687633 0.346384186868194 0.659494610491297 0.276728586965951 0.517421523968003 0.401806032643649 0.335648817954781 0.314362771839772 0.256162511005235 0.15606551373367 0.312968856870989 0.192140462645486 0.522922477506199 0.371576171687703 0.658941028637461 0.355835821227372 0.268064204922487 0.583634952498576 0.279511616191116 0.331314704660715 0.228532900287216 0.264964700891268 0.243830918834999 0.34021313173166 0.370667743060308 0.527950712613966 0.403612143143047 0.573506027764226 0.456530883588403 0.408442047068755 0.369051471820718 0.535909142255017 0.286952894887984 0.238570545637063 0.232961371769028 0.338049002831397 0.40365907309994 0.347347891187635 0.550672239879028 0.484196692184087 0.353594140383403 0.39466905352193 0.183588871069339 0.273565117376739 0.211527816524401 0.356430720117784 0.223659561442503 0.406761053402182 0.417121422059175 0.217708879213658 0.379038808984524 0.396066193908258 0.726717297696913 0.56225963165535 0.573916693603519 0.393469194510485 0.409140798792826 0.414703202985181 0.437457411205515 0.37656791446252 0.348500524750173 0.313651344706935 0.244236017837915 0.355588873619865 0.207388362710886 0.178734546146644 0.259864820985704 0.170169655050161 0.205952014514581 0.241412212897374 0.365707204209691 0.464056998136201 0.392478308284413 0.253401507549904 0.281456501139717 0.467574717635489 0.212894364130053 0.205533079623992 0.483537466819854 0.328253365446659 0.279193990221922 0.530417283371752 0.350363180990491 0.392210591282098 0.231736249674562 0.170076558407808 0.293240213554451 0.428122497644735 0.497891621580032 0.173102199284282 0.346614414470732 0.323686141334765 0.291759919370252 0.382740246797257 0.419597062324205 0.240307699980324 0.336784811392988 0.268070927246089 0.428463334137616 0.309693766915449 0.717722754720886 0.361973649508192 0.43302190784394 0.310362139853778 0.525597721509043 0.298656495686316 0.295389687967115 0.188835531841952 0.179738659932023 0.371701266439372 0.331437671044288 0.254242339930558 0.343448373138528 0.280613642675747 0.155846071648124 0.29817249207119 0.42693069442481 0.232948531882396 0.422954399478001 0.364272364889886 0.268756688433007 0.255095972520689 0.164962892838557 0.252832942305403 0.179798507773536 0.183329530422784 0.373988181250283 0.235806034493738 0.404660637322256 0.297717682813119 0.210358124348431 0.480563794535704 0.379529352128016 0.335033732155009 0.298081167957807 0.350663128674606 0.689079295797343 0.589541303320551 0.26723075176111 0.202593885629703 0.262799001116606 0.362377779760158 0.372517680902408 0.252015529397936 0.44447850356851 0.343402359890161 0.304831623880416 0.274515415016038 0.473325478379942 0.171326713319406 0.316875323085693 0.319054597044419 0.358954280968189 0.199634742354908 0.306948808269598 0.27844021608933 0.267208587579688 0.296551821257639 0.264498448083186 0.348612742274307 0.34830734908484 0.367203580286268 0.32225118522171 0.390396807365531 0.247838853813296 0.258866182957152 0.292397074914171 0.442435015671257 0.149781490374838 0.238842873525991 0.227816391653138 0.4671693974813 0.325437715935824 0.26030603965029 0.775976671312763 0.498111988161184 0.300204802740707 0.46829333129227 0.360190060372758 0.209303493639292 0.202166825131754 0.379322978190312 0.270005609202305 0.258130911699236 0.296554111625127 0.19627661347003 0.277610152487159 0.212693430129525 0.312202276538137 0.221819355740993 0.456109666550897 0.407681203898462 0.286869431185315 0.539816699817822 0.295517727015937 0.30531438755653 0.535276362379991 0.496388553325975 0.4693881435904 0.245221725416471 0.248245268198413 0.343215564500591 0.390856422688148 0.45867066061095 0.261471058721885 0.357049374261552 0.384410440184839 0.184213948525139 0.31328536306502 0.206244603961977 0.375050713321463 0.427513066294666 0.33079232077345 0.214182813455949 0.373259220751392 0.378212696558324 0.274278756976696 0.211862606573031 0.228926935895286 0.399122889667137 0.253451013020574 0.229040581126318 0.496181247528353 0.350887703425379 0.204685236048105 0.289944386556376 0.379751444907429 0.445979633097973 0.221884839877293 0.258502145603067 0.245173881427688 0.48457586852043 0.28658375328228 0.219233352605344 0.535461692352276 0.41779915704825 0.3584208341763 0.408177941073555 0.278871070747983 0.371675039735126 0.289427266294036 0.343809374429918 0.250790872863317 0.222376234560487 0.612942480369309 0.631804920981054 0.451395439215912 0.294791511549016 0.381156475968008 0.393718804437053 0.421775326751323 0.173946718825628 0.457233428475156 0.309059134923624 0.506026145046311 0.187698422853211 0.466148085019861 0.300190250066435 0.280264461655187 0.18192507194907 0.293682155139416 0.314340524808634 0.681527885622348 0.346184066246771 0.212289235954758 0.467825066817725 0.166252946311164 0.313133071402943 0.271657595857845 0.316075452103836 0.343147986978706 0.338139606325783 0.580334946042466 0.31740342267721 0.337684071969964 0.238447305962727 0.379494785074889 0.255146579051435 0.268392516700693 0.403275893937975 0.338947664466192 0.494619369452888 0.504781686708209 0.243295791256313 0.18085581964026 0.280422970359513 0.285285117419956 0.327490647526185 0.289488951829857 0.433711373993059 0.773278766667342 0.24062711673758 0.251032593284693 0.43961357183377 0.269995628785431 0.264219335531466 0.246430374817645 0.371608177206626 0.312426662494257 0.456189933787893 0.230783706767181 0.280794830829801 0.248628821352932 0.409139146167273 0.233520170794736 0.163420148479563 0.397889923587718 0.193383967796916 0.340998989663297 0.482120141174265 0.271686096903537 0.512409632402104 0.445018836417345 0.43796523835782 0.302979688419487 0.24790512468571 0.280989432856116 0.555361785474926 0.399532150764887 0.424686210823489 0.35612763036715 0.214839821595335 0.390282238943421 0.266691575208374 0.284601358328223 0.291644238863483 0.4352904742832 0.29624041913395 0.481485297497796 0.175216823017731 0.163041112149983 0.280523521514047 0.35177798712786 0.264334198516875 0.429936496627123 0.294894609364858 0.260910898058613 0.392589235344026 0.358890438137139 0.268411945427228 0.246081636070326 0.491633784750933 0.29200860555466 0.490645916233993 0.338869933192135 0.347905490017068 0.252986644494706 0.272235632083512 0.381869879662187 0.391750059796534 0.407693262386926 0.291714738469331 0.356723968931357 0.480624536261504 0.348616857312027 0.308796120320537 0.375499576572493 0.243692367404141 0.335481876365587 0.146596255254329 0.537143278315164 0.240001169887279 0.49908999254302 0.344183010414589 0.429344714147782 0.276137392398836 0.412808541275137 0.484560694688237 0.557826583147177 0.177377995072356 0.378149094436443 0.300453695497214 0.465504562086569 0.231383457693619 0.327617660148143 0.331007509730808 0.35852195400561 0.27861583738263 0.350741623637592 0.175815301432858 0.44309189703728 0.163393549438891 0.224406433840895 0.404179299979137 0.365713122754998 0.332571575625091 0.33527917675041 0.286970323127711 0.354615219830368 0.321659335973003 0.332219653603931 0.383399804003746 0.27839492949982 0.275038847455369 0.188468403956307 0.303580292889312 0.481392449576193 0.172982503601257 0.17477128908647 0.3850160407989 0.325757680817186 0.392247005643661 0.211132177685513 0.293886608915207 0.343250651220523 0.187877966377749 0.309082374254517 0.284104661742365 0.352573659539264 0.38263033401689 0.328767718309992 0.353762879270877 0.501783073659185 0.348243196396223 0.312488264303813 0.33832809787362 0.398808855640356 0.374195826068326 0.229564243356051 0.709055511073177 0.355780556836795 0.360728254382626 0.387600086587815 0.453078055926935 0.238170102528913 0.266399716452539 0.36389482078748 0.322128656988734 0.398807552983835 0.554452240862044 0.220762961805098 0.465094890296711 0.281479927349993 0.42123583809608 0.301020262214771 0.358584309925217 0.584771979672686 0.366142157138361 0.224819334971405 0.304244242199638 0.412936652522502 0.566781965130964 0.429164169213156 0.359338262451428 0.249032839554341 0.357202139987672 0.447215809909252 0.631828466727016 0.210104112845951 0.27214596349353 0.400057546150436 0.305482648753717 0.156956581596192 0.240090479594544 0.357679507077004 0.341718500610455 0.295063312861379 0.440950543151011 0.282411114820722 0.239761615838388 0.320165051172288 0.36578619449951 0.434576174257281 0.362715333590121 0.227204648011754 0.30056474858717 0.288450895031598 0.374465495426693 0.41299731687748 0.176094591359917 0.318574263523271 0.325657459331187 0.38540965741045 0.323872386176382 0.335050620576022 0.187086644917748 0.362297301692531 0.393748996302212 0.212895083982498 0.316936228116528 0.545130511570212 0.312117382465751 0.287678085249682 0.38523372805443 0.238982903255173 0.435000918772251 0.256129676121009 0.635318126673128 0.287581529016277 0.425107799014008 0.273197779441116 0.220262298396282 0.361510572143443 0.526484588703918 0.292150772737178 0.319170649478437 0.374010396446295 0.501233158783557 0.288218385366219 0.242930142067242 0.352763093859733 0.452735769870115 0.434814360279263 0.397093461809874 0.366969456378103 0.293373427731853 0.250268785310061 0.327351214494095 0.329594874155956 0.509687243284064 0.407753557860439 0.498132192511521 0.443368155656316

MHC_class_I 0.944465516289541 0.928579984314912 0.949279891483655 0.935721891780569 0.948380573465703 0.976416133288267 0.985947093329785 0.975681373168403 0.96952869128986 0.989296673755304 0.982392158718895 0.97125913402804 0.972999624319303 0.923038929912584 0.957647783482132 0.956473709662299 0.985299866849918 0.922156477412507 0.970414397491299 0.920919779865174 0.967154976231871 0.981667157897513 0.962538515437614 0.972963530051175 0.977960838804948 0.986890669479015 0.975258460083674 0.962436124519765 0.967644517576555 0.97833455099597 0.988273962303366 0.959757485553859 0.964881659444962 0.994625508688093 0.957174212124782 0.967430078935041 0.950743930485489 0.977581868725957 0.967440740377538 0.958979352460992 0.981408406062887 0.960983483516045 0.980166204514106 0.984261146731754 0.971504393870316 0.964022322156443 0.990437711653072 0.966903889798017 0.988001591642778 0.972768855349323 0.970957675967838 0.964942567466381 0.963990923765267 0.991159626842177 0.973562796877946 0.964820959561685 0.970786594632883 0.987598987403154 0.969021601948231 0.959057117937845 0.987544308092419 0.95196406051164 0.955865153243703 0.942199278253288 0.985240188477096 0.996143174039245 0.958831022957821 0.983563256219665 0.965000092435385 0.984747514821126 0.977317831001596 0.976269635284067 0.962458581029703 0.961550403947784 0.982556729989709 0.949347623346408 0.955919273951316 0.9827921066342 0.957892775667223 0.978551110237801 0.985885527196326 0.962647271525999 0.961961886050303 0.966476195537481 0.988190470877468 0.962549862286355 0.942014646382614 0.964080766879412 0.956488919312459 0.983102032750067 0.959859289415869 0.972461867353894 0.951284774514615 0.976471601706412 0.987715522638909 0.928761945973206 0.952880277853998 0.99585891714491 0.968326603563237 0.986691535822189 0.985368120460448 0.965335994241186 0.974418863675187 0.982976501928748 0.950577962027034 0.939840434801205 0.987405317798755 0.972395601661963 0.977029660334674 0.985454555201245 0.986486027976899 0.978102556209915 0.971363356896524 1 0.966351541246563 0.978302680585529 0.978181342212203 0.985061427900772 0.953736357674598 0.987863696234135 0.956721853535771 0.981971369574789 0.974349739028702 0.991062550212785 0.964880154511939 0.997203982867989 0.966606848542432 0.954477137241182 0.974208484521901 0.957548064891228 0.959809181737806 0.986979645784514 0.985326831533885 0.987522238149865 0.977062113428174 0.988191963412131 0.957243674545749 0.992034845113709 0.970899897980033 0.966317234708597 0.97157996274004 0.992715439188004 0.976836398819879 0.989666228485194 0.976014899385674 0.963416993234886 0.946337978413864 0.971426399228489 0.977311956238923 0.974769883561219 0.951364662574988 0.992490448224236 0.957698732785298 0.975352035390992 0.962563824715598 0.984652712370903 0.980322151560639 0.985514231148961 0.984534946617722 0.965983929839952 0.940224897528313 0.968782374900223 0.946057988831149 0.925811175557459 0.956221090272932 0.982033110472219 0.940369124290775 0.976604565467412 0.981483702282963 0.970206187117051 0.967734125530945 0.988230159479009 0.96169383251973 0.977116497568221 0.989830691023273 0.992210378539695 0.962497698928202 0.964946014682263 0.95351739717799 0.96542523604615 0.989405105043728 0.966508154348581 0.952979537048706 0.966699805889303 0.970788522610768 0.9699461954671 0.980981223807083 0.957543685619727 0.954879970974235 0.966065138504496 0.967555888693594 0.980347502347164 0.956439672058731 0.991192871183669 0.979819050964995 0.98286717247049 0.969409079570843 0.935619377877947 0.984718167140204 0.979079480991675 0.982734708943495 0.96666913590118 0.972010802328982 0.981496192890382 0.925278904006969 0.973732132340089 0.952222951066225 0.967757451835797 0.98875493464747 0.964252048378277 0.970657353621116 0.984952462286865 0.966058161776808 0.97256316947701 0.992575931399077 0.970310063923894 0.925640400450538 0.962827843728336 0.955239626249475 0.968846476466884 0.963639988122614 0.951036797786165 0.983787655596753 0.97243153258509 0.969887071149075 0.976341514574534 0.972908233442894 0.975336854940138 0.991947638415992 0.978856791594485 0.967748968692055 0.979559712072361 0.978479528280094 0.982013083190178 0.974337343821908 0.978762968361465 0.960109618310051 0.96698868561419 0.967408092726685 0.964600318803293 0.987305531860208 0.967589971690924 0.954903914440467 0.977743522881809 0.972440186405587 0.983745674121213 0.994194915875656 0.986303388458261 0.982251266341202 0.987672975349238 0.958649577594114 0.974609600978874 0.983717142067691 0.985225104684213 0.950264611746716 0.987000774061007 0.982101831830537 0.973463967074845 0.97426430959545 0.986467800193824 0.981244924398252 0.969353294057895 0.931060709627346 0.946561065164224 0.989893626533113 0.969503125271009 0.986495838317518 0.952250839848492 0.985330368545274 0.980386180339651 0.98129754247523 0.978619601619527 0.944317941180557 0.978400326578148 0.979731522725923 0.966712084945847 0.96643658202371 0.982030372828312 0.951876051053439 0.986054143385869 0.9828644996687 0.976734585446895 0.947886542125086 0.996050265171794 0.970519564778849 0.993277064862169 0.961509676791792 0.9717219811177 0.979347329811183 0.953352442960957 0.950759898039048 0.982101681583005 0.9559250294015 0.965341754685974 0.981635648158216 0.973446418070325 0.959435355658671 0.98680761992636 0.952686149497737 0.959036564472102 0.975545507110144 0.976765008681669 0.953963364004768 0.973477189895894 0.967898808550627 0.968979031971351 0.989428049581005 0.968374989837903 0.983487731414596 0.952957751438558 0.993263547841166 0.968243774038056 0.96797000040272 0.946362941559318 0.994565853036177 0.959755338917738 0.962408440670691 0.952028605741996 0.970368148296342 0.979731155710568 0.96044648615928 0.993711136049426 0.957820213495983 0.955285584132319 0.982395061041554 0.947922092546278 0.964319369768886 0.983654536308018 0.976489984720564 0.985572206887553 0.976231570621321 0.977358131961372 0.977086853509405 0.985848356900153 0.961578682537879 0.966821594396314 0.956311369938616 0.996474457093975 0.992992942067412 0.964115123703865 0.959188636143296 0.97868790618724 0.966797606286521 0.990535696111464 0.989604230588542 0.986803473619134 0.978338852504553 0.951149531497932 0.96950635123432 0.977999369702887 0.971497479092783 0.980043127467223 0.964878851685601 0.964129060642221 0.994487745005099 0.963848701077278 0.98683785960105 0.969554061162284 0.998066526027157 0.972642672540911 0.96760806407495 0.954407333401531 0.993613822955121 0.987809931358276 0.942058781269795 0.983070844651876 0.958931752108657 0.975714436746165 0.968189203293849 0.955337300084907 0.977509146067454 0.983847576918191 0.990685385250194 0.964658350743991 0.981125553129568 0.965923480957658 0.968315941766505 0.993420675125795 0.966411844612572 0.95461449728306 0.966918447224737 0.998059734334579 0.914533735287999 0.959641161008568 0.96022268106112 0.965965087202765 0.970047661190693 0.967428955736019 0.962532800702922 0.989333436746053 0.967605150287789 0.976045638004061 0.980384159861753 0.966164178874103 0.987982961481476 0.980837130267346 0.951038911843123 0.959026678254566 0.968362279072175 0.995605138694121 0.961380016898713 0.93592244997423 0.977495397696067 0.991366313772109 0.968997240749386 0.98182521042145 0.960814282646537 0.9691571277075 0.955254429331889 0.982969203787249 0.95288427758464 0.941080840500014 0.942330557599076 0.969014948142225 0.952600134352768 0.981928767390828 0.979146404500117 0.963505681987591 0.98681951103555 0.974093471021658 0.993766193509027 0.970748318951235 0.975958306412186 0.964745374713156 0.983062866731462 0.950599681844433 0.937948743340875 0.974682545308754 0.971546136442362 0.940018395598866 0.998339006688398 0.967609776738013 0.966298992378096 0.98917898016654 0.97768519258683 0.957466929508923 0.98947462838456 0.993938089569111 0.978090503009672 0.945121862316098 0.966585067537563 0.968668917404432 0.976195499853295 0.987547760929466 0.960206611551946 0.942813359509391 0.990432744584967 0.976914015403187 0.954296344162331 0.932269661358827 0.957529377739464 0.976819826233634 0.981447891490448 0.989872898156809 0.983627540895047 0.987794993609399 0.956379107054637 0.954690938685554 0.980920561405157 0.962669527590469 0.957966313598256 0.963342611553316 0.965383057849962 0.964481925053333 0.984431219530671 0.972642906462939 0.970901544073619 0.959045337933621 0.964867230076474 0.945753723287013 0.958247859377136 0.944233389063727 0.956263983350159 0.995394297393789 0.984636392150333 0.962640828711727 0.970674336644293 0.991069598128463 0.957342653586266 0.976357360875246 0.959946983363717 0.980303757502445 0.964775211739388 0.96679336983474 0.958886487731706 0.991200525172017 0.988989369582449 0.986171978347382 0.987635515962248 0.968505343564685 0.95696645294263 0.967666389516499 0.970601006502277 0.955587658887699 0.985936626631095 0.97327513198093 0.960886483643792 0.968674477228616 0.977975200128812 0.950899654727178 0.988376700123317 0.952437019173572 0.982619984669751 0.978213725462438 0.953445169703483 0.966987244636459 0.975118226763459 0.971954207192494 0.989231473876575 0.941667431481174 0.96947968761594 0.959381114615501 0.951780968929341 0.955825050339763 0.977242757886226 0.956092888652895 0.951126585617467 0.976933977016675 0.958210026594892 0.975496766378366 0.956569378430995 0.948487368218714 0.992409605262781 0.985448274102998 0.949077778081672 0.980450408916293 0.954822078719949 0.948706148073855 0.970821265675219 0.966232429425745 0.950826581568606 0.986176002110379 0.961068583381786 0.975274203464782 0.973686028859526 0.925891729684111 0.991235162901458 0.964984705865292 0.996347731618321 0.953816003108662 0.983029182067058 0.976848907186761 0.980396950013267 0.986047997641295 0.942565321762261 0.966780204882851 0.956211030910095 0.974712218580818 0.967668603407135 0.961774319691057 0.993998526983511 0.96948149808092 0.967932326950713 0.954079116265852 0.985175529541567 0.977251282167054 0.969505185067587 0.95168461484413 0.967688766098614 0.977041685633751 0.983659187386666 0.946115985498647 0.970631018685932 0.979608824578186 0.976937524665248 0.966003291131446 0.990504896258578 0.972677126420517 0.994806259615691 0.971478335808968 0.971403751868669 0.957888040991855 0.981717089342008 0.958222198535291 0.97768110002672 0.974553751844644 0.95896925695908 0.981114839399521 0.970206356163478 0.967034655936309 0.948086273968014 0.942311547654647 0.963437967790722 0.946829598454269 0.969209239978186 0.970659705076202 0.960085982676585 0.993791939902172 0.963018028096501 0.963087322185738 0.965226500145452 0.981380806410752 0.990077127368099 0.960899939453346 0.961644014957105 0.957389290435179 0.976357464133367 0.954029214503923 0.962981084103799 0.968638035807488 0.96160550687987 0.960422087758734 0.966677265077621 0.960845101625006 0.955421136301884 0.968513129536587 0.952914408765737 0.977036609138891 0.962407247149015 0.991295698214785 0.956964792715225 0.989008850672498 0.940228202188451 0.985351284763536 0.994626303779652 0.969307047644538 0.973995089450448 0.977672727620176 0.983209778449484 0.980205239579405 0.983601377606252 0.972632020448948 0.964053144241369 0.97441540276147 0.961849864984411 0.96733551488429 0.969883083511758 0.955889268160152 0.98754838392809 0.997568912775302 0.952696949065239 0.978660787363931 0.964729740429965 0.948021602715581 0.96120328925426 0.948972742870117 0.947403303723868 0.946977415120868 0.983188371892454 0.957112205891475 0.932503498893759 0.952497580096769 0.995633086062256 0.953924697181453 0.964944363131838 0.996673160493955 0.983112349801726 0.973848062616208 0.976939736695133 0.982712761410309 0.9776172707213 0.936232464213364 0.951518508116014 0.94345437251111 0.975271753518498 0.983245309847273 0.947406146807432 0.964479636907216 0.943005598377138 0.996268624906162 0.975411059760228 0.946937763438974 0.959071242961458 0.977369120258684 0.980921707827096 0.984105675454617 0.971418787797324 0.969806382332814 0.976703800762349 0.967644391332193 0.966256658922845 0.980451502859796 0.998059801431196 0.960070726424208 0.953963437936139 0.986967942095101 0.982363487718248 0.991982389864174 0.958718925531066 0.939759631325254 0.975018136064415 0.974994902899398 0.981855409484622 0.970068117030852 0.969639551297351 0.947510002487328 0.961460320072065 0.965610045358756 0.977721714244521 0.979821319139333 0.981281756084337 0.958120682060256 0.97323672087814 0.971057458221218 0.985740958071679 0.995711968133323 0.961762603073505 0.95697825483695 0.970504224488523 0.973098093674771 0.960768646195063 0.966323152142409 0.967507664970862 0.965577712550583 0.986107193792165 0.951422059337735 0.965644102306478 0.966626255501228 0.964147176456707 0.998524508691498

Neutrophils 0.729583485047973 0.729569703192113 0.67812712704859 0.746037777026457 0.729714142084488 0.731934334978239 0.771259835429442 0.742692459135868 0.757780770478102 0.772957223436751 0.758027453731603 0.752401264343868 0.708612666996373 0.677761857561304 0.660967425093408 0.717554618252543 0.75432747825298 0.676113152114121 0.689696768009223 0.654768441538729 0.665350969620559 0.795207381654715 0.73956487692823 0.748093043050909 0.757866692537519 0.698803495942003 0.818348215821398 0.72192573212218 0.758628918595347 0.763811112909973 0.748105885441748 0.772813418128778 0.727147183310916 0.779229464394844 0.765074349128408 0.734933600826392 0.768594577004724 0.734617877834162 0.746501414311499 0.759812799077156 0.717485591648113 0.721366992012043 0.738310520203595 0.739037147764934 0.702183257052154 0.76458101105467 0.754155615710704 0.732454594742479 0.761605869834556 0.749656171017044 0.740518859351159 0.708133934430031 0.763167250668132 0.774672837035914 0.730524602651337 0.756155305640851 0.734667737330089 0.756806477893956 0.739697784770557 0.745660007479246 0.793140065720286 0.760055277525118 0.764790141971977 0.745653367898922 0.771493011707579 0.751195736228644 0.734409389561974 0.780991441881414 0.720931478018882 0.767227592427947 0.75336190170797 0.755230858538393 0.741511318432114 0.7160505613747 0.646868398576631 0.777191126181238 0.736627156683059 0.784250011483933 0.688985106574004 0.748798791626143 0.716421669847187 0.759518368626352 0.747171694141815 0.772843151089527 0.776853650584646 0.70741855479448 0.698865249012013 0.755150297777899 0.740473897201822 0.753284836935852 0.739258809540984 0.726472267239961 0.682206202184731 0.760297856435306 0.735333946262059 0.691233585991628 0.731898351392856 0.753239815401966 0.748669376498019 0.75630323304034 0.744216687270594 0.78420291032457 0.718698743039474 0.726899444463468 0.753364672479876 0.761782288510311 0.688575716997991 0.796810159345826 0.734034793382448 0.756495486830305 0.746075928144452 0.727087048330485 0.741498399404123 0.763367491894891 0.734033297288287 0.77261185841504 0.765928461191763 0.767758831370664 0.762279021441383 0.783871000898743 0.776676348884283 0.798042319566912 0.746192649059358 0.739096222355043 0.756447930529474 0.791903778058525 0.707051556961148 0.717230696847478 0.777535934414788 0.705090274888455 0.729475410185604 0.769691960766089 0.810339815646197 0.732038940700871 0.781388997291853 0.766317025451067 0.732446670020491 0.73420884820692 0.738393051481533 0.731787271514437 0.696179349192751 0.765295544899161 0.756318314058595 0.749904775310371 0.764312866577316 0.707580062106857 0.737356310397902 0.741234634937039 0.74008764956521 0.78824251380167 0.735985635121057 0.654236727375953 0.723325994922338 0.762175199469658 0.749948593309987 0.799405578728448 0.768891141038473 0.775934917243581 0.759729219798848 0.724663322008081 0.696623209805679 0.730116286993681 0.75070363314905 0.739816996034997 0.752254427528228 0.697946398350374 0.741410486423902 0.7613000734158 0.70568363602367 0.747709115935714 0.750357691767004 0.784064037369884 0.71380834166072 0.752830425037335 0.737231016184903 0.711312902300551 0.765106637052339 0.747828375962692 0.738015676586945 0.73821987331292 0.788929713274906 0.717136012172992 0.713343333760639 0.768121831833985 0.756890520845416 0.741824738151769 0.747953998105556 0.737457767448843 0.712677881630967 0.740348199430552 0.695131440780098 0.783730459610008 0.718112343124976 0.770601695185687 0.742312217960463 0.801291981502799 0.752578902731214 0.746722516070593 0.724733772083793 0.761435212732779 0.813307783955493 0.740239833777058 0.785787045285917 0.805768442817612 0.694195717249108 0.709876496928074 0.720485508118364 0.783936232860954 0.76274591137097 0.792131288992095 0.725880915795119 0.766342999186844 0.760159095787211 0.791563822574555 0.788592163141211 0.75837246590307 0.725267964206097 0.707770279203629 0.778139817042196 0.723349658274469 0.750217642333534 0.749307258752324 0.761020342383917 0.748727461625027 0.665716330970036 0.801056626329699 0.742725205687217 0.787222440744597 0.679630111727186 0.772619215980543 0.723695688350715 0.71127619597139 0.689147713021112 0.780054578950185 0.808173378710612 0.751168039279804 0.711684319559989 0.702114243612391 0.719220202082477 0.759087919772548 0.714205073657778 0.725537121947966 0.768672775259422 0.703063279078064 0.741267303855084 0.79295077483847 0.750822500826785 0.691358471078497 0.776762949868734 0.810700457194003 0.767943567454175 0.754142498568636 0.801862785947005 0.777833859153665 0.802049721306282 0.774656267647602 0.828787491483034 0.757739924578645 0.723034841749013 0.749937866859085 0.70442622929436 0.784958327216252 0.705749391012702 0.765868516676907 0.718855048835321 0.768236340139917 0.714282295287475 0.720144724820826 0.759114846983255 0.837393003954332 0.752591566014498 0.769806605835638 0.749343078570057 0.742898451681586 0.731988680600682 0.741150990869563 0.811950090391115 0.735479935577364 0.733418252929485 0.763454188641776 0.786122728399538 0.777186271728229 0.762693590354096 0.735611877242183 0.778914415634757 0.776920895264227 0.768023803027627 0.736263235915804 0.749417597799081 0.782084420773925 0.729410818768282 0.727219849936159 0.766508637289181 0.712615019514387 0.726049938130483 0.736707347949479 0.752707545435015 0.744085342391416 0.76751460822507 0.750244969735018 0.743111522764522 0.800057978994321 0.72408251173852 0.762534101339289 0.722878766620492 0.710954864765006 0.779850845201615 0.703280081902298 0.794720704275353 0.761207881273632 0.740222533003522 0.742703147681595 0.7735389800723 0.739374494122409 0.73963626085913 0.77120891247727 0.715437495206251 0.78497813702289 0.73003483291596 0.75521288814543 0.711129810350583 0.75811282570251 0.724895495765251 0.755698518119609 0.771105272282936 0.72533090736776 0.771382308381332 0.776771426286532 0.723257291022583 0.754437995513058 0.747073390745467 0.793207449759758 0.744630195929308 0.842387734993565 0.761546602355058 0.738752719545689 0.757643643541545 0.818109580080501 0.743484061754644 0.68589931443817 0.728963478743313 0.710765330061468 0.760732750100676 0.765765002983088 0.787400721142789 0.888866411057862 0.701126265837325 0.779963747003575 0.717191020414789 0.761748404530444 0.777209535722653 0.68613812012386 0.719858814298392 0.795875754405975 0.662563683357062 0.748144311581449 0.717450987521468 0.761710330928338 0.74746563930939 0.77000556119611 0.721593726826447 0.666830006535282 0.73868779784681 0.776962650441277 0.795667747562694 0.707437438671494 0.721641630551133 0.696768988451902 0.780517979724761 0.757612841463981 0.798147723378152 0.799923212787757 0.733368359403873 0.733880181329485 0.702482659721358 0.731427282667216 0.728930025964486 0.768141230767656 0.718962155239295 0.734247904341848 0.756304272067626 0.812263947493001 0.652363234945006 0.720653823366135 0.760204178777457 0.725848258848695 0.738763163292547 0.753869559615748 0.676724864387377 0.807970107532316 0.723029128013908 0.77679761071035 0.741626335995463 0.719030597273182 0.781425791923556 0.792760146688914 0.734964026538689 0.777192009806907 0.789648496932616 0.765024140019208 0.753552908800355 0.685566413312072 0.768461673628109 0.780319259576873 0.699998391976889 0.744844080298368 0.751711590669113 0.762160055929155 0.696812529439595 0.731525092589688 0.717281566414956 0.739468216200257 0.666137369269651 0.736163539257851 0.708452213652395 0.710664254886382 0.758159466503145 0.748946218036565 0.775676185408914 0.736351178504935 0.72695828347393 0.795561192752739 0.769624307872269 0.783835250596786 0.735075947217365 0.734767661455343 0.737591710178182 0.725863969701239 0.769258179266516 0.74065376566474 0.729931549297968 0.678877352446822 0.729972811087704 0.761489446508361 0.735782981927476 0.754625417807008 0.784478019092943 0.755316991037908 0.743849634479991 0.705237139677483 0.738203982552257 0.759809854833116 0.719095628020976 0.726402618882071 0.729659269252597 0.742760986955271 0.733129868707125 0.760573372378206 0.746823083062301 0.712661746977796 0.653610806659883 0.71702230693296 0.718401103138595 0.777923560709377 0.724170887288903 0.732306144007705 0.76387660415816 0.68045151228991 0.801727541283228 0.707768591785489 0.750742636875948 0.709520143476189 0.789258774078011 0.726415485581243 0.731379418148586 0.768323219986658 0.755753515304328 0.760659460377395 0.668796728982525 0.769051764951476 0.735278257902275 0.753514259999939 0.707781518593318 0.749287217037855 0.707751465508503 0.732337244843552 0.744094253790393 0.774768014452651 0.732177912327408 0.767043639041835 0.741316379528253 0.726105610118813 0.782029469673677 0.766929781535158 0.755996380267967 0.778002525157261 0.714759466238656 0.802571951323113 0.772316179781982 0.73983882539904 0.730198671923033 0.791799511779867 0.704472263421137 0.758699315751047 0.756041114053924 0.755586871628123 0.788630103104091 0.747912039494774 0.749133098382502 0.714827969568476 0.805335835550449 0.733917465590392 0.788267510310072 0.728295083897074 0.759930494022911 0.78293463145155 0.750319845887697 0.788523036473495 0.795925684099485 0.700320729907657 0.703059274594593 0.701753253649376 0.732597973562688 0.714370381904121 0.749890011388591 0.783955681902689 0.774413368128813 0.729043597120433 0.695083118910999 0.758284694311795 0.706717826747434 0.691585678008644 0.75568016947145 0.805830980964245 0.751565016271189 0.642332051745337 0.734611736870709 0.72552298969773 0.663289948179899 0.727526901593822 0.732721307869409 0.776167699315881 0.733869923566207 0.723307566650686 0.699690369773835 0.712095956624055 0.761371667821071 0.713939412147317 0.771463882678281 0.760032916181989 0.734418817186064 0.774717084644136 0.738376348975891 0.778955951535787 0.743740285701895 0.785339091025266 0.738371179139114 0.748368133828844 0.756861061737708 0.710622838716122 0.738511197029538 0.753694555851215 0.722615920558596 0.739320738655422 0.772504748525017 0.762721667000398 0.725813433565131 0.76085830051516 0.782094367552347 0.77004723099157 0.755316814356696 0.667138714336441 0.76771529051809 0.733626868953216 0.773547235174003 0.744522877864863 0.812007295792549 0.710861346278311 0.750157918353944 0.740160338409917 0.71237164838132 0.736062087967643 0.727360863207959 0.742517814800163 0.776759314226764 0.771892325702513 0.766236827699091 0.71059695462649 0.753068663276404 0.726734659741776 0.744382581023511 0.735955310356265 0.708054022462483 0.731284245510237 0.736077580006108 0.721814124977646 0.736354694502132 0.720810210497995 0.760065539892916 0.708663857324128 0.735079067651264 0.747909865350174 0.754746931345485 0.736891369925618 0.731278633122429 0.700353472848233 0.763024808985532 0.760500260257337 0.750499763436948 0.685040139779173 0.774112530681709 0.761226050832784 0.73346938770698 0.772718090419873 0.694083651944561 0.747375802222516 0.70844279472965 0.800608765345005 0.711770665079362 0.829804457845326 0.762510987989663 0.756937968849414 0.72774230599758 0.715640997075813 0.75266192412227 0.741119837435755 0.775161135833217 0.739629391228789 0.755811008044788 0.787296981658431 0.721185602030029 0.785994373597693 0.784799420047209 0.749723825406045 0.739384839262278 0.706623996688783 0.781079510673321 0.741505468087008 0.81712800860807 0.743101418494428 0.764672855715805 0.757336171503023 0.769347218694182 0.707985766978868 0.773961324733802 0.706411933303021 0.775403295390004 0.730152442110517 0.770258263517711 0.799142538321251 0.679865666433149 0.720790198431631 0.745434326467008 0.755268958983977 0.752313167686337 0.689177249307745 0.737814884924945 0.766990991278125 0.737712479312589 0.693078221342702 0.687015227956163 0.779282031217412 0.7399161377192 0.779306529544553 0.760963730426219 0.742263839450259 0.752397652874658 0.788196328717374 0.716293072769458 0.761026096922398 0.684401743370517 0.708206920750462 0.7114692030623 0.714278568033538 0.735395499693517 0.759599648312098 0.741131970419469 0.759409491660776 0.717089843287797 0.749111084641993 0.723095574868354 0.770386368133624 0.684044606253535 0.767871473125795 0.759714043799723 0.666344764451653 0.693138644667337 0.778419967099774 0.745764633155885 0.704533350990392 0.792848972330539 0.693445240636817 0.66167819859277 0.735465818090461 0.737463461352901 0.733643225865743 0.705047160404134 0.738237307306837 0.765510651646543 0.743036610104257 0.806054982810969 0.777788115119147 0.71571036730786 0.826368183417846 0.739919348238722 0.796929823515392 0.770967068939262 0.719783451554606 0.672332258663927 0.799189443103643 0.734267626466179 0.753994310472864 0.764095197704486 0.734349197015588 0.763843808941863 0.714885111094475 0.692266068118215 0.757636707161273 0.759335593358774 0.761851802568894

NK_cells 0.47166837231916 0.432738794003618 0.366454977982494 0.479954306261667 0.519313754479381 0.3833915846406 0.515617610324466 0.636078812328846 0.589423048862752 0.68261192665946 0.464958048326736 0.509323428503354 0.538724866095672 0.52315557867699 0.608525420838406 0.449721706760929 0.620830106886575 0.415907537172891 0.700029052271898 0.305275086008768 0.496083000765559 0.670694804347309 0.423150272083742 0.554759475817702 0.62244157354159 0.475583337930247 0.61226834189547 0.267532693203464 0.587487774898385 0.539309055618401 0.544671081458551 0.546334709918898 0.551170403260568 0.505983651265171 0.497219161668635 0.460852852276392 0.532600886932789 0.59615416735147 0.497718404142149 0.422276140267404 0.620880498096791 0.492225743986802 0.590419947883227 0.461126002179528 0.634965255539656 0.577433983116406 0.423701002756135 0.587342734184863 0.338147705315485 0.545553265510436 0.604468514516837 0.441809666149826 0.516585506197526 0.500837063321997 0.699290238239881 0.518088566463622 0.569147476879489 0.450828948680652 0.56661739874053 0.51937607208551 0.458946898060051 0.564807547732935 0.290944540685905 0.566792205442707 0.624887605845041 0.292748881344874 0.56186254500141 0.534416827731596 0.653222406766337 0.45238863387611 0.558403657312638 0.524458574795071 0.587266295606609 0.591191075380313 0.309596473494796 0.440879139934205 0.645175947490262 0.545309616567593 0.537285056321965 0.545296110680456 0.224527961107517 0.473421495497288 0.514085173217334 0.602817426755745 0.554387891403159 0.591299318215392 0.421889879935711 0.434620000583825 0.458865207791209 0.238913317076084 0.526758574182992 0.621676624175745 0.476058816716876 0.552023880662182 0.528961079076938 0.50229734887465 0.426914922435302 0.533480420104999 0.332920452609461 0.510347197526968 0.474182399516714 0.599097169295969 0.580208262674322 0.54652958801575 0.543059606407024 0.572469808651068 0.460197327559596 0.581214429644578 0.548527777072078 0.525759896992317 0.52008391095738 0.540500145141412 0.57258190701461 0.472633368624508 0.600395018453293 0.581121971081818 0.540703133646955 0.395136931157002 0.611917262334645 0.466287605048512 0.542617951142484 0.602717832728801 0.601820436709143 0.645748039555701 0.531362248619269 0.560576459420085 0.563057514967526 0.540114889198831 0.587458768259049 0.467135791572023 0.564568225616076 0.566381402911336 0.656138233674234 0.485546228584985 0.612093512240098 0.614071987419186 0.52776411170792 0.446865955685215 0.449340384267464 0.489888399474013 0.45104828818979 0.566768214939881 0.573657626175649 0.364766869663123 0.465300219609003 0.581577396254085 0.56343913015134 0.584823205476046 0.570179428183636 0.614771189105472 0.557914125629589 0.499052268344468 0.588102112522756 0.583822070727718 0.545054587211899 0.583839109418542 0.527531127926617 0.535295897226661 0.552898317119543 0.569299169474318 0.582557371897154 0.679521797182994 0.545775922828071 0.281422674884903 0.545765428377482 0.485421164322272 0.55756020883695 0.647319554735811 0.556453573168212 0.5551066099172 0.456791968454666 0.504319447943927 0.609048583835566 0.615431208281218 0.491321188417281 0.424607186244561 0.52617076264883 0.56587452312165 0.500506057602924 0.618684109554022 0.625413669498633 0.657953611919577 0.603830097868518 0.522031210847615 0.485673835749446 0.598940089390983 0.445311467629036 0.576310777774392 0.632228978593464 0.520340853185809 0.60597688392207 0.553683601817289 0.546875904913744 0.600229562009113 0.487401255855017 0.557388571875716 0.560835087868466 0.498516425091753 0.502703648215768 0.576800000785343 0.47412656747978 0.514373527679419 0.637218867357374 0.450705018977016 0.388766723543505 0.515728068801727 0.56327513698674 0.577579588186959 0.604363740779137 0.633697667016987 0.733166896745639 0.514579313817909 0.534542114163767 0.553077793861093 0.645242034204684 0.565152211274365 0.398637263827078 0.570525484899159 0.582183631432215 0.638385077828997 0.593760845623671 0.493871676026468 0.552074517276709 0.607734763005044 0.511126014328305 0.610170722385799 0.565620878903607 0.360300802449418 0.545961689782159 0.390974482987921 0.517833482986454 0.598885632189731 0.539504245635868 0.498035040448167 0.524842346365319 0.530792024750939 0.625858168567512 0.608301647271831 0.597427052821201 0.294643017234247 0.517561633107619 0.457952676497338 0.531752999351649 0.493058596769097 0.542997898758532 0.576032471945815 0.453387111854828 0.728788638353016 0.572867100746869 0.606265238444276 0.597241480945561 0.581055291135572 0.547545639770149 0.542368389503963 0.507460585028092 0.53292561638948 0.723730977011057 0.590337152195131 0.536337286339678 0.651357750425244 0.487396423687287 0.547357004388214 0.434105585466862 0.537815913646185 0.502120135044595 0.503537978339622 0.334143320867017 0.563408856028922 0.531140351878817 0.620403202577686 0.549410320055933 0.503635665708743 0.563369280532275 0.299220428585995 0.614319658180044 0.602350155880085 0.599363280228289 0.62659677903851 0.59134121520902 0.61094782431497 0.506677652458012 0.590478521432137 0.478040825506095 0.52362763316392 0.610567962483964 0.569672930042566 0.569076242239742 0.495372183957667 0.486701203572735 0.578934976009295 0.465517800500167 0.467415418236664 0.501717751764085 0.579170912200359 0.508385551296776 0.536948554516281 0.55437881943982 0.513780243254986 0.594695765965667 0.584561862949199 0.63109653853759 0.575909013580797 0.491804887372888 0.44089868964632 0.481950811925521 0.519437899602021 0.501963179663656 0.52265398781584 0.221196993575538 0.445490269174562 0.470255189099185 0.542968686308145 0.539149303320235 0.490237736577486 0.443569055096294 0.588623206787716 0.481103622376394 0.466007304104255 0.478435325760821 0.439378333983494 0.527451375575661 0.532608466626932 0.622935936361306 0.549192548036186 0.532419842025423 0.594883242616473 0.582091927785675 0.53586504302752 0.461399236983938 0.611387195254554 0.547713561788521 0.529155305548265 0.600471339735616 0.608726908192077 0.543264496258343 0.531357486092695 0.546138463278253 0.494572816779438 0.511739350564278 0.640985702035064 0.490538309797255 0.453947102938409 0.598185097380537 0.555043402469448 0.497512201866248 0.573383092703492 0.437322488075699 0.482980736315116 0.408562759196769 0.526533908211173 0.602760226854635 0.640274428524402 0.615778689155515 0.696678100658383 0.423498957334487 0.553686544637047 0.41369221817848 0.467057576107373 0.608241299474427 0.588360273401596 0.292476279840623 0.355653725412964 0.526565049038273 0.63084804949679 0.562864034562047 0.447896044579768 0.564471668837415 0.428514115808632 0.447534053702213 0.557980580009168 0.610875212172466 0.58591602797869 0.401991321801615 0.463595900744884 0.618899383275231 0.524894351537792 0.52832424139871 0.488629300764115 0.534490458344859 0.591401268347433 0.634161806322187 0.692037734792874 0.554301337001148 0.553103808979404 0.606729421578927 0.486476501777948 0.562864789461853 0.570106787533452 0.583760499364894 0.649217720609211 0.453924040371247 0.543321382546229 0.652369243817734 0.499509575850408 0.474714923739205 0.500273535470618 0.542023121151874 0.537597381680994 0.533264162403661 0.501064965024949 0.544413516021531 0.52183759049676 0.591348781276619 0.599753053503694 0.633582909554837 0.646950947701198 0.530971494310018 0.588462274659156 0.560082455918507 0.473427072130254 0.582273878317352 0.444739508193322 0.621489674441889 0.516226698726792 0.52824505302852 0.540149235514542 0.559856181203586 0.622038657822357 0.591556283436095 0.599747391941945 0.545881960233537 0.521031848303057 0.43687192242877 0.506581804777261 0.547704935966842 0.497504103527893 0.476106124542312 0.571922360165231 0.558398746395775 0.465599267223954 0.478815512780966 0.537744761087487 0.49801639534337 0.503002995064105 0.558325588993664 0.296795310916803 0.49434431600307 0.477221432780826 0.619389258612691 0.622431591142792 0.623506633618741 0.67241436032461 0.657719416540733 0.477688435250616 0.519104989913273 0.521729051807931 0.5970658830513 0.558090570263073 0.602730104852926 0.602613813470901 0.399171279462752 0.455881015633553 0.262716242719125 0.5334661185289 0.345087103956487 0.632692176668632 0.56406946037812 0.52112819684097 0.521492038545288 0.630089320065517 0.518697412716456 0.52884825763412 0.588158412579643 0.422651888814797 0.547442286059493 0.525658908541427 0.530876593242591 0.490354700436717 0.548446867607866 0.672420694911776 0.436055415503591 0.617998713952889 0.532617921431161 0.473618731667941 0.233042465400088 0.516511413098158 0.549709701859993 0.531027084418964 0.453867535348126 0.522248144743785 0.550101929298748 0.483603825034684 0.597209522309276 0.524131817541111 0.468413167935147 0.53220751700075 0.480196271486965 0.574875792242529 0.585239754377477 0.493943160543891 0.619474636266522 0.525808209057508 0.634814328051301 0.519263549082939 0.505595703648837 0.468793940961225 0.570361479562444 0.552580395786429 0.518430878492987 0.535108249435285 0.664849008125006 0.556627366196821 0.567213287452951 0.459415396297659 0.52429006961959 0.430214004245589 0.557028293510074 0.625318359668941 0.501059776439697 0.477724193860199 0.295132820431349 0 0.580871534155665 0.53179224795526 0.510937712723196 0.521394010632939 0.515323732129331 0.516968246012824 0.548415317126955 0.386621073261567 0.46598145733288 0.573052640560798 0.634915015134946 0.490746357849427 0.521735219847511 0.627254209181772 0.529803644816344 0.555428047869874 0.626997922620412 0.46299903173941 0.615276761652036 0.273866337048226 0.541377760486283 0.577857024212359 0.373709969768901 0.52058485416244 0.621311740717592 0.391322612475546 0.521413219070286 0.504464912319743 0.609820639377951 0.491585850302198 0.428206132669699 0.498898710382265 0.456620615139411 0.512916594987517 0.46391152441886 0.526041861668649 0.427981362077359 0.598451618391775 0.581952596009441 0.625644970549874 0.582894166581795 0.619018478551834 0.464921382104306 0.601947035918751 0.572635589139498 0.617582471787286 0.484733366676259 0.598252179345227 0.451370636609696 0.47811022764914 0.567567017638343 0.560636581603042 0.612858631155748 0.642652442567676 0.430023411885208 0.527149980853039 0.532553782796927 0.473189692648533 0.595904260067302 0.389372717471371 0.508005474529492 0.517945399439871 0.562241416963256 0.480587532833131 0.536835036466587 0.487173898667332 0.476979580097295 0.551721412350609 0.549996764026635 0.496117095119626 0.518957624522438 0.53089676059845 0.523434791517045 0.520078540527931 0.57339838388061 0.52322680654909 0.547275740030064 0.502939380479762 0.591548324611606 0.5445686421273 0.567422458736727 0.502142948340096 0.0192507159880468 0.557551293589996 0.478535395516116 0.594815533020389 0.547451356274918 0.534911817469898 0.577023818648837 0.497464097329355 0.621743176218603 0.544822743160776 0.477909520783856 0.43803106895983 0.571014774140743 0.586324340350578 0.514099871718733 0.687847461365445 0.467799620893928 0.526761058218839 0.526310603038704 0.558803776653867 0.526083975694301 0.512265854034443 0.570159229248928 0.540904023056527 0.665089063679373 0.604813586242941 0.400486064602812 0.579237743548874 0.531885629947807 0.42201581095578 0.532989429476289 0.491751785535041 0.575278725833864 0.587042479473865 0.518704872290782 0.578529557231422 0.571184792752777 0.651408906878718 0.520913345751049 0.49225916956339 0.477351019201508 0.549691775370672 0.609919606969556 0.472812944156574 0.626123556390971 0.516746335798768 0.581810710610882 0.580165300033928 0.472923345713574 0.567279685589012 0.615359696705154 0.438155383373926 0.49809568129776 0.631063916152585 0.482971716685723 0.672857106358274 0.609897988525997 0.423906179740577 0.551581005814925 0.507121198173997 0.687577692635335 0.565158861465584 0.55326523540495 0.534256123329736 0.576573446801064 0.554105420740321 0.513436122244165 0.586930546067499 0.543698813288894 0.508978474993465 0.28584773553695 0.641457524790483 0.560645570168584 0.422985836280689 0.644188684733659 0.555153980294695 0.53193492759453 0.74207547545059 0.511739391281595 0.570786314686845 0.509215097518363 0.679144478002216 0.32002147762358 0.567841263118437 0.511626443503489 0.537217335597965 0.687740198957863 0.580259778896186 0.364794354028134 0.359145815154596 0.508014403544096 0.392733791658928 0.473393913322774 0.556263792648789 0.538114830351812 0.560455773454432 0.504390503410119 0.557961991104496 0.637084282702449 0.632402759352228 0.466435839377071 0.654218393775555 0.551014621074389 0.54726496933995 0.444488729136657 0.562601812374147 0.573930843396994 0.480869767699692 0.601300797111332 0.652760956400712 0.5828333179036 0.494001103951713 0.534531702021068 0.525022289241394 0.551811786064883 0.516522037780575

Parainflammation 0.786776879696614 0.735935856988391 0.743452575220298 0.763705183546757 0.775915020339012 0.834940803797942 0.851812671916922 0.796213899682746 0.805718249324817 0.843531016747669 0.819704122928844 0.74838978596379 0.721249004527998 0.708741586789484 0.720285419462869 0.794884598119719 0.845154095219062 0.757439653588761 0.735845774196646 0.732120418764741 0.727512999111567 0.853288166098098 0.773848189599302 0.837798837687224 0.817486422108017 0.816250836581121 0.814949296916274 0.725752631451241 0.776413199165233 0.83523886486861 0.863782009611869 0.78858813700807 0.743231316959684 0.865191955859704 0.843230866365788 0.733870814340772 0.724384654627546 0.769145062803195 0.771974558289533 0.769511088921693 0.780469058869743 0.77487958627162 0.742274902391606 0.825149282827094 0.764391901374934 0.76090435063986 0.840732437110304 0.742253085449045 0.833469056174214 0.776288661786373 0.792515809980457 0.731561339848293 0.77273985638699 0.87810901570414 0.752069078585099 0.811235266387472 0.772053566350742 0.865407318758349 0.730761601936211 0.787257335947628 0.858980765206034 0.767930914464273 0.791170365890496 0.797670499442445 0.864811988156597 0.857596918481061 0.742560382244978 0.843465565803033 0.755056789839046 0.833343631305964 0.780256991358345 0.777534314793033 0.815426586662668 0.721276243938693 0.808991460231285 0.738459338112458 0.765715158082568 0.835706260232666 0.766259239264921 0.817266128904484 0.816216932191876 0.82416215722123 0.777886136632758 0.812468102232129 0.855402405360843 0.758746415340702 0.783773840982928 0.749206305870312 0.746079336946314 0.843126270311891 0.724229399598639 0.767932068139089 0.770604305077716 0.809684589120722 0.853576144459206 0.72513598384682 0.758061395486342 0.86100384040213 0.806935292260555 0.848281038923408 0.825501880286463 0.84244157439793 0.779012354459234 0.789682628934083 0.748523027990095 0.759992663141783 0.80237997662955 0.808282602125523 0.820474257138792 0.834259862119219 0.815262199919165 0.761228307902447 0.773015988057383 0.858143598657286 0.771902273402374 0.822452900040613 0.846804258798586 0.85152541589558 0.770127657118837 0.847774698378372 0.776245680220723 0.835207017380913 0.785658634262387 0.850682299925127 0.790007168983842 0.851999203811406 0.80893446229431 0.750693498941605 0.791426048870499 0.734677976635726 0.773826948246104 0.82201409846463 0.823930291329701 0.848240451590235 0.845575058087059 0.788020028570729 0.750791303129573 0.81939597729094 0.747417906196629 0.798152438203272 0.768839205089682 0.862149234846469 0.83081056663033 0.81155400163486 0.79802735519755 0.753892414399478 0.780977931967969 0.771475393635326 0.773814284231967 0.795499293170826 0.753555312388644 0.824677107086893 0.784347905943882 0.814986237322727 0.771639089418235 0.839145445263203 0.830324601453562 0.826037872575612 0.811410556965495 0.76647748878261 0.739601715568029 0.748535645229446 0.731087940744442 0.720989242738318 0.787252329761488 0.802670892637978 0.75689820115246 0.789707887746707 0.827832291593256 0.759563232240494 0.768523267531444 0.86968819104706 0.731525118745632 0.77559309198141 0.859198591509776 0.833290005799089 0.759408105165357 0.767564162550059 0.726627355623494 0.770615203720087 0.835770707891224 0.745330808761481 0.754005731441642 0.785689677414122 0.84864560259237 0.744948106474651 0.825113116164978 0.751404200292853 0.743489860194416 0.785738907397111 0.727200224266067 0.850467490242815 0.77636653619811 0.844030718288162 0.84966433063456 0.834457526838232 0.775347872816752 0.746533104827187 0.796308727837848 0.759744993701008 0.841915622790598 0.796635694592544 0.801535556179054 0.868164964603014 0.704029129949504 0.780390713625262 0.741575476513976 0.793281773127988 0.817828212275873 0.845225736376784 0.748842586460824 0.861407576191223 0.762235614579813 0.81610728716239 0.837833651418813 0.77303200286959 0.724011373087081 0.727736473894871 0.802811791661602 0.73255177299165 0.796774637234922 0.739365702732005 0.814996734356064 0.786152519879955 0.755337149719802 0.871379030564132 0.788669922842766 0.844069103362844 0.836815393520547 0.849065642135509 0.80887878763138 0.768910965450762 0.783814464870917 0.842060591761491 0.797980486345427 0.856768006429529 0.724803892771289 0.742550794840518 0.732576682691073 0.75624927256491 0.846039664765636 0.774915382972077 0.79008356577798 0.76888681171057 0.808632306394815 0.853823552102849 0.813048999632539 0.793477371599916 0.775735756839584 0.879657418000831 0.823926860798492 0.805015314688232 0.832064181417586 0.8493612491327 0.774133724919607 0.847714156710578 0.88128640909719 0.817790741797064 0.762178623359845 0.82390973589226 0.807028296379083 0.788145364350243 0.730002883991283 0.747517227772904 0.798836649757383 0.770029029934198 0.810324274535663 0.714810074724798 0.846461223680193 0.874446517750318 0.833884188778777 0.793899577420602 0.729873139221501 0.803838042158539 0.726059721708388 0.831495820653947 0.802246744702638 0.813559841655286 0.743763333936578 0.825317896530773 0.859045093851791 0.802410605772179 0.735369816227492 0.843941410644766 0.807492440995899 0.860054628033587 0.771380273591177 0.727314386457601 0.840390159063717 0.76524301236981 0.741983613209682 0.803242849867802 0.766544421960101 0.745707658613148 0.78314583336417 0.819250375852268 0.745687127939726 0.811567866742256 0.754826472072369 0.746772155708787 0.821252894369256 0.839735537448668 0.749455589353724 0.823031997629557 0.821139572479186 0.777352637067685 0.844555654990803 0.745892673117337 0.834602983074698 0.750653155105368 0.851326891141521 0.761093986772741 0.776118200653372 0.752889086950468 0.835171403635423 0.798976579616046 0.74269997203142 0.757859655352109 0.783320979063188 0.83928950459172 0.791981362715449 0.833294488772906 0.760731496571896 0.756518613804978 0.8458394830607 0.753460519481314 0.782320967560787 0.834053716041652 0.840447395043025 0.859534047326765 0.765885555040639 0.838331567782535 0.81522998453932 0.87654030481714 0.787884025545116 0.795028742098759 0.758982908480666 0.877521238713827 0.854271130423906 0.739886346312773 0.752536355536289 0.810563989480052 0.75200072151913 0.862772905312119 0.81806274755318 0.894421202573762 0.77929955529694 0.758542935718276 0.803682100020074 0.83284798996074 0.775321927530073 0.724716849354261 0.756316496242375 0.793902391821408 0.820619683371746 0.764684106515648 0.831981201951811 0.839296943025737 0.848864748414064 0.799845352807778 0.765066590039516 0.811135214357015 0.855948713792765 0.835396981974007 0.797080934471094 0.814843267936001 0.75475523732143 0.797432997779701 0.853748838014175 0.759138738054227 0.833413511045994 0.866643665437753 0.84519457205516 0.733873186290986 0.788372398515704 0.765186498781838 0.776235198520646 0.86013164472804 0.834761580693317 0.784011206949788 0.801214744234844 0.884700785644194 0.732517596099051 0.746089328969788 0.778014453238309 0.803072328313576 0.771688903139951 0.820556083743905 0.724542038417942 0.867916299101225 0.779815834775398 0.789526507645557 0.809909525051789 0.749890384194945 0.855068462194294 0.848830920459478 0.772900328694557 0.799062732286646 0.79383173638649 0.84396221429125 0.753476930005473 0.742944645200865 0.804664470053529 0.845415825421717 0.720800598291035 0.767476243764438 0.774196110757882 0.814595473776668 0.782897684138872 0.813152674173933 0.779359786622592 0.730091374736713 0.705581683396682 0.80587512014782 0.740405030469987 0.819599492409604 0.727936903874707 0.776324402236369 0.854921084957682 0.767582239815466 0.800646680468324 0.779856925853174 0.798972743677309 0.77039629739466 0.840989311734019 0.787151511348658 0.752846184536629 0.764602960199944 0.760292242132514 0.732284970119971 0.830919463800897 0.751064352386686 0.817494469974457 0.840240982477185 0.755382761791692 0.760978915047593 0.859737318035713 0.861546107098623 0.749965812000283 0.771254776585089 0.842366698771526 0.773482916400206 0.773040781361316 0.809449778461469 0.759438279044099 0.747486951186642 0.853984883526727 0.765134541903375 0.785049773841674 0.789037302561868 0.78054811040813 0.777526308001482 0.815537297926748 0.860676755999986 0.806584310366726 0.850294786397488 0.792656403174496 0.717672073207611 0.838355956280346 0.775021010373974 0.75498610120803 0.781420102622324 0.804867099285808 0.725109698809831 0.828282167734762 0.778976135751937 0.814656519378919 0.749684045252844 0.808872249736794 0.776817561008258 0.740948332392726 0.752389779767181 0.728660598515946 0.853993923114006 0.828506170319054 0.778520943986565 0.774090887926886 0.815320743350585 0.75428564638245 0.837041346862215 0.776964823998839 0.8236759120483 0.777641196021198 0.771235807558668 0.759767297692601 0.812099953409625 0.793084376719743 0.838559052006799 0.824626076065343 0.737028409030379 0.788198692532041 0.78015075328396 0.756370457393385 0.744588011761422 0.808718370457457 0.792658676618805 0.817768405641201 0.802070238271597 0.769013794190404 0.724115520615505 0.890019859009099 0.732797340533201 0.851238185226361 0.815137840575581 0.749977201281555 0.78377601397548 0.796882942307153 0.795054547654881 0.860983125672838 0.74966367161377 0.790525813459585 0.788835817109484 0.74110843165575 0.750175557909976 0.780596175307963 0.770744806781299 0.750062903623105 0.778550237186491 0.732243923146295 0.840046460778827 0.78734865828761 0.735761629413747 0.849626715965861 0.855918741037795 0.751762232637598 0.742130122142828 0.758728992274707 0.734656495528806 0.724160866884014 0.767525378882722 0.783817512827276 0.843127971780002 0.749081422439749 0.751432282563316 0.802212834279063 0.732517874998657 0.862140699403393 0.750926484935566 0.856286390824182 0.782548997232818 0.770340537098516 0.792545317390516 0.837884047577999 0.824373509227821 0.769437304126599 0.841131854932751 0.774289571695844 0.829176954046463 0.798459817493713 0.748813770771694 0.850626903115691 0.780121434473705 0.769667643026138 0.768677546588326 0.820160927694826 0.792762483589636 0.735683629525394 0.747655070257839 0.811685719007583 0.855924077442757 0.812794640400512 0.716734012695018 0.843161014851789 0.82120864965543 0.805499540698228 0.76541180926995 0.859050416937245 0.783689757113747 0.8078997377084 0.827556545982972 0.733066955975499 0.745538294264563 0.803118431969661 0.748713131911416 0.814242604647751 0.810998810571795 0.779613750919772 0.807176249364229 0.835541826890076 0.759008983400785 0.768749752173812 0.744218517691434 0.81865395874828 0.764134155751244 0.746837167105762 0.749444662959318 0.758948866410206 0.845740026591821 0.792372430680923 0.751935437379535 0.77417700989394 0.77969080340693 0.785916649286425 0.773589051943939 0.740697190153367 0.725888914835979 0.831728278551723 0.747453494931998 0.758573206598365 0.806952888117896 0.785437112070503 0.750453321277813 0.774812241518145 0.762181574311644 0.764748129823229 0.808782119217805 0.745397035334502 0.77254098287178 0.748574375305988 0.858968811100364 0.759768104101277 0.860445635726229 0.733129512157343 0.804656923805382 0.834816492154002 0.753666713527489 0.821012756876718 0.7562631531132 0.788573702109431 0.844632947852203 0.792344704995005 0.793404706623621 0.733935138922325 0.765926739820476 0.757990306827559 0.817147880990151 0.805496229285803 0.761788463242767 0.868997832176132 0.873419684225857 0.764512445577488 0.824685267853478 0.733637721424766 0.734027971317036 0.77330911741576 0.755583857723798 0.761533856251958 0.754983710991114 0.809622684946818 0.80811993826142 0.704673136787456 0.728714929379382 0.849910211021302 0.747337892887426 0.790999616941072 0.834583061861159 0.791494499699352 0.853787254299541 0.836342255842665 0.819424130398044 0.802318087326578 0.732005800451895 0.766471389703162 0.737565995092709 0.812910762308641 0.840665348420291 0.787298788628103 0.818123248258753 0.776594475851132 0.8656002573362 0.796407145050754 0.760218171281049 0.749266799408955 0.742146691567954 0.808518987575591 0.830970817516372 0.78934073449099 0.821504150737478 0.749354206994347 0.755726267596252 0.74511624954771 0.793377932108113 0.840409870797434 0.757503201574665 0.764461186227049 0.727722930171687 0.811204829154984 0.889853187190242 0.746736255707473 0.75124326334567 0.826222628706593 0.74870375488416 0.744674543506659 0.763434700135081 0.773599896999999 0.728131167126591 0.785454096341791 0.770317810483044 0.81840245339833 0.799122130704731 0.875196527294093 0.754965122622391 0.728259669812194 0.851279303003085 0.830038621392557 0.856578631300407 0.774337089197359 0.745244187031673 0.707860998741814 0.828054005811738 0.81492536059874 0.830954518823312 0.796259297613561 0.818651096476202 0.844965136333436 0.763968109542848 0.767218997692673 0.841372294604406 0.79294051793379 0.865076834693914

pDCs 0.636131149951312 0.577053062604122 0.638310561004434 0.642038177674371 0.61101022012467 0.668093761347568 0.765868145461898 0.674597870517362 0.650526741399696 0.773515036131519 0.653642291550029 0.647202563018153 0.539660303767032 0.551877839806752 0.574852622082265 0.691095861705515 0.714117531237109 0.571898905744293 0.49916906819475 0.559145767545375 0.465048501988753 0.74490065330684 0.63123223413859 0.734084844695555 0.693748838195423 0.68859800097974 0.728488667707957 0.549012381214088 0.648243267951046 0.75202855667319 0.757188730300777 0.622341847034122 0.609005727800704 0.73562397618236 0.752232500895383 0.598108761150529 0.627648798666478 0.649447747445295 0.648220391175478 0.607455661630737 0.577325982535774 0.641908344662147 0.557893775936925 0.670540222688142 0.638101345164403 0.627308056224842 0.676544871210216 0.584403304917191 0.671803529010624 0.640262584821866 0.695274936164317 0.617946573782019 0.655391134079872 0.750809197007258 0.587380655404746 0.65180925149511 0.583169043406449 0.763286226767544 0.586486178232448 0.656022797488483 0.726740239005505 0.646691311006012 0.651590414715219 0.718056908651933 0.731097701502544 0.721001974131882 0.604852413793255 0.720753960828525 0.494221852452696 0.698908138264787 0.642579720914315 0.625447716249755 0.670944142892115 0.543076027906948 0.626999889334464 0.607327136852652 0.602526636973666 0.691162720836364 0.559601983956204 0.750024647851188 0.6424530507734 0.772445097475317 0.654984355711348 0.687114169700669 0.708052051113347 0.544702646895903 0.646471256633578 0.604892101790225 0.561136748355599 0.671757071447181 0.595310884990378 0.597526995883557 0.566798681143727 0.735438234315132 0.74196731059317 0.529791659308284 0.563038640463309 0.712562649429979 0.740726516214884 0.702318528999232 0.712321350151999 0.712404083892431 0.650865032009392 0.640052395333364 0.586390633376053 0.639546711906424 0.669624404608384 0.691724658089768 0.687889712137698 0.742818197541385 0.641498816162271 0.618840893279036 0.647227091419713 0.701803860371362 0.637450031668315 0.644742480940713 0.691157373588679 0.71233003963751 0.676483026268606 0.724827589484928 0.624877491145505 0.755152873935787 0.640536788188826 0.695961012648961 0.718935011898925 0.73386744391002 0.697633666643124 0.642199426812117 0.64661565604003 0.623474224545735 0.64956471783387 0.672918559788831 0.736937708939301 0.693477329258629 0.724699823883656 0.69469941522593 0.630716776293226 0.675505788236793 0.593053760403222 0.69548652694551 0.636191241809734 0.686633245500788 0.716948529179787 0.647372164541819 0.681378055204483 0.585676218099218 0.657211270462668 0.592131130055328 0.615594878697467 0.679500640592505 0.583563407521592 0.603709047355383 0.661966081331344 0.617914040047199 0.603028399252904 0.66874851914474 0.732085625386611 0.717751256373733 0.658401363669093 0.566359087417878 0.618076246987451 0.607016808842442 0.538958448377921 0.529189338504179 0.680190946332431 0.648388854856624 0.602261585089441 0.637602074122764 0.660608695890919 0.576899157914041 0.600618302478627 0.720685126952483 0.623255094486851 0.620179662150881 0.750609236716477 0.63266683583654 0.610617847870606 0.633426532898783 0.613755154333136 0.62664399427923 0.661336662587111 0.633211310065788 0.617400895334556 0.663782726814186 0.736389710404076 0.645725009643596 0.655396151961481 0.589262375600016 0.587385838856719 0.592187754149111 0.57508799205021 0.683380085387917 0.605044824970547 0.751848080329209 0.730081653544883 0.718010253115255 0.652582245456652 0.632937059705348 0.680634001294271 0.618080018764427 0.756954357462715 0.595690260514245 0.675321855267644 0.654694412759702 0.572875035460371 0.640008810836858 0.654799942963773 0.673110149923253 0.681998843790774 0.655117470037244 0.525129961568868 0.755889708385444 0.605035452964533 0.72149219362442 0.754886015365535 0.658278055695759 0.534415554046541 0.53338145100683 0.698706576150423 0.526811798782444 0.721102663753257 0.534961347435589 0.712549168344788 0.671372044368291 0.610039176060394 0.7880452264907 0.623858492123056 0.704660034333706 0.688230121429044 0.674989953306278 0.692443432711247 0.620136670033583 0.543238754301184 0.74620760019854 0.676345827039 0.716005556324947 0.523351855330418 0.595796097311832 0.573949947456247 0.659912089633626 0.701907142001032 0.615586040932731 0.667260593681486 0.633154098205511 0.629874371328792 0.738404403171354 0.685063779608974 0.619387889347248 0.632975736812104 0.805035845333185 0.734748069239554 0.732989920604081 0.694221402354568 0.771123622890683 0.661310445899393 0.723370930772161 0.778726976028036 0.714592668834045 0.606353481523757 0.702905463369961 0.658191442740938 0.723768091227155 0.482549935538013 0.643378716088432 0.643495816583207 0.586884196659027 0.59077668674326 0.512862710732924 0.623921730569832 0.757271656913369 0.719029412324775 0.615843180053188 0.604558622216141 0.668250904061991 0.542883349439404 0.743316025428933 0.674303144762708 0.737962051677645 0.63384056375088 0.735161635374795 0.716652090005151 0.713570127387563 0.542749751248272 0.717139154176954 0.708124095717054 0.791333297008194 0.682988171628782 0.534123340152328 0.715485579710483 0.610294856300176 0.585805942604189 0.653357832835261 0.668738873353253 0.519084074365816 0.665961617336796 0.634884592227485 0.57659596637261 0.64461355346685 0.620393889581913 0.580503387158913 0.750212085731403 0.699590378280066 0.573284961268651 0.676118480544168 0.732550171592825 0.621924578203289 0.684798202792062 0.622343788622715 0.644290088905445 0.577625939273009 0.758843603810398 0.643611811255033 0.667154634289252 0.513667430501288 0.70442705031245 0.684219376099153 0.525389817952545 0.604026904112401 0.592360221803378 0.639366057239414 0.619128125174933 0.742728418103109 0.64810039765438 0.600745119318565 0.765427367238121 0.521756300049269 0.61650715875999 0.689059873578634 0.692811143007516 0.686774138084775 0.642505282294098 0.706325332447958 0.703550507949104 0.708152287999472 0.609665702999036 0.679770380501002 0.652733592053197 0.788493687241408 0.706153291025884 0.626556283540074 0.605827063644388 0.602045634472454 0.612145006608828 0.752948509005276 0.699845905872716 0.760211494351673 0.60020729741201 0.64335495419867 0.602493104590131 0.630292514528277 0.709178416115339 0.483079941741066 0.653978182590002 0.601584023487595 0.64220370126104 0.632605107908379 0.69478892709222 0.711345039242699 0.67952520054942 0.673959301241345 0.573208246651069 0.538658994000967 0.656083578092324 0.755697203114302 0.725698051529701 0.599267266510095 0.604657358090388 0.656058582887369 0.645278388322524 0.610672359426138 0.694374949788968 0.664459542434668 0.701288077914446 0.561128955024582 0.609676564171635 0.622624050379858 0.677922715437061 0.711028282178413 0.697863080030562 0.679623053204576 0.651290194076845 0.764496768963894 0.563272496702727 0.612008440289387 0.655047042567629 0.694919642314655 0.645631101085908 0.687336511436779 0.527290609378326 0.756684414695756 0.628899126104604 0.672469837871769 0.715054085987158 0.563208880586358 0.695976341296606 0.710260485221043 0.641102615027466 0.692383295520732 0.655808916669854 0.713934357177582 0.606987684645289 0.627240196008892 0.711735508973824 0.727347181652568 0.603253684031859 0.608320751655912 0.651508609637212 0.695600107754067 0.619733108045411 0.687952452164842 0.665578670611331 0.535186822067616 0.510140247666151 0.691642004979722 0.556080577496335 0.717223105599959 0.514342655144558 0.709050190500624 0.723416469793918 0.604232685997934 0.671612386972615 0.694910317889747 0.692279135919158 0.640814303007925 0.782672780723794 0.660205460963236 0.583039508176513 0.577586480078956 0.577093903315183 0.563131658150176 0.657490070145719 0.602721137733095 0.681835905871387 0.709208326131519 0.560730329185647 0.601682805816775 0.752803828535856 0.792145971963435 0.614751162947414 0.612987779903458 0.702521006665959 0.65638611312194 0.614112424084234 0.688354018179951 0.632062946425232 0.603285635437706 0.722788458632453 0.653673293936978 0.72505150697737 0.705656942235006 0.589315419058456 0.620580234037559 0.651373538331714 0.701741975932902 0.681953923763719 0.737471993381948 0.666545112386088 0.534670157489137 0.730040821845826 0.657734298411731 0.626240310294607 0.689897093710641 0.697059956764591 0.559740087402128 0.658274370465038 0.652173674504886 0.685538604312257 0.608576084574154 0.673983720345479 0.683011367091137 0.55012930933381 0.674433381277229 0.559717657662285 0.693429270301023 0.69708330287936 0.533230543714428 0.617723725229957 0.73361013175292 0.549934937462429 0.726132113613454 0.645266523009393 0.718742941717137 0.675011858076564 0.6877194998387 0.618957266523691 0.695964554747704 0.677475639877519 0.757733733431567 0.734668236191999 0.654756155617732 0.58734114993748 0.675808545360095 0.555183550369395 0.612588252532837 0.676018187297415 0.701688650231848 0.732840090633517 0.663006814921211 0.612257839773317 0.550809148685115 0.812281648263279 0.616619010228024 0.757614849901931 0.659188249861386 0.640165505047972 0.676204752867641 0.650061646958723 0.627778688904027 0.744430053541572 0.547436537129425 0.595155344429126 0.565479357428654 0.618675876173963 0.637912600889613 0.650075952760109 0.59286587569625 0.569384078623132 0.62742109362922 0.591689815247453 0.711423139117786 0.643469666024468 0.577331313002368 0.699079750614674 0.726410864351031 0.611282613872923 0.587071037452359 0.625771779719783 0.590249223062444 0.498841134411941 0.624510196813405 0.655508201267318 0.653299493109277 0.60695786948668 0.619210555715487 0.644879872149096 0.524238580250709 0.782229575361475 0.56267216549039 0.699114576920062 0.610961487994944 0.612772463330768 0.645773472782231 0.687487021939739 0.703845709538593 0.588097459934162 0.703136877468247 0.625662737503325 0.63777547626921 0.664304488978471 0.634079824796568 0.712130080846116 0.673763710671727 0.624785206760302 0.569627335066758 0.701415871215531 0.7228285877955 0.585680349154873 0.579435115558013 0.727045795835285 0.754506328816005 0.740102297204689 0.486257761670114 0.712544285864987 0.734968199514963 0.660049225626579 0.580358890825096 0.706859191558934 0.70853205690605 0.632582208482066 0.706052352828802 0.570033909501795 0.607108868769289 0.720420648105162 0.55939602671317 0.632555249518465 0.693924739143913 0.603787761153998 0.694703241781151 0.741929549313609 0.626197713157508 0.62212985877268 0.582480836167829 0.749274774123276 0.61434575059728 0.562930175497065 0.556486307050161 0.609782809402132 0.79064737387641 0.642856081903767 0.572604043090027 0.629476731101928 0.668393439937927 0.617201523494119 0.641298851760466 0.577181158691397 0.591262119211936 0.719605412285845 0.596678577193572 0.674280369954045 0.722728347358748 0.698697261096459 0.633616399765477 0.657495691448089 0.695920234317212 0.541552301566408 0.668612474730429 0.568462718270175 0.641838304177728 0.618400792954128 0.785840636466691 0.658583612325297 0.701452929842602 0.583798459402321 0.706968281125961 0.71511343602976 0.611214783794317 0.734450635826019 0.647088729817153 0.639033026933838 0.662320520147719 0.617699551523104 0.691035675381259 0.582399094768402 0.651214824409267 0.621524224329519 0.613191390370539 0.691841661348932 0.598372114217968 0.657342178644296 0.678865276796513 0.675021988845115 0.686543053227619 0.639437593383314 0.533326935330524 0.6107107983721 0.588666444358067 0.648397052488307 0.56832680781527 0.694373976892998 0.613533791880411 0.535464719507779 0.572950232211076 0.681684703845882 0.649232332466371 0.629569203135211 0.640217395160302 0.627687144687134 0.707844565645716 0.771759565389547 0.670077259315609 0.681360705776788 0.583332085274568 0.625605476095854 0.673684030011055 0.744369174349749 0.693419944922126 0.658195394532831 0.718226697697803 0.632520896580336 0.766021919251624 0.659456058036088 0.606745273762467 0.626717491206921 0.562960296579361 0.68090135817815 0.704022095633789 0.63023349282713 0.720295930394686 0.558405496333071 0.587819567972457 0.636881424618309 0.687501706684488 0.710876560810023 0.590107756660622 0.650605634694366 0.546637488916157 0.603089319804264 0.781581694675175 0.591003983836381 0.618284546969403 0.727827846895815 0.533475202086372 0.513504745334569 0.522548183351508 0.617567256412604 0.583502964590536 0.660575209856206 0.678639658105949 0.697897079450659 0.640297625734491 0.785782863604544 0.615135094116318 0.532271576226303 0.72175221166584 0.698541646488547 0.689880715167391 0.652899629941789 0.576253790705759 0.498609841369953 0.742423224212569 0.66220094670681 0.69979991475985 0.694257401099937 0.687675920348141 0.742964480752051 0.643467996755799 0.674565597449318 0.747048515905861 0.684132711385818 0.770631982901371

T_cell_co-inhibition 0.632825013943354 0.591579630765034 0.591739424085076 0.598232273414216 0.605557299654851 0.654171234301819 0.739497619849214 0.659572612913445 0.694321216128827 0.700867945330613 0.694403033169113 0.575998058237289 0.570365923303955 0.569644975982436 0.521298796085446 0.642057370950315 0.699201381332235 0.604846112519529 0.555618752201022 0.562545593780232 0.588370384553666 0.728081169961223 0.625962366412055 0.686071438293218 0.667668648184117 0.646694596747191 0.676036690607774 0.551008342663562 0.622304922732368 0.67178121097649 0.674494169783881 0.634949684928676 0.618839628976868 0.67260199770589 0.62702025946562 0.586934062654665 0.603951234119935 0.615805691725661 0.648530622042234 0.623016791312097 0.564747896835972 0.633962382141099 0.536854381133171 0.621561570433809 0.586946519504608 0.625201450611195 0.676228612847513 0.575418420241967 0.636114172022288 0.60733883033477 0.652705053637031 0.579862090033705 0.62982073269267 0.713906490837452 0.574168820890309 0.660620616746537 0.570183544348588 0.657746088208551 0.582138155134275 0.603273023962062 0.712716629803977 0.632912656969744 0.622283611176872 0.662479574657766 0.725754772882336 0.640359248562587 0.580887818698734 0.68711478722302 0.540479115094478 0.664424131996991 0.621140619519021 0.610633413717398 0.628103979541685 0.549537730811097 0.623911001704357 0.608182463832522 0.609401810373969 0.665020146206707 0.570477177257338 0.685333261065905 0.610377011087717 0.667771180312811 0.633833868299304 0.674257426336723 0.666690710852211 0.572957292521323 0.625522306092778 0.599285409052837 0.569359187472279 0.663535712321867 0.574739702901069 0.617046838265056 0.602897023891375 0.668245068538269 0.65453456321936 0.591847735204305 0.60170556295308 0.658858240774869 0.647416483466089 0.664588055628494 0.687248039020667 0.71432674804593 0.619612048044998 0.664458029923305 0.600551018247266 0.636286548960994 0.647296745043215 0.676025760059269 0.670533301984229 0.648476991718845 0.656743011937525 0.610731723246722 0.622018048251308 0.650538141116098 0.648188779691799 0.632272965813219 0.660639671160709 0.657991182409095 0.622694129412864 0.658698070305223 0.652768343401325 0.697545507298199 0.623461763796138 0.658858660730014 0.650353753921489 0.651942637277344 0.676669466721415 0.609397222024024 0.643388997137292 0.626270706000777 0.612124042065993 0.653214239605037 0.697300884894809 0.637646138857248 0.671712350267932 0.613934604726181 0.593460893697587 0.631058846784102 0.591807293688782 0.604321986940241 0.600168370152883 0.678465170578869 0.65342537411724 0.586737416408565 0.650268338661845 0.574125790606252 0.592745647761602 0.599203176753355 0.635730938802016 0.654355048739204 0.617218316271892 0.592735892381372 0.62960526812779 0.64516078829532 0.609151240025772 0.684372127349984 0.648078824013974 0.671294979010789 0.674074625755283 0.583779372642234 0.596137498174002 0.648292480523002 0.602957369427218 0.581167582328684 0.64407123065355 0.614382690665058 0.61618084137247 0.64636778533053 0.652478216782959 0.564076602926212 0.613422570570253 0.713483137706864 0.572375155538678 0.577393020687222 0.712854305228753 0.617282955203995 0.606111656251578 0.617771975645114 0.588792720865018 0.601291284389345 0.671989570326491 0.560799297860956 0.565902454291883 0.675749701101924 0.651138455341396 0.597167194472421 0.662639660652566 0.631815630276759 0.585022359194207 0.625756939898261 0.558561321359103 0.658757674952773 0.612163062142321 0.658360888849043 0.655708041319602 0.675398112577112 0.611107550995755 0.615831676965954 0.586121096619745 0.624580903779279 0.715085999345204 0.651284646029014 0.690887281336389 0.672599932555672 0.584264039826724 0.601224692592737 0.622199523792214 0.652478287771058 0.604013380096325 0.759887295466441 0.564907597881378 0.699186236933324 0.588493310704806 0.679620691809438 0.730292869040825 0.646108955338003 0.578860012813402 0.585669555567898 0.68191628914262 0.526379210433208 0.706002846650334 0.595324858154361 0.606855671726177 0.637529188304257 0.573059743872418 0.770308665547568 0.639606862465182 0.662701925729268 0.664432387798964 0.668765372314406 0.672216200900827 0.588568653739138 0.576020057160815 0.665555997406702 0.628936415578927 0.68454568771578 0.575743225939472 0.597813392918914 0.590566492498557 0.636683009734061 0.664360952153327 0.590755470353903 0.642612943085223 0.609208361431144 0.642900226403872 0.676180949917182 0.595668636865473 0.580075366229523 0.642200682205727 0.783601480772968 0.695131682553345 0.673477918580133 0.673264290671137 0.683802470983073 0.652004032639214 0.697055129981322 0.722132618759909 0.625509000262725 0.591893750696763 0.71062167969345 0.600851812211201 0.662464226189519 0.556335873633468 0.625036128942215 0.630144026982925 0.650061185491797 0.546650200175009 0.563164195722946 0.664834046349493 0.713489370482685 0.682888351713768 0.612370449112733 0.610295443691796 0.647774599529814 0.559980369135054 0.684693527284203 0.669993959913746 0.667227749325099 0.57465211026798 0.716158862467455 0.695493606774872 0.659580336196175 0.586072102514789 0.668832446290592 0.694709772131989 0.738398165042137 0.655772192540005 0.598383430771027 0.64969217280604 0.617859106873062 0.602993417494547 0.608093828573644 0.631338245662542 0.565158990670903 0.644407832802478 0.617374137669252 0.619416703874991 0.667070218250353 0.606875458535486 0.588869632176493 0.623251086430687 0.667840452108013 0.60257056233487 0.608541438747682 0.648507557586645 0.597146222326546 0.699065078174239 0.572050277113234 0.656672662991308 0.589267078250901 0.709758932441028 0.639294609845942 0.640439896401448 0.584286352455979 0.67693624406768 0.680758143116475 0.594770285822628 0.602497789513753 0.610378345549083 0.658407707218328 0.627531158870549 0.677974942242841 0.591303455290144 0.593535229672306 0.690633347599307 0.600306251819783 0.650637157827599 0.680461134987076 0.658580148205117 0.70270000758255 0.628569192474241 0.680093663382478 0.661731885174888 0.742314489638839 0.652198820554686 0.62209576654944 0.620667626148133 0.766962714115526 0.659895410451373 0.570569343035555 0.574848669364889 0.590907574177719 0.605475332040543 0.716755755311008 0.728477409537849 0.773576280350567 0.601422891996018 0.612716369535756 0.643286754903821 0.671823866975894 0.651070513320221 0.551749194942938 0.590896707138639 0.638499286999697 0.610998121333297 0.626520163890621 0.641517832460772 0.666082845870408 0.690209088143843 0.663710714140116 0.578943915570342 0.538552388521678 0.619947196530318 0.722145074514394 0.679658026730015 0.614832909812816 0.616964326964091 0.629887906381859 0.694685629999812 0.594999875912039 0.703866977114096 0.718618357738606 0.649997781449516 0.550946636173264 0.60127294047589 0.616481027721698 0.643059784409209 0.678417285888582 0.658045747573146 0.637553254260683 0.639113755474062 0.766965721098026 0.558454476316103 0.565285263580564 0.628660815392686 0.658644007656322 0.609267796253955 0.675839612573889 0.572234924476563 0.692869944913025 0.572667385336597 0.632369625426718 0.655459359943889 0.613078811887456 0.663604743988664 0.688312796667717 0.633306716740798 0.655129160308461 0.664321783101488 0.72536361663424 0.630213974253205 0.602242537499766 0.645427230629912 0.703032400389937 0.553566559110614 0.590697348751461 0.66303537003481 0.644854315472108 0.573193255398837 0.61292002151294 0.626460882161433 0.582680119394553 0.573306871443867 0.663564475047396 0.593754596431048 0.652499958685311 0.55542521312606 0.625364740169493 0.714166826122555 0.618451932263771 0.676468061146664 0.643883065644171 0.618516525682922 0.622314442948669 0.629018229221022 0.667183478947323 0.581818018752435 0.607670873522078 0.586784037477397 0.577923431204109 0.656139625261985 0.634892449351453 0.609496052494934 0.678656433630238 0.616715698370916 0.608196562751339 0.713818190250883 0.686812532387154 0.590300327792048 0.616942375204251 0.677105382626548 0.628299582858282 0.618558066485084 0.642481456424258 0.616536634120034 0.598352762276147 0.681053793748267 0.633802501685475 0.62898623004537 0.680420610884396 0.599272068327676 0.625345355791661 0.617650949022698 0.690445243505686 0.571493541907093 0.663771563249396 0.634726938259436 0.517678299735812 0.679858405886404 0.581126825423316 0.602167536250598 0.640018099528551 0.67489923655893 0.554704466161192 0.623853737110545 0.638066691478388 0.649285526254034 0.580476204067993 0.606831391196744 0.661855392502207 0.577723132894698 0.63287983302955 0.552753352426341 0.640930601556274 0.625272166104047 0.587362392358336 0.589285102353783 0.661404688108144 0.617336725581719 0.694500641796065 0.629523847913771 0.628616404052169 0.645514577060416 0.603575414581148 0.604782606208897 0.64005061227422 0.638548792549352 0.731904629291425 0.662905615346912 0.616279682517591 0.601060239229744 0.675241792947023 0.560922294337176 0.592697197667193 0.61482965095885 0.620220342814696 0.687355077755106 0.650020193689289 0.597131234734297 0.605574128476631 0.793814268809068 0.579662113135283 0.703588304913527 0.639092825846749 0.618631278640782 0.65165445554943 0.617037438320313 0.634635611675341 0.699549129070402 0.551465452393964 0.611097140469034 0.583747741072215 0.616528307037596 0.607544794228559 0.661716285296223 0.60986264749541 0.575339745130584 0.606123292047547 0.558553419581368 0.622308333877112 0.595805362394869 0.553796666596511 0.664987601833945 0.693738751177629 0.626048994116613 0.548529137287925 0.619531720268988 0.579134345589508 0.535196827681345 0.592067862385367 0.606499443581364 0.682409038342146 0.593469432950884 0.590991177488282 0.607781707688539 0.590324514293436 0.679280260718234 0.56482631368965 0.648894769009577 0.649332840981611 0.594862919264038 0.632666379305658 0.6345912970554 0.667219781417389 0.602042205884867 0.68950050726525 0.610903441085441 0.678208607746308 0.611003123719742 0.589518788371043 0.666315423276287 0.625690600957382 0.598469068056245 0.612324749505947 0.644682492098042 0.673944393945952 0.609654649564858 0.594432774901072 0.669387416779868 0.726694974944359 0.648999951829874 0.53908705565189 0.721770491046126 0.64745569291958 0.660195211803389 0.605104225135427 0.666441028523806 0.677964244093597 0.606128069032519 0.656122433088787 0.592768065085394 0.586724591723617 0.648601217803387 0.51401311840579 0.655918809660017 0.636905218385525 0.651503310059312 0.625456569627406 0.648998550999217 0.591626648024874 0.609972440030637 0.583803797745196 0.67131284890428 0.607372031631921 0.56979655925823 0.559772857973212 0.599969152718773 0.658909159684438 0.571428446640793 0.566520745425405 0.636056352536242 0.603141242868801 0.575997820719542 0.587295417837263 0.560116363065002 0.563641973625319 0.658980163843132 0.59096577256269 0.628041550463934 0.609363309095671 0.659471486534495 0.627034034931941 0.64348543584865 0.633491966843172 0.609614285797207 0.590819216328986 0.610732549306078 0.605521008653789 0.595480875192925 0.798946338196931 0.641361828154809 0.681424493729164 0.607805502036383 0.644924836229425 0.686782986483691 0.629237340417295 0.686681633985617 0.599482964233014 0.663745264939743 0.657047324668399 0.61685177999262 0.639662841636661 0.583467967875475 0.653013142819832 0.607620180155826 0.583850362134142 0.659259146038393 0.581280330783503 0.731442893394208 0.707740405228552 0.626161880952037 0.662702705230338 0.552740846897263 0.586684657186196 0.63181421743708 0.609995954029444 0.614385341785983 0.58921892284075 0.689602120597643 0.690560518581536 0.513354533664004 0.546323136967696 0.655067310663129 0.618484303215266 0.622701748923741 0.630894118295808 0.589808854155151 0.682788584607009 0.69138295371049 0.634461522447591 0.653722310357206 0.595467983300857 0.626888389433856 0.636241918558561 0.707751510313754 0.661413034461768 0.640009163186413 0.646044512671523 0.636400052694592 0.755656818701522 0.638098641027545 0.643025474794432 0.60830725306698 0.568212738896481 0.631555909918474 0.68827680968421 0.62625890693487 0.661332368563957 0.567305039543179 0.604675023259911 0.625283695255788 0.683871902260593 0.673283283097329 0.596070805261153 0.614726582512251 0.566173923097762 0.600051765474146 0.729582325385687 0.599880392664141 0.569140224857427 0.709006426083171 0.56370122092257 0.583413327556281 0.622914941765214 0.626496384821647 0.577229778849595 0.570911764658157 0.598437011921551 0.657514271894 0.635251022337296 0.777149225786227 0.636906925128507 0.566100584642085 0.707813241195725 0.644759439766115 0.743135044809917 0.656162152088985 0.55048027341105 0.55866151598083 0.723683825821984 0.641208528215218 0.671695689754119 0.639321580832677 0.676500240303163 0.714276877754809 0.631219474251558 0.610904697281936 0.691120247580769 0.678697174366886 0.672501541061786

T_cell_co-stimulation 0.532874460170186 0.523908672126208 0.497659418066693 0.557744735488515 0.576071781116501 0.560191930161178 0.661662592254286 0.512931866735092 0.60049839555744 0.690122487718033 0.639898458220554 0.483248716536255 0.443174436516719 0.479887710655689 0.435376533536689 0.540066252996162 0.648814037272874 0.522252275733875 0.459289256664429 0.553342414008385 0.543989045377606 0.672451938175166 0.449637148947532 0.630029238410403 0.62814701033856 0.559029910914128 0.610723410440679 0.464235333851788 0.499665142064755 0.574990990879646 0.612537100395768 0.546048213802325 0.458754849153899 0.622652086317752 0.60851387696801 0.477883162626808 0.470211565341589 0.505157218269099 0.514740230928163 0.471716829246317 0.491253659642562 0.504970557834521 0.465865765655288 0.560193106994429 0.524016089167102 0.464923865201145 0.62244477407579 0.457776285695859 0.549036923852688 0.535681157382616 0.537990690619685 0.433305763999068 0.519192733858173 0.679942755180104 0.495206043214039 0.546299291720661 0.487850355446436 0.593546770064398 0.500992163751931 0.570932416037338 0.664146797627525 0.525360122910709 0.493534968773976 0.550558354135296 0.713847472916294 0.575844852278129 0.485833762264497 0.642434113821961 0.473764875734962 0.633607349596067 0.494308858333067 0.527935993811941 0.562189462325011 0.454757522057413 0.558712193664272 0.462334152063081 0.481725266785269 0.535956771090054 0.510111741657481 0.631948436334573 0.527356120733449 0.58222696531449 0.560326482936376 0.571788369665013 0.627588033571693 0.506380913543275 0.522582247526451 0.469817890669154 0.504955145890317 0.597348826148656 0.489230567439478 0.478985780734012 0.564805561227023 0.563269381147463 0.58722467572066 0.451320725775773 0.470030424323964 0.600380098439241 0.558374399565304 0.592794143806305 0.612249590181171 0.654662756106743 0.574313693605218 0.584213920058983 0.500833966581643 0.506270902768454 0.556468827561587 0.604361556512214 0.58700042562475 0.605716669538278 0.528285938202117 0.479107765012558 0.509542623056038 0.620723805206276 0.535601090629843 0.604958003050922 0.616796476000664 0.58649672048948 0.479447902780806 0.646334336639403 0.528519512837222 0.651504752956573 0.502648494406564 0.588346262332619 0.500547591821547 0.614104759031066 0.607037233914501 0.486051498281519 0.511846342039336 0.452219550468343 0.49661228637255 0.55584676138389 0.625253083478105 0.627967487005344 0.659427539565287 0.555638911760466 0.464028052941784 0.505538806794423 0.485307227517733 0.523545543161436 0.521935106386419 0.662067808553746 0.614726068303132 0.539098765848908 0.549756400084577 0.479814037871362 0.555146257011287 0.518272605408161 0.497490482324693 0.527469316051606 0.50412570792285 0.54766619477803 0.527771184287036 0.56830358983628 0.483603828940314 0.609991240235511 0.571610983056417 0.524327975317984 0.561530746865315 0.452278224494669 0.462664569715723 0.462182996755652 0.453113632504954 0.49801241745485 0.575318857516428 0.576021588845755 0.450669185467887 0.503040477007396 0.558162144228269 0.433560112509694 0.475487415756805 0.67269440594305 0.43256296389099 0.510585809426212 0.626214651863961 0.566628930257126 0.501627304500739 0.537637044782146 0.468168637810023 0.504778913105134 0.609629906096939 0.47922755283112 0.473174426817404 0.492062611946216 0.598646074611424 0.424442242659948 0.583413573430462 0.46275221726499 0.524409718902995 0.521361387543615 0.41666133517038 0.630307294882171 0.485471724394928 0.579465506972469 0.595036380017036 0.62782480072069 0.523801192782214 0.530437402625007 0.512276475105382 0.552918297214581 0.606236243131342 0.588439914361595 0.569287983450933 0.626648060946771 0.475600273873462 0.509593509741397 0.474258752226903 0.489737664541467 0.512688282186896 0.756700804290298 0.483978518543667 0.649267718208657 0.514674473260687 0.584725763662138 0.667751302343849 0.546227388255916 0.458711391254831 0.432647576289022 0.547055720938184 0.440884614880564 0.617985352372899 0.456600523230963 0.61223966669473 0.591142438207709 0.509883100397166 0.72060668046194 0.492024009172229 0.617015578895592 0.623134935796529 0.626915127401144 0.524155019935087 0.529436302482682 0.541368234498702 0.629800570560625 0.569131745667325 0.618503590238609 0.485396302035977 0.479085569954534 0.420204096165271 0.461174917485499 0.584675108053355 0.539673349688206 0.541281508609991 0.538818754011023 0.528547136110261 0.658403673910604 0.538405174736244 0.509813188359374 0.526803540206477 0.780081472757383 0.629806359360624 0.579687065648386 0.630980837867784 0.666054141761322 0.520899132049962 0.614342128283409 0.736639689927281 0.561274366864414 0.506801371213972 0.562147699840229 0.548430927163074 0.540173737647908 0.4559963898748 0.511780990073009 0.613920543094357 0.464964695990511 0.498904979042706 0.405603847026551 0.632894405559355 0.687120598056486 0.623871138030505 0.566643598379231 0.500413618092805 0.481635361253045 0.478644368002369 0.635550433793619 0.603354504942179 0.616128345706962 0.511471506539711 0.643075378547894 0.672220246465365 0.55612742717269 0.453253683666405 0.604841872288237 0.56538590620231 0.680337138936868 0.558896460902141 0.485952287142063 0.58090236576512 0.50546694448394 0.510194745656152 0.528847521497633 0.541798477890306 0.463824175239773 0.553870176740722 0.58281842945891 0.46660308526039 0.558461221452636 0.507023251795809 0.481426589538522 0.592969770625998 0.645008946587807 0.482951950240947 0.528162636038823 0.569906899244665 0.512026965417156 0.627834847433019 0.452768695148995 0.654060279620791 0.48985116831268 0.678420123441537 0.536879516936973 0.546992603731012 0.44004524154599 0.545658880867036 0.599075307631882 0.391571001311665 0.496961991787159 0.496804282623469 0.593533507033496 0.498875509914397 0.585307246361783 0.466755805139116 0.517680088745934 0.671316649933067 0.481973382260706 0.527985604023111 0.602236619092097 0.598759761638142 0.654380414593503 0.561162259383068 0.677532480332175 0.546024926658017 0.735232210169767 0.527113063199844 0.583682300976135 0.486465566452102 0.697341097196175 0.584553324189537 0.526630925886715 0.440426182463163 0.51053633535581 0.479266568409119 0.630624765021594 0.649786386359234 0.751350995697477 0.469252868544479 0.464537680233 0.535642508652669 0.551447656182005 0.550245826914384 0.460910053879781 0.452164225925051 0.525954225979261 0.560655019977664 0.539159911686892 0.594530334855363 0.623813233506426 0.617608765206786 0.535950829618321 0.482379258210423 0.571544301521727 0.582020504481102 0.70263065545207 0.630657925088133 0.527420761767978 0.502447979361603 0.512730751194406 0.639752568309151 0.507529268430316 0.638287506343602 0.727483892209314 0.606334594203983 0.478531039661258 0.542537886824848 0.514214638212327 0.539724030600428 0.632874117553953 0.600288893520013 0.525058389771527 0.560996741974026 0.721913043891214 0.4459316502993 0.466171832914973 0.49569451511886 0.512230151924574 0.474575805151956 0.600833599809811 0.468704253305634 0.618417227847836 0.50474933311425 0.510024426846934 0.61467677293204 0.48346378345918 0.624426878684046 0.684791358054251 0.530710314158202 0.560519202459216 0.534059100177523 0.573033595872311 0.492205157439104 0.480722901578107 0.590144828440152 0.640259694617002 0.460451749078554 0.556207728148628 0.539936268135098 0.581449042797979 0.518350323698548 0.574724445972996 0.55674916503997 0.458668062806448 0.425828398452123 0.540723458177987 0.491500053169471 0.604865513696413 0.484715442410368 0.53403267124332 0.628029562836014 0.574919992607112 0.690688701134746 0.527131917589926 0.508892566670204 0.555861028903074 0.540468757936115 0.513174214978667 0.487451360861754 0.508657449179291 0.52003834530399 0.472924967481921 0.556648056253229 0.45170289617476 0.542469965494944 0.619348778908208 0.51616313347903 0.453078923208787 0.648968796580088 0.610730531227671 0.49502262885998 0.514349724912963 0.62627980084529 0.555213030301037 0.579895626144223 0.57725278900251 0.511076522437093 0.524319701518106 0.606421879532086 0.496146749164983 0.545749219149013 0.547833065125506 0.505921907070152 0.497646056908886 0.554304947297341 0.63262446969944 0.556190947248789 0.591689089443289 0.511937846643279 0.472056642531659 0.551282583540626 0.555491609742027 0.49500104311655 0.495107229833563 0.563041562890377 0.461399227164845 0.564967870149033 0.49305366092773 0.580755134342719 0.45464738532606 0.53269507912505 0.56083085409626 0.432712551930189 0.509006658978465 0.488405767624056 0.568331263611254 0.556242753407186 0.533428631940829 0.533210378242613 0.554888693357832 0.479730791441449 0.651823540437564 0.513374282768595 0.551231660800241 0.543764231530964 0.539605801851798 0.525520025576335 0.519687838263307 0.599209431983296 0.695155075102287 0.56489345043273 0.503312021016418 0.512947047766647 0.532952885039319 0.429541069912081 0.480805349229062 0.546514770434204 0.506487505002565 0.592196337818922 0.540671936415414 0.479512236329179 0.461931201823965 0.744068634455455 0.477887248448555 0.636153748983995 0.550057820177389 0.484240418662535 0.551162468914708 0.553604942719614 0.522949780736993 0.692433015405937 0.530186353032597 0.460982034099191 0.48485576875657 0.490832012776783 0.48316237933075 0.518834723987871 0.508140584342533 0.506110072017251 0.543726570525271 0.467743313205136 0.560919775174191 0.507099940503264 0.548676288887476 0.580694822906321 0.676214693191903 0.457280629116449 0.489810251480608 0.489632590137968 0.446754635230697 0.428327832530221 0.531952632823883 0.521993736223059 0.676025094599684 0.481769873423206 0.456385561071016 0.551554754669202 0.466009579667383 0.646301806794132 0.462335871382855 0.589978736399761 0.499987137552834 0.572012842935281 0.528471363515298 0.574910501775006 0.543253217196444 0.511141253549774 0.645681334085083 0.511631168925502 0.656247266325532 0.558781976014001 0.468249154740385 0.61653119702623 0.522477345955824 0.512023014954662 0.454217635235682 0.568429863405307 0.577720799117384 0.513826383537934 0.557223108939899 0.615567576791641 0.677571618475604 0.600301910581261 0.506063260473436 0.622959311352972 0.606810238228386 0.561064032654373 0.52412216700312 0.559797990114358 0.544888123991703 0.505302783224821 0.637026794948943 0.439367161765164 0.507280316974511 0.550527360907083 0.454413969800094 0.568811030330661 0.554938825844121 0.44173247313133 0.54297898259233 0.644205049392368 0.484098609512509 0.523000330918062 0.498472137143835 0.612846543990125 0.496400086432573 0.496744091318083 0.461256775796276 0.43855563464725 0.654241584557976 0.507215455207824 0.435005540193148 0.522367694485543 0.458649744012143 0.498580259334138 0.507550067317356 0.434166504183484 0.460838711461579 0.536791419071819 0.470083191340719 0.505905423731304 0.542750760092316 0.629376839725188 0.499363380981161 0.529329706925652 0.526527610671953 0.476778840155671 0.544252809345162 0.492933082279723 0.538375853405502 0.461426285926502 0.779042495825902 0.485637317530286 0.630915300669298 0.523403244535851 0.618209545326674 0.600785025216611 0.485499246410551 0.680182501924528 0.498249414487534 0.581044495128718 0.631718227974821 0.49685369716462 0.540724360597097 0.505180568650785 0.517489782985998 0.452797726963161 0.53216069941657 0.593617486201195 0.495545071068651 0.661045487778996 0.611658728165238 0.474664694663262 0.550690794624434 0.494611056469184 0.473450016073623 0.515028222072864 0.501538067741452 0.481447983644334 0.482213626268477 0.576525840066284 0.538816858843347 0.427889143851282 0.456621019959457 0.563772368523383 0.500200183291174 0.54763991228701 0.596802033289243 0.495941865860599 0.644724155401375 0.644004513914204 0.585893449327798 0.56255151589327 0.527133106285307 0.501190681646602 0.470493930471663 0.666523914246894 0.620552425140295 0.508515284565172 0.545192172165335 0.564676168734289 0.75282992088706 0.527292410415874 0.42931412840272 0.515817237603635 0.449670567596778 0.532910090974656 0.620994127372064 0.498601134751629 0.541530857308286 0.483879654346091 0.498742751260469 0.47589253340723 0.609104009527119 0.585377453343908 0.516029478061784 0.508826559887262 0.461460431232484 0.571986610442642 0.682354221210759 0.469767855135506 0.472456679168227 0.632426101068265 0.447319884836433 0.497177425346585 0.450197384343136 0.547603969530273 0.457190178354506 0.502353048330226 0.511372074018443 0.565710593981068 0.493242157703923 0.719304722092134 0.496362076228132 0.437148030765491 0.673043076853612 0.600020258033564 0.581317982029499 0.504312630018728 0.484862090262219 0.448530179492959 0.60883092745936 0.553701486477003 0.561125688589181 0.551410888873176 0.580366329043622 0.690775872513199 0.49656083460351 0.477009138507042 0.636155588955034 0.602392044431636 0.613129803910532

T_helper_cells 0.89143974481485 0.862953177603926 0.868439036135441 0.878233787186256 0.855053541318543 0.905383799773138 0.972411016740019 0.977677440930274 0.930937926241761 0.961957962665422 0.961160019606292 0.89427244267476 0.824153196353752 0.807376443535554 0.879709981845646 0.941111700245663 0.964531329031115 0.800573978956475 0.811346210254724 0.84705416215077 0.830576637979745 0.977817080965621 0.91158780705787 0.947535141871656 0.972550656775366 0.88792879535468 0.948033856283611 0.827664145813922 0.925811142086854 0.97953265854275 0.965349220666722 0.944143883870355 0.920804049390816 0.959205059111425 0.96469091764294 0.846655190621205 0.820961424117234 0.911527961328436 0.966805466749634 0.901553673089317 0.929501628735328 0.92698810809907 0.838097251312041 0.921562095296989 0.901832953160012 0.921621941026424 0.960242385088294 0.91691407697756 0.960521665158989 0.883719645717772 0.961419351100509 0.867740835958703 0.953160640438519 0.979213481319098 0.85996089113219 0.928743582829155 0.843084395431601 0.96798243276185 0.871949985595611 0.878592861562864 0.987272706216306 0.895988020251888 0.925232633368985 0.932753246701281 0.971413587916107 0.951804137237999 0.841328920701516 0.96333441444242 0.850405522999114 0.942607843481531 0.86686309859366 0.900675935724274 0.929800857382502 0.82882116324966 0.885275634683074 0.847233699339074 0.907258965962093 0.973727622787582 0.898481592311668 0.97137369076315 0.886213217777551 0.964571226184071 0.953619457697518 0.922739061309205 0.961239813912205 0.848630099692551 0.865646235428488 0.88679172649542 0.921582043873468 0.951664497202651 0.83131473530944 0.872388854278132 0.865127572440054 0.9547365779803 0.92902286289985 0.84637591055051 0.825250368060056 0.940533191527794 0.920065952061122 0.943904500952616 0.967344078314546 0.958826036158338 0.919906363449296 0.973687725634626 0.911408269869566 0.955674161074777 0.918450117366384 0.973328651258018 0.954537092215517 0.951465011437868 0.936343990467364 0.952482388838259 0.921482300991076 0.924414741733378 0.930578851865153 0.942966917858139 0.959903259288163 0.962775854301029 0.937939876585623 0.97568258328245 0.958407116052295 0.968062227067763 0.924973301874768 0.894691362780802 0.971892353751584 0.959364647723251 0.97205194236341 0.917692071460211 0.965209580631375 0.892516967944675 0.944024192411486 0.923297621450596 0.975662634705971 0.948771953613306 0.977438058012535 0.892217739297501 0.908914697809786 0.90582266845566 0.858125622096192 0.941291237433967 0.884597383082814 0.973488239869843 0.948033856283611 0.907797577527005 0.945181209847223 0.882502782552599 0.949869125319609 0.885754400518552 0.921841375367685 0.959544184911555 0.885155943224205 0.874303917620043 0.928963017170416 0.934867795807974 0.877216409785866 0.96832155856198 0.928085279805373 0.944782238317659 0.959863362135207 0.893354808156761 0.875800060855911 0.914919219329736 0.881505353728687 0.854036163918153 0.921163123767425 0.922080758285424 0.857168090425237 0.937840133703232 0.947634884754047 0.814518033914763 0.885874091977421 0.987711574898827 0.850864340258113 0.924993250451246 0.972470862469453 0.907677886068136 0.958207630287513 0.895928174522453 0.85996089113219 0.873984740396391 0.942567946328574 0.871411374030699 0.934289287090105 0.935905121784843 0.959703773523381 0.869875333641874 0.952143263038129 0.904027296572618 0.855632050036412 0.947395501836308 0.83652131377026 0.961319608218118 0.934788001502061 0.942627792058009 0.959703773523381 0.947674781907003 0.889544630049417 0.890382470261503 0.891719024885545 0.904805291055269 0.976979240753535 0.86494803525175 0.960342127970685 0.977936772424491 0.835424142063957 0.869177133465136 0.958427064628774 0.986155585933525 0.938897408256578 0.967563512655807 0.873047157301914 0.98292391654405 0.838974988677083 0.978216052495186 0.966207009455287 0.924733918957029 0.801671150662778 0.860479554120624 0.961160019606292 0.838974988677083 0.959803516405772 0.832790929968829 0.951504908590825 0.900556244265405 0.881505353728687 0.991262421511954 0.963972768889724 0.959205059111425 0.88679172649542 0.94827323920135 0.984001139673875 0.84807153955116 0.891599333426676 0.981826744837747 0.909832332327785 0.976719909259318 0.838476274265127 0.867641093076312 0.822896436035623 0.972111788092845 0.91386194477639 0.903049816325184 0.925671502051507 0.849866911434201 0.945879410023962 0.985656871521569 0.915577522353518 0.835144861993262 0.967224386855677 0.988848643758087 0.954936063745082 0.957250098616558 0.962935442912855 0.985936151592264 0.950946348449434 0.937022242067624 0.988848643758087 0.950726914108174 0.881605096611078 0.980510138790183 0.887609618131028 0.96117996818277 0.775618309782199 0.879530444657342 0.83482568476961 0.915238396553388 0.851203466058243 0.824193093506709 0.94589935860044 0.992160107453474 0.960581510888423 0.885295583259552 0.867341864429138 0.926888365216679 0.923118084262292 0.959763619252816 0.958786139005382 0.958985624770164 0.872209317089828 0.986335123121829 0.97919353274262 0.93955571128036 0.826387436919315 0.934488772854888 0.979831887189923 0.985916203015786 0.929441783005894 0.84569765895025 0.970615644856977 0.959703773523381 0.864449320839794 0.902491256183794 0.90287027913688 0.851801923352591 0.942687637787444 0.927047953828505 0.857447370495932 0.917572380001342 0.895668843028236 0.84908891695155 0.950048662507914 0.969019758738718 0.90117465013623 0.919248060425514 0.924594278921682 0.856050970142455 0.979492761389793 0.879311010316081 0.959225007687903 0.834007793134002 0.980190961566531 0.940513242951316 0.942867174975748 0.909014440692178 0.977278469400709 0.981727001955356 0.82361458478884 0.889664321508287 0.870354099477352 0.956891024239949 0.895389562957541 0.968820272973936 0.879470598927907 0.886293012083464 0.956232721216167 0.888646944107896 0.94442316394105 0.980849264590313 0.95824752744047 0.981328030425791 0.882243451058382 0.961020379570944 0.961938014088944 0.993277227736256 0.928025434075939 0.904446216678661 0.935605893137669 0.982664585049833 0.909134132151047 0.853357912317893 0.912625133034739 0.909433360798221 0.894352236980673 0.959524236335077 0.959344699146773 0.99493295958395 0.846555447738814 0.914779579294388 0.892497019368196 0.963214722983551 0.92495335329829 0.810109398513073 0.892955836627196 0.899638609747406 0.841827635113471 0.939695351315708 0.952841463214867 0.944862032623572 0.959703773523381 0.956033235451385 0.8127426106082 0.855731792918804 0.924534433192247 0.958048041675687 0.96129965964164 0.917751917189646 0.935067281572757 0.847373339374422 0.966087317996417 0.919986157755209 0.970096981868543 0.982425202132094 0.942208871951966 0.925392221980811 0.912385750117 0.914839425023823 0.923417312909466 0.963713437395507 0.929102657205763 0.932972681042542 0.924354896003943 0.992698719018387 0.808234232324119 0.879989261916341 0.941171545975098 0.89562894587528 0.911787292822653 0.943724963764312 0.80617952894686 0.971313845033716 0.908136703327135 0.937461110750145 0.91996620917873 0.932613606665933 0.973687725634626 0.965688346466852 0.955434778157038 0.975882069047232 0.951385217131955 0.984759185580048 0.875640472244085 0.892337430756371 0.962855648606942 0.983402682379528 0.854933849859674 0.852978889364806 0.947634884754047 0.945520335647353 0.8892852985552 0.925432119133768 0.909174029304004 0.820662195470061 0.800494184650562 0.92076415223786 0.848450562504246 0.90536385119666 0.874443557655391 0.936423784773277 0.97658026922397 0.950846605567043 0.923876130168465 0.943246197928834 0.962556419959769 0.913423076093868 0.925172787639551 0.945919307176918 0.850964083140505 0.895928174522453 0.913143796023173 0.839473703089039 0.918509963095819 0.921382558108685 0.94340578654066 0.957708915875557 0.885295583259552 0.897524060640712 0.959883310711685 0.97284988542254 0.922639318426814 0.894711311357281 0.981188390390443 0.921901221097119 0.930778337629935 0.894172699792368 0.894791105663194 0.841408715007429 0.918689500284123 0.902551101913228 0.95246244026178 0.968521044326762 0.825449853824838 0.94408403814092 0.879490547504385 0.975044228835146 0.884457743047466 0.971353742186672 0.955195395239299 0.846016836173901 0.974425822964321 0.917851660072037 0.901593570242273 0.933251961113237 0.928404457029025 0.869735693606527 0.940972060210315 0.919387700460861 0.945799615718049 0.85644994167202 0.919926312025774 0.915118705094519 0.882961599811598 0.901813004583534 0.800095213120997 0.947914164824742 0.918589757401732 0.841967275148819 0.901374135901012 0.94907118226048 0.933690829795758 0.966466340949504 0.942009386187184 0.951465011437868 0.919188214696079 0.921203020920381 0.881006639316731 0.928244868417199 0.90151377593636 0.960003002170554 0.961678682594726 0.903368993548836 0.886372806389377 0.948472724966133 0.924973301874768 0.899957786971057 0.902571050489707 0.917412791389516 0.968680632938588 0.94023396288062 0.897623803523104 0.827584351508009 0.987412346251654 0.895469357263454 0.978016566730403 0.908695263468526 0.91974677483747 0.96888011870337 0.895828431640062 0.946418021588874 0.978535229718838 0.823634533365318 0.892477070791718 0.899897941241623 0.871491168336612 0.860519451273581 0.956551898439819 0.923537004368335 0.830217563603137 0.881186176505035 0.809131918265639 0.933232012536759 0.884737023118162 0.96028228224125 0.942408357716748 0.97217163382228 0.922719112732727 0.867062584358443 0.936324041890886 0.87887214163356 0.828063117343487 0.926150267886984 0.937141933526493 0.96333441444242 0.847971796668769 0.905762822726225 0.893015682356631 0.833110107192481 0.964910351984201 0.847173853609639 0.952661926026563 0.965788089349243 0.93027962321798 0.941770003269445 0.964331843266332 0.938438590997579 0.872767877231219 0.971054513539498 0.883779491447206 0.955155498086343 0.933710778372236 0.884677177388727 0.91386194477639 0.957429635804862 0.890003447308417 0.889025967060983 0.947954061977698 0.949529999519479 0.867561298770399 0.817310834621717 0.964411637572245 0.960900688112075 0.958965676193686 0.80334683108695 0.972710245387192 0.961439299676988 0.94340578654066 0.896127660287236 0.936084658973147 0.943306043658269 0.939236534056709 0.926748725181331 0.858903616578843 0.826068259695663 0.950248148272696 0.846575396315292 0.93151643495963 0.97115425642189 0.934648361466714 0.904366422372748 0.964790660525332 0.866603767099443 0.926908313793157 0.855651998612891 0.950946348449434 0.861217651450319 0.795387349072133 0.899718404053319 0.911408269869566 0.926609085145984 0.931815663606804 0.838655811453431 0.934289287090105 0.892995733780152 0.856769118895672 0.900895370065535 0.907598091762223 0.860599245579493 0.941809900422401 0.861975697356492 0.954896166592125 0.928244868417199 0.943146455046443 0.897504112064234 0.966586032408373 0.945300901306093 0.888966121331548 0.925591707745594 0.933910264137019 0.892417225062283 0.885016303188857 0.9927785133243 0.934728155772627 0.966825415326112 0.863052920486317 0.924135461662682 0.923776387286074 0.890043344461373 0.966985003937938 0.881664942340513 0.94919087371935 0.955674161074777 0.897384420605365 0.907298863115049 0.836082445087739 0.942528049175618 0.868119858911789 0.917512534271907 0.967782946997067 0.831693758262526 0.965269426360809 0.975822223317797 0.953619457697518 0.965548706431505 0.859561919602625 0.829060546167399 0.94238840914027 0.939715299892186 0.952402594532346 0.857347627613541 0.957170304310645 0.933471395454498 0.77232679466329 0.859801302520364 0.931596229265543 0.87558062651465 0.925551810592637 0.852739506447068 0.900655987147796 0.955694109651255 0.975802274741319 0.926409599381201 0.924334947427464 0.819485229457845 0.884158514400293 0.926070473581071 0.97624114342384 0.936663167691016 0.966406495220069 0.946797044541961 0.920903792273208 0.979133687013185 0.889385041437591 0.913482921823303 0.939416071245013 0.87105229965409 0.915378036588736 0.912385750117 0.920105849214078 0.950786759837608 0.808254180900597 0.882502782552599 0.953898737768214 0.965887832231635 0.930638697594588 0.901094855830317 0.955155498086343 0.816213662915414 0.937540905056058 0.980071270107662 0.831075352391701 0.899099998182493 0.980051321531184 0.880647564940123 0.870912659618743 0.829200186202747 0.922000963979511 0.894691362780802 0.912605184458261 0.906441074326485 0.904785342478791 0.898322003699842 0.985018517074265 0.892616710827066 0.817989086221977 0.975463148941189 0.940493294374837 0.993476713501038 0.937281573561841 0.830656432285658 0.80651865474699 0.974306131505451 0.95110593706126 0.953180589014997 0.949529999519479 0.968022329914806 0.957070561428253 0.908675314892048 0.910470686775089 0.963753334548463 0.96028228224125 0.952761668908954

Tfh 0.33798434255236 0.274997082321833 0.246281906412641 0.280033714921687 0.339338565522614 0.420332532309045 0.639703423214938 0.383679566553963 0.530010830858956 0.503224983814679 0.663094562394581 0.28866530619708 0.355478873954955 0.186824961988203 0.405115710689358 0.290325660814573 0.471168079208196 0.270446379578224 0.292938961284714 0.448111402518704 0.48037163224756 0.427952887156171 0.38999314424243 0.45761570031977 0.380171895942132 0.324535205539601 0.507837491417731 0.546001507007623 0.336420531874027 0.372662353569495 0.561259975768879 0.354092657757764 0.266099348034666 0.407094893424027 0.42279712916701 0.413683435184709 0.241796270902323 0.438804350699002 0.272244213696873 0.33428204168379 0.406078559131714 0.411788035270227 0.442382592472634 0.342920499139532 0.441930703135306 0.24536860563802 0.455834877844258 0.352823808490046 0.473258941835804 0.279017311425957 0.451006475013872 0.198441871683033 0.282665752052021 0.554913286476842 0.423871566379561 0.445654740065253 0.457130124714213 0.531794572771511 0.400280551312826 0.396079063961956 0.474201801202533 0.286466982873283 0.349843961021321 0.353170388894215 0.596859703857877 0.320078607158112 0.300420811612346 0.590208900299812 0.340260296287796 0.365137081947111 0.302200926074297 0.318319478704897 0.374108241919363 0.365917456113105 0.32149987850245 0.455176558285556 0.218495720851242 0.364126944004239 0.417787810991299 0.400371777318727 0.370580892142904 0.568330153534992 0.489091258427612 0.383177750739051 0.408887147934736 0.37758432474939 0.527109045877645 0.296284334713491 0.311632761774381 0.41013091083616 0.461370995864486 0.403411019738002 0.356022231848435 0.331664371875234 0.386895606428353 0.251077345169107 0.280203538145029 0.416506565433293 0.464149554927072 0.454153594319858 0.330193343184621 0.483110677102715 0.393552221358475 0.473950351123079 0.330339383718462 0.275212034362141 0.309758766944573 0.255282949602864 0.386575055311963 0.348811544700301 0.297526530149435 0.327652276084968 0.365100800661728 0.449370344265952 0.400357862124976 0.483172820527632 0.516256384195862 0.407823904338667 0.295590136692978 0.31538272786242 0.451409266519571 0.448782087963652 0.444054291692236 0.411611429547141 0.40114891114726 0.349628662193036 0.369772067974075 0.347195983500787 0.315540892402246 0.335096322769868 0.335586029018052 0.406660294081116 0.397511714426394 0.555885539210046 0.406279949638904 0.43568221292893 0.441913055351865 0.358148540232629 0.421605565292859 0.459852045801714 0.305525972760097 0.515429837178301 0.404122852915114 0.291910207803618 0.305652822103478 0.39813465499271 0.310221446749492 0.501002142188421 0.320026930340465 0.362573848220163 0.493711009955519 0.497441556452238 0.322940875346494 0.426441341095467 0.42129677454202 0.453143618529928 0.383427506695753 0.421796232786637 0.424855873634582 0.393728166503195 0.277124745077598 0.450449168731376 0.278914351115708 0.227718693696555 0.329552356068414 0.371409044879376 0.231485644777365 0.347172204733547 0.310581046994915 0.557919068837806 0.405454052780841 0.454364210124357 0.224150682711497 0.42371478946808 0.55904780584625 0.346464030383842 0.296543700960769 0.301666904358493 0.290485916610982 0.306016443314008 0.487717915065968 0.3389026407165 0.499532594162614 0.389622671067393 0.561569838025724 0.380342808959881 0.444118259340417 0.303412119092678 0.479456920222802 0.454089971492397 0.329112383175931 0.587196545578147 0.312152446459273 0.400071615528848 0.538883575259155 0.439758399459912 0.601925596422354 0.212481746290495 0.423509019192274 0.410020751608353 0.564072067520024 0.611863682927816 0.449690549703544 0.505467181478675 0.238151986232642 0.347853119247941 0.324072611700254 0.384693298759312 0.361933924515174 0.74812777147092 0.190590995304072 0.405708659314013 0.3950058309673 0.349553246568476 0.417618664357947 0.449992723482593 0.320015581384232 0.329459301380511 0.338613940064109 0.363682263609887 0.346908955620149 0.379887197552288 0.382361938792719 0.415253560127379 0.542712393342608 0.553544816168379 0.345465542549569 0.390093548978503 0.334570416084418 0.565887110894564 0.307357492202579 0.357792309943834 0.17018939333354 0.437620882710294 0.545692767975355 0.398323602116218 0.358142319451193 0.359305654599557 0.389054478174356 0.221138278443315 0.520127140250159 0.320715911539134 0.447151310961593 0.377255022120687 0.378920911035877 0.562480500512571 0.454002545209434 0.404690400193733 0.340485296433949 0.695084873959182 0.445823346668743 0.341696777266353 0.561286891676383 0.510964855747073 0.266437439850876 0.338725041967329 0.607443978285101 0.383725487248218 0.370468254600257 0.55097034374686 0.315971348518239 0.373085758167344 0.239868308310921 0.245014430280629 0.415051381428493 0.427868212676267 0.311343371177661 0.309238142914072 0.501960275741431 0.517855267706808 0.494016321558438 0.441197129327945 0.338851644731566 0.438880648291039 0.237291718283182 0.432152922424696 0.316205586427333 0.467303706773693 0.473258821655879 0.435583539525041 0.522431128562153 0.336787679607768 0.367207069500032 0.359569702006698 0.302262173134831 0.599346003395874 0.361431202558967 0.369599842450076 0.420796704576337 0.331460505528334 0.319622033985089 0.302647562890315 0.436715091053535 0.318488890941751 0.280964311239153 0.291966946239838 0.410123521003796 0.370181257373091 0.279008802993574 0.398670050492099 0.373801188260386 0.528215024235124 0.255362367717283 0.470437814015512 0.378724129244418 0.29126995365712 0.558903963704975 0.393656452365068 0.351353987468937 0.25536538221041 0.459849415134343 0.373733253764173 0.355870444833788 0.443571033273695 0.359047099307894 0.409527129632352 0.360871702188485 0.304574531552485 0.53048286345294 0.321174245328621 0.381001222230106 0.371720832912087 0.309134260369682 0.362431366805638 0.488867772733245 0.567691751961308 0.35046737544213 0.303970000327701 0.472547618474608 0.417376726677362 0.562498351822471 0.44609721571988 0.337426306748331 0.682428009733638 0.286543087241855 0.337331908899057 0.285365167576794 0.603096218742376 0.419429338255675 0.276798852933315 0.525368091436491 0.360376801006011 0.439166719297207 0.439294630480393 0.42721861161444 0.629420326840073 0.256650652590443 0.179296849510199 0.355397664296279 0.430912430037427 0.311293949712367 0.359538974964207 0.244885439268819 0.539965268251868 0.465552718490034 0.308049900878071 0.355230365808868 0.441956347596035 0.422640458680243 0.3742861225248 0.333861417827406 0.42489977726185 0.487701658026036 0.481434908048416 0.507755917556389 0.564612161359307 0.247394667257939 0.415635932942425 0.552833195213775 0.351839045382322 0.371875425920999 0.655146080578727 0.481731519550777 0.291844328639413 0.302940418579499 0.369796827726387 0.369741821746769 0.427981696053658 0.48369777430707 0.311104882316412 0.364494501475867 0.527852869014513 0.34365145781191 0.266472910429962 0.448154886228029 0.342518926325355 0.443565222984152 0.419836833499476 0.43065226797625 0.509651734216501 0.486775478055701 0.428910962377668 0.389128423754027 0.175406731876336 0.394077868646307 0.580389792336758 0.380245918275487 0.462120846649838 0.332508577447567 0.569400768126565 0.414050371021799 0.292446022451808 0.414963537847893 0.382755864336102 0.397929770726838 0.271420399591975 0.36650368485216 0.350051433655044 0.390883661329117 0.437070170001004 0.330096086494916 0.282818248520033 0.39135778287582 0.416816637469986 0.219763608948292 0.383198773768932 0.399446905774201 0.377520291113155 0.422402632723482 0.407426352720399 0.393205518145544 0.344970922665141 0.328503527964758 0.387778290801341 0.369513421403897 0.183253908288113 0.380434145737274 0.25958488030676 0.469825497036918 0.269591200011404 0.36392644899743 0.361371743317412 0.46026831499697 0.528832649852909 0.255296344765465 0.291675549432194 0.580136825037259 0.428036970622568 0.477202845362723 0.356049163974723 0.474156759540896 0.441617661308475 0.494388973111288 0.46144041078934 0.322397237609923 0.332286297070143 0.411753163058618 0.286870956815901 0.450422227933307 0.315004056053456 0.382786539790922 0.353668045628124 0.500829214428219 0.520678458334687 0.323743285263708 0.419166690984975 0.431829318144456 0.524798363590237 0.312128178969686 0.452701283066427 0.432135263433573 0.194244601226404 0.394014749574321 0.34730079892916 0.522828579504699 0.431605484901587 0.518243903321598 0.341274282482522 0.46089292130635 0.596606769506023 0.186140627415851 0.30528235895629 0.281956503591818 0.31773816130241 0.31368755435011 0.365106862167159 0.436088806767034 0.417718117511448 0.32505956464459 0.442085277505586 0.332700139788033 0.50191011475895 0.516413727415809 0.27046270073505 0.368077724845352 0.48148351581755 0.372142369269541 0.471051355641587 0.486180756207708 0.434731093552558 0.268514056472429 0.381878983531727 0.520808470183011 0.202675504154004 0.356876316097599 0.335884476524248 0.399018616938214 0.530592575887037 0.356986855607808 0.246540661476788 0.643789380015956 0.225890872593817 0.540937432688523 0.281309100721946 0.300873330617113 0.386590090964744 0.465278801222817 0.3797977296851 0.55766624705593 0.384134034407292 0.391351280655218 0.345713193797965 0.303192632914512 0.378605522087461 0.284966564469487 0.306082475518519 0.275475143467864 0.43152135287675 0.374699749982979 0.307104925300043 0.401991679649999 0.397725475097637 0.278217549916352 0.594779688794477 0.378552023364318 0.380130355007331 0.212422759066581 0.324777755074094 0.547911752276698 0.288276198042755 0.352574245007361 0.562297778221252 0.270882563107528 0.40131892571392 0.284865476348228 0.263092787676016 0.434921852887489 0.44513003660418 0.35549181081295 0.330627484910342 0.294211034181004 0.55276627330435 0.331380205293078 0.43302290821251 0.33323277553417 0.48147419725993 0.231958206338356 0.540263108753808 0.341961167208327 0.326208832213075 0.459430774544432 0.348839748385396 0.423133147691283 0.331364352028166 0.55651913572792 0.408982858287161 0.455798138965412 0.28429682381024 0.360450137376271 0.699776065593294 0.36961852942285 0.376148532261713 0.35531113159804 0.330346936318548 0.360844198079644 0.428833728690921 0.430619558121671 0.37222722083141 0.479629861280026 0.428221239893193 0.446561781363076 0.236670291487646 0.373937148093007 0.396722851585384 0.432090795988149 0.308616629134479 0.282396561841762 0.546852604407707 0.529572567938316 0.280989576704423 0.278932929118769 0.321974736742807 0.416775177566546 0.290757875292763 0.505296391551502 0.3413832935684 0.447397363275243 0.573457255081767 0.317537505511442 0.339883615631646 0.486608423490925 0.389241538190936 0.379557173200156 0.303549306285933 0.505083557691244 0.337790678882651 0.539491668037141 0.429408765792742 0.312067220243059 0.326402364152155 0.353631142209181 0.247682452948185 0.299515484432359 0.489570305475083 0.316470785730829 0.453946997651035 0.215239382507645 0.377405249102212 0.34534542026291 0.803717865741944 0.318186521358072 0.403229217672704 0.411349633258674 0.461652016773155 0.439514603363493 0.411829303715616 0.433844767910967 0.502123920373269 0.387060706801414 0.577955743417651 0.515509390853787 0.372578709039153 0.428618356710065 0.253603955668782 0.318838817404396 0.330158305294946 0.391863415959578 0.283614095529443 0.696215903382178 0.463074685949322 0.488392723179416 0.345249648784669 0.384357294657105 0.296182463804367 0.324115765436799 0.29145201495159 0.35757628482052 0.246648635261281 0.467041895544111 0.351861656802964 0.203371873427754 0.277978745768141 0.353700645925918 0.254823243095407 0.531213515489717 0.521406929885592 0.356323183798757 0.442909189763838 0.393623853879428 0.31764145821095 0.489763702830772 0.226496263881219 0.252916543173584 0.368539260234338 0.516763723512678 0.594584082743038 0.270273816053302 0.384102923938725 0.336531011575703 0.621282766475448 0.477305638508862 0.22175285803442 0.273721337350321 0.260664433167122 0.311943349016388 0.482058540037402 0.312340318366902 0.35109555156104 0.387251808292427 0.349003290334719 0.414507921355285 0.369397769917632 0.470778076994299 0.360691725911946 0.454845931335332 0.493888852206169 0.299294115028991 0.615476526721661 0.541563715899049 0.232503611481922 0.413443694830087 0.410341275495762 0.440445897839126 0.325213611439031 0.483020539854106 0.246322061211328 0.363073767030151 0.395096090352615 0.340641135084043 0.31300798914729 0.635839524810621 0.343110881351342 0.396988949333028 0.636861573671521 0.342948210187464 0.394012445486371 0.275138792881343 0.418092753788273 0.460442918618334 0.340226582896582 0.488676493267148 0.46626908902729 0.531632459098594 0.349461704059624 0.442238296078411 0.298149642178645 0.285009360638714 0.514358052776342 0.38079951581639 0.425091100054398

Th1_cells 0.562657110373273 0.564898025147575 0.555385581480843 0.529430753022835 0.569043926625369 0.520385157118571 0.634672844485747 0.574354567984474 0.569384813604752 0.620664525317533 0.575131512327782 0.520616470246335 0.484862340253927 0.46961824895754 0.452866529938535 0.492213813181483 0.530058183247158 0.468718392066898 0.465403108416066 0.501968734813113 0.525408043835494 0.606466685037547 0.514194018065218 0.541589155828558 0.559095855676441 0.550944235314178 0.600042496362096 0.536269232570001 0.505288602201238 0.541324485906967 0.569804113222628 0.488426044714522 0.523110101823977 0.514018375976579 0.518827630174179 0.496136571436651 0.574825001678761 0.539994560847788 0.539175260544195 0.483981574975378 0.491874017197218 0.507699075609339 0.513948255396049 0.443165881676735 0.523481310451952 0.540663560625644 0.536588167837221 0.480604119639139 0.518929059687056 0.580198870587123 0.542180177035394 0.510818027487388 0.563596701364109 0.563378127354166 0.514742552073587 0.607130242347212 0.485361992757998 0.521491213369475 0.575919144258323 0.503806381091619 0.582735908187228 0.556244445872401 0.501103813869836 0.564261038980771 0.614875392195736 0.52789269362726 0.575336505763682 0.568224311791079 0.478006101333898 0.531126418466954 0.567222426906327 0.488429207935741 0.530549851274494 0.528252413250854 0.496864151945237 0.536255227148194 0.5372376212991 0.515641203842898 0.465225885073096 0.518880705335051 0.46649116054335 0.512690753492711 0.510854536916977 0.54190250553488 0.529515714120997 0.497967874655428 0.546322073379196 0.443942133616176 0.477250337482983 0.528137836799757 0.525171000282086 0.531139469760149 0.520756855420583 0.526371609714765 0.539215701206792 0.556905014553451 0.575515706107424 0.525392401597338 0.512288090222475 0.509239332062984 0.526939948878137 0.608750994414156 0.581453612364786 0.556018199557635 0.501551903730541 0.511266343793073 0.478425278548609 0.565392341582034 0.554197759455016 0.521612433619321 0.485791464895792 0.495541183876671 0.50627769389908 0.485258190081968 0.4865847648188 0.535033964956826 0.563417132946995 0.53086075737108 0.541430194374136 0.563050383981048 0.526300742085807 0.59752320923795 0.519708999154643 0.539684736526251 0.500319969897451 0.490319736830223 0.552305420430206 0.537347322462704 0.493459524598998 0.498460175260308 0.489929260491188 0.580206318628975 0.604679632711638 0.549128493001479 0.591246418648809 0.480907227967901 0.547642991712543 0.532864102014533 0.563963370517282 0.473871452060796 0.495967096573201 0.599784545937226 0.545858375263143 0.497926437702057 0.531673545177308 0.495661765841356 0.487126607051547 0.461597284731395 0.52123657866999 0.499227503236624 0.516778608345049 0.525207613337317 0.536731377933058 0.57704737800938 0.545489351030693 0.557560212809842 0.553367605781739 0.587076441468524 0.527345992420095 0.495017629279725 0.468079297943362 0.515499918195057 0.56118762264097 0.515971621233514 0.605929213678158 0.494640650123759 0.555432569309615 0.534155909854152 0.502204415571746 0.493229929321329 0.55104852321024 0.580646875141451 0.472148500789412 0.506605798535731 0.58263693215507 0.485846924614278 0.495715978285573 0.519879329446035 0.513809939967574 0.591187611909168 0.565831234413867 0.49420137236621 0.450040598380527 0.528331898197009 0.613755761864048 0.490046743241749 0.502896207084909 0.550259253839507 0.547190125690506 0.510340561202268 0.465644916525567 0.575998214413509 0.48649958241153 0.482089589393303 0.519120073376201 0.621035947321566 0.5387074352461 0.516592686307235 0.538518026848743 0.504595890541111 0.566461587307011 0.50967990557579 0.578154413831557 0.55806416935778 0.548049117518531 0.594891493497535 0.492897147276238 0.475878478394194 0.538063161087679 0.684108240058074 0.445081558469665 0.563213335316433 0.478982492447144 0.526488173761329 0.608359001965374 0.558585439435772 0.531375814002655 0.521070073127808 0.524186685102865 0.492441246659623 0.537860881634914 0.543781115428119 0.486640727824802 0.585911876503069 0.508593792963398 0.64489703192249 0.563224251398007 0.523250400866082 0.577774935461539 0.526558653420707 0.493302910093634 0.548688363480216 0.491830870647504 0.528644163143617 0.538211164770632 0.532139288219966 0.534144398136536 0.552055731570997 0.467369441583035 0.517851554778157 0.636681086406857 0.523693840684817 0.56928340072835 0.525886706209759 0.537744327364614 0.581993956944459 0.556589346763321 0.534599938858769 0.507331512490493 0.694508964601796 0.544956611527767 0.60938579843241 0.48652635118562 0.569789755442547 0.517153814632976 0.555943631300566 0.644314507364481 0.547179354842441 0.503706320865861 0.567753584917338 0.537863460052991 0.514668850235946 0.547958744718371 0.584041822454083 0.537208410468739 0.51261686725226 0.436457121818022 0.543680552806435 0.544368401492645 0.612885561409039 0.579325083277551 0.495791940214771 0.548131796311238 0.530359462458539 0.488861370526485 0.591059181192971 0.564436927670645 0.588602760221736 0.512937315519963 0.649394686767215 0.54107881032543 0.544975023251744 0.533575238175333 0.528807184945758 0.567837968103543 0.598991243653882 0.526713035028704 0.566610342264662 0.545635203373337 0.505814788888737 0.541705180008037 0.525306200274877 0.540164447225801 0.50931814745769 0.566535783126533 0.528560594961176 0.537072173192725 0.526675299599517 0.570743482905324 0.525164306234827 0.526377612530242 0.557219931191406 0.472325628709451 0.492883577841139 0.547768567386668 0.57213592641516 0.540246022051529 0.515647698037699 0.553484893111102 0.550055213922989 0.572037059563156 0.480517075171975 0.55594146619161 0.538113887069857 0.532834551730639 0.525736231143948 0.529282238347981 0.558609040607981 0.497335159078642 0.531140993141616 0.550715387934609 0.555970773712503 0.452712909423501 0.540572901378623 0.60472051227764 0.509938140216704 0.56086619918939 0.520515768777405 0.550445925038889 0.58123342352117 0.5425913368253 0.568453705950745 0.588435046704373 0.692714213636981 0.583664924573846 0.523000099504117 0.51652768365635 0.644166125319855 0.580994056154467 0.485539166609864 0.518802414975977 0.458754159852902 0.514795607759721 0.641594806912 0.534898285625895 0.673458078958193 0.532652947561536 0.538550140725705 0.491372135891451 0.525044240453616 0.557344299957796 0.478255067043048 0.492631237050284 0.562051320783327 0.511232177589909 0.503202014827274 0.525657292522288 0.592054386104318 0.562224878135218 0.543794452181288 0.545208156799055 0.480462549595853 0.508834269895847 0.694229271575276 0.547745732962822 0.454921206222166 0.482654565501488 0.544060441889449 0.601772561736269 0.485529180230849 0.55774721137055 0.653053295585305 0.572000171316986 0.53117134698888 0.465506275890213 0.50214871828387 0.531914176781491 0.575253318285732 0.546002571811969 0.539826113110835 0.5817825109923 0.652290136075653 0.482135408647306 0.540951254748412 0.493297890035469 0.566590456558523 0.501432938027853 0.55371832745901 0.52852227665083 0.557364054868269 0.471291983489437 0.508691340630107 0.576217292877646 0.503816814207052 0.524612088450663 0.615884628995299 0.449192989647578 0.494251455331247 0.563728639737348 0.529355228580789 0.493770965715523 0.481435954291834 0.519833914342625 0.567100815570708 0.44482382391374 0.532400886907882 0.543738766895004 0.562884518604813 0.531325947765351 0.479826062955125 0.501221867206178 0.562734390192935 0.479408740495213 0.584657764498894 0.535425307327942 0.535682244184917 0.453643356426633 0.534581389881906 0.544360405898679 0.551085537496637 0.631628117639904 0.519849637548196 0.516393599290868 0.537258062072338 0.493314380941223 0.525561938371513 0.547068967250719 0.541626648495323 0.525705544551146 0.558662076102729 0.546401429448165 0.508225778964149 0.557987488914301 0.61960901309071 0.493036527546463 0.545301662865109 0.590906848882859 0.554342591499625 0.473977889818955 0.488337982346461 0.569654464524671 0.510034225701878 0.529540100911528 0.538054985283999 0.554182061896465 0.584934961210351 0.504411992645293 0.527762110329681 0.488717931436361 0.496674584701152 0.493867590424587 0.562090201104045 0.478917292532923 0.565500255843474 0.516490555140357 0.534120370215928 0.532331920470567 0.512568234599044 0.515012883233362 0.509333986411959 0.521329357640027 0.51371492237287 0.547259810188291 0.488968470698204 0.501252083692176 0.528011358072004 0.499263004823811 0.544579645282779 0.507893791500447 0.544972617175483 0.548150855126049 0.533731533906616 0.500014557582792 0.528487906570648 0.490517373195061 0.521610438507502 0.562560283438577 0.548467146590125 0.52766839552635 0.606318099898995 0.477691203393733 0.533005873856379 0.594968618290978 0.575694730099355 0.534801770274882 0.581588109235514 0.54768955815818 0.638004959770542 0.607493581600505 0.521057488457533 0.567080143452181 0.5604483130164 0.475261812433387 0.573170203791704 0.536145513487393 0.517445439926393 0.552754853939783 0.513727872674664 0.479572268409808 0.529182005324904 0.671292630978721 0.487561761860809 0.566529438345403 0.544725783097029 0.537163508282 0.513936892585896 0.584527853271441 0.539329117030637 0.585628137439702 0.433752142917076 0.473592093998023 0.475515668595883 0.538561143389524 0.534559331158396 0.493999941773285 0.537842890612218 0.520995463560951 0.528803271815528 0.567255262704311 0.508966443118606 0.512652278254038 0.582494707785386 0.557436226684956 0.577518439564448 0.550392257317236 0.424056634125854 0.512400469897357 0.465022479002985 0.478946451376891 0.511041800395686 0.49533973680327 0.570182255534818 0.45974738882198 0.478529158325623 0.447753928881203 0.563464049231579 0.512452389955993 0.515589870765173 0.569386616940281 0.535691876588214 0.543396583645272 0.533835190366593 0.446062966740295 0.56783434058943 0.575637043771461 0.494022948773551 0.553484198682308 0.57728895721475 0.545157977404916 0.488849014883439 0.573629232205986 0.509556966455085 0.487824203158257 0.518458882591146 0.498859614554005 0.601505344033085 0.523632373026431 0.56549012585105 0.528704851443436 0.58561881297833 0.519190931306927 0.549325361637478 0.581045436126107 0.524116813254641 0.59993452196618 0.503274239132916 0.552011294382314 0.573889941886607 0.483388472566859 0.596961441793531 0.505831844104642 0.487867363956689 0.515825048919228 0.485801640906221 0.555512725157012 0.481227186357422 0.49366265913011 0.480370588193615 0.558049106144624 0.492441099641536 0.507138278141159 0.538166575631751 0.586675543886111 0.533648014146163 0.597488216072385 0.49139488090284 0.471122983233438 0.637985915623692 0.481249932596276 0.519291745166039 0.530081352064255 0.509062056442409 0.427847596073975 0.497900820608613 0.504274090281694 0.481016164841178 0.50032350582148 0.497704654353922 0.555549313862039 0.469396392489485 0.53165197194478 0.545558341006675 0.53712744451929 0.498891250223551 0.52210496849953 0.468107394832647 0.51101519177476 0.573186428687375 0.498326821458064 0.695083802820751 0.510841713441381 0.494639417386854 0.546726476829507 0.505716071513431 0.571665372302509 0.534865676975462 0.600566093136416 0.530282829221648 0.522934732505034 0.620149860747472 0.52075548413375 0.544549439051143 0.53479924985215 0.561985400091474 0.555253854657037 0.537961057368096 0.509452991358431 0.51040552687037 0.613706524391715 0.557466633716936 0.506192103234351 0.489540737861176 0.449408740187091 0.516830853949359 0.539192485516975 0.467742551719189 0.469264870743598 0.575903605951312 0.522573661344301 0.55081388530886 0.541180016813583 0.534775853082394 0.456707417120315 0.546365259168374 0.574928071124772 0.547736927196124 0.534500738870902 0.493561487011631 0.594308941118031 0.531108437087361 0.566426032602397 0.575014864590256 0.538926915630607 0.505875467089662 0.610334588239172 0.584010057762419 0.493500087406769 0.520455006060571 0.552423047262119 0.687084089741665 0.546885201626607 0.541193245591466 0.52229910654018 0.47951679285086 0.449721931455552 0.54482672038142 0.490276948317561 0.512103963550746 0.511894558332506 0.529827395698045 0.470543065719866 0.591330324384916 0.48091774736659 0.562692119028156 0.513209266677785 0.469766741860888 0.507314864973723 0.608241635452883 0.544245754567388 0.489854536558429 0.615700376225339 0.474295060323531 0.462732435145624 0.50038184790203 0.54995734573398 0.459856739051748 0.422042740269443 0.556446267381729 0.524379067203742 0.550399622916985 0.671644816173385 0.546302187761262 0.503847014465427 0.579042454038692 0.540426228405904 0.535358281183553 0.533603797279176 0.486514632786888 0.505826914945745 0.573122187082558 0.559964429957256 0.603669993843614 0.528465421734166 0.552114739689392 0.62208170644909 0.544541880148875 0.482930067066745 0.582122241324816 0.557124895916328 0.554202451931253

Th2_cells 0.45749143580126 0.482433710765892 0.547657653837753 0.504868758971729 0.430916843425296 0.513254068199252 0.594203355802008 0.492901592151595 0.509524924202776 0.596553560809133 0.600828511056202 0.503276968988358 0.503723223537137 0.457232237043595 0.470058625083369 0.465272900075113 0.524800580982846 0.499028697088049 0.439818408119428 0.389729723244219 0.465378652880417 0.636631212424008 0.503750820563016 0.64580763520345 0.536813894859261 0.528597283481171 0.568092947188611 0.332322094020291 0.531595068599779 0.521079458230237 0.526364067628772 0.466273719654228 0.489495822657068 0.540731605408245 0.564115911694466 0.46798547349084 0.540657211196732 0.384393941863039 0.487683751645953 0.469606175786938 0.40775148395673 0.36329372991343 0.421751782088071 0.490322869401658 0.514659740055328 0.505531219938827 0.554807214405109 0.483817721270019 0.536717191942568 0.453984918675386 0.550992673112367 0.425001538376337 0.475253720049456 0.56495849465292 0.57586496603901 0.525807126279971 0.452271662764689 0.550394183482522 0.55030194410157 0.530727651388497 0.567411566355739 0.547280199339083 0.489566005202803 0.487173170477904 0.665439860720648 0.400331670428792 0.434349625092545 0.524950789622132 0.417580386434434 0.573456576959214 0.477266473291903 0.529714227131774 0.513209258720287 0.380517476687365 0.513064993973354 0.456061728052677 0.424222732404801 0.393935446004066 0.472776616730006 0.579712062235638 0.443681036514026 0.498004387727252 0.478437305165343 0.494270802670664 0.472900904597977 0.401126397932438 0.552777984923811 0.432113366642347 0.36259604446074 0.432983245319713 0.402774780724666 0.397087330950998 0.485449416476108 0.651643059888933 0.436096488567957 0.492300289144626 0.493376107179241 0.617597944674208 0.590374944040135 0.500870303565694 0.510973230024161 0.595961697666395 0.493598098105687 0.551191257273519 0.454295400104622 0.395907895879786 0.534088660146478 0.522958301304812 0.457839793946449 0.473892820348839 0.48422996983278 0.492799772152839 0.439571670332325 0.517950310877934 0.483407870945622 0.526904998900003 0.56339302338543 0.490060461416245 0.359230080690854 0.497162976019328 0.478000613204569 0.472213640718058 0.525543963711389 0.502479846249031 0.478254985636165 0.595457847490573 0.492638051171656 0.405634705863433 0.397301228058161 0.454683886192197 0.459126424984282 0.500458896774661 0.527692546025493 0.473269161040679 0.611130193038618 0.560502837834374 0.391161141655459 0.47257331260088 0.434974936632619 0.496441961755197 0.432694371321475 0.561445186025083 0.523449086373408 0.467534377872401 0.567998717211446 0.434010323049127 0.467766707440423 0.465289476644418 0.535999549400252 0.447731742718831 0.533544791057541 0.429467465820011 0.52632390830688 0.471198551457286 0.468365971038406 0.628912278556949 0.509738500065032 0.453359257945997 0.398138696939028 0.444362156176019 0.445910273566611 0.448123836921423 0.417034189271744 0.485896064140261 0.560212940273577 0.428577318944977 0.469413487685613 0.470190392789324 0.496271372981111 0.419577683794561 0.499809370928537 0.600733991604851 0.388181741274188 0.431037104940951 0.557992417804474 0.455772079836209 0.451356005298355 0.564798848324214 0.448040323261094 0.458530135321384 0.506098431178051 0.557705111400719 0.510935307460546 0.456865383845694 0.577328225036218 0.4307055682307 0.503216583378654 0.506190028741439 0.36668075006049 0.548502538725224 0.435917442810894 0.579238409448077 0.444808846122967 0.577014578643113 0.565097462402518 0.541737148405424 0.485677364260507 0.46354423357058 0.469860372851006 0.476044687018418 0.522587830448463 0.460391784803016 0.511950853521463 0.644184451570422 0.430167890372515 0.467019503213291 0.415237081278707 0.473098122570049 0.48371798271149 0.66790865138629 0.472139732754107 0.595656982202332 0.438842304666118 0.542055762452933 0.612513496182417 0.484368493201541 0.463217249869461 0.442789019719299 0.496460039878896 0.301839687179687 0.541127894550497 0.439036070080698 0.603906608748555 0.601430613199918 0.448008514267618 0.642785435470544 0.452829820721932 0.532914460716357 0.558807573990058 0.484476565933062 0.49977170384005 0.461413711290391 0.389195724071189 0.570888342051612 0.478224619845269 0.620201943392243 0.343847089102335 0.414145916082033 0.450478156104826 0.470473488828307 0.546468105356642 0.517441932136759 0.487228378648921 0.487565648545804 0.502454141856922 0.622705325412921 0.441291347396399 0.428698822731323 0.391214228002521 0.692423965252242 0.642978932838353 0.542332670843341 0.645519300669045 0.642032155647674 0.471680707893693 0.614398029264119 0.642065761495891 0.514133894780167 0.375373004789628 0.54929556002645 0.449369835496666 0.487263478362871 0.393808792773149 0.494167780236016 0.487191513748306 0.490098026602998 0.431162359507901 0.390509845055265 0.549610004138032 0.544446869180473 0.582680963361963 0.511992758855883 0.46137621589498 0.441978960192765 0.365554663974168 0.578707183634553 0.489172501977151 0.561084035594534 0.517317124866528 0.648532798171398 0.580386117330689 0.498614399052014 0.522658835425142 0.549285884784609 0.570888525653587 0.667915781742117 0.484079652357329 0.47782237509845 0.515287863681928 0.382532724312979 0.488872757846318 0.470281837564649 0.513813046244895 0.486813314730085 0.528226578098007 0.573158373277738 0.405907713535364 0.485916655150597 0.446817908608557 0.443181195811731 0.609344066098974 0.571953568672909 0.47706953297361 0.496723546014648 0.511820379347002 0.520681334613041 0.595898956075802 0.368097950432571 0.548470112731152 0.445232084205894 0.645707635064377 0.477024111029381 0.437260172692481 0.374744198201712 0.54178547015632 0.489139595533284 0.37748008279078 0.430907119699253 0.533376903112651 0.502344243357619 0.504277820561853 0.589595814319932 0.480422804286964 0.435489892948723 0.568231195162294 0.441407272549957 0.446149395923624 0.50295650541862 0.478524028112772 0.59200707049503 0.532820923916226 0.583256900947928 0.453308628799349 0.697894017471702 0.462023068866191 0.582298476097149 0.56128084880831 0.621888957734814 0.602129096077191 0.462447006529183 0.468242015510177 0.415543108829507 0.492084848515494 0.605442966232286 0.565055082984874 0.679913734163118 0.486231930931622 0.328131172660647 0.482330413204023 0.479361247289463 0.410147709313724 0.349164529481005 0.466841348237003 0.482502642946846 0.440621466699581 0.458609949788496 0.539459489965647 0.543250945255598 0.528212956079845 0.541278063098804 0.577770880450776 0.447065636256512 0.511476734618148 0.630307855647802 0.538387508555017 0.427121863352457 0.478788756709222 0.568874185005668 0.595361450588003 0.440806492647862 0.620353562896278 0.65667402203296 0.537552584488002 0.381890575882918 0.464273765965806 0.552946948901155 0.410554086695108 0.502517676990771 0.617212948012163 0.492559385325832 0.538203836151836 0.631827390365133 0.372019599390078 0.434779767340027 0.484323280397096 0.404614129845879 0.394472569722879 0.510360183707464 0.401408074446242 0.484535333479847 0.517789595116565 0.514837567331222 0.549877131808505 0.435787755568421 0.539476310461991 0.612254368805333 0.429522158304908 0.4996901247679 0.518345413424026 0.469030730452348 0.447320855797638 0.455783450620628 0.558446956365831 0.578402416183805 0.526405026908629 0.524423901935335 0.476944810384302 0.510974930072822 0.494280675157891 0.46113927820923 0.607067156171353 0.486923796369484 0.413599240677582 0.595555057860824 0.467405148221054 0.597753840174177 0.483849223703204 0.495856350000871 0.643473130176983 0.415655405465457 0.616894246766853 0.457226503136454 0.447650402458652 0.491409019814424 0.498754110474752 0.400128680649289 0.50597911239377 0.463804176213096 0.439466530381844 0.329886614830513 0.559581847872218 0.464344886914045 0.602513651941223 0.630251670975476 0.50161120489207 0.462962374354836 0.573332510777869 0.602298960999396 0.50021702305812 0.458905671222848 0.557361850589116 0.455746780624467 0.571499865908886 0.489972512313095 0.548191135287149 0.508120048326322 0.564442418299364 0.386579824298021 0.492469709188705 0.505783521507311 0.488353407882013 0.43045673335067 0.46259434186222 0.537683360257303 0.53268819362504 0.516223112120059 0.511707706948381 0.401971188081252 0.493893095859043 0.404946228908982 0.473391963443434 0.477288909702916 0.528776829727713 0.419826552078646 0.43656104246006 0.514499013050839 0.571523975234556 0.531775524300576 0.426831604463703 0.530538052738072 0.354425125266163 0.420893764604884 0.33017375983072 0.487853495488597 0.48407277247379 0.448817824833505 0.522437914511449 0.451946114063757 0.438503591526426 0.614316176937927 0.436887175126968 0.491950539222216 0.527510180029884 0.430406205820967 0.503986817856386 0.465832172371414 0.579805939656248 0.6219309619741 0.525106457695795 0.423501011175998 0.528087598158019 0.394289253529584 0.476800740517907 0.450975636295867 0.511654053826514 0.477517826964034 0.507366302374131 0.520529056560113 0.429649237584049 0.449474635929774 0.678362269328621 0.322835852242266 0.645739410756767 0.464608314650816 0.383032715845548 0.508039487997516 0.516102999913144 0.469378576059969 0.598731960256275 0.315677532750669 0.562530994932186 0.592962980918006 0.323726652289593 0.496468246829604 0.381446643886141 0.500409081027954 0.472338896131309 0.459451307601602 0.499932216426203 0.487866072882039 0.512476033377446 0.498937883347397 0.606451259358407 0.541679137987549 0.6156151287721 0.48454438792789 0.396726748730983 0.40841986376112 0.403385515201231 0.485159678878506 0.585489003530989 0.555688736219542 0.440517608064561 0.414868910377787 0.526381867374992 0.433820905688157 0.458767401618019 0.471021439620268 0.52638194335216 0.482650932673887 0.529093757132736 0.52982350269182 0.460722772981508 0.543141964609469 0.50548196152509 0.624734132030786 0.37999066252974 0.643220550798453 0.508739849947233 0.441430646364785 0.601364333148426 0.463948810997451 0.423039177674408 0.459434336485923 0.533051522331174 0.598091012714268 0.484141070667765 0.422008906744995 0.567629540813883 0.641147442592181 0.485754933019267 0.548807717734339 0.59204629404534 0.508459008414938 0.567953047350235 0.479257633522537 0.544570286308562 0.494386720396766 0.421008062658374 0.550481013510085 0.35219715377578 0.502821801976192 0.59570669210101 0.411142331235309 0.49822192684489 0.40127369226835 0.486584899490042 0.49121709956296 0.553229619088351 0.524712013995147 0.490354112591221 0.419950958532977 0.619098180450069 0.36328948366 0.511588534013741 0.444897402924631 0.500249294732418 0.694222726747933 0.437515634575754 0.358296583395831 0.491715581655826 0.480109712235517 0.469556391368774 0.417188534853252 0.53651437055673 0.292944945280455 0.520878060494295 0.442946864554885 0.454109986411952 0.426241997779516 0.590121603429506 0.507062649397077 0.480436765459121 0.48821030596359 0.453100428585311 0.354233229243841 0.451624204689072 0.542064540742335 0.375328269936493 0.721988384519321 0.500406801258441 0.527289191267031 0.483381267717158 0.500979587076069 0.550249294911945 0.424305234880548 0.675736237825322 0.483685416196488 0.540746604575531 0.556406256911643 0.458277272714435 0.620242602123882 0.529730290843754 0.534362952491017 0.429847499750526 0.364831504291422 0.561203362061311 0.474632513457608 0.660354799999803 0.533381083734732 0.440849271994956 0.517974232842261 0.441848456837101 0.441682303959543 0.454652834938122 0.442276808093837 0.476822744697945 0.41657622992517 0.545671581760765 0.528757171987717 0.571625493841864 0.458518237332846 0.478221068458378 0.489355837034242 0.448780834636705 0.437501445362565 0.424658082999771 0.59217260561059 0.586758718577478 0.500403669630545 0.423821053904927 0.499478891455244 0.529780637645361 0.456637687894062 0.533849076598732 0.559008902758887 0.448022385281165 0.481832159706735 0.524239404026679 0.635781490322171 0.491230928357482 0.392961753628643 0.403730304941255 0.212505022409294 0.435334456363148 0.549817546661457 0.462675152674953 0.522888028662255 0.455118593555583 0.398474211639741 0.403860605074402 0.53552026634893 0.514384920288542 0.481405474066257 0.423524724791291 0.502533339748684 0.559524613776663 0.576275440126287 0.381800966193272 0.425500103191882 0.562373274523303 0.457995367733563 0.452889759306657 0.479598008703952 0.505395555212404 0.409208260044779 0.423426106765482 0.512166438379996 0.58402713148816 0.344772610225552 0.717761336725343 0.49336283038335 0.528063656377521 0.593552592327733 0.48513146090289 0.558491337815073 0.449627755904512 0.484213012122025 0.455231267495453 0.519332120524925 0.44281230514614 0.552080713814493 0.49550696858629 0.487962012515424 0.642526481374665 0.455939540912653 0.482738721689428 0.697489448440367 0.54197875720581 0.518437990497224

TIL 0.653042380016286 0.640359878046478 0.641187724979813 0.665415597838832 0.658425443424183 0.672279910465318 0.769621217867849 0.705533602087561 0.690229924744991 0.768379035505168 0.724852472055952 0.64888139220177 0.620659631053715 0.605035834313651 0.614272982851693 0.680487551219045 0.745932119559431 0.62220496502993 0.619696238354294 0.612747608065092 0.63968169992211 0.778781529338213 0.651250071609023 0.74357241764049 0.737407860149369 0.675654952124975 0.736741603969927 0.611997249497916 0.672404669085907 0.716974532677026 0.725190600863399 0.697427284239005 0.639131126282536 0.724649352625606 0.717270351306706 0.637539317263729 0.636469536424198 0.673139808301193 0.680319044964195 0.644784256177258 0.66323034340136 0.665916806831013 0.620296750096223 0.678288136346921 0.665676510560321 0.659646391338991 0.707037924726915 0.624334619816866 0.68674374994981 0.678369587540011 0.689919223183261 0.598234516013705 0.679352233301595 0.753917664479289 0.656509280515428 0.695868728811615 0.633331110623293 0.715660501030547 0.639985904673837 0.673698204630766 0.760155898254825 0.673700714750083 0.658747965617132 0.702207647584047 0.761598273288764 0.688716838822422 0.628908835221181 0.746817015522062 0.623709780723776 0.718975197402215 0.667141662593603 0.662933685664837 0.679099831351133 0.604427026656351 0.643412377150575 0.632667979839362 0.644009215500813 0.699570299503645 0.645524162915743 0.735827136822031 0.632290926687955 0.706942677448559 0.678212445715622 0.708196578997755 0.716318606274754 0.64192210158231 0.647656017492424 0.634840954854451 0.63438379563771 0.700207854759547 0.634091917565083 0.660739500466572 0.646641047044871 0.702632112238047 0.701275766789508 0.599800844029195 0.625718515786234 0.711706347398445 0.685496007096996 0.706476198119139 0.724881885450899 0.756060919074097 0.683305594918342 0.691212104184144 0.644257218501502 0.683837052689102 0.669637408743559 0.727273544878061 0.709393105351471 0.699262432347917 0.685250927323347 0.640224049265109 0.653840365680178 0.717257566342323 0.67929084080584 0.7088766423485 0.726897552145535 0.707596772001493 0.659446351628305 0.721922395887982 0.671047639985643 0.75604451932574 0.66524773911899 0.710578509092693 0.696265314270204 0.720085328273629 0.71968894583905 0.627082049257772 0.692479602787815 0.638145823166363 0.656023771825139 0.687622241716515 0.760132446783449 0.702714249507114 0.742227573579916 0.681923923291091 0.636841168683655 0.65555436005898 0.640321208806784 0.662564058215077 0.650781368875049 0.74856694550749 0.720003131154684 0.649411997561193 0.688404790047594 0.636619961171603 0.686737813169889 0.675897066786371 0.668021970864552 0.696147160655219 0.67361217256734 0.660588518076166 0.673437658247115 0.687145812098519 0.655425065380517 0.727544448791582 0.693646324683466 0.664422952171852 0.708412539784233 0.646703218026089 0.631517403426224 0.617301521587678 0.638694914837904 0.622846976488391 0.685129414843078 0.680735762839555 0.626758278764539 0.666170743334056 0.687392596174246 0.624406859206498 0.636442345758162 0.755435420823476 0.628990829508326 0.665356969828835 0.741246553244923 0.662813939315082 0.659263738519248 0.678807467987023 0.616811833022135 0.652577382049424 0.73017684246422 0.65233537313826 0.634037638565415 0.671887383976499 0.726950020911536 0.619271460757865 0.689503518580693 0.626645127550441 0.64796146452325 0.666928102273376 0.606604149210946 0.729560828287894 0.651546553664559 0.711034727316275 0.71242458557671 0.732450109434498 0.655966092655205 0.655624083580933 0.670560631447825 0.691758508521243 0.740142976827441 0.682521364875653 0.724485054844915 0.731241862126563 0.618579256452169 0.648113108340704 0.654711504242651 0.692472181488884 0.682039297052796 0.807832966282349 0.632624141394594 0.752071107754451 0.665137075768263 0.730985353497695 0.786970204761224 0.672752780725664 0.601718839711258 0.612609100711043 0.700470331745007 0.615896932833441 0.740872700227978 0.61944421668057 0.710291084758518 0.706471027241683 0.632850920126565 0.804662553476235 0.668156138120656 0.724574637937346 0.716989606785576 0.714091546173603 0.688960461571441 0.659759642297024 0.651214277959369 0.732359729773765 0.701265879423631 0.737012052649442 0.615848422662099 0.628987476091608 0.625351565377757 0.658151314415208 0.69366676208328 0.653171022086132 0.681318187998031 0.666050595712019 0.676730307545543 0.753451908366388 0.681490441559556 0.650117085444308 0.677506223324581 0.83476781930698 0.747086913190315 0.725339990363957 0.72215007058188 0.762920780673449 0.686880448378103 0.732131548512329 0.801634867730012 0.701105205124491 0.648625821893362 0.727125284831966 0.649274251199139 0.699960007445086 0.595394888117526 0.66494378690105 0.681396545137226 0.658817102999929 0.62163371140323 0.596066486179683 0.712873807862818 0.786827858965184 0.716390387031495 0.689005044105712 0.627645045855039 0.651929710887655 0.630539620487455 0.753807674291102 0.744419539553565 0.730882504197238 0.658487443736689 0.75047475496424 0.760619156767525 0.69371796494494 0.628704311534298 0.707094664492795 0.7288907116009 0.792831817286679 0.690187545512288 0.620904058305155 0.707838037758673 0.67132215310273 0.626689339281543 0.660323384288115 0.676022255394039 0.619580563052209 0.676937156228198 0.699741485789491 0.653446677962382 0.693700280733744 0.650234756141938 0.639949889457932 0.711069204752192 0.738769763638557 0.630958145675749 0.666045753844639 0.686767650032677 0.648952939028958 0.721074611169708 0.627964300617791 0.715528403363837 0.639991760553455 0.752235046957932 0.653173243391883 0.6741033596169 0.615325224450721 0.708341604852888 0.708068535191773 0.605244687791783 0.654294758419273 0.637089080797698 0.684678696890442 0.670344647533679 0.726586586176284 0.648509216933666 0.671122848955354 0.750759554850697 0.621777322085998 0.677871694056442 0.728447386364901 0.705667076334493 0.731751448735822 0.6965168626609 0.735879534869331 0.696479952934832 0.806448777998645 0.668415631348379 0.700034897097657 0.649099370965771 0.809510854321817 0.703786121417907 0.638796178882954 0.63005402063693 0.6536382964857 0.643543157278985 0.742697320285899 0.735644013376116 0.826060062069869 0.646245196191964 0.654602397231274 0.651564268814224 0.703547903351457 0.691664657763673 0.601732542434802 0.638352626610871 0.670356216737367 0.668377651571446 0.673295414723401 0.689533987193539 0.725696151857374 0.722132327554494 0.690889301104789 0.623399607061177 0.637801048062101 0.682043508600985 0.774269418869355 0.741956210165848 0.638745097001636 0.665711437249236 0.661062929991672 0.741506106568436 0.657329335538941 0.753019643428875 0.784728450922126 0.699273499588415 0.626453812054585 0.666034361328354 0.672443628726468 0.702042308623055 0.719328053642209 0.704894699142043 0.678087201287794 0.69963981397769 0.803654220990708 0.613126297536949 0.634113815216739 0.669389941594872 0.656912439216806 0.660781933944144 0.717052982490026 0.589233967235161 0.731145712278737 0.650093070544707 0.686934958594 0.721756217246752 0.647813386853821 0.704890592898462 0.746501935082277 0.664535313543648 0.705352397096755 0.675102276517661 0.715898587448461 0.667122674938612 0.627332350801117 0.716452430178429 0.761651532951541 0.61356945916183 0.679991460333554 0.687204643043394 0.699035858220501 0.656275467888108 0.680292871692796 0.687526253575776 0.610549939370829 0.581825323703076 0.684723946146313 0.617670420656353 0.686187992723361 0.62248007702106 0.699870763294664 0.741544051628915 0.69131827259278 0.726858303846885 0.695046268757647 0.678737684306637 0.69051341725499 0.689165432857826 0.678965660670303 0.63251719521227 0.630106122866546 0.649440426782593 0.634932012788636 0.685511961379606 0.649532390492562 0.681641057795593 0.735795946780876 0.635195954054147 0.628993100099707 0.757922834443991 0.735019463260308 0.650147889238803 0.660314502977029 0.753739273057987 0.670065477508978 0.684114613847766 0.698947260813539 0.655699032395607 0.641103084132512 0.72476412471384 0.66521570539015 0.687949233602136 0.704345207490895 0.608855822342431 0.655564656167215 0.651371189409307 0.729155405392383 0.668372233917429 0.723404910935387 0.684622771865082 0.602027429347718 0.733558667145917 0.677069367016985 0.635466032945483 0.666352585219978 0.717780207749151 0.623829429177436 0.684255176600188 0.677615475515288 0.707973774782008 0.627012782678257 0.650333369497852 0.705876681858677 0.611728987905117 0.657720668415571 0.620552349226717 0.676877770041334 0.678314315774245 0.658827554830474 0.682536435105278 0.710906150218956 0.637596958630229 0.743650594110741 0.661903240343586 0.694763162829218 0.677728323927521 0.669336204427264 0.667870128680252 0.697104788530769 0.681435922998362 0.768671837973943 0.712585666270866 0.638303611525781 0.666171855765463 0.686776839786736 0.63442411066453 0.627119617654128 0.669182471005936 0.668060306715377 0.73350603063713 0.686998621384513 0.648896694959652 0.613319721376991 0.819358875342698 0.634147721423062 0.74573145202259 0.664996350806853 0.660481432774921 0.703234819509425 0.693760190404602 0.685109266795085 0.746654249984692 0.61599319665904 0.640470532114794 0.642944236924544 0.638655945166021 0.650684317331536 0.677769022016577 0.660930332694273 0.640782257843673 0.652916653454172 0.620833251547123 0.672247170931687 0.634541379689397 0.664515006035006 0.708538237821549 0.749904184739163 0.659947662994464 0.622705422925031 0.647067838267515 0.632130309542148 0.574662107233727 0.672544968489266 0.683212098169882 0.73052309029607 0.626915936697444 0.648864081128527 0.634719965833108 0.60698617824176 0.747198023861792 0.632471302888876 0.699852712623957 0.677950655891167 0.675918507132783 0.687847612651595 0.70371934707083 0.692633608939322 0.645815384993909 0.748577926395269 0.667551596413205 0.753074322201199 0.675125281561088 0.624946000680654 0.707827215720842 0.677908341556262 0.64870739356599 0.642223770979778 0.689061433165055 0.729862732093872 0.653428868107707 0.671881773530638 0.730448884560336 0.764816573550385 0.705993190348535 0.638470245986469 0.74232068933877 0.732053668839515 0.704740133299612 0.645737196117329 0.695404041935498 0.705771805820862 0.663554597266432 0.707859749861954 0.622286787593554 0.630664809919127 0.691491263854114 0.60450762935808 0.691666401946846 0.695150806301788 0.665347434715265 0.661915771986084 0.724359397562131 0.652015508813873 0.670546705825603 0.638989863623314 0.716964742743569 0.628892097099581 0.642133467775786 0.641495562865106 0.6275911509805 0.737278372218974 0.651895599345907 0.615204576995561 0.657899469656215 0.654635814884193 0.645531036478183 0.660699477707773 0.611416919142391 0.604244264557632 0.707577185257914 0.64790362988767 0.667961902066894 0.676792711881955 0.742868680415822 0.662053419271105 0.694924068068988 0.692882768991398 0.625694302025824 0.657160999990307 0.640454345389865 0.690630665107836 0.647786545342572 0.866477619331213 0.657119718512245 0.713374738694043 0.633784671363939 0.691837918147119 0.711145090549096 0.648302449592771 0.762346014477871 0.65375606161283 0.709468717011143 0.72539609453048 0.632737116151284 0.706768322551855 0.642936258921625 0.66756512256593 0.636944699390066 0.651779936965544 0.725787458439838 0.623608380555946 0.747728569654097 0.72226119534401 0.669750841173117 0.709561920471918 0.649412224560325 0.618789085397074 0.662574985876352 0.663527228495614 0.66622838293371 0.627009840017546 0.726352410088724 0.714773563297709 0.581474335819816 0.627035460067326 0.690279250571663 0.661436567454146 0.679179523335057 0.676942229067828 0.643933523059356 0.732783286485934 0.761368265770705 0.682838660447659 0.699126389619454 0.654365176230551 0.650273863872776 0.636246555246053 0.763235035969261 0.730835943579795 0.669497358122394 0.709862190459245 0.678544771645961 0.788573048577789 0.663184266554937 0.64285398031938 0.65265243242352 0.61845903520502 0.668653047996847 0.726501989389413 0.653147358201712 0.692991906239306 0.631390634170442 0.640225270346412 0.656906942200406 0.721481908812577 0.698539572427882 0.665056287580916 0.666444683312542 0.607429801658497 0.656824548249149 0.769276973999239 0.618368452988479 0.623347647535764 0.742621983303335 0.61529868481022 0.608141625815092 0.633074938854597 0.669120533658644 0.617681081807335 0.634091015761282 0.659917132627525 0.699068651965298 0.654887925971346 0.80245734209571 0.668024974609863 0.61608543894161 0.765797920304841 0.699711646236698 0.751997689477483 0.672712075589529 0.642049779925065 0.586700787655627 0.750127205768083 0.701015904399132 0.695631272034708 0.722623681453529 0.723735420346872 0.74851412148016 0.647501582841011 0.648948058739256 0.73955928504213 0.726524629245115 0.722207476589624

Treg 0.776136476543007 0.770676330886401 0.763583446881667 0.773770554377771 0.788936787947621 0.776440853922984 0.817314109137117 0.76361620923593 0.795197022097003 0.814693062834225 0.793797442153964 0.758152373894175 0.742633302541521 0.731016234742316 0.73579523191213 0.789129740508251 0.782762406102907 0.753254911687312 0.751426047165222 0.732353195060037 0.753670156756994 0.816534139150824 0.759341192019262 0.788748513096593 0.784763786664348 0.791136483185886 0.785023856285984 0.753375089405103 0.765182940007537 0.774163030712729 0.786719261995423 0.772488777293101 0.752334479479051 0.817982374820859 0.780544282917347 0.755064405642732 0.76867870252942 0.752900186130145 0.756439723014073 0.755972291586498 0.765678646859962 0.754140545648527 0.748800686484956 0.787240342722362 0.754145663486258 0.773808808143323 0.805664502207538 0.741590088887229 0.794359529553652 0.766441961282921 0.764555991093311 0.741446654276662 0.780968737328164 0.827084501475569 0.753168953877652 0.782356488983931 0.758487778157044 0.795743140446351 0.778063851481553 0.764471046764181 0.827802310709179 0.785450623516776 0.763575562088286 0.772558092278809 0.839346634113064 0.775232682578337 0.753753725608408 0.809131332978733 0.759694513807733 0.817566711964236 0.775916888580772 0.751765138613026 0.770623137308824 0.749740249780541 0.769017580970941 0.775807130250759 0.746085777779214 0.780504823762982 0.764619494638661 0.777696476209057 0.786954513865279 0.781228315176737 0.754055393740463 0.782240396165199 0.805605380904752 0.756145206599992 0.764461129679515 0.746725804433466 0.734516307014022 0.805153294496358 0.761789170857617 0.765513456346886 0.771031149829511 0.79458804694749 0.788012037332634 0.74976812934162 0.774453605529658 0.800121710479437 0.782210009070323 0.797479696586393 0.799071998847324 0.813418084468679 0.770072223584818 0.794632201999427 0.752752441878117 0.776121205557011 0.783604771314628 0.789889417210631 0.790910038594482 0.796761330276953 0.788557241753022 0.755133051862937 0.747992974389058 0.806084122128971 0.773688567869336 0.775825194492206 0.796595247324053 0.792265968888956 0.756070983285169 0.80799709695903 0.779315174324919 0.805639513362133 0.764010928977386 0.78395522973417 0.759891997433251 0.80842877443508 0.785394820558455 0.754987663838117 0.760520520172775 0.743382823285394 0.754792403825171 0.788159117532168 0.796726431177255 0.808080670996919 0.804013567203275 0.773615833398897 0.758826624359015 0.788010377782013 0.76521773535584 0.761187871207965 0.755782865510877 0.819945328078216 0.800513520359929 0.790085412705153 0.771295227279077 0.75727798649468 0.753378445132309 0.754712705528335 0.7665979958307 0.767476909903417 0.763349936745551 0.771422929751132 0.773418746657135 0.784875637599649 0.77460443369571 0.81767282776649 0.796442880710722 0.781159761516471 0.788875395525245 0.751492785475804 0.73840794138622 0.758352344696291 0.767101696228979 0.763375035818291 0.79246054181758 0.763389411599652 0.768411061028817 0.765883035332618 0.770603530539409 0.767215816221094 0.7596398257918 0.82115812683113 0.755376558569941 0.766584150449325 0.802523394374061 0.793255562546069 0.749949849644613 0.770309478359793 0.740566884607586 0.77302186211346 0.786586980062639 0.754522429127069 0.740408428252391 0.774759705405443 0.786612997176199 0.752483206584428 0.803012014291263 0.750191520660827 0.75322721316725 0.764936694552511 0.733838554886803 0.804614654385329 0.750860727937184 0.789714403478289 0.798573132885139 0.803785416676148 0.759741158992022 0.743077269744384 0.760643564735515 0.78127831072003 0.803028961165648 0.783746809103915 0.781484280072227 0.830454259932088 0.759933221822306 0.767267638229603 0.752778116682186 0.762987352603331 0.770566814834258 0.832624360168138 0.753076345847046 0.803392680134377 0.757633849904064 0.796606131411403 0.808314369907175 0.759996188651125 0.766599164877154 0.76132947778935 0.76993685489284 0.748167491886954 0.781198026736858 0.763434453048274 0.785051067432741 0.788946636371255 0.763452896419612 0.826451620354693 0.774066779738458 0.804906613359698 0.797280696585783 0.797304501562557 0.775849550429663 0.77107330724318 0.763100921595854 0.804102518625165 0.780672373326431 0.815777200856066 0.757324586569177 0.758068875215823 0.75520541581042 0.75310606509185 0.784288887909621 0.768006715869801 0.777549602772044 0.770387857688347 0.766668288905755 0.808597035110156 0.785247975331524 0.766970556209055 0.754225878928347 0.842696494358572 0.799156754375931 0.796170962396945 0.814706614596293 0.810062066011685 0.778997772648152 0.802208320152474 0.832961585647242 0.784914940116671 0.755887912503056 0.78284201182436 0.777588075430221 0.768939488480556 0.755468213472316 0.766315649767455 0.767925151818687 0.769935234943288 0.77911239124884 0.757140463324788 0.805174956635421 0.8395328812422 0.79437825563308 0.781055616811933 0.758156162025545 0.768723389012886 0.744290978940392 0.782127032686141 0.78051239140993 0.783592197582676 0.759723337458139 0.806221189965905 0.809918924364069 0.781106710618424 0.771783229608067 0.794077621525059 0.791217346092068 0.811578363200652 0.768432269881168 0.76163969792662 0.793574934885877 0.748861655142047 0.757427227624054 0.775825226528408 0.75630670363222 0.748891813845601 0.78031629749751 0.76871672884126 0.772593977405583 0.779946771647077 0.770927472795931 0.770561787282386 0.771847104661417 0.804989072876747 0.757789140623717 0.767957441178523 0.76367756893367 0.77149602303825 0.820066963686699 0.753969025597729 0.811178912645912 0.773860361321908 0.816706714136304 0.751214867960868 0.774462796552971 0.742329227817153 0.777656285457943 0.771912905613157 0.760937531108456 0.778252304565778 0.754052885765997 0.809010835236901 0.769743492128621 0.788515180660458 0.751456121777355 0.77513531191137 0.801404860536846 0.765175307820825 0.774477579525764 0.801709194077965 0.790944790781823 0.810960601028891 0.780900629337399 0.811659850103066 0.778791819405265 0.848810712274396 0.784670695841653 0.785000672731559 0.750261591575964 0.823833557076466 0.808170203142135 0.745720272005713 0.748339647597939 0.779753486051554 0.75496234892708 0.79729353182336 0.818303741818867 0.852282935895793 0.78048567862964 0.772616755503811 0.792053371631395 0.789745369010822 0.756596090111369 0.746011438883774 0.762328861404814 0.781701888751258 0.777363407180556 0.750152171876582 0.792117509388653 0.803430807581542 0.795622700899828 0.783426687470165 0.76465375526906 0.761379531715029 0.801768275160747 0.799510991460146 0.785650107689321 0.777493963812639 0.751200587551451 0.765092005989571 0.8053716973837 0.755460492542033 0.820840776397405 0.847750099297765 0.779732161625546 0.769928094563519 0.773969715365229 0.760344358736757 0.774022401918631 0.80848941460224 0.797303813254569 0.760494428871753 0.780716082719052 0.845023105211323 0.735834370825663 0.75768955345501 0.760062204030484 0.770485006719419 0.754761684118095 0.788868413214867 0.761888191123747 0.788072867216434 0.757362388910005 0.771654082859914 0.778458980496829 0.760344451504919 0.791530477965467 0.819705455758702 0.754027500041956 0.766439333720611 0.77420861327406 0.788097882241941 0.767334946832939 0.760290403380338 0.781620939668698 0.806139306970706 0.742115793860351 0.765216469007792 0.766770394869041 0.782482381538623 0.745882508002881 0.790602962619526 0.765367513470633 0.76309811845719 0.754142394665085 0.780649334594022 0.756201396163198 0.759791897298434 0.752326238501241 0.765094821314 0.80376375532465 0.771539440931594 0.804013561476441 0.766929485904709 0.755731022554538 0.78203939222353 0.76855338915878 0.775089464658541 0.764713305428179 0.776064807749699 0.78205848412631 0.762806190554319 0.796771013677219 0.759120003730031 0.771521871497316 0.790045506143287 0.759536045788422 0.759303552169889 0.802179063491424 0.796458916012237 0.750300490493924 0.747986969323356 0.789041119011972 0.767357300786479 0.761849945162297 0.785499365309882 0.773794355349432 0.763749710830882 0.794854625724953 0.768762417069911 0.751184840126214 0.756052110239725 0.750674298240521 0.766194449714254 0.781425041100058 0.805812714773168 0.780700131559868 0.793411979250193 0.759150404209663 0.75088503209203 0.778432705078087 0.760679315345471 0.763953620829438 0.757077662942929 0.783108524660037 0.750193393749592 0.777668358702086 0.775380070326076 0.784608539619389 0.773576412875307 0.761451123425423 0.767681040299161 0.760260494409784 0.764837306065017 0.755914200497838 0.801465474038051 0.785809615732977 0.778718838270946 0.779041518863111 0.777695793245584 0.760057926950621 0.798725801190112 0.750508943574858 0.787263550450721 0.76966750871469 0.779721656682929 0.759938346588994 0.783121091510916 0.776456137841898 0.81856465300087 0.779249217345934 0.764207629356278 0.760154520283683 0.762479003732953 0.75504355510594 0.750306209686003 0.786045432490408 0.780581693860452 0.773845908766922 0.761589662750214 0.759664280848634 0.750365545927227 0.838252275855423 0.747622044452221 0.815194034461064 0.791199362864324 0.762794598967324 0.760971186927779 0.788161570883954 0.771267228101475 0.841160745335003 0.757403082029045 0.76508370747129 0.748581772537051 0.757619263157997 0.760188647722638 0.771590789103554 0.781395220688932 0.761994410646318 0.768468316332419 0.754329666236945 0.786217684772583 0.764673148918894 0.764760509730265 0.797780059762681 0.829166575059378 0.774886662959545 0.737490456007855 0.753327777462574 0.745878564378543 0.744033674050968 0.768039248209454 0.752508443190383 0.822194545538467 0.763321159479188 0.755357891811038 0.77253388574173 0.7639953991218 0.797314842676761 0.761374066658863 0.798035277751111 0.773668674069656 0.768229115719553 0.765204488685727 0.772386225682441 0.786916429166049 0.770060200970599 0.806539859550992 0.781225229907992 0.800233656193789 0.779885038441556 0.750029079981921 0.77579908790805 0.760657244702905 0.757396166246623 0.752700654517766 0.794076489935991 0.77612459383493 0.761568421002683 0.75756207939806 0.784866094728752 0.807262583516136 0.803887761788731 0.761839532163684 0.799029511994929 0.793555360371691 0.800330009327513 0.760073492658444 0.805451750755064 0.784183286526609 0.762285631833239 0.781380593484108 0.755487533612473 0.766281084334033 0.777452918941793 0.743365256081921 0.774381745559937 0.775689606538513 0.749638280005307 0.773923065545225 0.787792570256022 0.752975510320017 0.771270992877921 0.774455545373056 0.772318111746939 0.770065954024583 0.767068340120406 0.752860620931933 0.741623982965046 0.811363782165779 0.762236609886875 0.760096639006056 0.753629073461798 0.762886190587321 0.773518571802713 0.768441928548154 0.747219418655006 0.741764515313376 0.774577404885631 0.762280590000068 0.766805715138629 0.776957722237704 0.770729984756783 0.769457095013212 0.753273958756901 0.769082989473701 0.738557675133856 0.774909421838618 0.751480191147878 0.77384019929216 0.750184302937478 0.845653518090724 0.752224443445667 0.806752196314827 0.768453961281341 0.783023156996234 0.796385173235266 0.765016851432322 0.802499654415812 0.744819404239034 0.779659226750579 0.801933553056139 0.770019718975916 0.773728777701681 0.772017735231618 0.771818411179626 0.752022023221143 0.768205783961965 0.777888844867369 0.752962038154676 0.835523873995749 0.811074366392809 0.772498585658349 0.772096839691321 0.760269758316528 0.767061329435358 0.763576309641921 0.742307214906718 0.750709309843747 0.76111472473032 0.789168326709326 0.786950831640694 0.748305043968597 0.741042111425179 0.794157526896788 0.770892784605029 0.779438006268008 0.794775101936963 0.756866339444871 0.801976224083274 0.795210725011544 0.792564789975484 0.778643992499212 0.771161869878811 0.780950303304961 0.752401352045567 0.787735623682297 0.785404345516701 0.760279584256302 0.784453203881925 0.774254645972444 0.8375692903502 0.776968724135988 0.753234160573057 0.757486545859118 0.748174124998537 0.780539816749047 0.797173616662197 0.770160925117334 0.790852129215155 0.758834339024835 0.75781520817766 0.754272345085907 0.78533673878686 0.795494334597529 0.766125358084371 0.760659279029552 0.739945751654967 0.755485773586017 0.819509737903425 0.769937058632712 0.741253549812206 0.783548384277593 0.752363380665883 0.75443865260798 0.770037887985995 0.779488530045262 0.736189209211205 0.745177040240338 0.774129971998488 0.769623097735862 0.756929864557983 0.83571457697355 0.775090276782454 0.738011826119442 0.824548665345339 0.803387181576136 0.793558970233694 0.755302246903659 0.741142683962378 0.749557072311588 0.788720145330654 0.771488696331535 0.788805533381777 0.776685101667723 0.782584985448762 0.825714971266607 0.773158685104034 0.747958378093899 0.807705327113593 0.773591528547431 0.805113048957077

Type_I_IFN_Reponse 0.819001617778118 0.762485311655619 0.792172931921591 0.778050681536683 0.755698368429187 0.803031682761026 0.869564236497858 0.792131550624751 0.764693602857205 0.820549071558311 0.773437553726434 0.764621412605919 0.72583657579632 0.694664373045739 0.754695823006635 0.747097684990734 0.840452096786395 0.787462333191056 0.768350771167831 0.731476247108351 0.715788001879673 0.817773197884056 0.78470255535907 0.825697616273317 0.827629696526623 0.790474128577282 0.803677323639135 0.759673099329147 0.760940853666032 0.892844746168736 0.872089093929328 0.74732688885892 0.760318139097244 0.837326666311059 0.812006788363283 0.765490788558181 0.752744381070059 0.772236067949169 0.761609734397631 0.762940832774442 0.78487663779255 0.810109298261355 0.74148281916905 0.772643920070753 0.800380393435038 0.756235938412591 0.773069882261456 0.742207058394216 0.808054961489479 0.841114481854147 0.765787433890009 0.75838526204808 0.771407320599176 0.850165571773342 0.750262705747798 0.808899665940528 0.758980007598899 0.885370604701949 0.738355472398862 0.766780655186589 0.795595780331152 0.771571737185459 0.781921782310871 0.808291957130459 0.787242159978833 0.859937984221612 0.779639883496532 0.800932322835624 0.761015590117423 0.781044439729542 0.780159084620543 0.774079184062352 0.836862933567978 0.749556478021174 0.830200868498767 0.765180954543748 0.823402762191131 0.854226259811987 0.751685045510738 0.83028048555207 0.820297394050823 0.824011183056499 0.76114599529087 0.792265061493928 0.804078117085248 0.760664207296592 0.719528197590775 0.749699034813014 0.711391454706042 0.787355255703747 0.714925005911599 0.773607007984477 0.743775653203419 0.782578957472415 0.847390544220859 0.725229851942526 0.792912794511303 0.889611437420059 0.755229997812623 0.865774338035748 0.826398918252568 0.818282663090817 0.796818757276782 0.770709693244727 0.731461341795445 0.758589034557888 0.756389221380306 0.819024684182809 0.78893190512312 0.771875204824427 0.821145567176918 0.761768642415167 0.775000600619696 0.885206788123806 0.766314931717608 0.775741104972364 0.803711480238535 0.85551938259607 0.761754555600504 0.825878215913442 0.738120564109241 0.800169557946262 0.769380905238476 0.86580208863249 0.766975149998889 0.800719301158189 0.773947051805368 0.74222293257665 0.78762134147919 0.739161081518418 0.770548815894967 0.877435927363032 0.806281706381137 0.798485699008014 0.81528472913404 0.798858645111101 0.75525478260052 0.767955894235283 0.786002167754355 0.759559374215615 0.758803814814734 0.843529140712635 0.769132892274777 0.731096799127359 0.772466960339454 0.764843171796909 0.77795308347151 0.759126513287383 0.766732709174998 0.814926813131657 0.740263778123513 0.837311637501444 0.760919630666997 0.778422976274207 0.788102522482098 0.775068424634607 0.815477368499852 0.858689256176855 0.794694004354251 0.78337325023948 0.699744877912575 0.753387215324579 0.747464553389332 0.77289549435269 0.767557271264281 0.79010639109537 0.759080736061466 0.771287876235191 0.840050487521014 0.765453801027822 0.804534906315906 0.827314029079869 0.750379238464073 0.766751766800862 0.884038200285142 0.780169664176312 0.749986337046278 0.782654731149494 0.741891847032502 0.829142652459112 0.859344484180109 0.737689456659888 0.788236973950875 0.765413982000871 0.850656603673195 0.769569724518255 0.773740055366033 0.751172926399015 0.753684148751282 0.779353907849313 0.740440733821768 0.783263715912081 0.750704994183604 0.819402084062246 0.812191273105626 0.839883278715966 0.762509872128905 0.751267057547658 0.802737172789162 0.756535048513592 0.815930564013383 0.789473515841837 0.773015857019988 0.787850512508581 0.751448783632671 0.818402080706526 0.734002007440478 0.781062343545908 0.860757670266104 0.792240880548527 0.757471445989068 0.79399754893859 0.754145362629169 0.822892845831839 0.874089496558146 0.794031666682883 0.771274823553913 0.742634684066798 0.799574501550177 0.766890045433702 0.790055865140497 0.739074376495301 0.75447449442107 0.80122896340015 0.74808458024203 0.843622381691372 0.785499587771262 0.818730393187328 0.830973701843347 0.813247199864256 0.783703820307301 0.756830396318412 0.801219230476596 0.822475795520369 0.790222509691438 0.820930695193453 0.726943244940495 0.760812876420937 0.722964154577001 0.753698780892492 0.877003505155383 0.716874474855154 0.754938798975351 0.715361966159965 0.822752600650741 0.813200513614965 0.822423414272752 0.76497037044099 0.783916657828146 0.84493867104825 0.781722095670282 0.786427355465889 0.758704534560426 0.786187300712039 0.764306071730298 0.857355578964751 0.841080071966061 0.788572114898232 0.779185175221651 0.854546972883245 0.756937674662538 0.787175409044394 0.771407148580342 0.768446838818494 0.802763245910422 0.795098689889428 0.80627022353934 0.737875207445544 0.822534664648512 0.826219278958229 0.795420727485192 0.794579152940405 0.761830173306553 0.839328576471205 0.740300350100825 0.822891561858737 0.770025000017795 0.788392515319152 0.747199204361886 0.815503180900387 0.831014098588259 0.77662432370776 0.775510958623835 0.845965677039385 0.796375690641714 0.858935706284479 0.784670352030204 0.740977252364735 0.80214023718189 0.740545313226045 0.748512388348764 0.814373673609881 0.790390525069764 0.747318199526702 0.765032655555239 0.817641301522178 0.763706224755992 0.831463789854911 0.751739180929675 0.768400441602529 0.773232056701688 0.784072386679891 0.732203544723312 0.838732296153257 0.853486540892513 0.816795181628233 0.806049937886976 0.790700575952225 0.77814782552513 0.778137781237132 0.854584201801697 0.754238514551439 0.803030908237056 0.738767603014454 0.861781091575618 0.798569747638749 0.768080544797317 0.774401982589949 0.778843869451603 0.765460253716198 0.766835948180906 0.841875589590734 0.749739074622873 0.761746329321394 0.834269572331207 0.73962630694234 0.811079083037645 0.802124625835448 0.811228310016019 0.837991381299402 0.754033388573529 0.775812930009293 0.789359282945955 0.841029307911787 0.773176470057337 0.805910507870627 0.760555983258281 0.933371690012339 0.825683232642108 0.744905866587754 0.743789047548561 0.766967097996158 0.7615058614306 0.915142770476276 0.817490365116011 0.878763037658427 0.748894716135238 0.781786767401592 0.725213700866249 0.781743056241377 0.776255618021794 0.761300606193018 0.775017440830831 0.774706724987167 0.835515130696008 0.753697569739803 0.839080679553464 0.792177068867668 0.859558648578917 0.748895248051522 0.798248762615984 0.775666205357326 0.853151616812806 0.847483807914047 0.774122113398216 0.757927679270931 0.760192459638581 0.780312448542682 0.844926904582557 0.749445279131043 0.769882342662645 0.832432172847245 0.820332702386016 0.752816379189469 0.744869662396658 0.746997042590043 0.797255039155973 0.816683638341504 0.798851006932932 0.804782035273315 0.813645530365654 0.870057454063708 0.7055026726429 0.758574456195538 0.791568690579842 0.836608840039852 0.782393286695413 0.779149215651098 0.731513573555521 0.864962445104876 0.727778756570038 0.818978198080927 0.809412495807498 0.734310787130298 0.805904688848134 0.77363395789298 0.755695043535825 0.769303206566807 0.798040369545594 0.905240457495708 0.733556302156868 0.715923224050157 0.762788009945562 0.884785547711735 0.739650036350794 0.780462507138689 0.762146950735858 0.768363959399598 0.735283544080699 0.750733760839738 0.780959931676613 0.757697850242339 0.711031861480212 0.824721992025824 0.733068512841672 0.847323891677969 0.76461284944083 0.76625167910488 0.869020339230808 0.756154135615276 0.740739835022378 0.785976850337365 0.814671798244051 0.761128966001951 0.849335463597645 0.771427493182132 0.781745054752375 0.74774835121058 0.755198482511127 0.771218757637263 0.81621217010794 0.731571469636246 0.778644017687682 0.845207690799428 0.760090875276867 0.756724930548458 0.890946393895648 0.881657753576205 0.750334633993577 0.738623325281189 0.817298723359198 0.751971687379079 0.811522164087952 0.801698216599998 0.76169585630292 0.776192946450211 0.85299926171568 0.772979503909804 0.782324135991827 0.783821400240844 0.757484963095387 0.780062684364111 0.798229991378321 0.849339104515041 0.809759676381398 0.863565009220135 0.76412285055336 0.728805984810872 0.869601405018358 0.771622521257476 0.724088629387802 0.763060962292299 0.802689552989304 0.74006061753954 0.756853504995291 0.802000696763605 0.780789009688635 0.757493474772654 0.746401520219493 0.785964390439072 0.743702686867177 0.766452814682876 0.775169656222019 0.825456517931175 0.816985226936704 0.760048359722536 0.784997940301711 0.857168652205266 0.739411898969283 0.788180160485702 0.753951663124919 0.800454416330062 0.79190389813316 0.753937852795477 0.754775781015525 0.837086546817153 0.763291910909877 0.828137638709425 0.8351684192137 0.753169665313513 0.772093784182253 0.785523285049056 0.759054974401755 0.753001086360755 0.778217254265079 0.790133502189752 0.810808763139945 0.7717866896464 0.778496843458212 0.757567796346631 0.890666070316619 0.73787403243478 0.827875488593412 0.773742651119564 0.788151072647297 0.771534094915119 0.810200340997839 0.775940432423797 0.80995169361187 0.687367105732484 0.764318262935085 0.767657046342008 0.729180418666209 0.73341601783084 0.779401567652582 0.761858165912684 0.776663565865331 0.76452928042546 0.744098394490636 0.812806737912094 0.743179778357245 0.770517213365803 0.854856046352505 0.803051356900296 0.737794624144375 0.743161037459223 0.758215902266377 0.735488708839888 0.708870767878316 0.745168238313835 0.752934486609018 0.750367443680542 0.760819809743548 0.740651274947336 0.731909641993102 0.756348619672852 0.862119533483001 0.770451232955567 0.84643404338263 0.770377283794291 0.739739423285679 0.780329424594686 0.776534913497706 0.831995493062096 0.792741374234457 0.78031545833166 0.785205837197578 0.768510552914628 0.760302967564684 0.77914261182446 0.884752074276191 0.771072138271803 0.771830887756866 0.758581247752967 0.752915636868421 0.8080548553159 0.737759714853303 0.765998958008595 0.789959899417006 0.842744880458245 0.737893536533847 0.688086911768706 0.817884603166956 0.796134412425891 0.78507772330284 0.753652947356827 0.895928199561347 0.78227059167993 0.879490080473488 0.776803262810373 0.755891684038522 0.735408600988515 0.780202781557439 0.749229196737116 0.804496140406391 0.76333002285418 0.74313569769468 0.741587611102492 0.803166384272354 0.786700076823888 0.743117768978256 0.751859840656259 0.820478631179864 0.767023672908048 0.743818159868613 0.74941355209972 0.717684128355556 0.847336965314774 0.761296686698925 0.772860408230376 0.76887272026549 0.81084716803384 0.785431785164552 0.78117993607681 0.729254170956837 0.728380550723879 0.858418052159641 0.759536213993625 0.748977502042681 0.807832016989659 0.791908013719167 0.769455193933493 0.755364098267641 0.746367307273114 0.730589871871101 0.764155606902587 0.706184178295973 0.790011019018801 0.752106561318693 0.824280478634747 0.740811199369681 0.799276083656685 0.770601260210838 0.746224474871633 0.885493603576653 0.792969837370585 0.773523573920236 0.787693086572839 0.766272794084198 0.779445678944906 0.742599010341044 0.805683878981672 0.769789740424529 0.772900696731286 0.779951460343029 0.761540273970752 0.785605050415318 0.767833052501503 0.826789696684338 0.847036830988647 0.75797553958375 0.83052575513717 0.74142469397016 0.740621013959262 0.723168762734268 0.744176117706803 0.747372378138133 0.76927737269742 0.822637067045332 0.803441383704514 0.736572586932799 0.756264023862356 0.871971370796267 0.750827146730349 0.742019431288878 0.851534252093235 0.765818112004321 0.806924257909452 0.84861858362522 0.786516285340426 0.789641538345352 0.769927318273448 0.766644484459858 0.727376115689844 0.799301878501703 0.82989119202764 0.772507229275614 0.814967323918231 0.775171746000379 0.817780897285546 0.756948812083309 0.774674382458333 0.75692981981014 0.748134988069767 0.759362806455363 0.803116214180293 0.797054785880298 0.825025796464598 0.754232071627147 0.786503887937692 0.7329928159913 0.814549173136152 0.837644200785607 0.794353991294306 0.757450669148175 0.777795250205141 0.760061730019125 0.913513800680791 0.755923897998193 0.74643389735979 0.81042859405268 0.746436226177729 0.721372962887084 0.757418917572088 0.750997913780868 0.731581747915372 0.728506501731209 0.773959963712798 0.882282118058551 0.825546262301096 0.8411754830479 0.768954615709728 0.773327541151398 0.815384368417174 0.762141397142673 0.912338774959431 0.771034920641755 0.730124137356453 0.72736865815821 0.814238457790353 0.799696762499769 0.792078737086131 0.784368511828351 0.797944947739433 0.779236341017 0.778259506715472 0.784540352845911 0.817016640516887 0.775343001866626 0.873929020468726

Type_II_IFN_Reponse 0.639831045303022 0.610622796012739 0.653579320801052 0.628910503384057 0.614344547831343 0.601720477338648 0.657292258291321 0.685871036158918 0.682915538872471 0.67585197328949 0.651908853159807 0.641489290316318 0.563716295133279 0.523465675212493 0.575272459613823 0.620132912233 0.646700847573178 0.660520427303104 0.58001633621226 0.488510368894566 0.544220367413664 0.706402609696122 0.624417912195777 0.621316647570597 0.65570135852543 0.605088166110717 0.76070741838049 0.592455483962359 0.621514008594478 0.620167530095603 0.588401460592821 0.648243398561741 0.571776173161288 0.560199476647083 0.587833911282463 0.599330143606213 0.621161811550679 0.486728715735957 0.595685649377607 0.678136694201205 0.630018668480122 0.665192335763787 0.551643017339076 0.634912327713956 0.537472942460838 0.638517265819559 0.653317441713376 0.564052936580397 0.652913334143576 0.6038936225437 0.592029017821026 0.536000859183498 0.629311132050221 0.698424358613113 0.633547020742964 0.618611547805582 0.567407850811095 0.627123984650892 0.65345395632825 0.69383315505413 0.580194253290114 0.627721676043807 0.560858427254571 0.607261830807512 0.740606950436941 0.568275569756593 0.54490563289805 0.754964838202511 0.571410056073721 0.682356666806294 0.653664205517468 0.604199503078147 0.541835663768269 0.557693649439465 0.597244651509093 0.623920740473157 0.585565472379683 0.591274149012135 0.618864069837119 0.532724317633244 0.639283775128988 0.565992493768663 0.590888856090633 0.693820000687222 0.684071930950215 0.598311436669017 0.548208041556026 0.625045025436051 0.564456371035062 0.689814143647667 0.681301339797891 0.570760986795247 0.549473509204883 0.63227145818556 0.601663734862162 0.528363773455558 0.609505967060018 0.639911998323257 0.645244416894335 0.617490900847049 0.578298569850393 0.599351469279217 0.626296649504152 0.58033458696379 0.628742662563168 0.637241390912033 0.648754089385163 0.602711323408039 0.644560889203666 0.677027448599189 0.594850688403196 0.545508126349049 0.626176109889143 0.550158819990879 0.660961868498956 0.643382771472751 0.636241158253593 0.58086976104573 0.613338013995096 0.681895849048411 0.631782684618597 0.661187910007185 0.627186190599921 0.699243912005886 0.649734657167529 0.686002564874769 0.655025398527216 0.609646761281737 0.658829293029181 0.606973621398609 0.635362116471436 0.623864041257882 0.631160560906951 0.678320588981068 0.673923937501422 0.682923042819786 0.618999650096076 0.613637335195423 0.59771675807508 0.655492125067318 0.580840414643894 0.669566330308587 0.605115713872708 0.63469542666519 0.62619035023707 0.592727891306878 0.599322214157964 0.553295101402713 0.602347059066566 0.59631900144578 0.605094904064231 0.618869352103216 0.6011211720632 0.630940132439652 0.609740835393822 0.662626706905423 0.546020878894749 0.612703456882991 0.64272809023198 0.505011075007723 0.552424878618062 0.663431634518356 0.57582520033864 0.614103257355673 0.602567603211 0.595614709108689 0.619543873334354 0.57552322313379 0.594685146637403 0.620378152810162 0.573073975676282 0.708480219087914 0.544914989276979 0.595084047264148 0.638015945695068 0.517773128195635 0.613745098388952 0.572356911193832 0.596053784308114 0.599940589372386 0.671084696873983 0.602222751249642 0.574835770618597 0.686082977971203 0.666993577187369 0.624933938890628 0.650995441085895 0.598818659989441 0.569930857469678 0.68497947296944 0.515265181717247 0.632512590893552 0.599662378721438 0.598722356934501 0.659894441579148 0.73842974769883 0.621360597934949 0.541375728105658 0.526664393983621 0.617447806291006 0.570956430697001 0.62502396425217 0.613270649961673 0.664123513484423 0.604800849019237 0.594686693775499 0.603351864803555 0.649531278807765 0.577574001394231 0.712409651477053 0.542651555275658 0.725646517254234 0.595867502680651 0.649149405367522 0.695003413613679 0.655327453576778 0.624946722406371 0.601033071566334 0.668821226060021 0.537294697151705 0.595093096051136 0.55513843928721 0.661998740731559 0.622841831104537 0.564499540372426 0.783606554929301 0.632476970559437 0.64630005409486 0.537550805980659 0.653473387976267 0.578467909066915 0.610090510738247 0.655459129305308 0.672973850943686 0.642090559851158 0.655754447873401 0.566327619672589 0.583437685844559 0.573747295413529 0.538548516700603 0.572569200249595 0.612870021833695 0.629374813198875 0.648943002177905 0.611998990209392 0.695141034355117 0.615015704616009 0.651670960060857 0.597213375260902 0.816139932547577 0.697162112844789 0.602858844454075 0.615451258866642 0.664205617488621 0.633945687035856 0.666325929667306 0.739005464187516 0.647366044097858 0.604073096512952 0.6393970758022 0.607637383930735 0.633401731770245 0.601140823474673 0.622637891587723 0.640047649516484 0.649490482323778 0.622188936379309 0.567320855732535 0.6542414829873 0.768063929836523 0.685864019407822 0.635016702596777 0.579881337858372 0.585092747338319 0.494493488521688 0.661591314609059 0.60901289671272 0.655887170240252 0.524993635594312 0.638403228206709 0.685951276691742 0.626853873506859 0.602725264120045 0.670904678426963 0.686276120518927 0.663633178274489 0.599448160635468 0.651897667469935 0.656232777618193 0.588427667242634 0.596258459653437 0.584258784272594 0.584184882654212 0.578029951069895 0.648729569912529 0.646951985449948 0.607308724008382 0.648860553237708 0.604411159882753 0.587323537635362 0.627654588336512 0.708991329189552 0.544018099494686 0.540033048341989 0.632144720694447 0.626672561723456 0.667533804520589 0.602769170127515 0.628938895662961 0.623683407140299 0.695083524521736 0.615290990530028 0.651242796830063 0.563873681949769 0.559568783658711 0.620915908979939 0.608756628872456 0.658148196859255 0.546908614359674 0.635226713314896 0.621517966247297 0.642010763610879 0.596597907601689 0.600273240839035 0.616302704528088 0.615971135951602 0.557534610339424 0.7280989261329 0.648735003941859 0.637527713642192 0.614438633298162 0.607158025867348 0.562169148991348 0.82682342406537 0.677462799850048 0.625242876367762 0.617385009317466 0.699400674402017 0.616253071548462 0.502475000764163 0.587575202820321 0.683548537239056 0.589544191876889 0.614376490329057 0.665530661809653 0.758284479034877 0.641996100057129 0.613675788259296 0.601886100889362 0.663000249519687 0.620809409318544 0.54798921211416 0.625816427143606 0.602802788490769 0.563835797290119 0.628390204642362 0.568011290168507 0.652534410007715 0.668265675309972 0.616148455881325 0.681275466085059 0.45824238469145 0.654481176658721 0.6300689275534 0.603199499152032 0.563309275477814 0.540182194722361 0.501838888291702 0.642459115857897 0.56654289057163 0.564836524016066 0.759503848864251 0.625706771046732 0.537566647110004 0.713451866285756 0.592083075922026 0.649409837139822 0.632274600440199 0.623454689613599 0.660394509248976 0.652873897981959 0.711118409142406 0.559120945122577 0.593556440981043 0.57943808693482 0.569005634217896 0.624311214853359 0.710099853525461 0.559352806317753 0.596307870540939 0.608214298992927 0.623069947782354 0.612596329048514 0.618596451771486 0.57615238053621 0.688528330712075 0.609740057274307 0.654554994728793 0.616063785596346 0.643794581553499 0.590141482625763 0.594802017688125 0.608748673812187 0.600004250249216 0.571151175408021 0.599866937774297 0.646413743638763 0.667896866501736 0.630773894127503 0.654309617617192 0.604165558777752 0.605135699607776 0.570966848678469 0.619501519779011 0.584328492111581 0.50442256464443 0.570961522352589 0.5800015966778 0.622121237775908 0.658769061273574 0.612801779414515 0.635014420132844 0.672322522581956 0.646161020905294 0.516320175742524 0.614905213595552 0.59263999801644 0.616110687377582 0.609784739481867 0.620870044375032 0.601380250289491 0.625804292122629 0.655704399666011 0.651424275401579 0.593133972690822 0.585455202407725 0.634791911650621 0.622318900872593 0.543164510968861 0.604579929130617 0.663648345810673 0.547669960267835 0.534052130733507 0.668360282549237 0.570144880918286 0.630486364919991 0.701044727421543 0.638109566887814 0.587400740752553 0.569911989237428 0.614695990213977 0.580321882679042 0.597448654038641 0.667342763785509 0.554446565930438 0.608384596461795 0.6189299998563 0.548530909862204 0.661832379585781 0.592631492951953 0.579799408220827 0.611802357168459 0.701207342265716 0.592808318589201 0.697463835405929 0.619299764212191 0.688204271972569 0.541769126440036 0.624755008886557 0.61330255316342 0.507003844489148 0.543897747909864 0.575328785371797 0.666906751498753 0.609142987311021 0.669649383260355 0.628354959487487 0.535173835459704 0.532445080707363 0.667070206413886 0.513789224531497 0.55044008052688 0.636325499649003 0.631498194157564 0.595271399342478 0.624253974072108 0.567649202161061 0.733484220318691 0.656489648203145 0.589223307761816 0.563505003906175 0.628629661050728 0.60888242779507 0.641720721274372 0.654307784899095 0.60221377689418 0.592394464570597 0.636013645356866 0.604355873127898 0.499657110398928 0.70592125417995 0.520815106097767 0.694681904194355 0.606250906582135 0.62723491269649 0.595464262735268 0.672073452403937 0.615594609134419 0.685969394227381 0.632287470010448 0.527756547030247 0.599230325314842 0.585092627664915 0.577261715998416 0.607523113177302 0.657820137533325 0.621892949272963 0.569192455333429 0.603297717174822 0.61879869014804 0.611531388624075 0.625384517189495 0.642449132357969 0.636051031303383 0.652283265369175 0.554769896323657 0.593945402245957 0.559558595739783 0.584890534811702 0.605539473866861 0.617368600776045 0.663552183632389 0.595580678822886 0.614506105932894 0.586017054724271 0.609634674347603 0.673646524205171 0.601453754832993 0.622571778918487 0.643031710587127 0.66340369158481 0.608661659396567 0.687660697465869 0.672459552404266 0.656690566668681 0.701707822306376 0.624607237126253 0.7218114468955 0.665479826195204 0.556294162972586 0.581345489690219 0.585177301324727 0.616983405808035 0.597029103008221 0.66980128364617 0.548678117013228 0.532894007860242 0.641046628624668 0.621907227342191 0.71228342333056 0.553260984718396 0.622686753862907 0.606070303461822 0.648806285758377 0.685665187905689 0.564775307298142 0.625102606159619 0.601186267517068 0.593547667584022 0.606829816625577 0.610063853013072 0.643858486872086 0.559662723663381 0.588035490441485 0.708946357504016 0.576623671717721 0.614347724696115 0.674814315910312 0.6625065424796 0.617066809252329 0.559254781717045 0.586869903177729 0.600295690059454 0.589787972913043 0.593662540843922 0.547131764369638 0.625161338155041 0.663564690627801 0.5878852290646 0.596287563233716 0.612973916760828 0.616305751811421 0.5757481766722 0.667556069326413 0.624828541187798 0.602290964231988 0.57784775047026 0.598565834038305 0.602667325014011 0.571527413157691 0.634688568743645 0.611962072528315 0.577763498682994 0.653255243716782 0.548485871449785 0.6369961346982 0.599922873499781 0.598905164811011 0.542180399096086 0.735587498391428 0.629501071941077 0.637290728071772 0.580634085618933 0.615483043134956 0.597404011482733 0.613321037516357 0.762120091242269 0.543754290156398 0.611240451823806 0.64829940077296 0.607972431109948 0.684785783699693 0.637608886915967 0.603792088684988 0.618043658067315 0.616143331338434 0.612621956067382 0.602414892492894 0.743027835507458 0.604521028365227 0.633294003753802 0.613623523745806 0.599675516229487 0.585694008688966 0.588514610431914 0.577616591345248 0.614079928527806 0.601590224847057 0.693589525139293 0.669947744923631 0.565578980840626 0.594318956326735 0.616203779701785 0.582051243783173 0.610108445895079 0.585421706414089 0.57826464598392 0.640136474676099 0.629443129028034 0.647099387251189 0.570787548262354 0.625101704597201 0.618795568887712 0.623697330453866 0.598402329732517 0.596689232090114 0.608578215797463 0.668821523236801 0.730361929620891 0.660071509458357 0.588192817031889 0.49379831695545 0.582594168861369 0.533246035670413 0.594463869210016 0.634137380877001 0.555114110580256 0.63995546334136 0.609364452392422 0.651825457728475 0.556250069289762 0.523385457731821 0.625469628746895 0.641717579913228 0.640615820430326 0.516813826890324 0.546416352158865 0.628880692015919 0.612806024013361 0.505146101872104 0.610179167527305 0.619686838742323 0.583318936581556 0.644923229936514 0.634155973173088 0.551991945622471 0.590154896274137 0.62872711087594 0.647454039539756 0.601372568939619 0.689281975016366 0.6108581676514 0.509629731611148 0.647882600804385 0.630724274967607 0.631616501119393 0.63409527210553 0.561978646734447 0.530730273604754 0.670969882143832 0.60832831682982 0.624899406691122 0.578669582051699 0.669324674215839 0.651721489579202 0.610570957929867 0.590777730653256 0.652620727598118 0.607284544681616 0.656870385454408
