# Supplementary material for: Associations between migrasome-related genes and long non-coding rnas in glioma and their prognostic relevance to the tumor microenvironment
Source: IBRO Neurosci Rep. 2026 Jun 24;21:279–90. doi: 10.1016/j.ibneur.2026.06.013 (PMC13356737; doi:10.1016/j.ibneur.2026.06.013)
Supplement: Supplementary file 9 — Supplementary material [file mmc9.docx]

| **cohort** | **feature** | **genes_used** | **n** | **events** | **HR** | **HR_95L** | **HR_95H** | **Cox_P** | **LogRank_P** |
| --- | --- | --- | --- | --- | --- | --- | --- | --- | --- |
| CGGA325 | available-gene partial score | CRNDE;AC007879.2;LINC00092 | 313 | 218 | 1.54474413635094 | 1.37299607098204 | 1.73797616557187 | 4.78281644727776e-13 | 9.32122974823161e-07 |
| CGGA325 | CRNDE | CRNDE | 313 | 218 | 1.42203501792073 | 1.30628473840373 | 1.54804196416159 | 4.36481416100338e-16 | 1.06634468992759e-27 |
| CGGA325 | AC007879.2 | AC007879.2 | 313 | 218 | 1.28079555417223 | 1.12404678674256 | 1.45940299899907 | 0.000202735562752277 | 0.0793406265828715 |
| CGGA325 | LINC00092 | LINC00092 | 313 | 218 | 1.20505544943352 | 1.06923030280022 | 1.35813456877003 | 0.00223518229497674 | 0.231102310169047 |
| CGGA693 | available-gene partial score | CRNDE;LINC00092 | 657 | 394 | 1.34003866511113 | 1.26728294210465 | 1.41697135212015 | 8.9757955885295e-25 | 3.2312013361461e-29 |
| CGGA693 | CRNDE | CRNDE | 657 | 394 | 1.35188843320164 | 1.27558460326448 | 1.43275666008134 | 2.66184084764068e-24 | 4.08966496267252e-27 |
| CGGA693 | LINC00092 | LINC00092 | 657 | 394 | 1.2917952395037 | 1.1954196629773 | 1.39594068299687 | 9.66740260250835e-11 | 2.94309625460854e-08 |
| GSE16011 | available-gene partial score | CRNDE | 240 | 213 | 1.43174434521692 | 1.24647556897968 | 1.64455037954623 | 3.85181003648406e-07 | 2.52428559298703e-08 |
| GSE16011 | CRNDE | CRNDE | 240 | 213 | 1.43174434521692 | 1.24647556897968 | 1.64455037954623 | 3.85181003648406e-07 | 2.52428559298703e-08 |
